# Tree diversity and standing carbon stocks in smallholder forests of Ciamis Regency, Indonesia: Effects of cropping patterns and elevations

**DOI:** 10.1371/journal.pone.0356872

**Published:** 2026-09-21

**Authors:** Mohamad Siarudin, San Afri Awang, Ronggo Sadono, Priyono Suryanto, Muhammad Abdul Qirom, Budi Mulyana, Antun Puspanti, Donny Wicaksono, Fatahul Azwar, Said Fahmi, Freddy Jontara Hutapea

**Affiliations:** 1 Research Center for Ecology, National Research and Innovation Agency (BRIN), Cibinong, Bogor, Indonesia; 2 Faculty of Forestry, Universitas Gadjah Mada (UGM), Depok, Sleman, DI Yogyakarta, Indonesia; Forest Survey of India, INDIA

## Abstract

Smallholder forests (SFs) provide critical ecosystem services by supporting biodiversity conservation and carbon (C) sequestration in tropical landscapes, yet how these functions vary across management systems and environmental gradients remains poorly understood. We investigated the effects of three main cropping patterns (tree crops, mixed-tree lots, and agrisilviculture) on tree diversity and aboveground C stocks across three elevation zones (< 200 masl, 200–500 masl, > 500 masl) in Ciamis, West Java, Indonesia. Data were collected from 150 plots (50 plots per elevation class), which were unevenly distributed across the three cropping patterns. Trees were classified into three growth phases (saplings, poles, and mature trees), and DBH and total height were measured to estimate C stocks, while tree diversity was assessed using standard diversity indices. The results showed that mixed-tree lots and agrisilviculture supported higher species richness than tree crops systems, which exhibited lower diversity and greater dominance by a limited number of species. Carbon stocks varied significantly among cropping patterns and growth phases, but not across elevation zones, suggesting that management-associated cropping patterns and stand structural attributes exerted a stronger influence on aboveground C storage than topographic variation. Mean C stocks ranged from 41–57 MgC ha^-1^ in mixed-tree lots and 37–51 MgC ha^-1^ in tree crops, compared with 23–32 MgC ha^-1^ in agrisilviculture systems. Overall, these findings highlight the potential of mixed-tree lots to simultaneously promote biodiversity conservation and C storage in tropical smallholder forest landscapes, though the results should be interpreted within the scope of this observational study, as economic performance and long-term sustainability were not evaluated.

## Introduction

Forests represent one of the Earth’s largest terrestrial carbon (C) reservoirs, storing approximately 870 PgC globally while supporting critical ecosystem functions, including biodiversity conservation and hydrological regulation [1,2]. However, continued forest conversion remains a major source of greenhouse gas (GHG) emissions, contributing nearly 2 PgC year^-1^ between 2015 and 2019 [3]. Increasing tree cover through afforestation, reforestation, and agroforestry has therefore become a key strategy for climate change mitigation [4]. Although these approaches are often implemented in public forests, privately-owned land, such as smallholder forests (SFs) also represent an important opportunity to enhance C sequestration while maintaining ecosystem functions.

Smallholder forests (SFs) have long existed in Indonesia [5,6] and are typically managed by households on privately owned land, often located within or around village landscapes [7]. In the Indonesian context, SFs are defined as privately managed forest areas of at least 0.25 ha with a minimum density of 500 trees ha^-1^ [8,9]. While these thresholds provide a regulatory framework, their application in practice varies across regions depending on local land-use history and management intensity. In recent decades, SFs have expanded steadily, driven largely by the growing demand for timber and rising timber prices [10]. On Java Island, SF area increased by 73% from 1.5 million ha in 2003 to 2.7 million ha in 2010 [11], with approximately 0.9 million ha are located in West Java Province [5].

Unlike intensively manage plantations, SFs are generally characterized by flexible and simple (traditional) management systems designed to balance household needs with long-term land productivity [12]. Farmers typically cultivate commercial timber species, such as teak (*Tectona grandis*), sengon (*Paraserianthes falcataria*), jabon (*Anthocephalus cadamba*), kayu afrika (*Maesopsis eminii*), and mahogany (*Swietenia macrophylla*), often intercropped with valuable understorey crops, such as coffee, cocoa, tea, and cardamom [13]. However, management practices vary widely depending on the socio-economic conditions of landowners [5,14].

Beyond their contribution to rural economies, SFs provide important ecological functions by diversifying landscapes, buffering protected areas [15]. Moreover, SFs contribute significantly to timber production and enhance the economic welfare of local communities. In Java, SFs produce approximately eight million m^3^ timber annually, equivalent to about 70% of the timber supply for furniture industry and generate around USD 360 million income annually for farmers [16]. In West Java, SFs contribute between 10% and 30% of total household income among farmers [14]. In addition, SFs provide a range of ecosystem services, such as improvement in soil quality and water system [10,17], provision of habitat for fauna, and mitigation of GHG emissions [18,19].

Compared with monoculture systems, agroforestry systems, including SFs, generally support greater plant diversity due to intentional integration of trees and crops or livestock within the same landscapes [20,21]. In these systems, species selection is influenced by economic value, ecological functions, and ethnobotanical values [21,22], resulting in distinct cropping patterns with different level of structural complexity and species composition [23]. These differences may have important implications for biodiversity and C storage because stand structure and species composition regulate biomass accumulation, resource availability, and habitat conditions. Furthermore, these relationships may be modified by environmental gradients such as elevation, which influence climatic conditions and ecological processes.

Recent ecological studies indicate that biodiversity-C relationships are mediated not only by species richness but also by variation in functional traits, functional diversity, resource-use strategies, and structural development [24,25]. Functional differences among tree species influence light interception, resource acquisition, growth, and biomass accumulation [26], whereas environmental and management filters, such as soil conditions, disturbance, and silvicultural practices, determine which species and functional traits persist within agroforestry systems [27]. Consequently, understanding how management interacts with environmental conditions to shape species composition and stand structure is essential for explaining variation in biodiversity and C storage across SFs.

Despite extensive research on the socio-economic characteristics, productivity, and sustainability of SFs [28–30], little is known about how different cropping patterns and environmental gradients influence tree diversity and C storage. In particular, the mechanisms underlying biodiversity-C relationships in SFs landscapes remain unclear because they are influenced by multiple interacting factors, including species composition, stand structure, soil conditions, disturbance history, and management intensity [31,32]. Recent evidences suggest that C storage in agroforestry systems is not determined by species richness alone but is strongly influenced by stand structure, species composition, management practices, and environmental conditions [33–36]. Therefore, understanding the relative importance of management and environmental variation is critical for identifying SF strategies that simultaneously support timber production, biodiversity conservation, and climate mitigation.

This study investigated how cropping patterns and elevation gradients influence tree diversity and standing C stocks in SFs in Ciamis, West Java, Indonesia. We compared three dominant cropping patterns (tree crops, mixed-tree lots, and agrisilviculture) across three elevation zones to assess the relative importance of management and environmental variation in shaping tree diversity and C storage. We hypothesized that (1) cropping patterns with greater structural and compositional complexity would support higher tree diversity and C stocks than simpler systems, and (2) cropping pattern would provide a stronger influence than elevation because management directly shapes stand structure and species composition. Our findings are expected to provide valuable insights for promoting more sustainable and multifunctional management of SF in Indonesia.

## Methods

### Study sites

**Fig 1:**
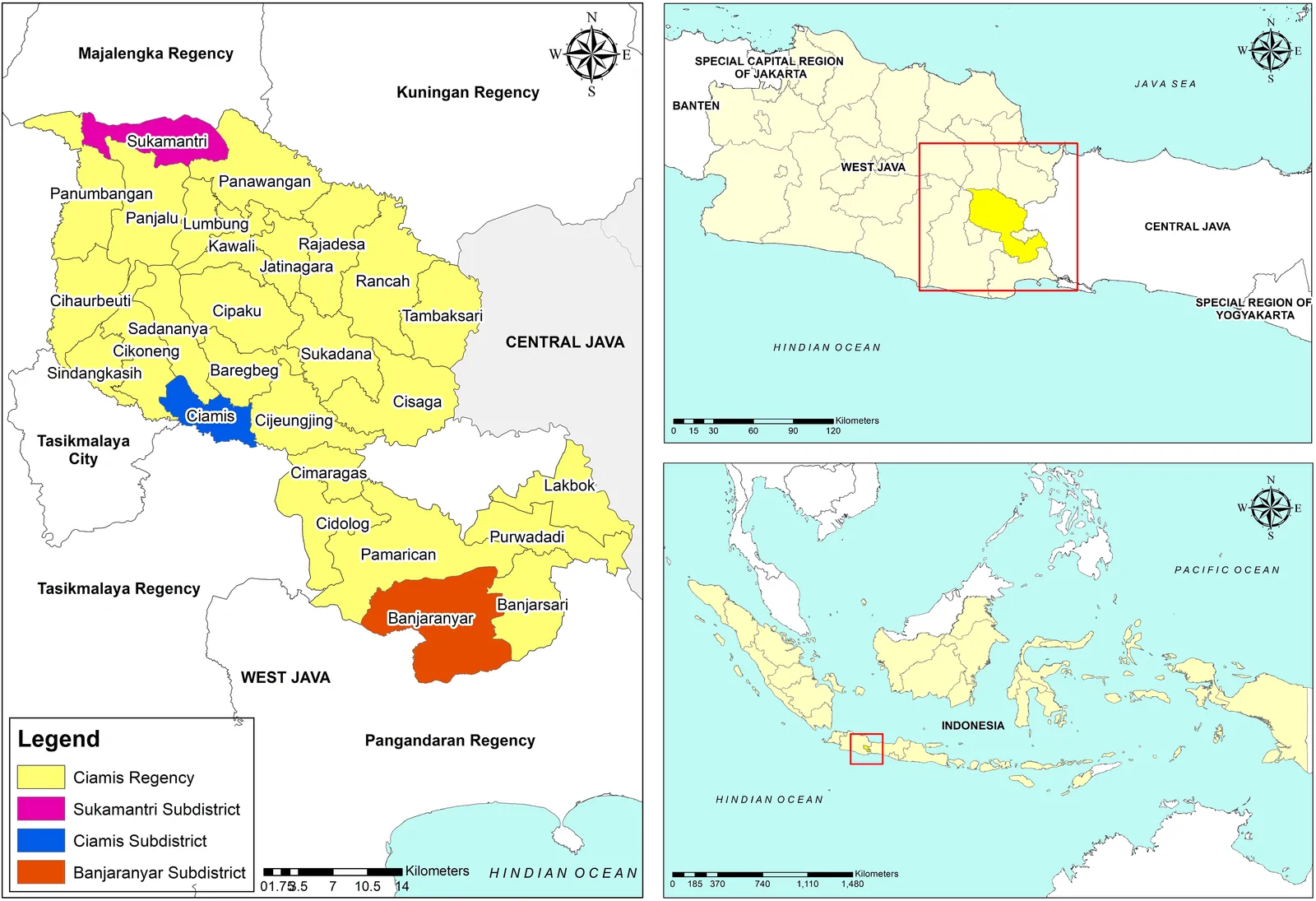
Map of study sites in Ciamis Regency, West Java Province, Indonesia.

**Fig 2:**
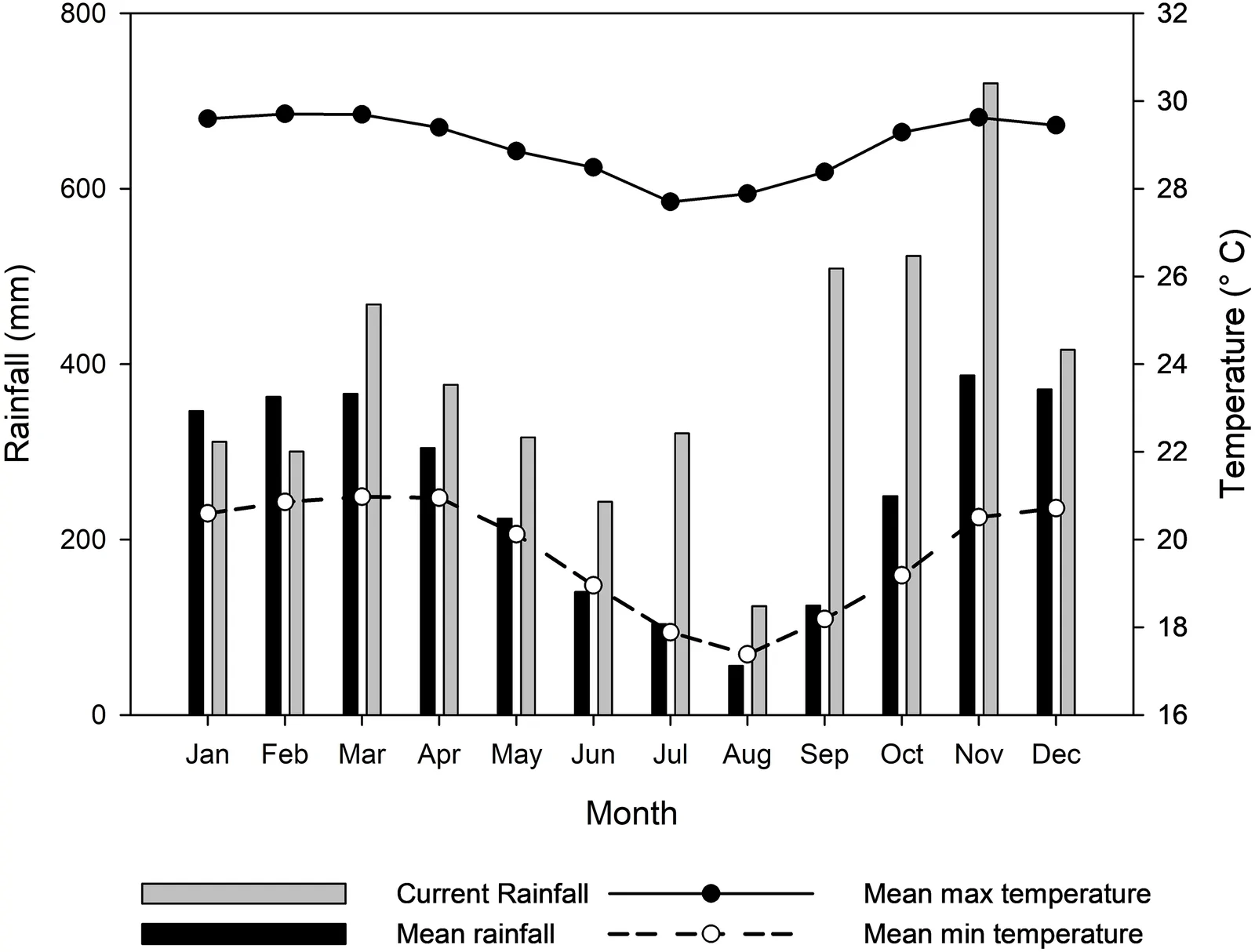
Mean monthly rainfall and temperature in Ciamis between 2000 and 2022. Developed from National Aeronautics and Space Administration (NASA) [38].

This study was performed in Ciamis Regency, West Java Province, Indonesia (Fig 1), located between 108°19’ - 108°43’ E and 7°40’30” - 7°41’30” S. Elevation ranges between 0–1,775 meters above sea levels (masl) and tends to increase from south to north, except in the western part of the regency, which is characterized by hilly terrain [37]. Mean annual temperature ranges between 19.7 and 29 °C, with a mean annual rainfall of about 3,037 mm (Fig 2). Based on the Scmidt Ferguson climate classification, the region has a type C (moderately wet) climate [37]. The dominant soil orders are Andisol in the northern region, Inceptisol in the southern and central region, and Ultisol in the southern and central region. Although recognized as important determinants of tree growth, species composition, and C accumulation, soil information is provided to characterize the environmental setting of the study area, thereby, it was not included in the statistical analysis because soil data were only available at a regional classification scale rather than for individual plots. As this study focused on the effects of cropping patterns and elevation on tree diversity and standing C stocks, analyses were restricted to variables consistently measured at the plot scale.

The classification of SF cropping patterns in Ciamis is based on stand structure and species composition as outlined by Siarudin et al. [5], who conducted a related study in the same sites. In that study, cropping patterns were identified using four categorical variables (tree species composition, tree age, tree spatial distribution, and intercropping) and two continuous variables (stand density and basal area). These variables were classified using a Two-Step Cluster algorithm with a log-likelihood distance measure and automatic cluster determination using Schwarz’s Bayesian Information Criterion. The analysis identified three primary patterns of SF: tree crops, mixed-tree lots, and agrisilviculture. As this study was conducted on the same plots using the identical field inventory dataset by Siarudin et al. [5], and no structural reclassification occurred between surveys, the previously established categories, tree crops, mixed-tree lots, and agrisilviculture, remain directly applicable and analytically valid.

The tree crops pattern typically consists of one to three predominantly even-aged tree species established without regular spacing, and it is often intercropped with understorey crops. Stand density averages approximately 1,800 trees ha^-1^. Common tree species include mahogany (*S. macrophylla*), teak (*T. grandis*), sengon (*P. falcataria*), jenitri (*Elaeocarpus sphaericus*), kayu afrika (*M. eminii*), and cempaka (*Magnolia champaca*).

The mixed-tree lot pattern comprises uneven-aged, multispecies stands without understorey crops and has a similar stand density of about 1,800 trees ha^-1^. Dominant tree species include mahogany (*S. macrophylla*), largeleaf rosemallow (*Hibiscus macrophyllus*), sengon (*P. falcataria*), durian (*Durio zibethinus*), langsat (*Lansium domesticum*), teak (*T. grandis*), coconut (*Cocos nucifera*), kayu afrika (*M. eminii*), jenitri (*E. sphaericus*), and needlewood tree (*Schima wallichii*).

The agrisilviculture pattern consists of clump-distributed, uneven-aged, multispecies tree stands integrated with understorey crops. It has a lower stand density (around 1,223 trees ha^-1^) than the other cropping patterns. The canopy composition partially overlaps with the mixed-tree lots, as both systems commonly cultivate teak (*T. grandis*), sengon (*P. falcataria*), kayu afrika (*M. eminii*), mahogany (*S. macrophylla*), and jenitri (*E. sphaericus*). However, the agrisilviculture pattern generally exhibits a more selective set of canopy species and greater emphasis on crop integration. Major understorey crops include cardamom (*Elettaria cardamomum*), banana (*Musa* sp.), cassava (*Manihot esculenta*), taro (*Colocasia esculenta*), salak (*Salacca acehensis*), and fingerroot (*Boesenbergia pandurata*).

### Field data collection

Data were collected across three cropping patterns (tree crops, mixed-tree lots, and agrisilviculture) and three elevation classes (< 200 masl, 200–500 masl, and > 500 masl), following the protocol provided by Siarudin et al [29]. A total of 150 plots were established across all cropping patterns and elevations to assess tree diversity and C stocks, with roughly 50 plots per elevation class, distributed proportionally to their field occurrence (Table 1). The uneven plot distribution across cropping patterns and elevation classes reflect field condition, thereby enhancing the ecological relevance of the study. As a result, the design is not fully balanced across factors. While this may reduce the ability to detect interaction effects, the analysis remains suitable for evaluating the main effects.

**Table 1:** The number of plots in different cropping patterns and elevations.

Cropping pattern	Elevation		
	< 200 masl	200-500 masl	> 500 masl
Agrisilviculture	10	16	16
Mixed-tree lots	23	24	17
Tree crops	17	10	17

Trees were classified into three growth classes: saplings (height > 1.5 m and diameter at breast height [DBH at 1.3 m] < 10 cm), poles (DBH 10–20 cm), and trees (DBH > 20 cm). In brief, field measurements were conducted within 20 m x 20 m plots at each location. This plot size (400 m^2^) represents a commonly used fixed-area sampling unit for assessing tree diversity and forest structure in vegetation studies [39] and is consistent with forest inventory approaches recommended in the Indonesian national standards for forest C measurement [40]. Plots were separated by a minimum distance of 200 m to reduce potential spatial autocorrelation. Within each main plot, nested subplots of 10 m x 10 m and 5 m x 5 m were installed to measure poles and saplings, respectively. All individuals within the three growth phases were recorded for species identity, DBH, and total height. In this study, tree diversity encompasses all perennial plant components recorded within plots, including tree species, palms (e.g., coconut and areca), and perennial crops (e.g., coffee and cacao), reflecting the integrated management practices typical of SFs. Tree height was measured using a Haga altimeter.

### Data analysis

Species richness (*S*), Simpson’s dominance (*D*), Shannon’s diversity (*H’*), Simpson’s diversity (*D1*), Pielou’s evenness (*J’*), and Margalef index (*d*) in each plot was calculated using Eq. (1), Eq. (2) [41], Eq. (3) [42], Eq. (4) [43], Eq. (5) [44], and Eq. (6) [45], respectively.


$$\begin{array}{c} S=the\;number\;of\;species \end{array}$$ (1)

$$\begin{array}{c} D=\;\frac{\text{1}}{\Sigma P_{i}^{\text{2}}} \end{array}$$ (2)

$$\begin{array}{c} H^{\prime }=-\;\Sigma P_{i}\text{ln}\left(P_{i}\right) \end{array}$$ (3)

$$\begin{array}{c} D\text{1}=\text{1}-\;\Sigma P_{i}^{\text{2}} \end{array}$$ (4)

$$\begin{array}{c} J^{\prime }=\frac{H^{\prime }}{\text{ln}\left(S\right)} \end{array}$$ (5)

$$\begin{array}{c} d=\frac{S-\;\text{1}}{\text{ln}\left(N\right)} \end{array}$$ (6)

where *S* = species richness, *D* = Simpson’s dominance, *H’* = Shannon’s diversity, *P*_*i*_ = the proportion of individuals belonging to species *i*, *D1* = Simpson’s diversity, *J’* = Evenness Index, *d* = Margalef index, and *N* = the total number of individuals in a sample/community.

Although species composition in SFs is strongly influenced by management choices, naturally regenerated species also contribute to overall diversity. Consequently, the observed pattern of tree diversity represent the combined effects of deliberate species selection and natural ecological processes.

Stand density and basal area were calculated for each plot following standard forest inventory protocols. Stand density expressed as the number of trees ha^-1^, while basal area was calculated as the sum of individual tree basal areas (π × DBH^2^/4) ha^-1^. Standing woody biomass of trees, coffee, cacao, and palm (coconut and areca) was estimated using non-destructive methods (allometric equations). Standing woody biomass for different tree species was calculated using Eq. (7) [46], Eq. (8) [47], Eq. (9) [48], and Eq. (10) [49], respectively. Estimated biomass was converted to C stocks using a conversion factor of 0.47 [50], and C stocks were subsequently converted to CO_2_ equivalent (CO_2_-eq) using a factor of 3.67 [51].


$$\begin{array}{c} {AGB}_{tree}=\text{0}.\text{0}\text{5}\text{0}\text{9}\;x\;WD\;x\;{DBH}^{\text{2}}\;x\;H \end{array}$$ (7)

$$\begin{array}{c} {AGB}_{coffee}=\text{0}.\text{2}\text{8}\text{1}\;x\;{DBH}^{\text{2}.\text{0}\text{6}} \end{array}$$ (8)

$$\begin{array}{c} {AGB}_{caccao}=\text{0}.\text{1}\text{2}\text{0}\text{8}\;x\;{DBH}^{\text{1}.\text{9}\text{8}} \end{array}$$ (9)

$$\begin{array}{c} {AGB}_{palm}=\text{4}.\text{5}+\text{7}.\text{7}\;H \end{array}$$ (10)

where *AGB* = tree biomass (kg tree^-1^), *WD* = wood density (g cm^-3^), *DBH* = diameter at breast height (cm), and *H* = tree height (m).

Multiple diversity indices were calculated to capture complementary dimensions of community diversity, including species richness, dominance, evenness, and richness standardized by sample size, providing a comprehensive assessment of tree diversity responses rather than relying on a single metric [39]. Two-way analysis of variance (ANOVA) was performed to assess the effects of cropping pattern and elevation on tree diversity and standing C stocks, followed by Holm-Sidak post hoc comparison test (*emmeans* package [52]. Data were either log or square root transformed to meet normality assumptions. Since the number of samples among different tree crop patterns and elevations was not equal, the Type III sums of squares were applied to account for the unbalanced factorial designs. Variables that satisfied normality assumptions were retained for ANOVA, whereas those that continued to violate normality assumptions were analysed using the non-parametric Scheirer-Ray-Hare test (*rcompanion* package) [53,54]. The Scheirer-Ray-Hare test was applied to investigate the impact of cropping pattern and elevation on Simpson’s diversity index in saplings, poles, and trees, Pielou’s evenness index in poles and trees, Simpson’s dominance index in trees, Shannon’s diversity index in trees, and standing woody biomass, standing C stocks, and CO_2_-eq in different growth phases.

Relationships between standing C stocks (MgC ha^-1^) and stand density, basal area, species richness, the number of individuals, Simpson’s dominance index, Simpson’s diversity index, Shannon’s diversity index, Pielou’s evenness index, and Margalef index were evaluated using linear models to examine first-order associations between standing C stocks and tree diversity or stand structural attributes. This approach was adopted to provide an initial assessment of the direction and strength of these relationships, rather than to develop predictive models or identify potential non-linear responses. Model assumptions were evaluated using standard residual diagnostics to assess normality and homoscedasticity, and no major deviations were observed. Data analysis was conducted in R version 4.4.3 [55], with the level of statistical significance set to p *<* 0.05. Tree diversity indicators were analysed in PAST version 4.03.

## Results

### Forest structure

**Fig 3:**
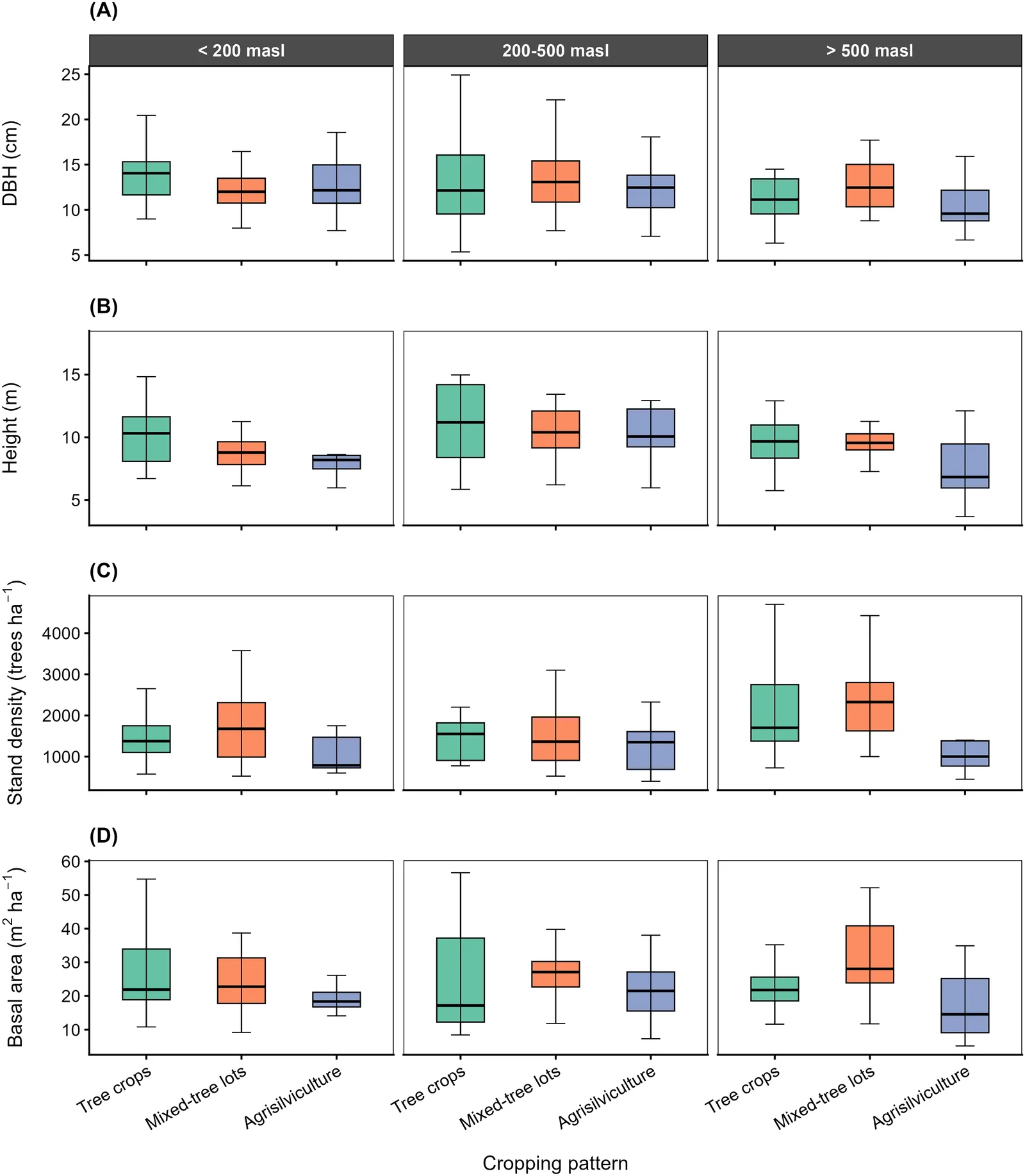
Boxplots of DBH (A), tree height (B), stand density (C), and basal area (D) in different cropping patterns and elevations. The central line represents the median, boxes indicate the interquartile range (IQR), and whiskers extend to 1.5 x IQR.

A total of 7,971 individuals belonging to 108 species were recorded across 150 plots (Data not shown). Mean DBH ranged from 12–14 cm in tree crops, 12–13 cm in mixed-tree lots, and 11–13 cm in agrisilviculture (Fig 3A), with no significant differences among cropping patterns or elevation classes (p > 0.05). Similarly, mean tree height ranged between 10–11 m in tree crops, 9–11 m in mixed tree lots, and 8–11 m in agrisilviculture (Fig 3B), with no significant effects of cropping patterns or elevation classes (p > 0.05). These findings suggest that individual tree size was relatively comparable across cropping patterns and elevation gradients. Consequently, differences in stand structure observed among cropping patterns were more likely associated with stand-level characteristics than with differences in the average size of individual trees.

Stand density ranged between 1,540−2,162 trees ha^-1^ in tree crops, 1,412−2,288 trees ha^-1^ in mixed-tree lots, and 1,065−1,289 trees ha^-1^ in agrisilviculture (Fig 3C). Basal area ranged between 23−28 m^2^ ha^-1^ in tree crops, 24−30 m^2^ ha^-1^ in mixed-tree lots, and 17−21 m^2^ ha^-1^ in agrisilviculture (Fig 3D). Both stand density and basal area showed apparent variation among cropping patterns, whereas variation across elevation classes were less pronounced. These findings confirm that differences among cropping patterns were reflected more strongly in stand density and basal area than in the average dimensions of individual trees.

### Tree diversity in different cropping patterns and growth phases

Species richness and diversity indices varied across growth phases and cropping patterns. Across growth phases, trees had the lowest species richness (2–3 species), while saplings showed the highest species richness (4–6 species) followed by poles (3–5 species). Across cropping patterns, mixed-tree lots and agrisilviculture consistently had higher species richness (3–6 species) than tree crops (2–4 species).

Similar patterns were observed for diversity indices. Shannon’s diversity and Simpson’s diversity indices were higher in agrisilviculture and mixed-tree lots than in tree crops across growth phases. In contrast, Simpson’s dominance index showed the opposite pattern, with higher values in tree crops. Within each cropping pattern, diversity indices tended to be lower in trees than in poles and saplings (Table 2).

**Table 2:** Species richness (*S*), Simpson’s dominance index (*D*), Shannon’s diversity (*H’*), Simpson’s diversity (*D1*), Pielou’s evenness (*J’*), and Margalef index (*d*) in different cropping patterns and growth phases.

Cropping pattern	Growth phase	*S*	*D*	*H’*	*D1*	*J’*	*d*
Tree crops	Saplings	4 ± 0.4Ac	0.5 ± 0.04Ba	0.9 ± 0.09Ab	0.5 ± 0.04Ab	0.7 ± 0.03Aa	1.1 ± 0.12Ab
	Poles	3 ± 0.3Ab	0.7 ± 0.04Cab	0.4 ± 0.09Aa	0.3 ± 0.04Aab	0.7 ± 0.03Aa	0.6 ± 0.10Aa
	Trees	2 ± 0.2Aa	0.6 ± 0.04Bb	0.6 ± 0.08Aa	0.4 ± 0.04Aa	0.9 ± 0.02Ab	0.6 ± 0.11Aa
Mixed-tree lots	Saplings	6 ± 0.4Bc	0.3 ± 0.03Aa	1.4 ± 0.07Bb	0.7 ± 0.03Bb	0.7 ± 0.02Ba	1.7 ± 0.11Bb
	Poles	4.5 ± 0.2Bb	0.4 ± 0.03Bab	1.1 ± 0.06Ba	0.6 ± 0.03Bab	0.7 ± 0.02Ab	1.3 ± 0.08Ba
	Trees	3 ± 0.2Ba	0.4 ± 0.03Ab	1.0 ± 0.07Ba	0.6 ± 0.03Ba	0.9 ± 0.01Ac	1.2 ± 0.09Ba
Agrisilviculture	Saplings	6 ± 0.5Bc	0.3 ± 0.03Aa	1.4 ± 0.08Bb	0.7 ± 0.03Bb	0.7 ± 0.03ABa	1.8 ± 0.13Cb
	Poles	5 ± 0.3Cb	0.3 ± 0.03Aab	1.3 ± 0.07Cb	0.7 ± 0.03 Cab	0.8 ± 0.03Ab	1.6 ± 0.10Cb
	Trees	3 ± 0.3Ba	0.4 ± 0.04Ab	1.0 ± 0.08Ba	0.6 ± 0.04Ba	0.9 ± 0.02Ac	1.3 ± 0.11Ba
Cropping pattern effect		[44.40] < 0.001	[44.87] < 0.001	[43.14] < 0.001	[44.87] < 0.001	ns	[54.78] < 0.001
Growth phase effect		[62.74] < 0.01	[13.08] < 0.01	[26.64] < 0.001	[13.08] < 0.01	[72.86] < 0.001	[22.85] < 0.001

Values are median ± SE (Dunn test with Bonferroni corrections). Different capital letters indicate significant differences among cropping patterns within the same growth phase, and different lowercase letters indicate significant differences among growth phases within the same cropping pattern at p < 0.05. The bottom lines are the results of the Scheirer-Ray-Hare test. The H value (Scheirer-RayHare test) is given in brackets, followed by the p value. ns: non-significant.

Pielou’s evenness index varied significantly among growth phases but not among cropping patterns. In contrast, the Margalef index was significantly varied between cropping patterns and growth phases, with higher values in agrisilviculture (1.3–1.8) and mixed-tree lots (1.2–1.7) than in tree crops (0.6–1.1). Across growth phases, Margalef index values were higher in saplings (1.1–1.8) than in poles (0.6–1.6) and trees (0.6–1.3) (Table 2).

### Tree diversity in different cropping patterns and elevations

#### Saplings.

Cropping pattern had strong and consistent effects on most diversity indices, whereas elevation effects were significant but comparatively weaker (Table 3). Species richness in tree crops (3.3–4.8) was significantly lower than that in mixed-tree lots (5.0–6.8) and agrisilviculture (5.3–7.2). Across cropping patterns, species richness at > 500 masl (3.3–5.3) was slightly lower than that at < 200 masl (4.1–6.3) and significantly lower than that at 200–500 masl (4.8–7.2).

**Table 3:** Species richness (*S*), Simpson’s dominance index (*D*), Shannon’s diversity (*H’*), Simpson’s diversity (*D1*), Pielou’s evenness (*J’*), and Margalef index (*d*) of saplings in different cropping patterns and elevations.

Cropping pattern	Elevation (masl)	*S* ^a^	*D* ^a^	*H’* ^a^	*D1* ^b^	*J’* ^a^	*d* ^a^
Tree crops	< 200	4.1 ± 0.5Aab	0.5 ± 0.04Bab	1.0 ± 0.1Aab	0.5 ± 0.07Aa	0.8 ± 0.03	1.2 ± 0.15Aab
	200-500	4.8 ± 0.5Ab	0.4 ± 0.04Ba	1.1 ± 0.1Ab	0.6 ± 0.08Aa	0.7 ± 0.03	1.4 ± 0.16Ab
	> 500	3.3 ± 0.4Aa	0.6 ± 0.05Bb	0.7 ± 0.1Aa	0.4 ± 0.06Aa	0.7 ± 0.03	0.8 ± 0.15Aa
Mixed-tree lots	< 200	6.0 ± 0.5Bab	0.3 ± 0.03Aab	1.3 ± 0.1Bab	0.7 ± 0.03Bb	0.7 ± 0.03	1.7 ± 0.14Bab
	200-500	6.8 ± 0.6Bb	0.3 ± 0.03Aa	1.4 ± 0.1Bb	0.7 ± 0.05ABb	0.7 ± 0.03	1.9 ± 0.14Bb
	> 500	5.0 ± 0.5Ba	0.4 ± 0.04Ab	1.1 ± 0.1Ba	0.6 ± 0.06Aa	0.7 ± 0.03	1.4 ± 0.15Ba
Agrisilviculture	< 200	6.3 ± 0.7Bab	0.3 ± 0.03Aab	1.4 ± 0.1Bab	0.7 ± 0.08Bb	0.7 ± 0.03	1.8 ± 0.17Bab
	200-500	7.2 ± 0.7Bb	0.3 ± 0.03Aa	1.5 ± 0.1Bb	0.7 ± 0.03Bb	0.7 ± 0.03	2.0 ± 0.16Bb
	> 500	5.3 ± 0.6Ba	0.4 ± 0.04Ab	1.2 ± 0.1Ba	0.6 ± 0.06Aa	0.7 ± 0.03	1.5 ± 0.16Ba
Cropping pattern effect		[8.15] < 0.001	[7.12] < 0.01	[8.95] < 0.001	[12.82] < 0.01	ns	[7.99] < 0.001
Elevation effect		[4.24] <0.05	[4.84] < 0.01	[4.92] < 0.01	[8.59] < 0.05	ns	[5.25] < 0.01

^a^Values are estimated means ± SE. ^b^Values are median ± SE (Dunn test with Bonferroni corrections). Different capital letters indicate significant differences among cropping patterns in each elevation, and different lowercase letters indicate significant differences among elevations in each cropping pattern at p < 0.05. The bottom lines are the results of two-way ANOVA or Scheirer-Ray-Hare test. The F value (ANOVA) or H value (Scheirer-RayHare test) is given in brackets, followed by the p value. ns: non-significant.

Simpson’s dominance index was significantly higher in tree crops (0.4–0.6) than in mixed-tree lots (0.3–0.4) and agrisilviculture (0.3–0.4) across all elevation classes. Across cropping patterns, Simpson’s dominance index at > 500 masl (0.4–0.6) was significantly higher than at 200−500 masl (0.3–0.4) and slightly higher than that at < 200 masl (0.3–0.5) (Table 3).

Shannon’s diversity index was significantly lower in tree crops (0.7–1.1) than in mixed-tree lots (1.1–1.4) and agrisilviculture (1.2–1.5) across all elevation classes. Across cropping patterns, Shannon’s diversity index was highest at 200–500 masl (1.1–1.5) and lowest at > 500 masl (0.7–1.2) (Table 3).

Simpson’s diversity index was higher in mixed tree lots and agrisilviculture than in tree crops at < 200 and 200–500 masl, although these differences were not significant > 500 masl. Within mixed-tree lots and agrisilviculture, Simpson’s diversity index was significantly lower at elevation > 500 masl than at lower elevations. In contrast, Simpson’s diversity index showed little variation across elevation classes in tree crop systems (Table 3).

Pielou’s evenness index (0.7 and 0.8) did not differ significantly among cropping patterns and elevation classes. In contrast, the Margalef index was significantly lower in tree crops (0.8–1.4) than in mixed-tree lots (1.4–1.9) and agrisilviculture (1.5–2.0) across elevation classes. Within each cropping pattern, the Margalef index was slightly higher at 200–500 masl (1.4–2.0) than at < 200 masl (1.2–1.8) and was significantly higher than that at > 500 masl (0.8–1.5) (Table 3).

#### Poles.

Diversity indices for poles were generally not influenced by elevation, whereas cropping patterns significantly influenced most diversity indices (Table 4). The only exception was Pielou’s evenness index, which did not differ among cropping patterns or elevation classes. Species richness, Shannon’s diversity, Simpson’s diversity, and Margalef indices were significantly higher in agrisilviculture and mixed-tree lots than in tree crops. In contrast, Simpson’s dominance index was significantly higher in tree crops than in mixed-tree lots and agrisilviculture.

**Table 4:** Species richness (*S*), Simpson’s dominance index (*D*), Shannon’s diversity (*H’*), Simpson’s diversity (*D1*), Pielou’s evenness (*J’*), and Margalef index (*d*) of poles in different cropping patterns and elevations.

Cropping pattern	Elevation (masl)	*S* ^a^	*D* ^a^	*H’* ^a^	*D1* ^b^	*J’* ^b^	*d* ^a^
Tree crops	< 200	3.0 ± 0.3A	0.6 ± 0.05B	0.7 ± 0.1A	0.2 ± 0.07A	0.7 ± 0.05	0.8 ± 0.1A
	200-500	3.0 ± 0.3A	0.6 ± 0.05B	0.7 ± 0.1A	0.6 ± 0.09A	0.9 ± 0.06	0.9 ± 0.1A
	> 500	2.7 ± 0.3A	0.7 ± 0.05B	0.6 ± 0.1A	0.2 ± 0.06A	0.7 ± 0.04	0.6 ± 0.1A
Mixed-tree lots	< 200	4.5 ± 0.3B	0.4 ± 0.03A	1.1 ± 0.1B	0.7 ± 0.03B	0.8 ± 0.02	1.3 ± 0.1B
	200-500	4.4 ± 0.3B	0.4 ± 0.04A	1.1 ± 0.1B	0.6 ± 0.05A	0.8 ± 0.04	1.4 ± 0.1B
	> 500	4.1 ± 0.4B	0.5 ± 0.04A	1.0 ± 0.1B	0.6 ± 0.05B	0.7 ± 0.03	1.1 ± 0.1B
Agrisilviculture	< 200	4.9 ± 0.4B	0.3 ± 0.04A	1.3 ± 0.1B	0.7 ± 0.05B	0.8 ± 0.04	1.6 ± 0.1B
	200-500	4.9 ± 0.4B	0.4 ± 0.04A	1.3 ± 0.1B	0.6 ± 0.03A	0.7 ± 0.05	1.6 ± 0.1B
	> 500	4.5 ± 0.4B	0.4 ± 0.04A	1.1 ± 0.1B	0.6 ± 0.06B	0.8 ± 0.04	1.3 ± 0.1B
Cropping pattern effect		[12.45] < 0.001	[13.13] < 0.001	[13.97] < 0.001	[18.52] < 0.001	ns	[15.14] < 0.001
Elevation effect		ns	ns	ns	ns	ns	ns

^a^Values are estimated means ± SE. ^b^Values are median ± SE (Dunn test with Bonferroni corrections). Different capital letters indicate significant differences among cropping patterns in each elevation, and different lowercase letters indicate significant differences among elevations in each cropping pattern at p < 0.05. The bottom lines are the results of two-way ANOVA or Scheirer-Ray-Hare test. The F value (ANOVA) or H value (Scheirer-RayHare test) is given in brackets, followed by the p value. ns: non-significant.

#### Trees.

Species richness in tree crops (1.9–2.8) was consistently lower than in mixed-tree lots (2.6–3.8) and agrisilviculture (2.8–3.9) across all elevation classes. Across cropping patterns, species richness was lowest at > 500 masl (1.9–2.8) and highest at 200–500 masl (2.8–3.9). Simpson’s dominance index tended to be lower at 200–500 masl (0.3–0.4) than at < 200 masl (0.4–0.6) and > 500 masl (0.5–0.7) across cropping patterns. At < 200 masl, Simpson’s dominance index was higher in tree crops than in mixed-tree lots and agrisilviculture, whereas no differences among cropping patterns were observed at 200–500 and > 500 masl (Table 5).

**Table 5:** Species richness (*S*), Simpson’s dominance index (*D*), Shannon’s diversity (*H’*), Simpson’s diversity (*D1*), Pielou’s evenness (*J’*), and Margalef index (*d*) of trees in different cropping patterns and elevations.

Cropping pattern	Elevation (masl)	*S* ^a^	*D* ^b^	*H’* ^b^	*D1* ^b^	*J’* ^b^	*d* ^a^
Tree crops	< 200	2.3 ± 0.3Aab	0.6 ± 0.06Ba	0.6 ± 0.1Aa	0.4 ± 0.06Aa	0.9 ± 0.03	0.7 ± 0.13Aab
	200-500	2.8 ± 0.3Ab	0.4 ± 0.12Aa	1.1 ± 0.2Aa	0.6 ± 0.12Aa	0.9 ± 0.04	1.0 ± 0.1Ab
	> 500	1.9 ± 0.2Aa	0.6 ± 0.07Aa	0.6 ± 0.1Aa	0.4 ± 0.07Aa	1.0 ± 0.03	0.5 ± 0.1Aa
Mixed-tree lots	< 200	3.2 ± 0.3Bab	0.4 ± 0.06Aa	1.0 ± 0.1Ba	0.6 ± 0.06Ba	0.9 ± 0.02	1.1 ± 0.1Bab
	200-500	3.8 ± 0.3Bb	0.4 ± 0.05Aa	1.0 ± 0.1Aa	0.6 ± 0.05Aa	0.9 ± 0.02	1.4 ± 0.1Bb
	> 500	2.6 ± 0.3Ba	0.5 ± 0.08Aa	0.9 ± 0.1Aa	0.5 ± 0.08Aa	0.9 ± 0.03	0.9 ± 0.1Ba
Agrisilviculture	< 200	3.3 ± 0.3Bab	0.5 ± 0.04Aab	0.9 ± 0.1Bab	0.5 ± 0.04Bab	0.8 ± 0.04	1.1 ± 0.1Bab
	200-500	3.9 ± 0.4Bb	0.3 ± 0.03Aa	1.2 ± 0.1Ab	0.7 ± 0.03Ab	0.9 ± 0.03	1.5 ± 0.1Bb
	> 500	2.8 ± 0.3Ba	0.7 ± 0.08Ab	0.6 ± 0.2Aa	0.3 ± 0.08Aa	0.8 ± 0.05	1.0 ± 0.1Ba
Cropping pattern effect		[5.49] < 0.01	[10.25] < 0.01	[9.61] < 0.01	[10.25] < 0.01	ns	[5.63] < 0.01
Elevation effect		[5.43] < 0.01	[9.82]< 0.01	[10.43] < 0.01	[9.82] < 0.01	ns	[5.89] < 0.01

^a^Values are estimated means ± SE. ^b^Values are median ± SE (Dunn test with Bonferroni corrections). Different capital letters indicate significant differences among cropping patterns in each elevation, and different lowercase letters indicate significant differences among elevations in each cropping pattern at p < 0.05. The bottom lines are the results of two-way ANOVA or Scheirer-Ray-Hare test. The F value (ANOVA) or H value (Scheirer-RayHare test) is given in brackets, followed by the p value. ns: non-significant.

Shannon’s diversity and Simpson’s diversity indices followed similar patterns, with higher values in mixed-tree lots and agrisilviculture than in tree crops, particularly at < 200 masl. Across cropping patterns, both indices tended to be higher at 200–500 masl and lower at > 500 masl, with the clearest elevation-related trend observed in agrisilviculture.

Pielou’s evenness index did not differ significantly among cropping patterns and elevation classes. In contrast, the Margalef index varied significantly with both cropping patterns and elevations. Across all elevation classes, the Margalef index was significantly lower in tree crops (0.5–1.0) than in mixed-tree lots (0.9–1.4) and agrisilviculture (1.0–1.5). Within each cropping pattern, the Margalef index was highest at 200–500 masl and lower at both < 200 masl and > 500 masl (Table 5).

Collectively, the diversity indices showed a consistent pattern of higher species richness and diversity in mixed-tree lots and agrisilviculture than in tree crops, accompanied by lower species dominance. Although the magnitude of differences varied among indices and growth phases, the overall pattern indicated greater diversity and lower dominance in mixed-species cropping systems compared with tree crops.

### Standing woody biomass, C stocks, and CO_2_-eq in different cropping patterns and elevations

**Fig 4:**
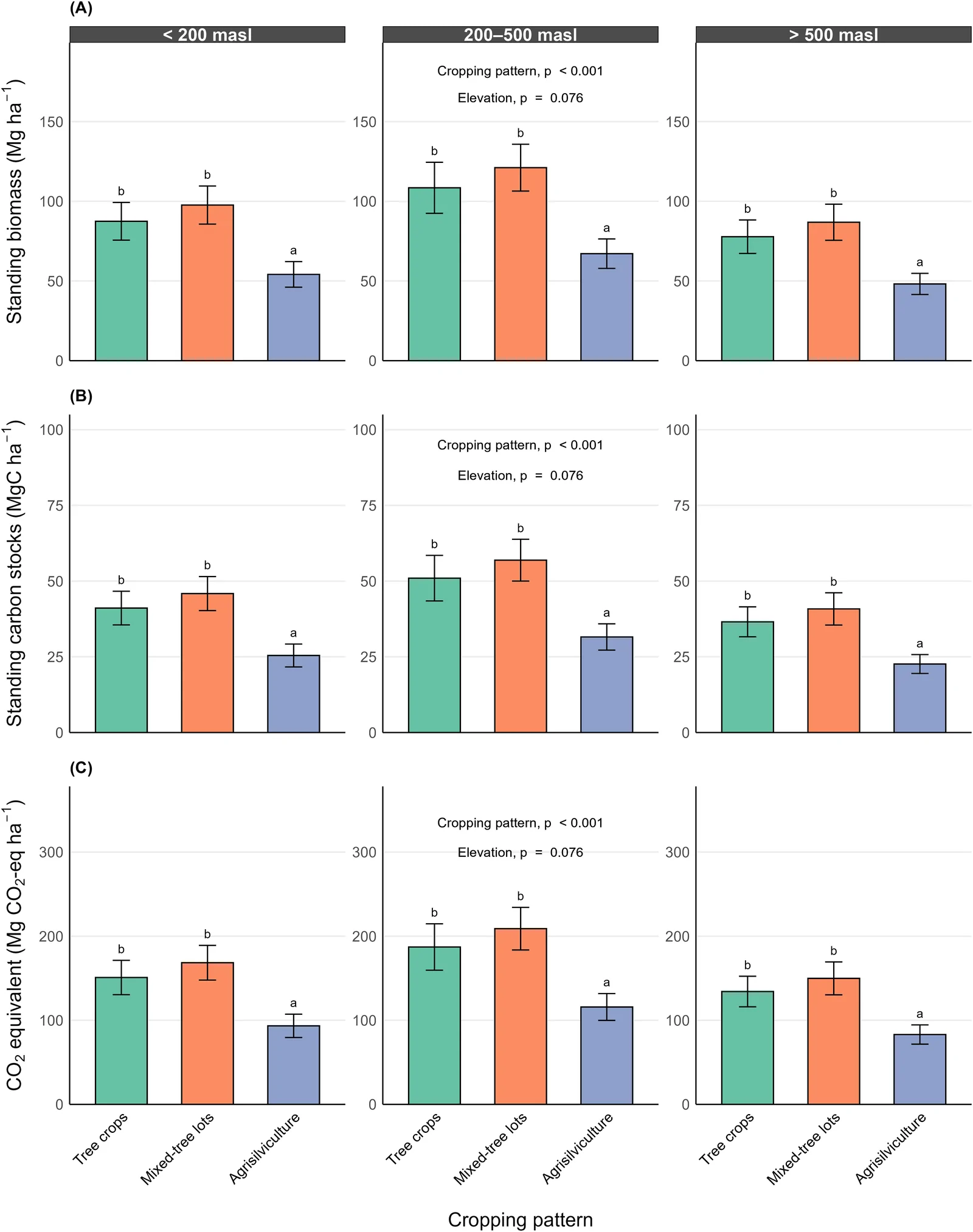
Standing woody biomass (A), standing C stocks (B), and CO_2_ equivalent (C) in different cropping patterns and elevation. Values represent mean ± SE. Different letters indicate significant differences among cropping patterns in each elevation.

Cropping pattern significantly influenced standing woody biomass, but elevation did not have a significant effect (Fig 4A). Standing woody biomass in tree crops (78−108 Mg ha^-1^) and mixed-tree lots (87−121 Mg ha^-1^) was 61−62% and 80−81% higher, respectively, than that in agrisilviculture (48−67 Mg ha^-1^) across elevation classes. However, substantial variation among plots was observed within each cropping pattern.

A similar pattern was observed for standing C stocks (Fig 4B). Standing C stocks in tree crops (37−51 MgC ha^-1^) and mixed-tree lots (41−57 MgC ha^-1^) were consistently higher than those in agrisilviculture (23−32 MgC ha^-1^), although variability among plots was observed within cropping patterns. CO_2_-eq also varied significantly between tree crops but did not vary with elevations. CO_2_-eq showed a similar pattern, with significant differences among cropping patterns but no significant effect of elevation (Fig 4C). Carbon storage expressed as CO_2_-eq in tree crops (134−187 Mg CO_2_-eq ha^-1^) and mixed-tree lots (150−209 Mg CO_2_-eq ha^-1^) was significantly higher than that in agrisilviculture (83−116 Mg CO_2_-eq ha^-1^) across elevation classes.

### Standing woody biomass, C stocks, and CO_2_-eq in different cropping patterns and growth phases

**Fig 5:**
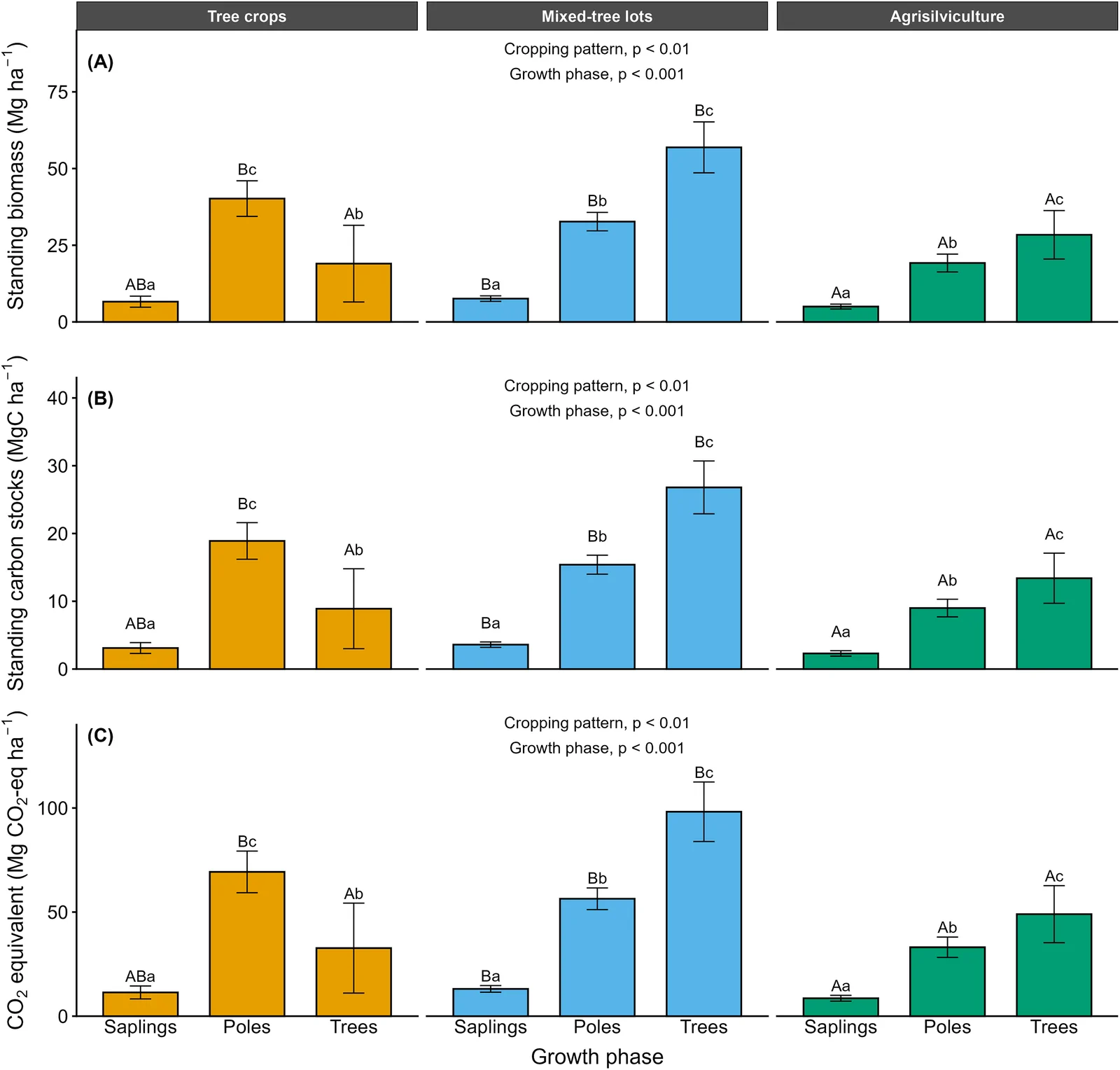
Standing woody biomass (A), standing C stocks (B), and CO_2_ equivalent (C) in different cropping patterns and growth phases. Values represent median ± SE. Different letters indicate significant differences among cropping patterns in each elevation.

Cropping pattern and growth phase significantly influenced standing woody biomass, C stocks, and CO_2_-eq. Across cropping patterns, saplings had significantly lower standing woody biomass, C stocks, and CO_2_-eq than poles and trees. In mixed-tree lots, trees showed the highest values, with standing woody biomass, C stocks, and CO_2_-eq being more than 600% higher than those in saplings and 74% higher than those in poles. Similarly, in agrisilviculture, trees showed more than 450% higher values than saplings and 48% higher values than poles. In tree crops, standing woody biomass, C stocks, and CO_2_-eq were highest in poles, which were more than five times higher than saplings and more than 1.7 times higher than trees (Fig 5).

### Relationships between standing C stocks and stand density, basal area, and tree diversity indices

**Fig 6:**
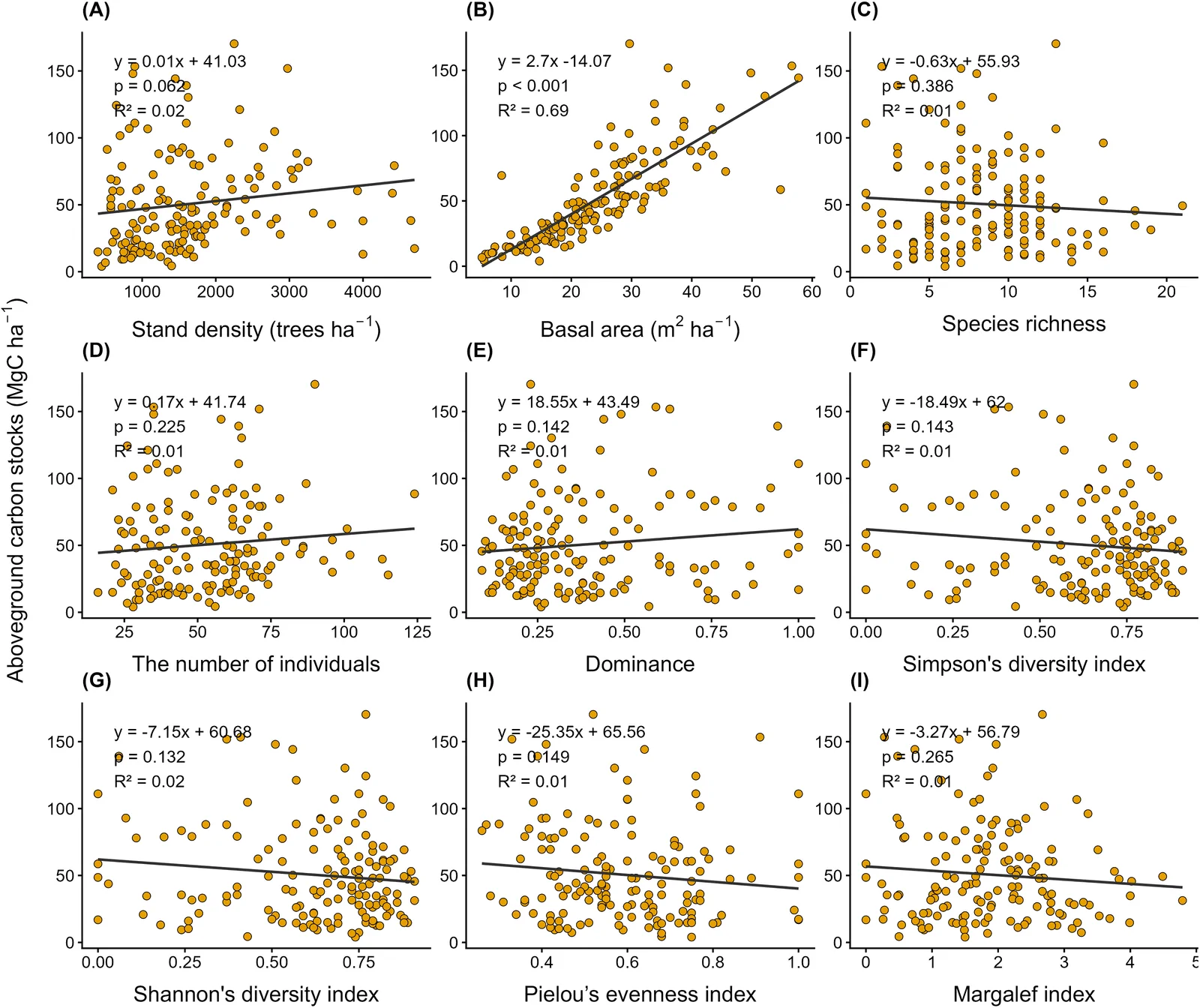
The relationship between standing C stocks and stand density (A), basal area (B), species rich ness (C), the number of individuals (D), Simpson’s dominance index (E), Simpson’s diversity index (F), Shannon’s diversity index (G), Pielou’s evenness index (H), and Margalef index (I). Lines represent fitted linear models.

Standing C stocks were not correlated with stand density, species richness, the number of individuals, Simpson’s dominance, Simpson’s diversity, Shannon’s diversity, Pielou’s evenness, and Margalef indices (Fig 6). In contrast, standing C stocks showed a strong positive relationship with basal area, which explained 69% of the variation in standing C stocks (Fig 6B). These results indicate that basal area was more closely associated with standing C stock than tree diversity indices evaluated in this study.

## Discussions

### Tree diversity across cropping patterns, growth phases, and elevations

The total number of tree species recorded in this study (108 species) exceeds those recorded for traditional agroforestry in Gunung Halimun Salak National Park, West Java (32 species), forest garden in Kampung Birang (26 species) and Kampung Merabu (88 species) in East Kalimantan [56], teak-based agroforestry system in Central Java (12 species) [18], *pekarangans* (traditional homestead garden) in Cianjur Watershed (26–44 species) [57]. The number of tree species found in this study is comparable to that reported for home gardens in Sri Lanka (107 woody species) [58] and a West African cacao landscape (109 species) [59]. These comparisons suggest that SFs in the present study support relatively high levels of woody plant diversity.

Species richness in agroforestry systems is generally categorized as low to moderate, and often approximating that of secondary forests [18]. Our results showed that species richness and diversity indices varied markedly among cropping patterns, highlighting the strong influence of management practices in tree diversity outcomes. The tree crops pattern exhibited the lowest species richness in different growth phases, reflecting its limited species composition (one to three tree species). In contrast, mixed-tree lots and agrisilviculture patterns support more diverse species assemblages through the integration of fruit, timber, and shade-tolerant species [5]. Shannon’s diversity index in different growth phases was low in tree crops pattern (*H’* < 1), while mixed-tree lots and agrisilviculture patterns showed moderate diversity (1 < *H’* < 3). The same pattern was observed across elevation classes, with tree-crop systems consistently showing the lowest diversity. However, Shannon’s diversity index in this study is lower than those reported for *Cabruca* agroforest in Bahia (2.21–3.52) [60] and forest garden in Saparua, Maluku (2.73–3.12) [61], possibly due to the higher structural complexity and longer management continuity in those systems. Similar trends were observed for Simpson’s and Margalef’s indices, both of which were consistently higher in mixed-tree lots and agrisilviculture patterns, suggesting greater stand-level tree diversity in these cropping patterns.

Higher diversity in mixed-tree lots and agrisilviculture likely reflects the integration of multiple tree species maintained for different functions. Diverse tree assemblages are known to confer greater ecosystem stability by buffering against climatic and biological disturbances through functional complementarity and trait diversity [62,63]. Similarly, higher tree diversity may enhance resistance to drought, pests, and diseases, providing a foundation for more resilient smallholder forestry systems [5,64]. However, the present study did not directly test these mechanisms. In contrast, the lower species richness observed in tree-crop pattern leads to higher Simpson’s dominance indices [39], indicating greater dominance by a few species. By comparison, the lower dominance indices in mixed-tree lots and agrisilviculture reflect a more balanced species composition and reduced dependence on a narrow set of species. Although this study assessed taxonomic rather than functional diversity, greater taxonomic diversity may also reflect broader variation in species functional traits, which influence resource-use strategies, productivity, and ecosystem functioning. Recent evidence indicates that changes in functional traits, rather than species richness alone, can explain variation in ecosystem productivity across environmental gradients, highlighting the importance of trait-based mechanisms in forest ecosystem functioning [65]. However, because functional traits were not measured in the present study, these mechanisms could not be evaluated directly and therefore warrant further investigation.

The absence of significant difference in Pielou’s evenness index among cropping patterns suggests that, despite variation in species richness, the relative distribution of individuals among tree species remained broadly uniform [66]. Thus, differences in diversity among cropping patterns were primarily driven by species richness rather than species evenness.

A tendency toward higher species richness at mid-elevation (200–500 masl) was observed across cropping patterns and growth phases, though this effect was generally modest and not statistically significant. This pattern contrast with findings from Ethiopian home gardens, where species richness declined with increasing elevation [67]. The pattern observed in the present study is consistent with the commonly reported “mid-elevation peak” in tree diversity across taxa and ecosystems [68,69], which has been attributed to favourable microclimatic conditions or greater landscape heterogeneity. However, the observed differences may also partly reflect variation in the distribution of cropping patterns across elevation classes or differences in local management history rather than a strong ecological effect of elevation itself. Collectively, these results suggest that while elevation may exert a secondary influence on species richness, cropping pattern remains the more dominant factor shaping tree diversity within SFs.

### Standing C stocks

Standing C stocks in SFs in Indonesia range between 30 and 123 MgC ha^-1^, comparable to those of secondary forests of similar age [15,70]. Although generally lower than those in forest plantations and natural forests, standing C stocks in agroforestry system (including SFs) are higher than those in croplands, grasslands, and pastures [71,72]. The standing woody biomass and C stocks observed across cropping patterns in this study are higher than those reported for community forests of Gunung Kidul (38 Mg ha^-1^ and 19 MgC ha^-1^) [73], and comparable to those reported for Lampung (34.7 MgC ha^-1^) [70], cacao monoculture in West Sulawesi (47.7 MgC ha^-1^) [74], and *Manglieta glauca*-based agroforestry in West Java (44 MgC ha^-1^) [75]. However, these estimates were lower than those reported for cacao agroforests in West Sulawesi (60.7 MgC ha^-1^) [74] and teak-based agroforestry in Central Java (64.4 MgC ha^-1^) [18]. Differences among systems likely reflect variation in stand age, species composition, and structural development, which strongly influence biomass accumulation in agroforestry landscapes.

Our results showed that cropping pattern strongly influenced biomass and C storage, consistent with Budiadi & Ishii [76]. Tree crops and mixed-tree lots stored more C than agrisilviculture, which may be related to wider spacing commonly used in agrisilviculture systems to accommodate understory crops, thereby reducing basal area and fewer large-diameter trees. As large-diameter trees contribute disproportionately to stand biomass and C storage, stand structure likely plays a greater role in determining C stocks than the number of individuals or tree diversity alone.

However, elevation did not significantly affect C sequestration in SF, consistent with Lowe et al. [58] and Mensah et al. [25], suggesting that cropping pattern and structural attributes are more influential than topographic position within the elevation range examined. Our results also showed that standing C stocks are positively correlated with stand basal area, which is in line with Hartoyo et al. [56], but weakly correlated with stand density and species richness.

Although Reich et al. [77] and Mensah et al. [25] observed positive correlations between standing C stocks and species richness, our results indicate that stand structural attributes, such as DBH, height, and wood density play a more important role in determining standing C stocks than tree diversity indices [44,55,68–70]. This pattern may arise because a relatively small number of large trees contributes disproportionately to stand biomass in SFs, meaning that higher tree diversity does not necessarily translate into higher C storage unless accompanied by greater structural development. Therefore, under the conditions examined in this study, tree diversity was not a strong predictor of standing C stocks. However, this does not imply that tree diversity is unimportant for C sequestration, as it may contribute indirectly through long-term stand development, species interactions, and functional complementarity. Greater tree diversity may also enhance ecosystem resilience and stability [34,78,79], thereby contributing to the long-term maintenance of C stocks

As this study is mainly focused on standing trees, total ecosystem biomass and C stocks may have been underestimated across cropping patterns and elevations. Future studies should quantify biomass additional C pools, including soil, litter, deadwood, and non-woody species (understorey). Future research should also investigate the effects of management intensity, soil type, topography, and plantation age to better understand the factors influencing tree diversity and C storage in SFs.

### Management-filtered tree diversity and standing C stocks in SFs

Community assembly theory proposes that species composition is determined by a series of ecological filters operating across spatial and temporal scales, including dispersal limitation, environmental conditions, and biotic interactions [80,81]. In managed ecosystems such as SFs, these ecological filters are complemented by anthropogenic filters arising from farmers’ decisions regarding species selection, harvesting, enrichment planting, and the retention of naturally regenerated trees [78]. Consequently, tree diversity patterns and standing C stocks in the SFs of Ciamis Regency reflect not only ecological processes but also farmers’ management decisions. Planting choices, selective retention of naturally regenerated trees, and harvesting practices together act as management filters that shape species composition alongside natural ecological assembly [21,82].

Higher species richness in mixed-tree lots does not always result from deliberate diversification strategies. In many cases, this pattern emerges from low management intensity, especially in distant or infrequently visited sites. Under such conditions, farmers tend to harvest selectively or intermittently, retaining both planted and naturally regenerated species and producing structurally and taxonomically heterogeneous stands. This supports earlier findings that high diversity in traditional systems often reflects low-intensity management rather than carefully designed planting schemes [83].

Moreover, cropping patterns in SFs are dynamic and may shift from tree crops to mix-tree lots through selective harvesting and enrichment planting, shifting species composition and biomass accumulation over time [5]. In line with the concept of ‘domestic forests’ [84], tree diversity and C stocks should therefore be viewed as management-associated outcomes that integrate both deliberate and incidental diversity while supporting landscape heterogeneity and ecosystem services.

### Management implications and future research direction

Our results clearly demonstrate that mixed-tree lots provide a favourable balance between tree diversity and standing C stocks, highlighting their potential as a nature-based solution for multifunctional SF management. Improving the ecological performances of other cropping patterns may involve diversifying tree crops patterns, increasing tree density in agrisilviculture where appropriate, and selecting tree species with high biomass potential without compromising agricultural productivity.

As cropping patterns in SFs are ultimately shaped by farmers’ decision-making, understanding the socio-economic drivers of management choices is essential. Further studies should quantify the trade-offs among tree diversity, stand C storage, ecosystem services, and farmers’ income while also evaluating the effects of species selection, stand density, rotation regimes, and management intensity. Public policy interventions, such as incentive schemes, subsidies for tree planting, and ecosystem service valuation, could accelerate the wider adoption of agroforestry practices [85,86]. These efforts would benefit from an interdisciplinary approach integrating ecological, silvicultural, and socio-economic perspectives to ensure that agroforestry practices are ecologically sound, socially acceptable, and economically viable for smallholder farmers. Cross-sectoral coordination among forestry, agriculture, and rural development agencies will be essential to streamline policy implementation and avoid institutional fragmentation [87,88].

## Conclusions

This study demonstrates that cropping patterns exerted a stronger influence than elevation on tree diversity and standing C stocks in SFs of Ciamis Regency, West Java. Among the evaluated cropping patterns, mixed-tree lots consistently supported higher tree diversity and greater standing C stocks than tree crops and agrisilviculture, highlighting their potential to enhance both biodiversity conservation and climate mitigation under smallholder management. In contrast, the relatively high standing C stocks in tree-crop systems were more closely associated with stand structural attributes, particularly basal area, than with tree diversity.

Standing C stocks were strongly associated with basal area but not with tree diversity indices, highlighting that stand structure was a better predictor of aboveground C storage than tree diversity alone. Although elevation had little influence on standing C stocks, it showed modest effects on tree diversity, with species richness generally peaking at mid-elevations. Together, these findings suggest that management practices shaping stand structure and species composition play a greater role than elevation in determining tree diversity and C storage in SFs.

Future research should evaluate the socio-economic trade-offs among cropping pattern, including farmers’ livelihood, management objectives, and long-term sustainability. Quantifying additional ecosystem C pools and assessing the effects of management intensity across broader environmental gradients will further improve understanding of the climate mitigation potential of SFs and support the development of more sustainable and multifunctional management strategies.

## Supporting information

S1 Table Dataset of tree inventory and aboveground biomass carbon estimates in Ciamis Regency. This dataset contains the individual tree-level inventory data and derived estimates of aboveground biomass, carbon stock, and CO_2_ equivalent used in the analyses presented in this study.
(PDF)
