# Supplementary material for: Tree diversity and standing carbon stocks in smallholder forests of Ciamis Regency, Indonesia: Effects of cropping patterns and elevations
Source: PLoS One. 2026 Sep 21;21(9):e0356872. doi: 10.1371/journal.pone.0356872 (PMC13592580; doi:10.1371/journal.pone.0356872)
Supplement: Supplementary file 1 [file pone.0356872.s001.pdf]

S1 Table. Dataset of tree inventory and aboveground biomass carbon estimates in Ciamis Regency

No	Plot	Pattern goup	Sub-District	Elevation	Local name	Latin name	Family	WD	Diameter (cm)	Tbc	Ttot	Luas plot (ha)	Basal Area (cm2)	AGB_kg/tree	AGB_Mg/tree	AGC (MgC/ha)	MgCO2e/ha
1	1	3	Banjaranyar	<200	Afrika	Maesopsis eminii Engl.	Rhamnaceae	0,43	15,6	11	13	0,04	0,02	83,02	0,08	0,98	3,58
2	1	3	Banjaranyar	<200	Sengon	Paraserianthes falcataria (L.) I.C	Leguminosae / Fabaceae	0,33	14,6	9	13	0,04	0,02	58,03	0,06	0,68	2,50
3	1	3	Banjaranyar	<200	Afrika	Maesopsis eminii Engl.	Rhamnaceae	0,43	9,9	8	10	0,04	0,01	28,22	0,03	0,33	1,22
4	1	3	Banjaranyar	<200	Afrika	Maesopsis eminii Engl.	Rhamnaceae	0,43	38,2	18	21	0,04	0,11	664,64	0,66	7,81	28,66
5	1	3	Banjaranyar	<200	Afrika	Maesopsis eminii Engl.	Rhamnaceae	0,43	13,0	11	12	0,04	0,01	55,66	0,06	0,65	2,40
6	1	3	Banjaranyar	<200	Jabon	Neolamarckia cadamba (Roxb.) I	Rubiaceae	0,42	32,8	14	16	0,04	0,08	383,27	0,38	4,50	16,53
7	1	3	Banjaranyar	<200	Afrika	Maesopsis eminii Engl.	Rhamnaceae	0,43	13,4	10	11,5	0,04	0,01	55,95	0,06	0,66	2,41
8	1	3	Banjaranyar	<200	Mahoni	Swietenia macrophylla King	Meliaceae	0,61	12,7	4	11	0,04	0,01	67,67	0,07	0,80	2,92
9	1	3	Banjaranyar	<200	Mahoni	Swietenia macrophylla King	Meliaceae	0,61	9,5	3	7	0,04	0,01	26,40	0,03	0,31	1,14
10	1	3	Banjaranyar	<200	Jabon	Neolamarckia cadamba (Roxb.) I	Rubiaceae	0,42	40,1	15	18	0,04	0,13	617,62	0,62	7,26	26,63
11	1	3	Banjaranyar	<200	Mahoni	Swietenia macrophylla King	Meliaceae	0,61	22,6	8	110	0,04	0,04	1.595,52	1,60	18,75	68,80
12	1	3	Banjaranyar	<200	Kisereh	Cinnamomum parthenoxylon (Ja	Lauraceae	0,5	16,5	3	10	0,04	0,02	83,58	0,08	0,98	3,60
13	1	3	Banjaranyar	<200	Cengkeh	Syzygium aromaticum (L.) Merr. &	Myrtaceae	0,79	4,1	1,5	4	0,04	0,00	4,33	0,00	0,05	0,19
14	1	3	Banjaranyar	<200	Mahoni	Swietenia macrophylla King	Meliaceae	0,61	21,3	6	13	0,04	0,04	202,87	0,20	2,38	8,75
15	1	3	Banjaranyar	<200	Mahoni	Swietenia macrophylla King	Meliaceae	0,61	18,1	9	13	0,04	0,03	150,87	0,15	1,77	6,51
16	1	3	Banjaranyar	<200	Manggis	Mangostana garcinia Gaertn.	Clusiaceae	0,81	3,2	0,5	2,5	0,04	0,00	1,78	0,00	0,02	0,08
17	1	3	Banjaranyar	<200	Alpukat	Persea americana Mill.	Lauraceae	0,54	14,0	5	9	0,04	0,02	59,96	0,06	0,70	2,59
18	1	3	Banjaranyar	<200	Mahoni	Swietenia macrophylla King	Meliaceae	0,61	21,0	3,5	11	0,04	0,03	169,36	0,17	1,99	7,30
19	1	3	Banjaranyar	<200	Afrika	Maesopsis eminii Engl.	Rhamnaceae	0,43	13,0	9	10	0,04	0,01	47,10	0,05	0,55	2,03
20	1	3	Banjaranyar	<200	Manggis	Mangostana garcinia Gaertn.	Clusiaceae	0,81	3,2	0,5	3	0,04	0,00	2,11	0,00	0,02	0,09
21	1	3	Banjaranyar	<200	Jabon	Neolamarckia cadamba (Roxb.) I	Rubiaceae	0,42	34,7	11	15	0,04	0,09	400,76	0,40	4,71	17,28
22	1	3	Banjaranyar	<200	Afrika	Maesopsis eminii Engl.	Rhamnaceae	0,43	10,5	9	10,5	0,04	0,01	33,09	0,03	0,39	1,43
23	1	3	Banjaranyar	<200	Afrika	Maesopsis eminii Engl.	Rhamnaceae	0,43	9,9	9	10,5	0,04	0,01	29,51	0,03	0,35	1,27
24	1	3	Banjaranyar	<200	Afrika	Maesopsis eminii Engl.	Rhamnaceae	0,43	12,1	9	10	0,04	0,01	40,98	0,04	0,48	1,77
25	1	3	Banjaranyar	<200	Manggis	Mangostana garcinia Gaertn.	Clusiaceae	0,81	3,8	0,5	3	0,04	0,00	2,94	0,00	0,03	0,13
26	1	3	Banjaranyar	<200	Mahoni	Swietenia macrophylla King	Meliaceae	0,61	27,0	9	12	0,04	0,06	291,55	0,29	3,43	12,57
27	1	3	Banjaranyar	<200	Sengon	Paraserianthes falcataria (L.) I.C	Leguminosae / Fabaceae	0,33	13,0	9	11	0,04	0,01	40,33	0,04	0,47	1,74
28	1	3	Banjaranyar	<200	Afrika	Maesopsis eminii Engl.	Rhamnaceae	0,43	17,8	8	10	0,04	0,02	83,38	0,08	0,98	3,60
29	2	1	Banjaranyar	<200	Sengon	Paraserianthes falcataria (L.) I.C	Leguminosae / Fabaceae	0,33	12,1	4	8	0,04	0,01	26,21	0,03	0,31	1,13
30	2	1	Banjaranyar	<200	Manggis	Mangostana garcinia Gaertn.	Clusiaceae	0,81	5,4	0,5	3	0,04	0,00	5,57	0,01	0,07	0,24
31	2	1	Banjaranyar	<200	Petai	Parkia speciosa Hassk.	Leguminosae / Fabaceae	0,45	25,8	4	11	0,04	0,05	186,52	0,19	2,19	8,04
32	2	1	Banjaranyar	<200	Pala	Myristica fragrans Houtt.	Myristicaceae	0,5	3,2	0,5	2	0,04	0,00	0,93	0,00	0,01	0,04
33	2	1	Banjaranyar	<200	Pala	Myristica fragrans Houtt.	Myristicaceae	0,5	8,3	0,5	4	0,04	0,01	10,14	0,01	0,12	0,44
34	2	1	Banjaranyar	<200	Kopi	Coffea canephora Pierre ex A.Fro	Rubiaceae	0,41	3,2	0,5	2,2	0,04	0,00	3,01	0,00	0,04	0,13
35	2	1	Banjaranyar	<200	Cengkeh	Syzygium aromaticum (L.) Merr. &	Myrtaceae	0,79	16,9	3	8	0,04	0,02	107,26	0,11	1,26	4,63
36	2	1	Banjaranyar	<200	Cengkeh	Syzygium aromaticum (L.) Merr. &	Myrtaceae	0,79	15,6		8	0,04	0,02	92,90	0,09	1,09	4,01
37	2	1	Banjaranyar	<200	Cengkeh	Syzygium aromaticum (L.) Merr. &	Myrtaceae	0,79	16,5		8	0,04	0,02	103,58	0,10	1,22	4,47
38	2	1	Banjaranyar	<200	Cengkeh	Syzygium aromaticum (L.) Merr. &	Myrtaceae	0,79	18,1		8	0,04	0,03	122,56	0,12	1,44	5,28
39	2	1	Banjaranyar	<200	Cengkeh	Syzygium aromaticum (L.) Merr. &	Myrtaceae	0,79	24,2	3,5	10	0,04	0,05	254,68	0,25	2,99	10,98
40	2	1	Banjaranyar	<200	Cengkeh	Syzygium aromaticum (L.) Merr. &	Myrtaceae	0,79	27,7		10	0,04	0,06	326,25	0,33	3,83	14,07
41	2	1	Banjaranyar	<200	Cengkeh	Syzygium aromaticum (L.) Merr. &	Myrtaceae	0,79	11,1		10	0,04	0,01	61,53	0,06	0,72	2,65
42	2	1	Banjaranyar	<200	Cengkeh	Syzygium aromaticum (L.) Merr. &	Myrtaceae	0,79	16,2		10	0,04	0,02	122,63	0,12	1,44	5,29
43	2	1	Banjaranyar	<200	Cengkeh	Syzygium aromaticum (L.) Merr. &	Myrtaceae	0,79	14,6	3	9	0,04	0,02	92,17	0,09	1,08	3,97
44	2	1	Banjaranyar	<200	Cengkeh	Syzygium aromaticum (L.) Merr. &	Myrtaceae	0,79	12,7		9	0,04	0,01	71,35	0,07	0,84	3,08
45	2	1	Banjaranyar	<200	Cengkeh	Syzygium aromaticum (L.) Merr. &	Myrtaceae	0,79	14,3		9	0,04	0,02	88,54	0,09	1,04	3,82
46	2	1	Banjaranyar	<200	Cengkeh	Syzygium aromaticum (L.) Merr. &	Myrtaceae	0,79	13,7		9	0,04	0,01	81,46	0,08	0,96	3,51
47	2	1	Banjaranyar	<200	Cengkeh	Syzygium aromaticum (L.) Merr. &	Myrtaceae	0,79	15,6	5	10	0,04	0,02	113,97	0,11	1,34	4,91
48	2	1	Banjaranyar	<200	Cengkeh	Syzygium aromaticum (L.) Merr. &	Myrtaceae	0,79	16,9		10	0,04	0,02	131,59	0,13	1,55	5,67
49	2	1	Banjaranyar	<200	Cengkeh	Syzygium aromaticum (L.) Merr. &	Myrtaceae	0,79	30,2	6	12	0,04	0,07	452,97	0,45	5,32	19,53
50	2	1	Banjaranyar	<200	Cengkeh	Syzygium aromaticum (L.) Merr. &	Myrtaceae	0,79	27,0	3,5	11	0,04	0,06	341,16	0,34	4,01	14,71
51	2	1	Banjaranyar	<200	Cengkeh	Syzygium aromaticum (L.) Merr. &	Myrtaceae	0,79	17,8		11	0,04	0,02	158,83	0,16	1,87	6,85
52	2	1	Banjaranyar	<200	Cengkeh	Syzygium aromaticum (L.) Merr. &	Myrtaceae	0,79	21,0		11	0,04	0,03	214,62	0,21	2,52	9,25
53	2	1	Banjaranyar	<200	Cengkeh	Syzygium aromaticum (L.) Merr. &	Myrtaceae	0,79	21,3	3	12	0,04	0,04	238,92	0,24	2,81	10,30
54	2	1	Banjaranyar	<200	Cengkeh	Syzygium aromaticum (L.) Merr. &	Myrtaceae	0,79	19,1		12	0,04	0,03	195,19	0,20	2,29	8,42
55	2	1	Banjaranyar	<200	Manggis	Mangostana garcinia Gaertn.	Clusiaceae	0,81	3,5	0,5	2	0,04	0,00	1,73	0,00	0,02	0,07
56	2	1	Banjaranyar	<200	Manggis	Mangostana garcinia Gaertn.	Clusiaceae	0,81	4,8	0,5	3	0,04	0,00	4,43	0,00	0,05	0,19
57	2	1	Banjaranyar	<200	Afrika	Maesopsis eminii Engl.	Rhamnaceae	0,43	3,2	1	2	0,04	0,00	0,81	0,00	0,01	0,04
58	2	1	Banjaranyar	<200	Cengkeh	Syzygium aromaticum (L.) Merr. &	Myrtaceae	0,79	18,1	2	8	0,04	0,03	122,56	0,12	1,44	5,28
59	2	1	Banjaranyar	<200	Cengkeh	Syzygium aromaticum (L.) Merr. &	Myrtaceae	0,79	15,0		8	0,04	0,02	86,07	0,09	1,01	3,71
60	2	1	Banjaranyar	<200	Cengkeh	Syzygium aromaticum (L.) Merr. &	Myrtaceae	0,79	24,5		8	0,04	0,05	212,63	0,21	2,50	9,17
61	2	1	Banjaranyar	<200	Cengkeh	Syzygium aromaticum (L.) Merr. &	Myrtaceae	0,79	17,8	2,5	12	0,04	0,02	172,01	0,17	2,02	7,42
62	2	1	Banjaranyar	<200	Cengkeh	Syzygium aromaticum (L.) Merr. &	Myrtaceae	0,79	19,7		12	0,04	0,03	207,27	0,21	2,44	8,94
63	2	1	Banjaranyar	<200	Cengkeh	Syzygium aromaticum (L.) Merr. &	Myrtaceae	0,79	23,5		12	0,04	0,04	286,62	0,29	3,37	12,36
64	2	1	Banjaranyar	<200	Manggis	Mangostana garcinia Gaertn.	Clusiaceae	0,81	4,1	0,5	2,5	0,04	0,00	2,88	0,00	0,03	0,12
65	2	1	Banjaranyar	<200	Kopi	Coffea canephora Pierre ex A.Fro	Rubiaceae	0,41	4,1	0,5	2,5	0,04	0,00	5,16	0,01	0,06	0,22
66	3	1	Banjaranyar	<200	Mahoni	Swietenia macrophylla King	Meliaceae	0,61	15,9	4	10	0,04	0,02	93,32	0,09	1,10	4,02
67	3	1	Banjaranyar	<200	Mahoni	Swietenia macrophylla King	Meliaceae	0,61	14,0	4	10	0,04	0,02	73,84	0,07	0,87	3,18
68	3	1	Banjaranyar	<200	Sirsak	Annona muricata L.	Annonaceae	0,36	9,9	1,5	4	0,04	0,01	10,36	0,01	0,12	0,45
69	3	1	Banjaranyar	<200	Manggis	Mangostana garcinia Gaertn.	Clusiaceae	0,81	4,5	0,5	2,5	0,04	0,00	3,30	0,00	0,04	0,14
70	3	1	Banjaranyar	<200	Sengon	Paraserianthes falcataria (L.) I.C	Leguminosae / Fabaceae	0,33	11,5	7	10	0,04	0,01	29,12	0,03	0,34	1,26
71	3	1	Banjaranyar	<200	Mahoni	Swietenia macrophylla King	Meliaceae	0,61	14,0	3	9	0,04	0,02	67,05	0,07	0,79	2,89
72	3	1	Banjaranyar	<200	Manggis	Mangostana garcinia Gaertn.	Clusiaceae	0,81	3,8	0,5	2,5	0,04	0,00	2,49	0,00	0,03	0,11
73	3	1	Banjaranyar	<200	Sengon	Paraserianthes falcataria (L.) I.C	Leguminosae / Fabaceae	0,33	18,5	6	13	0,04	0,03	88,73	0,09	1,04	3,83
74	3	1	Banjaranyar	<200	Sirsak	Annona muricata L.	Annonaceae	0,36	7,0	2	5	0,04	0,00	6,78	0,01	0,08	0,29
75	3	1	Banjaranyar	<200	Manggis	Mangostana garcinia Gaertn.	Clusiaceae	0,81	4,1	0,5	3	0,04	0,00	3,40	0,00	0,04	0,15
76	3	1	Banjaranyar	<200	Sirsak	Annona muricata L.	Annonaceae	0,36	9,9	1,8	6	0,04	0,01	15,02	0,02	0,18	0,65
77	3	1	Banjaranyar	<200	Sengon	Paraserianthes falcataria (L.) I.C	Leguminosae / Fabaceae	0,33	10,5	9	11	0,04	0,01	27,10	0,03	0,32	1,17
78	3	1	Banjaranyar	<200	Sengon	Paraserianthes falcataria (L.) I.C	Leguminosae / Fabaceae	0,33	19,4	10	14	0,04	0,03	104,15	0,10	1,22	4,49
79	3	1	Banjaranyar	<200	Sengon	Paraserianthes falcataria (L.) I.C	Leguminosae / Fabaceae	0,33	14,0	12	15	0,04	0,02	60,98	0,06	0,72	2,63
80	3	1	Banjaranyar	<200	Sirsak	Annona muricata L.	Annonaceae	0,36	7,0	1,5	6	0,04	0,00	8,01	0,01	0,09	0,35
81	3	1	Banjaranyar	<200	Sengon	Paraserianthes falcataria (L.) I.C	Leguminosae / Fabaceae	0,33	7,3	7	9	0,04	0,00	11,64	0,01	0,14	0,50
82	3	1	Banjaranyar	<200	Sengon	Paraserianthes falcataria (L.) I.C	Leguminosae / Fabaceae	0,33	15,0	7	11	0,04	0,02	51,79	0,05	0,61	2,23
83	3	1	Banjaranyar	<200	Sengon	Paraserianthes falcataria (L.) I.C	Leguminosae / Fabaceae	0,33	17,5	10	14	0,04	0,02	86,15	0,09	1,01	3,72
84	3	1	Banjaranyar	<200	Sengon	Paraserianthes falcataria (L.) I.C	Leguminosae / Fabaceae	0,33	17,2	6	13	0,04	0,02	77,84	0,08	0,91	3,36
85	3	1	Banjaranyar	<200	Manggis	Mangostana garcinia Gaertn.	Clusiaceae	0,81	4,5	0,5	2,5	0,04	0,00	3,30	0,00	0,04	0,14
86	3	1	Banjaranyar	<200	Sengon	Paraserianthes falcataria (L.) I.C	Leguminosae / Fabaceae	0,33	21,3	7	11	0,04	0,04	99,17	0,10	1,17	4,28
87	3	1	Banjaranyar	<200	Sirsak	Annona muricata L.	Annonaceae	0,36	5,7	1,5	3,5	0,04	0,00	3,39	0,00	0,04	0,15
88	3	1	Banjaranyar	<200	Manggis	Mangostana garcinia Gaertn.	Clusiaceae	0,81	4,5	0,5	2	0,04	0,00	2,69	0,00	0,03	0,12
89	3	1	Banjaranyar	<200	Sengon	Paraserianthes falcataria (L.) I.C	Leguminosae / Fabaceae	0,33	20,0	9	11	0,04	0,03	88,59	0,09	1,04	3,82
90	3	1	Banjaranyar	<200	Sengon	Paraserianthes falcataria (L.) I.C	Leguminosae / Fabaceae	0,33	15,9	12	15	0,04	0,02	77,07	0,08	0,91	3,32
91	3	1	Banjaranyar	<200	Sirsak	Annona muricata L.	Annonaceae	0,36	13,7	1,8	5	0,04	0,01	23,15	0,02	0,27	1,00
92	3	1	Banjaranyar	<200	Sengon	Paraserianthes falcataria (L.) I.C	Leguminosae / Fabaceae	0,33	17,5	7	13	0,04	0,02	80,50	0,08	0,95	3,47
93	3	1	Banjaranyar	<200	Sengon	Paraserianthes falcataria (L.) I.C	Leguminosae / Fabaceae	0,33	15,6	9	12	0,04	0,02	60,54	0,06	0,71	2,61
94	3	1	Banjaranyar	<200	Sengon	Paraserianthes falcataria (L.) I.C	Leguminosae / Fabaceae	0,33	14,6	5	11	0,04	0,02	49,79	0,05	0,59	2,15
95	3	1	Banjaranyar	<200	Sengon	Paraserianthes falcataria (L.) I.C	Leguminosae / Fabaceae	0,33	18,8	8	12	0,04	0,03	85,08	0,09	1,00	3,67
96	3	1	Banjaranyar	<200	Mahoni	Swietenia macrophylla King	Meliaceae	0,61	14,0	6	10	0,04	0,02	73,84	0,07	0,87	3,18
97	3	1	Banjaranyar	<200	Sengon	Paraserianthes falcataria (L.) I.C	Leguminosae / Fabaceae	0,33	12,7	9	14	0,04	0,01	48,07	0,05	0,56	2,07
98	3	1	Banjaranyar	<200	Sengon	Paraserianthes falcataria (L.) I.C	Leguminosae / Fabaceae	0,33	8,6	8	9	0,04	0,01	15,61	0,02	0,18	0,67
99	3	1	Banjaranyar	<200	Sengon	Paraserianthes falcataria (L.) I.C	Leguminosae / Fabaceae	0,33	12,1	7	9	0,04	0,01	29,20	0,03	0,34	1,26
100	3	1	Banjaranyar	<200	Sengon	Paraserianthes falcataria (L.) I.C	Leguminosae / Fabaceae	0,33	21,3	9	14	0,04	0,04	123,68	0,12	1,45	5,33
101	3	1	Banjaranyar	<200	Sengon	Paraserianthes falcataria (L.) I.C	Leguminosae / Fabaceae	0,33	19,7	9	13	0,04	0,03	100,26	0,10	1,18	4,32
102	3	1	Banjaranyar	<200	Sengon	Paraserianthes falcataria (L.) I.C	Leguminosae / Fabaceae	0,33	13,7	10	12	0,04	0,01	47,66	0,05	0,56	2,06
103	3	1	Banjaranyar	<200	Sengon	Paraserianthes falcataria (L.) I.C	Leguminosae / Fabaceae	0,33	21,3	5	13	0,04	0,04	115,56	0,12	1,36	4,98
104	3	1	Banjaranyar	<200	Sengon	Paraserianthes falcataria (L.) I.C	Leguminosae / Fabaceae	0,33	19,7	8	14	0,04	0,03	107,30	0,11	1,26	4,63
105	3	1	Banjaranyar	<200	Kopi	Coffea canephora Pierre ex A.Fro	Rubiaceae	0,41	3,2	0,5	2,2	0,04	0,00	3,01	0,00	0,04	0,13
106	4	1	Banjaranyar	<200	Karet	Hevea brasiliensis (Willd. ex A.Ju	Euphorbiaceae	0,53	11,5	6	9	0,04	0,01	40,81	0,04	0,48	1,76
107	4	1	Banjaranyar	<200	Karet	Hevea brasiliensis (Willd. ex A.Ju	Euphorbiaceae	0,53	9,9	6	8	0,04	0,01	27,86	0,03	0,33	1,20
108	4	1	Banjaranyar	<200	Jabon	Neolamarckia cadamba (Roxb.) I	Rubiaceae	0,42	8,0	5,5	6,5	0,04	0,00	12,55	0,01	0,15	0,54
109	4	1	Banjaranyar	<200	Karet	Hevea brasiliensis (Willd. ex A.Ju	Euphorbiaceae	0,53	6,0	5	7	0,04	0,00	10,05	0,01	0,12	0,43
110	4	1	Banjaranyar	<200	Karet	Hevea brasiliensis (Willd. ex A.Ju	Euphorbiaceae	0,53	7,3	6	8	0,04	0,00	16,12	0,02	0,19	0,70
111	4	1	Banjaranyar	<200	Kelapa	Cocos nucifera L.	Arecaceae	0,5	21,0	5	8	0,04	0,03	66,10	0,07	0,78	2,85
112	4	1	Banjaranyar	<200	Sengon	Paraserianthes falcataria (L.) I.C	Leguminosae / Fabaceae	0,33	16,5	9	13	0,04	0,02	72,64	0,07	0,85	3,13
113	4	1	Banjaranyar	<200	Karet	Hevea brasiliensis (Willd. ex A.Ju	Euphorbiaceae	0,53	17,2	7	12	0,04	0,02	111,64	0,11	1,31	4,81
114	4	1	Banjaranyar	<200	Kelapa	Cocos nucifera L.	Arecaceae	0,5	25,1	5	8	0,04	0,05	66,10	0,07	0,78	2,85
115	4	1	Banjaranyar	<200	Karet	Hevea brasiliensis (Willd. ex A.Ju	Euphorbiaceae	0,53	14,3	7	11	0,04	0,02	73,82	0,07	0,87	3,18
116	4	1	Banjaranyar	<200	Karet	Hevea brasiliensis (Willd. ex A.Ju	Euphorbiaceae	0,53	10,8	7	9	0,04	0,01	36,75	0,04	0,43	1,58
117	4	1	Banjaranyar	<200	Karet	Hevea brasiliensis (Willd. ex A.Ju	Euphorbiaceae	0,53	3,2	3	4	0,04	0,00	1,86	0,00	0,02	0,08
118	4	1	Banjaranyar	<200	Sengon	Paraserianthes falcataria (L.) I.C	Leguminosae / Fabaceae	0,33	9,5	9	10	0,04	0,01	20,85	0,02	0,25	0,90

119	4	1	Banjaranyar	<200	Karet	Hevea brasiliensis (Willd. ex A.Ju	Euphorbiaceae	0,53	12,1	8	10	0,04	0,01	49,63	0,05	0,58	2,14
120	4	1	Banjaranyar	<200	Karet	Hevea brasiliensis (Willd. ex A.Ju	Euphorbiaceae	0,53	11,8	5	7,5	0,04	0,01	36,31	0,04	0,43	1,57
121	4	1	Banjaranyar	<200	Karet	Hevea brasiliensis (Willd. ex A.Ju	Euphorbiaceae	0,53	9,9	5	7	0,04	0,01	24,65	0,02	0,29	1,06
122	4	1	Banjaranyar	<200	Sengon	Paraserianthes falcataria (L.) I.C.	Leguminosae / Fabaceae	0,33	15,3	10	12	0,04	0,02	58,30	0,06	0,68	2,51
123	4	1	Banjaranyar	<200	Sengon	Paraserianthes falcataria (L.) I.C.	Leguminosae / Fabaceae	0,33	8,0	6	7	0,04	0,00	10,77	0,01	0,13	0,46
124	4	1	Banjaranyar	<200	Karet	Hevea brasiliensis (Willd. ex A.Ju	Euphorbiaceae	0,53	11,8	5	9	0,04	0,01	42,91	0,04	0,50	1,85
125	4	1	Banjaranyar	<200	Sengon	Paraserianthes falcataria (L.) I.C.	Leguminosae / Fabaceae	0,33	9,5	8	9	0,04	0,01	18,93	0,02	0,22	0,82
126	4	1	Banjaranyar	<200	Karet	Hevea brasiliensis (Willd. ex A.Ju	Euphorbiaceae	0,53	20,0	5	11	0,04	0,03	136,73	0,14	1,61	5,90
127	4	1	Banjaranyar	<200	Karet	Hevea brasiliensis (Willd. ex A.Ju	Euphorbiaceae	0,53	11,8	9	11	0,04	0,01	51,57	0,05	0,61	2,22
128	4	1	Banjaranyar	<200	Karet	Hevea brasiliensis (Willd. ex A.Ju	Euphorbiaceae	0,53	12,4	4	9	0,04	0,01	47,26	0,05	0,56	2,04
129	4	1	Banjaranyar	<200	Sengon	Paraserianthes falcataria (L.) I.C.	Leguminosae / Fabaceae	0,33	13,4	11	13	0,04	0,01	49,12	0,05	0,58	2,12
130	4	1	Banjaranyar	<200	Karet	Hevea brasiliensis (Willd. ex A.Ju	Euphorbiaceae	0,53	10,5	4	9	0,04	0,01	34,80	0,03	0,41	1,50
131	4	1	Banjaranyar	<200	Karet	Hevea brasiliensis (Willd. ex A.Ju	Euphorbiaceae	0,53	4,5	2	5	0,04	0,00	4,22	0,00	0,05	0,18
132	4	1	Banjaranyar	<200	Karet	Hevea brasiliensis (Willd. ex A.Ju	Euphorbiaceae	0,53	8,0	3	7	0,04	0,00	16,62	0,02	0,20	0,72
133	4	1	Banjaranyar	<200	Kelapa	Cocos nucifera L.	Arecaceae	0,5	21,3	4	7	0,04	0,04	58,40	0,06	0,69	2,52
134	4	1	Banjaranyar	<200	Karet	Hevea brasiliensis (Willd. ex A.Ju	Euphorbiaceae	0,53	4,1	4	6	0,04	0,00	4,36	0,00	0,05	0,19
135	4	1	Banjaranyar	<200	Karet	Hevea brasiliensis (Willd. ex A.Ju	Euphorbiaceae	0,53	11,5	4,5	9	0,04	0,01	40,81	0,04	0,48	1,76
136	4	1	Banjaranyar	<200	Karet	Hevea brasiliensis (Willd. ex A.Ju	Euphorbiaceae	0,53	5,4	3	6	0,04	0,00	7,12	0,01	0,08	0,31
137	4	1	Banjaranyar	<200	Sengon	Paraserianthes falcataria (L.) I.C.	Leguminosae / Fabaceae	0,33	15,6	9	11	0,04	0,02	55,90	0,06	0,66	2,41
138	4	1	Banjaranyar	<200	Karet	Hevea brasiliensis (Willd. ex A.Ju	Euphorbiaceae	0,53	8,3	4,5	8,5	0,04	0,01	21,34	0,02	0,25	0,92
139	4	1	Banjaranyar	<200	Karet	Hevea brasiliensis (Willd. ex A.Ju	Euphorbiaceae	0,53	9,2	4	8	0,04	0,01	24,65	0,02	0,29	1,06
140	4	1	Banjaranyar	<200	Sengon	Paraserianthes falcataria (L.) I.C.	Leguminosae / Fabaceae	0,33	5,7	4	6	0,04	0,00	5,12	0,01	0,06	0,22
141	4	1	Banjaranyar	<200	Karet	Hevea brasiliensis (Willd. ex A.Ju	Euphorbiaceae	0,53	7,0	4	9	0,04	0,00	16,56	0,02	0,19	0,71
142	4	1	Banjaranyar	<200	Kelapa	Cocos nucifera L.	Arecaceae	0,5	23,2	4	7	0,04	0,04	58,40	0,06	0,69	2,52
143	4	1	Banjaranyar	<200	Sengon	Paraserianthes falcataria (L.) I.C.	Leguminosae / Fabaceae	0,33	4,8	6	7	0,04	0,00	4,22	0,00	0,05	0,18
144	4	1	Banjaranyar	<200	Karet	Hevea brasiliensis (Willd. ex A.Ju	Euphorbiaceae	0,53	16,5	6	10	0,04	0,02	88,16	0,09	1,04	3,80
145	4	1	Banjaranyar	<200	Karet	Hevea brasiliensis (Willd. ex A.Ju	Euphorbiaceae	0,53	12,7	7	10	0,04	0,01	54,52	0,05	0,64	2,35
146	4	1	Banjaranyar	<200	Sengon	Paraserianthes falcataria (L.) I.C.	Leguminosae / Fabaceae	0,33	15,3	12	15	0,04	0,02	71,52	0,07	0,84	3,08
147	4	1	Banjaranyar	<200	Karet	Hevea brasiliensis (Willd. ex A.Ju	Euphorbiaceae	0,53	12,4	4	9	0,04	0,01	47,26	0,05	0,56	2,04
148	4	1	Banjaranyar	<200	Karet	Hevea brasiliensis (Willd. ex A.Ju	Euphorbiaceae	0,53	9,9	5	9	0,04	0,01	31,03	0,03	0,36	1,34
149	4	1	Banjaranyar	<200	Karet	Hevea brasiliensis (Willd. ex A.Ju	Euphorbiaceae	0,53	8,9	6	10	0,04	0,01	28,36	0,03	0,33	1,22
150	4	1	Banjaranyar	<200	Karet	Hevea brasiliensis (Willd. ex A.Ju	Euphorbiaceae	0,53	16,9	3	11	0,04	0,02	99,62	0,10	1,17	4,30
151	4	1	Banjaranyar	<200	Karet	Hevea brasiliensis (Willd. ex A.Ju	Euphorbiaceae	0,53	12,4	6	11	0,04	0,01	56,79	0,06	0,67	2,45
152	4	1	Banjaranyar	<200	Karet	Hevea brasiliensis (Willd. ex A.Ju	Euphorbiaceae	0,53	9,2	4	8	0,04	0,01	24,65	0,02	0,29	1,06
153	4	1	Banjaranyar	<200	Jabon	Neolamarckia cadamba (Roxb.) I	Rubiaceae	0,42	17,2	10	11	0,04	0,02	83,31	0,08	0,98	3,59
154	4	1	Banjaranyar	<200	Karet	Hevea brasiliensis (Willd. ex A.Ju	Euphorbiaceae	0,53	4,8	3	5	0,04	0,00	4,79	0,00	0,06	0,21
155	4	1	Banjaranyar	<200	Karet	Hevea brasiliensis (Willd. ex A.Ju	Euphorbiaceae	0,53	12,1	4	8	0,04	0,01	40,45	0,04	0,48	1,74
156	4	1	Banjaranyar	<200	Jabon	Neolamarckia cadamba (Roxb.) I	Rubiaceae	0,42	12,4	6	7	0,04	0,01	30,34	0,03	0,36	1,31
157	4	1	Banjaranyar	<200	Karet	Hevea brasiliensis (Willd. ex A.Ju	Euphorbiaceae	0,53	7,0	4	6	0,04	0,00	11,42	0,01	0,13	0,49
158	4	1	Banjaranyar	<200	Karet	Hevea brasiliensis (Willd. ex A.Ju	Euphorbiaceae	0,53	7,3	3,5	6,5	0,04	0,00	13,33	0,01	0,16	0,57
159	4	1	Banjaranyar	<200	Jati	Tectona grandis L.f.	Lamiaceae / Verbenaceae	0,7	3,8	4	4,5	0,04	0,00	3,73	0,00	0,04	0,16
160	4	1	Banjaranyar	<200	Karet	Hevea brasiliensis (Willd. ex A.Ju	Euphorbiaceae	0,53	12,1	4	7	0,04	0,01	35,80	0,04	0,42	1,54
161	4	1	Banjaranyar	<200	Karet	Hevea brasiliensis (Willd. ex A.Ju	Euphorbiaceae	0,53	11,5	4	9	0,04	0,01	40,81	0,04	0,48	1,76
162	4	1	Banjaranyar	<200	Karet	Hevea brasiliensis (Willd. ex A.Ju	Euphorbiaceae	0,53	4,5	2,5	4	0,04	0,00	3,44	0,00	0,04	0,15
163	4	1	Banjaranyar	<200	Alpukat	Persea americana Mill.	Lauraceae	0,54	19,1	3,5	7	0,04	0,03	84,08	0,08	0,99	3,63
164	4	1	Banjaranyar	<200	Kelapa	Cocos nucifera L.	Arecaceae	0,5	24,2	3	6	0,04	0,05	50,70	0,05	0,60	2,19
165	4	1	Banjaranyar	<200	Jati	Tectona grandis L.f.	Lamiaceae / Verbenaceae	0,7	5,4	2,5	4	0,04	0,00	6,34	0,01	0,07	0,27
166	4	1	Banjaranyar	<200	Karet	Hevea brasiliensis (Willd. ex A.Ju	Euphorbiaceae	0,53	3,5	2,5	3,5	0,04	0,00	1,96	0,00	0,02	0,08
167	4	1	Banjaranyar	<200	Karet	Hevea brasiliensis (Willd. ex A.Ju	Euphorbiaceae	0,53	15,0	4	10	0,04	0,02	73,26	0,07	0,86	3,16
168	4	1	Banjaranyar	<200	Jabon	Neolamarckia cadamba (Roxb.) I	Rubiaceae	0,42	11,8	9	10	0,04	0,01	38,19	0,04	0,45	1,65
169	4	1	Banjaranyar	<200	Jabon	Neolamarckia cadamba (Roxb.) I	Rubiaceae	0,42	7,3	5	6	0,04	0,00	10,01	0,01	0,12	0,43
170	4	1	Banjaranyar	<200	Karet	Hevea brasiliensis (Willd. ex A.Ju	Euphorbiaceae	0,53	2,5	3	3,5	0,04	0,00	1,09	0,00	0,01	0,05
171	4	1	Banjaranyar	<200	Jabon	Neolamarckia cadamba (Roxb.) I	Rubiaceae	0,42	3,8	3,5	1,5	0,04	0,00	0,85	0,00	0,01	0,04
172	4	1	Banjaranyar	<200	Kelapa	Cocos nucifera L.	Arecaceae	0,5	20,0	5	3	0,04	0,03	27,60	0,03	0,32	1,19
173	4	1	Banjaranyar	<200	karet	Hevea brasiliensis (Willd. ex A.Ju	Euphorbiaceae	0,53	2,5	3	4	0,04	0,00	1,23	0,00	0,01	0,05
174	4	1	Banjaranyar	<200	Karet	Hevea brasiliensis (Willd. ex A.Ju	Euphorbiaceae	0,53	11,8	6	11	0,04	0,01	51,57	0,05	0,61	2,22
175	4	1	Banjaranyar	<200	Jabon	Neolamarckia cadamba (Roxb.) I	Rubiaceae	0,42	6,0	5	6	0,04	0,00	7,05	0,01	0,08	0,30
176	4	1	Banjaranyar	<200	Jabon	Neolamarckia cadamba (Roxb.) I	Rubiaceae	0,42	20,7	11	13	0,04	0,03	136,35	0,14	1,60	5,88
177	4	1	Banjaranyar	<200	Karet	Hevea brasiliensis (Willd. ex A.Ju	Euphorbiaceae	0,53	7,6	3	8	0,04	0,00	17,43	0,02	0,20	0,75
178	5	2	Banjaranyar	<200	Mahoni	Swietenia macrophylla King	Meliaceae	0,61	6,0	2	4	0,04	0,00	6,85	0,01	0,08	0,30
179	5	2	Banjaranyar	<200	Mahoni	Swietenia macrophylla King	Meliaceae	0,61	26,4	7	11	0,04	0,05	257,72	0,26	3,03	11,11
180	5	2	Banjaranyar	<200	Dukuh	Lansium domesticum Corrêa	Meliaceae	0,85	5,7	3	7	0,04	0,00	14,04	0,01	0,16	0,61
181	5	2	Banjaranyar	<200	Mahoni	Swietenia macrophylla King	Meliaceae	0,61	9,5	4	8	0,04	0,01	29,84	0,03	0,35	1,29
182	5	2	Banjaranyar	<200	Mangga	Mangifera indica L.	Anacardiaceae	0,6	10,5	3	7	0,04	0,01	30,97	0,03	0,36	1,34
183	5	2	Banjaranyar	<200	Jati	Tectona grandis L.f.	Lamiaceae / Verbenaceae	0,7	27,0	6	12	0,04	0,06	330,72	0,33	3,89	14,26
184	5	2	Banjaranyar	<200	Mahoni	Swietenia macrophylla King	Meliaceae	0,61	12,7	7	10	0,04	0,01	62,01	0,06	0,73	2,67
185	5	2	Banjaranyar	<200	Mahoni	Swietenia macrophylla King	Meliaceae	0,61	9,5	3	6	0,04	0,01	22,93	0,02	0,27	0,99
186	5	2	Banjaranyar	<200	Jati	Tectona grandis L.f.	Lamiaceae / Verbenaceae	0,7	22,3	7	12	0,04	0,04	231,73	0,23	2,72	9,99
187	5	2	Banjaranyar	<200	Mahoni	Swietenia macrophylla King	Meliaceae	0,61	22,6	7	11	0,04	0,04	193,60	0,19	2,27	8,35
188	5	2	Banjaranyar	<200	Dukuh	Lansium domesticum Corrêa	Meliaceae	0,85	15,3	4	9	0,04	0,02	106,56	0,11	1,25	4,60
189	5	2	Banjaranyar	<200	Mahoni	Swietenia macrophylla King	Meliaceae	0,61	5,7	3	7	0,04	0,00	10,36	0,01	0,12	0,45
190	5	2	Banjaranyar	<200	Langsat	Lansium domesticum	Meliaceae	0,7	4,5	2	5	0,04	0,00	5,45	0,01	0,06	0,23
191	5	2	Banjaranyar	<200	Mahoni	Swietenia macrophylla King	Meliaceae	0,61	5,1	3	5	0,04	0,00	6,13	0,01	0,07	0,26
192	5	2	Banjaranyar	<200	Sawo hijau	Chrysophyllum cainito L.	Sapotaceae	0,69	27,4	7	10	0,04	0,06	282,17	0,28	3,32	12,17
193	5	2	Banjaranyar	<200	Dukuh	Lansium domesticum Corrêa	Meliaceae	0,85	11,5	4	8	0,04	0,01	56,47	0,06	0,66	2,44
194	5	2	Banjaranyar	<200	Mahoni	Swietenia macrophylla King	Meliaceae	0,61	13,4	5	11	0,04	0,01	73,99	0,07	0,87	3,19
195	5	2	Banjaranyar	<200	Mahoni	Swietenia macrophylla King	Meliaceae	0,61	5,4	2	7	0,04	0,00	9,33	0,01	0,11	0,40
196	5	2	Banjaranyar	<200	Mahoni	Swietenia macrophylla King	Meliaceae	0,61	28,0	13	17	0,04	0,06	427,43	0,43	5,02	18,43
197	5	2	Banjaranyar	<200	Mahoni	Swietenia macrophylla King	Meliaceae	0,61	8,9	7	8,5	0,04	0,01	27,80	0,03	0,33	1,20
198	5	2	Banjaranyar	<200	Mahoni	Swietenia macrophylla King	Meliaceae	0,61	10,5	5	9	0,04	0,01	39,58	0,04	0,47	1,71
199	5	2	Banjaranyar	<200	Dukuh	Lansium domesticum Corrêa	Meliaceae	0,85	23,2	6	14	0,04	0,04	344,29	0,34	4,05	14,85
200	5	2	Banjaranyar	<200	Mahoni	Swietenia macrophylla King	Meliaceae	0,61	8,3	4	8	0,04	0,01	22,96	0,02	0,27	0,99
201	5	2	Banjaranyar	<200	Mahoni	Swietenia macrophylla King	Meliaceae	0,61	7,0	4	9	0,04	0,00	18,83	0,02	0,22	0,81
202	5	2	Banjaranyar	<200	Mahoni	Swietenia macrophylla King	Meliaceae	0,61	6,4	5	9	0,04	0,00	15,81	0,02	0,19	0,68
203	5	2	Banjaranyar	<200	Mahoni	Swietenia macrophylla King	Meliaceae	0,61	28,0	15	19	0,04	0,06	473,27	0,47	5,56	20,41
204	5	2	Banjaranyar	<200	Dukuh	Lansium domesticum Corrêa	Meliaceae	0,85	17,2	6	12	0,04	0,02	172,08	0,17	2,02	7,42
205	5	2	Banjaranyar	<200	Mahoni	Swietenia macrophylla King	Meliaceae	0,61	29,0	14	19	0,04	0,07	503,25	0,50	5,91	21,70
206	5	2	Banjaranyar	<200	Kelapa	Cocos nucifera L.	Arecaceae	0,5	28,6	12	15	0,04	0,06	120,00	0,12	1,41	5,17
207	5	2	Banjaranyar	<200	Mahoni	Swietenia macrophylla King	Meliaceae	0,61	11,5	5	6	0,04	0,01	32,02	0,03	0,38	1,38
208	5	2	Banjaranyar	<200	Mahoni	Swietenia macrophylla King	Meliaceae	0,61	36,3	15	19	0,04	0,10	760,45	0,76	8,94	32,79
209	5	2	Banjaranyar	<200	Mahoni	Swietenia macrophylla King	Meliaceae	0,61	6,4	4	8	0,04	0,00	14,20	0,01	0,17	0,61
210	5	2	Banjaranyar	<200	Nangka	Artocarpus heterophyllus Lam.	Moraceae	0,61	25,1	10	15	0,04	0,05	312,78	0,31	3,68	13,49
211	5	2	Banjaranyar	<200	Mahoni	Swietenia macrophylla King	Meliaceae	0,61	18,5	8	11	0,04	0,03	133,66	0,13	1,57	5,76
212	5	2	Banjaranyar	<200	Nyamplung	Calophyllum inophyllum L.	Clusiaceae	0,61	40,7	15	19	0,04	0,13	940,22	0,94	11,05	40,54
213	5	2	Banjaranyar	<200	Mahoni	Swietenia macrophylla King	Meliaceae	0,61	6,4	4	6	0,04	0,00	10,91	0,01	0,13	0,47
214	5	2	Banjaranyar	<200	Mahoni	Swietenia macrophylla King	Meliaceae	0,61	38,8	12	18	0,04	0,12	819,45	0,82	9,63	35,34
215	5	2	Banjaranyar	<200	Dukuh	Lansium domesticum Corrêa	Meliaceae	0,85	10,5	7	12	0,04	0,01	69,81	0,07	0,82	3,01
216	5	2	Banjaranyar	<200	Mahoni	Swietenia macrophylla King	Meliaceae	0,61	3,5	4	5,5	0,04	0,00	3,37	0,00	0,04	0,15
217	5	2	Banjaranyar	<200	Mahoni	Swietenia macrophylla King	Meliaceae	0,61	11,5	6	11	0,04	0,01	55,79	0,06	0,66	2,41
218	5	2	Banjaranyar	<200	Mahoni	Swietenia macrophylla King	Meliaceae	0,61	5,1	2,5	5,5	0,04	0,00	6,69	0,01	0,08	0,29
219	5	2	Banjaranyar	<200	Dukuh	Lansium domesticum Corrêa	Meliaceae	0,85	13,7	6	10	0,04	0,01	95,94	0,10	1,13	4,14
220	5	2	Banjaranyar	<200	Dukuh	Lansium domesticum Corrêa	Meliaceae	0,85	8,0	4	9	0,04	0,00	32,25	0,03	0,38	1,39
221	5	2	Banjaranyar	<200	Mahoni	Swietenia macrophylla King	Meliaceae	0,61	6,4	3,5	6	0,04	0,00	10,91	0,01	0,13	0,47
222	5	2	Banjaranyar	<200	Langsat	Lansium domesticum	Meliaceae	0,7	12,7	3	9	0,04	0,01	63,87	0,06	0,75	2,75
223	5	2	Banjaranyar	<200	Nangka	Artocarpus heterophyllus Lam.	Moraceae	0,61	8,9	6	8	0,04	0,01	26,30	0,03	0,31	1,13
224	5	2	Banjaranyar	<200	Dukuh	Lansium domesticum Corrêa	Meliaceae	0,85	12,1	4	8	0,04	0,01	62,35	0,06	0,73	2,69
225	5	2	Banjaranyar	<200	Mahoni	Swietenia macrophylla King	Meliaceae	0,61	11,1	7	9	0,04	0,01	44,09	0,04	0,52	1,90
226	5	2	Banjaranyar	<200	Dukuh	Lansium domesticum Corrêa	Meliaceae	0,85	11,5	4	8	0,04	0,01	56,47	0,06	0,66	2,44
227	5	2	Banjaranyar	<200	Mahoni	Swietenia macrophylla King	Meliaceae	0,61	11,8	5	8	0,04	0,01	43,82	0,04	0,51	1,89
228	5	2	Banjaranyar	<200	Manggis	Mangostana garcinia Gaertn.	Clusiaceae	0,81	8,0	1	4	0,04	0,00	14,68	0,01	0,17	0,63
229	5	2	Banjaranyar	<200	Dukuh	Lansium domesticum Corrêa	Meliaceae	0,85	12,4	5	9	0,04	0,01	72,84	0,07	0,86	3,14
230	5	2	Banjaranyar	<200	Mahoni	Swietenia macrophylla King	Meliaceae	0,61	4,1	3	3,5	0,04	0,00	3,02	0,00	0,04	0,13
231	5	2	Banjaranyar	<200	Kelapa	Cocos nucifera L.	Arecaceae	0,5	26,1	11	14	0,04	0,05	112,30	0,11	1,32	4,84
232	5	2	Banjaranyar	<200	Mahoni	Swietenia macrophylla King	Meliaceae	0,61	12,1	4	7	0,04	0,01	40,72	0,04	0,48	1,76
233	5	2	Banjaranyar	<200	Mahoni	Swietenia macrophylla King	Meliaceae	0,61	14,0	5	8	0,04	0,02	60,19	0,06	0,71	2,60
234	5	2	Banjaranyar	<200	Petai	Parkia speciosa Hassk.	Leguminosae / Fabaceae	0,45	23,2	9	12	0,04	0,04	166,96	0,17	1,96	7,20
235	5	2	Banjaranyar	<200	Dukuh	Lansium domesticum Corrêa	Meliaceae	0,85	4,5	4	5,5	0,04	0,00	7,10	0,01	0,08	0,31
236	5	2	Banjaranyar	<200	Langsat	Lansium domesticum	Meliaceae	0,7	10,8	3	8	0,04	0,01	42,57	0,04	0,50	1,84
237	5	2	Banjaranyar	<200	Nangka	Artocarpus heterophyllus Lam.	Moraceae	0,61	32,5	7	11	0,04	0,08	375,97	0,38	4,42	16,21
238	5	2	Banjaranyar	<200	Mahoni	Swietenia macrophylla King	Meliaceae	0,61	6,7	2,5	6	0,04	0,00	11,93	0,01	0,14	0,51
239	5	2	Banjaranyar	<200	Mahoni	Swietenia macrophylla King	Meliaceae	0,61	5,7	2,5	5	0,04	0,00	7,61	0,01	0,09	0,33
240	6	3	Banjaranyar	<200	Kelapa	Cocos nucifera L.	Arecaceae	0,5	28,6	7	10	0,04	0,06	81,50	0,08	0,96	3,51
241	6	3	Banjaranyar	<200	Langsat	Lansium domesticum	Meliaceae	0,7	14,6	7	9	0,04	0,02	82,51	0,08	0,97	3,56
242	6	3	Banjaranyar	<200	Manggis	Mangostana garcinia Gaertn.	Clusiaceae	0,81	7,3	1	4	0,04	0,00	12,60	0,01	0,15	0,54

243	6	3	Banjaranyar	<200	Caruy	Pterospermum javanicum Jungh.	Malvaceae	0,53	11,8	6	9	0,04	0,01	42,91	0,04	0,50	1,85
244	6	3	Banjaranyar	<200	Pala	Myristica fragrans Houtt.	Myristicaceae	0,5	5,1	1	3	0,04	0,00	3,20	0,00	0,04	0,14
245	6	3	Banjaranyar	<200	Durian	Durio zibethinus L.	Malvaceae	0,57	21,0	9	12	0,04	0,03	172,36	0,17	2,03	7,43
246	6	3	Banjaranyar	<200	Pala	Myristica fragrans Houtt.	Myristicaceae	0,5	3,2	1	3	0,04	0,00	1,35	0,00	0,02	0,06
247	6	3	Banjaranyar	<200	Langsat	Lansium domesticum	Meliaceae	0,7	8,3	3,5	6,5	0,04	0,01	21,53	0,02	0,25	0,93
248	6	3	Banjaranyar	<200	Kelapa	Cocos nucifera L.	Arecaceae	0,5	28,0	9	12	0,04	0,06	96,90	0,10	1,14	4,18
249	6	3	Banjaranyar	<200	Kedondong	Spondias dulcis Parkinson	Anacardiaceae	0,3	26,4	7	11	0,04	0,05	134,53	0,13	1,58	5,80
250	6	3	Banjaranyar	<200	Manggis	Mangostana garcinia Gaertn.	Clusiaceae	0,81	9,9	2	6	0,04	0,01	31,57	0,03	0,37	1,36
251	6	3	Banjaranyar	<200	Kelapa	Cocos nucifera L.	Arecaceae	0,5	29,0	13	16	0,04	0,07	127,70	0,13	1,50	5,51
252	6	3	Banjaranyar	<200	Pala	Myristica fragrans Houtt.	Myristicaceae	0,5	4,1	1	3	0,04	0,00	2,19	0,00	0,03	0,09
253	6	3	Banjaranyar	<200	Manggis	Mangostana garcinia Gaertn.	Clusiaceae	0,81	9,2	1	4	0,04	0,01	19,27	0,02	0,23	0,83
254	6	3	Banjaranyar	<200	Kelapa	Cocos nucifera L.	Arecaceae	0,5	24,8	13	16	0,04	0,05	127,70	0,13	1,50	5,51
255	6	3	Banjaranyar	<200	Sengon	Paraserianthes falcataria (L.) I.C.	Leguminosae / Fabaceae	0,33	3,2	1,5	3	0,04	0,00	0,92	0,00	0,01	0,04
256	6	3	Banjaranyar	<200	Sengon	Paraserianthes falcataria (L.) I.C.	Leguminosae / Fabaceae	0,33	2,9	1,5	3	0,04	0,00	0,76	0,00	0,01	0,03
257	6	3	Banjaranyar	<200	Sengon	Paraserianthes falcataria (L.) I.C.	Leguminosae / Fabaceae	0,33	3,2	2	3,5	0,04	0,00	1,07	0,00	0,01	0,05
258	6	3	Banjaranyar	<200	Sengon	Paraserianthes falcataria (L.) I.C.	Leguminosae / Fabaceae	0,33	2,5	1,5	3	0,04	0,00	0,61	0,00	0,01	0,03
259	6	3	Banjaranyar	<200	Petai	Parkia speciosa Hassk.	Leguminosae / Fabaceae	0,45	14,3	4	1	0,04	0,02	7,07	0,01	0,08	0,30
260	6	3	Banjaranyar	<200	Gamal/Kalkiria	Gliricidia sepium (Jacq.) Walp.	Leguminosae / Fabaceae	0,58	5,1	2	3	0,04	0,00	3,67	0,00	0,04	0,16
261	6	3	Banjaranyar	<200	Hantap	Sterculia oblongata R.Br.	Malvaceae	0,28	5,4	3	4	0,04	0,00	2,74	0,00	0,03	0,12
262	6	3	Banjaranyar	<200	Kelapa	Cocos nucifera L.	Arecaceae	0,5	28,6	7	10	0,04	0,06	81,50	0,08	0,96	3,51
263	6	3	Banjaranyar	<200	Pala	Myristica fragrans Houtt.	Myristicaceae	0,5	18,8	1,5	7,5	0,04	0,03	80,93	0,08	0,95	3,49
264	6	3	Banjaranyar	<200	Pala	Myristica fragrans Houtt.	Myristicaceae	0,5	15,3	1,5	8	0,04	0,02	58,84	0,06	0,69	2,54
265	6	3	Banjaranyar	<200	Manggis	Mangostana garcinia Gaertn.	Clusiaceae	0,81	12,7	2	8	0,04	0,01	65,54	0,07	0,77	2,83
266	6	3	Banjaranyar	<200	Pala	Myristica fragrans Houtt.	Myristicaceae	0,5	20,7	2	11	0,04	0,03	137,26	0,14	1,61	5,92
267	6	3	Banjaranyar	<200	Kelapa	Cocos nucifera L.	Arecaceae	0,5	30,5	9	12	0,04	0,07	96,90	0,10	1,14	4,18
268	6	3	Banjaranyar	<200	Langsat	Lansium domesticum	Meliaceae	0,7	3,2	2	3	0,04	0,00	1,84	0,00	0,02	0,08
269	6	3	Banjaranyar	<200	Mangga	Mangifera indica L.	Anacardiaceae	0,6	8,9	5	7	0,04	0,01	22,92	0,02	0,27	0,99
270	6	3	Banjaranyar	<200	Pinang	Areca catechu L.	Arecaceae	0,66	14,3	6	8	0,04	0,02	67,41	0,07	0,79	2,91
271	6	3	Banjaranyar	<200	Cempedak	Artocarpus integer (Thunb.) Merr	Moraceae	0,56	10,5	5	8	0,04	0,01	32,86	0,03	0,39	1,42
272	6	3	Banjaranyar	<200	Pinang	Areca catechu L.	Arecaceae	0,66	14,0	5	7	0,04	0,02	57,24	0,06	0,67	2,47
273	6	3	Banjaranyar	<200	Pala	Myristica fragrans Houtt.	Myristicaceae	0,5	4,8	1	4	0,04	0,00	3,70	0,00	0,04	0,16
274	7	2	Banjaranyar	<200	Mahoni	Swietenia macrophylla King	Meliaceae	0,61	18,1	5	10	0,04	0,03	118,64	0,12	1,39	5,12
275	7	2	Banjaranyar	<200	Kelapa	Cocos nucifera L.	Arecaceae	0,5	24,8	12	15	0,04	0,05	120,00	0,12	1,41	5,17
276	7	2	Banjaranyar	<200	Sengon	Paraserianthes falcataria (L.) I.C.	Leguminosae / Fabaceae	0,33	19,1	13	15	0,04	0,03	107,63	0,11	1,26	4,64
277	7	2	Banjaranyar	<200	Tisuk	Hibiscus macrophyllus Roxb. ex	Malvaceae	0,4	14,3	10	12	0,04	0,02	61,78	0,06	0,73	2,66
278	7	2	Banjaranyar	<200	Mahoni	Swietenia macrophylla King	Meliaceae	0,61	9,9	4	7	0,04	0,01	28,04	0,03	0,33	1,21
279	7	2	Banjaranyar	<200	Manggis	Mangostana garcinia Gaertn.	Clusiaceae	0,81	2,9	0,5	2,5	0,04	0,00	1,47	0,00	0,02	0,06
280	7	2	Banjaranyar	<200	Sengon	Paraserianthes falcataria (L.) I.C.	Leguminosae / Fabaceae	0,33	16,9	14	17	0,04	0,02	96,17	0,10	1,13	4,15
281	7	2	Banjaranyar	<200	Sengon	Paraserianthes falcataria (L.) I.C.	Leguminosae / Fabaceae	0,33	6,0	6	8	0,04	0,00	7,36	0,01	0,09	0,32
282	7	2	Banjaranyar	<200	Mahoni	Swietenia macrophylla King	Meliaceae	0,61	9,5	3	8	0,04	0,01	29,84	0,03	0,35	1,29
283	7	2	Banjaranyar	<200	Mahoni	Swietenia macrophylla King	Meliaceae	0,61	13,0	4	8	0,04	0,01	52,88	0,05	0,62	2,28
284	7	2	Banjaranyar	<200	Tisuk	Hibiscus macrophyllus Roxb. ex	Malvaceae	0,4	6,7	7	10	0,04	0,00	12,94	0,01	0,15	0,56
285	7	2	Banjaranyar	<200	Mahoni	Swietenia macrophylla King	Meliaceae	0,61	15,0	3	8	0,04	0,02	67,92	0,07	0,80	2,93
286	7	2	Banjaranyar	<200	Sengon	Paraserianthes falcataria (L.) I.C.	Leguminosae / Fabaceae	0,33	13,4	12	14	0,04	0,01	52,57	0,05	0,62	2,27
287	7	2	Banjaranyar	<200	Tisuk	Hibiscus macrophyllus Roxb. ex	Malvaceae	0,4	18,5	14	17	0,04	0,03	135,30	0,14	1,59	5,83
288	7	2	Banjaranyar	<200	Mahoni	Swietenia macrophylla King	Meliaceae	0,61	14,3	5	10	0,04	0,02	76,94	0,08	0,90	3,32
289	7	2	Banjaranyar	<200	Sengon	Paraserianthes falcataria (L.) I.C.	Leguminosae / Fabaceae	0,33	13,7	12	15	0,04	0,01	58,46	0,06	0,69	2,52
290	7	2	Banjaranyar	<200	Mahoni	Swietenia macrophylla King	Meliaceae	0,61	15,0	5	9	0,04	0,02	75,66	0,08	0,89	3,26
291	7	2	Banjaranyar	<200	Mahoni	Swietenia macrophylla King	Meliaceae	0,61	13,7	6	8	0,04	0,01	57,71	0,06	0,68	2,49
292	7	2	Banjaranyar	<200	Manggis	Mangostana garcinia Gaertn.	Clusiaceae	0,81	3,2	1	3	0,04	0,00	2,11	0,00	0,02	0,09
293	7	2	Banjaranyar	<200	Karet	Hevea brasiliensis (Willd. ex A.Ju	Euphorbiaceae	0,53	5,4	3	8	0,04	0,00	9,27	0,01	0,11	0,40
294	7	2	Banjaranyar	<200	Mahoni	Swietenia macrophylla King	Meliaceae	0,61	7,6	3	6	0,04	0,00	15,23	0,02	0,18	0,66
295	7	2	Banjaranyar	<200	Mahoni	Swietenia macrophylla King	Meliaceae	0,61	4,8	4	5	0,04	0,00	5,45	0,01	0,06	0,23
296	7	2	Banjaranyar	<200	Tisuk	Hibiscus macrophyllus Roxb. ex	Malvaceae	0,4	21,0	12	16	0,04	0,03	162,18	0,16	1,91	6,99
297	7	2	Banjaranyar	<200	Sengon	Paraserianthes falcataria (L.) I.C.	Leguminosae / Fabaceae	0,33	9,9	8	10	0,04	0,01	22,14	0,02	0,26	0,95
298	7	2	Banjaranyar	<200	Mahoni	Swietenia macrophylla King	Meliaceae	0,61	10,8	5	7	0,04	0,01	33,21	0,03	0,39	1,43
299	7	2	Banjaranyar	<200	Karet	Hevea brasiliensis (Willd. ex A.Ju	Euphorbiaceae	0,53	2,5	2	3	0,04	0,00	0,95	0,00	0,01	0,04
300	7	2	Banjaranyar	<200	Karet	Hevea brasiliensis (Willd. ex A.Ju	Euphorbiaceae	0,53	13,7	9	12	0,04	0,01	73,55	0,07	0,86	3,17
301	7	2	Banjaranyar	<200	Langsat	Lansium domesticum	Meliaceae	0,7	3,2	3	4	0,04	0,00	2,40	0,00	0,03	0,10
302	7	2	Banjaranyar	<200	Mahoni	Swietenia macrophylla King	Meliaceae	0,61	9,2	5	8	0,04	0,01	28,04	0,03	0,33	1,21
303	7	2	Banjaranyar	<200	Manggis	Mangostana garcinia Gaertn.	Clusiaceae	0,81	2,5	0,8	2	0,04	0,00	0,96	0,00	0,01	0,04
304	7	2	Banjaranyar	<200	Sengon	Paraserianthes falcataria (L.) I.C.	Leguminosae / Fabaceae	0,33	33,1	15	19	0,04	0,09	366,12	0,37	4,30	15,79
305	7	2	Banjaranyar	<200	Tisuk	Hibiscus macrophyllus Roxb. ex	Malvaceae	0,4	11,1	10	12	0,04	0,01	38,98	0,04	0,46	1,68
306	7	2	Banjaranyar	<200	Mahoni	Swietenia macrophylla King	Meliaceae	0,61	9,2	4	6	0,04	0,01	21,55	0,02	0,25	0,93
307	7	2	Banjaranyar	<200	Manggis	Mangostana garcinia Gaertn.	Clusiaceae	0,81	3,2	0,5	2	0,04	0,00	1,45	0,00	0,02	0,06
308	7	2	Banjaranyar	<200	Mahoni	Swietenia macrophylla King	Meliaceae	0,61	15,3	6	9	0,04	0,02	78,63	0,08	0,92	3,39
309	7	2	Banjaranyar	<200	Mahoni	Swietenia macrophylla King	Meliaceae	0,61	12,1	5	9	0,04	0,01	51,25	0,05	0,60	2,21
310	7	2	Banjaranyar	<200	Sengon	Paraserianthes falcataria (L.) I.C.	Leguminosae / Fabaceae	0,33	16,9	12	15	0,04	0,02	85,75	0,09	1,01	3,70
311	7	2	Banjaranyar	<200	Sengon	Paraserianthes falcataria (L.) I.C.	Leguminosae / Fabaceae	0,33	21,0	12	15	0,04	0,03	128,17	0,13	1,51	5,53
312	7	2	Banjaranyar	<200	Mahoni	Swietenia macrophylla King	Meliaceae	0,61	8,0	5	8	0,04	0,00	21,37	0,02	0,25	0,92
313	7	2	Banjaranyar	<200	Kelapa	Cocos nucifera L.	Arecaceae	0,5	27,4	10	13	0,04	0,06	104,60	0,10	1,23	4,51
314	7	2	Banjaranyar	<200	Sengon	Paraserianthes falcataria (L.) I.C.	Leguminosae / Fabaceae	0,33	16,5	12	15	0,04	0,02	82,81	0,08	0,97	3,57
315	7	2	Banjaranyar	<200	Sengon	Paraserianthes falcataria (L.) I.C.	Leguminosae / Fabaceae	0,33	23,9	13	15	0,04	0,04	161,99	0,16	1,90	6,99
316	7	2	Banjaranyar	<200	Sengon	Paraserianthes falcataria (L.) I.C.	Leguminosae / Fabaceae	0,33	13,4	13	14	0,04	0,01	52,57	0,05	0,62	2,27
317	7	2	Banjaranyar	<200	Mahoni	Swietenia macrophylla King	Meliaceae	0,61	21,0	4	9	0,04	0,03	140,92	0,14	1,66	6,08
318	7	2	Banjaranyar	<200	Mahoni	Swietenia macrophylla King	Meliaceae	0,61	8,0	4	7	0,04	0,00	18,91	0,02	0,22	0,82
319	7	2	Banjaranyar	<200	Kelapa	Cocos nucifera L.	Arecaceae	0,5	24,8	10	13	0,04	0,05	104,60	0,10	1,23	4,51
320	7	2	Banjaranyar	<200	Tisuk	Hibiscus macrophyllus Roxb. ex	Malvaceae	0,4	12,1	10	14	0,04	0,01	52,19	0,05	0,61	2,25
321	7	2	Banjaranyar	<200	Durian	Durio zibethinus L.	Malvaceae	0,57	44,5	10	16	0,04	0,16	889,57	0,89	10,45	38,36
322	7	2	Banjaranyar	<200	Tisuk	Hibiscus macrophyllus Roxb. ex	Malvaceae	0,4	9,2	6	9	0,04	0,01	21,22	0,02	0,25	0,92
323	7	2	Banjaranyar	<200	Manggis	Mangostana garcinia Gaertn.	Clusiaceae	0,81	2,5	0,5	2	0,04	0,00	0,96	0,00	0,01	0,04
324	7	2	Banjaranyar	<200	Nangka	Artocarpus heterophyllus Lam.	Moraceae	0,61	17,2	5	9	0,04	0,02	97,57	0,10	1,15	4,21
325	7	2	Banjaranyar	<200	Mahoni	Swietenia macrophylla King	Meliaceae	0,61	7,6	4	8	0,04	0,00	19,83	0,02	0,23	0,85
326	7	2	Banjaranyar	<200	Mahoni	Swietenia macrophylla King	Meliaceae	0,61	10,2	4	8	0,04	0,01	33,59	0,03	0,39	1,45
327	7	2	Banjaranyar	<200	Kelapa	Cocos nucifera L.	Arecaceae	0,5	22,3	12	15	0,04	0,04	120,00	0,12	1,41	5,17
328	7	2	Banjaranyar	<200	Mahoni	Swietenia macrophylla King	Meliaceae	0,61	5,4	3	4	0,04	0,00	5,59	0,01	0,07	0,24
329	7	2	Banjaranyar	<200	Tisuk	Hibiscus macrophyllus Roxb. ex	Malvaceae	0,4	11,8	8	10	0,04	0,01	36,52	0,04	0,43	1,57
330	7	2	Banjaranyar	<200	Manggis	Mangostana garcinia Gaertn.	Clusiaceae	0,81	2,5	0,5	2,5	0,04	0,00	1,18	0,00	0,01	0,05
331	7	2	Banjaranyar	<200	Mahoni	Swietenia macrophylla King	Meliaceae	0,61	7,0	3	6	0,04	0,00	12,99	0,01	0,15	0,56
332	7	2	Banjaranyar	<200	Kelapa	Cocos nucifera L.	Arecaceae	0,5	18,8	8	11	0,04	0,03	89,20	0,09	1,05	3,85
333	7	2	Banjaranyar	<200	Kelapa	Cocos nucifera L.	Arecaceae	0,5	27,4	10	12	0,04	0,06	96,90	0,10	1,14	4,18
334	7	2	Banjaranyar	<200	Mahoni	Swietenia macrophylla King	Meliaceae	0,61	24,2	4	10	0,04	0,05	200,97	0,20	2,36	8,67
335	7	2	Banjaranyar	<200	Tisuk	Hibiscus macrophyllus Roxb. ex	Malvaceae	0,4	15,9	11	13	0,04	0,02	80,63	0,08	0,95	3,48
336	7	2	Banjaranyar	<200	Sengon	Paraserianthes falcataria (L.) I.C.	Leguminosae / Fabaceae	0,33	19,7	12	14	0,04	0,03	107,30	0,11	1,26	4,63
337	7	2	Banjaranyar	<200	Mahoni	Swietenia macrophylla King	Meliaceae	0,61	8,0	4	7	0,04	0,00	18,91	0,02	0,22	0,82
338	7	2	Banjaranyar	<200	Dukuh	Lansium domesticum Corrêa	Meliaceae	0,85	4,8	3	5	0,04	0,00	7,38	0,01	0,09	0,32
339	7	2	Banjaranyar	<200	Jati	Tectona grandis L.f.	Lamiaceae / Verbenaceae	0,7	20,0	4	9	0,04	0,03	146,79	0,15	1,72	6,33
340	7	2	Banjaranyar	<200	Mahoni	Swietenia macrophylla King	Meliaceae	0,61	20,7	6	9	0,04	0,03	137,03	0,14	1,61	5,91
341	7	2	Banjaranyar	<200	Manggis	Mangostana garcinia Gaertn.	Clusiaceae	0,81	2,5	0,5	2	0,04	0,00	0,96	0,00	0,01	0,04
342	7	2	Banjaranyar	<200	Kelapa	Cocos nucifera L.	Arecaceae	0,5	24,8	12	15	0,04	0,05	120,00	0,12	1,41	5,17
343	7	2	Banjaranyar	<200	Mahoni	Swietenia macrophylla King	Meliaceae	0,61	7,6	4	7	0,04	0,00	17,54	0,02	0,21	0,76
344	7	2	Banjaranyar	<200	Tisuk	Hibiscus macrophyllus Roxb. ex	Malvaceae	0,4	17,8	10	14	0,04	0,02	106,20	0,11	1,25	4,58
345	7	2	Banjaranyar	<200	Petai	Parkia speciosa Hassk.	Leguminosae / Fabaceae	0,45	8,0	4	3	0,04	0,00	6,58	0,01	0,08	0,28
346	8	2	Banjaranyar	<200	Pala	Myristica fragrans Houtt.	Myristicaceae	0,5	5,4	1	4	0,04	0,00	4,66	0,00	0,05	0,20
347	8	2	Banjaranyar	<200	Pala	Myristica fragrans Houtt.	Myristicaceae	0,5	11,5	1,5	6,5	0,04	0,01	28,72	0,03	0,34	1,24
348	8	2	Banjaranyar	<200	Petai	Parkia speciosa Hassk.	Leguminosae / Fabaceae	0,45	8,0	4	6	0,04	0,00	12,42	0,01	0,15	0,54
349	8	2	Banjaranyar	<200	Kelapa	Cocos nucifera L.	Arecaceae	0,5	28,0	2	5	0,04	0,06	43,00	0,04	0,51	1,85
350	8	2	Banjaranyar	<200	Pala	Myristica fragrans Houtt.	Myristicaceae	0,5	14,3	2	7	0,04	0,02	46,26	0,05	0,54	1,99
351	8	2	Banjaranyar	<200	Cengkeh	Syzygium aromaticum (L.) Merr. &	Myrtaceae	0,79	37,5	7	11	0,04	0,11	622,23	0,62	7,31	26,83
352	8	2	Banjaranyar	<200	Manggis	Mangostana garcinia Gaertn.	Clusiaceae	0,81	20,7	2	9	0,04	0,03	177,68	0,18	2,09	7,66
353	8	2	Banjaranyar	<200	Sengon	Paraserianthes falcataria (L.) I.C.	Leguminosae / Fabaceae	0,33	3,8	3	4,5	0,04	0,00	1,87	0,00	0,02	0,08
354	8	2	Banjaranyar	<200	Sengon	Paraserianthes falcataria (L.) I.C.	Leguminosae / Fabaceae	0,33	34,4	12	18	0,04	0,09	373,37	0,37	4,39	16,10
355	8	2	Banjaranyar	<200	Pala	Myristica fragrans Houtt.	Myristicaceae	0,5	4,8	1	3	0,04	0,00	2,84	0,00	0,03	0,12
356	8	2	Banjaranyar	<200	Pala	Myristica fragrans Houtt.	Myristicaceae	0,5	4,8	1,8	4	0,04	0,00	3,70	0,00	0,04	0,16
357	8	2	Banjaranyar	<200	Sirsak	Annona muricata L.	Annonaceae	0,36	3,2	0,5	2	0,04	0,00	0,69	0,00	0,01	0,03
358	8	2	Banjaranyar	<200	Pala	Myristica fragrans Houtt.	Myristicaceae	0,5	3,5	1	3	0,04	0,00	1,61	0,00	0,02	0,07
359	8	2	Banjaranyar	<200	Cengkeh	Syzygium aromaticum (L.) Merr. &	Myrtaceae	0,79	3,2	1	3	0,04	0,00	2,06	0,00	0,02	0,09
360	8	2	Banjaranyar	<200	Cempedak	Artocarpus integer (Thunb.) Merr	Moraceae	0,56	2,9	1	2	0,04	0,00	0,85	0,00	0,01	0,04
361	8	2	Banjaranyar	<200	Jengkol	Archidendron pauciflorum (Benth	Leguminosae / Fabaceae	0,4	2,2	1,8	2	0,04	0,00	0,40	0,00	0,00	0,02
362	8	2	Banjaranyar	<200	Durian	Durio zibethinus L.	Malvaceae	0,57	44,9	8	15	0,04	0,16	849,51	0,85	9,98	36,63
363	8	2	Banjaranyar	<200	Pala	Myristica fragrans Houtt.	Myristicaceae	0,5	7,6	1,8	5	0,04	0,00	10,74	0,01	0,13	0,46
364	8	2	Banjaranyar	<200	Pala	Myristica fragrans Houtt.	Myristicaceae	0,5	11,1	1,8	7	0,04	0,01	29,19	0,03	0,34	1,26
365	8	2	Banjaranyar	<200	Petai	Parkia speciosa Hassk.	Leguminosae / Fabaceae	0,45	5,1	3	4	0,04	0,00	3,78	0,00	0,04	0,16
366	8	2	Banjaranyar	<200	Manggis	Mangostana garcinia Gaertn.	Clusiaceae	0,81	7,6	1,5	4,5	0,04	0,00	15,18	0,02	0,18	0,65

367	8	2	Banjaranyar	<200	Sengon	Paraserianthes falcataria (L.) I.C.	Leguminosae / Fabaceae	0,33	31,2	10	16	0,04	0,08	280,53	0,28	3,30	12,10
368	8	2	Banjaranyar	<200	Petai	Parkia speciosa Hassk.	Leguminosae / Fabaceae	0,45	10,8	3	4,5	0,04	0,01	16,77	0,02	0,20	0,72
369	8	2	Banjaranyar	<200	Kelapa	Cocos nucifera L.	Arecaceae	0,5	25,8	6	8	0,04	0,05	66,10	0,07	0,78	2,85
370	8	2	Banjaranyar	<200	Pala	Myristica fragrans Houtt.	Myristicaceae	0,5	10,5	1,5	6	0,04	0,01	22,76	0,02	0,27	0,98
371	9	1	Banjaranyar	<200	Sengon	Paraserianthes falcataria (L.) I.C.	Leguminosae / Fabaceae	0,33	18,1	10	15	0,04	0,03	97,98	0,10	1,15	4,23
372	9	1	Banjaranyar	<200	Aren	Arenga pinnata (Wurmb) Merr.	Arecaceae	0,71	45,8	2	7	0,04	0,16	58,40	0,06	0,69	2,52
373	9	1	Banjaranyar	<200	Kelapa	Cocos nucifera L.	Arecaceae	0,5	22,3	8	11	0,04	0,04	89,20	0,09	1,05	3,85
374	9	1	Banjaranyar	<200	Sengon	Paraserianthes falcataria (L.) I.C.	Leguminosae / Fabaceae	0,33	16,5	8	10	0,04	0,02	57,12	0,06	0,67	2,46
375	9	1	Banjaranyar	<200	Alpukat	Persea americana Mill.	Lauraceae	0,54	7,3	2,5	5	0,04	0,00	10,66	0,01	0,13	0,46
376	9	1	Banjaranyar	<200	Sengon	Paraserianthes falcataria (L.) I.C.	Leguminosae / Fabaceae	0,33	11,8	9	11	0,04	0,01	33,41	0,03	0,39	1,44
377	9	1	Banjaranyar	<200	Sengon	Paraserianthes falcataria (L.) I.C.	Leguminosae / Fabaceae	0,33	15,6	8	11	0,04	0,02	55,90	0,06	0,66	2,41
378	9	1	Banjaranyar	<200	Sengon	Paraserianthes falcataria (L.) I.C.	Leguminosae / Fabaceae	0,33	12,7	6	13	0,04	0,01	44,92	0,04	0,53	1,94
379	9	1	Banjaranyar	<200	Sengon	Paraserianthes falcataria (L.) I.C.	Leguminosae / Fabaceae	0,33	10,8	8	14	0,04	0,01	35,69	0,04	0,42	1,54
380	9	1	Banjaranyar	<200	Sengon	Paraserianthes falcataria (L.) I.C.	Leguminosae / Fabaceae	0,33	12,7	7	12	0,04	0,01	41,74	0,04	0,49	1,80
381	9	1	Banjaranyar	<200	Sengon	Paraserianthes falcataria (L.) I.C.	Leguminosae / Fabaceae	0,33	4,5	4	6	0,04	0,00	3,23	0,00	0,04	0,14
382	9	1	Banjaranyar	<200	Sengon	Paraserianthes falcataria (L.) I.C.	Leguminosae / Fabaceae	0,33	5,7	5	7	0,04	0,00	5,90	0,01	0,07	0,25
383	9	1	Banjaranyar	<200	Kelapa	Cocos nucifera L.	Arecaceae	0,5	22,9	9	12	0,04	0,04	96,90	0,10	1,14	4,18
384	9	1	Banjaranyar	<200	Sengon	Paraserianthes falcataria (L.) I.C.	Leguminosae / Fabaceae	0,33	15,9	8	12	0,04	0,02	62,82	0,06	0,74	2,71
385	9	1	Banjaranyar	<200	Sengon	Paraserianthes falcataria (L.) I.C.	Leguminosae / Fabaceae	0,33	14,3	4	10	0,04	0,02	43,83	0,04	0,51	1,89
386	9	1	Banjaranyar	<200	Sengon	Paraserianthes falcataria (L.) I.C.	Leguminosae / Fabaceae	0,33	4,5	10	13	0,04	0,00	6,56	0,01	0,08	0,28
387	9	1	Banjaranyar	<200	Kelapa	Cocos nucifera L.	Arecaceae	0,5	24,8	10	13	0,04	0,05	104,60	0,10	1,23	4,51
388	9	1	Banjaranyar	<200	Sengon	Paraserianthes falcataria (L.) I.C.	Leguminosae / Fabaceae	0,33	10,8	10	14	0,04	0,01	35,69	0,04	0,42	1,54
389	9	1	Banjaranyar	<200	Sengon	Paraserianthes falcataria (L.) I.C.	Leguminosae / Fabaceae	0,33	7,6	6	8	0,04	0,00	11,29	0,01	0,13	0,49
390	9	1	Banjaranyar	<200	Sengon	Paraserianthes falcataria (L.) I.C.	Leguminosae / Fabaceae	0,33	17,8	10	15	0,04	0,02	94,85	0,09	1,11	4,09
391	9	1	Banjaranyar	<200	Sengon	Paraserianthes falcataria (L.) I.C.	Leguminosae / Fabaceae	0,33	10,2	9	11	0,04	0,01	25,61	0,03	0,30	1,10
392	9	1	Banjaranyar	<200	Sengon	Paraserianthes falcataria (L.) I.C.	Leguminosae / Fabaceae	0,33	4,8	4	5	0,04	0,00	3,10	0,00	0,04	0,13
393	9	1	Banjaranyar	<200	Sengon	Paraserianthes falcataria (L.) I.C.	Leguminosae / Fabaceae	0,33	10,2	7	9	0,04	0,01	21,31	0,02	0,25	0,92
394	9	1	Banjaranyar	<200	Sengon	Paraserianthes falcataria (L.) I.C.	Leguminosae / Fabaceae	0,33	7,0	6	7,5	0,04	0,00	9,08	0,01	0,11	0,39
395	9	1	Banjaranyar	<200	Tisuk	Hibiscus macrophyllus Roxb. ex I	Malvaceae	0,4	17,2	5	11	0,04	0,02	79,66	0,08	0,94	3,44
396	9	1	Banjaranyar	<200	Sengon	Paraserianthes falcataria (L.) I.C.	Leguminosae / Fabaceae	0,33	9,5	9	10	0,04	0,01	20,85	0,02	0,25	0,90
397	9	1	Banjaranyar	<200	Mahoni	Swietenia macrophylla King	Meliaceae	0,61	9,5	4	7	0,04	0,01	26,40	0,03	0,31	1,14
398	9	1	Banjaranyar	<200	Tisuk	Hibiscus macrophyllus Roxb. ex I	Malvaceae	0,4	11,8	5	9	0,04	0,01	33,16	0,03	0,39	1,43
399	9	1	Banjaranyar	<200	Kelapa	Cocos nucifera L.	Arecaceae	0,5	27,7	12	15	0,04	0,06	120,00	0,12	1,41	5,17
400	9	1	Banjaranyar	<200	Aren	Arenga pinnata (Wurmb) Merr.	Arecaceae	0,71	41,4	10	13	0,04	0,13	104,60	0,10	1,23	4,51
401	10	3	Banjaranyar	<200	Mahoni	Swietenia macrophylla King	Meliaceae	0,61	2,9	1,5	3	0,04	0,00	1,34	0,00	0,02	0,06
402	10	3	Banjaranyar	<200	Alpukat	Persea americana Mill.	Lauraceae	0,54	8,6	5	7	0,04	0,01	19,47	0,02	0,23	0,84
403	10	3	Banjaranyar	<200	Mahoni	Swietenia macrophylla King	Meliaceae	0,61	2,5	1,5	3	0,04	0,00	1,08	0,00	0,01	0,05
404	10	3	Banjaranyar	<200	Manggis	Mangostana garcinia Gaertn.	Clusiaceae	0,81	9,5	2	4	0,04	0,01	20,51	0,02	0,24	0,88
405	10	3	Banjaranyar	<200	Mahoni	Swietenia macrophylla King	Meliaceae	0,61	33,1	7	14	0,04	0,09	485,89	0,49	5,71	20,95
406	10	3	Banjaranyar	<200	Mahoni	Swietenia macrophylla King	Meliaceae	0,61	3,2	2	3	0,04	0,00	1,62	0,00	0,02	0,07
407	10	3	Banjaranyar	<200	Mahoni	Swietenia macrophylla King	Meliaceae	0,61	35,0	7	14	0,04	0,10	538,48	0,54	6,33	23,22
408	10	3	Banjaranyar	<200	Petai	Parkia speciosa Hassk.	Leguminosae / Fabaceae	0,45	10,2	3	6	0,04	0,01	19,53	0,02	0,23	0,84
409	10	3	Banjaranyar	<200	Kelapa	Cocos nucifera L.	Arecaceae	0,5	20,4	11	14	0,04	0,03	112,30	0,11	1,32	4,84
410	10	3	Banjaranyar	<200	Manggis	Mangostana garcinia Gaertn.	Clusiaceae	0,81	13,0	2	7	0,04	0,01	60,68	0,06	0,71	2,62
411	10	3	Banjaranyar	<200	Mahoni	Swietenia macrophylla King	Meliaceae	0,61	12,7	9	11	0,04	0,01	67,67	0,07	0,80	2,92
412	10	3	Banjaranyar	<200	Mahoni	Swietenia macrophylla King	Meliaceae	0,61	16,9	10	14	0,04	0,02	141,32	0,14	1,66	6,09
413	10	3	Banjaranyar	<200	Sengon	Paraserianthes falcataria (L.) I.C.	Leguminosae / Fabaceae	0,33	9,2	9	12	0,04	0,01	23,16	0,02	0,27	1,00
414	10	3	Banjaranyar	<200	Mahoni	Swietenia macrophylla King	Meliaceae	0,61	57,3	6	18	0,04	0,26	1.670,98	1,67	19,63	72,06
415	10	3	Banjaranyar	<200	Kelapa	Cocos nucifera L.	Arecaceae	0,5	25,8	10	13	0,04	0,05	104,60	0,10	1,23	4,51
416	10	3	Banjaranyar	<200	Mahoni	Swietenia macrophylla King	Meliaceae	0,61	3,8	1,5	3	0,04	0,00	2,27	0,00	0,03	0,10
417	10	3	Banjaranyar	<200	Mahoni	Swietenia macrophylla King	Meliaceae	0,61	4,1	2	3,5	0,04	0,00	3,02	0,00	0,04	0,13
418	10	3	Banjaranyar	<200	Mahoni	Swietenia macrophylla King	Meliaceae	0,61	4,5	2	3,5	0,04	0,00	3,46	0,00	0,04	0,15
419	10	3	Banjaranyar	<200	Mahoni	Swietenia macrophylla King	Meliaceae	0,61	10,8	6	8	0,04	0,01	37,53	0,04	0,44	1,62
420	10	3	Banjaranyar	<200	Kelapa	Cocos nucifera L.	Arecaceae	0,5	23,2	12	15	0,04	0,04	120,00	0,12	1,41	5,17
421	10	3	Banjaranyar	<200	Mangga	Mangifera indica L.	Anacardiaceae	0,6	20,0	4,5	6,5	0,04	0,03	94,61	0,09	1,11	4,08
422	10	3	Banjaranyar	<200	Petai	Parkia speciosa Hassk.	Leguminosae / Fabaceae	0,45	6,0	3	5	0,04	0,00	6,36	0,01	0,07	0,27
423	10	3	Banjaranyar	<200	Jengkol	Archidendron pauciflorum (Bentl	Leguminosae / Fabaceae	0,4	20,4	5	9	0,04	0,03	90,49	0,09	1,06	3,90
424	10	3	Banjaranyar	<200	Mahoni	Swietenia macrophylla King	Meliaceae	0,61	5,1	2,5	3	0,04	0,00	3,84	0,00	0,05	0,17
425	10	3	Banjaranyar	<200	Cengkeh	Syzygium aromaticum (L.) Merr. &	Myrtaceae	0,79	9,5	2,5	6	0,04	0,01	29,05	0,03	0,34	1,25
426	10	3	Banjaranyar	<200	Sengon	Paraserianthes falcataria (L.) I.C.	Leguminosae / Fabaceae	0,33	13,0	9	12	0,04	0,01	43,67	0,04	0,51	1,88
427	10	3	Banjaranyar	<200	Kelapa	Cocos nucifera L.	Arecaceae	0,5	27,7	7	10	0,04	0,06	81,50	0,08	0,96	3,51
428	10	3	Banjaranyar	<200	Petai	Parkia speciosa Hassk.	Leguminosae / Fabaceae	0,45	22,3	10	14	0,04	0,04	178,05	0,18	2,09	7,68
429	10	3	Banjaranyar	<200	Dukuh	Lansium domesticum Corrêa	Meliaceae	0,85	20,0	2	9	0,04	0,03	175,37	0,18	2,06	7,56
430	11	2	Banjaranyar	<200	Kelapa	Cocos nucifera L.	Arecaceae	0,5	25,5	15	19	0,04	0,05	150,80	0,15	1,77	6,50
431	11	2	Banjaranyar	<200	Cengkeh	Syzygium aromaticum (L.) Merr. &	Myrtaceae	0,79	24,8	3	10	0,04	0,05	267,09	0,27	3,14	11,52
432	11	2	Banjaranyar	<200	Sengon	Paraserianthes falcataria (L.) I.C.	Leguminosae / Fabaceae	0,33	12,7	10	12	0,04	0,01	41,74	0,04	0,49	1,80
433	11	2	Banjaranyar	<200	Afrika	Maesopsis eminii Engl.	Rhamnaceae	0,43	15,9	14	16	0,04	0,02	104,20	0,10	1,22	4,49
434	11	2	Banjaranyar	<200	Afrika	Maesopsis eminii Engl.	Rhamnaceae	0,43	15,6	12	15	0,04	0,02	94,65	0,09	1,11	4,08
435	11	2	Banjaranyar	<200	Mahoni	Swietenia macrophylla King	Meliaceae	0,61	11,5	6	10	0,04	0,01	51,12	0,05	0,60	2,20
436	11	2	Banjaranyar	<200	Mahoni	Swietenia macrophylla King	Meliaceae	0,61	5,7	4	7	0,04	0,00	10,36	0,01	0,12	0,45
437	11	2	Banjaranyar	<200	Mahoni	Swietenia macrophylla King	Meliaceae	0,61	6,0	5	7	0,04	0,00	11,44	0,01	0,13	0,49
438	11	2	Banjaranyar	<200	Mahoni	Swietenia macrophylla King	Meliaceae	0,61	10,2	9	11	0,04	0,01	44,96	0,04	0,53	1,94
439	11	2	Banjaranyar	<200	Petai	Parkia speciosa Hassk.	Leguminosae / Fabaceae	0,45	23,9	6	13	0,04	0,04	188,78	0,19	2,22	8,14
440	11	2	Banjaranyar	<200	Mahoni	Swietenia macrophylla King	Meliaceae	0,61	5,1	3	4,5	0,04	0,00	5,57	0,01	0,07	0,24
441	11	2	Banjaranyar	<200	Kelapa	Cocos nucifera L.	Arecaceae	0,5	30,9	12	15	0,04	0,07	120,00	0,12	1,41	5,17
442	11	2	Banjaranyar	<200	Pala	Myristica fragrans Houtt.	Myristicaceae	0,5	3,8	0,5	3	0,04	0,00	1,89	0,00	0,02	0,08
443	11	2	Banjaranyar	<200	Afrika	Maesopsis eminii Engl.	Rhamnaceae	0,43	17,2	13	15	0,04	0,02	113,09	0,11	1,33	4,88
444	11	2	Banjaranyar	<200	Manggis	Mangostana garcinia Gaertn.	Clusiaceae	0,81	5,7	0,8	3	0,04	0,00	6,18	0,01	0,07	0,27
445	11	2	Banjaranyar	<200	Jengkol	Archidendron pauciflorum (Bentl	Leguminosae / Fabaceae	0,4	4,1	2,5	3,5	0,04	0,00	2,05	0,00	0,02	0,09
446	11	2	Banjaranyar	<200	Kelapa	Cocos nucifera L.	Arecaceae	0,5	28,0	10	13	0,04	0,06	104,60	0,10	1,23	4,51
447	11	2	Banjaranyar	<200	Kelapa	Cocos nucifera L.	Arecaceae	0,5	24,2	12	15	0,04	0,05	120,00	0,12	1,41	5,17
448	11	2	Banjaranyar	<200	Pala	Myristica fragrans Houtt.	Myristicaceae	0,5	4,5	0,5	2,5	0,04	0,00	2,12	0,00	0,02	0,09
449	11	2	Banjaranyar	<200	Sengon	Paraserianthes falcataria (L.) I.C.	Leguminosae / Fabaceae	0,33	19,4	12	16	0,04	0,03	117,70	0,12	1,38	5,08
450	11	2	Banjaranyar	<200	Manggis	Mangostana garcinia Gaertn.	Clusiaceae	0,81	5,7	0,5	2,5	0,04	0,00	5,23	0,01	0,06	0,23
451	11	2	Banjaranyar	<200	Cengkeh	Syzygium aromaticum (L.) Merr. &	Myrtaceae	0,79	3,8	0,5	2	0,04	0,00	1,98	0,00	0,02	0,09
452	11	2	Banjaranyar	<200	Kelapa	Cocos nucifera L.	Arecaceae	0,5	22,3	9	12	0,04	0,04	96,90	0,10	1,14	4,18
453	11	2	Banjaranyar	<200	Pasang	Quercus sundaica Blume	Fagaceae	0,68	19,1	8	13	0,04	0,03	183,08	0,18	2,15	7,89
454	11	2	Banjaranyar	<200	Manggis	Mangostana garcinia Gaertn.	Clusiaceae	0,81	4,5	0,5	2,5	0,04	0,00	3,30	0,00	0,04	0,14
455	11	2	Banjaranyar	<200	Mahoni	Swietenia macrophylla King	Meliaceae	0,61	11,5	8	10	0,04	0,01	51,12	0,05	0,60	2,20
456	11	2	Banjaranyar	<200	Mahoni	Swietenia macrophylla King	Meliaceae	0,61	5,7	4	6	0,04	0,00	8,99	0,01	0,11	0,39
457	11	2	Banjaranyar	<200	Sengon	Paraserianthes falcataria (L.) I.C.	Leguminosae / Fabaceae	0,33	7,0	9	10	0,04	0,00	11,81	0,01	0,14	0,51
458	11	2	Banjaranyar	<200	Mahoni	Swietenia macrophylla King	Meliaceae	0,61	5,7	3	4	0,04	0,00	6,20	0,01	0,07	0,27
459	11	2	Banjaranyar	<200	Afrika	Maesopsis eminii Engl.	Rhamnaceae	0,43	13,7	10	13	0,04	0,01	65,35	0,07	0,77	2,82
460	11	2	Banjaranyar	<200	Mahoni	Swietenia macrophylla King	Meliaceae	0,61	5,7	3	4,5	0,04	0,00	6,91	0,01	0,08	0,30
461	11	2	Banjaranyar	<200	Tisuk	Hibiscus macrophyllus Roxb. ex I	Malvaceae	0,4	8,9	7	10	0,04	0,01	21,92	0,02	0,26	0,95
462	11	2	Banjaranyar	<200	Afrika	Maesopsis eminii Engl.	Rhamnaceae	0,43	8,6	9	10	0,04	0,01	21,91	0,02	0,26	0,94
463	11	2	Banjaranyar	<200	Mahoni	Swietenia macrophylla King	Meliaceae	0,61	13,7	6	9	0,04	0,01	64,28	0,06	0,76	2,77
464	11	2	Banjaranyar	<200	Sengon	Paraserianthes falcataria (L.) I.C.	Leguminosae / Fabaceae	0,33	19,1	10	14	0,04	0,03	101,04	0,10	1,19	4,36
465	11	2	Banjaranyar	<200	Mahoni	Swietenia macrophylla King	Meliaceae	0,61	9,2	5	6	0,04	0,01	21,55	0,02	0,25	0,93
466	12	1	Banjaranyar	<200	Jati	Tectona grandis L.f.	Lamiaceae / Verbenaceae	0,7	21,3	10	15	0,04	0,04	262,36	0,26	3,08	11,31
467	12	1	Banjaranyar	<200	Jati	Tectona grandis L.f.	Lamiaceae / Verbenaceae	0,7	15,6	9	12	0,04	0,02	120,56	0,12	1,42	5,20
468	12	1	Banjaranyar	<200	Jati	Tectona grandis L.f.	Lamiaceae / Verbenaceae	0,7	8,0	5	7	0,04	0,00	21,45	0,02	0,25	0,92
469	12	1	Banjaranyar	<200	Jati	Tectona grandis L.f.	Lamiaceae / Verbenaceae	0,7	20,7	10	15	0,04	0,03	248,19	0,25	2,92	10,70
470	12	1	Banjaranyar	<200	Jati	Tectona grandis L.f.	Lamiaceae / Verbenaceae	0,7	13,7	6	11	0,04	0,01	87,63	0,09	1,03	3,78
471	12	1	Banjaranyar	<200	Jati	Tectona grandis L.f.	Lamiaceae / Verbenaceae	0,7	15,3	10	15	0,04	0,02	142,42	0,14	1,67	6,14
472	12	1	Banjaranyar	<200	Mahoni	Swietenia macrophylla King	Meliaceae	0,61	7,0	4	5	0,04	0,00	10,99	0,01	0,13	0,47
473	12	1	Banjaranyar	<200	Jati	Tectona grandis L.f.	Lamiaceae / Verbenaceae	0,7	9,9	5	7	0,04	0,01	31,81	0,03	0,37	1,37
474	12	1	Banjaranyar	<200	Jati	Tectona grandis L.f.	Lamiaceae / Verbenaceae	0,7	11,5	9	12	0,04	0,01	68,53	0,07	0,81	2,96
475	12	1	Banjaranyar	<200	Jati	Tectona grandis L.f.	Lamiaceae / Verbenaceae	0,7	8,9	5	10	0,04	0,01	36,60	0,04	0,43	1,58
476	12	1	Banjaranyar	<200	Jati	Tectona grandis L.f.	Lamiaceae / Verbenaceae	0,7	19,7	10	14	0,04	0,03	213,67	0,21	2,51	9,21
477	12	1	Banjaranyar	<200	Jati	Tectona grandis L.f.	Lamiaceae / Verbenaceae	0,7	18,1	9	15	0,04	0,03	195,12	0,20	2,29	8,41
478	12	1	Banjaranyar	<200	Jati	Tectona grandis L.f.	Lamiaceae / Verbenaceae	0,7	17,2	9	15	0,04	0,02	176,72	0,18	2,08	7,62
479	12	1	Banjaranyar	<200	Mahoni	Swietenia macrophylla King	Meliaceae	0,61	4,5	3	4	0,04	0,00	3,91	0,00	0,05	0,17
480	12	1	Banjaranyar	<200	Jati	Tectona grandis L.f.	Lamiaceae / Verbenaceae	0,7	12,4	6	10	0,04	0,01	67,15	0,07	0,79	2,90
481	12	1	Banjaranyar	<200	Mahoni	Swietenia macrophylla King	Meliaceae	0,61	7,3	4	7	0,04	0,00	16,23	0,02	0,19	0,70
482	12	1	Banjaranyar	<200	Mahoni	Swietenia macrophylla King	Meliaceae	0,61	27,4	7	14	0,04	0,06	343,03	0,34	4,03	14,79
483	12	1	Banjaranyar	<200	Jati	Tectona grandis L.f.	Lamiaceae / Verbenaceae	0,7	8,0	5	8	0,04	0,00	24,24	0,02	0,28	1,05
484	12	1	Banjaranyar	<200	Jati	Tectona grandis L.f.	Lamiaceae / Verbenaceae	0,7	7,6	4	7	0,04	0,00	19,90	0,02	0,23	0,86
485	12	1	Banjaranyar	<200	Jati	Tectona grandis L.f.	Lamiaceae / Verbenaceae	0,7	7,0	3	6	0,04	0,00	14,73	0,01	0,17	0,64
486	12	1	Banjaranyar	<200	Jati	Tectona grandis L.f.	Lamiaceae / Verbenaceae	0,7	12,7	6	8	0,04	0,01	57,34	0,06	0,67	2,47
487	12	1	Banjaranyar	<200	Jati	Tectona grandis L.f.	Lamiaceae / Verbenaceae	0,7	19,4	8	14	0,04	0,03	207,40	0,21	2,44	8,94
488	12	1	Banjaranyar	<200	jati	Tectona grandis L.f.	Lamiaceae / Verbenaceae	0,7	8,9	6	9	0,04	0,01	33,23	0,03	0,39	1,43
489	12	1	Banjaranyar	<200	Mahoni	Swietenia macrophylla King	Meliaceae	0,61	21,0	7	10	0,04	0,03	155,20	0,16	1,82	6,69
490	12	1	Banjaranyar	<200	Jati	Tectona grandis L.f.	Lamiaceae / Verbenaceae	0,7	6,4	4	9	0,04	0,00	17,94	0,02	0,21	0,77

491	12	1	Banjaranyar	<200	Jati	Tectona grandis L.f.	Lamiaceae / Verbenaceae	0,7	15,9	8	14	0,04	0,02	144,08	0,14	1,69	6,21
492	12	1	Banjaranyar	<200	Jati	Tectona grandis L.f.	Lamiaceae / Verbenaceae	0,7	21,0	10	15	0,04	0,03	255,23	0,26	3,00	11,01
493	12	1	Banjaranyar	<200	Jati	Tectona grandis L.f.	Lamiaceae / Verbenaceae	0,7	14,6	8	12	0,04	0,02	107,38	0,11	1,26	4,63
494	12	1	Banjaranyar	<200	Jati	Tectona grandis L.f.	Lamiaceae / Verbenaceae	0,7	12,1	5	9	0,04	0,01	58,14	0,06	0,68	2,51
495	12	1	Banjaranyar	<200	Jati	Tectona grandis L.f.	Lamiaceae / Verbenaceae	0,7	12,7	6	9	0,04	0,01	63,87	0,06	0,75	2,75
496	12	1	Banjaranyar	<200	Jati	Tectona grandis L.f.	Lamiaceae / Verbenaceae	0,7	18,5	10	13	0,04	0,03	176,69	0,18	2,08	7,62
497	12	1	Banjaranyar	<200	Jati	Tectona grandis L.f.	Lamiaceae / Verbenaceae	0,7	22,6	9	13	0,04	0,04	255,92	0,26	3,01	11,04
498	12	1	Banjaranyar	<200	Jati	Tectona grandis L.f.	Lamiaceae / Verbenaceae	0,7	11,1	7	10	0,04	0,01	55,08	0,06	0,65	2,38
499	12	1	Banjaranyar	<200	Jati	Tectona grandis L.f.	Lamiaceae / Verbenaceae	0,7	9,5	5	8	0,04	0,01	33,85	0,03	0,40	1,46
500	12	1	Banjaranyar	<200	Mahoni	Swietenia macrophylla King	Meliaceae	0,61	11,8	6	10	0,04	0,01	53,76	0,05	0,63	2,32
501	12	1	Banjaranyar	<200	Jati	Tectona grandis L.f.	Lamiaceae / Verbenaceae	0,7	9,5	6	10	0,04	0,01	41,53	0,04	0,49	1,79
502	12	1	Banjaranyar	<200	Jati	Tectona grandis L.f.	Lamiaceae / Verbenaceae	0,7	11,5	9	12	0,04	0,01	68,53	0,07	0,81	2,96
503	12	1	Banjaranyar	<200	Tisuk	Hibiscus macrophyllus Roxb. ex I	Malvaceae	0,4	12,1	10	11	0,04	0,01	41,85	0,04	0,49	1,80
504	12	1	Banjaranyar	<200	Jati	Tectona grandis L.f.	Lamiaceae / Verbenaceae	0,7	6,4	6	8	0,04	0,00	16,10	0,02	0,19	0,69
505	12	1	Banjaranyar	<200	Durian	Durio zibethinus L.	Malvaceae	0,57	20,7	10	13	0,04	0,03	180,36	0,18	2,12	7,78
506	12	1	Banjaranyar	<200	Kelapa	Cocos nucifera L.	Arecaceae	0,5	22,9	3	6	0,04	0,04	50,70	0,05	0,60	2,19
507	12	1	Banjaranyar	<200	Jati	Tectona grandis L.f.	Lamiaceae / Verbenaceae	0,7	19,7	9	14	0,04	0,03	213,67	0,21	2,51	9,21
508	12	1	Banjaranyar	<200	Jati	Tectona grandis L.f.	Lamiaceae / Verbenaceae	0,7	10,8	9	12	0,04	0,01	61,72	0,06	0,73	2,66
509	12	1	Banjaranyar	<200	Jati	Tectona grandis L.f.	Lamiaceae / Verbenaceae	0,7	23,2	9	13	0,04	0,04	269,28	0,27	3,16	11,61
510	12	1	Banjaranyar	<200	Jati	Tectona grandis L.f.	Lamiaceae / Verbenaceae	0,7	9,5	7	11	0,04	0,01	45,31	0,05	0,53	1,95
511	12	1	Banjaranyar	<200	Jati	Tectona grandis L.f.	Lamiaceae / Verbenaceae	0,7	19,1	9	14	0,04	0,03	201,21	0,20	2,36	8,68
512	12	1	Banjaranyar	<200	Kelapa	Cocos nucifera L.	Arecaceae	0,5	24,8	4	7	0,04	0,05	58,40	0,06	0,69	2,52
513	12	1	Banjaranyar	<200	Jati	Tectona grandis L.f.	Lamiaceae / Verbenaceae	0,7	17,5	10	14	0,04	0,02	171,56	0,17	2,02	7,40
514	12	1	Banjaranyar	<200	Jati	Tectona grandis L.f.	Lamiaceae / Verbenaceae	0,7	9,9	6	9	0,04	0,01	40,04	0,04	0,47	1,73
515	12	1	Banjaranyar	<200	Jati	Tectona grandis L.f.	Lamiaceae / Verbenaceae	0,7	20,4	10	15	0,04	0,03	241,24	0,24	2,83	10,40
516	12	1	Banjaranyar	<200	Jati	Tectona grandis L.f.	Lamiaceae / Verbenaceae	0,7	14,6	9	12	0,04	0,02	107,38	0,11	1,26	4,63
517	12	1	Banjaranyar	<200	Jati	Tectona grandis L.f.	Lamiaceae / Verbenaceae	0,7	22,0	12	15	0,04	0,04	276,89	0,28	3,25	11,94
518	12	1	Banjaranyar	<200	Jati	Tectona grandis L.f.	Lamiaceae / Verbenaceae	0,7	21,6	11	14	0,04	0,04	253,07	0,25	2,97	10,91
519	12	1	Banjaranyar	<200	Jati	Tectona grandis L.f.	Lamiaceae / Verbenaceae	0,7	9,2	5	8	0,04	0,01	31,81	0,03	0,37	1,37
520	12	1	Banjaranyar	<200	Jati	Tectona grandis L.f.	Lamiaceae / Verbenaceae	0,7	15,0	8	12	0,04	0,02	111,70	0,11	1,31	4,82
521	12	1	Banjaranyar	<200	Jati	Tectona grandis L.f.	Lamiaceae / Verbenaceae	0,7	14,0	7	10	0,04	0,02	83,76	0,08	0,98	3,61
522	12	1	Banjaranyar	<200	Jati	Tectona grandis L.f.	Lamiaceae / Verbenaceae	0,7	16,5	9	12	0,04	0,02	134,42	0,13	1,58	5,80
523	12	1	Banjaranyar	<200	Jati	Tectona grandis L.f.	Lamiaceae / Verbenaceae	0,7	18,5	8	13	0,04	0,03	176,69	0,18	2,08	7,62
524	12	1	Banjaranyar	<200	Jati	Tectona grandis L.f.	Lamiaceae / Verbenaceae	0,7	12,7	7	12	0,04	0,01	83,13	0,08	0,98	3,58
525	12	1	Banjaranyar	<200	Jati	Tectona grandis L.f.	Lamiaceae / Verbenaceae	0,7	10,8	5	10	0,04	0,01	52,23	0,05	0,61	2,25
526	12	1	Banjaranyar	<200	Jati	Tectona grandis L.f.	Lamiaceae / Verbenaceae	0,7	16,2	9	14	0,04	0,02	149,40	0,15	1,76	6,44
527	12	1	Banjaranyar	<200	Jati	Tectona grandis L.f.	Lamiaceae / Verbenaceae	0,7	8,0	5	7	0,04	0,00	21,45	0,02	0,25	0,92
528	12	1	Banjaranyar	<200	Jati	Tectona grandis L.f.	Lamiaceae / Verbenaceae	0,7	19,4	10	14	0,04	0,03	207,40	0,21	2,44	8,94
529	12	1	Banjaranyar	<200	Jati	Tectona grandis L.f.	Lamiaceae / Verbenaceae	0,7	10,5	6	10	0,04	0,01	49,45	0,05	0,58	2,13
530	12	1	Banjaranyar	<200	Mahoni	Swietenia macrophylla King	Meliaceae	0,61	3,2	3	4	0,04	0,00	2,11	0,00	0,02	0,09
531	12	1	Banjaranyar	<200	Jati	Tectona grandis L.f.	Lamiaceae / Verbenaceae	0,7	10,2	6	10	0,04	0,01	46,74	0,05	0,55	2,02
532	12	1	Banjaranyar	<200	Jati	Tectona grandis L.f.	Lamiaceae / Verbenaceae	0,7	12,1	6	8	0,04	0,01	52,19	0,05	0,61	2,25
533	12	1	Banjaranyar	<200	Jati	Tectona grandis L.f.	Lamiaceae / Verbenaceae	0,7	21,3	10	14	0,04	0,04	246,29	0,25	2,89	10,62
534	12	1	Banjaranyar	<200	Jati	Tectona grandis L.f.	Lamiaceae / Verbenaceae	0,7	8,9	5	7	0,04	0,01	26,40	0,03	0,31	1,14
535	12	1	Banjaranyar	<200	Jati	Tectona grandis L.f.	Lamiaceae / Verbenaceae	0,7	11,5	6	11	0,04	0,01	63,28	0,06	0,74	2,73
536	12	1	Banjaranyar	<200	Jati	Tectona grandis L.f.	Lamiaceae / Verbenaceae	0,7	8,9	4	5	0,04	0,01	19,39	0,02	0,23	0,84
537	13	1	Banjaranyar	<200	Sengon	Paraserianthes falcataria (L.) I.C	Leguminosae / Fabaceae	0,33	7,3	6	8	0,04	0,00	10,45	0,01	0,12	0,45
538	13	1	Banjaranyar	<200	Sengon	Paraserianthes falcataria (L.) I.C	Leguminosae / Fabaceae	0,33	3,8	4	5,5	0,04	0,00	2,25	0,00	0,03	0,10
539	13	1	Banjaranyar	<200	Sengon	Paraserianthes falcataria (L.) I.C	Leguminosae / Fabaceae	0,33	4,5	4	5	0,04	0,00	2,74	0,00	0,03	0,12
540	13	1	Banjaranyar	<200	Sengon	Paraserianthes falcataria (L.) I.C	Leguminosae / Fabaceae	0,33	15,3	10	12	0,04	0,02	58,30	0,06	0,68	2,51
541	13	1	Banjaranyar	<200	Sengon	Paraserianthes falcataria (L.) I.C	Leguminosae / Fabaceae	0,33	10,5	9	11	0,04	0,01	27,10	0,03	0,32	1,17
542	13	1	Banjaranyar	<200	Cengkeh	Syzygium aromaticum (L.) Merr. a	Myrtaceae	0,79	2,5	1	3	0,04	0,00	1,37	0,00	0,02	0,06
543	13	1	Banjaranyar	<200	Manggis	Mangostana garcinia Gaertn.	Clusiaceae	0,81	3,8	0,5	3	0,04	0,00	2,94	0,00	0,03	0,13
544	13	1	Banjaranyar	<200	Sengon	Paraserianthes falcataria (L.) I.C	Leguminosae / Fabaceae	0,33	6,0	4	6	0,04	0,00	5,66	0,01	0,07	0,24
545	13	1	Banjaranyar	<200	Sengon	Paraserianthes falcataria (L.) I.C	Leguminosae / Fabaceae	0,33	4,5	4	5	0,04	0,00	2,74	0,00	0,03	0,12
546	13	1	Banjaranyar	<200	Sengon	Paraserianthes falcataria (L.) I.C	Leguminosae / Fabaceae	0,33	15,9	7	10	0,04	0,02	53,16	0,05	0,62	2,29
547	13	1	Banjaranyar	<200	Sengon	Paraserianthes falcataria (L.) I.C	Leguminosae / Fabaceae	0,33	7,6	6	7,5	0,04	0,00	10,65	0,01	0,13	0,46
548	13	1	Banjaranyar	<200	Tisuk	Hibiscus macrophyllus Roxb. ex I	Malvaceae	0,4	3,5	1,8	3	0,04	0,00	1,31	0,00	0,02	0,06
549	13	1	Banjaranyar	<200	Sengon	Paraserianthes falcataria (L.) I.C	Leguminosae / Fabaceae	0,33	14,0	5	8	0,04	0,02	34,29	0,03	0,40	1,48
550	13	1	Banjaranyar	<200	Sengon	Paraserianthes falcataria (L.) I.C	Leguminosae / Fabaceae	0,33	5,7	5	6	0,04	0,00	5,12	0,01	0,06	0,22
551	13	1	Banjaranyar	<200	Sengon	Paraserianthes falcataria (L.) I.C	Leguminosae / Fabaceae	0,33	7,3	5	6	0,04	0,00	8,03	0,01	0,09	0,35
552	13	1	Banjaranyar	<200	Sengon	Paraserianthes falcataria (L.) I.C	Leguminosae / Fabaceae	0,33	6,4	5	6	0,04	0,00	6,21	0,01	0,07	0,27
553	13	1	Banjaranyar	<200	Sengon	Paraserianthes falcataria (L.) I.C	Leguminosae / Fabaceae	0,33	6,7	5	6	0,04	0,00	6,79	0,01	0,08	0,29
554	13	1	Banjaranyar	<200	Sengon	Paraserianthes falcataria (L.) I.C	Leguminosae / Fabaceae	0,33	9,5	5	7	0,04	0,01	15,04	0,02	0,18	0,65
555	13	1	Banjaranyar	<200	Sengon	Paraserianthes falcataria (L.) I.C	Leguminosae / Fabaceae	0,33	5,4	5	7	0,04	0,00	5,31	0,01	0,06	0,23
556	13	1	Banjaranyar	<200	Sengon	Paraserianthes falcataria (L.) I.C	Leguminosae / Fabaceae	0,33	5,7	5	6	0,04	0,00	5,12	0,01	0,06	0,22
557	13	1	Banjaranyar	<200	Karet	Hevea brasiliensis (Willd. ex A.Ju	Euphorbiaceae	0,53	2,2	1	2	0,04	0,00	0,51	0,00	0,01	0,02
558	13	1	Banjaranyar	<200	Manggis	Mangostana garcinia Gaertn.	Clusiaceae	0,81	15,3	1,8	5	0,04	0,02	59,51	0,06	0,70	2,57
559	13	1	Banjaranyar	<200	Tisuk	Hibiscus macrophyllus Roxb. ex I	Malvaceae	0,4	3,2	2	3	0,04	0,00	1,10	0,00	0,01	0,05
560	13	1	Banjaranyar	<200	Tisuk	Hibiscus macrophyllus Roxb. ex I	Malvaceae	0,4	3,2	2	3	0,04	0,00	1,10	0,00	0,01	0,05
561	13	1	Banjaranyar	<200	Cengkeh	Syzygium aromaticum (L.) Merr. a	Myrtaceae	0,79	2,2	1,8	2,5	0,04	0,00	0,91	0,00	0,01	0,04
562	13	1	Banjaranyar	<200	Sengon	Paraserianthes falcataria (L.) I.C	Leguminosae / Fabaceae	0,33	12,1	5	7	0,04	0,01	23,19	0,02	0,27	1,00
563	13	1	Banjaranyar	<200	Sengon	Paraserianthes falcataria (L.) I.C	Leguminosae / Fabaceae	0,33	6,4	4	6	0,04	0,00	6,21	0,01	0,07	0,27
564	13	1	Banjaranyar	<200	Sengon	Paraserianthes falcataria (L.) I.C	Leguminosae / Fabaceae	0,33	7,6	4	7	0,04	0,00	9,99	0,01	0,12	0,43
565	13	1	Banjaranyar	<200	Manggis	Mangostana garcinia Gaertn.	Clusiaceae	0,81	12,4	1	5	0,04	0,01	40,68	0,04	0,48	1,75
566	13	1	Banjaranyar	<200	Sengon	Paraserianthes falcataria (L.) I.C	Leguminosae / Fabaceae	0,33	3,8	4	5	0,04	0,00	2,06	0,00	0,02	0,09
567	13	1	Banjaranyar	<200	Sengon	Paraserianthes falcataria (L.) I.C	Leguminosae / Fabaceae	0,33	8,0	5	7	0,04	0,00	10,77	0,01	0,13	0,46
568	13	1	Banjaranyar	<200	Sengon	Paraserianthes falcataria (L.) I.C	Leguminosae / Fabaceae	0,33	5,1	4	6	0,04	0,00	4,13	0,00	0,05	0,18
569	13	1	Banjaranyar	<200	Sengon	Paraserianthes falcataria (L.) I.C	Leguminosae / Fabaceae	0,33	9,2	7	10	0,04	0,01	19,60	0,02	0,23	0,85
570	13	1	Banjaranyar	<200	Kelapa	Cocos nucifera L.	Arecaceae	0,5	23,2	9	12	0,04	0,04	96,90	0,10	1,14	4,18
571	13	1	Banjaranyar	<200	Sengon	Paraserianthes falcataria (L.) I.C	Leguminosae / Fabaceae	0,33	5,4	5	7	0,04	0,00	5,31	0,01	0,06	0,23
572	13	1	Banjaranyar	<200	Karet	Hevea brasiliensis (Willd. ex A.Ju	Euphorbiaceae	0,53	5,1	4	5	0,04	0,00	5,39	0,01	0,06	0,23
573	13	1	Banjaranyar	<200	Kueni	Mangifera odorata Griff.	Anacardiaceae	0,47	14,6	3,5	6,5	0,04	0,02	42,52	0,04	0,50	1,83
574	13	1	Banjaranyar	<200	Sengon	Paraserianthes falcataria (L.) I.C	Leguminosae / Fabaceae	0,33	10,2	8	10	0,04	0,01	23,47	0,02	0,28	1,01
575	13	1	Banjaranyar	<200	Sengon	Paraserianthes falcataria (L.) I.C	Leguminosae / Fabaceae	0,33	16,9	8	10	0,04	0,02	59,15	0,06	0,70	2,55
576	13	1	Banjaranyar	<200	Cempedak	Artocarpus integer (Thunb.) Merr	Moraceae	0,56	14,0	3	7	0,04	0,02	49,25	0,05	0,58	2,12
577	13	1	Banjaranyar	<200	Kelapa	Cocos nucifera L.	Arecaceae	0,5	22,3	8	11	0,04	0,04	89,20	0,09	1,05	3,85
578	13	1	Banjaranyar	<200	Durian	Durio zibethinus L.	Malvaceae	0,57	7,6	1,8	6	0,04	0,00	14,32	0,01	0,17	0,62
579	13	1	Banjaranyar	<200	Petai	Parkia speciosa Hassk.	Leguminosae / Fabaceae	0,45	22,0	8	11	0,04	0,04	139,04	0,14	1,63	6,00
580	13	1	Banjaranyar	<200	Sengon	Paraserianthes falcataria (L.) I.C	Leguminosae / Fabaceae	0,33	11,5	7	9	0,04	0,01	26,44	0,03	0,31	1,14
581	13	1	Banjaranyar	<200	Sengon	Paraserianthes falcataria (L.) I.C	Leguminosae / Fabaceae	0,33	10,8	6	7,5	0,04	0,01	20,15	0,02	0,24	0,87
582	13	1	Banjaranyar	<200	Karet	Hevea brasiliensis (Willd. ex A.Ju	Euphorbiaceae	0,53	3,5	3	4,5	0,04	0,00	2,46	0,00	0,03	0,11
583	13	1	Banjaranyar	<200	Sengon	Paraserianthes falcataria (L.) I.C	Leguminosae / Fabaceae	0,33	12,4	9	11	0,04	0,01	36,80	0,04	0,43	1,59
584	13	1	Banjaranyar	<200	Manggis	Mangostana garcinia Gaertn.	Clusiaceae	0,81	18,5	1	6	0,04	0,03	99,47	0,10	1,17	4,29
585	13	1	Banjaranyar	<200	Sengon	Paraserianthes falcataria (L.) I.C	Leguminosae / Fabaceae	0,33	8,6	4	7	0,04	0,01	12,40	0,01	0,15	0,53
586	13	1	Banjaranyar	<200	Karet	Hevea brasiliensis (Willd. ex A.Ju	Euphorbiaceae	0,53	6,4	5	6	0,04	0,00	9,59	0,01	0,11	0,41
587	14	3	Banjaranyar	<200	Mahoni afrika	Khaya anthotheca (Welw.) C.DC.	Meliaceae	0,49	19,7	9	12	0,04	0,03	133,82	0,13	1,57	5,77
588	14	3	Banjaranyar	<200	Sengon	Paraserianthes falcataria (L.) I.C	Leguminosae / Fabaceae	0,33	5,7	4	5	0,04	0,00	4,34	0,00	0,05	0,19
589	14	3	Banjaranyar	<200	Mahoni afrika	Khaya anthotheca (Welw.) C.DC.	Meliaceae	0,49	19,7	10	14	0,04	0,03	154,12	0,15	1,81	6,65
590	14	3	Banjaranyar	<200	Sengon	Paraserianthes falcataria (L.) I.C	Leguminosae / Fabaceae	0,33	6,4	9	12	0,04	0,00	11,72	0,01	0,14	0,51
591	14	3	Banjaranyar	<200	Sirsak	Annona muricata L.	Annonaceae	0,36	3,2	0,5	2	0,04	0,00	0,69	0,00	0,01	0,03
592	14	3	Banjaranyar	<200	Sengon	Paraserianthes falcataria (L.) I.C	Leguminosae / Fabaceae	0,33	14,0	10	12	0,04	0,02	49,71	0,05	0,58	2,14
593	14	3	Banjaranyar	<200	Sampang	Melicope denhamii (Seem.) T.G.I	Rutaceae	0,366	14,0	9	12	0,04	0,02	54,65	0,05	0,64	2,36
594	14	3	Banjaranyar	<200	Kueni	Mangifera odorata Griff.	Anacardiaceae	0,47	3,8	1,5	4	0,04	0,00	2,32	0,00	0,03	0,10
595	14	3	Banjaranyar	<200	Manggis	Mangostana garcinia Gaertn.	Clusiaceae	0,81	4,5	1	4	0,04	0,00	5,08	0,01	0,06	0,22
596	14	3	Banjaranyar	<200	Jambu bol	Syzygium malaccense (L.) Merr. a	Myrtaceae	0,56	5,4	2	5	0,04	0,00	6,34	0,01	0,07	0,27
597	14	3	Banjaranyar	<200	Jengkol	Archidendron pauciflorum (Bentl	Leguminosae / Fabaceae	0,4	11,8	10	13	0,04	0,01	46,44	0,05	0,55	2,00
598	14	3	Banjaranyar	<200	Mahoni	Swietenia macrophylla King	Meliaceae	0,61	13,0	6	10	0,04	0,01	64,88	0,06	0,76	2,80
599	14	3	Banjaranyar	<200	Kupa	Syzygium polycephalum (Miq.) M	Myrtaceae	0,614	2,5	2	2,5	0,04	0,00	0,92	0,00	0,01	0,04
600	14	3	Banjaranyar	<200	Langsat	Lansium domesticum	Meliaceae	0,7	5,4	4	5,5	0,04	0,00	8,48	0,01	0,10	0,37
601	14	3	Banjaranyar	<200	Mahoni	Swietenia macrophylla King	Meliaceae	0,61	11,8	6	8	0,04	0,01	43,82	0,04	0,51	1,89
602	14	3	Banjaranyar	<200	Kupa	Syzygium polycephalum (Miq.) M	Myrtaceae	0,614	5,4	3	4	0,04	0,00	5,62	0,01	0,07	0,24
603	14	3	Banjaranyar	<200	Langsat	Lansium domesticum	Meliaceae	0,7	2,9	3	4	0,04	0,00	1,98	0,00	0,02	0,09
604	14	3	Banjaranyar	<200	Mahoni	Swietenia macrophylla King	Meliaceae	0,61	11,1	4	6	0,04	0,01	30,41	0,03	0,36	1,31
605	14	3	Banjaranyar	<200	Mahoni	Swietenia macrophylla King	Meliaceae	0,61	4,1	3	3,5	0,04	0,00	3,02	0,00	0,04	0,13
606	14	3	Banjaranyar	<200	Mahoni	Swietenia macrophylla King	Meliaceae	0,61	6,4	4	5	0,04	0,00	9,23	0,01	0,11	0,40
607	14	3	Banjaranyar	<200	Mahoni	Swietenia macrophylla King	Meliaceae	0,61	6,7	4	6	0,04	0,00	11,93	0,01	0,14	0,51
608	14	3	Banjaranyar	<200	Mahoni	Swietenia macrophylla King	Meliaceae	0,61	6,4	4	6	0,04	0,00	10,91	0,01	0,13	0,47
609	14	3	Banjaranyar	<200	Mahoni	Swietenia macrophylla King	Meliaceae	0,61	16,2	6	9	0,04	0,02	87,87	0,09	1,03	3,79
610	14	3	Banjaranyar	<200	Mahoni	Swietenia macrophylla King	Meliaceae	0,61	6,0	4	7	0,04	0,00	11,44	0,01	0,13	0,49
611	14	3	Banjaranyar	<200	Tisuk	Hibiscus macrophyllus Roxb. ex I	Malvaceae	0,4	21,0	12	15	0,04	0,03	152,87	0,15	1,80	6,59
612	14	3	Banjaranyar	<200	Durian	Durio zibethinus L.	Malvaceae	0,57	19,4	2	12	0,04	0,03	149,20	0,15	1,75	6,43
613	14	3	Banjaranyar	<200	Mahoni	Swietenia macrophylla King	Meliaceae	0,61	26,7	8	12	0,04	0,06	285,29	0,29	3,35	12,30
614	14	3	Banjaranyar	<200	Petai	Parkia speciosa Hassk.	Leguminosae / Fabaceae	0,45	7,0	7	9	0,04	0,00	14,25	0,01	0,17	0,61

615	14	3	Banjaranyar	<200	Jengkol	Archidendron pauciflorum (Bentl	Leguminosae / Fabaceae	0,4	6,4	4	6	0,04	0,00	7,41	0,01	0,09	0,32
616	14	3	Banjaranyar	<200	Mahoni	Swietenia macrophylla King	Meliaceae	0,61	11,1	5	9	0,04	0,01	44,09	0,04	0,52	1,90
617	14	3	Banjaranyar	<200	Mahoni	Swietenia macrophylla King	Meliaceae	0,61	6,4	4	5,5	0,04	0,00	10,07	0,01	0,12	0,43
618	14	3	Banjaranyar	<200	Mahoni	Swietenia macrophylla King	Meliaceae	0,61	22,3	8	13	0,04	0,04	219,82	0,22	2,58	9,48
619	14	3	Banjaranyar	<200	Mahoni	Swietenia macrophylla King	Meliaceae	0,61	18,5	8	11	0,04	0,03	133,66	0,13	1,57	5,76
620	14	3	Banjaranyar	<200	Sengon	Paraserianthes falcataria (L.) I.C.	Leguminosae / Fabaceae	0,33	15,3	10	13	0,04	0,02	62,73	0,06	0,74	2,71
621	14	3	Banjaranyar	<200	Sengon	Paraserianthes falcataria (L.) I.C.	Leguminosae / Fabaceae	0,33	10,5	6	12	0,04	0,01	29,34	0,03	0,34	1,27
622	14	3	Banjaranyar	<200	Sengon	Paraserianthes falcataria (L.) I.C.	Leguminosae / Fabaceae	0,33	10,8	7	11	0,04	0,01	28,62	0,03	0,34	1,23
623	14	3	Banjaranyar	<200	Sengon	Paraserianthes falcataria (L.) I.C.	Leguminosae / Fabaceae	0,33	14,3	6	10	0,04	0,02	43,83	0,04	0,51	1,89
624	14	3	Banjaranyar	<200	Sengon	Paraserianthes falcataria (L.) I.C.	Leguminosae / Fabaceae	0,33	9,5	6	8	0,04	0,01	17,00	0,02	0,20	0,73
625	14	3	Banjaranyar	<200	Mahoni	Swietenia macrophylla King	Meliaceae	0,61	8,0	4	6	0,04	0,00	16,42	0,02	0,19	0,71
626	14	3	Banjaranyar	<200	Mahoni	Swietenia macrophylla King	Meliaceae	0,61	3,8	3	4	0,04	0,00	2,95	0,00	0,03	0,13
627	14	3	Banjaranyar	<200	Mahoni	Swietenia macrophylla King	Meliaceae	0,61	21,6	7	10	0,04	0,04	163,92	0,16	1,93	7,07
628	14	3	Banjaranyar	<200	Mahoni	Swietenia macrophylla King	Meliaceae	0,61	3,2	2	2,5	0,04	0,00	1,37	0,00	0,02	0,06
629	14	3	Banjaranyar	<200	Mahoni	Swietenia macrophylla King	Meliaceae	0,61	5,7	4	5,5	0,04	0,00	8,30	0,01	0,10	0,36
630	14	3	Banjaranyar	<200	Mahoni	Swietenia macrophylla King	Meliaceae	0,61	4,5	3	4,5	0,04	0,00	4,36	0,00	0,05	0,19
631	14	3	Banjaranyar	<200	Mahoni	Swietenia macrophylla King	Meliaceae	0,61	3,8	2,5	3,5	0,04	0,00	2,61	0,00	0,03	0,11
632	14	3	Banjaranyar	<200	Kelapa	Cocos nucifera L.	Arecaceae	0,5	24,8	6	9	0,04	0,05	73,80	0,07	0,87	3,18
633	14	3	Banjaranyar	<200	Petai	Parkia speciosa Hassk.	Leguminosae / Fabaceae	0,45	2,5	2	2,5	0,04	0,00	0,69	0,00	0,01	0,03
634	14	3	Banjaranyar	<200	Sengon	Paraserianthes falcataria (L.) I.C.	Leguminosae / Fabaceae	0,33	3,8	4	5	0,04	0,00	2,06	0,00	0,02	0,09
635	14	3	Banjaranyar	<200	Sampang	Melicope denhamii (Seem.) T.G.I	Rutaceae	0,366	18,1	6	10	0,04	0,03	74,31	0,07	0,87	3,20
636	14	3	Banjaranyar	<200	Mindi	Melia azedarach L.	Meliaceae	0,53	7,0	6	6,5	0,04	0,00	12,29	0,01	0,14	0,53
637	14	3	Banjaranyar	<200	Langsat	Lansium domesticum	Meliaceae	0,7	2,5	3	3,5	0,04	0,00	1,41	0,00	0,02	0,06
638	14	3	Banjaranyar	<200	Sengon	Paraserianthes falcataria (L.) I.C.	Leguminosae / Fabaceae	0,33	3,5	4	5	0,04	0,00	1,76	0,00	0,02	0,08
639	14	3	Banjaranyar	<200	Mahoni	Swietenia macrophylla King	Meliaceae	0,61	19,1	6	11	0,04	0,03	142,22	0,14	1,67	6,13
640	14	3	Banjaranyar	<200	Mahoni afrika	Khaya anthotheca (Welw.) C.DC.	Meliaceae	0,49	19,1	14	17	0,04	0,03	173,38	0,17	2,04	7,48
641	14	3	Banjaranyar	<200	Mahoni	Swietenia macrophylla King	Meliaceae	0,61	20,0	7	13	0,04	0,03	181,24	0,18	2,13	7,82
642	14	3	Banjaranyar	<200	Kakao	Theobroma cacao L.	Malvaceae	0,36	10,8	2,5	6	0,04	0,01	13,48	0,01	0,16	0,58
643	14	3	Banjaranyar	<200	Kakao	Theobroma cacao L.	Malvaceae	0,36	10,2	1,8	3	0,04	0,01	11,96	0,01	0,14	0,52
644	14	3	Banjaranyar	<200	Karet	Hevea brasiliensis (Willd. ex A.Ju	Euphorbiaceae	0,53	12,7	4	10	0,04	0,01	54,52	0,05	0,64	2,35
645	14	3	Banjaranyar	<200	Mahoni	Swietenia macrophylla King	Meliaceae	0,61	12,7	5	9	0,04	0,01	56,30	0,06	0,66	2,43
646	14	3	Banjaranyar	<200	Mahoni	Swietenia macrophylla King	Meliaceae	0,61	19,4	6	13	0,04	0,03	170,84	0,17	2,01	7,37
647	14	3	Banjaranyar	<200	Sengon	Paraserianthes falcataria (L.) I.C.	Leguminosae / Fabaceae	0,33	15,9	11	14	0,04	0,02	72,35	0,07	0,85	3,12
648	14	3	Banjaranyar	<200	Karet	Hevea brasiliensis (Willd. ex A.Ju	Euphorbiaceae	0,53	7,6	4	6	0,04	0,00	13,39	0,01	0,16	0,58
649	14	3	Banjaranyar	<200	Mahoni	Swietenia macrophylla King	Meliaceae	0,61	4,1	4	5	0,04	0,00	4,19	0,00	0,05	0,18
650	14	3	Banjaranyar	<200	Sengon	Paraserianthes falcataria (L.) I.C.	Leguminosae / Fabaceae	0,33	14,0	12	15	0,04	0,02	60,98	0,06	0,72	2,63
651	14	3	Banjaranyar	<200	Sengon	Paraserianthes falcataria (L.) I.C.	Leguminosae / Fabaceae	0,33	12,7	12	15	0,04	0,01	51,21	0,05	0,60	2,21
652	14	3	Banjaranyar	<200	Sengon	Paraserianthes falcataria (L.) I.C.	Leguminosae / Fabaceae	0,33	10,8	7	9	0,04	0,01	23,81	0,02	0,28	1,03
653	14	3	Banjaranyar	<200	Sirsak	Annona muricata L.	Annonaceae	0,36	4,5	2	3	0,04	0,00	1,86	0,00	0,02	0,08
654	14	3	Banjaranyar	<200	Tisuk	Hibiscus macrophyllus Roxb. ex I	Malvaceae	0,4	19,7	8	12	0,04	0,03	111,12	0,11	1,31	4,79
655	14	3	Banjaranyar	<200	Sengon	Paraserianthes falcataria (L.) I.C.	Leguminosae / Fabaceae	0,33	4,8	7	8	0,04	0,00	4,77	0,00	0,06	0,21
656	14	3	Banjaranyar	<200	Sengon	Paraserianthes falcataria (L.) I.C.	Leguminosae / Fabaceae	0,33	9,9	7	10	0,04	0,01	22,14	0,02	0,26	0,95
657	15	2	Banjaranyar	<200	Afrika	Maesopsis eminii Engl.	Rhamnaceae	0,43	10,2	5	7	0,04	0,01	21,57	0,02	0,25	0,93
658	15	2	Banjaranyar	<200	Sengon	Paraserianthes falcataria (L.) I.C.	Leguminosae / Fabaceae	0,33	6,7	7	8	0,04	0,00	8,84	0,01	0,10	0,38
659	15	2	Banjaranyar	<200	Sengon	Paraserianthes falcataria (L.) I.C.	Leguminosae / Fabaceae	0,33	6,4	8	10	0,04	0,00	9,92	0,01	0,12	0,43
660	15	2	Banjaranyar	<200	Kelapa	Cocos nucifera L.	Arecaceae	0,5	22,6	9	12	0,04	0,04	96,90	0,10	1,14	4,18
661	15	2	Banjaranyar	<200	Afrika	Maesopsis eminii Engl.	Rhamnaceae	0,43	13,0	8	9	0,04	0,01	42,76	0,04	0,50	1,84
662	15	2	Banjaranyar	<200	Afrika	Maesopsis eminii Engl.	Rhamnaceae	0,43	10,5	8	10	0,04	0,01	31,64	0,03	0,37	1,36
663	15	2	Banjaranyar	<200	Sengon	Paraserianthes falcataria (L.) I.C.	Leguminosae / Fabaceae	0,33	8,0	10	12	0,04	0,00	17,65	0,02	0,21	0,76
664	15	2	Banjaranyar	<200	Afrika	Maesopsis eminii Engl.	Rhamnaceae	0,43	10,5	8	10	0,04	0,01	31,64	0,03	0,37	1,36
665	15	2	Banjaranyar	<200	Afrika	Maesopsis eminii Engl.	Rhamnaceae	0,43	10,8	8	10	0,04	0,01	33,42	0,03	0,39	1,44
666	15	2	Banjaranyar	<200	Kelapa	Cocos nucifera L.	Arecaceae	0,5	18,1	10	13	0,04	0,03	104,60	0,10	1,23	4,51
667	15	2	Banjaranyar	<200	Afrika	Maesopsis eminii Engl.	Rhamnaceae	0,43	12,4	9	12	0,04	0,01	50,79	0,05	0,60	2,19
668	15	2	Banjaranyar	<200	Afrika	Maesopsis eminii Engl.	Rhamnaceae	0,43	10,8	9	12	0,04	0,01	39,50	0,04	0,46	1,70
669	15	2	Banjaranyar	<200	Sengon	Paraserianthes falcataria (L.) I.C.	Leguminosae / Fabaceae	0,33	9,5	9	10	0,04	0,01	20,85	0,02	0,25	0,90
670	15	2	Banjaranyar	<200	kakao	Theobroma cacao L.	Malvaceae	0,36	11,8	2	5	0,04	0,01	15,94	0,02	0,19	0,69
671	15	2	Banjaranyar	<200	jengkol	Archidendron pauciflorum (Bentl	Leguminosae / Fabaceae	0,4	11,5	8	11	0,04	0,01	37,90	0,04	0,45	1,63
672	15	2	Banjaranyar	<200	Afrika	Maesopsis eminii Engl.	Rhamnaceae	0,43	14,0	8	12	0,04	0,02	63,34	0,06	0,74	2,73
673	15	2	Banjaranyar	<200	mahoni	Swietenia macrophylla King	Meliaceae	0,61	12,4	8	11	0,04	0,01	64,60	0,06	0,76	2,79
674	15	2	Banjaranyar	<200	Afrika	Maesopsis eminii Engl.	Rhamnaceae	0,43	3,5	3	4	0,04	0,00	1,83	0,00	0,02	0,08
675	15	2	Banjaranyar	<200	mahoni	Swietenia macrophylla King	Meliaceae	0,61	13,0	6	12	0,04	0,01	76,67	0,08	0,90	3,31
676	15	2	Banjaranyar	<200	Afrika	Maesopsis eminii Engl.	Rhamnaceae	0,43	4,5	4	5	0,04	0,00	3,49	0,00	0,04	0,15
677	15	2	Banjaranyar	<200	Afrika	Maesopsis eminii Engl.	Rhamnaceae	0,43	8,9	8	11	0,04	0,01	25,56	0,03	0,30	1,10
678	15	2	Banjaranyar	<200	Afrika	Maesopsis eminii Engl.	Rhamnaceae	0,43	9,5	8	11	0,04	0,01	29,00	0,03	0,34	1,25
679	15	2	Banjaranyar	<200	Sengon	Paraserianthes falcataria (L.) I.C.	Leguminosae / Fabaceae	0,33	4,5	5	6	0,04	0,00	3,23	0,00	0,04	0,14
680	15	2	Banjaranyar	<200	cengkeh	Syzygium aromaticum (L.) Merr. &	Myrtaceae	0,79	3,2	2	3	0,04	0,00	2,06	0,00	0,02	0,09
681	15	2	Banjaranyar	<200	Sengon	Paraserianthes falcataria (L.) I.C.	Leguminosae / Fabaceae	0,33	13,0	15	18	0,04	0,01	63,32	0,06	0,74	2,73
682	15	2	Banjaranyar	<200	Afrika	Maesopsis eminii Engl.	Rhamnaceae	0,43	18,5	8	12	0,04	0,03	105,08	0,11	1,23	4,53
683	15	2	Banjaranyar	<200	Kelapa	Cocos nucifera L.	Arecaceae	0,5	23,2	14	17	0,04	0,04	135,40	0,14	1,59	5,84
684	15	2	Banjaranyar	<200	Sengon	Paraserianthes falcataria (L.) I.C.	Leguminosae / Fabaceae	0,33	12,7	12	15	0,04	0,01	51,21	0,05	0,60	2,21
685	15	2	Banjaranyar	<200	Afrika	Maesopsis eminii Engl.	Rhamnaceae	0,43	4,5	5	6	0,04	0,00	4,12	0,00	0,05	0,18
686	15	2	Banjaranyar	<200	Afrika	Maesopsis eminii Engl.	Rhamnaceae	0,43	8,6	7	10	0,04	0,01	21,91	0,02	0,26	0,94
687	15	2	Banjaranyar	<200	Sengon	Paraserianthes falcataria (L.) I.C.	Leguminosae / Fabaceae	0,33	5,7	6	8	0,04	0,00	6,67	0,01	0,08	0,29
688	15	2	Banjaranyar	<200	Kelapa	Cocos nucifera L.	Arecaceae	0,5	24,2	11	14	0,04	0,05	112,30	0,11	1,32	4,84
689	15	2	Banjaranyar	<200	mahoni	Swietenia macrophylla King	Meliaceae	0,61	10,8	6	11	0,04	0,01	50,24	0,05	0,59	2,17
690	15	2	Banjaranyar	<200	Sengon	Paraserianthes falcataria (L.) I.C.	Leguminosae / Fabaceae	0,33	3,2	6	7	0,04	0,00	2,01	0,00	0,02	0,09
691	15	2	Banjaranyar	<200	Sengon	Paraserianthes falcataria (L.) I.C.	Leguminosae / Fabaceae	0,33	11,1	13	15	0,04	0,01	40,10	0,04	0,47	1,73
692	15	2	Banjaranyar	<200	Sengon	Paraserianthes falcataria (L.) I.C.	Leguminosae / Fabaceae	0,33	6,7	10	11	0,04	0,00	11,84	0,01	0,14	0,51
693	15	2	Banjaranyar	<200	Sengon	Paraserianthes falcataria (L.) I.C.	Leguminosae / Fabaceae	0,33	6,0	9	10	0,04	0,00	9,03	0,01	0,11	0,39
694	15	2	Banjaranyar	<200	Sengon	Paraserianthes falcataria (L.) I.C.	Leguminosae / Fabaceae	0,33	11,1	13	14,5	0,04	0,01	38,87	0,04	0,46	1,68
695	15	2	Banjaranyar	<200	Nangka	Artocarpus heterophyllus Lam.	Moraceae	0,61	6,4	3	5	0,04	0,00	9,23	0,01	0,11	0,40
696	15	2	Banjaranyar	<200	kakao	Theobroma cacao L.	Malvaceae	0,36	5,4	1,8	3	0,04	0,00	3,42	0,00	0,04	0,15
697	15	2	Banjaranyar	<200	Sengon	Paraserianthes falcataria (L.) I.C.	Leguminosae / Fabaceae	0,33	3,2	3	4	0,04	0,00	1,20	0,00	0,01	0,05
698	15	2	Banjaranyar	<200	cengkeh	Syzygium aromaticum (L.) Merr. &	Myrtaceae	0,79	14,0	4	9	0,04	0,02	84,96	0,08	1,00	3,66
699	15	2	Banjaranyar	<200	Afrika	Maesopsis eminii Engl.	Rhamnaceae	0,43	15,0	10	15	0,04	0,02	87,69	0,09	1,03	3,78
700	15	2	Banjaranyar	<200	Langsat	Lansium domesticum	Meliaceae	0,7	7,3	4	7	0,04	0,00	18,41	0,02	0,22	0,79
701	15	2	Banjaranyar	<200	Afrika	Maesopsis eminii Engl.	Rhamnaceae	0,43	5,7	5	7	0,04	0,00	7,52	0,01	0,09	0,32
702	15	2	Banjaranyar	<200	Kelapa	Cocos nucifera L.	Arecaceae	0,5	22,3	12	15	0,04	0,04	120,00	0,12	1,41	5,17
703	15	2	Banjaranyar	<200	Sengon	Paraserianthes falcataria (L.) I.C.	Leguminosae / Fabaceae	0,33	11,1	12	15	0,04	0,01	40,10	0,04	0,47	1,73
704	15	2	Banjaranyar	<200	kakao	Theobroma cacao L.	Malvaceae	0,36	5,4	2	2,5	0,04	0,00	3,42	0,00	0,04	0,15
705	15	2	Banjaranyar	<200	Afrika	Maesopsis eminii Engl.	Rhamnaceae	0,43	8,9	7	9	0,04	0,01	21,26	0,02	0,25	0,92
706	15	2	Banjaranyar	<200	Afrika	Maesopsis eminii Engl.	Rhamnaceae	0,43	9,5	7	10	0,04	0,01	26,57	0,03	0,31	1,15
707	15	2	Banjaranyar	<200	cengkeh	Syzygium aromaticum (L.) Merr. &	Myrtaceae	0,79	4,5	3	5	0,04	0,00	6,09	0,01	0,07	0,26
708	15	2	Banjaranyar	<200	kakao	Theobroma cacao L.	Malvaceae	0,36	3,5	1,8	2	0,04	0,00	1,44	0,00	0,02	0,06
709	15	2	Banjaranyar	<200	Sengon	Paraserianthes falcataria (L.) I.C.	Leguminosae / Fabaceae	0,33	8,3	9	10	0,04	0,01	16,04	0,02	0,19	0,69
710	15	2	Banjaranyar	<200	Afrika	Maesopsis eminii Engl.	Rhamnaceae	0,43	12,7	6	9	0,04	0,01	40,87	0,04	0,48	1,76
711	15	2	Banjaranyar	<200	Sengon	Paraserianthes falcataria (L.) I.C.	Leguminosae / Fabaceae	0,33	11,1	12	14	0,04	0,01	37,64	0,04	0,44	1,62
712	15	2	Banjaranyar	<200	Kelapa	Cocos nucifera L.	Arecaceae	0,5	27,4	13	16	0,04	0,06	127,70	0,13	1,50	5,51
713	15	2	Banjaranyar	<200	jengkol	Archidendron pauciflorum (Bentl	Leguminosae / Fabaceae	0,4	7,3	4	8	0,04	0,00	12,46	0,01	0,15	0,54
714	15	2	Banjaranyar	<200	jengkol	Archidendron pauciflorum (Bentl	Leguminosae / Fabaceae	0,4	5,1	3	5	0,04	0,00	4,17	0,00	0,05	0,18
715	15	2	Banjaranyar	<200	Kelapa	Cocos nucifera L.	Arecaceae	0,5	24,8	16	19	0,04	0,05	150,80	0,15	1,77	6,50
716	15	2	Banjaranyar	<200	mahoni	Swietenia macrophylla King	Meliaceae	0,61	9,5	6	12	0,04	0,01	43,26	0,04	0,51	1,87
717	15	2	Banjaranyar	<200	Sengon	Paraserianthes falcataria (L.) I.C.	Leguminosae / Fabaceae	0,33	8,3	12	14	0,04	0,01	21,84	0,02	0,26	0,94
718	15	2	Banjaranyar	<200	kakao	Theobroma cacao L.	Malvaceae	0,36	4,5	2	2,5	0,04	0,00	2,33	0,00	0,03	0,10
719	15	2	Banjaranyar	<200	cengkeh	Syzygium aromaticum (L.) Merr. &	Myrtaceae	0,79	13,7	5	11	0,04	0,01	97,90	0,10	1,15	4,22
720	15	2	Banjaranyar	<200	Afrika	Maesopsis eminii Engl.	Rhamnaceae	0,43	9,5	6	9	0,04	0,01	24,13	0,02	0,28	1,04
721	15	2	Banjaranyar	<200	Sengon	Paraserianthes falcataria (L.) I.C.	Leguminosae / Fabaceae	0,33	6,4	8	9	0,04	0,00	9,01	0,01	0,11	0,39
722	15	2	Banjaranyar	<200	kakao	Theobroma cacao L.	Malvaceae	0,36	3,5	2	2,5	0,04	0,00	1,44	0,00	0,02	0,06
723	15	2	Banjaranyar	<200	cengkeh	Syzygium aromaticum (L.) Merr. &	Myrtaceae	0,79	13,0	7	11	0,04	0,01	89,72	0,09	1,05	3,87
724	15	2	Banjaranyar	<200	Afrika	Maesopsis eminii Engl.	Rhamnaceae	0,43	5,7	5	7	0,04	0,00	7,52	0,01	0,09	0,32
725	15	2	Banjaranyar	<200	Sengon	Paraserianthes falcataria (L.) I.C.	Leguminosae / Fabaceae	0,33	3,8	4	5	0,04	0,00	2,06	0,00	0,02	0,09
726	16	1	Banjaranyar	<200	Mahoni	Swietenia macrophylla King	Meliaceae	0,61	12,1	6	8	0,04	0,01	46,01	0,05	0,54	1,98
727	16	1	Banjaranyar	<200	Kakao	Theobroma cacao L.	Malvaceae	0,36	6,4	1,8	3	0,04	0,00	4,71	0,00	0,06	0,20
728	16	1	Banjaranyar	<200	Kelapa	Cocos nucifera L.	Arecaceae	0,5	24,8	5	8	0,04	0,05	66,10	0,07	0,78	2,85
729	16	1	Banjaranyar	<200	Mahoni	Swietenia macrophylla King	Meliaceae	0,61	12,1	4	7	0,04	0,01	40,72	0,04	0,48	1,76
730	16	1	Banjaranyar	<200	Cengkeh	Syzygium aromaticum (L.) Merr. &	Myrtaceae	0,79	6,4	2	4	0,04	0,00	9,53	0,01	0,11	0,41
731	16	1	Banjaranyar	<200	Mahoni	Swietenia macrophylla King	Meliaceae	0,61	17,5	4	8	0,04	0,02	90,58	0,09	1,06	3,91
732	16	1	Banjaranyar	<200	Kelapa	Cocos nucifera L.	Arecaceae	0,5	19,1	2	4	0,04	0,03	35,30	0,04	0,41	1,52
733	16	1	Banjaranyar	<200	Kakao	Theobroma cacao L.	Malvaceae	0,36	6,4	2	3	0,04	0,00	4,71	0,00	0,06	0,20
734	16	1	Banjaranyar	<200	Jengkol	Archidendron pauciflorum (Bentl	Leguminosae / Fabaceae	0,4	5,7	2	4	0,04	0,00	4,21	0,00	0,05	0,18
735	16	1	Banjaranyar	<200	Cengkeh	Syzygium aromaticum (L.) Merr. &	Myrtaceae	0,79	10,8	2	6	0,04	0,01	36,54	0,04	0,43	1,58
736	16	1	Banjaranyar	<200	Kelapa	Cocos nucifera L.	Arecaceae	0,5	23,5	7	10	0,04	0,04	81,50	0,08	0,96	3,51
737	16	1	Banjaranyar	<200	Sengon	Paraserianthes falcataria (L.) I.C.	Leguminosae / Fabaceae	0,33	19,1	10	13	0,04	0,03	94,41	0,09	1,11	4,07
738	16	1	Banjaranyar	<200	Sengon	Paraserianthes falcataria (L.) I.C.	Leguminosae / Fabaceae	0,33	8,9	10	12	0,04	0,01	21,72	0,02	0,26	0,94

739	16	1	Banjaranyar	<200	Manggis	Mangostana garcinia Gaertn.	Clusiaceae	0,81	5,1	1	3	0,04	0,00	4,98	0,00	0,06	0,21
740	16	1	Banjaranyar	<200	Sengon	Paraserianthes falcataria (L.) I.C.	Leguminosae / Fabaceae	0,33	5,7	4	5	0,04	0,00	4,34	0,00	0,05	0,19
741	16	1	Banjaranyar	<200	Mahoni	Swietenia macrophylla King	Meliaceae	0,61	21,0	9	13	0,04	0,03	197,36	0,20	2,32	8,51
742	16	1	Banjaranyar	<200	Kakao	Theobroma cacao L.	Malvaceae	0,36	4,5	1	2	0,04	0,00	2,33	0,00	0,03	0,10
743	16	1	Banjaranyar	<200	Sengon	Paraserianthes falcataria (L.) I.C.	Leguminosae / Fabaceae	0,33	3,2	3,5	4,5	0,04	0,00	1,34	0,00	0,02	0,06
744	16	1	Banjaranyar	<200	Manggis	Mangostana garcinia Gaertn.	Clusiaceae	0,81	3,5	0,5	2,5	0,04	0,00	2,12	0,00	0,02	0,09
745	16	1	Banjaranyar	<200	Tisuk	Hibiscus macrophyllus Roxb. ex	Malvaceae	0,4	12,7	7	11	0,04	0,01	45,97	0,05	0,54	1,98
746	16	1	Banjaranyar	<200	Sengon	Paraserianthes falcataria (L.) I.C.	Leguminosae / Fabaceae	0,33	14,0	6	11	0,04	0,02	45,90	0,05	0,54	1,98
747	16	1	Banjaranyar	<200	Sengon	Paraserianthes falcataria (L.) I.C.	Leguminosae / Fabaceae	0,33	4,8	7	8	0,04	0,00	4,77	0,00	0,06	0,21
748	16	1	Banjaranyar	<200	Sengon	Paraserianthes falcataria (L.) I.C.	Leguminosae / Fabaceae	0,33	6,0	7	8	0,04	0,00	7,36	0,01	0,09	0,32
749	16	1	Banjaranyar	<200	Cengkeh	Syzygium aromaticum (L.) Merr. &	Myrtaceae	0,79	16,5	3	7	0,04	0,02	91,66	0,09	1,08	3,95
750	16	1	Banjaranyar	<200	Sengon	Paraserianthes falcataria (L.) I.C.	Leguminosae / Fabaceae	0,33	5,4	5	6,5	0,04	0,00	4,96	0,00	0,06	0,21
751	16	1	Banjaranyar	<200	Sengon	Paraserianthes falcataria (L.) I.C.	Leguminosae / Fabaceae	0,33	6,4	8	10	0,04	0,00	9,92	0,01	0,12	0,43
752	16	1	Banjaranyar	<200	Sengon	Paraserianthes falcataria (L.) I.C.	Leguminosae / Fabaceae	0,33	3,8	3	4	0,04	0,00	1,68	0,00	0,02	0,07
753	16	1	Banjaranyar	<200	Sengon	Paraserianthes falcataria (L.) I.C.	Leguminosae / Fabaceae	0,33	8,0	10	9	0,04	0,00	13,56	0,01	0,16	0,58
754	16	1	Banjaranyar	<200	Kelapa	Cocos nucifera L.	Arecaceae	0,5	26,7	8	13	0,04	0,06	104,60	0,10	1,23	4,51
755	16	1	Banjaranyar	<200	Sengon	Paraserianthes falcataria (L.) I.C.	Leguminosae / Fabaceae	0,33	10,8	9	11	0,04	0,01	28,62	0,03	0,34	1,23
756	16	1	Banjaranyar	<200	Sengon	Paraserianthes falcataria (L.) I.C.	Leguminosae / Fabaceae	0,33	13,7	6	12	0,04	0,01	47,66	0,05	0,56	2,06
757	16	1	Banjaranyar	<200	Sengon	Paraserianthes falcataria (L.) I.C.	Leguminosae / Fabaceae	0,33	5,4	3	7	0,04	0,00	5,31	0,01	0,06	0,23
758	16	1	Banjaranyar	<200	Cengkeh	Syzygium aromaticum (L.) Merr. &	Myrtaceae	0,79	14,3	9	10	0,04	0,02	97,51	0,10	1,15	4,20
759	16	1	Banjaranyar	<200	Kelapa	Cocos nucifera L.	Arecaceae	0,5	27,0	6	12	0,04	0,06	96,90	0,10	1,14	4,18
760	16	1	Banjaranyar	<200	Sengon	Paraserianthes falcataria (L.) I.C.	Leguminosae / Fabaceae	0,33	9,5	6	8	0,04	0,01	17,00	0,02	0,20	0,73
761	16	1	Banjaranyar	<200	Sengon	Paraserianthes falcataria (L.) I.C.	Leguminosae / Fabaceae	0,33	5,1	6	9	0,04	0,00	5,99	0,01	0,07	0,26
762	16	1	Banjaranyar	<200	Kelapa	Cocos nucifera L.	Arecaceae	0,5	27,4	10	13	0,04	0,06	104,60	0,10	1,23	4,51
763	16	1	Banjaranyar	<200	Sengon	Paraserianthes falcataria (L.) I.C.	Leguminosae / Fabaceae	0,33	3,8	1,5	3	0,04	0,00	1,29	0,00	0,02	0,06
764	16	1	Banjaranyar	<200	Kelapa	Cocos nucifera L.	Arecaceae	0,5	23,5	6	9	0,04	0,04	73,80	0,07	0,87	3,18
765	16	1	Banjaranyar	<200	Cengkeh	Syzygium aromaticum (L.) Merr. &	Myrtaceae	0,79	4,8	2,5	4,5	0,04	0,00	6,27	0,01	0,07	0,27
766	16	1	Banjaranyar	<200	Kakao	Theobroma cacao L.	Malvaceae	0,36	8,9	1,5	3	0,04	0,01	9,18	0,01	0,11	0,40
767	16	1	Banjaranyar	<200	Kakao	Theobroma cacao L.	Malvaceae	0,36	9,5	1,5	5,5	0,04	0,01	10,52	0,01	0,12	0,45
768	16	1	Banjaranyar	<200	Kelapa	Cocos nucifera L.	Arecaceae	0,5	24,5	10	13	0,04	0,05	104,60	0,10	1,23	4,51
769	16	1	Banjaranyar	<200	Cengkeh	Syzygium aromaticum (L.) Merr. &	Myrtaceae	0,79	15,3	2	9	0,04	0,02	99,65	0,10	1,17	4,30
770	17	2	Banjaranyar	<200	Sengon	Paraserianthes falcataria (L.) I.C.	Leguminosae / Fabaceae	0,33	8,0	7	8	0,04	0,00	12,17	0,01	0,14	0,52
771	17	2	Banjaranyar	<200	Durian	Durio zibethinus L.	Malvaceae	0,57	13,7	4	9	0,04	0,01	60,41	0,06	0,71	2,60
772	17	2	Banjaranyar	<200	Sengon	Paraserianthes falcataria (L.) I.C.	Leguminosae / Fabaceae	0,33	5,7	7	8	0,04	0,00	6,67	0,01	0,08	0,29
773	17	2	Banjaranyar	<200	Petai	Parkia speciosa Hassk.	Leguminosae / Fabaceae	0,45	3,5	4	5	0,04	0,00	2,34	0,00	0,03	0,10
774	17	2	Banjaranyar	<200	Sengon	Paraserianthes falcataria (L.) I.C.	Leguminosae / Fabaceae	0,33	5,4	4	6	0,04	0,00	4,61	0,00	0,05	0,20
775	17	2	Banjaranyar	<200	Kelapa	Cocos nucifera L.	Arecaceae	0,5	25,5	10	13	0,04	0,05	104,60	0,10	1,23	4,51
776	17	2	Banjaranyar	<200	Sengon	Paraserianthes falcataria (L.) I.C.	Leguminosae / Fabaceae	0,33	8,9	5	7	0,04	0,01	13,26	0,01	0,16	0,57
777	17	2	Banjaranyar	<200	Sp8	Sp8	0	0,5	2,9	2	3	0,04	0,00	1,12	0,00	0,01	0,05
778	17	2	Banjaranyar	<200	Sengon	Paraserianthes falcataria (L.) I.C.	Leguminosae / Fabaceae	0,33	5,1	4	6	0,04	0,00	4,13	0,00	0,05	0,18
779	17	2	Banjaranyar	<200	Cempedak	Artocarpus integer (Thunb.) Merr	Moraceae	0,56	10,5	4	8	0,04	0,01	32,86	0,03	0,39	1,42
780	17	2	Banjaranyar	<200	Mindi	Melia azedarach L.	Meliaceae	0,53	11,5	8	9	0,04	0,01	40,81	0,04	0,48	1,76
781	17	2	Banjaranyar	<200	Manggis	Mangostana garcinia Gaertn.	Clusiaceae	0,81	8,0	1	4	0,04	0,00	14,68	0,01	0,17	0,63
782	17	2	Banjaranyar	<200	Kelapa	Cocos nucifera L.	Arecaceae	0,5	27,0	6	9	0,04	0,06	73,80	0,07	0,87	3,18
783	17	2	Banjaranyar	<200	Sengon	Paraserianthes falcataria (L.) I.C.	Leguminosae / Fabaceae	0,33	8,6	6	7,5	0,04	0,01	13,21	0,01	0,16	0,57
784	17	2	Banjaranyar	<200	Durian	Durio zibethinus L.	Malvaceae	0,57	14,6	3	7	0,04	0,02	54,30	0,05	0,64	2,34
785	17	2	Banjaranyar	<200	Sengon	Paraserianthes falcataria (L.) I.C.	Leguminosae / Fabaceae	0,33	12,1	7	9	0,04	0,01	29,20	0,03	0,34	1,26
786	17	2	Banjaranyar	<200	Durian	Durio zibethinus L.	Malvaceae	0,57	13,7	2	7	0,04	0,01	47,99	0,05	0,56	2,07
787	17	2	Banjaranyar	<200	Kelapa	Cocos nucifera L.	Arecaceae	0,5	24,5	6	9	0,04	0,05	73,80	0,07	0,87	3,18
788	17	2	Banjaranyar	<200	Petai	Parkia speciosa Hassk.	Leguminosae / Fabaceae	0,45	7,3	5	6	0,04	0,00	10,66	0,01	0,13	0,46
789	17	2	Banjaranyar	<200	Sengon	Paraserianthes falcataria (L.) I.C.	Leguminosae / Fabaceae	0,33	7,6	7	8	0,04	0,00	11,29	0,01	0,13	0,49
790	17	2	Banjaranyar	<200	Langsat	Lansium domesticum	Meliaceae	0,7	13,0	4	8	0,04	0,01	59,99	0,06	0,70	2,59
791	17	2	Banjaranyar	<200	Sengon	Paraserianthes falcataria (L.) I.C.	Leguminosae / Fabaceae	0,33	4,5	2,5	5	0,04	0,00	2,74	0,00	0,03	0,12
792	17	2	Banjaranyar	<200	Durian	Durio zibethinus L.	Malvaceae	0,57	6,4	3	5	0,04	0,00	8,67	0,01	0,10	0,37
793	17	2	Banjaranyar	<200	Manggis	Mangostana garcinia Gaertn.	Clusiaceae	0,81	3,2	0,5	2	0,04	0,00	1,45	0,00	0,02	0,06
794	17	2	Banjaranyar	<200	Kelapa	Cocos nucifera L.	Arecaceae	0,5	25,5	12	15	0,04	0,05	120,00	0,12	1,41	5,17
795	17	2	Banjaranyar	<200	Durian	Durio zibethinus L.	Malvaceae	0,57	16,5	7	10	0,04	0,02	94,24	0,09	1,11	4,06
796	17	2	Banjaranyar	<200	Durian	Durio zibethinus L.	Malvaceae	0,57	14,0	3	11	0,04	0,02	75,72	0,08	0,89	3,27
797	17	2	Banjaranyar	<200	Sengon	Paraserianthes falcataria (L.) I.C.	Leguminosae / Fabaceae	0,33	10,5	7	11	0,04	0,01	27,10	0,03	0,32	1,17
798	17	2	Banjaranyar	<200	Tisuk	Hibiscus macrophyllus Roxb. ex	Malvaceae	0,4	4,5	3	4	0,04	0,00	2,66	0,00	0,03	0,11
799	17	2	Banjaranyar	<200	Sengon	Paraserianthes falcataria (L.) I.C.	Leguminosae / Fabaceae	0,33	10,8	6	10	0,04	0,01	26,23	0,03	0,31	1,13
800	17	2	Banjaranyar	<200	Pulai	Alstonia scholaris (L.) R. Br.	Apocynaceae	0,3	13,4	3	7	0,04	0,01	25,53	0,03	0,30	1,10
801	17	2	Banjaranyar	<200	Pulai	Alstonia scholaris (L.) R. Br.	Apocynaceae	0,3	11,1	3	7	0,04	0,01	18,28	0,02	0,21	0,79
802	17	2	Banjaranyar	<200	Manggis	Mangostana garcinia Gaertn.	Clusiaceae	0,81	4,1	0,5	2,5	0,04	0,00	2,88	0,00	0,03	0,12
803	17	2	Banjaranyar	<200	Kelapa	Cocos nucifera L.	Arecaceae	0,5	24,5	14	17	0,04	0,05	135,40	0,14	1,59	5,84
804	17	2	Banjaranyar	<200	Sengon	Paraserianthes falcataria (L.) I.C.	Leguminosae / Fabaceae	0,33	5,4	6	9	0,04	0,00	6,69	0,01	0,08	0,29
805	18	2	Banjaranyar	<200	Cengkeh	Syzygium aromaticum (L.) Merr. &	Myrtaceae	0,79	16,9	3	9	0,04	0,02	119,48	0,12	1,40	5,15
806	18	2	Banjaranyar	<200	Mahoni	Swietenia macrophylla King	Meliaceae	0,61	22,9	5	14	0,04	0,04	247,72	0,25	2,91	10,68
807	18	2	Banjaranyar	<200	Sengon	Paraserianthes falcataria (L.) I.C.	Leguminosae / Fabaceae	0,33	3,5	4	6	0,04	0,00	2,08	0,00	0,02	0,09
808	18	2	Banjaranyar	<200	Tisuk	Hibiscus macrophyllus Roxb. ex	Malvaceae	0,4	16,5	12	16	0,04	0,02	104,78	0,10	1,23	4,52
809	18	2	Banjaranyar	<200	Tisuk	Hibiscus macrophyllus Roxb. ex	Malvaceae	0,4	25,5	12	16	0,04	0,05	230,70	0,23	2,71	9,95
810	18	2	Banjaranyar	<200	Akasia mangium	Acacia mangium Willd.	Leguminosae / Fabaceae	0,5	9,5	9	14	0,04	0,01	41,53	0,04	0,49	1,79
811	18	2	Banjaranyar	<200	Tisuk	Hibiscus macrophyllus Roxb. ex	Malvaceae	0,4	17,5	13	16	0,04	0,02	116,12	0,12	1,36	5,01
812	18	2	Banjaranyar	<200	Tisuk	Hibiscus macrophyllus Roxb. ex	Malvaceae	0,4	9,5	9	12	0,04	0,01	29,39	0,03	0,35	1,27
813	18	2	Banjaranyar	<200	Akasia mangium	Acacia mangium Willd.	Leguminosae / Fabaceae	0,5	23,9	12	15	0,04	0,04	237,02	0,24	2,79	10,22
814	18	2	Banjaranyar	<200	Akasia mangium	Acacia mangium Willd.	Leguminosae / Fabaceae	0,5	21,3	12	15	0,04	0,04	192,77	0,19	2,27	8,31
815	18	2	Banjaranyar	<200	Mahoni	Swietenia macrophylla King	Meliaceae	0,61	21,6	7	12	0,04	0,04	193,72	0,19	2,28	8,35
816	18	2	Banjaranyar	<200	Mahoni	Swietenia macrophylla King	Meliaceae	0,61	11,1	4	9	0,04	0,01	44,09	0,04	0,52	1,90
817	18	2	Banjaranyar	<200	Langsat	Lansium domesticum	Meliaceae	0,7	22,3	4	9	0,04	0,04	178,05	0,18	2,09	7,68
818	18	2	Banjaranyar	<200	Akasia mangium	Acacia mangium Willd.	Leguminosae / Fabaceae	0,5	20,0	11	15	0,04	0,03	172,21	0,17	2,02	7,43
819	18	2	Banjaranyar	<200	Kelapa	Cocos nucifera L.	Arecaceae	0,5	30,5	12	15	0,04	0,07	120,00	0,12	1,41	5,17
820	18	2	Banjaranyar	<200	Sengon	Paraserianthes falcataria (L.) I.C.	Leguminosae / Fabaceae	0,33	4,5	5	6	0,04	0,00	3,23	0,00	0,04	0,14
821	18	2	Banjaranyar	<200	Sengon	Paraserianthes falcataria (L.) I.C.	Leguminosae / Fabaceae	0,33	5,4	5	7	0,04	0,00	5,31	0,01	0,06	0,23
822	18	2	Banjaranyar	<200	Tisuk	Hibiscus macrophyllus Roxb. ex	Malvaceae	0,4	22,3	11	15	0,04	0,04	170,27	0,17	2,00	7,34
823	18	2	Banjaranyar	<200	Tisuk	Hibiscus macrophyllus Roxb. ex	Malvaceae	0,4	17,5	4	8	0,04	0,02	61,54	0,06	0,72	2,65
824	18	2	Banjaranyar	<200	Limus	Mangifera foetida Lour.	Anacardiaceae	0,73	5,1	3	4	0,04	0,00	5,89	0,01	0,07	0,25
825	18	2	Banjaranyar	<200	Sengon	Paraserianthes falcataria (L.) I.C.	Leguminosae / Fabaceae	0,33	19,7	9	15	0,04	0,03	114,30	0,11	1,34	4,93
826	18	2	Banjaranyar	<200	Akasia mangium	Acacia mangium Willd.	Leguminosae / Fabaceae	0,5	22,3	5	15	0,04	0,04	208,88	0,21	2,45	9,01
827	18	2	Banjaranyar	<200	Akasia mangium	Acacia mangium Willd.	Leguminosae / Fabaceae	0,5	19,1	5	14	0,04	0,03	147,84	0,15	1,74	6,38
828	18	2	Banjaranyar	<200	Akasia mangium	Acacia mangium Willd.	Leguminosae / Fabaceae	0,5	7,0	5	15	0,04	0,00	25,06	0,03	0,29	1,08
829	18	2	Banjaranyar	<200	Kelapa	Cocos nucifera L.	Arecaceae	0,5	24,5	11	14	0,04	0,05	112,30	0,11	1,32	4,84
830	18	2	Banjaranyar	<200	Sengon	Paraserianthes falcataria (L.) I.C.	Leguminosae / Fabaceae	0,33	5,4	5	9	0,04	0,00	6,69	0,01	0,08	0,29
831	18	2	Banjaranyar	<200	Kelapa	Cocos nucifera L.	Arecaceae	0,5	28,0	2	5	0,04	0,06	43,00	0,04	0,51	1,85
832	18	2	Banjaranyar	<200	Alpukat	Persea americana Mill.	Lauraceae	0,54	24,8	7	12	0,04	0,05	222,76	0,22	2,62	9,61
833	18	2	Banjaranyar	<200	Alpukat	Persea americana Mill.	Lauraceae	0,54	4,8	2,5	5	0,04	0,00	4,87	0,00	0,06	0,21
834	18	2	Banjaranyar	<200	Sengon	Paraserianthes falcataria (L.) I.C.	Leguminosae / Fabaceae	0,33	2,5	3	3,5	0,04	0,00	0,71	0,00	0,01	0,03
835	18	2	Banjaranyar	<200	Kakao	Theobroma cacao L.	Malvaceae	0,36	4,5	2	3	0,04	0,00	2,33	0,00	0,03	0,10
836	18	2	Banjaranyar	<200	Sengon	Paraserianthes falcataria (L.) I.C.	Leguminosae / Fabaceae	0,33	8,0	9	10	0,04	0,00	14,93	0,01	0,18	0,64
837	18	2	Banjaranyar	<200	Mahoni	Swietenia macrophylla King	Meliaceae	0,61	21,0	6	15	0,04	0,03	225,00	0,23	2,64	9,70
838	18	2	Banjaranyar	<200	Manggis	Mangostana garcinia Gaertn.	Clusiaceae	0,81	11,5	1,5	5	0,04	0,01	35,13	0,04	0,41	1,51
839	18	2	Banjaranyar	<200	Manggis	Mangostana garcinia Gaertn.	Clusiaceae	0,81	9,5	1	5	0,04	0,01	25,16	0,03	0,30	1,08
840	19	3	Banjaranyar	<200	Kelapa	Cocos nucifera L.	Arecaceae	0,5	24,5	9	12	0,04	0,05	96,90	0,10	1,14	4,18
841	19	3	Banjaranyar	<200	Jeruk ragi	Citrus microcarpa	Rutaceae	0,7	10,2	2	5	0,04	0,01	24,77	0,02	0,29	1,07
842	19	3	Banjaranyar	<200	Mahoni	Swietenia macrophylla King	Meliaceae	0,61	4,5	2	3	0,04	0,00	3,01	0,00	0,04	0,13
843	19	3	Banjaranyar	<200	Jeruk ragi	Citrus microcarpa	Rutaceae	0,7	11,1	2,5	4,5	0,04	0,01	26,50	0,03	0,31	1,14
844	19	3	Banjaranyar	<200	Nangka	Artocarpus heterophyllus Lam.	Moraceae	0,61	21,6	2,5	6,5	0,04	0,04	110,48	0,11	1,30	4,76
845	19	3	Banjaranyar	<200	Tisuk	Hibiscus macrophyllus Roxb. ex	Malvaceae	0,4	28,3	12	21	0,04	0,06	359,78	0,36	4,23	15,51
846	19	3	Banjaranyar	<200	Petai	Parkia speciosa Hassk.	Leguminosae / Fabaceae	0,45	10,2	4	8	0,04	0,01	25,42	0,03	0,30	1,10
847	19	3	Banjaranyar	<200	Kelapa	Cocos nucifera L.	Arecaceae	0,5	28,6	14	17	0,04	0,06	135,40	0,14	1,59	5,84
848	19	3	Banjaranyar	<200	Tisuk	Hibiscus macrophyllus Roxb. ex	Malvaceae	0,4	4,1	3	5	0,04	0,00	2,85	0,00	0,03	0,12
849	19	3	Banjaranyar	<200	Sengon	Paraserianthes falcataria (L.) I.C.	Leguminosae / Fabaceae	0,33	28,6	10	16	0,04	0,06	240,01	0,24	2,82	10,35
850	19	3	Banjaranyar	<200	Kopi	Coffea canephora Pierre ex A.Fro	Rubiaceae	0,41	2,2	2	2,5	0,04	0,00	1,45	0,00	0,02	0,06
851	19	3	Banjaranyar	<200	Caruy	Pterospermum javanicum Jungh.	Malvaceae	0,53	5,1	3	4,5	0,04	0,00	4,90	0,00	0,06	0,21
852	19	3	Banjaranyar	<200	Caruy	Pterospermum javanicum Jungh.	Malvaceae	0,53	20,4	10	15	0,04	0,03	186,97	0,19	2,20	8,06
853	19	3	Banjaranyar	<200	Gamal/Kalkiria	Gliricidia sepium (Jacq.) Walp.	Leguminosae / Fabaceae	0,58	8,6	5	7	0,04	0,01	20,79	0,02	0,24	0,90
854	19	3	Banjaranyar	<200	Mahoni	Swietenia macrophylla King	Meliaceae	0,61	2,9	1,8	3	0,04	0,00	1,34	0,00	0,02	0,06
855	19	3	Banjaranyar	<200	Langsat	Lansium domesticum	Meliaceae	0,7	8,9	2	6	0,04	0,01	22,92	0,02	0,27	0,99
856	19	3	Banjaranyar	<200	Kelapa	Cocos nucifera L.	Arecaceae	0,5	20,0	10	13	0,04	0,03	104,60	0,10	1,23	4,51
857	19	3	Banjaranyar	<200	Tisuk	Hibiscus macrophyllus Roxb. ex	Malvaceae	0,4	3,2	2	3	0,04	0,00	1,10	0,00	0,01	0,05
858	19	3	Banjaranyar	<200	Tisuk	Hibiscus macrophyllus Roxb. ex	Malvaceae	0,4	4,1	2	3,5	0,04	0,00	2,05	0,00	0,02	0,09
859	19	3	Banjaranyar	<200	Sengon	Paraserianthes falcataria (L.) I.C.	Leguminosae / Fabaceae	0,33	19,7	7	14	0,04	0,03	107,30	0,11	1,26	4,63
860	19	3	Banjaranyar	<200	Tisuk	Hibiscus macrophyllus Roxb. ex	Malvaceae	0,4	5,7	2,5	6,5	0,04	0,00	6,57	0,01	0,08	0,28
861	19	3	Banjaranyar	<200	Langsat	Lansium domesticum	Meliaceae	0,7	7,6	2	5	0,04	0,00	14,62	0,01	0,17	0,63
862	19	3	Banjaranyar	<200	Sengon	Paraserianthes falcataria (L.) I.C.	Leguminosae / Fabaceae	0,33	25,1	12	15	0,04	0,05	178,17	0,18	2,09	7,68

863	19	3	Banjaranyar	<200	Tisuk	Hibiscus macrophyllus Roxb. ex I	Malvaceae	0,4	4,8	2	4	0,04	0,00	3,02	0,00	0,04	0,13
864	19	3	Banjaranyar	<200	Kelapa	Cocos nucifera L.	Arecaceae	0,5	19,7	2	5	0,04	0,03	43,00	0,04	0,51	1,85
865	19	3	Banjaranyar	<200	Kelapa	Cocos nucifera L.	Arecaceae	0,5	21,3	11	14	0,04	0,04	112,30	0,11	1,32	4,84
866	19	3	Banjaranyar	<200	Tisuk	Hibiscus macrophyllus Roxb. ex I	Malvaceae	0,4	7,3	6	10	0,04	0,00	15,29	0,02	0,18	0,66
867	19	3	Banjaranyar	<200	Gamal/Kalkiria	Gliricidia sepium (Jacq.) Walp.	Leguminosae / Fabaceae	0,58	12,7	3	4	0,04	0,01	25,58	0,03	0,30	1,10
868	19	3	Banjaranyar	<200	Langsat	Lansium domesticum	Meliaceae	0,7	8,3	4	8	0,04	0,01	26,04	0,03	0,31	1,12
869	20	3	Banjaranyar	<200	Sengon	Paraserianthes falcataria (L.) I.C.	Leguminosae / Fabaceae	0,33	16,9	10	13	0,04	0,02	75,22	0,08	0,88	3,24
870	20	3	Banjaranyar	<200	Tisuk	Hibiscus macrophyllus Roxb. ex I	Malvaceae	0,4	3,5	3,5	6	0,04	0,00	2,48	0,00	0,03	0,11
871	20	3	Banjaranyar	<200	Kelapa	Cocos nucifera L.	Arecaceae	0,5	24,8	11	14	0,04	0,05	112,30	0,11	1,32	4,84
872	20	3	Banjaranyar	<200	Tisuk	Hibiscus macrophyllus Roxb. ex I	Malvaceae	0,4	5,7	4	7	0,04	0,00	7,04	0,01	0,08	0,30
873	20	3	Banjaranyar	<200	Tisuk	Hibiscus macrophyllus Roxb. ex I	Malvaceae	0,4	6,7	5	7	0,04	0,00	9,33	0,01	0,11	0,40
874	20	3	Banjaranyar	<200	Baros	Manglietia glauca Blume	Magnoliaceae	0,38	2,5	2	2,5	0,04	0,00	0,59	0,00	0,01	0,03
875	20	3	Banjaranyar	<200	Kakao	Theobroma cacao L.	Malvaceae	0,36	4,5	0,5	2	0,04	0,00	2,33	0,00	0,03	0,10
876	20	3	Banjaranyar	<200	Mangga	Mangifera indica L.	Anacardiaceae	0,6	4,5	2	3	0,04	0,00	2,96	0,00	0,03	0,13
877	20	3	Banjaranyar	<200	Mahoni	Swietenia macrophylla King	Meliaceae	0,61	13,4	8	14	0,04	0,01	92,28	0,09	1,08	3,98
878	20	3	Banjaranyar	<200	Sengon	Paraserianthes falcataria (L.) I.C.	Leguminosae / Fabaceae	0,33	14,3	10	14	0,04	0,02	59,65	0,06	0,70	2,57
879	20	3	Banjaranyar	<200	Mahoni	Swietenia macrophylla King	Meliaceae	0,61	11,5	4	9	0,04	0,01	46,42	0,05	0,55	2,00
880	20	3	Banjaranyar	<200	Kelapa	Cocos nucifera L.	Arecaceae	0,5	22,9	4	7	0,04	0,04	58,40	0,06	0,69	2,52
881	20	3	Banjaranyar	<200	Kelapa	Cocos nucifera L.	Arecaceae	0,5	24,2	6	9	0,04	0,05	73,80	0,07	0,87	3,18
882	20	3	Banjaranyar	<200	Mahoni	Swietenia macrophylla King	Meliaceae	0,61	16,5	7	14	0,04	0,02	136,47	0,14	1,60	5,89
883	20	3	Banjaranyar	<200	Rambutan	Nephelium lappaceum L.	Sapindaceae	0,71	8,3	4	7	0,04	0,01	23,35	0,02	0,27	1,01
884	20	3	Banjaranyar	<200	Sengon	Paraserianthes falcataria (L.) I.C.	Leguminosae / Fabaceae	0,33	19,4	10	14	0,04	0,03	104,15	0,10	1,22	4,49
885	20	3	Banjaranyar	<200	Kelapa	Cocos nucifera L.	Arecaceae	0,5	24,2	8	12	0,04	0,05	96,90	0,10	1,14	4,18
886	20	3	Banjaranyar	<200	Sengon	Paraserianthes falcataria (L.) I.C.	Leguminosae / Fabaceae	0,33	7,6	7	11	0,04	0,00	15,12	0,02	0,18	0,65
887	20	3	Banjaranyar	<200	Kelapa	Cocos nucifera L.	Arecaceae	0,5	21,6	4	7	0,04	0,04	58,40	0,06	0,69	2,52
888	20	3	Banjaranyar	<200	Sengon	Paraserianthes falcataria (L.) I.C.	Leguminosae / Fabaceae	0,33	8,6	5	10	0,04	0,01	17,19	0,02	0,20	0,74
889	20	3	Banjaranyar	<200	Mahoni	Swietenia macrophylla King	Meliaceae	0,61	4,1	3	4	0,04	0,00	3,42	0,00	0,04	0,15
890	20	3	Banjaranyar	<200	Jati	Tectona grandis L.f.	Lamiaceae / Verbenaceae	0,7	4,1	3	3,5	0,04	0,00	3,43	0,00	0,04	0,15
891	20	3	Banjaranyar	<200	Heuras	Vitex pubescens Vahl	Lamiaceae / Verbenaceae	0,88	6,0	5	8	0,04	0,00	18,08	0,02	0,21	0,78
892	20	3	Banjaranyar	<200	Nangka	Artocarpus heterophyllus Lam.	Moraceae	0,61	6,7	4	6	0,04	0,00	11,93	0,01	0,14	0,51
893	20	3	Banjaranyar	<200	Mahoni	Swietenia macrophylla King	Meliaceae	0,61	4,8	2,5	5,5	0,04	0,00	5,95	0,01	0,07	0,26
894	20	3	Banjaranyar	<200	Kelapa	Cocos nucifera L.	Arecaceae	0,5	31,8	11	14	0,04	0,08	112,30	0,11	1,32	4,84
895	20	3	Banjaranyar	<200	Tisuk	Hibiscus macrophyllus Roxb. ex I	Malvaceae	0,4	5,4	3	7	0,04	0,00	6,34	0,01	0,07	0,27
896	20	3	Banjaranyar	<200	Kakao	Theobroma cacao L.	Malvaceae	0,36	7,0	0,5	3	0,04	0,00	5,69	0,01	0,07	0,25
897	20	3	Banjaranyar	<200	Kelapa	Cocos nucifera L.	Arecaceae	0,5	28,6	8	11	0,04	0,06	89,20	0,09	1,05	3,85
898	20	3	Banjaranyar	<200	Mahoni	Swietenia macrophylla King	Meliaceae	0,61	11,8	3	9	0,04	0,01	48,81	0,05	0,57	2,10
899	20	3	Banjaranyar	<200	Heuras	Vitex pubescens Vahl	Lamiaceae / Verbenaceae	0,88	3,8	4	5	0,04	0,00	5,07	0,01	0,06	0,22
900	20	3	Banjaranyar	<200	Kelapa	Cocos nucifera L.	Arecaceae	0,5	26,1	8	11	0,04	0,05	89,20	0,09	1,05	3,85
901	20	3	Banjaranyar	<200	Tisuk	Hibiscus macrophyllus Roxb. ex I	Malvaceae	0,4	3,2	4	5	0,04	0,00	1,76	0,00	0,02	0,08
902	20	3	Banjaranyar	<200	Heuras	Vitex pubescens Vahl	Lamiaceae / Verbenaceae	0,88	17,2	4	10	0,04	0,02	150,32	0,15	1,77	6,48
903	20	3	Banjaranyar	<200	Sengon	Paraserianthes falcataria (L.) I.C.	Leguminosae / Fabaceae	0,33	3,8	4	5	0,04	0,00	2,06	0,00	0,02	0,09
904	20	3	Banjaranyar	<200	pangsor	Ficus callosa Willd.	Moraceae	0,33	3,2	2,5	3,5	0,04	0,00	1,07	0,00	0,01	0,05
905	20	3	Banjaranyar	<200	Sengon	Paraserianthes falcataria (L.) I.C.	Leguminosae / Fabaceae	0,33	7,0	10	12	0,04	0,00	13,96	0,01	0,16	0,60
906	20	3	Banjaranyar	<200	Tisuk	Hibiscus macrophyllus Roxb. ex I	Malvaceae	0,4	3,2	2	2,5	0,04	0,00	0,93	0,00	0,01	0,04
907	20	3	Banjaranyar	<200	Baros	Manglietia glauca Blume	Magnoliaceae	0,38	2,5	3,5	4	0,04	0,00	0,91	0,00	0,01	0,04
908	20	3	Banjaranyar	<200	Kelapa	Cocos nucifera L.	Arecaceae	0,5	24,8	3	6	0,04	0,05	50,70	0,05	0,60	2,19
909	20	3	Banjaranyar	<200	Kelapa	Cocos nucifera L.	Arecaceae	0,5	23,2	4	7	0,04	0,04	58,40	0,06	0,69	2,52
910	20	3	Banjaranyar	<200	Cengkeh	Syzygium aromaticum (L.) Merr. ε	Myrtaceae	0,79	3,2	2	3	0,04	0,00	2,06	0,00	0,02	0,09
911	20	3	Banjaranyar	<200	Kakao	Theobroma cacao L.	Malvaceae	0,36	3,8	0,8	2,5	0,04	0,00	1,71	0,00	0,02	0,07
912	20	3	Banjaranyar	<200	Mahoni	Swietenia macrophylla King	Meliaceae	0,61	23,9	10	15	0,04	0,04	284,38	0,28	3,34	12,26
913	20	3	Banjaranyar	<200	Tisuk	Hibiscus macrophyllus Roxb. ex I	Malvaceae	0,4	9,2	7	10	0,04	0,01	23,37	0,02	0,27	1,01
914	20	3	Banjaranyar	<200	Sengon	Paraserianthes falcataria (L.) I.C.	Leguminosae / Fabaceae	0,33	25,5	12	16	0,04	0,05	193,43	0,19	2,27	8,34
915	20	3	Banjaranyar	<200	Jati	Tectona grandis L.f.	Lamiaceae / Verbenaceae	0,7	8,6	3	8	0,04	0,01	27,91	0,03	0,33	1,20
916	20	3	Banjaranyar	<200	Tisuk	Hibiscus macrophyllus Roxb. ex I	Malvaceae	0,4	7,0	4	8	0,04	0,00	11,49	0,01	0,13	0,50
917	20	3	Banjaranyar	<200	Kelapa	Cocos nucifera L.	Arecaceae	0,5	25,5	11	14	0,04	0,05	112,30	0,11	1,32	4,84
918	20	3	Banjaranyar	<200	Heuras	Vitex pubescens Vahl	Lamiaceae / Verbenaceae	0,88	6,7	7	8,5	0,04	0,00	22,96	0,02	0,27	0,99
919	20	3	Banjaranyar	<200	Tisuk	Hibiscus macrophyllus Roxb. ex I	Malvaceae	0,4	5,4	4	5	0,04	0,00	4,66	0,00	0,05	0,20
920	20	3	Banjaranyar	<200	Tisuk	Hibiscus macrophyllus Roxb. ex I	Malvaceae	0,4	8,9	7	10	0,04	0,01	21,92	0,02	0,26	0,95
921	20	3	Banjaranyar	<200	Salam	Syzygium polyanthum (Wight) W	Myrtaceae	0,66	5,4	4	7	0,04	0,00	10,03	0,01	0,12	0,43
922	20	3	Banjaranyar	<200	Sengon	Paraserianthes falcataria (L.) I.C.	Leguminosae / Fabaceae	0,33	10,5	10	13	0,04	0,01	31,58	0,03	0,37	1,36
923	20	3	Banjaranyar	<200	Tisuk	Hibiscus macrophyllus Roxb. ex I	Malvaceae	0,4	4,1	4	5	0,04	0,00	2,85	0,00	0,03	0,12
924	20	3	Banjaranyar	<200	Heuras	Vitex pubescens Vahl	Lamiaceae / Verbenaceae	0,88	5,4	5	7	0,04	0,00	13,05	0,01	0,15	0,56
925	20	3	Banjaranyar	<200	Tisuk	Hibiscus macrophyllus Roxb. ex I	Malvaceae	0,4	9,5	7	10	0,04	0,01	24,87	0,02	0,29	1,07
926	20	3	Banjaranyar	<200	Cengkeh	Syzygium aromaticum (L.) Merr. ε	Myrtaceae	0,79	2,5	2	3	0,04	0,00	1,37	0,00	0,02	0,06
927	20	3	Banjaranyar	<200	Nangka	Artocarpus heterophyllus Lam.	Moraceae	0,61	3,5	1	3	0,04	0,00	1,93	0,00	0,02	0,08
928	20	3	Banjaranyar	<200	Jati	Tectona grandis L.f.	Lamiaceae / Verbenaceae	0,7	13,7	7	12	0,04	0,01	94,90	0,09	1,12	4,09
929	20	3	Banjaranyar	<200	Tisuk	Hibiscus macrophyllus Roxb. ex I	Malvaceae	0,4	16,9	10	13	0,04	0,02	89,71	0,09	1,05	3,87
930	20	3	Banjaranyar	<200	Tisuk	Hibiscus macrophyllus Roxb. ex I	Malvaceae	0,4	6,4	5	8	0,04	0,00	9,65	0,01	0,11	0,42
931	20	3	Banjaranyar	<200	Sengon	Paraserianthes falcataria (L.) I.C.	Leguminosae / Fabaceae	0,33	9,5	9	11	0,04	0,01	22,76	0,02	0,27	0,98
932	20	3	Banjaranyar	<200	Kelapa	Cocos nucifera L.	Arecaceae	0,5	24,8	10	13	0,04	0,05	104,60	0,10	1,23	4,51
933	20	3	Banjaranyar	<200	Tisuk	Hibiscus macrophyllus Roxb. ex I	Malvaceae	0,4	4,8	4	6	0,04	0,00	4,38	0,00	0,05	0,19
934	20	3	Banjaranyar	<200	Tisuk	Hibiscus macrophyllus Roxb. ex I	Malvaceae	0,4	3,2	3	3,5	0,04	0,00	1,27	0,00	0,01	0,05
935	20	3	Banjaranyar	<200	Mahoni	Swietenia macrophylla King	Meliaceae	0,61	22,3	2	3	0,04	0,04	57,38	0,06	0,67	2,47
936	20	3	Banjaranyar	<200	Tisuk	Hibiscus macrophyllus Roxb. ex I	Malvaceae	0,4	3,2	3	3,5	0,04	0,00	1,27	0,00	0,01	0,05
937	21	1	Banjaranyar	<200	Jati	Tectona grandis L.f.	Lamiaceae / Verbenaceae	0,7	43,0	9	16	0,04	0,14	1.004,56	1,00	11,80	43,32
938	21	1	Banjaranyar	<200	Sengon	Paraserianthes falcataria (L.) I.C.	Leguminosae / Fabaceae	0,33	35,6	14	20	0,04	0,10	439,53	0,44	5,16	18,95
939	21	1	Banjaranyar	<200	Mahoni	Swietenia macrophylla King	Meliaceae	0,61	11,1	5	10	0,04	0,01	48,55	0,05	0,57	2,09
940	21	1	Banjaranyar	<200	Mahoni	Swietenia macrophylla King	Meliaceae	0,61	9,9	5	9	0,04	0,01	35,30	0,04	0,41	1,52
941	21	1	Banjaranyar	<200	Kelapa	Cocos nucifera L.	Arecaceae	0,5	25,5	10	14	0,04	0,05	112,30	0,11	1,32	4,84
942	21	1	Banjaranyar	<200	Sengon	Paraserianthes falcataria (L.) I.C.	Leguminosae / Fabaceae	0,33	19,7	12	15	0,04	0,03	114,30	0,11	1,34	4,93
943	21	1	Banjaranyar	<200	Mahoni	Swietenia macrophylla King	Meliaceae	0,61	11,1	5	9	0,04	0,01	44,09	0,04	0,52	1,90
944	21	1	Banjaranyar	<200	Sengon	Paraserianthes falcataria (L.) I.C.	Leguminosae / Fabaceae	0,33	21,3	15	18	0,04	0,04	155,70	0,16	1,83	6,71
945	21	1	Banjaranyar	<200	Sengon	Paraserianthes falcataria (L.) I.C.	Leguminosae / Fabaceae	0,33	29,9	14	18	0,04	0,07	289,52	0,29	3,40	12,48
946	21	1	Banjaranyar	<200	Jati	Tectona grandis L.f.	Lamiaceae / Verbenaceae	0,7	8,6	5	8	0,04	0,01	27,91	0,03	0,33	1,20
947	21	1	Banjaranyar	<200	Sengon	Paraserianthes falcataria (L.) I.C.	Leguminosae / Fabaceae	0,33	7,3	11	14	0,04	0,00	17,44	0,02	0,20	0,75
948	21	1	Banjaranyar	<200	Jati	Tectona grandis L.f.	Lamiaceae / Verbenaceae	0,7	9,9	5	7	0,04	0,01	31,81	0,03	0,37	1,37
949	21	1	Banjaranyar	<200	Sengon	Paraserianthes falcataria (L.) I.C.	Leguminosae / Fabaceae	0,33	34,7	18	23	0,04	0,09	475,32	0,48	5,59	20,50
950	21	1	Banjaranyar	<200	Jati	Tectona grandis L.f.	Lamiaceae / Verbenaceae	0,7	33,4	10	18	0,04	0,09	706,12	0,71	8,30	30,45
951	21	1	Banjaranyar	<200	Jati	Tectona grandis L.f.	Lamiaceae / Verbenaceae	0,7	9,9	5	7	0,04	0,01	31,81	0,03	0,37	1,37
952	21	1	Banjaranyar	<200	Jati	Tectona grandis L.f.	Lamiaceae / Verbenaceae	0,7	13,4	8	12	0,04	0,01	90,90	0,09	1,07	3,92
953	21	1	Banjaranyar	<200	Jati	Tectona grandis L.f.	Lamiaceae / Verbenaceae	0,7	30,9	10	17	0,04	0,07	579,54	0,58	6,81	24,99
954	21	1	Banjaranyar	<200	Jati	Tectona grandis L.f.	Lamiaceae / Verbenaceae	0,7	30,2	9	15	0,04	0,07	497,41	0,50	5,84	21,45
955	21	1	Banjaranyar	<200	Jati	Tectona grandis L.f.	Lamiaceae / Verbenaceae	0,7	34,0	8	17	0,04	0,09	693,67	0,69	8,15	29,91
956	21	1	Banjaranyar	<200	Kelapa	Cocos nucifera L.	Arecaceae	0,5	27,0	13	16	0,04	0,06	127,70	0,13	1,50	5,51
957	21	1	Banjaranyar	<200	Salam	Syzygium polyanthum (Wight) W	Myrtaceae	0,66	8,6	6	12	0,04	0,01	38,34	0,04	0,45	1,65
958	21	1	Banjaranyar	<200	Kedondong	Spondias dulcis Parkinson	Anacardiaceae	0,3	8,6	9	9,5	0,04	0,01	15,03	0,02	0,18	0,65
959	21	1	Banjaranyar	<200	Jati	Tectona grandis L.f.	Lamiaceae / Verbenaceae	0,7	32,8	10	14	0,04	0,08	541,50	0,54	6,36	23,35
960	21	1	Banjaranyar	<200	Kelapa	Cocos nucifera L.	Arecaceae	0,5	21,0	10	13	0,04	0,03	104,60	0,10	1,23	4,51
961	21	1	Banjaranyar	<200	Jati	Tectona grandis L.f.	Lamiaceae / Verbenaceae	0,7	33,7	7	16	0,04	0,09	645,01	0,65	7,58	27,81
962	21	1	Banjaranyar	<200	Kelapa	Cocos nucifera L.	Arecaceae	0,5	21,6	7	10	0,04	0,04	81,50	0,08	0,96	3,51
963	21	1	Banjaranyar	<200	Jati	Tectona grandis L.f.	Lamiaceae / Verbenaceae	0,7	32,5	8	16	0,04	0,08	601,12	0,60	7,06	25,92
964	21	1	Banjaranyar	<200	Jati	Tectona grandis L.f.	Lamiaceae / Verbenaceae	0,7	8,6	5	9	0,04	0,01	31,09	0,03	0,37	1,34
965	21	1	Banjaranyar	<200	Jati	Tectona grandis L.f.	Lamiaceae / Verbenaceae	0,7	26,7	10	17	0,04	0,06	445,25	0,45	5,23	19,20
966	21	1	Banjaranyar	<200	Wangkal	Albizia procera Benth	Leguminosae / Fabaceae	0,77	17,2	15	18	0,04	0,02	227,89	0,23	2,68	9,83
967	21	1	Banjaranyar	<200	jati	Tectona grandis L.f.	Lamiaceae / Verbenaceae	0,7	25,8	8	15	0,04	0,05	371,43	0,37	4,36	16,02
968	21	1	Banjaranyar	<200	jati	Tectona grandis L.f.	Lamiaceae / Verbenaceae	0,7	28,6	12	18	0,04	0,06	532,39	0,53	6,26	22,96
969	21	1	Banjaranyar	<200	jati	Tectona grandis L.f.	Lamiaceae / Verbenaceae	0,7	19,4	7	14	0,04	0,03	207,40	0,21	2,44	8,94
970	22	1	Banjaranyar	<200	Jati	Tectona grandis L.f.	Lamiaceae / Verbenaceae	0,7	19,1	2	11	0,04	0,03	161,33	0,16	1,90	6,96
971	22	1	Banjaranyar	<200	Jati	Tectona grandis L.f.	Lamiaceae / Verbenaceae	0,7	20,0	2	11	0,04	0,03	176,42	0,18	2,07	7,61
972	22	1	Banjaranyar	<200	Jati	Tectona grandis L.f.	Lamiaceae / Verbenaceae	0,7	12,1	3	7	0,04	0,01	46,19	0,05	0,54	1,99
973	22	1	Banjaranyar	<200	Jati	Tectona grandis L.f.	Lamiaceae / Verbenaceae	0,7	9,5	2	8	0,04	0,01	33,85	0,03	0,40	1,46
974	22	1	Banjaranyar	<200	Jati	Tectona grandis L.f.	Lamiaceae / Verbenaceae	0,7	14,6	2,5	9,5	0,04	0,02	86,70	0,09	1,02	3,74
975	22	1	Banjaranyar	<200	Tisuk	Hibiscus macrophyllus Roxb. ex I	Malvaceae	0,4	12,7	8	11	0,04	0,01	45,97	0,05	0,54	1,98
976	22	1	Banjaranyar	<200	Tisuk	Hibiscus macrophyllus Roxb. ex I	Malvaceae	0,4	3,2	2	3	0,04	0,00	1,10	0,00	0,01	0,05
977	22	1	Banjaranyar	<200	Kelapa	Cocos nucifera L.	Arecaceae	0,5	22,6	4	7	0,04	0,04	58,40	0,06	0,69	2,52
978	22	1	Banjaranyar	<200	Jati	Tectona grandis L.f.	Lamiaceae / Verbenaceae	0,7	11,5	3	8	0,04	0,01	47,27	0,05	0,56	2,04
979	22	1	Banjaranyar	<200	Jati	Tectona grandis L.f.	Lamiaceae / Verbenaceae	0,7	11,8	2,5	4,5	0,04	0,01	29,34	0,03	0,34	1,27
980	22	1	Banjaranyar	<200	Jati	Tectona grandis L.f.	Lamiaceae / Verbenaceae	0,7	6,4	2,5	4	0,04	0,00	8,53	0,01	0,10	0,37
981	22	1	Banjaranyar	<200	Kelapa	Cocos nucifera L.	Arecaceae	0,5	23,5	4	7	0,04	0,04	58,40	0,06	0,69	2,52
982	22	1	Banjaranyar	<200	Jati	Tectona grandis L.f.	Lamiaceae / Verbenaceae	0,7	7,6	1	4	0,04	0,00	11,92	0,01	0,14	0,51
983	22	1	Banjaranyar	<200	Mahoni	Swietenia macrophylla King	Meliaceae	0,61	5,4	4	6	0,04	0,00	8,10	0,01	0,10	0,35
984	22	1	Banjaranyar	<200	Mahoni	Swietenia macrophylla King	Meliaceae	0,61	18,5	4	9	0,04	0,03	111,22	0,11	1,31	4,80
985	22	1	Banjaranyar	<200	Sengon	Paraserianthes falcataria (L.) I.C.	Leguminosae / Fabaceae	0,33	3,8	5	6	0,04	0,00	2,44	0,00	0,03	0,11
986	22	1	Banjaranyar	<200	Jati	Tectona grandis L.f.	Lamiaceae / Verbenaceae	0,7	14,6	2	8	0,04	0,02	74,07	0,07	0,87	3,19

987	22	1	Banjaranyar	<200	Sengon	Paraserianthes falcataria (L.) I.C.	Leguminosae / Fabaceae	0,33	19,4	9	14	0,04	0,03	104,15	0,10	1,22	4,49
988	22	1	Banjaranyar	<200	Sengon	Paraserianthes falcataria (L.) I.C.	Leguminosae / Fabaceae	0,33	11,8	9	12	0,04	0,01	36,19	0,04	0,43	1,56
989	22	1	Banjaranyar	<200	Sengon	Paraserianthes falcataria (L.) I.C.	Leguminosae / Fabaceae	0,33	8,9	9	11	0,04	0,01	20,05	0,02	0,24	0,86
990	22	1	Banjaranyar	<200	Sengon	Paraserianthes falcataria (L.) I.C.	Leguminosae / Fabaceae	0,33	18,8	10	13	0,04	0,03	91,55	0,09	1,08	3,95
991	22	1	Banjaranyar	<200	Bintinu	Melochia umbellata (Houtt.) Stap	Malvaceae	0,25	11,8	4	9	0,04	0,01	21,56	0,02	0,25	0,93
992	22	1	Banjaranyar	<200	Kelapa	Cocos nucifera L.	Arecaceae	0,5	17,8	6	9	0,04	0,02	73,80	0,07	0,87	3,18
993	22	1	Banjaranyar	<200	Tisuk	Hibiscus macrophyllus Roxb. ex	Malvaceae	0,4	6,7	4	7	0,04	0,00	9,33	0,01	0,11	0,40
994	22	1	Banjaranyar	<200	Tisuk	Hibiscus macrophyllus Roxb. ex	Malvaceae	0,4	4,5	4	6	0,04	0,00	3,86	0,00	0,05	0,17
995	22	1	Banjaranyar	<200	Langsat	Lansium domesticum	Meliaceae	0,7	7,6	2	6	0,04	0,00	17,28	0,02	0,20	0,75
996	22	1	Banjaranyar	<200	Nangka	Artocarpus heterophyllus Lam.	Moraceae	0,61	11,5	3	9	0,04	0,01	46,42	0,05	0,55	2,00
997	22	1	Banjaranyar	<200	Mahoni	Swietenia macrophylla King	Meliaceae	0,61	8,6	6	7	0,04	0,01	21,77	0,02	0,26	0,94
998	22	1	Banjaranyar	<200	Sengon	Paraserianthes falcataria (L.) I.C.	Leguminosae / Fabaceae	0,33	5,1	6	7,5	0,04	0,00	5,07	0,01	0,06	0,22
999	22	1	Banjaranyar	<200	Alpukat	Persea americana Mill.	Lauraceae	0,54	5,7	2,5	6	0,04	0,00	8,04	0,01	0,09	0,35
1000	22	1	Banjaranyar	<200	Sengon	Paraserianthes falcataria (L.) I.C.	Leguminosae / Fabaceae	0,33	9,2	8	10	0,04	0,01	19,60	0,02	0,23	0,85
1001	22	1	Banjaranyar	<200	Tisuk	Hibiscus macrophyllus Roxb. ex	Malvaceae	0,4	12,7	7	10	0,04	0,01	42,13	0,04	0,50	1,82
1002	22	1	Banjaranyar	<200	Tisuk	Hibiscus macrophyllus Roxb. ex	Malvaceae	0,4	3,8	4	5	0,04	0,00	2,46	0,00	0,03	0,11
1003	22	1	Banjaranyar	<200	Sengon	Paraserianthes falcataria (L.) I.C.	Leguminosae / Fabaceae	0,33	17,2	10	13	0,04	0,02	77,84	0,08	0,91	3,36
1004	22	1	Banjaranyar	<200	Sengon	Paraserianthes falcataria (L.) I.C.	Leguminosae / Fabaceae	0,33	9,2	8	10	0,04	0,01	19,60	0,02	0,23	0,85
1005	22	1	Banjaranyar	<200	Sengon	Paraserianthes falcataria (L.) I.C.	Leguminosae / Fabaceae	0,33	17,2	8	9,5	0,04	0,02	58,40	0,06	0,69	2,52
1006	22	1	Banjaranyar	<200	Binong	Tetrameles nudiflora R. Br.	Tetramelaceae	0,3	3,2	2	2,5	0,04	0,00	0,72	0,00	0,01	0,03
1007	22	1	Banjaranyar	<200	Mahoni	Swietenia macrophylla King	Meliaceae	0,61	5,4	4	6	0,04	0,00	8,10	0,01	0,10	0,35
1008	22	1	Banjaranyar	<200	Jati	Tectona grandis L.f.	Lamiaceae / Verbenaceae	0,7	9,5	4	7	0,04	0,01	29,95	0,03	0,35	1,29
1009	22	1	Banjaranyar	<200	Kelapa	Cocos nucifera L.	Arecaceae	0,5	21,6	3,5	6,5	0,04	0,04	54,55	0,05	0,64	2,35
1010	22	1	Banjaranyar	<200	Tisuk	Hibiscus macrophyllus Roxb. ex	Malvaceae	0,4	8,3	7	10	0,04	0,01	19,14	0,02	0,22	0,83
1011	22	1	Banjaranyar	<200	Jati	Tectona grandis L.f.	Lamiaceae / Verbenaceae	0,7	9,2	6	10	0,04	0,01	39,02	0,04	0,46	1,68
1012	22	1	Banjaranyar	<200	Tisuk	Hibiscus macrophyllus Roxb. ex	Malvaceae	0,4	5,1	6	9	0,04	0,00	7,14	0,01	0,08	0,31
1013	22	1	Banjaranyar	<200	Jati	Tectona grandis L.f.	Lamiaceae / Verbenaceae	0,7	13,4	3	10	0,04	0,01	76,92	0,08	0,90	3,32
1014	22	1	Banjaranyar	<200	Wangkal	Albizia procera Benth	Leguminosae / Fabaceae	0,77	4,1	6	9	0,04	0,00	8,89	0,01	0,10	0,38
1015	22	1	Banjaranyar	<200	Tisuk	Hibiscus macrophyllus Roxb. ex	Malvaceae	0,4	4,5	4	6	0,04	0,00	3,86	0,00	0,05	0,17
1016	22	1	Banjaranyar	<200	Tisuk	Hibiscus macrophyllus Roxb. ex	Malvaceae	0,4	4,1	4	5	0,04	0,00	2,85	0,00	0,03	0,12
1017	22	1	Banjaranyar	<200	Jati	Tectona grandis L.f.	Lamiaceae / Verbenaceae	0,7	11,8	8	12	0,04	0,01	72,06	0,07	0,85	3,11
1018	22	1	Banjaranyar	<200	Tisuk	Hibiscus macrophyllus Roxb. ex	Malvaceae	0,4	17,8	8	10	0,04	0,02	78,03	0,08	0,92	3,37
1019	22	1	Banjaranyar	<200	Tisuk	Hibiscus macrophyllus Roxb. ex	Malvaceae	0,4	3,8	3	4	0,04	0,00	2,01	0,00	0,02	0,09
1020	22	1	Banjaranyar	<200	Tisuk	Hibiscus macrophyllus Roxb. ex	Malvaceae	0,4	4,8	4	6	0,04	0,00	4,38	0,00	0,05	0,19
1021	22	1	Banjaranyar	<200	Jati	Tectona grandis L.f.	Lamiaceae / Verbenaceae	0,7	13,7	7	11	0,04	0,01	87,63	0,09	1,03	3,78
1022	22	1	Banjaranyar	<200	Tisuk	Hibiscus macrophyllus Roxb. ex	Malvaceae	0,4	10,5	6	9	0,04	0,01	26,89	0,03	0,32	1,16
1023	22	1	Banjaranyar	<200	Jati	Tectona grandis L.f.	Lamiaceae / Verbenaceae	0,7	8,3	2,5	7	0,04	0,01	23,04	0,02	0,27	0,99
1024	22	1	Banjaranyar	<200	Tisuk	Hibiscus macrophyllus Roxb. ex	Malvaceae	0,4	10,8	5	9	0,04	0,01	28,40	0,03	0,33	1,22
1025	22	1	Banjaranyar	<200	Jati	Tectona grandis L.f.	Lamiaceae / Verbenaceae	0,7	21,0	4	9	0,04	0,03	159,85	0,16	1,88	6,89
1026	22	1	Banjaranyar	<200	Jati	Tectona grandis L.f.	Lamiaceae / Verbenaceae	0,7	14,6	3,5	8	0,04	0,02	74,07	0,07	0,87	3,19
1027	22	1	Banjaranyar	<200	Mahoni	Swietenia macrophylla King	Meliaceae	0,61	3,5	3	4	0,04	0,00	2,52	0,00	0,03	0,11
1028	22	1	Banjaranyar	<200	Sengon	Paraserianthes falcataria (L.) I.C.	Leguminosae / Fabaceae	0,33	7,0	7	9	0,04	0,00	10,73	0,01	0,13	0,46
1029	22	1	Banjaranyar	<200	jeungjing	Albizia falcata (L.) Backer	Leguminosae / Fabaceae	0,37	12,7	7	10	0,04	0,01	39,23	0,04	0,46	1,69
1030	22	1	Banjaranyar	<200	jeungjing	Albizia falcata (L.) Backer	Leguminosae / Fabaceae	0,37	9,9	8	9	0,04	0,01	22,33	0,02	0,26	0,96
1031	22	1	Banjaranyar	<200	Mahoni	Swietenia macrophylla King	Meliaceae	0,61	5,1	3,5	5,5	0,04	0,00	6,69	0,01	0,08	0,29
1032	22	1	Banjaranyar	<200	Tisuk	Hibiscus macrophyllus Roxb. ex	Malvaceae	0,4	4,1	4	5	0,04	0,00	2,85	0,00	0,03	0,12
1033	22	1	Banjaranyar	<200	Sengon	Paraserianthes falcataria (L.) I.C.	Leguminosae / Fabaceae	0,33	4,1	5	6	0,04	0,00	2,82	0,00	0,03	0,12
1034	22	1	Banjaranyar	<200	Kelapa	Cocos nucifera L.	Arecaceae	0,5	24,2	7	10	0,04	0,05	81,50	0,08	0,96	3,51
1035	22	1	Banjaranyar	<200	Wangkal	Albizia procera Benth	Leguminosae / Fabaceae	0,77	6,0	3	5	0,04	0,00	10,40	0,01	0,12	0,45
1036	22	1	Banjaranyar	<200	Mahoni	Swietenia macrophylla King	Meliaceae	0,61	10,2	5	8	0,04	0,01	33,59	0,03	0,39	1,45
1037	22	1	Banjaranyar	<200	Mahoni	Swietenia macrophylla King	Meliaceae	0,61	11,5	5	9	0,04	0,01	46,42	0,05	0,55	2,00
1038	22	1	Banjaranyar	<200	Sengon	Paraserianthes falcataria (L.) I.C.	Leguminosae / Fabaceae	0,33	6,4	6	9	0,04	0,00	9,01	0,01	0,11	0,39
1039	22	1	Banjaranyar	<200	Wangkal	Albizia procera Benth	Leguminosae / Fabaceae	0,77	8,3	6	9	0,04	0,01	31,66	0,03	0,37	1,37
1040	23	2	Banjaranyar	<200	Kelapa	Cocos nucifera L.	Arecaceae	0,5	31,2	11	14	0,04	0,08	112,30	0,11	1,32	4,84
1041	23	2	Banjaranyar	<200	Tisuk	Hibiscus macrophyllus Roxb. ex	Malvaceae	0,4	11,1	6	8	0,04	0,01	26,89	0,03	0,32	1,16
1042	23	2	Banjaranyar	<200	Jati	Tectona grandis L.f.	Lamiaceae / Verbenaceae	0,7	4,5	2	3	0,04	0,00	3,41	0,00	0,04	0,15
1043	23	2	Banjaranyar	<200	Tisuk	Hibiscus macrophyllus Roxb. ex	Malvaceae	0,4	8,3	6	8	0,04	0,01	15,60	0,02	0,18	0,67
1044	23	2	Banjaranyar	<200	Gmelina	Gmelina arborea Roxb.	Lamiaceae / Verbenaceae	0,47	10,5	7	8,5	0,04	0,01	29,58	0,03	0,35	1,28
1045	23	2	Banjaranyar	<200	Jati	Tectona grandis L.f.	Lamiaceae / Verbenaceae	0,7	3,8	2	4	0,04	0,00	3,35	0,00	0,04	0,14
1046	23	2	Banjaranyar	<200	Gmelina	Gmelina arborea Roxb.	Lamiaceae / Verbenaceae	0,47	13,7	3	10	0,04	0,01	55,75	0,06	0,66	2,40
1047	23	2	Banjaranyar	<200	Gmelina	Gmelina arborea Roxb.	Lamiaceae / Verbenaceae	0,47	6,4	6	7	0,04	0,00	9,89	0,01	0,12	0,43
1048	23	2	Banjaranyar	<200	Sengon	Paraserianthes falcataria (L.) I.C.	Leguminosae / Fabaceae	0,33	39,5	12	19	0,04	0,12	505,32	0,51	5,94	21,79
1049	23	2	Banjaranyar	<200	Jati	Tectona grandis L.f.	Lamiaceae / Verbenaceae	0,7	3,8	2	4	0,04	0,00	3,35	0,00	0,04	0,14
1050	23	2	Banjaranyar	<200	Jati	Tectona grandis L.f.	Lamiaceae / Verbenaceae	0,7	3,8	2	3	0,04	0,00	2,57	0,00	0,03	0,11
1051	23	2	Banjaranyar	<200	Gmelina	Gmelina arborea Roxb.	Lamiaceae / Verbenaceae	0,47	11,5	6	9	0,04	0,01	36,56	0,04	0,43	1,58
1052	23	2	Banjaranyar	<200	Pangsor	Ficus callosa Willd.	Moraceae	0,33	19,7	12	14	0,04	0,03	107,30	0,11	1,26	4,63
1053	23	2	Banjaranyar	<200	Tisuk	Hibiscus macrophyllus Roxb. ex	Malvaceae	0,4	3,8	4	5	0,04	0,00	2,46	0,00	0,03	0,11
1054	23	2	Banjaranyar	<200	Kelapa	Cocos nucifera L.	Arecaceae	0,5	20,0	4	7	0,04	0,03	58,40	0,06	0,69	2,52
1055	23	2	Banjaranyar	<200	Gmelina	Gmelina arborea Roxb.	Lamiaceae / Verbenaceae	0,47	3,5	3	4	0,04	0,00	1,98	0,00	0,02	0,09
1056	23	2	Banjaranyar	<200	Tisuk	Hibiscus macrophyllus Roxb. ex	Malvaceae	0,4	8,9	7	9	0,04	0,01	19,90	0,02	0,23	0,86
1057	23	2	Banjaranyar	<200	Jati	Tectona grandis L.f.	Lamiaceae / Verbenaceae	0,7	8,6	2	6	0,04	0,01	21,44	0,02	0,25	0,92
1058	23	2	Banjaranyar	<200	Jati	Tectona grandis L.f.	Lamiaceae / Verbenaceae	0,7	5,1	2	5	0,04	0,00	6,96	0,01	0,08	0,30
1059	23	2	Banjaranyar	<200	Kelapa	Cocos nucifera L.	Arecaceae	0,5	24,2	10	13	0,04	0,05	104,60	0,10	1,23	4,51
1060	23	2	Banjaranyar	<200	Mahoni	Swietenia macrophylla King	Meliaceae	0,61	11,8	4	10	0,04	0,01	53,76	0,05	0,63	2,32
1061	23	2	Banjaranyar	<200	Jati	Tectona grandis L.f.	Lamiaceae / Verbenaceae	0,7	3,8	3	4	0,04	0,00	3,35	0,00	0,04	0,14
1062	23	2	Banjaranyar	<200	Jati	Tectona grandis L.f.	Lamiaceae / Verbenaceae	0,7	10,8	4	7	0,04	0,01	37,67	0,04	0,44	1,62
1063	23	2	Banjaranyar	<200	Jati	Tectona grandis L.f.	Lamiaceae / Verbenaceae	0,7	6,4	3,5	5,5	0,04	0,00	11,43	0,01	0,13	0,49
1064	23	2	Banjaranyar	<200	Jati	Tectona grandis L.f.	Lamiaceae / Verbenaceae	0,7	7,3	2	6	0,04	0,00	15,98	0,02	0,19	0,69
1065	23	2	Banjaranyar	<200	Sengon	Paraserianthes falcataria (L.) I.C.	Leguminosae / Fabaceae	0,33	3,5	4	5	0,04	0,00	1,76	0,00	0,02	0,08
1066	23	2	Banjaranyar	<200	Mahoni	Swietenia macrophylla King	Meliaceae	0,61	4,5	3	4	0,04	0,00	3,91	0,00	0,05	0,17
1067	23	2	Banjaranyar	<200	Jati	Tectona grandis L.f.	Lamiaceae / Verbenaceae	0,7	10,8	4	7	0,04	0,01	37,67	0,04	0,44	1,62
1068	23	2	Banjaranyar	<200	Sengon	Paraserianthes falcataria (L.) I.C.	Leguminosae / Fabaceae	0,33	9,5	7	9	0,04	0,01	18,93	0,02	0,22	0,82
1069	23	2	Banjaranyar	<200	Kelapa	Cocos nucifera L.	Arecaceae	0,5	31,8	13	16	0,04	0,08	127,70	0,13	1,50	5,51
1070	23	2	Banjaranyar	<200	Tisuk	Hibiscus macrophyllus Roxb. ex	Malvaceae	0,4	5,1	5	6,5	0,04	0,00	5,30	0,01	0,06	0,23
1071	23	2	Banjaranyar	<200	Jati	Tectona grandis L.f.	Lamiaceae / Verbenaceae	0,7	5,7	2,5	6	0,04	0,00	10,20	0,01	0,12	0,44
1072	23	2	Banjaranyar	<200	Sengon	Paraserianthes falcataria (L.) I.C.	Leguminosae / Fabaceae	0,33	14,3	9	12	0,04	0,02	51,80	0,05	0,61	2,23
1073	23	2	Banjaranyar	<200	Gmelina	Gmelina arborea Roxb.	Lamiaceae / Verbenaceae	0,47	24,8	9	13	0,04	0,05	211,08	0,21	2,48	9,10
1074	23	2	Banjaranyar	<200	Sengon	Paraserianthes falcataria (L.) I.C.	Leguminosae / Fabaceae	0,33	17,2	10	13	0,04	0,02	77,84	0,08	0,91	3,36
1075	23	2	Banjaranyar	<200	Mahoni	Swietenia macrophylla King	Meliaceae	0,61	4,5	3	3,5	0,04	0,00	3,46	0,00	0,04	0,15
1076	23	2	Banjaranyar	<200	Tisuk	Hibiscus macrophyllus Roxb. ex	Malvaceae	0,4	13,0	7	11	0,04	0,01	48,10	0,05	0,57	2,07
1077	23	2	Banjaranyar	<200	Gmelina	Gmelina arborea Roxb.	Lamiaceae / Verbenaceae	0,47	17,2	7	11	0,04	0,02	92,35	0,09	1,09	3,98
1078	23	2	Banjaranyar	<200	Tisuk	Hibiscus macrophyllus Roxb. ex	Malvaceae	0,4	8,0	7	9	0,04	0,00	16,17	0,02	0,19	0,70
1079	23	2	Banjaranyar	<200	Sengon	Paraserianthes falcataria (L.) I.C.	Leguminosae / Fabaceae	0,33	10,5	10	12	0,04	0,01	29,34	0,03	0,34	1,27
1080	23	2	Banjaranyar	<200	Jati	Tectona grandis L.f.	Lamiaceae / Verbenaceae	0,7	11,1	8	11	0,04	0,01	60,10	0,06	0,71	2,59
1081	23	2	Banjaranyar	<200	Sengon	Paraserianthes falcataria (L.) I.C.	Leguminosae / Fabaceae	0,33	18,5	14	16	0,04	0,03	107,31	0,11	1,26	4,63
1082	23	2	Banjaranyar	<200	Jati	Tectona grandis L.f.	Lamiaceae / Verbenaceae	0,7	9,9	3	4	0,04	0,01	19,05	0,02	0,22	0,82
1083	23	2	Banjaranyar	<200	Tisuk	Hibiscus macrophyllus Roxb. ex	Malvaceae	0,4	11,8	6	10	0,04	0,01	36,52	0,04	0,43	1,57
1084	23	2	Banjaranyar	<200	Kelapa	Cocos nucifera L.	Arecaceae	0,5	28,6	13	16	0,04	0,06	127,70	0,13	1,50	5,51
1085	23	2	Banjaranyar	<200	Tisuk	Hibiscus macrophyllus Roxb. ex	Malvaceae	0,4	5,1	5	6	0,04	0,00	4,92	0,00	0,06	0,21
1086	23	2	Banjaranyar	<200	Gmelina	Gmelina arborea Roxb.	Lamiaceae / Verbenaceae	0,47	28,6	9	15	0,04	0,06	312,78	0,31	3,68	13,49
1087	23	2	Banjaranyar	<200	Tisuk	Hibiscus macrophyllus Roxb. ex	Malvaceae	0,4	8,0	5	7	0,04	0,00	12,85	0,01	0,15	0,55
1088	23	2	Banjaranyar	<200	Gmelina	Gmelina arborea Roxb.	Lamiaceae / Verbenaceae	0,47	17,2	11	13	0,04	0,02	107,62	0,11	1,26	4,64
1089	23	2	Banjaranyar	<200	Mahoni	Swietenia macrophylla King	Meliaceae	0,61	3,2	3	4	0,04	0,00	2,11	0,00	0,02	0,09
1090	23	2	Banjaranyar	<200	Pulai	Alstonia scholaris (L.) R. Br.	Apocynaceae	0,3	13,4	6	10	0,04	0,01	35,40	0,04	0,42	1,53
1091	23	2	Banjaranyar	<200	Gmelina	Gmelina arborea Roxb.	Lamiaceae / Verbenaceae	0,47	17,2	9	15	0,04	0,02	122,69	0,12	1,44	5,29
1092	23	2	Banjaranyar	<200	Sengon	Paraserianthes falcataria (L.) I.C.	Leguminosae / Fabaceae	0,33	7,6	9	10	0,04	0,00	13,86	0,01	0,16	0,60
1093	23	2	Banjaranyar	<200	Jati	Tectona grandis L.f.	Lamiaceae / Verbenaceae	0,7	7,0	3	7	0,04	0,00	16,97	0,02	0,20	0,73
1094	23	2	Banjaranyar	<200	Tisuk	Hibiscus macrophyllus Roxb. ex	Malvaceae	0,4	10,5	8	11	0,04	0,01	32,32	0,03	0,38	1,39
1095	23	2	Banjaranyar	<200	Jati	Tectona grandis L.f.	Lamiaceae / Verbenaceae	0,7	7,3	3	6	0,04	0,00	15,98	0,02	0,19	0,69
1096	23	2	Banjaranyar	<200	Jati	Tectona grandis L.f.	Lamiaceae / Verbenaceae	0,7	5,7	3	6	0,04	0,00	10,20	0,01	0,12	0,44
1097	23	2	Banjaranyar	<200	Sengon	Paraserianthes falcataria (L.) I.C.	Leguminosae / Fabaceae	0,33	12,4	10	12	0,04	0,01	39,85	0,04	0,47	1,72
1098	23	2	Banjaranyar	<200	Sengon	Paraserianthes falcataria (L.) I.C.	Leguminosae / Fabaceae	0,33	8,6	6	7	0,04	0,01	12,40	0,01	0,15	0,53
1099	23	2	Banjaranyar	<200	Jati	Tectona grandis L.f.	Lamiaceae / Verbenaceae	0,7	6,4	3	7	0,04	0,00	14,25	0,01	0,17	0,61
1100	23	2	Banjaranyar	<200	Sengon	Paraserianthes falcataria (L.) I.C.	Leguminosae / Fabaceae	0,33	10,2	9	11	0,04	0,01	25,61	0,03	0,30	1,10
1101	23	2	Banjaranyar	<200	Jati	Tectona grandis L.f.	Lamiaceae / Verbenaceae	0,7	4,1	3	5	0,04	0,00	4,76	0,00	0,06	0,21
1102	23	2	Banjaranyar	<200	Tisuk	Hibiscus macrophyllus Roxb. ex	Malvaceae	0,4	4,5	5	6,5	0,04	0,00	4,15	0,00	0,05	0,18
1103	23	2	Banjaranyar	<200	Sengon	Paraserianthes falcataria (L.) I.C.	Leguminosae / Fabaceae	0,33	12,7	12	15	0,04	0,01	51,21	0,05	0,60	2,21
1104	23	2	Banjaranyar	<200	Kelapa	Cocos nucifera L.	Arecaceae	0,5	30,2	14	17	0,04	0,07	135,40	0,14	1,59	5,84
1105	23	2	Banjaranyar	<200	Jati	Tectona grandis L.f.	Lamiaceae / Verbenaceae	0,7	7,3	3	7	0,04	0,00	18,41	0,02	0,22	0,79
1106	23	2	Banjaranyar	<200	Sengon	Paraserianthes falcataria (L.) I.C.	Leguminosae / Fabaceae	0,33	15,9	10	12	0,04	0,02	62,82	0,06	0,74	2,71
1107	23	2	Banjaranyar	<200	Jati	Tectona grandis L.f.	Lamiaceae / Verbenaceae	0,7	8,0	4	8	0,04	0,00	24,24	0,02	0,28	1,05
1108	23	2	Banjaranyar	<200	Gmelina	Gmelina arborea Roxb.	Lamiaceae / Verbenaceae	0,47	26,4	11	14	0,04	0,05	253,14	0,25	2,97	10,92
1109	23	2	Banjaranyar	<200	Jati	Tectona grandis L.f.	Lamiaceae / Verbenaceae	0,7	3,8	4	5	0,04	0,00	4,11	0,00	0,05	0,18
1110	23	2	Banjaranyar	<200	Jati	Tectona grandis L.f.	Lamiaceae / Verbenaceae	0,7	4,5	3	5	0,04	0,00	5,45	0,01	0,06	0,23

1111	23	2	Banjaranyar	<200	Jati	Tectona grandis L.f.	Lamiaceae / Verbenaceae	0,7	5,7	3	5	0,04	0,00	8,63	0,01	0,10	0,37
1112	23	2	Banjaranyar	<200	Sengon	Paraserianthes falcataria (L.) I.C	Leguminosae / Fabaceae	0,33	7,6	7	10	0,04	0,00	13,86	0,01	0,16	0,60
1113	23	2	Banjaranyar	<200	Jati	Tectona grandis L.f.	Lamiaceae / Verbenaceae	0,7	5,4	3	6	0,04	0,00	9,19	0,01	0,11	0,40
1114	23	2	Banjaranyar	<200	Sengon	Paraserianthes falcataria (L.) I.C	Leguminosae / Fabaceae	0,33	6,7	6	8	0,04	0,00	8,84	0,01	0,10	0,38
1115	23	2	Banjaranyar	<200	Jati	Tectona grandis L.f.	Lamiaceae / Verbenaceae	0,7	7,0	2,5	6,5	0,04	0,00	15,86	0,02	0,19	0,68
1116	23	2	Banjaranyar	<200	Sengon	Paraserianthes falcataria (L.) I.C	Leguminosae / Fabaceae	0,33	9,9	12	16	0,04	0,01	34,06	0,03	0,40	1,47
1117	23	2	Banjaranyar	<200	Tisuk	Hibiscus macrophyllus Roxb. ex I	Malvaceae	0,4	4,5	5	6	0,04	0,00	3,86	0,00	0,05	0,17
1118	23	2	Banjaranyar	<200	Sengon	Paraserianthes falcataria (L.) I.C	Leguminosae / Fabaceae	0,33	17,8	10	12	0,04	0,02	77,32	0,08	0,91	3,33
1119	23	2	Banjaranyar	<200	Jati	Tectona grandis L.f.	Lamiaceae / Verbenaceae	0,7	7,6	4	6	0,04	0,00	17,28	0,02	0,20	0,75
1120	23	2	Banjaranyar	<200	Sengon	Paraserianthes falcataria (L.) I.C	Leguminosae / Fabaceae	0,33	11,5	9	12	0,04	0,01	34,42	0,03	0,40	1,48
1121	23	2	Banjaranyar	<200	Jati	Tectona grandis L.f.	Lamiaceae / Verbenaceae	0,7	4,1	4	5	0,04	0,00	4,76	0,00	0,06	0,21
1122	23	2	Banjaranyar	<200	Gmelina	Gmelina arborea Roxb.	Lamiaceae / Verbenaceae	0,47	28,6	12	15	0,04	0,06	312,78	0,31	3,68	13,49
1123	23	2	Banjaranyar	<200	Jati	Tectona grandis L.f.	Lamiaceae / Verbenaceae	0,7	6,4	3	7	0,04	0,00	14,25	0,01	0,17	0,61
1124	23	2	Banjaranyar	<200	Jati	Tectona grandis L.f.	Lamiaceae / Verbenaceae	0,7	5,4	2,5	3	0,04	0,00	4,87	0,00	0,06	0,21
1125	24	3	Banjaranyar	<200	Sengon	Paraserianthes falcataria (L.) I.C	Leguminosae / Fabaceae	0,33	27,0	6	16	0,04	0,06	216,15	0,22	2,54	9,32
1126	24	3	Banjaranyar	<200	Kakao	Theobroma cacao L.	Malvaceae	0,36	4,8	2	3	0,04	0,00	2,67	0,00	0,03	0,12
1127	24	3	Banjaranyar	<200	Kelapa	Cocos nucifera L.	Arecaceae	0,5	27,0	9	11	0,04	0,06	89,20	0,09	1,05	3,85
1128	24	3	Banjaranyar	<200	Kelapa	Cocos nucifera L.	Arecaceae	0,5	22,9	6	9	0,04	0,04	73,80	0,07	0,87	3,18
1129	24	3	Banjaranyar	<200	Sengon	Paraserianthes falcataria (L.) I.C	Leguminosae / Fabaceae	0,33	15,0	10	15	0,04	0,02	68,81	0,07	0,81	2,97
1130	24	3	Banjaranyar	<200	Sengon	Paraserianthes falcataria (L.) I.C	Leguminosae / Fabaceae	0,33	10,8	10	13	0,04	0,01	33,35	0,03	0,39	1,44
1131	24	3	Banjaranyar	<200	Sengon	Paraserianthes falcataria (L.) I.C	Leguminosae / Fabaceae	0,33	14,6	10	14	0,04	0,02	62,10	0,06	0,73	2,68
1132	24	3	Banjaranyar	<200	Kelapa	Cocos nucifera L.	Arecaceae	0,5	21,3	7	10	0,04	0,04	81,50	0,08	0,96	3,51
1133	24	3	Banjaranyar	<200	Sengon	Paraserianthes falcataria (L.) I.C	Leguminosae / Fabaceae	0,33	21,6	13	19	0,04	0,04	168,10	0,17	1,98	7,25
1134	24	3	Banjaranyar	<200	Sengon	Paraserianthes falcataria (L.) I.C	Leguminosae / Fabaceae	0,33	18,5	8	15	0,04	0,03	101,15	0,10	1,19	4,36
1135	24	3	Banjaranyar	<200	Sengon	Paraserianthes falcataria (L.) I.C	Leguminosae / Fabaceae	0,33	12,7	10	13	0,04	0,01	44,92	0,04	0,53	1,94
1136	24	3	Banjaranyar	<200	Sengon	Paraserianthes falcataria (L.) I.C	Leguminosae / Fabaceae	0,33	15,9	9	15	0,04	0,02	77,07	0,08	0,91	3,32
1137	24	3	Banjaranyar	<200	Durian	Durio zibethinus L.	Malvaceae	0,57	14,3	2	8	0,04	0,02	58,94	0,06	0,69	2,54
1138	24	3	Banjaranyar	<200	Sengon	Paraserianthes falcataria (L.) I.C	Leguminosae / Fabaceae	0,33	25,5	10	7	0,04	0,05	90,71	0,09	1,07	3,91
1139	24	3	Banjaranyar	<200	Kakao	Theobroma cacao L.	Malvaceae	0,36	5,4	1	2,5	0,04	0,00	3,42	0,00	0,04	0,15
1140	24	3	Banjaranyar	<200	Kakao	Theobroma cacao L.	Malvaceae	0,36	12,7	1	6	0,04	0,01	18,60	0,02	0,22	0,80
1141	24	3	Banjaranyar	<200	Kelapa	Cocos nucifera L.	Arecaceae	0,5	28,6	10	13	0,04	0,06	104,60	0,10	1,23	4,51
1142	24	3	Banjaranyar	<200	Sengon	Paraserianthes falcataria (L.) I.C	Leguminosae / Fabaceae	0,33	13,7	8	13	0,04	0,01	51,28	0,05	0,60	2,21
1143	24	3	Banjaranyar	<200	Kelapa	Cocos nucifera L.	Arecaceae	0,5	23,2	9	12	0,04	0,04	96,90	0,10	1,14	4,18
1144	24	3	Banjaranyar	<200	Kelapa	Cocos nucifera L.	Arecaceae	0,5	24,2	3	7	0,04	0,05	58,40	0,06	0,69	2,52
1145	24	3	Banjaranyar	<200	Kelapa	Cocos nucifera L.	Arecaceae	0,5	22,6	16	20	0,04	0,04	158,50	0,16	1,86	6,83
1146	24	3	Banjaranyar	<200	Kelapa	Cocos nucifera L.	Arecaceae	0,5	21,6	9	13	0,04	0,04	104,60	0,10	1,23	4,51
1147	24	3	Banjaranyar	<200	Sengon	Paraserianthes falcataria (L.) I.C	Leguminosae / Fabaceae	0,33	14,6	10	14	0,04	0,02	62,10	0,06	0,73	2,68
1148	24	3	Banjaranyar	<200	Sengon	Paraserianthes falcataria (L.) I.C	Leguminosae / Fabaceae	0,33	26,7	13	18	0,04	0,06	235,61	0,24	2,77	10,16
1149	25	2	Banjaranyar	<200	Kelapa	Cocos nucifera L.	Arecaceae	0,5	19,4	5	8	0,04	0,03	66,10	0,07	0,78	2,85
1150	25	2	Banjaranyar	<200	Nangka	Artocarpus heterophyllus Lam.	Moraceae	0,61	16,5	4	9	0,04	0,02	91,05	0,09	1,07	3,93
1151	25	2	Banjaranyar	<200	Mahoni	Swietenia macrophylla King	Meliaceae	0,61	15,0	6	11	0,04	0,02	90,92	0,09	1,07	3,92
1152	25	2	Banjaranyar	<200	Melinjo	Gnetum gnemon L.	Gnetaceae	0,76	6,4	1	5	0,04	0,00	11,29	0,01	0,13	0,49
1153	25	2	Banjaranyar	<200	Mangga	Mangifera indica L.	Anacardiaceae	0,6	10,2	5	7	0,04	0,01	29,27	0,03	0,34	1,26
1154	25	2	Banjaranyar	<200	Manggis	Mangostana garcinia Gaertn.	Clusiaceae	0,81	9,5	1,5	5	0,04	0,01	25,16	0,03	0,30	1,08
1155	25	2	Banjaranyar	<200	Jati	Tectona grandis L.f.	Lamiaceae / Verbenaceae	0,7	3,2	2	3	0,04	0,00	1,84	0,00	0,02	0,08
1156	25	2	Banjaranyar	<200	Pala	Myristica fragrans Houtt.	Myristicaceae	0,5	6,4	1	4	0,04	0,00	6,27	0,01	0,07	0,27
1157	25	2	Banjaranyar	<200	Sawo / sawo manila	Manilkara zapota (L.) P.Royen	Sapotaceae	0,81	25,5	5	11	0,04	0,05	312,37	0,31	3,67	13,47
1158	25	2	Banjaranyar	<200	Sengon	Paraserianthes falcataria (L.) I.C	Leguminosae / Fabaceae	0,33	23,9	11	17	0,04	0,04	181,67	0,18	2,13	7,83
1159	25	2	Banjaranyar	<200	Tisuk	Hibiscus macrophyllus Roxb. ex I	Malvaceae	0,4	4,5	4	6	0,04	0,00	3,86	0,00	0,05	0,17
1160	25	2	Banjaranyar	<200	Tisuk	Hibiscus macrophyllus Roxb. ex I	Malvaceae	0,4	12,7	6	9	0,04	0,01	38,25	0,04	0,45	1,65
1161	25	2	Banjaranyar	<200	Pala	Myristica fragrans Houtt.	Myristicaceae	0,5	5,4	1	4	0,04	0,00	4,66	0,00	0,05	0,20
1162	25	2	Banjaranyar	<200	Tisuk	Hibiscus macrophyllus Roxb. ex I	Malvaceae	0,4	4,1	3	4,5	0,04	0,00	2,59	0,00	0,03	0,11
1163	25	2	Banjaranyar	<200	Sawo / sawo manila	Manilkara zapota (L.) P.Royen	Sapotaceae	0,81	23,9	4	9	0,04	0,04	230,94	0,23	2,71	9,96
1164	25	2	Banjaranyar	<200	Sengon	Paraserianthes falcataria (L.) I.C	Leguminosae / Fabaceae	0,33	34,0	12	19	0,04	0,09	385,70	0,39	4,53	16,63
1165	25	2	Banjaranyar	<200	Tisuk	Hibiscus macrophyllus Roxb. ex I	Malvaceae	0,4	4,1	2	3,5	0,04	0,00	2,05	0,00	0,02	0,09
1166	25	2	Banjaranyar	<200	Jeruk ragi	Citrus microcarpa	Rutaceae	0,7	5,7	2	5	0,04	0,00	8,63	0,01	0,10	0,37
1167	25	2	Banjaranyar	<200	Tisuk	Hibiscus macrophyllus Roxb. ex I	Malvaceae	0,4	29,3	10	15	0,04	0,07	280,91	0,28	3,30	12,11
1168	25	2	Banjaranyar	<200	Heuras	Vitex pubescens Vahl	Lamiaceae / Verbenaceae	0,88	4,8	5	6	0,04	0,00	9,01	0,01	0,11	0,39
1169	25	2	Banjaranyar	<200	Jati	Tectona grandis L.f.	Lamiaceae / Verbenaceae	0,7	11,5	3	8	0,04	0,01	47,27	0,05	0,56	2,04
1170	25	2	Banjaranyar	<200	Ketapang	Terminalia catappa L.	Combretaceae	0,65	3,2	2	2,5	0,04	0,00	1,46	0,00	0,02	0,06
1171	25	2	Banjaranyar	<200	Sawo / sawo manila	Manilkara zapota (L.) P.Royen	Sapotaceae	0,81	21,6	3,5	8,5	0,04	0,04	183,15	0,18	2,15	7,90
1172	25	2	Banjaranyar	<200	Tisuk	Hibiscus macrophyllus Roxb. ex I	Malvaceae	0,4	8,6	5	8	0,04	0,01	16,71	0,02	0,20	0,72
1173	25	2	Banjaranyar	<200	Tisuk	Hibiscus macrophyllus Roxb. ex I	Malvaceae	0,4	3,5	4	5	0,04	0,00	2,10	0,00	0,02	0,09
1174	25	2	Banjaranyar	<200	Jati	Tectona grandis L.f.	Lamiaceae / Verbenaceae	0,7	3,8	3	4	0,04	0,00	3,35	0,00	0,04	0,14
1175	25	2	Banjaranyar	<200	Pala	Myristica fragrans Houtt.	Myristicaceae	0,5	9,5	1	5	0,04	0,01	16,17	0,02	0,19	0,70
1176	25	2	Banjaranyar	<200	Kelapa	Cocos nucifera L.	Arecaceae	0,5	23,2	2	5	0,04	0,04	43,00	0,04	0,51	1,85
1177	25	2	Banjaranyar	<200	Nangka	Artocarpus heterophyllus Lam.	Moraceae	0,61	5,4	2,5	5	0,04	0,00	6,85	0,01	0,08	0,30
1178	25	2	Banjaranyar	<200	Jati	Tectona grandis L.f.	Lamiaceae / Verbenaceae	0,7	29,3	8	11	0,04	0,07	353,02	0,35	4,15	15,22
1179	25	2	Banjaranyar	<200	Sengon	Paraserianthes falcataria (L.) I.C	Leguminosae / Fabaceae	0,33	32,8	10	16	0,04	0,08	307,31	0,31	3,61	13,25
1180	25	2	Banjaranyar	<200	Pala	Myristica fragrans Houtt.	Myristicaceae	0,5	5,7	0,5	4	0,04	0,00	5,17	0,01	0,06	0,22
1181	25	2	Banjaranyar	<200	Rambutan	Nephelium lappaceum L.	Sapindaceae	0,71	5,4	2	4	0,04	0,00	6,42	0,01	0,08	0,28
1182	25	2	Banjaranyar	<200	Heuras	Vitex pubescens Vahl	Lamiaceae / Verbenaceae	0,88	3,8	3	4	0,04	0,00	4,13	0,00	0,05	0,18
1183	25	2	Banjaranyar	<200	Heuras	Vitex pubescens Vahl	Lamiaceae / Verbenaceae	0,88	5,7	4	7	0,04	0,00	14,49	0,01	0,17	0,62
1184	25	2	Banjaranyar	<200	Durian	Durio zibethinus L.	Malvaceae	0,57	8,3	2	6	0,04	0,01	16,58	0,02	0,19	0,71
1185	25	2	Banjaranyar	<200	Mangga	Mangifera indica L.	Anacardiaceae	0,6	6,4	3	5	0,04	0,00	9,09	0,01	0,11	0,39
1186	25	2	Banjaranyar	<200	Binong	Tetrameles nudiflora R. Br.	Tetramelaceae	0,3	13,0	4	6	0,04	0,01	21,21	0,02	0,25	0,91
1187	25	2	Banjaranyar	<200	Tisuk	Hibiscus macrophyllus Roxb. ex I	Malvaceae	0,4	4,1	2,5	3,5	0,04	0,00	2,05	0,00	0,02	0,09
1188	25	2	Banjaranyar	<200	Wangkal	Albizia procera Benth	Leguminosae / Fabaceae	0,77	7,6	4	7	0,04	0,00	21,72	0,02	0,26	0,94
1189	25	2	Banjaranyar	<200	Tisuk	Hibiscus macrophyllus Roxb. ex I	Malvaceae	0,4	13,7	6	9	0,04	0,01	43,67	0,04	0,51	1,88
1190	25	2	Banjaranyar	<200	Cengkeh	Syzygium aromaticum (L.) Merr. &	Myrtaceae	0,79	5,7	2	4	0,04	0,00	7,86	0,01	0,09	0,34
1191	25	2	Banjaranyar	<200	Durian	Durio zibethinus L.	Malvaceae	0,57	16,9	2	6	0,04	0,02	61,12	0,06	0,72	2,64
1192	26	1	Banjaranyar	<200	Sengon	Paraserianthes falcataria (L.) I.C	Leguminosae / Fabaceae	0,33	19,1	14	17	0,04	0,03	120,71	0,12	1,42	5,21
1193	26	1	Banjaranyar	<200	mahoni	Swietenia macrophylla King	Meliaceae	0,61	6,7	3	4	0,04	0,00	8,23	0,01	0,10	0,35
1194	26	1	Banjaranyar	<200	mahoni	Swietenia macrophylla King	Meliaceae	0,61	19,1	9	14	0,04	0,03	177,38	0,18	2,08	7,65
1195	26	1	Banjaranyar	<200	tisuk	Hibiscus macrophyllus Roxb. ex I	Malvaceae	0,4	5,4	4	7	0,04	0,00	6,34	0,01	0,07	0,27
1196	26	1	Banjaranyar	<200	Sengon	Paraserianthes falcataria (L.) I.C	Leguminosae / Fabaceae	0,33	43,9	12	21	0,04	0,15	673,74	0,67	7,92	29,05
1197	26	1	Banjaranyar	<200	Sengon	Paraserianthes falcataria (L.) I.C	Leguminosae / Fabaceae	0,33	18,5	10	15	0,04	0,03	101,15	0,10	1,19	4,36
1198	26	1	Banjaranyar	<200	tisuk	Hibiscus macrophyllus Roxb. ex I	Malvaceae	0,4	5,1	5	8	0,04	0,00	6,41	0,01	0,08	0,28
1199	26	1	Banjaranyar	<200	Sengon	Paraserianthes falcataria (L.) I.C	Leguminosae / Fabaceae	0,33	42,0	14	23	0,04	0,14	675,01	0,68	7,93	29,11
1200	26	1	Banjaranyar	<200	tisuk	Hibiscus macrophyllus Roxb. ex I	Malvaceae	0,4	11,8	11	14	0,04	0,01	49,71	0,05	0,58	2,14
1201	26	1	Banjaranyar	<200	Kelapa	Cocos nucifera L.	Arecaceae	0,5	24,8	12	15	0,04	0,05	120,00	0,12	1,41	5,17
1202	26	1	Banjaranyar	<200	tisuk	Hibiscus macrophyllus Roxb. ex I	Malvaceae	0,4	24,2	13	17	0,04	0,05	222,00	0,22	2,61	9,57
1203	26	1	Banjaranyar	<200	Rambutan	Nephelium lappaceum L.	Sapindaceae	0,71	12,4	6	11	0,04	0,01	74,24	0,07	0,87	3,20
1204	26	1	Banjaranyar	<200	Sengon	Paraserianthes falcataria (L.) I.C	Leguminosae / Fabaceae	0,33	13,0	10	13	0,04	0,01	47,00	0,05	0,55	2,03
1205	26	1	Banjaranyar	<200	mahoni	Swietenia macrophylla King	Meliaceae	0,61	13,7	4	8	0,04	0,01	57,71	0,06	0,68	2,49
1206	26	1	Banjaranyar	<200	mahoni	Swietenia macrophylla King	Meliaceae	0,61	10,2	3	6	0,04	0,01	25,80	0,03	0,30	1,11
1207	26	1	Banjaranyar	<200	Kokosan	Lansium domesticum var. aquae	Meliaceae	0,7	36,9	6	10	0,04	0,11	494,67	0,49	5,81	21,33
1208	26	1	Banjaranyar	<200	Kelapa	Cocos nucifera L.	Arecaceae	0,5	32,8	12	15	0,04	0,08	120,00	0,12	1,41	5,17
1209	26	1	Banjaranyar	<200	mahoni	Swietenia macrophylla King	Meliaceae	0,61	20,7	4	10	0,04	0,03	150,92	0,15	1,77	6,51
1210	26	1	Banjaranyar	<200	Sengon	Paraserianthes falcataria (L.) I.C	Leguminosae / Fabaceae	0,33	41,4	12	18	0,04	0,13	524,39	0,52	6,16	22,61
1211	26	1	Banjaranyar	<200	Sengon	Paraserianthes falcataria (L.) I.C	Leguminosae / Fabaceae	0,33	24,8	11	14	0,04	0,05	163,40	0,16	1,92	7,05
1212	26	1	Banjaranyar	<200	mahoni	Swietenia macrophylla King	Meliaceae	0,61	10,5	4	9	0,04	0,01	39,58	0,04	0,47	1,71
1213	26	1	Banjaranyar	<200	tisuk	Hibiscus macrophyllus Roxb. ex I	Malvaceae	0,4	11,5	9	14	0,04	0,01	47,27	0,05	0,56	2,04
1214	26	1	Banjaranyar	<200	Sengon	Paraserianthes falcataria (L.) I.C	Leguminosae / Fabaceae	0,33	28,0	15	18	0,04	0,06	256,57	0,26	3,01	11,06
1215	26	1	Banjaranyar	<200	Sengon	Paraserianthes falcataria (L.) I.C	Leguminosae / Fabaceae	0,33	9,2	5	10	0,04	0,01	19,60	0,02	0,23	0,85
1216	26	1	Banjaranyar	<200	Sengon	Paraserianthes falcataria (L.) I.C	Leguminosae / Fabaceae	0,33	42,6	12	20	0,04	0,14	610,49	0,61	7,17	26,33
1217	26	1	Banjaranyar	<200	mahoni	Swietenia macrophylla King	Meliaceae	0,61	16,2	9	15	0,04	0,02	140,30	0,14	1,65	6,05
1218	26	1	Banjaranyar	<200	tisuk	Hibiscus macrophyllus Roxb. ex I	Malvaceae	0,4	10,2	9	12	0,04	0,01	33,08	0,03	0,39	1,43
1219	26	1	Banjaranyar	<200	Kelapa	Cocos nucifera L.	Arecaceae	0,5	4,1	3	3,5	0,04	0,00	31,45	0,03	0,37	1,36
1220	26	1	Banjaranyar	<200	Kelapa	Cocos nucifera L.	Arecaceae	0,5	40,7	12	18	0,04	0,13	143,10	0,14	1,68	6,17
1221	26	1	Banjaranyar	<200	tisuk	Hibiscus macrophyllus Roxb. ex I	Malvaceae	0,4	5,1	3	6	0,04	0,00	4,92	0,00	0,06	0,21
1222	26	1	Banjaranyar	<200	Kelapa	Cocos nucifera L.	Arecaceae	0,5	27,4	7	10	0,04	0,06	81,50	0,08	0,96	3,51
1223	26	1	Banjaranyar	<200	Kelapa	Cocos nucifera L.	Arecaceae	0,5	30,5	12	15	0,04	0,07	120,00	0,12	1,41	5,17
1224	26	1	Banjaranyar	<200	mahoni	Swietenia macrophylla King	Meliaceae	0,61	21,6	4	10	0,04	0,04	163,92	0,16	1,93	7,07
1225	26	1	Banjaranyar	<200	Sengon	Paraserianthes falcataria (L.) I.C	Leguminosae / Fabaceae	0,33	9,2	9	11	0,04	0,01	21,38	0,02	0,25	0,92
1226	26	1	Banjaranyar	<200	Kelapa	Cocos nucifera L.	Arecaceae	0,5	22,6	3	7	0,04	0,04	58,40	0,06	0,69	2,52
1227	26	1	Banjaranyar	<200	Janglot	Miliusa horsfieldii (Benn.) Baill. e	Annonaceae	0,61	6,4	1,8	4	0,04	0,00	7,52	0,01	0,09	0,32
1228	26	1	Banjaranyar	<200	Janglot	Miliusa horsfieldii (Benn.) Baill. e	Annonaceae	0,61	3,2	1,5	3	0,04	0,00	1,62	0,00	0,02	0,07
1229	26	1	Banjaranyar	<200	Sengon	Paraserianthes falcataria (L.) I.C	Leguminosae / Fabaceae	0,33	8,6	10	13	0,04	0,01	21,86	0,02	0,26	0,94
1230	26	1	Banjaranyar	<200	Sengon	Paraserianthes falcataria (L.) I.C	Leguminosae / Fabaceae	0,33	37,5	12	19	0,04	0,11	461,43	0,46	5,42	19,90
1231	26	1	Banjaranyar	<200	Sengon	Paraserianthes falcataria (L.) I.C	Leguminosae / Fabaceae	0,33	30,2	10	18	0,04	0,07	295,19	0,30	3,47	12,73
1232	26	1	Banjaranyar	<200	Janglot	Miliusa horsfieldii (Benn.) Baill. e	Annonaceae	0,61	5,1	1	3	0,04	0,00	3,84	0,00	0,05	0,17
1233	26	1	Banjaranyar	<200	Bungur	Lagerstroemia speciosa (L.) Pers	Lythraceae	0,69	4,1	1	2	0,04	0,00	2,03	0,00	0,02	0,09
1234	26	1	Banjaranyar	<200	Kelapa	Cocos nucifera L.	Arecaceae	0,5	27,4	7	10	0,04	0,06	81,50	0,08	0,96	3,51

1235	26	1	Banjaranyar	<200	Janglot	Miliusa horsfieldii (Benn.) Baill. e	Annonaceae	0,61	4,1	18	3	0,04	0,00	2,63	0,00	0,03	0,11
1236	26	1	Banjaranyar	<200	Sengon	Paraserianthes falcataria (L.) I.C.	Leguminosae / Fabaceae	0,33	12,4	11	14	0,04	0,01	45,89	0,05	0,54	1,98
1237	26	1	Banjaranyar	<200	mahoni	Swietenia macrophylla King	Meliaceae	0,61	8,3	3	8	0,04	0,01	22,96	0,02	0,27	0,99
1238	27	3	Banjaranyar	<200	Sengon	Paraserianthes falcataria (L.) I.C.	Leguminosae / Fabaceae	0,33	10,2	4	8	0,04	0,01	19,13	0,02	0,22	0,82
1239	27	3	Banjaranyar	<200	Tisuk	Hibiscus macrophyllus Roxb. ex	Malvaceae	0,4	15,0	9	12	0,04	0,02	66,90	0,07	0,79	2,88
1240	27	3	Banjaranyar	<200	Sengon	Paraserianthes falcataria (L.) I.C.	Leguminosae / Fabaceae	0,33	3,5	4	6	0,04	0,00	2,08	0,00	0,02	0,09
1241	27	3	Banjaranyar	<200	Heuras	Vitex pubescens Vahl	Lamiaceae / Verbenaceae	0,88	17,8	4	8	0,04	0,02	130,97	0,13	1,54	5,65
1242	27	3	Banjaranyar	<200	Tisuk	Hibiscus macrophyllus Roxb. ex	Malvaceae	0,4	11,8	8	12	0,04	0,01	43,16	0,04	0,51	1,86
1243	27	3	Banjaranyar	<200	Tisuk	Hibiscus macrophyllus Roxb. ex	Malvaceae	0,4	7,3	4	6	0,04	0,00	9,57	0,01	0,11	0,41
1244	27	3	Banjaranyar	<200	Heuras	Vitex pubescens Vahl	Lamiaceae / Verbenaceae	0,88	16,5	5	9	0,04	0,02	127,37	0,13	1,50	5,49
1245	27	3	Banjaranyar	<200	Heuras	Vitex pubescens Vahl	Lamiaceae / Verbenaceae	0,88	8,9	5	9	0,04	0,01	40,98	0,04	0,48	1,77
1246	27	3	Banjaranyar	<200	Tisuk	Hibiscus macrophyllus Roxb. ex	Malvaceae	0,4	7,0	4	3	0,04	0,00	4,68	0,00	0,05	0,20
1247	27	3	Banjaranyar	<200	Tisuk	Hibiscus macrophyllus Roxb. ex	Malvaceae	0,4	10,5	5	9	0,04	0,01	26,89	0,03	0,32	1,16
1248	27	3	Banjaranyar	<200	Sengon	Paraserianthes falcataria (L.) I.C.	Leguminosae / Fabaceae	0,33	3,8	3	6	0,04	0,00	2,44	0,00	0,03	0,11
1249	27	3	Banjaranyar	<200	Sengon	Paraserianthes falcataria (L.) I.C.	Leguminosae / Fabaceae	0,33	8,6	7	9	0,04	0,01	15,61	0,02	0,18	0,67
1250	27	3	Banjaranyar	<200	Tisuk	Hibiscus macrophyllus Roxb. ex	Malvaceae	0,4	11,8	6	10	0,04	0,01	36,52	0,04	0,43	1,57
1251	27	3	Banjaranyar	<200	Sengon	Paraserianthes falcataria (L.) I.C.	Leguminosae / Fabaceae	0,33	20,0	8	11	0,04	0,03	88,59	0,09	1,04	3,82
1252	27	3	Banjaranyar	<200	Sengon	Paraserianthes falcataria (L.) I.C.	Leguminosae / Fabaceae	0,33	4,5	3,5	5,5	0,04	0,00	2,99	0,00	0,04	0,13
1253	27	3	Banjaranyar	<200	Sengon	Paraserianthes falcataria (L.) I.C.	Leguminosae / Fabaceae	0,33	6,7	5	8	0,04	0,00	8,84	0,01	0,10	0,38
1254	27	3	Banjaranyar	<200	Sengon	Paraserianthes falcataria (L.) I.C.	Leguminosae / Fabaceae	0,33	13,7	10	14	0,04	0,01	54,88	0,05	0,64	2,37
1255	27	3	Banjaranyar	<200	Sengon	Paraserianthes falcataria (L.) I.C.	Leguminosae / Fabaceae	0,33	6,0	4	6	0,04	0,00	5,66	0,01	0,07	0,24
1256	27	3	Banjaranyar	<200	Sengon	Paraserianthes falcataria (L.) I.C.	Leguminosae / Fabaceae	0,33	22,0	7	13	0,04	0,04	121,96	0,12	1,43	5,26
1257	27	3	Banjaranyar	<200	Tisuk	Hibiscus macrophyllus Roxb. ex	Malvaceae	0,4	8,6	4	7	0,04	0,01	14,79	0,01	0,17	0,64
1258	27	3	Banjaranyar	<200	Sengon	Paraserianthes falcataria (L.) I.C.	Leguminosae / Fabaceae	0,33	7,6	6	9	0,04	0,00	12,58	0,01	0,15	0,54
1259	27	3	Banjaranyar	<200	Sengon	Paraserianthes falcataria (L.) I.C.	Leguminosae / Fabaceae	0,33	6,0	4	6	0,04	0,00	5,66	0,01	0,07	0,24
1260	27	3	Banjaranyar	<200	Heuras	Vitex pubescens Vahl	Lamiaceae / Verbenaceae	0,88	2,5	1	3,5	0,04	0,00	1,74	0,00	0,02	0,07
1261	27	3	Banjaranyar	<200	Sengon	Paraserianthes falcataria (L.) I.C.	Leguminosae / Fabaceae	0,33	4,1	3	5	0,04	0,00	2,39	0,00	0,03	0,10
1262	27	3	Banjaranyar	<200	Kelapa	Cocos nucifera L.	Arecaceae	0,5	28,6	10	14	0,04	0,06	112,30	0,11	1,32	4,84
1263	27	3	Banjaranyar	<200	Sengon	Paraserianthes falcataria (L.) I.C.	Leguminosae / Fabaceae	0,33	9,2	8	9	0,04	0,01	17,79	0,02	0,21	0,77
1264	27	3	Banjaranyar	<200	Heuras	Vitex pubescens Vahl	Lamiaceae / Verbenaceae	0,88	9,5	4	7	0,04	0,01	36,94	0,04	0,43	1,59
1265	27	3	Banjaranyar	<200	Sengon	Paraserianthes falcataria (L.) I.C.	Leguminosae / Fabaceae	0,33	8,0	8	10	0,04	0,00	14,93	0,01	0,18	0,64
1266	27	3	Banjaranyar	<200	Sengon	Paraserianthes falcataria (L.) I.C.	Leguminosae / Fabaceae	0,33	11,1	7	11	0,04	0,01	30,18	0,03	0,35	1,30
1267	27	3	Banjaranyar	<200	Tisuk	Hibiscus macrophyllus Roxb. ex	Malvaceae	0,4	10,8	5	9	0,04	0,01	28,40	0,03	0,33	1,22
1268	27	3	Banjaranyar	<200	Salam	Syzygium polyanthum (Wight) W.	Myrtaceae	0,66	3,8	1,8	4	0,04	0,00	3,17	0,00	0,04	0,14
1269	27	3	Banjaranyar	<200	Tisuk	Hibiscus macrophyllus Roxb. ex	Malvaceae	0,4	7,3	5	7	0,04	0,00	11,03	0,01	0,13	0,48
1270	27	3	Banjaranyar	<200	Jati	Tectona grandis L.f.	Lamiaceae / Verbenaceae	0,7	6,4	2	4	0,04	0,00	8,53	0,01	0,10	0,37
1271	27	3	Banjaranyar	<200	Jati	Tectona grandis L.f.	Lamiaceae / Verbenaceae	0,7	5,1	2,5	4,5	0,04	0,00	6,32	0,01	0,07	0,27
1272	27	3	Banjaranyar	<200	Sengon	Paraserianthes falcataria (L.) I.C.	Leguminosae / Fabaceae	0,33	11,1	7	9	0,04	0,01	25,11	0,03	0,30	1,08
1273	27	3	Banjaranyar	<200	Tisuk	Hibiscus macrophyllus Roxb. ex	Malvaceae	0,4	8,3	3	7	0,04	0,01	13,80	0,01	0,16	0,60
1274	27	3	Banjaranyar	<200	Sengon	Paraserianthes falcataria (L.) I.C.	Leguminosae / Fabaceae	0,33	10,5	5	7	0,04	0,01	17,91	0,02	0,21	0,77
1275	27	3	Banjaranyar	<200	Tisuk	Hibiscus macrophyllus Roxb. ex	Malvaceae	0,4	8,6	3	7	0,04	0,01	14,79	0,01	0,17	0,64
1276	27	3	Banjaranyar	<200	Sengon	Paraserianthes falcataria (L.) I.C.	Leguminosae / Fabaceae	0,33	8,3	4	8	0,04	0,01	13,08	0,01	0,15	0,56
1277	27	3	Banjaranyar	<200	Sengon	Paraserianthes falcataria (L.) I.C.	Leguminosae / Fabaceae	0,33	12,7	5	9	0,04	0,01	32,07	0,03	0,38	1,38
1278	27	3	Banjaranyar	<200	Jati	Tectona grandis L.f.	Lamiaceae / Verbenaceae	0,7	13,4	4	9	0,04	0,01	69,84	0,07	0,82	3,01
1279	27	3	Banjaranyar	<200	Jati	Tectona grandis L.f.	Lamiaceae / Verbenaceae	0,7	5,4	5	7	0,04	0,00	10,58	0,01	0,12	0,46
1280	27	3	Banjaranyar	<200	Sengon	Paraserianthes falcataria (L.) I.C.	Leguminosae / Fabaceae	0,33	4,5	4	6	0,04	0,00	3,23	0,00	0,04	0,14
1281	27	3	Banjaranyar	<200	Kelapa	Cocos nucifera L.	Arecaceae	0,5	28,6	9	12	0,04	0,06	96,90	0,10	1,14	4,18
1282	27	3	Banjaranyar	<200	Sengon	Paraserianthes falcataria (L.) I.C.	Leguminosae / Fabaceae	0,33	13,4	9	12	0,04	0,01	45,65	0,05	0,54	1,97
1283	27	3	Banjaranyar	<200	Kelapa	Cocos nucifera L.	Arecaceae	0,5	29,6	11	14	0,04	0,07	112,30	0,11	1,32	4,84
1284	27	3	Banjaranyar	<200	Jati	Tectona grandis L.f.	Lamiaceae / Verbenaceae	0,7	11,1	3	7	0,04	0,01	39,73	0,04	0,47	1,71
1285	27	3	Banjaranyar	<200	Sengon	Paraserianthes falcataria (L.) I.C.	Leguminosae / Fabaceae	0,33	8,3	7	8	0,04	0,01	13,08	0,01	0,15	0,56
1286	27	3	Banjaranyar	<200	Jati	Tectona grandis L.f.	Lamiaceae / Verbenaceae	0,7	4,5	2	3	0,04	0,00	3,41	0,00	0,04	0,15
1287	27	3	Banjaranyar	<200	Sengon	Paraserianthes falcataria (L.) I.C.	Leguminosae / Fabaceae	0,33	12,7	8	10	0,04	0,01	35,32	0,04	0,42	1,52
1288	27	3	Banjaranyar	<200	Rambutan	Nephelium lappaceum L.	Sapindaceae	0,71	4,5	2	4	0,04	0,00	4,50	0,00	0,05	0,19
1289	27	3	Banjaranyar	<200	Jati	Tectona grandis L.f.	Lamiaceae / Verbenaceae	0,7	11,1	3	11	0,04	0,01	60,10	0,06	0,71	2,59
1290	27	3	Banjaranyar	<200	Sengon	Paraserianthes falcataria (L.) I.C.	Leguminosae / Fabaceae	0,33	10,5	8	10	0,04	0,01	24,83	0,02	0,29	1,07
1291	27	3	Banjaranyar	<200	Sengon	Paraserianthes falcataria (L.) I.C.	Leguminosae / Fabaceae	0,33	3,2	5	6	0,04	0,00	1,75	0,00	0,02	0,08
1292	27	3	Banjaranyar	<200	Tisuk	Hibiscus macrophyllus Roxb. ex	Malvaceae	0,4	11,1	3,5	10	0,04	0,01	32,99	0,03	0,39	1,42
1293	27	3	Banjaranyar	<200	Sengon	Paraserianthes falcataria (L.) I.C.	Leguminosae / Fabaceae	0,33	10,2	4	5	0,04	0,01	12,44	0,01	0,15	0,54
1294	27	3	Banjaranyar	<200	Sengon	Paraserianthes falcataria (L.) I.C.	Leguminosae / Fabaceae	0,33	10,2	3	11	0,04	0,01	25,61	0,03	0,30	1,10
1295	27	3	Banjaranyar	<200	Sengon	Paraserianthes falcataria (L.) I.C.	Leguminosae / Fabaceae	0,33	5,7	6	8	0,04	0,00	6,67	0,01	0,08	0,29
1296	27	3	Banjaranyar	<200	Tisuk	Hibiscus macrophyllus Roxb. ex	Malvaceae	0,4	8,3	3	8	0,04	0,01	15,60	0,02	0,18	0,67
1297	27	3	Banjaranyar	<200	Jati	Tectona grandis L.f.	Lamiaceae / Verbenaceae	0,7	16,5	3	9	0,04	0,02	103,28	0,10	1,21	4,45
1298	28	2	Banjaranyar	<200	Mahoni	Swietenia macrophylla King	Meliaceae	0,61	16,2	5	9	0,04	0,02	87,87	0,09	1,03	3,79
1299	28	2	Banjaranyar	<200	Jati	Tectona grandis L.f.	Lamiaceae / Verbenaceae	0,7	6,4	2	4	0,04	0,00	8,53	0,01	0,10	0,37
1300	28	2	Banjaranyar	<200	Mahoni	Swietenia macrophylla King	Meliaceae	0,61	20,4	2,5	12,5	0,04	0,03	179,96	0,18	2,11	7,76
1301	28	2	Banjaranyar	<200	Sengon	Paraserianthes falcataria (L.) I.C.	Leguminosae / Fabaceae	0,33	17,5	12	17	0,04	0,02	102,92	0,10	1,21	4,44
1302	28	2	Banjaranyar	<200	Jati	Tectona grandis L.f.	Lamiaceae / Verbenaceae	0,7	18,1	4	11	0,04	0,03	146,86	0,15	1,73	6,33
1303	28	2	Banjaranyar	<200	Jati	Tectona grandis L.f.	Lamiaceae / Verbenaceae	0,7	28,6	3	11	0,04	0,06	339,09	0,34	3,98	14,62
1304	28	2	Banjaranyar	<200	Jati	Tectona grandis L.f.	Lamiaceae / Verbenaceae	0,7	25,1	4	12	0,04	0,05	289,21	0,29	3,40	12,47
1305	28	2	Banjaranyar	<200	Sengon	Paraserianthes falcataria (L.) I.C.	Leguminosae / Fabaceae	0,33	23,2	9	15	0,04	0,04	154,16	0,15	1,81	6,65
1306	28	2	Banjaranyar	<200	Lamtoro	Leucaena leucocephala (Lam.) d	Leguminosae / Fabaceae	0,64	3,2	3	4	0,04	0,00	2,21	0,00	0,03	0,10
1307	28	2	Banjaranyar	<200	Jati	Tectona grandis L.f.	Lamiaceae / Verbenaceae	0,7	6,4	2	4,5	0,04	0,00	9,51	0,01	0,11	0,41
1308	28	2	Banjaranyar	<200	Tisuk	Hibiscus macrophyllus Roxb. ex	Malvaceae	0,4	12,7	6	11	0,04	0,01	45,97	0,05	0,54	1,98
1309	28	2	Banjaranyar	<200	Rambutan	Nephelium lappaceum L.	Sapindaceae	0,71	4,1	2	5	0,04	0,00	4,82	0,00	0,06	0,21
1310	28	2	Banjaranyar	<200	Jati	Tectona grandis L.f.	Lamiaceae / Verbenaceae	0,7	23,9	4	11	0,04	0,04	242,80	0,24	2,85	10,47
1311	28	2	Banjaranyar	<200	Jati	Tectona grandis L.f.	Lamiaceae / Verbenaceae	0,7	22,6	3	10	0,04	0,04	201,25	0,20	2,36	8,68
1312	28	2	Banjaranyar	<200	Sengon	Paraserianthes falcataria (L.) I.C.	Leguminosae / Fabaceae	0,33	17,2	10	15	0,04	0,02	88,74	0,09	1,04	3,83
1313	28	2	Banjaranyar	<200	Kelapa	Cocos nucifera L.	Arecaceae	0,5	30,2	14	18	0,04	0,07	143,10	0,14	1,68	6,17
1314	28	2	Banjaranyar	<200	Sengon	Paraserianthes falcataria (L.) I.C.	Leguminosae / Fabaceae	0,33	19,1	10	15	0,04	0,03	107,63	0,11	1,26	4,64
1315	28	2	Banjaranyar	<200	Jati	Tectona grandis L.f.	Lamiaceae / Verbenaceae	0,7	10,5	3	7	0,04	0,01	35,67	0,04	0,42	1,54
1316	28	2	Banjaranyar	<200	Pangsor	Ficus callosa Willd.	Moraceae	0,33	25,5	10	14	0,04	0,05	171,16	0,17	2,01	7,38
1317	28	2	Banjaranyar	<200	Sengon	Paraserianthes falcataria (L.) I.C.	Leguminosae / Fabaceae	0,33	26,1	12	15	0,04	0,05	190,76	0,19	2,24	8,23
1318	28	2	Banjaranyar	<200	Sengon	Paraserianthes falcataria (L.) I.C.	Leguminosae / Fabaceae	0,33	11,1	11	13	0,04	0,01	35,17	0,04	0,41	1,52
1319	28	2	Banjaranyar	<200	Kelapa	Cocos nucifera L.	Arecaceae	0,5	27,7	2,5	6	0,04	0,06	50,70	0,05	0,60	2,19
1320	28	2	Banjaranyar	<200	Heuras	Vitex pubescens Vahl	Lamiaceae / Verbenaceae	0,88	19,1	3,5	10,5	0,04	0,03	190,65	0,19	2,24	8,22
1321	28	2	Banjaranyar	<200	Sengon	Paraserianthes falcataria (L.) I.C.	Leguminosae / Fabaceae	0,33	9,5	9	11	0,04	0,01	22,76	0,02	0,27	0,98
1322	28	2	Banjaranyar	<200	Sengon	Paraserianthes falcataria (L.) I.C.	Leguminosae / Fabaceae	0,33	21,0	10	13	0,04	0,03	112,42	0,11	1,32	4,85
1323	28	2	Banjaranyar	<200	Jati	Tectona grandis L.f.	Lamiaceae / Verbenaceae	0,7	9,5	3	8	0,04	0,01	33,85	0,03	0,40	1,46
1324	28	2	Banjaranyar	<200	Jati	Tectona grandis L.f.	Lamiaceae / Verbenaceae	0,7	23,5	6	14	0,04	0,04	295,47	0,30	3,47	12,74
1325	28	2	Banjaranyar	<200	Kelapa	Cocos nucifera L.	Arecaceae	0,5	22,3	35	6	0,04	0,04	50,70	0,05	0,60	2,19
1326	28	2	Banjaranyar	<200	Jati	Tectona grandis L.f.	Lamiaceae / Verbenaceae	0,7	4,8	3	4,5	0,04	0,00	5,61	0,01	0,07	0,24
1327	28	2	Banjaranyar	<200	Sengon	Paraserianthes falcataria (L.) I.C.	Leguminosae / Fabaceae	0,33	13,7	12	15	0,04	0,01	58,46	0,06	0,69	2,52
1328	28	2	Banjaranyar	<200	Mahoni	Swietenia macrophylla King	Meliaceae	0,61	7,6	3	7	0,04	0,00	17,54	0,02	0,21	0,76
1329	28	2	Banjaranyar	<200	Tisuk	Hibiscus macrophyllus Roxb. ex	Malvaceae	0,4	22,3	10	14	0,04	0,04	159,84	0,16	1,88	6,89
1330	28	2	Banjaranyar	<200	Jati	Tectona grandis L.f.	Lamiaceae / Verbenaceae	0,7	23,5	4	10	0,04	0,04	217,10	0,22	2,55	9,36
1331	28	2	Banjaranyar	<200	Kelapa	Cocos nucifera L.	Arecaceae	0,5	27,0	4	8	0,04	0,06	66,10	0,07	0,78	2,85
1332	28	2	Banjaranyar	<200	Jati	Tectona grandis L.f.	Lamiaceae / Verbenaceae	0,7	5,7	3	5	0,04	0,00	8,63	0,01	0,10	0,37
1333	28	2	Banjaranyar	<200	Jati	Tectona grandis L.f.	Lamiaceae / Verbenaceae	0,7	4,8	2	3	0,04	0,00	3,87	0,00	0,05	0,17
1334	28	2	Banjaranyar	<200	Mahoni	Swietenia macrophylla King	Meliaceae	0,61	10,2	3	8	0,04	0,01	33,59	0,03	0,39	1,45
1335	28	2	Banjaranyar	<200	Jati	Tectona grandis L.f.	Lamiaceae / Verbenaceae	0,7	12,7	3	9	0,04	0,01	63,87	0,06	0,75	2,75
1336	28	2	Banjaranyar	<200	Sengon	Paraserianthes falcataria (L.) I.C.	Leguminosae / Fabaceae	0,33	13,4	12	15	0,04	0,01	56,00	0,06	0,66	2,41
1337	28	2	Banjaranyar	<200	Jati	Tectona grandis L.f.	Lamiaceae / Verbenaceae	0,7	23,5	4	12	0,04	0,04	256,56	0,26	3,01	11,06
1338	28	2	Banjaranyar	<200	Jati	Tectona grandis L.f.	Lamiaceae / Verbenaceae	0,7	11,1	3	5	0,04	0,01	29,19	0,03	0,34	1,26
1339	28	2	Banjaranyar	<200	Jati	Tectona grandis L.f.	Lamiaceae / Verbenaceae	0,7	7,6	3	5	0,04	0,00	14,62	0,01	0,17	0,63
1340	28	2	Banjaranyar	<200	Tisuk	Hibiscus macrophyllus Roxb. ex	Malvaceae	0,4	17,2	10	13	0,04	0,02	92,84	0,09	1,09	4,00
1341	28	2	Banjaranyar	<200	Sengon	Paraserianthes falcataria (L.) I.C.	Leguminosae / Fabaceae	0,33	17,5	11	15	0,04	0,02	91,77	0,09	1,08	3,96
1342	28	2	Banjaranyar	<200	Jati	Tectona grandis L.f.	Lamiaceae / Verbenaceae	0,7	27,4	8	13	0,04	0,06	363,58	0,36	4,27	15,68
1343	28	2	Banjaranyar	<200	Mahoni	Swietenia macrophylla King	Meliaceae	0,61	6,0	2	5	0,04	0,00	8,40	0,01	0,10	0,36
1344	28	2	Banjaranyar	<200	Jati	Tectona grandis L.f.	Lamiaceae / Verbenaceae	0,7	4,8	2,5	4	0,04	0,00	5,04	0,01	0,06	0,22
1345	28	2	Banjaranyar	<200	Aren	Arenga pinnata (Wurmb) Merr.	Arecaceae	0,71	30,2	7	12	0,04	0,07	96,90	0,10	1,14	4,18
1346	28	2	Banjaranyar	<200	Jati	Tectona grandis L.f.	Lamiaceae / Verbenaceae	0,7	14,0	6	8	0,04	0,02	68,28	0,07	0,80	2,94
1347	29	2	Banjaranyar	<200	Sengon	Paraserianthes falcataria (L.) I.C.	Leguminosae / Fabaceae	0,33	22,6	12	17	0,04	0,04	164,31	0,16	1,93	7,09
1348	29	2	Banjaranyar	<200	Tisuk	Hibiscus macrophyllus Roxb. ex	Malvaceae	0,4	3,5	3	4	0,04	0,00	1,71	0,00	0,02	0,07
1349	29	2	Banjaranyar	<200	Jati	Tectona grandis L.f.	Lamiaceae / Verbenaceae	0,7	14,6	5	9	0,04	0,02	82,51	0,08	0,97	3,56
1350	29	2	Banjaranyar	<200	Kelapa	Cocos nucifera L.	Arecaceae	0,5	22,9	10	14	0,04	0,04	112,30	0,11	1,32	4,84
1351	29	2	Banjaranyar	<200	Tisuk	Hibiscus macrophyllus Roxb. ex	Malvaceae	0,4	3,2	2	3	0,04	0,00	1,10	0,00	0,01	0,05
1352	29	2	Banjaranyar	<200	Sengon	Paraserianthes falcataria (L.) I.C.	Leguminosae / Fabaceae	0,33	12,7	12	14	0,04	0,01	48,07	0,05	0,56	2,07
1353	29	2	Banjaranyar	<200	Heuras	Vitex pubescens Vahl	Lamiaceae / Verbenaceae	0,88	8,9	4	7	0,04	0,01	32,55	0,03	0,38	1,40
1354	29	2	Banjaranyar	<200	Sengon	Paraserianthes falcataria (L.) I.C.	Leguminosae / Fabaceae	0,33	12,1	10	12	0,04	0,01	38,00	0,04	0,45	1,64
1355	29	2	Banjaranyar	<200	Kelapa	Cocos nucifera L.	Arecaceae	0,5	19,1	9	14	0,04	0,03	112,30	0,11	1,32	4,84
1356	29	2	Banjaranyar	<200	Jati	Tectona grandis L.f.	Lamiaceae / Verbenaceae	0,7	4,1	2	4	0,04	0,00	3,88	0,00	0,05	0,17
1357	29	2	Banjaranyar	<200	Sengon	Paraserianthes falcataria (L.) I.C.	Leguminosae / Fabaceae	0,33	21,0	9	14	0,04	0,03	120,32	0,12	1,41	5,19
1358	29	2	Banjaranyar	<200	Jati	Tectona grandis L.f.	Lamiaceae / Verbenaceae	0,7	10,2	4	8	0,04	0,01	38,10	0,04	0,45	1,64

1359	29	2	Banjaranyar	<200	Kipari	Mangifera macrocarpa Blume	Anacardiaceae	0,489	10,2	4	7	0,04	0,01	24,27	0,02	0,29	1,05
1360	29	2	Banjaranyar	<200	Sengon	Paraserianthes falcataria (L.) I.C.	Leguminosae / Fabaceae	0,33	25,5	7	13	0,04	0,05	159,92	0,16	1,88	6,90
1361	29	2	Banjaranyar	<200	Jati	Tectona grandis L.f.	Lamiaceae / Verbenaceae	0,7	9,9	2	4	0,04	0,01	19,05	0,02	0,22	0,82
1362	29	2	Banjaranyar	<200	Jati	Tectona grandis L.f.	Lamiaceae / Verbenaceae	0,7	11,1	2	6	0,04	0,01	34,49	0,03	0,41	1,49
1363	29	2	Banjaranyar	<200	Kelapa	Cocos nucifera L.	Arecaceae	0,5	26,7	4	8	0,04	0,06	66,10	0,07	0,78	2,85
1364	29	2	Banjaranyar	<200	Sengon	Paraserianthes falcataria (L.) I.C.	Leguminosae / Fabaceae	0,33	22,3	9	14	0,04	0,04	134,01	0,13	1,57	5,78
1365	29	2	Banjaranyar	<200	Sengon	Paraserianthes falcataria (L.) I.C.	Leguminosae / Fabaceae	0,33	16,5	10	13	0,04	0,02	72,64	0,07	0,85	3,13
1366	29	2	Banjaranyar	<200	Mahoni	Swietenia macrophylla King	Meliaceae	0,61	3,5	3,5	4,5	0,04	0,00	2,80	0,00	0,03	0,12
1367	29	2	Banjaranyar	<200	Sengon	Paraserianthes falcataria (L.) I.C.	Leguminosae / Fabaceae	0,33	6,4	5	7	0,04	0,00	7,16	0,01	0,08	0,31
1368	29	2	Banjaranyar	<200	Mahoni	Swietenia macrophylla King	Meliaceae	0,61	5,4	3	4,5	0,04	0,00	6,22	0,01	0,07	0,27
1369	29	2	Banjaranyar	<200	Rambutan	Nephelium lappaceum L.	Sapindaceae	0,71	16,5	6	8	0,04	0,02	93,93	0,09	1,10	4,05
1370	29	2	Banjaranyar	<200	Mahoni	Swietenia macrophylla King	Meliaceae	0,61	5,1	3	4,5	0,04	0,00	5,57	0,01	0,07	0,24
1371	29	2	Banjaranyar	<200	Jati	Tectona grandis L.f.	Lamiaceae / Verbenaceae	0,7	16,9	7	10	0,04	0,02	117,79	0,12	1,38	5,08
1372	29	2	Banjaranyar	<200	Mahoni	Swietenia macrophylla King	Meliaceae	0,61	5,1	2	4	0,04	0,00	5,00	0,00	0,06	0,22
1373	29	2	Banjaranyar	<200	Rambutan	Nephelium lappaceum L.	Sapindaceae	0,71	6,7	2	6	0,04	0,00	13,71	0,01	0,16	0,59
1374	29	2	Banjaranyar	<200	Sengon	Paraserianthes falcataria (L.) I.C.	Leguminosae / Fabaceae	0,33	5,4	5	6	0,04	0,00	4,61	0,00	0,05	0,20
1375	29	2	Banjaranyar	<200	Mahoni	Swietenia macrophylla King	Meliaceae	0,61	8,9	4	7	0,04	0,01	23,27	0,02	0,27	1,00
1376	29	2	Banjaranyar	<200	Tisuk	Hibiscus macrophyllus Roxb. ex I	Malvaceae	0,4	20,0	12	15	0,04	0,03	140,38	0,14	1,65	6,05
1377	29	2	Banjaranyar	<200	Salam	Syzygium polyanthum (Wight) Wa	Myrtaceae	0,66	21,3	9	16	0,04	0,04	263,73	0,26	3,10	11,37
1378	29	2	Banjaranyar	<200	Tisuk	Hibiscus macrophyllus Roxb. ex I	Malvaceae	0,4	3,2	2	3	0,04	0,00	1,10	0,00	0,01	0,05
1379	29	2	Banjaranyar	<200	Sengon	Paraserianthes falcataria (L.) I.C.	Leguminosae / Fabaceae	0,33	7,0	7	11	0,04	0,00	12,89	0,01	0,15	0,56
1380	29	2	Banjaranyar	<200	Kelapa	Cocos nucifera L.	Arecaceae	0,5	24,5	12	16	0,04	0,05	127,70	0,13	1,50	5,51
1381	29	2	Banjaranyar	<200	Tisuk	Hibiscus macrophyllus Roxb. ex I	Malvaceae	0,4	2,9	3	4	0,04	0,00	1,18	0,00	0,01	0,05
1382	29	2	Banjaranyar	<200	Tisuk	Hibiscus macrophyllus Roxb. ex I	Malvaceae	0,4	4,5	2,5	4	0,04	0,00	2,66	0,00	0,03	0,11
1383	29	2	Banjaranyar	<200	Sengon	Paraserianthes falcataria (L.) I.C.	Leguminosae / Fabaceae	0,33	8,9	6	10	0,04	0,01	18,38	0,02	0,22	0,79
1384	29	2	Banjaranyar	<200	Tisuk	Hibiscus macrophyllus Roxb. ex I	Malvaceae	0,4	3,2	3	5	0,04	0,00	1,76	0,00	0,02	0,08
1385	29	2	Banjaranyar	<200	Sengon	Paraserianthes falcataria (L.) I.C.	Leguminosae / Fabaceae	0,33	6,4	8	11	0,04	0,00	10,83	0,01	0,13	0,47
1386	29	2	Banjaranyar	<200	Kelapa	Cocos nucifera L.	Arecaceae	0,5	20,7	9	13	0,04	0,03	104,60	0,10	1,23	4,51
1387	29	2	Banjaranyar	<200	Mahoni	Swietenia macrophylla King	Meliaceae	0,61	10,2	5	8	0,04	0,01	33,59	0,03	0,39	1,45
1388	29	2	Banjaranyar	<200	Mahoni	Swietenia macrophylla King	Meliaceae	0,61	12,1	3	8	0,04	0,01	46,01	0,05	0,54	1,98
1389	29	2	Banjaranyar	<200	Mahoni	Swietenia macrophylla King	Meliaceae	0,61	4,8	3	5	0,04	0,00	5,45	0,01	0,06	0,23
1390	29	2	Banjaranyar	<200	Mahoni	Swietenia macrophylla King	Meliaceae	0,61	11,8	3	8	0,04	0,01	43,82	0,04	0,51	1,89
1391	29	2	Banjaranyar	<200	Heuras	Vitex pubescens Vahl	Lamiaceae / Verbenaceae	0,88	8,6	3	9	0,04	0,01	38,34	0,04	0,45	1,65
1392	29	2	Banjaranyar	<200	Jati	Tectona grandis L.f.	Lamiaceae / Verbenaceae	0,7	28,6	6	15	0,04	0,06	450,51	0,45	5,29	19,43
1393	29	2	Banjaranyar	<200	Mahoni	Swietenia macrophylla King	Meliaceae	0,61	3,5	3	4	0,04	0,00	2,52	0,00	0,03	0,11
1394	29	2	Banjaranyar	<200	Mahoni	Swietenia macrophylla King	Meliaceae	0,61	4,8	3	4,5	0,04	0,00	4,95	0,00	0,06	0,21
1395	29	2	Banjaranyar	<200	Tisuk	Hibiscus macrophyllus Roxb. ex I	Malvaceae	0,4	30,2	12	17	0,04	0,07	334,11	0,33	3,93	14,41
1396	29	2	Banjaranyar	<200	Mahoni	Swietenia macrophylla King	Meliaceae	0,61	8,6	3	9	0,04	0,01	27,40	0,03	0,32	1,18
1397	29	2	Banjaranyar	<200	Kelapa	Cocos nucifera L.	Arecaceae	0,5	24,5	12	16	0,04	0,05	127,70	0,13	1,50	5,51
1398	29	2	Banjaranyar	<200	Huru kacang	Neolitsea triplinervia Merr.	Lauraceae	0,56	10,5	4	10	0,04	0,01	40,31	0,04	0,47	1,74
1399	29	2	Banjaranyar	<200	Mahoni	Swietenia macrophylla King	Meliaceae	0,61	7,0	5	8	0,04	0,00	16,91	0,02	0,20	0,73
1400	29	2	Banjaranyar	<200	Mahoni	Swietenia macrophylla King	Meliaceae	0,61	6,0	3	7	0,04	0,00	11,44	0,01	0,13	0,49
1401	29	2	Banjaranyar	<200	Mahoni	Swietenia macrophylla King	Meliaceae	0,61	8,9	4	9	0,04	0,01	29,29	0,03	0,34	1,26
1402	29	2	Banjaranyar	<200	Mahoni	Swietenia macrophylla King	Meliaceae	0,61	4,5	2,5	5	0,04	0,00	4,80	0,00	0,06	0,21
1403	29	2	Banjaranyar	<200	Pangsor	Ficus callosa Willd.	Moraceae	0,33	11,8	9	11	0,04	0,01	33,41	0,03	0,39	1,44
1404	29	2	Banjaranyar	<200	Mahoni	Swietenia macrophylla King	Meliaceae	0,61	4,8	3	5	0,04	0,00	5,45	0,01	0,06	0,23
1405	29	2	Banjaranyar	<200	Kelapa	Cocos nucifera L.	Arecaceae	0,5	26,4	12	16	0,04	0,05	127,70	0,13	1,50	5,51
1406	29	2	Banjaranyar	<200	Sirsak	Annona muricata L.	Annonaceae	0,36	7,0	4	7	0,04	0,00	9,23	0,01	0,11	0,40
1407	29	2	Banjaranyar	<200	Sengon	Paraserianthes falcataria (L.) I.C.	Leguminosae / Fabaceae	0,33	8,9	6	9	0,04	0,01	16,69	0,02	0,20	0,72
1408	29	2	Banjaranyar	<200	Mahoni	Swietenia macrophylla King	Meliaceae	0,61	4,8	3	4,5	0,04	0,00	4,95	0,00	0,06	0,21
1409	30	1	Banjaranyar	<200	Jati	Tectona grandis L.f.	Lamiaceae / Verbenaceae	0,7	7,3	3	5	0,04	0,00	13,53	0,01	0,16	0,58
1410	30	1	Banjaranyar	<200	Jati	Tectona grandis L.f.	Lamiaceae / Verbenaceae	0,7	14,0	4	8	0,04	0,02	68,28	0,07	0,80	2,94
1411	30	1	Banjaranyar	<200	Jati	Tectona grandis L.f.	Lamiaceae / Verbenaceae	0,7	5,4	3	5	0,04	0,00	7,77	0,01	0,09	0,34
1412	30	1	Banjaranyar	<200	Jati	Tectona grandis L.f.	Lamiaceae / Verbenaceae	0,7	24,8	9	13	0,04	0,05	304,03	0,30	3,57	13,11
1413	30	1	Banjaranyar	<200	Jati	Tectona grandis L.f.	Lamiaceae / Verbenaceae	0,7	17,2	5	9	0,04	0,02	110,68	0,11	1,30	4,77
1414	30	1	Banjaranyar	<200	Jati	Tectona grandis L.f.	Lamiaceae / Verbenaceae	0,7	12,7	3	9	0,04	0,01	63,87	0,06	0,75	2,75
1415	30	1	Banjaranyar	<200	Jati	Tectona grandis L.f.	Lamiaceae / Verbenaceae	0,7	13,7	4	9	0,04	0,01	72,92	0,07	0,86	3,14
1416	30	1	Banjaranyar	<200	Jati	Tectona grandis L.f.	Lamiaceae / Verbenaceae	0,7	13,7	6	9	0,04	0,01	72,92	0,07	0,86	3,14
1417	30	1	Banjaranyar	<200	Jati	Tectona grandis L.f.	Lamiaceae / Verbenaceae	0,7	14,3	7	9	0,04	0,02	79,25	0,08	0,93	3,42
1418	30	1	Banjaranyar	<200	Jati	Tectona grandis L.f.	Lamiaceae / Verbenaceae	0,7	22,0	7	12	0,04	0,04	225,70	0,23	2,65	9,73
1419	30	1	Banjaranyar	<200	Jati	Tectona grandis L.f.	Lamiaceae / Verbenaceae	0,7	20,0	9	14	0,04	0,03	220,03	0,22	2,59	9,49
1420	30	1	Banjaranyar	<200	Jati	Tectona grandis L.f.	Lamiaceae / Verbenaceae	0,7	8,3	4	7	0,04	0,01	23,04	0,02	0,27	0,99
1421	30	1	Banjaranyar	<200	Jati	Tectona grandis L.f.	Lamiaceae / Verbenaceae	0,7	9,9	7	10	0,04	0,01	44,10	0,04	0,52	1,90
1422	30	1	Banjaranyar	<200	Jati	Tectona grandis L.f.	Lamiaceae / Verbenaceae	0,7	11,5	7	10	0,04	0,01	57,99	0,06	0,68	2,50
1423	30	1	Banjaranyar	<200	Jati	Tectona grandis L.f.	Lamiaceae / Verbenaceae	0,7	10,5	3	8	0,04	0,01	40,31	0,04	0,47	1,74
1424	30	1	Banjaranyar	<200	Jati	Tectona grandis L.f.	Lamiaceae / Verbenaceae	0,7	8,0	6	10	0,04	0,00	29,73	0,03	0,35	1,28
1425	30	1	Banjaranyar	<200	Jati	Tectona grandis L.f.	Lamiaceae / Verbenaceae	0,7	7,0	2	6	0,04	0,00	14,73	0,01	0,17	0,64
1426	30	1	Banjaranyar	<200	Jati	Tectona grandis L.f.	Lamiaceae / Verbenaceae	0,7	13,7	5	10	0,04	0,01	80,31	0,08	0,94	3,46
1427	30	1	Banjaranyar	<200	Jati	Tectona grandis L.f.	Lamiaceae / Verbenaceae	0,7	22,6	10	15	0,04	0,04	291,77	0,29	3,43	12,58
1428	30	1	Banjaranyar	<200	Jati	Tectona grandis L.f.	Lamiaceae / Verbenaceae	0,7	7,0	5	8	0,04	0,00	19,18	0,02	0,23	0,83
1429	30	1	Banjaranyar	<200	Jati	Tectona grandis L.f.	Lamiaceae / Verbenaceae	0,7	8,3	4	8	0,04	0,01	26,04	0,03	0,31	1,12
1430	30	1	Banjaranyar	<200	Jati	Tectona grandis L.f.	Lamiaceae / Verbenaceae	0,7	10,5	7	9	0,04	0,01	44,90	0,04	0,53	1,94
1431	30	1	Banjaranyar	<200	Jati	Tectona grandis L.f.	Lamiaceae / Verbenaceae	0,7	14,3	6	12	0,04	0,02	103,14	0,10	1,21	4,45
1432	30	1	Banjaranyar	<200	Jati	Tectona grandis L.f.	Lamiaceae / Verbenaceae	0,7	7,3	4	7	0,04	0,00	18,41	0,02	0,22	0,79
1433	30	1	Banjaranyar	<200	Jati	Tectona grandis L.f.	Lamiaceae / Verbenaceae	0,7	7,6	3	5	0,04	0,00	14,62	0,01	0,17	0,63
1434	30	1	Banjaranyar	<200	Jati	Tectona grandis L.f.	Lamiaceae / Verbenaceae	0,7	6,7	7	10	0,04	0,00	21,60	0,02	0,25	0,93
1435	30	1	Banjaranyar	<200	Jati	Tectona grandis L.f.	Lamiaceae / Verbenaceae	0,7	9,2	6	10	0,04	0,01	39,02	0,04	0,46	1,68
1436	30	1	Banjaranyar	<200	Jati	Tectona grandis L.f.	Lamiaceae / Verbenaceae	0,7	20,4	9	14	0,04	0,03	226,47	0,23	2,66	9,77
1437	30	1	Banjaranyar	<200	Jati	Tectona grandis L.f.	Lamiaceae / Verbenaceae	0,7	18,8	6	12	0,04	0,03	169,42	0,17	1,99	7,31
1438	30	1	Banjaranyar	<200	Jati	Tectona grandis L.f.	Lamiaceae / Verbenaceae	0,7	6,4	2	3	0,04	0,00	6,56	0,01	0,08	0,28
1439	30	1	Banjaranyar	<200	Jati	Tectona grandis L.f.	Lamiaceae / Verbenaceae	0,7	6,7	5	7	0,04	0,00	15,58	0,02	0,18	0,67
1440	30	1	Banjaranyar	<200	Jati	Tectona grandis L.f.	Lamiaceae / Verbenaceae	0,7	16,9	6	10	0,04	0,02	117,79	0,12	1,38	5,08
1441	30	1	Banjaranyar	<200	Jati	Tectona grandis L.f.	Lamiaceae / Verbenaceae	0,7	7,0	4	6	0,04	0,00	14,73	0,01	0,17	0,64
1442	30	1	Banjaranyar	<200	Jati	Tectona grandis L.f.	Lamiaceae / Verbenaceae	0,7	6,0	5	7	0,04	0,00	12,97	0,01	0,15	0,56
1443	30	1	Banjaranyar	<200	Jati	Tectona grandis L.f.	Lamiaceae / Verbenaceae	0,7	5,4	4	7	0,04	0,00	10,58	0,01	0,12	0,46
1444	30	1	Banjaranyar	<200	Jati	Tectona grandis L.f.	Lamiaceae / Verbenaceae	0,7	13,4	7	12	0,04	0,01	90,90	0,09	1,07	3,92
1445	30	1	Banjaranyar	<200	Jati	Tectona grandis L.f.	Lamiaceae / Verbenaceae	0,7	14,6	8	13	0,04	0,02	115,55	0,12	1,36	4,98
1446	30	1	Banjaranyar	<200	Jati	Tectona grandis L.f.	Lamiaceae / Verbenaceae	0,7	6,7	6	11	0,04	0,00	23,57	0,02	0,28	1,02
1447	30	1	Banjaranyar	<200	Jati	Tectona grandis L.f.	Lamiaceae / Verbenaceae	0,7	8,3	3	7	0,04	0,01	23,04	0,02	0,27	0,99
1448	30	1	Banjaranyar	<200	Jati	Tectona grandis L.f.	Lamiaceae / Verbenaceae	0,7	21,3	8	14	0,04	0,04	246,29	0,25	2,89	10,62
1449	30	1	Banjaranyar	<200	Jati	Tectona grandis L.f.	Lamiaceae / Verbenaceae	0,7	9,9	7	10	0,04	0,01	44,10	0,04	0,52	1,90
1450	30	1	Banjaranyar	<200	Jati	Tectona grandis L.f.	Lamiaceae / Verbenaceae	0,7	10,5	6	9	0,04	0,01	44,90	0,04	0,53	1,94
1451	30	1	Banjaranyar	<200	Jati	Tectona grandis L.f.	Lamiaceae / Verbenaceae	0,7	6,7	3	5	0,04	0,00	11,45	0,01	0,13	0,49
1452	30	1	Banjaranyar	<200	Jati	Tectona grandis L.f.	Lamiaceae / Verbenaceae	0,7	4,8	3,5	5	0,04	0,00	6,18	0,01	0,07	0,27
1453	30	1	Banjaranyar	<200	Jati	Tectona grandis L.f.	Lamiaceae / Verbenaceae	0,7	11,8	3	10	0,04	0,01	60,98	0,06	0,72	2,63
1454	30	1	Banjaranyar	<200	Jati	Tectona grandis L.f.	Lamiaceae / Verbenaceae	0,7	8,3	4	8	0,04	0,01	26,04	0,03	0,31	1,12
1455	30	1	Banjaranyar	<200	Jati	Tectona grandis L.f.	Lamiaceae / Verbenaceae	0,7	12,4	6	12	0,04	0,01	79,36	0,08	0,93	3,42
1456	30	1	Banjaranyar	<200	Jati	Tectona grandis L.f.	Lamiaceae / Verbenaceae	0,7	5,1	3	9	0,04	0,00	11,92	0,01	0,14	0,51
1457	30	1	Banjaranyar	<200	Jati	Tectona grandis L.f.	Lamiaceae / Verbenaceae	0,7	20,0	9	14	0,04	0,03	220,03	0,22	2,59	9,49
1458	30	1	Banjaranyar	<200	Jati	Tectona grandis L.f.	Lamiaceae / Verbenaceae	0,7	27,0	7	14	0,04	0,06	380,87	0,38	4,48	16,42
1459	30	1	Banjaranyar	<200	Jati	Tectona grandis L.f.	Lamiaceae / Verbenaceae	0,7	7,3	7	10	0,04	0,00	25,52	0,03	0,30	1,10
1460	30	1	Banjaranyar	<200	Jati	Tectona grandis L.f.	Lamiaceae / Verbenaceae	0,7	8,9	6	10	0,04	0,01	36,60	0,04	0,43	1,58
1461	30	1	Banjaranyar	<200	Jati	Tectona grandis L.f.	Lamiaceae / Verbenaceae	0,7	7,6	7	10	0,04	0,00	27,59	0,03	0,32	1,19
1462	30	1	Banjaranyar	<200	Jati	Tectona grandis L.f.	Lamiaceae / Verbenaceae	0,7	12,4	7	12	0,04	0,01	79,36	0,08	0,93	3,42
1463	30	1	Banjaranyar	<200	Jati	Tectona grandis L.f.	Lamiaceae / Verbenaceae	0,7	9,2	5	7	0,04	0,01	28,15	0,03	0,33	1,21
1464	30	1	Banjaranyar	<200	Jati	Tectona grandis L.f.	Lamiaceae / Verbenaceae	0,7	19,1	6	12	0,04	0,03	174,72	0,17	2,05	7,53
1465	30	1	Banjaranyar	<200	Jati	Tectona grandis L.f.	Lamiaceae / Verbenaceae	0,7	9,9	6	9	0,04	0,01	40,04	0,04	0,47	1,73
1466	30	1	Banjaranyar	<200	Jati	Tectona grandis L.f.	Lamiaceae / Verbenaceae	0,7	14,6	6	11	0,04	0,02	99,16	0,10	1,17	4,28
1467	30	1	Banjaranyar	<200	Jati	Tectona grandis L.f.	Lamiaceae / Verbenaceae	0,7	7,0	6	9	0,04	0,00	21,36	0,02	0,25	0,92
1468	30	1	Banjaranyar	<200	Jati	Tectona grandis L.f.	Lamiaceae / Verbenaceae	0,7	16,9	7	12	0,04	0,02	139,20	0,14	1,64	6,00
1469	30	1	Banjaranyar	<200	Jati	Tectona grandis L.f.	Lamiaceae / Verbenaceae	0,7	8,0	5	8	0,04	0,00	24,24	0,02	0,28	1,05
1470	30	1	Banjaranyar	<200	Jati	Tectona grandis L.f.	Lamiaceae / Verbenaceae	0,7	10,2	4	9	0,04	0,01	42,44	0,04	0,50	1,83
1471	31	2	Banjaranyar	<200	Jati	Tectona grandis L.f.	Lamiaceae / Verbenaceae	0,7	12,1	3	7	0,04	0,01	46,19	0,05	0,54	1,99
1472	31	2	Banjaranyar	<200	Mahoni	Swietenia macrophylla King	Meliaceae	0,61	19,1	7	11	0,04	0,03	142,22	0,14	1,67	6,13
1473	31	2	Banjaranyar	<200	Jati	Tectona grandis L.f.	Lamiaceae / Verbenaceae	0,7	6,0	3	7	0,04	0,00	12,97	0,01	0,15	0,56
1474	31	2	Banjaranyar	<200	Tisuk	Hibiscus macrophyllus Roxb. ex I	Malvaceae	0,4	34,0	12	18	0,04	0,09	437,79	0,44	5,14	18,88
1475	31	2	Banjaranyar	<200	Kelapa	Cocos nucifera L.	Arecaceae	0,5	26,7	10	14	0,04	0,06	112,30	0,11	1,32	4,84
1476	31	2	Banjaranyar	<200	Heuras	Vitex pubescens Vahl	Lamiaceae / Verbenaceae	0,88	13,4	7	11	0,04	0,01	103,51	0,10	1,22	4,46
1477	31	2	Banjaranyar	<200	Mahoni	Swietenia macrophylla King	Meliaceae	0,61	22,9	4	12	0,04	0,04	215,10	0,22	2,53	9,28
1478	31	2	Banjaranyar	<200	Jati	Tectona grandis L.f.	Lamiaceae / Verbenaceae	0,7	53,5	9	20	0,04	0,22	1.839,67	1,84	21,62	79,33
1479	31	2	Banjaranyar	<200	Tisuk	Hibiscus macrophyllus Roxb. ex I	Malvaceae	0,4	2,5	3	3,5	0,04	0,00	0,84	0,00	0,01	0,04
1480	31	2	Banjaranyar	<200	Kelapa	Cocos nucifera L.	Arecaceae	0,5	28,6	14	18	0,04	0,06	143,10	0,14	1,68	6,17
1481	31	2	Banjaranyar	<200	Mahoni	Swietenia macrophylla King	Meliaceae	0,61	11,1	4	8	0,04	0,01	39,58	0,04	0,47	1,71
1482	31	2	Banjaranyar	<200	Mahoni	Swietenia macrophylla King	Meliaceae	0,61	19,7	7	12	0,04	0,03	163,56	0,16	1,92	7,05

1483	31	2	Banjaranyar	<200	Tisuk	Hibiscus macrophyllus Roxb. ex I	Malvaceae	0,4	3,8	4	4,5	0,04	0,00	2,23	0,00	0,03	0,10
1484	31	2	Banjaranyar	<200	Tisuk	Hibiscus macrophyllus Roxb. ex I	Malvaceae	0,4	2,5	2	2,5	0,04	0,00	0,62	0,00	0,01	0,03
1485	31	2	Banjaranyar	<200	Tisuk	Hibiscus macrophyllus Roxb. ex I	Malvaceae	0,4	2,5	1,8	2	0,04	0,00	0,51	0,00	0,01	0,02
1486	31	2	Banjaranyar	<200	Jati	Tectona grandis L.f.	Lamiaceae / Verbenaceae	0,7	2,9	2	2,5	0,04	0,00	1,29	0,00	0,02	0,06
1487	31	2	Banjaranyar	<200	Tisuk	Hibiscus macrophyllus Roxb. ex I	Malvaceae	0,4	3,2	2,5	3	0,04	0,00	1,10	0,00	0,01	0,05
1488	31	2	Banjaranyar	<200	Caruy	Pterospermum javanicum Jungh.	Malvaceae	0,53	27,4	10	17	0,04	0,06	360,29	0,36	4,23	15,54
1489	31	2	Banjaranyar	<200	Mahoni	Swietenia macrophylla King	Meliaceae	0,61	45,8	10	18	0,04	0,16	1.110,28	1,11	13,05	47,88
1490	31	2	Banjaranyar	<200	Mahoni	Swietenia macrophylla King	Meliaceae	0,61	43,6	12	20	0,04	0,15	1.116,08	1,12	13,11	48,13
1491	31	2	Banjaranyar	<200	Jati	Tectona grandis L.f.	Lamiaceae / Verbenaceae	0,7	11,1	3	8	0,04	0,01	44,89	0,04	0,53	1,94
1492	31	2	Banjaranyar	<200	Jati	Tectona grandis L.f.	Lamiaceae / Verbenaceae	0,7	33,4	10	15	0,04	0,09	597,51	0,60	7,02	25,77
1493	31	2	Banjaranyar	<200	Caruy	Pterospermum javanicum Jungh.	Malvaceae	0,53	39,1	18	23	0,04	0,12	915,39	0,92	10,76	39,47
1494	31	2	Banjaranyar	<200	Jati	Tectona grandis L.f.	Lamiaceae / Verbenaceae	0,7	34,4	7	14	0,04	0,09	590,63	0,59	6,94	25,47
1495	31	2	Banjaranyar	<200	Tisuk	Hibiscus macrophyllus Roxb. ex I	Malvaceae	0,4	23,2	9	15	0,04	0,04	183,87	0,18	2,16	7,93
1496	31	2	Banjaranyar	<200	Mahoni	Swietenia macrophylla King	Meliaceae	0,61	17,5	6	10	0,04	0,02	111,13	0,11	1,31	4,79
1497	31	2	Banjaranyar	<200	Tisuk	Hibiscus macrophyllus Roxb. ex I	Malvaceae	0,4	25,5	13	17	0,04	0,05	243,87	0,24	2,87	10,52
1498	31	2	Banjaranyar	<200	Jati	Tectona grandis L.f.	Lamiaceae / Verbenaceae	0,7	33,1	10	16	0,04	0,09	622,89	0,62	7,32	26,86
1499	31	2	Banjaranyar	<200	Jati	Tectona grandis L.f.	Lamiaceae / Verbenaceae	0,7	2,9	3	4	0,04	0,00	1,98	0,00	0,02	0,09
1500	31	2	Banjaranyar	<200	Jati	Tectona grandis L.f.	Lamiaceae / Verbenaceae	0,7	3,8	2	3	0,04	0,00	2,57	0,00	0,03	0,11
1501	31	2	Banjaranyar	<200	Jati	Tectona grandis L.f.	Lamiaceae / Verbenaceae	0,7	10,5	4	8	0,04	0,01	40,31	0,04	0,47	1,74
1502	31	2	Banjaranyar	<200	Kelapa	Cocos nucifera L.	Arecaceae	0,5	26,4	11	15	0,04	0,05	120,00	0,12	1,41	5,17
1503	31	2	Banjaranyar	<200	Mahoni	Swietenia macrophylla King	Meliaceae	0,61	10,2	3	6	0,04	0,01	25,80	0,03	0,30	1,11
1504	31	2	Banjaranyar	<200	Jati	Tectona grandis L.f.	Lamiaceae / Verbenaceae	0,7	3,2	2	2,5	0,04	0,00	1,56	0,00	0,02	0,07
1505	31	2	Banjaranyar	<200	Jati	Tectona grandis L.f.	Lamiaceae / Verbenaceae	0,7	4,1	2	2,5	0,04	0,00	2,52	0,00	0,03	0,11
1506	31	2	Banjaranyar	<200	Mahoni	Swietenia macrophylla King	Meliaceae	0,61	9,5	2	8	0,04	0,01	29,84	0,03	0,35	1,29
1507	32	3	Banjaranyar	<200	Jati	Tectona grandis L.f.	Lamiaceae / Verbenaceae	0,7	9,9	2	6	0,04	0,01	27,62	0,03	0,32	1,19
1508	32	3	Banjaranyar	<200	Kakao	Theobroma cacao L.	Malvaceae	0,36	7,6	1,5	2	0,04	0,00	6,76	0,01	0,08	0,29
1509	32	3	Banjaranyar	<200	Akasia mangium	Acacia mangium Willd.	Leguminosae / Fabaceae	0,5	26,4	12	15	0,04	0,05	285,38	0,29	3,35	12,31
1510	32	3	Banjaranyar	<200	Rambutan	Nephelium lappaceum L.	Sapindaceae	0,71	7,3	3	6	0,04	0,00	16,19	0,02	0,19	0,70
1511	32	3	Banjaranyar	<200	Mahoni	Swietenia macrophylla King	Meliaceae	0,61	8,6	4	7	0,04	0,01	21,77	0,02	0,26	0,94
1512	32	3	Banjaranyar	<200	Mahoni	Swietenia macrophylla King	Meliaceae	0,61	6,4	3	6	0,04	0,00	10,91	0,01	0,13	0,47
1513	32	3	Banjaranyar	<200	Akasia mangium	Acacia mangium Willd.	Leguminosae / Fabaceae	0,5	27,0	12	15	0,04	0,06	298,11	0,30	3,50	12,86
1514	32	3	Banjaranyar	<200	Mahoni	Swietenia macrophylla King	Meliaceae	0,61	6,4	3	5	0,04	0,00	9,23	0,01	0,11	0,40
1515	32	3	Banjaranyar	<200	Akasia mangium	Acacia mangium Willd.	Leguminosae / Fabaceae	0,5	34,0	8	14	0,04	0,09	426,64	0,43	5,01	18,40
1516	32	3	Banjaranyar	<200	Mahoni	Swietenia macrophylla King	Meliaceae	0,61	18,1	3	11	0,04	0,03	129,47	0,13	1,52	5,58
1517	32	3	Banjaranyar	<200	Jati	Tectona grandis L.f.	Lamiaceae / Verbenaceae	0,7	27,4	4	13	0,04	0,06	363,58	0,36	4,27	15,68
1518	32	3	Banjaranyar	<200	Tisuk	Hibiscus macrophyllus Roxb. ex I	Malvaceae	0,4	8,0	8	10	0,04	0,00	17,81	0,02	0,21	0,77
1519	32	3	Banjaranyar	<200	Kayu Gunung	Sp1	0	0,5	4,1	2	4	0,04	0,00	2,85	0,00	0,03	0,12
1520	32	3	Banjaranyar	<200	Jati	Tectona grandis L.f.	Lamiaceae / Verbenaceae	0,7	11,5	4	11	0,04	0,01	63,28	0,06	0,74	2,73
1521	32	3	Banjaranyar	<200	Jati	Tectona grandis L.f.	Lamiaceae / Verbenaceae	0,7	4,8	3	4	0,04	0,00	5,04	0,01	0,06	0,22
1522	32	3	Banjaranyar	<200	Jati	Tectona grandis L.f.	Lamiaceae / Verbenaceae	0,7	10,8	5	8	0,04	0,01	42,57	0,04	0,50	1,84
1523	32	3	Banjaranyar	<200	Mahoni	Swietenia macrophylla King	Meliaceae	0,61	5,1	3	5	0,04	0,00	6,13	0,01	0,07	0,26
1524	32	3	Banjaranyar	<200	Kayu Gunung	Sp1	0	0,5	7,3	4	9	0,04	0,00	17,03	0,02	0,20	0,73
1525	32	3	Banjaranyar	<200	Jati	Tectona grandis L.f.	Lamiaceae / Verbenaceae	0,7	5,7	2	4	0,04	0,00	7,04	0,01	0,08	0,30
1526	32	3	Banjaranyar	<200	Mahoni	Swietenia macrophylla King	Meliaceae	0,61	7,0	3	5	0,04	0,00	10,99	0,01	0,13	0,47
1527	32	3	Banjaranyar	<200	Mahoni	Swietenia macrophylla King	Meliaceae	0,61	12,4	3	7	0,04	0,01	42,70	0,04	0,50	1,84
1528	32	3	Banjaranyar	<200	Mahoni	Swietenia macrophylla King	Meliaceae	0,61	12,1	4	8	0,04	0,01	46,01	0,05	0,54	1,98
1529	32	3	Banjaranyar	<200	Mahoni	Swietenia macrophylla King	Meliaceae	0,61	11,1	3,5	8,5	0,04	0,01	41,84	0,04	0,49	1,80
1530	32	3	Banjaranyar	<200	Mahoni	Swietenia macrophylla King	Meliaceae	0,61	11,8	3	8	0,04	0,01	43,82	0,04	0,51	1,89
1531	32	3	Banjaranyar	<200	Mahoni	Swietenia macrophylla King	Meliaceae	0,61	9,5	3	7	0,04	0,01	26,40	0,03	0,31	1,14
1532	32	3	Banjaranyar	<200	Kelapa	Cocos nucifera L.	Arecaceae	0,5	25,8	8	12	0,04	0,05	96,90	0,10	1,14	4,18
1533	32	3	Banjaranyar	<200	Mahoni	Swietenia macrophylla King	Meliaceae	0,61	12,7	5	9	0,04	0,01	56,30	0,06	0,66	2,43
1534	32	3	Banjaranyar	<200	Jati	Tectona grandis L.f.	Lamiaceae / Verbenaceae	0,7	3,8	2,5	3,5	0,04	0,00	2,96	0,00	0,03	0,13
1535	32	3	Banjaranyar	<200	Jati	Tectona grandis L.f.	Lamiaceae / Verbenaceae	0,7	3,2	2	2,5	0,04	0,00	1,56	0,00	0,02	0,07
1536	32	3	Banjaranyar	<200	Tisuk	Hibiscus macrophyllus Roxb. ex I	Malvaceae	0,4	5,4	5	6,5	0,04	0,00	5,92	0,01	0,07	0,26
1537	32	3	Banjaranyar	<200	Jati	Tectona grandis L.f.	Lamiaceae / Verbenaceae	0,7	4,8	2	3	0,04	0,00	3,87	0,00	0,05	0,17
1538	32	3	Banjaranyar	<200	Kayu Gunung	Sp1	0	0,5	5,4	3	5	0,04	0,00	5,71	0,01	0,07	0,25
1539	32	3	Banjaranyar	<200	Jati	Tectona grandis L.f.	Lamiaceae / Verbenaceae	0,7	4,1	4	2,5	0,04	0,00	2,52	0,00	0,03	0,11
1540	32	3	Banjaranyar	<200	Jati	Tectona grandis L.f.	Lamiaceae / Verbenaceae	0,7	8,9	3	7	0,04	0,01	26,40	0,03	0,31	1,14
1541	32	3	Banjaranyar	<200	Pangsor	Ficus callosa Willd.	Moraceae	0,33	5,4	4	7	0,04	0,00	5,31	0,01	0,06	0,23
1542	32	3	Banjaranyar	<200	Jati	Tectona grandis L.f.	Lamiaceae / Verbenaceae	0,7	7,0	3	5	0,04	0,00	12,47	0,01	0,15	0,54
1543	32	3	Banjaranyar	<200	Tisuk	Hibiscus macrophyllus Roxb. ex I	Malvaceae	0,4	11,8	6	9	0,04	0,01	33,16	0,03	0,39	1,43
1544	32	3	Banjaranyar	<200	Jati	Tectona grandis L.f.	Lamiaceae / Verbenaceae	0,7	5,7	3	5	0,04	0,00	8,63	0,01	0,10	0,37
1545	32	3	Banjaranyar	<200	Jati	Tectona grandis L.f.	Lamiaceae / Verbenaceae	0,7	3,2	1,8	2,5	0,04	0,00	1,56	0,00	0,02	0,07
1546	32	3	Banjaranyar	<200	Cengkeh	Syzygium aromaticum (L.) Merr. a	Myrtaceae	0,79	11,8	4	7	0,04	0,01	49,14	0,05	0,58	2,12
1547	32	3	Banjaranyar	<200	Mahoni	Swietenia macrophylla King	Meliaceae	0,61	12,7	4	9	0,04	0,01	56,30	0,06	0,66	2,43
1548	32	3	Banjaranyar	<200	Cengkeh	Syzygium aromaticum (L.) Merr. a	Myrtaceae	0,79	10,2	4	5	0,04	0,01	27,67	0,03	0,33	1,19
1549	32	3	Banjaranyar	<200	Mahoni	Swietenia macrophylla King	Meliaceae	0,61	14,6	4	8	0,04	0,02	65,30	0,07	0,77	2,82
1550	32	3	Banjaranyar	<200	Mahoni	Swietenia macrophylla King	Meliaceae	0,61	14,0	4	8	0,04	0,02	60,19	0,06	0,71	2,60
1551	32	3	Banjaranyar	<200	Mahoni	Swietenia macrophylla King	Meliaceae	0,61	13,7	4	9	0,04	0,01	64,28	0,06	0,76	2,77
1552	32	3	Banjaranyar	<200	Mahoni	Swietenia macrophylla King	Meliaceae	0,61	17,5	5	12	0,04	0,02	131,33	0,13	1,54	5,66
1553	32	3	Banjaranyar	<200	Mahoni	Swietenia macrophylla King	Meliaceae	0,61	11,1	4	8	0,04	0,01	39,58	0,04	0,47	1,71
1554	32	3	Banjaranyar	<200	Mahoni	Swietenia macrophylla King	Meliaceae	0,61	11,8	3,5	7,5	0,04	0,01	41,30	0,04	0,49	1,78
1555	32	3	Banjaranyar	<200	Mahoni	Swietenia macrophylla King	Meliaceae	0,61	11,8	4	8	0,04	0,01	43,82	0,04	0,51	1,89
1556	32	3	Banjaranyar	<200	Heuras	Vitex pubescens Vahl	Lamiaceae / Verbenaceae	0,88	29,6	9	12	0,04	0,07	480,90	0,48	5,65	20,74
1557	32	3	Banjaranyar	<200	Sengon	Paraserianthes falcataria (L.) I.C.	Leguminosae / Fabaceae	0,33	7,6	5	7	0,04	0,00	9,99	0,01	0,12	0,43
1558	32	3	Banjaranyar	<200	Tisuk	Hibiscus macrophyllus Roxb. ex I	Malvaceae	0,4	6,0	4	7	0,04	0,00	7,77	0,01	0,09	0,34
1559	32	3	Banjaranyar	<200	Sengon	Paraserianthes falcataria (L.) I.C.	Leguminosae / Fabaceae	0,33	4,1	4	5	0,04	0,00	2,39	0,00	0,03	0,10
1560	32	3	Banjaranyar	<200	Mahoni	Swietenia macrophylla King	Meliaceae	0,61	13,4	4	7	0,04	0,01	48,91	0,05	0,57	2,11
1561	32	3	Banjaranyar	<200	Kayu Gunung	Sp1	0	0,5	5,1	2	5	0,04	0,00	5,11	0,01	0,06	0,22
1562	33	2	Banjaranyar	<200	Tisuk	Hibiscus macrophyllus Roxb. ex I	Malvaceae	0,4	4,5	5	6	0,01	0,00	3,86	0,00	0,18	0,67
1563	33	2	Banjaranyar	<200	Tisuk	Hibiscus macrophyllus Roxb. ex I	Malvaceae	0,4	5,7	5	6	0,01	0,00	6,11	0,01	0,29	1,05
1564	33	2	Banjaranyar	<200	Tisuk	Hibiscus macrophyllus Roxb. ex I	Malvaceae	0,4	10,2	6	8	0,01	0,00	22,82	0,02	1,07	3,94
1565	33	2	Banjaranyar	<200	Sengon	Paraserianthes falcataria (L.) I.C.	Leguminosae / Fabaceae	0,33	11,1	9	11	0,01	0,00	30,18	0,03	1,42	5,21
1566	33	2	Banjaranyar	<200	Pangsor	Ficus callosa Willd.	Moraceae	0,33	10,2	4	7	0,01	0,00	16,93	0,02	0,80	2,92
1567	33	2	Banjaranyar	<200	Pangsor	Ficus callosa Willd.	Moraceae	0,33	10,5	4	7	0,01	0,00	17,91	0,02	0,84	3,09
1568	33	2	Banjaranyar	<200	Sengon	Paraserianthes falcataria (L.) I.C.	Leguminosae / Fabaceae	0,33	7,0	5	7	0,01	0,00	8,52	0,01	0,40	1,47
1569	33	2	Banjaranyar	<200	Tisuk	Hibiscus macrophyllus Roxb. ex I	Malvaceae	0,4	3,2	3	5	0,01	0,00	1,76	0,00	0,08	0,30
1570	33	2	Banjaranyar	<200	Tisuk	Hibiscus macrophyllus Roxb. ex I	Malvaceae	0,4	4,8	5	8	0,01	0,00	5,69	0,01	0,27	0,98
1571	33	2	Banjaranyar	<200	Angsana/Sonokembang	Pterocarpus indicus Willd.	Leguminosae / Fabaceae	0,65	9,5	6	8	0,01	0,00	31,63	0,03	1,49	5,46
1572	33	2	Banjaranyar	<200	Jati	Tectona grandis L.f.	Lamiaceae / Verbenaceae	0,7	13,7	4	7	0,01	0,00	57,92	0,06	2,72	9,99
1573	33	2	Banjaranyar	<200	Pisitan	Dysoxylum parasiticum (Osbeck	Meliaceae	0,6	12,7	4	7	0,01	0,00	44,05	0,04	2,07	7,60
1574	33	2	Banjaranyar	<200	Pangsor	Ficus callosa Willd.	Moraceae	0,33	4,8	3	4	0,01	0,00	2,53	0,00	0,12	0,44
1575	33	2	Banjaranyar	<200	Durian	Durio zibethinus L.	Malvaceae	0,57	16,5	4	8	0,01	0,01	76,82	0,08	3,61	13,25
1576	33	2	Banjaranyar	<200	Tisuk	Hibiscus macrophyllus Roxb. ex I	Malvaceae	0,4	17,8	7	11	0,01	0,01	85,15	0,09	4,00	14,69
1577	33	2	Banjaranyar	<200	Sengon	Paraserianthes falcataria (L.) I.C.	Leguminosae / Fabaceae	0,33	3,2	3	4	0,01	0,00	1,20	0,00	0,06	0,21
1578	33	2	Banjaranyar	<200	Sengon	Paraserianthes falcataria (L.) I.C.	Leguminosae / Fabaceae	0,33	5,7	7	8,5	0,01	0,00	7,05	0,01	0,33	1,22
1579	33	2	Banjaranyar	<200	Tisuk	Hibiscus macrophyllus Roxb. ex I	Malvaceae	0,4	4,8	3	4	0,01	0,00	3,02	0,00	0,14	0,52
1580	33	2	Banjaranyar	<200	Jati	Tectona grandis L.f.	Lamiaceae / Verbenaceae	0,7	12,7	5	9	0,01	0,00	63,87	0,06	3,00	11,02
1581	33	2	Banjaranyar	<200	Pisitan	Dysoxylum parasiticum (Osbeck	Meliaceae	0,6	3,8	1,8	3	0,01	0,00	2,23	0,00	0,10	0,39
1582	33	2	Banjaranyar	<200	Tisuk	Hibiscus macrophyllus Roxb. ex I	Malvaceae	0,4	3,8	3,5	5	0,01	0,00	2,46	0,00	0,12	0,42
1583	33	2	Banjaranyar	<200	Jati	Tectona grandis L.f.	Lamiaceae / Verbenaceae	0,7	16,5	6	8	0,04	0,02	92,72	0,09	1,09	4,00
1584	33	2	Banjaranyar	<200	Jati	Tectona grandis L.f.	Lamiaceae / Verbenaceae	0,7	11,5	3	8	0,04	0,01	47,27	0,05	0,56	2,04
1585	33	2	Banjaranyar	<200	Jati	Tectona grandis L.f.	Lamiaceae / Verbenaceae	0,7	11,8	3	8	0,04	0,01	49,71	0,05	0,58	2,14
1586	33	2	Banjaranyar	<200	Tisuk	Hibiscus macrophyllus Roxb. ex I	Malvaceae	0,4	15,9	7	9	0,04	0,02	57,57	0,06	0,68	2,48
1587	33	2	Banjaranyar	<200	Jati	Tectona grandis L.f.	Lamiaceae / Verbenaceae	0,7	11,1	3	7	0,04	0,01	39,73	0,04	0,47	1,71
1588	33	2	Banjaranyar	<200	Jati	Tectona grandis L.f.	Lamiaceae / Verbenaceae	0,7	12,7	5	9	0,04	0,01	63,87	0,06	0,75	2,75
1589	33	2	Banjaranyar	<200	Jati	Tectona grandis L.f.	Lamiaceae / Verbenaceae	0,7	14,6	6	11	0,04	0,02	99,16	0,10	1,17	4,28
1590	33	2	Banjaranyar	<200	Durian	Durio zibethinus L.	Malvaceae	0,57	16,9	8	11	0,04	0,02	106,49	0,11	1,25	4,59
1591	33	2	Banjaranyar	<200	Jati	Tectona grandis L.f.	Lamiaceae / Verbenaceae	0,7	16,2	3	9	0,04	0,02	99,67	0,10	1,17	4,30
1592	33	2	Banjaranyar	<200	Tisuk	Hibiscus macrophyllus Roxb. ex I	Malvaceae	0,4	7,6	8	11	0,04	0,00	18,03	0,02	0,21	0,78
1593	33	2	Banjaranyar	<200	Jati	Tectona grandis L.f.	Lamiaceae / Verbenaceae	0,7	15,6	7	10	0,04	0,02	102,02	0,10	1,20	4,40
1594	33	2	Banjaranyar	<200	Tisuk	Hibiscus macrophyllus Roxb. ex I	Malvaceae	0,4	7,0	6	9	0,04	0,00	12,79	0,01	0,15	0,55
1595	33	2	Banjaranyar	<200	Cangcaratan	Nauclea subdita (Korth.) Steud	Rubiaceae	0,32	16,9	7	10	0,04	0,02	57,51	0,06	0,68	2,48
1596	33	2	Banjaranyar	<200	Jati	Tectona grandis L.f.	Lamiaceae / Verbenaceae	0,7	15,6	6	9	0,04	0,02	92,63	0,09	1,09	3,99
1597	33	2	Banjaranyar	<200	Tisuk	Hibiscus macrophyllus Roxb. ex I	Malvaceae	0,4	8,0	7	10	0,04	0,00	17,81	0,02	0,21	0,77
1598	33	2	Banjaranyar	<200	Tisuk	Hibiscus macrophyllus Roxb. ex I	Malvaceae	0,4	6,4	7	10	0,04	0,00	11,83	0,01	0,14	0,51
1599	33	2	Banjaranyar	<200	Tisuk	Hibiscus macrophyllus Roxb. ex I	Malvaceae	0,4	6,7	7	8	0,04	0,00	10,55	0,01	0,12	0,45
1600	33	2	Banjaranyar	<200	Tisuk	Hibiscus macrophyllus Roxb. ex I	Malvaceae	0,4	8,0	7	9	0,04	0,00	16,17	0,02	0,19	0,70
1601	33	2	Banjaranyar	<200	Tisuk	Hibiscus macrophyllus Roxb. ex I	Malvaceae	0,4	6,0	6	7	0,04	0,00	7,77	0,01	0,09	0,34
1602	33	2	Banjaranyar	<200	Sengon	Paraserianthes falcataria (L.) I.C.	Leguminosae / Fabaceae	0,33	7,6	8	9	0,04	0,00	12,58	0,01	0,15	0,54
1603	33	2	Banjaranyar	<200	Tisuk	Hibiscus macrophyllus Roxb. ex I	Malvaceae	0,4	7,6	7	8	0,04	0,00	13,47	0,01	0,16	0,58
1604	33	2	Banjaranyar	<200	Sengon	Paraserianthes falcataria (L.) I.C.	Leguminosae / Fabaceae	0,33	8,0	5	8	0,04	0,00	12,17	0,01	0,14	0,52
1605	33	2	Banjaranyar	<200	Sengon	Paraserianthes falcataria (L.) I.C.	Leguminosae / Fabaceae	0,33	9,5	10	11	0,04	0,01	22,76	0,02	0,27	0,98
1606	33	2	Banjaranyar	<200	Jati	Tectona grandis L.f.	Lamiaceae / Verbenaceae	0,7	6,7	3	6	0,04	0,00	13,53	0,01	0,16	0,58

1607	33	2	Banjaranyar	<200	Sengon	Paraserianthes falcataria (L.) I.C.	Leguminosae / Fabaceae	0,33	10,8	9	12	0,04	0,01	30,99	0,03	0,36	1,34
1608	33	2	Banjaranyar	<200	Durian	Durio zibethinus L.	Malvaceae	0,57	11,1	7	11	0,04	0,01	49,79	0,05	0,59	2,15
1609	33	2	Banjaranyar	<200	Sengon	Paraserianthes falcataria (L.) I.C.	Leguminosae / Fabaceae	0,33	7,3	8	10	0,04	0,00	12,82	0,01	0,15	0,55
1610	33	2	Banjaranyar	<200	Sengon	Paraserianthes falcataria (L.) I.C.	Leguminosae / Fabaceae	0,33	15,9	10	14	0,04	0,02	72,35	0,07	0,85	3,12
1611	33	2	Banjaranyar	<200	Jati	Tectona grandis L.f.	Lamiaceae / Verbenaceae	0,7	7,3	3	7	0,04	0,00	18,41	0,02	0,22	0,79
1612	33	2	Banjaranyar	<200	Pisitan	Dysoxylum parasiticum (Osbeck	Meliaceae	0,6	17,2	6	11	0,04	0,02	115,50	0,12	1,36	4,98
1613	33	2	Banjaranyar	<200	Pangsor	Ficus callosa Willd.	Moraceae	0,33	8,3	6	8	0,04	0,01	13,08	0,01	0,15	0,56
1614	33	2	Banjaranyar	<200	kelapa	Cocos nucifera L.	Arecaceae	0,5	28,6	10	14	0,04	0,06	112,30	0,11	1,32	4,84
1615	33	2	Banjaranyar	<200	Jati	Tectona grandis L.f.	Lamiaceae / Verbenaceae	0,7	10,2	3	6	0,04	0,01	29,27	0,03	0,34	1,26
1616	33	2	Banjaranyar	<200	Sengon	Paraserianthes falcataria (L.) I.C.	Leguminosae / Fabaceae	0,33	15,0	11	13	0,04	0,02	60,36	0,06	0,71	2,60
1617	33	2	Banjaranyar	<200	Sengon	Paraserianthes falcataria (L.) I.C.	Leguminosae / Fabaceae	0,33	20,7	10	14	0,04	0,03	117,00	0,12	1,37	5,05
1618	33	2	Banjaranyar	<200	mahoni	Swietenia macrophylla King	Meliaceae	0,61	15,3	4	8	0,04	0,02	70,59	0,07	0,83	3,04
1619	33	2	Banjaranyar	<200	Pisitan	Dysoxylum parasiticum (Osbeck	Meliaceae	0,6	16,5	5	8	0,04	0,02	80,51	0,08	0,95	3,47
1620	33	2	Banjaranyar	<200	mahoni	Swietenia macrophylla King	Meliaceae	0,61	14,3	6	9	0,04	0,02	69,86	0,07	0,82	3,01
1621	33	2	Banjaranyar	<200	Pisitan	Dysoxylum parasiticum (Osbeck	Meliaceae	0,6	16,2	7	11	0,04	0,02	104,01	0,10	1,22	4,49
1622	33	2	Banjaranyar	<200	kelapa	Cocos nucifera L.	Arecaceae	0,5	26,7	10	14	0,04	0,06	112,30	0,11	1,32	4,84
1623	33	2	Banjaranyar	<200	mahoni	Swietenia macrophylla King	Meliaceae	0,61	59,2	11	16	0,04	0,28	1.592,95	1,59	18,72	68,69
1624	33	2	Banjaranyar	<200	Pisitan	Dysoxylum parasiticum (Osbeck	Meliaceae	0,6	23,9	4	10	0,04	0,04	193,20	0,19	2,27	8,33
1625	33	2	Banjaranyar	<200	Jati	Tectona grandis L.f.	Lamiaceae / Verbenaceae	0,7	7,0	3	6	0,04	0,00	14,73	0,01	0,17	0,64
1626	33	2	Banjaranyar	<200	Jati	Tectona grandis L.f.	Lamiaceae / Verbenaceae	0,7	7,6	3	5	0,04	0,00	14,62	0,01	0,17	0,63
1627	34	1	Banjaranyar	<200	Jati	Tectona grandis L.f.	Lamiaceae / Verbenaceae	0,7	8,6	4	9	0,04	0,01	31,09	0,03	0,37	1,34
1628	34	1	Banjaranyar	<200	Jati	Tectona grandis L.f.	Lamiaceae / Verbenaceae	0,7	13,4	6	12	0,04	0,01	90,90	0,09	1,07	3,92
1629	34	1	Banjaranyar	<200	Jati	Tectona grandis L.f.	Lamiaceae / Verbenaceae	0,7	24,8	8	13	0,04	0,05	304,03	0,30	3,57	13,11
1630	34	1	Banjaranyar	<200	Jati	Tectona grandis L.f.	Lamiaceae / Verbenaceae	0,7	19,1	5	10	0,04	0,03	147,84	0,15	1,74	6,38
1631	34	1	Banjaranyar	<200	Jati	Tectona grandis L.f.	Lamiaceae / Verbenaceae	0,7	8,0	6	7	0,04	0,00	21,45	0,02	0,25	0,92
1632	34	1	Banjaranyar	<200	Jati	Tectona grandis L.f.	Lamiaceae / Verbenaceae	0,7	21,3	8	13	0,04	0,04	230,13	0,23	2,70	9,92
1633	34	1	Banjaranyar	<200	Jati	Tectona grandis L.f.	Lamiaceae / Verbenaceae	0,7	19,1	8	12	0,04	0,03	174,72	0,17	2,05	7,53
1634	34	1	Banjaranyar	<200	Mahoni	Swietenia macrophylla King	Meliaceae	0,61	4,5	3	4	0,04	0,00	3,91	0,00	0,05	0,17
1635	34	1	Banjaranyar	<200	Jati	Tectona grandis L.f.	Lamiaceae / Verbenaceae	0,7	8,0	2,5	3,5	0,04	0,00	11,37	0,01	0,13	0,49
1636	34	1	Banjaranyar	<200	Jati	Tectona grandis L.f.	Lamiaceae / Verbenaceae	0,7	6,4	2	53	0,04	0,00	91,02	0,09	1,07	3,93
1637	34	1	Banjaranyar	<200	Jati	Tectona grandis L.f.	Lamiaceae / Verbenaceae	0,7	8,0	2	3	0,04	0,00	9,87	0,01	0,12	0,43
1638	34	1	Banjaranyar	<200	Jati	Tectona grandis L.f.	Lamiaceae / Verbenaceae	0,7	7,6	2	4	0,04	0,00	11,92	0,01	0,14	0,51
1639	34	1	Banjaranyar	<200	Jati	Tectona grandis L.f.	Lamiaceae / Verbenaceae	0,7	15,3	7	11	0,04	0,02	107,20	0,11	1,26	4,62
1640	34	1	Banjaranyar	<200	Jati	Tectona grandis L.f.	Lamiaceae / Verbenaceae	0,7	19,1	5	11	0,04	0,03	161,33	0,16	1,90	6,96
1641	34	1	Banjaranyar	<200	Jati	Tectona grandis L.f.	Lamiaceae / Verbenaceae	0,7	14,0	6	11	0,04	0,02	91,40	0,09	1,07	3,94
1642	34	1	Banjaranyar	<200	Jati	Tectona grandis L.f.	Lamiaceae / Verbenaceae	0,7	7,6	4	5	0,04	0,00	14,62	0,01	0,17	0,63
1643	34	1	Banjaranyar	<200	Jati	Tectona grandis L.f.	Lamiaceae / Verbenaceae	0,7	15,3	6	13	0,04	0,02	124,92	0,12	1,47	5,39
1644	34	1	Banjaranyar	<200	Jati	Tectona grandis L.f.	Lamiaceae / Verbenaceae	0,7	9,5	2,5	6,5	0,04	0,01	27,99	0,03	0,33	1,21
1645	34	1	Banjaranyar	<200	Jati	Tectona grandis L.f.	Lamiaceae / Verbenaceae	0,7	9,9	2	2,5	0,04	0,01	12,39	0,01	0,15	0,53
1646	34	1	Banjaranyar	<200	Jati	Tectona grandis L.f.	Lamiaceae / Verbenaceae	0,7	5,7	4	5	0,04	0,00	8,63	0,01	0,10	0,37
1647	34	1	Banjaranyar	<200	Jati	Tectona grandis L.f.	Lamiaceae / Verbenaceae	0,7	11,5	2	2,5	0,04	0,01	16,29	0,02	0,19	0,70
1648	34	1	Banjaranyar	<200	Jati	Tectona grandis L.f.	Lamiaceae / Verbenaceae	0,7	3,8	2	3	0,04	0,00	2,57	0,00	0,03	0,11
1649	34	1	Banjaranyar	<200	Jati	Tectona grandis L.f.	Lamiaceae / Verbenaceae	0,7	4,5	2	5	0,04	0,00	5,45	0,01	0,06	0,23
1650	34	1	Banjaranyar	<200	Jati	Tectona grandis L.f.	Lamiaceae / Verbenaceae	0,7	3,2	2	2,5	0,04	0,00	1,56	0,00	0,02	0,07
1651	34	1	Banjaranyar	<200	Jati	Tectona grandis L.f.	Lamiaceae / Verbenaceae	0,7	11,5	3,5	6	0,04	0,01	36,32	0,04	0,43	1,57
1652	34	1	Banjaranyar	<200	Jati	Tectona grandis L.f.	Lamiaceae / Verbenaceae	0,7	15,6	5	11	0,04	0,02	111,32	0,11	1,31	4,80
1653	34	1	Banjaranyar	<200	Jati	Tectona grandis L.f.	Lamiaceae / Verbenaceae	0,7	20,4	8	12	0,04	0,03	196,64	0,20	2,31	8,48
1654	34	1	Banjaranyar	<200	Jati	Tectona grandis L.f.	Lamiaceae / Verbenaceae	0,7	7,0	2,5	4	0,04	0,00	10,16	0,01	0,12	0,44
1655	34	1	Banjaranyar	<200	Jati	Tectona grandis L.f.	Lamiaceae / Verbenaceae	0,7	11,8	7	10	0,04	0,01	60,98	0,06	0,72	2,63
1656	34	1	Banjaranyar	<200	Jati	Tectona grandis L.f.	Lamiaceae / Verbenaceae	0,7	17,8	5	9	0,04	0,02	118,30	0,12	1,39	5,10
1657	34	1	Banjaranyar	<200	Jati	Tectona grandis L.f.	Lamiaceae / Verbenaceae	0,7	11,5	5	8	0,04	0,01	47,27	0,05	0,56	2,04
1658	34	1	Banjaranyar	<200	Sengon	Paraserianthes falcataria (L.) I.C.	Leguminosae / Fabaceae	0,33	4,8	5	7	0,04	0,00	4,22	0,00	0,05	0,18
1659	34	1	Banjaranyar	<200	Jeungjing	Albizia falcata (L.) Backer	Leguminosae / Fabaceae	0,37	9,2	12	16	0,04	0,01	33,47	0,03	0,39	1,44
1660	34	1	Banjaranyar	<200	Pala	Myristica fragrans Houtt.	Myristicaceae	0,5	3,5	1	2,5	0,04	0,00	1,36	0,00	0,02	0,06
1661	34	1	Banjaranyar	<200	Jati	Tectona grandis L.f.	Lamiaceae / Verbenaceae	0,7	14,6	5	10	0,04	0,02	90,87	0,09	1,07	3,92
1662	34	1	Banjaranyar	<200	Jati	Tectona grandis L.f.	Lamiaceae / Verbenaceae	0,7	14,3	4	6	0,04	0,02	54,66	0,05	0,64	2,36
1663	34	1	Banjaranyar	<200	Mahoni	Swietenia macrophylla King	Meliaceae	0,61	20,4	6	12	0,04	0,03	173,35	0,17	2,04	7,48
1664	34	1	Banjaranyar	<200	Jati	Tectona grandis L.f.	Lamiaceae / Verbenaceae	0,7	6,7	3	7	0,04	0,00	15,58	0,02	0,18	0,67
1665	34	1	Banjaranyar	<200	Jati	Tectona grandis L.f.	Lamiaceae / Verbenaceae	0,7	13,4	8	10	0,04	0,01	76,92	0,08	0,90	3,32
1666	34	1	Banjaranyar	<200	Jati	Tectona grandis L.f.	Lamiaceae / Verbenaceae	0,7	12,1	5	10	0,04	0,01	64,03	0,06	0,75	2,76
1667	34	1	Banjaranyar	<200	Sengon	Paraserianthes falcataria (L.) I.C.	Leguminosae / Fabaceae	0,33	5,1	7	9	0,04	0,00	5,99	0,01	0,07	0,26
1668	34	1	Banjaranyar	<200	Jati	Tectona grandis L.f.	Lamiaceae / Verbenaceae	0,7	4,5	2	5	0,04	0,00	5,45	0,01	0,06	0,23
1669	34	1	Banjaranyar	<200	Sengon	Paraserianthes falcataria (L.) I.C.	Leguminosae / Fabaceae	0,33	8,6	7	12	0,04	0,01	20,32	0,02	0,24	0,88
1670	34	1	Banjaranyar	<200	Pala	Myristica fragrans Houtt.	Myristicaceae	0,5	4,8	0,5	3	0,04	0,00	2,84	0,00	0,03	0,12
1671	34	1	Banjaranyar	<200	Jati	Tectona grandis L.f.	Lamiaceae / Verbenaceae	0,7	17,8	8	12	0,04	0,02	153,97	0,15	1,81	6,64
1672	34	1	Banjaranyar	<200	Jati	Tectona grandis L.f.	Lamiaceae / Verbenaceae	0,7	18,1	8	13	0,04	0,03	171,14	0,17	2,01	7,38
1673	34	1	Banjaranyar	<200	Jati	Tectona grandis L.f.	Lamiaceae / Verbenaceae	0,7	17,5	7	11	0,04	0,02	137,56	0,14	1,62	5,93
1674	35	1	Banjaranyar	<200	Jati	Tectona grandis L.f.	Lamiaceae / Verbenaceae	0,7	10,5	6	13	0,04	0,01	62,88	0,06	0,74	2,71
1675	35	1	Banjaranyar	<200	Jati	Tectona grandis L.f.	Lamiaceae / Verbenaceae	0,7	19,1	10	15	0,04	0,03	214,34	0,21	2,52	9,24
1676	35	1	Banjaranyar	<200	Jati	Tectona grandis L.f.	Lamiaceae / Verbenaceae	0,7	16,5	11	16	0,04	0,02	174,95	0,17	2,06	7,54
1677	35	1	Banjaranyar	<200	Jati	Tectona grandis L.f.	Lamiaceae / Verbenaceae	0,7	18,8	9	15	0,04	0,03	207,84	0,21	2,44	8,96
1678	35	1	Banjaranyar	<200	Jati	Tectona grandis L.f.	Lamiaceae / Verbenaceae	0,7	14,3	9	14	0,04	0,02	118,79	0,12	1,40	5,12
1679	35	1	Banjaranyar	<200	Jati	Tectona grandis L.f.	Lamiaceae / Verbenaceae	0,7	17,2	9	14	0,04	0,02	165,89	0,17	1,95	7,15
1680	35	1	Banjaranyar	<200	Jati	Tectona grandis L.f.	Lamiaceae / Verbenaceae	0,7	13,4	10	14	0,04	0,01	104,68	0,10	1,23	4,51
1681	35	1	Banjaranyar	<200	Jati	Tectona grandis L.f.	Lamiaceae / Verbenaceae	0,7	6,4	2	3	0,04	0,00	6,56	0,01	0,08	0,28
1682	35	1	Banjaranyar	<200	Jati	Tectona grandis L.f.	Lamiaceae / Verbenaceae	0,7	5,1	9	15	0,04	0,00	19,03	0,02	0,22	0,82
1683	35	1	Banjaranyar	<200	Jati	Tectona grandis L.f.	Lamiaceae / Verbenaceae	0,7	20,7	10	15	0,04	0,03	248,19	0,25	2,92	10,70
1684	35	1	Banjaranyar	<200	Jati	Tectona grandis L.f.	Lamiaceae / Verbenaceae	0,7	16,5	8	15	0,04	0,02	164,91	0,16	1,94	7,11
1685	35	1	Banjaranyar	<200	Jati	Tectona grandis L.f.	Lamiaceae / Verbenaceae	0,7	18,5	11	15	0,04	0,03	201,43	0,20	2,37	8,69
1686	35	1	Banjaranyar	<200	Jati	Tectona grandis L.f.	Lamiaceae / Verbenaceae	0,7	15,3	11	14	0,04	0,02	133,70	0,13	1,57	5,77
1687	35	1	Banjaranyar	<200	Jati	Tectona grandis L.f.	Lamiaceae / Verbenaceae	0,7	17,2	8	14	0,04	0,02	165,89	0,17	1,95	7,15
1688	35	1	Banjaranyar	<200	Jati	Tectona grandis L.f.	Lamiaceae / Verbenaceae	0,7	19,7	9	15	0,04	0,03	227,61	0,23	2,67	9,82
1689	35	1	Banjaranyar	<200	Jati	Tectona grandis L.f.	Lamiaceae / Verbenaceae	0,7	11,1	9	14	0,04	0,01	74,96	0,07	0,88	3,23
1690	35	1	Banjaranyar	<200	Jati	Tectona grandis L.f.	Lamiaceae / Verbenaceae	0,7	11,8	6	12	0,04	0,01	72,06	0,07	0,85	3,11
1691	35	1	Banjaranyar	<200	Jati	Tectona grandis L.f.	Lamiaceae / Verbenaceae	0,7	15,9	7	14	0,04	0,02	144,08	0,14	1,69	6,21
1692	35	1	Banjaranyar	<200	Jati	Tectona grandis L.f.	Lamiaceae / Verbenaceae	0,7	17,2	8	15	0,04	0,02	176,72	0,18	2,08	7,62
1693	35	1	Banjaranyar	<200	Jati	Tectona grandis L.f.	Lamiaceae / Verbenaceae	0,7	15,6	7	15	0,04	0,02	147,90	0,15	1,74	6,38
1694	35	1	Banjaranyar	<200	Jati	Tectona grandis L.f.	Lamiaceae / Verbenaceae	0,7	14,3	8	14	0,04	0,02	118,79	0,12	1,40	5,12
1695	35	1	Banjaranyar	<200	Jati	Tectona grandis L.f.	Lamiaceae / Verbenaceae	0,7	16,9	8	15	0,04	0,02	170,77	0,17	2,01	7,36
1696	35	1	Banjaranyar	<200	Jati	Tectona grandis L.f.	Lamiaceae / Verbenaceae	0,7	18,8	9	15	0,04	0,03	207,84	0,21	2,44	8,96
1697	35	1	Banjaranyar	<200	Jati	Tectona grandis L.f.	Lamiaceae / Verbenaceae	0,7	17,5	10	14	0,04	0,02	171,56	0,17	2,02	7,40
1698	35	1	Banjaranyar	<200	Jati	Tectona grandis L.f.	Lamiaceae / Verbenaceae	0,7	18,1	10	16	0,04	0,03	207,00	0,21	2,43	8,93
1699	35	1	Banjaranyar	<200	Jati	Tectona grandis L.f.	Lamiaceae / Verbenaceae	0,7	17,5	10	16	0,04	0,02	193,89	0,19	2,28	8,36
1700	35	1	Banjaranyar	<200	Jati	Tectona grandis L.f.	Lamiaceae / Verbenaceae	0,7	15,6	11	15	0,04	0,02	147,90	0,15	1,74	6,38
1701	35	1	Banjaranyar	<200	Jati	Tectona grandis L.f.	Lamiaceae / Verbenaceae	0,7	15,0	10	15	0,04	0,02	137,03	0,14	1,61	5,91
1702	35	1	Banjaranyar	<200	Jati	Tectona grandis L.f.	Lamiaceae / Verbenaceae	0,7	19,4	10	16	0,04	0,03	234,38	0,23	2,75	10,11
1703	35	1	Banjaranyar	<200	Jati	Tectona grandis L.f.	Lamiaceae / Verbenaceae	0,7	20,0	10	16	0,04	0,03	248,65	0,25	2,92	10,72
1704	35	1	Banjaranyar	<200	Jati	Tectona grandis L.f.	Lamiaceae / Verbenaceae	0,7	18,5	7	15	0,04	0,03	201,43	0,20	2,37	8,69
1705	35	1	Banjaranyar	<200	Jati	Tectona grandis L.f.	Lamiaceae / Verbenaceae	0,7	8,0	6	10	0,04	0,00	29,73	0,03	0,35	1,28
1706	35	1	Banjaranyar	<200	Jati	Tectona grandis L.f.	Lamiaceae / Verbenaceae	0,7	15,0	10	14	0,04	0,02	128,64	0,13	1,51	5,55
1707	35	1	Banjaranyar	<200	Jati	Tectona grandis L.f.	Lamiaceae / Verbenaceae	0,7	16,2	7	15	0,04	0,02	159,15	0,16	1,87	6,86
1708	35	1	Banjaranyar	<200	Jati	Tectona grandis L.f.	Lamiaceae / Verbenaceae	0,7	8,3	3	8	0,04	0,01	26,04	0,03	0,31	1,12
1709	35	1	Banjaranyar	<200	Jati	Tectona grandis L.f.	Lamiaceae / Verbenaceae	0,7	21,0	10	16	0,04	0,03	270,77	0,27	3,18	11,68
1710	35	1	Banjaranyar	<200	Jati	Tectona grandis L.f.	Lamiaceae / Verbenaceae	0,7	14,3	9	15	0,04	0,02	126,54	0,13	1,49	5,46
1711	35	1	Banjaranyar	<200	Jati	Tectona grandis L.f.	Lamiaceae / Verbenaceae	0,7	17,5	10	17	0,04	0,02	204,96	0,20	2,41	8,84
1712	35	1	Banjaranyar	<200	Jati	Tectona grandis L.f.	Lamiaceae / Verbenaceae	0,7	19,7	11	17	0,04	0,03	255,26	0,26	3,00	11,01
1713	35	1	Banjaranyar	<200	Jati	Tectona grandis L.f.	Lamiaceae / Verbenaceae	0,7	18,5	10	17	0,04	0,03	225,90	0,23	2,65	9,74
1714	35	1	Banjaranyar	<200	Jati	Tectona grandis L.f.	Lamiaceae / Verbenaceae	0,7	19,1	10	17	0,04	0,03	240,38	0,24	2,82	10,37
1715	35	1	Banjaranyar	<200	Jati	Tectona grandis L.f.	Lamiaceae / Verbenaceae	0,7	14,0	10	17	0,04	0,02	136,18	0,14	1,60	5,87
1716	35	1	Banjaranyar	<200	Jati	Tectona grandis L.f.	Lamiaceae / Verbenaceae	0,7	15,3	9	17	0,04	0,02	159,72	0,16	1,88	6,89
1717	35	1	Banjaranyar	<200	Jati	Tectona grandis L.f.	Lamiaceae / Verbenaceae	0,7	15,6	10	17	0,04	0,02	165,87	0,17	1,95	7,15
1718	35	1	Banjaranyar	<200	Jati	Tectona grandis L.f.	Lamiaceae / Verbenaceae	0,7	20,0	10	17	0,04	0,03	262,85	0,26	3,09	11,33
1719	35	1	Banjaranyar	<200	Jati	Tectona grandis L.f.	Lamiaceae / Verbenaceae	0,7	18,5	13	16	0,04	0,03	213,70	0,21	2,51	9,22
1720	35	1	Banjaranyar	<200	Jati	Tectona grandis L.f.	Lamiaceae / Verbenaceae	0,7	18,1	12	16	0,04	0,03	207,00	0,21	2,43	8,93
1721	35	1	Banjaranyar	<200	Jati	Tectona grandis L.f.	Lamiaceae / Verbenaceae	0,7	12,7	4	14	0,04	0,01	95,73	0,10	1,12	4,13
1722	35	1	Banjaranyar	<200	Jati	Tectona grandis L.f.	Lamiaceae / Verbenaceae	0,7	14,0	13	16	0,04	0,02	128,83	0,13	1,51	5,56
1723	35	1	Banjaranyar	<200	Jati	Tectona grandis L.f.	Lamiaceae / Verbenaceae	0,7	18,8	7	16	0,04	0,03	220,50	0,22	2,59	9,51
1724	35	1	Banjaranyar	<200	Jati	Tectona grandis L.f.	Lamiaceae / Verbenaceae	0,7	10,5	7	13	0,04	0,01	62,88	0,06	0,74	2,71
1725	35	1	Banjaranyar	<200	Jati	Tectona grandis L.f.	Lamiaceae / Verbenaceae	0,7	8,6	6	9	0,04	0,01	31,09	0,03	0,37	1,34
1726	35	1	Banjaranyar	<200	Jati	Tectona grandis L.f.	Lamiaceae / Verbenaceae	0,7	18,1	10	16	0,04	0,03	207,00	0,21	2,43	8,93
1727	35	1	Banjaranyar	<200	Jati	Tectona grandis L.f.	Lamiaceae / Verbenaceae	0,7	19,4	10	17	0,04	0,03	247,77	0,25	2,91	10,68
1728	35	1	Banjaranyar	<200	Jati	Tectona grandis L.f.	Lamiaceae / Verbenaceae	0,7	18,8	9	17	0,04	0,03	233,09	0,23	2,74	10,05
1729	35	1	Banjaranyar	<200	Jati	Tectona grandis L.f.	Lamiaceae / Verbenaceae	0,7	17,8	9	16	0,04	0,02	200,39	0,20	2,35	8,64
1730	35	1	Banjaranyar	<200	Jati	Tectona grandis L.f.	Lamiaceae / Verbenaceae	0,7	22,0	10	17	0,04	0,04	310,52	0,31	3,65	13,39

1731	35	1	Banjaranyar	<200	Jati	Tectona grandis L.f.	Lamiaceae / Verbenaceae	0,7	15,6	9	16	0,04	0,02	156,91	0,16	1,84	6,77
1732	35	1	Banjaranyar	<200	Jati	Tectona grandis L.f.	Lamiaceae / Verbenaceae	0,7	20,7	10	17	0,04	0,03	278,34	0,28	3,27	12,00
1733	35	1	Banjaranyar	<200	Jati	Tectona grandis L.f.	Lamiaceae / Verbenaceae	0,7	17,8	9	16	0,04	0,02	200,39	0,20	2,35	8,64
1734	35	1	Banjaranyar	<200	Jati	Tectona grandis L.f.	Lamiaceae / Verbenaceae	0,7	16,9	10	16	0,04	0,02	181,17	0,18	2,13	7,81
1735	35	1	Banjaranyar	<200	Jati	Tectona grandis L.f.	Lamiaceae / Verbenaceae	0,7	10,5	6	13	0,04	0,01	62,88	0,06	0,74	2,71
1736	35	1	Banjaranyar	<200	Jati	Tectona grandis L.f.	Lamiaceae / Verbenaceae	0,7	14,6	8	14	0,04	0,02	123,67	0,12	1,45	5,33
1737	35	1	Banjaranyar	<200	Jati	Tectona grandis L.f.	Lamiaceae / Verbenaceae	0,7	17,5	10	16	0,04	0,02	193,89	0,19	2,28	8,36
1738	36	2	Banjaranyar	<200	Jati	Tectona grandis L.f.	Lamiaceae / Verbenaceae	0,7	10,8	8	11	0,01	0,00	56,99	0,06	2,68	9,83
1739	36	2	Banjaranyar	<200	Sengon	Paraserianthes falcataria (L.) I.C.	Leguminosae / Fabaceae	0,33	12,7	11	14	0,01	0,00	48,07	0,05	2,26	8,29
1740	36	2	Banjaranyar	<200	Jati	Tectona grandis L.f.	Lamiaceae / Verbenaceae	0,7	8,6	9	11	0,01	0,00	37,36	0,04	1,76	6,44
1741	36	2	Banjaranyar	<200	Sengon	Paraserianthes falcataria (L.) I.C.	Leguminosae / Fabaceae	0,33	7,0	10	12	0,01	0,00	13,96	0,01	0,66	2,41
1742	36	2	Banjaranyar	<200	Jati	Tectona grandis L.f.	Lamiaceae / Verbenaceae	0,7	10,5	9	12	0,01	0,00	58,44	0,06	2,75	10,08
1743	36	2	Banjaranyar	<200	Tisuk	Hibiscus macrophyllus Roxb. ex I	Malvaceae	0,4	5,4	5	9	0,01	0,00	7,98	0,01	0,37	1,38
1744	36	2	Banjaranyar	<200	Jati	Tectona grandis L.f.	Lamiaceae / Verbenaceae	0,7	8,9	5	9	0,01	0,00	33,23	0,03	1,56	5,73
1745	36	2	Banjaranyar	<200	Nangka	Artocarpus heterophyllus Lam.	Moraceae	0,61	9,2	5	8	0,01	0,00	28,04	0,03	1,32	4,84
1746	36	2	Banjaranyar	<200	Pangsor	Ficus callosa Willd.	Moraceae	0,33	3,8	4	5	0,01	0,00	2,06	0,00	0,10	0,36
1747	36	2	Banjaranyar	<200	Akasia mangium	Acacia mangium Willd.	Leguminosae / Fabaceae	0,5	8,3	12	14	0,01	0,00	31,95	0,03	1,50	5,51
1748	36	2	Banjaranyar	<200	Sengon	Paraserianthes falcataria (L.) I.C.	Leguminosae / Fabaceae	0,33	12,7	10	12	0,01	0,00	41,74	0,04	1,96	7,20
1749	36	2	Banjaranyar	<200	Tisuk	Hibiscus macrophyllus Roxb. ex I	Malvaceae	0,4	3,5	5	6	0,01	0,00	2,48	0,00	0,12	0,43
1750	36	2	Banjaranyar	<200	Pisitan	Dysoxylum parasiticum (Osbeck	Meliaceae	0,6	6,4	3	6	0,01	0,00	10,74	0,01	0,50	1,85
1751	36	2	Banjaranyar	<200	Jati	Tectona grandis L.f.	Lamiaceae / Verbenaceae	0,7	9,2	7	10	0,01	0,00	39,02	0,04	1,83	6,73
1752	36	2	Banjaranyar	<200	Jati	Tectona grandis L.f.	Lamiaceae / Verbenaceae	0,7	9,5	6	10	0,01	0,00	41,53	0,04	1,95	7,16
1753	36	2	Banjaranyar	<200	Jati	Tectona grandis L.f.	Lamiaceae / Verbenaceae	0,7	13,0	9	12	0,01	0,00	86,97	0,09	4,09	15,00
1754	36	2	Banjaranyar	<200	Jati	Tectona grandis L.f.	Lamiaceae / Verbenaceae	0,7	12,7	9	12	0,01	0,00	83,13	0,08	3,91	14,34
1755	36	2	Banjaranyar	<200	Jati	Tectona grandis L.f.	Lamiaceae / Verbenaceae	0,7	10,5	8	11	0,01	0,00	53,96	0,05	2,54	9,31
1756	36	2	Banjaranyar	<200	Jati	Tectona grandis L.f.	Lamiaceae / Verbenaceae	0,7	10,2	6	12	0,01	0,00	55,23	0,06	2,60	9,53
1757	36	2	Banjaranyar	<200	Jati	Tectona grandis L.f.	Lamiaceae / Verbenaceae	0,7	8,0	6	8	0,01	0,00	24,24	0,02	1,14	4,18
1758	36	2	Banjaranyar	<200	Jati	Tectona grandis L.f.	Lamiaceae / Verbenaceae	0,7	7,6	6	9	0,01	0,00	25,05	0,03	1,18	4,32
1759	36	2	Banjaranyar	<200	Kelapa	Cocos nucifera L.	Arecaceae	0,5	23,5	9	14	0,01	0,01	112,30	0,11	5,28	19,37
1760	36	2	Banjaranyar	<200	Heuras	Vitex pubescens Vahl	Lamiaceae / Verbenaceae	0,88	5,7	5	8	0,01	0,00	16,37	0,02	0,77	2,82
1761	36	2	Banjaranyar	<200	Jati	Tectona grandis L.f.	Lamiaceae / Verbenaceae	0,7	7,3	7	10	0,01	0,00	25,52	0,03	1,20	4,40
1762	36	2	Banjaranyar	<200	Tisuk	Hibiscus macrophyllus Roxb. ex I	Malvaceae	0,4	4,8	7	8	0,01	0,00	5,69	0,01	0,27	0,98
1763	36	2	Banjaranyar	<200	Jati	Tectona grandis L.f.	Lamiaceae / Verbenaceae	0,7	14,0	7	12	0,01	0,00	98,98	0,10	4,65	17,07
1764	36	2	Banjaranyar	<200	Jati	Tectona grandis L.f.	Lamiaceae / Verbenaceae	0,7	8,3	5	9	0,01	0,00	29,01	0,03	1,36	5,00
1765	36	2	Banjaranyar	<200	Jati	Tectona grandis L.f.	Lamiaceae / Verbenaceae	0,7	7,3	6	9	0,01	0,00	23,17	0,02	1,09	4,00
1766	36	2	Banjaranyar	<200	Jati	Tectona grandis L.f.	Lamiaceae / Verbenaceae	0,7	8,0	7	9	0,01	0,00	27,00	0,03	1,27	4,66
1767	36	2	Banjaranyar	<200	Jati	Tectona grandis L.f.	Lamiaceae / Verbenaceae	0,7	9,5	9	12	0,01	0,00	49,07	0,05	2,31	8,46
1768	36	2	Banjaranyar	<200	Sengon	Paraserianthes falcataria (L.) I.C.	Leguminosae / Fabaceae	0,33	21,6	15	19	0,04	0,04	168,10	0,17	1,98	7,25
1769	36	2	Banjaranyar	<200	Sengon	Paraserianthes falcataria (L.) I.C.	Leguminosae / Fabaceae	0,33	48,4	13	19	0,04	0,18	733,76	0,73	8,62	31,64
1770	36	2	Banjaranyar	<200	Sengon	Paraserianthes falcataria (L.) I.C.	Leguminosae / Fabaceae	0,33	27,0	14	19	0,04	0,06	252,99	0,25	2,97	10,91
1771	36	2	Banjaranyar	<200	Salam	Syzygium polyanthum (Wight) W.	Myrtaceae	0,66	29,3	9	16	0,04	0,07	471,47	0,47	5,54	20,33
1772	36	2	Banjaranyar	<200	Mahoni	Swietenia macrophylla King	Meliaceae	0,61	33,4	10	17	0,04	0,09	590,73	0,59	6,94	25,47
1773	37	1	Banjaranyar	<200	Sengon	Paraserianthes falcataria (L.) I.C.	Leguminosae / Fabaceae	0,33	10,5	9	12	0,01	0,00	29,34	0,03	1,38	5,06
1774	37	1	Banjaranyar	<200	Sengon	Paraserianthes falcataria (L.) I.C.	Leguminosae / Fabaceae	0,33	17,2	13	16	0,01	0,01	94,15	0,09	4,42	16,24
1775	37	1	Banjaranyar	<200	Sengon	Paraserianthes falcataria (L.) I.C.	Leguminosae / Fabaceae	0,33	16,2	13	16	0,01	0,01	84,79	0,08	3,98	14,62
1776	37	1	Banjaranyar	<200	Sengon	Paraserianthes falcataria (L.) I.C.	Leguminosae / Fabaceae	0,33	12,7	12	15	0,01	0,00	51,21	0,05	2,41	8,83
1777	37	1	Banjaranyar	<200	Sengon	Paraserianthes falcataria (L.) I.C.	Leguminosae / Fabaceae	0,33	9,2	9	12	0,01	0,00	23,16	0,02	1,09	3,99
1778	37	1	Banjaranyar	<200	Sengon	Paraserianthes falcataria (L.) I.C.	Leguminosae / Fabaceae	0,33	13,4	10	13	0,01	0,00	49,12	0,05	2,31	8,47
1779	37	1	Banjaranyar	<200	Sengon	Paraserianthes falcataria (L.) I.C.	Leguminosae / Fabaceae	0,33	16,9	14	17	0,01	0,01	96,17	0,10	4,52	16,59
1780	37	1	Banjaranyar	<200	Sengon	Paraserianthes falcataria (L.) I.C.	Leguminosae / Fabaceae	0,33	10,8	10	15	0,01	0,00	38,02	0,04	1,79	6,56
1781	37	1	Banjaranyar	<200	Sengon	Paraserianthes falcataria (L.) I.C.	Leguminosae / Fabaceae	0,33	15,3	11	14	0,01	0,00	67,14	0,07	3,16	11,58
1782	37	1	Banjaranyar	<200	Sengon	Paraserianthes falcataria (L.) I.C.	Leguminosae / Fabaceae	0,33	15,0	11	14	0,01	0,00	64,60	0,06	3,04	11,14
1783	37	1	Banjaranyar	<200	Sengon	Paraserianthes falcataria (L.) I.C.	Leguminosae / Fabaceae	0,33	12,1	13	15	0,01	0,00	46,62	0,05	2,19	8,04
1784	37	1	Banjaranyar	<200	sengon	Paraserianthes falcataria (L.) I.C.	Leguminosae / Fabaceae	0,33	19,1	11	14	0,01	0,01	101,04	0,10	4,75	17,43
1785	37	1	Banjaranyar	<200	sengon	Paraserianthes falcataria (L.) I.C.	Leguminosae / Fabaceae	0,33	14,3	12	14	0,01	0,00	59,65	0,06	2,80	10,29
1786	37	1	Banjaranyar	<200	sengon	Paraserianthes falcataria (L.) I.C.	Leguminosae / Fabaceae	0,33	17,8	10	13	0,01	0,01	83,20	0,08	3,91	14,35
1787	37	1	Banjaranyar	<200	sengon	Paraserianthes falcataria (L.) I.C.	Leguminosae / Fabaceae	0,33	17,2	12	14	0,01	0,01	83,31	0,08	3,92	14,37
1788	37	1	Banjaranyar	<200	sengon	Paraserianthes falcataria (L.) I.C.	Leguminosae / Fabaceae	0,33	13,7	12	14	0,01	0,00	54,88	0,05	2,58	9,47
1789	37	1	Banjaranyar	<200	Sengon	Paraserianthes falcataria (L.) I.C.	Leguminosae / Fabaceae	0,33	13,4	11	15	0,01	0,00	56,00	0,06	2,63	9,66
1790	37	1	Banjaranyar	<200	Sengon	Paraserianthes falcataria (L.) I.C.	Leguminosae / Fabaceae	0,33	14,3	11	15	0,01	0,00	63,54	0,06	2,99	10,96
1791	37	1	Banjaranyar	<200	Sengon	Paraserianthes falcataria (L.) I.C.	Leguminosae / Fabaceae	0,33	10,2	10	14	0,01	0,00	31,94	0,03	1,50	5,51
1792	37	1	Banjaranyar	<200	Sengon	Paraserianthes falcataria (L.) I.C.	Leguminosae / Fabaceae	0,33	20,7	8	15	0,01	0,01	124,63	0,12	5,86	21,50
1793	37	1	Banjaranyar	<200	Sengon	Paraserianthes falcataria (L.) I.C.	Leguminosae / Fabaceae	0,33	7,3	7	8	0,01	0,00	10,45	0,01	0,49	1,80
1794	37	1	Banjaranyar	<200	Sengon	Paraserianthes falcataria (L.) I.C.	Leguminosae / Fabaceae	0,33	7,6	6	9	0,01	0,00	12,58	0,01	0,59	2,17
1795	37	1	Banjaranyar	<200	Sengon	Paraserianthes falcataria (L.) I.C.	Leguminosae / Fabaceae	0,33	17,5	10	15	0,01	0,01	91,77	0,09	4,31	15,83
1796	37	1	Banjaranyar	<200	Sengon	Paraserianthes falcataria (L.) I.C.	Leguminosae / Fabaceae	0,33	13,0	10	14	0,01	0,00	50,30	0,05	2,36	8,68
1797	37	1	Banjaranyar	<200	Sengon	Paraserianthes falcataria (L.) I.C.	Leguminosae / Fabaceae	0,33	15,9	10	13	0,01	0,00	67,60	0,07	3,18	11,66
1798	38	2	Banjaranyar	<200	Mahoni	Swietenia macrophylla King	Meliaceae	0,61	11,1	5	8	0,04	0,00	39,58	0,04	0,47	1,71
1799	38	2	Banjaranyar	<200	Mahoni	Swietenia macrophylla King	Meliaceae	0,61	20,7	4	9	0,04	0,03	137,03	0,14	1,61	5,91
1800	38	2	Banjaranyar	<200	Bungbulang	Premna tomentosa Willd.	Lamiaceae / Verbenaceae	0,96	11,1	5	8	0,04	0,01	59,96	0,06	0,70	2,59
1801	38	2	Banjaranyar	<200	Jati	Tectona grandis L.f.	Lamiaceae / Verbenaceae	0,7	3,8	2	5	0,04	0,00	4,11	0,00	0,05	0,18
1802	38	2	Banjaranyar	<200	Ketapang	Terminalia catappa L.	Combretaceae	0,65	4,1	2	4	0,04	0,00	3,62	0,00	0,04	0,16
1803	38	2	Banjaranyar	<200	Bungbulang	Premna tomentosa Willd.	Lamiaceae / Verbenaceae	0,96	9,5	6	9	0,04	0,01	50,36	0,05	0,59	2,17
1804	38	2	Banjaranyar	<200	Jati	Tectona grandis L.f.	Lamiaceae / Verbenaceae	0,7	4,8	3	5	0,04	0,00	6,18	0,01	0,07	0,27
1805	38	2	Banjaranyar	<200	Jati	Tectona grandis L.f.	Lamiaceae / Verbenaceae	0,7	14,3	7	12	0,04	0,02	103,14	0,10	1,21	4,45
1806	38	2	Banjaranyar	<200	Ketapang	Terminalia catappa L.	Combretaceae	0,65	3,5	4	5	0,04	0,00	3,27	0,00	0,04	0,14
1807	38	2	Banjaranyar	<200	Mangga	Mangifera indica L.	Anacardiaceae	0,6	8,3	4	6	0,04	0,01	17,37	0,02	0,20	0,75
1808	38	2	Banjaranyar	<200	Rambutan	Nephelium lappaceum L.	Sapindaceae	0,71	4,1	3	5	0,04	0,00	4,82	0,00	0,06	0,21
1809	38	2	Banjaranyar	<200	Heuras	Vitex pubescens Vahl	Lamiaceae / Verbenaceae	0,88	4,8	6	7	0,04	0,00	10,37	0,01	0,12	0,45
1810	38	2	Banjaranyar	<200	Mahoni	Swietenia macrophylla King	Meliaceae	0,61	10,2	5	8	0,04	0,01	33,59	0,03	0,39	1,45
1811	38	2	Banjaranyar	<200	Mahoni	Swietenia macrophylla King	Meliaceae	0,61	19,4	7	12	0,04	0,03	158,76	0,16	1,87	6,85
1812	38	2	Banjaranyar	<200	Kelapa	Cocos nucifera L.	Arecaceae	0,5	29,6	12	16	0,04	0,07	127,70	0,13	1,50	5,51
1813	38	2	Banjaranyar	<200	Tisuk	Hibiscus macrophyllus Roxb. ex I	Malvaceae	0,4	5,1	6	7	0,04	0,00	5,67	0,01	0,07	0,24
1814	38	2	Banjaranyar	<200	Cangcaratan	Nauclea subdita (Korth.) Steud	Rubiaceae	0,32	5,7	2	5	0,04	0,00	4,21	0,00	0,05	0,18
1815	38	2	Banjaranyar	<200	Huru keling	Litsea sp.	Lauraceae	0,425	13,4	7	10	0,04	0,01	48,70	0,05	0,57	2,10
1816	38	2	Banjaranyar	<200	Mahoni	Swietenia macrophylla King	Meliaceae	0,61	17,2	5	9	0,04	0,02	97,57	0,10	1,15	4,21
1817	38	2	Banjaranyar	<200	Pinang	Areca catechu L.	Arecaceae	0,66	9,5	4	6	0,04	0,01	24,64	0,02	0,29	1,06
1818	38	2	Banjaranyar	<200	Jati	Tectona grandis L.f.	Lamiaceae / Verbenaceae	0,7	3,8	2	3	0,04	0,00	2,57	0,00	0,03	0,11
1819	38	2	Banjaranyar	<200	Heuras	Vitex pubescens Vahl	Lamiaceae / Verbenaceae	0,88	3,8	7	9	0,04	0,00	8,68	0,01	0,10	0,37
1820	38	2	Banjaranyar	<200	Pala	Myristica fragrans Houtt.	Myristicaceae	0,5	6,4	6	8	0,04	0,00	11,83	0,01	0,14	0,51
1821	38	2	Banjaranyar	<200	Mahoni	Swietenia macrophylla King	Meliaceae	0,61	5,1	2	4	0,04	0,00	5,00	0,00	0,06	0,22
1822	38	2	Banjaranyar	<200	Mahoni	Swietenia macrophylla King	Meliaceae	0,61	4,8	4	7	0,04	0,00	7,42	0,01	0,09	0,32
1823	38	2	Banjaranyar	<200	Bungbulang	Premna tomentosa Willd.	Lamiaceae / Verbenaceae	0,96	7,6	3	5	0,04	0,00	19,53	0,02	0,23	0,84
1824	38	2	Banjaranyar	<200	Ketapang	Terminalia catappa L.	Combretaceae	0,65	4,8	2	4	0,04	0,00	4,71	0,00	0,06	0,20
1825	38	2	Banjaranyar	<200	Bungbulang	Premna tomentosa Willd.	Lamiaceae / Verbenaceae	0,96	10,2	5	8	0,04	0,01	50,88	0,05	0,60	2,19
1826	38	2	Banjaranyar	<200	Jati	Tectona grandis L.f.	Lamiaceae / Verbenaceae	0,7	7,6	4	7	0,04	0,00	19,90	0,02	0,23	0,86
1827	38	2	Banjaranyar	<200	Jati	Tectona grandis L.f.	Lamiaceae / Verbenaceae	0,7	4,8	3	5	0,04	0,00	6,18	0,01	0,07	0,27
1828	38	2	Banjaranyar	<200	Jati	Tectona grandis L.f.	Lamiaceae / Verbenaceae	0,7	5,4	4	8	0,04	0,00	11,96	0,01	0,14	0,52
1829	38	2	Banjaranyar	<200	Jati	Tectona grandis L.f.	Lamiaceae / Verbenaceae	0,7	6,4	3	7	0,04	0,00	14,25	0,01	0,17	0,61
1830	38	2	Banjaranyar	<200	Mahoni	Swietenia macrophylla King	Meliaceae	0,61	3,2	3	4	0,04	0,00	2,11	0,00	0,02	0,09
1831	38	2	Banjaranyar	<200	Jati	Tectona grandis L.f.	Lamiaceae / Verbenaceae	0,7	7,0	5	7	0,04	0,00	16,97	0,02	0,20	0,73
1832	38	2	Banjaranyar	<200	Tisuk	Hibiscus macrophyllus Roxb. ex I	Malvaceae	0,4	4,5	4	6	0,04	0,00	3,86	0,00	0,05	0,17
1833	38	2	Banjaranyar	<200	Mahoni	Swietenia macrophylla King	Meliaceae	0,61	21,3	7	16	0,04	0,04	245,37	0,25	2,88	10,58
1834	38	2	Banjaranyar	<200	Mahoni	Swietenia macrophylla King	Meliaceae	0,61	13,7	6	9	0,04	0,01	64,28	0,06	0,76	2,77
1835	38	2	Banjaranyar	<200	Mahoni	Swietenia macrophylla King	Meliaceae	0,61	2,9	2	3	0,04	0,00	1,34	0,00	0,02	0,06
1836	38	2	Banjaranyar	<200	Huru keling	Litsea sp.	Lauraceae	0,425	7,0	6	9	0,04	0,00	13,52	0,01	0,16	0,58
1837	38	2	Banjaranyar	<200	Mahoni	Swietenia macrophylla King	Meliaceae	0,61	13,7	4	8	0,04	0,01	57,71	0,06	0,68	2,49
1838	38	2	Banjaranyar	<200	Huru keling	Litsea sp.	Lauraceae	0,425	7,0	5	9	0,04	0,00	13,52	0,01	0,16	0,58
1839	38	2	Banjaranyar	<200	Mahoni	Swietenia macrophylla King	Meliaceae	0,61	5,4	4	6	0,04	0,00	8,10	0,01	0,10	0,35
1840	38	2	Banjaranyar	<200	Mahoni	Swietenia macrophylla King	Meliaceae	0,61	3,5	3	4	0,04	0,00	2,52	0,00	0,03	0,11
1841	38	2	Banjaranyar	<200	Huru keling	Litsea sp.	Lauraceae	0,425	8,9	6	11	0,04	0,01	25,28	0,03	0,30	1,09
1842	38	2	Banjaranyar	<200	Jati	Tectona grandis L.f.	Lamiaceae / Verbenaceae	0,7	4,5	4	5	0,04	0,00	5,45	0,01	0,06	0,23
1843	38	2	Banjaranyar	<200	Ketapang	Terminalia catappa L.	Combretaceae	0,65	2,5	2	3	0,04	0,00	1,14	0,00	0,01	0,05
1844	38	2	Banjaranyar	<200	Mahoni	Swietenia macrophylla King	Meliaceae	0,61	15,9	8	13	0,04	0,02	118,68	0,12	1,39	5,12
1845	38	2	Banjaranyar	<200	Ketapang	Terminalia catappa L.	Combretaceae	0,65	3,5	3	4	0,04	0,00	2,67	0,00	0,03	0,12
1846	38	2	Banjaranyar	<200	Tisuk	Hibiscus macrophyllus Roxb. ex I	Malvaceae	0,4	24,2	10	14	0,04	0,05	185,83	0,19	2,18	8,01
1847	38	2	Banjaranyar	<200	Salam	Syzygium polyanthum (Wight) W.	Myrtaceae	0,66	4,8	4	7	0,04	0,00	7,97	0,01	0,09	0,34
1848	38	2	Banjaranyar	<200	Mahoni	Swietenia macrophylla King	Meliaceae	0,61	15,9	4	9	0,04	0,02	84,74	0,08	1,00	3,65
1849	38	2	Banjaranyar	<200	Mahoni	Swietenia macrophylla King	Meliaceae	0,61	9,5	5	8	0,04	0,01	29,84	0,03	0,35	1,29
1850	38	2	Banjaranyar	<200	Mahoni	Swietenia macrophylla King	Meliaceae	0,61	6,4	5	6	0,04	0,00	10,91	0,01	0,13	0,47
1851	38	2	Banjaranyar	<200	Mahoni	Swietenia macrophylla King	Meliaceae	0,61	20,4	4	11	0,04	0,03	160,07	0,16	1,88	6,90
1852	38	2	Banjaranyar	<200	Mahoni	Swietenia macrophylla King	Meliaceae	0,61	5,1	5	5	0,04	0,00	6,13	0,01	0,07	0,26
1853	38	2	Banjaranyar	<200	Mahoni	Swietenia macrophylla King	Meliaceae	0,61	17,8	5	10	0,04	0,02	114,86	0,11	1,35	4,95
1854	38	2	Banjaranyar	<200	Kenanga	Cananga odorata (Lam.) Hook.f.	Annonaceae	0,33	8,0	7	9	0,04	0,00	13,56	0,01	0,16	0,58

1855	38	2	Banjaranyar	<200	Kelapa	Cocos nucifera L.	Arecaceae	0,5	27,0	15	19	0,04	0,06	150,80	0,15	1,77	6,50
1856	38	2	Banjaranyar	<200	Mahoni	Swietenia macrophylla King	Meliaceae	0,61	13,4	5	9	0,04	0,01	61,57	0,06	0,72	2,65
1857	38	2	Banjaranyar	<200	Ketapang	Terminalia catappa L.	Combretaceae	0,65	8,0	8	10	0,04	0,00	27,78	0,03	0,33	1,20
1858	38	2	Banjaranyar	<200	Mahoni	Swietenia macrophylla King	Meliaceae	0,61	9,5	7	11	0,04	0,01	39,95	0,04	0,47	1,72
1859	38	2	Banjaranyar	<200	Tisuk	Hibiscus macrophyllus Roxb. ex l	Malvaceae	0,4	6,0	7	9	0,04	0,00	9,78	0,01	0,11	0,42
1860	38	2	Banjaranyar	<200	Mahoni	Swietenia macrophylla King	Meliaceae	0,61	19,1	11	14	0,04	0,03	177,38	0,18	2,08	7,65
1861	39	2	Banjaranyar	<200	Sawo / sawo manila	Manilkara zapota (L.) P.Royen	Sapotaceae	0,81	18,5	1,5	2	0,04	0,03	36,36	0,04	0,43	1,57
1862	39	2	Banjaranyar	<200	Pala	Myristica fragrans Houtt.	Myristicaceae	0,5	4,5	0,5	2	0,04	0,00	1,73	0,00	0,02	0,07
1863	39	2	Banjaranyar	<200	Jati	Tectona grandis L.f.	Lamiaceae / Verbenaceae	0,7	15,9	7	11	0,04	0,02	115,52	0,12	1,36	4,98
1864	39	2	Banjaranyar	<200	Sawo / sawo manila	Manilkara zapota (L.) P.Royen	Sapotaceae	0,81	8,9	3	5	0,04	0,01	22,17	0,02	0,26	0,96
1865	39	2	Banjaranyar	<200	Sawo / sawo manila	Manilkara zapota (L.) P.Royen	Sapotaceae	0,81	11,5	3	6	0,04	0,01	41,52	0,04	0,49	1,79
1866	39	2	Banjaranyar	<200	Sawo / sawo manila	Manilkara zapota (L.) P.Royen	Sapotaceae	0,81	17,2	4	5	0,04	0,02	73,84	0,07	0,87	3,18
1867	39	2	Banjaranyar	<200	Jati	Tectona grandis L.f.	Lamiaceae / Verbenaceae	0,7	18,5	5	10	0,04	0,03	138,94	0,14	1,63	5,99
1868	39	2	Banjaranyar	<200	Pala	Myristica fragrans Houtt.	Myristicaceae	0,5	3,8	0,5	2	0,04	0,00	1,30	0,00	0,02	0,06
1869	39	2	Banjaranyar	<200	Sawo / sawo manila	Manilkara zapota (L.) P.Royen	Sapotaceae	0,81	13,4	1,8	4	0,04	0,01	37,98	0,04	0,45	1,64
1870	39	2	Banjaranyar	<200	Sawo / sawo manila	Manilkara zapota (L.) P.Royen	Sapotaceae	0,81	20,4	4	10	0,04	0,03	190,20	0,19	2,23	8,20
1871	39	2	Banjaranyar	<200	Sawo / sawo manila	Manilkara zapota (L.) P.Royen	Sapotaceae	0,81	17,8	4	10	0,04	0,02	148,93	0,15	1,75	6,42
1872	39	2	Banjaranyar	<200	Sawo / sawo manila	Manilkara zapota (L.) P.Royen	Sapotaceae	0,81	14,0	4	9	0,04	0,02	86,93	0,09	1,02	3,75
1873	39	2	Banjaranyar	<200	Sawo / sawo manila	Manilkara zapota (L.) P.Royen	Sapotaceae	0,81	23,9	3	9	0,04	0,04	230,94	0,23	2,71	9,96
1874	39	2	Banjaranyar	<200	Pala	Myristica fragrans Houtt.	Myristicaceae	0,5	2,9	0,5	2	0,04	0,00	0,77	0,00	0,01	0,03
1875	39	2	Banjaranyar	<200	Mahoni	Swietenia macrophylla King	Meliaceae	0,61	21,6	4	8	0,04	0,04	133,62	0,13	1,57	5,76
1876	39	2	Banjaranyar	<200	Mahoni	Swietenia macrophylla King	Meliaceae	0,61	19,7	4	8	0,04	0,03	112,82	0,11	1,33	4,86
1877	39	2	Banjaranyar	<200	Pala	Myristica fragrans Houtt.	Myristicaceae	0,5	3,5	0,5	2	0,04	0,00	1,11	0,00	0,01	0,05
1878	39	2	Banjaranyar	<200	Sawo / sawo manila	Manilkara zapota (L.) P.Royen	Sapotaceae	0,81	6,4	1	4	0,04	0,00	9,76	0,01	0,11	0,42
1879	39	2	Banjaranyar	<200	Sawo / sawo manila	Manilkara zapota (L.) P.Royen	Sapotaceae	0,81	6,4	1	6	0,04	0,00	14,14	0,01	0,17	0,61
1880	39	2	Banjaranyar	<200	Sirsak	Annona muricata L.	Annonaceae	0,36	16,5	4	7	0,04	0,02	44,62	0,04	0,52	1,92
1881	39	2	Banjaranyar	<200	Sirsak	Annona muricata L.	Annonaceae	0,36	17,2	4	7	0,04	0,02	47,81	0,05	0,56	2,06
1882	40	2	Banjaranyar	<200	Mahoni	Swietenia macrophylla King	Meliaceae	0,61	6,7	3	5	0,04	0,00	10,09	0,01	0,12	0,44
1883	40	2	Banjaranyar	<200	Tisuk	Hibiscus macrophyllus Roxb. ex l	Malvaceae	0,4	20,4	4	9	0,04	0,03	90,49	0,09	1,06	3,90
1884	40	2	Banjaranyar	<200	Mahoni	Swietenia macrophylla King	Meliaceae	0,61	7,6	3	6	0,04	0,00	15,23	0,02	0,18	0,66
1885	40	2	Banjaranyar	<200	Tisuk	Hibiscus macrophyllus Roxb. ex l	Malvaceae	0,4	2,9	2	3	0,04	0,00	0,91	0,00	0,01	0,04
1886	40	2	Banjaranyar	<200	Tisuk	Hibiscus macrophyllus Roxb. ex l	Malvaceae	0,4	14,3	9	13	0,04	0,02	66,48	0,07	0,78	2,87
1887	40	2	Banjaranyar	<200	Salam	Syzygium polyanthum (Wight) W.	Myrtaceae	0,66	15,6	6	10	0,04	0,02	96,66	0,10	1,14	4,17
1888	40	2	Banjaranyar	<200	Mahoni	Swietenia macrophylla King	Meliaceae	0,61	5,1	4	6	0,04	0,00	7,25	0,01	0,09	0,31
1889	40	2	Banjaranyar	<200	Salam	Syzygium polyanthum (Wight) W.	Myrtaceae	0,66	6,4	6	7	0,04	0,00	13,50	0,01	0,16	0,58
1890	40	2	Banjaranyar	<200	Mahoni	Swietenia macrophylla King	Meliaceae	0,61	2,5	1	2,5	0,04	0,00	0,91	0,00	0,01	0,04
1891	40	2	Banjaranyar	<200	Jati	Tectona grandis L.f.	Lamiaceae / Verbenaceae	0,7	8,6	4	9	0,04	0,01	31,09	0,03	0,37	1,34
1892	40	2	Banjaranyar	<200	Mahoni	Swietenia macrophylla King	Meliaceae	0,61	8,0	4	6	0,04	0,00	16,42	0,02	0,19	0,71
1893	40	2	Banjaranyar	<200	Mahoni	Swietenia macrophylla King	Meliaceae	0,61	19,1	7	12	0,04	0,03	154,02	0,15	1,81	6,64
1894	40	2	Banjaranyar	<200	Mahoni	Swietenia macrophylla King	Meliaceae	0,61	5,1	3	5	0,04	0,00	6,13	0,01	0,07	0,26
1895	40	2	Banjaranyar	<200	Mahoni	Swietenia macrophylla King	Meliaceae	0,61	9,2	4	6	0,04	0,01	21,55	0,02	0,25	0,93
1896	40	2	Banjaranyar	<200	Mahoni	Swietenia macrophylla King	Meliaceae	0,61	6,4	4	5	0,04	0,00	9,23	0,01	0,11	0,40
1897	40	2	Banjaranyar	<200	Mahoni	Swietenia macrophylla King	Meliaceae	0,61	10,2	5	7	0,04	0,01	29,72	0,03	0,35	1,28
1898	40	2	Banjaranyar	<200	Mahoni	Swietenia macrophylla King	Meliaceae	0,61	2,5	3	5	0,04	0,00	1,72	0,00	0,02	0,07
1899	40	2	Banjaranyar	<200	Salam	Syzygium polyanthum (Wight) W.	Myrtaceae	0,66	8,9	8	11	0,04	0,01	37,84	0,04	0,44	1,63
1900	40	2	Banjaranyar	<200	Salam	Syzygium polyanthum (Wight) W.	Myrtaceae	0,66	6,0	4	7	0,04	0,00	12,29	0,01	0,14	0,53
1901	40	2	Banjaranyar	<200	Salam	Syzygium polyanthum (Wight) W.	Myrtaceae	0,66	4,1	4	5	0,04	0,00	4,51	0,00	0,05	0,19
1902	40	2	Banjaranyar	<200	Mahoni	Swietenia macrophylla King	Meliaceae	0,61	19,1	6	11	0,04	0,03	142,22	0,14	1,67	6,13
1903	40	2	Banjaranyar	<200	Mahoni	Swietenia macrophylla King	Meliaceae	0,61	11,1	3,5	7,5	0,04	0,01	37,31	0,04	0,44	1,61
1904	40	2	Banjaranyar	<200	Mahoni	Swietenia macrophylla King	Meliaceae	0,61	6,4	3,5	5	0,04	0,00	9,23	0,01	0,11	0,40
1905	40	2	Banjaranyar	<200	Salam	Syzygium polyanthum (Wight) W.	Myrtaceae	0,66	5,1	5	6	0,04	0,00	7,79	0,01	0,09	0,34
1906	40	2	Banjaranyar	<200	Mahoni	Swietenia macrophylla King	Meliaceae	0,61	6,4	3,5	6,5	0,04	0,00	11,74	0,01	0,14	0,51
1907	40	2	Banjaranyar	<200	Salam	Syzygium polyanthum (Wight) W.	Myrtaceae	0,66	17,5	7	11	0,04	0,02	130,34	0,13	1,53	5,62
1908	40	2	Banjaranyar	<200	Gmelina	Gmelina arborea Roxb.	Lamiaceae / Verbenaceae	0,47	6,4	4	5	0,04	0,00	7,27	0,01	0,09	0,31
1909	40	2	Banjaranyar	<200	Gmelina	Gmelina arborea Roxb.	Lamiaceae / Verbenaceae	0,47	16,9	9	14	0,04	0,02	111,30	0,11	1,31	4,80
1910	40	2	Banjaranyar	<200	Gmelina	Gmelina arborea Roxb.	Lamiaceae / Verbenaceae	0,47	5,1	5	6	0,04	0,00	5,71	0,01	0,07	0,25
1911	40	2	Banjaranyar	<200	Mahoni	Swietenia macrophylla King	Meliaceae	0,61	2,5	1,8	2	0,04	0,00	0,74	0,00	0,01	0,03
1912	40	2	Banjaranyar	<200	Kelapa	Cocos nucifera L.	Arecaceae	0,5	28,6	10	14	0,04	0,06	112,30	0,11	1,32	4,84
1913	40	2	Banjaranyar	<200	Mahoni	Swietenia macrophylla King	Meliaceae	0,61	8,0	4	8	0,04	0,00	21,37	0,02	0,25	0,92
1914	40	2	Banjaranyar	<200	Mahoni	Swietenia macrophylla King	Meliaceae	0,61	11,8	4	9	0,04	0,01	48,81	0,05	0,57	2,10
1915	40	2	Banjaranyar	<200	Mahoni	Swietenia macrophylla King	Meliaceae	0,61	12,7	5	10	0,04	0,01	62,01	0,06	0,73	2,67
1916	40	2	Banjaranyar	<200	Mahoni	Swietenia macrophylla King	Meliaceae	0,61	8,6	3	5	0,04	0,01	16,00	0,02	0,19	0,69
1917	40	2	Banjaranyar	<200	Gmelina	Gmelina arborea Roxb.	Lamiaceae / Verbenaceae	0,47	7,3	6	8	0,04	0,00	14,44	0,01	0,17	0,62
1918	40	2	Banjaranyar	<200	Jati	Tectona grandis L.f.	Lamiaceae / Verbenaceae	0,7	3,5	3	5	0,04	0,00	3,50	0,00	0,04	0,15
1919	40	2	Banjaranyar	<200	Gmelina	Gmelina arborea Roxb.	Lamiaceae / Verbenaceae	0,47	6,4	7	9	0,04	0,00	12,45	0,01	0,15	0,54
1920	40	2	Banjaranyar	<200	Mahoni	Swietenia macrophylla King	Meliaceae	0,61	6,4	4	7	0,04	0,00	12,56	0,01	0,15	0,54
1921	40	2	Banjaranyar	<200	Gmelina	Gmelina arborea Roxb.	Lamiaceae / Verbenaceae	0,47	5,7	9	11	0,04	0,00	12,34	0,01	0,15	0,53
1922	40	2	Banjaranyar	<200	Mahoni	Swietenia macrophylla King	Meliaceae	0,61	9,2	4	8	0,04	0,01	28,04	0,03	0,33	1,21
1923	40	2	Banjaranyar	<200	Mahoni	Swietenia macrophylla King	Meliaceae	0,61	12,7	5	9	0,04	0,01	56,30	0,06	0,66	2,43
1924	40	2	Banjaranyar	<200	Mahoni	Swietenia macrophylla King	Meliaceae	0,61	15,3	6	10	0,04	0,02	86,60	0,09	1,02	3,73
1925	40	2	Banjaranyar	<200	Tisuk	Hibiscus macrophyllus Roxb. ex l	Malvaceae	0,4	5,7	4,5	6	0,04	0,00	6,11	0,01	0,07	0,26
1926	40	2	Banjaranyar	<200	Wangkal	Albizia procera Benth	Leguminosae / Fabaceae	0,77	6,4	4	6	0,04	0,00	13,50	0,01	0,16	0,58
1927	40	2	Banjaranyar	<200	Jati	Tectona grandis L.f.	Lamiaceae / Verbenaceae	0,7	8,3	3	7	0,04	0,01	23,04	0,02	0,27	0,99
1928	40	2	Banjaranyar	<200	Mahoni	Swietenia macrophylla King	Meliaceae	0,61	3,5	3	4	0,04	0,00	2,52	0,00	0,03	0,11
1929	40	2	Banjaranyar	<200	Mangga	Mangifera indica L.	Anacardiaceae	0,6	5,4	2	3	0,04	0,00	4,23	0,00	0,05	0,18
1930	40	2	Banjaranyar	<200	Salam	Syzygium polyanthum (Wight) W.	Myrtaceae	0,66	4,1	3	4	0,04	0,00	3,67	0,00	0,04	0,16
1931	40	2	Banjaranyar	<200	Tisuk	Hibiscus macrophyllus Roxb. ex l	Malvaceae	0,4	17,2	8	11	0,04	0,02	79,66	0,08	0,94	3,44
1932	40	2	Banjaranyar	<200	Tisuk	Hibiscus macrophyllus Roxb. ex l	Malvaceae	0,4	4,5	5	6	0,04	0,00	3,86	0,00	0,05	0,17
1933	40	2	Banjaranyar	<200	Tisuk	Hibiscus macrophyllus Roxb. ex l	Malvaceae	0,4	15,0	8	11	0,04	0,02	61,77	0,06	0,73	2,66
1934	40	2	Banjaranyar	<200	Tisuk	Hibiscus macrophyllus Roxb. ex l	Malvaceae	0,4	5,1	6	8	0,04	0,00	6,41	0,01	0,08	0,28
1935	40	2	Banjaranyar	<200	Nangka	Artocarpus heterophyllus Lam.	Moraceae	0,61	16,5	2	8	0,04	0,02	81,74	0,08	0,96	3,52
1936	40	2	Banjaranyar	<200	Salam	Syzygium polyanthum (Wight) W.	Myrtaceae	0,66	11,8	7	10	0,04	0,01	57,78	0,06	0,68	2,49
1937	40	2	Banjaranyar	<200	Tisuk	Hibiscus macrophyllus Roxb. ex l	Malvaceae	0,4	10,8	8	10	0,04	0,01	31,28	0,03	0,37	1,35
1938	40	2	Banjaranyar	<200	Mahoni	Swietenia macrophylla King	Meliaceae	0,61	6,4	3	7	0,04	0,00	12,56	0,01	0,15	0,54
1939	40	2	Banjaranyar	<200	Wisnu	Sp7	0	0,5	8,6	6	9	0,04	0,01	22,84	0,02	0,27	0,98
1940	40	2	Banjaranyar	<200	Mangga	Mangifera indica L.	Anacardiaceae	0,6	19,7	4	11	0,04	0,03	148,76	0,15	1,75	6,41
1941	40	2	Banjaranyar	<200	Kelapa	Cocos nucifera L.	Arecaceae	0,5	29,3	10	14	0,04	0,07	112,30	0,11	1,32	4,84
1942	40	2	Banjaranyar	<200	Salam	Syzygium polyanthum (Wight) W.	Myrtaceae	0,66	14,0	5	10	0,04	0,02	79,36	0,08	0,93	3,42
1943	40	2	Banjaranyar	<200	Mahoni	Swietenia macrophylla King	Meliaceae	0,61	8,0	4	8	0,04	0,00	21,37	0,02	0,25	0,92
1944	40	2	Banjaranyar	<200	Mahoni	Swietenia macrophylla King	Meliaceae	0,61	12,7	3	8	0,04	0,01	50,55	0,05	0,59	2,18
1945	40	2	Banjaranyar	<200	Mahoni	Swietenia macrophylla King	Meliaceae	0,61	8,9	3	5	0,04	0,01	17,10	0,02	0,20	0,74
1946	40	2	Banjaranyar	<200	Mahoni	Swietenia macrophylla King	Meliaceae	0,61	15,0	6	9	0,04	0,02	75,66	0,08	0,89	3,26
1947	40	2	Banjaranyar	<200	Mahoni	Swietenia macrophylla King	Meliaceae	0,61	12,4	6	9	0,04	0,01	53,75	0,05	0,63	2,32
1948	40	2	Banjaranyar	<200	Mahoni	Swietenia macrophylla King	Meliaceae	0,61	8,9	4	8	0,04	0,01	26,30	0,03	0,31	1,13
1949	40	2	Banjaranyar	<200	Mahoni	Swietenia macrophylla King	Meliaceae	0,61	6,4	3	6	0,04	0,00	10,91	0,01	0,13	0,47
1950	40	2	Banjaranyar	<200	Mahoni	Swietenia macrophylla King	Meliaceae	0,61	8,0	4	7	0,04	0,00	18,91	0,02	0,22	0,82
1951	40	2	Banjaranyar	<200	Mahoni	Swietenia macrophylla King	Meliaceae	0,61	15,9	3,5	8,5	0,04	0,02	80,42	0,08	0,94	3,47
1952	40	2	Banjaranyar	<200	Kelapa	Cocos nucifera L.	Arecaceae	0,5	28,6	10	14	0,04	0,06	112,30	0,11	1,32	4,84
1953	40	2	Banjaranyar	<200	Sirsak	Annona muricata L.	Annonaceae	0,36	4,5	3,5	4,5	0,04	0,00	2,69	0,00	0,03	0,12
1954	40	2	Banjaranyar	<200	Mangga	Mangifera indica L.	Anacardiaceae	0,6	16,9	6	10	0,04	0,02	102,28	0,10	1,20	4,41
1955	40	2	Banjaranyar	<200	Salam	Syzygium polyanthum (Wight) W.	Myrtaceae	0,66	23,9	10	15	0,04	0,04	305,66	0,31	3,59	13,18
1956	41	2	Banjaranyar	<200	Mahoni	Swietenia macrophylla King	Meliaceae	0,61	26,4	10	17	0,01	0,01	383,99	0,38	18,05	66,24
1957	41	2	Banjaranyar	<200	Mahoni	Swietenia macrophylla King	Meliaceae	0,61	29,6	9	16	0,01	0,02	447,42	0,45	21,03	77,18
1958	41	2	Banjaranyar	<200	Kelapa	Cocos nucifera L.	Arecaceae	0,5	24,2	5	9	0,01	0,01	73,80	0,07	3,47	12,73
1959	41	2	Banjaranyar	<200	Sengon	Paraserianthes falcataria (L.) I.C	Leguminosae / Fabaceae	0,33	9,9	10	14	0,01	0,00	30,14	0,03	1,42	5,20
1960	41	2	Banjaranyar	<200	Kelapa	Cocos nucifera L.	Arecaceae	0,5	28,6	3	7	0,01	0,02	58,40	0,06	2,74	10,07
1961	41	2	Banjaranyar	<200	Tisuk	Hibiscus macrophyllus Roxb. ex l	Malvaceae	0,4	3,2	2	5	0,01	0,00	1,76	0,00	0,08	0,30
1962	41	2	Banjaranyar	<200	Mahoni	Swietenia macrophylla King	Meliaceae	0,61	3,2	3	4	0,01	0,00	2,11	0,00	0,10	0,36
1963	41	2	Banjaranyar	<200	Tisuk	Hibiscus macrophyllus Roxb. ex l	Malvaceae	0,4	3,2	3	4	0,01	0,00	1,44	0,00	0,07	0,25
1964	41	2	Banjaranyar	<200	Mahoni	Swietenia macrophylla King	Meliaceae	0,61	3,5	2	3,5	0,01	0,00	2,23	0,00	0,10	0,38
1965	41	2	Banjaranyar	<200	Tisuk	Hibiscus macrophyllus Roxb. ex l	Malvaceae	0,4	4,8	4	6	0,01	0,00	4,38	0,00	0,21	0,75
1966	41	2	Banjaranyar	<200	Sengon	Paraserianthes falcataria (L.) I.C	Leguminosae / Fabaceae	0,33	5,1	7	8	0,01	0,00	5,37	0,01	0,25	0,93
1967	41	2	Banjaranyar	<200	Tisuk	Hibiscus macrophyllus Roxb. ex l	Malvaceae	0,4	3,5	3	4,5	0,01	0,00	1,90	0,00	0,09	0,33
1968	41	2	Banjaranyar	<200	Sengon	Paraserianthes falcataria (L.) I.C	Leguminosae / Fabaceae	0,33	24,2	12	17	0,01	0,01	186,13	0,19	8,75	32,11
1969	41	2	Banjaranyar	<200	Sengon	Paraserianthes falcataria (L.) I.C	Leguminosae / Fabaceae	0,33	7,0	7	9	0,01	0,00	10,73	0,01	0,50	1,85
1970	41	2	Banjaranyar	<200	Mahoni	Swietenia macrophylla King	Meliaceae	0,61	3,8	2	4	0,01	0,00	2,95	0,00	0,14	0,51
1971	41	2	Banjaranyar	<200	Mahoni	Swietenia macrophylla King	Meliaceae	0,61	2,5	2	2,5	0,01	0,00	0,91	0,00	0,04	0,16
1972	41	2	Banjaranyar	<200	Sengon	Paraserianthes falcataria (L.) I.C	Leguminosae / Fabaceae	0,33	4,8	6	8	0,01	0,00	4,77	0,00	0,22	0,82
1973	41	2	Banjaranyar	<200	Mahoni	Swietenia macrophylla King	Meliaceae	0,61	3,8	4	5	0,01	0,00	3,62	0,00	0,17	0,62
1974	41	2	Banjaranyar	<200	Mahoni	Swietenia macrophylla King	Meliaceae	0,61	23,2	10	15	0,01	0,01	270,64	0,27	12,72	46,68
1975	41	2	Banjaranyar	<200	Salam	Syzygium polyanthum (Wight) W.	Myrtaceae	0,66	2,5	2	3,5	0,01	0,00	1,34	0,00	0,06	0,23
1976	41	2	Banjaranyar	<200	Mahoni	Swietenia macrophylla King	Meliaceae	0,61	3,5	3	3,5	0,01	0,00	2,23	0,00	0,10	0,38
1977	41	2	Banjaranyar	<200	Mahoni	Swietenia macrophylla King	Meliaceae	0,61	16,2	9	12	0,01	0,01	114,36	0,11	5,37	19,73
1978	41	2	Banjaranyar	<200	Mahoni	Swietenia macrophylla King	Meliaceae	0,61	3,8	2	3	0,01	0,00	2,27	0,00	0,11	0,39

1979	41	2	Banjaranyar	<200	Mahoni	Swietenia macrophylla King	Meliaceae	0,61	24,5	7	13	0,01	0,01	261,76	0,26	12,30	45,15
1980	41	2	Banjaranyar	<200	Mahoni	Swietenia macrophylla King	Meliaceae	0,61	12,7	7	10	0,01	0,00	62,01	0,06	2,91	10,70
1981	41	2	Banjaranyar	<200	Mahoni	Swietenia macrophylla King	Meliaceae	0,61	2,9	2	3	0,01	0,00	1,34	0,00	0,06	0,23
1982	41	2	Banjaranyar	<200	Mahoni	Swietenia macrophylla King	Meliaceae	0,61	10,2	7	10	0,01	0,00	41,20	0,04	1,94	7,11
1983	41	2	Banjaranyar	<200	Mahoni	Swietenia macrophylla King	Meliaceae	0,61	4,1	3,5	4	0,01	0,00	3,42	0,00	0,16	0,59
1984	41	2	Banjaranyar	<200	Mahoni	Swietenia macrophylla King	Meliaceae	0,61	18,8	9	14	0,01	0,01	172,00	0,17	8,08	29,67
1985	41	2	Banjaranyar	<200	Mahoni	Swietenia macrophylla King	Meliaceae	0,61	2,5	2	3	0,01	0,00	1,08	0,00	0,05	0,19
1986	41	2	Banjaranyar	<200	Mahoni	Swietenia macrophylla King	Meliaceae	0,61	18,5	8	12	0,04	0,03	144,75	0,14	1,70	6,24
1987	41	2	Banjaranyar	<200	Mahoni	Swietenia macrophylla King	Meliaceae	0,61	14,3	6	8	0,04	0,02	62,72	0,06	0,74	2,70
1988	41	2	Banjaranyar	<200	Mahoni	Swietenia macrophylla King	Meliaceae	0,61	6,4	5	6,5	0,04	0,00	11,74	0,01	0,14	0,51
1989	41	2	Banjaranyar	<200	Mahoni	Swietenia macrophylla King	Meliaceae	0,61	12,4	5	9	0,04	0,01	53,75	0,05	0,63	2,32
1990	41	2	Banjaranyar	<200	Mahoni	Swietenia macrophylla King	Meliaceae	0,61	19,1	7	11	0,04	0,03	142,22	0,14	1,67	6,13
1991	41	2	Banjaranyar	<200	Mahoni	Swietenia macrophylla King	Meliaceae	0,61	7,3	5	7	0,04	0,00	16,23	0,02	0,19	0,70
1992	41	2	Banjaranyar	<200	Mahoni	Swietenia macrophylla King	Meliaceae	0,61	25,5	10	14	0,04	0,05	300,47	0,30	3,53	12,96
1993	41	2	Banjaranyar	<200	Mahoni	Swietenia macrophylla King	Meliaceae	0,61	13,4	10	13	0,04	0,01	86,23	0,09	1,01	3,72
1994	41	2	Banjaranyar	<200	Mahoni	Swietenia macrophylla King	Meliaceae	0,61	10,5	7	10	0,04	0,01	43,59	0,04	0,51	1,88
1995	41	2	Banjaranyar	<200	Mahoni	Swietenia macrophylla King	Meliaceae	0,61	15,0	10	14	0,04	0,02	113,40	0,11	1,33	4,89
1996	41	2	Banjaranyar	<200	Mahoni	Swietenia macrophylla King	Meliaceae	0,61	5,4	4	5	0,04	0,00	6,85	0,01	0,08	0,30
1997	41	2	Banjaranyar	<200	Mahoni	Swietenia macrophylla King	Meliaceae	0,61	12,7	4	10	0,04	0,01	62,01	0,06	0,73	2,67
1998	41	2	Banjaranyar	<200	Mahoni	Swietenia macrophylla King	Meliaceae	0,61	8,9	6	10	0,04	0,01	32,26	0,03	0,38	1,39
1999	41	2	Banjaranyar	<200	Mahoni	Swietenia macrophylla King	Meliaceae	0,61	30,5	12	18	0,04	0,07	528,24	0,53	6,21	22,78
2000	41	2	Banjaranyar	<200	Rambutan	Nephelium lappaceum L.	Sapindaceae	0,71	19,7	3	10	0,04	0,03	159,05	0,16	1,87	6,86
2001	41	2	Banjaranyar	<200	Mahoni	Swietenia macrophylla King	Meliaceae	0,61	6,0	5	6	0,04	0,00	9,93	0,01	0,12	0,43
2002	41	2	Banjaranyar	<200	Mahoni	Swietenia macrophylla King	Meliaceae	0,61	6,4	4	6	0,04	0,00	10,91	0,01	0,13	0,47
2003	41	2	Banjaranyar	<200	Mahoni	Swietenia macrophylla King	Meliaceae	0,61	5,4	3	4	0,04	0,00	5,59	0,01	0,07	0,24
2004	41	2	Banjaranyar	<200	Mahoni	Swietenia macrophylla King	Meliaceae	0,61	12,7	5	8	0,04	0,01	50,55	0,05	0,59	2,18
2005	41	2	Banjaranyar	<200	Mahoni	Swietenia macrophylla King	Meliaceae	0,61	13,0	7	10	0,04	0,01	64,88	0,06	0,76	2,80
2006	41	2	Banjaranyar	<200	Mahoni	Swietenia macrophylla King	Meliaceae	0,61	16,2	9	12	0,04	0,02	114,36	0,11	1,34	4,93
2007	41	2	Banjaranyar	<200	Mahoni	Swietenia macrophylla King	Meliaceae	0,61	9,5	7	10	0,04	0,01	36,61	0,04	0,43	1,58
2008	41	2	Banjaranyar	<200	Mahoni	Swietenia macrophylla King	Meliaceae	0,61	5,7	4	6	0,04	0,00	8,99	0,01	0,11	0,39
2009	41	2	Banjaranyar	<200	Mahoni	Swietenia macrophylla King	Meliaceae	0,61	37,2	10	17	0,04	0,11	720,26	0,72	8,46	31,06
2010	41	2	Banjaranyar	<200	Kelapa	Cocos nucifera L.	Arecaceae	0,5	22,3	12	16	0,04	0,04	127,70	0,13	1,50	5,51
2011	41	2	Banjaranyar	<200	Mahoni	Swietenia macrophylla King	Meliaceae	0,61	9,5	5	6	0,04	0,01	22,93	0,02	0,27	0,99
2012	41	2	Banjaranyar	<200	Mangga	Mangifera indica L.	Anacardiaceae	0,6	20,4	5	11	0,04	0,03	157,67	0,16	1,85	6,80
2013	41	2	Banjaranyar	<200	Mahoni	Swietenia macrophylla King	Meliaceae	0,61	5,4	3	5	0,04	0,00	6,85	0,01	0,08	0,30
2014	41	2	Banjaranyar	<200	Mahoni	Swietenia macrophylla King	Meliaceae	0,61	15,9	8	12	0,04	0,02	110,29	0,11	1,30	4,76
2015	41	2	Banjaranyar	<200	Mahoni	Swietenia macrophylla King	Meliaceae	0,61	32,8	12	18	0,04	0,08	600,94	0,60	7,06	25,91
2016	41	2	Banjaranyar	<200	Mahoni	Swietenia macrophylla King	Meliaceae	0,61	23,2	11	14	0,04	0,04	254,06	0,25	2,99	10,96
2017	41	2	Banjaranyar	<200	Mahoni	Swietenia macrophylla King	Meliaceae	0,61	31,2	12	17	0,04	0,08	520,59	0,52	6,12	22,45
2018	41	2	Banjaranyar	<200	Mahoni	Swietenia macrophylla King	Meliaceae	0,61	7,0	5	6	0,04	0,00	12,99	0,01	0,15	0,56
2019	41	2	Banjaranyar	<200	Mahoni	Swietenia macrophylla King	Meliaceae	0,61	19,7	11	15	0,04	0,03	200,65	0,20	2,36	8,65
2020	41	2	Banjaranyar	<200	Mahoni	Swietenia macrophylla King	Meliaceae	0,61	5,7	3	5	0,04	0,00	7,61	0,01	0,09	0,33
2021	41	2	Banjaranyar	<200	Mahoni	Swietenia macrophylla King	Meliaceae	0,61	5,4	3	4,5	0,04	0,00	6,22	0,01	0,07	0,27
2022	41	2	Banjaranyar	<200	Mahoni	Swietenia macrophylla King	Meliaceae	0,61	9,9	6	10	0,04	0,01	38,87	0,04	0,46	1,68
2023	41	2	Banjaranyar	<200	Mahoni	Swietenia macrophylla King	Meliaceae	0,61	19,1	9	14	0,04	0,03	177,38	0,18	2,08	7,65
2024	41	2	Banjaranyar	<200	Mahoni	Swietenia macrophylla King	Meliaceae	0,61	9,5	5	8	0,04	0,01	29,84	0,03	0,35	1,29
2025	41	2	Banjaranyar	<200	Mahoni	Swietenia macrophylla King	Meliaceae	0,61	27,0	12	16	0,04	0,06	379,45	0,38	4,46	16,36
2026	41	2	Banjaranyar	<200	Mahoni	Swietenia macrophylla King	Meliaceae	0,61	5,4	3,5	4,5	0,04	0,00	6,22	0,01	0,07	0,27
2027	42	1	Banjaranyar	<200	Jati	Tectona grandis L.f.	Lamiaceae / Verbenaceae	0,7	36,3	10	15	0,04	0,10	694,67	0,69	8,16	29,96
2028	42	1	Banjaranyar	<200	Jati	Tectona grandis L.f.	Lamiaceae / Verbenaceae	0,7	7,3	4	5	0,04	0,00	13,53	0,01	0,16	0,58
2029	42	1	Banjaranyar	<200	Jati	Tectona grandis L.f.	Lamiaceae / Verbenaceae	0,7	16,5	7	10	0,04	0,02	113,75	0,11	1,34	4,91
2030	42	1	Banjaranyar	<200	Jati	Tectona grandis L.f.	Lamiaceae / Verbenaceae	0,7	8,0	6	8	0,04	0,00	24,24	0,02	0,28	1,05
2031	42	1	Banjaranyar	<200	Jati	Tectona grandis L.f.	Lamiaceae / Verbenaceae	0,7	10,2	7	12	0,04	0,01	55,23	0,06	0,65	2,38
2032	42	1	Banjaranyar	<200	Jati	Tectona grandis L.f.	Lamiaceae / Verbenaceae	0,7	27,0	10	16	0,04	0,06	430,43	0,43	5,06	18,56
2033	42	1	Banjaranyar	<200	Jati	Tectona grandis L.f.	Lamiaceae / Verbenaceae	0,7	5,1	2	3	0,04	0,00	4,36	0,00	0,05	0,19
2034	42	1	Banjaranyar	<200	Jati	Tectona grandis L.f.	Lamiaceae / Verbenaceae	0,7	4,8	3	4	0,04	0,00	5,04	0,01	0,06	0,22
2035	42	1	Banjaranyar	<200	Jati	Tectona grandis L.f.	Lamiaceae / Verbenaceae	0,7	15,6	9	10	0,04	0,02	102,02	0,10	1,20	4,40
2036	42	1	Banjaranyar	<200	Jati	Tectona grandis L.f.	Lamiaceae / Verbenaceae	0,7	8,9	6	7	0,04	0,01	26,40	0,03	0,31	1,14
2037	42	1	Banjaranyar	<200	Jati	Tectona grandis L.f.	Lamiaceae / Verbenaceae	0,7	7,0	6	8	0,04	0,00	19,18	0,02	0,23	0,83
2038	42	1	Banjaranyar	<200	Jati	Tectona grandis L.f.	Lamiaceae / Verbenaceae	0,7	10,5	3	10	0,04	0,01	49,45	0,05	0,58	2,13
2039	42	1	Banjaranyar	<200	Jati	Tectona grandis L.f.	Lamiaceae / Verbenaceae	0,7	7,6	5	6	0,04	0,00	17,28	0,02	0,20	0,75
2040	42	1	Banjaranyar	<200	Jati	Tectona grandis L.f.	Lamiaceae / Verbenaceae	0,7	5,7	2,5	4,5	0,04	0,00	7,84	0,01	0,09	0,34
2041	42	1	Banjaranyar	<200	Jati	Tectona grandis L.f.	Lamiaceae / Verbenaceae	0,7	18,5	9	14	0,04	0,03	189,10	0,19	2,22	8,15
2042	42	1	Banjaranyar	<200	Jati	Tectona grandis L.f.	Lamiaceae / Verbenaceae	0,7	8,6	5	7	0,04	0,01	24,69	0,02	0,29	1,06
2043	42	1	Banjaranyar	<200	Jati	Tectona grandis L.f.	Lamiaceae / Verbenaceae	0,7	22,9	9	16	0,04	0,04	317,57	0,32	3,73	13,69
2044	42	1	Banjaranyar	<200	Jati	Tectona grandis L.f.	Lamiaceae / Verbenaceae	0,7	26,4	12	16	0,04	0,05	412,05	0,41	4,84	17,77
2045	42	1	Banjaranyar	<200	Jati	Tectona grandis L.f.	Lamiaceae / Verbenaceae	0,7	5,4	4	6	0,04	0,00	9,19	0,01	0,11	0,40
2046	42	1	Banjaranyar	<200	Wangkal	Albizia procera Benth	Leguminosae / Fabaceae	0,77	5,7	4	5	0,04	0,00	9,42	0,01	0,11	0,41
2047	42	1	Banjaranyar	<200	Mahoni	Swietenia macrophylla King	Meliaceae	0,61	3,2	1	3	0,04	0,00	1,62	0,00	0,02	0,07
2048	42	1	Banjaranyar	<200	Jati	Tectona grandis L.f.	Lamiaceae / Verbenaceae	0,7	8,0	4	7	0,04	0,00	21,45	0,02	0,25	0,92
2049	42	1	Banjaranyar	<200	Jati	Tectona grandis L.f.	Lamiaceae / Verbenaceae	0,7	7,6	4	7	0,04	0,00	19,90	0,02	0,23	0,86
2050	42	1	Banjaranyar	<200	Jati	Tectona grandis L.f.	Lamiaceae / Verbenaceae	0,7	9,9	5	7	0,04	0,01	31,81	0,03	0,37	1,37
2051	42	1	Banjaranyar	<200	Jati	Tectona grandis L.f.	Lamiaceae / Verbenaceae	0,7	8,9	5	9	0,04	0,01	33,23	0,03	0,39	1,43
2052	42	1	Banjaranyar	<200	Jati	Tectona grandis L.f.	Lamiaceae / Verbenaceae	0,7	29,0	10	16	0,04	0,07	487,72	0,49	5,73	21,03
2053	42	1	Banjaranyar	<200	Jati	Tectona grandis L.f.	Lamiaceae / Verbenaceae	0,7	7,0	2	5	0,04	0,00	12,47	0,01	0,15	0,54
2054	42	1	Banjaranyar	<200	Jati	Tectona grandis L.f.	Lamiaceae / Verbenaceae	0,7	17,8	9	13	0,04	0,02	165,68	0,17	1,95	7,14
2055	42	1	Banjaranyar	<200	Jati	Tectona grandis L.f.	Lamiaceae / Verbenaceae	0,7	5,1	2	4	0,04	0,00	5,67	0,01	0,07	0,24
2056	42	1	Banjaranyar	<200	Jati	Tectona grandis L.f.	Lamiaceae / Verbenaceae	0,7	22,3	10	16	0,04	0,04	301,59	0,30	3,54	13,01
2057	42	1	Banjaranyar	<200	Jati	Tectona grandis L.f.	Lamiaceae / Verbenaceae	0,7	17,2	9	14	0,04	0,02	165,89	0,17	1,95	7,15
2058	42	1	Banjaranyar	<200	Jati	Tectona grandis L.f.	Lamiaceae / Verbenaceae	0,7	14,3	5	11	0,04	0,02	95,24	0,10	1,12	4,11
2059	42	1	Banjaranyar	<200	Jati	Tectona grandis L.f.	Lamiaceae / Verbenaceae	0,7	21,3	11	14	0,04	0,04	246,29	0,25	2,89	10,62
2060	42	1	Banjaranyar	<200	Jati Belanda	Guazuma ulmifolia Lamk.	Malvaceae	0,52	5,7	5	6	0,04	0,00	7,77	0,01	0,09	0,34
2061	42	1	Banjaranyar	<200	jati	Tectona grandis L.f.	Lamiaceae / Verbenaceae	0,7	30,5	12	16	0,04	0,07	537,93	0,54	6,32	23,20
2062	42	1	Banjaranyar	<200	Kelapa	Cocos nucifera L.	Arecaceae	0,5	31,8	8	17	0,04	0,08	135,40	0,14	1,59	5,84
2063	42	1	Banjaranyar	<200	Melinjo	Gnetum gnemon L.	Gnetaceae	0,76	4,5	4	7	0,04	0,00	7,99	0,01	0,09	0,34
2064	42	1	Banjaranyar	<200	Jati	Tectona grandis L.f.	Lamiaceae / Verbenaceae	0,7	20,4	9	15	0,04	0,03	241,24	0,24	2,83	10,40
2065	42	1	Banjaranyar	<200	Jati	Tectona grandis L.f.	Lamiaceae / Verbenaceae	0,7	7,3	5	7	0,04	0,00	18,41	0,02	0,22	0,79
2066	42	1	Banjaranyar	<200	Jati	Tectona grandis L.f.	Lamiaceae / Verbenaceae	0,7	12,7	6	10	0,04	0,01	70,34	0,07	0,83	3,03
2067	42	1	Banjaranyar	<200	Jati	Tectona grandis L.f.	Lamiaceae / Verbenaceae	0,7	7,6	2	8	0,04	0,00	22,49	0,02	0,26	0,97
2068	42	1	Banjaranyar	<200	Jati	Tectona grandis L.f.	Lamiaceae / Verbenaceae	0,7	15,3	8	11	0,04	0,02	107,20	0,11	1,26	4,62
2069	42	1	Banjaranyar	<200	Jati	Tectona grandis L.f.	Lamiaceae / Verbenaceae	0,7	29,3	9	15	0,04	0,07	469,02	0,47	5,51	20,23
2070	42	1	Banjaranyar	<200	Jati	Tectona grandis L.f.	Lamiaceae / Verbenaceae	0,7	21,6	10	14	0,04	0,04	253,07	0,25	2,97	10,91
2071	42	1	Banjaranyar	<200	Jati	Tectona grandis L.f.	Lamiaceae / Verbenaceae	0,7	19,7	10	14	0,04	0,03	213,67	0,21	2,51	9,21
2072	42	1	Banjaranyar	<200	Jati	Tectona grandis L.f.	Lamiaceae / Verbenaceae	0,7	10,2	5	8	0,04	0,01	38,10	0,04	0,45	1,64
2073	42	1	Banjaranyar	<200	Jati	Tectona grandis L.f.	Lamiaceae / Verbenaceae	0,7	27,0	11	15	0,04	0,06	405,72	0,41	4,77	17,50
2074	42	1	Banjaranyar	<200	Mahoni	Swietenia macrophylla King	Meliaceae	0,61	3,5	4	5	0,04	0,00	3,09	0,00	0,04	0,13
2075	42	1	Banjaranyar	<200	Jati	Tectona grandis L.f.	Lamiaceae / Verbenaceae	0,7	15,0	10	14	0,04	0,02	128,64	0,13	1,51	5,55
2076	42	1	Banjaranyar	<200	Jati	Tectona grandis L.f.	Lamiaceae / Verbenaceae	0,7	22,9	8	15	0,04	0,04	299,34	0,30	3,52	12,91
2077	42	1	Banjaranyar	<200	Jati	Tectona grandis L.f.	Lamiaceae / Verbenaceae	0,7	18,1	10	14	0,04	0,03	183,17	0,18	2,15	7,90
2078	42	1	Banjaranyar	<200	Jati	Tectona grandis L.f.	Lamiaceae / Verbenaceae	0,7	13,4	10	14	0,04	0,01	104,68	0,10	1,23	4,51
2079	42	1	Banjaranyar	<200	Tisuk	Hibiscus macrophyllus Roxb. ex I	Malvaceae	0,4	13,7	11	15	0,04	0,01	69,73	0,07	0,82	3,01
2080	42	1	Banjaranyar	<200	Jati	Tectona grandis L.f.	Lamiaceae / Verbenaceae	0,7	8,0	3	7	0,04	0,00	21,45	0,02	0,25	0,92
2081	42	1	Banjaranyar	<200	Jati	Tectona grandis L.f.	Lamiaceae / Verbenaceae	0,7	23,2	8	12	0,04	0,04	250,25	0,25	2,94	10,79
2082	43	2	Banjaranyar	<200	Mahoni	Swietenia macrophylla King	Meliaceae	0,61	4,8	3	4	0,01	0,00	4,44	0,00	0,21	0,77
2083	43	2	Banjaranyar	<200	Jati	Tectona grandis L.f.	Lamiaceae / Verbenaceae	0,7	8,6	6	9	0,01	0,00	31,09	0,03	1,46	5,36
2084	43	2	Banjaranyar	<200	Jati	Tectona grandis L.f.	Lamiaceae / Verbenaceae	0,7	5,4	3	4	0,01	0,00	6,34	0,01	0,30	1,09
2085	43	2	Banjaranyar	<200	Mahoni	Swietenia macrophylla King	Meliaceae	0,61	3,2	3	4	0,01	0,00	2,11	0,00	0,10	0,36
2086	43	2	Banjaranyar	<200	Jati	Tectona grandis L.f.	Lamiaceae / Verbenaceae	0,7	10,2	3	7	0,01	0,00	33,71	0,03	1,58	5,81
2087	43	2	Banjaranyar	<200	Mahoni	Swietenia macrophylla King	Meliaceae	0,61	15,0	4	7	0,01	0,00	60,10	0,06	2,82	10,37
2088	43	2	Banjaranyar	<200	Mahoni	Swietenia macrophylla King	Meliaceae	0,61	3,8	3	4	0,01	0,00	2,95	0,00	0,14	0,51
2089	43	2	Banjaranyar	<200	Mahoni	Swietenia macrophylla King	Meliaceae	0,61	3,5	3	4,5	0,01	0,00	2,80	0,00	0,13	0,48
2090	43	2	Banjaranyar	<200	Jati	Tectona grandis L.f.	Lamiaceae / Verbenaceae	0,7	8,6	4	6	0,01	0,00	21,44	0,02	1,01	3,70
2091	43	2	Banjaranyar	<200	Mahoni	Swietenia macrophylla King	Meliaceae	0,61	4,8	4	5	0,01	0,00	5,45	0,01	0,26	0,94
2092	43	2	Banjaranyar	<200	Mahoni	Swietenia macrophylla King	Meliaceae	0,61	6,4	3	5	0,01	0,00	9,23	0,01	0,43	1,59
2093	43	2	Banjaranyar	<200	Jati	Tectona grandis L.f.	Lamiaceae / Verbenaceae	0,7	12,7	3	5	0,01	0,00	37,28	0,04	1,75	6,43
2094	43	2	Banjaranyar	<200	Mahoni	Swietenia macrophylla King	Meliaceae	0,61	11,5	4	8	0,01	0,00	41,67	0,04	1,96	7,19
2095	43	2	Banjaranyar	<200	Jati	Tectona grandis L.f.	Lamiaceae / Verbenaceae	0,7	8,3	6	7	0,01	0,00	23,04	0,02	1,08	3,97
2096	43	2	Banjaranyar	<200	Sengon	Paraserianthes falcataria (L.) I.C.	Leguminosae / Fabaceae	0,33	2,2	3	3,5	0,01	0,00	0,55	0,00	0,03	0,10
2097	43	2	Banjaranyar	<200	Heuras	Vitex pubescens Vahl	Lamiaceae / Verbenaceae	0,88	4,1	4	5	0,01	0,00	5,86	0,01	0,28	1,01
2098	43	2	Banjaranyar	<200	Jati	Tectona grandis L.f.	Lamiaceae / Verbenaceae	0,7	7,6	5	7	0,01	0,00	19,90	0,02	0,94	3,43
2099	43	2	Banjaranyar	<200	Mahoni	Swietenia macrophylla King	Meliaceae	0,61	11,8	4	7	0,01	0,00	38,77	0,04	1,82	6,69
2100	43	2	Banjaranyar	<200	Mahoni	Swietenia macrophylla King	Meliaceae	0,61	6,7	3	6	0,01	0,00	11,93	0,01	0,56	2,06
2101	43	2	Banjaranyar	<200	Jati	Tectona grandis L.f.	Lamiaceae / Verbenaceae	0,7	7,6	5	7	0,01	0,00	19,90	0,02	0,94	3,43
2102	43	2	Banjaranyar	<200	Jati	Tectona grandis L.f.	Lamiaceae / Verbenaceae	0,7	6,0	4	6	0,01	0,00	11,26	0,01	0,53	1,94

2103	43	2	Banjaranyar	<200	Jati	Tectona grandis L.f.	Lamiaceae / Verbenaceae	0,7	4,8	3	5	0,01	0,00	6,18	0,01	0,29	1,07
2104	43	2	Banjaranyar	<200	Mahoni	Swietenia macrophylla King	Meliaceae	0,61	2,5	1,8	2	0,01	0,00	0,74	0,00	0,03	0,13
2105	43	2	Banjaranyar	<200	Mahoni	Swietenia macrophylla King	Meliaceae	0,61	14,0	5	9	0,01	0,00	67,05	0,07	3,15	11,56
2106	43	2	Banjaranyar	<200	Mahoni	Swietenia macrophylla King	Meliaceae	0,61	3,8	3	4	0,01	0,00	2,95	0,00	0,14	0,51
2107	43	2	Banjaranyar	<200	Jati	Tectona grandis L.f.	Lamiaceae / Verbenaceae	0,7	3,2	2	3	0,01	0,00	1,84	0,00	0,09	0,32
2108	43	2	Banjaranyar	<200	Jati	Tectona grandis L.f.	Lamiaceae / Verbenaceae	0,7	4,8	3	5	0,01	0,00	6,18	0,01	0,29	1,07
2109	43	2	Banjaranyar	<200	Mahoni	Swietenia macrophylla King	Meliaceae	0,61	5,4	3	4	0,01	0,00	5,59	0,01	0,26	0,96
2110	43	2	Banjaranyar	<200	Jati	Tectona grandis L.f.	Lamiaceae / Verbenaceae	0,7	3,2	2	3	0,01	0,00	1,84	0,00	0,09	0,32
2111	43	2	Banjaranyar	<200	Jati	Tectona grandis L.f.	Lamiaceae / Verbenaceae	0,7	11,5	3	9	0,01	0,00	52,66	0,05	2,47	9,08
2112	43	2	Banjaranyar	<200	Jati	Tectona grandis L.f.	Lamiaceae / Verbenaceae	0,7	7,6	4	8	0,01	0,00	22,49	0,02	1,06	3,88
2113	43	2	Banjaranyar	<200	Jati	Tectona grandis L.f.	Lamiaceae / Verbenaceae	0,7	7,0	2	5	0,01	0,00	12,47	0,01	0,59	2,15
2114	43	2	Banjaranyar	<200	Tisuk	Hibiscus macrophyllus Roxb. ex I	Malvaceae	0,4	9,5	7	9	0,01	0,00	22,58	0,02	1,06	3,90
2115	43	2	Banjaranyar	<200	Jati	Tectona grandis L.f.	Lamiaceae / Verbenaceae	0,7	7,0	5	7	0,01	0,00	16,97	0,02	0,80	2,93
2116	43	2	Banjaranyar	<200	Jati	Tectona grandis L.f.	Lamiaceae / Verbenaceae	0,7	5,7	4	7	0,01	0,00	11,75	0,01	0,55	2,03
2117	43	2	Banjaranyar	<200	Tisuk	Hibiscus macrophyllus Roxb. ex I	Malvaceae	0,4	3,5	4	5	0,01	0,00	2,10	0,00	0,10	0,36
2118	43	2	Banjaranyar	<200	Sengon	Paraserianthes falcataria (L.) I.C.	Leguminosae / Fabaceae	0,33	13,4	5	11	0,01	0,00	42,15	0,04	1,98	7,27
2119	43	2	Banjaranyar	<200	Jati	Tectona grandis L.f.	Lamiaceae / Verbenaceae	0,7	6,4	2	4	0,01	0,00	8,53	0,01	0,40	1,47
2120	43	2	Banjaranyar	<200	Sengon	Paraserianthes falcataria (L.) I.C.	Leguminosae / Fabaceae	0,33	24,8	9	15	0,04	0,05	174,06	0,17	2,05	7,51
2121	43	2	Banjaranyar	<200	Sengon	Paraserianthes falcataria (L.) I.C.	Leguminosae / Fabaceae	0,33	20,7	9	14	0,04	0,03	117,00	0,12	1,37	5,05
2122	43	2	Banjaranyar	<200	Jati	Tectona grandis L.f.	Lamiaceae / Verbenaceae	0,7	19,1	7	11	0,04	0,03	161,33	0,16	1,90	6,96
2123	43	2	Banjaranyar	<200	Sengon	Paraserianthes falcataria (L.) I.C.	Leguminosae / Fabaceae	0,33	20,4	9	14	0,04	0,03	113,72	0,11	1,34	4,90
2124	43	2	Banjaranyar	<200	Mahoni	Swietenia macrophylla King	Meliaceae	0,61	19,1	7	11	0,04	0,03	142,22	0,14	1,67	6,13
2125	43	2	Banjaranyar	<200	Jati	Tectona grandis L.f.	Lamiaceae / Verbenaceae	0,7	15,3	3	10	0,04	0,02	98,23	0,10	1,15	4,24
2126	43	2	Banjaranyar	<200	Mahoni	Swietenia macrophylla King	Meliaceae	0,61	10,8	5	9	0,04	0,01	41,81	0,04	0,49	1,80
2127	43	2	Banjaranyar	<200	Jati	Tectona grandis L.f.	Lamiaceae / Verbenaceae	0,7	11,1	4	8	0,04	0,01	44,89	0,04	0,53	1,94
2128	43	2	Banjaranyar	<200	Jati	Tectona grandis L.f.	Lamiaceae / Verbenaceae	0,7	12,7	4	8	0,04	0,01	57,34	0,06	0,67	2,47
2129	43	2	Banjaranyar	<200	Jati	Tectona grandis L.f.	Lamiaceae / Verbenaceae	0,7	12,4	5	7	0,04	0,01	48,44	0,05	0,57	2,09
2130	43	2	Banjaranyar	<200	Mahoni	Swietenia macrophylla King	Meliaceae	0,61	11,1	4	8	0,04	0,01	39,58	0,04	0,47	1,71
2131	43	2	Banjaranyar	<200	Tisuk	Hibiscus macrophyllus Roxb. ex I	Malvaceae	0,4	11,5	5	10	0,04	0,01	34,73	0,03	0,41	1,50
2132	43	2	Banjaranyar	<200	Mahoni	Swietenia macrophylla King	Meliaceae	0,61	13,0	5	8	0,04	0,01	52,88	0,05	0,62	2,28
2133	43	2	Banjaranyar	<200	Jati	Tectona grandis L.f.	Lamiaceae / Verbenaceae	0,7	11,1	3,5	4,5	0,04	0,01	26,50	0,03	0,31	1,14
2134	43	2	Banjaranyar	<200	Jati	Tectona grandis L.f.	Lamiaceae / Verbenaceae	0,7	11,1	4	9	0,04	0,01	50,01	0,05	0,59	2,16
2135	44	2	Banjaranyar	<200	Karet	Hevea brasiliensis (Willd. ex A.Ju	Euphorbiaceae	0,53	11,8	9	13	0,01	0,00	60,10	0,06	2,82	10,37
2136	44	2	Banjaranyar	<200	Karet	Hevea brasiliensis (Willd. ex A.Ju	Euphorbiaceae	0,53	6,4	3	8	0,01	0,00	12,48	0,01	0,59	2,15
2137	44	2	Banjaranyar	<200	Sengon	Paraserianthes falcataria (L.) I.C.	Leguminosae / Fabaceae	0,33	24,5	10	14	0,01	0,01	159,58	0,16	7,50	27,53
2138	44	2	Banjaranyar	<200	Karet	Hevea brasiliensis (Willd. ex A.Ju	Euphorbiaceae	0,53	5,1	5	7	0,01	0,00	7,34	0,01	0,34	1,27
2139	44	2	Banjaranyar	<200	Karet	Hevea brasiliensis (Willd. ex A.Ju	Euphorbiaceae	0,53	12,1	9	11	0,01	0,00	54,15	0,05	2,55	9,34
2140	44	2	Banjaranyar	<200	Sengon	Paraserianthes falcataria (L.) I.C.	Leguminosae / Fabaceae	0,33	6,4	5	7	0,01	0,00	7,16	0,01	0,34	1,23
2141	44	2	Banjaranyar	<200	Sengon	Paraserianthes falcataria (L.) I.C.	Leguminosae / Fabaceae	0,33	11,8	10	14	0,01	0,00	41,68	0,04	1,96	7,19
2142	44	2	Banjaranyar	<200	Jati	Tectona grandis L.f.	Lamiaceae / Verbenaceae	0,7	6,4	4	6	0,01	0,00	12,37	0,01	0,58	2,13
2143	44	2	Banjaranyar	<200	Sengon	Paraserianthes falcataria (L.) I.C.	Leguminosae / Fabaceae	0,33	14,0	9	12	0,01	0,00	49,71	0,05	2,34	8,57
2144	44	2	Banjaranyar	<200	Mahoni	Swietenia macrophylla King	Meliaceae	0,61	9,5	6	9	0,01	0,00	33,24	0,03	1,56	5,73
2145	44	2	Banjaranyar	<200	Sengon	Paraserianthes falcataria (L.) I.C.	Leguminosae / Fabaceae	0,33	6,4	9	11	0,01	0,00	10,83	0,01	0,51	1,87
2146	44	2	Banjaranyar	<200	Karet	Hevea brasiliensis (Willd. ex A.Ju	Euphorbiaceae	0,53	9,9	5	9	0,01	0,00	31,03	0,03	1,46	5,35
2147	44	2	Banjaranyar	<200	Mahoni	Swietenia macrophylla King	Meliaceae	0,61	2,5	2	3	0,01	0,00	1,08	0,00	0,05	0,19
2148	44	2	Banjaranyar	<200	Sengon	Paraserianthes falcataria (L.) I.C.	Leguminosae / Fabaceae	0,33	14,3	12	16	0,01	0,00	67,41	0,07	3,17	11,63
2149	44	2	Banjaranyar	<200	Mahoni	Swietenia macrophylla King	Meliaceae	0,61	7,6	4	8	0,01	0,00	19,83	0,02	0,93	3,42
2150	44	2	Banjaranyar	<200	Karet	Hevea brasiliensis (Willd. ex A.Ju	Euphorbiaceae	0,53	14,3	6	10	0,01	0,00	67,65	0,07	3,18	11,67
2151	44	2	Banjaranyar	<200	Tisuk	Hibiscus macrophyllus Roxb. ex I	Malvaceae	0,4	8,9	8	10	0,01	0,00	21,92	0,02	1,03	3,78
2152	44	2	Banjaranyar	<200	Mahoni	Swietenia macrophylla King	Meliaceae	0,61	2,5	2	3	0,01	0,00	1,08	0,00	0,05	0,19
2153	44	2	Banjaranyar	<200	Karet	Hevea brasiliensis (Willd. ex A.Ju	Euphorbiaceae	0,53	10,8	3	8	0,01	0,00	33,00	0,03	1,55	5,69
2154	44	2	Banjaranyar	<200	Sengon	Paraserianthes falcataria (L.) I.C.	Leguminosae / Fabaceae	0,33	10,2	10	13	0,01	0,00	29,85	0,03	1,40	5,15
2155	44	2	Banjaranyar	<200	Mahoni	Swietenia macrophylla King	Meliaceae	0,61	3,2	3	4	0,01	0,00	2,11	0,00	0,10	0,36
2156	44	2	Banjaranyar	<200	Karet	Hevea brasiliensis (Willd. ex A.Ju	Euphorbiaceae	0,53	10,2	4	9	0,01	0,00	32,89	0,03	1,55	5,67
2157	44	2	Banjaranyar	<200	Mahoni	Swietenia macrophylla King	Meliaceae	0,61	2,5	2	3	0,01	0,00	1,08	0,00	0,05	0,19
2158	44	2	Banjaranyar	<200	Mahoni	Swietenia macrophylla King	Meliaceae	0,61	2,2	2	3	0,01	0,00	0,84	0,00	0,04	0,15
2159	44	2	Banjaranyar	<200	Sengon	Paraserianthes falcataria (L.) I.C.	Leguminosae / Fabaceae	0,33	10,2	8	12	0,01	0,00	27,74	0,03	1,30	4,78
2160	44	2	Banjaranyar	<200	Karet	Hevea brasiliensis (Willd. ex A.Ju	Euphorbiaceae	0,53	5,7	2	6	0,01	0,00	7,91	0,01	0,37	1,36
2161	44	2	Banjaranyar	<200	Sengon	Paraserianthes falcataria (L.) I.C.	Leguminosae / Fabaceae	0,33	20,7	11	15	0,01	0,01	124,63	0,12	5,86	21,50
2162	44	2	Banjaranyar	<200	Karet	Hevea brasiliensis (Willd. ex A.Ju	Euphorbiaceae	0,53	10,2	5	9	0,01	0,00	32,89	0,03	1,55	5,67
2163	44	2	Banjaranyar	<200	Mahoni	Swietenia macrophylla King	Meliaceae	0,61	3,5	2	3	0,01	0,00	1,93	0,00	0,09	0,33
2164	44	2	Banjaranyar	<200	Mahoni	Swietenia macrophylla King	Meliaceae	0,61	3,8	3	5	0,01	0,00	3,62	0,00	0,17	0,62
2165	44	2	Banjaranyar	<200	Karet	Hevea brasiliensis (Willd. ex A.Ju	Euphorbiaceae	0,53	20,4	7	10	0,04	0,03	128,97	0,13	1,52	5,56
2166	44	2	Banjaranyar	<200	Sengon	Paraserianthes falcataria (L.) I.C.	Leguminosae / Fabaceae	0,33	19,1	7	11	0,04	0,03	81,02	0,08	0,95	3,49
2167	44	2	Banjaranyar	<200	Sengon	Paraserianthes falcataria (L.) I.C.	Leguminosae / Fabaceae	0,33	22,3	9	14	0,04	0,04	134,01	0,13	1,57	5,78
2168	44	2	Banjaranyar	<200	Sengon	Paraserianthes falcataria (L.) I.C.	Leguminosae / Fabaceae	0,33	21,3	15	18	0,04	0,04	155,70	0,16	1,83	6,71
2169	44	2	Banjaranyar	<200	Karet	Hevea brasiliensis (Willd. ex A.Ju	Euphorbiaceae	0,53	19,4	6	11	0,04	0,03	128,88	0,13	1,51	5,56
2170	44	2	Banjaranyar	<200	Sengon	Paraserianthes falcataria (L.) I.C.	Leguminosae / Fabaceae	0,33	19,1	10	15	0,04	0,03	107,63	0,11	1,26	4,64
2171	44	2	Banjaranyar	<200	Sengon	Paraserianthes falcataria (L.) I.C.	Leguminosae / Fabaceae	0,33	19,7	12	16	0,04	0,03	121,26	0,12	1,42	5,23
2172	44	2	Banjaranyar	<200	Karet	Hevea brasiliensis (Willd. ex A.Ju	Euphorbiaceae	0,53	20,4	10	15	0,04	0,03	186,97	0,19	2,20	8,06
2173	44	2	Banjaranyar	<200	Kelapa	Cocos nucifera L.	Arecaceae	0,5	31,8	12	16	0,04	0,08	127,70	0,13	1,50	5,51
2174	44	2	Banjaranyar	<200	Sengon	Paraserianthes falcataria (L.) I.C.	Leguminosae / Fabaceae	0,33	20,7	12	17	0,04	0,03	139,77	0,14	1,64	6,03
2175	44	2	Banjaranyar	<200	Karet	Hevea brasiliensis (Willd. ex A.Ju	Euphorbiaceae	0,53	20,7	10	14	0,04	0,03	180,58	0,18	2,12	7,79
2176	44	2	Banjaranyar	<200	Tisuk	Hibiscus macrophyllus Roxb. ex I	Malvaceae	0,4	17,2	7	10	0,04	0,02	73,00	0,07	0,86	3,15
2177	44	2	Banjaranyar	<200	Mahoni	Swietenia macrophylla King	Meliaceae	0,61	13,0	6	11	0,04	0,01	70,80	0,07	0,83	3,05
2178	44	2	Banjaranyar	<200	Karet	Hevea brasiliensis (Willd. ex A.Ju	Euphorbiaceae	0,53	10,2	5	8	0,04	0,01	29,53	0,03	0,35	1,27
2179	44	2	Banjaranyar	<200	Tisuk	Hibiscus macrophyllus Roxb. ex I	Malvaceae	0,4	11,1	7	10	0,04	0,01	32,99	0,03	0,39	1,42
2180	44	2	Banjaranyar	<200	Karet	Hevea brasiliensis (Willd. ex A.Ju	Euphorbiaceae	0,53	10,2	8	11	0,04	0,01	39,53	0,04	0,46	1,70
2181	44	2	Banjaranyar	<200	Tisuk	Hibiscus macrophyllus Roxb. ex I	Malvaceae	0,4	15,9	9	14	0,04	0,02	86,29	0,09	1,01	3,72
2182	44	2	Banjaranyar	<200	Karet	Hevea brasiliensis (Willd. ex A.Ju	Euphorbiaceae	0,53	14,0	4	10	0,04	0,02	64,92	0,06	0,76	2,80
2183	44	2	Banjaranyar	<200	Karet	Hevea brasiliensis (Willd. ex A.Ju	Euphorbiaceae	0,53	10,2	5	8	0,04	0,01	29,53	0,03	0,35	1,27
2184	44	2	Banjaranyar	<200	Sengon	Paraserianthes falcataria (L.) I.C.	Leguminosae / Fabaceae	0,33	13,0	10	12	0,04	0,01	43,67	0,04	0,51	1,88
2185	44	2	Banjaranyar	<200	Karet	Hevea brasiliensis (Willd. ex A.Ju	Euphorbiaceae	0,53	16,5	7	12	0,04	0,02	104,18	0,10	1,22	4,49
2186	44	2	Banjaranyar	<200	Sengon	Paraserianthes falcataria (L.) I.C.	Leguminosae / Fabaceae	0,33	15,9	10	14	0,04	0,02	72,35	0,07	0,85	3,12
2187	44	2	Banjaranyar	<200	Karet	Hevea brasiliensis (Willd. ex A.Ju	Euphorbiaceae	0,53	17,5	9	14	0,04	0,02	132,97	0,13	1,56	5,73
2188	44	2	Banjaranyar	<200	Sengon	Paraserianthes falcataria (L.) I.C.	Leguminosae / Fabaceae	0,33	14,3	10	14	0,04	0,02	59,65	0,06	0,70	2,57
2189	44	2	Banjaranyar	<200	Karet	Hevea brasiliensis (Willd. ex A.Ju	Euphorbiaceae	0,53	11,8	5	9	0,04	0,01	42,91	0,04	0,50	1,85
2190	44	2	Banjaranyar	<200	Kelapa	Cocos nucifera L.	Arecaceae	0,5	14,6	4	8	0,04	0,02	66,10	0,07	0,78	2,85
2191	44	2	Banjaranyar	<200	Karet	Hevea brasiliensis (Willd. ex A.Ju	Euphorbiaceae	0,53	10,2	4	7	0,04	0,01	26,13	0,03	0,31	1,13
2192	44	2	Banjaranyar	<200	Sengon	Paraserianthes falcataria (L.) I.C.	Leguminosae / Fabaceae	0,33	17,2	9	12	0,04	0,02	72,34	0,07	0,85	3,12
2193	44	2	Banjaranyar	<200	Karet	Hevea brasiliensis (Willd. ex A.Ju	Euphorbiaceae	0,53	11,8	5	10	0,04	0,01	47,26	0,05	0,56	2,04
2194	44	2	Banjaranyar	<200	Karet	Hevea brasiliensis (Willd. ex A.Ju	Euphorbiaceae	0,53	14,3	6	12	0,04	0,02	79,94	0,08	0,94	3,45
2195	44	2	Banjaranyar	<200	Karet	Hevea brasiliensis (Willd. ex A.Ju	Euphorbiaceae	0,53	15,9	7	11	0,04	0,02	89,53	0,09	1,05	3,86
2196	44	2	Banjaranyar	<200	Sengon	Paraserianthes falcataria (L.) I.C.	Leguminosae / Fabaceae	0,33	17,5	10	14	0,04	0,02	86,15	0,09	1,01	3,72
2197	45	3	Banjaranyar	<200	Jati	Tectona grandis L.f.	Lamiaceae / Verbenaceae	0,7	3,2	2	3	0,04	0,00	1,84	0,00	0,02	0,08
2198	45	3	Banjaranyar	<200	Sengon	Paraserianthes falcataria (L.) I.C.	Leguminosae / Fabaceae	0,33	2,2	2	3	0,04	0,00	0,48	0,00	0,01	0,02
2199	45	3	Banjaranyar	<200	Jati	Tectona grandis L.f.	Lamiaceae / Verbenaceae	0,7	6,4	3	5	0,04	0,00	10,47	0,01	0,12	0,45
2200	45	3	Banjaranyar	<200	Jati	Tectona grandis L.f.	Lamiaceae / Verbenaceae	0,7	3,8	2	3	0,04	0,00	2,57	0,00	0,03	0,11
2201	45	3	Banjaranyar	<200	Jati	Tectona grandis L.f.	Lamiaceae / Verbenaceae	0,7	10,8	4	7	0,04	0,01	37,67	0,04	0,44	1,62
2202	45	3	Banjaranyar	<200	Jati	Tectona grandis L.f.	Lamiaceae / Verbenaceae	0,7	8,9	5	7	0,04	0,01	26,40	0,03	0,31	1,14
2203	45	3	Banjaranyar	<200	Tisuk	Hibiscus macrophyllus Roxb. ex I	Malvaceae	0,4	2,5	2	2,5	0,04	0,00	0,62	0,00	0,01	0,03
2204	45	3	Banjaranyar	<200	Jati	Tectona grandis L.f.	Lamiaceae / Verbenaceae	0,7	3,8	2	3	0,04	0,00	2,57	0,00	0,03	0,11
2205	45	3	Banjaranyar	<200	Jati	Tectona grandis L.f.	Lamiaceae / Verbenaceae	0,7	3,5	3	4	0,04	0,00	2,85	0,00	0,03	0,12
2206	45	3	Banjaranyar	<200	Jati	Tectona grandis L.f.	Lamiaceae / Verbenaceae	0,7	5,4	4	6	0,04	0,00	9,19	0,01	0,11	0,40
2207	45	3	Banjaranyar	<200	Jati	Tectona grandis L.f.	Lamiaceae / Verbenaceae	0,7	5,1	3	5	0,04	0,00	6,96	0,01	0,08	0,30
2208	45	3	Banjaranyar	<200	Tisuk	Hibiscus macrophyllus Roxb. ex I	Malvaceae	0,4	8,0	3	7	0,04	0,00	12,85	0,01	0,15	0,55
2209	45	3	Banjaranyar	<200	Kelapa	Cocos nucifera L.	Arecaceae	0,5	22,9	8	12	0,04	0,04	96,90	0,10	1,14	4,18
2210	45	3	Banjaranyar	<200	Tisuk	Hibiscus macrophyllus Roxb. ex I	Malvaceae	0,4	4,1	3	4	0,04	0,00	2,32	0,00	0,03	0,10
2211	45	3	Banjaranyar	<200	Jati	Tectona grandis L.f.	Lamiaceae / Verbenaceae	0,7	7,0	3	6	0,04	0,00	14,73	0,01	0,17	0,64
2212	45	3	Banjaranyar	<200	Jati	Tectona grandis L.f.	Lamiaceae / Verbenaceae	0,7	7,3	3	6	0,04	0,00	15,98	0,02	0,19	0,69
2213	45	3	Banjaranyar	<200	Jati	Tectona grandis L.f.	Lamiaceae / Verbenaceae	0,7	6,4	2,5	4	0,04	0,00	8,53	0,01	0,10	0,37
2214	45	3	Banjaranyar	<200	Tisuk	Hibiscus macrophyllus Roxb. ex I	Malvaceae	0,4	33,7	11	18	0,04	0,09	430,32	0,43	5,06	18,56
2215	45	3	Banjaranyar	<200	Tisuk	Hibiscus macrophyllus Roxb. ex I	Malvaceae	0,4	2,5	3	5	0,04	0,00	1,17	0,00	0,01	0,05
2216	45	3	Banjaranyar	<200	Tisuk	Hibiscus macrophyllus Roxb. ex I	Malvaceae	0,4	4,1	4	5	0,04	0,00	2,85	0,00	0,03	0,12
2217	45	3	Banjaranyar	<200	Tisuk	Hibiscus macrophyllus Roxb. ex I	Malvaceae	0,4	4,1	3	5	0,04	0,00	2,85	0,00	0,03	0,12
2218	45	3	Banjaranyar	<200	Jati	Tectona grandis L.f.	Lamiaceae / Verbenaceae	0,7	11,1	3	7	0,04	0,01	39,73	0,04	0,47	1,71
2219	45	3	Banjaranyar	<200	Mahoni	Swietenia macrophylla King	Meliaceae	0,61	9,5	2,5	7	0,04	0,01	26,40	0,03	0,31	1,14
2220	45	3	Banjaranyar	<200	Manglid	Magnolia champaca (L.) Baill. ex	Magnoliaceae	0,53	6,4	1,8	4	0,04	0,00	6,62	0,01	0,08	0,29
2221	45	3	Banjaranyar	<200	Jati	Tectona grandis L.f.	Lamiaceae / Verbenaceae	0,7	3,8	2	5	0,04	0,00	4,11	0,00	0,05	0,18
2222	45	3	Banjaranyar	<200	Manglid	Magnolia champaca (L.) Baill. ex	Magnoliaceae	0,53	5,1	2	3	0,04	0,00	3,38	0,00	0,04	0,15
2223	45	3	Banjaranyar	<200	Jati	Tectona grandis L.f.	Lamiaceae / Verbenaceae	0,7	8,9	2,5	9	0,04	0,01	33,23	0,03	0,39	1,43
2224	45	3	Banjaranyar	<200	Jati	Tectona grandis L.f.	Lamiaceae / Verbenaceae	0,7	8,6	4	8	0,04	0,01	27,91	0,03	0,33	1,20
2225	45	3	Banjaranyar	<200	mahoni	Swietenia macrophylla King	Meliaceae	0,61	14,0	6	10	0,04	0,02	73,84	0,07	0,87	3,18
2226	46	2	Banjaranyar	<200	Jati	Tectona grandis L.f.	Lamiaceae / Verbenaceae	0,7	8,0	4	6	0,01	0,00	18,62	0,02	0,88	3,21

2227	46	2	Banjaranyar	<200	Jati	Tectona grandis L.f.	Lamiaceae / Verbenaceae	0,7	5,1	3	4	0,01	0,00	5,67	0,01	0,27	0,98
2228	46	2	Banjaranyar	<200	Jati	Tectona grandis L.f.	Lamiaceae / Verbenaceae	0,7	2,9	2	3	0,01	0,00	1,52	0,00	0,07	0,26
2229	46	2	Banjaranyar	<200	Tisuk	Hibiscus macrophyllus Roxb. ex	Malvaceae	0,4	2,9	3	4	0,01	0,00	1,18	0,00	0,06	0,20
2230	46	2	Banjaranyar	<200	Jati	Tectona grandis L.f.	Lamiaceae / Verbenaceae	0,7	3,5	3	4	0,01	0,00	2,85	0,00	0,13	0,49
2231	46	2	Banjaranyar	<200	Jati	Tectona grandis L.f.	Lamiaceae / Verbenaceae	0,7	3,5	2,5	3,5	0,01	0,00	2,53	0,00	0,12	0,44
2232	46	2	Banjaranyar	<200	Jati	Tectona grandis L.f.	Lamiaceae / Verbenaceae	0,7	3,8	2	2,5	0,01	0,00	2,18	0,00	0,10	0,38
2233	46	2	Banjaranyar	<200	Tisuk	Hibiscus macrophyllus Roxb. ex	Malvaceae	0,4	8,9	4	7	0,01	0,00	15,81	0,02	0,74	2,73
2234	46	2	Banjaranyar	<200	Jati	Tectona grandis L.f.	Lamiaceae / Verbenaceae	0,7	7,6	4	7	0,01	0,00	19,90	0,02	0,94	3,43
2235	46	2	Banjaranyar	<200	Tisuk	Hibiscus macrophyllus Roxb. ex	Malvaceae	0,4	13,4	5	8	0,01	0,00	37,55	0,04	1,76	6,48
2236	46	2	Banjaranyar	<200	Jati	Tectona grandis L.f.	Lamiaceae / Verbenaceae	0,7	7,6	3	5	0,01	0,00	14,62	0,01	0,69	2,52
2237	46	2	Banjaranyar	<200	Jati	Tectona grandis L.f.	Lamiaceae / Verbenaceae	0,7	5,4	2	4	0,01	0,00	6,34	0,01	0,30	1,09
2238	46	2	Banjaranyar	<200	Tisuk	Hibiscus macrophyllus Roxb. ex	Malvaceae	0,4	2,2	2	2,5	0,01	0,00	0,49	0,00	0,02	0,08
2239	46	2	Banjaranyar	<200	Tisuk	Hibiscus macrophyllus Roxb. ex	Malvaceae	0,4	2,5	3	5	0,01	0,00	1,17	0,00	0,06	0,20
2240	46	2	Banjaranyar	<200	Jati	Tectona grandis L.f.	Lamiaceae / Verbenaceae	0,7	6,0	2,5	5	0,01	0,00	9,53	0,01	0,45	1,64
2241	46	2	Banjaranyar	<200	Jati	Tectona grandis L.f.	Lamiaceae / Verbenaceae	0,7	3,5	4	4,5	0,01	0,00	3,18	0,00	0,15	0,55
2242	46	2	Banjaranyar	<200	Jati	Tectona grandis L.f.	Lamiaceae / Verbenaceae	0,7	5,7	3	6	0,01	0,00	10,20	0,01	0,48	1,76
2243	46	2	Banjaranyar	<200	Jati	Tectona grandis L.f.	Lamiaceae / Verbenaceae	0,7	4,1	2	4,5	0,01	0,00	4,32	0,00	0,20	0,74
2244	46	2	Banjaranyar	<200	Tisuk	Hibiscus macrophyllus Roxb. ex	Malvaceae	0,4	2,9	2	3	0,01	0,00	0,91	0,00	0,04	0,16
2245	46	2	Banjaranyar	<200	Jati	Tectona grandis L.f.	Lamiaceae / Verbenaceae	0,7	2,5	7	2,5	0,01	0,00	1,04	0,00	0,05	0,18
2246	46	2	Banjaranyar	<200	Mahoni	Swietenia macrophylla King	Meliaceae	0,61	14,6	5	10	0,01	0,00	80,10	0,08	3,76	13,82
2247	46	2	Banjaranyar	<200	Jati	Tectona grandis L.f.	Lamiaceae / Verbenaceae	0,7	17,5	5	9	0,01	0,01	114,46	0,11	5,38	19,74
2248	46	2	Banjaranyar	<200	Jati	Tectona grandis L.f.	Lamiaceae / Verbenaceae	0,7	10,2	4	8	0,01	0,00	38,10	0,04	1,79	6,57
2249	46	2	Banjaranyar	<200	Jati	Tectona grandis L.f.	Lamiaceae / Verbenaceae	0,7	5,7	5	5,5	0,01	0,00	9,42	0,01	0,44	1,62
2250	46	2	Banjaranyar	<200	Mahoni	Swietenia macrophylla King	Meliaceae	0,61	7,6	5	6	0,01	0,00	15,23	0,02	0,72	2,63
2251	46	2	Banjaranyar	<200	Jati	Tectona grandis L.f.	Lamiaceae / Verbenaceae	0,7	2,5	2	2,5	0,01	0,00	1,04	0,00	0,05	0,18
2252	46	2	Banjaranyar	<200	Jati	Tectona grandis L.f.	Lamiaceae / Verbenaceae	0,7	4,5	2,5	3,5	0,01	0,00	3,93	0,00	0,18	0,68
2253	46	2	Banjaranyar	<200	Jati	Tectona grandis L.f.	Lamiaceae / Verbenaceae	0,7	4,8	3	5	0,01	0,00	6,18	0,01	0,29	1,07
2254	46	2	Banjaranyar	<200	Jati	Tectona grandis L.f.	Lamiaceae / Verbenaceae	0,7	4,1	2,5	3,5	0,01	0,00	3,43	0,00	0,16	0,59
2255	46	2	Banjaranyar	<200	Jati	Tectona grandis L.f.	Lamiaceae / Verbenaceae	0,7	2,5	3	3,5	0,01	0,00	1,41	0,00	0,07	0,24
2256	46	2	Banjaranyar	<200	Jati	Tectona grandis L.f.	Lamiaceae / Verbenaceae	0,7	3,2	2	2,5	0,01	0,00	1,56	0,00	0,07	0,27
2257	46	2	Banjaranyar	<200	Mahoni	Swietenia macrophylla King	Meliaceae	0,61	8,9	5	7	0,01	0,00	23,27	0,02	1,09	4,01
2258	46	2	Banjaranyar	<200	Jati	Tectona grandis L.f.	Lamiaceae / Verbenaceae	0,7	18,1	5	9	0,01	0,01	122,20	0,12	5,74	21,08
2259	46	2	Banjaranyar	<200	Jati	Tectona grandis L.f.	Lamiaceae / Verbenaceae	0,7	2,5	2	2,5	0,01	0,00	1,04	0,00	0,05	0,18
2260	46	2	Banjaranyar	<200	Mahoni	Swietenia macrophylla King	Meliaceae	0,61	7,6	5	8	0,01	0,00	19,83	0,02	0,93	3,42
2261	46	2	Banjaranyar	<200	Jati	Tectona grandis L.f.	Lamiaceae / Verbenaceae	0,7	4,1	3	4	0,01	0,00	3,88	0,00	0,18	0,67
2262	46	2	Banjaranyar	<200	Heuras	Vitex pubescens Vahl	Lamiaceae / Verbenaceae	0,88	8,3	5	7	0,01	0,00	28,42	0,03	1,34	4,90
2263	46	2	Banjaranyar	<200	Jati	Tectona grandis L.f.	Lamiaceae / Verbenaceae	0,7	4,5	2,5	4	0,01	0,00	4,44	0,00	0,21	0,77
2264	46	2	Banjaranyar	<200	Jati	Tectona grandis L.f.	Lamiaceae / Verbenaceae	0,7	2,5	1,8	2,5	0,01	0,00	1,04	0,00	0,05	0,18
2265	46	2	Banjaranyar	<200	Jati	Tectona grandis L.f.	Lamiaceae / Verbenaceae	0,7	5,1	3,5	5	0,01	0,00	6,96	0,01	0,33	1,20
2266	46	2	Banjaranyar	<200	Tisuk	Hibiscus macrophyllus Roxb. ex	Malvaceae	0,4	1,9	2	2,5	0,01	0,00	0,37	0,00	0,02	0,06
2267	46	2	Banjaranyar	<200	Jati	Tectona grandis L.f.	Lamiaceae / Verbenaceae	0,7	4,1	3	3,5	0,01	0,00	3,43	0,00	0,16	0,59
2268	46	2	Banjaranyar	<200	Jati	Tectona grandis L.f.	Lamiaceae / Verbenaceae	0,7	3,2	2	2,5	0,01	0,00	1,56	0,00	0,07	0,27
2269	46	2	Banjaranyar	<200	Tisuk	Hibiscus macrophyllus Roxb. ex	Malvaceae	0,4	2,2	2	2,5	0,01	0,00	0,49	0,00	0,02	0,08
2270	46	2	Banjaranyar	<200	Jati	Tectona grandis L.f.	Lamiaceae / Verbenaceae	0,7	3,2	2	3	0,01	0,00	1,84	0,00	0,09	0,32
2271	46	2	Banjaranyar	<200	Tisuk	Hibiscus macrophyllus Roxb. ex	Malvaceae	0,4	10,5	7	9	0,04	0,01	26,89	0,03	0,32	1,16
2272	46	2	Banjaranyar	<200	Jati	Tectona grandis L.f.	Lamiaceae / Verbenaceae	0,7	11,1	5	9	0,04	0,01	50,01	0,05	0,59	2,16
2273	46	2	Banjaranyar	<200	Mahoni	Swietenia macrophylla King	Meliaceae	0,61	10,5	7	11	0,04	0,01	47,57	0,05	0,56	2,05
2274	46	2	Banjaranyar	<200	Nangka	Artocarpus heterophyllus Lam.	Moraceae	0,61	10,2	4	5	0,04	0,01	21,84	0,02	0,26	0,94
2275	46	2	Banjaranyar	<200	Jati	Tectona grandis L.f.	Lamiaceae / Verbenaceae	0,7	11,1	6	9	0,04	0,01	50,01	0,05	0,59	2,16
2276	46	2	Banjaranyar	<200	Mahoni	Swietenia macrophylla King	Meliaceae	0,61	10,2	6	9	0,04	0,01	37,41	0,04	0,44	1,61
2277	46	2	Banjaranyar	<200	Tisuk	Hibiscus macrophyllus Roxb. ex	Malvaceae	0,4	12,7	9	14	0,04	0,01	57,34	0,06	0,67	2,47
2278	46	2	Banjaranyar	<200	Jati	Tectona grandis L.f.	Lamiaceae / Verbenaceae	0,7	16,9	4	11	0,04	0,02	128,53	0,13	1,51	5,54
2279	46	2	Banjaranyar	<200	Jati	Tectona grandis L.f.	Lamiaceae / Verbenaceae	0,7	15,0	9	17	0,04	0,02	153,68	0,15	1,81	6,63
2280	46	2	Banjaranyar	<200	Jati	Tectona grandis L.f.	Lamiaceae / Verbenaceae	0,7	7,0	5	7	0,04	0,00	16,97	0,02	0,20	0,73
2281	46	2	Banjaranyar	<200	Heuras	Vitex pubescens Vahl	Lamiaceae / Verbenaceae	0,88	10,2	5	8	0,04	0,01	46,98	0,05	0,55	2,03
2282	46	2	Banjaranyar	<200	Mahoni	Swietenia macrophylla King	Meliaceae	0,61	10,2	5	8	0,04	0,01	33,59	0,03	0,39	1,45
2283	46	2	Banjaranyar	<200	Tisuk	Hibiscus macrophyllus Roxb. ex	Malvaceae	0,4	10,2	6	9	0,04	0,01	25,42	0,03	0,30	1,10
2284	46	2	Banjaranyar	<200	Mahoni	Swietenia macrophylla King	Meliaceae	0,61	20,0	8	12	0,04	0,03	168,42	0,17	1,98	7,26
2285	46	2	Banjaranyar	<200	Jati	Tectona grandis L.f.	Lamiaceae / Verbenaceae	0,7	19,1	6	10	0,04	0,03	147,84	0,15	1,74	6,38
2286	46	2	Banjaranyar	<200	Mahoni	Swietenia macrophylla King	Meliaceae	0,61	19,4	7	11	0,04	0,03	146,60	0,15	1,72	6,32
2287	46	2	Banjaranyar	<200	Jati	Tectona grandis L.f.	Lamiaceae / Verbenaceae	0,7	19,1	5	10	0,04	0,03	147,84	0,15	1,74	6,38
2288	46	2	Banjaranyar	<200	Kelapa	Cocos nucifera L.	Arecaceae	0,5	23,5	12	16	0,04	0,04	127,70	0,13	1,50	5,51
2289	47	1	Banjaranyar	<200	jati	Tectona grandis L.f.	Lamiaceae / Verbenaceae	0,7	13,4	6	12	0,01	0,00	90,90	0,09	4,27	15,68
2290	47	1	Banjaranyar	<200	jati	Tectona grandis L.f.	Lamiaceae / Verbenaceae	0,7	8,0	6	10	0,01	0,00	29,73	0,03	1,40	5,13
2291	47	1	Banjaranyar	<200	jati	Tectona grandis L.f.	Lamiaceae / Verbenaceae	0,7	8,3	6	9	0,01	0,00	29,01	0,03	1,36	5,00
2292	47	1	Banjaranyar	<200	jati	Tectona grandis L.f.	Lamiaceae / Verbenaceae	0,7	11,8	6	10	0,01	0,00	60,98	0,06	2,87	10,52
2293	47	1	Banjaranyar	<200	jati	Tectona grandis L.f.	Lamiaceae / Verbenaceae	0,7	6,0	4	5	0,01	0,00	9,53	0,01	0,45	1,64
2294	47	1	Banjaranyar	<200	Tisuk	Hibiscus macrophyllus Roxb. ex	Malvaceae	0,4	15,6	9	12	0,01	0,00	72,21	0,07	3,39	12,45
2295	47	1	Banjaranyar	<200	jati	Tectona grandis L.f.	Lamiaceae / Verbenaceae	0,7	7,3	6	9	0,01	0,00	23,17	0,02	1,09	4,00
2296	47	1	Banjaranyar	<200	jati	Tectona grandis L.f.	Lamiaceae / Verbenaceae	0,7	8,9	8	11	0,01	0,00	39,93	0,04	1,88	6,89
2297	47	1	Banjaranyar	<200	jati	Tectona grandis L.f.	Lamiaceae / Verbenaceae	0,7	9,2	6	10	0,01	0,00	39,02	0,04	1,83	6,73
2298	47	1	Banjaranyar	<200	jati	Tectona grandis L.f.	Lamiaceae / Verbenaceae	0,7	9,5	6	9	0,01	0,00	37,71	0,04	1,77	6,50
2299	47	1	Banjaranyar	<200	jati	Tectona grandis L.f.	Lamiaceae / Verbenaceae	0,7	11,8	10	12	0,01	0,00	72,06	0,07	3,39	12,43
2300	47	1	Banjaranyar	<200	jati	Tectona grandis L.f.	Lamiaceae / Verbenaceae	0,7	9,9	9	11	0,01	0,00	48,12	0,05	2,26	8,30
2301	47	1	Banjaranyar	<200	jati	Tectona grandis L.f.	Lamiaceae / Verbenaceae	0,7	15,0	8	12	0,01	0,00	111,70	0,11	5,25	19,27
2302	47	1	Banjaranyar	<200	jati	Tectona grandis L.f.	Lamiaceae / Verbenaceae	0,7	11,8	7	11	0,01	0,00	66,54	0,07	3,13	11,48
2303	47	1	Banjaranyar	<200	jati	Tectona grandis L.f.	Lamiaceae / Verbenaceae	0,7	10,8	8	12	0,01	0,00	61,72	0,06	2,90	10,65
2304	47	1	Banjaranyar	<200	jati	Tectona grandis L.f.	Lamiaceae / Verbenaceae	0,7	12,4	9	12	0,01	0,00	79,36	0,08	3,73	13,69
2305	47	1	Banjaranyar	<200	jati	Tectona grandis L.f.	Lamiaceae / Verbenaceae	0,7	12,7	8	11	0,01	0,00	76,76	0,08	3,61	13,24
2306	47	1	Banjaranyar	<200	jati	Tectona grandis L.f.	Lamiaceae / Verbenaceae	0,7	9,9	7	10	0,01	0,00	44,10	0,04	2,07	7,61
2307	47	1	Banjaranyar	<200	jati	Tectona grandis L.f.	Lamiaceae / Verbenaceae	0,7	10,2	5	9	0,01	0,00	42,44	0,04	1,99	7,32
2308	47	1	Banjaranyar	<200	jati	Tectona grandis L.f.	Lamiaceae / Verbenaceae	0,7	8,0	4	8	0,01	0,00	24,24	0,02	1,14	4,18
2309	47	1	Banjaranyar	<200	jati	Tectona grandis L.f.	Lamiaceae / Verbenaceae	0,7	12,1	8	11	0,01	0,00	69,87	0,07	3,28	12,05
2310	47	1	Banjaranyar	<200	jati	Tectona grandis L.f.	Lamiaceae / Verbenaceae	0,7	11,8	6	12	0,01	0,00	72,06	0,07	3,39	12,43
2311	47	1	Banjaranyar	<200	jati	Tectona grandis L.f.	Lamiaceae / Verbenaceae	0,7	13,0	7	11	0,01	0,00	80,31	0,08	3,77	13,85
2312	47	1	Banjaranyar	<200	jati	Tectona grandis L.f.	Lamiaceae / Verbenaceae	0,7	7,6	7	9	0,01	0,00	25,05	0,03	1,18	4,32
2313	47	1	Banjaranyar	<200	jati	Tectona grandis L.f.	Lamiaceae / Verbenaceae	0,7	8,0	7	10	0,01	0,00	29,73	0,03	1,40	5,13
2314	47	1	Banjaranyar	<200	jati	Tectona grandis L.f.	Lamiaceae / Verbenaceae	0,7	11,1	7	11	0,01	0,00	60,10	0,06	2,82	10,37
2315	47	1	Banjaranyar	<200	jati	Tectona grandis L.f.	Lamiaceae / Verbenaceae	0,7	12,7	9	13	0,01	0,00	89,45	0,09	4,20	15,43
2316	47	1	Banjaranyar	<200	jati	Tectona grandis L.f.	Lamiaceae / Verbenaceae	0,7	11,5	9	13	0,01	0,00	73,75	0,07	3,47	12,72
2317	47	1	Banjaranyar	<200	jati	Tectona grandis L.f.	Lamiaceae / Verbenaceae	0,7	11,5	8	11	0,01	0,00	63,28	0,06	2,97	10,92
2318	47	1	Banjaranyar	<200	jati	Tectona grandis L.f.	Lamiaceae / Verbenaceae	0,7	10,5	6	9	0,01	0,00	44,90	0,04	2,11	7,74
2319	47	1	Banjaranyar	<200	jati	Tectona grandis L.f.	Lamiaceae / Verbenaceae	0,7	12,4	7	12	0,01	0,00	79,36	0,08	3,73	13,69
2320	47	1	Banjaranyar	<200	Kelapa	Cocos nucifera L.	Arecaceae	0,5	21,6	8	12	0,04	0,04	96,90	0,10	1,14	4,18
2321	47	1	Banjaranyar	<200	jati	Tectona grandis L.f.	Lamiaceae / Verbenaceae	0,7	11,5	6	10	0,04	0,01	57,99	0,06	0,68	2,50
2322	47	1	Banjaranyar	<200	jati	Tectona grandis L.f.	Lamiaceae / Verbenaceae	0,7	10,2	6	11	0,04	0,01	51,00	0,05	0,60	2,20
2323	47	1	Banjaranyar	<200	jati	Tectona grandis L.f.	Lamiaceae / Verbenaceae	0,7	10,2	6	10	0,04	0,01	46,74	0,05	0,55	2,02
2324	47	1	Banjaranyar	<200	jati	Tectona grandis L.f.	Lamiaceae / Verbenaceae	0,7	14,0	6	10	0,04	0,02	83,76	0,08	0,98	3,61
2325	47	1	Banjaranyar	<200	jati	Tectona grandis L.f.	Lamiaceae / Verbenaceae	0,7	14,3	6	11	0,04	0,02	95,24	0,10	1,12	4,11
2326	47	1	Banjaranyar	<200	jati	Tectona grandis L.f.	Lamiaceae / Verbenaceae	0,7	12,7	9	11	0,04	0,01	76,76	0,08	0,90	3,31
2327	47	1	Banjaranyar	<200	jati	Tectona grandis L.f.	Lamiaceae / Verbenaceae	0,7	10,2	8	11	0,04	0,01	51,00	0,05	0,60	2,20
2328	47	1	Banjaranyar	<200	jati	Tectona grandis L.f.	Lamiaceae / Verbenaceae	0,7	10,8	7	10	0,04	0,01	52,23	0,05	0,61	2,25
2329	47	1	Banjaranyar	<200	jati	Tectona grandis L.f.	Lamiaceae / Verbenaceae	0,7	10,2	6	10	0,04	0,01	46,74	0,05	0,55	2,02
2330	47	1	Banjaranyar	<200	jati	Tectona grandis L.f.	Lamiaceae / Verbenaceae	0,7	15,3	7	10	0,04	0,02	98,23	0,10	1,15	4,24
2331	47	1	Banjaranyar	<200	jati	Tectona grandis L.f.	Lamiaceae / Verbenaceae	0,7	10,2	5	9	0,04	0,01	42,44	0,04	0,50	1,83
2332	47	1	Banjaranyar	<200	jati	Tectona grandis L.f.	Lamiaceae / Verbenaceae	0,7	11,8	6	10	0,04	0,01	60,98	0,06	0,72	2,63
2333	47	1	Banjaranyar	<200	jati	Tectona grandis L.f.	Lamiaceae / Verbenaceae	0,7	11,5	6	10	0,04	0,01	57,99	0,06	0,68	2,50
2334	47	1	Banjaranyar	<200	jati	Tectona grandis L.f.	Lamiaceae / Verbenaceae	0,7	14,3	9	14	0,04	0,02	118,79	0,12	1,40	5,12
2335	47	1	Banjaranyar	<200	jati	Tectona grandis L.f.	Lamiaceae / Verbenaceae	0,7	10,5	6	11	0,04	0,01	53,96	0,05	0,63	2,33
2336	47	1	Banjaranyar	<200	jati	Tectona grandis L.f.	Lamiaceae / Verbenaceae	0,7	13,4	10	12	0,04	0,01	90,90	0,09	1,07	3,92
2337	47	1	Banjaranyar	<200	jati	Tectona grandis L.f.	Lamiaceae / Verbenaceae	0,7	11,5	9	12	0,04	0,01	68,53	0,07	0,81	2,96
2338	47	1	Banjaranyar	<200	jati	Tectona grandis L.f.	Lamiaceae / Verbenaceae	0,7	13,7	7	11	0,04	0,01	87,63	0,09	1,03	3,78
2339	47	1	Banjaranyar	<200	jati	Tectona grandis L.f.	Lamiaceae / Verbenaceae	0,7	10,8	5	10	0,04	0,01	52,23	0,05	0,61	2,25
2340	47	1	Banjaranyar	<200	jati	Tectona grandis L.f.	Lamiaceae / Verbenaceae	0,7	13,4	6	11	0,04	0,01	83,93	0,08	0,99	3,62
2341	47	1	Banjaranyar	<200	jati	Tectona grandis L.f.	Lamiaceae / Verbenaceae	0,7	13,0	6	11	0,04	0,01	80,31	0,08	0,94	3,46
2342	47	1	Banjaranyar	<200	jati	Tectona grandis L.f.	Lamiaceae / Verbenaceae	0,7	13,0	6	11	0,04	0,01	80,31	0,08	0,94	3,46
2343	47	1	Banjaranyar	<200	jati	Tectona grandis L.f.	Lamiaceae / Verbenaceae	0,7	12,1	7	12	0,04	0,01	75,67	0,08	0,89	3,26
2344	47	1	Banjaranyar	<200	jati	Tectona grandis L.f.	Lamiaceae / Verbenaceae	0,7	10,2	8	10	0,04	0,01	46,74	0,05	0,55	2,02
2345	47	1	Banjaranyar	<200	jati	Tectona grandis L.f.	Lamiaceae / Verbenaceae	0,7	11,5	6	11	0,04	0,01	63,28	0,06	0,74	2,73
2346	47	1	Banjaranyar	<200	jati	Tectona grandis L.f.	Lamiaceae / Verbenaceae	0,7	12,4	6	11	0,04	0,01	73,28	0,07	0,86	3,16
2347	47	1	Banjaranyar	<200	jati	Tectona grandis L.f.	Lamiaceae / Verbenaceae	0,7	11,8	7	11	0,04	0,01	66,54	0,07	0,78	2,87
2348	47	1	Banjaranyar	<200	jati	Tectona grandis L.f.	Lamiaceae / Verbenaceae	0,7	12,4	7	12	0,04	0,01	79,36	0,08	0,93	3,42
2349	47	1	Banjaranyar	<200	jati	Tectona grandis L.f.	Lamiaceae / Verbenaceae	0,7	10,2	7	11	0,04	0,01	51,00	0,05	0,60	2,20
2350	47	1	Banjaranyar	<200	jati	Tectona grandis L.f.	Lamiaceae / Verbenaceae	0,7	10,5	8	11	0,04	0,01	53,96	0,05	0,63	2,33

2351	47	1	Banjaranyar	<200	jati	Tectona grandis L.f.	Lamiaceae / Verbenaceae	0,7	14,3	8	12	0,04	0,02	103,14	0,10	1,21	4,45
2352	47	1	Banjaranyar	<200	jati	Tectona grandis L.f.	Lamiaceae / Verbenaceae	0,7	10,8	7	12	0,04	0,01	61,72	0,06	0,73	2,66
2353	47	1	Banjaranyar	<200	jati	Tectona grandis L.f.	Lamiaceae / Verbenaceae	0,7	13,7	7	11	0,04	0,01	87,63	0,09	1,03	3,78
2354	47	1	Banjaranyar	<200	Tisuk	Hibiscus macrophyllus Roxb. ex	Malvaceae	0,4	14,3	7	11	0,04	0,02	57,04	0,06	0,67	2,46
2355	47	1	Banjaranyar	<200	jati	Tectona grandis L.f.	Lamiaceae / Verbenaceae	0,7	10,2	7	10	0,04	0,01	46,74	0,05	0,55	2,02
2356	47	1	Banjaranyar	<200	jati	Tectona grandis L.f.	Lamiaceae / Verbenaceae	0,7	12,7	9	12	0,04	0,01	83,13	0,08	0,98	3,58
2357	47	1	Banjaranyar	<200	jati	Tectona grandis L.f.	Lamiaceae / Verbenaceae	0,7	16,2	6	11	0,04	0,02	119,79	0,12	1,41	5,17
2358	47	1	Banjaranyar	<200	jati	Tectona grandis L.f.	Lamiaceae / Verbenaceae	0,7	11,5	9	11	0,04	0,01	63,28	0,06	0,74	2,73
2359	48	1	Banjaranyar	<200	Mahoni	Swietenia macrophylla King	Meliaceae	0,61	19,1	8	12	0,04	0,03	154,02	0,15	1,81	6,64
2360	48	1	Banjaranyar	<200	Mahoni	Swietenia macrophylla King	Meliaceae	0,61	9,5	4	5	0,04	0,01	19,40	0,02	0,23	0,84
2361	48	1	Banjaranyar	<200	Kelapa	Cocos nucifera L.	Arecaceae	0,5	26,4	10	14	0,04	0,05	112,30	0,11	1,32	4,84
2362	48	1	Banjaranyar	<200	Mahoni	Swietenia macrophylla King	Meliaceae	0,61	16,9	6	10	0,04	0,02	103,84	0,10	1,22	4,48
2363	48	1	Banjaranyar	<200	Mahoni	Swietenia macrophylla King	Meliaceae	0,61	6,4	3	5	0,04	0,00	9,23	0,01	0,11	0,40
2364	48	1	Banjaranyar	<200	Mahoni	Swietenia macrophylla King	Meliaceae	0,61	34,4	9	13	0,04	0,09	486,50	0,49	5,72	20,98
2365	48	1	Banjaranyar	<200	Mahoni	Swietenia macrophylla King	Meliaceae	0,61	19,7	9	12	0,04	0,03	163,56	0,16	1,92	7,05
2366	48	1	Banjaranyar	<200	Mahoni	Swietenia macrophylla King	Meliaceae	0,61	23,9	7	12	0,04	0,04	231,81	0,23	2,72	10,00
2367	48	1	Banjaranyar	<200	Mahoni	Swietenia macrophylla King	Meliaceae	0,61	13,0	7	9	0,04	0,01	58,91	0,06	0,69	2,54
2368	48	1	Banjaranyar	<200	Kelapa	Cocos nucifera L.	Arecaceae	0,5	23,9	10	14	0,04	0,04	112,30	0,11	1,32	4,84
2369	48	1	Banjaranyar	<200	Kelapa	Cocos nucifera L.	Arecaceae	0,5	25,5	10	14	0,04	0,05	112,30	0,11	1,32	4,84
2370	48	1	Banjaranyar	<200	Mahoni	Swietenia macrophylla King	Meliaceae	0,61	20,7	9	12	0,04	0,03	178,35	0,18	2,10	7,69
2371	48	1	Banjaranyar	<200	Mahoni	Swietenia macrophylla King	Meliaceae	0,61	11,1	5	8	0,04	0,01	39,58	0,04	0,47	1,71
2372	48	1	Banjaranyar	<200	Mahoni	Swietenia macrophylla King	Meliaceae	0,61	27,7	8	13	0,04	0,06	327,38	0,33	3,85	14,12
2373	48	1	Banjaranyar	<200	Mahoni	Swietenia macrophylla King	Meliaceae	0,61	20,0	7	11	0,04	0,03	155,52	0,16	1,83	6,71
2374	48	1	Banjaranyar	<200	Rambutan	Nephelium lappaceum L.	Sapindaceae	0,71	7,3	6	8	0,04	0,00	21,08	0,02	0,25	0,91
2375	48	1	Banjaranyar	<200	Mahoni	Swietenia macrophylla King	Meliaceae	0,61	24,5	8	12	0,04	0,05	243,26	0,24	2,86	10,49
2376	48	1	Banjaranyar	<200	Jati	Tectona grandis L.f.	Lamiaceae / Verbenaceae	0,7	14,6	4	6	0,04	0,02	56,91	0,06	0,67	2,45
2377	48	1	Banjaranyar	<200	Jati	Tectona grandis L.f.	Lamiaceae / Verbenaceae	0,7	8,0	6	10	0,04	0,00	29,73	0,03	0,35	1,28
2378	48	1	Banjaranyar	<200	Mahoni	Swietenia macrophylla King	Meliaceae	0,61	25,5	6	12	0,04	0,05	260,90	0,26	3,07	11,25
2379	48	1	Banjaranyar	<200	Jati	Tectona grandis L.f.	Lamiaceae / Verbenaceae	0,7	29,0	9	16	0,04	0,07	487,72	0,49	5,73	21,03
2380	48	1	Banjaranyar	<200	Mahoni	Swietenia macrophylla King	Meliaceae	0,61	32,5	6	13	0,04	0,08	438,14	0,44	5,15	18,89
2381	48	1	Banjaranyar	<200	Jati	Tectona grandis L.f.	Lamiaceae / Verbenaceae	0,7	22,9	7	11	0,04	0,04	225,31	0,23	2,65	9,72
2382	48	1	Banjaranyar	<200	Mahoni	Swietenia macrophylla King	Meliaceae	0,61	26,4	10	14	0,04	0,05	321,43	0,32	3,78	13,86
2383	48	1	Banjaranyar	<200	Mahoni	Swietenia macrophylla King	Meliaceae	0,61	27,7	9	14	0,04	0,06	350,38	0,35	4,12	15,11
2384	48	1	Banjaranyar	<200	Jati	Tectona grandis L.f.	Lamiaceae / Verbenaceae	0,7	19,1	7	11	0,04	0,03	161,33	0,16	1,90	6,96
2385	48	1	Banjaranyar	<200	Mahoni	Swietenia macrophylla King	Meliaceae	0,61	36,0	9	14	0,04	0,10	565,68	0,57	6,65	24,39
2386	48	1	Banjaranyar	<200	Jati	Tectona grandis L.f.	Lamiaceae / Verbenaceae	0,7	34,7	6	15	0,04	0,09	639,87	0,64	7,52	27,59
2387	48	1	Banjaranyar	<200	Mahoni	Swietenia macrophylla King	Meliaceae	0,61	17,2	7	11	0,04	0,02	117,26	0,12	1,38	5,06
2388	48	1	Banjaranyar	<200	Jati	Tectona grandis L.f.	Lamiaceae / Verbenaceae	0,7	17,5	9	12	0,04	0,02	148,97	0,15	1,75	6,42
2389	48	1	Banjaranyar	<200	Mahoni	Swietenia macrophylla King	Meliaceae	0,61	12,7	5	8	0,04	0,01	50,55	0,05	0,59	2,18
2390	48	1	Banjaranyar	<200	Jati	Tectona grandis L.f.	Lamiaceae / Verbenaceae	0,7	22,6	7	12	0,04	0,04	237,83	0,24	2,79	10,26
2391	48	1	Banjaranyar	<200	Jati	Tectona grandis L.f.	Lamiaceae / Verbenaceae	0,7	9,2	6	9	0,04	0,01	35,43	0,04	0,42	1,53
2392	48	1	Banjaranyar	<200	Jati	Tectona grandis L.f.	Lamiaceae / Verbenaceae	0,7	30,9	12	17	0,04	0,07	579,54	0,58	6,81	24,99
2393	48	1	Banjaranyar	<200	Jati	Tectona grandis L.f.	Lamiaceae / Verbenaceae	0,7	13,7	7	10	0,04	0,01	80,31	0,08	0,94	3,46
2394	48	1	Banjaranyar	<200	Jati	Tectona grandis L.f.	Lamiaceae / Verbenaceae	0,7	19,4	10	15	0,04	0,03	220,93	0,22	2,60	9,53
2395	48	1	Banjaranyar	<200	Jati	Tectona grandis L.f.	Lamiaceae / Verbenaceae	0,7	7,3	5	6	0,04	0,00	15,98	0,02	0,19	0,69
2396	48	1	Banjaranyar	<200	Jati	Tectona grandis L.f.	Lamiaceae / Verbenaceae	0,7	12,1	9	13	0,04	0,01	81,43	0,08	0,96	3,51
2397	48	1	Banjaranyar	<200	Jati	Tectona grandis L.f.	Lamiaceae / Verbenaceae	0,7	10,5	10	11	0,04	0,01	53,96	0,05	0,63	2,33
2398	48	1	Banjaranyar	<200	Jati	Tectona grandis L.f.	Lamiaceae / Verbenaceae	0,7	9,9	7	9	0,04	0,01	40,04	0,04	0,47	1,73
2399	48	1	Banjaranyar	<200	Jati	Tectona grandis L.f.	Lamiaceae / Verbenaceae	0,7	23,2	11	15	0,04	0,04	307,00	0,31	3,61	13,24
2400	48	1	Banjaranyar	<200	Jati	Tectona grandis L.f.	Lamiaceae / Verbenaceae	0,7	20,0	11	14	0,04	0,03	220,03	0,22	2,59	9,49
2401	48	1	Banjaranyar	<200	Jati	Tectona grandis L.f.	Lamiaceae / Verbenaceae	0,7	18,1	8	15	0,04	0,03	195,12	0,20	2,29	8,41
2402	48	1	Banjaranyar	<200	Jati	Tectona grandis L.f.	Lamiaceae / Verbenaceae	0,7	14,0	10	14	0,04	0,02	114,00	0,11	1,34	4,92
2403	48	1	Banjaranyar	<200	Jati	Tectona grandis L.f.	Lamiaceae / Verbenaceae	0,7	12,4	8	12	0,04	0,01	79,36	0,08	0,93	3,42
2404	48	1	Banjaranyar	<200	Jati	Tectona grandis L.f.	Lamiaceae / Verbenaceae	0,7	10,8	6	8	0,04	0,01	42,57	0,04	0,50	1,84
2405	48	1	Banjaranyar	<200	Jati	Tectona grandis L.f.	Lamiaceae / Verbenaceae	0,7	23,9	11	14	0,04	0,04	302,83	0,30	3,56	13,06
2406	48	1	Banjaranyar	<200	Mahoni	Swietenia macrophylla King	Meliaceae	0,61	17,5	7	9	0,04	0,02	100,90	0,10	1,19	4,35
2407	48	1	Banjaranyar	<200	Jati	Tectona grandis L.f.	Lamiaceae / Verbenaceae	0,7	31,2	8	15	0,04	0,08	526,57	0,53	6,19	22,71
2408	48	1	Banjaranyar	<200	Mahoni	Swietenia macrophylla King	Meliaceae	0,61	55,4	10	16	0,04	0,24	1.409,75	1,41	16,56	60,79
2409	48	1	Banjaranyar	<200	Mahoni	Swietenia macrophylla King	Meliaceae	0,61	16,9	6	9	0,04	0,02	94,28	0,09	1,11	4,07
2410	48	1	Banjaranyar	<200	Mahoni	Swietenia macrophylla King	Meliaceae	0,61	29,0	7	12	0,04	0,07	330,35	0,33	3,88	14,25
2411	48	1	Banjaranyar	<200	Mahoni	Swietenia macrophylla King	Meliaceae	0,61	0,5	8	12	0,04	0,00	0,23	0,00	0,00	0,01
2412	48	1	Banjaranyar	<200	Mahoni	Swietenia macrophylla King	Meliaceae	0,61	21,3	8	11	0,04	0,04	174,09	0,17	2,05	7,51
2413	48	1	Banjaranyar	<200	Jati	Tectona grandis L.f.	Lamiaceae / Verbenaceae	0,7	33,7	10	14	0,04	0,09	570,75	0,57	6,71	24,61
2414	48	1	Banjaranyar	<200	Mahoni	Swietenia macrophylla King	Meliaceae	0,61	20,7	7	9	0,04	0,03	137,03	0,14	1,61	5,91
2415	48	1	Banjaranyar	<200	Jati	Tectona grandis L.f.	Lamiaceae / Verbenaceae	0,7	31,2	7	15	0,04	0,08	526,57	0,53	6,19	22,71
2416	48	1	Banjaranyar	<200	Mahoni	Swietenia macrophylla King	Meliaceae	0,61	17,2	6	9	0,04	0,02	97,57	0,10	1,15	4,21
2417	49	2	Banjaranyar	<200	Sengon	Paraserianthes falcataria (L.) I.C	Leguminosae / Fabaceae	0,33	9,9	9	13	0,04	0,01	28,16	0,03	0,33	1,21
2418	49	2	Banjaranyar	<200	Angsana/Sonokembang	Pterocarpus indicus Willd.	Leguminosae / Fabaceae	0,65	12,4	9	10	0,04	0,01	62,74	0,06	0,74	2,71
2419	49	2	Banjaranyar	<200	Mahoni	Swietenia macrophylla King	Meliaceae	0,61	4,5	3	4,5	0,04	0,00	4,36	0,00	0,05	0,19
2420	49	2	Banjaranyar	<200	Mahoni	Swietenia macrophylla King	Meliaceae	0,61	3,8	3	4	0,04	0,00	2,95	0,00	0,03	0,13
2421	49	2	Banjaranyar	<200	Salam	Syzygium polyanthum (Wight) W	Myrtaceae	0,66	19,1	11	14	0,04	0,03	190,65	0,19	2,24	8,22
2422	49	2	Banjaranyar	<200	Sengon	Paraserianthes falcataria (L.) I.C	Leguminosae / Fabaceae	0,33	15,9	10	13	0,04	0,02	67,60	0,07	0,79	2,92
2423	49	2	Banjaranyar	<200	Mahoni	Swietenia macrophylla King	Meliaceae	0,61	7,0	5	8	0,04	0,00	16,91	0,02	0,20	0,73
2424	49	2	Banjaranyar	<200	Mahoni	Swietenia macrophylla King	Meliaceae	0,61	3,8	3	4	0,04	0,00	2,95	0,00	0,03	0,13
2425	49	2	Banjaranyar	<200	Salam	Syzygium polyanthum (Wight) W	Myrtaceae	0,66	4,1	5	6,5	0,04	0,00	5,73	0,01	0,07	0,25
2426	49	2	Banjaranyar	<200	Mahoni	Swietenia macrophylla King	Meliaceae	0,61	9,9	5	8	0,04	0,01	31,69	0,03	0,37	1,37
2427	49	2	Banjaranyar	<200	Mahoni	Swietenia macrophylla King	Meliaceae	0,61	9,2	4	8	0,04	0,01	28,04	0,03	0,33	1,21
2428	49	2	Banjaranyar	<200	Mahoni	Swietenia macrophylla King	Meliaceae	0,61	5,7	4	7	0,04	0,00	10,36	0,01	0,12	0,45
2429	49	2	Banjaranyar	<200	Jati	Tectona grandis L.f.	Lamiaceae / Verbenaceae	0,7	6,7	5	7	0,04	0,00	15,58	0,02	0,18	0,67
2430	49	2	Banjaranyar	<200	Sengon	Paraserianthes falcataria (L.) I.C	Leguminosae / Fabaceae	0,33	13,7	8	14	0,04	0,01	54,88	0,05	0,64	2,37
2431	49	2	Banjaranyar	<200	Jati	Tectona grandis L.f.	Lamiaceae / Verbenaceae	0,7	14,3	6	15	0,04	0,02	126,54	0,13	1,49	5,46
2432	49	2	Banjaranyar	<200	Sengon	Paraserianthes falcataria (L.) I.C	Leguminosae / Fabaceae	0,33	10,5	9	12	0,04	0,01	29,34	0,03	0,34	1,27
2433	49	2	Banjaranyar	<200	Jati	Tectona grandis L.f.	Lamiaceae / Verbenaceae	0,7	7,3	5	10	0,04	0,00	25,52	0,03	0,30	1,10
2434	49	2	Banjaranyar	<200	Jati	Tectona grandis L.f.	Lamiaceae / Verbenaceae	0,7	11,5	6	9	0,04	0,01	52,66	0,05	0,62	2,27
2435	49	2	Banjaranyar	<200	Mahoni	Swietenia macrophylla King	Meliaceae	0,61	3,8	3	3,5	0,04	0,00	2,61	0,00	0,03	0,11
2436	49	2	Banjaranyar	<200	Mahoni	Swietenia macrophylla King	Meliaceae	0,61	12,1	7	11	0,04	0,01	61,60	0,06	0,72	2,66
2437	49	2	Banjaranyar	<200	Jati	Tectona grandis L.f.	Lamiaceae / Verbenaceae	0,7	12,1	6	10	0,04	0,01	64,03	0,06	0,75	2,76
2438	49	2	Banjaranyar	<200	Heuras	Vitex pubescens Vahl	Lamiaceae / Verbenaceae	0,88	10,2	7	10	0,04	0,01	57,64	0,06	0,68	2,49
2439	49	2	Banjaranyar	<200	Mahoni	Swietenia macrophylla King	Meliaceae	0,61	3,5	3	4	0,04	0,00	2,52	0,00	0,03	0,11
2440	49	2	Banjaranyar	<200	Mahoni	Swietenia macrophylla King	Meliaceae	0,61	11,5	5	9	0,04	0,01	46,42	0,05	0,55	2,00
2441	49	2	Banjaranyar	<200	Sengon	Paraserianthes falcataria (L.) I.C	Leguminosae / Fabaceae	0,33	16,2	9	12	0,04	0,02	65,14	0,07	0,77	2,81
2442	49	2	Banjaranyar	<200	Mahoni	Swietenia macrophylla King	Meliaceae	0,61	8,6	4	8	0,04	0,01	24,60	0,02	0,29	1,06
2443	49	2	Banjaranyar	<200	Jati	Tectona grandis L.f.	Lamiaceae / Verbenaceae	0,7	6,4	5	8	0,04	0,00	16,10	0,02	0,19	0,69
2444	49	2	Banjaranyar	<200	Mahoni	Swietenia macrophylla King	Meliaceae	0,61	4,1	3	4	0,04	0,00	3,42	0,00	0,04	0,15
2445	49	2	Banjaranyar	<200	Sengon	Paraserianthes falcataria (L.) I.C	Leguminosae / Fabaceae	0,33	14,3	11	16	0,04	0,02	67,41	0,07	0,79	2,91
2446	49	2	Banjaranyar	<200	Mahoni	Swietenia macrophylla King	Meliaceae	0,61	4,1	4	5	0,04	0,00	4,19	0,00	0,05	0,18
2447	49	2	Banjaranyar	<200	Jati	Tectona grandis L.f.	Lamiaceae / Verbenaceae	0,7	27,0	7	14	0,04	0,06	380,87	0,38	4,48	16,42
2448	49	2	Banjaranyar	<200	Jati	Tectona grandis L.f.	Lamiaceae / Verbenaceae	0,7	15,0	3	9	0,04	0,02	85,82	0,09	1,01	3,70
2449	49	2	Banjaranyar	<200	Jati	Tectona grandis L.f.	Lamiaceae / Verbenaceae	0,7	12,1	5	9	0,04	0,01	58,14	0,06	0,68	2,51
2450	49	2	Banjaranyar	<200	Jati	Tectona grandis L.f.	Lamiaceae / Verbenaceae	0,7	13,4	5	9	0,04	0,01	69,84	0,07	0,82	3,01
2451	49	2	Banjaranyar	<200	Jati	Tectona grandis L.f.	Lamiaceae / Verbenaceae	0,7	3,8	3	4	0,04	0,00	3,35	0,00	0,04	0,14
2452	49	2	Banjaranyar	<200	Angsana/Sonokembang	Pterocarpus indicus Willd.	Leguminosae / Fabaceae	0,65	7,3	6	7	0,04	0,00	17,20	0,02	0,20	0,74
2453	49	2	Banjaranyar	<200	Jati	Tectona grandis L.f.	Lamiaceae / Verbenaceae	0,7	4,1	2	3	0,04	0,00	2,98	0,00	0,03	0,13
2454	49	2	Banjaranyar	<200	Jati	Tectona grandis L.f.	Lamiaceae / Verbenaceae	0,7	32,1	7	12	0,04	0,08	453,60	0,45	5,33	19,56
2455	49	2	Banjaranyar	<200	Kelapa	Cocos nucifera L.	Arecaceae	0,5	20,0	3	7	0,04	0,03	58,40	0,06	0,69	2,52
2456	49	2	Banjaranyar	<200	Jati	Tectona grandis L.f.	Lamiaceae / Verbenaceae	0,7	17,2	7	14	0,04	0,02	165,89	0,17	1,95	7,15
2457	49	2	Banjaranyar	<200	Mahoni	Swietenia macrophylla King	Meliaceae	0,61	12,7	6	10	0,04	0,01	62,01	0,06	0,73	2,67
2458	49	2	Banjaranyar	<200	Mahoni	Swietenia macrophylla King	Meliaceae	0,61	8,6	5	8	0,04	0,01	24,60	0,02	0,29	1,06
2459	49	2	Banjaranyar	<200	Jati	Tectona grandis L.f.	Lamiaceae / Verbenaceae	0,7	20,7	5	11	0,04	0,03	186,81	0,19	2,20	8,06
2460	49	2	Banjaranyar	<200	Tisuk	Hibiscus macrophyllus Roxb. ex	Malvaceae	0,4	5,4	6	8	0,04	0,00	7,16	0,01	0,08	0,31
2461	49	2	Banjaranyar	<200	Mahoni	Swietenia macrophylla King	Meliaceae	0,61	9,5	5	8	0,04	0,01	29,84	0,03	0,35	1,29
2462	49	2	Banjaranyar	<200	Mahoni	Swietenia macrophylla King	Meliaceae	0,61	3,2	2	2,5	0,04	0,00	1,37	0,00	0,02	0,06
2463	49	2	Banjaranyar	<200	Mahoni	Swietenia macrophylla King	Meliaceae	0,61	3,2	2	2,5	0,04	0,00	1,37	0,00	0,02	0,06
2464	49	2	Banjaranyar	<200	Mahoni	Swietenia macrophylla King	Meliaceae	0,61	15,0	6	9	0,04	0,02	75,66	0,08	0,89	3,26
2465	49	2	Banjaranyar	<200	Mahoni	Swietenia macrophylla King	Meliaceae	0,61	7,3	4	7	0,04	0,00	16,23	0,02	0,19	0,70
2466	49	2	Banjaranyar	<200	Jati	Tectona grandis L.f.	Lamiaceae / Verbenaceae	0,7	8,0	3	6	0,04	0,00	18,62	0,02	0,22	0,80
2467	49	2	Banjaranyar	<200	Jati	Tectona grandis L.f.	Lamiaceae / Verbenaceae	0,7	5,4	2	3	0,04	0,00	4,87	0,00	0,06	0,21
2468	49	2	Banjaranyar	<200	Sawo / sawo manila	Manilkara zapota (L.) P.Royen	Sapotaceae	0,81	3,2	2	3	0,04	0,00	2,11	0,00	0,02	0,09
2469	49	2	Banjaranyar	<200	Sengon	Paraserianthes falcataria (L.) I.C	Leguminosae / Fabaceae	0,33	23,2	10	16	0,04	0,04	163,55	0,16	1,92	7,05
2470	49	2	Banjaranyar	<200	Jati	Tectona grandis L.f.	Lamiaceae / Verbenaceae	0,7	6,4	4	6	0,04	0,00	12,37	0,01	0,15	0,53
2471	49	2	Banjaranyar	<200	Sengon	Paraserianthes falcataria (L.) I.C	Leguminosae / Fabaceae	0,33	15,6	10	15	0,04	0,02	74,27	0,07	0,87	3,20
2472	49	2	Banjaranyar	<200	Mahoni	Swietenia macrophylla King	Meliaceae	0,61	19,7	10	14	0,04	0,03	188,36	0,19	2,21	8,12
2473	49	2	Banjaranyar	<200	Jati	Tectona grandis L.f.	Lamiaceae / Verbenaceae	0,7	14,0	8	11	0,04	0,02	91,40	0,09	1,07	3,94
2474	49	2	Banjaranyar	<200	Jati	Tectona grandis L.f.	Lamiaceae / Verbenaceae	0,7	9,5	7	10	0,04	0,01	41,53	0,04	0,49	1,79

2475	49	2	Banjaranyar	<200	Sawo / sawo manila	Manilkara zapota (L.) P.Royen	Sapotaceae	0,81	2,9	1	3	0,04	0,00	1,74	0,00	0,02	0,07
2476	49	2	Banjaranyar	<200	Jati	Tectona grandis L.f.	Lamiaceae / Verbenaceae	0,7	6,0	3	5	0,04	0,00	9,53	0,01	0,11	0,41
2477	49	2	Banjaranyar	<200	Sengon	Paraserianthes falcataria (L.) I.C.	Leguminosae / Fabaceae	0,33	23,5	10	15	0,04	0,04	158,06	0,16	1,86	6,82
2478	49	2	Banjaranyar	<200	Jati	Tectona grandis L.f.	Lamiaceae / Verbenaceae	0,7	12,1	6	9	0,04	0,01	58,14	0,06	0,68	2,51
2479	49	2	Banjaranyar	<200	Mahoni	Swietenia macrophylla King	Meliaceae	0,61	2,9	2	3	0,04	0,00	1,34	0,00	0,02	0,06
2480	49	2	Banjaranyar	<200	Sengon	Paraserianthes falcataria (L.) I.C.	Leguminosae / Fabaceae	0,33	18,1	10	15	0,04	0,03	97,98	0,10	1,15	4,23
2481	49	2	Banjaranyar	<200	Heuras	Vitex pubescens Vahl	Lamiaceae / Verbenaceae	0,88	2,5	2	3	0,04	0,00	1,51	0,00	0,02	0,07
2482	49	2	Banjaranyar	<200	Angsana/Sonokembang	Pterocarpus indicus Willd.	Leguminosae / Fabaceae	0,65	12,1	8	9	0,04	0,01	54,32	0,05	0,64	2,34
2483	49	2	Banjaranyar	<200	Mahoni	Swietenia macrophylla King	Meliaceae	0,61	14,3	4	10	0,04	0,02	76,94	0,08	0,90	3,32
2484	49	2	Banjaranyar	<200	Mahoni	Swietenia macrophylla King	Meliaceae	0,61	13,4	7	11	0,04	0,01	73,99	0,07	0,87	3,19
2485	49	2	Banjaranyar	<200	Mahoni	Swietenia macrophylla King	Meliaceae	0,61	7,6	4	6	0,04	0,00	15,23	0,02	0,18	0,66
2486	49	2	Banjaranyar	<200	Mahoni	Swietenia macrophylla King	Meliaceae	0,61	3,8	3	4	0,04	0,00	2,95	0,00	0,03	0,13
2487	49	2	Banjaranyar	<200	Mahoni	Swietenia macrophylla King	Meliaceae	0,61	12,1	6	9	0,04	0,01	51,25	0,05	0,60	2,21
2488	49	2	Banjaranyar	<200	Mahoni	Swietenia macrophylla King	Meliaceae	0,61	3,5	2	2,5	0,04	0,00	1,64	0,00	0,02	0,07
2489	49	2	Banjaranyar	<200	Tisuk	Hibiscus macrophyllus Roxb. ex I	Malvaceae	0,4	3,5	3	5	0,04	0,00	2,10	0,00	0,02	0,09
2490	49	2	Banjaranyar	<200	Tisuk	Hibiscus macrophyllus Roxb. ex I	Malvaceae	0,4	13,0	8	11	0,04	0,01	48,10	0,05	0,57	2,07
2491	49	2	Banjaranyar	<200	Jati	Tectona grandis L.f.	Lamiaceae / Verbenaceae	0,7	10,8	4	5	0,04	0,01	27,68	0,03	0,33	1,19
2492	49	2	Banjaranyar	<200	Heuras	Vitex pubescens Vahl	Lamiaceae / Verbenaceae	0,88	2,9	3	5	0,04	0,00	2,99	0,00	0,04	0,13
2493	49	2	Banjaranyar	<200	Mahoni	Swietenia macrophylla King	Meliaceae	0,61	4,5	3	4	0,04	0,00	3,91	0,00	0,05	0,17
2494	49	2	Banjaranyar	<200	Mahoni	Swietenia macrophylla King	Meliaceae	0,61	6,7	3	5	0,04	0,00	10,09	0,01	0,12	0,44
2495	49	2	Banjaranyar	<200	Mahoni	Swietenia macrophylla King	Meliaceae	0,61	7,3	4	6	0,04	0,00	14,09	0,01	0,17	0,61
2496	49	2	Banjaranyar	<200	Mahoni	Swietenia macrophylla King	Meliaceae	0,61	3,5	3	4	0,04	0,00	2,52	0,00	0,03	0,11
2497	49	2	Banjaranyar	<200	Jati	Tectona grandis L.f.	Lamiaceae / Verbenaceae	0,7	23,5	7	10	0,04	0,04	217,10	0,22	2,55	9,36
2498	49	2	Banjaranyar	<200	salam	Syzygium polyanthum (Wight) W.	Myrtaceae	0,66	4,5	4	5,5	0,04	0,00	5,63	0,01	0,07	0,24
2499	49	2	Banjaranyar	<200	Jati	Tectona grandis L.f.	Lamiaceae / Verbenaceae	0,7	14,0	8	14	0,04	0,02	114,00	0,11	1,34	4,92
2500	49	2	Banjaranyar	<200	Jati	Tectona grandis L.f.	Lamiaceae / Verbenaceae	0,7	12,4	4	10	0,04	0,01	67,15	0,07	0,79	2,90
2501	49	2	Banjaranyar	<200	Sengon	Paraserianthes falcataria (L.) I.C.	Leguminosae / Fabaceae	0,33	5,7	6	9	0,04	0,00	7,43	0,01	0,09	0,32
2502	49	2	Banjaranyar	<200	Sengon	Paraserianthes falcataria (L.) I.C.	Leguminosae / Fabaceae	0,33	39,8	11	18	0,04	0,12	488,03	0,49	5,73	21,05
2503	49	2	Banjaranyar	<200	Mahoni	Swietenia macrophylla King	Meliaceae	0,61	7,3	3	6	0,04	0,00	14,09	0,01	0,17	0,61
2504	49	2	Banjaranyar	<200	Jati	Tectona grandis L.f.	Lamiaceae / Verbenaceae	0,7	11,1	4	8	0,04	0,01	44,89	0,04	0,53	1,94
2505	49	2	Banjaranyar	<200	Jati	Tectona grandis L.f.	Lamiaceae / Verbenaceae	0,7	11,1	6	8	0,04	0,01	44,89	0,04	0,53	1,94
2506	49	2	Banjaranyar	<200	Jati	Tectona grandis L.f.	Lamiaceae / Verbenaceae	0,7	12,7	5	9	0,04	0,01	63,87	0,06	0,75	2,75
2507	49	2	Banjaranyar	<200	Jati	Tectona grandis L.f.	Lamiaceae / Verbenaceae	0,7	8,3	4	6	0,04	0,01	20,01	0,02	0,24	0,86
2508	49	2	Banjaranyar	<200	Jati	Tectona grandis L.f.	Lamiaceae / Verbenaceae	0,7	3,5	3	3,5	0,04	0,00	2,53	0,00	0,03	0,11
2509	49	2	Banjaranyar	<200	Jambu bol	Syzygium malaccense (L.) Merr. &	Myrtaceae	0,56	14,3	4	9	0,04	0,02	64,60	0,06	0,76	2,79
2510	49	2	Banjaranyar	<200	Sawo / sawo manila	Manilkara zapota (L.) P.Royen	Sapotaceae	0,81	2,5	2	3	0,04	0,00	1,40	0,00	0,02	0,06
2511	49	2	Banjaranyar	<200	Karet	Hevea brasiliensis (Willd. ex A.Ju	Euphorbiaceae	0,53	7,0	5	6	0,04	0,00	11,42	0,01	0,13	0,49
2512	49	2	Banjaranyar	<200	karet	Hevea brasiliensis (Willd. ex A.Ju	Euphorbiaceae	0,53	4,5	4	5	0,04	0,00	4,22	0,00	0,05	0,18
2513	50	2	Banjaranyar	<200	Jati	Tectona grandis L.f.	Lamiaceae / Verbenaceae	0,7	23,9	8	12	0,04	0,04	262,95	0,26	3,09	11,34
2514	50	2	Banjaranyar	<200	Tisuk	Hibiscus macrophyllus Roxb. ex I	Malvaceae	0,4	8,0	6	9	0,04	0,00	16,17	0,02	0,19	0,70
2515	50	2	Banjaranyar	<200	Tisuk	Hibiscus macrophyllus Roxb. ex I	Malvaceae	0,4	6,0	3	6	0,04	0,00	6,75	0,01	0,08	0,29
2516	50	2	Banjaranyar	<200	Jati	Tectona grandis L.f.	Lamiaceae / Verbenaceae	0,7	22,6	8	14	0,04	0,04	273,90	0,27	3,22	11,81
2517	50	2	Banjaranyar	<200	Tisuk	Hibiscus macrophyllus Roxb. ex I	Malvaceae	0,4	3,2	2	3	0,04	0,00	1,10	0,00	0,01	0,05
2518	50	2	Banjaranyar	<200	Mahoni	Swietenia macrophylla King	Meliaceae	0,61	4,5	3	4	0,04	0,00	3,91	0,00	0,05	0,17
2519	50	2	Banjaranyar	<200	Jati	Tectona grandis L.f.	Lamiaceae / Verbenaceae	0,7	15,3	7	10	0,04	0,02	98,23	0,10	1,15	4,24
2520	50	2	Banjaranyar	<200	Jati	Tectona grandis L.f.	Lamiaceae / Verbenaceae	0,7	12,1	6	9	0,04	0,01	58,14	0,06	0,68	2,51
2521	50	2	Banjaranyar	<200	Jati	Tectona grandis L.f.	Lamiaceae / Verbenaceae	0,7	9,5	5	7	0,04	0,01	29,95	0,03	0,35	1,29
2522	50	2	Banjaranyar	<200	Mahoni	Swietenia macrophylla King	Meliaceae	0,61	4,5	2,5	3,5	0,04	0,00	3,46	0,00	0,04	0,15
2523	50	2	Banjaranyar	<200	tisuk	Hibiscus macrophyllus Roxb. ex I	Malvaceae	0,4	5,7	4	5	0,04	0,00	5,17	0,01	0,06	0,22
2524	50	2	Banjaranyar	<200	Tisuk	Hibiscus macrophyllus Roxb. ex I	Malvaceae	0,4	6,0	5	6,5	0,04	0,00	7,26	0,01	0,09	0,31
2525	50	2	Banjaranyar	<200	Jati	Tectona grandis L.f.	Lamiaceae / Verbenaceae	0,7	23,2	5	9	0,04	0,04	192,27	0,19	2,26	8,29
2526	50	2	Banjaranyar	<200	Jati	Tectona grandis L.f.	Lamiaceae / Verbenaceae	0,7	24,5	7	11	0,04	0,05	254,80	0,25	2,99	10,99
2527	50	2	Banjaranyar	<200	Jati	Tectona grandis L.f.	Lamiaceae / Verbenaceae	0,7	10,8	4	5	0,04	0,01	27,68	0,03	0,33	1,19
2528	50	2	Banjaranyar	<200	Jati	Tectona grandis L.f.	Lamiaceae / Verbenaceae	0,7	9,2	3,5	6,5	0,04	0,01	26,30	0,03	0,31	1,13
2529	50	2	Banjaranyar	<200	Mangga	Mangifera indica L.	Anacardiaceae	0,6	2,9	2	3	0,04	0,00	1,32	0,00	0,02	0,06
2530	50	2	Banjaranyar	<200	Jati	Tectona grandis L.f.	Lamiaceae / Verbenaceae	0,7	12,1	4	8	0,04	0,01	52,19	0,05	0,61	2,25
2531	50	2	Banjaranyar	<200	Jati	Tectona grandis L.f.	Lamiaceae / Verbenaceae	0,7	21,3	9	12	0,04	0,04	213,86	0,21	2,51	9,22
2532	50	2	Banjaranyar	<200	Tisuk	Hibiscus macrophyllus Roxb. ex I	Malvaceae	0,4	14,3	9	13	0,04	0,02	66,48	0,07	0,78	2,87
2533	50	2	Banjaranyar	<200	Tisuk	Hibiscus macrophyllus Roxb. ex I	Malvaceae	0,4	9,5	3	5	0,04	0,01	13,18	0,01	0,15	0,57
2534	50	2	Banjaranyar	<200	Jati	Tectona grandis L.f.	Lamiaceae / Verbenaceae	0,7	18,1	6	10	0,04	0,03	134,58	0,13	1,58	5,80
2535	50	2	Banjaranyar	<200	Tisuk	Hibiscus macrophyllus Roxb. ex I	Malvaceae	0,4	8,9	6	8	0,04	0,01	17,87	0,02	0,21	0,77
2536	50	2	Banjaranyar	<200	Jati	Tectona grandis L.f.	Lamiaceae / Verbenaceae	0,7	19,4	9	3	0,04	0,03	50,58	0,05	0,59	2,18
2537	50	2	Banjaranyar	<200	Jati	Tectona grandis L.f.	Lamiaceae / Verbenaceae	0,7	15,9	8	11	0,04	0,02	115,52	0,12	1,36	4,98
2538	50	2	Banjaranyar	<200	Jati	Tectona grandis L.f.	Lamiaceae / Verbenaceae	0,7	18,1	9	12	0,04	0,03	159,05	0,16	1,87	6,86
2539	50	2	Banjaranyar	<200	tisuk	Hibiscus macrophyllus Roxb. ex I	Malvaceae	0,4	10,8	7	9	0,04	0,01	28,40	0,03	0,33	1,22
2540	50	2	Banjaranyar	<200	Jati	Tectona grandis L.f.	Lamiaceae / Verbenaceae	0,7	9,9	4	6	0,04	0,01	27,62	0,03	0,32	1,19
2541	50	2	Banjaranyar	<200	Tisuk	Hibiscus macrophyllus Roxb. ex I	Malvaceae	0,4	15,3	8	10	0,04	0,02	58,84	0,06	0,69	2,54
2542	50	2	Banjaranyar	<200	Tisuk	Hibiscus macrophyllus Roxb. ex I	Malvaceae	0,4	6,0	4	6	0,04	0,00	6,75	0,01	0,08	0,29
2543	50	2	Banjaranyar	<200	Tisuk	Hibiscus macrophyllus Roxb. ex I	Malvaceae	0,4	7,3	5	8	0,04	0,00	12,46	0,01	0,15	0,54
2544	50	2	Banjaranyar	<200	Tisuk	Hibiscus macrophyllus Roxb. ex I	Malvaceae	0,4	7,0	4	6	0,04	0,00	8,82	0,01	0,10	0,38
2545	50	2	Banjaranyar	<200	Jati	Tectona grandis L.f.	Lamiaceae / Verbenaceae	0,7	24,5	7	12	0,04	0,05	275,94	0,28	3,24	11,90
2546	50	2	Banjaranyar	<200	Jati	Tectona grandis L.f.	Lamiaceae / Verbenaceae	0,7	8,9	5	7	0,04	0,01	26,40	0,03	0,31	1,14
2547	50	2	Banjaranyar	<200	Jati	Tectona grandis L.f.	Lamiaceae / Verbenaceae	0,7	12,7	6	10	0,04	0,01	70,34	0,07	0,83	3,03
2548	50	2	Banjaranyar	<200	Jati	Tectona grandis L.f.	Lamiaceae / Verbenaceae	0,7	17,2	7	11	0,04	0,02	133,01	0,13	1,56	5,74
2549	50	2	Banjaranyar	<200	Tisuk	Hibiscus macrophyllus Roxb. ex I	Malvaceae	0,4	7,3	5	7	0,04	0,00	11,03	0,01	0,13	0,48
2550	50	2	Banjaranyar	<200	Jati	Tectona grandis L.f.	Lamiaceae / Verbenaceae	0,7	17,5	7	11	0,04	0,02	137,56	0,14	1,62	5,93
2551	50	2	Banjaranyar	<200	Tisuk	Hibiscus macrophyllus Roxb. ex I	Malvaceae	0,4	14,3	10	12	0,04	0,02	61,78	0,06	0,73	2,66
2552	50	2	Banjaranyar	<200	Jati	Tectona grandis L.f.	Lamiaceae / Verbenaceae	0,7	21,3	8	10	0,04	0,04	180,97	0,18	2,13	7,80
2553	50	2	Banjaranyar	<200	Jati	Tectona grandis L.f.	Lamiaceae / Verbenaceae	0,7	11,5	4	7	0,04	0,01	41,83	0,04	0,49	1,80
2554	50	2	Banjaranyar	<200	Mahoni	Swietenia macrophylla King	Meliaceae	0,61	3,2	3	4	0,04	0,00	2,11	0,00	0,02	0,09
2555	50	2	Banjaranyar	<200	Jati	Tectona grandis L.f.	Lamiaceae / Verbenaceae	0,7	9,5	4	6	0,04	0,01	26,01	0,03	0,31	1,12
2556	50	2	Banjaranyar	<200	Mahoni	Swietenia macrophylla King	Meliaceae	0,61	4,8	3	5	0,04	0,00	5,45	0,01	0,06	0,23
2557	50	2	Banjaranyar	<200	Caruy	Pterospermum javanicum Jungh.	Malvaceae	0,53	5,4	4	5	0,04	0,00	6,03	0,01	0,07	0,26
2558	50	2	Banjaranyar	<200	Sengon	Paraserianthes falcataria (L.) I.C.	Leguminosae / Fabaceae	0,33	8,6	7	10	0,04	0,01	17,19	0,02	0,20	0,74
2559	50	2	Banjaranyar	<200	Rambutan	Nephelium lappaceum L.	Sapindaceae	0,71	13,0	2	5	0,04	0,01	39,51	0,04	0,46	1,70
2560	50	2	Banjaranyar	<200	Jati	Tectona grandis L.f.	Lamiaceae / Verbenaceae	0,7	17,5	8	10	0,04	0,02	126,06	0,13	1,48	5,44
2561	50	2	Banjaranyar	<200	Mahoni	Swietenia macrophylla King	Meliaceae	0,61	6,4	3	5	0,04	0,00	9,23	0,01	0,11	0,40
2562	50	2	Banjaranyar	<200	Sengon	Paraserianthes falcataria (L.) I.C.	Leguminosae / Fabaceae	0,33	10,2	10	12	0,04	0,01	27,74	0,03	0,33	1,20
2563	50	2	Banjaranyar	<200	Jati	Tectona grandis L.f.	Lamiaceae / Verbenaceae	0,7	14,0	5	9	0,04	0,02	76,05	0,08	0,89	3,28
2564	50	2	Banjaranyar	<200	Alpukat	Persea americana Mill.	Lauraceae	0,54	7,6	4	7	0,04	0,00	15,69	0,02	0,18	0,68
2565	50	2	Banjaranyar	<200	Mahoni	Swietenia macrophylla King	Meliaceae	0,61	11,8	4	7	0,04	0,01	38,77	0,04	0,46	1,67
2566	50	2	Banjaranyar	<200	Mahoni	Swietenia macrophylla King	Meliaceae	0,61	3,5	3	4	0,04	0,00	2,52	0,00	0,03	0,11
2567	50	2	Banjaranyar	<200	tisuk	Hibiscus macrophyllus Roxb. ex I	Malvaceae	0,4	8,0	7	8	0,04	0,00	14,52	0,01	0,17	0,63
2568	50	2	Banjaranyar	<200	Mahoni	Swietenia macrophylla King	Meliaceae	0,61	9,5	3	7	0,04	0,01	26,40	0,03	0,31	1,14
2569	50	2	Banjaranyar	<200	Jati	Tectona grandis L.f.	Lamiaceae / Verbenaceae	0,7	19,1	10	14	0,04	0,03	201,21	0,20	2,36	8,68
2570	50	2	Banjaranyar	<200	Jati	Tectona grandis L.f.	Lamiaceae / Verbenaceae	0,7	8,0	3	5	0,04	0,00	15,76	0,02	0,19	0,68
2571	50	2	Banjaranyar	<200	tisuk	Hibiscus macrophyllus Roxb. ex I	Malvaceae	0,4	14,0	8	11	0,04	0,02	54,74	0,05	0,64	2,36
2572	50	2	Banjaranyar	<200	Jati	Tectona grandis L.f.	Lamiaceae / Verbenaceae	0,7	15,9	6	11	0,04	0,02	115,52	0,12	1,36	4,98
2573	50	2	Banjaranyar	<200	Jati	Tectona grandis L.f.	Lamiaceae / Verbenaceae	0,7	7,6	3	8	0,04	0,00	22,49	0,02	0,26	0,97
2574	50	2	Banjaranyar	<200	Jati	Tectona grandis L.f.	Lamiaceae / Verbenaceae	0,7	14,0	6	10	0,04	0,02	83,76	0,08	0,98	3,61
2575	50	2	Banjaranyar	<200	Mahoni	Swietenia macrophylla King	Meliaceae	0,61	3,2	2	2,5	0,04	0,00	1,37	0,00	0,02	0,06
2576	50	2	Banjaranyar	<200	Jati	Tectona grandis L.f.	Lamiaceae / Verbenaceae	0,7	18,8	7	12	0,04	0,03	169,42	0,17	1,99	7,31
2577	50	2	Banjaranyar	<200	Jati	Tectona grandis L.f.	Lamiaceae / Verbenaceae	0,7	16,9	6	9	0,04	0,02	106,95	0,11	1,26	4,61
2578	50	2	Banjaranyar	<200	Tisuk	Hibiscus macrophyllus Roxb. ex I	Malvaceae	0,4	11,8	6	8	0,04	0,01	29,77	0,03	0,35	1,28
2579	50	2	Banjaranyar	<200	Mahoni	Swietenia macrophylla King	Meliaceae	0,61	3,5	2	3	0,04	0,00	1,93	0,00	0,02	0,08
2580	51	2	Ciamis	200-500	Mahoni	Swietenia macrophylla King	Meliaceae	0,61	19,7	10	16	0,04	0,03	212,87	0,21	2,50	9,18
2581	51	2	Ciamis	200-500	Durian	Durio zibethinus L.	Malvaceae	0,57	27,0	16	18	0,04	0,06	397,22	0,40	4,67	17,13
2582	51	2	Ciamis	200-500	Mahoni	Swietenia macrophylla King	Meliaceae	0,61	20,0	11	17	0,04	0,03	231,72	0,23	2,72	9,99
2583	51	2	Ciamis	200-500	Mahoni	Swietenia macrophylla King	Meliaceae	0,61	16,2	4	12	0,04	0,02	114,36	0,11	1,34	4,93
2584	51	2	Ciamis	200-500	Mahoni	Swietenia macrophylla King	Meliaceae	0,61	14,0	1,5	9	0,04	0,02	67,05	0,07	0,79	2,89
2585	51	2	Ciamis	200-500	Tisuk	Hibiscus macrophyllus Roxb. ex I	Malvaceae	0,4	5,4	4	8	0,04	0,00	7,16	0,01	0,08	0,31
2586	51	2	Ciamis	200-500	tisuk	Hibiscus macrophyllus Roxb. ex I	Malvaceae	0,4	5,4	4	8	0,04	0,00	7,16	0,01	0,08	0,31
2587	51	2	Ciamis	200-500	Tisuk	Hibiscus macrophyllus Roxb. ex I	Malvaceae	0,4	4,8	5	9	0,04	0,00	6,34	0,01	0,07	0,27
2588	51	2	Ciamis	200-500	Mahoni	Swietenia macrophylla King	Meliaceae	0,61	16,9	6	12	0,04	0,02	122,71	0,12	1,44	5,29
2589	51	2	Ciamis	200-500	Tisuk	Hibiscus macrophyllus Roxb. ex I	Malvaceae	0,4	4,8	4	15	0,04	0,00	10,13	0,01	0,12	0,44
2590	51	2	Ciamis	200-500	Tisuk	Hibiscus macrophyllus Roxb. ex I	Malvaceae	0,4	6,0	5	9	0,04	0,00	9,78	0,01	0,11	0,42
2591	51	2	Ciamis	200-500	Sengon	Paraserianthes falcataria (L.) I.C.	Leguminosae / Fabaceae	0,33	9,9	12	15	0,04	0,01	32,10	0,03	0,38	1,38
2592	51	2	Ciamis	200-500	Mahoni	Swietenia macrophylla King	Meliaceae	0,61	14,3	8	14	0,04	0,02	104,72	0,10	1,23	4,52
2593	51	2	Ciamis	200-500	Kokosan	Lansium domesticum var. aquae	Meliaceae	0,7	4,1	1,7	6	0,04	0,00	5,62	0,01	0,07	0,24
2594	51	2	Ciamis	200-500	kelapa	Cocos nucifera L.	Arecaceae	0,5	27,7	15	17	0,04	0,06	135,40	0,14	1,59	5,84
2595	51	2	Ciamis	200-500	Tisuk	Hibiscus macrophyllus Roxb. ex I	Malvaceae	0,4	5,1	3	5	0,04	0,00	4,17	0,00	0,05	0,18
2596	51	2	Ciamis	200-500	Sengon	Paraserianthes falcataria (L.) I.C.	Leguminosae / Fabaceae	0,33	30,2	18	24	0,04	0,07	384,19	0,38	4,51	16,57
2597	51	2	Ciamis	200-500	Tisuk	Hibiscus macrophyllus Roxb. ex I	Malvaceae	0,4	5,7	6	9	0,04	0,00	8,86	0,01	0,10	0,38
2598	51	2	Ciamis	200-500	Tisuk	Hibiscus macrophyllus Roxb. ex I	Malvaceae	0,4	4,8	5	8	0,04	0,00	5,69	0,01	0,07	0,25

2599	51	2	Ciamis	200-500	Sengon	Paraserianthes falcataria (L.) I.C.	Leguminosae / Fabaceae		0,33	9,5	10	12	0,04	0,01	24,64	0,02	0,29	1,06
2600	51	2	Ciamis	200-500	Tisuk	Hibiscus macrophyllus Roxb. ex I	Malvaceae		0,4	15,6	10	12	0,04	0,02	72,21	0,07	0,85	3,11
2601	51	2	Ciamis	200-500	Durian	Durio zibethinus L.	Malvaceae		0,57	5,4	1	3,5	0,04	0,00	4,65	0,00	0,05	0,20
2602	51	2	Ciamis	200-500	Tisuk	Hibiscus macrophyllus Roxb. ex I	Malvaceae		0,4	7,0	8	10	0,04	0,00	14,09	0,01	0,17	0,61
2603	51	2	Ciamis	200-500	Sengon	Paraserianthes falcataria (L.) I.C.	Leguminosae / Fabaceae		0,33	36,3	12	18	0,04	0,10	412,25	0,41	4,84	17,78
2604	51	2	Ciamis	200-500	Tisuk	Hibiscus macrophyllus Roxb. ex I	Malvaceae		0,4	5,7	7	11	0,04	0,00	10,65	0,01	0,13	0,46
2605	51	2	Ciamis	200-500	Mahoni	Swietenia macrophylla King	Meliaceae		0,61	17,8	7	14	0,04	0,02	156,32	0,16	1,84	6,74
2606	51	2	Ciamis	200-500	Nangka	Artocarpus heterophyllus Lam.	Moraceae		0,61	12,1	2,5	5	0,04	0,01	29,92	0,03	0,35	1,29
2607	51	2	Ciamis	200-500	Tisuk	Hibiscus macrophyllus Roxb. ex I	Malvaceae		0,4	4,8	4	8	0,04	0,00	5,69	0,01	0,07	0,25
2608	51	2	Ciamis	200-500	Mahoni	Swietenia macrophylla King	Meliaceae		0,61	17,8	3,5	12,5	0,04	0,02	140,91	0,14	1,66	6,08
2609	51	2	Ciamis	200-500	Tisuk	Hibiscus macrophyllus Roxb. ex I	Malvaceae		0,4	4,5	6	7,5	0,04	0,00	4,73	0,00	0,06	0,20
2610	51	2	Ciamis	200-500	kelapa	Cocos nucifera L.	Arecaceae		0,5	27,7	12	16	0,04	0,06	127,70	0,13	1,50	5,51
2611	51	2	Ciamis	200-500	Tisuk	Hibiscus macrophyllus Roxb. ex I	Malvaceae		0,4	3,2	4	5,5	0,04	0,00	1,92	0,00	0,02	0,08
2612	51	2	Ciamis	200-500	Tisuk	Hibiscus macrophyllus Roxb. ex I	Malvaceae		0,4	6,0	8	9	0,04	0,00	9,78	0,01	0,11	0,42
2613	51	2	Ciamis	200-500	Kanyere	Bridelia stipularis (L.) Blume	Phyllanthaceae		0,44	2,2	5	6	0,04	0,00	1,18	0,00	0,01	0,05
2614	51	2	Ciamis	200-500	Mahoni	Swietenia macrophylla King	Meliaceae		0,61	5,1	3	6	0,04	0,00	7,25	0,01	0,09	0,31
2615	51	2	Ciamis	200-500	Mahoni	Swietenia macrophylla King	Meliaceae		0,61	4,5	2,5	5	0,04	0,00	4,80	0,00	0,06	0,21
2616	51	2	Ciamis	200-500	Mahoni	Swietenia macrophylla King	Meliaceae		0,61	12,4	6	11	0,04	0,01	64,60	0,06	0,76	2,79
2617	51	2	Ciamis	200-500	Mahoni	Swietenia macrophylla King	Meliaceae		0,61	7,0	5	9	0,04	0,00	18,83	0,02	0,22	0,81
2618	51	2	Ciamis	200-500	kelapa	Cocos nucifera L.	Arecaceae		0,5	25,8	11	14	0,04	0,05	112,30	0,11	1,32	4,84
2619	51	2	Ciamis	200-500	Melinjo	Gnetum gnemon L.	Gnetaceae		0,76	11,1	2	12	0,04	0,01	70,18	0,07	0,82	3,03
2620	51	2	Ciamis	200-500	Mahoni	Swietenia macrophylla King	Meliaceae		0,61	15,9	7	13	0,04	0,02	118,68	0,12	1,39	5,12
2621	51	2	Ciamis	200-500	Durian	Durio zibethinus L.	Malvaceae		0,57	2,2	2	3	0,04	0,00	0,79	0,00	0,01	0,03
2622	51	2	Ciamis	200-500	Mahoni	Swietenia macrophylla King	Meliaceae		0,61	7,0	5	8	0,04	0,00	16,91	0,02	0,20	0,73
2623	51	2	Ciamis	200-500	Mahoni	Swietenia macrophylla King	Meliaceae		0,61	2,9	4	13	0,04	0,00	5,13	0,01	0,06	0,22
2624	51	2	Ciamis	200-500	Sengon	Paraserianthes falcataria (L.) I.C.	Leguminosae / Fabaceae		0,33	9,9	14	17	0,04	0,01	36,00	0,04	0,42	1,55
2625	51	2	Ciamis	200-500	Mahoni	Swietenia macrophylla King	Meliaceae		0,61	11,8	3	11	0,04	0,01	58,66	0,06	0,69	2,53
2626	51	2	Ciamis	200-500	Mahoni	Swietenia macrophylla King	Meliaceae		0,61	10,8	4	10	0,04	0,01	46,04	0,05	0,54	1,99
2627	51	2	Ciamis	200-500	kelapa	Cocos nucifera L.	Arecaceae		0,5	28,0	11	15	0,04	0,06	120,00	0,12	1,41	5,17
2628	51	2	Ciamis	200-500	Mahoni	Swietenia macrophylla King	Meliaceae		0,61	11,5	6	11	0,04	0,01	55,79	0,06	0,66	2,41
2629	51	2	Ciamis	200-500	Mahoni	Swietenia macrophylla King	Meliaceae		0,61	12,4	5	10	0,04	0,01	59,20	0,06	0,70	2,55
2630	51	2	Ciamis	200-500	Durian	Durio zibethinus L.	Malvaceae		0,57	5,4	8	10	0,04	0,00	12,15	0,01	0,14	0,52
2631	51	2	Ciamis	200-500	Mahoni	Swietenia macrophylla King	Meliaceae		0,61	6,0	3,5	6,5	0,04	0,00	10,69	0,01	0,13	0,46
2632	51	2	Ciamis	200-500	Mahoni	Swietenia macrophylla King	Meliaceae		0,61	7,0	2	8	0,04	0,00	16,91	0,02	0,20	0,73
2633	51	2	Ciamis	200-500	Mahoni	Swietenia macrophylla King	Meliaceae		0,61	12,1	8	13	0,04	0,01	71,78	0,07	0,84	3,10
2634	51	2	Ciamis	200-500	Mahoni	Swietenia macrophylla King	Meliaceae		0,61	11,8	7	13	0,04	0,01	68,36	0,07	0,80	2,95
2635	51	2	Ciamis	200-500	Tisuk	Hibiscus macrophyllus Roxb. ex I	Malvaceae		0,4	14,3	12	16	0,04	0,02	80,40	0,08	0,94	3,47
2636	51	2	Ciamis	200-500	Mahoni	Swietenia macrophylla King	Meliaceae		0,61	13,7	6	12	0,04	0,01	83,66	0,08	0,98	3,61
2637	51	2	Ciamis	200-500	Mahoni	Swietenia macrophylla King	Meliaceae		0,61	15,3	7	15	0,04	0,02	125,55	0,13	1,48	5,41
2638	51	2	Ciamis	200-500	Mahoni	Swietenia macrophylla King	Meliaceae		0,61	5,7	3	9	0,04	0,00	13,04	0,01	0,15	0,56
2639	51	2	Ciamis	200-500	Mahoni	Swietenia macrophylla King	Meliaceae		0,61	10,2	3	7	0,04	0,01	29,72	0,03	0,35	1,28
2640	51	2	Ciamis	200-500	Mahoni	Swietenia macrophylla King	Meliaceae		0,61	10,2	3	9	0,04	0,01	37,41	0,04	0,44	1,61
2641	51	2	Ciamis	200-500	Durian	Durio zibethinus L.	Malvaceae		0,57	4,1	4	8	0,04	0,00	6,06	0,01	0,07	0,26
2642	51	2	Ciamis	200-500	Mahoni	Swietenia macrophylla King	Meliaceae		0,61	7,0	3	8	0,04	0,00	16,91	0,02	0,20	0,73
2643	51	2	Ciamis	200-500	Mahoni	Swietenia macrophylla King	Meliaceae		0,61	14,6	5	11	0,04	0,02	87,41	0,09	1,03	3,77
2644	51	2	Ciamis	200-500	Tisuk	Hibiscus macrophyllus Roxb. ex I	Malvaceae		0,4	4,5	3	7	0,04	0,00	4,44	0,00	0,05	0,19
2645	51	2	Ciamis	200-500	kelapa	Cocos nucifera L.	Arecaceae		0,5	26,4	10	13	0,04	0,05	104,60	0,10	1,23	4,51
2646	51	2	Ciamis	200-500	Jengkol	Archidendron pauciflorum (Bentl	Leguminosae / Fabaceae		0,4	4,5	5	8	0,04	0,00	5,02	0,01	0,06	0,22
2647	51	2	Ciamis	200-500	Mahoni	Swietenia macrophylla King	Meliaceae		0,61	15,6	5	11	0,04	0,02	98,14	0,10	1,15	4,23
2648	51	2	Ciamis	200-500	Jati	Tectona grandis L.f.	Lamiaceae / Verbenaceae		0,7	4,5	2	6	0,04	0,00	6,44	0,01	0,08	0,28
2649	51	2	Ciamis	200-500	Sengon	Paraserianthes falcataria (L.) I.C.	Leguminosae / Fabaceae		0,33	23,9	12	19	0,04	0,04	201,15	0,20	2,36	8,67
2650	51	2	Ciamis	200-500	Jati	Tectona grandis L.f.	Lamiaceae / Verbenaceae		0,7	11,5	3,5	15	0,04	0,01	84,08	0,08	0,99	3,63
2651	51	2	Ciamis	200-500	Durian	Durio zibethinus L.	Malvaceae		0,57	4,8	4	6	0,04	0,00	6,05	0,01	0,07	0,26
2652	51	2	Ciamis	200-500	Mahoni	Swietenia macrophylla King	Meliaceae		0,61	14,0	3	10	0,04	0,02	73,84	0,07	0,87	3,18
2653	51	2	Ciamis	200-500	Alpukat	Persea americana Mill.	Lauraceae		0,54	4,5	2,5	6	0,04	0,00	5,08	0,01	0,06	0,22
2654	51	2	Ciamis	200-500	Mahoni	Swietenia macrophylla King	Meliaceae		0,61	9,9	6	8	0,04	0,01	31,69	0,03	0,37	1,37
2655	51	2	Ciamis	200-500	Mahoni	Swietenia macrophylla King	Meliaceae		0,61	7,6	4	5,5	0,04	0,00	14,07	0,01	0,17	0,61
2656	51	2	Ciamis	200-500	Mahoni	Swietenia macrophylla King	Meliaceae		0,61	13,7	6	11	0,04	0,01	77,25	0,08	0,91	3,33
2657	51	2	Ciamis	200-500	Mahoni	Swietenia macrophylla King	Meliaceae		0,61	11,5	4	5,5	0,04	0,01	29,57	0,03	0,35	1,27
2658	51	2	Ciamis	200-500	Mahoni	Swietenia macrophylla King	Meliaceae		0,61	9,5	2,5	5	0,04	0,01	19,40	0,02	0,23	0,84
2659	51	2	Ciamis	200-500	Mahoni	Swietenia macrophylla King	Meliaceae		0,61	8,6	5	9	0,04	0,01	27,40	0,03	0,32	1,18
2660	52	2	Ciamis	200-500	Namnam/Pukih	Cynometra cauliflora L.	Leguminosae / Fabaceae		0,72	9,9	2,5	6,5	0,04	0,01	30,50	0,03	0,36	1,32
2661	52	2	Ciamis	200-500	Sawo / sawo manila	Manilkara zapota (L.) P.Royen	Sapotaceae		0,81	25,5	4	9	0,04	0,05	259,92	0,26	3,05	11,21
2662	52	2	Ciamis	200-500	Dukuh	Lansium domesticum Corrêa	Meliaceae		0,85	32,8	4	15	0,04	0,08	689,10	0,69	8,10	29,72
2663	52	2	Ciamis	200-500	Pisitan	Dysoxylum parasiticum (Osbeck	Meliaceae		0,6	8,6	4	10	0,04	0,01	29,73	0,03	0,35	1,28
2664	52	2	Ciamis	200-500	Kokosan	Lansium domesticum var. aquae	Meliaceae		0,7	7,3	4	7	0,04	0,00	18,41	0,02	0,22	0,79
2665	52	2	Ciamis	200-500	Tisuk	Hibiscus macrophyllus Roxb. ex I	Malvaceae		0,4	29,0	14	19	0,04	0,07	341,91	0,34	4,02	14,74
2666	52	2	Ciamis	200-500	Kelapa	Cocos nucifera L.	Arecaceae		0,5	26,4	12	15	0,04	0,05	120,00	0,12	1,41	5,17
2667	52	2	Ciamis	200-500	Melinjo	Gnetum gnemon L.	Gnetaceae		0,76	28,0	11	18	0,04	0,06	550,89	0,55	6,47	23,76
2668	52	2	Ciamis	200-500	Namnam/Pukih	Cynometra cauliflora L.	Leguminosae / Fabaceae		0,72	15,0	2	8	0,04	0,02	79,06	0,08	0,93	3,41
2669	52	2	Ciamis	200-500	Mahoni	Swietenia macrophylla King	Meliaceae		0,61	9,9	5	9	0,04	0,01	35,30	0,04	0,41	1,52
2670	52	2	Ciamis	200-500	Kokosan	Lansium domesticum var. aquae	Meliaceae		0,7	14,0	7	9	0,04	0,02	76,05	0,08	0,89	3,28
2671	52	2	Ciamis	200-500	Dukuh	Lansium domesticum Corrêa	Meliaceae		0,85	36,0	5	13	0,04	0,10	716,27	0,72	8,42	30,89
2672	52	2	Ciamis	200-500	Nangka	Artocarpus heterophyllus Lam.	Moraceae		0,61	40,4	8	15	0,04	0,13	746,36	0,75	8,77	32,18
2673	52	2	Ciamis	200-500	Kokosan	Lansium domesticum var. aquae	Meliaceae		0,7	27,0	4	13	0,04	0,06	355,87	0,36	4,18	15,35
2674	52	2	Ciamis	200-500	Namnam/Pukih	Cynometra cauliflora L.	Leguminosae / Fabaceae		0,72	7,6	2,5	7	0,04	0,00	20,42	0,02	0,24	0,88
2675	52	2	Ciamis	200-500	Dukuh	Lansium domesticum Corrêa	Meliaceae		0,85	39,5	4	13	0,04	0,12	849,16	0,85	9,98	36,62
2676	52	2	Ciamis	200-500	Melinjo	Gnetum gnemon L.	Gnetaceae		0,76	21,3	10	15	0,04	0,04	282,89	0,28	3,32	12,20
2677	52	2	Ciamis	200-500	Kelapa	Cocos nucifera L.	Arecaceae		0,5	26,7	17	20	0,04	0,06	158,50	0,16	1,86	6,83
2678	52	2	Ciamis	200-500	Dukuh	Lansium domesticum Corrêa	Meliaceae		0,85	12,7	5	10	0,04	0,01	84,03	0,08	0,99	3,62
2679	52	2	Ciamis	200-500	Kokosan	Lansium domesticum var. aquae	Meliaceae		0,7	18,0	10	16	0,04	0,03	203,68	0,20	2,39	8,78
2680	52	2	Ciamis	200-500	Kelapa	Cocos nucifera L.	Arecaceae		0,5	33,7	14	17	0,04	0,09	135,40	0,14	1,59	5,84
2681	52	2	Ciamis	200-500	Balam	Palaquium warsurifolium	Sapotaceae		0,6	21,0	10	15	0,04	0,03	221,62	0,22	2,60	9,56
2682	52	2	Ciamis	200-500	Aren	Arenga pinnata (Wurmb) Merr.	Arecaceae		0,71	29,0	7	10	0,04	0,07	81,50	0,08	0,96	3,51
2683	53	2	Ciamis	200-500	Caruy	Pterospermum javanicum Jungh.	Malvaceae		0,53	6,7	6	8	0,04	0,00	13,65	0,01	0,16	0,59
2684	53	2	Ciamis	200-500	Kelapa	Cocos nucifera L.	Arecaceae		0,5	29,9	15	18	0,04	0,07	143,10	0,14	1,68	6,17
2685	53	2	Ciamis	200-500	Caruy	Pterospermum javanicum Jungh.	Malvaceae		0,53	9,5	8	10	0,04	0,01	32,18	0,03	0,38	1,39
2686	53	2	Ciamis	200-500	Kelapa	Cocos nucifera L.	Arecaceae		0,5	29,0	15	18	0,04	0,07	143,10	0,14	1,68	6,17
2687	53	2	Ciamis	200-500	Caruy	Pterospermum javanicum Jungh.	Malvaceae		0,53	7,5	2	9	0,04	0,00	18,68	0,02	0,22	0,81
2688	53	2	Ciamis	200-500	Caruy	Pterospermum javanicum Jungh.	Malvaceae		0,53	17,5	9	17	0,04	0,02	158,85	0,16	1,87	6,85
2689	53	2	Ciamis	200-500	Sengon	Paraserianthes falcataria (L.) I.C.	Leguminosae / Fabaceae		0,33	29,6	17	21	0,04	0,07	326,96	0,33	3,84	14,10
2690	53	2	Ciamis	200-500	Caruy	Pterospermum javanicum Jungh.	Malvaceae		0,53	16,2	7	12	0,04	0,02	100,54	0,10	1,18	4,34
2691	53	2	Ciamis	200-500	Caruy	Pterospermum javanicum Jungh.	Malvaceae		0,53	23,9	6	12	0,04	0,04	203,80	0,20	2,39	8,79
2692	53	2	Ciamis	200-500	Caruy	Pterospermum javanicum Jungh.	Malvaceae		0,53	21,3	12	16	0,04	0,04	215,72	0,22	2,53	9,30
2693	53	2	Ciamis	200-500	Suren	Toona sureni (Blume) Merr.	Meliaceae		0,39	10,8	6	8	0,04	0,01	24,91	0,02	0,29	1,07
2694	53	2	Ciamis	200-500	Caruy	Pterospermum javanicum Jungh.	Malvaceae		0,53	12,4	2	10	0,04	0,01	52,05	0,05	0,61	2,24
2695	53	2	Ciamis	200-500	Durian	Durio zibethinus L.	Malvaceae		0,57	19,7	2,5	11,5	0,04	0,03	147,83	0,15	1,74	6,37
2696	53	2	Ciamis	200-500	Caruy	Pterospermum javanicum Jungh.	Malvaceae		0,53	8,0	3,5	8,5	0,04	0,00	19,86	0,02	0,23	0,86
2697	53	2	Ciamis	200-500	Caruy	Pterospermum javanicum Jungh.	Malvaceae		0,53	13,7	8	14	0,04	0,01	84,71	0,08	1,00	3,65
2698	53	2	Ciamis	200-500	Sengon	Paraserianthes falcataria (L.) I.C.	Leguminosae / Fabaceae		0,33	17,5	14	17	0,04	0,02	102,92	0,10	1,21	4,44
2699	53	2	Ciamis	200-500	Caruy	Pterospermum javanicum Jungh.	Malvaceae		0,53	7,0	7	9	0,04	0,00	16,56	0,02	0,19	0,71
2700	53	2	Ciamis	200-500	Sengon	Paraserianthes falcataria (L.) I.C.	Leguminosae / Fabaceae		0,33	33,1	18	21	0,04	0,09	401,27	0,40	4,71	17,30
2701	53	2	Ciamis	200-500	Caruy	Pterospermum javanicum Jungh.	Malvaceae		0,53	7,3	6	8	0,04	0,00	16,12	0,02	0,19	0,70
2702	53	2	Ciamis	200-500	Kelapa	Cocos nucifera L.	Arecaceae		0,5	29,9	14	17	0,04	0,07	135,40	0,14	1,59	5,84
2703	53	2	Ciamis	200-500	Caruy	Pterospermum javanicum Jungh.	Malvaceae		0,53	10,2	7	10	0,04	0,01	36,22	0,04	0,43	1,56
2704	53	2	Ciamis	200-500	Sengon	Paraserianthes falcataria (L.) I.C.	Leguminosae / Fabaceae		0,33	19,1	15	18	0,04	0,03	127,20	0,13	1,49	5,49
2705	53	2	Ciamis	200-500	Sengon	Paraserianthes falcataria (L.) I.C.	Leguminosae / Fabaceae		0,33	25,1	15	19	0,04	0,05	221,24	0,22	2,60	9,54
2706	53	2	Ciamis	200-500	Suren	Toona sureni (Blume) Merr.	Meliaceae		0,39	9,5	6	10	0,04	0,01	24,30	0,02	0,29	1,05
2707	53	2	Ciamis	200-500	Caruy	Pterospermum javanicum Jungh.	Malvaceae		0,53	11,8	9	13	0,04	0,01	60,10	0,06	0,71	2,59
2708	53	2	Ciamis	200-500	Suren	Toona sureni (Blume) Merr.	Meliaceae		0,39	8,0	5	10	0,04	0,00	17,40	0,02	0,20	0,75
2709	53	2	Ciamis	200-500	Caruy	Pterospermum javanicum Jungh.	Malvaceae		0,53	10,3	4	12	0,04	0,01	44,04	0,04	0,52	1,90
2710	53	2	Ciamis	200-500	Sengon	Paraserianthes falcataria (L.) I.C.	Leguminosae / Fabaceae		0,33	9,2	4	8	0,04	0,01	15,97	0,02	0,19	0,69
2711	53	2	Ciamis	200-500	Sp8	Sp8		0	0,5	6,0	5	6,5	0,04	0,00	8,91	0,01	0,10	0,38
2712	53	2	Ciamis	200-500	Caruy	Pterospermum javanicum Jungh.	Malvaceae		0,53	19,1	9	15	0,04	0,03	166,12	0,17	1,95	7,16
2713	53	2	Ciamis	200-500	Caruy	Pterospermum javanicum Jungh.	Malvaceae		0,53	18,8	7	14	0,04	0,03	151,22	0,15	1,78	6,52
2714	53	2	Ciamis	200-500	Suren	Toona sureni (Blume) Merr.	Meliaceae		0,39	3,5	4	4,5	0,04	0,00	1,86	0,00	0,02	0,08
2715	53	2	Ciamis	200-500	Caruy	Pterospermum javanicum Jungh.	Malvaceae		0,53	12,1	7	12	0,04	0,01	58,65	0,06	0,69	2,53
2716	53	2	Ciamis	200-500	Sengon	Paraserianthes falcataria (L.) I.C.	Leguminosae / Fabaceae		0,33	14,6	11	15	0,04	0,02	66,15	0,07	0,78	2,85
2717	53	2	Ciamis	200-500	Durian	Durio zibethinus L.	Malvaceae		0,57	43,9	10	16	0,04	0,15	866,43	0,87	10,18	37,36
2718	53	2	Ciamis	200-500	Caruy	Pterospermum javanicum Jungh.	Malvaceae		0,53	18,1	8	14	0,04	0,03	141,96	0,14	1,67	6,12
2719	53	2	Ciamis	200-500	Suren	Toona sureni (Blume) Merr.	Meliaceae		0,39	5,7	4	6	0,04	0,00	5,97	0,01	0,07	0,26
2720	53	2	Ciamis	200-500	Caruy	Pterospermum javanicum Jungh.	Malvaceae		0,53	7,0	2,5	6,5	0,04	0,00	12,29	0,01	0,14	0,53
2721	53	2	Ciamis	200-500	Caruy	Pterospermum javanicum Jungh.	Malvaceae		0,53	8,9	8	10,5	0,04	0,01	29,66	0,03	0,35	1,28
2722	53	2	Ciamis	200-500	Caruy	Pterospermum javanicum Jungh.	Malvaceae		0,53	3,8	2	4	0,04	0,00	2,59	0,00	0,03	0,11

2723	53	2	Ciamis	200-500	Caruy	Pterospermum javanicum Jungh.	Malvaceae	0,53	15,0	10	14	0,04	0,02	99,70	0,10	1,17	4,30
2724	53	2	Ciamis	200-500	Caruy	Pterospermum javanicum Jungh.	Malvaceae	0,53	3,5	1,8	3,5	0,04	0,00	1,96	0,00	0,02	0,08
2725	53	2	Ciamis	200-500	Dukuh	Lansium domesticum Corrêa	Meliaceae	0,85	53,5	1,8	13,8	0,04	0,22	1.564,45	1,56	18,38	67,46
2726	53	2	Ciamis	200-500	Caruy	Pterospermum javanicum Jungh.	Malvaceae	0,53	3,2	1,5	3,5	0,04	0,00	1,64	0,00	0,02	0,07
2727	53	2	Ciamis	200-500	Caruy	Pterospermum javanicum Jungh.	Malvaceae	0,53	7,3	2,5	9,5	0,04	0,00	18,87	0,02	0,22	0,81
2728	53	2	Ciamis	200-500	Sengon	Paraserianthes falcataria (L.) I.C.	Leguminosae / Fabaceae	0,33	39,1	12	21	0,04	0,12	545,68	0,55	6,41	23,53
2729	53	2	Ciamis	200-500	Tisuk	Hibiscus macrophyllus Roxb. ex	Malvaceae	0,4	20,0	12	17	0,04	0,03	157,43	0,16	1,85	6,79
2730	53	2	Ciamis	200-500	Caruy	Pterospermum javanicum Jungh.	Malvaceae	0,53	8,3	9	10,5	0,04	0,01	25,89	0,03	0,30	1,12
2731	53	2	Ciamis	200-500	Sengon	Paraserianthes falcataria (L.) I.C.	Leguminosae / Fabaceae	0,33	46,8	13	24	0,04	0,17	854,82	0,85	10,04	36,86
2732	53	2	Ciamis	200-500	Sengon	Paraserianthes falcataria (L.) I.C.	Leguminosae / Fabaceae	0,33	28,3	16	21	0,04	0,06	301,66	0,30	3,54	13,01
2733	53	2	Ciamis	200-500	Caruy	Pterospermum javanicum Jungh.	Malvaceae	0,53	7,3	8	10	0,04	0,00	19,78	0,02	0,23	0,85
2734	53	2	Ciamis	200-500	Caruy	Pterospermum javanicum Jungh.	Malvaceae	0,53	6,7	5	6,5	0,04	0,00	11,28	0,01	0,13	0,49
2735	53	2	Ciamis	200-500	Caruy	Pterospermum javanicum Jungh.	Malvaceae	0,53	5,7	3	5	0,04	0,00	6,69	0,01	0,08	0,29
2736	53	2	Ciamis	200-500	Caruy	Pterospermum javanicum Jungh.	Malvaceae	0,53	8,0	7	8,5	0,04	0,00	19,86	0,02	0,23	0,86
2737	53	2	Ciamis	200-500	Tisuk	Hibiscus macrophyllus Roxb. ex	Malvaceae	0,4	12,7	6	14	0,04	0,01	57,34	0,06	0,67	2,47
2738	53	2	Ciamis	200-500	Sengon	Paraserianthes falcataria (L.) I.C.	Leguminosae / Fabaceae	0,33	15,3	14	15	0,04	0,02	71,52	0,07	0,84	3,08
2739	54	2	Ciamis	200-500	Mahoni	Swietenia macrophylla King	Meliaceae	0,61	20,7	6	15	0,04	0,03	218,80	0,22	2,57	9,44
2740	54	2	Ciamis	200-500	Durian	Durio zibethinus L.	Malvaceae	0,57	24,5	22	27	0,04	0,05	480,49	0,48	5,65	20,72
2741	54	2	Ciamis	200-500	Durian	Durio zibethinus L.	Malvaceae	0,57	19,7	14	21	0,04	0,03	256,63	0,26	3,02	11,07
2742	54	2	Ciamis	200-500	Mahoni	Swietenia macrophylla King	Meliaceae	0,61	3,2	2	3,5	0,04	0,00	1,87	0,00	0,02	0,08
2743	54	2	Ciamis	200-500	Durian	Durio zibethinus L.	Malvaceae	0,57	6,7	7	10	0,04	0,00	17,90	0,02	0,21	0,77
2744	54	2	Ciamis	200-500	Durian	Durio zibethinus L.	Malvaceae	0,57	9,5	6,5	10	0,04	0,01	34,40	0,03	0,40	1,48
2745	54	2	Ciamis	200-500	Mahoni	Swietenia macrophylla King	Meliaceae	0,61	34,0	12	21	0,04	0,09	742,11	0,74	8,72	32,00
2746	54	2	Ciamis	200-500	Durian	Durio zibethinus L.	Malvaceae	0,57	24,5	25	31	0,04	0,05	545,30	0,55	6,41	23,51
2747	54	2	Ciamis	200-500	Mahoni	Swietenia macrophylla King	Meliaceae	0,61	41,0	7	19	0,04	0,13	953,72	0,95	11,21	41,13
2748	54	2	Ciamis	200-500	Mahoni	Swietenia macrophylla King	Meliaceae	0,61	17,8	6	14	0,04	0,02	156,32	0,16	1,84	6,74
2749	54	2	Ciamis	200-500	Kokosan	Lansium domesticum var. aquae	Meliaceae	0,7	3,8	4	7	0,04	0,00	5,59	0,01	0,07	0,24
2750	54	2	Ciamis	200-500	Mahoni	Swietenia macrophylla King	Meliaceae	0,61	6,0	2,5	6,5	0,04	0,00	10,69	0,01	0,13	0,46
2751	54	2	Ciamis	200-500	Melinjo	Gnetum gnemon L.	Gnetaceae	0,76	3,5	2,5	5	0,04	0,00	3,78	0,00	0,04	0,16
2752	54	2	Ciamis	200-500	Dukuh	Lansium domesticum Corrêa	Meliaceae	0,85	11,1	4	12	0,04	0,01	77,76	0,08	0,91	3,35
2753	54	2	Ciamis	200-500	Kemiri	Aleurites moluccanus (L.) Willd.	Euphorbiaceae	0,31	47,4	10	20	0,04	0,18	158,50	0,16	1,86	6,83
2754	54	2	Ciamis	200-500	Mahoni	Swietenia macrophylla King	Meliaceae	0,61	10,2	4,5	8,5	0,04	0,01	35,50	0,04	0,42	1,53
2755	54	2	Ciamis	200-500	Kelapa	Cocos nucifera L.	Arecaceae	0,5	29,3	12	15	0,04	0,07	120,00	0,12	1,41	5,17
2756	54	2	Ciamis	200-500	Mahoni	Swietenia macrophylla King	Meliaceae	0,61	10,2	7	11	0,04	0,01	44,96	0,04	0,53	1,94
2757	54	2	Ciamis	200-500	Mahoni	Swietenia macrophylla King	Meliaceae	0,61	13,7	9	14	0,04	0,01	96,35	0,10	1,13	4,15
2758	54	2	Ciamis	200-500	Mahoni	Swietenia macrophylla King	Meliaceae	0,61	12,7	9	14	0,04	0,01	84,39	0,08	0,99	3,64
2759	54	2	Ciamis	200-500	Mahoni	Swietenia macrophylla King	Meliaceae	0,61	7,3	4	8	0,04	0,00	18,34	0,02	0,22	0,79
2760	54	2	Ciamis	200-500	Mahoni	Swietenia macrophylla King	Meliaceae	0,61	7,3	7	12	0,04	0,00	26,59	0,03	0,31	1,15
2761	54	2	Ciamis	200-500	Kemiri	Aleurites moluccanus (L.) Willd.	Euphorbiaceae	0,31	40,4	12	17	0,04	0,13	135,40	0,14	1,59	5,84
2762	54	2	Ciamis	200-500	Mahoni	Swietenia macrophylla King	Meliaceae	0,61	5,7	4,5	6,5	0,04	0,00	9,68	0,01	0,11	0,42
2763	54	2	Ciamis	200-500	Mahoni	Swietenia macrophylla King	Meliaceae	0,61	0,2	6	9	0,04	0,00	0,02	0,00	0,00	0,00
2764	54	2	Ciamis	200-500	Mahoni	Swietenia macrophylla King	Meliaceae	0,61	5,7	5	8	0,04	0,00	11,70	0,01	0,14	0,50
2765	54	2	Ciamis	200-500	Mahoni	Swietenia macrophylla King	Meliaceae	0,61	12,4	6	11	0,04	0,01	64,60	0,06	0,76	2,79
2766	54	2	Ciamis	200-500	Mahoni	Swietenia macrophylla King	Meliaceae	0,61	6,4	2	7	0,04	0,00	12,56	0,01	0,15	0,54
2767	54	2	Ciamis	200-500	Mahoni	Swietenia macrophylla King	Meliaceae	0,61	5,7	3	8,5	0,04	0,00	12,37	0,01	0,15	0,53
2768	54	2	Ciamis	200-500	Mahoni	Swietenia macrophylla King	Meliaceae	0,61	11,8	6	11	0,04	0,01	58,66	0,06	0,69	2,53
2769	54	2	Ciamis	200-500	Mahoni	Swietenia macrophylla King	Meliaceae	0,61	5,7	5	8	0,04	0,00	11,70	0,01	0,14	0,50
2770	54	2	Ciamis	200-500	Mahoni	Swietenia macrophylla King	Meliaceae	0,61	10,2	8	12	0,04	0,01	48,69	0,05	0,57	2,10
2771	54	2	Ciamis	200-500	Mahoni	Swietenia macrophylla King	Meliaceae	0,61	6,0	8	11	0,04	0,00	17,30	0,02	0,20	0,75
2772	54	2	Ciamis	200-500	Mahoni	Swietenia macrophylla King	Meliaceae	0,61	7,0	6	10	0,04	0,00	20,74	0,02	0,24	0,89
2773	54	2	Ciamis	200-500	Mahoni	Swietenia macrophylla King	Meliaceae	0,61	17,8	7	13	0,04	0,02	146,06	0,15	1,72	6,30
2774	54	2	Ciamis	200-500	Mahoni	Swietenia macrophylla King	Meliaceae	0,61	8,3	2,5	7,5	0,04	0,01	21,64	0,02	0,25	0,93
2775	54	2	Ciamis	200-500	Mahoni	Swietenia macrophylla King	Meliaceae	0,61	11,5	5	6	0,04	0,01	32,02	0,03	0,38	1,38
2776	54	2	Ciamis	200-500	Mahoni	Swietenia macrophylla King	Meliaceae	0,61	6,7	4	6,5	0,04	0,00	12,84	0,01	0,15	0,55
2777	54	2	Ciamis	200-500	Mahoni	Swietenia macrophylla King	Meliaceae	0,61	8,3	2,5	7,5	0,04	0,01	21,64	0,02	0,25	0,93
2778	54	2	Ciamis	200-500	Mahoni	Swietenia macrophylla King	Meliaceae	0,61	8,3	3,5	9,5	0,04	0,01	26,87	0,03	0,32	1,16
2779	54	2	Ciamis	200-500	Mahoni	Swietenia macrophylla King	Meliaceae	0,61	22,3	4	13	0,04	0,04	219,82	0,22	2,58	9,48
2780	54	2	Ciamis	200-500	Mahoni	Swietenia macrophylla King	Meliaceae	0,61	8,6	4	9	0,04	0,01	27,40	0,03	0,32	1,18
2781	54	2	Ciamis	200-500	Mahoni	Swietenia macrophylla King	Meliaceae	0,61	10,2	9	14	0,04	0,01	56,08	0,06	0,66	2,42
2782	54	2	Ciamis	200-500	Mahoni	Swietenia macrophylla King	Meliaceae	0,61	12,4	6	4	0,04	0,01	25,57	0,03	0,30	1,10
2783	54	2	Ciamis	200-500	Mahoni	Swietenia macrophylla King	Meliaceae	0,61	6,7	5	8	0,04	0,00	15,52	0,02	0,18	0,67
2784	54	2	Ciamis	200-500	Mahoni	Swietenia macrophylla King	Meliaceae	0,61	11,5	8	13	0,04	0,01	65,01	0,07	0,76	2,80
2785	54	2	Ciamis	200-500	Mahoni	Swietenia macrophylla King	Meliaceae	0,61	19,1	12	19	0,04	0,03	234,64	0,23	2,76	10,12
2786	54	2	Ciamis	200-500	Mahoni	Swietenia macrophylla King	Meliaceae	0,61	10,8	5	10	0,04	0,01	46,04	0,05	0,54	1,99
2787	54	2	Ciamis	200-500	Mahoni	Swietenia macrophylla King	Meliaceae	0,61	16,9	5	11	0,04	0,02	113,31	0,11	1,33	4,89
2788	54	2	Ciamis	200-500	Mahoni	Swietenia macrophylla King	Meliaceae	0,61	8,3	6	10	0,04	0,01	28,17	0,03	0,33	1,21
2789	54	2	Ciamis	200-500	Mahoni	Swietenia macrophylla King	Meliaceae	0,61	24,5	8	17	0,04	0,05	334,68	0,33	3,93	14,43
2790	54	2	Ciamis	200-500	Mahoni	Swietenia macrophylla King	Meliaceae	0,61	9,5	3,5	8,5	0,04	0,01	31,54	0,03	0,37	1,36
2791	54	2	Ciamis	200-500	Mahoni	Swietenia macrophylla King	Meliaceae	0,61	24,5	9	18	0,04	0,05	352,66	0,35	4,14	15,21
2792	54	2	Ciamis	200-500	Mahoni	Swietenia macrophylla King	Meliaceae	0,61	10,2	5	10	0,04	0,01	41,20	0,04	0,48	1,78
2793	54	2	Ciamis	200-500	Mahoni	Swietenia macrophylla King	Meliaceae	0,61	21,6	5	17	0,04	0,04	266,52	0,27	3,13	11,49
2794	54	2	Ciamis	200-500	Mahoni	Swietenia macrophylla King	Meliaceae	0,61	8,9	6	11	0,04	0,01	35,20	0,04	0,41	1,52
2795	54	2	Ciamis	200-500	Mahoni	Swietenia macrophylla King	Meliaceae	0,61	6,4	4	9	0,04	0,00	15,81	0,02	0,19	0,68
2796	54	2	Ciamis	200-500	Mahoni	Swietenia macrophylla King	Meliaceae	0,61	5,1	4,5	7	0,04	0,00	8,35	0,01	0,10	0,36
2797	54	2	Ciamis	200-500	Mahoni	Swietenia macrophylla King	Meliaceae	0,61	8,3	4	9	0,04	0,01	25,57	0,03	0,30	1,10
2798	54	2	Ciamis	200-500	Mahoni	Swietenia macrophylla King	Meliaceae	0,61	4,5	3	4,5	0,04	0,00	4,36	0,00	0,05	0,19
2799	54	2	Ciamis	200-500	Mahoni	Swietenia macrophylla King	Meliaceae	0,61	10,8	7	12	0,04	0,01	54,41	0,05	0,64	2,35
2800	54	2	Ciamis	200-500	Durian	Durio zibethinus L.	Malvaceae	0,57	11,1	9	15	0,04	0,01	66,15	0,07	0,78	2,85
2801	54	2	Ciamis	200-500	Mahoni	Swietenia macrophylla King	Meliaceae	0,61	9,5	4	8	0,04	0,01	29,84	0,03	0,35	1,29
2802	54	2	Ciamis	200-500	Mahoni	Swietenia macrophylla King	Meliaceae	0,61	10,2	5	7	0,04	0,01	29,72	0,03	0,35	1,28
2803	54	2	Ciamis	200-500	Jengkol	Archidendron pauciflorum (Bentl	Leguminosae / Fabaceae	0,4	28,0	7	17	0,04	0,06	290,40	0,29	3,41	12,52
2804	54	2	Ciamis	200-500	Kihuut	Aporosa fructescens Blume	Phyllanthaceae	0,37	3,8	4	8	0,04	0,00	3,52	0,00	0,04	0,15
2805	55	1	Ciamis	200-500	Jati	Tectona grandis L.f.	Lamiaceae / Verbenaceae	0,7	8,6	2,5	7,5	0,04	0,01	26,31	0,03	0,31	1,13
2806	55	1	Ciamis	200-500	Jati	Tectona grandis L.f.	Lamiaceae / Verbenaceae	0,7	20,7	12	18	0,04	0,03	293,30	0,29	3,45	12,65
2807	55	1	Ciamis	200-500	Jati	Tectona grandis L.f.	Lamiaceae / Verbenaceae	0,7	21,6	12	18	0,04	0,04	318,58	0,32	3,74	13,74
2808	55	1	Ciamis	200-500	Jati	Tectona grandis L.f.	Lamiaceae / Verbenaceae	0,7	9,2	5	11	0,04	0,01	42,59	0,04	0,50	1,84
2809	55	1	Ciamis	200-500	Jati	Tectona grandis L.f.	Lamiaceae / Verbenaceae	0,7	21,3	12	18	0,04	0,04	310,05	0,31	3,64	13,37
2810	55	1	Ciamis	200-500	Jati	Tectona grandis L.f.	Lamiaceae / Verbenaceae	0,7	3,8	1,8	3,6	0,04	0,00	3,04	0,00	0,04	0,13
2811	55	1	Ciamis	200-500	Jati	Tectona grandis L.f.	Lamiaceae / Verbenaceae	0,7	18,5	12	18	0,04	0,03	238,04	0,24	2,80	10,27
2812	55	1	Ciamis	200-500	Jati	Tectona grandis L.f.	Lamiaceae / Verbenaceae	0,7	17,8	8	18	0,04	0,02	223,22	0,22	2,62	9,63
2813	55	1	Ciamis	200-500	Jati	Tectona grandis L.f.	Lamiaceae / Verbenaceae	0,7	20,4	9	18	0,04	0,03	285,09	0,29	3,35	12,29
2814	55	1	Ciamis	200-500	Jati	Tectona grandis L.f.	Lamiaceae / Verbenaceae	0,7	12,1	4	13	0,04	0,01	81,43	0,08	0,96	3,51
2815	55	1	Ciamis	200-500	Jati	Tectona grandis L.f.	Lamiaceae / Verbenaceae	0,7	19,1	7	18	0,04	0,03	253,30	0,25	2,98	10,92
2816	55	1	Ciamis	200-500	Jati	Tectona grandis L.f.	Lamiaceae / Verbenaceae	0,7	8,9	5	10	0,04	0,01	36,60	0,04	0,43	1,58
2817	55	1	Ciamis	200-500	Jati	Tectona grandis L.f.	Lamiaceae / Verbenaceae	0,7	17,5	8	18	0,04	0,02	215,97	0,22	2,54	9,31
2818	55	1	Ciamis	200-500	Jati	Tectona grandis L.f.	Lamiaceae / Verbenaceae	0,7	21,0	8	18	0,04	0,03	301,62	0,30	3,54	13,01
2819	55	1	Ciamis	200-500	Jati	Tectona grandis L.f.	Lamiaceae / Verbenaceae	0,7	21,6	8	18	0,04	0,04	318,58	0,32	3,74	13,74
2820	55	1	Ciamis	200-500	Jati	Tectona grandis L.f.	Lamiaceae / Verbenaceae	0,7	11,5	3	12	0,04	0,01	68,53	0,07	0,81	2,96
2821	55	1	Ciamis	200-500	Jati	Tectona grandis L.f.	Lamiaceae / Verbenaceae	0,7	10,8	3	11	0,04	0,01	56,99	0,06	0,67	2,46
2822	55	1	Ciamis	200-500	Jati	Tectona grandis L.f.	Lamiaceae / Verbenaceae	0,7	15,6	8	14	0,04	0,02	138,84	0,14	1,63	5,99
2823	55	1	Ciamis	200-500	Jati	Tectona grandis L.f.	Lamiaceae / Verbenaceae	0,7	7,3	3	9	0,04	0,00	23,17	0,02	0,27	1,00
2824	55	1	Ciamis	200-500	Jati	Tectona grandis L.f.	Lamiaceae / Verbenaceae	0,7	17,5	11	18	0,04	0,02	215,97	0,22	2,54	9,31
2825	55	1	Ciamis	200-500	Jati	Tectona grandis L.f.	Lamiaceae / Verbenaceae	0,7	7,6	2,5	8,5	0,04	0,00	23,78	0,02	0,28	1,03
2826	55	1	Ciamis	200-500	Jati	Tectona grandis L.f.	Lamiaceae / Verbenaceae	0,7	17,5	11	18	0,04	0,02	215,97	0,22	2,54	9,31
2827	55	1	Ciamis	200-500	Jati	Tectona grandis L.f.	Lamiaceae / Verbenaceae	0,7	7,6	2,5	8,5	0,04	0,00	23,78	0,02	0,28	1,03
2828	55	1	Ciamis	200-500	Jati	Tectona grandis L.f.	Lamiaceae / Verbenaceae	0,7	20,4	10	17	0,04	0,03	270,55	0,27	3,18	11,67
2829	55	1	Ciamis	200-500	Jati	Tectona grandis L.f.	Lamiaceae / Verbenaceae	0,7	22,6	9	17	0,04	0,04	327,21	0,33	3,84	14,11
2830	55	1	Ciamis	200-500	Jati	Tectona grandis L.f.	Lamiaceae / Verbenaceae	0,7	18,5	7	16	0,04	0,03	213,70	0,21	2,51	9,22
2831	55	1	Ciamis	200-500	Jati	Tectona grandis L.f.	Lamiaceae / Verbenaceae	0,7	21,6	7	16	0,04	0,04	286,00	0,29	3,36	12,33
2832	55	1	Ciamis	200-500	Jati	Tectona grandis L.f.	Lamiaceae / Verbenaceae	0,7	9,5	3,5	9,5	0,04	0,01	39,62	0,04	0,47	1,71
2833	55	1	Ciamis	200-500	Jati	Tectona grandis L.f.	Lamiaceae / Verbenaceae	0,7	16,5	6	16	0,04	0,02	174,95	0,17	2,06	7,54
2834	55	1	Ciamis	200-500	Jati	Tectona grandis L.f.	Lamiaceae / Verbenaceae	0,7	29,3	14	19	0,04	0,07	582,41	0,58	6,84	25,11
2835	55	1	Ciamis	200-500	Jati	Tectona grandis L.f.	Lamiaceae / Verbenaceae	0,7	12,1	8	18	0,04	0,01	109,70	0,11	1,29	4,73
2836	55	1	Ciamis	200-500	Jati	Tectona grandis L.f.	Lamiaceae / Verbenaceae	0,7	24,2	11	19	0,04	0,05	410,41	0,41	4,82	17,70
2837	55	1	Ciamis	200-500	Jati	Tectona grandis L.f.	Lamiaceae / Verbenaceae	0,7	7,6	2,5	6,5	0,04	0,00	18,60	0,02	0,22	0,80
2838	55	1	Ciamis	200-500	Jati	Tectona grandis L.f.	Lamiaceae / Verbenaceae	0,7	10,8	3,5	9,5	0,04	0,01	49,83	0,05	0,59	2,15
2839	55	1	Ciamis	200-500	Jati	Tectona grandis L.f.	Lamiaceae / Verbenaceae	0,7	12,7	6	13	0,04	0,01	89,45	0,09	1,05	3,86
2840	55	1	Ciamis	200-500	Mahoni	Swietenia macrophylla King	Meliaceae	0,61	15,9	6	12	0,04	0,02	110,29	0,11	1,30	4,76
2841	55	1	Ciamis	200-500	Jati	Tectona grandis L.f.	Lamiaceae / Verbenaceae	0,7	24,8	13	19	0,04	0,05	430,41	0,43	5,06	18,56
2842	55	1	Ciamis	200-500	Jati	Tectona grandis L.f.	Lamiaceae / Verbenaceae	0,7	11,8	3,5	13,5	0,04	0,01	80,27	0,08	0,94	3,46
2843	55	1	Ciamis	200-500	Jati	Tectona grandis L.f.	Lamiaceae / Verbenaceae	0,7	24,5	12	18	0,04	0,05	400,05	0,40	4,70	17,25
2844	55	1	Ciamis	200-500	Jati	Tectona grandis L.f.	Lamiaceae / Verbenaceae	0,7	17,2	7	16	0,04	0,02	187,48	0,19	2,20	8,08
2845	55	1	Ciamis	200-500	Jati	Tectona grandis L.f.	Lamiaceae / Verbenaceae	0,7	16,9	4	16	0,04	0,02	181,17	0,18	2,13	7,81
2846	55	1	Ciamis	200-500	Petai	Parkia speciosa Hassk.	Leguminosae / Fabaceae	0,45	17,2	5	16	0,04	0,02	125,08	0,13	1,47	5,39

2847	55	1	Ciamis	200-500	Jati	Tectona grandis L.f.	Lamiaceae / Verbenaceae	0,7	10,5	2,5	12	0,04	0,01	58,44	0,06	0,69	2,52
2848	55	1	Ciamis	200-500	Jati	Tectona grandis L.f.	Lamiaceae / Verbenaceae	0,7	2,2	14	20	0,04	0,00	5,17	0,01	0,06	0,22
2849	55	1	Ciamis	200-500	Jati	Tectona grandis L.f.	Lamiaceae / Verbenaceae	0,7	22,3	8	20	0,04	0,04	369,99	0,37	4,35	15,95
2850	55	1	Ciamis	200-500	Jati	Tectona grandis L.f.	Lamiaceae / Verbenaceae	0,7	8,0	2,5	6,5	0,04	0,00	20,04	0,02	0,24	0,86
2851	55	1	Ciamis	200-500	Jati	Tectona grandis L.f.	Lamiaceae / Verbenaceae	0,7	19,1	9	18	0,04	0,03	253,30	0,25	2,98	10,92
2852	55	1	Ciamis	200-500	Jati	Tectona grandis L.f.	Lamiaceae / Verbenaceae	0,7	26,1	14	19	0,04	0,05	471,71	0,47	5,54	20,34
2853	55	1	Ciamis	200-500	Jati	Tectona grandis L.f.	Lamiaceae / Verbenaceae	0,7	17,8	7	17	0,04	0,02	211,84	0,21	2,49	9,13
2854	55	1	Ciamis	200-500	Jati	Tectona grandis L.f.	Lamiaceae / Verbenaceae	0,7	22,3	11	17	0,04	0,04	318,82	0,32	3,75	13,75
2855	55	1	Ciamis	200-500	Jati	Tectona grandis L.f.	Lamiaceae / Verbenaceae	0,7	15,3	7	17	0,04	0,02	159,72	0,16	1,88	6,89
2856	55	1	Ciamis	200-500	Jati	Tectona grandis L.f.	Lamiaceae / Verbenaceae	0,7	28,6	12	18	0,04	0,06	532,39	0,53	6,26	22,96
2857	55	1	Ciamis	200-500	Jati	Tectona grandis L.f.	Lamiaceae / Verbenaceae	0,7	24,8	12	18	0,04	0,05	409,62	0,41	4,81	17,66
2858	55	1	Ciamis	200-500	Jati	Tectona grandis L.f.	Lamiaceae / Verbenaceae	0,7	17,2	10	18	0,04	0,02	208,84	0,21	2,45	9,01
2859	55	1	Ciamis	200-500	Jati	Tectona grandis L.f.	Lamiaceae / Verbenaceae	0,7	23,2	11	18	0,04	0,04	362,80	0,36	4,26	15,64
2860	55	1	Ciamis	200-500	Jati	Tectona grandis L.f.	Lamiaceae / Verbenaceae	0,7	19,4	11	16	0,04	0,03	234,38	0,23	2,75	10,11
2861	55	1	Ciamis	200-500	Jati	Tectona grandis L.f.	Lamiaceae / Verbenaceae	0,7	14,0	7	15	0,04	0,02	121,43	0,12	1,43	5,24
2862	55	1	Ciamis	200-500	Jati	Tectona grandis L.f.	Lamiaceae / Verbenaceae	0,7	27,7	13	19	0,04	0,06	525,73	0,53	6,18	22,67
2863	55	1	Ciamis	200-500	Jati	Tectona grandis L.f.	Lamiaceae / Verbenaceae	0,7	18,1	8	16	0,04	0,03	207,00	0,21	2,43	8,93
2864	55	1	Ciamis	200-500	Jati	Tectona grandis L.f.	Lamiaceae / Verbenaceae	0,7	9,5	3,5	9,5	0,04	0,01	39,62	0,04	0,47	1,71
2865	55	1	Ciamis	200-500	Jati	Tectona grandis L.f.	Lamiaceae / Verbenaceae	0,7	12,7	4	9,5	0,04	0,01	67,11	0,07	0,79	2,89
2866	55	1	Ciamis	200-500	Jati	Tectona grandis L.f.	Lamiaceae / Verbenaceae	0,7	14,0	10	16	0,04	0,02	128,83	0,13	1,51	5,56
2867	55	1	Ciamis	200-500	Jati	Tectona grandis L.f.	Lamiaceae / Verbenaceae	0,7	9,5	2,5	9,5	0,04	0,01	39,62	0,04	0,47	1,71
2868	55	1	Ciamis	200-500	Jati	Tectona grandis L.f.	Lamiaceae / Verbenaceae	0,7	21,0	10	18	0,04	0,03	301,62	0,30	3,54	13,01
2869	56	2	Ciamis	200-500	Kokosan	Lansium domesticum var. aquae	Meliaceae	0,7	21,0	6	11	0,04	0,03	192,11	0,19	2,26	8,28
2870	56	2	Ciamis	200-500	Sengon	Paraserianthes falcataria (L.) I.C	Leguminosae / Fabaceae	0,33	5,4	8	9	0,04	0,00	6,69	0,01	0,08	0,29
2871	56	2	Ciamis	200-500	Sengon	Paraserianthes falcataria (L.) I.C	Leguminosae / Fabaceae	0,33	14,6	8	9	0,04	0,02	41,43	0,04	0,49	1,79
2872	56	2	Ciamis	200-500	Tisuk	Hibiscus macrophyllus Roxb. ex	Malvaceae	0,4	5,4	4	5,5	0,04	0,00	5,08	0,01	0,06	0,22
2873	56	2	Ciamis	200-500	Sengon	Paraserianthes falcataria (L.) I.C	Leguminosae / Fabaceae	0,33	5,7	4	9	0,04	0,00	7,43	0,01	0,09	0,32
2874	56	2	Ciamis	200-500	Sengon	Paraserianthes falcataria (L.) I.C	Leguminosae / Fabaceae	0,33	17,8	10	12	0,04	0,02	77,32	0,08	0,91	3,33
2875	56	2	Ciamis	200-500	Sengon	Paraserianthes falcataria (L.) I.C	Leguminosae / Fabaceae	0,33	15,0	7	13	0,04	0,02	60,36	0,06	0,71	2,60
2876	56	2	Ciamis	200-500	Sengon	Paraserianthes falcataria (L.) I.C	Leguminosae / Fabaceae	0,33	15,3	3,5	4,5	0,04	0,02	23,74	0,02	0,28	1,02
2877	56	2	Ciamis	200-500	Sengon	Paraserianthes falcataria (L.) I.C	Leguminosae / Fabaceae	0,33	12,7	8	11	0,04	0,01	38,54	0,04	0,45	1,66
2878	56	2	Ciamis	200-500	Sengon	Paraserianthes falcataria (L.) I.C	Leguminosae / Fabaceae	0,33	7,3	7	8	0,04	0,00	10,45	0,01	0,12	0,45
2879	56	2	Ciamis	200-500	Mangga	Mangifera indica L.	Anacardiaceae	0,6	6,7	3,5	5	0,04	0,00	9,94	0,01	0,12	0,43
2880	56	2	Ciamis	200-500	Dukuh	Lansium domesticum Corrêa	Meliaceae	0,85	6,7	1	4	0,04	0,00	11,15	0,01	0,13	0,48
2881	56	2	Ciamis	200-500	Dukuh	Lansium domesticum Corrêa	Meliaceae	0,85	7,0	1,5	5	0,04	0,00	14,90	0,01	0,18	0,64
2882	56	2	Ciamis	200-500	Rambutan	Nephelium lappaceum L.	Sapindaceae	0,71	9,5	3,5	7,5	0,04	0,01	32,32	0,03	0,38	1,39
2883	56	2	Ciamis	200-500	Durian	Durio zibethinus L.	Malvaceae	0,57	25,1	6	1	0,04	0,05	24,60	0,02	0,29	1,06
2884	56	2	Ciamis	200-500	Durian	Durio zibethinus L.	Malvaceae	0,57	15,0	5	10	0,04	0,02	78,30	0,08	0,92	3,38
2885	56	2	Ciamis	200-500	Sengon	Paraserianthes falcataria (L.) I.C	Leguminosae / Fabaceae	0,33	19,1	11	13,5	0,04	0,03	97,73	0,10	1,15	4,21
2886	56	2	Ciamis	200-500	Sengon	Paraserianthes falcataria (L.) I.C	Leguminosae / Fabaceae	0,33	39,1	17	26	0,04	0,12	663,59	0,66	7,80	28,62
2887	56	2	Ciamis	200-500	Rambutan	Nephelium lappaceum L.	Sapindaceae	0,71	11,5	3	7	0,04	0,01	42,38	0,04	0,50	1,83
2888	56	2	Ciamis	200-500	Manggis	Mangostana garcinia Gaertn.	Clusiaceae	0,81	8,0	1,5	3	0,04	0,00	11,28	0,01	0,13	0,49
2889	56	2	Ciamis	200-500	Sengon	Paraserianthes falcataria (L.) I.C	Leguminosae / Fabaceae	0,33	12,7	12	18	0,04	0,01	60,52	0,06	0,71	2,61
2890	56	2	Ciamis	200-500	Sengon	Paraserianthes falcataria (L.) I.C	Leguminosae / Fabaceae	0,33	38,2	13	19	0,04	0,11	475,86	0,48	5,59	20,52
2891	56	2	Ciamis	200-500	Sengon	Paraserianthes falcataria (L.) I.C	Leguminosae / Fabaceae	0,33	15,0	8	14	0,04	0,02	64,60	0,06	0,76	2,79
2892	56	2	Ciamis	200-500	Sengon	Paraserianthes falcataria (L.) I.C	Leguminosae / Fabaceae	0,33	13,4	8	14	0,04	0,01	52,57	0,05	0,62	2,27
2893	56	2	Ciamis	200-500	Petai	Parkia speciosa Hassk.	Leguminosae / Fabaceae	0,45	27,0	15	22	0,04	0,06	384,43	0,38	4,52	16,58
2894	56	2	Ciamis	200-500	Rambutan	Nephelium lappaceum L.	Sapindaceae	0,71	7,6	3	8	0,04	0,00	22,79	0,02	0,27	0,98
2895	56	2	Ciamis	200-500	Kelapa	Cocos nucifera L.	Arecaceae	0,5	24,8	3	6	0,04	0,05	50,70	0,05	0,60	2,19
2896	56	2	Ciamis	200-500	Jengkol	Archidendron pauciflorum (Bentl	Leguminosae / Fabaceae	0,4	7,3	7	8	0,04	0,00	12,46	0,01	0,15	0,54
2897	56	2	Ciamis	200-500	Kokosan	Lansium domesticum var. aquae	Meliaceae	0,7	6,4	3,5	6,5	0,04	0,00	13,32	0,01	0,16	0,57
2898	56	2	Ciamis	200-500	Kokosan	Lansium domesticum var. aquae	Meliaceae	0,7	4,5	3,5	5,5	0,04	0,00	5,94	0,01	0,07	0,26
2899	56	2	Ciamis	200-500	Nangka	Artocarpus heterophyllus Lam.	Moraceae	0,61	10,2	4	6,5	0,04	0,01	27,77	0,03	0,33	1,20
2900	56	2	Ciamis	200-500	Kokosan	Lansium domesticum var. aquae	Meliaceae	0,7	6,4	4	7	0,04	0,00	14,25	0,01	0,17	0,61
2901	56	2	Ciamis	200-500	Jengkol	Archidendron pauciflorum (Bentl	Leguminosae / Fabaceae	0,4	8,9	4	8	0,04	0,01	17,87	0,02	0,21	0,77
2902	57	2	Ciamis	200-500	Mahoni	Swietenia macrophylla King	Meliaceae	0,61	16,9	7	12	0,04	0,02	122,71	0,12	1,44	5,29
2903	57	2	Ciamis	200-500	Mahoni	Swietenia macrophylla King	Meliaceae	0,61	9,9	5	8	0,04	0,01	31,69	0,03	0,37	1,37
2904	57	2	Ciamis	200-500	Kopo	Syzygium pycnanthum Merr. & L.	Myrtaceae	0,59	4,1	2	3,5	0,04	0,00	2,93	0,00	0,03	0,13
2905	57	2	Ciamis	200-500	Mahoni	Swietenia macrophylla King	Meliaceae	0,61	11,8	5	9	0,04	0,01	48,81	0,05	0,57	2,10
2906	57	2	Ciamis	200-500	Mahoni	Swietenia macrophylla King	Meliaceae	0,61	11,8	4	6	0,04	0,01	33,67	0,03	0,40	1,45
2907	57	2	Ciamis	200-500	Jati	Tectona grandis L.f.	Lamiaceae / Verbenaceae	0,7	3,2	1	2	0,04	0,00	1,27	0,00	0,01	0,05
2908	57	2	Ciamis	200-500	Sengon	Paraserianthes falcataria (L.) I.C	Leguminosae / Fabaceae	0,33	25,5	20	23	0,04	0,05	269,70	0,27	3,17	11,63
2909	57	2	Ciamis	200-500	Mahoni	Swietenia macrophylla King	Meliaceae	0,61	12,1	3,5	8,5	0,04	0,01	48,64	0,05	0,57	2,10
2910	57	2	Ciamis	200-500	Mahoni	Swietenia macrophylla King	Meliaceae	0,61	16,9	6	10	0,04	0,02	103,84	0,10	1,22	4,48
2911	57	2	Ciamis	200-500	Mahoni	Swietenia macrophylla King	Meliaceae	0,61	5,4	3	1,5	0,04	0,00	2,27	0,00	0,03	0,10
2912	57	2	Ciamis	200-500	Mahoni	Swietenia macrophylla King	Meliaceae	0,61	12,1	6	11	0,04	0,01	61,60	0,06	0,72	2,66
2913	57	2	Ciamis	200-500	Mahoni	Swietenia macrophylla King	Meliaceae	0,61	7,0	6	11	0,04	0,00	22,63	0,02	0,27	0,98
2914	57	2	Ciamis	200-500	Mahoni	Swietenia macrophylla King	Meliaceae	0,61	6,7	3,5	7,5	0,04	0,00	14,63	0,01	0,17	0,63
2915	57	2	Ciamis	200-500	Mahoni	Swietenia macrophylla King	Meliaceae	0,61	16,2	8	12	0,04	0,02	114,36	0,11	1,34	4,93
2916	57	2	Ciamis	200-500	Mahoni	Swietenia macrophylla King	Meliaceae	0,61	7,0	4	7	0,04	0,00	14,96	0,01	0,18	0,65
2917	57	2	Ciamis	200-500	Mahoni	Swietenia macrophylla King	Meliaceae	0,61	11,5	7	11	0,04	0,01	55,79	0,06	0,66	2,41
2918	57	2	Ciamis	200-500	Mahoni	Swietenia macrophylla King	Meliaceae	0,61	8,3	4	8	0,04	0,01	22,96	0,02	0,27	0,99
2919	57	2	Ciamis	200-500	Mahoni	Swietenia macrophylla King	Meliaceae	0,61	9,5	4	7	0,04	0,01	26,40	0,03	0,31	1,14
2920	57	2	Ciamis	200-500	Mahoni	Swietenia macrophylla King	Meliaceae	0,61	7,0	2,5	5,5	0,04	0,00	11,99	0,01	0,14	0,52
2921	57	2	Ciamis	200-500	Mahoni	Swietenia macrophylla King	Meliaceae	0,61	7,6	3	6	0,04	0,00	15,23	0,02	0,18	0,66
2922	57	2	Ciamis	200-500	Mahoni	Swietenia macrophylla King	Meliaceae	0,61	8,6	2	6	0,04	0,01	18,90	0,02	0,22	0,82
2923	57	2	Ciamis	200-500	Kelapa	Cocos nucifera L.	Arecaceae	0,5	21,6	8	11	0,04	0,04	89,20	0,09	1,05	3,85
2924	57	2	Ciamis	200-500	Sengon	Paraserianthes falcataria (L.) I.C	Leguminosae / Fabaceae	0,33	18,1	9	4	0,04	0,03	29,20	0,03	0,34	1,26
2925	57	2	Ciamis	200-500	Mahoni	Swietenia macrophylla King	Meliaceae	0,61	17,8	4	12	0,04	0,02	135,74	0,14	1,59	5,85
2926	57	2	Ciamis	200-500	Mahoni	Swietenia macrophylla King	Meliaceae	0,61	12,4	6	10	0,04	0,01	59,20	0,06	0,70	2,55
2927	57	2	Ciamis	200-500	Mahoni	Swietenia macrophylla King	Meliaceae	0,61	9,5	3	7	0,04	0,01	26,40	0,03	0,31	1,14
2928	57	2	Ciamis	200-500	Sengon	Paraserianthes falcataria (L.) I.C	Leguminosae / Fabaceae	0,33	15,6	11	15	0,04	0,02	74,27	0,07	0,87	3,20
2929	57	2	Ciamis	200-500	Mahoni	Swietenia macrophylla King	Meliaceae	0,61	7,3	2,5	5,5	0,04	0,00	13,01	0,01	0,15	0,56
2930	57	2	Ciamis	200-500	Mahoni	Swietenia macrophylla King	Meliaceae	0,61	6,4	3	6	0,04	0,00	10,91	0,01	0,13	0,47
2931	57	2	Ciamis	200-500	Mahoni	Swietenia macrophylla King	Meliaceae	0,61	7,6	3,5	2	0,04	0,00	5,57	0,01	0,07	0,24
2932	57	2	Ciamis	200-500	Mahoni	Swietenia macrophylla King	Meliaceae	0,61	8,0	3,5	7,5	0,04	0,00	20,14	0,02	0,24	0,87
2933	57	2	Ciamis	200-500	Mahoni	Swietenia macrophylla King	Meliaceae	0,61	13,7	3,5	9	0,04	0,01	64,28	0,06	0,76	2,77
2934	57	2	Ciamis	200-500	Rambutan	Nephelium lappaceum L.	Sapindaceae	0,71	15,0	5	13	0,04	0,02	121,77	0,12	1,43	5,25
2935	57	2	Ciamis	200-500	Mahoni	Swietenia macrophylla King	Meliaceae	0,61	12,1	6	11	0,04	0,01	61,60	0,06	0,72	2,66
2936	57	2	Ciamis	200-500	Mahoni	Swietenia macrophylla King	Meliaceae	0,61	12,7	6	10	0,04	0,01	62,01	0,06	0,73	2,67
2937	57	2	Ciamis	200-500	Sengon	Paraserianthes falcataria (L.) I.C	Leguminosae / Fabaceae	0,33	20,0	15	19	0,04	0,03	146,15	0,15	1,72	6,30
2938	57	2	Ciamis	200-500	Mahoni	Swietenia macrophylla King	Meliaceae	0,61	5,1	6	12	0,04	0,00	13,68	0,01	0,16	0,59
2939	57	2	Ciamis	200-500	Bungbulang	Premna tomentosa Willd.	Lamiaceae / Verbenaceae	0,96	8,9	7	10	0,04	0,01	48,87	0,05	0,57	2,11
2940	57	2	Ciamis	200-500	Mahoni	Swietenia macrophylla King	Meliaceae	0,61	8,9	7	11	0,04	0,01	35,20	0,04	0,41	1,52
2941	57	2	Ciamis	200-500	Mahoni	Swietenia macrophylla King	Meliaceae	0,61	7,0	2	6	0,04	0,00	12,99	0,01	0,15	0,56
2942	57	2	Ciamis	200-500	Mahoni	Swietenia macrophylla King	Meliaceae	0,61	7,3	2	7	0,04	0,00	16,23	0,02	0,19	0,70
2943	57	2	Ciamis	200-500	Mahoni	Swietenia macrophylla King	Meliaceae	0,61	11,1	3	10	0,04	0,01	48,55	0,05	0,57	2,09
2944	57	2	Ciamis	200-500	Kelapa	Cocos nucifera L.	Arecaceae	0,5	24,2	7	10	0,04	0,05	81,50	0,08	0,96	3,51
2945	57	2	Ciamis	200-500	Mahoni	Swietenia macrophylla King	Meliaceae	0,61	10,5	7	12	0,04	0,01	51,51	0,05	0,61	2,22
2946	57	2	Ciamis	200-500	Mahoni	Swietenia macrophylla King	Meliaceae	0,61	8,3	3	11	0,04	0,01	30,73	0,03	0,36	1,33
2947	57	2	Ciamis	200-500	Mahoni	Swietenia macrophylla King	Meliaceae	0,61	10,2	3,5	8,5	0,04	0,01	35,50	0,04	0,42	1,53
2948	57	2	Ciamis	200-500	Mahoni	Swietenia macrophylla King	Meliaceae	0,61	12,4	3,5	10	0,04	0,01	59,20	0,06	0,70	2,55
2949	57	2	Ciamis	200-500	Mahoni	Swietenia macrophylla King	Meliaceae	0,61	9,2	5	10	0,04	0,01	34,40	0,03	0,40	1,48
2950	57	2	Ciamis	200-500	Mahoni	Swietenia macrophylla King	Meliaceae	0,61	8,0	5	8	0,04	0,00	21,37	0,02	0,25	0,92
2951	57	2	Ciamis	200-500	Mahoni	Swietenia macrophylla King	Meliaceae	0,61	5,7	3	7	0,04	0,00	10,36	0,01	0,12	0,45
2952	57	2	Ciamis	200-500	Nangka	Artocarpus heterophyllus Lam.	Moraceae	0,61	12,1	6	10	0,04	0,01	56,45	0,06	0,66	2,43
2953	57	2	Ciamis	200-500	Kopo	Syzygium pycnanthum Merr. & L.	Myrtaceae	0,59	15,0	8	12	0,04	0,02	95,51	0,10	1,12	4,12
2954	57	2	Ciamis	200-500	Mahoni	Swietenia macrophylla King	Meliaceae	0,61	21,3	9	12	0,04	0,04	188,53	0,19	2,22	8,13
2955	57	2	Ciamis	200-500	Mahoni	Swietenia macrophylla King	Meliaceae	0,61	14,0	2	9	0,04	0,02	67,05	0,07	0,79	2,89
2956	57	2	Ciamis	200-500	Mahoni	Swietenia macrophylla King	Meliaceae	0,61	17,8	7	12	0,04	0,02	135,74	0,14	1,59	5,85
2957	57	2	Ciamis	200-500	Mahoni	Swietenia macrophylla King	Meliaceae	0,61	13,4	3	10	0,04	0,01	67,81	0,07	0,80	2,92
2958	58	2	Ciamis	200-500	Petai	Parkia speciosa Hassk.	Leguminosae / Fabaceae	0,45	24,2	12	17	0,04	0,05	247,29	0,25	2,91	10,66
2959	58	2	Ciamis	200-500	Kokosan	Lansium domesticum var. aquae	Meliaceae	0,7	22,6	7	13	0,04	0,04	255,92	0,26	3,01	11,04
2960	58	2	Ciamis	200-500	Dukuh	Lansium domesticum Corrêa	Meliaceae	0,85	16,2	5	6	0,04	0,02	82,13	0,08	0,97	3,54
2961	58	2	Ciamis	200-500	Kokosan	Lansium domesticum var. aquae	Meliaceae	0,7	22,0	8	14	0,04	0,04	259,93	0,26	3,05	11,21
2962	58	2	Ciamis	200-500	Tisuk	Hibiscus macrophyllus Roxb. ex	Malvaceae	0,4	9,5	9	12	0,04	0,01	29,39	0,03	0,35	1,27
2963	58	2	Ciamis	200-500	Tisuk	Hibiscus macrophyllus Roxb. ex	Malvaceae	0,4	15,0	15	20	0,04	0,02	106,82	0,11	1,26	4,61
2964	58	2	Ciamis	200-500	Petai	Parkia speciosa Hassk.	Leguminosae / Fabaceae	0,45	23,9	14	22	0,04	0,04	305,66	0,31	3,59	13,18
2965	58	2	Ciamis	200-500	Petai	Parkia speciosa Hassk.	Leguminosae / Fabaceae	0,45	33,4	5	14	0,04	0,09	374,23	0,37	4,40	16,14
2966	58	2	Ciamis	200-500	Tisuk	Hibiscus macrophyllus Roxb. ex	Malvaceae	0,4	5,1	4	6	0,04	0,00	4,92	0,00	0,06	0,21
2967	58	2	Ciamis	200-500	Rambutan	Nephelium lappaceum L.	Sapindaceae	0,71	15,0	10	13	0,04	0,02	121,77	0,12	1,43	5,25
2968	58	2	Ciamis	200-500	Aren	Arenga pinnata (Wurmb) Merr.	Arecaceae	0,71	41,4	9	14	0,04	0,13	112,30	0,11	1,32	4,84
2969	58	2	Ciamis	200-500	Rambutan	Nephelium lappaceum L.	Sapindaceae	0,71	14,0	6	13	0,04	0,02	107,91	0,11	1,27	4,65
2970	58	2	Ciamis	200-500	Dukuh	Lansium domesticum Corrêa	Meliaceae	0,85	4,1	3	7	0,04	0,00	7,73	0,01	0,09	0,33

2971	58	2	Ciamis	200-500	Dukuh	Lansium domesticum Corrêa	Meliaceae	0,85	4,5	2,5	5,5	0,04	0,00	7,10	0,01	0,08	0,31
2972	58	2	Ciamis	200-500	Tisuk	Hibiscus macrophyllus Roxb. ex I	Malvaceae	0,4	9,5	9	14	0,04	0,01	33,85	0,03	0,40	1,46
2973	58	2	Ciamis	200-500	Rambutan	Nephelium lappaceum L.	Sapindaceae	0,71	8,0	6	10	0,04	0,00	30,12	0,03	0,35	1,30
2974	58	2	Ciamis	200-500	Tisuk	Hibiscus macrophyllus Roxb. ex I	Malvaceae	0,4	8,9	9	13	0,04	0,01	27,87	0,03	0,33	1,20
2975	58	2	Ciamis	200-500	Nangka	Artocarpus heterophyllus Lam.	Moraceae	0,61	4,5	3	5	0,04	0,00	4,80	0,00	0,06	0,21
2976	58	2	Ciamis	200-500	Nangka	Artocarpus heterophyllus Lam.	Moraceae	0,61	6,4	3,5	5,5	0,04	0,00	10,07	0,01	0,12	0,43
2977	58	2	Ciamis	200-500	Mahoni	Swietenia macrophylla King	Meliaceae	0,61	6,7	3,5	6,5	0,04	0,00	12,84	0,01	0,15	0,55
2978	58	2	Ciamis	200-500	Mahoni	Swietenia macrophylla King	Meliaceae	0,61	7,0	3	7	0,04	0,00	14,96	0,01	0,18	0,65
2979	58	2	Ciamis	200-500	Mahoni	Swietenia macrophylla King	Meliaceae	0,61	4,8	3,5	6,5	0,04	0,00	6,93	0,01	0,08	0,30
2980	58	2	Ciamis	200-500	Sengon	Paraserianthes falcataria (L.) I.C.	Leguminosae / Fabaceae	0,33	18,5	13	30	0,04	0,03	190,86	0,19	2,24	8,23
2981	58	2	Ciamis	200-500	Tisuk	Hibiscus macrophyllus Roxb. ex I	Malvaceae	0,4	9,9	12	16	0,04	0,01	40,62	0,04	0,48	1,75
2982	58	2	Ciamis	200-500	Tisuk	Hibiscus macrophyllus Roxb. ex I	Malvaceae	0,4	11,1	10	13	0,04	0,01	41,95	0,04	0,49	1,81
2983	58	2	Ciamis	200-500	Tisuk	Hibiscus macrophyllus Roxb. ex I	Malvaceae	0,4	10,2	8	15	0,04	0,01	40,58	0,04	0,48	1,75
2984	58	2	Ciamis	200-500	Tisuk	Hibiscus macrophyllus Roxb. ex I	Malvaceae	0,4	6,7	8	12	0,04	0,00	15,29	0,02	0,18	0,66
2985	58	2	Ciamis	200-500	Tisuk	Hibiscus macrophyllus Roxb. ex I	Malvaceae	0,4	6,4	9	12	0,04	0,00	13,98	0,01	0,16	0,60
2986	58	2	Ciamis	200-500	Mahoni	Swietenia macrophylla King	Meliaceae	0,61	5,7	4	6	0,04	0,00	8,99	0,01	0,11	0,39
2987	58	2	Ciamis	200-500	Tisuk	Hibiscus macrophyllus Roxb. ex I	Malvaceae	0,4	4,5	6	13,5	0,04	0,00	8,10	0,01	0,10	0,35
2988	58	2	Ciamis	200-500	Tisuk	Hibiscus macrophyllus Roxb. ex I	Malvaceae	0,4	7,3	3,5	9	0,04	0,00	13,88	0,01	0,16	0,60
2989	58	2	Ciamis	200-500	Sengon	Paraserianthes falcataria (L.) I.C.	Leguminosae / Fabaceae	0,33	12,4	12	17	0,04	0,01	54,83	0,05	0,64	2,36
2990	58	2	Ciamis	200-500	Manglid	Magnolia champaca (L.) Baill. ex	Magnoliaceae	0,53	6,4	4	9	0,04	0,00	13,90	0,01	0,16	0,60
2991	58	2	Ciamis	200-500	Dukuh	Lansium domesticum Corrêa	Meliaceae	0,85	63,3	4	14	0,04	0,32	2.161,82	2,16	25,40	93,22
2992	58	2	Ciamis	200-500	Kelapa	Cocos nucifera L.	Arecaceae	0,5	33,4	10	13	0,04	0,09	104,60	0,10	1,23	4,51
2993	58	2	Ciamis	200-500	Kelapa	Cocos nucifera L.	Arecaceae	0,5	26,4	3	5	0,04	0,05	43,00	0,04	0,51	1,85
2994	58	2	Ciamis	200-500	Rambutan	Nephelium lappaceum L.	Sapindaceae	0,71	22,3	7	17	0,04	0,04	322,99	0,32	3,80	13,93
2995	59	2	Ciamis	200-500	Mahoni	Swietenia macrophylla King	Meliaceae	0,61	6,4	4	6	0,04	0,00	10,91	0,01	0,13	0,47
2996	59	2	Ciamis	200-500	Mahoni	Swietenia macrophylla King	Meliaceae	0,61	3,8	3	5	0,04	0,00	3,62	0,00	0,04	0,16
2997	59	2	Ciamis	200-500	Mahoni	Swietenia macrophylla King	Meliaceae	0,61	3,8	2	3,5	0,04	0,00	2,61	0,00	0,03	0,11
2998	59	2	Ciamis	200-500	Melinjo	Gnetum gnemon L.	Gnetaceae	0,76	4,8	1	5	0,04	0,00	6,67	0,01	0,08	0,29
2999	59	2	Ciamis	200-500	Mahoni	Swietenia macrophylla King	Meliaceae	0,61	3,8	3,5	5	0,04	0,00	3,62	0,00	0,04	0,16
3000	59	2	Ciamis	200-500	Mahoni	Swietenia macrophylla King	Meliaceae	0,61	4,5	4	6	0,04	0,00	5,68	0,01	0,07	0,24
3001	59	2	Ciamis	200-500	Mahoni	Swietenia macrophylla King	Meliaceae	0,61	3,8	2	5	0,04	0,00	3,62	0,00	0,04	0,16
3002	59	2	Ciamis	200-500	Mahoni	Swietenia macrophylla King	Meliaceae	0,61	7,6	3	7	0,04	0,00	17,54	0,02	0,21	0,76
3003	59	2	Ciamis	200-500	Melinjo	Gnetum gnemon L.	Gnetaceae	0,76	1,6	0,5	2,5	0,04	0,00	0,47	0,00	0,01	0,02
3004	59	2	Ciamis	200-500	Mahoni	Swietenia macrophylla King	Meliaceae	0,61	6,4	6	9	0,04	0,00	15,81	0,02	0,19	0,68
3005	59	2	Ciamis	200-500	Mahoni	Swietenia macrophylla King	Meliaceae	0,61	5,1	4	7	0,04	0,00	8,35	0,01	0,10	0,36
3006	59	2	Ciamis	200-500	Mahoni	Swietenia macrophylla King	Meliaceae	0,61	3,8	3	6	0,04	0,00	4,28	0,00	0,05	0,18
3007	59	2	Ciamis	200-500	Mahoni	Swietenia macrophylla King	Meliaceae	0,61	4,8	4	11	0,04	0,00	11,22	0,01	0,13	0,48
3008	59	2	Ciamis	200-500	Mahoni	Swietenia macrophylla King	Meliaceae	0,61	6,7	5	9	0,04	0,00	17,29	0,02	0,20	0,75
3009	59	2	Ciamis	200-500	Mahoni	Swietenia macrophylla King	Meliaceae	0,61	3,8	2	5	0,04	0,00	3,62	0,00	0,04	0,16
3010	59	2	Ciamis	200-500	Mahoni	Swietenia macrophylla King	Meliaceae	0,61	6,4	3,5	7,5	0,04	0,00	13,38	0,01	0,16	0,58
3011	59	2	Ciamis	200-500	Mahoni	Swietenia macrophylla King	Meliaceae	0,61	4,1	4	7	0,04	0,00	5,71	0,01	0,07	0,25
3012	59	2	Ciamis	200-500	Mahoni	Swietenia macrophylla King	Meliaceae	0,61	3,8	3	7	0,04	0,00	4,93	0,00	0,06	0,21
3013	59	2	Ciamis	200-500	Mahoni	Swietenia macrophylla King	Meliaceae	0,61	5,1	5	8	0,04	0,00	9,43	0,01	0,11	0,41
3014	59	2	Ciamis	200-500	Rambutan	Nephelium lappaceum L.	Sapindaceae	0,71	4,8	4	7	0,04	0,00	8,52	0,01	0,10	0,37
3015	59	2	Ciamis	200-500	Mahoni	Swietenia macrophylla King	Meliaceae	0,61	7,3	4	10	0,04	0,00	22,50	0,02	0,26	0,97
3016	59	2	Ciamis	200-500	Mahoni	Swietenia macrophylla King	Meliaceae	0,61	5,7	2,5	5	0,04	0,00	7,61	0,01	0,09	0,33
3017	59	2	Ciamis	200-500	Mahoni	Swietenia macrophylla King	Meliaceae	0,61	2,2	2	3	0,04	0,00	0,84	0,00	0,01	0,04
3018	59	2	Ciamis	200-500	Mahoni	Swietenia macrophylla King	Meliaceae	0,61	3,2	3	4	0,04	0,00	2,11	0,00	0,02	0,09
3019	59	2	Ciamis	200-500	Kelapa	Cocos nucifera L.	Arecaceae	0,5	27,0	9	12	0,04	0,06	96,90	0,10	1,14	4,18
3020	59	2	Ciamis	200-500	Mahoni	Swietenia macrophylla King	Meliaceae	0,61	3,2	3	5	0,04	0,00	2,59	0,00	0,03	0,11
3021	59	2	Ciamis	200-500	Mahoni	Swietenia macrophylla King	Meliaceae	0,61	6,0	4	8	0,04	0,00	12,92	0,01	0,15	0,56
3022	59	2	Ciamis	200-500	Jati	Tectona grandis L.f.	Lamiaceae / Verbenaceae	0,7	13,4	6	11	0,04	0,01	83,93	0,08	0,99	3,62
3023	59	2	Ciamis	200-500	Melinjo	Gnetum gnemon L.	Gnetaceae	0,76	6,4	0,5	5	0,04	0,00	11,29	0,01	0,13	0,49
3024	59	2	Ciamis	200-500	Jati	Tectona grandis L.f.	Lamiaceae / Verbenaceae	0,7	4,5	3	5	0,04	0,00	5,45	0,01	0,06	0,23
3025	59	2	Ciamis	200-500	Mahoni	Swietenia macrophylla King	Meliaceae	0,61	2,2	2,5	45	0,04	0,00	10,09	0,01	0,12	0,44
3026	59	2	Ciamis	200-500	Matoa	Pometia pinnata J.R.Forst. & G.Fo	Sapindaceae	0,77	18,5	5	13	0,04	0,03	192,80	0,19	2,27	8,31
3027	59	2	Ciamis	200-500	Lamtoro	Leucaena leucocephala (Lam.) d	Leguminosae / Fabaceae	0,64	6,4	5	9	0,04	0,00	16,53	0,02	0,19	0,71
3028	59	2	Ciamis	200-500	Jati	Tectona grandis L.f.	Lamiaceae / Verbenaceae	0,7	3,5	2	5	0,04	0,00	3,50	0,00	0,04	0,15
3029	59	2	Ciamis	200-500	Melinjo	Gnetum gnemon L.	Gnetaceae	0,76	4,1	0,5	4,5	0,04	0,00	4,66	0,00	0,05	0,20
3030	59	2	Ciamis	200-500	Mahoni	Swietenia macrophylla King	Meliaceae	0,61	6,7	5	9	0,04	0,00	17,29	0,02	0,20	0,75
3031	59	2	Ciamis	200-500	Jati	Tectona grandis L.f.	Lamiaceae / Verbenaceae	0,7	7,0	2	6	0,04	0,00	14,73	0,01	0,17	0,64
3032	59	2	Ciamis	200-500	Mahoni	Swietenia macrophylla King	Meliaceae	0,61	5,1	3	7	0,04	0,00	8,35	0,01	0,10	0,36
3033	59	2	Ciamis	200-500	Mahoni	Swietenia macrophylla King	Meliaceae	0,61	5,7	4	8	0,04	0,00	11,70	0,01	0,14	0,50
3034	59	2	Ciamis	200-500	Jati	Tectona grandis L.f.	Lamiaceae / Verbenaceae	0,7	11,5	4	10	0,04	0,01	57,99	0,06	0,68	2,50
3035	59	2	Ciamis	200-500	Kecapi	Sandoricum koetjape (Burm.f.) M	Meliaceae	0,44	17,2	5	9	0,04	0,02	72,34	0,07	0,85	3,12
3036	59	2	Ciamis	200-500	Kecapi	Sandoricum koetjape (Burm.f.) M	Meliaceae	0,44	3,2	2	3,5	0,04	0,00	1,39	0,00	0,02	0,06
3037	59	2	Ciamis	200-500	Mahoni	Swietenia macrophylla King	Meliaceae	0,61	5,7	3	6	0,04	0,00	8,99	0,01	0,11	0,39
3038	59	2	Ciamis	200-500	Kecapi	Sandoricum koetjape (Burm.f.) M	Meliaceae	0,44	5,4	3	6	0,04	0,00	6,00	0,01	0,07	0,26
3039	59	2	Ciamis	200-500	Kecapi	Sandoricum koetjape (Burm.f.) M	Meliaceae	0,44	8,6	6	9	0,04	0,01	20,32	0,02	0,24	0,88
3040	59	2	Ciamis	200-500	Kecapi	Sandoricum koetjape (Burm.f.) M	Meliaceae	0,44	5,4	4	7	0,04	0,00	6,92	0,01	0,08	0,30
3041	59	2	Ciamis	200-500	Kecapi	Sandoricum koetjape (Burm.f.) M	Meliaceae	0,44	3,5	3,5	5,5	0,04	0,00	2,50	0,00	0,03	0,11
3042	59	2	Ciamis	200-500	Mahoni	Swietenia macrophylla King	Meliaceae	0,61	7,6	3,5	8,5	0,04	0,00	20,96	0,02	0,25	0,90
3043	59	2	Ciamis	200-500	Karet	Hevea brasiliensis (Willd. ex A.Ju	Euphorbiaceae	0,53	3,8	5	6,5	0,04	0,00	4,05	0,00	0,05	0,17
3044	59	2	Ciamis	200-500	Mahoni	Swietenia macrophylla King	Meliaceae	0,61	9,9	4	9	0,04	0,01	35,30	0,04	0,41	1,52
3045	59	2	Ciamis	200-500	Mahoni	Swietenia macrophylla King	Meliaceae	0,61	6,7	3	2,5	0,04	0,00	5,35	0,01	0,06	0,23
3046	59	2	Ciamis	200-500	Saga	Adenanthera pavonina L.	Leguminosae / Fabaceae	0,7	21,3	9	15	0,04	0,04	262,36	0,26	3,08	11,31
3047	59	2	Ciamis	200-500	Mahoni	Swietenia macrophylla King	Meliaceae	0,61	9,9	7	12	0,04	0,01	45,94	0,05	0,54	1,98
3048	59	2	Ciamis	200-500	Mahoni	Swietenia macrophylla King	Meliaceae	0,61	8,6	5	8	0,04	0,01	24,60	0,02	0,29	1,06
3049	59	2	Ciamis	200-500	Rambutan	Nephelium lappaceum L.	Sapindaceae	0,71	5,7	6	10	0,04	0,00	16,50	0,02	0,19	0,71
3050	59	2	Ciamis	200-500	Nangka	Artocarpus heterophyllus Lam.	Moraceae	0,61	5,7	6	8	0,04	0,00	11,70	0,01	0,14	0,50
3051	59	2	Ciamis	200-500	Rambutan	Nephelium lappaceum L.	Sapindaceae	0,71	5,1	5	10	0,04	0,00	13,30	0,01	0,16	0,57
3052	59	2	Ciamis	200-500	Saga	Adenanthera pavonina L.	Leguminosae / Fabaceae	0,7	12,1	7	13	0,04	0,01	81,43	0,08	0,96	3,51
3053	59	2	Ciamis	200-500	Sengon	Paraserianthes falcataria (L.) I.C.	Leguminosae / Fabaceae	0,33	5,4	8	10,5	0,04	0,00	7,70	0,01	0,09	0,33
3054	59	2	Ciamis	200-500	Mahoni	Swietenia macrophylla King	Meliaceae	0,61	5,7	3	7	0,04	0,00	10,36	0,01	0,12	0,45
3055	59	2	Ciamis	200-500	Sengon	Paraserianthes falcataria (L.) I.C.	Leguminosae / Fabaceae	0,33	8,6	9	13	0,04	0,01	21,86	0,02	0,26	0,94
3056	59	2	Ciamis	200-500	Saga	Adenanthera pavonina L.	Leguminosae / Fabaceae	0,7	11,5	9	15	0,04	0,01	84,08	0,08	0,99	3,63
3057	59	2	Ciamis	200-500	Mahoni	Swietenia macrophylla King	Meliaceae	0,61	6,7	5	25	0,04	0,00	44,09	0,04	0,52	1,90
3058	59	2	Ciamis	200-500	Saga	Adenanthera pavonina L.	Leguminosae / Fabaceae	0,7	17,5	8	14	0,04	0,02	171,56	0,17	2,02	7,40
3059	59	2	Ciamis	200-500	Nangka	Artocarpus heterophyllus Lam.	Moraceae	0,61	6,4	3	7	0,04	0,00	12,56	0,01	0,15	0,54
3060	59	2	Ciamis	200-500	Afrika	Maesopsis eminii Engl.	Rhamnaceae	0,43	15,3	14	16	0,04	0,02	96,69	0,10	1,14	4,17
3061	59	2	Ciamis	200-500	Melinjo	Gnetum gnemon L.	Gnetaceae	0,76	10,2	1	4	0,04	0,01	21,77	0,02	0,26	0,94
3062	59	2	Ciamis	200-500	Jati	Tectona grandis L.f.	Lamiaceae / Verbenaceae	0,7	10,8	5	11	0,04	0,01	56,99	0,06	0,67	2,46
3063	59	2	Ciamis	200-500	Mahoni	Swietenia macrophylla King	Meliaceae	0,61	8,3	6	8,5	0,04	0,01	24,27	0,02	0,29	1,05
3064	59	2	Ciamis	200-500	Mahoni	Swietenia macrophylla King	Meliaceae	0,61	4,1	3	6	0,04	0,00	4,95	0,00	0,06	0,21
3065	59	2	Ciamis	200-500	Petai	Parkia speciosa Hassk.	Leguminosae / Fabaceae	0,45	4,1	5	6	0,04	0,00	3,75	0,00	0,04	0,16
3066	59	2	Ciamis	200-500	Mahoni	Swietenia macrophylla King	Meliaceae	0,61	3,5	2,5	5,5	0,04	0,00	3,37	0,00	0,04	0,15
3067	59	2	Ciamis	200-500	Petai	Parkia speciosa Hassk.	Leguminosae / Fabaceae	0,45	5,4	5	7,5	0,04	0,00	7,52	0,01	0,09	0,32
3068	59	2	Ciamis	200-500	Kokosan	Lansium domesticum var. aquae	Meliaceae	0,7	12,1	2,5	7,5	0,04	0,01	49,20	0,05	0,58	2,12
3069	59	2	Ciamis	200-500	Mahoni	Swietenia macrophylla King	Meliaceae	0,61	8,0	2,5	7,5	0,04	0,00	20,14	0,02	0,24	0,87
3070	59	2	Ciamis	200-500	Mahoni	Swietenia macrophylla King	Meliaceae	0,61	8,3	3,5	7,5	0,04	0,01	21,64	0,02	0,25	0,93
3071	59	2	Ciamis	200-500	Mahoni	Swietenia macrophylla King	Meliaceae	0,61	12,4	5	10	0,04	0,01	59,20	0,06	0,70	2,55
3072	59	2	Ciamis	200-500	Saga	Adenanthera pavonina L.	Leguminosae / Fabaceae	0,7	18,1	9	14	0,04	0,03	183,17	0,18	2,15	7,90
3073	59	2	Ciamis	200-500	Mahoni	Swietenia macrophylla King	Meliaceae	0,61	12,7	5	10	0,04	0,01	62,01	0,06	0,73	2,67
3074	59	2	Ciamis	200-500	Melinjo	Gnetum gnemon L.	Gnetaceae	0,76	6,4	1	7	0,04	0,00	15,37	0,02	0,18	0,66
3075	59	2	Ciamis	200-500	Salam	Syzygium polyanthum (Wight) Wa	Myrtaceae	0,66	5,1	3	6	0,04	0,00	7,79	0,01	0,09	0,34
3076	59	2	Ciamis	200-500	Saga	Adenanthera pavonina L.	Leguminosae / Fabaceae	0,7	6,7	8	11	0,04	0,00	23,57	0,02	0,28	1,02
3077	59	2	Ciamis	200-500	Mahoni	Swietenia macrophylla King	Meliaceae	0,61	16,2	7	13	0,04	0,02	123,06	0,12	1,45	5,31
3078	59	2	Ciamis	200-500	Mahoni	Swietenia macrophylla King	Meliaceae	0,61	6,4	2,5	4	0,04	0,00	7,52	0,01	0,09	0,32
3079	59	2	Ciamis	200-500	Mahoni	Swietenia macrophylla King	Meliaceae	0,61	4,8	5	7	0,04	0,00	7,42	0,01	0,09	0,32
3080	59	2	Ciamis	200-500	Rambutan	Nephelium lappaceum L.	Sapindaceae	0,71	9,9	5	9	0,04	0,01	40,56	0,04	0,48	1,75
3081	59	2	Ciamis	200-500	Mahoni	Swietenia macrophylla King	Meliaceae	0,61	14,3	7	12	0,04	0,02	90,93	0,09	1,07	3,92
3082	59	2	Ciamis	200-500	Mahoni	Swietenia macrophylla King	Meliaceae	0,61	11,8	1,5	7,5	0,04	0,01	41,30	0,04	0,49	1,78
3083	59	2	Ciamis	200-500	Mahoni	Swietenia macrophylla King	Meliaceae	0,61	14,0	8	13	0,04	0,02	93,90	0,09	1,10	4,05
3084	59	2	Ciamis	200-500	Mahoni	Swietenia macrophylla King	Meliaceae	0,61	12,4	7	12	0,04	0,01	69,96	0,07	0,82	3,02
3085	59	2	Ciamis	200-500	Saga	Adenanthera pavonina L.	Leguminosae / Fabaceae	0,7	12,1	11	16	0,04	0,01	98,48	0,10	1,16	4,25
3086	59	2	Ciamis	200-500	Mahoni	Swietenia macrophylla King	Meliaceae	0,61	6,4	9	12	0,04	0,00	20,58	0,02	0,24	0,89
3087	59	2	Ciamis	200-500	Mahoni	Swietenia macrophylla King	Meliaceae	0,61	13,0	3	10	0,04	0,01	64,88	0,06	0,76	2,80
3088	59	2	Ciamis	200-500	Mahoni	Swietenia macrophylla King	Meliaceae	0,61	7,3	7	11	0,04	0,00	24,55	0,02	0,29	1,06
3089	59	2	Ciamis	200-500	Mahoni	Swietenia macrophylla King	Meliaceae	0,61	9,9	5	10	0,04	0,01	38,87	0,04	0,46	1,68
3090	59	2	Ciamis	200-500	Mahoni	Swietenia macrophylla King	Meliaceae	0,61	8,3	8	3	0,04	0,01	9,35	0,01	0,11	0,40
3091	60	3	Ciamis	200-500	Jati	Tectona grandis L.f.	Lamiaceae / Verbenaceae	0,7	3,5	1,5	4,5	0,04	0,00	3,18	0,00	0,04	0,14
3092	60	3	Ciamis	200-500	Jati	Tectona grandis L.f.	Lamiaceae / Verbenaceae	0,7	3,8	1,8	4,8	0,04	0,00	3,96	0,00	0,05	0,17
3093	60	3	Ciamis	200-500	Jati	Tectona grandis L.f.	Lamiaceae / Verbenaceae	0,7	3,2	2	5	0,04	0,00	2,94	0,00	0,03	0,13
3094	60	3	Ciamis	200-500	Jati	Tectona grandis L.f.	Lamiaceae / Verbenaceae	0,7	4,1	1,8	5	0,04	0,00	4,76	0,00	0,06	0,21

3095	60	3	Ciamis	200-500	Sengon	Paraserianthes falcataria (L.) I.C.	Leguminosae / Fabaceae	0,33	8,9	11	14	0,04	0,01	25,01	0,03	0,29	1,08
3096	60	3	Ciamis	200-500	Jati	Tectona grandis L.f.	Lamiaceae / Verbenaceae	0,7	3,2	1	2	0,04	0,00	1,27	0,00	0,01	0,05
3097	60	3	Ciamis	200-500	Kokosan	Lansium domesticum var. aquae	Meliaceae	0,7	5,1	5	12	0,04	0,00	15,51	0,02	0,18	0,67
3098	60	3	Ciamis	200-500	Jati	Tectona grandis L.f.	Lamiaceae / Verbenaceae	0,7	2,5	1	3	0,04	0,00	1,22	0,00	0,01	0,05
3099	60	3	Ciamis	200-500	Kokosan	Lansium domesticum var. aquae	Meliaceae	0,7	1,9	3	4	0,04	0,00	0,94	0,00	0,01	0,04
3100	60	3	Ciamis	200-500	Nangka	Artocarpus heterophyllus Lam.	Moraceae	0,61	3,8	3,5	6,5	0,04	0,00	4,60	0,00	0,05	0,20
3101	60	3	Ciamis	200-500	Kokosan	Lansium domesticum var. aquae	Meliaceae	0,7	27,4	7	13	0,04	0,06	363,58	0,36	4,27	15,68
3102	60	3	Ciamis	200-500	Manggis	Mangostana garcinia Gaertn.	Clusiaceae	0,81	5,1	2	6	0,04	0,00	9,40	0,01	0,11	0,41
3103	60	3	Ciamis	200-500	Dukuh	Lansium domesticum Corrêa	Meliaceae	0,85	30,9	9	16	0,04	0,07	654,95	0,65	7,70	28,24
3104	60	3	Ciamis	200-500	Dukuh	Lansium domesticum Corrêa	Meliaceae	0,85	16,2	5	13	0,04	0,02	166,77	0,17	1,96	7,19
3105	60	3	Ciamis	200-500	Dukuh	Lansium domesticum Corrêa	Meliaceae	0,85	9,9	3	8	0,04	0,01	42,94	0,04	0,50	1,85
3106	60	3	Ciamis	200-500	Nangka	Artocarpus heterophyllus Lam.	Moraceae	0,61	7,6	7	16	0,04	0,00	37,41	0,04	0,44	1,61
3107	60	3	Ciamis	200-500	Manggis	Mangostana garcinia Gaertn.	Clusiaceae	0,81	7,0	2,5	7,5	0,04	0,00	20,66	0,02	0,24	0,89
3108	60	3	Ciamis	200-500	Dukuh	Lansium domesticum Corrêa	Meliaceae	0,85	2,9	2	5,5	0,04	0,00	3,16	0,00	0,04	0,14
3109	60	3	Ciamis	200-500	Rambutan	Nephelium lappaceum L.	Sapindaceae	0,71	12,7	10	15	0,04	0,01	103,31	0,10	1,21	4,46
3110	60	3	Ciamis	200-500	Kokosan	Lansium domesticum var. aquae	Meliaceae	0,7	9,5	7	11	0,04	0,01	45,31	0,05	0,53	1,95
3111	60	3	Ciamis	200-500	Dukuh	Lansium domesticum Corrêa	Meliaceae	0,85	6,7	2,5	3	0,04	0,00	8,57	0,01	0,10	0,37
3112	60	3	Ciamis	200-500	Dukuh	Lansium domesticum Corrêa	Meliaceae	0,85	41,0	2	14	0,04	0,13	977,06	0,98	11,48	42,13
3113	60	3	Ciamis	200-500	Mahoni	Swietenia macrophylla King	Meliaceae	0,61	4,5	5	8	0,04	0,00	7,39	0,01	0,09	0,32
3114	60	3	Ciamis	200-500	Aren	Arenga pinnata (Wurmb) Merr.	Arecaceae	0,71	55,4	5	9	0,04	0,24	73,80	0,07	0,87	3,18
3115	60	3	Ciamis	200-500	Jengkol	Archidendron pauciflorum (Bentl	Leguminosae / Fabaceae	0,4	4,1	4	7	0,04	0,00	3,88	0,00	0,05	0,17
3116	60	3	Ciamis	200-500	Kokosan	Lansium domesticum var. aquae	Meliaceae	0,7	12,4	4	10	0,04	0,01	67,15	0,07	0,79	2,90
3117	60	3	Ciamis	200-500	Dukuh	Lansium domesticum Corrêa	Meliaceae	0,85	10,8	2	7	0,04	0,01	45,00	0,05	0,53	1,94
3118	60	3	Ciamis	200-500	Kelapa	Cocos nucifera L.	Arecaceae	0,5	29,3	12	27	0,04	0,07	212,40	0,21	2,50	9,16
3119	60	3	Ciamis	200-500	Nangka	Artocarpus heterophyllus Lam.	Moraceae	0,61	16,9	7	12	0,04	0,02	122,71	0,12	1,44	5,29
3120	60	3	Ciamis	200-500	Dukuh	Lansium domesticum Corrêa	Meliaceae	0,85	29,3	6	14	0,04	0,07	525,99	0,53	6,18	22,68
3121	60	3	Ciamis	200-500	Durian	Durio zibethinus L.	Malvaceae	0,57	10,2	12	14	0,04	0,01	52,70	0,05	0,62	2,27
3122	60	3	Ciamis	200-500	Dukuh	Lansium domesticum Corrêa	Meliaceae	0,85	8,0	2	5	0,04	0,00	18,83	0,02	0,22	0,81
3123	60	3	Ciamis	200-500	Kokosan	Lansium domesticum var. aquae	Meliaceae	0,7	8,0	5	9	0,04	0,00	27,00	0,03	0,32	1,16
3124	60	3	Ciamis	200-500	Tisuk	Hibiscus macrophyllus Roxb. ex I	Malvaceae	0,4	11,1	8	14	0,04	0,01	44,89	0,04	0,53	1,94
3125	60	3	Ciamis	200-500	Kokosan	Lansium domesticum var. aquae	Meliaceae	0,7	3,8	3	8,5	0,04	0,00	6,68	0,01	0,08	0,29
3126	60	3	Ciamis	200-500	Dukuh	Lansium domesticum Corrêa	Meliaceae	0,85	24,2	2,5	135	0,04	0,05	2.954,63	2,95	34,72	127,41
3127	60	3	Ciamis	200-500	Dukuh	Lansium domesticum Corrêa	Meliaceae	0,85	36,0	2	25	0,04	0,10	1.303,83	1,30	15,32	56,22
3128	60	3	Ciamis	200-500	Jengkol	Archidendron pauciflorum (Bentl	Leguminosae / Fabaceae	0,4	3,8	3	4,5	0,04	0,00	2,23	0,00	0,03	0,10
3129	60	3	Ciamis	200-500	Dukuh	Lansium domesticum Corrêa	Meliaceae	0,85	11,5	8	12	0,04	0,01	81,87	0,08	0,96	3,53
3130	60	3	Ciamis	200-500	Dukuh	Lansium domesticum Corrêa	Meliaceae	0,85	5,7	3	5,5	0,04	0,00	11,25	0,01	0,13	0,49
3131	60	3	Ciamis	200-500	Dukuh	Lansium domesticum Corrêa	Meliaceae	0,85	32,8	3	13	0,04	0,08	604,45	0,60	7,10	26,07
3132	60	3	Ciamis	200-500	Kokosan	Lansium domesticum var. aquae	Meliaceae	0,7	6,4	4	10	0,04	0,00	19,76	0,02	0,23	0,85
3133	60	3	Ciamis	200-500	Kokosan	Lansium domesticum var. aquae	Meliaceae	0,7	15,3	4,5	10,5	0,04	0,02	102,72	0,10	1,21	4,43
3134	61	3	Ciamis	200-500	Mahoni	Swietenia macrophylla King	Meliaceae	0,61	5,7	4	4	0,04	0,00	6,20	0,01	0,07	0,27
3135	61	3	Ciamis	200-500	Mahoni	Swietenia macrophylla King	Meliaceae	0,61	3,5	3,5	5,5	0,04	0,00	3,37	0,00	0,04	0,15
3136	61	3	Ciamis	200-500	Mahoni	Swietenia macrophylla King	Meliaceae	0,61	10,5	3	8	0,04	0,01	35,53	0,04	0,42	1,53
3137	61	3	Ciamis	200-500	Mahoni	Swietenia macrophylla King	Meliaceae	0,61	1,9	2	3	0,04	0,00	0,64	0,00	0,01	0,03
3138	61	3	Ciamis	200-500	Mahoni	Swietenia macrophylla King	Meliaceae	0,61	8,0	7	9	0,04	0,00	23,80	0,02	0,28	1,03
3139	61	3	Ciamis	200-500	Mahoni	Swietenia macrophylla King	Meliaceae	0,61	10,5	5	11	0,04	0,01	47,57	0,05	0,56	2,05
3140	61	3	Ciamis	200-500	Mahoni	Swietenia macrophylla King	Meliaceae	0,61	4,1	4	5	0,04	0,00	4,19	0,00	0,05	0,18
3141	61	3	Ciamis	200-500	Tisuk	Hibiscus macrophyllus Roxb. ex I	Malvaceae	0,4	8,6	8	10	0,04	0,01	20,51	0,02	0,24	0,88
3142	61	3	Ciamis	200-500	Tisuk	Hibiscus macrophyllus Roxb. ex I	Malvaceae	0,4	11,5	10	14	0,04	0,01	47,27	0,05	0,56	2,04
3143	61	3	Ciamis	200-500	Nangka	Artocarpus heterophyllus Lam.	Moraceae	0,61	16,5	4	9	0,04	0,02	91,05	0,09	1,07	3,93
3144	61	3	Ciamis	200-500	Kecapi	Sandoricum koetjape (Burm.f.) M	Meliaceae	0,44	6,4	8	10	0,04	0,00	12,91	0,01	0,15	0,56
3145	61	3	Ciamis	200-500	Kecapi	Sandoricum koetjape (Burm.f.) M	Meliaceae	0,44	3,8	5	7	0,04	0,00	3,65	0,00	0,04	0,16
3146	61	3	Ciamis	200-500	Kecapi	Sandoricum koetjape (Burm.f.) M	Meliaceae	0,44	7,3	4	7	0,04	0,00	12,03	0,01	0,14	0,52
3147	61	3	Ciamis	200-500	Kelapa	Cocos nucifera L.	Arecaceae	0,5	29,6	12	15	0,04	0,07	120,00	0,12	1,41	5,17
3148	61	3	Ciamis	200-500	Mahoni	Swietenia macrophylla King	Meliaceae	0,61	15,6	5	10	0,04	0,02	89,93	0,09	1,06	3,88
3149	61	3	Ciamis	200-500	Matoa	Pometia pinnata J.R.Forst. & G.Fo	Sapindaceae	0,77	10,2	6	11	0,04	0,01	55,65	0,06	0,65	2,40
3150	61	3	Ciamis	200-500	Manggis	Mangostana garcinia Gaertn.	Clusiaceae	0,81	2,9	1	2,5	0,04	0,00	1,47	0,00	0,02	0,06
3151	61	3	Ciamis	200-500	Pulai	Alstonia scholaris (L.) R. Br.	Apocynaceae	0,3	29,3	11	15	0,04	0,07	215,83	0,22	2,54	9,31
3152	61	3	Ciamis	200-500	Kelapa	Cocos nucifera L.	Arecaceae	0,5	28,6	11	14	0,04	0,06	112,30	0,11	1,32	4,84
3153	61	3	Ciamis	200-500	Mahoni	Swietenia macrophylla King	Meliaceae	0,61	8,9	7	10	0,04	0,01	32,26	0,03	0,38	1,39
3154	61	3	Ciamis	200-500	Sengon	Paraserianthes falcataria (L.) I.C.	Leguminosae / Fabaceae	0,33	27,0	15	20	0,04	0,06	265,17	0,27	3,12	11,43
3155	61	3	Ciamis	200-500	Mahoni	Swietenia macrophylla King	Meliaceae	0,61	15,0	7	5	0,04	0,02	44,16	0,04	0,52	1,90
3156	61	3	Ciamis	200-500	Mahoni	Swietenia macrophylla King	Meliaceae	0,61	8,9	4	8	0,04	0,01	26,30	0,03	0,31	1,13
3157	61	3	Ciamis	200-500	Sengon	Paraserianthes falcataria (L.) I.C.	Leguminosae / Fabaceae	0,33	12,7	10	13	0,04	0,01	44,92	0,04	0,53	1,94
3158	61	3	Ciamis	200-500	Mahoni	Swietenia macrophylla King	Meliaceae	0,61	2,9	2,5	5	0,04	0,00	2,14	0,00	0,03	0,09
3159	61	3	Ciamis	200-500	Mahoni	Swietenia macrophylla King	Meliaceae	0,61	4,5	4	6	0,04	0,00	5,68	0,01	0,07	0,24
3160	61	3	Ciamis	200-500	Mahoni	Swietenia macrophylla King	Meliaceae	0,61	4,5	4	5,5	0,04	0,00	5,24	0,01	0,06	0,23
3161	61	3	Ciamis	200-500	Mahoni	Swietenia macrophylla King	Meliaceae	0,61	5,4	5	7	0,04	0,00	9,33	0,01	0,11	0,40
3162	61	3	Ciamis	200-500	Mahoni	Swietenia macrophylla King	Meliaceae	0,61	4,1	3	5	0,04	0,00	4,19	0,00	0,05	0,18
3163	61	3	Ciamis	200-500	Sp8	Sp8	0	0,5	3,8	2,5	5	0,04	0,00	3,02	0,00	0,04	0,13
3164	61	3	Ciamis	200-500	Mahoni	Swietenia macrophylla King	Meliaceae	0,61	6,0	5	6	0,04	0,00	9,93	0,01	0,12	0,43
3165	61	3	Ciamis	200-500	Mahoni	Swietenia macrophylla King	Meliaceae	0,61	6,0	3,5	4,5	0,04	0,00	7,63	0,01	0,09	0,33
3166	61	3	Ciamis	200-500	Mahoni	Swietenia macrophylla King	Meliaceae	0,61	10,8	5	8	0,04	0,01	37,53	0,04	0,44	1,62
3167	61	3	Ciamis	200-500	Mahoni	Swietenia macrophylla King	Meliaceae	0,61	14,3	7	11	0,04	0,02	83,96	0,08	0,99	3,62
3168	61	3	Ciamis	200-500	Mahoni	Swietenia macrophylla King	Meliaceae	0,61	4,1	4	7	0,04	0,00	5,71	0,01	0,07	0,25
3169	61	3	Ciamis	200-500	Mahoni	Swietenia macrophylla King	Meliaceae	0,61	3,8	3	5	0,04	0,00	3,62	0,00	0,04	0,16
3170	61	3	Ciamis	200-500	Kecapi	Sandoricum koetjape (Burm.f.) M	Meliaceae	0,44	13,7	3,5	9	0,04	0,01	47,66	0,05	0,56	2,06
3171	61	3	Ciamis	200-500	Mahoni	Swietenia macrophylla King	Meliaceae	0,61	4,1	4	5,5	0,04	0,00	4,58	0,00	0,05	0,20
3172	61	3	Ciamis	200-500	Tisuk	Hibiscus macrophyllus Roxb. ex I	Malvaceae	0,4	6,0	9	11	0,04	0,00	11,75	0,01	0,14	0,51
3173	61	3	Ciamis	200-500	Mahoni	Swietenia macrophylla King	Meliaceae	0,61	10,2	7	10	0,04	0,01	41,20	0,04	0,48	1,78
3174	61	3	Ciamis	200-500	Mahoni afrika	Khaya anthotheca (Welw.) C.DC.	Meliaceae	0,49	9,9	12	1	0,04	0,01	3,86	0,00	0,05	0,17
3175	61	3	Ciamis	200-500	Mahoni	Swietenia macrophylla King	Meliaceae	0,61	4,8	3,5	5	0,04	0,00	5,45	0,01	0,06	0,23
3176	61	3	Ciamis	200-500	Mahoni	Swietenia macrophylla King	Meliaceae	0,61	6,4	4	9	0,04	0,00	15,81	0,02	0,19	0,68
3177	61	3	Ciamis	200-500	Mahoni	Swietenia macrophylla King	Meliaceae	0,61	10,5	7	13	0,04	0,01	55,43	0,06	0,65	2,39
3178	61	3	Ciamis	200-500	Mahoni	Swietenia macrophylla King	Meliaceae	0,61	8,0	6	10	0,04	0,00	26,21	0,03	0,31	1,13
3179	61	3	Ciamis	200-500	Mahoni	Swietenia macrophylla King	Meliaceae	0,61	1,9	2	3,5	0,04	0,00	0,73	0,00	0,01	0,03
3180	61	3	Ciamis	200-500	Mahoni	Swietenia macrophylla King	Meliaceae	0,61	13,4	5	9	0,04	0,01	61,57	0,06	0,72	2,65
3181	61	3	Ciamis	200-500	Kelapa	Cocos nucifera L.	Arecaceae	0,5	30,5	10	13	0,04	0,07	104,60	0,10	1,23	4,51
3182	61	3	Ciamis	200-500	Mahoni	Swietenia macrophylla King	Meliaceae	0,61	4,8	3,5	5	0,04	0,00	5,45	0,01	0,06	0,23
3183	61	3	Ciamis	200-500	Mahoni	Swietenia macrophylla King	Meliaceae	0,61	4,5	6	7,3	0,04	0,00	6,79	0,01	0,08	0,29
3184	61	3	Ciamis	200-500	Kecapi	Sandoricum koetjape (Burm.f.) M	Meliaceae	0,44	11,1	9	14	0,04	0,01	48,99	0,05	0,58	2,11
3185	61	3	Ciamis	200-500	Mahoni	Swietenia macrophylla King	Meliaceae	0,61	3,2	2	3	0,04	0,00	1,62	0,00	0,02	0,07
3186	61	3	Ciamis	200-500	Mahoni	Swietenia macrophylla King	Meliaceae	0,61	7,0	3,5	5,5	0,04	0,00	11,99	0,01	0,14	0,52
3187	61	3	Ciamis	200-500	Mahoni afrika	Khaya anthotheca (Welw.) C.DC.	Meliaceae	0,49	9,2	9	1	0,04	0,01	3,42	0,00	0,04	0,15
3188	61	3	Ciamis	200-500	Kihuut	Aporosa fructescens Blume	Phyllanthaceae	0,37	4,5	3,5	7	0,04	0,00	4,13	0,00	0,05	0,18
3189	61	3	Ciamis	200-500	Mahoni	Swietenia macrophylla King	Meliaceae	0,61	10,5	7	12	0,04	0,01	51,51	0,05	0,61	2,22
3190	61	3	Ciamis	200-500	Kecapi	Sandoricum koetjape (Burm.f.) M	Meliaceae	0,44	16,2	11	15	0,04	0,02	104,01	0,10	1,22	4,49
3191	61	3	Ciamis	200-500	Kihuut	Aporosa fructescens Blume	Phyllanthaceae	0,37	13,0	4	8	0,04	0,01	33,45	0,03	0,39	1,44
3192	61	3	Ciamis	200-500	Mahoni	Swietenia macrophylla King	Meliaceae	0,61	10,8	3	8	0,04	0,01	37,53	0,04	0,44	1,62
3193	61	3	Ciamis	200-500	Jengkol	Archidendron pauciflorum (Bentl	Leguminosae / Fabaceae	0,4	5,1	3	5	0,04	0,00	4,17	0,00	0,05	0,18
3194	62	3	Ciamis	200-500	Mahoni	Swietenia macrophylla King	Meliaceae	0,61	6,4	3	11	0,04	0,00	19,01	0,02	0,22	0,82
3195	62	3	Ciamis	200-500	Mahoni	Swietenia macrophylla King	Meliaceae	0,61	12,4	3,5	10,5	0,04	0,01	61,90	0,06	0,73	2,67
3196	62	3	Ciamis	200-500	Mahoni	Swietenia macrophylla King	Meliaceae	0,61	20,7	5,5	14,5	0,04	0,03	212,11	0,21	2,49	9,15
3197	62	3	Ciamis	200-500	Jati	Tectona grandis L.f.	Lamiaceae / Verbenaceae	0,7	15,9	7	12	0,04	0,02	125,10	0,13	1,47	5,39
3198	62	3	Ciamis	200-500	Jati	Tectona grandis L.f.	Lamiaceae / Verbenaceae	0,7	4,8	2,5	6,5	0,04	0,00	7,86	0,01	0,09	0,34
3199	62	3	Ciamis	200-500	Jati	Tectona grandis L.f.	Lamiaceae / Verbenaceae	0,7	8,0	3,5	6,4	0,04	0,00	19,76	0,02	0,23	0,85
3200	62	3	Ciamis	200-500	Mahoni	Swietenia macrophylla King	Meliaceae	0,61	11,8	9	13	0,04	0,01	68,36	0,07	0,80	2,95
3201	62	3	Ciamis	200-500	Mahoni	Swietenia macrophylla King	Meliaceae	0,61	12,7	7	12	0,04	0,01	73,28	0,07	0,86	3,16
3202	62	3	Ciamis	200-500	Jati	Tectona grandis L.f.	Lamiaceae / Verbenaceae	0,7	12,1	2	9	0,04	0,01	58,14	0,06	0,68	2,51
3203	62	3	Ciamis	200-500	Mahoni	Swietenia macrophylla King	Meliaceae	0,61	19,1	8	15	0,04	0,03	188,95	0,19	2,22	8,15
3204	62	3	Ciamis	200-500	Jati	Tectona grandis L.f.	Lamiaceae / Verbenaceae	0,7	19,7	10	16	0,04	0,03	241,47	0,24	2,84	10,41
3205	62	3	Ciamis	200-500	Jati	Tectona grandis L.f.	Lamiaceae / Verbenaceae	0,7	22,9	10	17	0,04	0,04	335,70	0,34	3,94	14,48
3206	62	3	Ciamis	200-500	Jati	Tectona grandis L.f.	Lamiaceae / Verbenaceae	0,7	17,5	5	14	0,04	0,02	171,56	0,17	2,02	7,40
3207	62	3	Ciamis	200-500	Jati	Tectona grandis L.f.	Lamiaceae / Verbenaceae	0,7	26,7	7	20	0,04	0,06	516,72	0,52	6,07	22,28
3208	62	3	Ciamis	200-500	Tisuk	Hibiscus macrophyllus Roxb. ex I	Malvaceae	0,4	21,6	15	22	0,04	0,04	229,31	0,23	2,69	9,89
3209	62	3	Ciamis	200-500	Jati	Tectona grandis L.f.	Lamiaceae / Verbenaceae	0,7	12,1	4	13	0,04	0,01	81,43	0,08	0,96	3,51
3210	62	3	Ciamis	200-500	Jati	Tectona grandis L.f.	Lamiaceae / Verbenaceae	0,7	24,5	12	22	0,04	0,05	480,77	0,48	5,65	20,73
3211	62	3	Ciamis	200-500	Durian	Durio zibethinus L.	Malvaceae	0,57	4,1	2	3,5	0,04	0,00	2,84	0,00	0,03	0,12
3212	62	3	Ciamis	200-500	Jati	Tectona grandis L.f.	Lamiaceae / Verbenaceae	0,7	19,4	10	19	0,04	0,03	274,34	0,27	3,22	11,83
3213	62	3	Ciamis	200-500	Jati	Tectona grandis L.f.	Lamiaceae / Verbenaceae	0,7	17,2	17	24	0,04	0,02	271,80	0,27	3,19	11,72
3214	62	3	Ciamis	200-500	Jati	Tectona grandis L.f.	Lamiaceae / Verbenaceae	0,7	10,2	7	12	0,04	0,01	55,23	0,06	0,65	2,38
3215	62	3	Ciamis	200-500	Jati	Tectona grandis L.f.	Lamiaceae / Verbenaceae	0,7	17,8	9	16	0,04	0,02	200,39	0,20	2,35	8,64
3216	62	3	Ciamis	200-500	Manglid	Magnolia champaca (L.) Baill. ex	Magnoliaceae	0,53	18,5	8	17	0,04	0,03	175,08	0,18	2,06	7,55
3217	62	3	Ciamis	200-500	Jati	Tectona grandis L.f.	Lamiaceae / Verbenaceae	0,7	8,6	3,5	7	0,04	0,01	24,69	0,02	0,29	1,06
3218	62	3	Ciamis	200-500	Tisuk	Hibiscus macrophyllus Roxb. ex I	Malvaceae	0,4	11,8	12	17	0,04	0,01	59,38	0,06	0,70	2,56

3219	62	3	Ciamis	200-500	Mahoni	Swietenia macrophylla King	Meliaceae	0,61	4,5	3,5	4,5	0,04	0,00	4,36	0,00	0,05	0,19
3220	62	3	Ciamis	200-500	Mahoni	Swietenia macrophylla King	Meliaceae	0,61	5,4	3,5	7	0,04	0,00	9,33	0,01	0,11	0,40
3221	62	3	Ciamis	200-500	Jati	Tectona grandis L.f.	Lamiaceae / Verbenaceae	0,7	9,5	4	11	0,04	0,01	45,31	0,05	0,53	1,95
3222	62	3	Ciamis	200-500	Jati	Tectona grandis L.f.	Lamiaceae / Verbenaceae	0,7	13,0	5	12	0,04	0,01	86,97	0,09	1,02	3,75
3223	62	3	Ciamis	200-500	Jati	Tectona grandis L.f.	Lamiaceae / Verbenaceae	0,7	19,7	6	14	0,04	0,03	213,67	0,21	2,51	9,21
3224	62	3	Ciamis	200-500	Gmelina	Gmelina arborea Roxb.	Lamiaceae / Verbenaceae	0,47	6,0	8	11	0,04	0,00	13,63	0,01	0,16	0,59
3225	62	3	Ciamis	200-500	Jati	Tectona grandis L.f.	Lamiaceae / Verbenaceae	0,7	9,2	3	9	0,04	0,01	35,43	0,04	0,42	1,53
3226	62	3	Ciamis	200-500	Durian	Durio zibethinus L.	Malvaceae	0,57	2,9	1	3,5	0,04	0,00	1,45	0,00	0,02	0,06
3227	62	3	Ciamis	200-500	Jati	Tectona grandis L.f.	Lamiaceae / Verbenaceae	0,7	12,1	3,5	10,5	0,04	0,01	66,96	0,07	0,79	2,89
3228	62	3	Ciamis	200-500	Gmelina	Gmelina arborea Roxb.	Lamiaceae / Verbenaceae	0,47	8,3	9	12	0,04	0,01	26,21	0,03	0,31	1,13
3229	62	3	Ciamis	200-500	Gmelina	Gmelina arborea Roxb.	Lamiaceae / Verbenaceae	0,47	14,3	13	16	0,04	0,02	93,20	0,09	1,10	4,02
3230	62	3	Ciamis	200-500	Sengon	Paraserianthes falcataria (L.) I.C.	Leguminosae / Fabaceae	0,33	29,9	18	24	0,04	0,07	376,81	0,38	4,43	16,25
3231	62	3	Ciamis	200-500	Gmelina	Gmelina arborea Roxb.	Lamiaceae / Verbenaceae	0,47	10,2	7	8	0,04	0,01	26,45	0,03	0,31	1,14
3232	62	3	Ciamis	200-500	Tisuk	Hibiscus macrophyllus Roxb. ex I	Malvaceae	0,4	13,4	9	15	0,04	0,01	66,79	0,07	0,78	2,88
3233	62	3	Ciamis	200-500	Durian	Durio zibethinus L.	Malvaceae	0,57	17,8	13	17	0,04	0,02	175,50	0,18	2,06	7,57
3234	62	3	Ciamis	200-500	Kelapa	Cocos nucifera L.	Arecaceae	0,5	26,1	17	20	0,04	0,05	158,50	0,16	1,86	6,83
3235	62	3	Ciamis	200-500	Petai	Parkia speciosa Hassk.	Leguminosae / Fabaceae	0,45	21,6	19	22	0,04	0,04	255,43	0,26	3,00	11,01
3236	62	3	Ciamis	200-500	Durian	Durio zibethinus L.	Malvaceae	0,57	24,8	13	18	0,04	0,05	339,35	0,34	3,99	14,63
3237	62	3	Ciamis	200-500	Gmelina	Gmelina arborea Roxb.	Lamiaceae / Verbenaceae	0,47	6,4	9	10	0,04	0,00	13,72	0,01	0,16	0,59
3238	62	3	Ciamis	200-500	Gmelina	Gmelina arborea Roxb.	Lamiaceae / Verbenaceae	0,47	9,5	9	10	0,04	0,01	28,83	0,03	0,34	1,24
3239	62	3	Ciamis	200-500	Gmelina	Gmelina arborea Roxb.	Lamiaceae / Verbenaceae	0,47	6,0	8	9	0,04	0,00	11,34	0,01	0,13	0,49
3240	62	3	Ciamis	200-500	Jati	Tectona grandis L.f.	Lamiaceae / Verbenaceae	0,7	18,5	7	19	0,04	0,03	250,13	0,25	2,94	10,79
3241	62	3	Ciamis	200-500	Tisuk	Hibiscus macrophyllus Roxb. ex I	Malvaceae	0,4	6,7	53	8	0,04	0,00	10,55	0,01	0,12	0,45
3242	62	3	Ciamis	200-500	Jati	Tectona grandis L.f.	Lamiaceae / Verbenaceae	0,7	10,5	5	8	0,04	0,01	40,31	0,04	0,47	1,74
3243	62	3	Ciamis	200-500	Kelapa	Cocos nucifera L.	Arecaceae	0,5	22,9	12	15	0,04	0,04	120,00	0,12	1,41	5,17
3244	62	3	Ciamis	200-500	Tisuk	Hibiscus macrophyllus Roxb. ex I	Malvaceae	0,4	17,5	18	11,5	0,04	0,02	85,81	0,09	1,01	3,70
3245	62	3	Ciamis	200-500	Gmelina	Gmelina arborea Roxb.	Lamiaceae / Verbenaceae	0,47	3,8	3	6	0,04	0,00	3,37	0,00	0,04	0,15
3246	62	3	Ciamis	200-500	Tisuk	Hibiscus macrophyllus Roxb. ex I	Malvaceae	0,4	18,1	8	24	0,04	0,03	179,74	0,18	2,11	7,75
3247	62	3	Ciamis	200-500	Gmelina	Gmelina arborea Roxb.	Lamiaceae / Verbenaceae	0,47	18,5	14	17	0,04	0,03	156,84	0,16	1,84	6,76
3248	62	3	Ciamis	200-500	Tisuk	Hibiscus macrophyllus Roxb. ex I	Malvaceae	0,4	12,1	9	7	0,04	0,01	27,66	0,03	0,33	1,19
3249	62	3	Ciamis	200-500	Tisuk	Hibiscus macrophyllus Roxb. ex I	Malvaceae	0,4	9,9	12	15	0,04	0,01	38,29	0,04	0,45	1,65
3250	62	3	Ciamis	200-500	Jati	Tectona grandis L.f.	Lamiaceae / Verbenaceae	0,7	6,4	3	6	0,04	0,00	12,37	0,01	0,15	0,53
3251	62	3	Ciamis	200-500	Gmelina	Gmelina arborea Roxb.	Lamiaceae / Verbenaceae	0,47	8,0	5	11	0,04	0,00	22,53	0,02	0,26	0,97
3252	62	3	Ciamis	200-500	Manglid	Magnolia champaca (L.) Baill. ex	Magnoliaceae	0,53	2,9	4	5	0,04	0,00	1,88	0,00	0,02	0,08
3253	62	3	Ciamis	200-500	Gmelina	Gmelina arborea Roxb.	Lamiaceae / Verbenaceae	0,47	7,3	9	12	0,04	0,00	20,94	0,02	0,25	0,90
3254	62	3	Ciamis	200-500	Gmelina	Gmelina arborea Roxb.	Lamiaceae / Verbenaceae	0,47	16,5	10	14	0,04	0,02	107,48	0,11	1,26	4,63
3255	62	3	Ciamis	200-500	Jati	Tectona grandis L.f.	Lamiaceae / Verbenaceae	0,7	8,0	5	12	0,04	0,00	35,14	0,04	0,41	1,52
3256	62	3	Ciamis	200-500	Jati	Tectona grandis L.f.	Lamiaceae / Verbenaceae	0,7	5,7	1	3	0,04	0,00	5,41	0,01	0,06	0,23
3257	62	3	Ciamis	200-500	Gmelina	Gmelina arborea Roxb.	Lamiaceae / Verbenaceae	0,47	25,5	13	19	0,04	0,05	313,01	0,31	3,68	13,50
3258	62	3	Ciamis	200-500	Gmelina	Gmelina arborea Roxb.	Lamiaceae / Verbenaceae	0,47	4,1	5	7	0,04	0,00	4,49	0,00	0,05	0,19
3259	62	3	Ciamis	200-500	Gmelina	Gmelina arborea Roxb.	Lamiaceae / Verbenaceae	0,47	14,3	12	17	0,04	0,02	98,52	0,10	1,16	4,25
3260	62	3	Ciamis	200-500	Jati	Tectona grandis L.f.	Lamiaceae / Verbenaceae	0,7	8,6	9	13	0,04	0,01	43,54	0,04	0,51	1,88
3261	62	3	Ciamis	200-500	Gmelina	Gmelina arborea Roxb.	Lamiaceae / Verbenaceae	0,47	4,1	2	4	0,04	0,00	2,69	0,00	0,03	0,12
3262	63	3	Ciamis	200-500	Jati	Tectona grandis L.f.	Lamiaceae / Verbenaceae	0,7	27,7	3	12	0,04	0,06	345,11	0,35	4,06	14,88
3263	63	3	Ciamis	200-500	Durian	Durio zibethinus L.	Malvaceae	0,57	6,4	2	5	0,04	0,00	8,67	0,01	0,10	0,37
3264	63	3	Ciamis	200-500	Petai	Parkia speciosa Hassk.	Leguminosae / Fabaceae	0,45	5,1	3	5	0,04	0,00	4,64	0,00	0,05	0,20
3265	63	3	Ciamis	200-500	Jati	Tectona grandis L.f.	Lamiaceae / Verbenaceae	0,7	25,5	5	14	0,04	0,05	340,83	0,34	4,00	14,70
3266	63	3	Ciamis	200-500	Sengon	Paraserianthes falcataria (L.) I.C.	Leguminosae / Fabaceae	0,33	13,7	10	13	0,04	0,01	51,28	0,05	0,60	2,21
3267	63	3	Ciamis	200-500	Sengon	Paraserianthes falcataria (L.) I.C.	Leguminosae / Fabaceae	0,33	22,3	9	16	0,04	0,04	151,45	0,15	1,78	6,53
3268	63	3	Ciamis	200-500	Sengon	Paraserianthes falcataria (L.) I.C.	Leguminosae / Fabaceae	0,33	28,0	10	17	0,04	0,06	243,48	0,24	2,86	10,50
3269	63	3	Ciamis	200-500	Mahoni	Swietenia macrophylla King	Meliaceae	0,61	13,7	3,5	9,5	0,04	0,01	67,54	0,07	0,79	2,91
3270	63	3	Ciamis	200-500	Jati	Tectona grandis L.f.	Lamiaceae / Verbenaceae	0,7	5,4	3	5	0,04	0,00	7,77	0,01	0,09	0,34
3271	63	3	Ciamis	200-500	Jati	Tectona grandis L.f.	Lamiaceae / Verbenaceae	0,7	6,4	3	6	0,04	0,00	12,37	0,01	0,15	0,53
3272	63	3	Ciamis	200-500	Sengon	Paraserianthes falcataria (L.) I.C.	Leguminosae / Fabaceae	0,33	6,4	7	8,5	0,04	0,00	8,55	0,01	0,10	0,37
3273	63	3	Ciamis	200-500	Jati	Tectona grandis L.f.	Lamiaceae / Verbenaceae	0,7	13,0	3,5	10,5	0,04	0,01	76,96	0,08	0,90	3,32
3274	63	3	Ciamis	200-500	Mahoni	Swietenia macrophylla King	Meliaceae	0,61	9,9	3,5	8,5	0,04	0,01	33,50	0,03	0,39	1,44
3275	63	3	Ciamis	200-500	Mahoni	Swietenia macrophylla King	Meliaceae	0,61	9,5	3,5	8,5	0,04	0,01	31,54	0,03	0,37	1,36
3276	63	3	Ciamis	200-500	Mahoni	Swietenia macrophylla King	Meliaceae	0,61	3,2	2	4	0,04	0,00	2,11	0,00	0,02	0,09
3277	63	3	Ciamis	200-500	Kokosan	Lansium domesticum var. aquae	Meliaceae	0,7	10,2	4	6,5	0,04	0,01	31,50	0,03	0,37	1,36
3278	64	2	Ciamis	200-500	Sengon	Paraserianthes falcataria (L.) I.C.	Leguminosae / Fabaceae	0,33	19,1	12	15	0,04	0,03	107,63	0,11	1,26	4,64
3279	64	2	Ciamis	200-500	Tisuk	Hibiscus macrophyllus Roxb. ex I	Malvaceae	0,4	7,6	9	12	0,04	0,00	19,53	0,02	0,23	0,84
3280	64	2	Ciamis	200-500	Caruy	Pterospermum javanicum Jungh.	Malvaceae	0,53	3,2	2	3	0,04	0,00	1,43	0,00	0,02	0,06
3281	64	2	Ciamis	200-500	Sengon	Paraserianthes falcataria (L.) I.C.	Leguminosae / Fabaceae	0,33	6,7	3	8	0,04	0,00	8,84	0,01	0,10	0,38
3282	64	2	Ciamis	200-500	Sengon	Paraserianthes falcataria (L.) I.C.	Leguminosae / Fabaceae	0,33	11,1	9	14	0,04	0,01	37,64	0,04	0,44	1,62
3283	64	2	Ciamis	200-500	Petai	Parkia speciosa Hassk.	Leguminosae / Fabaceae	0,45	6,0	3	7	0,04	0,00	8,65	0,01	0,10	0,37
3284	64	2	Ciamis	200-500	Sengon	Paraserianthes falcataria (L.) I.C.	Leguminosae / Fabaceae	0,33	22,3	15	20	0,04	0,04	185,80	0,19	2,18	8,01
3285	64	2	Ciamis	200-500	Tisuk	Hibiscus macrophyllus Roxb. ex I	Malvaceae	0,4	5,4	4	8	0,04	0,00	7,16	0,01	0,08	0,31
3286	64	2	Ciamis	200-500	Mahoni	Swietenia macrophylla King	Meliaceae	0,61	6,0	8	6	0,04	0,00	9,93	0,01	0,12	0,43
3287	64	2	Ciamis	200-500	Sengon	Paraserianthes falcataria (L.) I.C.	Leguminosae / Fabaceae	0,33	23,5	12	17	0,04	0,04	177,26	0,18	2,08	7,64
3288	64	2	Ciamis	200-500	Sengon	Paraserianthes falcataria (L.) I.C.	Leguminosae / Fabaceae	0,33	26,4	12	17	0,04	0,05	218,74	0,22	2,57	9,43
3289	64	2	Ciamis	200-500	Mahoni	Swietenia macrophylla King	Meliaceae	0,61	4,1	2	5	0,04	0,00	4,19	0,00	0,05	0,18
3290	64	2	Ciamis	200-500	Mahoni	Swietenia macrophylla King	Meliaceae	0,61	3,5	2	1,5	0,04	0,00	1,02	0,00	0,01	0,04
3291	64	2	Ciamis	200-500	Caruy	Pterospermum javanicum Jungh.	Malvaceae	0,53	4,8	3,5	5,5	0,04	0,00	5,23	0,01	0,06	0,23
3292	64	2	Ciamis	200-500	Sengon	Paraserianthes falcataria (L.) I.C.	Leguminosae / Fabaceae	0,33	4,8	3	4	0,04	0,00	2,53	0,00	0,03	0,11
3293	64	2	Ciamis	200-500	Mahoni	Swietenia macrophylla King	Meliaceae	0,61	3,5	2	3,5	0,04	0,00	2,23	0,00	0,03	0,10
3294	64	2	Ciamis	200-500	Sengon	Paraserianthes falcataria (L.) I.C.	Leguminosae / Fabaceae	0,33	9,9	10	12	0,04	0,01	26,17	0,03	0,31	1,13
3295	64	2	Ciamis	200-500	Sengon	Paraserianthes falcataria (L.) I.C.	Leguminosae / Fabaceae	0,33	8,0	8	11	0,04	0,00	16,29	0,02	0,19	0,70
3296	64	2	Ciamis	200-500	Kihuut	Aporosa fructescens Blume	Phyllanthaceae	0,37	4,8	3	6	0,04	0,00	4,07	0,00	0,05	0,18
3297	64	2	Ciamis	200-500	Tisuk	Hibiscus macrophyllus Roxb. ex I	Malvaceae	0,4	20,4	10	14	0,04	0,03	135,64	0,14	1,59	5,85
3298	64	2	Ciamis	200-500	Tisuk	Hibiscus macrophyllus Roxb. ex I	Malvaceae	0,4	3,8	3	6	0,04	0,00	2,91	0,00	0,03	0,13
3299	64	2	Ciamis	200-500	Sengon	Paraserianthes falcataria (L.) I.C.	Leguminosae / Fabaceae	0,33	15,0	10	15	0,04	0,02	68,81	0,07	0,81	2,97
3300	64	2	Ciamis	200-500	Tisuk	Hibiscus macrophyllus Roxb. ex I	Malvaceae	0,4	12,7	7	12	0,04	0,01	49,79	0,05	0,58	2,15
3301	64	2	Ciamis	200-500	Caruy	Pterospermum javanicum Jungh.	Malvaceae	0,53	5,4	3	6	0,04	0,00	7,12	0,01	0,08	0,31
3302	64	2	Ciamis	200-500	Tisuk	Hibiscus macrophyllus Roxb. ex I	Malvaceae	0,4	13,7	4,5	9,5	0,04	0,01	45,89	0,05	0,54	1,98
3303	64	2	Ciamis	200-500	Tisuk	Hibiscus macrophyllus Roxb. ex I	Malvaceae	0,4	3,5	3,5	4,5	0,04	0,00	1,90	0,00	0,02	0,08
3304	64	2	Ciamis	200-500	Tisuk	Hibiscus macrophyllus Roxb. ex I	Malvaceae	0,4	3,8	3,5	5,5	0,04	0,00	2,68	0,00	0,03	0,12
3305	64	2	Ciamis	200-500	Tisuk	Hibiscus macrophyllus Roxb. ex I	Malvaceae	0,4	4,1	2	5	0,04	0,00	2,85	0,00	0,03	0,12
3306	64	2	Ciamis	200-500	Sengon	Paraserianthes falcataria (L.) I.C.	Leguminosae / Fabaceae	0,33	6,0	5	6	0,04	0,00	5,66	0,01	0,07	0,24
3307	64	2	Ciamis	200-500	Sengon	Paraserianthes falcataria (L.) I.C.	Leguminosae / Fabaceae	0,33	17,5	3,5	11,5	0,04	0,02	71,95	0,07	0,85	3,10
3308	64	2	Ciamis	200-500	Sengon	Paraserianthes falcataria (L.) I.C.	Leguminosae / Fabaceae	0,33	10,8	7	9	0,04	0,01	23,81	0,02	0,28	1,03
3309	64	2	Ciamis	200-500	Sengon	Paraserianthes falcataria (L.) I.C.	Leguminosae / Fabaceae	0,33	0,6	5	6,5	0,04	0,00	0,10	0,00	0,00	0,00
3310	64	2	Ciamis	200-500	Sengon	Paraserianthes falcataria (L.) I.C.	Leguminosae / Fabaceae	0,33	2,5	9	10,5	0,04	0,00	1,94	0,00	0,02	0,08
3311	64	2	Ciamis	200-500	Petai	Parkia speciosa Hassk.	Leguminosae / Fabaceae	0,45	9,2	6	8	0,04	0,01	21,22	0,02	0,25	0,92
3312	64	2	Ciamis	200-500	Sengon	Paraserianthes falcataria (L.) I.C.	Leguminosae / Fabaceae	0,33	17,2	10	15	0,04	0,02	88,74	0,09	1,04	3,83
3313	64	2	Ciamis	200-500	Sengon	Paraserianthes falcataria (L.) I.C.	Leguminosae / Fabaceae	0,33	7,3	7	10	0,04	0,00	12,82	0,01	0,15	0,55
3314	64	2	Ciamis	200-500	Caruy	Pterospermum javanicum Jungh.	Malvaceae	0,53	3,8	1	4,5	0,04	0,00	2,89	0,00	0,03	0,12
3315	64	2	Ciamis	200-500	Sengon	Paraserianthes falcataria (L.) I.C.	Leguminosae / Fabaceae	0,33	12,7	10	13	0,04	0,01	44,92	0,04	0,53	1,94
3316	64	2	Ciamis	200-500	Sengon	Paraserianthes falcataria (L.) I.C.	Leguminosae / Fabaceae	0,33	7,0	9	10,5	0,04	0,00	12,35	0,01	0,15	0,53
3317	65	2	Ciamis	200-500	Jengkol	Archidendron pauciflorum (Bentl	Leguminosae / Fabaceae	0,4	4,1	3,5	4,5	0,04	0,00	2,59	0,00	0,03	0,11
3318	65	2	Ciamis	200-500	Durian	Durio zibethinus L.	Malvaceae	0,57	6,7	5	6,5	0,04	0,00	12,06	0,01	0,14	0,52
3319	65	2	Ciamis	200-500	Dukuh	Lansium domesticum Corrêa	Meliaceae	0,85	47,7	4	16	0,04	0,18	1.455,59	1,46	17,10	62,77
3320	65	2	Ciamis	200-500	Mahoni	Swietenia macrophylla King	Meliaceae	0,61	4,5	1,7	3,7	0,04	0,00	3,64	0,00	0,04	0,16
3321	65	2	Ciamis	200-500	Jengkol	Archidendron pauciflorum (Bentl	Leguminosae / Fabaceae	0,4	20,4	9	15	0,04	0,03	144,49	0,14	1,70	6,23
3322	65	2	Ciamis	200-500	Durian	Durio zibethinus L.	Malvaceae	0,57	98,3	12	24	0,04	0,76	5.500,19	5,50	64,63	237,18
3323	65	2	Ciamis	200-500	Durian	Durio zibethinus L.	Malvaceae	0,57	46,8	11	18	0,04	0,17	1.083,57	1,08	12,73	46,73
3324	65	2	Ciamis	200-500	Tisuk	Hibiscus macrophyllus Roxb. ex I	Malvaceae	0,4	4,1	1,5	4,5	0,04	0,00	2,59	0,00	0,03	0,11
3325	65	2	Ciamis	200-500	Tisuk	Hibiscus macrophyllus Roxb. ex I	Malvaceae	0,4	5,7	4	6	0,04	0,00	6,11	0,01	0,07	0,26
3326	65	2	Ciamis	200-500	Tisuk	Hibiscus macrophyllus Roxb. ex I	Malvaceae	0,4	4,1	3,5	4,5	0,04	0,00	2,59	0,00	0,03	0,11
3327	65	2	Ciamis	200-500	Rambutan	Nephelium lappaceum L.	Sapindaceae	0,71	3,2	1	2,5	0,04	0,00	1,58	0,00	0,02	0,07
3328	65	2	Ciamis	200-500	Mahoni	Swietenia macrophylla King	Meliaceae	0,61	24,2	4	14	0,04	0,05	273,52	0,27	3,21	11,79
3329	65	2	Ciamis	200-500	Mahoni	Swietenia macrophylla King	Meliaceae	0,61	20,7	6	16	0,04	0,03	232,12	0,23	2,73	10,01
3330	65	2	Ciamis	200-500	Rambutan	Nephelium lappaceum L.	Sapindaceae	0,71	3,8	1	2,5	0,04	0,00	2,21	0,00	0,03	0,10
3331	65	2	Ciamis	200-500	Durian	Durio zibethinus L.	Malvaceae	0,57	10,2	4	11	0,04	0,01	42,25	0,04	0,50	1,82
3332	65	2	Ciamis	200-500	Durian	Durio zibethinus L.	Malvaceae	0,57	8,3	5	7	0,04	0,01	19,09	0,02	0,22	0,82
3333	65	2	Ciamis	200-500	Durian	Durio zibethinus L.	Malvaceae	0,57	29,3	9	16	0,04	0,07	412,22	0,41	4,84	17,78
3334	65	2	Ciamis	200-500	Tisuk	Hibiscus macrophyllus Roxb. ex I	Malvaceae	0,4	5,1	2,5	6,5	0,04	0,00	5,30	0,01	0,06	0,23
3335	65	2	Ciamis	200-500	Tisuk	Hibiscus macrophyllus Roxb. ex I	Malvaceae	0,4	2,5	2,5	3,5	0,04	0,00	0,84	0,00	0,01	0,04
3336	65	2	Ciamis	200-500	Rambutan	Nephelium lappaceum L.	Sapindaceae	0,71	2,5	2	3,5	0,04	0,00	1,43	0,00	0,02	0,06
3337	65	2	Ciamis	200-500	Tisuk	Hibiscus macrophyllus Roxb. ex I	Malvaceae	0,4	4,8	3	7	0,04	0,00	5,04	0,01	0,06	0,22
3338	65	2	Ciamis	200-500	Tisuk	Hibiscus macrophyllus Roxb. ex I	Malvaceae	0,4	5,7	3,5	6,5	0,04	0,00	6,57	0,01	0,08	0,28
3339	65	2	Ciamis	200-500	Tisuk	Hibiscus macrophyllus Roxb. ex I	Malvaceae	0,4	4,1	3,5	4,5	0,04	0,00	2,59	0,00	0,03	0,11
3340	65	2	Ciamis	200-500	Tisuk	Hibiscus macrophyllus Roxb. ex I	Malvaceae	0,4	7,0	2,8	7	0,04	0,00	10,16	0,01	0,12	0,44
3341	65	2	Ciamis	200-500	Durian	Durio zibethinus L.	Malvaceae	0,57	14,6	11	20	0,04	0,02	142,04	0,14	1,67	6,13
3342	65	2	Ciamis	200-500	Petai	Parkia speciosa Hassk.	Leguminosae / Fabaceae	0,45	8,3	4	7,5	0,04	0,01	16,38	0,02	0,19	0,71

3343	66	3	Ciamis	200-500	Gmelina	Gmelina arborea Roxb.	Lamiaceae / Verbenaceae	0,47	7,3	7	11	0,04	0,00	19,34	0,02	0,23	0,83
3344	66	3	Ciamis	200-500	Gmelina	Gmelina arborea Roxb.	Lamiaceae / Verbenaceae	0,47	10,5	9	14	0,04	0,01	46,72	0,05	0,55	2,01
3345	66	3	Ciamis	200-500	Tisuk	Hibiscus macrophyllus Roxb. ex l	Malvaceae	0,4	3,2	4	5	0,04	0,00	1,76	0,00	0,02	0,08
3346	66	3	Ciamis	200-500	Gmelina	Gmelina arborea Roxb.	Lamiaceae / Verbenaceae	0,47	20,7	16	20	0,04	0,03	224,27	0,22	2,64	9,67
3347	66	3	Ciamis	200-500	Gmelina	Gmelina arborea Roxb.	Lamiaceae / Verbenaceae	0,47	13,4	14	23	0,04	0,01	114,52	0,11	1,35	4,94
3348	66	3	Ciamis	200-500	Sengon	Paraserianthes falcataria (L.) I.C.	Leguminosae / Fabaceae	0,33	3,8	5	7	0,04	0,00	2,81	0,00	0,03	0,12
3349	66	3	Ciamis	200-500	Gmelina	Gmelina arborea Roxb.	Lamiaceae / Verbenaceae	0,47	9,5	10	16	0,04	0,01	44,34	0,04	0,52	1,91
3350	66	3	Ciamis	200-500	Gmelina	Gmelina arborea Roxb.	Lamiaceae / Verbenaceae	0,47	18,5	11	16	0,04	0,03	148,37	0,15	1,74	6,40
3351	66	3	Ciamis	200-500	Gmelina	Gmelina arborea Roxb.	Lamiaceae / Verbenaceae	0,47	19,1	13	19	0,04	0,03	184,79	0,18	2,17	7,97
3352	66	3	Ciamis	200-500	Gmelina	Gmelina arborea Roxb.	Lamiaceae / Verbenaceae	0,47	19,7	12	18	0,04	0,03	186,75	0,19	2,19	8,05
3353	66	3	Ciamis	200-500	Mahoni	Swietenia macrophylla King	Meliaceae	0,61	7,0	2	9	0,04	0,00	18,83	0,02	0,22	0,81
3354	66	3	Ciamis	200-500	Dukuh	Lansium domesticum Corrêa	Meliaceae	0,85	3,5	2,5	4,5	0,04	0,00	3,80	0,00	0,04	0,16
3355	66	3	Ciamis	200-500	Gmelina	Gmelina arborea Roxb.	Lamiaceae / Verbenaceae	0,47	8,0	10	13	0,04	0,00	26,25	0,03	0,31	1,13
3356	66	3	Ciamis	200-500	Gmelina	Gmelina arborea Roxb.	Lamiaceae / Verbenaceae	0,47	6,7	8	14	0,04	0,00	20,41	0,02	0,24	0,88
3357	66	3	Ciamis	200-500	Gmelina	Gmelina arborea Roxb.	Lamiaceae / Verbenaceae	0,47	7,6	9	15	0,04	0,00	27,77	0,03	0,33	1,20
3358	66	3	Ciamis	200-500	Gmelina	Gmelina arborea Roxb.	Lamiaceae / Verbenaceae	0,47	8,3	8	12	0,04	0,01	26,21	0,03	0,31	1,13
3359	66	3	Ciamis	200-500	Gmelina	Gmelina arborea Roxb.	Lamiaceae / Verbenaceae	0,47	19,1	11	16	0,04	0,03	157,87	0,16	1,86	6,81
3360	66	3	Ciamis	200-500	Gmelina	Gmelina arborea Roxb.	Lamiaceae / Verbenaceae	0,47	12,4	5	13	0,04	0,01	59,29	0,06	0,70	2,56
3361	66	3	Ciamis	200-500	Gmelina	Gmelina arborea Roxb.	Lamiaceae / Verbenaceae	0,47	7,0	6	11	0,04	0,00	17,82	0,02	0,21	0,77
3362	66	3	Ciamis	200-500	Gmelina	Gmelina arborea Roxb.	Lamiaceae / Verbenaceae	0,47	9,5	9	13	0,04	0,01	36,66	0,04	0,43	1,58
3363	66	3	Ciamis	200-500	Sengon	Paraserianthes falcataria (L.) I.C.	Leguminosae / Fabaceae	0,33	40,7	15	24	0,04	0,13	663,38	0,66	7,79	28,61
3364	66	3	Ciamis	200-500	Gmelina	Gmelina arborea Roxb.	Lamiaceae / Verbenaceae	0,47	29,0	9	19	0,04	0,07	396,34	0,40	4,66	17,09
3365	66	3	Ciamis	200-500	Gmelina	Gmelina arborea Roxb.	Lamiaceae / Verbenaceae	0,47	8,0	7	13	0,04	0,00	26,25	0,03	0,31	1,13
3366	66	3	Ciamis	200-500	Gmelina	Gmelina arborea Roxb.	Lamiaceae / Verbenaceae	0,47	23,2	10	16	0,04	0,04	226,12	0,23	2,66	9,75
3367	66	3	Ciamis	200-500	Tisuk	Hibiscus macrophyllus Roxb. ex l	Malvaceae	0,4	3,2	3,5	5	0,04	0,00	1,76	0,00	0,02	0,08
3368	66	3	Ciamis	200-500	Tisuk	Hibiscus macrophyllus Roxb. ex l	Malvaceae	0,4	3,8	3,5	5	0,04	0,00	2,46	0,00	0,03	0,11
3369	66	3	Ciamis	200-500	Tisuk	Hibiscus macrophyllus Roxb. ex l	Malvaceae	0,4	2,9	3	4	0,04	0,00	1,18	0,00	0,01	0,05
3370	66	3	Ciamis	200-500	Sengon	Paraserianthes falcataria (L.) I.C.	Leguminosae / Fabaceae	0,33	10,5	14	18	0,04	0,01	42,54	0,04	0,50	1,83
3371	66	3	Ciamis	200-500	Gmelina	Gmelina arborea Roxb.	Lamiaceae / Verbenaceae	0,47	8,3	11	14	0,04	0,01	30,19	0,03	0,35	1,30
3372	66	3	Ciamis	200-500	Sengon	Paraserianthes falcataria (L.) I.C.	Leguminosae / Fabaceae	0,33	22,3	17	24	0,04	0,04	219,57	0,22	2,58	9,47
3373	66	3	Ciamis	200-500	Tisuk	Hibiscus macrophyllus Roxb. ex l	Malvaceae	0,4	2,9	3,5	4,5	0,04	0,00	1,32	0,00	0,02	0,06
3374	66	3	Ciamis	200-500	Gmelina	Gmelina arborea Roxb.	Lamiaceae / Verbenaceae	0,47	24,5	15	21	0,04	0,05	319,86	0,32	3,76	13,79
3375	66	3	Ciamis	200-500	Gmelina	Gmelina arborea Roxb.	Lamiaceae / Verbenaceae	0,47	8,6	9	13	0,04	0,01	30,23	0,03	0,36	1,30
3376	66	3	Ciamis	200-500	Gmelina	Gmelina arborea Roxb.	Lamiaceae / Verbenaceae	0,47	17,8	16	22	0,04	0,02	186,25	0,19	2,19	8,03
3377	66	3	Ciamis	200-500	Tisuk	Hibiscus macrophyllus Roxb. ex l	Malvaceae	0,4	4,8	4	6	0,04	0,00	4,38	0,00	0,05	0,19
3378	66	3	Ciamis	200-500	Gmelina	Gmelina arborea Roxb.	Lamiaceae / Verbenaceae	0,47	12,7	10	17	0,04	0,01	79,40	0,08	0,93	3,42
3379	66	3	Ciamis	200-500	Gmelina	Gmelina arborea Roxb.	Lamiaceae / Verbenaceae	0,47	18,1	13	22	0,04	0,03	192,39	0,19	2,26	8,30
3380	66	3	Ciamis	200-500	Gmelina	Gmelina arborea Roxb.	Lamiaceae / Verbenaceae	0,47	15,3	14	20	0,04	0,02	128,69	0,13	1,51	5,55
3381	66	3	Ciamis	200-500	Tisuk	Hibiscus macrophyllus Roxb. ex l	Malvaceae	0,4	3,8	6	7,5	0,04	0,00	3,57	0,00	0,04	0,15
3382	66	3	Ciamis	200-500	Gmelina	Gmelina arborea Roxb.	Lamiaceae / Verbenaceae	0,47	11,1	10	16	0,04	0,01	58,81	0,06	0,69	2,54
3383	66	3	Ciamis	200-500	Sengon	Paraserianthes falcataria (L.) I.C.	Leguminosae / Fabaceae	0,33	35,3	15	25	0,04	0,10	530,42	0,53	6,23	22,87
3384	66	3	Ciamis	200-500	Gmelina	Gmelina arborea Roxb.	Lamiaceae / Verbenaceae	0,47	15,9	12	18	0,04	0,02	125,92	0,13	1,48	5,43
3385	66	3	Ciamis	200-500	Petai	Parkia speciosa Hassk.	Leguminosae / Fabaceae	0,45	2,9	3	4	0,04	0,00	1,32	0,00	0,02	0,06
3386	66	3	Ciamis	200-500	Gmelina	Gmelina arborea Roxb.	Lamiaceae / Verbenaceae	0,47	11,1	9	16	0,04	0,01	58,81	0,06	0,69	2,54
3387	66	3	Ciamis	200-500	Gmelina	Gmelina arborea Roxb.	Lamiaceae / Verbenaceae	0,47	7,3	7	10	0,04	0,00	17,72	0,02	0,21	0,76
3388	66	3	Ciamis	200-500	Kokosan	Lansium domesticum var. aquae	Meliaceae	0,7	3,2	4	5,5	0,04	0,00	3,21	0,00	0,04	0,14
3389	66	3	Ciamis	200-500	Gmelina	Gmelina arborea Roxb.	Lamiaceae / Verbenaceae	0,47	7,3	7	11	0,04	0,00	19,34	0,02	0,23	0,83
3390	66	3	Ciamis	200-500	Gmelina	Gmelina arborea Roxb.	Lamiaceae / Verbenaceae	0,47	6,0	4	7	0,04	0,00	9,01	0,01	0,11	0,39
3391	66	3	Ciamis	200-500	Gmelina	Gmelina arborea Roxb.	Lamiaceae / Verbenaceae	0,47	11,1	4	8	0,04	0,01	31,17	0,03	0,37	1,34
3392	66	3	Ciamis	200-500	Sengon	Paraserianthes falcataria (L.) I.C.	Leguminosae / Fabaceae	0,33	24,2	15	21	0,04	0,05	225,88	0,23	2,65	9,74
3393	66	3	Ciamis	200-500	Tisuk	Hibiscus macrophyllus Roxb. ex l	Malvaceae	0,4	4,1	3,5	5	0,04	0,00	2,85	0,00	0,03	0,12
3394	66	3	Ciamis	200-500	Sengon	Paraserianthes falcataria (L.) I.C.	Leguminosae / Fabaceae	0,33	9,2	6	11	0,04	0,01	21,38	0,02	0,25	0,92
3395	66	3	Ciamis	200-500	Gmelina	Gmelina arborea Roxb.	Lamiaceae / Verbenaceae	0,47	8,9	10	15	0,04	0,01	36,83	0,04	0,43	1,59
3396	66	3	Ciamis	200-500	Gmelina	Gmelina arborea Roxb.	Lamiaceae / Verbenaceae	0,47	19,7	15	18	0,04	0,03	186,75	0,19	2,19	8,05
3397	66	3	Ciamis	200-500	Gmelina	Gmelina arborea Roxb.	Lamiaceae / Verbenaceae	0,47	5,7	6	10	0,04	0,00	11,31	0,01	0,13	0,49
3398	66	3	Ciamis	200-500	Gmelina	Gmelina arborea Roxb.	Lamiaceae / Verbenaceae	0,47	13,7	13	16	0,04	0,01	85,75	0,09	1,01	3,70
3399	66	3	Ciamis	200-500	Mahoni	Swietenia macrophylla King	Meliaceae	0,61	3,2	3	4	0,04	0,00	2,11	0,00	0,02	0,09
3400	66	3	Ciamis	200-500	Pongporang	Oroxylum indicum (L.) Kurz	Bignoniaceae	0,32	6,7	9	10	0,04	0,00	10,55	0,01	0,12	0,45
3401	66	3	Ciamis	200-500	Mahoni	Swietenia macrophylla King	Meliaceae	0,61	3,2	3,5	4,5	0,04	0,00	2,35	0,00	0,03	0,10
3402	66	3	Ciamis	200-500	Sengon	Paraserianthes falcataria (L.) I.C.	Leguminosae / Fabaceae	0,33	22,9	14	21	0,04	0,04	204,58	0,20	2,40	8,82
3403	66	3	Ciamis	200-500	Gmelina	Gmelina arborea Roxb.	Lamiaceae / Verbenaceae	0,47	22,3	14	21	0,04	0,04	268,62	0,27	3,16	11,58
3404	66	3	Ciamis	200-500	Gmelina	Gmelina arborea Roxb.	Lamiaceae / Verbenaceae	0,47	5,7	6	8	0,04	0,00	9,22	0,01	0,11	0,40
3405	66	3	Ciamis	200-500	Gmelina	Gmelina arborea Roxb.	Lamiaceae / Verbenaceae	0,47	7,3	6	11	0,04	0,00	19,34	0,02	0,23	0,83
3406	66	3	Ciamis	200-500	Dukuh	Lansium domesticum Corrêa	Meliaceae	0,85	32,8	5	14	0,04	0,08	646,90	0,65	7,60	27,90
3407	66	3	Ciamis	200-500	Durian	Durio zibethinus L.	Malvaceae	0,57	3,2	2	4	0,04	0,00	1,99	0,00	0,02	0,09
3408	66	3	Ciamis	200-500	Sp8	Sp8	0	0,5	3,2	1	4	0,04	0,00	1,76	0,00	0,02	0,08
3409	66	3	Ciamis	200-500	Kokosan	Lansium domesticum var. aquae	Meliaceae	0,7	2,9	2,5	3,5	0,04	0,00	1,75	0,00	0,02	0,08
3410	66	3	Ciamis	200-500	Kelapa	Cocos nucifera L.	Arecaceae	0,5	3,2	13	17,5	0,04	0,00	139,25	0,14	1,64	6,00
3411	66	3	Ciamis	200-500	Jengkol	Archidendron pauciflorum (Bentl	Leguminosae / Fabaceae	0,4	6,4	7	8	0,04	0,00	9,65	0,01	0,11	0,42
3412	66	3	Ciamis	200-500	Dukuh	Lansium domesticum Corrêa	Meliaceae	0,85	17,2	4	10	0,04	0,02	145,62	0,15	1,71	6,28
3413	66	3	Ciamis	200-500	Rambutan	Nephelium lappaceum L.	Sapindaceae	0,71	5,4	5	9	0,04	0,00	13,49	0,01	0,16	0,58
3414	66	3	Ciamis	200-500	Nangka	Artocarpus heterophyllus Lam.	Moraceae	0,61	1,9	3	6	0,04	0,00	1,20	0,00	0,01	0,05
3415	66	3	Ciamis	200-500	Jati	Tectona grandis L.f.	Lamiaceae / Verbenaceae	0,7	29,0	5	14	0,04	0,07	431,56	0,43	5,07	18,61
3416	66	3	Ciamis	200-500	Durian	Durio zibethinus L.	Malvaceae	0,57	3,8	3,5	5	0,04	0,00	3,40	0,00	0,04	0,15
3417	66	3	Ciamis	200-500	Kokosan	Lansium domesticum var. aquae	Meliaceae	0,7	5,1	5	7,5	0,04	0,00	10,09	0,01	0,12	0,43
3418	66	3	Ciamis	200-500	Mahoni	Swietenia macrophylla King	Meliaceae	0,61	10,2	3	9	0,04	0,01	37,41	0,04	0,44	1,61
3419	66	3	Ciamis	200-500	Mahoni	Swietenia macrophylla King	Meliaceae	0,61	35,0	9	16	0,04	0,10	608,54	0,61	7,15	26,24
3420	66	3	Ciamis	200-500	Jengkol	Archidendron pauciflorum (Bentl	Leguminosae / Fabaceae	0,4	11,8	5	7	0,04	0,01	26,34	0,03	0,31	1,14
3421	66	3	Ciamis	200-500	Durian	Durio zibethinus L.	Malvaceae	0,57	12,4	7	13	0,04	0,01	70,75	0,07	0,83	3,05
3422	66	3	Ciamis	200-500	Melinjo	Gnetum gnemon L.	Gnetaceae	0,76	5,1	1	3	0,04	0,00	4,70	0,00	0,06	0,20
3423	66	3	Ciamis	200-500	Kelapa	Cocos nucifera L.	Arecaceae	0,5	22,0	1	4	0,04	0,04	35,30	0,04	0,41	1,52
3424	66	3	Ciamis	200-500	Kokosan	Lansium domesticum var. aquae	Meliaceae	0,7	9,5	3,5	5,5	0,04	0,01	24,02	0,02	0,28	1,04
3425	66	3	Ciamis	200-500	Dukuh	Lansium domesticum Corrêa	Meliaceae	0,85	24,2	4	11	0,04	0,05	297,19	0,30	3,49	12,82
3426	66	3	Ciamis	200-500	Dukuh	Lansium domesticum Corrêa	Meliaceae	0,85	15,6	4	10	0,04	0,02	121,87	0,12	1,43	5,26
3427	66	3	Ciamis	200-500	Kokosan	Lansium domesticum var. aquae	Meliaceae	0,7	9,5	3	10	0,04	0,01	41,53	0,04	0,49	1,79
3428	66	3	Ciamis	200-500	Dukuh	Lansium domesticum Corrêa	Meliaceae	0,85	27,4	3,5	11,5	0,04	0,06	388,21	0,39	4,56	16,74
3429	66	3	Ciamis	200-500	Melinjo	Gnetum gnemon L.	Gnetaceae	0,76	6,4	2	9	0,04	0,00	19,34	0,02	0,23	0,83
3430	67	3	Ciamis	200-500	Dukuh	Lansium domesticum Corrêa	Meliaceae	0,85	24,8	4	11	0,04	0,05	311,67	0,31	3,66	13,44
3431	67	3	Ciamis	200-500	Mahoni	Swietenia macrophylla King	Meliaceae	0,61	42,6	10	19	0,04	0,14	1.022,53	1,02	12,01	44,09
3432	67	3	Ciamis	200-500	Mahoni	Swietenia macrophylla King	Meliaceae	0,61	27,7	13	22	0,04	0,06	530,08	0,53	6,23	22,86
3433	67	3	Ciamis	200-500	Mahoni	Swietenia macrophylla King	Meliaceae	0,61	63,3	15	27	0,04	0,32	2.911,45	2,91	34,21	125,55
3434	67	3	Ciamis	200-500	Nangka	Artocarpus heterophyllus Lam.	Moraceae	0,61	21,0	13	18	0,04	0,03	265,90	0,27	3,12	11,47
3435	67	3	Ciamis	200-500	Melinjo	Gnetum gnemon L.	Gnetaceae	0,76	27,7	4,5	7,5	0,04	0,06	241,94	0,24	2,84	10,43
3436	67	3	Ciamis	200-500	Melinjo	Gnetum gnemon L.	Gnetaceae	0,76	17,8	10	14	0,04	0,02	191,20	0,19	2,25	8,24
3437	67	3	Ciamis	200-500	Dukuh	Lansium domesticum Corrêa	Meliaceae	0,85	41,4	4	12	0,04	0,13	860,48	0,86	10,11	37,11
3438	67	3	Ciamis	200-500	Nangka	Artocarpus heterophyllus Lam.	Moraceae	0,61	19,7	5	12	0,04	0,03	163,56	0,16	1,92	7,05
3439	67	3	Ciamis	200-500	Durian	Durio zibethinus L.	Malvaceae	0,57	5,7	3,5	6	0,04	0,00	8,45	0,01	0,10	0,36
3440	67	3	Ciamis	200-500	Kelapa	Cocos nucifera L.	Arecaceae	0,5	31,2	15	18	0,04	0,08	143,10	0,14	1,68	6,17
3441	67	3	Ciamis	200-500	Tisuk	Hibiscus macrophyllus Roxb. ex l	Malvaceae	0,4	27,7	13	18	0,04	0,06	299,66	0,30	3,52	12,92
3442	67	3	Ciamis	200-500	Manggis	Mangostana garcinia Gaertn.	Clusiaceae	0,81	4,1	1	4	0,04	0,00	4,43	0,00	0,05	0,19
3443	67	3	Ciamis	200-500	Dukuh	Lansium domesticum Corrêa	Meliaceae	0,85	44,5	4,5	15	0,04	0,16	1.209,13	1,21	14,21	52,14
3444	67	3	Ciamis	200-500	Tisuk	Hibiscus macrophyllus Roxb. ex l	Malvaceae	0,4	2,9	2,5	3,5	0,04	0,00	1,05	0,00	0,01	0,05
3445	67	3	Ciamis	200-500	Durian	Durio zibethinus L.	Malvaceae	0,57	2,9	1,8	4	0,04	0,00	1,64	0,00	0,02	0,07
3446	67	3	Ciamis	200-500	Durian	Durio zibethinus L.	Malvaceae	0,57	4,8	1,8	5	0,04	0,00	5,12	0,01	0,06	0,22
3447	67	3	Ciamis	200-500	Tisuk	Hibiscus macrophyllus Roxb. ex l	Malvaceae	0,4	8,3	5	7,5	0,04	0,01	14,70	0,01	0,17	0,63
3448	67	3	Ciamis	200-500	Petai	Parkia speciosa Hassk.	Leguminosae / Fabaceae	0,45	3,8	5	6	0,04	0,00	3,24	0,00	0,04	0,14
3449	67	3	Ciamis	200-500	Durian	Durio zibethinus L.	Malvaceae	0,57	8,9	6	11	0,04	0,01	33,08	0,03	0,39	1,43
3450	67	3	Ciamis	200-500	Dukuh	Lansium domesticum Corrêa	Meliaceae	0,85	4,8	2	3,5	0,04	0,00	5,33	0,01	0,06	0,23
3451	67	3	Ciamis	200-500	Tisuk	Hibiscus macrophyllus Roxb. ex l	Malvaceae	0,4	4,8	2,5	4	0,04	0,00	3,02	0,00	0,04	0,13
3452	67	3	Ciamis	200-500	Alpukat	Persea americana Mill.	Lauraceae	0,54	3,5	1,3	2,5	0,04	0,00	1,46	0,00	0,02	0,06
3453	67	3	Ciamis	200-500	Tisuk	Hibiscus macrophyllus Roxb. ex l	Malvaceae	0,4	7,0	4	5,5	0,04	0,00	8,15	0,01	0,10	0,35
3454	67	3	Ciamis	200-500	Tisuk	Hibiscus macrophyllus Roxb. ex l	Malvaceae	0,4	8,6	2	7	0,04	0,01	14,79	0,01	0,17	0,64
3455	67	3	Ciamis	200-500	Dukuh	Lansium domesticum Corrêa	Meliaceae	0,85	3,2	1	2,5	0,04	0,00	1,86	0,00	0,02	0,08
3456	67	3	Ciamis	200-500	Tisuk	Hibiscus macrophyllus Roxb. ex l	Malvaceae	0,4	2,9	1,8	2,5	0,04	0,00	0,77	0,00	0,01	0,03
3457	67	3	Ciamis	200-500	Tisuk	Hibiscus macrophyllus Roxb. ex l	Malvaceae	0,4	7,3	3,5	7	0,04	0,00	11,03	0,01	0,13	0,48
3458	68	2	Ciamis	200-500	Dukuh	Lansium domesticum Corrêa	Meliaceae	0,85	13,7	4	11	0,04	0,01	104,69	0,10	1,23	4,51
3459	68	2	Ciamis	200-500	Dukuh	Lansium domesticum Corrêa	Meliaceae	0,85	41,0	4,5	6,5	0,04	0,13	483,83	0,48	5,69	20,86
3460	68	2	Ciamis	200-500	Durian	Durio zibethinus L.	Malvaceae	0,57	33,7	16	23	0,04	0,09	745,08	0,75	8,75	32,13
3461	68	2	Ciamis	200-500	Durian	Durio zibethinus L.	Malvaceae	0,57	3,2	2	3	0,04	0,00	1,53	0,00	0,02	0,07
3462	68	2	Ciamis	200-500	Durian	Durio zibethinus L.	Malvaceae	0,57	41,4	16	24	0,04	0,13	1.125,95	1,13	13,23	48,55
3463	68	2	Ciamis	200-500	Mahoni	Swietenia macrophylla King	Meliaceae	0,61	13,4	4	14	0,04	0,01	92,28	0,09	1,08	3,98
3464	68	2	Ciamis	200-500	Tisuk	Hibiscus macrophyllus Roxb. ex l	Malvaceae	0,4	6,7	5	10	0,04	0,00	12,94	0,01	0,15	0,56
3465	68	2	Ciamis	200-500	Dukuh	Lansium domesticum Corrêa	Meliaceae	0,85	20,4	4	14	0,04	0,03	270,55	0,27	3,18	11,67
3466	68	2	Ciamis	200-500	Durian	Durio zibethinus L.	Malvaceae	0,57	3,8	3	4,5	0,04	0,00	3,09	0,00	0,04	0,13

3467	68	2	Ciamis	200-500	Durian	Durio zibethinus L.	Malvaceae	0,57	20,0	5	6,5	0,04	0,03	90,27	0,09	1,06	3,89
3468	68	2	Ciamis	200-500	Durian	Durio zibethinus L.	Malvaceae	0,57	5,4	3	4,5	0,04	0,00	5,85	0,01	0,07	0,25
3469	68	2	Ciamis	200-500	Nangka	Artocarpus heterophyllus Lam.	Moraceae	0,61	30,9	5	14	0,04	0,07	427,66	0,43	5,03	18,44
3470	68	2	Ciamis	200-500	Dukuh	Lansium domesticum Corrêa	Meliaceae	0,85	37,9	3,5	16,5	0,04	0,11	979,68	0,98	11,51	42,25
3471	68	2	Ciamis	200-500	Manggis	Mangostana garcinia Gaertn.	Clusiaceae	0,81	7,0	2,5	6	0,04	0,00	16,84	0,02	0,20	0,73
3472	68	2	Ciamis	200-500	Dukuh	Lansium domesticum Corrêa	Meliaceae	0,85	39,8	5,5	14,5	0,04	0,12	952,40	0,95	11,19	41,07
3473	68	2	Ciamis	200-500	Durian	Durio zibethinus L.	Malvaceae	0,57	8,6	6	12	0,04	0,01	33,52	0,03	0,39	1,45
3474	68	2	Ciamis	200-500	Dukuh	Lansium domesticum Corrêa	Meliaceae	0,85	29,0	8	16	0,04	0,07	582,65	0,58	6,85	25,13
3475	68	2	Ciamis	200-500	Kokosan	Lansium domesticum var. aquae	Meliaceae	0,7	24,8	10	14	0,04	0,05	325,39	0,33	3,82	14,03
3476	68	2	Ciamis	200-500	Dukuh	Lansium domesticum Corrêa	Meliaceae	0,85	25,5	10	15	0,04	0,05	433,74	0,43	5,10	18,70
3477	68	2	Ciamis	200-500	Dukuh	Lansium domesticum Corrêa	Meliaceae	0,85	32,8	5	15	0,04	0,08	689,10	0,69	8,10	29,72
3478	68	2	Ciamis	200-500	Dukuh	Lansium domesticum Corrêa	Meliaceae	0,85	26,7	5	14	0,04	0,06	445,25	0,45	5,23	19,20
3479	69	1	Ciamis	200-500	Jati	Tectona grandis L.f.	Lamiaceae / Verbenaceae	0,7	15,3	10	18	0,04	0,02	168,30	0,17	1,98	7,26
3480	69	1	Ciamis	200-500	Jati	Tectona grandis L.f.	Lamiaceae / Verbenaceae	0,7	12,4	8	17	0,04	0,01	109,18	0,11	1,28	4,71
3481	69	1	Ciamis	200-500	Sengon	Paraserianthes falcataria (L.) I.C.	Leguminosae / Fabaceae	0,33	12,7	18	21	0,04	0,01	69,70	0,07	0,82	3,01
3482	69	1	Ciamis	200-500	Jati	Tectona grandis L.f.	Lamiaceae / Verbenaceae	0,7	14,0	11	18	0,04	0,02	143,50	0,14	1,69	6,19
3483	69	1	Ciamis	200-500	Sengon	Paraserianthes falcataria (L.) I.C.	Leguminosae / Fabaceae	0,33	15,0	15	18	0,04	0,02	81,32	0,08	0,96	3,51
3484	69	1	Ciamis	200-500	Jati	Tectona grandis L.f.	Lamiaceae / Verbenaceae	0,7	12,7	11	19	0,04	0,01	126,63	0,13	1,49	5,46
3485	69	1	Ciamis	200-500	Jati	Tectona grandis L.f.	Lamiaceae / Verbenaceae	0,7	10,2	11	16	0,04	0,01	71,88	0,07	0,84	3,10
3486	69	1	Ciamis	200-500	Jati	Tectona grandis L.f.	Lamiaceae / Verbenaceae	0,7	9,9	4	13	0,04	0,01	56,08	0,06	0,66	2,42
3487	69	1	Ciamis	200-500	Sengon	Paraserianthes falcataria (L.) I.C.	Leguminosae / Fabaceae	0,33	8,9	12	15	0,04	0,01	26,64	0,03	0,31	1,15
3488	69	1	Ciamis	200-500	Jati	Tectona grandis L.f.	Lamiaceae / Verbenaceae	0,7	16,2	11	17	0,04	0,02	178,48	0,18	2,10	7,70
3489	69	1	Ciamis	200-500	Sengon	Paraserianthes falcataria (L.) I.C.	Leguminosae / Fabaceae	0,33	6,7	9	10	0,04	0,00	10,85	0,01	0,13	0,47
3490	69	1	Ciamis	200-500	Sengon	Paraserianthes falcataria (L.) I.C.	Leguminosae / Fabaceae	0,33	15,6	16	19	0,04	0,02	92,23	0,09	1,08	3,98
3491	69	1	Ciamis	200-500	Sengon	Paraserianthes falcataria (L.) I.C.	Leguminosae / Fabaceae	0,33	11,5	14	17	0,04	0,01	47,35	0,05	0,56	2,04
3492	69	1	Ciamis	200-500	Jati	Tectona grandis L.f.	Lamiaceae / Verbenaceae	0,7	8,6	4	9	0,04	0,01	31,09	0,03	0,37	1,34
3493	69	1	Ciamis	200-500	Sengon	Paraserianthes falcataria (L.) I.C.	Leguminosae / Fabaceae	0,33	9,5	14	16	0,04	0,01	32,07	0,03	0,38	1,38
3494	69	1	Ciamis	200-500	Sengon	Paraserianthes falcataria (L.) I.C.	Leguminosae / Fabaceae	0,33	16,9	16	19	0,04	0,02	106,49	0,11	1,25	4,59
3495	69	1	Ciamis	200-500	Jati	Tectona grandis L.f.	Lamiaceae / Verbenaceae	0,7	4,8	2	6	0,04	0,00	7,30	0,01	0,09	0,32
3496	69	1	Ciamis	200-500	Jati	Tectona grandis L.f.	Lamiaceae / Verbenaceae	0,7	15,3	8	15	0,04	0,02	142,42	0,14	1,67	6,14
3497	69	1	Ciamis	200-500	Sengon	Paraserianthes falcataria (L.) I.C.	Leguminosae / Fabaceae	0,33	8,0	11	13	0,04	0,00	18,99	0,02	0,22	0,82
3498	69	1	Ciamis	200-500	Jati	Tectona grandis L.f.	Lamiaceae / Verbenaceae	0,7	9,5	7	10	0,04	0,01	41,53	0,04	0,49	1,79
3499	69	1	Ciamis	200-500	Sengon	Paraserianthes falcataria (L.) I.C.	Leguminosae / Fabaceae	0,33	8,6	13	14	0,04	0,01	23,40	0,02	0,27	1,01
3500	69	1	Ciamis	200-500	Jati	Tectona grandis L.f.	Lamiaceae / Verbenaceae	0,7	3,8	2	5,5	0,04	0,00	4,48	0,00	0,05	0,19
3501	69	1	Ciamis	200-500	Jati	Tectona grandis L.f.	Lamiaceae / Verbenaceae	0,7	8,3	2,5	6,5	0,04	0,01	21,53	0,02	0,25	0,93
3502	69	1	Ciamis	200-500	Jati	Tectona grandis L.f.	Lamiaceae / Verbenaceae	0,7	16,9	5,5	14,5	0,04	0,02	165,54	0,17	1,95	7,14
3503	69	1	Ciamis	200-500	Jati	Tectona grandis L.f.	Lamiaceae / Verbenaceae	0,7	15,9	5	15	0,04	0,02	153,48	0,15	1,80	6,62
3504	69	1	Ciamis	200-500	Jati	Tectona grandis L.f.	Lamiaceae / Verbenaceae	0,7	14,6	5	13	0,04	0,02	115,55	0,12	1,36	4,98
3505	69	1	Ciamis	200-500	Sengon	Paraserianthes falcataria (L.) I.C.	Leguminosae / Fabaceae	0,33	10,5	14	7	0,04	0,01	17,91	0,02	0,21	0,77
3506	69	1	Ciamis	200-500	Jati	Tectona grandis L.f.	Lamiaceae / Verbenaceae	0,7	9,9	3	11	0,04	0,01	48,12	0,05	0,57	2,08
3507	69	1	Ciamis	200-500	Sengon	Paraserianthes falcataria (L.) I.C.	Leguminosae / Fabaceae	0,33	8,3	12	14	0,04	0,01	21,84	0,02	0,26	0,94
3508	69	1	Ciamis	200-500	Jati	Tectona grandis L.f.	Lamiaceae / Verbenaceae	0,7	9,2	4	11	0,04	0,01	42,59	0,04	0,50	1,84
3509	69	1	Ciamis	200-500	Jati	Tectona grandis L.f.	Lamiaceae / Verbenaceae	0,7	9,5	7	17	0,04	0,01	67,52	0,07	0,79	2,91
3510	69	1	Ciamis	200-500	Jati	Tectona grandis L.f.	Lamiaceae / Verbenaceae	0,7	9,2	13	15	0,04	0,01	56,58	0,06	0,66	2,44
3511	69	1	Ciamis	200-500	Sengon	Paraserianthes falcataria (L.) I.C.	Leguminosae / Fabaceae	0,33	9,9	2,5	7,5	0,04	0,01	17,01	0,02	0,20	0,73
3512	69	1	Ciamis	200-500	Jati	Tectona grandis L.f.	Lamiaceae / Verbenaceae	0,7	6,7	13	16	0,04	0,00	33,23	0,03	0,39	1,43
3513	69	1	Ciamis	200-500	Sengon	Paraserianthes falcataria (L.) I.C.	Leguminosae / Fabaceae	0,33	13,7	6	10	0,04	0,01	40,33	0,04	0,47	1,74
3514	69	1	Ciamis	200-500	Sengon	Paraserianthes falcataria (L.) I.C.	Leguminosae / Fabaceae	0,33	16,2	6	12	0,04	0,02	65,14	0,07	0,77	2,81
3515	69	1	Ciamis	200-500	Sengon	Paraserianthes falcataria (L.) I.C.	Leguminosae / Fabaceae	0,33	8,6	3	4,5	0,04	0,01	8,27	0,01	0,10	0,36
3516	70	2	Ciamis	200-500	Tisuk	Hibiscus macrophyllus Roxb. ex I	Malvaceae	0,4	3,8	9	10	0,01	0,00	4,64	0,00	0,22	0,80
3517	70	2	Ciamis	200-500	Tisuk	Hibiscus macrophyllus Roxb. ex I	Malvaceae	0,4	3,2	4	6	0,01	0,00	2,08	0,00	0,10	0,36
3518	70	2	Ciamis	200-500	Tisuk	Hibiscus macrophyllus Roxb. ex I	Malvaceae	0,4	4,5	6	7,5	0,01	0,00	4,73	0,00	0,22	0,82
3519	70	2	Ciamis	200-500	Tisuk	Hibiscus macrophyllus Roxb. ex I	Malvaceae	0,4	4,1	6	7,5	0,01	0,00	4,13	0,00	0,19	0,71
3520	70	2	Ciamis	200-500	Tisuk	Hibiscus macrophyllus Roxb. ex I	Malvaceae	0,4	5,7	8	9	0,01	0,00	8,86	0,01	0,42	1,53
3521	70	2	Ciamis	200-500	Tisuk	Hibiscus macrophyllus Roxb. ex I	Malvaceae	0,4	3,2	6,5	7,5	0,01	0,00	2,55	0,00	0,12	0,44
3522	70	2	Ciamis	200-500	Tisuk	Hibiscus macrophyllus Roxb. ex I	Malvaceae	0,4	6,4	8	9,5	0,01	0,00	11,29	0,01	0,53	1,95
3523	70	2	Ciamis	200-500	Tisuk	Hibiscus macrophyllus Roxb. ex I	Malvaceae	0,4	2,9	4	4,5	0,01	0,00	1,32	0,00	0,06	0,23
3524	70	2	Ciamis	200-500	Tisuk	Hibiscus macrophyllus Roxb. ex I	Malvaceae	0,4	6,4	8	10	0,01	0,00	11,83	0,01	0,56	2,04
3525	70	2	Ciamis	200-500	Tisuk	Hibiscus macrophyllus Roxb. ex I	Malvaceae	0,4	4,5	9	10	0,01	0,00	6,16	0,01	0,29	1,06
3526	70	2	Ciamis	200-500	Tisuk	Hibiscus macrophyllus Roxb. ex I	Malvaceae	0,4	4,1	8	9	0,01	0,00	4,88	0,00	0,23	0,84
3527	70	2	Ciamis	200-500	Tisuk	Hibiscus macrophyllus Roxb. ex I	Malvaceae	0,4	4,8	9	10,5	0,01	0,00	7,30	0,01	0,34	1,26
3528	70	2	Ciamis	200-500	Tisuk	Hibiscus macrophyllus Roxb. ex I	Malvaceae	0,4	3,2	8,5	9	0,01	0,00	3,02	0,00	0,14	0,52
3529	70	2	Ciamis	200-500	Tisuk	Hibiscus macrophyllus Roxb. ex I	Malvaceae	0,4	4,1	9	10	0,01	0,00	5,37	0,01	0,25	0,93
3530	70	2	Ciamis	200-500	Tisuk	Hibiscus macrophyllus Roxb. ex I	Malvaceae	0,4	6,7	10	11,5	0,01	0,00	14,71	0,01	0,69	2,54
3531	70	2	Ciamis	200-500	Tisuk	Hibiscus macrophyllus Roxb. ex I	Malvaceae	0,4	7,0	9	10,5	0,01	0,00	14,73	0,01	0,69	2,54
3532	70	2	Ciamis	200-500	Tisuk	Hibiscus macrophyllus Roxb. ex I	Malvaceae	0,4	4,8	7	8,5	0,01	0,00	6,02	0,01	0,28	1,04
3533	70	2	Ciamis	200-500	Tisuk	Hibiscus macrophyllus Roxb. ex I	Malvaceae	0,4	8,0	10	12	0,01	0,00	21,05	0,02	0,99	3,63
3534	70	2	Ciamis	200-500	Tisuk	Hibiscus macrophyllus Roxb. ex I	Malvaceae	0,4	3,8	6	7,5	0,01	0,00	3,57	0,00	0,17	0,62
3535	70	2	Ciamis	200-500	Tisuk	Hibiscus macrophyllus Roxb. ex I	Malvaceae	0,4	6,0	10	12	0,01	0,00	12,73	0,01	0,60	2,20
3536	70	2	Ciamis	200-500	Tisuk	Hibiscus macrophyllus Roxb. ex I	Malvaceae	0,4	3,5	5,5	6,3	0,01	0,00	2,59	0,00	0,12	0,45
3537	70	2	Ciamis	200-500	Tisuk	Hibiscus macrophyllus Roxb. ex I	Malvaceae	0,4	5,7	9	10,5	0,01	0,00	10,20	0,01	0,48	1,76
3538	70	2	Ciamis	200-500	Tisuk	Hibiscus macrophyllus Roxb. ex I	Malvaceae	0,4	6,7	9	10,5	0,01	0,00	13,53	0,01	0,64	2,33
3539	70	2	Ciamis	200-500	Tisuk	Hibiscus macrophyllus Roxb. ex I	Malvaceae	0,4	6,0	8	9,5	0,01	0,00	10,28	0,01	0,48	1,77
3540	70	2	Ciamis	200-500	Tisuk	Hibiscus macrophyllus Roxb. ex I	Malvaceae	0,4	4,8	8	9,5	0,01	0,00	6,67	0,01	0,31	1,15
3541	70	2	Ciamis	200-500	Tisuk	Hibiscus macrophyllus Roxb. ex I	Malvaceae	0,4	4,5	8	9	0,01	0,00	5,59	0,01	0,26	0,96
3542	70	2	Ciamis	200-500	Tisuk	Hibiscus macrophyllus Roxb. ex I	Malvaceae	0,4	4,1	8	9	0,01	0,00	4,88	0,00	0,23	0,84
3543	70	2	Ciamis	200-500	Tisuk	Hibiscus macrophyllus Roxb. ex I	Malvaceae	0,4	5,4	10	11	0,01	0,00	9,59	0,01	0,45	1,65
3544	70	2	Ciamis	200-500	Tisuk	Hibiscus macrophyllus Roxb. ex I	Malvaceae	0,4	2,9	4,5	6	0,01	0,00	1,72	0,00	0,08	0,30
3545	70	2	Ciamis	200-500	Tisuk	Hibiscus macrophyllus Roxb. ex I	Malvaceae	0,4	5,1	7	9	0,01	0,00	7,14	0,01	0,34	1,23
3546	70	2	Ciamis	200-500	Tisuk	Hibiscus macrophyllus Roxb. ex I	Malvaceae	0,4	3,2	4,5	5,5	0,01	0,00	1,92	0,00	0,09	0,33
3547	70	2	Ciamis	200-500	Tisuk	Hibiscus macrophyllus Roxb. ex I	Malvaceae	0,4	7,0	9	12	0,01	0,00	16,65	0,02	0,78	2,87
3548	70	2	Ciamis	200-500	Tisuk	Hibiscus macrophyllus Roxb. ex I	Malvaceae	0,4	2,5	3,5	4,5	0,01	0,00	1,06	0,00	0,05	0,18
3549	70	2	Ciamis	200-500	Tisuk	Hibiscus macrophyllus Roxb. ex I	Malvaceae	0,4	4,8	6,5	8	0,01	0,00	5,69	0,01	0,27	0,98
3550	70	2	Ciamis	200-500	Tisuk	Hibiscus macrophyllus Roxb. ex I	Malvaceae	0,4	4,8	8	9,5	0,01	0,00	6,67	0,01	0,31	1,15
3551	70	2	Ciamis	200-500	Tisuk	Hibiscus macrophyllus Roxb. ex I	Malvaceae	0,4	4,5	3,5	5,3	0,01	0,00	3,44	0,00	0,16	0,59
3552	70	2	Ciamis	200-500	Mahoni	Swietenia macrophylla King	Meliaceae	0,61	9,2	4,5	7	0,01	0,00	24,81	0,02	1,17	4,28
3553	70	2	Ciamis	200-500	Mahoni	Swietenia macrophylla King	Meliaceae	0,61	11,1	8	11	0,01	0,00	52,98	0,05	2,49	9,14
3554	70	2	Ciamis	200-500	Mahoni	Swietenia macrophylla King	Meliaceae	0,61	5,4	5	7,5	0,01	0,00	9,94	0,01	0,47	1,71
3555	70	2	Ciamis	200-500	Mahoni	Swietenia macrophylla King	Meliaceae	0,61	6,4	3	7	0,01	0,00	12,56	0,01	0,59	2,17
3556	70	2	Ciamis	200-500	Mahoni	Swietenia macrophylla King	Meliaceae	0,61	6,0	4	4,5	0,01	0,00	7,63	0,01	0,36	1,32
3557	70	2	Ciamis	200-500	Mahoni	Swietenia macrophylla King	Meliaceae	0,61	5,7	3	4	0,01	0,00	6,20	0,01	0,29	1,07
3558	70	2	Ciamis	200-500	Mahoni	Swietenia macrophylla King	Meliaceae	0,61	9,9	6	9	0,01	0,00	35,30	0,04	1,66	6,09
3559	70	2	Ciamis	200-500	Mahoni	Swietenia macrophylla King	Meliaceae	0,61	3,2	4	5,5	0,01	0,00	2,83	0,00	0,13	0,49
3560	70	2	Ciamis	200-500	Mahoni	Swietenia macrophylla King	Meliaceae	0,61	8,9	6	10	0,01	0,00	32,26	0,03	1,52	5,56
3561	70	2	Ciamis	200-500	Mahoni	Swietenia macrophylla King	Meliaceae	0,61	3,8	3,5	5,5	0,01	0,00	3,95	0,00	0,19	0,68
3562	70	2	Ciamis	200-500	Mahoni	Swietenia macrophylla King	Meliaceae	0,61	5,7	3,5	6,5	0,01	0,00	9,68	0,01	0,45	1,67
3563	70	2	Ciamis	200-500	Mahoni	Swietenia macrophylla King	Meliaceae	0,61	5,7	3,5	6,5	0,01	0,00	9,68	0,01	0,45	1,67
3564	70	2	Ciamis	200-500	Mahoni	Swietenia macrophylla King	Meliaceae	0,61	3,8	2,5	5,5	0,01	0,00	3,95	0,00	0,19	0,68
3565	70	2	Ciamis	200-500	Mahoni	Swietenia macrophylla King	Meliaceae	0,61	7,6	3,5	7	0,01	0,00	17,54	0,02	0,82	3,03
3566	70	2	Ciamis	200-500	Mahoni	Swietenia macrophylla King	Meliaceae	0,61	11,8	5	10	0,01	0,00	53,76	0,05	2,53	9,27
3567	70	2	Ciamis	200-500	Mahoni	Swietenia macrophylla King	Meliaceae	0,61	3,8	3,5	6	0,01	0,00	4,28	0,00	0,20	0,74
3568	70	2	Ciamis	200-500	Mahoni	Swietenia macrophylla King	Meliaceae	0,61	7,0	4	8	0,01	0,00	16,91	0,02	0,79	2,92
3569	70	2	Ciamis	200-500	Mahoni	Swietenia macrophylla King	Meliaceae	0,61	7,3	7	10	0,01	0,00	22,50	0,02	1,06	3,88
3570	70	2	Ciamis	200-500	Mahoni	Swietenia macrophylla King	Meliaceae	0,61	7,6	4	9	0,01	0,00	22,09	0,02	1,04	3,81
3571	70	2	Ciamis	200-500	Jengkol	Archidendron pauciflorum (Bentl	Leguminosae / Fabaceae	0,4	6,0	5	9	0,01	0,00	9,78	0,01	0,46	1,69
3572	70	2	Ciamis	200-500	Nangka	Artocarpus heterophyllus Lam.	Moraceae	0,61	3,8	3,5	5	0,01	0,00	3,62	0,00	0,17	0,62
3573	70	2	Ciamis	200-500	Sengon	Paraserianthes falcataria (L.) I.C.	Leguminosae / Fabaceae	0,33	21,3	8	15	0,01	0,01	131,75	0,13	6,19	22,73
3574	70	2	Ciamis	200-500	Sengon	Paraserianthes falcataria (L.) I.C.	Leguminosae / Fabaceae	0,33	14,6	9	12	0,01	0,00	53,92	0,05	2,53	9,30
3575	70	2	Ciamis	200-500	Durian	Durio zibethinus L.	Malvaceae	0,57	12,7	8,5	11,5	0,01	0,00	66,23	0,07	3,11	11,42
3576	70	2	Ciamis	200-500	Kokosan	Lansium domesticum var. aquae	Meliaceae	0,7	7,6	4	7	0,01	0,00	19,90	0,02	0,94	3,43
3577	70	2	Ciamis	200-500	Petai	Parkia speciosa Hassk.	Leguminosae / Fabaceae	0,45	4,5	6	7	0,01	0,00	4,95	0,00	0,23	0,85
3578	70	2	Ciamis	200-500	Durian	Durio zibethinus L.	Malvaceae	0,57	2,9	1,7	3,5	0,01	0,00	1,45	0,00	0,07	0,25
3579	70	2	Ciamis	200-500	Melinjo	Gnetum gnemon L.	Gnetaceae	0,76	3,8	0,5	4	0,03	0,00	3,61	0,00	0,06	0,21
3580	70	2	Ciamis	200-500	Sengon	Paraserianthes falcataria (L.) I.C.	Leguminosae / Fabaceae	0,33	5,1	9	16	0,03	0,00	10,14	0,01	0,16	0,58
3581	70	2	Ciamis	200-500	Rambutan	Nephelium lappaceum L.	Sapindaceae	0,71	4,8	3	5	0,03	0,00	6,26	0,01	0,10	0,36
3582	70	2	Ciamis	200-500	Dukuh	Lansium domesticum Corrêa	Meliaceae	0,85	30,5	7	16	0,03	0,05	642,63	0,64	10,07	36,95
3583	70	2	Ciamis	200-500	Dukuh	Lansium domesticum Corrêa	Meliaceae	0,85	24,5	11	19	0,03	0,04	502,18	0,50	7,87	28,87
3584	70	2	Ciamis	200-500	Manggis	Mangostana garcinia Gaertn.	Clusiaceae	0,81	14,3	5,5	10	0,03	0,01	99,76	0,10	1,56	5,74
3585	70	2	Ciamis	200-500	Manggis	Mangostana garcinia Gaertn.	Clusiaceae	0,81	8,3	3	8	0,03	0,00	29,77	0,03	0,47	1,71
3586	70	2	Ciamis	200-500	Dukuh	Lansium domesticum Corrêa	Meliaceae	0,85	38,8	8	18	0,03	0,09	1.110,47	1,11	17,40	63,85
3587	70	2	Ciamis	200-500	Dukuh	Lansium domesticum Corrêa	Meliaceae	0,85	7,0	3,5	7,5	0,03	0,00	21,59	0,02	0,34	1,24
3588	70	2	Ciamis	200-500	Dukuh	Lansium domesticum Corrêa	Meliaceae	0,85	38,2	9	18	0,03	0,09	1.077,35	1,08	16,88	61,94
3589	70	2	Ciamis	200-500	Dukuh	Lansium domesticum Corrêa	Meliaceae	0,85	26,4	7	16	0,03	0,04	492,26	0,49	7,71	28,30
3590	70	2	Ciamis	200-500	Manggis	Mangostana garcinia Gaertn.	Clusiaceae	0,81	12,4	3,5	11	0,03	0,01	83,76	0,08	1,31	4,82

3591	70	2	Ciamis	200-500	Kokosan	Lansium domesticum var. aquae	Meliaceae		0,7	10,5	6,5	9,5	0,03	0,01	47,18	0,05	0,74	2,71
3592	70	2	Ciamis	200-500	Dukuh	Lansium domesticum Corrêa	Meliaceae		0,85	40,4	5	16	0,03	0,10	1.073,02	1,07	16,81	61,70
3593	70	2	Ciamis	200-500	Kokosan	Lansium domesticum var. aquae	Meliaceae		0,7	14,3	6	10	0,03	0,01	87,28	0,09	1,37	5,02
3594	70	2	Ciamis	200-500	Dukuh	Lansium domesticum Corrêa	Meliaceae		0,85	49,3	8	18	0,03	0,14	1.721,81	1,72	26,97	99,00
3595	70	2	Ciamis	200-500	Durian	Durio zibethinus L.	Malvaceae		0,57	45,8	15	23	0,03	0,12	1.306,06	1,31	20,46	75,09
3596	70	2	Ciamis	200-500	Manggis	Mangostana garcinia Gaertn.	Clusiaceae		0,81	5,7	2	5	0,03	0,00	9,87	0,01	0,15	0,57
3597	70	2	Ciamis	200-500	Dukuh	Lansium domesticum Corrêa	Meliaceae		0,85	6,4	3	5	0,03	-	12,51	0,01	0,20	0,72
3598	70	2	Ciamis	200-500	Rambutan	Nephelium lappaceum L.	Sapindaceae		0,71	4,5	2,5	5	0,03	-	5,52	0,01	0,09	0,32
3599	70	2	Ciamis	200-500	Jati	Tectona grandis L.f.	Lamiaceae / Verbenaceae		0,7	7,6	3,5	7,5	0,03	-	21,20	0,02	0,33	1,22
3600	70	2	Ciamis	200-500	Jengkol	Archidendron pauciflorum (Bentl	Leguminosae / Fabaceae		0,4	6,4	4	6	0,03	-	7,41	0,01	0,12	0,43
3601	70	2	Ciamis	200-500	Jati	Tectona grandis L.f.	Lamiaceae / Verbenaceae		0,7	8,0	2,5	5	0,03	-	15,76	0,02	0,25	0,91
3602	70	2	Ciamis	200-500	Durian	Durio zibethinus L.	Malvaceae		0,57	9,5	8	10	0,03	-	34,40	0,03	0,54	1,98
3603	70	2	Ciamis	200-500	Jengkol	Archidendron pauciflorum (Bentl	Leguminosae / Fabaceae		0,4	5,7	4,5	6,5	0,03	-	6,57	0,01	0,10	0,38
3604	70	2	Ciamis	200-500	Dukuh	Lansium domesticum Corrêa	Meliaceae		0,85	19,1	7	11	0,03	-	192,73	0,19	3,02	11,08
3605	70	2	Ciamis	200-500	Melinjo	Gnetum gnemon L.	Gnetaceae		0,76	3,8	1,5	7	0,03	-	6,03	0,01	0,09	0,35
3606	71	1	Ciamis	200-500	Sp8	Sp8		0	0,5	5,1	3	4	0,04	0,00	4,17	0,00	0,05	0,18
3607	71	1	Ciamis	200-500	Mahoni	Swietenia macrophylla King	Meliaceae		0,61	38,2	14	20	0,04	0,11	875,56	0,88	10,29	37,76
3608	71	1	Ciamis	200-500	Mahoni	Swietenia macrophylla King	Meliaceae		0,61	30,2	9	17	0,04	0,07	491,77	0,49	5,78	21,21
3609	71	1	Ciamis	200-500	Mahoni	Swietenia macrophylla King	Meliaceae		0,61	10,2	3	6	0,04	0,01	25,80	0,03	0,30	1,11
3610	71	1	Ciamis	200-500	Mahoni	Swietenia macrophylla King	Meliaceae		0,61	44,5	4	14	0,04	0,16	837,61	0,84	9,84	36,12
3611	71	1	Ciamis	200-500	Kopi	Coffea canephora Pierre ex A.Fro	Rubiaceae		0,41	3,8	1	5	0,04	0,00	4,38	0,00	0,05	0,19
3612	71	1	Ciamis	200-500	Mahoni	Swietenia macrophylla King	Meliaceae		0,61	8,0	3	7	0,04	0,00	18,91	0,02	0,22	0,82
3613	71	1	Ciamis	200-500	Kopi	Coffea canephora Pierre ex A.Fro	Rubiaceae		0,41	6,7	1,5	5	0,04	0,00	13,80	0,01	0,16	0,59
3614	71	1	Ciamis	200-500	Dukuh	Lansium domesticum Corrêa	Meliaceae		0,85	6,7	3	5	0,04	0,00	13,68	0,01	0,16	0,59
3615	71	1	Ciamis	200-500	Kopi	Coffea canephora Pierre ex A.Fro	Rubiaceae		0,41	6,4	1	4	0,04	0,00	12,48	0,01	0,15	0,54
3616	71	1	Ciamis	200-500	Kelapa	Cocos nucifera L.	Arecaceae		0,5	28,0	11	12	0,04	0,06	96,90	0,10	1,14	4,18
3617	71	1	Ciamis	200-500	Mangga	Mangifera indica L.	Anacardiaceae		0,6	13,0	6	16	0,04	0,01	98,29	0,10	1,15	4,24
3618	71	1	Ciamis	200-500	Wangkal	Albizia procera Benth	Leguminosae / Fabaceae		0,77	33,4	11	15	0,04	0,09	652,02	0,65	7,66	28,12
3619	71	1	Ciamis	200-500	Mahoni	Swietenia macrophylla King	Meliaceae		0,61	31,8	6	18	0,04	0,08	569,26	0,57	6,69	24,55
3620	71	1	Ciamis	200-500	Mahoni	Swietenia macrophylla King	Meliaceae		0,61	64,6	6	21	0,04	0,33	2.398,65	2,40	28,18	103,44
3621	71	1	Ciamis	200-500	Mahoni	Swietenia macrophylla King	Meliaceae		0,61	19,4	4	10	0,04	0,03	134,34	0,13	1,58	5,79
3622	71	1	Ciamis	200-500	Kelapa	Cocos nucifera L.	Arecaceae		0,5	30,9	14	17	0,04	0,07	135,40	0,14	1,59	5,84
3623	71	1	Ciamis	200-500	Mahoni	Swietenia macrophylla King	Meliaceae		0,61	4,1	2,5	4	0,04	0,00	3,42	0,00	0,04	0,15
3624	71	1	Ciamis	200-500	Mahoni	Swietenia macrophylla King	Meliaceae		0,61	15,3	2,5	13	0,04	0,02	110,13	0,11	1,29	4,75
3625	71	1	Ciamis	200-500	Mahoni	Swietenia macrophylla King	Meliaceae		0,61	28,6	10	19	0,04	0,06	493,16	0,49	5,79	21,27
3626	71	1	Ciamis	200-500	Mahoni	Swietenia macrophylla King	Meliaceae		0,61	38,2	4	19	0,04	0,11	835,37	0,84	9,82	36,02
3627	71	1	Ciamis	200-500	Mahoni	Swietenia macrophylla King	Meliaceae		0,61	8,3	4	7	0,04	0,01	20,32	0,02	0,24	0,88
3628	71	1	Ciamis	200-500	Mahoni	Swietenia macrophylla King	Meliaceae		0,61	28,3	4	12	0,04	0,06	317,17	0,32	3,73	13,68
3629	71	1	Ciamis	200-500	Mahoni	Swietenia macrophylla King	Meliaceae		0,61	34,0	14	19	0,04	0,09	677,10	0,68	7,96	29,20
3630	71	1	Ciamis	200-500	Mahoni	Swietenia macrophylla King	Meliaceae		0,61	10,2	5	10	0,04	0,01	41,20	0,04	0,48	1,78
3631	71	1	Ciamis	200-500	Kelapa	Cocos nucifera L.	Arecaceae		0,5	32,5	13	16	0,04	0,08	127,70	0,13	1,50	5,51
3632	71	1	Ciamis	200-500	Mahoni	Swietenia macrophylla King	Meliaceae		0,61	5,7	3	5	0,04	0,00	7,61	0,01	0,09	0,33
3633	71	1	Ciamis	200-500	Mahoni	Swietenia macrophylla King	Meliaceae		0,61	6,7	4	6	0,04	0,00	11,93	0,01	0,14	0,51
3634	71	1	Ciamis	200-500	Mahoni	Swietenia macrophylla King	Meliaceae		0,61	33,1	9	16	0,04	0,09	549,11	0,55	6,45	23,68
3635	71	1	Ciamis	200-500	Mahoni	Swietenia macrophylla King	Meliaceae		0,61	15,6	3	8	0,04	0,02	73,31	0,07	0,86	3,16
3636	71	1	Ciamis	200-500	Mahoni	Swietenia macrophylla King	Meliaceae		0,61	8,6	3,5	5,5	0,04	0,01	17,45	0,02	0,21	0,75
3637	71	1	Ciamis	200-500	Mahoni	Swietenia macrophylla King	Meliaceae		0,61	22,3	10	16	0,04	0,04	265,87	0,27	3,12	11,47
3638	71	1	Ciamis	200-500	Rambutan	Nephelium lappaceum L.	Sapindaceae		0,71	4,8	3,5	6	0,04	0,00	7,40	0,01	0,09	0,32
3639	71	1	Ciamis	200-500	Mahoni	Swietenia macrophylla King	Meliaceae		0,61	49,3	10	19	0,04	0,19	1.335,08	1,34	15,69	57,57
3640	71	1	Ciamis	200-500	Mahoni	Swietenia macrophylla King	Meliaceae		0,61	43,9	12	18	0,04	0,15	1.027,00	1,03	12,07	44,29
3641	72	1	Ciamis	200-500	Gmelina	Gmelina arborea Roxb.	Lamiaceae / Verbenaceae		0,47	4,5	1,7	4,5	0,04	-	3,43	0,00	0,04	0,15
3642	72	1	Ciamis	200-500	Mahoni	Swietenia macrophylla King	Meliaceae		0,61	13,0	4	9	0,04	0,01	58,91	0,06	0,69	2,54
3643	72	1	Ciamis	200-500	Mahoni	Swietenia macrophylla King	Meliaceae		0,61	8,9	5	8	0,04	0,01	26,30	0,03	0,31	1,13
3644	72	1	Ciamis	200-500	Mahoni	Swietenia macrophylla King	Meliaceae		0,61	10,8	5	9	0,04	0,01	41,81	0,04	0,49	1,80
3645	72	1	Ciamis	200-500	Mahoni	Swietenia macrophylla King	Meliaceae		0,61	8,9	4	9	0,04	0,01	29,29	0,03	0,34	1,26
3646	72	1	Ciamis	200-500	Mahoni	Swietenia macrophylla King	Meliaceae		0,61	11,1	6	10	0,04	0,01	48,55	0,05	0,57	2,09
3647	72	1	Ciamis	200-500	Rambutan	Nephelium lappaceum L.	Sapindaceae		0,71	7,6	8	13	0,04	0,00	35,55	0,04	0,42	1,53
3648	72	1	Ciamis	200-500	Mahoni	Swietenia macrophylla King	Meliaceae		0,61	17,2	4,5	11	0,04	0,02	117,26	0,12	1,38	5,06
3649	72	1	Ciamis	200-500	Rambutan	Nephelium lappaceum L.	Sapindaceae		0,71	9,2	6	9	0,04	0,01	35,90	0,04	0,42	1,55
3650	72	1	Ciamis	200-500	Mahoni	Swietenia macrophylla King	Meliaceae		0,61	7,6	4	7,5	0,04	0,00	18,69	0,02	0,22	0,81
3651	72	1	Ciamis	200-500	Mahoni	Swietenia macrophylla King	Meliaceae		0,61	12,7	4	8	0,04	0,01	50,55	0,05	0,59	2,18
3652	72	1	Ciamis	200-500	Mahoni	Swietenia macrophylla King	Meliaceae		0,61	16,5	4	11	0,04	0,02	109,42	0,11	1,29	4,72
3653	72	1	Ciamis	200-500	Mahoni	Swietenia macrophylla King	Meliaceae		0,61	9,9	3	6	0,04	0,01	24,35	0,02	0,29	1,05
3654	72	1	Ciamis	200-500	Mahoni	Swietenia macrophylla King	Meliaceae		0,61	8,6	3,5	7	0,04	0,01	21,77	0,02	0,26	0,94
3655	72	1	Ciamis	200-500	Mahoni	Swietenia macrophylla King	Meliaceae		0,61	15,0	7	12	0,04	0,02	98,47	0,10	1,16	4,25
3656	72	1	Ciamis	200-500	Mahoni	Swietenia macrophylla King	Meliaceae		0,61	13,0	3	10	0,04	0,01	64,88	0,06	0,76	2,80
3657	72	1	Ciamis	200-500	Mahoni	Swietenia macrophylla King	Meliaceae		0,61	14,0	3	2	0,04	0,02	16,91	0,02	0,20	0,73
3658	72	1	Ciamis	200-500	Mahoni	Swietenia macrophylla King	Meliaceae		0,61	13,0	2,5	10	0,04	0,01	64,88	0,06	0,76	2,80
3659	72	1	Ciamis	200-500	Melinjo	Gnetum gnemon L.	Gnetaceae		0,76	3,2	0,8	2	0,04	0,00	1,37	0,00	0,02	0,06
3660	72	1	Ciamis	200-500	Mahoni	Swietenia macrophylla King	Meliaceae		0,61	3,5	2	4	0,04	0,00	2,52	0,00	0,03	0,11
3661	72	1	Ciamis	200-500	Kokosan	Lansium domesticum var. aquae	Meliaceae		0,7	4,8	4	5,5	0,04	0,00	6,75	0,01	0,08	0,29
3662	72	1	Ciamis	200-500	Mahoni	Swietenia macrophylla King	Meliaceae		0,61	10,8	3	7	0,04	0,01	33,21	0,03	0,39	1,43
3663	72	1	Ciamis	200-500	Mahoni	Swietenia macrophylla King	Meliaceae		0,61	13,0	5	9	0,04	0,01	58,91	0,06	0,69	2,54
3664	72	1	Ciamis	200-500	Mahoni	Swietenia macrophylla King	Meliaceae		0,61	9,9	5	9	0,04	0,01	35,30	0,04	0,41	1,52
3665	72	1	Ciamis	200-500	Mahoni	Swietenia macrophylla King	Meliaceae		0,61	12,4	3,5	10,5	0,04	0,01	61,90	0,06	0,73	2,67
3666	72	1	Ciamis	200-500	Mahoni	Swietenia macrophylla King	Meliaceae		0,61	10,8	3,5	9,5	0,04	0,01	43,93	0,04	0,52	1,89
3667	72	1	Ciamis	200-500	Mahoni	Swietenia macrophylla King	Meliaceae		0,61	8,6	3,5	8,5	0,04	0,01	26,01	0,03	0,31	1,12
3668	72	1	Ciamis	200-500	Mahoni	Swietenia macrophylla King	Meliaceae		0,61	15,9	4,5	11,5	0,04	0,02	106,07	0,11	1,25	4,57
3669	72	1	Ciamis	200-500	Mahoni	Swietenia macrophylla King	Meliaceae		0,61	7,0	3	5	0,04	0,00	10,99	0,01	0,13	0,47
3670	72	1	Ciamis	200-500	Mahoni	Swietenia macrophylla King	Meliaceae		0,61	7,0	2,5	4,5	0,04	0,00	9,98	0,01	0,12	0,43
3671	72	1	Ciamis	200-500	Mahoni	Swietenia macrophylla King	Meliaceae		0,61	10,2	4	8	0,04	0,01	33,59	0,03	0,39	1,45
3672	72	1	Ciamis	200-500	Mahoni	Swietenia macrophylla King	Meliaceae		0,61	12,1	3,5	10,5	0,04	0,01	59,03	0,06	0,69	2,55
3673	72	1	Ciamis	200-500	Nangka	Artocarpus heterophyllus Lam.	Moraceae		0,61	16,2	7	12	0,04	0,02	114,36	0,11	1,34	4,93
3674	72	1	Ciamis	200-500	Mahoni	Swietenia macrophylla King	Meliaceae		0,61	7,0	3,5	5,5	0,04	0,00	11,99	0,01	0,14	0,52
3675	72	1	Ciamis	200-500	Petai	Parkia speciosa Hassk.	Leguminosae / Fabaceae		0,45	19,1	14	17	0,04	0,03	160,37	0,16	1,88	6,92
3676	72	1	Ciamis	200-500	Mahoni	Swietenia macrophylla King	Meliaceae		0,61	15,9	7	13	0,04	0,02	118,68	0,12	1,39	5,12
3677	72	1	Ciamis	200-500	Mahoni	Swietenia macrophylla King	Meliaceae		0,61	10,8	5	10	0,04	0,01	46,04	0,05	0,54	1,99
3678	72	1	Ciamis	200-500	Kokosan	Lansium domesticum var. aquae	Meliaceae		0,7	7,6	4	5,5	0,04	0,00	15,96	0,02	0,19	0,69
3679	72	1	Ciamis	200-500	Mahoni	Swietenia macrophylla King	Meliaceae		0,61	6,4	3,5	4,5	0,04	0,00	8,38	0,01	0,10	0,36
3680	72	1	Ciamis	200-500	Mahoni	Swietenia macrophylla King	Meliaceae		0,61	10,5	4	8	0,04	0,01	35,53	0,04	0,42	1,53
3681	72	1	Ciamis	200-500	Kelapa	Cocos nucifera L.	Arecaceae		0,5	26,7	8	10	0,04	0,06	81,50	0,08	0,96	3,51
3682	72	1	Ciamis	200-500	Mahoni	Swietenia macrophylla King	Meliaceae		0,61	9,5	4,5	9,5	0,04	0,01	34,93	0,03	0,41	1,51
3683	72	1	Ciamis	200-500	Mahoni	Swietenia macrophylla King	Meliaceae		0,61	9,5	4	8	0,04	0,01	29,84	0,03	0,35	1,29
3684	72	1	Ciamis	200-500	Mahoni	Swietenia macrophylla King	Meliaceae		0,61	7,0	4	5,5	0,04	0,00	11,99	0,01	0,14	0,52
3685	72	1	Ciamis	200-500	Mahoni	Swietenia macrophylla King	Meliaceae		0,61	9,9	6	11	0,04	0,01	42,42	0,04	0,50	1,83
3686	72	1	Ciamis	200-500	Mahoni	Swietenia macrophylla King	Meliaceae		0,61	15,3	7	12	0,04	0,02	102,34	0,10	1,20	4,41
3687	72	1	Ciamis	200-500	Mahoni	Swietenia macrophylla King	Meliaceae		0,61	9,2	3	4,5	0,04	0,01	16,56	0,02	0,19	0,71
3688	72	1	Ciamis	200-500	Mahoni	Swietenia macrophylla King	Meliaceae		0,61	9,2	5	8	0,04	0,01	28,04	0,03	0,33	1,21
3689	72	1	Ciamis	200-500	Kelapa	Cocos nucifera L.	Arecaceae		0,5	26,7	10	13	0,04	0,06	104,60	0,10	1,23	4,51
3690	72	1	Ciamis	200-500	Kokosan	Lansium domesticum var. aquae	Meliaceae		0,7	4,1	3	6	0,04	0,00	5,62	0,01	0,07	0,24
3691	72	1	Ciamis	200-500	Mahoni	Swietenia macrophylla King	Meliaceae		0,61	7,3	3	6	0,04	0,00	14,09	0,01	0,17	0,61
3692	72	1	Ciamis	200-500	Mahoni	Swietenia macrophylla King	Meliaceae		0,61	9,9	6	9	0,04	0,01	35,30	0,04	0,41	1,52
3693	72	1	Ciamis	200-500	Mahoni	Swietenia macrophylla King	Meliaceae		0,61	10,5	7	11	0,04	0,01	47,57	0,05	0,56	2,05
3694	72	1	Ciamis	200-500	Mahoni	Swietenia macrophylla King	Meliaceae		0,61	14,0	4	11	0,04	0,02	80,58	0,08	0,95	3,47
3695	72	1	Ciamis	200-500	Renghas	Gluta renghas L.	Anacardiaceae		0,69	9,5	11	12,5	0,04	0,01	50,28	0,05	0,59	2,17
3696	72	1	Ciamis	200-500	Mahoni	Swietenia macrophylla King	Meliaceae		0,61	18,5	8	13	0,04	0,03	155,76	0,16	1,83	6,72
3697	72	1	Ciamis	200-500	Mahoni	Swietenia macrophylla King	Meliaceae		0,61	9,9	3	8,5	0,04	0,01	33,50	0,03	0,39	1,44
3698	72	1	Ciamis	200-500	Mahoni	Swietenia macrophylla King	Meliaceae		0,61	17,2	8	12	0,04	0,02	126,99	0,13	1,49	5,48
3699	72	1	Ciamis	200-500	Mahoni	Swietenia macrophylla King	Meliaceae		0,61	4,5	2,5	4,5	0,04	0,00	4,36	0,00	0,05	0,19
3700	72	1	Ciamis	200-500	Mahoni	Swietenia macrophylla King	Meliaceae		0,61	8,6	5	10	0,04	0,01	30,18	0,03	0,35	1,30
3701	72	1	Ciamis	200-500	Mahoni	Swietenia macrophylla King	Meliaceae		0,61	11,1	4	9	0,04	0,01	44,09	0,04	0,52	1,90
3702	72	1	Ciamis	200-500	Mahoni	Swietenia macrophylla King	Meliaceae		0,61	6,7	2,5	5	0,04	0,00	10,09	0,01	0,12	0,44
3703	72	1	Ciamis	200-500	Kelapa	Cocos nucifera L.	Arecaceae		0,5	22,3	5	7	0,04	0,04	58,40	0,06	0,69	2,52
3704	72	1	Ciamis	200-500	Mahoni	Swietenia macrophylla King	Meliaceae		0,61	15,6	5	11	0,04	0,02	98,14	0,10	1,15	4,23
3705	72	1	Ciamis	200-500	Melinjo	Gnetum gnemon L.	Gnetaceae		0,76	4,5	1,5	4,5	0,04	0,00	5,33	0,01	0,06	0,23
3706	72	1	Ciamis	200-500	Mahoni	Swietenia macrophylla King	Meliaceae		0,61	1,3	3	8	0,04	0,00	0,74	0,00	0,01	0,03
3707	72	1	Ciamis	200-500	Melinjo	Gnetum gnemon L.	Gnetaceae		0,76	2,2	1	2	0,04	0,00	0,71	0,00	0,01	0,03
3708	72	1	Ciamis	200-500	Mahoni	Swietenia macrophylla King	Meliaceae		0,61	9,9	3,5	7	0,04	0,01	28,04	0,03	0,33	1,21
3709	72	1	Ciamis	200-500	Mahoni	Swietenia macrophylla King	Meliaceae		0,61	5,4	3,5	4,5	0,04	0,00	6,22	0,01	0,07	0,27
3710	72	1	Ciamis	200-500	Mahoni	Swietenia macrophylla King	Meliaceae		0,61	13,4	4,5	9,5	0,04	0,01	64,69	0,06	0,76	2,79
3711	72	1	Ciamis	200-500	Mahoni	Swietenia macrophylla King	Meliaceae		0,61	15,3	3	9	0,04	0,02	78,63	0,08	0,92	3,39
3712	72	1	Ciamis	200-500	Mahoni	Swietenia macrophylla King	Meliaceae		0,61	15,9	5	14	0,04	0,02	127,01	0,13	1,49	5,48
3713	72	1	Ciamis	200-500	Melinjo	Gnetum gnemon L.	Gnetaceae		0,76	15,6	1,5	8,5	0,04	0,02	94,78	0,09	1,11	4,09
3714	72	1	Ciamis	200-500	Mahoni	Swietenia macrophylla King	Meliaceae		0,61	9,5	3	7	0,04	0,01	26,40	0,03	0,31	1,14

3715	72	1	Ciamis	200-500	Mahoni	Swietenia macrophylla King	Meliaceae	0,61	11,1	3	9	0,04	0,01	44,09	0,04	0,52	1,90
3716	73	1	Ciamis	200-500	Sengon	Paraserianthes falcataria (L.) I.C.	Leguminosae / Fabaceae	0,33	8,3	6	9	0,04	-	14,57	0,01	0,17	0,63
3717	73	1	Ciamis	200-500	Sengon	Paraserianthes falcataria (L.) I.C.	Leguminosae / Fabaceae	0,33	6,7	8	9,5	0,04	0,00	10,35	0,01	0,12	0,45
3718	73	1	Ciamis	200-500	Sengon	Paraserianthes falcataria (L.) I.C.	Leguminosae / Fabaceae	0,33	11,8	11	12,5	0,04	0,01	37,57	0,04	0,44	1,62
3719	73	1	Ciamis	200-500	Sengon	Paraserianthes falcataria (L.) I.C.	Leguminosae / Fabaceae	0,33	12,1	6	12	0,04	0,01	38,00	0,04	0,45	1,64
3720	73	1	Ciamis	200-500	Sengon	Paraserianthes falcataria (L.) I.C.	Leguminosae / Fabaceae	0,33	6,7	7	17,5	0,04	0,00	18,11	0,02	0,21	0,78
3721	73	1	Ciamis	200-500	Sengon	Paraserianthes falcataria (L.) I.C.	Leguminosae / Fabaceae	0,33	8,3	9	10,5	0,04	0,01	16,78	0,02	0,20	0,72
3722	73	1	Ciamis	200-500	Sengon	Paraserianthes falcataria (L.) I.C.	Leguminosae / Fabaceae	0,33	7,6	7	10	0,04	0,00	13,86	0,01	0,16	0,60
3723	73	1	Ciamis	200-500	Sengon	Paraserianthes falcataria (L.) I.C.	Leguminosae / Fabaceae	0,33	5,4	8	9,5	0,04	0,00	7,03	0,01	0,08	0,30
3724	73	1	Ciamis	200-500	Sengon	Paraserianthes falcataria (L.) I.C.	Leguminosae / Fabaceae	0,33	9,9	9	11	0,04	0,01	24,16	0,02	0,28	1,04
3725	73	1	Ciamis	200-500	Sengon	Paraserianthes falcataria (L.) I.C.	Leguminosae / Fabaceae	0,33	7,6	7	10	0,04	0,00	13,86	0,01	0,16	0,60
3726	73	1	Ciamis	200-500	Sengon	Paraserianthes falcataria (L.) I.C.	Leguminosae / Fabaceae	0,33	3,5	5	6	0,04	0,00	2,08	0,00	0,02	0,09
3727	73	1	Ciamis	200-500	Sengon	Paraserianthes falcataria (L.) I.C.	Leguminosae / Fabaceae	0,33	9,9	8	10	0,04	0,01	22,14	0,02	0,26	0,95
3728	73	1	Ciamis	200-500	Sengon	Paraserianthes falcataria (L.) I.C.	Leguminosae / Fabaceae	0,33	4,1	4	6	0,04	0,00	2,82	0,00	0,03	0,12
3729	73	1	Ciamis	200-500	Sengon	Paraserianthes falcataria (L.) I.C.	Leguminosae / Fabaceae	0,33	7,6	8	10	0,04	0,00	13,86	0,01	0,16	0,60
3730	73	1	Ciamis	200-500	Sengon	Paraserianthes falcataria (L.) I.C.	Leguminosae / Fabaceae	0,33	5,7	7	8	0,04	0,00	6,67	0,01	0,08	0,29
3731	73	1	Ciamis	200-500	Sengon	Paraserianthes falcataria (L.) I.C.	Leguminosae / Fabaceae	0,33	6,7	8	9	0,04	0,00	9,85	0,01	0,12	0,42
3732	73	1	Ciamis	200-500	Sengon	Paraserianthes falcataria (L.) I.C.	Leguminosae / Fabaceae	0,33	10,5	6	11	0,04	0,01	27,10	0,03	0,32	1,17
3733	73	1	Ciamis	200-500	Sengon	Paraserianthes falcataria (L.) I.C.	Leguminosae / Fabaceae	0,33	9,5	9	11	0,04	0,01	22,76	0,02	0,27	0,98
3734	73	1	Ciamis	200-500	Sengon	Paraserianthes falcataria (L.) I.C.	Leguminosae / Fabaceae	0,33	11,1	9	11	0,04	0,01	30,18	0,03	0,35	1,30
3735	73	1	Ciamis	200-500	Sengon	Paraserianthes falcataria (L.) I.C.	Leguminosae / Fabaceae	0,33	10,2	6,5	10,5	0,04	0,01	24,54	0,02	0,29	1,06
3736	73	1	Ciamis	200-500	Sengon	Paraserianthes falcataria (L.) I.C.	Leguminosae / Fabaceae	0,33	6,0	8,5	11,5	0,04	0,00	10,26	0,01	0,12	0,44
3737	73	1	Ciamis	200-500	Sengon	Paraserianthes falcataria (L.) I.C.	Leguminosae / Fabaceae	0,33	7,0	10	12	0,04	0,00	13,96	0,01	0,16	0,60
3738	73	1	Ciamis	200-500	Sengon	Paraserianthes falcataria (L.) I.C.	Leguminosae / Fabaceae	0,33	8,6	8	10	0,04	0,01	17,19	0,02	0,20	0,74
3739	73	1	Ciamis	200-500	Sengon	Paraserianthes falcataria (L.) I.C.	Leguminosae / Fabaceae	0,33	10,2	8	12	0,04	0,01	27,74	0,03	0,33	1,20
3740	73	1	Ciamis	200-500	Sengon	Paraserianthes falcataria (L.) I.C.	Leguminosae / Fabaceae	0,33	9,5	6	12	0,04	0,01	24,64	0,02	0,29	1,06
3741	73	1	Ciamis	200-500	Sengon	Paraserianthes falcataria (L.) I.C.	Leguminosae / Fabaceae	0,33	9,9	5	11	0,04	0,01	24,16	0,02	0,28	1,04
3742	73	1	Ciamis	200-500	Sengon	Paraserianthes falcataria (L.) I.C.	Leguminosae / Fabaceae	0,33	3,5	4,5	5,5	0,04	0,00	1,92	0,00	0,02	0,08
3743	73	1	Ciamis	200-500	Sengon	Paraserianthes falcataria (L.) I.C.	Leguminosae / Fabaceae	0,33	2,2	4,5	5,5	0,04	0,00	0,84	0,00	0,01	0,04
3744	73	1	Ciamis	200-500	Sengon	Paraserianthes falcataria (L.) I.C.	Leguminosae / Fabaceae	0,33	4,8	5	6,5	0,04	0,00	3,95	0,00	0,05	0,17
3745	73	1	Ciamis	200-500	Sengon	Paraserianthes falcataria (L.) I.C.	Leguminosae / Fabaceae	0,33	9,2	7	12	0,04	0,01	23,16	0,02	0,27	1,00
3746	73	1	Ciamis	200-500	Sengon	Paraserianthes falcataria (L.) I.C.	Leguminosae / Fabaceae	0,33	6,7	4	10	0,04	0,00	10,85	0,01	0,13	0,47
3747	73	1	Ciamis	200-500	Sengon	Paraserianthes falcataria (L.) I.C.	Leguminosae / Fabaceae	0,33	8,9	8	11	0,04	0,01	20,05	0,02	0,24	0,86
3748	73	1	Ciamis	200-500	Sengon	Paraserianthes falcataria (L.) I.C.	Leguminosae / Fabaceae	0,33	6,4	8	10,5	0,04	0,00	10,37	0,01	0,12	0,45
3749	73	1	Ciamis	200-500	Sengon	Paraserianthes falcataria (L.) I.C.	Leguminosae / Fabaceae	0,33	16,2	10	14	0,04	0,02	75,02	0,08	0,88	3,24
3750	73	1	Ciamis	200-500	Sengon	Paraserianthes falcataria (L.) I.C.	Leguminosae / Fabaceae	0,33	4,8	8	9	0,04	0,00	5,32	0,01	0,06	0,23
3751	73	1	Ciamis	200-500	Sengon	Paraserianthes falcataria (L.) I.C.	Leguminosae / Fabaceae	0,33	0,3	5	6,5	0,04	0,00	0,03	0,00	0,00	0,00
3752	73	1	Ciamis	200-500	Sengon	Paraserianthes falcataria (L.) I.C.	Leguminosae / Fabaceae	0,33	12,1	7	15	0,04	0,01	46,62	0,05	0,55	2,01
3753	73	1	Ciamis	200-500	Sengon	Paraserianthes falcataria (L.) I.C.	Leguminosae / Fabaceae	0,33	16,2	10	15	0,04	0,02	79,92	0,08	0,94	3,45
3754	73	1	Ciamis	200-500	Sengon	Paraserianthes falcataria (L.) I.C.	Leguminosae / Fabaceae	0,33	17,5	4	16	0,04	0,02	97,36	0,10	1,14	4,20
3755	73	1	Ciamis	200-500	Sengon	Paraserianthes falcataria (L.) I.C.	Leguminosae / Fabaceae	0,33	15,0	5	16	0,04	0,02	73,00	0,07	0,86	3,15
3756	73	1	Ciamis	200-500	Sengon	Paraserianthes falcataria (L.) I.C.	Leguminosae / Fabaceae	0,33	19,1	6	16	0,04	0,03	114,19	0,11	1,34	4,92
3757	73	1	Ciamis	200-500	Sengon	Paraserianthes falcataria (L.) I.C.	Leguminosae / Fabaceae	0,33	14,0	4	14	0,04	0,02	57,24	0,06	0,67	2,47
3758	73	1	Ciamis	200-500	Sengon	Paraserianthes falcataria (L.) I.C.	Leguminosae / Fabaceae	0,33	8,3	9	11	0,04	0,01	17,51	0,02	0,21	0,75
3759	73	1	Ciamis	200-500	Sengon	Paraserianthes falcataria (L.) I.C.	Leguminosae / Fabaceae	0,33	4,8	6	9	0,04	0,00	5,32	0,01	0,06	0,23
3760	73	1	Ciamis	200-500	Sengon	Paraserianthes falcataria (L.) I.C.	Leguminosae / Fabaceae	0,33	13,4	8	17	0,04	0,01	62,80	0,06	0,74	2,71
3761	73	1	Ciamis	200-500	Sengon	Paraserianthes falcataria (L.) I.C.	Leguminosae / Fabaceae	0,33	5,4	5	9	0,04	0,00	6,69	0,01	0,08	0,29
3762	73	1	Ciamis	200-500	Sengon	Paraserianthes falcataria (L.) I.C.	Leguminosae / Fabaceae	0,33	3,2	4	5	0,04	0,00	1,48	0,00	0,02	0,06
3763	73	1	Ciamis	200-500	Sengon	Paraserianthes falcataria (L.) I.C.	Leguminosae / Fabaceae	0,33	2,9	3	4	0,04	0,00	0,99	0,00	0,01	0,04
3764	73	1	Ciamis	200-500	Sengon	Paraserianthes falcataria (L.) I.C.	Leguminosae / Fabaceae	0,33	4,8	5	8	0,04	0,00	4,77	0,00	0,06	0,21
3765	73	1	Ciamis	200-500	Sengon	Paraserianthes falcataria (L.) I.C.	Leguminosae / Fabaceae	0,33	8,6	8	14	0,04	0,01	23,40	0,02	0,27	1,01
3766	73	1	Ciamis	200-500	Sengon	Paraserianthes falcataria (L.) I.C.	Leguminosae / Fabaceae	0,33	17,2	3	15	0,04	0,02	88,74	0,09	1,04	3,83
3767	73	1	Ciamis	200-500	Sengon	Paraserianthes falcataria (L.) I.C.	Leguminosae / Fabaceae	0,33	18,1	4	10	0,04	0,03	67,58	0,07	0,79	2,91
3768	73	1	Ciamis	200-500	Sengon	Paraserianthes falcataria (L.) I.C.	Leguminosae / Fabaceae	0,33	5,4	8	13	0,04	0,00	9,37	0,01	0,11	0,40
3769	73	1	Ciamis	200-500	Sengon	Paraserianthes falcataria (L.) I.C.	Leguminosae / Fabaceae	0,33	15,0	4	15	0,04	0,02	68,81	0,07	0,81	2,97
3770	73	1	Ciamis	200-500	Sengon	Paraserianthes falcataria (L.) I.C.	Leguminosae / Fabaceae	0,33	9,5	4	11	0,04	0,01	22,76	0,02	0,27	0,98
3771	73	1	Ciamis	200-500	Sengon	Paraserianthes falcataria (L.) I.C.	Leguminosae / Fabaceae	0,33	3,5	3,5	4,5	0,04	0,00	1,60	0,00	0,02	0,07
3772	73	1	Ciamis	200-500	Sengon	Paraserianthes falcataria (L.) I.C.	Leguminosae / Fabaceae	0,33	8,9	4	7	0,04	0,01	13,26	0,01	0,16	0,57
3773	73	1	Ciamis	200-500	Sengon	Paraserianthes falcataria (L.) I.C.	Leguminosae / Fabaceae	0,33	2,9	0,1	1,1	0,04	0,00	0,30	0,00	0,00	0,01
3774	73	1	Ciamis	200-500	Sengon	Paraserianthes falcataria (L.) I.C.	Leguminosae / Fabaceae	0,33	15,9	4,5	13,5	0,04	0,02	69,98	0,07	0,82	3,02
3775	73	1	Ciamis	200-500	Sengon	Paraserianthes falcataria (L.) I.C.	Leguminosae / Fabaceae	0,33	7,6	5	7	0,04	0,00	9,99	0,01	0,12	0,43
3776	73	1	Ciamis	200-500	Sengon	Paraserianthes falcataria (L.) I.C.	Leguminosae / Fabaceae	0,33	4,5	3,5	5	0,04	0,00	2,74	0,00	0,03	0,12
3777	73	1	Ciamis	200-500	Sengon	Paraserianthes falcataria (L.) I.C.	Leguminosae / Fabaceae	0,33	13,4	3,5	12,5	0,04	0,01	47,38	0,05	0,56	2,04
3778	73	1	Ciamis	200-500	Sengon	Paraserianthes falcataria (L.) I.C.	Leguminosae / Fabaceae	0,33	9,9	5	9	0,04	0,01	20,11	0,02	0,24	0,87
3779	73	1	Ciamis	200-500	Sengon	Paraserianthes falcataria (L.) I.C.	Leguminosae / Fabaceae	0,33	17,8	5	14	0,04	0,02	89,05	0,09	1,05	3,84
3780	73	1	Ciamis	200-500	Sengon	Paraserianthes falcataria (L.) I.C.	Leguminosae / Fabaceae	0,33	10,5	5	12	0,04	0,01	29,34	0,03	0,34	1,27
3781	73	1	Ciamis	200-500	Sengon	Paraserianthes falcataria (L.) I.C.	Leguminosae / Fabaceae	0,33	16,2	8	15	0,04	0,02	79,92	0,08	0,94	3,45
3782	74	2	Ciamis	200-500	Sengon	Paraserianthes falcataria (L.) I.C.	Leguminosae / Fabaceae	0,33	42,3	14	23	0,04	-	684,41	0,68	8,04	29,51
3783	74	2	Ciamis	200-500	Jati	Tectona grandis L.f.	Lamiaceae / Verbenaceae	0,7	7,6	3,5	6,5	0,04	0,00	18,60	0,02	0,22	0,80
3784	74	2	Ciamis	200-500	Tisuk	Hibiscus macrophyllus Roxb. ex I	Malvaceae	0,4	7,3	4	6	0,04	0,00	9,57	0,01	0,11	0,41
3785	74	2	Ciamis	200-500	Kelapa	Cocos nucifera L.	Arecaceae	0,5	28,6	9	10	0,04	0,06	81,50	0,08	0,96	3,51
3786	74	2	Ciamis	200-500	Durian	Durio zibethinus L.	Malvaceae	0,57	27,0	6	16	0,04	0,06	356,59	0,36	4,19	15,38
3787	74	2	Ciamis	200-500	Kelapa	Cocos nucifera L.	Arecaceae	0,5	28,0	12	15	0,04	0,06	120,00	0,12	1,41	5,17
3788	74	2	Ciamis	200-500	Jati	Tectona grandis L.f.	Lamiaceae / Verbenaceae	0,7	4,1	4	4,5	0,04	0,00	4,32	0,00	0,05	0,19
3789	74	2	Ciamis	200-500	Tisuk	Hibiscus macrophyllus Roxb. ex I	Malvaceae	0,4	8,9	8	11	0,04	0,01	23,92	0,02	0,28	1,03
3790	74	2	Ciamis	200-500	Tisuk	Hibiscus macrophyllus Roxb. ex I	Malvaceae	0,4	6,4	6	9	0,04	0,00	10,74	0,01	0,13	0,46
3791	74	2	Ciamis	200-500	Durian	Durio zibethinus L.	Malvaceae	0,57	5,1	3,5	5	0,04	0,00	5,76	0,01	0,07	0,25
3792	74	2	Ciamis	200-500	Jati	Tectona grandis L.f.	Lamiaceae / Verbenaceae	0,7	8,9	3,5	6,5	0,04	0,01	24,66	0,02	0,29	1,06
3793	74	2	Ciamis	200-500	Tisuk	Hibiscus macrophyllus Roxb. ex I	Malvaceae	0,4	16,9	12	15	0,04	0,02	102,28	0,10	1,20	4,41
3794	74	2	Ciamis	200-500	Jati	Tectona grandis L.f.	Lamiaceae / Verbenaceae	0,7	2,5	2	3	0,04	0,00	1,22	0,00	0,01	0,05
3795	74	2	Ciamis	200-500	Jati	Tectona grandis L.f.	Lamiaceae / Verbenaceae	0,7	11,5	8	15	0,04	0,01	84,08	0,08	0,99	3,63
3796	74	2	Ciamis	200-500	Jati	Tectona grandis L.f.	Lamiaceae / Verbenaceae	0,7	3,2	2	3	0,04	0,00	1,84	0,00	0,02	0,08
3797	74	2	Ciamis	200-500	Jati	Tectona grandis L.f.	Lamiaceae / Verbenaceae	0,7	3,2	2	3,5	0,04	0,00	2,12	0,00	0,02	0,09
3798	74	2	Ciamis	200-500	Jati	Tectona grandis L.f.	Lamiaceae / Verbenaceae	0,7	7,0	2	5,5	0,04	0,00	13,61	0,01	0,16	0,59
3799	74	2	Ciamis	200-500	Sengon	Paraserianthes falcataria (L.) I.C.	Leguminosae / Fabaceae	0,33	34,7	9	20	0,04	0,09	418,20	0,42	4,91	18,03
3800	74	2	Ciamis	200-500	Jati	Tectona grandis L.f.	Lamiaceae / Verbenaceae	0,7	4,1	1,6	2,6	0,04	0,00	2,61	0,00	0,03	0,11
3801	74	2	Ciamis	200-500	Durian	Durio zibethinus L.	Malvaceae	0,57	5,4	3,5	4,5	0,04	0,00	5,85	0,01	0,07	0,25
3802	74	2	Ciamis	200-500	Sengon	Paraserianthes falcataria (L.) I.C.	Leguminosae / Fabaceae	0,33	7,6	9	11	0,04	0,00	15,12	0,02	0,18	0,65
3803	74	2	Ciamis	200-500	Sengon	Paraserianthes falcataria (L.) I.C.	Leguminosae / Fabaceae	0,33	41,4	15	2,5	0,04	0,13	85,97	0,09	1,01	3,71
3804	74	2	Ciamis	200-500	Sengon	Paraserianthes falcataria (L.) I.C.	Leguminosae / Fabaceae	0,33	11,1	8	15	0,04	0,01	40,10	0,04	0,47	1,73
3805	74	2	Ciamis	200-500	Kelapa	Cocos nucifera L.	Arecaceae	0,5	29,3	16	19	0,04	0,07	150,80	0,15	1,77	6,50
3806	74	2	Ciamis	200-500	Tisuk	Hibiscus macrophyllus Roxb. ex I	Malvaceae	0,4	5,1	3	6,5	0,04	0,00	5,30	0,01	0,06	0,23
3807	74	2	Ciamis	200-500	Tisuk	Hibiscus macrophyllus Roxb. ex I	Malvaceae	0,4	23,5	18	24	0,04	0,04	289,94	0,29	3,41	12,50
3808	74	2	Ciamis	200-500	Kelapa	Cocos nucifera L.	Arecaceae	0,5	25,5	18	21	0,04	0,05	166,20	0,17	1,95	7,17
3809	74	2	Ciamis	200-500	Durian	Durio zibethinus L.	Malvaceae	0,57	3,2	1,5	3	0,04	0,00	1,53	0,00	0,02	0,07
3810	74	2	Ciamis	200-500	Tisuk	Hibiscus macrophyllus Roxb. ex I	Malvaceae	0,4	5,7	6	9	0,04	0,00	8,86	0,01	0,10	0,38
3811	74	2	Ciamis	200-500	Jati	Tectona grandis L.f.	Lamiaceae / Verbenaceae	0,7	28,6	6	18	0,04	0,06	532,39	0,53	6,26	22,96
3812	74	2	Ciamis	200-500	Sengon	Paraserianthes falcataria (L.) I.C.	Leguminosae / Fabaceae	0,33	44,9	10	22	0,04	0,16	731,32	0,73	8,59	31,54
3813	74	2	Ciamis	200-500	Jati	Tectona grandis L.f.	Lamiaceae / Verbenaceae	0,7	3,8	2	5	0,04	0,00	4,11	0,00	0,05	0,18
3814	74	2	Ciamis	200-500	Jati	Tectona grandis L.f.	Lamiaceae / Verbenaceae	0,7	6,7	4	7	0,04	0,00	15,58	0,02	0,18	0,67
3815	74	2	Ciamis	200-500	Jati	Tectona grandis L.f.	Lamiaceae / Verbenaceae	0,7	8,0	3,5	7,5	0,04	0,00	22,85	0,02	0,27	0,99
3816	74	2	Ciamis	200-500	Tisuk	Hibiscus macrophyllus Roxb. ex I	Malvaceae	0,4	11,5	6	12	0,04	0,01	41,05	0,04	0,48	1,77
3817	74	2	Ciamis	200-500	Durian	Durio zibethinus L.	Malvaceae	0,57	3,8	3,5	4,5	0,04	0,00	3,09	0,00	0,04	0,13
3818	74	2	Ciamis	200-500	Durian	Durio zibethinus L.	Malvaceae	0,57	4,8	3	5	0,04	0,00	5,12	0,01	0,06	0,22
3819	74	2	Ciamis	200-500	Durian	Durio zibethinus L.	Malvaceae	0,57	4,1	2	4	0,04	0,00	3,21	0,00	0,04	0,14
3820	74	2	Ciamis	200-500	Kelapa	Cocos nucifera L.	Arecaceae	0,5	31,8	14	19	0,04	0,08	150,80	0,15	1,77	6,50
3821	74	2	Ciamis	200-500	Durian	Durio zibethinus L.	Malvaceae	0,57	9,9	7	10	0,04	0,01	36,53	0,04	0,43	1,58
3822	74	2	Ciamis	200-500	Dukuh	Lansium domesticum Corrêa	Meliaceae	0,85	2,9	2	3	0,04	0,00	1,81	0,00	0,02	0,08
3823	74	2	Ciamis	200-500	Dukuh	Lansium domesticum Corrêa	Meliaceae	0,85	2,9	2	3,5	0,04	0,00	2,09	0,00	0,02	0,09
3824	75	3	Ciamis	200-500	Mahoni	Swietenia macrophylla King	Meliaceae	0,61	14,0	6	13	0,04	-	93,90	0,09	1,10	4,05
3825	75	3	Ciamis	200-500	Kemiri	Aleurites moluccanus (L.) Willd.	Euphorbiaceae	0,31	21,0	9	15	0,04	0,03	120,00	0,12	1,41	5,17
3826	75	3	Ciamis	200-500	Kokosan	Lansium domesticum var. aquae	Meliaceae	0,7	4,1	2,5	5	0,04	0,00	4,76	0,00	0,06	0,21
3827	75	3	Ciamis	200-500	Gamal/Kalkiria	Gliricidia sepium (Jacq.) Walp.	Leguminosae / Fabaceae	0,58	15,3	4	13	0,04	0,02	105,15	0,11	1,24	4,53
3828	75	3	Ciamis	200-500	Sengon	Paraserianthes falcataria (L.) I.C.	Leguminosae / Fabaceae	0,33	12,4	14	18	0,04	0,01	57,77	0,06	0,68	2,49
3829	75	3	Ciamis	200-500	Tisuk	Hibiscus macrophyllus Roxb. ex I	Malvaceae	0,4	19,7	15	18	0,04	0,03	161,10	0,16	1,89	6,95
3830	75	3	Ciamis	200-500	Jengkol	Archidendron pauciflorum (Bentl	Leguminosae / Fabaceae	0,4	23,9	10	17	0,04	0,04	216,68	0,22	2,55	9,34
3831	75	3	Ciamis	200-500	Petai	Parkia speciosa Hassk.	Leguminosae / Fabaceae	0,45	29,0	15	23	0,04	0,07	453,70	0,45	5,33	19,56
3832	75	3	Ciamis	200-500	Kopi	Coffea canephora Pierre ex A.Frc	Rubiaceae	0,41	9,9	1,5	4,5	0,04	0,01	30,65	0,03	0,36	1,32
3833	75	3	Ciamis	200-500	Kokosan	Lansium domesticum var. aquae	Meliaceae	0,7	7,6	5	8	0,04	0,00	22,49	0,02	0,26	0,97
3834	75	3	Ciamis	200-500	Dukuh	Lansium domesticum Corrêa	Meliaceae	0,85	9,2	4	7	0,04	0,01	33,63	0,03	0,40	1,45
3835	75	3	Ciamis	200-500	Dukuh	Lansium domesticum Corrêa	Meliaceae	0,85	7,6	4	8	0,04	0,00	26,87	0,03	0,32	1,16
3836	75	3	Ciamis	200-500	Tisuk	Hibiscus macrophyllus Roxb. ex I	Malvaceae	0,4	38,5	10	18	0,04	0,12	548,40	0,55	6,44	23,65
3837	75	3	Ciamis	200-500	Rambutan	Nephelium lappaceum L.	Sapindaceae	0,71	11,5	6	12	0,04	0,01	69,43	0,07	0,82	2,99
3838	75	3	Ciamis	200-500	Tisuk	Hibiscus macrophyllus Roxb. ex I	Malvaceae	0,4	9,9	8	14	0,04	0,01	35,94	0,04	0,42	1,55

3839	75	3	Ciamis	200-500	Tisuk	Hibiscus macrophyllus Roxb. ex	Malvaceae	0,4	17,8	10	16	0,04	0,02	120,02	0,12	1,41	5,18
3840	75	3	Ciamis	200-500	Mahoni	Swietenia macrophylla King	Meliaceae	0,61	11,5	5	11	0,04	0,01	55,79	0,06	0,66	2,41
3841	75	3	Ciamis	200-500	Mahoni	Swietenia macrophylla King	Meliaceae	0,61	9,9	5	10	0,04	0,01	38,87	0,04	0,46	1,68
3842	75	3	Ciamis	200-500	Gamal/Kalkiria	Gliricidia sepium (Jacq.) Walp.	Leguminosae / Fabaceae	0,58	37,5	6	16	0,04	0,11	660,82	0,66	7,76	28,50
3843	75	3	Ciamis	200-500	Gamal/Kalkiria	Gliricidia sepium (Jacq.) Walp.	Leguminosae / Fabaceae	0,58	35,6	4	16	0,04	0,10	600,57	0,60	7,06	25,90
3844	75	3	Ciamis	200-500	Gamal/Kalkiria	Gliricidia sepium (Jacq.) Walp.	Leguminosae / Fabaceae	0,58	36,3	3	12	0,04	0,10	476,65	0,48	5,60	20,55
3845	75	3	Ciamis	200-500	Nangka	Artocarpus heterophyllus Lam.	Moraceae	0,61	25,1	11	15	0,04	0,05	312,78	0,31	3,68	13,49
3846	75	3	Ciamis	200-500	Namnam/Pukih	Cynometra cauliflora L.	Leguminosae / Fabaceae	0,72	8,3	5	8	0,04	0,01	26,72	0,03	0,31	1,15
3847	76	1	Ciamis	200-500	Sengon	Paraserianthes falcataria (L.) I.C	Leguminosae / Fabaceae	0,33	8,6	9	11	0,01	-	18,76	0,02	0,88	3,24
3848	76	1	Ciamis	200-500	Sengon	Paraserianthes falcataria (L.) I.C	Leguminosae / Fabaceae	0,33	6,0	8	9	0,01	0,00	8,20	0,01	0,39	1,41
3849	76	1	Ciamis	200-500	Sengon	Paraserianthes falcataria (L.) I.C	Leguminosae / Fabaceae	0,33	3,5	4,5	5,5	0,01	0,00	1,92	0,00	0,09	0,33
3850	76	1	Ciamis	200-500	Durian	Durio zibethinus L.	Malvaceae	0,57	3,5	1	3	0,01	0,00	1,82	0,00	0,09	0,31
3851	76	1	Ciamis	200-500	Sengon	Paraserianthes falcataria (L.) I.C	Leguminosae / Fabaceae	0,33	17,5	7	13	0,01	0,01	80,50	0,08	3,78	13,89
3852	76	1	Ciamis	200-500	Sengon	Paraserianthes falcataria (L.) I.C	Leguminosae / Fabaceae	0,33	3,8	5	6	0,01	0,00	2,44	0,00	0,11	0,42
3853	76	1	Ciamis	200-500	Sengon	Paraserianthes falcataria (L.) I.C	Leguminosae / Fabaceae	0,33	3,5	5	6	0,01	0,00	2,08	0,00	0,10	0,36
3854	76	1	Ciamis	200-500	Sengon	Paraserianthes falcataria (L.) I.C	Leguminosae / Fabaceae	0,33	2,9	3	4	0,01	0,00	0,99	0,00	0,05	0,17
3855	76	1	Ciamis	200-500	Sengon	Paraserianthes falcataria (L.) I.C	Leguminosae / Fabaceae	0,33	1,9	1,5	2	0,01	0,00	0,25	0,00	0,01	0,04
3856	76	1	Ciamis	200-500	Sengon	Paraserianthes falcataria (L.) I.C	Leguminosae / Fabaceae	0,33	2,2	3,5	5	0,01	0,00	0,77	0,00	0,04	0,13
3857	76	1	Ciamis	200-500	Sengon	Paraserianthes falcataria (L.) I.C	Leguminosae / Fabaceae	0,33	5,4	7	8	0,01	0,00	6,00	0,01	0,28	1,04
3858	76	1	Ciamis	200-500	Sengon	Paraserianthes falcataria (L.) I.C	Leguminosae / Fabaceae	0,33	1,9	3,5	4	0,01	0,00	0,47	0,00	0,02	0,08
3859	76	1	Ciamis	200-500	Sengon	Paraserianthes falcataria (L.) I.C	Leguminosae / Fabaceae	0,33	13,4	7	11	0,01	0,00	42,15	0,04	1,98	7,27
3860	76	1	Ciamis	200-500	Sengon	Paraserianthes falcataria (L.) I.C	Leguminosae / Fabaceae	0,33	4,1	4	5,5	0,01	0,00	2,61	0,00	0,12	0,45
3861	76	1	Ciamis	200-500	Sengon	Paraserianthes falcataria (L.) I.C	Leguminosae / Fabaceae	0,33	2,9	3,5	4	0,01	0,00	0,99	0,00	0,05	0,17
3862	76	1	Ciamis	200-500	Dukuh	Lansium domesticum Corrêa	Meliaceae	0,85	1,9	4,5	5,5	0,01	0,00	1,50	0,00	0,07	0,26
3863	76	1	Ciamis	200-500	Dukuh	Lansium domesticum Corrêa	Meliaceae	0,85	3,5	3	5	0,01	0,00	4,18	0,00	0,20	0,72
3864	76	1	Ciamis	200-500	Sengon	Paraserianthes falcataria (L.) I.C	Leguminosae / Fabaceae	0,33	1,9	4	5,5	0,01	0,00	0,63	0,00	0,03	0,11
3865	76	1	Ciamis	200-500	Sengon	Paraserianthes falcataria (L.) I.C	Leguminosae / Fabaceae	0,33	1,9	3	3,5	0,01	0,00	0,42	0,00	0,02	0,07
3866	76	1	Ciamis	200-500	Sengon	Paraserianthes falcataria (L.) I.C	Leguminosae / Fabaceae	0,33	3,2	5	6	0,01	0,00	1,75	0,00	0,08	0,30
3867	76	1	Ciamis	200-500	Sengon	Paraserianthes falcataria (L.) I.C	Leguminosae / Fabaceae	0,33	8,6	5	9	0,01	0,00	15,61	0,02	0,73	2,69
3868	76	1	Ciamis	200-500	Sengon	Paraserianthes falcataria (L.) I.C	Leguminosae / Fabaceae	0,33	7,6	9	10,5	0,01	0,00	14,49	0,01	0,68	2,50
3869	76	1	Ciamis	200-500	Sengon	Paraserianthes falcataria (L.) I.C	Leguminosae / Fabaceae	0,33	3,8	2,5	4	0,01	0,00	1,68	0,00	0,08	0,29
3870	76	1	Ciamis	200-500	Sengon	Paraserianthes falcataria (L.) I.C	Leguminosae / Fabaceae	0,33	8,9	8	10	0,01	0,00	18,38	0,02	0,86	3,17
3871	76	1	Ciamis	200-500	Sengon	Paraserianthes falcataria (L.) I.C	Leguminosae / Fabaceae	0,33	7,0	6	13	0,01	0,00	15,02	0,02	0,71	2,59
3872	76	1	Ciamis	200-500	Sengon	Paraserianthes falcataria (L.) I.C	Leguminosae / Fabaceae	0,33	4,5	8	9	0,01	0,00	4,69	0,00	0,22	0,81
3873	76	1	Ciamis	200-500	Sengon	Paraserianthes falcataria (L.) I.C	Leguminosae / Fabaceae	0,33	1,9	3,5	7,54	0,01	0,00	0,84	0,00	0,04	0,15
3874	76	1	Ciamis	200-500	Sengon	Paraserianthes falcataria (L.) I.C	Leguminosae / Fabaceae	0,33	10,2	8,5	10	0,01	0,00	23,47	0,02	1,10	4,05
3875	76	1	Ciamis	200-500	Sengon	Paraserianthes falcataria (L.) I.C	Leguminosae / Fabaceae	0,33	3,2	3	4	0,01	0,00	1,20	0,00	0,06	0,21
3876	76	1	Ciamis	200-500	Sengon	Paraserianthes falcataria (L.) I.C	Leguminosae / Fabaceae	0,33	6,0	9	10	0,01	0,00	9,03	0,01	0,42	1,56
3877	76	1	Ciamis	200-500	Dukuh	Lansium domesticum Corrêa	Meliaceae	0,85	2,5	2	3,5	0,01	0,00	1,68	0,00	0,08	0,29
3878	76	1	Ciamis	200-500	Sengon	Paraserianthes falcataria (L.) I.C	Leguminosae / Fabaceae	0,33	4,8	6	7	0,01	0,00	4,22	0,00	0,20	0,73
3879	76	1	Ciamis	200-500	Sengon	Paraserianthes falcataria (L.) I.C	Leguminosae / Fabaceae	0,33	10,2	8	11	0,01	0,00	25,61	0,03	1,20	4,42
3880	76	1	Ciamis	200-500	Sengon	Paraserianthes falcataria (L.) I.C	Leguminosae / Fabaceae	0,33	6,7	10	11	0,01	0,00	11,84	0,01	0,56	2,04
3881	76	1	Ciamis	200-500	Sengon	Paraserianthes falcataria (L.) I.C	Leguminosae / Fabaceae	0,33	1,9	2	3	0,01	0,00	0,36	0,00	0,02	0,06
3882	76	1	Ciamis	200-500	Sengon	Paraserianthes falcataria (L.) I.C	Leguminosae / Fabaceae	0,33	8,6	9	12	0,01	0,00	20,32	0,02	0,95	3,50
3883	76	1	Ciamis	200-500	Sengon	Paraserianthes falcataria (L.) I.C	Leguminosae / Fabaceae	0,33	5,1	8	9,5	0,01	0,00	6,29	0,01	0,30	1,08
3884	76	1	Ciamis	200-500	Sengon	Paraserianthes falcataria (L.) I.C	Leguminosae / Fabaceae	0,33	2,5	2,5	4,5	0,01	0,00	0,89	0,00	0,04	0,15
3885	76	1	Ciamis	200-500	Sengon	Paraserianthes falcataria (L.) I.C	Leguminosae / Fabaceae	0,33	8,9	8	9,5	0,01	0,00	17,53	0,02	0,82	3,02
3886	76	1	Ciamis	200-500	Sengon	Paraserianthes falcataria (L.) I.C	Leguminosae / Fabaceae	0,33	7,0	7	8	0,01	0,00	9,63	0,01	0,45	1,66
3887	77	2	Ciamis	200-500	Mahoni	Swietenia macrophylla King	Meliaceae	0,61	8,3	2,5	6	0,04	-	17,64	0,02	0,21	0,76
3888	77	2	Ciamis	200-500	Jati	Tectona grandis L.f.	Lamiaceae / Verbenaceae	0,7	17,8	2,5	9,5	0,04	0,02	124,31	0,12	1,46	5,36
3889	77	2	Ciamis	200-500	Mahoni	Swietenia macrophylla King	Meliaceae	0,61	18,1	4	9	0,04	0,03	107,73	0,11	1,27	4,65
3890	77	2	Ciamis	200-500	Jati	Tectona grandis L.f.	Lamiaceae / Verbenaceae	0,7	3,5	2	5	0,04	0,00	3,50	0,00	0,04	0,15
3891	77	2	Ciamis	200-500	Mangga	Mangifera indica L.	Anacardiaceae	0,6	8,6	4	6	0,04	0,01	18,62	0,02	0,22	0,80
3892	77	2	Ciamis	200-500	Jati	Tectona grandis L.f.	Lamiaceae / Verbenaceae	0,7	6,7	3	5	0,04	0,00	11,45	0,01	0,13	0,49
3893	77	2	Ciamis	200-500	Jati	Tectona grandis L.f.	Lamiaceae / Verbenaceae	0,7	9,5	3	7	0,04	0,01	29,95	0,03	0,35	1,29
3894	77	2	Ciamis	200-500	Jati	Tectona grandis L.f.	Lamiaceae / Verbenaceae	0,7	15,9	5	11	0,04	0,02	115,52	0,12	1,36	4,98
3895	77	2	Ciamis	200-500	Sengon	Paraserianthes falcataria (L.) I.C	Leguminosae / Fabaceae	0,33	6,0	17	9	0,04	0,00	8,20	0,01	0,10	0,35
3896	77	2	Ciamis	200-500	Durian	Durio zibethinus L.	Malvaceae	0,57	2,5	1,5	2,5	0,04	0,00	0,86	0,00	0,01	0,04
3897	77	2	Ciamis	200-500	Sengon	Paraserianthes falcataria (L.) I.C	Leguminosae / Fabaceae	0,33	24,8	13	18	0,04	0,05	205,70	0,21	2,42	8,87
3898	77	2	Ciamis	200-500	Sengon	Paraserianthes falcataria (L.) I.C	Leguminosae / Fabaceae	0,33	19,4	12	17	0,04	0,03	124,42	0,12	1,46	5,37
3899	77	2	Ciamis	200-500	Kokosan	Lansium domesticum var. aquae	Meliaceae	0,7	19,1	5	8	0,04	0,03	120,51	0,12	1,42	5,20
3900	77	2	Ciamis	200-500	Kokosan	Lansium domesticum var. aquae	Meliaceae	0,7	9,5	4	8	0,04	0,01	33,85	0,03	0,40	1,46
3901	77	2	Ciamis	200-500	Kokosan	Lansium domesticum var. aquae	Meliaceae	0,7	9,5	3	7	0,04	0,01	29,95	0,03	0,35	1,29
3902	77	2	Ciamis	200-500	Tisuk	Hibiscus macrophyllus Roxb. ex	Malvaceae	0,4	6,4	5	7	0,04	0,00	8,53	0,01	0,10	0,37
3903	77	2	Ciamis	200-500	Tisuk	Hibiscus macrophyllus Roxb. ex	Malvaceae	0,4	10,5	8	11	0,04	0,01	32,32	0,03	0,38	1,39
3904	77	2	Ciamis	200-500	Sawo / sawo manila	Manilkara zapota (L.) P.Royen	Sapotaceae	0,81	3,8	0,5	2,5	0,04	0,00	2,49	0,00	0,03	0,11
3905	77	2	Ciamis	200-500	Sengon	Paraserianthes falcataria (L.) I.C	Leguminosae / Fabaceae	0,33	15,0	17	23	0,04	0,02	101,79	0,10	1,20	4,39
3906	77	2	Ciamis	200-500	Sengon	Paraserianthes falcataria (L.) I.C	Leguminosae / Fabaceae	0,33	33,1	12	22	0,04	0,09	418,74	0,42	4,92	18,06
3907	77	2	Ciamis	200-500	Dukuh	Lansium domesticum Corrêa	Meliaceae	0,85	5,4	1,5	5,5	0,04	0,00	10,13	0,01	0,12	0,44
3908	77	2	Ciamis	200-500	Nangka	Artocarpus heterophyllus Lam.	Moraceae	0,61	12,1	3	5	0,04	0,01	29,92	0,03	0,35	1,29
3909	77	2	Ciamis	200-500	Mahoni	Swietenia macrophylla King	Meliaceae	0,61	12,4	4	7	0,04	0,01	42,70	0,04	0,50	1,84
3910	77	2	Ciamis	200-500	Mahoni	Swietenia macrophylla King	Meliaceae	0,61	16,2	4	9	0,04	0,02	87,87	0,09	1,03	3,79
3911	77	2	Ciamis	200-500	Sengon	Paraserianthes falcataria (L.) I.C	Leguminosae / Fabaceae	0,33	15,9	9	1,5	0,04	0,02	9,35	0,01	0,11	0,40
3912	77	2	Ciamis	200-500	Nangka	Artocarpus heterophyllus Lam.	Moraceae	0,61	3,2	1,5	3	0,04	0,00	1,62	0,00	0,02	0,07
3913	77	2	Ciamis	200-500	Limus	Mangifera foetida Lour.	Anacardiaceae	0,73	4,1	3	4,5	0,04	0,00	4,49	0,00	0,05	0,19
3914	77	2	Ciamis	200-500	Mahoni	Swietenia macrophylla King	Meliaceae	0,61	10,8	4	8	0,04	0,01	37,53	0,04	0,44	1,62
3915	77	2	Ciamis	200-500	Sengon	Paraserianthes falcataria (L.) I.C	Leguminosae / Fabaceae	0,33	11,1	17	22	0,04	0,01	56,95	0,06	0,67	2,46
3916	77	2	Ciamis	200-500	Rambutan	Nephelium lappaceum L.	Sapindaceae	0,71	3,8	2,5	5,5	0,04	0,00	4,54	0,00	0,05	0,20
3917	77	2	Ciamis	200-500	Durian	Durio zibethinus L.	Malvaceae	0,57	2,5	1,5	2	0,04	0,00	0,70	0,00	0,01	0,03
3918	77	2	Ciamis	200-500	Tisuk	Hibiscus macrophyllus Roxb. ex	Malvaceae	0,4	5,1	4	7	0,04	0,00	5,67	0,01	0,07	0,24
3919	77	2	Ciamis	200-500	Tisuk	Hibiscus macrophyllus Roxb. ex	Malvaceae	0,4	5,7	6	8	0,04	0,00	7,95	0,01	0,09	0,34
3920	77	2	Ciamis	200-500	Sengon	Paraserianthes falcataria (L.) I.C	Leguminosae / Fabaceae	0,33	12,4	19	22	0,04	0,01	69,43	0,07	0,82	2,99
3921	77	2	Ciamis	200-500	Kokosan	Lansium domesticum var. aquae	Meliaceae	0,7	6,0	4	7	0,04	0,00	12,97	0,01	0,15	0,56
3922	77	2	Ciamis	200-500	Jati	Tectona grandis L.f.	Lamiaceae / Verbenaceae	0,7	9,5	4	8	0,04	0,01	33,85	0,03	0,40	1,46
3923	77	2	Ciamis	200-500	Tisuk	Hibiscus macrophyllus Roxb. ex	Malvaceae	0,4	5,1	6	7	0,04	0,00	5,67	0,01	0,07	0,24
3924	77	2	Ciamis	200-500	Sengon	Paraserianthes falcataria (L.) I.C	Leguminosae / Fabaceae	0,33	15,0	11	17	0,04	0,02	77,17	0,08	0,91	3,33
3925	77	2	Ciamis	200-500	Rasamala	Altingia excelsa Noronha	Altingiaceae	0,81	8,0	4	7	0,04	0,00	24,51	0,02	0,29	1,06
3926	77	2	Ciamis	200-500	Sengon	Paraserianthes falcataria (L.) I.C	Leguminosae / Fabaceae	0,33	12,4	10	13	0,04	0,01	42,88	0,04	0,50	1,85
3927	77	2	Ciamis	200-500	Manglid	Magnolia champaca (L.) Baill. ex	Magnoliaceae	0,53	4,1	2	4,5	0,04	0,00	3,35	0,00	0,04	0,14
3928	77	2	Ciamis	200-500	Sengon	Paraserianthes falcataria (L.) I.C	Leguminosae / Fabaceae	0,33	12,4	10	13	0,04	0,01	42,88	0,04	0,50	1,85
3929	77	2	Ciamis	200-500	Kokosan	Lansium domesticum var. aquae	Meliaceae	0,7	4,1	3	5	0,04	0,00	4,76	0,00	0,06	0,21
3930	77	2	Ciamis	200-500	Rambutan	Nephelium lappaceum L.	Sapindaceae	0,71	10,8	3,5	8,5	0,04	0,01	45,59	0,05	0,54	1,97
3931	77	2	Ciamis	200-500	Dukuh	Lansium domesticum Corrêa	Meliaceae	0,85	5,1	4	6	0,04	0,00	9,82	0,01	0,12	0,42
3932	77	2	Ciamis	200-500	Renghas	Gluta renghas L.	Anacardiaceae	0,69	6,0	2	5	0,04	0,00	9,41	0,01	0,11	0,41
3933	77	2	Ciamis	200-500	Tisuk	Hibiscus macrophyllus Roxb. ex	Malvaceae	0,4	5,1	5	7	0,04	0,00	5,67	0,01	0,07	0,24
3934	77	2	Ciamis	200-500	Kokosan	Lansium domesticum var. aquae	Meliaceae	0,7	5,1	3,5	5,5	0,04	0,00	7,59	0,01	0,09	0,33
3935	77	2	Ciamis	200-500	Nangka	Artocarpus heterophyllus Lam.	Moraceae	0,61	7,3	2,5	5,5	0,04	0,00	13,01	0,01	0,15	0,56
3936	77	2	Ciamis	200-500	Tisuk	Hibiscus macrophyllus Roxb. ex	Malvaceae	0,4	7,0	7	9,5	0,04	0,00	13,44	0,01	0,16	0,58
3937	77	2	Ciamis	200-500	Tisuk	Hibiscus macrophyllus Roxb. ex	Malvaceae	0,4	4,1	3,5	4,5	0,04	0,00	2,59	0,00	0,03	0,11
3938	77	2	Ciamis	200-500	Sengon	Paraserianthes falcataria (L.) I.C	Leguminosae / Fabaceae	0,33	20,4	10	16	0,04	0,03	128,52	0,13	1,51	5,54
3939	77	2	Ciamis	200-500	Tisuk	Hibiscus macrophyllus Roxb. ex	Malvaceae	0,4	3,2	3,5	4,5	0,04	0,00	1,60	0,00	0,02	0,07
3940	77	2	Ciamis	200-500	Tisuk	Hibiscus macrophyllus Roxb. ex	Malvaceae	0,4	7,0	7	9	0,04	0,00	12,79	0,01	0,15	0,55
3941	77	2	Ciamis	200-500	Kokosan	Lansium domesticum var. aquae	Meliaceae	0,7	3,8	2,5	5,5	0,04	0,00	4,48	0,00	0,05	0,19
3942	77	2	Ciamis	200-500	Sengon	Paraserianthes falcataria (L.) I.C	Leguminosae / Fabaceae	0,33	34,0	11	20	0,04	0,09	404,25	0,40	4,75	17,43
3943	77	2	Ciamis	200-500	Tisuk	Hibiscus macrophyllus Roxb. ex	Malvaceae	0,4	3,2	3,5	4,5	0,04	0,00	1,60	0,00	0,02	0,07
3944	77	2	Ciamis	200-500	Tisuk	Hibiscus macrophyllus Roxb. ex	Malvaceae	0,4	5,4	4,5	7,5	0,04	0,00	6,75	0,01	0,08	0,29
3945	77	2	Ciamis	200-500	Namnam/Pukih	Cynometra cauliflora L.	Leguminosae / Fabaceae	0,72	10,2	1,5	3,5	0,04	0,01	18,33	0,02	0,22	0,79
3946	77	2	Ciamis	200-500	Aren	Arenga pinnata (Wurmb) Merr.	Arecaceae	0,71	44,5	0,5	4,5	0,04	0,16	39,15	0,04	0,46	1,69
3947	77	2	Ciamis	200-500	Kokosan	Lansium domesticum var. aquae	Meliaceae	0,7	15,0	4	8	0,04	0,02	77,04	0,08	0,91	3,32
3948	77	2	Ciamis	200-500	Sengon	Paraserianthes falcataria (L.) I.C	Leguminosae / Fabaceae	0,33	22,3	10	19	0,04	0,04	177,27	0,18	2,08	7,64
3949	77	2	Ciamis	200-500	Tisuk	Hibiscus macrophyllus Roxb. ex	Malvaceae	0,4	6,0	5	7,5	0,04	0,00	8,28	0,01	0,10	0,36
3950	77	2	Ciamis	200-500	Samolo / bisbul	Diospyros discolor Willd.	Ebenaceae	0,88	3,8	3	4,5	0,04	0,00	4,60	0,00	0,05	0,20
3951	77	2	Ciamis	200-500	Tisuk	Hibiscus macrophyllus Roxb. ex	Malvaceae	0,4	3,8	7	9	0,04	0,00	4,21	0,00	0,05	0,18
3952	77	2	Ciamis	200-500	Mahoni	Swietenia macrophylla King	Meliaceae	0,61	9,9	3,5	7	0,04	0,01	28,04	0,03	0,33	1,21
3953	77	2	Ciamis	200-500	Mahoni	Swietenia macrophylla King	Meliaceae	0,61	9,5	3,5	7,5	0,04	0,01	28,13	0,03	0,33	1,21
3954	77	2	Ciamis	200-500	Mahoni	Swietenia macrophylla King	Meliaceae	0,61	9,5	3,5	7,5	0,04	0,01	28,13	0,03	0,33	1,21
3955	77	2	Ciamis	200-500	Jati	Tectona grandis L.f.	Lamiaceae / Verbenaceae	0,7	28,3	3,5	11,5	0,04	0,06	346,03	0,35	4,07	14,92
3956	77	2	Ciamis	200-500	Mahoni	Swietenia macrophylla King	Meliaceae	0,61	18,8	8	11	0,04	0,03	137,91	0,14	1,62	5,95
3957	77	2	Ciamis	200-500	Mahoni	Swietenia macrophylla King	Meliaceae	0,61	18,5	8	11	0,04	0,03	133,66	0,13	1,57	5,76
3958	77	2	Ciamis	200-500	Mahoni	Swietenia macrophylla King	Meliaceae	0,61	18,5	5	12	0,04	0,03	144,75	0,14	1,70	6,24
3959	77	2	Ciamis	200-500	Jengkol	Archidendron pauciflorum (Bent	Leguminosae / Fabaceae	0,4	8,9	2,5	3,5	0,04	0,01	8,38	0,01	0,10	0,36
3960	77	2	Ciamis	200-500	Kokosan	Lansium domesticum var. aquae	Meliaceae	0,7	2,9	3,5	5	0,04	0,00	2,42	0,00	0,03	0,10
3961	77	2	Ciamis	200-500	Kokosan	Lansium domesticum var. aquae	Meliaceae	0,7	2,2	2	3	0,04	0,00	0,96	0,00	0,01	0,04
3962	77	2	Ciamis	200-500	Kokosan	Lansium domesticum var. aquae	Meliaceae	0,7	3,8	3,5	5	0,04	0,00	4,11	0,00	0,05	0,18

3963	77	2	Ciamis	200-500	Aren	Arenga pinnata (Wurmb) Merr.	Arecaceae		0,71	28,0	4	7	0,04	0,06	58,40	0,06	0,69	2,52
3964	77	2	Ciamis	200-500	Mahoni	Swietenia macrophylla King	Meliaceae		0,61	22,6	3	10	0,04	0,04	177,41	0,18	2,08	7,65
3965	77	2	Ciamis	200-500	Mahoni	Swietenia macrophylla King	Meliaceae		0,61	4,1	2,5	4	0,04	0,00	3,42	0,00	0,04	0,15
3966	77	2	Ciamis	200-500	Mahoni	Swietenia macrophylla King	Meliaceae		0,61	21,6	3,5	11,5	0,04	0,04	186,31	0,19	2,19	8,03
3967	77	2	Ciamis	200-500	Petai	Parkia speciosa Hassk.	Leguminosae / Fabaceae		0,45	6,7	3,5	6,5	0,04	0,00	9,71	0,01	0,11	0,42
3968	77	2	Ciamis	200-500	Mahoni	Swietenia macrophylla King	Meliaceae		0,61	24,8	10	15	0,04	0,05	305,56	0,31	3,59	13,18
3969	77	2	Ciamis	200-500	Sirsak	Annona muricata L.	Annonaceae		0,36	6,7	4	7	0,04	0,00	8,47	0,01	0,10	0,37
3970	77	2	Ciamis	200-500	Sirsak	Annona muricata L.	Annonaceae		0,36	6,7	3,5	6,5	0,04	0,00	7,92	0,01	0,09	0,34
3971	77	2	Ciamis	200-500	Mahoni	Swietenia macrophylla King	Meliaceae		0,61	2,9	3	4	0,04	0,00	1,74	0,00	0,02	0,08
3972	77	2	Ciamis	200-500	Mahoni	Swietenia macrophylla King	Meliaceae		0,61	17,8	3	7	0,04	0,02	82,85	0,08	0,97	3,57
3973	78	2	Ciamis	200-500	Kokosan	Lansium domesticum var. aquae	Meliaceae		0,7	6,7	4	7	0,04	-	15,58	0,02	0,18	0,67
3974	78	2	Ciamis	200-500	Manglid	Magnolia champaca (L.) Baill. ex	Magnoliaceae		0,53	12,1	5	9	0,04	0,01	45,06	0,05	0,53	1,94
3975	78	2	Ciamis	200-500	Sp8	Sp8		0	0,5	4,1	2,5	3,5	0,04	0,00	2,52	0,00	0,03	0,11
3976	78	2	Ciamis	200-500	Tisuk	Hibiscus macrophyllus Roxb. ex I	Malvaceae		0,4	15,3	10	13	0,04	0,02	74,82	0,07	0,88	3,23
3977	78	2	Ciamis	200-500	Manglid	Magnolia champaca (L.) Baill. ex	Magnoliaceae		0,53	8,9	7	11	0,04	0,01	30,95	0,03	0,36	1,33
3978	78	2	Ciamis	200-500	Durian	Durio zibethinus L.	Malvaceae		0,57	4,5	1,5	3	0,04	0,00	2,83	0,00	0,03	0,12
3979	78	2	Ciamis	200-500	Sp8	Sp8		0	0,5	8,9	10	11	0,04	0,01	29,34	0,03	0,34	1,27
3980	78	2	Ciamis	200-500	Durian	Durio zibethinus L.	Malvaceae		0,57	2,5	1,5	3	0,04	0,00	1,01	0,00	0,01	0,04
3981	78	2	Ciamis	200-500	Durian	Durio zibethinus L.	Malvaceae		0,57	4,1	1,5	3,5	0,04	0,00	2,84	0,00	0,03	0,12
3982	78	2	Ciamis	200-500	Pangsor	Ficus callosa Willd.	Moraceae		0,33	4,8	6,5	7,5	0,04	0,00	4,50	0,00	0,05	0,19
3983	78	2	Ciamis	200-500	Mahoni	Swietenia macrophylla King	Meliaceae		0,61	5,4	3,5	5,5	0,04	0,00	7,48	0,01	0,09	0,32
3984	78	2	Ciamis	200-500	Pangsor	Ficus callosa Willd.	Moraceae		0,33	5,1	5	6,5	0,04	0,00	4,44	0,00	0,05	0,19
3985	78	2	Ciamis	200-500	Pangsor	Ficus callosa Willd.	Moraceae		0,33	21,6	13	20	0,04	0,04	176,19	0,18	2,07	7,60
3986	78	2	Ciamis	200-500	Pangsor	Ficus callosa Willd.	Moraceae		0,33	13,4	11	12,5	0,04	0,01	47,38	0,05	0,56	2,04
3987	78	2	Ciamis	200-500	Sengon	Paraserianthes falcataria (L.) I.C.	Leguminosae / Fabaceae		0,33	28,6	15	20	0,04	0,06	294,44	0,29	3,46	12,70
3988	78	2	Ciamis	200-500	Mahoni	Swietenia macrophylla King	Meliaceae		0,61	8,0	3	7	0,04	0,00	18,91	0,02	0,22	0,82
3989	78	2	Ciamis	200-500	Pangsor	Ficus callosa Willd.	Moraceae		0,33	6,7	7	8	0,04	0,00	8,84	0,01	0,10	0,38
3990	78	2	Ciamis	200-500	Pangsor	Ficus callosa Willd.	Moraceae		0,33	18,8	15	19	0,04	0,03	129,60	0,13	1,52	5,59
3991	78	2	Ciamis	200-500	Mahoni	Swietenia macrophylla King	Meliaceae		0,61	6,7	2,5	6,5	0,04	0,00	12,84	0,01	0,15	0,55
3992	78	2	Ciamis	200-500	Pangsor	Ficus callosa Willd.	Moraceae		0,33	15,3	12	16	0,04	0,02	75,87	0,08	0,89	3,27
3993	78	2	Ciamis	200-500	Mahoni	Swietenia macrophylla King	Meliaceae		0,61	12,7	6	13	0,04	0,01	78,85	0,08	0,93	3,40
3994	78	2	Ciamis	200-500	Sengon	Paraserianthes falcataria (L.) I.C.	Leguminosae / Fabaceae		0,33	20,0	12	19	0,04	0,03	146,15	0,15	1,72	6,30
3995	78	2	Ciamis	200-500	Pangsor	Ficus callosa Willd.	Moraceae		0,33	10,5	10	14	0,04	0,01	33,79	0,03	0,40	1,46
3996	78	2	Ciamis	200-500	Pangsor	Ficus callosa Willd.	Moraceae		0,33	14,3	14	17	0,04	0,02	71,26	0,07	0,84	3,07
3997	78	2	Ciamis	200-500	Mahoni	Swietenia macrophylla King	Meliaceae		0,61	15,9	8	12	0,04	0,02	110,29	0,11	1,30	4,76
3998	78	2	Ciamis	200-500	Nangka	Artocarpus heterophyllus Lam.	Moraceae		0,61	9,9	4	8	0,04	0,01	31,69	0,03	0,37	1,37
3999	78	2	Ciamis	200-500	Pangsor	Ficus callosa Willd.	Moraceae		0,33	17,2	13	17	0,04	0,02	99,52	0,10	1,17	4,29
4000	78	2	Ciamis	200-500	Pangsor	Ficus callosa Willd.	Moraceae		0,33	13,4	12	15	0,04	0,01	56,00	0,06	0,66	2,41
4001	78	2	Ciamis	200-500	Pangsor	Ficus callosa Willd.	Moraceae		0,33	8,6	8	10	0,04	0,01	17,19	0,02	0,20	0,74
4002	78	2	Ciamis	200-500	Mahoni	Swietenia macrophylla King	Meliaceae		0,61	12,7	4	11	0,04	0,01	67,67	0,07	0,80	2,92
4003	78	2	Ciamis	200-500	Pangsor	Ficus callosa Willd.	Moraceae		0,33	4,8	5	6,5	0,04	0,00	3,95	0,00	0,05	0,17
4004	78	2	Ciamis	200-500	Mahoni	Swietenia macrophylla King	Meliaceae		0,61	4,8	3,5	5	0,04	0,00	5,45	0,01	0,06	0,23
4005	78	2	Ciamis	200-500	Pangsor	Ficus callosa Willd.	Moraceae		0,33	13,4	11	14	0,04	0,01	52,57	0,05	0,62	2,27
4006	78	2	Ciamis	200-500	Durian	Durio zibethinus L.	Malvaceae		0,57	2,5	0,5	2	0,04	0,00	0,70	0,00	0,01	0,03
4007	78	2	Ciamis	200-500	Mahoni	Swietenia macrophylla King	Meliaceae		0,61	5,7	5,5	7	0,04	0,00	10,36	0,01	0,12	0,45
4008	78	2	Ciamis	200-500	Mahoni	Swietenia macrophylla King	Meliaceae		0,61	15,3	5	11	0,04	0,02	94,50	0,09	1,11	4,08
4009	78	2	Ciamis	200-500	Kokosan	Lansium domesticum var. aquae	Meliaceae		0,7	2,9	3	4,5	0,04	0,00	2,20	0,00	0,03	0,09
4010	78	2	Ciamis	200-500	Sengon	Paraserianthes falcataria (L.) I.C.	Leguminosae / Fabaceae		0,33	9,9	11	14	0,04	0,01	30,14	0,03	0,35	1,30
4011	78	2	Ciamis	200-500	Kelapa	Cocos nucifera L.	Arecaceae		0,5	27,4	15	18	0,04	0,06	143,10	0,14	1,68	6,17
4012	78	2	Ciamis	200-500	Kokosan	Lansium domesticum var. aquae	Meliaceae		0,7	3,8	3,5	4,5	0,04	0,00	3,73	0,00	0,04	0,16
4013	78	2	Ciamis	200-500	Petai	Parkia speciosa Hassk.	Leguminosae / Fabaceae		0,45	1,9	3	3,8	0,04	0,00	0,60	0,00	0,01	0,03
4014	78	2	Ciamis	200-500	Nangka	Artocarpus heterophyllus Lam.	Moraceae		0,61	1,9	1,5	2,3	0,04	0,00	0,50	0,00	0,01	0,02
4015	78	2	Ciamis	200-500	Nangka	Artocarpus heterophyllus Lam.	Moraceae		0,61	4,1	3	6	0,04	0,00	4,95	0,00	0,06	0,21
4016	78	2	Ciamis	200-500	Sengon	Paraserianthes falcataria (L.) I.C.	Leguminosae / Fabaceae		0,33	28,0	12	22	0,04	0,06	308,34	0,31	3,62	13,30
4017	78	2	Ciamis	200-500	Mahoni	Swietenia macrophylla King	Meliaceae		0,61	10,5	3	10	0,04	0,01	43,59	0,04	0,51	1,88
4018	78	2	Ciamis	200-500	Petai	Parkia speciosa Hassk.	Leguminosae / Fabaceae		0,45	7,3	6	7,5	0,04	0,00	13,08	0,01	0,15	0,56
4019	78	2	Ciamis	200-500	Mahoni	Swietenia macrophylla King	Meliaceae		0,61	3,2	2	3,5	0,04	0,00	1,87	0,00	0,02	0,08
4020	78	2	Ciamis	200-500	Pangsor	Ficus callosa Willd.	Moraceae		0,33	5,7	7	10	0,04	0,00	8,18	0,01	0,10	0,35
4021	78	2	Ciamis	200-500	Pangsor	Ficus callosa Willd.	Moraceae		0,33	16,9	9	11	0,04	0,02	64,55	0,06	0,76	2,78
4022	78	2	Ciamis	200-500	Tisuk	Hibiscus macrophyllus Roxb. ex I	Malvaceae		0,4	7,0	2	7	0,04	0,00	10,16	0,01	0,12	0,44
4023	78	2	Ciamis	200-500	Pangsor	Ficus callosa Willd.	Moraceae		0,33	13,7	11	14	0,04	0,01	54,88	0,05	0,64	2,37
4024	78	2	Ciamis	200-500	Pangsor	Ficus callosa Willd.	Moraceae		0,33	4,8	3,5	4	0,04	0,00	2,53	0,00	0,03	0,11
4025	78	2	Ciamis	200-500	Pangsor	Ficus callosa Willd.	Moraceae		0,33	5,4	5	6	0,04	0,00	4,61	0,00	0,05	0,20
4026	78	2	Ciamis	200-500	Mahoni	Swietenia macrophylla King	Meliaceae		0,61	8,0	3,5	9,5	0,04	0,00	25,01	0,03	0,29	1,08
4027	78	2	Ciamis	200-500	Mahoni	Swietenia macrophylla King	Meliaceae		0,61	6,4	3,5	6,5	0,04	0,00	11,74	0,01	0,14	0,51
4028	78	2	Ciamis	200-500	Pangsor	Ficus callosa Willd.	Moraceae		0,33	4,5	4	5,5	0,04	0,00	2,99	0,00	0,04	0,13
4029	78	2	Ciamis	200-500	Mahoni	Swietenia macrophylla King	Meliaceae		0,61	14,3	5	13	0,04	0,02	97,85	0,10	1,15	4,22
4030	78	2	Ciamis	200-500	Kelapa	Cocos nucifera L.	Arecaceae		0,5	30,5	13	18	0,04	0,07	143,10	0,14	1,68	6,17
4031	78	2	Ciamis	200-500	Pangsor	Ficus callosa Willd.	Moraceae		0,33	0,8	8	11	0,04	0,00	0,24	0,00	0,00	0,01
4032	78	2	Ciamis	200-500	Pangsor	Ficus callosa Willd.	Moraceae		0,33	10,8	10	14	0,04	0,01	35,69	0,04	0,42	1,54
4033	78	2	Ciamis	200-500	Pangsor	Ficus callosa Willd.	Moraceae		0,33	8,6	7	10	0,04	0,01	17,19	0,02	0,20	0,74
4034	78	2	Ciamis	200-500	Pangsor	Ficus callosa Willd.	Moraceae		0,33	13,4	12	15	0,04	0,01	56,00	0,06	0,66	2,41
4035	78	2	Ciamis	200-500	Mahoni	Swietenia macrophylla King	Meliaceae		0,61	8,9	5	12	0,04	0,01	38,12	0,04	0,45	1,64
4036	78	2	Ciamis	200-500	Pangsor	Ficus callosa Willd.	Moraceae		0,33	4,8	4	5,5	0,04	0,00	3,39	0,00	0,04	0,15
4037	78	2	Ciamis	200-500	Pangsor	Ficus callosa Willd.	Moraceae		0,33	3,5	4	4,8	0,04	0,00	1,69	0,00	0,02	0,07
4038	78	2	Ciamis	200-500	Pangsor	Ficus callosa Willd.	Moraceae		0,33	18,5	11	15	0,04	0,03	101,15	0,10	1,19	4,36
4039	78	2	Ciamis	200-500	Durian	Durio zibethinus L.	Malvaceae		0,57	5,4	5	5	0,04	0,00	6,44	0,01	0,08	0,28
4040	78	2	Ciamis	200-500	Mahoni	Swietenia macrophylla King	Meliaceae		0,61	14,6	7	13	0,04	0,02	101,87	0,10	1,20	4,39
4041	78	2	Ciamis	200-500	Mahoni	Swietenia macrophylla King	Meliaceae		0,61	13,4	6	13	0,04	0,01	86,23	0,09	1,01	3,72
4042	78	2	Ciamis	200-500	Petai	Parkia speciosa Hassk.	Leguminosae / Fabaceae		0,45	8,0	5	7	0,04	0,00	14,31	0,01	0,17	0,62
4043	78	2	Ciamis	200-500	Kopi	Coffea canephora Pierre ex A.Fro	Rubiaceae		0,41	3,5	2	4	0,04	0,00	3,66	0,00	0,04	0,16
4044	78	2	Ciamis	200-500	Sengon	Paraserianthes falcataria (L.) I.C.	Leguminosae / Fabaceae		0,33	32,5	12	21	0,04	0,08	387,25	0,39	4,55	16,70
4045	78	2	Ciamis	200-500	Kelapa	Cocos nucifera L.	Arecaceae		0,5	30,5	16	23	0,04	0,07	181,60	0,18	2,13	7,83
4046	78	2	Ciamis	200-500	Manglid	Magnolia champaca (L.) Baill. ex	Magnoliaceae		0,53	12,1	5	14	0,04	0,01	67,54	0,07	0,79	2,91
4047	78	2	Ciamis	200-500	Caruy	Pterospermum javanicum Jungh.	Malvaceae		0,53	2,5	3,5	5	0,04	0,00	1,51	0,00	0,02	0,07
4048	78	2	Ciamis	200-500	Kokosan	Lansium domesticum var. aquae	Meliaceae		0,7	7,0	4	7	0,04	0,00	16,97	0,02	0,20	0,73
4049	78	2	Ciamis	200-500	Tisuk	Hibiscus macrophyllus Roxb. ex I	Malvaceae		0,4	2,9	3	38	0,04	0,00	9,31	0,01	0,11	0,40
4050	78	2	Ciamis	200-500	Sp8	Sp8		0	0,5	11,1	8	12	0,04	0,01	47,82	0,05	0,56	2,06
4051	78	2	Ciamis	200-500	Petai	Parkia speciosa Hassk.	Leguminosae / Fabaceae		0,45	5,7	3,5	5	0,04	0,00	5,76	0,01	0,07	0,25
4052	78	2	Ciamis	200-500	Manglid	Magnolia champaca (L.) Baill. ex	Magnoliaceae		0,53	12,4	7	14	0,04	0,01	70,83	0,07	0,83	3,05
4053	78	2	Ciamis	200-500	Mahoni	Swietenia macrophylla King	Meliaceae		0,61	14,3	7	13	0,04	0,02	97,85	0,10	1,15	4,22
4054	78	2	Ciamis	200-500	Pangsor	Ficus callosa Willd.	Moraceae		0,33	5,4	4	7	0,04	0,00	5,31	0,01	0,06	0,23
4055	78	2	Ciamis	200-500	Pangsor	Ficus callosa Willd.	Moraceae		0,33	3,5	3,5	4	0,04	0,00	1,43	0,00	0,02	0,06
4056	78	2	Ciamis	200-500	Durian	Durio zibethinus L.	Malvaceae		0,57	8,6	2	4,5	0,04	0,01	13,65	0,01	0,16	0,59
4057	78	2	Ciamis	200-500	Pangsor	Ficus callosa Willd.	Moraceae		0,33	15,3	10	14	0,04	0,02	67,14	0,07	0,79	2,90
4058	78	2	Ciamis	200-500	Sengon	Paraserianthes falcataria (L.) I.C.	Leguminosae / Fabaceae		0,33	35,0	13	20	0,04	0,10	425,26	0,43	5,00	18,34
4059	79	3	Ciamis	200-500	Tisuk	Hibiscus macrophyllus Roxb. ex I	Malvaceae		0,4	10,2	7	9	0,04	-	25,42	0,03	0,30	1,10
4060	79	3	Ciamis	200-500	Sengon	Paraserianthes falcataria (L.) I.C.	Leguminosae / Fabaceae		0,33	27,7	11	17	0,04	0,06	238,43	0,24	2,80	10,28
4061	79	3	Ciamis	200-500	Jati	Tectona grandis L.f.	Lamiaceae / Verbenaceae		0,7	2,9	2	3	0,04	0,00	1,52	0,00	0,02	0,07
4062	79	3	Ciamis	200-500	Melinjo	Gnetum gnemon L.	Gnetaceae		0,76	11,8	1,5	6	0,04	0,01	41,18	0,04	0,48	1,78
4063	79	3	Ciamis	200-500	Kelapa	Cocos nucifera L.	Arecaceae		0,5	28,6	14	17	0,04	0,06	135,40	0,14	1,59	5,84
4064	79	3	Ciamis	200-500	Caruy	Pterospermum javanicum Jungh.	Malvaceae		0,53	2,5	2	3	0,04	0,00	0,95	0,00	0,01	0,04
4065	79	3	Ciamis	200-500	Tisuk	Hibiscus macrophyllus Roxb. ex I	Malvaceae		0,4	7,6	7	11	0,04	0,00	18,03	0,02	0,21	0,78
4066	79	3	Ciamis	200-500	Dukuh	Lansium domesticum Corrêa	Meliaceae		0,85	2,9	1,5	2,5	0,04	0,00	1,54	0,00	0,02	0,07
4067	79	3	Ciamis	200-500	Sengon	Paraserianthes falcataria (L.) I.C.	Leguminosae / Fabaceae		0,33	2,2	1	2	0,04	0,00	0,33	0,00	0,00	0,01
4068	79	3	Ciamis	200-500	Tisuk	Hibiscus macrophyllus Roxb. ex I	Malvaceae		0,4	6,4	2,5	6,5	0,04	0,00	7,97	0,01	0,09	0,34
4069	79	3	Ciamis	200-500	Caruy	Pterospermum javanicum Jungh.	Malvaceae		0,53	3,5	2	3,5	0,04	0,00	1,96	0,00	0,02	0,08
4070	79	3	Ciamis	200-500	Cangcaratan	Nauclea subdita (Korth.) Steud	Rubiaceae		0,32	22,6	12	3	0,04	0,04	32,61	0,03	0,38	1,41
4071	79	3	Ciamis	200-500	Tisuk	Hibiscus macrophyllus Roxb. ex I	Malvaceae		0,4	12,1	7	13	0,04	0,01	48,77	0,05	0,57	2,10
4072	79	3	Ciamis	200-500	Mahoni	Swietenia macrophylla King	Meliaceae		0,61	8,6	3	6	0,04	0,01	18,90	0,02	0,22	0,82
4073	79	3	Ciamis	200-500	Mahoni	Swietenia macrophylla King	Meliaceae		0,61	11,8	6	7,5	0,04	0,01	41,30	0,04	0,49	1,78
4074	79	3	Ciamis	200-500	Manglid	Magnolia champaca (L.) Baill. ex	Magnoliaceae		0,53	11,5	11	13	0,04	0,01	57,16	0,06	0,67	2,46
4075	79	3	Ciamis	200-500	Tisuk	Hibiscus macrophyllus Roxb. ex I	Malvaceae		0,4	4,5	5	7	0,04	0,00	4,44	0,00	0,05	0,19
4076	79	3	Ciamis	200-500	Tisuk	Hibiscus macrophyllus Roxb. ex I	Malvaceae		0,4	13,7	6	10	0,04	0,01	48,10	0,05	0,57	2,07
4077	79	3	Ciamis	200-500	Dukuh	Lansium domesticum Corrêa	Meliaceae		0,85	4,8	4	5,5	0,04	0,00	8,06	0,01	0,09	0,35
4078	79	3	Ciamis	200-500	Tisuk	Hibiscus macrophyllus Roxb. ex I	Malvaceae		0,4	2,9	3	4	0,04	0,00	1,18	0,00	0,01	0,05
4079	79	3	Ciamis	200-500	Tisuk	Hibiscus macrophyllus Roxb. ex I	Malvaceae		0,4	2,5	2	3	0,04	0,00	0,73	0,00	0,01	0,03
4080	79	3	Ciamis	200-500	Dukuh	Lansium domesticum Corrêa	Meliaceae		0,85	2,2	2	3	0,04	0,00	1,14	0,00	0,01	0,05
4081	79	3	Ciamis	200-500	Tisuk	Hibiscus macrophyllus Roxb. ex I	Malvaceae		0,4	9,5	4	6,5	0,04	0,01	16,76	0,02	0,20	0,72
4082	79	3	Ciamis	200-500	Kelapa	Cocos nucifera L.	Arecaceae		0,5	27,7	8	2	0,04	0,06	19,90	0,02	0,23	0,86
4083	79	3	Ciamis	200-500	Tisuk	Hibiscus macrophyllus Roxb. ex I	Malvaceae		0,4	2,2	1,7	2,5	0,04	0,00	0,49	0,00	0,01	0,02
4084	79	3	Ciamis	200-500	Tisuk	Hibiscus macrophyllus Roxb. ex I	Malvaceae		0,4	3,8	3,5	4	0,04	0,00	2,01	0,00	0,02	0,09
4085	79	3	Ciamis	200-500	Caruy	Pterospermum javanicum Jungh.	Malvaceae		0,53	2,9	2,5	3,5	0,04	0,00	1,36	0,00	0,02	0,06
4086	79	3	Ciamis	200-500	Tisuk	Hibiscus macrophyllus Roxb. ex I	Malvaceae		0,4	6,4	4	6	0,04	0,00	7,41	0,01	0,09	0,32

4087	79	3	Ciamis	200-500	Tisuk	Hibiscus macrophyllus Roxb. ex I	Malvaceae	0,4	3,2	2	3,5	0,04	0,00	1,27	0,00	0,01	0,05
4088	79	3	Ciamis	200-500	Tisuk	Hibiscus macrophyllus Roxb. ex I	Malvaceae	0,4	2,2	2	3	0,04	0,00	0,57	0,00	0,01	0,02
4089	79	3	Ciamis	200-500	Tisuk	Hibiscus macrophyllus Roxb. ex I	Malvaceae	0,4	4,8	3,5	5,5	0,04	0,00	4,04	0,00	0,05	0,17
4090	79	3	Ciamis	200-500	Kelapa	Cocos nucifera L.	Arecaceae	0,5	29,3	12	15	0,04	0,07	120,00	0,12	1,41	5,17
4091	79	3	Ciamis	200-500	Tisuk	Hibiscus macrophyllus Roxb. ex I	Malvaceae	0,4	6,4	5	6	0,04	0,00	7,41	0,01	0,09	0,32
4092	79	3	Ciamis	200-500	Tisuk	Hibiscus macrophyllus Roxb. ex I	Malvaceae	0,4	18,5	10	16	0,04	0,03	127,99	0,13	1,50	5,52
4093	79	3	Ciamis	200-500	Sengon	Paraserianthes falcataria (L.) I.C.	Leguminosae / Fabaceae	0,33	36,9	15	21	0,04	0,11	490,14	0,49	5,76	21,14
4094	79	3	Ciamis	200-500	Sengon	Paraserianthes falcataria (L.) I.C.	Leguminosae / Fabaceae	0,33	9,5	7	9	0,04	0,01	18,93	0,02	0,22	0,82
4095	79	3	Ciamis	200-500	Caruy	Pterospermum javanicum Jungh.	Malvaceae	0,53	2,9	1,5	3	0,04	0,00	1,18	0,00	0,01	0,05
4096	79	3	Ciamis	200-500	Tisuk	Hibiscus macrophyllus Roxb. ex I	Malvaceae	0,4	9,9	6	8	0,04	0,01	21,53	0,02	0,25	0,93
4097	79	3	Ciamis	200-500	Sengon	Paraserianthes falcataria (L.) I.C.	Leguminosae / Fabaceae	0,33	10,8	13	17	0,04	0,01	42,64	0,04	0,50	1,84
4098	79	3	Ciamis	200-500	Tisuk	Hibiscus macrophyllus Roxb. ex I	Malvaceae	0,4	3,2	2,5	3,5	0,04	0,00	1,27	0,00	0,01	0,05
4099	79	3	Ciamis	200-500	Mahoni	Swietenia macrophylla King	Meliaceae	0,61	11,5	3	6	0,04	0,01	32,02	0,03	0,38	1,38
4100	79	3	Ciamis	200-500	Sengon	Paraserianthes falcataria (L.) I.C.	Leguminosae / Fabaceae	0,33	12,7	11	17	0,04	0,01	57,43	0,06	0,67	2,48
4101	79	3	Ciamis	200-500	Sengon	Paraserianthes falcataria (L.) I.C.	Leguminosae / Fabaceae	0,33	11,1	11	16	0,04	0,01	42,54	0,04	0,50	1,83
4102	79	3	Ciamis	200-500	Sengon	Paraserianthes falcataria (L.) I.C.	Leguminosae / Fabaceae	0,33	9,5	10	14	0,04	0,01	28,38	0,03	0,33	1,22
4103	79	3	Ciamis	200-500	Sengon	Paraserianthes falcataria (L.) I.C.	Leguminosae / Fabaceae	0,33	25,8	16	21	0,04	0,05	253,85	0,25	2,98	10,95
4104	79	3	Ciamis	200-500	Sengon	Paraserianthes falcataria (L.) I.C.	Leguminosae / Fabaceae	0,33	13,7	13	16	0,04	0,01	62,02	0,06	0,73	2,67
4105	79	3	Ciamis	200-500	Sengon	Paraserianthes falcataria (L.) I.C.	Leguminosae / Fabaceae	0,33	11,8	13	16	0,04	0,01	47,10	0,05	0,55	2,03
4106	79	3	Ciamis	200-500	Sengon	Paraserianthes falcataria (L.) I.C.	Leguminosae / Fabaceae	0,33	20,7	13	17	0,04	0,03	139,77	0,14	1,64	6,03
4107	79	3	Ciamis	200-500	Tisuk	Hibiscus macrophyllus Roxb. ex I	Malvaceae	0,4	5,7	3	5	0,04	0,00	5,17	0,01	0,06	0,22
4108	79	3	Ciamis	200-500	Mahoni	Swietenia macrophylla King	Meliaceae	0,61	22,3	6	12	0,04	0,04	204,28	0,20	2,40	8,81
4109	79	3	Ciamis	200-500	Sengon	Paraserianthes falcataria (L.) I.C.	Leguminosae / Fabaceae	0,33	21,6	9	14	0,04	0,04	127,08	0,13	1,49	5,48
4110	79	3	Ciamis	200-500	Sengon	Paraserianthes falcataria (L.) I.C.	Leguminosae / Fabaceae	0,33	13,7	14	17	0,04	0,01	65,57	0,07	0,77	2,83
4111	79	3	Ciamis	200-500	Sengon	Paraserianthes falcataria (L.) I.C.	Leguminosae / Fabaceae	0,33	20,7	12	21	0,04	0,03	169,62	0,17	1,99	7,31
4112	79	3	Ciamis	200-500	Sengon	Paraserianthes falcataria (L.) I.C.	Leguminosae / Fabaceae	0,33	15,3	10	14	0,04	0,02	67,14	0,07	0,79	2,90
4113	79	3	Ciamis	200-500	Sengon	Paraserianthes falcataria (L.) I.C.	Leguminosae / Fabaceae	0,33	34,0	14	21	0,04	0,09	422,73	0,42	4,97	18,23
4114	79	3	Ciamis	200-500	Sengon	Paraserianthes falcataria (L.) I.C.	Leguminosae / Fabaceae	0,33	14,6	14	17	0,04	0,02	74,19	0,07	0,87	3,20
4115	80	3	Ciamis	200-500	Tisuk	Hibiscus macrophyllus Roxb. ex I	Malvaceae	0,4	8,9	4	7	0,04	0,01	15,81	0,02	0,19	0,68
4116	80	3	Ciamis	200-500	Rambutan	Nephelium lappaceum L.	Sapindaceae	0,71	19,4	4	11	0,04	0,03	168,47	0,17	1,98	7,26
4117	80	3	Ciamis	200-500	Rambutan	Nephelium lappaceum L.	Sapindaceae	0,71	5,7	3	6	0,04	0,00	10,34	0,01	0,12	0,45
4118	80	3	Ciamis	200-500	Kelapa	Cocos nucifera L.	Arecaceae	0,5	30,2	13	16	0,04	0,07	127,70	0,13	1,50	5,51
4119	80	3	Ciamis	200-500	Rambutan	Nephelium lappaceum L.	Sapindaceae	0,71	12,7	6	10	0,04	0,01	71,26	0,07	0,84	3,07
4120	80	3	Ciamis	200-500	Rambutan	Nephelium lappaceum L.	Sapindaceae	0,71	6,7	3,5	7	0,04	0,00	15,79	0,02	0,19	0,68
4121	80	3	Ciamis	200-500	Rambutan	Nephelium lappaceum L.	Sapindaceae	0,71	8,9	4	5	0,04	0,01	19,65	0,02	0,23	0,85
4122	80	3	Ciamis	200-500	Petai	Parkia speciosa Hassk.	Leguminosae / Fabaceae	0,45	1,3	13	16	0,04	0,00	1,06	0,00	0,01	0,05
4123	80	3	Ciamis	200-500	Rambutan	Nephelium lappaceum L.	Sapindaceae	0,71	1,6	6	7	0,04	0,00	1,14	0,00	0,01	0,05
4124	80	3	Ciamis	200-500	Nangka	Artocarpus heterophyllus Lam.	Moraceae	0,61	29,0	4	11	0,04	0,07	305,04	0,31	3,58	13,15
4125	80	3	Ciamis	200-500	Durian	Durio zibethinus L.	Malvaceae	0,57	20,4	4	11	0,04	0,03	150,43	0,15	1,77	6,49
4126	80	3	Ciamis	200-500	Rambutan	Nephelium lappaceum L.	Sapindaceae	0,71	5,1	4	5,5	0,04	0,00	7,69	0,01	0,09	0,33
4127	80	3	Ciamis	200-500	Caruy	Pterospermum javanicum Jungh.	Malvaceae	0,53	3,5	3	4	0,04	0,00	2,21	0,00	0,03	0,10
4128	80	3	Ciamis	200-500	Sengon	Paraserianthes falcataria (L.) I.C.	Leguminosae / Fabaceae	0,33	22,6	18	21	0,04	0,04	199,41	0,20	2,34	8,60
4129	80	3	Ciamis	200-500	Dukuh	Lansium domesticum Corrêa	Meliaceae	0,85	9,9	7	11	0,04	0,01	57,49	0,06	0,68	2,48
4130	80	3	Ciamis	200-500	Dukuh	Lansium domesticum Corrêa	Meliaceae	0,85	3,5	3	5	0,04	0,00	4,18	0,00	0,05	0,18
4131	80	3	Ciamis	200-500	Sengon	Paraserianthes falcataria (L.) I.C.	Leguminosae / Fabaceae	0,33	24,5	16	21	0,04	0,05	231,36	0,23	2,72	9,98
4132	80	3	Ciamis	200-500	Mahoni	Swietenia macrophylla King	Meliaceae	0,61	14,3	5,5	11	0,04	0,02	83,96	0,08	0,99	3,62
4133	80	3	Ciamis	200-500	Bungbulang	Premna tomentosa Willd.	Lamiaceae / Verbenaceae	0,96	19,1	10	14	0,04	0,03	268,72	0,27	3,16	11,59
4134	80	3	Ciamis	200-500	Mahoni	Swietenia macrophylla King	Meliaceae	0,61	22,6	9	14	0,04	0,04	241,46	0,24	2,84	10,41
4135	80	3	Ciamis	200-500	Pangsor	Ficus callosa Willd.	Moraceae	0,33	5,1	5	7	0,04	0,00	4,75	0,00	0,06	0,21
4136	80	3	Ciamis	200-500	Kelapa	Cocos nucifera L.	Arecaceae	0,5	30,5	16	19	0,04	0,07	150,80	0,15	1,77	6,50
4137	80	3	Ciamis	200-500	Mahoni	Swietenia macrophylla King	Meliaceae	0,61	17,5	8	12	0,04	0,02	131,33	0,13	1,54	5,66
4138	80	3	Ciamis	200-500	Kokosan	Lansium domesticum var. aquae	Meliaceae	0,7	12,7	4	10	0,04	0,01	70,34	0,07	0,83	3,03
4139	80	3	Ciamis	200-500	Kokosan	Lansium domesticum var. aquae	Meliaceae	0,7	3,2	4	5,5	0,04	0,00	3,21	0,00	0,04	0,14
4140	80	3	Ciamis	200-500	Mahoni	Swietenia macrophylla King	Meliaceae	0,61	11,5	7	10	0,04	0,01	51,12	0,05	0,60	2,20
4141	80	3	Ciamis	200-500	Randu	Ceiba pentandra (L.) Gaertn.	Malvaceae	0,23	3,8	3,5	4,5	0,04	0,00	1,35	0,00	0,02	0,06
4142	80	3	Ciamis	200-500	Pangsor	Ficus callosa Willd.	Moraceae	0,33	11,5	11	14	0,04	0,01	39,63	0,04	0,47	1,71
4143	80	3	Ciamis	200-500	Pangsor	Ficus callosa Willd.	Moraceae	0,33	9,9	9	12	0,04	0,01	26,17	0,03	0,31	1,13
4144	80	3	Ciamis	200-500	Kelapa	Cocos nucifera L.	Arecaceae	0,5	29,3	13	16	0,04	0,07	127,70	0,13	1,50	5,51
4145	80	3	Ciamis	200-500	Tisuk	Hibiscus macrophyllus Roxb. ex I	Malvaceae	0,4	9,9	16	20	0,04	0,01	49,83	0,05	0,59	2,15
4146	80	3	Ciamis	200-500	Kokosan	Lansium domesticum var. aquae	Meliaceae	0,7	9,9	7	13	0,04	0,01	56,08	0,06	0,66	2,42
4147	80	3	Ciamis	200-500	Tisuk	Hibiscus macrophyllus Roxb. ex I	Malvaceae	0,4	6,4	9	11	0,04	0,00	12,91	0,01	0,15	0,56
4148	80	3	Ciamis	200-500	Jengkol	Archidendron pauciflorum (Bentl	Leguminosae / Fabaceae	0,4	4,8	5	7	0,04	0,00	5,04	0,01	0,06	0,22
4149	80	3	Ciamis	200-500	Petai	Parkia speciosa Hassk.	Leguminosae / Fabaceae	0,45	24,2	11	15	0,04	0,05	220,50	0,22	2,59	9,51
4150	80	3	Ciamis	200-500	Pangsor	Ficus callosa Willd.	Moraceae	0,33	9,9	4	10	0,04	0,01	22,14	0,02	0,26	0,95
4151	80	3	Ciamis	200-500	Pangsor	Ficus callosa Willd.	Moraceae	0,33	6,4	7	10	0,04	0,00	9,92	0,01	0,12	0,43
4152	80	3	Ciamis	200-500	Nangka	Artocarpus heterophyllus Lam.	Moraceae	0,61	39,1	6	13	0,04	0,12	617,39	0,62	7,25	26,62
4153	80	3	Ciamis	200-500	Pangsor	Ficus callosa Willd.	Moraceae	0,33	10,2	14	14	0,04	0,01	31,94	0,03	0,38	1,38
4154	80	3	Ciamis	200-500	Pangsor	Ficus callosa Willd.	Moraceae	0,33	13,4	9	13	0,04	0,01	49,12	0,05	0,58	2,12
4155	80	3	Ciamis	200-500	Kihampelas	Ficus ampelas Burm.f.	Moraceae	0,38	3,8	2	4	0,04	0,00	1,91	0,00	0,02	0,08
4156	80	3	Ciamis	200-500	Caruy	Pterospermum javanicum Jungh.	Malvaceae	0,53	16,5	14	20	0,04	0,02	166,35	0,17	1,95	7,17
4157	80	3	Ciamis	200-500	Kelapa	Cocos nucifera L.	Arecaceae	0,5	27,4	16	19	0,04	0,06	150,80	0,15	1,77	6,50
4158	80	3	Ciamis	200-500	Tisuk	Hibiscus macrophyllus Roxb. ex I	Malvaceae	0,4	17,8	13	17	0,04	0,02	126,88	0,13	1,49	5,47
4159	80	3	Ciamis	200-500	Mahoni	Swietenia macrophylla King	Meliaceae	0,61	8,3	3	6	0,04	0,01	17,64	0,02	0,21	0,76
4160	80	3	Ciamis	200-500	Tisuk	Hibiscus macrophyllus Roxb. ex I	Malvaceae	0,4	9,2	11	14	0,04	0,01	31,81	0,03	0,37	1,37
4161	80	3	Ciamis	200-500	Pangsor	Ficus callosa Willd.	Moraceae	0,33	12,4	9	16	0,04	0,01	51,87	0,05	0,61	2,24
4162	80	3	Ciamis	200-500	Kelapa	Cocos nucifera L.	Arecaceae	0,5	32,5	15	18	0,04	0,08	143,10	0,14	1,68	6,17
4163	80	3	Ciamis	200-500	Tisuk	Hibiscus macrophyllus Roxb. ex I	Malvaceae	0,4	10,2	12	16	0,04	0,01	43,05	0,04	0,51	1,86
4164	80	3	Ciamis	200-500	Petai	Parkia speciosa Hassk.	Leguminosae / Fabaceae	0,45	31,5	15	19	0,04	0,08	444,43	0,44	5,22	19,16
4165	80	3	Ciamis	200-500	Mahoni	Swietenia macrophylla King	Meliaceae	0,61	13,4	8	11	0,04	0,01	73,99	0,07	0,87	3,19
4166	80	3	Ciamis	200-500	Kokosan	Lansium domesticum var. aquae	Meliaceae	0,7	10,2	4	11	0,04	0,01	51,00	0,05	0,60	2,20
4167	80	3	Ciamis	200-500	Tisuk	Hibiscus macrophyllus Roxb. ex I	Malvaceae	0,4	6,0	10	12	0,04	0,00	12,73	0,01	0,15	0,55
4168	80	3	Ciamis	200-500	Kokosan	Lansium domesticum var. aquae	Meliaceae	0,7	4,5	3	4,5	0,04	0,00	4,95	0,00	0,06	0,21
4169	81	2	Ciamis	200-500	Jengkol	Archidendron pauciflorum (Bentl	Leguminosae / Fabaceae	0,4	10,8	7	4,5	0,04	0,01	15,05	0,02	0,18	0,65
4170	81	2	Ciamis	200-500	Bungbulang	Premna tomentosa Willd.	Lamiaceae / Verbenaceae	0,96	5,4	4	7	0,04	0,00	14,13	0,01	0,17	0,61
4171	81	2	Ciamis	200-500	Sp8	Sp8	0	0,5	6,4	2	8	0,04	0,00	11,83	0,01	0,14	0,51
4172	81	2	Ciamis	200-500	Petai	Parkia speciosa Hassk.	Leguminosae / Fabaceae	0,45	6,0	4	6	0,04	0,00	7,51	0,01	0,09	0,32
4173	81	2	Ciamis	200-500	Mangga	Mangifera indica L.	Anacardiaceae	0,6	2,5	1,5	2,5	0,04	0,00	0,90	0,00	0,01	0,04
4174	81	2	Ciamis	200-500	Petai	Parkia speciosa Hassk.	Leguminosae / Fabaceae	0,45	4,8	4	5,5	0,04	0,00	4,50	0,00	0,05	0,19
4175	81	2	Ciamis	200-500	Bungbulang	Premna tomentosa Willd.	Lamiaceae / Verbenaceae	0,96	3,5	3,5	5,5	0,04	0,00	5,10	0,01	0,06	0,22
4176	81	2	Ciamis	200-500	Kokosan	Lansium domesticum var. aquae	Meliaceae	0,7	10,8	2,5	7,5	0,04	0,01	40,13	0,04	0,47	1,73
4177	81	2	Ciamis	200-500	Kokosan	Lansium domesticum var. aquae	Meliaceae	0,7	5,7	3	6	0,04	0,00	10,20	0,01	0,12	0,44
4178	81	2	Ciamis	200-500	Rambutan	Nephelium lappaceum L.	Sapindaceae	0,71	5,4	3	6	0,04	0,00	9,31	0,01	0,11	0,40
4179	81	2	Ciamis	200-500	Rambutan	Nephelium lappaceum L.	Sapindaceae	0,71	2,9	3	4	0,04	0,00	2,00	0,00	0,02	0,09
4180	81	2	Ciamis	200-500	Tisuk	Hibiscus macrophyllus Roxb. ex I	Malvaceae	0,4	3,2	1,8	3,5	0,04	0,00	1,27	0,00	0,01	0,05
4181	81	2	Ciamis	200-500	Jengkol	Archidendron pauciflorum (Bentl	Leguminosae / Fabaceae	0,4	15,6	10	13	0,04	0,02	77,70	0,08	0,91	3,35
4182	81	2	Ciamis	200-500	Tisuk	Hibiscus macrophyllus Roxb. ex I	Malvaceae	0,4	4,1	3	5	0,04	0,00	2,85	0,00	0,03	0,12
4183	81	2	Ciamis	200-500	Nangka	Artocarpus heterophyllus Lam.	Moraceae	0,61	2,9	1	1,5	0,04	0,00	0,71	0,00	0,01	0,03
4184	81	2	Ciamis	200-500	Petai	Parkia speciosa Hassk.	Leguminosae / Fabaceae	0,45	3,5	3,5	5	0,04	0,00	2,34	0,00	0,03	0,10
4185	81	2	Ciamis	200-500	Tisuk	Hibiscus macrophyllus Roxb. ex I	Malvaceae	0,4	2,5	3	3,5	0,04	0,00	0,84	0,00	0,01	0,04
4186	81	2	Ciamis	200-500	Rambutan	Nephelium lappaceum L.	Sapindaceae	0,71	6,4	3	7	0,04	0,00	14,44	0,01	0,17	0,62
4187	81	2	Ciamis	200-500	Rambutan	Nephelium lappaceum L.	Sapindaceae	0,71	2,5	2,5	3,5	0,04	0,00	1,43	0,00	0,02	0,06
4188	81	2	Ciamis	200-500	Tisuk	Hibiscus macrophyllus Roxb. ex I	Malvaceae	0,4	2,2	2	3	0,04	0,00	0,57	0,00	0,01	0,02
4189	81	2	Ciamis	200-500	Tisuk	Hibiscus macrophyllus Roxb. ex I	Malvaceae	0,4	3,8	3	5	0,04	0,00	2,46	0,00	0,03	0,11
4190	81	2	Ciamis	200-500	Tisuk	Hibiscus macrophyllus Roxb. ex I	Malvaceae	0,4	2,5	3	3,5	0,04	0,00	0,84	0,00	0,01	0,04
4191	81	2	Ciamis	200-500	Jengkol	Archidendron pauciflorum (Bentl	Leguminosae / Fabaceae	0,4	5,7	4,5	6	0,04	0,00	6,11	0,01	0,07	0,26
4192	81	2	Ciamis	200-500	Rambutan	Nephelium lappaceum L.	Sapindaceae	0,71	3,8	2	3	0,04	0,00	2,61	0,00	0,03	0,11
4193	81	2	Ciamis	200-500	Rambutan	Nephelium lappaceum L.	Sapindaceae	0,71	4,8	3,5	5	0,04	0,00	6,26	0,01	0,07	0,27
4194	81	2	Ciamis	200-500	Jengkol	Archidendron pauciflorum (Bentl	Leguminosae / Fabaceae	0,4	3,5	2	3	0,04	0,00	1,31	0,00	0,02	0,06
4195	81	2	Ciamis	200-500	Tisuk	Hibiscus macrophyllus Roxb. ex I	Malvaceae	0,4	4,8	3,5	5	0,04	0,00	3,70	0,00	0,04	0,16
4196	81	2	Ciamis	200-500	Petai	Parkia speciosa Hassk.	Leguminosae / Fabaceae	0,45	6,0	7	8	0,04	0,00	9,78	0,01	0,11	0,42
4197	81	2	Ciamis	200-500	Nangka	Artocarpus heterophyllus Lam.	Moraceae	0,61	3,2	2	4	0,04	0,00	2,11	0,00	0,02	0,09
4198	81	2	Ciamis	200-500	Dukuh	Lansium domesticum Corrêa	Meliaceae	0,85	24,8	4	11	0,04	0,05	311,67	0,31	3,66	13,44
4199	81	2	Ciamis	200-500	Tisuk	Hibiscus macrophyllus Roxb. ex I	Malvaceae	0,4	5,7	3	6	0,04	0,00	6,11	0,01	0,07	0,26
4200	81	2	Ciamis	200-500	Rambutan	Nephelium lappaceum L.	Sapindaceae	0,71	6,4	3	5,5	0,04	0,00	11,58	0,01	0,14	0,50
4201	81	2	Ciamis	200-500	Jengkol	Archidendron pauciflorum (Bentl	Leguminosae / Fabaceae	0,4	22,0	8	12	0,04	0,04	135,18	0,14	1,59	5,83
4202	81	2	Ciamis	200-500	Kokosan	Lansium domesticum var. aquae	Meliaceae	0,7	7,0	5	7,5	0,04	0,00	18,08	0,02	0,21	0,78
4203	81	2	Ciamis	200-500	Kokosan	Lansium domesticum var. aquae	Meliaceae	0,7	4,5	3,5	4,5	0,04	0,00	4,95	0,00	0,06	0,21
4204	81	2	Ciamis	200-500	Sp8	Sp8	0	0,5	4,1	2,5	4	0,04	0,00	2,85	0,00	0,03	0,12
4205	81	2	Ciamis	200-500	Sp8	Sp8	0	0,5	4,1	4	5,5	0,04	0,00	3,81	0,00	0,04	0,16
4206	81	2	Ciamis	200-500	Mangga	Mangifera indica L.	Anacardiaceae	0,6	6,0	3	5	0,04	0,00	8,28	0,01	0,10	0,36
4207	81	2	Ciamis	200-500	Kimunding / kemuning	Ficus lyrata Warb.	Moraceae	0,393	4,8	3,5	5,5	0,04	0,00	3,98	0,00	0,05	0,17
4208	81	2	Ciamis	200-500	Dukuh	Lansium domesticum Corrêa	Meliaceae	0,85	14,0	5	9	0,04	0,02	90,86	0,09	1,07	3,92
4209	81	2	Ciamis	200-500	Kokosan	Lansium domesticum var. aquae	Meliaceae	0,7	9,5	3,5	6,5	0,04	0,01	27,99	0,03	0,33	1,21
4210	81	2	Ciamis	200-500	Kokosan	Lansium domesticum var. aquae	Meliaceae	0,7	5,1	3,5	4,5	0,04	0,00	6,32	0,01	0,07	0,27

4211	81	2	Ciamis	200-500	Dukuh	Lansium domesticum Corrêa	Meliaceae		0,85	10,5	3,5	8,5	0,04	0,01	50,90	0,05	0,60	2,20
4212	81	2	Ciamis	200-500	Jengkol	Archidendron pauciflorum (Bentl	Leguminosae / Fabaceae		0,4	5,1	3	6	0,04	0,00	4,92	0,00	0,06	0,21
4213	81	2	Ciamis	200-500	Rambutan	Nephelium lappaceum L.	Sapindaceae		0,71	5,4	4	6	0,04	0,00	9,31	0,01	0,11	0,40
4214	81	2	Ciamis	200-500	Bintinu	Melochia umbellata (Houtt.) Stap	Malvaceae		0,25	12,7	4	8	0,04	0,01	22,33	0,02	0,26	0,96
4215	81	2	Ciamis	200-500	Kelapa	Cocos nucifera L.	Arecaceae		0,5	26,7	15	18	0,04	0,06	143,10	0,14	1,68	6,17
4216	81	2	Ciamis	200-500	Sp8	Sp8		0	0,5	3,2	2	3	0,04	0,00	1,35	0,00	0,02	0,06
4217	81	2	Ciamis	200-500	Kelapa	Cocos nucifera L.	Arecaceae		0,5	33,7	10	13	0,04	0,09	104,60	0,10	1,23	4,51
4218	81	2	Ciamis	200-500	Mahoni	Swietenia macrophylla King	Meliaceae		0,61	6,0	3	4	0,04	0,00	6,85	0,01	0,08	0,30
4219	81	2	Ciamis	200-500	Rambutan	Nephelium lappaceum L.	Sapindaceae		0,71	6,7	4	6	0,04	0,00	13,71	0,01	0,16	0,59
4220	81	2	Ciamis	200-500	Rambutan	Nephelium lappaceum L.	Sapindaceae		0,71	8,6	6	8	0,04	0,01	28,27	0,03	0,33	1,22
4221	81	2	Ciamis	200-500	Rambutan	Nephelium lappaceum L.	Sapindaceae		0,71	3,5	2,5	3,5	0,04	0,00	2,56	0,00	0,03	0,11
4222	81	2	Ciamis	200-500	Jengkol	Archidendron pauciflorum (Bentl	Leguminosae / Fabaceae		0,4	6,0	4	7	0,04	0,00	7,77	0,01	0,09	0,34
4223	81	2	Ciamis	200-500	Jengkol	Archidendron pauciflorum (Bentl	Leguminosae / Fabaceae		0,4	14,6	6	10	0,04	0,02	54,42	0,05	0,64	2,35
4224	81	2	Ciamis	200-500	Kelapa	Cocos nucifera L.	Arecaceae		0,5	26,7	9	12	0,04	0,06	96,90	0,10	1,14	4,18
4225	81	2	Ciamis	200-500	Rambutan	Nephelium lappaceum L.	Sapindaceae		0,71	8,3	3,5	6,5	0,04	0,01	21,81	0,02	0,26	0,94
4226	81	2	Ciamis	200-500	Rambutan	Nephelium lappaceum L.	Sapindaceae		0,71	4,8	3	5	0,04	0,00	6,26	0,01	0,07	0,27
4227	82	2	Ciamis	200-500	Caruy	Pterospermum javanicum Jungh.	Malvaceae		0,53	16,2	10	15	0,04	0,02	123,35	0,12	1,45	5,32
4228	82	2	Ciamis	200-500	Sengon	Paraserianthes falcataria (L.) I.C.	Leguminosae / Fabaceae		0,33	57,9	14	24	0,04	0,26	1.264,16	1,26	14,85	54,51
4229	82	2	Ciamis	200-500	Kelapa	Cocos nucifera L.	Arecaceae		0,5	24,8	14	17	0,04	0,05	135,40	0,14	1,59	5,84
4230	82	2	Ciamis	200-500	Mahoni	Swietenia macrophylla King	Meliaceae		0,61	9,5	3	8	0,04	0,01	29,84	0,03	0,35	1,29
4231	82	2	Ciamis	200-500	Tisuk	Hibiscus macrophyllus Roxb. ex I	Malvaceae		0,4	10,8	5	10	0,04	0,01	31,28	0,03	0,37	1,35
4232	82	2	Ciamis	200-500	Mahoni	Swietenia macrophylla King	Meliaceae		0,61	18,8	4	8	0,04	0,03	103,02	0,10	1,21	4,44
4233	82	2	Ciamis	200-500	Kokosan	Lansium domesticum var. aquae	Meliaceae		0,7	10,5	5,5	9,5	0,04	0,01	47,18	0,05	0,55	2,03
4234	82	2	Ciamis	200-500	Tisuk	Hibiscus macrophyllus Roxb. ex I	Malvaceae		0,4	31,2	10	18	0,04	0,08	372,70	0,37	4,38	16,07
4235	82	2	Ciamis	200-500	Kelapa	Cocos nucifera L.	Arecaceae		0,5	25,8	17	20	0,04	0,05	158,50	0,16	1,86	6,83
4236	82	2	Ciamis	200-500	Mahoni	Swietenia macrophylla King	Meliaceae		0,61	34,0	8	15	0,04	0,09	545,27	0,55	6,41	23,51
4237	82	2	Ciamis	200-500	Mahoni	Swietenia macrophylla King	Meliaceae		0,61	21,3	8	14	0,04	0,04	217,12	0,22	2,55	9,36
4238	82	2	Ciamis	200-500	Caruy	Pterospermum javanicum Jungh.	Malvaceae		0,53	7,0	3	7,5	0,04	0,00	14,01	0,01	0,16	0,60
4239	82	2	Ciamis	200-500	Kokosan	Lansium domesticum var. aquae	Meliaceae		0,7	6,7	3	6,5	0,04	0,00	14,56	0,01	0,17	0,63
4240	82	2	Ciamis	200-500	Kelapa	Cocos nucifera L.	Arecaceae		0,5	29,6	17	20	0,04	0,07	158,50	0,16	1,86	6,83
4241	82	2	Ciamis	200-500	Durian	Durio zibethinus L.	Malvaceae		0,57	18,1	10	13	0,04	0,03	141,79	0,14	1,67	6,11
4242	82	2	Ciamis	200-500	Tisuk	Hibiscus macrophyllus Roxb. ex I	Malvaceae		0,4	25,5	15	23	0,04	0,05	321,67	0,32	3,78	13,87
4243	82	2	Ciamis	200-500	Mahoni	Swietenia macrophylla King	Meliaceae		0,61	24,5	12	19	0,04	0,05	370,57	0,37	4,35	15,98
4244	82	2	Ciamis	200-500	Wangkal	Albizia procera Benth	Leguminosae / Fabaceae		0,77	33,4	14	24	0,04	0,09	1.002,85	1,00	11,78	43,25
4245	82	2	Ciamis	200-500	Jati	Tectona grandis L.f.	Lamiaceae / Verbenaceae		0,7	12,7	3,5	9,5	0,04	0,01	67,11	0,07	0,79	2,89
4246	82	2	Ciamis	200-500	Tisuk	Hibiscus macrophyllus Roxb. ex I	Malvaceae		0,4	10,5	7	9	0,04	0,01	26,89	0,03	0,32	1,16
4247	82	2	Ciamis	200-500	Nangka	Artocarpus heterophyllus Lam.	Moraceae		0,61	7,0	3,5	6,5	0,04	0,00	13,98	0,01	0,16	0,60
4248	82	2	Ciamis	200-500	Mahoni	Swietenia macrophylla King	Meliaceae		0,61	10,5	3	7	0,04	0,01	31,44	0,03	0,37	1,36
4249	82	2	Ciamis	200-500	Mahoni	Swietenia macrophylla King	Meliaceae		0,61	9,5	4	7	0,04	0,01	26,40	0,03	0,31	1,14
4250	82	2	Ciamis	200-500	Mahoni	Swietenia macrophylla King	Meliaceae		0,61	9,5	4	9	0,04	0,01	33,24	0,03	0,39	1,43
4251	83	2	Ciamis	200-500	Kokosan	Lansium domesticum var. aquae	Meliaceae		0,7	12,1	4	6	0,04	0,01	40,10	0,04	0,47	1,73
4252	83	2	Ciamis	200-500	Kokosan	Lansium domesticum var. aquae	Meliaceae		0,7	3,2	2,5	3,5	0,04	0,00	2,12	0,00	0,02	0,09
4253	83	2	Ciamis	200-500	Kelapa	Cocos nucifera L.	Arecaceae		0,5	27,7	13	16	0,04	0,06	127,70	0,13	1,50	5,51
4254	83	2	Ciamis	200-500	Petai	Parkia speciosa Hassk.	Leguminosae / Fabaceae		0,45	26,7	14	18	0,04	0,06	313,02	0,31	3,68	13,50
4255	83	2	Ciamis	200-500	Dukuh	Lansium domesticum Corrêa	Meliaceae		0,85	13,0	3,5	11,5	0,04	0,01	99,93	0,10	1,17	4,31
4256	83	2	Ciamis	200-500	Kokosan	Lansium domesticum var. aquae	Meliaceae		0,7	3,8	3,5	7	0,04	0,00	5,59	0,01	0,07	0,24
4257	83	2	Ciamis	200-500	Dukuh	Lansium domesticum Corrêa	Meliaceae		0,85	36,0	3,5	13,5	0,04	0,10	741,47	0,74	8,71	31,97
4258	83	2	Ciamis	200-500	Rambutan	Nephelium lappaceum L.	Sapindaceae		0,71	5,7	2,5	5,5	0,04	0,00	9,54	0,01	0,11	0,41
4259	83	2	Ciamis	200-500	Tisuk	Hibiscus macrophyllus Roxb. ex I	Malvaceae		0,4	10,5	9	11	0,04	0,01	32,32	0,03	0,38	1,39
4260	83	2	Ciamis	200-500	Durian	Durio zibethinus L.	Malvaceae		0,57	3,5	1,5	6	0,04	0,00	3,43	0,00	0,04	0,15
4261	83	2	Ciamis	200-500	Petai	Parkia speciosa Hassk.	Leguminosae / Fabaceae		0,45	4,5	3	4	0,04	0,00	2,96	0,00	0,03	0,13
4262	83	2	Ciamis	200-500	Tisuk	Hibiscus macrophyllus Roxb. ex I	Malvaceae		0,4	6,0	3	7	0,04	0,00	7,77	0,01	0,09	0,34
4263	83	2	Ciamis	200-500	Tisuk	Hibiscus macrophyllus Roxb. ex I	Malvaceae		0,4	11,5	9	12	0,04	0,01	41,05	0,04	0,48	1,77
4264	83	2	Ciamis	200-500	Mahoni	Swietenia macrophylla King	Meliaceae		0,61	15,6	11	17	0,04	0,02	146,22	0,15	1,72	6,31
4265	83	2	Ciamis	200-500	Dukuh	Lansium domesticum Corrêa	Meliaceae		0,85	47,7	7	14	0,04	0,18	1.288,01	1,29	15,13	55,54
4266	83	2	Ciamis	200-500	Dukuh	Lansium domesticum Corrêa	Meliaceae		0,85	33,7	3,5	10,5	0,04	0,09	523,89	0,52	6,16	22,59
4267	83	2	Ciamis	200-500	Kokosan	Lansium domesticum var. aquae	Meliaceae		0,7	0,8	3,5	7,5	0,04	0,00	0,36	0,00	0,00	0,02
4268	83	2	Ciamis	200-500	Dukuh	Lansium domesticum Corrêa	Meliaceae		0,85	4,8	1,5	2,5	0,04	0,00	3,91	0,00	0,05	0,17
4269	83	2	Ciamis	200-500	Sengon	Paraserianthes falcataria (L.) I.C.	Leguminosae / Fabaceae		0,33	27,4	18	24	0,04	0,06	320,15	0,32	3,76	13,81
4270	83	2	Ciamis	200-500	Sengon	Paraserianthes falcataria (L.) I.C.	Leguminosae / Fabaceae		0,33	18,1	12	16	0,04	0,03	103,95	0,10	1,22	4,48
4271	83	2	Ciamis	200-500	Sengon	Paraserianthes falcataria (L.) I.C.	Leguminosae / Fabaceae		0,33	24,2	12	19	0,04	0,05	206,09	0,21	2,42	8,89
4272	83	2	Ciamis	200-500	Alpukat	Persea americana Mill.	Lauraceae		0,54	3,2	2,5	4	0,04	0,00	1,89	0,00	0,02	0,08
4273	83	2	Ciamis	200-500	Dukuh	Lansium domesticum Corrêa	Meliaceae		0,85	2,5	2	2,8	0,04	0,00	1,37	0,00	0,02	0,06
4274	83	2	Ciamis	200-500	Sengon	Paraserianthes falcataria (L.) I.C.	Leguminosae / Fabaceae		0,33	12,4	3,5	15,5	0,04	0,01	50,38	0,05	0,59	2,17
4275	83	2	Ciamis	200-500	Petai	Parkia speciosa Hassk.	Leguminosae / Fabaceae		0,45	2,5	1,8	3,3	0,04	0,00	0,89	0,00	0,01	0,04
4276	83	2	Ciamis	200-500	Dukuh	Lansium domesticum Corrêa	Meliaceae		0,85	4,5	2	3,5	0,04	0,00	4,69	0,00	0,06	0,20
4277	83	2	Ciamis	200-500	Durian	Durio zibethinus L.	Malvaceae		0,57	4,5	1,5	3	0,04	0,00	2,83	0,00	0,03	0,12
4278	83	2	Ciamis	200-500	Sengon	Paraserianthes falcataria (L.) I.C.	Leguminosae / Fabaceae		0,33	43,6	12	22	0,04	0,15	693,76	0,69	8,15	29,92
4279	83	2	Ciamis	200-500	Sengon	Paraserianthes falcataria (L.) I.C.	Leguminosae / Fabaceae		0,33	30,5	12	22	0,04	0,07	361,62	0,36	4,25	15,59
4280	83	2	Ciamis	200-500	Sengon	Paraserianthes falcataria (L.) I.C.	Leguminosae / Fabaceae		0,33	18,5	11	18	0,04	0,03	119,54	0,12	1,40	5,15
4281	83	2	Ciamis	200-500	Petai	Parkia speciosa Hassk.	Leguminosae / Fabaceae		0,45	3,8	5	6	0,04	0,00	3,24	0,00	0,04	0,14
4282	83	2	Ciamis	200-500	Rambutan	Nephelium lappaceum L.	Sapindaceae		0,71	7,3	7	10	0,04	0,00	25,86	0,03	0,30	1,11
4283	83	2	Ciamis	200-500	Tisuk	Hibiscus macrophyllus Roxb. ex I	Malvaceae		0,4	4,5	3	6	0,04	0,00	3,86	0,00	0,05	0,17
4284	83	2	Ciamis	200-500	Durian	Durio zibethinus L.	Malvaceae		0,57	4,5	2,5	4	0,04	0,00	3,68	0,00	0,04	0,16
4285	83	2	Ciamis	200-500	Tisuk	Hibiscus macrophyllus Roxb. ex I	Malvaceae		0,4	3,5	4,5	5,5	0,04	0,00	2,29	0,00	0,03	0,10
4286	83	2	Ciamis	200-500	Tisuk	Hibiscus macrophyllus Roxb. ex I	Malvaceae		0,4	6,0	5	9	0,04	0,00	9,78	0,01	0,11	0,42
4287	83	2	Ciamis	200-500	Durian	Durio zibethinus L.	Malvaceae		0,57	4,5	2,5	5,5	0,04	0,00	4,92	0,00	0,06	0,21
4288	83	2	Ciamis	200-500	Sengon	Paraserianthes falcataria (L.) I.C.	Leguminosae / Fabaceae		0,33	14,6	13	16	0,04	0,02	70,18	0,07	0,82	3,03
4289	83	2	Ciamis	200-500	Sengon	Paraserianthes falcataria (L.) I.C.	Leguminosae / Fabaceae		0,33	17,5	11	15	0,04	0,02	91,77	0,09	1,08	3,96
4290	84	3	Ciamis	200-500	Aren	Arenga pinnata (Wurmb) Merr.	Arecaceae		0,71	39,5	4	7	0,04	0,12	58,40	0,06	0,69	2,52
4291	84	3	Ciamis	200-500	Mahoni	Swietenia macrophylla King	Meliaceae		0,61	4,5	2,5	5,5	0,04	0,00	5,24	0,01	0,06	0,23
4292	84	3	Ciamis	200-500	Dukuh	Lansium domesticum Corrêa	Meliaceae		0,85	18,5	5	9	0,04	0,03	150,71	0,15	1,77	6,50
4293	84	3	Ciamis	200-500	Jati	Tectona grandis L.f.	Lamiaceae / Verbenaceae		0,7	3,8	2	4	0,04	0,00	3,35	0,00	0,04	0,14
4294	84	3	Ciamis	200-500	Tisuk	Hibiscus macrophyllus Roxb. ex I	Malvaceae		0,4	3,2	2,5	3,5	0,04	0,00	1,27	0,00	0,01	0,05
4295	84	3	Ciamis	200-500	Tisuk	Hibiscus macrophyllus Roxb. ex I	Malvaceae		0,4	2,9	2	3,5	0,04	0,00	1,05	0,00	0,01	0,05
4296	84	3	Ciamis	200-500	Mahoni	Swietenia macrophylla King	Meliaceae		0,61	9,9	3,5	6,5	0,04	0,01	26,20	0,03	0,31	1,13
4297	84	3	Ciamis	200-500	Mahoni	Swietenia macrophylla King	Meliaceae		0,61	5,1	2,5	3,5	0,04	0,00	4,42	0,00	0,05	0,19
4298	84	3	Ciamis	200-500	Mahoni	Swietenia macrophylla King	Meliaceae		0,61	8,0	3,5	4,5	0,04	0,00	12,61	0,01	0,15	0,54
4299	84	3	Ciamis	200-500	Jati	Tectona grandis L.f.	Lamiaceae / Verbenaceae		0,7	4,1	3,5	4,5	0,04	0,00	4,32	0,00	0,05	0,19
4300	84	3	Ciamis	200-500	Mahoni	Swietenia macrophylla King	Meliaceae		0,61	5,7	3,5	4,5	0,04	0,00	6,91	0,01	0,08	0,30
4301	84	3	Ciamis	200-500	Tisuk	Hibiscus macrophyllus Roxb. ex I	Malvaceae		0,4	3,8	3,5	4,5	0,04	0,00	2,23	0,00	0,03	0,10
4302	84	3	Ciamis	200-500	Jati	Tectona grandis L.f.	Lamiaceae / Verbenaceae		0,7	4,5	3	4	0,04	0,00	4,44	0,00	0,05	0,19
4303	84	3	Ciamis	200-500	Tisuk	Hibiscus macrophyllus Roxb. ex I	Malvaceae		0,4	4,5	4,5	5,5	0,04	0,00	3,56	0,00	0,04	0,15
4304	84	3	Ciamis	200-500	Mahoni	Swietenia macrophylla King	Meliaceae		0,61	6,4	4	6	0,04	0,00	10,91	0,01	0,13	0,47
4305	84	3	Ciamis	200-500	Petai	Parkia speciosa Hassk.	Leguminosae / Fabaceae		0,45	4,1	4	6,5	0,04	0,00	4,03	0,00	0,05	0,17
4306	84	3	Ciamis	200-500	Sp8	Sp8		0	0,5	6,7	4,5	5,5	0,04	0,00	9,18	0,01	0,11	0,40
4307	84	3	Ciamis	200-500	Mahoni	Swietenia macrophylla King	Meliaceae		0,61	3,8	3,5	4	0,04	0,00	2,95	0,00	0,03	0,13
4308	84	3	Ciamis	200-500	Tisuk	Hibiscus macrophyllus Roxb. ex I	Malvaceae		0,4	6,7	6	7	0,04	0,00	9,33	0,01	0,11	0,40
4309	84	3	Ciamis	200-500	Kokosan	Lansium domesticum var. aquae	Meliaceae		0,7	5,7	2,5	6,5	0,04	0,00	10,98	0,01	0,13	0,47
4310	84	3	Ciamis	200-500	Tisuk	Hibiscus macrophyllus Roxb. ex I	Malvaceae		0,4	8,3	7	11	0,04	0,01	20,88	0,02	0,25	0,90
4311	84	3	Ciamis	200-500	Jati	Tectona grandis L.f.	Lamiaceae / Verbenaceae		0,7	13,4	4	7	0,04	0,01	55,48	0,06	0,65	2,39
4312	84	3	Ciamis	200-500	Tisuk	Hibiscus macrophyllus Roxb. ex I	Malvaceae		0,4	4,1	2,5	5,5	0,04	0,00	3,11	0,00	0,04	0,13
4313	84	3	Ciamis	200-500	Caruy	Pterospermum javanicum Jungh.	Malvaceae		0,53	8,6	4	7	0,04	0,01	19,14	0,02	0,22	0,83
4314	84	3	Ciamis	200-500	Nangka	Artocarpus heterophyllus Lam.	Moraceae		0,61	10,5	4	8	0,04	0,01	35,53	0,04	0,42	1,53
4315	84	3	Ciamis	200-500	Tisuk	Hibiscus macrophyllus Roxb. ex I	Malvaceae		0,4	4,5	3	5	0,04	0,00	3,26	0,00	0,04	0,14
4316	84	3	Ciamis	200-500	Mahoni	Swietenia macrophylla King	Meliaceae		0,61	7,3	3	6	0,04	0,00	14,09	0,01	0,17	0,61
4317	84	3	Ciamis	200-500	Tisuk	Hibiscus macrophyllus Roxb. ex I	Malvaceae		0,4	6,0	4	6,5	0,04	0,00	7,26	0,01	0,09	0,31
4318	84	3	Ciamis	200-500	Mahoni	Swietenia macrophylla King	Meliaceae		0,61	8,0	2,5	6,5	0,04	0,00	17,67	0,02	0,21	0,76
4319	84	3	Ciamis	200-500	Tisuk	Hibiscus macrophyllus Roxb. ex I	Malvaceae		0,4	5,1	4	6	0,04	0,00	4,92	0,00	0,06	0,21
4320	84	3	Ciamis	200-500	Tisuk	Hibiscus macrophyllus Roxb. ex I	Malvaceae		0,4	6,4	4	7	0,04	0,00	8,53	0,01	0,10	0,37
4321	84	3	Ciamis	200-500	Tisuk	Hibiscus macrophyllus Roxb. ex I	Malvaceae		0,4	3,8	3,5	5,5	0,04	0,00	2,68	0,00	0,03	0,12
4322	84	3	Ciamis	200-500	Tisuk	Hibiscus macrophyllus Roxb. ex I	Malvaceae		0,4	3,5	3,5	4,5	0,04	0,00	1,90	0,00	0,02	0,08
4323	84	3	Ciamis	200-500	Sp8	Sp8		0	0,5	9,5	6,5	8	0,04	0,01	24,87	0,02	0,29	1,07
4324	84	3	Ciamis	200-500	Tisuk	Hibiscus macrophyllus Roxb. ex I	Malvaceae		0,4	6,0	4,5	7,5	0,04	0,00	8,28	0,01	0,10	0,36
4325	84	3	Ciamis	200-500	Tisuk	Hibiscus macrophyllus Roxb. ex I	Malvaceae		0,4	7,0	6	9	0,04	0,00	12,79	0,01	0,15	0,55
4326	84	3	Ciamis	200-500	Tisuk	Hibiscus macrophyllus Roxb. ex I	Malvaceae		0,4	5,7	4	6	0,04	0,00	6,11	0,01	0,07	0,26
4327	84	3	Ciamis	200-500	Tisuk	Hibiscus macrophyllus Roxb. ex I	Malvaceae		0,4	7,0	4,5	6,5	0,04	0,00	9,50	0,01	0,11	0,41
4328	84	3	Ciamis	200-500	Sengon	Paraserianthes falcataria (L.) I.C.	Leguminosae / Fabaceae		0,33	13,0	8	12	0,04	0,01	43,67	0,04	0,51	1,88
4329	84	3	Ciamis	200-500	Tisuk	Hibiscus macrophyllus Roxb. ex I	Malvaceae		0,4	7,6	5	7,5	0,04	0,00	12,70	0,01	0,15	0,55
4330	84	3	Ciamis	200-500	Mahoni	Swietenia macrophylla King	Meliaceae		0,61	4,5	3	4,5	0,04	0,00	4,36	0,00	0,05	0,19
4331	84	3	Ciamis	200-500	Jati	Tectona grandis L.f.	Lamiaceae / Verbenaceae		0,7	10,2	3	8	0,04	0,01	38,10	0,04	0,45	1,64
4332	84	3	Ciamis	200-500	Tisuk	Hibiscus macrophyllus Roxb. ex I	Malvaceae		0,4	3,8	3,5	4,5	0,04	0,00	2,23	0,00	0,03	0,10
4333	84	3	Ciamis	200-500	Mahoni	Swietenia macrophylla King	Meliaceae		0,61	5,1	3	7	0,04	0,00	8,35	0,01	0,10	0,36
4334	84	3	Ciamis	200-500	Kihampelas	Ficus ampelas Burm.f.	Moraceae		0,38	13,7	3,5	7,5	0,04	0,01	35,26	0,04	0,41	1,52

4335	84	3	Ciamis	200-500	Mahoni	Swietenia macrophylla King	Meliaceae	0,61	3,2	2,5	3,5	0,04	0,00	1,87	0,00	0,02	0,08
4336	84	3	Ciamis	200-500	Dukuh	Lansium domesticum Corrêa	Meliaceae	0,85	3,8	2	3,5	0,04	0,00	3,54	0,00	0,04	0,15
4337	84	3	Ciamis	200-500	Benda/teureup	Artocarpus elasticus Reinw. ex B	Moraceae	0,37	8,0	4	5,5	0,04	0,00	9,59	0,01	0,11	0,41
4338	84	3	Ciamis	200-500	Tisuk	Hibiscus macrophyllus Roxb. ex	Malvaceae	0,4	3,2	3,5	4	0,04	0,00	1,44	0,00	0,02	0,06
4339	84	3	Ciamis	200-500	Tisuk	Hibiscus macrophyllus Roxb. ex	Malvaceae	0,4	4,1	2	4	0,04	0,00	2,32	0,00	0,03	0,10
4340	84	3	Ciamis	200-500	Tisuk	Hibiscus macrophyllus Roxb. ex	Malvaceae	0,4	5,1	2,5	5	0,04	0,00	4,17	0,00	0,05	0,18
4341	84	3	Ciamis	200-500	Tisuk	Hibiscus macrophyllus Roxb. ex	Malvaceae	0,4	3,5	3	4,5	0,04	0,00	1,90	0,00	0,02	0,08
4342	84	3	Ciamis	200-500	Afrika	Maesopsis eminii Engl.	Rhamnaceae	0,43	13,4	8	10	0,04	0,01	49,22	0,05	0,58	2,12
4343	84	3	Ciamis	200-500	Benda/teureup	Artocarpus elasticus Reinw. ex B	Moraceae	0,37	3,8	2,5	4	0,04	0,00	1,87	0,00	0,02	0,08
4344	85	1	Ciamis	200-500	Mahoni	Swietenia macrophylla King	Meliaceae	0,61	5,7	2,5	5,5	0,04	0,00	8,30	0,01	0,10	0,36
4345	85	1	Ciamis	200-500	Mahoni	Swietenia macrophylla King	Meliaceae	0,61	50,9	4	16	0,04	0,20	1.208,94	1,21	14,20	52,13
4346	85	1	Ciamis	200-500	Mahoni	Swietenia macrophylla King	Meliaceae	0,61	22,6	8	16	0,04	0,04	272,87	0,27	3,21	11,77
4347	85	1	Ciamis	200-500	Mahoni	Swietenia macrophylla King	Meliaceae	0,61	20,4	4,5	14,5	0,04	0,03	206,17	0,21	2,42	8,89
4348	85	1	Ciamis	200-500	Mahoni	Swietenia macrophylla King	Meliaceae	0,61	12,7	4	9	0,04	0,01	56,30	0,06	0,66	2,43
4349	85	1	Ciamis	200-500	Mahoni	Swietenia macrophylla King	Meliaceae	0,61	38,8	15	23	0,04	0,12	1.025,74	1,03	12,05	44,23
4350	85	1	Ciamis	200-500	Mahoni	Swietenia macrophylla King	Meliaceae	0,61	24,2	5	12	0,04	0,05	237,50	0,24	2,79	10,24
4351	85	1	Ciamis	200-500	Mahoni	Swietenia macrophylla King	Meliaceae	0,61	25,5	7	17	0,04	0,05	358,95	0,36	4,22	15,48
4352	85	1	Ciamis	200-500	Mahoni	Swietenia macrophylla King	Meliaceae	0,61	46,5	10	20	0,04	0,17	1.254,06	1,25	14,74	54,08
4353	85	1	Ciamis	200-500	Mahoni	Swietenia macrophylla King	Meliaceae	0,61	22,0	5,5	17,5	0,04	0,04	281,11	0,28	3,30	12,12
4354	85	1	Ciamis	200-500	Mahoni	Swietenia macrophylla King	Meliaceae	0,61	12,7	3,5	8,5	0,04	0,01	53,43	0,05	0,63	2,30
4355	85	1	Ciamis	200-500	Mahoni	Swietenia macrophylla King	Meliaceae	0,61	28,3	9	21	0,04	0,06	529,56	0,53	6,22	22,84
4356	85	1	Ciamis	200-500	Mahoni	Swietenia macrophylla King	Meliaceae	0,61	42,3	5	19	0,04	0,14	1.008,59	1,01	11,85	43,49
4357	85	1	Ciamis	200-500	Mahoni	Swietenia macrophylla King	Meliaceae	0,61	8,6	3,5	9,5	0,04	0,01	28,80	0,03	0,34	1,24
4358	85	1	Ciamis	200-500	Mahoni	Swietenia macrophylla King	Meliaceae	0,61	12,4	9	13	0,04	0,01	75,28	0,08	0,88	3,25
4359	85	1	Ciamis	200-500	Mahoni	Swietenia macrophylla King	Meliaceae	0,61	28,6	10	18	0,04	0,06	469,34	0,47	5,51	20,24
4360	85	1	Ciamis	200-500	Mahoni	Swietenia macrophylla King	Meliaceae	0,61	44,5	7	20	0,04	0,16	1.161,27	1,16	13,64	50,08
4361	85	1	Ciamis	200-500	Mahoni	Swietenia macrophylla King	Meliaceae	0,61	25,5	9	15	0,04	0,05	320,07	0,32	3,76	13,80
4362	85	1	Ciamis	200-500	Mahoni	Swietenia macrophylla King	Meliaceae	0,61	11,8	3	8	0,04	0,01	43,82	0,04	0,51	1,89
4363	85	1	Ciamis	200-500	Mahoni	Swietenia macrophylla King	Meliaceae	0,61	11,5	3,5	8,5	0,04	0,01	44,05	0,04	0,52	1,90
4364	85	1	Ciamis	200-500	Mahoni	Swietenia macrophylla King	Meliaceae	0,61	58,9	11	19	0,04	0,27	1.846,20	1,85	21,69	79,61
4365	85	1	Ciamis	200-500	Mahoni	Swietenia macrophylla King	Meliaceae	0,61	13,4	5	14	0,04	0,01	92,28	0,09	1,08	3,98
4366	85	1	Ciamis	200-500	Mahoni	Swietenia macrophylla King	Meliaceae	0,61	10,8	4	8	0,04	0,01	37,53	0,04	0,44	1,62
4367	85	1	Ciamis	200-500	Mahoni	Swietenia macrophylla King	Meliaceae	0,61	15,0	10	13	0,04	0,02	105,96	0,11	1,25	4,57
4368	85	1	Ciamis	200-500	Mahoni	Swietenia macrophylla King	Meliaceae	0,61	24,5	10	15	0,04	0,05	298,42	0,30	3,51	12,87
4369	85	1	Ciamis	200-500	Sengon	Paraserianthes falcataria (L.) I.C	Leguminosae / Fabaceae	0,33	17,2	5	11	0,04	0,02	66,79	0,07	0,78	2,88
4370	85	1	Ciamis	200-500	Sengon	Paraserianthes falcataria (L.) I.C	Leguminosae / Fabaceae	0,33	37,2	17	24	0,04	0,11	562,69	0,56	6,61	24,26
4371	85	1	Ciamis	200-500	Sengon	Paraserianthes falcataria (L.) I.C	Leguminosae / Fabaceae	0,33	22,9	5	10	0,04	0,04	103,68	0,10	1,22	4,47
4372	85	1	Ciamis	200-500	Sengon	Paraserianthes falcataria (L.) I.C	Leguminosae / Fabaceae	0,33	31,5	10	16	0,04	0,08	285,80	0,29	3,36	12,32
4373	85	1	Ciamis	200-500	Sengon	Paraserianthes falcataria (L.) I.C	Leguminosae / Fabaceae	0,33	29,3	9	16	0,04	0,07	249,87	0,25	2,94	10,77
4374	85	1	Ciamis	200-500	Sengon	Paraserianthes falcataria (L.) I.C	Leguminosae / Fabaceae	0,33	32,8	6	14	0,04	0,08	271,92	0,27	3,20	11,73
4375	85	1	Ciamis	200-500	Sengon	Paraserianthes falcataria (L.) I.C	Leguminosae / Fabaceae	0,33	18,5	2,5	11,5	0,04	0,03	79,30	0,08	0,93	3,42
4376	85	1	Ciamis	200-500	Sengon	Paraserianthes falcataria (L.) I.C	Leguminosae / Fabaceae	0,33	24,5	8	14	0,04	0,05	159,58	0,16	1,88	6,88
4377	85	1	Ciamis	200-500	Sengon	Paraserianthes falcataria (L.) I.C	Leguminosae / Fabaceae	0,33	19,7	12	16	0,04	0,03	121,26	0,12	1,42	5,23
4378	85	1	Ciamis	200-500	Sengon	Paraserianthes falcataria (L.) I.C	Leguminosae / Fabaceae	0,33	20,7	2,5	12,5	0,04	0,03	105,46	0,11	1,24	4,55
4379	86	3	Ciamis	200-500	Sengon	Paraserianthes falcataria (L.) I.C	Leguminosae / Fabaceae	0,33	39,5	12	18	0,04	0,12	480,90	0,48	5,65	20,74
4380	86	3	Ciamis	200-500	Tisuk	Hibiscus macrophyllus Roxb. ex	Malvaceae	0,4	8,6	4	9	0,04	0,01	18,62	0,02	0,22	0,80
4381	86	3	Ciamis	200-500	Bihbul	Vitex glabrata R.Br.	Lamiaceae / Verbenaceae	0,58	38,8	13	20	0,04	0,12	861,74	0,86	10,13	37,16
4382	86	3	Ciamis	200-500	Sengon	Paraserianthes falcataria (L.) I.C	Leguminosae / Fabaceae	0,33	25,5	16	24	0,04	0,05	280,42	0,28	3,29	12,09
4383	86	3	Ciamis	200-500	Sengon	Paraserianthes falcataria (L.) I.C	Leguminosae / Fabaceae	0,33	24,2	16	24	0,04	0,05	255,27	0,26	3,00	11,01
4384	86	3	Ciamis	200-500	Sengon	Paraserianthes falcataria (L.) I.C	Leguminosae / Fabaceae	0,33	14,0	13	18	0,04	0,02	72,06	0,07	0,85	3,11
4385	86	3	Ciamis	200-500	Tisuk	Hibiscus macrophyllus Roxb. ex	Malvaceae	0,4	4,8	3	5	0,04	0,00	3,70	0,00	0,04	0,16
4386	86	3	Ciamis	200-500	Wangkal	Albizia procera Benth	Leguminosae / Fabaceae	0,77	3,2	2,5	3,5	0,04	0,00	2,31	0,00	0,03	0,10
4387	86	3	Ciamis	200-500	Tisuk	Hibiscus macrophyllus Roxb. ex	Malvaceae	0,4	11,8	9	13	0,04	0,01	46,44	0,05	0,55	2,00
4388	86	3	Ciamis	200-500	Tisuk	Hibiscus macrophyllus Roxb. ex	Malvaceae	0,4	7,0	8	9,5	0,04	0,00	13,44	0,01	0,16	0,58
4389	86	3	Ciamis	200-500	Tisuk	Hibiscus macrophyllus Roxb. ex	Malvaceae	0,4	11,1	10	15	0,04	0,01	47,82	0,05	0,56	2,06
4390	86	3	Ciamis	200-500	sengon	Paraserianthes falcataria (L.) I.C	Leguminosae / Fabaceae	0,33	33,1	12	24	0,04	0,09	453,48	0,45	5,33	19,56
4391	86	3	Ciamis	200-500	sengon	Paraserianthes falcataria (L.) I.C	Leguminosae / Fabaceae	0,33	39,1	18	26	0,04	0,12	663,59	0,66	7,80	28,62
4392	86	3	Ciamis	200-500	sengon	Paraserianthes falcataria (L.) I.C	Leguminosae / Fabaceae	0,33	28,0	18	22	0,04	0,06	308,34	0,31	3,62	13,30
4393	86	3	Ciamis	200-500	Wangkal	Albizia procera Benth	Leguminosae / Fabaceae	0,77	4,8	35	5	0,04	0,00	6,75	0,01	0,08	0,29
4394	86	3	Ciamis	200-500	Sengon	Paraserianthes falcataria (L.) I.C	Leguminosae / Fabaceae	0,33	35,6	18	24	0,04	0,10	519,42	0,52	6,10	22,40
4395	86	3	Ciamis	200-500	Mahoni	Swietenia macrophylla King	Meliaceae	0,61	7,3	7	11	0,04	0,00	24,55	0,02	0,29	1,06
4396	86	3	Ciamis	200-500	Mahoni	Swietenia macrophylla King	Meliaceae	0,61	10,2	3	7	0,04	0,01	29,72	0,03	0,35	1,28
4397	86	3	Ciamis	200-500	Mahoni	Swietenia macrophylla King	Meliaceae	0,61	10,8	3,5	7	0,04	0,01	33,21	0,03	0,39	1,43
4398	86	3	Ciamis	200-500	Mahoni	Swietenia macrophylla King	Meliaceae	0,61	21,0	9	13	0,04	0,03	197,36	0,20	2,32	8,51
4399	86	3	Ciamis	200-500	Kokosan	Lansium domesticum var. aquae	Meliaceae	0,7	6,7	3	10	0,04	0,00	21,60	0,02	0,25	0,93
4400	86	3	Ciamis	200-500	mahoni	Swietenia macrophylla King	Meliaceae	0,61	15,9	4	11	0,04	0,02	101,84	0,10	1,20	4,39
4401	86	3	Ciamis	200-500	mahoni	Swietenia macrophylla King	Meliaceae	0,61	12,1	6,5	105	0,04	0,01	486,47	0,49	5,72	20,98
4402	86	3	Ciamis	200-500	mahoni	Swietenia macrophylla King	Meliaceae	0,61	4,1	3,5	4	0,04	0,00	3,42	0,00	0,04	0,15
4403	86	3	Ciamis	200-500	Bihbul	Vitex glabrata R.Br.	Lamiaceae / Verbenaceae	0,58	22,3	13	20	0,04	0,04	311,45	0,31	3,66	13,43
4404	86	3	Ciamis	200-500	Dukuh	Lansium domesticum Corrêa	Meliaceae	0,85	29,3	5	19	0,04	0,07	695,77	0,70	8,18	30,00
4405	86	3	Ciamis	200-500	Bihbul	Vitex glabrata R.Br.	Lamiaceae / Verbenaceae	0,58	13,7	9	13	0,04	0,01	85,96	0,09	1,01	3,71
4406	87	3	Ciamis	200-500	Sengon	Paraserianthes falcataria (L.) I.C	Leguminosae / Fabaceae	0,33	6,7	10	12	0,04	0,00	12,82	0,01	0,15	0,55
4407	87	3	Ciamis	200-500	Sengon	Paraserianthes falcataria (L.) I.C	Leguminosae / Fabaceae	0,33	3,5	9	12	0,04	0,00	3,92	0,00	0,05	0,17
4408	87	3	Ciamis	200-500	Sengon	Paraserianthes falcataria (L.) I.C	Leguminosae / Fabaceae	0,33	4,5	7	8	0,04	0,00	4,21	0,00	0,05	0,18
4409	87	3	Ciamis	200-500	Mahoni	Swietenia macrophylla King	Meliaceae	0,61	12,7	4	10	0,04	0,01	62,01	0,06	0,73	2,67
4410	87	3	Ciamis	200-500	Mahoni	Swietenia macrophylla King	Meliaceae	0,61	4,1	2	4	0,04	0,00	3,42	0,00	0,04	0,15
4411	87	3	Ciamis	200-500	Kokosan	Lansium domesticum var. aquae	Meliaceae	0,7	7,6	4,5	7	0,04	0,00	19,90	0,02	0,23	0,86
4412	87	3	Ciamis	200-500	Sengon	Paraserianthes falcataria (L.) I.C	Leguminosae / Fabaceae	0,33	0,6	510	13	0,04	0,00	0,19	0,00	0,00	0,01
4413	87	3	Ciamis	200-500	Sengon	Paraserianthes falcataria (L.) I.C	Leguminosae / Fabaceae	0,33	5,7	9	10	0,04	0,00	8,18	0,01	0,10	0,35
4414	87	3	Ciamis	200-500	Sengon	Paraserianthes falcataria (L.) I.C	Leguminosae / Fabaceae	0,33	10,8	10	12	0,04	0,01	30,99	0,03	0,36	1,34
4415	87	3	Ciamis	200-500	Sengon	Paraserianthes falcataria (L.) I.C	Leguminosae / Fabaceae	0,33	13,4	10	13	0,04	0,01	49,12	0,05	0,58	2,12
4416	87	3	Ciamis	200-500	Sengon	Paraserianthes falcataria (L.) I.C	Leguminosae / Fabaceae	0,33	16,9	11	15	0,04	0,02	85,75	0,09	1,01	3,70
4417	87	3	Ciamis	200-500	Sengon	Paraserianthes falcataria (L.) I.C	Leguminosae / Fabaceae	0,33	7,0	10	12	0,04	0,00	13,96	0,01	0,16	0,60
4418	87	3	Ciamis	200-500	Kokosan	Lansium domesticum var. aquae	Meliaceae	0,7	22,6	6	9	0,04	0,04	182,73	0,18	2,15	7,88
4419	87	3	Ciamis	200-500	Sengon	Paraserianthes falcataria (L.) I.C	Leguminosae / Fabaceae	0,33	19,7	10	13	0,04	0,03	100,26	0,10	1,18	4,32
4420	87	3	Ciamis	200-500	Sengon	Paraserianthes falcataria (L.) I.C	Leguminosae / Fabaceae	0,33	5,1	5	7	0,04	0,00	4,75	0,00	0,06	0,21
4421	87	3	Ciamis	200-500	Sengon	Paraserianthes falcataria (L.) I.C	Leguminosae / Fabaceae	0,33	3,5	4	7	0,04	0,00	2,39	0,00	0,03	0,10
4422	87	3	Ciamis	200-500	Mahoni	Swietenia macrophylla King	Meliaceae	0,61	17,8	3,5	10,5	0,04	0,02	120,11	0,12	1,41	5,18
4423	87	3	Ciamis	200-500	Sengon	Paraserianthes falcataria (L.) I.C	Leguminosae / Fabaceae	0,33	8,9	11	13	0,04	0,01	23,37	0,02	0,27	1,01
4424	87	3	Ciamis	200-500	Sengon	Paraserianthes falcataria (L.) I.C	Leguminosae / Fabaceae	0,33	15,3	11	14	0,04	0,02	67,14	0,07	0,79	2,90
4425	87	3	Ciamis	200-500	Sengon	Paraserianthes falcataria (L.) I.C	Leguminosae / Fabaceae	0,33	4,1	7	9	0,04	0,00	4,09	0,00	0,05	0,18
4426	87	3	Ciamis	200-500	Sengon	Paraserianthes falcataria (L.) I.C	Leguminosae / Fabaceae	0,33	6,4	8	11	0,04	0,00	10,83	0,01	0,13	0,47
4427	87	3	Ciamis	200-500	Rambutan	Nephelium lappaceum L.	Sapindaceae	0,71	4,5	2	4	0,04	0,00	4,50	0,00	0,05	0,19
4428	87	3	Ciamis	200-500	Sengon	Paraserianthes falcataria (L.) I.C	Leguminosae / Fabaceae	0,33	5,7	7	10	0,04	0,00	8,18	0,01	0,10	0,35
4429	87	3	Ciamis	200-500	Tisuk	Hibiscus macrophyllus Roxb. ex	Malvaceae	0,4	3,5	3,5	5,5	0,04	0,00	2,29	0,00	0,03	0,10
4430	87	3	Ciamis	200-500	Sengon	Paraserianthes falcataria (L.) I.C	Leguminosae / Fabaceae	0,33	5,1	7	9	0,04	0,00	5,99	0,01	0,07	0,26
4431	87	3	Ciamis	200-500	Tisuk	Hibiscus macrophyllus Roxb. ex	Malvaceae	0,4	3,8	4	6	0,04	0,00	2,91	0,00	0,03	0,13
4432	87	3	Ciamis	200-500	Sengon	Paraserianthes falcataria (L.) I.C	Leguminosae / Fabaceae	0,33	4,8	5	8	0,04	0,00	4,77	0,00	0,06	0,21
4433	87	3	Ciamis	200-500	Tisuk	Hibiscus macrophyllus Roxb. ex	Malvaceae	0,4	4,1	5	6	0,04	0,00	3,37	0,00	0,04	0,15
4434	87	3	Ciamis	200-500	Sengon	Paraserianthes falcataria (L.) I.C	Leguminosae / Fabaceae	0,33	9,2	11	14	0,04	0,01	26,67	0,03	0,31	1,15
4435	87	3	Ciamis	200-500	Sengon	Paraserianthes falcataria (L.) I.C	Leguminosae / Fabaceae	0,33	4,1	6	8	0,04	0,00	3,67	0,00	0,04	0,16
4436	87	3	Ciamis	200-500	Tisuk	Hibiscus macrophyllus Roxb. ex	Malvaceae	0,4	4,1	3,5	6,5	0,04	0,00	3,62	0,00	0,04	0,16
4437	87	3	Ciamis	200-500	Tisuk	Hibiscus macrophyllus Roxb. ex	Malvaceae	0,4	3,2	3,5	7	0,04	0,00	2,40	0,00	0,03	0,10
4438	87	3	Ciamis	200-500	Sengon	Paraserianthes falcataria (L.) I.C	Leguminosae / Fabaceae	0,33	8,9	10	12	0,04	0,01	21,72	0,02	0,26	0,94
4439	87	3	Ciamis	200-500	Manglid	Magnolia champaca (L.) Baill. ex	Magnoliaceae	0,53	7,0	2,5	7,5	0,04	0,00	14,01	0,01	0,16	0,60
4440	87	3	Ciamis	200-500	Kokosan	Lansium domesticum var. aquae	Meliaceae	0,7	1,6	5	9	0,04	0,00	1,42	0,00	0,02	0,06
4441	87	3	Ciamis	200-500	Mahoni	Swietenia macrophylla King	Meliaceae	0,61	4,1	6	8	0,04	0,00	6,45	0,01	0,08	0,28
4442	87	3	Ciamis	200-500	Sengon	Paraserianthes falcataria (L.) I.C	Leguminosae / Fabaceae	0,33	6,4	8	10	0,04	0,00	9,92	0,01	0,12	0,43
4443	87	3	Ciamis	200-500	Sengon	Paraserianthes falcataria (L.) I.C	Leguminosae / Fabaceae	0,33	6,4	7	9	0,04	0,00	9,01	0,01	0,11	0,39
4444	87	3	Ciamis	200-500	Sengon	Paraserianthes falcataria (L.) I.C	Leguminosae / Fabaceae	0,33	9,5	10	12	0,04	0,01	24,64	0,02	0,29	1,06
4445	87	3	Ciamis	200-500	Sengon	Paraserianthes falcataria (L.) I.C	Leguminosae / Fabaceae	0,33	6,0	8	9	0,04	0,00	8,20	0,01	0,10	0,35
4446	87	3	Ciamis	200-500	Sengon	Paraserianthes falcataria (L.) I.C	Leguminosae / Fabaceae	0,33	9,2	12	16	0,04	0,01	30,14	0,03	0,35	1,30
4447	87	3	Ciamis	200-500	Sengon	Paraserianthes falcataria (L.) I.C	Leguminosae / Fabaceae	0,33	15,3	12	16	0,04	0,02	75,87	0,08	0,89	3,27
4448	87	3	Ciamis	200-500	Kokosan	Lansium domesticum var. aquae	Meliaceae	0,7	7,0	10	12	0,04	0,00	27,80	0,03	0,33	1,20
4449	87	3	Ciamis	200-500	Sengon	Paraserianthes falcataria (L.) I.C	Leguminosae / Fabaceae	0,33	5,1	4	5,5	0,04	0,00	3,81	0,00	0,04	0,16
4450	87	3	Ciamis	200-500	Sengon	Paraserianthes falcataria (L.) I.C	Leguminosae / Fabaceae	0,33	2,9	7	10	0,04	0,00	2,30	0,00	0,03	0,10
4451	87	3	Ciamis	200-500	Sengon	Paraserianthes falcataria (L.) I.C	Leguminosae / Fabaceae	0,33	5,1	4	7	0,04	0,00	4,75	0,00	0,06	0,21
4452	87	3	Ciamis	200-500	Sengon	Paraserianthes falcataria (L.) I.C	Leguminosae / Fabaceae	0,33	4,1	6	8	0,04	0,00	3,67	0,00	0,04	0,16
4453	87	3	Ciamis	200-500	Pulai	Alstonia scholaris (L.) R. Br.	Apocynaceae	0,3	8,9	3,5	5,5	0,04	0,01	9,74	0,01	0,11	0,42
4454	87	3	Ciamis	200-500	Sengon	Paraserianthes falcataria (L.) I.C	Leguminosae / Fabaceae	0,33	8,3	8	10	0,04	0,01	16,04	0,02	0,19	0,69
4455	87	3	Ciamis	200-500	Mahoni	Swietenia macrophylla King	Meliaceae	0,61	4,5	3	5	0,04	0,00	4,80	0,00	0,06	0,21
4456	87	3	Ciamis	200-500	Sengon	Paraserianthes falcataria (L.) I.C	Leguminosae / Fabaceae	0,33	4,1	4	6	0,04	0,00	2,82	0,00	0,03	0,12
4457	87	3	Ciamis	200-500	Mahoni	Swietenia macrophylla King	Meliaceae	0,61	4,8	4	5	0,04	0,00	5,45	0,01	0,06	0,23
4458	87	3	Ciamis	200-500	Sengon	Paraserianthes falcataria (L.) I.C	Leguminosae / Fabaceae	0,33	13,7	13	18	0,04	0,01	69,09	0,07	0,81	2,98

4459	87	3	Ciamis	200-500	Tisuk	Hibiscus macrophyllus Roxb. ex I	Malvaceae	0,4	8,9	9	11	0,04	0,01	23,92	0,02	0,28	1,03
4460	87	3	Ciamis	200-500	Sengon	Paraserianthes falcataria (L.) I.C.	Leguminosae / Fabaceae	0,33	7,3	7	10	0,04	0,00	12,82	0,01	0,15	0,55
4461	87	3	Ciamis	200-500	Sengon	Paraserianthes falcataria (L.) I.C.	Leguminosae / Fabaceae	0,33	17,5	13	16	0,04	0,02	97,36	0,10	1,14	4,20
4462	87	3	Ciamis	200-500	Mahoni	Swietenia macrophylla King	Meliaceae	0,61	6,7	3,5	7,5	0,04	0,00	14,63	0,01	0,17	0,63
4463	87	3	Ciamis	200-500	Mahoni	Swietenia macrophylla King	Meliaceae	0,61	6,0	4	7	0,04	0,00	11,44	0,01	0,13	0,49
4464	87	3	Ciamis	200-500	Mahoni	Swietenia macrophylla King	Meliaceae	0,61	5,1	3,5	6,5	0,04	0,00	7,80	0,01	0,09	0,34
4465	87	3	Ciamis	200-500	Durian	Durio zibethinus L.	Malvaceae	0,57	11,5	10	13	0,04	0,01	61,10	0,06	0,72	2,63
4466	87	3	Ciamis	200-500	Mahoni	Swietenia macrophylla King	Meliaceae	0,61	6,7	3	7	0,04	0,00	13,74	0,01	0,16	0,59
4467	87	3	Ciamis	200-500	Mahoni	Swietenia macrophylla King	Meliaceae	0,61	4,5	3	7	0,04	0,00	6,54	0,01	0,08	0,28
4468	87	3	Ciamis	200-500	Dukuh	Lansium domesticum Corrêa	Meliaceae	0,85	9,5	6	9	0,04	0,01	45,04	0,05	0,53	1,94
4469	87	3	Ciamis	200-500	Mahoni	Swietenia macrophylla King	Meliaceae	0,61	12,4	3	8	0,04	0,01	48,25	0,05	0,57	2,08
4470	87	3	Ciamis	200-500	Mahoni	Swietenia macrophylla King	Meliaceae	0,61	19,1	6	8	0,04	0,03	106,24	0,11	1,25	4,58
4471	87	3	Ciamis	200-500	Mahoni	Swietenia macrophylla King	Meliaceae	0,61	28,6	6	12	0,04	0,06	323,73	0,32	3,80	13,96
4472	87	3	Ciamis	200-500	Tisuk	Hibiscus macrophyllus Roxb. ex I	Malvaceae	0,4	13,4	5	11	0,04	0,01	50,27	0,05	0,59	2,17
4473	87	3	Ciamis	200-500	Durian	Durio zibethinus L.	Malvaceae	0,57	4,5	2	4,5	0,04	0,00	4,10	0,00	0,05	0,18
4474	87	3	Ciamis	200-500	Mahoni	Swietenia macrophylla King	Meliaceae	0,61	13,7	4	10	0,04	0,01	70,79	0,07	0,83	3,05
4475	87	3	Ciamis	200-500	Mahoni	Swietenia macrophylla King	Meliaceae	0,61	3,8	2,5	4,5	0,04	0,00	3,29	0,00	0,04	0,14
4476	87	3	Ciamis	200-500	Sengon	Paraserianthes falcataria (L.) I.C.	Leguminosae / Fabaceae	0,33	43,9	13	22	0,04	0,15	703,07	0,70	8,26	30,32
4477	87	3	Ciamis	200-500	Durian	Durio zibethinus L.	Malvaceae	0,57	17,5	9	15	0,04	0,02	151,41	0,15	1,78	6,53
4478	87	3	Ciamis	200-500	Dukuh	Lansium domesticum Corrêa	Meliaceae	0,85	3,8	3,5	4,5	0,04	0,00	4,46	0,00	0,05	0,19
4479	87	3	Ciamis	200-500	Dukuh	Lansium domesticum Corrêa	Meliaceae	0,85	15,3	3,5	11,5	0,04	0,02	133,38	0,13	1,57	5,75
4480	87	3	Ciamis	200-500	Durian	Durio zibethinus L.	Malvaceae	0,57	15,9	7	12	0,04	0,02	103,64	0,10	1,22	4,47
4481	87	3	Ciamis	200-500	Kelapa	Cocos nucifera L.	Arecaceae	0,5	29,6	6	9	0,04	0,07	73,80	0,07	0,87	3,18
4482	87	3	Ciamis	200-500	Durian	Durio zibethinus L.	Malvaceae	0,57	3,5	2,5	4	0,04	0,00	2,36	0,00	0,03	0,10
4483	87	3	Ciamis	200-500	Dukuh	Lansium domesticum Corrêa	Meliaceae	0,85	3,8	2	3,5	0,04	0,00	3,54	0,00	0,04	0,15
4484	87	3	Ciamis	200-500	Tisuk	Hibiscus macrophyllus Roxb. ex I	Malvaceae	0,4	8,0	8	10	0,04	0,00	17,81	0,02	0,21	0,77
4485	87	3	Ciamis	200-500	Mahoni	Swietenia macrophylla King	Meliaceae	0,61	5,4	2	5	0,04	0,00	6,85	0,01	0,08	0,30
4486	87	3	Ciamis	200-500	Mahoni	Swietenia macrophylla King	Meliaceae	0,61	15,3	4	10	0,04	0,02	86,60	0,09	1,02	3,73
4487	87	3	Ciamis	200-500	Mahoni	Swietenia macrophylla King	Meliaceae	0,61	3,8		3,5	0,04	0,00	2,61	0,00	0,03	0,11
4488	87	3	Ciamis	200-500	Kelapa	Cocos nucifera L.	Arecaceae	0,5	22,9	6	9	0,04	0,04	73,80	0,07	0,87	3,18
4489	87	3	Ciamis	200-500	Sengon	Paraserianthes falcataria (L.) I.C.	Leguminosae / Fabaceae	0,33	10,5	8	10	0,04	0,01	24,83	0,02	0,29	1,07
4490	87	3	Ciamis	200-500	Tisuk	Hibiscus macrophyllus Roxb. ex I	Malvaceae	0,4	7,6	6	9	0,04	0,00	15,01	0,02	0,18	0,65
4491	87	3	Ciamis	200-500	Mahoni	Swietenia macrophylla King	Meliaceae	0,61	9,9	4	7	0,04	0,01	28,04	0,03	0,33	1,21
4492	87	3	Ciamis	200-500	Sengon	Paraserianthes falcataria (L.) I.C.	Leguminosae / Fabaceae	0,33	8,3	10	12	0,04	0,01	18,96	0,02	0,22	0,82
4493	87	3	Ciamis	200-500	Rambutan	Nephelium lappaceum L.	Sapindaceae	0,71	11,1	7	11	0,04	0,01	60,89	0,06	0,72	2,63
4494	87	3	Ciamis	200-500	Mahoni	Swietenia macrophylla King	Meliaceae	0,61	15,3	4	9	0,04	0,02	78,63	0,08	0,92	3,39
4495	87	3	Ciamis	200-500	Dukuh	Lansium domesticum Corrêa	Meliaceae	0,85	5,1	3	6	0,04	0,00	9,82	0,01	0,12	0,42
4496	87	3	Ciamis	200-500	Dukuh	Lansium domesticum Corrêa	Meliaceae	0,85	13,7	3,5	8,5	0,04	0,01	82,67	0,08	0,97	3,56
4497	87	3	Ciamis	200-500	Kokosan	Lansium domesticum var. aquae	Meliaceae	0,7	5,7	3	6	0,04	0,00	10,20	0,01	0,12	0,44
4498	87	3	Ciamis	200-500	Sengon	Paraserianthes falcataria (L.) I.C.	Leguminosae / Fabaceae	0,33	8,6	9	12	0,04	0,01	20,32	0,02	0,24	0,88
4499	88	3	Ciamis	200-500	Bihbul	Vitex glabrata R.Br.	Lamiaceae / Verbenaceae	0,58	10,5	10	13	0,04	0,01	52,93	0,05	0,62	2,28
4500	88	3	Ciamis	200-500	Kelapa	Cocos nucifera L.	Arecaceae	0,5	27,4	12	15	0,04	0,06	120,00	0,12	1,41	5,17
4501	88	3	Ciamis	200-500	Mahoni	Swietenia macrophylla King	Meliaceae	0,61	17,5	7	14	0,04	0,02	151,24	0,15	1,78	6,52
4502	88	3	Ciamis	200-500	Sp8	Sp8	0	0,5	6,0	2	3,5	0,04	0,00	5,05	0,01	0,06	0,22
4503	88	3	Ciamis	200-500	Pisitan	Dysoxylum parasiticum (Osbeck	Meliaceae	0,6	10,5	6	10	0,04	0,01	42,94	0,04	0,50	1,85
4504	88	3	Ciamis	200-500	Jengkol	Archidendron pauciflorum (Bentl	Leguminosae / Fabaceae	0,4	25,5	9	15	0,04	0,05	217,45	0,22	2,56	9,38
4505	88	3	Ciamis	200-500	Caruy	Pterospermum javanicum Jungh.	Malvaceae	0,53	3,8	5	7	0,04	0,00	4,33	0,00	0,05	0,19
4506	88	3	Ciamis	200-500	Kokosan	Lansium domesticum var. aquae	Meliaceae	0,7	3,2	4	5,5	0,04	0,00	3,21	0,00	0,04	0,14
4507	88	3	Ciamis	200-500	Rambutan	Nephelium lappaceum L.	Sapindaceae	0,71	6,7	7	10	0,04	0,00	21,89	0,02	0,26	0,94
4508	88	3	Ciamis	200-500	Bihbul	Vitex glabrata R.Br.	Lamiaceae / Verbenaceae	0,58	19,7	11	18	0,04	0,03	226,42	0,23	2,66	9,76
4509	88	3	Ciamis	200-500	Mahoni	Swietenia macrophylla King	Meliaceae	0,61	19,7	8	16	0,04	0,03	212,87	0,21	2,50	9,18
4510	88	3	Ciamis	200-500	Kokosan	Lansium domesticum var. aquae	Meliaceae	0,7	4,5	3,5	4,5	0,04	0,00	4,95	0,00	0,06	0,21
4511	88	3	Ciamis	200-500	Kokosan	Lansium domesticum var. aquae	Meliaceae	0,7	4,5	3,5	4,5	0,04	0,00	4,95	0,00	0,06	0,21
4512	88	3	Ciamis	200-500	Bungbulang	Premna tomentosa Willd.	Lamiaceae / Verbenaceae	0,96	8,9	6	8	0,04	0,01	39,84	0,04	0,47	1,72
4513	88	3	Ciamis	200-500	Mahoni	Swietenia macrophylla King	Meliaceae	0,61	23,9	4,5	11	0,04	0,04	214,05	0,21	2,52	9,23
4514	88	3	Ciamis	200-500	Heuras	Vitex pubescens Vahl	Lamiaceae / Verbenaceae	0,88	13,0	7	13	0,04	0,01	115,41	0,12	1,36	4,98
4515	88	3	Ciamis	200-500	Heuras	Vitex pubescens Vahl	Lamiaceae / Verbenaceae	0,88	12,4	7	13	0,04	0,01	105,31	0,11	1,24	4,54
4516	88	3	Ciamis	200-500	Jengkol	Archidendron pauciflorum (Bentl	Leguminosae / Fabaceae	0,4	8,6	6	9	0,04	0,01	18,62	0,02	0,22	0,80
4517	88	3	Ciamis	200-500	Pisitan	Dysoxylum parasiticum (Osbeck	Meliaceae	0,6	3,5	2	3,5	0,04	0,00	2,19	0,00	0,03	0,09
4518	88	3	Ciamis	200-500	Tisuk	Hibiscus macrophyllus Roxb. ex I	Malvaceae	0,4	8,0	10	14	0,04	0,00	24,24	0,02	0,28	1,05
4519	88	3	Ciamis	200-500	Heuras	Vitex pubescens Vahl	Lamiaceae / Verbenaceae	0,88	10,5	8	14	0,04	0,01	82,99	0,08	0,98	3,58
4520	88	3	Ciamis	200-500	Rambutan	Nephelium lappaceum L.	Sapindaceae	0,71	4,5	4	6	0,04	0,00	6,52	0,01	0,08	0,28
4521	88	3	Ciamis	200-500	Melinjo	Gnetum gnemon L.	Gnetaceae	0,76	3,5	2	3,5	0,04	0,00	2,72	0,00	0,03	0,12
4522	88	3	Ciamis	200-500	Bihbul	Vitex glabrata R.Br.	Lamiaceae / Verbenaceae	0,58	11,5	10	13	0,04	0,01	62,08	0,06	0,73	2,68
4523	88	3	Ciamis	200-500	Bihbul	Vitex glabrata R.Br.	Lamiaceae / Verbenaceae	0,58	18,1	14	17	0,04	0,03	184,19	0,18	2,16	7,94
4524	88	3	Ciamis	200-500	Afrika	Maesopsis eminii Engl.	Rhamnaceae	0,43	8,0	10	11	0,04	0,00	20,76	0,02	0,24	0,90
4525	88	3	Ciamis	200-500	Melinjo	Gnetum gnemon L.	Gnetaceae	0,76	7,6	2,5	8,5	0,04	0,00	25,64	0,03	0,30	1,11
4526	88	3	Ciamis	200-500	Kihampelas	Ficus ampelas Burm.f.	Moraceae	0,38	7,6	5	11	0,04	0,00	17,21	0,02	0,20	0,74
4527	88	3	Ciamis	200-500	Heuras	Vitex pubescens Vahl	Lamiaceae / Verbenaceae	0,88	4,5	6,5	7,5	0,04	0,00	9,74	0,01	0,11	0,42
4528	88	3	Ciamis	200-500	Tisuk	Hibiscus macrophyllus Roxb. ex I	Malvaceae	0,4	8,9	8	14	0,04	0,01	29,83	0,03	0,35	1,29
4529	88	3	Ciamis	200-500	Sengon	Paraserianthes falcataria (L.) I.C.	Leguminosae / Fabaceae	0,33	19,7	13	20	0,04	0,03	148,76	0,15	1,75	6,41
4530	88	3	Ciamis	200-500	Heuras	Vitex pubescens Vahl	Lamiaceae / Verbenaceae	0,88	7,6	6	11	0,04	0,00	37,13	0,04	0,44	1,60
4531	88	3	Ciamis	200-500	Sengon	Paraserianthes falcataria (L.) I.C.	Leguminosae / Fabaceae	0,33	27,7	18	22	0,04	0,06	301,95	0,30	3,55	13,02
4532	88	3	Ciamis	200-500	Sengon	Paraserianthes falcataria (L.) I.C.	Leguminosae / Fabaceae	0,33	13,7	9,5	13,5	0,04	0,01	53,09	0,05	0,62	2,29
4533	88	3	Ciamis	200-500	Rambutan	Nephelium lappaceum L.	Sapindaceae	0,71	6,7	6	10	0,04	0,00	21,89	0,02	0,26	0,94
4534	88	3	Ciamis	200-500	Jengkol	Archidendron pauciflorum (Bentl	Leguminosae / Fabaceae	0,4	5,1	6	7	0,04	0,00	5,67	0,01	0,07	0,24
4535	88	3	Ciamis	200-500	Sengon	Paraserianthes falcataria (L.) I.C.	Leguminosae / Fabaceae	0,33	8,0	12	14	0,04	0,00	20,32	0,02	0,24	0,88
4536	88	3	Ciamis	200-500	Bihbul	Vitex glabrata R.Br.	Lamiaceae / Verbenaceae	0,58	15,6	12	16	0,04	0,02	132,08	0,13	1,55	5,70
4537	88	3	Ciamis	200-500	Bihbul	Vitex glabrata R.Br.	Lamiaceae / Verbenaceae	0,58	9,5	12	15	0,04	0,01	50,68	0,05	0,60	2,19
4538	88	3	Ciamis	200-500	Melinjo	Gnetum gnemon L.	Gnetaceae	0,76	4,5	3	5	0,04	0,00	5,87	0,01	0,07	0,25
4539	88	3	Ciamis	200-500	Pisitan	Dysoxylum parasiticum (Osbeck	Meliaceae	0,6	8,9	10	11	0,04	0,01	34,67	0,03	0,41	1,50
4540	88	3	Ciamis	200-500	Rambutan	Nephelium lappaceum L.	Sapindaceae	0,71	6,0	6,5	10	0,04	0,00	18,22	0,02	0,21	0,79
4541	88	3	Ciamis	200-500	Jengkol	Archidendron pauciflorum (Bentl	Leguminosae / Fabaceae	0,4	16,5	12	15	0,04	0,02	98,77	0,10	1,16	4,26
4542	88	3	Ciamis	200-500	Tisuk	Hibiscus macrophyllus Roxb. ex I	Malvaceae	0,4	13,4	12	15	0,04	0,01	66,79	0,07	0,78	2,88
4543	88	3	Ciamis	200-500	Melinjo	Gnetum gnemon L.	Gnetaceae	0,76	4,1	2	6	0,04	0,00	6,06	0,01	0,07	0,26
4544	88	3	Ciamis	200-500	Heuras	Vitex pubescens Vahl	Lamiaceae / Verbenaceae	0,88	4,8	2	5	0,04	0,00	7,62	0,01	0,09	0,33
4545	88	3	Ciamis	200-500	Heuras	Vitex pubescens Vahl	Lamiaceae / Verbenaceae	0,88	6,0	6	9	0,04	0,00	20,14	0,02	0,24	0,87
4546	88	3	Ciamis	200-500	Melinjo	Gnetum gnemon L.	Gnetaceae	0,76	15,9	3	11	0,04	0,02	124,56	0,12	1,46	5,37
4547	88	3	Ciamis	200-500	Sengon	Paraserianthes falcataria (L.) I.C.	Leguminosae / Fabaceae	0,33	16,9	14	18	0,04	0,02	101,34	0,10	1,19	4,37
4548	88	3	Ciamis	200-500	Sengon	Paraserianthes falcataria (L.) I.C.	Leguminosae / Fabaceae	0,33	11,5	14	17	0,04	0,01	47,35	0,05	0,56	2,04
4549	88	3	Ciamis	200-500	Bihbul	Vitex glabrata R.Br.	Lamiaceae / Verbenaceae	0,58	13,4	13	15	0,04	0,01	93,87	0,09	1,10	4,05
4550	88	3	Ciamis	200-500	Kayu Gunung	Sp1	0	0,5	7,3	6	9	0,04	0,00	17,03	0,02	0,20	0,73
4551	88	3	Ciamis	200-500	Mahoni	Swietenia macrophylla King	Meliaceae	0,61	5,1	5	6	0,04	0,00	7,25	0,01	0,09	0,31
4552	88	3	Ciamis	200-500	Mahoni	Swietenia macrophylla King	Meliaceae	0,61	5,1	4	5,5	0,04	0,00	6,69	0,01	0,08	0,29
4553	88	3	Ciamis	200-500	Tisuk	Hibiscus macrophyllus Roxb. ex I	Malvaceae	0,4	6,0	6	11	0,04	0,00	11,75	0,01	0,14	0,51
4554	88	3	Ciamis	200-500	Heuras	Vitex pubescens Vahl	Lamiaceae / Verbenaceae	0,88	6,4	6,5	7,5	0,04	0,00	18,72	0,02	0,22	0,81
4555	88	3	Ciamis	200-500	Pisitan	Dysoxylum parasiticum (Osbeck	Meliaceae	0,6	13,4	7	11	0,04	0,01	72,88	0,07	0,86	3,14
4556	88	3	Ciamis	200-500	Tisuk	Hibiscus macrophyllus Roxb. ex I	Malvaceae	0,4	10,8	7	12	0,04	0,01	36,97	0,04	0,43	1,59
4557	88	3	Ciamis	200-500	Tisuk	Hibiscus macrophyllus Roxb. ex I	Malvaceae	0,4	6,4	6	8	0,04	0,00	9,65	0,01	0,11	0,42
4558	88	3	Ciamis	200-500	Heuras	Vitex pubescens Vahl	Lamiaceae / Verbenaceae	0,88	3,2	3,5	5,5	0,04	0,00	3,96	0,00	0,05	0,17
4559	88	3	Ciamis	200-500	Rambutan	Nephelium lappaceum L.	Sapindaceae	0,71	13,4	7	11	0,04	0,01	85,03	0,09	1,00	3,67
4560	88	3	Ciamis	200-500	Heuras	Vitex pubescens Vahl	Lamiaceae / Verbenaceae	0,88	5,4	5	9	0,04	0,00	16,43	0,02	0,19	0,71
4561	88	3	Ciamis	200-500	Mahoni	Swietenia macrophylla King	Meliaceae	0,61	8,3	4	8	0,04	0,01	22,96	0,02	0,27	0,99
4562	88	3	Ciamis	200-500	Bihbul	Vitex glabrata R.Br.	Lamiaceae / Verbenaceae	0,58	17,5	13	19	0,04	0,02	191,03	0,19	2,24	8,24
4563	88	3	Ciamis	200-500	Mahoni	Swietenia macrophylla King	Meliaceae	0,61	16,9	8	14	0,04	0,02	141,32	0,14	1,66	6,09
4564	88	3	Ciamis	200-500	Mahoni	Swietenia macrophylla King	Meliaceae	0,61	15,0	4	9	0,04	0,02	75,66	0,08	0,89	3,26
4565	88	3	Ciamis	200-500	Mahoni	Swietenia macrophylla King	Meliaceae	0,61	20,7	11	18	0,04	0,03	258,56	0,26	3,04	11,15
4566	88	3	Ciamis	200-500	Nangka	Artocarpus heterophyllus Lam.	Moraceae	0,61	3,5	2	5	0,04	0,00	3,09	0,00	0,04	0,13
4567	88	3	Ciamis	200-500	Kelapa	Cocos nucifera L.	Arecaceae	0,5	29,0	11	14	0,04	0,07	112,30	0,11	1,32	4,84
4568	89	1	Ciamis	200-500	Kayu Gunung	Sp1	0	0,5	30,9	18	23	0,04	0,07	561,67	0,56	6,60	24,22
4569	89	1	Ciamis	200-500	Kokosan	Lansium domesticum var. aquae	Meliaceae	0,7	11,1	3	70	0,04	0,01	327,39	0,33	3,85	14,12
4570	89	1	Ciamis	200-500	Pangsor	Ficus callosa Willd.	Moraceae	0,33	25,5	12	18	0,04	0,05	215,46	0,22	2,53	9,29
4571	89	1	Ciamis	200-500	Mahoni	Swietenia macrophylla King	Meliaceae	0,61	4,1	5	8	0,04	0,00	6,45	0,01	0,08	0,28
4572	89	1	Ciamis	200-500	Renghas	Gluta renghas L.	Anacardiaceae	0,69	28,6	11	16	0,04	0,06	471,68	0,47	5,54	20,34
4573	89	1	Ciamis	200-500	Mahoni	Swietenia macrophylla King	Meliaceae	0,61	14,0	5	11	0,04	0,02	80,58	0,08	0,95	3,47
4574	89	1	Ciamis	200-500	Mahoni	Swietenia macrophylla King	Meliaceae	0,61	12,1	5	10	0,04	0,01	56,45	0,06	0,66	2,43
4575	89	1	Ciamis	200-500	Sengon	Paraserianthes falcataria (L.) I.C.	Leguminosae / Fabaceae	0,33	18,1	15	21	0,04	0,03	133,35	0,13	1,57	5,75
4576	89	1	Ciamis	200-500	Tisuk	Hibiscus macrophyllus Roxb. ex I	Malvaceae	0,4	5,7	5	7	0,04	0,00	7,04	0,01	0,08	0,30
4577	89	1	Ciamis	200-500	Pisitan	Dysoxylum parasiticum (Osbeck	Meliaceae	0,6	10,2	6	9	0,04	0,01	36,85	0,04	0,43	1,59
4578	89	1	Ciamis	200-500	Tisuk	Hibiscus macrophyllus Roxb. ex I	Malvaceae	0,4	4,8	2,5	5,5	0,04	0,00	4,04	0,00	0,05	0,17
4579	89	1	Ciamis	200-500	Kokosan	Lansium domesticum var. aquae	Meliaceae	0,7	8,3	4	8	0,04	0,01	26,04	0,03	0,31	1,12
4580	89	1	Ciamis	200-500	Tisuk	Hibiscus macrophyllus Roxb. ex I	Malvaceae	0,4	17,5	12	15	0,04	0,02	109,46	0,11	1,29	4,72
4581	89	1	Ciamis	200-500	Kokosan	Lansium domesticum var. aquae	Meliaceae	0,7	7,3	4	8	0,04	0,00	20,80	0,02	0,24	0,90
4582	89	1	Ciamis	200-500	Jati	Tectona grandis L.f.	Lamiaceae / Verbenaceae	0,7	9,9	7	10	0,04	0,01	44,10	0,04	0,52	1,90

4583	89	1	Ciamis	200-500	Jati	Tectona grandis L.f.	Lamiaceae / Verbenaceae	0,7	21,0	3	14	0,04	0,03	239,60	0,24	2,82	10,33
4584	89	1	Ciamis	200-500	Tisuk	Hibiscus macrophyllus Roxb. ex	Malvaceae	0,4	7,6	7	12	0,04	0,00	19,53	0,02	0,23	0,84
4585	89	1	Ciamis	200-500	Jati	Tectona grandis L.f.	Lamiaceae / Verbenaceae	0,7	19,7	4	13	0,04	0,03	199,65	0,20	2,35	8,61
4586	89	1	Ciamis	200-500	Jati	Tectona grandis L.f.	Lamiaceae / Verbenaceae	0,7	19,1	5	16	0,04	0,03	227,39	0,23	2,67	9,81
4587	89	1	Ciamis	200-500	Mahoni	Swietenia macrophylla King	Meliaceae	0,61	13,7	7	11	0,04	0,01	77,25	0,08	0,91	3,33
4588	89	1	Ciamis	200-500	Mahoni	Swietenia macrophylla King	Meliaceae	0,61	7,6	5	8	0,04	0,00	19,83	0,02	0,23	0,85
4589	89	1	Ciamis	200-500	Jati	Tectona grandis L.f.	Lamiaceae / Verbenaceae	0,7	16,9	5	14	0,04	0,02	160,31	0,16	1,88	6,91
4590	89	1	Ciamis	200-500	Jati	Tectona grandis L.f.	Lamiaceae / Verbenaceae	0,7	11,1	5	12	0,04	0,01	65,09	0,07	0,76	2,81
4591	89	1	Ciamis	200-500	Tisuk	Hibiscus macrophyllus Roxb. ex	Malvaceae	0,4	9,9	10	14	0,04	0,01	35,94	0,04	0,42	1,55
4592	89	1	Ciamis	200-500	Jati	Tectona grandis L.f.	Lamiaceae / Verbenaceae	0,7	14,6	4	14	0,04	0,02	123,67	0,12	1,45	5,33
4593	89	1	Ciamis	200-500	Jati	Tectona grandis L.f.	Lamiaceae / Verbenaceae	0,7	15,3	7	14	0,04	0,02	133,70	0,13	1,57	5,77
4594	89	1	Ciamis	200-500	Tisuk	Hibiscus macrophyllus Roxb. ex	Malvaceae	0,4	8,3	6,5	10,5	0,04	0,01	20,01	0,02	0,24	0,86
4595	89	1	Ciamis	200-500	Jati	Tectona grandis L.f.	Lamiaceae / Verbenaceae	0,7	8,3	3,5	8,5	0,04	0,01	27,53	0,03	0,32	1,19
4596	89	1	Ciamis	200-500	Jati	Tectona grandis L.f.	Lamiaceae / Verbenaceae	0,7	19,7	5	14	0,04	0,03	213,67	0,21	2,51	9,21
4597	89	1	Ciamis	200-500	Jati	Tectona grandis L.f.	Lamiaceae / Verbenaceae	0,7	19,4	4	13	0,04	0,03	193,79	0,19	2,28	8,36
4598	89	1	Ciamis	200-500	Mahoni	Swietenia macrophylla King	Meliaceae	0,61	7,6	7	10	0,04	0,00	24,32	0,02	0,29	1,05
4599	89	1	Ciamis	200-500	Sengon	Paraserianthes falcataria (L.) I.C.	Leguminosae / Fabaceae	0,33	18,5	15	20	0,04	0,03	131,65	0,13	1,55	5,68
4600	89	1	Ciamis	200-500	Mahoni	Swietenia macrophylla King	Meliaceae	0,61	8,3	7	10	0,04	0,01	28,17	0,03	0,33	1,21
4601	89	1	Ciamis	200-500	Mahoni	Swietenia macrophylla King	Meliaceae	0,61	19,1	9	17	0,04	0,03	211,91	0,21	2,49	9,14
4602	89	1	Ciamis	200-500	Jati	Tectona grandis L.f.	Lamiaceae / Verbenaceae	0,7	22,3	10	18	0,04	0,04	335,95	0,34	3,95	14,49
4603	89	1	Ciamis	200-500	Jati	Tectona grandis L.f.	Lamiaceae / Verbenaceae	0,7	19,7	10	16	0,04	0,03	241,47	0,24	2,84	10,41
4604	89	1	Ciamis	200-500	Jati	Tectona grandis L.f.	Lamiaceae / Verbenaceae	0,7	21,0	7	16	0,04	0,03	270,77	0,27	3,18	11,68
4605	89	1	Ciamis	200-500	Mahoni	Swietenia macrophylla King	Meliaceae	0,61	15,6	6	13	0,04	0,02	114,36	0,11	1,34	4,93
4606	89	1	Ciamis	200-500	Jati	Tectona grandis L.f.	Lamiaceae / Verbenaceae	0,7	10,5	9	12	0,04	0,01	58,44	0,06	0,69	2,52
4607	89	1	Ciamis	200-500	Jati	Tectona grandis L.f.	Lamiaceae / Verbenaceae	0,7	19,1	9	16	0,04	0,03	227,39	0,23	2,67	9,81
4608	89	1	Ciamis	200-500	Jati	Tectona grandis L.f.	Lamiaceae / Verbenaceae	0,7	15,6	7	16	0,04	0,02	156,91	0,16	1,84	6,77
4609	89	1	Ciamis	200-500	Jati	Tectona grandis L.f.	Lamiaceae / Verbenaceae	0,7	16,2	7	14	0,04	0,02	149,40	0,15	1,76	6,44
4610	89	1	Ciamis	200-500	Jati	Tectona grandis L.f.	Lamiaceae / Verbenaceae	0,7	14,3	3,5	8,5	0,04	0,02	75,21	0,08	0,88	3,24
4611	89	1	Ciamis	200-500	Jati	Tectona grandis L.f.	Lamiaceae / Verbenaceae	0,7	11,8	8	12	0,04	0,01	72,06	0,07	0,85	3,11
4612	89	1	Ciamis	200-500	Mahoni	Swietenia macrophylla King	Meliaceae	0,61	18,5	8	16	0,04	0,03	188,39	0,19	2,21	8,12
4613	89	1	Ciamis	200-500	Kelapa	Cocos nucifera L.	Arecaceae	0,5	29,0	13	16	0,04	0,07	127,70	0,13	1,50	5,51
4614	89	1	Ciamis	200-500	Jati	Tectona grandis L.f.	Lamiaceae / Verbenaceae	0,7	11,1	4	11	0,04	0,01	60,10	0,06	0,71	2,59
4615	89	1	Ciamis	200-500	Jati	Tectona grandis L.f.	Lamiaceae / Verbenaceae	0,7	19,4	8	15	0,04	0,03	220,93	0,22	2,60	9,53
4616	89	1	Ciamis	200-500	Jati	Tectona grandis L.f.	Lamiaceae / Verbenaceae	0,7	32,8	6	16	0,04	0,08	611,96	0,61	7,19	26,39
4617	89	1	Ciamis	200-500	Jati	Tectona grandis L.f.	Lamiaceae / Verbenaceae	0,7	20,7	6	15	0,04	0,03	248,19	0,25	2,92	10,70
4618	89	1	Ciamis	200-500	Mahoni	Swietenia macrophylla King	Meliaceae	0,61	9,9	3,5	8,5	0,04	0,01	33,50	0,03	0,39	1,44
4619	89	1	Ciamis	200-500	Tisuk	Hibiscus macrophyllus Roxb. ex	Malvaceae	0,4	6,4	9	11	0,04	0,00	12,91	0,01	0,15	0,56
4620	89	1	Ciamis	200-500	Mahoni	Swietenia macrophylla King	Meliaceae	0,61	9,5	4	7	0,04	0,01	26,40	0,03	0,31	1,14
4621	89	1	Ciamis	200-500	Mahoni	Swietenia macrophylla King	Meliaceae	0,61	9,5	4	11	0,04	0,01	39,95	0,04	0,47	1,72
4622	89	1	Ciamis	200-500	Pangsor	Ficus callosa Willd.	Moraceae	0,33	20,7	12	77	0,04	0,03	557,65	0,56	6,55	24,05
4623	89	1	Ciamis	200-500	Kelapa	Cocos nucifera L.	Arecaceae	0,5	31,5	15	18	0,04	0,08	143,10	0,14	1,68	6,17
4624	89	1	Ciamis	200-500	Mahoni	Swietenia macrophylla King	Meliaceae	0,61	5,4	4	7	0,04	0,00	9,33	0,01	0,11	0,40
4625	89	1	Ciamis	200-500	Mahoni	Swietenia macrophylla King	Meliaceae	0,61	9,2	6	10	0,04	0,01	34,40	0,03	0,40	1,48
4626	89	1	Ciamis	200-500	Mahoni	Swietenia macrophylla King	Meliaceae	0,61	11,5	8	12	0,04	0,01	60,42	0,06	0,71	2,61
4627	89	1	Ciamis	200-500	Mahoni	Swietenia macrophylla King	Meliaceae	0,61	4,5	6	8	0,04	0,00	7,39	0,01	0,09	0,32
4628	90	2	Ciamis	200-500	Mahoni	Swietenia macrophylla King	Meliaceae	0,61	16,9	9	13	0,04	0,02	132,05	0,13	1,55	5,69
4629	90	2	Ciamis	200-500	Sengon	Paraserianthes falcataria (L.) I.C.	Leguminosae / Fabaceae	0,33	23,9	12	19	0,04	0,04	201,15	0,20	2,36	8,67
4630	90	2	Ciamis	200-500	Sengon	Paraserianthes falcataria (L.) I.C.	Leguminosae / Fabaceae	0,33	22,3	18	25	0,04	0,04	227,93	0,23	2,68	9,83
4631	90	2	Ciamis	200-500	Tisuk	Hibiscus macrophyllus Roxb. ex	Malvaceae	0,4	10,2	11	15	0,04	0,01	40,58	0,04	0,48	1,75
4632	90	2	Ciamis	200-500	Mahoni	Swietenia macrophylla King	Meliaceae	0,61	13,4	9	13	0,04	0,01	86,23	0,09	1,01	3,72
4633	90	2	Ciamis	200-500	Tisuk	Hibiscus macrophyllus Roxb. ex	Malvaceae	0,4	8,9	12	15	0,04	0,01	31,78	0,03	0,37	1,37
4634	90	2	Ciamis	200-500	Mahoni	Swietenia macrophylla King	Meliaceae	0,61	13,0	8	15	0,04	0,01	94,06	0,09	1,11	4,06
4635	90	2	Ciamis	200-500	Petai	Parkia speciosa Hassk.	Leguminosae / Fabaceae	0,45	13,7	11	15	0,04	0,01	77,67	0,08	0,91	3,35
4636	90	2	Ciamis	200-500	Mahoni	Swietenia macrophylla King	Meliaceae	0,61	12,7	8	12	0,04	0,01	73,28	0,07	0,86	3,16
4637	90	2	Ciamis	200-500	Mahoni	Swietenia macrophylla King	Meliaceae	0,61	4,1	1,5	4	0,04	0,00	3,42	0,00	0,04	0,15
4638	90	2	Ciamis	200-500	Pisitan	Dysoxylum parasiticum (Osbeck	Meliaceae	0,6	17,5	8	14	0,04	0,02	148,97	0,15	1,75	6,42
4639	90	2	Ciamis	200-500	Tisuk	Hibiscus macrophyllus Roxb. ex	Malvaceae	0,4	16,9	18	22	0,04	0,02	145,26	0,15	1,71	6,26
4640	90	2	Ciamis	200-500	Mahoni	Swietenia macrophylla King	Meliaceae	0,61	4,5	3	4	0,04	0,00	3,91	0,00	0,05	0,17
4641	90	2	Ciamis	200-500	Mahoni	Swietenia macrophylla King	Meliaceae	0,61	5,4	7	9	0,04	0,00	11,74	0,01	0,14	0,51
4642	90	2	Ciamis	200-500	Mahoni	Swietenia macrophylla King	Meliaceae	0,61	8,6	3	10	0,04	0,01	30,18	0,03	0,35	1,30
4643	90	2	Ciamis	200-500	Sengon	Paraserianthes falcataria (L.) I.C.	Leguminosae / Fabaceae	0,33	13,4	16	19	0,04	0,01	69,54	0,07	0,82	3,00
4644	90	2	Ciamis	200-500	Tisuk	Hibiscus macrophyllus Roxb. ex	Malvaceae	0,4	6,7	11	12	0,04	0,00	15,29	0,02	0,18	0,66
4645	90	2	Ciamis	200-500	Mahoni	Swietenia macrophylla King	Meliaceae	0,61	9,9	10	13	0,04	0,01	49,43	0,05	0,58	2,13
4646	90	2	Ciamis	200-500	Mahoni	Swietenia macrophylla King	Meliaceae	0,61	6,7	4	7	0,04	0,00	13,74	0,01	0,16	0,59
4647	90	2	Ciamis	200-500	Mahoni	Swietenia macrophylla King	Meliaceae	0,61	12,7	9	14	0,04	0,01	84,39	0,08	0,99	3,64
4648	90	2	Ciamis	200-500	Mahoni	Swietenia macrophylla King	Meliaceae	0,61	10,2	4	5	0,04	0,01	21,84	0,02	0,26	0,94
4649	90	2	Ciamis	200-500	Mahoni	Swietenia macrophylla King	Meliaceae	0,61	17,5	11	15	0,04	0,02	161,11	0,16	1,89	6,95
4650	90	2	Ciamis	200-500	Mahoni	Swietenia macrophylla King	Meliaceae	0,61	7,6	6	10	0,04	0,00	24,32	0,02	0,29	1,05
4651	90	2	Ciamis	200-500	Sengon	Paraserianthes falcataria (L.) I.C.	Leguminosae / Fabaceae	0,33	29,0	12	21	0,04	0,07	314,19	0,31	3,69	13,55
4652	90	2	Ciamis	200-500	Mahoni	Swietenia macrophylla King	Meliaceae	0,61	7,6	3	8	0,04	0,00	19,83	0,02	0,23	0,85
4653	90	2	Ciamis	200-500	Mahoni	Swietenia macrophylla King	Meliaceae	0,61	20,0	10	15	0,04	0,03	206,62	0,21	2,43	8,91
4654	90	2	Ciamis	200-500	Mahoni	Swietenia macrophylla King	Meliaceae	0,61	3,8	3	5	0,04	0,00	3,62	0,00	0,04	0,16
4655	90	2	Ciamis	200-500	Mahoni	Swietenia macrophylla King	Meliaceae	0,61	3,2	3	4	0,04	0,00	2,11	0,00	0,02	0,09
4656	90	2	Ciamis	200-500	Mahoni	Swietenia macrophylla King	Meliaceae	0,61	10,8	5	10	0,04	0,01	46,04	0,05	0,54	1,99
4657	90	2	Ciamis	200-500	Sengon	Paraserianthes falcataria (L.) I.C.	Leguminosae / Fabaceae	0,33	23,5	16	21	0,04	0,04	215,11	0,22	2,53	9,28
4658	90	2	Ciamis	200-500	Mahoni	Swietenia macrophylla King	Meliaceae	0,61	6,7	9	9,5	0,04	0,00	18,17	0,02	0,21	0,78
4659	90	2	Ciamis	200-500	Mahoni	Swietenia macrophylla King	Meliaceae	0,61	12,1	9	13	0,04	0,01	71,78	0,07	0,84	3,10
4660	90	2	Ciamis	200-500	Sengon	Paraserianthes falcataria (L.) I.C.	Leguminosae / Fabaceae	0,33	20,7	18	22	0,04	0,03	177,01	0,18	2,08	7,63
4661	90	2	Ciamis	200-500	Mahoni	Swietenia macrophylla King	Meliaceae	0,61	8,0	7	11	0,04	0,00	28,60	0,03	0,34	1,23
4662	90	2	Ciamis	200-500	Tisuk	Hibiscus macrophyllus Roxb. ex	Malvaceae	0,4	7,3	9	11	0,04	0,00	16,68	0,02	0,20	0,72
4663	90	2	Ciamis	200-500	Mahoni	Swietenia macrophylla King	Meliaceae	0,61	7,6	5	9	0,04	0,00	22,09	0,02	0,26	0,95
4664	90	2	Ciamis	200-500	Mahoni	Swietenia macrophylla King	Meliaceae	0,61	3,8	4	5	0,04	0,00	3,62	0,00	0,04	0,16
4665	90	2	Ciamis	200-500	Mahoni	Swietenia macrophylla King	Meliaceae	0,61	6,7	6	9	0,04	0,00	17,29	0,02	0,20	0,75
4666	90	2	Ciamis	200-500	Mahoni	Swietenia macrophylla King	Meliaceae	0,61	5,7	6	9	0,04	0,00	13,04	0,01	0,15	0,56
4667	90	2	Ciamis	200-500	Sengon	Paraserianthes falcataria (L.) I.C.	Leguminosae / Fabaceae	0,33	33,1	11	20	0,04	0,09	383,73	0,38	4,51	16,55
4668	90	2	Ciamis	200-500	Mahoni	Swietenia macrophylla King	Meliaceae	0,61	5,7	5	8	0,04	0,00	11,70	0,01	0,14	0,50
4669	90	2	Ciamis	200-500	Tisuk	Hibiscus macrophyllus Roxb. ex	Malvaceae	0,4	7,6	13	15	0,04	0,00	23,96	0,02	0,28	1,03
4670	90	2	Ciamis	200-500	Kokosan	Lansium domesticum var. aquae	Meliaceae	0,7	4,5	4	7	0,04	0,00	7,41	0,01	0,09	0,32
4671	90	2	Ciamis	200-500	Mahoni	Swietenia macrophylla King	Meliaceae	0,61	11,1	7	12	0,04	0,01	57,38	0,06	0,67	2,47
4672	90	2	Ciamis	200-500	Mahoni	Swietenia macrophylla King	Meliaceae	0,61	7,3	6	10	0,04	0,00	22,50	0,02	0,26	0,97
4673	90	2	Ciamis	200-500	Mahoni	Swietenia macrophylla King	Meliaceae	0,61	16,2	7	13	0,04	0,02	123,06	0,12	1,45	5,31
4674	90	2	Ciamis	200-500	Mahoni	Swietenia macrophylla King	Meliaceae	0,61	5,1	4	6	0,04	0,00	7,25	0,01	0,09	0,31
4675	90	2	Ciamis	200-500	Mahoni	Swietenia macrophylla King	Meliaceae	0,61	13,0	5	11	0,04	0,01	70,80	0,07	0,83	3,05
4676	90	2	Ciamis	200-500	Mahoni	Swietenia macrophylla King	Meliaceae	0,61	11,8	7	12	0,04	0,01	63,53	0,06	0,75	2,74
4677	90	2	Ciamis	200-500	Mahoni	Swietenia macrophylla King	Meliaceae	0,61	9,5	7	10	0,04	0,01	36,61	0,04	0,43	1,58
4678	90	2	Ciamis	200-500	Mahoni	Swietenia macrophylla King	Meliaceae	0,61	12,7	5	12	0,04	0,01	73,28	0,07	0,86	3,16
4679	90	2	Ciamis	200-500	Tisuk	Hibiscus macrophyllus Roxb. ex	Malvaceae	0,4	12,4	10	13	0,04	0,01	51,15	0,05	0,60	2,21
4680	90	2	Ciamis	200-500	Tisuk	Hibiscus macrophyllus Roxb. ex	Malvaceae	0,4	6,0	10	11,5	0,04	0,00	12,24	0,01	0,14	0,53
4681	90	2	Ciamis	200-500	Sengon	Paraserianthes falcataria (L.) I.C.	Leguminosae / Fabaceae	0,33	16,2	18	24	0,04	0,02	122,92	0,12	1,44	5,30
4682	90	2	Ciamis	200-500	Mahoni	Swietenia macrophylla King	Meliaceae	0,61	10,8	10	14	0,04	0,01	62,66	0,06	0,74	2,70
4683	90	2	Ciamis	200-500	Mahoni	Swietenia macrophylla King	Meliaceae	0,61	5,4	7	9	0,04	0,00	11,74	0,01	0,14	0,51
4684	90	2	Ciamis	200-500	Mahoni	Swietenia macrophylla King	Meliaceae	0,61	10,2	10	14	0,04	0,01	56,08	0,06	0,66	2,42
4685	90	2	Ciamis	200-500	Mahoni	Swietenia macrophylla King	Meliaceae	0,61	8,3	7	11	0,04	0,01	30,73	0,03	0,36	1,33
4686	90	2	Ciamis	200-500	Mahoni	Swietenia macrophylla King	Meliaceae	0,61	13,0	9	13	0,04	0,01	82,50	0,08	0,97	3,56
4687	90	2	Ciamis	200-500	Mahoni	Swietenia macrophylla King	Meliaceae	0,61	8,6	6	12	0,04	0,01	35,67	0,04	0,42	1,54
4688	90	2	Ciamis	200-500	Sengon	Paraserianthes falcataria (L.) I.C.	Leguminosae / Fabaceae	0,33	28,3	15	20	0,04	0,06	288,47	0,29	3,39	12,44
4689	90	2	Ciamis	200-500	Sengon	Paraserianthes falcataria (L.) I.C.	Leguminosae / Fabaceae	0,33	10,5	9	13	0,04	0,01	31,58	0,03	0,37	1,36
4690	90	2	Ciamis	200-500	Sengon	Paraserianthes falcataria (L.) I.C.	Leguminosae / Fabaceae	0,33	16,2	12	15	0,04	0,02	79,92	0,08	0,94	3,45
4691	90	2	Ciamis	200-500	Mahoni	Swietenia macrophylla King	Meliaceae	0,61	5,7	8	10	0,04	0,00	14,36	0,01	0,17	0,62
4692	90	2	Ciamis	200-500	Mahoni	Swietenia macrophylla King	Meliaceae	0,61	3,8	3	4,5	0,04	0,00	3,29	0,00	0,04	0,14
4693	90	2	Ciamis	200-500	Mahoni	Swietenia macrophylla King	Meliaceae	0,61	5,4	2,5	5	0,04	0,00	6,85	0,01	0,08	0,30
4694	90	2	Ciamis	200-500	Mahoni	Swietenia macrophylla King	Meliaceae	0,61	4,5	3,5	4,5	0,04	0,00	4,36	0,00	0,05	0,19
4695	90	2	Ciamis	200-500	Mahoni	Swietenia macrophylla King	Meliaceae	0,61	5,4	2	6	0,04	0,00	8,10	0,01	0,10	0,35
4696	90	2	Ciamis	200-500	Mahoni	Swietenia macrophylla King	Meliaceae	0,61	3,8	2	3	0,04	0,00	2,27	0,00	0,03	0,10
4697	90	2	Ciamis	200-500	Mahoni	Swietenia macrophylla King	Meliaceae	0,61	7,0	4	8	0,04	0,00	16,91	0,02	0,20	0,73
4698	90	2	Ciamis	200-500	Tisuk	Hibiscus macrophyllus Roxb. ex	Malvaceae	0,4	15,9	12	16	0,04	0,02	97,52	0,10	1,15	4,21
4699	90	2	Ciamis	200-500	Mahoni	Swietenia macrophylla King	Meliaceae	0,61	5,4	4	6	0,04	0,00	8,10	0,01	0,10	0,35
4700	90	2	Ciamis	200-500	Mahoni	Swietenia macrophylla King	Meliaceae	0,61	7,3	6	8	0,04	0,00	18,34	0,02	0,22	0,79
4701	90	2	Ciamis	200-500	Mahoni	Swietenia macrophylla King	Meliaceae	0,61	14,3	8	12	0,04	0,02	90,93	0,09	1,07	3,92
4702	90	2	Ciamis	200-500	Tisuk	Hibiscus macrophyllus Roxb. ex	Malvaceae	0,4	10,8	8	11	0,04	0,01	34,13	0,03	0,40	1,47
4703	90	2	Ciamis	200-500	Mahoni	Swietenia macrophylla King	Meliaceae	0,61	8,0	5	7	0,04	0,00	18,91	0,02	0,22	0,82
4704	90	2	Ciamis	200-500	Mahoni	Swietenia macrophylla King	Meliaceae	0,61	3,2	1,5	4,5	0,04	0,00	2,35	0,00	0,03	0,10
4705	90	2	Ciamis	200-500	Mahoni	Swietenia macrophylla King	Meliaceae	0,61	6,4	6	8	0,04	0,00	14,20	0,01	0,17	0,61
4706	90	2	Ciamis	200-500	Kelapa	Cocos nucifera L.	Arecaceae	0,5	25,5	11	4	0,04	0,05	35,30	0,04	0,41	1,52

4707	90	2	Ciamis	200-500	Melinjo	Gnetum gnemon L.	Gnetaceae	0,76	16,9	4	11	0,04	0,02	138,59	0,14	1,63	5,98
4708	90	2	Ciamis	200-500	Mahoni	Swietenia macrophylla King	Meliaceae	0,61	8,9	7	10	0,04	0,01	32,26	0,03	0,38	1,39
4709	90	2	Ciamis	200-500	Tisuk	Hibiscus macrophyllus Roxb. ex l	Malvaceae	0,4	5,7	8	9	0,04	0,00	8,86	0,01	0,10	0,38
4710	90	2	Ciamis	200-500	Mahoni	Swietenia macrophylla King	Meliaceae	0,61	19,7	7	14	0,04	0,03	188,36	0,19	2,21	8,12
4711	90	2	Ciamis	200-500	Sulasan	Sp5	0	0,5	8,3	7	9	0,04	0,01	21,32	0,02	0,25	0,92
4712	90	2	Ciamis	200-500	Mahoni	Swietenia macrophylla King	Meliaceae	0,61	11,1	7	11	0,04	0,01	52,98	0,05	0,62	2,28
4713	90	2	Ciamis	200-500	Sengon	Paraserianthes falcataria (L.) I.C	Leguminosae / Fabaceae	0,33	8,6	12	16	0,04	0,01	26,44	0,03	0,31	1,14
4714	90	2	Ciamis	200-500	Dukuh	Lansium domesticum Corrêa	Meliaceae	0,85	10,8	4	10	0,04	0,01	62,39	0,06	0,73	2,69
4715	90	2	Ciamis	200-500	Durian	Durio zibethinus L.	Malvaceae	0,57	4,5	5	6	0,04	0,00	5,33	0,01	0,06	0,23
4716	90	2	Ciamis	200-500	Sengon	Paraserianthes falcataria (L.) I.C	Leguminosae / Fabaceae	0,33	32,8	14	22	0,04	0,08	411,39	0,41	4,83	17,74
4717	90	2	Ciamis	200-500	Tisuk	Hibiscus macrophyllus Roxb. ex l	Malvaceae	0,4	7,6	7	9	0,04	0,00	15,01	0,02	0,18	0,65
4718	90	2	Ciamis	200-500	Mahoni	Swietenia macrophylla King	Meliaceae	0,61	5,1	5	7	0,04	0,00	8,35	0,01	0,10	0,36
4719	90	2	Ciamis	200-500	Mahoni	Swietenia macrophylla King	Meliaceae	0,61	10,5	6	10	0,04	0,01	43,59	0,04	0,51	1,88
4720	90	2	Ciamis	200-500	Kopi	Coffea canephora Pierre ex A.Fro	Rubiaceae	0,41	4,8	3	5	0,04	0,00	6,92	0,01	0,08	0,30
4721	90	2	Ciamis	200-500	Tisuk	Hibiscus macrophyllus Roxb. ex l	Malvaceae	0,4	8,0	11	12	0,04	0,00	21,05	0,02	0,25	0,91
4722	90	2	Ciamis	200-500	Mahoni	Swietenia macrophylla King	Meliaceae	0,61	8,3	4	7	0,04	0,01	20,32	0,02	0,24	0,88
4723	90	2	Ciamis	200-500	Mahoni	Swietenia macrophylla King	Meliaceae	0,61	12,4	5	9	0,04	0,01	53,75	0,05	0,63	2,32
4724	90	2	Ciamis	200-500	Kokosan	Lansium domesticum var. aquae	Meliaceae	0,7	3,2	3	6	0,04	0,00	3,48	0,00	0,04	0,15
4725	90	2	Ciamis	200-500	Mahoni	Swietenia macrophylla King	Meliaceae	0,61	15,0	4	11	0,04	0,02	90,92	0,09	1,07	3,92
4726	90	2	Ciamis	200-500	Mahoni	Swietenia macrophylla King	Meliaceae	0,61	12,7	6	10	0,04	0,01	62,01	0,06	0,73	2,67
4727	90	2	Ciamis	200-500	Petai	Parkia speciosa Hassk.	Leguminosae / Fabaceae	0,45	6,0	8	9	0,04	0,00	10,89	0,01	0,13	0,47
4728	90	2	Ciamis	200-500	Mahoni	Swietenia macrophylla King	Meliaceae	0,61	19,4	9	12	0,04	0,03	158,76	0,16	1,87	6,85
4729	90	2	Ciamis	200-500	Tisuk	Hibiscus macrophyllus Roxb. ex l	Malvaceae	0,4	15,3	12	15	0,04	0,02	85,30	0,09	1,00	3,68
4730	90	2	Ciamis	200-500	Tisuk	Hibiscus macrophyllus Roxb. ex l	Malvaceae	0,4	5,4	9	11	0,04	0,00	9,59	0,01	0,11	0,41
4731	90	2	Ciamis	200-500	Mahoni	Swietenia macrophylla King	Meliaceae	0,61	4,8	3,5	5	0,04	0,00	5,45	0,01	0,06	0,23
4732	90	2	Ciamis	200-500	Mahoni	Swietenia macrophylla King	Meliaceae	0,61	7,6	7	8,5	0,04	0,00	20,96	0,02	0,25	0,90
4733	90	2	Ciamis	200-500	Tisuk	Hibiscus macrophyllus Roxb. ex l	Malvaceae	0,4	10,8	12	15	0,04	0,01	45,35	0,05	0,53	1,96
4734	90	2	Ciamis	200-500	Mahoni	Swietenia macrophylla King	Meliaceae	0,61	8,3	6	8	0,04	0,01	22,96	0,02	0,27	0,99
4735	90	2	Ciamis	200-500	Sengon	Paraserianthes falcataria (L.) I.C	Leguminosae / Fabaceae	0,33	7,6	9	11	0,04	0,00	15,12	0,02	0,18	0,65
4736	90	2	Ciamis	200-500	Mahoni	Swietenia macrophylla King	Meliaceae	0,61	14,0	7	10	0,04	0,02	73,84	0,07	0,87	3,18
4737	90	2	Ciamis	200-500	Sengon	Paraserianthes falcataria (L.) I.C	Leguminosae / Fabaceae	0,33	22,0	12	18	0,04	0,04	164,32	0,16	1,93	7,09
4738	90	2	Ciamis	200-500	Mahoni	Swietenia macrophylla King	Meliaceae	0,61	11,8	3,5	10	0,04	0,01	53,76	0,05	0,63	2,32
4739	90	2	Ciamis	200-500	Tisuk	Hibiscus macrophyllus Roxb. ex l	Malvaceae	0,4	4,5	5	6,5	0,04	0,00	4,15	0,00	0,05	0,18
4740	90	2	Ciamis	200-500	Tisuk	Hibiscus macrophyllus Roxb. ex l	Malvaceae	0,4	9,2	10	12	0,04	0,01	27,62	0,03	0,32	1,19
4741	90	2	Ciamis	200-500	Sengon	Paraserianthes falcataria (L.) I.C	Leguminosae / Fabaceae	0,33	23,5	18	22	0,04	0,04	224,48	0,22	2,64	9,68
4742	90	2	Ciamis	200-500	Mahoni	Swietenia macrophylla King	Meliaceae	0,61	6,4	6	7,5	0,04	0,00	13,38	0,01	0,16	0,58
4743	90	2	Ciamis	200-500	Tisuk	Hibiscus macrophyllus Roxb. ex l	Malvaceae	0,4	8,3	10	12	0,04	0,01	22,61	0,02	0,27	0,98
4744	90	2	Ciamis	200-500	Mahoni	Swietenia macrophylla King	Meliaceae	0,61	8,9	3,5	7,5	0,04	0,01	24,79	0,02	0,29	1,07
4745	90	2	Ciamis	200-500	Mahoni	Swietenia macrophylla King	Meliaceae	0,61	5,1	4	6	0,04	0,00	7,25	0,01	0,09	0,31
4746	90	2	Ciamis	200-500	Mahoni	Swietenia macrophylla King	Meliaceae	0,61	5,4	5	7	0,04	0,00	9,33	0,01	0,11	0,40
4747	90	2	Ciamis	200-500	Mahoni	Swietenia macrophylla King	Meliaceae	0,61	4,5	2	3,5	0,04	0,00	3,46	0,00	0,04	0,15
4748	90	2	Ciamis	200-500	Dukuh	Lansium domesticum Corrêa	Meliaceae	0,85	47,7	3	13	0,04	0,18	1.203,48	1,20	14,14	51,90
4749	90	2	Ciamis	200-500	mahoni	Swietenia macrophylla King	Meliaceae	0,61	5,4	3	4	0,04	0,00	5,59	0,01	0,07	0,24
4750	90	2	Ciamis	200-500	mahoni	Swietenia macrophylla King	Meliaceae	0,61	4,1	3	5	0,04	0,00	4,19	0,00	0,05	0,18
4751	90	2	Ciamis	200-500	mahoni	Swietenia macrophylla King	Meliaceae	0,61	3,8	3	5	0,04	0,00	3,62	0,00	0,04	0,16
4752	91	3	Ciamis	200-500	Mahoni	Swietenia macrophylla King	Meliaceae	0,61	3,8	1,5	2	0,04	0,00	1,56	0,00	0,02	0,07
4753	91	3	Ciamis	200-500	Mahoni	Swietenia macrophylla King	Meliaceae	0,61	20,0	9	13	0,04	0,03	181,24	0,18	2,13	7,82
4754	91	3	Ciamis	200-500	Mahoni	Swietenia macrophylla King	Meliaceae	0,61	16,9	5	14	0,04	0,02	141,32	0,14	1,66	6,09
4755	91	3	Ciamis	200-500	Mahoni	Swietenia macrophylla King	Meliaceae	0,61	12,7	7	10	0,04	0,01	62,01	0,06	0,73	2,67
4756	91	3	Ciamis	200-500	Mahoni	Swietenia macrophylla King	Meliaceae	0,61	13,7	5	12	0,04	0,01	83,66	0,08	0,98	3,61
4757	91	3	Ciamis	200-500	Mahoni	Swietenia macrophylla King	Meliaceae	0,61	15,3	5	12	0,04	0,02	102,34	0,10	1,20	4,41
4758	91	3	Ciamis	200-500	Mahoni	Swietenia macrophylla King	Meliaceae	0,61	15,9	5	12	0,04	0,02	110,29	0,11	1,30	4,76
4759	91	3	Ciamis	200-500	Mahoni	Swietenia macrophylla King	Meliaceae	0,61	12,1	3	9	0,04	0,01	51,25	0,05	0,60	2,21
4760	91	3	Ciamis	200-500	Mahoni	Swietenia macrophylla King	Meliaceae	0,61	12,1	7	13	0,04	0,01	71,78	0,07	0,84	3,10
4761	91	3	Ciamis	200-500	Bungbulang	Premna tomentosa Willd.	Lamiaceae / Verbenaceae	0,96	22,3	3	12	0,04	0,04	309,48	0,31	3,64	13,35
4762	91	3	Ciamis	200-500	Caruy	Pterospermum javanicum Jungh.	Malvaceae	0,53	23,5	9	13	0,04	0,04	213,97	0,21	2,51	9,23
4763	91	3	Ciamis	200-500	Caruy	Pterospermum javanicum Jungh.	Malvaceae	0,53	19,4	11	17	0,04	0,03	192,03	0,19	2,26	8,28
4764	91	3	Ciamis	200-500	Caruy	Pterospermum javanicum Jungh.	Malvaceae	0,53	20,0	11	15	0,04	0,03	181,66	0,18	2,13	7,83
4765	91	3	Ciamis	200-500	Caruy	Pterospermum javanicum Jungh.	Malvaceae	0,53	19,1	5	12	0,04	0,03	135,41	0,14	1,59	5,84
4766	91	3	Ciamis	200-500	Mahoni	Swietenia macrophylla King	Meliaceae	0,61	22,0	5	14	0,04	0,04	229,14	0,23	2,69	9,88
4767	91	3	Ciamis	200-500	Mahoni	Swietenia macrophylla King	Meliaceae	0,61	12,4	4	11	0,04	0,01	64,60	0,06	0,76	2,79
4768	91	3	Ciamis	200-500	Mahoni	Swietenia macrophylla King	Meliaceae	0,61	17,5	3	13	0,04	0,02	141,32	0,14	1,66	6,09
4769	91	3	Ciamis	200-500	Tisuk	Hibiscus macrophyllus Roxb. ex l	Malvaceae	0,4	6,0	3	5,5	0,04	0,00	6,23	0,01	0,07	0,27
4770	91	3	Ciamis	200-500	Tisuk	Hibiscus macrophyllus Roxb. ex l	Malvaceae	0,4	12,7	10	14	0,04	0,01	57,34	0,06	0,67	2,47
4771	91	3	Ciamis	200-500	Mahoni	Swietenia macrophylla King	Meliaceae	0,61	6,0	4	5,5	0,04	0,00	9,17	0,01	0,11	0,40
4772	91	3	Ciamis	200-500	Tisuk	Hibiscus macrophyllus Roxb. ex l	Malvaceae	0,4	24,8	7	10	0,04	0,05	143,19	0,14	1,68	6,17
4773	91	3	Ciamis	200-500	Mahoni	Swietenia macrophylla King	Meliaceae	0,61	3,5	1,7	3	0,04	0,00	1,93	0,00	0,02	0,08
4774	91	3	Ciamis	200-500	Bungbulang	Premna tomentosa Willd.	Lamiaceae / Verbenaceae	0,96	3,2	1,7	3,5	0,04	0,00	2,83	0,00	0,03	0,12
4775	91	3	Ciamis	200-500	Rambutan	Nephelium lappaceum L.	Sapindaceae	0,71	3,5	1,7	3,5	0,04	0,00	2,56	0,00	0,03	0,11
4776	91	3	Ciamis	200-500	Mahoni	Swietenia macrophylla King	Meliaceae	0,61	8,9	3	7	0,04	0,01	23,27	0,02	0,27	1,00
4777	91	3	Ciamis	200-500	Mahoni	Swietenia macrophylla King	Meliaceae	0,61	18,1	6	12	0,04	0,03	140,21	0,14	1,65	6,05
4778	92	1	Ciamis	200-500	Mahoni	Swietenia macrophylla King	Meliaceae	0,61	17,5	3	4	0,04	0,02	48,01	0,05	0,56	2,07
4779	92	1	Ciamis	200-500	Mahoni	Swietenia macrophylla King	Meliaceae	0,61	4,5	2,5	4,5	0,04	0,00	4,36	0,00	0,05	0,19
4780	92	1	Ciamis	200-500	Mahoni	Swietenia macrophylla King	Meliaceae	0,61	20,0	9	12	0,04	0,03	168,42	0,17	1,98	7,26
4781	92	1	Ciamis	200-500	Mahoni	Swietenia macrophylla King	Meliaceae	0,61	20,0	4	9	0,04	0,03	129,41	0,13	1,52	5,58
4782	92	1	Ciamis	200-500	Mahoni	Swietenia macrophylla King	Meliaceae	0,61	20,4	4	8	0,04	0,03	119,57	0,12	1,40	5,16
4783	92	1	Ciamis	200-500	Mahoni	Swietenia macrophylla King	Meliaceae	0,61	13,0	4	8	0,04	0,01	52,88	0,05	0,62	2,28
4784	92	1	Ciamis	200-500	Mahoni	Swietenia macrophylla King	Meliaceae	0,61	18,8	3	10	0,04	0,03	126,38	0,13	1,48	5,45
4785	92	1	Ciamis	200-500	Mahoni	Swietenia macrophylla King	Meliaceae	0,61	24,5	4,5	9,5	0,04	0,05	196,39	0,20	2,31	8,47
4786	92	1	Ciamis	200-500	Mahoni	Swietenia macrophylla King	Meliaceae	0,61	15,0	4,5	8,5	0,04	0,02	71,80	0,07	0,84	3,10
4787	92	1	Ciamis	200-500	Mahoni	Swietenia macrophylla King	Meliaceae	0,61	17,2	4	9	0,04	0,02	97,57	0,10	1,15	4,21
4788	92	1	Ciamis	200-500	Mahoni	Swietenia macrophylla King	Meliaceae	0,61	18,8	4	11	0,04	0,03	137,91	0,14	1,62	5,95
4789	92	1	Ciamis	200-500	Mahoni	Swietenia macrophylla King	Meliaceae	0,61	13,4	5	9	0,04	0,01	61,57	0,06	0,72	2,65
4790	92	1	Ciamis	200-500	Mahoni	Swietenia macrophylla King	Meliaceae	0,61	14,0	5	10	0,04	0,02	73,84	0,07	0,87	3,18
4791	92	1	Ciamis	200-500	Mahoni	Swietenia macrophylla King	Meliaceae	0,61	18,1	8	10	0,04	0,03	118,64	0,12	1,39	5,12
4792	92	1	Ciamis	200-500	Gamal/Kalkiria	Gliricidia sepium (Jacq.) Walp.	Leguminosae / Fabaceae	0,58	10,8	1,3	4,3	0,04	0,01	20,29	0,02	0,24	0,88
4793	92	1	Ciamis	200-500	Mahoni	Swietenia macrophylla King	Meliaceae	0,61	21,3	7	12	0,04	0,04	188,53	0,19	2,22	8,13
4794	92	1	Ciamis	200-500	Pongporang	Oroxylum indicum (L.) Kurz	Bignoniaceae	0,32	7,3	6	7,5	0,04	0,00	9,57	0,01	0,11	0,41
4795	92	1	Ciamis	200-500	Mahoni	Swietenia macrophylla King	Meliaceae	0,61	14,3	5	9	0,04	0,02	69,86	0,07	0,82	3,01
4796	92	1	Ciamis	200-500	Mahoni	Swietenia macrophylla King	Meliaceae	0,61	15,0	5	12	0,04	0,02	98,47	0,10	1,16	4,25
4797	92	1	Ciamis	200-500	Mahoni	Swietenia macrophylla King	Meliaceae	0,61	3,5	3	4	0,04	0,00	2,52	0,00	0,03	0,11
4798	92	1	Ciamis	200-500	Mahoni	Swietenia macrophylla King	Meliaceae	0,61	10,5	4	10	0,04	0,01	43,59	0,04	0,51	1,88
4799	92	1	Ciamis	200-500	Mahoni	Swietenia macrophylla King	Meliaceae	0,61	15,0	5	8	0,04	0,02	67,92	0,07	0,80	2,93
4800	92	1	Ciamis	200-500	Mahoni	Swietenia macrophylla King	Meliaceae	0,61	12,1	4	8	0,04	0,01	46,01	0,05	0,54	1,98
4801	92	1	Ciamis	200-500	Renghas	Gluta renghas L.	Anacardiaceae	0,69	9,5	7	11	0,04	0,01	44,72	0,04	0,53	1,93
4802	92	1	Ciamis	200-500	Mahoni	Swietenia macrophylla King	Meliaceae	0,61	3,8	1,6	2,5	0,04	0,00	1,92	0,00	0,02	0,08
4803	92	1	Ciamis	200-500	Mahoni	Swietenia macrophylla King	Meliaceae	0,61	16,9	7	12	0,04	0,02	122,71	0,12	1,44	5,29
4804	92	1	Ciamis	200-500	Mahoni	Swietenia macrophylla King	Meliaceae	0,61	9,9	3	9	0,04	0,01	35,30	0,04	0,41	1,52
4805	92	1	Ciamis	200-500	Gamal/Kalkiria	Gliricidia sepium (Jacq.) Walp.	Leguminosae / Fabaceae	0,58	4,5	2,5	6	0,04	0,00	5,42	0,01	0,06	0,23
4806	92	1	Ciamis	200-500	Gamal/Kalkiria	Gliricidia sepium (Jacq.) Walp.	Leguminosae / Fabaceae	0,58	4,5	3	6	0,04	0,00	5,42	0,01	0,06	0,23
4807	92	1	Ciamis	200-500	Mahoni	Swietenia macrophylla King	Meliaceae	0,61	5,7	2,5	6,5	0,04	0,00	9,68	0,01	0,11	0,42
4808	92	1	Ciamis	200-500	Mahoni	Swietenia macrophylla King	Meliaceae	0,61	6,4	3	9	0,04	0,00	15,81	0,02	0,19	0,68
4809	93	2	Ciamis	200-500	Tisuk	Hibiscus macrophyllus Roxb. ex l	Malvaceae	0,4	16,2	6	12	0,04	0,02	77,70	0,08	0,91	3,35
4810	93	2	Ciamis	200-500	Sengon	Paraserianthes falcataria (L.) I.C	Leguminosae / Fabaceae	0,33	15,0	6	11	0,04	0,02	51,79	0,05	0,61	2,23
4811	93	2	Ciamis	200-500	Nangka	Artocarpus heterophyllus Lam.	Moraceae	0,61	5,7	1,5	4,5	0,04	0,00	6,91	0,01	0,08	0,30
4812	93	2	Ciamis	200-500	Sengon	Paraserianthes falcataria (L.) I.C	Leguminosae / Fabaceae	0,33	9,5	10	13	0,04	0,01	26,52	0,03	0,31	1,14
4813	93	2	Ciamis	200-500	Sengon	Paraserianthes falcataria (L.) I.C	Leguminosae / Fabaceae	0,33	8,9	9	11	0,04	0,01	20,05	0,02	0,24	0,86
4814	93	2	Ciamis	200-500	Sengon	Paraserianthes falcataria (L.) I.C	Leguminosae / Fabaceae	0,33	7,0	7	9	0,04	0,00	10,73	0,01	0,13	0,46
4815	93	2	Ciamis	200-500	Sengon	Paraserianthes falcataria (L.) I.C	Leguminosae / Fabaceae	0,33	11,5	9	9	0,04	0,01	26,44	0,03	0,31	1,14
4816	93	2	Ciamis	200-500	Bintinu	Melochia umbellata (Houtt.) Stap	Malvaceae	0,25	3,8	4	10	0,04	0,00	3,02	0,00	0,04	0,13
4817	93	2	Ciamis	200-500	Bintinu	Melochia umbellata (Houtt.) Stap	Malvaceae	0,25	3,5	4	5	0,04	0,00	1,36	0,00	0,02	0,06
4818	93	2	Ciamis	200-500	Bintinu	Melochia umbellata (Houtt.) Stap	Malvaceae	0,25	3,8	3	6	0,04	0,00	1,89	0,00	0,02	0,08
4819	93	2	Ciamis	200-500	Johar	Cassia siamea Lam.	Leguminosae / Fabaceae	0,84	8,3	3	8	0,04	0,01	30,78	0,03	0,36	1,33
4820	93	2	Ciamis	200-500	Sengon	Paraserianthes falcataria (L.) I.C	Leguminosae / Fabaceae	0,33	5,4	4	6	0,04	0,00	4,61	0,00	0,05	0,20
4821	93	2	Ciamis	200-500	Sengon	Paraserianthes falcataria (L.) I.C	Leguminosae / Fabaceae	0,33	29,3	12	19	0,04	0,07	292,47	0,29	3,44	12,61
4822	93	2	Ciamis	200-500	Mahoni	Swietenia macrophylla King	Meliaceae	0,61	6,0	3	8	0,04	0,00	12,92	0,01	0,15	0,56
4823	93	2	Ciamis	200-500	Tisuk	Hibiscus macrophyllus Roxb. ex l	Malvaceae	0,4	7,0	2,5	6,5	0,04	0,00	9,50	0,01	0,11	0,41
4824	93	2	Ciamis	200-500	Sengon	Paraserianthes falcataria (L.) I.C	Leguminosae / Fabaceae	0,33	9,5	9	11	0,04	0,01	22,76	0,02	0,27	0,98
4825	93	2	Ciamis	200-500	Sengon	Paraserianthes falcataria (L.) I.C	Leguminosae / Fabaceae	0,33	20,7	12	15	0,04	0,03	124,63	0,12	1,46	5,37
4826	93	2	Ciamis	200-500	Sengon	Paraserianthes falcataria (L.) I.C	Leguminosae / Fabaceae	0,33	13,0	10	14	0,04	0,01	50,30	0,05	0,59	2,17
4827	93	2	Ciamis	200-500	Sengon	Paraserianthes falcataria (L.) I.C	Leguminosae / Fabaceae	0,33	9,5	8	13	0,04	0,01	26,52	0,03	0,31	1,14
4828	93	2	Ciamis	200-500	Tisuk	Hibiscus macrophyllus Roxb. ex l	Malvaceae	0,4	7,6	3	8	0,04	0,00	13,47	0,01	0,16	0,58
4829	93	2	Ciamis	200-500	Sengon	Paraserianthes falcataria (L.) I.C	Leguminosae / Fabaceae	0,33	7,6	6	10	0,04	0,00	13,86	0,01	0,16	0,60
4830	93	2	Ciamis	200-500	Bintinu	Melochia umbellata (Houtt.) Stap	Malvaceae	0,25	5,4	5	4	0,04	0,00	2,47	0,00	0,03	0,11

4831	93	2	Ciamis	200-500	Bintinu	Melochia umbellata (Houtt.) Sta	Malvaceae	0,25	4,1	5	7	0,04	0,00	2,52	0,00	0,03	0,11
4832	93	2	Ciamis	200-500	Bintinu	Melochia umbellata (Houtt.) Sta	Malvaceae	0,25	5,4	5	7	0,04	0,00	4,12	0,00	0,05	0,18
4833	93	2	Ciamis	200-500	Sengon	Paraserianthes falcataria (L.) I.C.	Leguminosae / Fabaceae	0,33	6,0	7	8	0,04	0,00	7,36	0,01	0,09	0,32
4834	93	2	Ciamis	200-500	Sengon	Paraserianthes falcataria (L.) I.C.	Leguminosae / Fabaceae	0,33	11,5	10	3	0,04	0,01	9,67	0,01	0,11	0,42
4835	93	2	Ciamis	200-500	Sengon	Paraserianthes falcataria (L.) I.C.	Leguminosae / Fabaceae	0,33	6,0	6	7,5	0,04	0,00	6,94	0,01	0,08	0,30
4836	93	2	Ciamis	200-500	Johar	Cassia siamea Lam.	Leguminosae / Fabaceae	0,84	5,1	3	6	0,04	0,00	9,72	0,01	0,11	0,42
4837	93	2	Ciamis	200-500	Sengon	Paraserianthes falcataria (L.) I.C.	Leguminosae / Fabaceae	0,33	13,4	9	13	0,04	0,01	49,12	0,05	0,58	2,12
4838	93	2	Ciamis	200-500	Sengon	Paraserianthes falcataria (L.) I.C.	Leguminosae / Fabaceae	0,33	7,3	5	7	0,04	0,00	9,24	0,01	0,11	0,40
4839	93	2	Ciamis	200-500	Tisuk	Hibiscus macrophyllus Roxb. ex	Malvaceae	0,4	15,6	6	10	0,04	0,02	61,10	0,06	0,72	2,63
4840	93	2	Ciamis	200-500	Sengon	Paraserianthes falcataria (L.) I.C.	Leguminosae / Fabaceae	0,33	4,8	6	7	0,04	0,00	4,22	0,00	0,05	0,18
4841	93	2	Ciamis	200-500	Sengon	Paraserianthes falcataria (L.) I.C.	Leguminosae / Fabaceae	0,33	31,8	10	17	0,04	0,08	307,73	0,31	3,62	13,27
4842	93	2	Ciamis	200-500	Sengon	Paraserianthes falcataria (L.) I.C.	Leguminosae / Fabaceae	0,33	11,1	11	14	0,04	0,01	37,64	0,04	0,44	1,62
4843	93	2	Ciamis	200-500	Tisuk	Hibiscus macrophyllus Roxb. ex	Malvaceae	0,4	4,1	4	6	0,04	0,00	3,37	0,00	0,04	0,15
4844	93	2	Ciamis	200-500	Sengon	Paraserianthes falcataria (L.) I.C.	Leguminosae / Fabaceae	0,33	6,0	6	10	0,04	0,00	9,03	0,01	0,11	0,39
4845	93	2	Ciamis	200-500	Sengon	Paraserianthes falcataria (L.) I.C.	Leguminosae / Fabaceae	0,33	6,4	5	9	0,04	0,00	9,01	0,01	0,11	0,39
4846	93	2	Ciamis	200-500	Johar	Cassia siamea Lam.	Leguminosae / Fabaceae	0,84	5,1	4	6	0,04	0,00	9,72	0,01	0,11	0,42
4847	93	2	Ciamis	200-500	Sengon	Paraserianthes falcataria (L.) I.C.	Leguminosae / Fabaceae	0,33	9,5	12	15	0,04	0,01	30,23	0,03	0,36	1,30
4848	93	2	Ciamis	200-500	Johar	Cassia siamea Lam.	Leguminosae / Fabaceae	0,84	5,1	3	5	0,04	0,00	8,22	0,01	0,10	0,35
4849	93	2	Ciamis	200-500	Sengon	Paraserianthes falcataria (L.) I.C.	Leguminosae / Fabaceae	0,33	10,2	12	15	0,04	0,01	34,03	0,03	0,40	1,47
4850	93	2	Ciamis	200-500	Sengon	Paraserianthes falcataria (L.) I.C.	Leguminosae / Fabaceae	0,33	19,4	12	18	0,04	0,03	131,11	0,13	1,54	5,65
4851	93	2	Ciamis	200-500	Nangka	Artocarpus heterophyllus Lam.	Moraceae	0,61	18,8	3	8	0,04	0,03	103,02	0,10	1,21	4,44
4852	93	2	Ciamis	200-500	Nangka	Artocarpus heterophyllus Lam.	Moraceae	0,61	9,2	3	6	0,04	0,01	21,55	0,02	0,25	0,93
4853	93	2	Ciamis	200-500	Nangka	Artocarpus heterophyllus Lam.	Moraceae	0,61	16,9	4	10	0,04	0,02	103,84	0,10	1,22	4,48
4854	93	2	Ciamis	200-500	Nangka	Artocarpus heterophyllus Lam.	Moraceae	0,61	15,6	3	8	0,04	0,02	73,31	0,07	0,86	3,16
4855	93	2	Ciamis	200-500	Sengon	Paraserianthes falcataria (L.) I.C.	Leguminosae / Fabaceae	0,33	31,2	12	19	0,04	0,08	328,35	0,33	3,86	14,16
4856	93	2	Ciamis	200-500	Sengon	Paraserianthes falcataria (L.) I.C.	Leguminosae / Fabaceae	0,33	37,2	12	19	0,04	0,11	454,29	0,45	5,34	19,59
4857	93	2	Ciamis	200-500	Bintinu	Melochia umbellata (Houtt.) Sta	Malvaceae	0,25	7,6	6	9	0,04	0,00	9,76	0,01	0,11	0,42
4858	93	2	Ciamis	200-500	Mahoni	Swietenia macrophylla King	Meliaceae	0,61	6,4	3	6	0,04	0,00	10,91	0,01	0,13	0,47
4859	93	2	Ciamis	200-500	Tisuk	Hibiscus macrophyllus Roxb. ex	Malvaceae	0,4	19,1	7	14	0,04	0,03	120,51	0,12	1,42	5,20
4860	93	2	Ciamis	200-500	Tisuk	Hibiscus macrophyllus Roxb. ex	Malvaceae	0,4	10,2	5	9	0,04	0,01	25,42	0,03	0,30	1,10
4861	93	2	Ciamis	200-500	Johar	Cassia siamea Lam.	Leguminosae / Fabaceae	0,84	7,0	2,5	4	0,04	0,00	12,01	0,01	0,14	0,52
4862	93	2	Ciamis	200-500	Sengon	Paraserianthes falcataria (L.) I.C.	Leguminosae / Fabaceae	0,33	10,2	6	9	0,04	0,01	21,31	0,02	0,25	0,92
4863	93	2	Ciamis	200-500	Bintinu	Melochia umbellata (Houtt.) Sta	Malvaceae	0,25	6,4	3	6	0,04	0,00	4,82	0,00	0,06	0,21
4864	93	2	Ciamis	200-500	Sengon	Paraserianthes falcataria (L.) I.C.	Leguminosae / Fabaceae	0,33	6,4	2	8	0,04	0,00	8,09	0,01	0,10	0,35
4865	93	2	Ciamis	200-500	Sengon	Paraserianthes falcataria (L.) I.C.	Leguminosae / Fabaceae	0,33	6,7	6	10	0,04	0,00	10,85	0,01	0,13	0,47
4866	93	2	Ciamis	200-500	Bintinu	Melochia umbellata (Houtt.) Sta	Malvaceae	0,25	4,8	3,5	5	0,04	0,00	2,41	0,00	0,03	0,10
4867	93	2	Ciamis	200-500	Bintinu	Melochia umbellata (Houtt.) Sta	Malvaceae	0,25	6,4	4	6	0,04	0,00	4,82	0,00	0,06	0,21
4868	93	2	Ciamis	200-500	Sengon	Paraserianthes falcataria (L.) I.C.	Leguminosae / Fabaceae	0,33	27,0	9	16	0,04	0,06	216,15	0,22	2,54	9,32
4869	93	2	Ciamis	200-500	Kokosan	Lansium domesticum var. aquae	Meliaceae	0,7	4,1	1,5	3	0,04	0,00	2,98	0,00	0,03	0,13
4870	93	2	Ciamis	200-500	Sengon	Paraserianthes falcataria (L.) I.C.	Leguminosae / Fabaceae	0,33	9,9	7	14	0,04	0,01	30,14	0,03	0,35	1,30
4871	93	2	Ciamis	200-500	Tisuk	Hibiscus macrophyllus Roxb. ex	Malvaceae	0,4	10,2	3	9	0,04	0,01	25,42	0,03	0,30	1,10
4872	93	2	Ciamis	200-500	Nangka	Artocarpus heterophyllus Lam.	Moraceae	0,61	7,0	2	3,5	0,04	0,00	7,93	0,01	0,09	0,34
4873	93	2	Ciamis	200-500	Durian	Durio zibethinus L.	Malvaceae	0,57	7,3	4	6	0,04	0,00	13,24	0,01	0,16	0,57
4874	93	2	Ciamis	200-500	Sengon	Paraserianthes falcataria (L.) I.C.	Leguminosae / Fabaceae	0,33	20,7	10	15	0,04	0,03	124,63	0,12	1,46	5,37
4875	93	2	Ciamis	200-500	Jengkol	Archidendron pauciflorum (Bent	Leguminosae / Fabaceae	0,4	15,0	7	9	0,04	0,02	51,40	0,05	0,60	2,22
4876	93	2	Ciamis	200-500	Rambutan	Nephelium lappaceum L.	Sapindaceae	0,71	7,3	3	6	0,04	0,00	16,19	0,02	0,19	0,70
4877	93	2	Ciamis	200-500	Sengon	Paraserianthes falcataria (L.) I.C.	Leguminosae / Fabaceae	0,33	16,9	10	16	0,04	0,02	90,98	0,09	1,07	3,92
4878	93	2	Ciamis	200-500	Tisuk	Hibiscus macrophyllus Roxb. ex	Malvaceae	0,4	20,4	7	15	0,04	0,03	144,49	0,14	1,70	6,23
4879	93	2	Ciamis	200-500	Sengon	Paraserianthes falcataria (L.) I.C.	Leguminosae / Fabaceae	0,33	19,7	12	16	0,04	0,03	121,26	0,12	1,42	5,23
4880	93	2	Ciamis	200-500	Tisuk	Hibiscus macrophyllus Roxb. ex	Malvaceae	0,4	4,1	4	6	0,04	0,00	3,37	0,00	0,04	0,15
4881	93	2	Ciamis	200-500	Sengon	Paraserianthes falcataria (L.) I.C.	Leguminosae / Fabaceae	0,33	22,6	13	18	0,04	0,04	173,15	0,17	2,03	7,47
4882	93	2	Ciamis	200-500	Nangka	Artocarpus heterophyllus Lam.	Moraceae	0,61	9,2	3,5	6,5	0,04	0,01	23,19	0,02	0,27	1,00
4883	93	2	Ciamis	200-500	Tisuk	Hibiscus macrophyllus Roxb. ex	Malvaceae	0,4	6,7	7	8	0,04	0,00	10,55	0,01	0,12	0,45
4884	93	2	Ciamis	200-500	Renghas	Gluta renghas L.	Anacardiaceae	0,69	3,8	2	4	0,04	0,00	3,30	0,00	0,04	0,14
4885	93	2	Ciamis	200-500	Mahoni	Swietenia macrophylla King	Meliaceae	0,61	16,2	4	10	0,04	0,02	96,77	0,10	1,14	4,17
4886	93	2	Ciamis	200-500	Tisuk	Hibiscus macrophyllus Roxb. ex	Malvaceae	0,4	17,2	4	11	0,04	0,02	79,66	0,08	0,94	3,44
4887	94	2	Ciamis	200-500	Mahoni	Swietenia macrophylla King	Meliaceae	0,61	46,1	6	14	0,04	0,17	893,23	0,89	10,50	38,52
4888	94	2	Ciamis	200-500	Mahoni	Swietenia macrophylla King	Meliaceae	0,61	17,2	7	13	0,04	0,02	136,65	0,14	1,61	5,89
4889	94	2	Ciamis	200-500	Mahoni	Swietenia macrophylla King	Meliaceae	0,61	33,1	7	14	0,04	0,09	485,89	0,49	5,71	20,95
4890	94	2	Ciamis	200-500	Mahoni	Swietenia macrophylla King	Meliaceae	0,61	26,4	4	12	0,04	0,05	279,10	0,28	3,28	12,04
4891	94	2	Ciamis	200-500	Mahoni	Swietenia macrophylla King	Meliaceae	0,61	21,6	4	11	0,04	0,04	178,88	0,18	2,10	7,71
4892	94	2	Ciamis	200-500	Kelapa	Cocos nucifera L.	Arecaceae	0,5	27,4	4	7	0,04	0,06	58,40	0,06	0,69	2,52
4893	94	2	Ciamis	200-500	Tisuk	Hibiscus macrophyllus Roxb. ex	Malvaceae	0,4	5,4	4	5,5	0,04	0,00	5,08	0,01	0,06	0,22
4894	94	2	Ciamis	200-500	Sengon	Paraserianthes falcataria (L.) I.C.	Leguminosae / Fabaceae	0,33	11,8	12	15	0,04	0,01	44,39	0,04	0,52	1,91
4895	94	2	Ciamis	200-500	Jengkol	Archidendron pauciflorum (Bent	Leguminosae / Fabaceae	0,4	4,1	3	4,5	0,04	0,00	2,59	0,00	0,03	0,11
4896	94	2	Ciamis	200-500	Limus	Mangifera foetida Lour.	Anacardiaceae	0,73	10,8	7	10	0,04	0,01	54,27	0,05	0,64	2,34
4897	94	2	Ciamis	200-500	Sengon	Paraserianthes falcataria (L.) I.C.	Leguminosae / Fabaceae	0,33	15,9	11	17	0,04	0,02	86,43	0,09	1,02	3,73
4898	94	2	Ciamis	200-500	Sengon	Paraserianthes falcataria (L.) I.C.	Leguminosae / Fabaceae	0,33	10,8	9	13	0,04	0,01	33,35	0,03	0,39	1,44
4899	94	2	Ciamis	200-500	Sengon	Paraserianthes falcataria (L.) I.C.	Leguminosae / Fabaceae	0,33	9,9	9	15	0,04	0,01	32,10	0,03	0,38	1,38
4900	94	2	Ciamis	200-500	Jengkol	Archidendron pauciflorum (Bent	Leguminosae / Fabaceae	0,4	3,8	2,5	4	0,04	0,00	2,01	0,00	0,02	0,09
4901	94	2	Ciamis	200-500	Sengon	Paraserianthes falcataria (L.) I.C.	Leguminosae / Fabaceae	0,33	17,5	15	19	0,04	0,02	113,96	0,11	1,34	4,91
4902	94	2	Ciamis	200-500	Sengon	Paraserianthes falcataria (L.) I.C.	Leguminosae / Fabaceae	0,33	15,6	15	18	0,04	0,02	87,77	0,09	1,03	3,78
4903	94	2	Ciamis	200-500	Tisuk	Hibiscus macrophyllus Roxb. ex	Malvaceae	0,4	8,6	2,5	7,5	0,04	0,01	15,76	0,02	0,19	0,68
4904	94	2	Ciamis	200-500	Sengon	Paraserianthes falcataria (L.) I.C.	Leguminosae / Fabaceae	0,33	8,6	8	9,5	0,04	0,01	16,40	0,02	0,19	0,71
4905	94	2	Ciamis	200-500	Sengon	Paraserianthes falcataria (L.) I.C.	Leguminosae / Fabaceae	0,33	7,6	13	15	0,04	0,00	20,09	0,02	0,24	0,87
4906	94	2	Ciamis	200-500	Sengon	Paraserianthes falcataria (L.) I.C.	Leguminosae / Fabaceae	0,33	12,7	13	17	0,04	0,01	57,43	0,06	0,67	2,48
4907	94	2	Ciamis	200-500	Sengon	Paraserianthes falcataria (L.) I.C.	Leguminosae / Fabaceae	0,33	18,5	16	19	0,04	0,03	125,61	0,13	1,48	5,42
4908	94	2	Ciamis	200-500	Sengon	Paraserianthes falcataria (L.) I.C.	Leguminosae / Fabaceae	0,33	22,9	15	21	0,04	0,04	204,58	0,20	2,40	8,82
4909	94	2	Ciamis	200-500	Tisuk	Hibiscus macrophyllus Roxb. ex	Malvaceae	0,4	6,0	4	7	0,04	0,00	7,77	0,01	0,09	0,34
4910	94	2	Ciamis	200-500	Tisuk	Hibiscus macrophyllus Roxb. ex	Malvaceae	0,4	8,6	5	9	0,04	0,01	18,62	0,02	0,22	0,80
4911	94	2	Ciamis	200-500	Tisuk	Hibiscus macrophyllus Roxb. ex	Malvaceae	0,4	15,6	10	15	0,04	0,02	88,58	0,09	1,04	3,82
4912	94	2	Ciamis	200-500	Sengon	Paraserianthes falcataria (L.) I.C.	Leguminosae / Fabaceae	0,33	8,0	10	13	0,04	0,00	18,99	0,02	0,22	0,82
4913	94	2	Ciamis	200-500	Sengon	Paraserianthes falcataria (L.) I.C.	Leguminosae / Fabaceae	0,33	26,4	10	19	0,04	0,05	242,20	0,24	2,85	10,44
4914	94	2	Ciamis	200-500	Sengon	Paraserianthes falcataria (L.) I.C.	Leguminosae / Fabaceae	0,33	24,2	16	19	0,04	0,05	206,09	0,21	2,42	8,89
4915	94	2	Ciamis	200-500	Suren	Toona sureni (Blume) Merr.	Meliaceae	0,39	8,9	9	12	0,04	0,01	25,31	0,03	0,30	1,09
4916	94	2	Ciamis	200-500	Sengon	Paraserianthes falcataria (L.) I.C.	Leguminosae / Fabaceae	0,33	18,1	13	17	0,04	0,03	109,88	0,11	1,29	4,74
4917	94	2	Ciamis	200-500	Sengon	Paraserianthes falcataria (L.) I.C.	Leguminosae / Fabaceae	0,33	27,0	15	21	0,04	0,06	277,29	0,28	3,26	11,96
4918	94	2	Ciamis	200-500	Jengkol	Archidendron pauciflorum (Bent	Leguminosae / Fabaceae	0,4	7,6	4	6	0,04	0,00	10,35	0,01	0,12	0,45
4919	94	2	Ciamis	200-500	Tisuk	Hibiscus macrophyllus Roxb. ex	Malvaceae	0,4	18,8	12	19	0,04	0,03	154,58	0,15	1,82	6,67
4920	94	2	Ciamis	200-500	Sengon	Paraserianthes falcataria (L.) I.C.	Leguminosae / Fabaceae	0,33	8,3	11	14	0,04	0,01	21,84	0,02	0,26	0,94
4921	94	2	Ciamis	200-500	Sengon	Paraserianthes falcataria (L.) I.C.	Leguminosae / Fabaceae	0,33	8,9	10	13	0,04	0,01	23,37	0,02	0,27	1,01
4922	94	2	Ciamis	200-500	Sengon	Paraserianthes falcataria (L.) I.C.	Leguminosae / Fabaceae	0,33	5,1	7	8	0,04	0,00	5,37	0,01	0,06	0,23
4923	94	2	Ciamis	200-500	Sengon	Paraserianthes falcataria (L.) I.C.	Leguminosae / Fabaceae	0,33	23,5	17	21	0,04	0,04	215,11	0,22	2,53	9,28
4924	94	2	Ciamis	200-500	Kelapa	Cocos nucifera L.	Arecaceae	0,5	28,6	12	15	0,04	0,06	120,00	0,12	1,41	5,17
4925	94	2	Ciamis	200-500	Jati	Tectona grandis L.f.	Lamiaceae / Verbenaceae	0,7	14,0	2	10	0,04	0,02	83,76	0,08	0,98	3,61
4926	94	2	Ciamis	200-500	Jengkol	Archidendron pauciflorum (Bent	Leguminosae / Fabaceae	0,4	17,5	9	6	0,04	0,02	47,29	0,05	0,56	2,04
4927	94	2	Ciamis	200-500	Jati	Tectona grandis L.f.	Lamiaceae / Verbenaceae	0,7	9,9	2	6	0,04	0,01	27,62	0,03	0,32	1,19
4928	94	2	Ciamis	200-500	Sengon	Paraserianthes falcataria (L.) I.C.	Leguminosae / Fabaceae	0,33	6,4	8	13	0,04	0,00	12,62	0,01	0,15	0,54
4929	94	2	Ciamis	200-500	Tisuk	Hibiscus macrophyllus Roxb. ex	Malvaceae	0,4	18,5	15	14	0,04	0,03	113,26	0,11	1,33	4,88
4930	94	2	Ciamis	200-500	Kelapa	Cocos nucifera L.	Arecaceae	0,5	31,8	11	14	0,04	0,08	112,30	0,11	1,32	4,84
4931	94	2	Ciamis	200-500	Mahoni	Swietenia macrophylla King	Meliaceae	0,61	34,7	7	14	0,04	0,09	529,54	0,53	6,22	22,84
4932	94	2	Ciamis	200-500	Tisuk	Hibiscus macrophyllus Roxb. ex	Malvaceae	0,4	5,4	4	5	0,04	0,00	4,66	0,00	0,05	0,20
4933	94	2	Ciamis	200-500	Sengon	Paraserianthes falcataria (L.) I.C.	Leguminosae / Fabaceae	0,33	19,1	12	16	0,04	0,03	114,19	0,11	1,34	4,92
4934	94	2	Ciamis	200-500	Sengon	Paraserianthes falcataria (L.) I.C.	Leguminosae / Fabaceae	0,33	9,5	8	10,5	0,04	0,01	21,81	0,02	0,26	0,94
4935	94	2	Ciamis	200-500	Tisuk	Hibiscus macrophyllus Roxb. ex	Malvaceae	0,4	12,1	9	15	0,04	0,01	55,60	0,06	0,65	2,40
4936	94	2	Ciamis	200-500	Sengon	Paraserianthes falcataria (L.) I.C.	Leguminosae / Fabaceae	0,33	12,4	15	18	0,04	0,01	57,77	0,06	0,68	2,49
4937	95	3	Ciamis	200-500	Dukuh	Lansium domesticum Corrêa	Meliaceae	0,85	3,5	2	3	0,04	0,00	2,62	0,00	0,03	0,11
4938	95	3	Ciamis	200-500	Mahoni	Swietenia macrophylla King	Meliaceae	0,61	5,4	3,5	5	0,04	0,00	6,85	0,01	0,08	0,30
4939	95	3	Ciamis	200-500	Petai	Parkia speciosa Hassk.	Leguminosae / Fabaceae	0,45	8,9	4	8	0,04	0,01	19,90	0,02	0,23	0,86
4940	95	3	Ciamis	200-500	Mahoni	Swietenia macrophylla King	Meliaceae	0,61	4,5	3	4,5	0,04	0,00	4,36	0,00	0,05	0,19
4941	95	3	Ciamis	200-500	Mahoni	Swietenia macrophylla King	Meliaceae	0,61	3,5	2	2,5	0,04	0,00	1,64	0,00	0,02	0,07
4942	95	3	Ciamis	200-500	Mahoni	Swietenia macrophylla King	Meliaceae	0,61	23,9	3	10	0,04	0,04	196,15	0,20	2,30	8,46
4943	95	3	Ciamis	200-500	Jati	Tectona grandis L.f.	Lamiaceae / Verbenaceae	0,7	20,4	6	10	0,04	0,03	166,40	0,17	1,96	7,18
4944	95	3	Ciamis	200-500	Tisuk	Hibiscus macrophyllus Roxb. ex	Malvaceae	0,4	22,6	10	15	0,04	0,04	174,75	0,17	2,05	7,54
4945	95	3	Ciamis	200-500	Mahoni	Swietenia macrophylla King	Meliaceae	0,61	5,7	3	8	0,04	0,00	11,70	0,01	0,14	0,50
4946	95	3	Ciamis	200-500	Jati	Tectona grandis L.f.	Lamiaceae / Verbenaceae	0,7	16,5	3,5	12	0,04	0,02	134,42	0,13	1,58	5,80
4947	95	3	Ciamis	200-500	Mahoni	Swietenia macrophylla King	Meliaceae	0,61	3,5	4	5	0,04	0,00	3,09	0,00	0,04	0,13
4948	95	3	Ciamis	200-500	Jati	Tectona grandis L.f.	Lamiaceae / Verbenaceae	0,7	7,6	3,5	5	0,04	0,00	14,62	0,01	0,17	0,63
4949	95	3	Ciamis	200-500	Rambutan	Nephelium lappaceum L.	Sapindaceae	0,71	3,8	4	5	0,04	0,00	4,16	0,00	0,05	0,18
4950	95	3	Ciamis	200-500	Mahoni	Swietenia macrophylla King	Meliaceae	0,61	8,3	4	7	0,04	0,01	20,32	0,02	0,24	0,88
4951	95	3	Ciamis	200-500	Tisuk	Hibiscus macrophyllus Roxb. ex	Malvaceae	0,4	3,8	2	3	0,04	0,00	1,54	0,00	0,02	0,07
4952	95	3	Ciamis	200-500	Tisuk	Hibiscus macrophyllus Roxb. ex	Malvaceae	0,4	11,1	9	13	0,04	0,01	41,95	0,04	0,49	1,81
4953	95	3	Ciamis	200-500	Jati	Tectona grandis L.f.	Lamiaceae / Verbenaceae	0,7	8,9	4,5	6	0,04	0,01	22,92	0,02	0,27	0,99
4954	95	3	Ciamis	200-500	Sengon	Paraserianthes falcataria (L.) I.C.	Leguminosae / Fabaceae	0,33	26,1	10	17	0,04	0,05	213,93	0,21	2,51	9,23

4955	95	3	Ciamis	200-500	Sengon	Paraserianthes falcataria (L.) I.C.	Leguminosae / Fabaceae	0,33	9,5	10	11,5	0,04	0,01	23,70	0,02	0,28	1,02
4956	95	3	Ciamis	200-500	Jati	Tectona grandis L.f.	Lamiaceae / Verbenaceae	0,7	10,8	4	6	0,04	0,01	32,71	0,03	0,38	1,41
4957	95	3	Ciamis	200-500	Tisuk	Hibiscus macrophyllus Roxb. ex	Malvaceae	0,4	17,8	9	16	0,04	0,02	120,02	0,12	1,41	5,18
4958	95	3	Ciamis	200-500	Dukuh	Lansium domesticum Corrêa	Meliaceae	0,85	5,1	3	5	0,04	0,00	8,31	0,01	0,10	0,36
4959	95	3	Ciamis	200-500	Dukuh	Lansium domesticum Corrêa	Meliaceae	0,85	3,2	1,5	3	0,04	0,00	2,20	0,00	0,03	0,09
4960	95	3	Ciamis	200-500	Sengon	Paraserianthes falcataria (L.) I.C.	Leguminosae / Fabaceae	0,33	15,9	10	13	0,04	0,02	67,60	0,07	0,79	2,92
4961	95	3	Ciamis	200-500	Tisuk	Hibiscus macrophyllus Roxb. ex	Malvaceae	0,4	9,5	6	9	0,04	0,01	22,58	0,02	0,27	0,97
4962	95	3	Ciamis	200-500	Sengon	Paraserianthes falcataria (L.) I.C.	Leguminosae / Fabaceae	0,33	16,2	12	15	0,04	0,02	79,92	0,08	0,94	3,45
4963	95	3	Ciamis	200-500	Jati	Tectona grandis L.f.	Lamiaceae / Verbenaceae	0,7	11,8	6	9	0,04	0,01	55,37	0,06	0,65	2,39
4964	95	3	Ciamis	200-500	Jati	Tectona grandis L.f.	Lamiaceae / Verbenaceae	0,7	9,9	3	6	0,04	0,01	27,62	0,03	0,32	1,19
4965	95	3	Ciamis	200-500	Dukuh	Lansium domesticum Corrêa	Meliaceae	0,85	3,5	2	3	0,04	0,00	2,62	0,00	0,03	0,11
4966	95	3	Ciamis	200-500	Tisuk	Hibiscus macrophyllus Roxb. ex	Malvaceae	0,4	15,0	10	14	0,04	0,02	77,04	0,08	0,91	3,32
4967	95	3	Ciamis	200-500	Dukuh	Lansium domesticum Corrêa	Meliaceae	0,85	3,2	2	3	0,04	0,00	2,20	0,00	0,03	0,09
4968	95	3	Ciamis	200-500	Tisuk	Hibiscus macrophyllus Roxb. ex	Malvaceae	0,4	13,0	5	9	0,04	0,01	40,02	0,04	0,47	1,73
4969	95	3	Ciamis	200-500	Jati	Tectona grandis L.f.	Lamiaceae / Verbenaceae	0,7	6,4	3	5	0,04	0,00	10,47	0,01	0,12	0,45
4970	95	3	Ciamis	200-500	Tisuk	Hibiscus macrophyllus Roxb. ex	Malvaceae	0,4	4,1	2,5	5	0,04	0,00	2,85	0,00	0,03	0,12
4971	95	3	Ciamis	200-500	Tisuk	Hibiscus macrophyllus Roxb. ex	Malvaceae	0,4	5,1	5	7	0,04	0,00	5,67	0,01	0,07	0,24
4972	95	3	Ciamis	200-500	Jati	Tectona grandis L.f.	Lamiaceae / Verbenaceae	0,7	8,9	5	8	0,04	0,01	29,83	0,03	0,35	1,29
4973	95	3	Ciamis	200-500	Sengon	Paraserianthes falcataria (L.) I.C.	Leguminosae / Fabaceae	0,33	23,5	13	17	0,04	0,04	177,26	0,18	2,08	7,64
4974	95	3	Ciamis	200-500	Dukuh	Lansium domesticum Corrêa	Meliaceae	0,85	3,5	2	5	0,04	0,00	4,18	0,00	0,05	0,18
4975	95	3	Ciamis	200-500	Jati	Tectona grandis L.f.	Lamiaceae / Verbenaceae	0,7	10,8	6	12	0,04	0,01	61,72	0,06	0,73	2,66
4976	95	3	Ciamis	200-500	Sengon	Paraserianthes falcataria (L.) I.C.	Leguminosae / Fabaceae	0,33	18,1	13	19	0,04	0,03	121,67	0,12	1,43	5,25
4977	95	3	Ciamis	200-500	Sengon	Paraserianthes falcataria (L.) I.C.	Leguminosae / Fabaceae	0,33	9,9	11	13	0,04	0,01	28,16	0,03	0,33	1,21
4978	95	3	Ciamis	200-500	Jati	Tectona grandis L.f.	Lamiaceae / Verbenaceae	0,7	14,3	9	11	0,04	0,02	95,24	0,10	1,12	4,11
4979	95	3	Ciamis	200-500	Sengon	Paraserianthes falcataria (L.) I.C.	Leguminosae / Fabaceae	0,33	17,8	17	21	0,04	0,02	129,10	0,13	1,52	5,57
4980	95	3	Ciamis	200-500	Jati	Tectona grandis L.f.	Lamiaceae / Verbenaceae	0,7	14,6	8	13	0,04	0,02	115,55	0,12	1,36	4,98
4981	95	3	Ciamis	200-500	Petai	Parkia speciosa Hassk.	Leguminosae / Fabaceae	0,45	8,0	7	9	0,04	0,00	18,01	0,02	0,21	0,78
4982	95	3	Ciamis	200-500	Sengon	Paraserianthes falcataria (L.) I.C.	Leguminosae / Fabaceae	0,33	19,7	17	20	0,04	0,03	148,76	0,15	1,75	6,41
4983	95	3	Ciamis	200-500	Sengon	Paraserianthes falcataria (L.) I.C.	Leguminosae / Fabaceae	0,33	19,4	17	20	0,04	0,03	144,39	0,14	1,70	6,23
4984	95	3	Ciamis	200-500	Dukuh	Lansium domesticum Corrêa	Meliaceae	0,85	3,2	2,5	3	0,04	0,00	2,20	0,00	0,03	0,09
4985	95	3	Ciamis	200-500	Mahoni	Swietenia macrophylla King	Meliaceae	0,61	3,5	2	3	0,04	0,00	1,93	0,00	0,02	0,08
4986	95	3	Ciamis	200-500	Tisuk	Hibiscus macrophyllus Roxb. ex	Malvaceae	0,4	5,4	6	7	0,04	0,00	6,34	0,01	0,07	0,27
4987	95	3	Ciamis	200-500	Mahoni	Swietenia macrophylla King	Meliaceae	0,61	19,7	7	13	0,04	0,03	176,00	0,18	2,07	7,59
4988	95	3	Ciamis	200-500	Mahoni	Swietenia macrophylla King	Meliaceae	0,61	3,2	3	4	0,04	0,00	2,11	0,00	0,02	0,09
4989	95	3	Ciamis	200-500	Kelapa	Cocos nucifera L.	Arecaceae	0,5	28,0	13	16	0,04	0,06	127,70	0,13	1,50	5,51
4990	95	3	Ciamis	200-500	Tisuk	Hibiscus macrophyllus Roxb. ex	Malvaceae	0,4	4,5	4	5	0,04	0,00	3,26	0,00	0,04	0,14
4991	95	3	Ciamis	200-500	Jati	Tectona grandis L.f.	Lamiaceae / Verbenaceae	0,7	8,3	3,5	8,5	0,04	0,01	27,53	0,03	0,32	1,19
4992	95	3	Ciamis	200-500	Jati	Tectona grandis L.f.	Lamiaceae / Verbenaceae	0,7	11,5	4	8	0,04	0,01	47,27	0,05	0,56	2,04
4993	95	3	Ciamis	200-500	Sengon	Paraserianthes falcataria (L.) I.C.	Leguminosae / Fabaceae	0,33	10,5	9	10	0,04	0,01	24,83	0,02	0,29	1,07
4994	95	3	Ciamis	200-500	Jati	Tectona grandis L.f.	Lamiaceae / Verbenaceae	0,7	10,8	3	7	0,04	0,01	37,67	0,04	0,44	1,62
4995	95	3	Ciamis	200-500	Petai	Parkia speciosa Hassk.	Leguminosae / Fabaceae	0,45	4,5	5	6	0,04	0,00	4,29	0,00	0,05	0,19
4996	95	3	Ciamis	200-500	Jati	Tectona grandis L.f.	Lamiaceae / Verbenaceae	0,7	4,8	4	5	0,04	0,00	6,18	0,01	0,07	0,27
4997	95	3	Ciamis	200-500	Tisuk	Hibiscus macrophyllus Roxb. ex	Malvaceae	0,4	7,6	7	13	0,04	0,00	21,01	0,02	0,25	0,91
4998	95	3	Ciamis	200-500	Tisuk	Hibiscus macrophyllus Roxb. ex	Malvaceae	0,4	8,0	6	9	0,04	0,00	16,17	0,02	0,19	0,70
4999	95	3	Ciamis	200-500	Mahoni	Swietenia macrophylla King	Meliaceae	0,61	4,5	4	5	0,04	0,00	4,80	0,00	0,06	0,21
5000	96	2	Ciamis	200-500	Kelapa	Cocos nucifera L.	Arecaceae	0,5	23,9	3	6	0,04	0,04	50,70	0,05	0,60	2,19
5001	96	2	Ciamis	200-500	Sengon	Paraserianthes falcataria (L.) I.C.	Leguminosae / Fabaceae	0,33	13,4	12	2	0,04	0,01	8,84	0,01	0,10	0,38
5002	96	2	Ciamis	200-500	Sengon	Paraserianthes falcataria (L.) I.C.	Leguminosae / Fabaceae	0,33	41,0	13	23	0,04	0,13	647,17	0,65	7,60	27,91
5003	96	2	Ciamis	200-500	Sengon	Paraserianthes falcataria (L.) I.C.	Leguminosae / Fabaceae	0,33	16,5	10	12	0,04	0,02	67,50	0,07	0,79	2,91
5004	96	2	Ciamis	200-500	Sengon	Paraserianthes falcataria (L.) I.C.	Leguminosae / Fabaceae	0,33	25,1	14	24	0,04	0,05	274,03	0,27	3,22	11,82
5005	96	2	Ciamis	200-500	Sengon	Paraserianthes falcataria (L.) I.C.	Leguminosae / Fabaceae	0,33	36,3	15	26	0,04	0,10	577,36	0,58	6,78	24,90
5006	96	2	Ciamis	200-500	Sengon	Paraserianthes falcataria (L.) I.C.	Leguminosae / Fabaceae	0,33	12,1	9	13	0,04	0,01	40,89	0,04	0,48	1,76
5007	96	2	Ciamis	200-500	Kiciat	Ficus septica Burm.f.	Moraceae	0,42	6,4	4	6	0,04	0,00	7,75	0,01	0,09	0,33
5008	96	2	Ciamis	200-500	Tisuk	Hibiscus macrophyllus Roxb. ex	Malvaceae	0,4	8,0	5	8	0,04	0,00	14,52	0,01	0,17	0,63
5009	96	2	Ciamis	200-500	Sengon	Paraserianthes falcataria (L.) I.C.	Leguminosae / Fabaceae	0,33	24,8	14	23	0,04	0,05	257,48	0,26	3,03	11,10
5010	96	2	Ciamis	200-500	Tisuk	Hibiscus macrophyllus Roxb. ex	Malvaceae	0,4	8,9	2	3	0,04	0,01	7,28	0,01	0,09	0,31
5011	96	2	Ciamis	200-500	Tisuk	Hibiscus macrophyllus Roxb. ex	Malvaceae	0,4	4,1	2	4	0,04	0,00	2,32	0,00	0,03	0,10
5012	96	2	Ciamis	200-500	Sengon	Paraserianthes falcataria (L.) I.C.	Leguminosae / Fabaceae	0,33	16,9	10	15	0,04	0,02	85,75	0,09	1,01	3,70
5013	96	2	Ciamis	200-500	Sengon	Paraserianthes falcataria (L.) I.C.	Leguminosae / Fabaceae	0,33	36,9	11	20	0,04	0,11	468,71	0,47	5,51	20,21
5014	96	2	Ciamis	200-500	Tisuk	Hibiscus macrophyllus Roxb. ex	Malvaceae	0,4	9,9	3	9	0,04	0,01	23,98	0,02	0,28	1,03
5015	96	2	Ciamis	200-500	Nangka	Artocarpus heterophyllus Lam.	Moraceae	0,61	11,1	2	7	0,04	0,01	35,02	0,04	0,41	1,51
5016	96	2	Ciamis	200-500	Sengon	Paraserianthes falcataria (L.) I.C.	Leguminosae / Fabaceae	0,33	14,6	10	14	0,04	0,02	62,10	0,06	0,73	2,68
5017	96	2	Ciamis	200-500	Sengon	Paraserianthes falcataria (L.) I.C.	Leguminosae / Fabaceae	0,33	17,8	14	21	0,04	0,02	129,10	0,13	1,52	5,57
5018	96	2	Ciamis	200-500	Sengon	Paraserianthes falcataria (L.) I.C.	Leguminosae / Fabaceae	0,33	13,4	9	14	0,04	0,01	52,57	0,05	0,62	2,27
5019	96	2	Ciamis	200-500	Sengon	Paraserianthes falcataria (L.) I.C.	Leguminosae / Fabaceae	0,33	8,6	6	11	0,04	0,01	18,76	0,02	0,22	0,81
5020	96	2	Ciamis	200-500	Sengon	Paraserianthes falcataria (L.) I.C.	Leguminosae / Fabaceae	0,33	13,0	10	15	0,04	0,01	53,58	0,05	0,63	2,31
5021	96	2	Ciamis	200-500	Kelapa	Cocos nucifera L.	Arecaceae	0,5	24,2	0,2	5	0,04	0,05	43,00	0,04	0,51	1,85
5022	96	2	Ciamis	200-500	Jengkol	Archidendron pauciflorum (Bent	Leguminosae / Fabaceae	0,4	8,6	4,5	6	0,04	0,01	12,84	0,01	0,15	0,55
5023	96	2	Ciamis	200-500	Sengon	Paraserianthes falcataria (L.) I.C.	Leguminosae / Fabaceae	0,33	25,8	21	27	0,04	0,05	319,56	0,32	3,75	13,78
5024	96	2	Ciamis	200-500	Sengon	Paraserianthes falcataria (L.) I.C.	Leguminosae / Fabaceae	0,33	20,7	15	22	0,04	0,03	177,01	0,18	2,08	7,63
5025	96	2	Ciamis	200-500	Kakao	Theobroma cacao L.	Malvaceae	0,36	3,2	1,3	2,5	0,04	0,00	0,85	0,00	0,01	0,04
5026	96	2	Ciamis	200-500	Petai	Parkia speciosa Hassk.	Leguminosae / Fabaceae	0,45	7,6	3	5	0,04	0,00	9,76	0,01	0,11	0,42
5027	96	2	Ciamis	200-500	Sengon	Paraserianthes falcataria (L.) I.C.	Leguminosae / Fabaceae	0,33	12,4	6	11	0,04	0,01	36,80	0,04	0,43	1,59
5028	96	2	Ciamis	200-500	Sengon	Paraserianthes falcataria (L.) I.C.	Leguminosae / Fabaceae	0,33	27,7	22	28	0,04	0,06	376,59	0,38	4,42	16,24
5029	96	2	Ciamis	200-500	Sengon	Paraserianthes falcataria (L.) I.C.	Leguminosae / Fabaceae	0,33	28,3	19	27	0,04	0,06	379,74	0,38	4,46	16,38
5030	96	2	Ciamis	200-500	Pangsor	Ficus callosa Willd.	Moraceae	0,33	3,8	2	3	0,04	0,00	1,29	0,00	0,02	0,06
5031	96	2	Ciamis	200-500	Sengon	Paraserianthes falcataria (L.) I.C.	Leguminosae / Fabaceae	0,33	9,9	7	10	0,04	0,01	22,14	0,02	0,26	0,95
5032	96	2	Ciamis	200-500	Sengon	Paraserianthes falcataria (L.) I.C.	Leguminosae / Fabaceae	0,33	12,7	11	14	0,04	0,01	48,07	0,05	0,56	2,07
5033	96	2	Ciamis	200-500	Sengon	Paraserianthes falcataria (L.) I.C.	Leguminosae / Fabaceae	0,33	28,0	12	20	0,04	0,06	282,56	0,28	3,32	12,18
5034	97	3	Ciamis	200-500	Sengon	Paraserianthes falcataria (L.) I.C.	Leguminosae / Fabaceae	0,33	6,7	3	7	0,04	0,00	7,83	0,01	0,09	0,34
5035	97	3	Ciamis	200-500	Sengon	Paraserianthes falcataria (L.) I.C.	Leguminosae / Fabaceae	0,33	8,0	3	7	0,04	0,00	10,77	0,01	0,13	0,46
5036	97	3	Ciamis	200-500	Sengon	Paraserianthes falcataria (L.) I.C.	Leguminosae / Fabaceae	0,33	4,1	3	4,5	0,04	0,00	2,17	0,00	0,03	0,09
5037	97	3	Ciamis	200-500	Dukuh	Lansium domesticum Corrêa	Meliaceae	0,85	7,3	2	5	0,04	0,00	16,16	0,02	0,19	0,70
5038	97	3	Ciamis	200-500	Sengon	Paraserianthes falcataria (L.) I.C.	Leguminosae / Fabaceae	0,33	5,1	4	7	0,04	0,00	4,75	0,00	0,06	0,21
5039	97	3	Ciamis	200-500	Sengon	Paraserianthes falcataria (L.) I.C.	Leguminosae / Fabaceae	0,33	8,9	3	7	0,04	0,01	13,26	0,01	0,16	0,57
5040	97	3	Ciamis	200-500	Sengon	Paraserianthes falcataria (L.) I.C.	Leguminosae / Fabaceae	0,33	5,7	3,5	5	0,04	0,00	4,34	0,00	0,05	0,19
5041	97	3	Ciamis	200-500	Sengon	Paraserianthes falcataria (L.) I.C.	Leguminosae / Fabaceae	0,33	3,2	3,5	4,5	0,04	0,00	1,34	0,00	0,02	0,06
5042	97	3	Ciamis	200-500	Sengon	Paraserianthes falcataria (L.) I.C.	Leguminosae / Fabaceae	0,33	4,5	3	4,5	0,04	0,00	2,48	0,00	0,03	0,11
5043	97	3	Ciamis	200-500	Sengon	Paraserianthes falcataria (L.) I.C.	Leguminosae / Fabaceae	0,33	9,9	3	7	0,04	0,01	15,97	0,02	0,19	0,69
5044	97	3	Ciamis	200-500	Sengon	Paraserianthes falcataria (L.) I.C.	Leguminosae / Fabaceae	0,33	8,9	6	8	0,04	0,01	14,98	0,01	0,18	0,65
5045	97	3	Ciamis	200-500	Tisuk	Hibiscus macrophyllus Roxb. ex	Malvaceae	0,4	7,3	3,5	8,5	0,04	0,00	13,17	0,01	0,15	0,57
5046	97	3	Ciamis	200-500	Sengon	Paraserianthes falcataria (L.) I.C.	Leguminosae / Fabaceae	0,33	22,3	10	14	0,04	0,04	134,01	0,13	1,57	5,78
5047	97	3	Ciamis	200-500	Tisuk	Hibiscus macrophyllus Roxb. ex	Malvaceae	0,4	4,8	3	10	0,04	0,00	6,99	0,01	0,08	0,30
5048	97	3	Ciamis	200-500	Sengon	Paraserianthes falcataria (L.) I.C.	Leguminosae / Fabaceae	0,33	18,5	7	13	0,04	0,03	88,73	0,09	1,04	3,83
5049	97	3	Ciamis	200-500	Sengon	Paraserianthes falcataria (L.) I.C.	Leguminosae / Fabaceae	0,33	2,9	3	4	0,04	0,00	0,99	0,00	0,01	0,04
5050	97	3	Ciamis	200-500	Sengon	Paraserianthes falcataria (L.) I.C.	Leguminosae / Fabaceae	0,33	6,7	3,5	7,5	0,04	0,00	8,34	0,01	0,10	0,36
5051	97	3	Ciamis	200-500	Sengon	Paraserianthes falcataria (L.) I.C.	Leguminosae / Fabaceae	0,33	7,3	6	8	0,04	0,00	10,45	0,01	0,12	0,45
5052	97	3	Ciamis	200-500	Sengon	Paraserianthes falcataria (L.) I.C.	Leguminosae / Fabaceae	0,33	8,0	3,5	7	0,04	0,00	10,77	0,01	0,13	0,46
5053	97	3	Ciamis	200-500	Sengon	Paraserianthes falcataria (L.) I.C.	Leguminosae / Fabaceae	0,33	6,0	4	5	0,04	0,00	4,79	0,00	0,06	0,21
5054	97	3	Ciamis	200-500	Sengon	Paraserianthes falcataria (L.) I.C.	Leguminosae / Fabaceae	0,33	5,4	4	6	0,04	0,00	4,61	0,00	0,05	0,20
5055	97	3	Ciamis	200-500	Tisuk	Hibiscus macrophyllus Roxb. ex	Malvaceae	0,4	23,5	9	15	0,04	0,04	188,51	0,19	2,22	8,13
5056	97	3	Ciamis	200-500	Sengon	Paraserianthes falcataria (L.) I.C.	Leguminosae / Fabaceae	0,33	3,2	4	5	0,04	0,00	1,48	0,00	0,02	0,06
5057	97	3	Ciamis	200-500	Petai	Parkia speciosa Hassk.	Leguminosae / Fabaceae	0,45	11,8	7	8,5	0,04	0,01	35,06	0,04	0,41	1,51
5058	97	3	Ciamis	200-500	Sengon	Paraserianthes falcataria (L.) I.C.	Leguminosae / Fabaceae	0,33	4,8	4	5	0,04	0,00	3,10	0,00	0,04	0,13
5059	97	3	Ciamis	200-500	Tisuk	Hibiscus macrophyllus Roxb. ex	Malvaceae	0,4	20,7	10	14	0,04	0,03	139,55	0,14	1,64	6,02
5060	97	3	Ciamis	200-500	Sengon	Paraserianthes falcataria (L.) I.C.	Leguminosae / Fabaceae	0,33	6,0	4	6	0,04	0,00	5,66	0,01	0,07	0,24
5061	97	3	Ciamis	200-500	Tisuk	Hibiscus macrophyllus Roxb. ex	Malvaceae	0,4	15,6	10	14	0,04	0,02	83,16	0,08	0,98	3,59
5062	97	3	Ciamis	200-500	Tisuk	Hibiscus macrophyllus Roxb. ex	Malvaceae	0,4	16,5	10	13	0,04	0,02	86,64	0,09	1,02	3,74
5063	97	3	Ciamis	200-500	Tisuk	Hibiscus macrophyllus Roxb. ex	Malvaceae	0,4	17,2	10	14	0,04	0,02	99,36	0,10	1,17	4,28
5064	97	3	Ciamis	200-500	Tisuk	Hibiscus macrophyllus Roxb. ex	Malvaceae	0,4	19,7	9	14	0,04	0,03	127,97	0,13	1,50	5,52
5065	97	3	Ciamis	200-500	Petai	Parkia speciosa Hassk.	Leguminosae / Fabaceae	0,45	22,3	8	14	0,04	0,04	178,05	0,18	2,09	7,68
5066	97	3	Ciamis	200-500	Sengon	Paraserianthes falcataria (L.) I.C.	Leguminosae / Fabaceae	0,33	6,7	3,5	5,5	0,04	0,00	6,27	0,01	0,07	0,27
5067	97	3	Ciamis	200-500	Dukuh	Lansium domesticum Corrêa	Meliaceae	0,85	14,0	1,7	8	0,04	0,02	81,56	0,08	0,96	3,52
5068	97	3	Ciamis	200-500	Dukuh	Lansium domesticum Corrêa	Meliaceae	0,85	5,7	2	4	0,04	0,00	8,41	0,01	0,10	0,36
5069	97	3	Ciamis	200-500	Dukuh	Lansium domesticum Corrêa	Meliaceae	0,85	11,1	1,5	6	0,04	0,01	41,21	0,04	0,48	1,78
5070	97	3	Ciamis	200-500	Dukuh	Lansium domesticum Corrêa	Meliaceae	0,85	7,6	1,5	5	0,04	0,00	17,47	0,02	0,21	0,75
5071	97	3	Ciamis	200-500	Sengon	Paraserianthes falcataria (L.) I.C.	Leguminosae / Fabaceae	0,33	4,1	3	5	0,04	0,00	2,39	0,00	0,03	0,10
5072	98	1	Ciamis	200-500	Mahoni	Swietenia macrophylla King	Meliaceae	0,61	6,7	6	8	0,0025	0,00	15,52	0,02	2,92	10,71
5073	98	1	Ciamis	200-500	Mahoni	Swietenia macrophylla King	Meliaceae	0,61	2,2	3	3,3	0,0025	0,00	0,92	0,00	0,17	0,64
5074	98	1	Ciamis	200-500	Mahoni	Swietenia macrophylla King	Meliaceae	0,61	4,8	4	6	0,0025	0,00	6,44	0,01	1,21	4,44
5075	98	1	Ciamis	200-500	Mahoni	Swietenia macrophylla King	Meliaceae	0,61	4,8	6	7	0,0025	0,00	7,42	0,01	1,39	5,12
5076	98	1	Ciamis	200-500	Kiciat	Ficus septica Burm.f.	Moraceae	0,42	5,4	4	5	0,0025	0,00	4,87	0,00	0,92	3,36
5077	98	1	Ciamis	200-500	Mahoni	Swietenia macrophylla King	Meliaceae	0,61	7,6	5	7	0,0025	0,00	17,54	0,02	3,30	12,10
5078	98	1	Ciamis	200-500	Tisuk	Hibiscus macrophyllus Roxb. ex	Malvaceae	0,4	13,4	9	14	0,0025	0,00	62,70	0,06	11,79	43,26

5079	98	1	Ciamis	200-500	Petai	Parkia speciosa Hassk.	Leguminosae / Fabaceae	0,45	2,2	2,5	3	0,0025	0,00	0,64	0,00	0,12	0,44
5080	98	1	Ciamis	200-500	Mahoni	Swietenia macrophylla King	Meliaceae	0,61	3,5	4,5	5	0,0025	0,00	3,09	0,00	0,58	2,13
5081	98	1	Ciamis	200-500	Mahoni	Swietenia macrophylla King	Meliaceae	0,61	2,2	2	3	0,0025	0,00	0,84	0,00	0,16	0,58
5082	98	1	Ciamis	200-500	Mahoni	Swietenia macrophylla King	Meliaceae	0,61	6,0	5	7	0,0025	0,00	11,44	0,01	2,15	7,89
5083	98	1	Ciamis	200-500	Sengon	Paraserianthes falcataria (L.) I.C.	Leguminosae / Fabaceae	0,33	15,6	9	12	0,0025	0,00	60,54	0,06	11,38	41,77
5084	98	1	Ciamis	200-500	Heuras	Vitex pubescens Vahl	Lamiaceae / Verbenaceae	0,88	2,9	3	3,5	0,0025	0,00	2,16	0,00	0,41	1,49
5085	98	1	Ciamis	200-500	Petai	Parkia speciosa Hassk.	Leguminosae / Fabaceae	0,45	4,8	4	5	0,0025	0,00	4,12	0,00	0,78	2,85
5086	98	1	Ciamis	200-500	Heuras	Vitex pubescens Vahl	Lamiaceae / Verbenaceae	0,88	2,9	4	5	0,0025	0,00	2,99	0,00	0,56	2,06
5087	98	1	Ciamis	200-500	Mahoni	Swietenia macrophylla King	Meliaceae	0,61	4,8	3,5	6	0,0025	0,00	6,44	0,01	1,21	4,44
5088	98	1	Ciamis	200-500	Mahoni	Swietenia macrophylla King	Meliaceae	0,61	4,1	3	4,5	0,0025	0,00	3,81	0,00	0,72	2,63
5089	98	1	Ciamis	200-500	Mahoni	Swietenia macrophylla King	Meliaceae	0,61	4,8	2	3	0,0025	0,00	3,41	0,00	0,64	2,35
5090	98	1	Ciamis	200-500	Heuras	Vitex pubescens Vahl	Lamiaceae / Verbenaceae	0,88	2,9	4	4,5	0,0025	0,00	2,72	0,00	0,51	1,87
5091	98	1	Ciamis	200-500	Heuras	Vitex pubescens Vahl	Lamiaceae / Verbenaceae	0,88	2,2	3	3,5	0,0025	0,00	1,36	0,00	0,26	0,94
5092	98	1	Ciamis	200-500	Mahoni	Swietenia macrophylla King	Meliaceae	0,61	3,5	3,5	4,5	0,0025	0,00	2,80	0,00	0,53	1,93
5093	98	1	Ciamis	200-500	Mahoni	Swietenia macrophylla King	Meliaceae	0,61	3,8	3,5	0,5	0,0025	0,00	0,44	0,00	0,08	0,30
5094	98	1	Ciamis	200-500	Mahoni	Swietenia macrophylla King	Meliaceae	0,61	2,5	2	3	0,0025	0,00	1,08	0,00	0,20	0,74
5095	98	1	Ciamis	200-500	Mahoni	Swietenia macrophylla King	Meliaceae	0,61	3,2	3,5	5	0,0025	0,00	2,59	0,00	0,49	1,79
5096	98	1	Ciamis	200-500	Mahoni	Swietenia macrophylla King	Meliaceae	0,61	5,7	3,5	5,5	0,0025	0,00	8,30	0,01	1,56	5,73
5097	98	1	Ciamis	200-500	Mahoni	Swietenia macrophylla King	Meliaceae	0,61	7,0	5	6,5	0,0025	0,00	13,98	0,01	2,63	9,64
5098	98	1	Ciamis	200-500	Mahoni	Swietenia macrophylla King	Meliaceae	0,61	2,9	3	4,5	0,0025	0,00	1,94	0,00	0,36	1,34
5099	98	1	Ciamis	200-500	Mahoni	Swietenia macrophylla King	Meliaceae	0,61	4,1	4	6,5	0,0025	0,00	5,33	0,01	1,00	3,68
5100	98	1	Ciamis	200-500	Mahoni	Swietenia macrophylla King	Meliaceae	0,61	2,2	2	2,3	0,0025	0,00	0,66	0,00	0,12	0,46
5101	98	1	Ciamis	200-500	Kiciat	Ficus septica Burm.f.	Moraceae	0,42	3,2	2,5	2,5	0,0025	0,00	0,98	0,00	0,18	0,67
5102	98	1	Ciamis	200-500	Mahoni	Swietenia macrophylla King	Meliaceae	0,61	6,7	4	6	0,0025	0,00	11,93	0,01	2,24	8,23
5103	98	1	Ciamis	200-500	Tisuk	Hibiscus macrophyllus Roxb. ex I	Malvaceae	0,4	8,9	6	8	0,0025	0,00	17,87	0,02	3,36	12,33
5104	98	1	Ciamis	200-500	Mahoni	Swietenia macrophylla King	Meliaceae	0,61	3,2	3	4	0,0025	0,00	2,11	0,00	0,40	1,46
5105	98	1	Ciamis	200-500	Mahoni	Swietenia macrophylla King	Meliaceae	0,61	2,2	1,8	2	0,0025	0,00	0,58	0,00	0,11	0,40
5106	98	1	Ciamis	200-500	Tisuk	Hibiscus macrophyllus Roxb. ex I	Malvaceae	0,4	15,3	9	12	0,0025	0,00	69,53	0,07	13,07	47,97
5107	98	1	Ciamis	200-500	Kokosan	Lansium domesticum var. aquae	Meliaceae	0,7	2,2	3	4	0,0025	0,00	1,25	0,00	0,23	0,86
5108	98	1	Ciamis	200-500	Mahoni	Swietenia macrophylla King	Meliaceae	0,61	3,8	3,5	4,5	0,0025	0,00	3,29	0,00	0,62	2,27
5109	98	1	Ciamis	200-500	Mahoni	Swietenia macrophylla King	Meliaceae	0,61	2,9	3,5	4	0,0025	0,00	1,74	0,00	0,33	1,20
5110	98	1	Ciamis	200-500	Mahoni	Swietenia macrophylla King	Meliaceae	0,61	2,2	2	2,5	0,0025	0,00	0,71	0,00	0,13	0,49
5111	98	1	Ciamis	200-500	Kiciat	Ficus septica Burm.f.	Moraceae	0,42	4,5	3	5	0,0025	0,00	3,41	0,00	0,64	2,35
5112	98	1	Ciamis	200-500	Kokosan	Lansium domesticum var. aquae	Meliaceae	0,7	1,6	1	2,5	0,0025	0,00	0,44	0,00	0,08	0,30
5113	98	1	Ciamis	200-500	Mahoni	Swietenia macrophylla King	Meliaceae	0,61	2,9	3	4,5	0,0025	0,00	1,94	0,00	0,36	1,34
5114	98	1	Ciamis	200-500	Tikukuran	Sp6	0	0,5	4,1	5	6,5	0,0025	0,00	4,44	0,00	0,84	3,07
5115	98	1	Ciamis	200-500	Mahoni	Swietenia macrophylla King	Meliaceae	0,61	1,9	1,5	2	0,0025	0,00	0,44	0,00	0,08	0,30
5116	98	1	Ciamis	200-500	Mahoni	Swietenia macrophylla King	Meliaceae	0,61	2,5	2	3	0,0025	0,00	1,08	0,00	0,20	0,74
5117	98	1	Ciamis	200-500	Mahoni	Swietenia macrophylla King	Meliaceae	0,61	6,7	6	7	0,01	0,00	13,74	0,01	0,65	2,37
5118	98	1	Ciamis	200-500	Mahoni	Swietenia macrophylla King	Meliaceae	0,61	5,4	4,5	5,5	0,01	0,00	7,48	0,01	0,35	1,29
5119	98	1	Ciamis	200-500	Mahoni	Swietenia macrophylla King	Meliaceae	0,61	9,2	5	7	0,01	0,00	24,81	0,02	1,17	4,28
5120	98	1	Ciamis	200-500	Tisuk	Hibiscus macrophyllus Roxb. ex I	Malvaceae	0,4	11,8	7	10	0,01	0,00	36,52	0,04	1,72	6,30
5121	98	1	Ciamis	200-500	Mahoni	Swietenia macrophylla King	Meliaceae	0,61	5,1	3,5	5	0,01	0,00	6,13	0,01	0,29	1,06
5122	98	1	Ciamis	200-500	Mahoni	Swietenia macrophylla King	Meliaceae	0,61	9,2	5	6	0,01	0,00	21,55	0,02	1,01	3,72
5123	98	1	Ciamis	200-500	Sengon	Paraserianthes falcataria (L.) I.C.	Leguminosae / Fabaceae	0,33	17,8	9	12	0,01	0,01	77,32	0,08	3,63	13,34
5124	98	1	Ciamis	200-500	Mahoni	Swietenia macrophylla King	Meliaceae	0,61	9,5	6	8	0,01	0,00	29,84	0,03	1,40	5,15
5125	98	1	Ciamis	200-500	Mahoni	Swietenia macrophylla King	Meliaceae	0,61	6,4	6	7,5	0,01	0,00	13,38	0,01	0,63	2,31
5126	98	1	Ciamis	200-500	Mahoni	Swietenia macrophylla King	Meliaceae	0,61	8,0	5	9	0,01	0,00	23,80	0,02	1,12	4,11
5127	98	1	Ciamis	200-500	Mahoni	Swietenia macrophylla King	Meliaceae	0,61	10,8	4	6	0,01	0,00	28,84	0,03	1,36	4,97
5128	98	1	Ciamis	200-500	Mahoni	Swietenia macrophylla King	Meliaceae	0,61	7,6	6	8	0,01	0,00	19,83	0,02	0,93	3,42
5129	98	1	Ciamis	200-500	Petai	Parkia speciosa Hassk.	Leguminosae / Fabaceae	0,45	9,9	9	11	0,01	0,00	32,10	0,03	1,51	5,54
5130	98	1	Ciamis	200-500	Mahoni	Swietenia macrophylla King	Meliaceae	0,61	8,3	4	6	0,01	0,00	17,64	0,02	0,83	3,04
5131	98	1	Ciamis	200-500	Mahoni	Swietenia macrophylla King	Meliaceae	0,61	7,0	6	7,5	0,01	0,00	15,94	0,02	0,75	2,75
5132	98	1	Ciamis	200-500	Mahoni	Swietenia macrophylla King	Meliaceae	0,61	6,4	4	5,5	0,01	0,00	10,07	0,01	0,47	1,74
5133	98	1	Ciamis	200-500	Sengon	Paraserianthes falcataria (L.) I.C.	Leguminosae / Fabaceae	0,33	13,0	10	12	0,01	0,00	43,67	0,04	2,05	7,53
5134	98	1	Ciamis	200-500	Mahoni	Swietenia macrophylla King	Meliaceae	0,61	6,4	5	6,5	0,01	0,00	11,74	0,01	0,55	2,02
5135	98	1	Ciamis	200-500	Mahoni	Swietenia macrophylla King	Meliaceae	0,61	6,0	6	7,5	0,01	0,00	12,18	0,01	0,57	2,10
5136	98	1	Ciamis	200-500	Mahoni	Swietenia macrophylla King	Meliaceae	0,61	8,0	5	6,5	0,01	0,00	17,67	0,02	0,83	3,05
5137	98	1	Ciamis	200-500	Mahoni	Swietenia macrophylla King	Meliaceae	0,61	6,7	5	6	0,01	0,00	11,93	0,01	0,56	2,06
5138	98	1	Ciamis	200-500	Mahoni	Swietenia macrophylla King	Meliaceae	0,61	7,0	4,5	6,5	0,01	0,00	13,98	0,01	0,66	2,41
5139	99	2	Ciamis	200-500	Meranti	Shorea sp	Dipterocarpaceae	0,57	4,8	3	4	0,04	0,00	4,17	0,00	0,05	0,18
5140	99	2	Ciamis	200-500	Mahoni	Swietenia macrophylla King	Meliaceae	0,61	16,2	3	9	0,04	0,02	87,87	0,09	1,03	3,79
5141	99	2	Ciamis	200-500	Sengon	Paraserianthes falcataria (L.) I.C.	Leguminosae / Fabaceae	0,33	17,2	12	16	0,04	0,02	94,15	0,09	1,11	4,06
5142	99	2	Ciamis	200-500	Ganitri	Elaeocarpus ganitrus Roxb. ex G.	Elaeocarpaceae	0,49	11,8	6	11	0,04	0,01	48,00	0,05	0,56	2,07
5143	99	2	Ciamis	200-500	Petai	Parkia speciosa Hassk.	Leguminosae / Fabaceae	0,45	10,8	3,5	7,5	0,04	0,01	26,77	0,03	0,31	1,15
5144	99	2	Ciamis	200-500	Cempaka	Magnolia champaca var. champa	Magnoliaceae	0,51	4,8	2	3,5	0,04	0,00	3,34	0,00	0,04	0,14
5145	99	2	Ciamis	200-500	Manglid	Magnolia champaca (L.) Baill. ex	Magnoliaceae	0,53	18,5	10	15	0,04	0,03	156,12	0,16	1,83	6,73
5146	99	2	Ciamis	200-500	Sengon	Paraserianthes falcataria (L.) I.C.	Leguminosae / Fabaceae	0,33	12,7	10	13	0,04	0,01	44,92	0,04	0,53	1,94
5147	99	2	Ciamis	200-500	Tisuk	Hibiscus macrophyllus Roxb. ex I	Malvaceae	0,4	4,8	4	6	0,04	0,00	4,38	0,00	0,05	0,19
5148	99	2	Ciamis	200-500	Mahoni	Swietenia macrophylla King	Meliaceae	0,61	7,0	3	7	0,04	0,00	14,96	0,01	0,18	0,65
5149	99	2	Ciamis	200-500	Sengon	Paraserianthes falcataria (L.) I.C.	Leguminosae / Fabaceae	0,33	13,7	11	13	0,04	0,01	51,28	0,05	0,60	2,21
5150	99	2	Ciamis	200-500	Sengon	Paraserianthes falcataria (L.) I.C.	Leguminosae / Fabaceae	0,33	9,2	9	11	0,04	0,01	21,38	0,02	0,25	0,92
5151	99	2	Ciamis	200-500	Mahoni	Swietenia macrophylla King	Meliaceae	0,61	9,5	3	7	0,04	0,01	26,40	0,03	0,31	1,14
5152	99	2	Ciamis	200-500	Kelapa	Cocos nucifera L.	Arecaceae	0,5	26,7	13	16	0,04	0,06	127,70	0,13	1,50	5,51
5153	99	2	Ciamis	200-500	Mahoni	Swietenia macrophylla King	Meliaceae	0,61	7,6	3	7	0,04	0,00	17,54	0,02	0,21	0,76
5154	99	2	Ciamis	200-500	Mahoni	Swietenia macrophylla King	Meliaceae	0,61	6,4	3	6	0,04	0,00	10,91	0,01	0,13	0,47
5155	99	2	Ciamis	200-500	Sengon	Paraserianthes falcataria (L.) I.C.	Leguminosae / Fabaceae	0,33	8,9	10	11,5	0,04	0,01	20,89	0,02	0,25	0,90
5156	99	2	Ciamis	200-500	Sengon	Paraserianthes falcataria (L.) I.C.	Leguminosae / Fabaceae	0,33	10,2	9	10,5	0,04	0,01	24,54	0,02	0,29	1,06
5157	99	2	Ciamis	200-500	Tisuk	Hibiscus macrophyllus Roxb. ex I	Malvaceae	0,4	5,4	3,5	7,5	0,04	0,00	6,75	0,01	0,08	0,29
5158	99	2	Ciamis	200-500	Sengon	Paraserianthes falcataria (L.) I.C.	Leguminosae / Fabaceae	0,33	8,9	8	11	0,04	0,01	20,05	0,02	0,24	0,86
5159	99	2	Ciamis	200-500	Sengon	Paraserianthes falcataria (L.) I.C.	Leguminosae / Fabaceae	0,33	10,2	9	11	0,04	0,01	25,61	0,03	0,30	1,10
5160	99	2	Ciamis	200-500	Ganitri	Elaeocarpus ganitrus Roxb. ex G.	Elaeocarpaceae	0,49	2,5	2	3	0,04	0,00	0,88	0,00	0,01	0,04
5161	99	2	Ciamis	200-500	Heuras	Vitex pubescens Vahl	Lamiaceae / Verbenaceae	0,88	5,1	2,5	5,5	0,04	0,00	9,36	0,01	0,11	0,40
5162	99	2	Ciamis	200-500	Manglid	Magnolia champaca (L.) Baill. ex	Magnoliaceae	0,53	10,5	7	9	0,04	0,01	34,80	0,03	0,41	1,50
5163	99	2	Ciamis	200-500	Akasia mangium	Acacia mangium Willd.	Leguminosae / Fabaceae	0,5	12,7	5	10	0,04	0,01	51,68	0,05	0,61	2,23
5164	99	2	Ciamis	200-500	Akasia mangium	Acacia mangium Willd.	Leguminosae / Fabaceae	0,5	9,5	6	9	0,04	0,01	27,70	0,03	0,33	1,19
5165	99	2	Ciamis	200-500	Rukem	Flacourtia rukam Zoll. & Moritzi	Salicaceae	0,75	6,4	2	6	0,04	0,00	13,18	0,01	0,15	0,57
5166	99	2	Ciamis	200-500	Tisuk	Hibiscus macrophyllus Roxb. ex I	Malvaceae	0,4	7,3	3	3,5	0,04	0,00	5,84	0,01	0,07	0,25
5167	99	2	Ciamis	200-500	Heuras	Vitex pubescens Vahl	Lamiaceae / Verbenaceae	0,88	21,0	3	10	0,04	0,03	217,11	0,22	2,55	9,36
5168	99	2	Ciamis	200-500	Mahoni	Swietenia macrophylla King	Meliaceae	0,61	7,3	3	7	0,04	0,00	16,23	0,02	0,19	0,70
5169	99	2	Ciamis	200-500	Ganitri	Elaeocarpus ganitrus Roxb. ex G.	Elaeocarpaceae	0,49	14,3	8	12	0,04	0,02	74,40	0,07	0,87	3,21
5170	99	2	Ciamis	200-500	Ganitri	Elaeocarpus ganitrus Roxb. ex G.	Elaeocarpaceae	0,49	12,1	8	12	0,04	0,01	54,58	0,05	0,64	2,35
5171	99	2	Ciamis	200-500	Sengon	Paraserianthes falcataria (L.) I.C.	Leguminosae / Fabaceae	0,33	9,2	9	11	0,04	0,01	21,38	0,02	0,25	0,92
5172	99	2	Ciamis	200-500	Sengon	Paraserianthes falcataria (L.) I.C.	Leguminosae / Fabaceae	0,33	8,3	8	10	0,04	0,01	16,04	0,02	0,19	0,69
5173	99	2	Ciamis	200-500	Tisuk	Hibiscus macrophyllus Roxb. ex I	Malvaceae	0,4	3,2	1,5	2,5	0,04	0,00	0,93	0,00	0,01	0,04
5174	99	2	Ciamis	200-500	Kemiri	Aleurites moluccanus (L.) Willd.	Euphorbiaceae	0,31	21,6	4	13	0,04	0,04	104,60	0,10	1,23	4,51
5175	99	2	Ciamis	200-500	Sengon	Paraserianthes falcataria (L.) I.C.	Leguminosae / Fabaceae	0,33	6,4	9	11	0,04	0,00	10,83	0,01	0,13	0,47
5176	99	2	Ciamis	200-500	Sengon	Paraserianthes falcataria (L.) I.C.	Leguminosae / Fabaceae	0,33	13,0	10	12	0,04	0,01	43,67	0,04	0,51	1,88
5177	99	2	Ciamis	200-500	Sengon	Paraserianthes falcataria (L.) I.C.	Leguminosae / Fabaceae	0,33	14,3	10	12	0,04	0,02	51,80	0,05	0,61	2,23
5178	99	2	Ciamis	200-500	Akasia mangium	Acacia mangium Willd.	Leguminosae / Fabaceae	0,5	16,5	5	11	0,04	0,02	91,20	0,09	1,07	3,93
5179	99	2	Ciamis	200-500	Sengon	Paraserianthes falcataria (L.) I.C.	Leguminosae / Fabaceae	0,33	15,3	9	11	0,04	0,02	53,83	0,05	0,63	2,32
5180	99	2	Ciamis	200-500	Tisuk	Hibiscus macrophyllus Roxb. ex I	Malvaceae	0,4	8,6	7	8	0,04	0,01	16,71	0,02	0,20	0,72
5181	99	2	Ciamis	200-500	Tisuk	Hibiscus macrophyllus Roxb. ex I	Malvaceae	0,4	8,9	4	6	0,04	0,01	13,73	0,01	0,16	0,59
5182	99	2	Ciamis	200-500	Gmelina	Gmelina arborea Roxb.	Lamiaceae / Verbenaceae	0,47	13,0	7	10	0,04	0,01	51,10	0,05	0,60	2,20
5183	99	2	Ciamis	200-500	Sengon	Paraserianthes falcataria (L.) I.C.	Leguminosae / Fabaceae	0,33	12,4	9	10,5	0,04	0,01	35,26	0,04	0,41	1,52
5184	99	2	Ciamis	200-500	Suren	Toona sureni (Blume) Merr.	Meliaceae	0,39	3,2	5	5,5	0,04	0,00	1,88	0,00	0,02	0,08
5185	99	2	Ciamis	200-500	Sengon	Paraserianthes falcataria (L.) I.C.	Leguminosae / Fabaceae	0,33	14,0	10	11,5	0,04	0,02	47,81	0,05	0,56	2,06
5186	99	2	Ciamis	200-500	Mahoni	Swietenia macrophylla King	Meliaceae	0,61	5,7	2	3	0,04	0,00	4,77	0,00	0,06	0,21
5187	99	2	Ciamis	200-500	Akasia mangium	Acacia mangium Willd.	Leguminosae / Fabaceae	0,5	17,2	6	7	0,04	0,02	64,60	0,06	0,76	2,79
5188	99	2	Ciamis	200-500	Sengon	Paraserianthes falcataria (L.) I.C.	Leguminosae / Fabaceae	0,33	11,1	12	14	0,04	0,01	37,64	0,04	0,44	1,62
5189	99	2	Ciamis	200-500	Mahoni	Swietenia macrophylla King	Meliaceae	0,61	8,6	2	7	0,04	0,01	21,77	0,02	0,26	0,94
5190	99	2	Ciamis	200-500	Mahoni	Swietenia macrophylla King	Meliaceae	0,61	8,0	2	8	0,04	0,00	21,37	0,02	0,25	0,92
5191	99	2	Ciamis	200-500	Mahoni	Swietenia macrophylla King	Meliaceae	0,61	3,8	3,5	4,5	0,04	0,00	3,29	0,00	0,04	0,14
5192	100	2	Ciamis	200-500	Mahoni	Swietenia macrophylla King	Meliaceae	0,61	4,5	2	3	0,04	0,00	3,01	0,00	0,04	0,13
5193	100	2	Ciamis	200-500	Mahoni	Swietenia macrophylla King	Meliaceae	0,61	15,6	3,5	7,5	0,04	0,02	69,10	0,07	0,81	2,98
5194	100	2	Ciamis	200-500	Mahoni	Swietenia macrophylla King	Meliaceae	0,61	31,5	7	13	0,04	0,08	414,82	0,41	4,87	17,89
5195	100	2	Ciamis	200-500	Mahoni	Swietenia macrophylla King	Meliaceae	0,61	13,4	7	10	0,04	0,01	67,81	0,07	0,80	2,92
5196	100	2	Ciamis	200-500	Tisuk	Hibiscus macrophyllus Roxb. ex I	Malvaceae	0,4	13,4	12	15	0,04	0,01	66,79	0,07	0,78	2,88
5197	100	2	Ciamis	200-500	Mahoni	Swietenia macrophylla King	Meliaceae	0,61	9,5	3	8	0,04	0,01	29,84	0,03	0,35	1,29
5198	100	2	Ciamis	200-500	Mahoni	Swietenia macrophylla King	Meliaceae	0,61	6,0	3	5	0,04	0,00	8,40	0,01	0,10	0,36
5199	100	2	Ciamis	200-500	Mahoni	Swietenia macrophylla King	Meliaceae	0,61	3,2	2	3	0,04	0,00	1,62	0,00	0,02	0,07
5200	100	2	Ciamis	200-500	Mahoni	Swietenia macrophylla King	Meliaceae	0,61	16,5	7	11	0,04	0,02	109,42	0,11	1,29	4,72
5201	100	2	Ciamis	200-500	Mahoni	Swietenia macrophylla King	Meliaceae	0,61	13,7	3	8	0,04	0,01	57,71	0,06	0,68	2,49
5202	100	2	Ciamis	200-500	Mahoni	Swietenia macrophylla King	Meliaceae	0,61	16,9	4	10	0,04	0,02	103,84	0,10	1,22	4,48

5203	100	2	Ciamis	200-500	Kelapa	Cocos nucifera L.	Arecaceae	0,5	25,8	11	14	0,04	0,05	112,30	0,11	1,32	4,84
5204	100	2	Ciamis	200-500	Jengkol	Archidendron pauciflorum (Bentl	Leguminosae / Fabaceae	0,4	2,9	2,5	3,5	0,04	0,00	1,05	0,00	0,01	0,05
5205	100	2	Ciamis	200-500	Mahoni	Swietenia macrophylla King	Meliaceae	0,61	16,2	6	10	0,04	0,02	96,77	0,10	1,14	4,17
5206	100	2	Ciamis	200-500	Mahoni	Swietenia macrophylla King	Meliaceae	0,61	13,4	5	8	0,04	0,01	55,27	0,06	0,65	2,38
5207	100	2	Ciamis	200-500	Mahoni	Swietenia macrophylla King	Meliaceae	0,61	18,8	6	10	0,04	0,03	126,38	0,13	1,48	5,45
5208	100	2	Ciamis	200-500	Kelapa	Cocos nucifera L.	Arecaceae	0,5	23,5	10	13	0,04	0,04	104,60	0,10	1,23	4,51
5209	100	2	Ciamis	200-500	Sengon	Paraserianthes falcataria (L.) I.C	Leguminosae / Fabaceae	0,33	9,9	10	12	0,04	0,01	26,17	0,03	0,31	1,13
5210	100	2	Ciamis	200-500	Sengon	Paraserianthes falcataria (L.) I.C	Leguminosae / Fabaceae	0,33	23,5	10	16	0,04	0,04	167,68	0,17	1,97	7,23
5211	100	2	Ciamis	200-500	Tisuk	Hibiscus macrophyllus Roxb. ex I	Malvaceae	0,4	8,9	8	14	0,04	0,01	29,83	0,03	0,35	1,29
5212	100	2	Ciamis	200-500	Sengon	Paraserianthes falcataria (L.) I.C	Leguminosae / Fabaceae	0,33	26,1	10	18	0,04	0,05	225,43	0,23	2,65	9,72
5213	100	2	Ciamis	200-500	Sengon	Paraserianthes falcataria (L.) I.C	Leguminosae / Fabaceae	0,33	10,2	10	13	0,04	0,01	29,85	0,03	0,35	1,29
5214	100	2	Ciamis	200-500	Mahoni	Swietenia macrophylla King	Meliaceae	0,61	9,2	3,5	7,5	0,04	0,01	26,43	0,03	0,31	1,14
5215	100	2	Ciamis	200-500	Tisuk	Hibiscus macrophyllus Roxb. ex I	Malvaceae	0,4	10,5	11	15	0,04	0,01	42,94	0,04	0,50	1,85
5216	100	2	Ciamis	200-500	Mahoni	Swietenia macrophylla King	Meliaceae	0,61	5,7	3	7	0,04	0,00	10,36	0,01	0,12	0,45
5217	100	2	Ciamis	200-500	Sengon	Paraserianthes falcataria (L.) I.C	Leguminosae / Fabaceae	0,33	13,0	12	16	0,04	0,01	56,84	0,06	0,67	2,45
5218	100	2	Ciamis	200-500	Tisuk	Hibiscus macrophyllus Roxb. ex I	Malvaceae	0,4	7,3	6	9	0,04	0,00	13,88	0,01	0,16	0,60
5219	100	2	Ciamis	200-500	Sengon	Paraserianthes falcataria (L.) I.C	Leguminosae / Fabaceae	0,33	25,5	13	19	0,04	0,05	226,40	0,23	2,66	9,76
5220	100	2	Ciamis	200-500	Sengon	Paraserianthes falcataria (L.) I.C	Leguminosae / Fabaceae	0,33	8,3	9	12	0,04	0,01	18,96	0,02	0,22	0,82
5221	100	2	Ciamis	200-500	Sengon	Paraserianthes falcataria (L.) I.C	Leguminosae / Fabaceae	0,33	17,5	12	19	0,04	0,02	113,96	0,11	1,34	4,91
5222	100	2	Ciamis	200-500	Kihampelas	Ficus ampelas Burm.f.	Moraceae	0,38	3,2	1,5	4,5	0,04	0,00	1,53	0,00	0,02	0,07
5223	100	2	Ciamis	200-500	Sengon	Paraserianthes falcataria (L.) I.C	Leguminosae / Fabaceae	0,33	16,5	11	17	0,04	0,02	92,87	0,09	1,09	4,00
5224	100	2	Ciamis	200-500	Tisuk	Hibiscus macrophyllus Roxb. ex I	Malvaceae	0,4	10,2	9	13	0,04	0,01	35,60	0,04	0,42	1,54
5225	100	2	Ciamis	200-500	Sengon	Paraserianthes falcataria (L.) I.C	Leguminosae / Fabaceae	0,33	19,7	13	19	0,04	0,03	141,93	0,14	1,67	6,12
5226	100	2	Ciamis	200-500	Tisuk	Hibiscus macrophyllus Roxb. ex I	Malvaceae	0,4	12,4	12	15	0,04	0,01	58,31	0,06	0,69	2,51
5227	100	2	Ciamis	200-500	Kiciat	Ficus septica Burm.f.	Moraceae	0,42	13,4	7	10	0,04	0,01	48,17	0,05	0,57	2,08
5228	100	2	Ciamis	200-500	Hantap	Sterculia oblongata R.Br.	Malvaceae	0,28	3,2	3	4	0,04	0,00	1,04	0,00	0,01	0,04
5229	100	2	Ciamis	200-500	Tisuk	Hibiscus macrophyllus Roxb. ex I	Malvaceae	0,4	9,2	4	6	0,04	0,01	14,64	0,01	0,17	0,63
5230	100	2	Ciamis	200-500	Sengon	Paraserianthes falcataria (L.) I.C	Leguminosae / Fabaceae	0,33	8,6	4	9	0,04	0,01	15,61	0,02	0,18	0,67
5231	100	2	Ciamis	200-500	Sengon	Paraserianthes falcataria (L.) I.C	Leguminosae / Fabaceae	0,33	31,5	10	18	0,04	0,08	318,35	0,32	3,74	13,73
5232	100	2	Ciamis	200-500	Sengon	Paraserianthes falcataria (L.) I.C	Leguminosae / Fabaceae	0,33	9,9	9	13	0,04	0,01	28,16	0,03	0,33	1,21
5233	100	2	Ciamis	200-500	Dukuh	Lansium domesticum Corrêa	Meliaceae	0,85	3,5	3	4	0,04	0,00	3,41	0,00	0,04	0,15
5234	100	2	Ciamis	200-500	Tisuk	Hibiscus macrophyllus Roxb. ex I	Malvaceae	0,4	7,0	6	8	0,04	0,00	11,49	0,01	0,13	0,50
5235	100	2	Ciamis	200-500	Sengon	Paraserianthes falcataria (L.) I.C	Leguminosae / Fabaceae	0,33	13,7	9	13	0,04	0,01	51,28	0,05	0,60	2,21
5236	100	2	Ciamis	200-500	Sengon	Paraserianthes falcataria (L.) I.C	Leguminosae / Fabaceae	0,33	24,8	14	18	0,04	0,05	205,70	0,21	2,42	8,87
5237	100	2	Ciamis	200-500	Kiciat	Ficus septica Burm.f.	Moraceae	0,42	4,8	4	5	0,04	0,00	3,87	0,00	0,05	0,17
5238	100	2	Ciamis	200-500	Sengon	Paraserianthes falcataria (L.) I.C	Leguminosae / Fabaceae	0,33	8,9	9	10	0,04	0,01	18,38	0,02	0,22	0,79
5239	100	2	Ciamis	200-500	Sengon	Paraserianthes falcataria (L.) I.C	Leguminosae / Fabaceae	0,33	15,3	10	14	0,04	0,02	67,14	0,07	0,79	2,90
5240	100	2	Ciamis	200-500	Pulai	Alstonia scholaris (L.) R. Br.	Apocynaceae	0,3	4,8	3	4	0,04	0,00	2,32	0,00	0,03	0,10
5241	100	2	Ciamis	200-500	Mahoni	Swietenia macrophylla King	Meliaceae	0,61	4,8	3,5	4,5	0,04	0,00	4,95	0,00	0,06	0,21
5242	100	2	Ciamis	200-500	Sengon	Paraserianthes falcataria (L.) I.C	Leguminosae / Fabaceae	0,33	12,1	10	13	0,04	0,01	40,89	0,04	0,48	1,76
5243	100	2	Ciamis	200-500	Sengon	Paraserianthes falcataria (L.) I.C	Leguminosae / Fabaceae	0,33	7,3	7	10	0,04	0,00	12,82	0,01	0,15	0,55
5244	100	2	Ciamis	200-500	Dukuh	Lansium domesticum Corrêa	Meliaceae	0,85	6,7	1,5	5,5	0,04	0,00	14,93	0,01	0,18	0,64
5245	100	2	Ciamis	200-500	Tisuk	Hibiscus macrophyllus Roxb. ex I	Malvaceae	0,4	15,3	9	15	0,04	0,02	85,30	0,09	1,00	3,68
5246	100	2	Ciamis	200-500	Sengon	Paraserianthes falcataria (L.) I.C	Leguminosae / Fabaceae	0,33	14,0	11	14	0,04	0,02	57,24	0,06	0,67	2,47
5247	100	2	Ciamis	200-500	Kelapa	Cocos nucifera L.	Arecaceae	0,5	27,0	16	19	0,04	0,06	150,80	0,15	1,77	6,50
5248	100	2	Ciamis	200-500	Mahoni	Swietenia macrophylla King	Meliaceae	0,61	7,3	2,5	5,5	0,04	0,00	13,01	0,01	0,15	0,56
5249	100	2	Ciamis	200-500	Mahoni	Swietenia macrophylla King	Meliaceae	0,61	8,0	2	6	0,04	0,00	16,42	0,02	0,19	0,71
5250	100	2	Ciamis	200-500	Kelapa	Cocos nucifera L.	Arecaceae	0,5	25,5	12	15	0,04	0,05	120,00	0,12	1,41	5,17
5251	101	1	Sukamantri	>500	Ganitri	Elaeocarpus ganitrus Roxb. ex G.	Elaeocarpaceae	0,49	11,1	9	14	0,04	0,01	54,07	0,05	0,64	2,33
5252	101	1	Sukamantri	>500	Ganitri	Elaeocarpus ganitrus Roxb. ex G.	Elaeocarpaceae	0,49	9,5	7	10	0,04	0,01	29,95	0,03	0,35	1,29
5253	101	1	Sukamantri	>500	Ganitri	Elaeocarpus ganitrus Roxb. ex G.	Elaeocarpaceae	0,49	15,6	11	13	0,04	0,02	93,57	0,09	1,10	4,04
5254	101	1	Sukamantri	>500	Afrika	Maesopsis eminii Engl.	Rhamnaceae	0,43	5,7	1	4	0,04	0,00	4,50	0,00	0,05	0,19
5255	101	1	Sukamantri	>500	Ganitri	Elaeocarpus ganitrus Roxb. ex G.	Elaeocarpaceae	0,49	7,3	7	9	0,04	0,00	16,72	0,02	0,20	0,72
5256	101	1	Sukamantri	>500	Ganitri	Elaeocarpus ganitrus Roxb. ex G.	Elaeocarpaceae	0,49	10,8	9	11	0,04	0,01	41,11	0,04	0,48	1,77
5257	101	1	Sukamantri	>500	Ganitri	Elaeocarpus ganitrus Roxb. ex G.	Elaeocarpaceae	0,49	8,6	5	7	0,04	0,01	17,81	0,02	0,21	0,77
5258	101	1	Sukamantri	>500	Ganitri	Elaeocarpus ganitrus Roxb. ex G.	Elaeocarpaceae	0,49	6,7	4	6	0,04	0,00	9,76	0,01	0,11	0,42
5259	101	1	Sukamantri	>500	Ganitri	Elaeocarpus ganitrus Roxb. ex G.	Elaeocarpaceae	0,49	6,7	4	6	0,04	0,00	9,76	0,01	0,11	0,42
5260	101	1	Sukamantri	>500	Ganitri	Elaeocarpus ganitrus Roxb. ex G.	Elaeocarpaceae	0,49	17,2	12	11	0,04	0,02	95,94	0,10	1,13	4,14
5261	101	1	Sukamantri	>500	Afrika	Maesopsis eminii Engl.	Rhamnaceae	0,43	9,5	8	9	0,04	0,01	24,13	0,02	0,28	1,04
5262	101	1	Sukamantri	>500	Ganitri	Elaeocarpus ganitrus Roxb. ex G.	Elaeocarpaceae	0,49	11,8	10	12	0,04	0,01	51,98	0,05	0,61	2,24
5263	101	1	Sukamantri	>500	Ganitri	Elaeocarpus ganitrus Roxb. ex G.	Elaeocarpaceae	0,49	10,2	9	12	0,04	0,01	39,84	0,04	0,47	1,72
5264	101	1	Sukamantri	>500	Ganitri	Elaeocarpus ganitrus Roxb. ex G.	Elaeocarpaceae	0,49	14,0	12	14	0,04	0,02	82,22	0,08	0,97	3,55
5265	101	1	Sukamantri	>500	Ganitri	Elaeocarpus ganitrus Roxb. ex G.	Elaeocarpaceae	0,49	18,1	12	14	0,04	0,03	132,12	0,13	1,55	5,70
5266	101	1	Sukamantri	>500	Afrika	Maesopsis eminii Engl.	Rhamnaceae	0,43	10,8	8	9,5	0,04	0,01	31,89	0,03	0,37	1,38
5267	101	1	Sukamantri	>500	Afrika	Maesopsis eminii Engl.	Rhamnaceae	0,43	9,9	7	9	0,04	0,01	25,62	0,03	0,30	1,10
5268	101	1	Sukamantri	>500	Ganitri	Elaeocarpus ganitrus Roxb. ex G.	Elaeocarpaceae	0,49	10,2	9	11	0,04	0,01	36,79	0,04	0,43	1,59
5269	101	1	Sukamantri	>500	Ganitri	Elaeocarpus ganitrus Roxb. ex G.	Elaeocarpaceae	0,49	8,0	9	10	0,04	0,00	21,45	0,02	0,25	0,92
5270	101	1	Sukamantri	>500	Ganitri	Elaeocarpus ganitrus Roxb. ex G.	Elaeocarpaceae	0,49	14,0	12	13,5	0,04	0,02	79,53	0,08	0,93	3,43
5271	101	1	Sukamantri	>500	Ganitri	Elaeocarpus ganitrus Roxb. ex G.	Elaeocarpaceae	0,49	14,3	12	14	0,04	0,02	85,68	0,09	1,01	3,69
5272	101	1	Sukamantri	>500	Ganitri	Elaeocarpus ganitrus Roxb. ex G.	Elaeocarpaceae	0,49	11,5	6	10	0,04	0,01	41,83	0,04	0,49	1,80
5273	101	1	Sukamantri	>500	Ganitri	Elaeocarpus ganitrus Roxb. ex G.	Elaeocarpaceae	0,49	15,0	114	13	0,04	0,02	86,70	0,09	1,02	3,74
5274	101	1	Sukamantri	>500	Ganitri	Elaeocarpus ganitrus Roxb. ex G.	Elaeocarpaceae	0,49	18,8	12	14	0,04	0,03	140,73	0,14	1,65	6,07
5275	101	1	Sukamantri	>500	Afrika	Maesopsis eminii Engl.	Rhamnaceae	0,43	9,9	6	9	0,04	0,01	25,62	0,03	0,30	1,10
5276	101	1	Sukamantri	>500	Ganitri	Elaeocarpus ganitrus Roxb. ex G.	Elaeocarpaceae	0,49	18,5	13	12	0,04	0,03	118,43	0,12	1,39	5,11
5277	101	1	Sukamantri	>500	Ganitri	Elaeocarpus ganitrus Roxb. ex G.	Elaeocarpaceae	0,49	15,3	13	15	0,04	0,02	102,72	0,10	1,21	4,43
5278	101	1	Sukamantri	>500	Ganitri	Elaeocarpus ganitrus Roxb. ex G.	Elaeocarpaceae	0,49	3,5	4	13	0,04	0,00	6,06	0,01	0,07	0,26
5279	101	1	Sukamantri	>500	Ganitri	Elaeocarpus ganitrus Roxb. ex G.	Elaeocarpaceae	0,49	13,4	10	12	0,04	0,01	65,56	0,07	0,77	2,83
5280	101	1	Sukamantri	>500	Ganitri	Elaeocarpus ganitrus Roxb. ex G.	Elaeocarpaceae	0,49	7,6	5	6,5	0,04	0,00	13,41	0,01	0,16	0,58
5281	101	1	Sukamantri	>500	Ganitri	Elaeocarpus ganitrus Roxb. ex G.	Elaeocarpaceae	0,49	15,9	11	12,5	0,04	0,02	93,67	0,09	1,10	4,04
5282	101	1	Sukamantri	>500	Ganitri	Elaeocarpus ganitrus Roxb. ex G.	Elaeocarpaceae	0,49	13,0	11	12,5	0,04	0,01	65,12	0,07	0,77	2,81
5283	101	1	Sukamantri	>500	Ganitri	Elaeocarpus ganitrus Roxb. ex G.	Elaeocarpaceae	0,49	17,5	9	12	0,04	0,02	107,45	0,11	1,26	4,63
5284	101	1	Sukamantri	>500	Ganitri	Elaeocarpus ganitrus Roxb. ex G.	Elaeocarpaceae	0,49	17,8	11	13	0,04	0,02	119,51	0,12	1,40	5,15
5285	101	1	Sukamantri	>500	Ganitri	Elaeocarpus ganitrus Roxb. ex G.	Elaeocarpaceae	0,49	9,2	12	13	0,04	0,01	35,80	0,04	0,42	1,54
5286	101	1	Sukamantri	>500	Ganitri	Elaeocarpus ganitrus Roxb. ex G.	Elaeocarpaceae	0,49	14,0	11	13	0,04	0,02	76,83	0,08	0,90	3,31
5287	101	1	Sukamantri	>500	Ganitri	Elaeocarpus ganitrus Roxb. ex G.	Elaeocarpaceae	0,49	14,6	4	5	0,04	0,02	34,74	0,03	0,41	1,50
5288	101	1	Sukamantri	>500	Ganitri	Elaeocarpus ganitrus Roxb. ex G.	Elaeocarpaceae	0,49	13,4	11	13	0,04	0,01	70,55	0,07	0,83	3,04
5289	101	1	Sukamantri	>500	Ganitri	Elaeocarpus ganitrus Roxb. ex G.	Elaeocarpaceae	0,49	2,9	1	3	0,04	0,00	1,10	0,00	0,01	0,05
5290	101	1	Sukamantri	>500	Ganitri	Elaeocarpus ganitrus Roxb. ex G.	Elaeocarpaceae	0,49	3,5	1,5	3	0,04	0,00	1,58	0,00	0,02	0,07
5291	101	1	Sukamantri	>500	Ganitri	Elaeocarpus ganitrus Roxb. ex G.	Elaeocarpaceae	0,49	4,5	2	3,5	0,04	0,00	2,83	0,00	0,03	0,12
5292	101	1	Sukamantri	>500	Afrika	Maesopsis eminii Engl.	Rhamnaceae	0,43	5,7	3	4,5	0,04	0,00	5,02	0,01	0,06	0,22
5293	101	1	Sukamantri	>500	Ganitri	Elaeocarpus ganitrus Roxb. ex G.	Elaeocarpaceae	0,49	15,9	12	14	0,04	0,02	103,92	0,10	1,22	4,48
5294	101	1	Sukamantri	>500	Ganitri	Elaeocarpus ganitrus Roxb. ex G.	Elaeocarpaceae	0,49	9,9	7	8,5	0,04	0,01	27,41	0,03	0,32	1,18
5295	101	1	Sukamantri	>500	Sp8	Sp8	0	0,5	28,6	6	11	0,04	0,06	249,15	0,25	2,93	10,74
5296	101	1	Sukamantri	>500	Ganitri	Elaeocarpus ganitrus Roxb. ex G.	Elaeocarpaceae	0,49	15,9	11	13	0,04	0,02	97,10	0,10	1,14	4,19
5297	101	1	Sukamantri	>500	Petai	Parkia speciosa Hassk.	Leguminosae / Fabaceae	0,45	11,5	6	9	0,04	0,01	35,13	0,04	0,41	1,51
5298	101	1	Sukamantri	>500	Ganitri	Elaeocarpus ganitrus Roxb. ex G.	Elaeocarpaceae	0,49	11,5	9	12	0,04	0,01	49,43	0,05	0,58	2,13
5299	101	1	Sukamantri	>500	Ganitri	Elaeocarpus ganitrus Roxb. ex G.	Elaeocarpaceae	0,49	12,1	10	11	0,04	0,01	50,40	0,05	0,59	2,17
5300	101	1	Sukamantri	>500	Ganitri	Elaeocarpus ganitrus Roxb. ex G.	Elaeocarpaceae	0,49	13,4	11	12	0,04	0,01	65,56	0,07	0,77	2,83
5301	101	1	Sukamantri	>500	Mahoni afrika	Khaya anthotheca (Welw.) C.DC.	Meliaceae	0,49	6,4	3	6	0,04	0,00	8,93	0,01	0,10	0,38
5302	101	1	Sukamantri	>500	Ganitri	Elaeocarpus ganitrus Roxb. ex G.	Elaeocarpaceae	0,49	14,0	10	12	0,04	0,02	71,40	0,07	0,84	3,08
5303	101	1	Sukamantri	>500	Ganitri	Elaeocarpus ganitrus Roxb. ex G.	Elaeocarpaceae	0,49	14,3	11	12,5	0,04	0,02	77,23	0,08	0,91	3,33
5304	101	1	Sukamantri	>500	Ganitri	Elaeocarpus ganitrus Roxb. ex G.	Elaeocarpaceae	0,49	7,6	3	5	0,04	0,00	10,55	0,01	0,12	0,45
5305	101	1	Sukamantri	>500	Ganitri	Elaeocarpus ganitrus Roxb. ex G.	Elaeocarpaceae	0,49	12,7	10	11	0,04	0,01	55,36	0,06	0,65	2,39
5306	101	1	Sukamantri	>500	Ganitri	Elaeocarpus ganitrus Roxb. ex G.	Elaeocarpaceae	0,49	15,0	11	12	0,04	0,02	80,57	0,08	0,95	3,47
5307	101	1	Sukamantri	>500	Ganitri	Elaeocarpus ganitrus Roxb. ex G.	Elaeocarpaceae	0,49	7,0	5	7	0,04	0,00	12,24	0,01	0,14	0,53
5308	101	1	Sukamantri	>500	Ganitri	Elaeocarpus ganitrus Roxb. ex G.	Elaeocarpaceae	0,49	15,0	6	9	0,04	0,02	61,90	0,06	0,73	2,67
5309	101	1	Sukamantri	>500	Ganitri	Elaeocarpus ganitrus Roxb. ex G.	Elaeocarpaceae	0,49	9,5	6	9	0,04	0,01	27,20	0,03	0,32	1,17
5310	102	3	Sukamantri	>500	Afrika	Maesopsis eminii Engl.	Rhamnaceae	0,43	15,9	9	11	0,04	0,02	73,93	0,07	0,87	3,19
5311	102	3	Sukamantri	>500	Afrika	Maesopsis eminii Engl.	Rhamnaceae	0,43	9,9	6	7	0,04	0,01	20,35	0,02	0,24	0,88
5312	102	3	Sukamantri	>500	Afrika	Maesopsis eminii Engl.	Rhamnaceae	0,43	16,9	7	8	0,04	0,02	61,44	0,06	0,72	2,65
5313	102	3	Sukamantri	>500	Puspa	Schima wallichii Choisy	Theaceae	0,74	13,7	3	8	0,04	0,01	68,88	0,07	0,81	2,97
5314	102	3	Sukamantri	>500	Sengon	Paraserianthes falcataria (L.) I.C	Leguminosae / Fabaceae	0,33	11,8	7	9	0,04	0,01	27,80	0,03	0,33	1,20
5315	102	3	Sukamantri	>500	Sengon	Paraserianthes falcataria (L.) I.C	Leguminosae / Fabaceae	0,33	11,8	7	8	0,04	0,01	24,96	0,02	0,29	1,08
5316	102	3	Sukamantri	>500	Sengon	Paraserianthes falcataria (L.) I.C	Leguminosae / Fabaceae	0,33	3,5	7	8	0,04	0,00	2,70	0,00	0,03	0,12
5317	102	3	Sukamantri	>500	Puspa	Schima wallichii Choisy	Theaceae	0,74	7,3	2	5	0,04	0,00	14,23	0,01	0,17	0,61
5318	102	3	Sukamantri	>500	Sengon	Paraserianthes falcataria (L.) I.C	Leguminosae / Fabaceae	0,33	11,5	7	8	0,04	0,01	23,74	0,02	0,28	1,02
5319	102	3	Sukamantri	>500	Sengon	Paraserianthes falcataria (L.) I.C	Leguminosae / Fabaceae	0,33	6,7	7	8	0,04	0,00	8,84	0,01	0,10	0,38
5320	102	3	Sukamantri	>500	Sengon	Paraserianthes falcataria (L.) I.C	Leguminosae / Fabaceae	0,33	7,3	8	9	0,04	0,00	11,64	0,01	0,14	0,50
5321	102	3	Sukamantri	>500	Sengon	Paraserianthes falcataria (L.) I.C	Leguminosae / Fabaceae	0,33	3,8	5	6	0,04	0,00	2,44	0,00	0,03	0,11
5322	102	3	Sukamantri	>500	Sengon	Paraserianthes falcataria (L.) I.C	Leguminosae / Fabaceae	0,33	3,5	5	5,5	0,04	0,00	1,92	0,00	0,02	0,08
5323	102	3	Sukamantri	>500	Sengon	Paraserianthes falcataria (L.) I.C	Leguminosae / Fabaceae	0,33	3,5	4	5	0,04	0,00	1,76	0,00	0,02	0,08
5324	102	3	Sukamantri	>500	Sengon	Paraserianthes falcataria (L.) I.C	Leguminosae / Fabaceae	0,33	4,8	5	6	0,04	0,00	3,67	0,00	0,04	0,16
5325	102	3	Sukamantri	>500	Kihuut	Aporosa fructescens Blume	Phyllanthaceae	0,37	4,5	2	3	0,04	0,00	1,90	0,00	0,02	0,08
5326	102	3	Sukamantri	>500	Sawo / sawo manila	Manilkara zapota (L.) P.Royen	Sapotaceae	0,81	3,5	2,5	3	0,04	0,00	2,51	0,00	0,03	0,11

5327	102	3	Sukamantri	>500	Huru keling	Litsea sp.	Lauraceae	0,425	9,5	6	9	0,04	0,01	23,87	0,02	0,28	1,03
5328	102	3	Sukamantri	>500	Afrika	Maesopsis eminii Engl.	Rhamnaceae	0,43	9,5	6	7	0,04	0,01	19,17	0,02	0,23	0,83
5329	102	3	Sukamantri	>500	Afrika	Maesopsis eminii Engl.	Rhamnaceae	0,43	4,1	4	5	0,04	0,00	3,04	0,00	0,04	0,13
5330	102	3	Sukamantri	>500	Sengon	Paraserianthes falcataria (L.) I.C.	Leguminosae / Fabaceae	0,33	13,4	8	10	0,04	0,01	38,63	0,04	0,45	1,67
5331	102	3	Sukamantri	>500	Sengon	Paraserianthes falcataria (L.) I.C.	Leguminosae / Fabaceae	0,33	4,8	6	7	0,04	0,00	4,22	0,00	0,05	0,18
5332	102	3	Sukamantri	>500	Gmelina	Gmelina arborea Roxb.	Lamiaceae / Verbenaceae	0,47	4,5	4	6	0,04	0,00	4,47	0,00	0,05	0,19
5333	102	3	Sukamantri	>500	Gmelina	Gmelina arborea Roxb.	Lamiaceae / Verbenaceae	0,47	4,5	3	4	0,04	0,00	3,08	0,00	0,04	0,13
5334	102	3	Sukamantri	>500	Sengon	Paraserianthes falcataria (L.) I.C.	Leguminosae / Fabaceae	0,33	4,8	6	7	0,04	0,00	4,22	0,00	0,05	0,18
5335	102	3	Sukamantri	>500	Sengon	Paraserianthes falcataria (L.) I.C.	Leguminosae / Fabaceae	0,33	4,8	6	7	0,04	0,00	4,22	0,00	0,05	0,18
5336	102	3	Sukamantri	>500	Sawo / sawo manila	Manilkara zapota (L.) P.Royen	Sapotaceae	0,81	5,4	2	4	0,04	0,00	7,24	0,01	0,09	0,31
5337	102	3	Sukamantri	>500	Mahoni	Swietenia macrophylla King	Meliaceae	0,61	9,2	3	4	0,04	0,01	14,86	0,01	0,17	0,64
5338	102	3	Sukamantri	>500	Huru keling	Litsea sp.	Lauraceae	0,425	15,6	7	8,5	0,04	0,02	55,66	0,06	0,65	2,40
5339	102	3	Sukamantri	>500	Afrika	Maesopsis eminii Engl.	Rhamnaceae	0,43	12,7	7	8	0,04	0,01	36,69	0,04	0,43	1,58
5340	102	3	Sukamantri	>500	Mahoni	Swietenia macrophylla King	Meliaceae	0,61	8,0	2	3,5	0,04	0,00	10,02	0,01	0,12	0,43
5341	102	3	Sukamantri	>500	Huru keling	Litsea sp.	Lauraceae	0,425	9,2	6	7	0,04	0,01	17,82	0,02	0,21	0,77
5342	102	3	Sukamantri	>500	Huru keling	Litsea sp.	Lauraceae	0,425	12,1	5	7	0,04	0,01	29,24	0,03	0,34	1,26
5343	102	3	Sukamantri	>500	Puspa	Schima wallichii Choisy	Theaceae	0,74	13,4	4	7	0,04	0,01	58,38	0,06	0,69	2,52
5344	102	3	Sukamantri	>500	Sawo / sawo manila	Manilkara zapota (L.) P.Royen	Sapotaceae	0,81	6,7	3	5	0,04	0,00	13,09	0,01	0,15	0,56
5345	102	3	Sukamantri	>500	Huru keling	Litsea sp.	Lauraceae	0,425	15,3	6	8	0,04	0,02	50,70	0,05	0,60	2,19
5346	102	3	Sukamantri	>500	Afrika	Maesopsis eminii Engl.	Rhamnaceae	0,43	7,3	6	7,5	0,04	0,00	12,55	0,01	0,15	0,54
5347	102	3	Sukamantri	>500	Afrika	Maesopsis eminii Engl.	Rhamnaceae	0,43	18,5	8	9	0,04	0,03	80,74	0,08	0,95	3,48
5348	102	3	Sukamantri	>500	Puspa	Schima wallichii Choisy	Theaceae	0,74	13,7	6	8	0,04	0,01	68,88	0,07	0,81	2,97
5349	102	3	Sukamantri	>500	Afrika	Maesopsis eminii Engl.	Rhamnaceae	0,43	7,3	7	8	0,04	0,00	13,31	0,01	0,16	0,57
5350	102	3	Sukamantri	>500	Afrika	Maesopsis eminii Engl.	Rhamnaceae	0,43	7,6	7	8	0,04	0,00	14,39	0,01	0,17	0,62
5351	102	3	Sukamantri	>500	Afrika	Maesopsis eminii Engl.	Rhamnaceae	0,43	13,7	7	8	0,04	0,01	41,89	0,04	0,49	1,81
5352	102	3	Sukamantri	>500	Afrika	Maesopsis eminii Engl.	Rhamnaceae	0,43	7,6	7	8	0,04	0,00	14,39	0,01	0,17	0,62
5353	102	3	Sukamantri	>500	Afrika	Maesopsis eminii Engl.	Rhamnaceae	0,43	9,9	6	7	0,04	0,01	20,35	0,02	0,24	0,88
5354	102	3	Sukamantri	>500	Puspa	Schima wallichii Choisy	Theaceae	0,74	11,5	5	7	0,04	0,01	44,01	0,04	0,52	1,90
5355	102	3	Sukamantri	>500	Afrika	Maesopsis eminii Engl.	Rhamnaceae	0,43	8,9	5	6	0,04	0,01	14,67	0,01	0,17	0,63
5356	102	3	Sukamantri	>500	Mahoni	Swietenia macrophylla King	Meliaceae	0,61	8,6	2	4	0,04	0,01	13,04	0,01	0,15	0,56
5357	102	3	Sukamantri	>500	Afrika	Maesopsis eminii Engl.	Rhamnaceae	0,43	7,3	6	7	0,04	0,00	11,78	0,01	0,14	0,51
5358	102	3	Sukamantri	>500	Mahoni	Swietenia macrophylla King	Meliaceae	0,61	7,3	3	5	0,04	0,00	11,92	0,01	0,14	0,51
5359	102	3	Sukamantri	>500	Puspa	Schima wallichii Choisy	Theaceae	0,74	13,7	4	8	0,04	0,01	68,88	0,07	0,81	2,97
5360	102	3	Sukamantri	>500	Sengon	Paraserianthes falcataria (L.) I.C.	Leguminosae / Fabaceae	0,33	10,8	7	9	0,04	0,01	23,81	0,02	0,28	1,03
5361	102	3	Sukamantri	>500	Afrika	Maesopsis eminii Engl.	Rhamnaceae	0,43	7,6	6	7	0,04	0,00	12,74	0,01	0,15	0,55
5362	102	3	Sukamantri	>500	Nangka	Artocarpus heterophyllus Lam.	Moraceae	0,61	6,0	2,5	4,5	0,04	0,00	7,63	0,01	0,09	0,33
5363	102	3	Sukamantri	>500	Alpukat	Persea americana Mill.	Lauraceae	0,54	8,6	3	6	0,04	0,01	16,91	0,02	0,20	0,73
5364	102	3	Sukamantri	>500	Afrika	Maesopsis eminii Engl.	Rhamnaceae	0,43	17,8	9	10	0,04	0,02	83,38	0,08	0,98	3,60
5365	102	3	Sukamantri	>500	Alpukat	Persea americana Mill.	Lauraceae	0,54	4,8	5	6	0,04	0,00	5,76	0,01	0,07	0,25
5366	102	3	Sukamantri	>500	Afrika	Maesopsis eminii Engl.	Rhamnaceae	0,43	7,3	8	9	0,04	0,00	14,83	0,01	0,17	0,64
5367	102	3	Sukamantri	>500	Afrika	Maesopsis eminii Engl.	Rhamnaceae	0,43	4,5	6	6,5	0,04	0,00	4,43	0,00	0,05	0,19
5368	102	3	Sukamantri	>500	Afrika	Maesopsis eminii Engl.	Rhamnaceae	0,43	6,4	6	6,5	0,04	0,00	8,52	0,01	0,10	0,37
5369	102	3	Sukamantri	>500	Nangka	Artocarpus heterophyllus Lam.	Moraceae	0,61	6,4	2	4	0,04	0,00	7,52	0,01	0,09	0,32
5370	102	3	Sukamantri	>500	Afrika	Maesopsis eminii Engl.	Rhamnaceae	0,43	8,0	6	7	0,04	0,00	13,72	0,01	0,16	0,59
5371	102	3	Sukamantri	>500	Sengon	Paraserianthes falcataria (L.) I.C.	Leguminosae / Fabaceae	0,33	10,8	6	7	0,04	0,01	18,92	0,02	0,22	0,82
5372	102	3	Sukamantri	>500	Puspa	Schima wallichii Choisy	Theaceae	0,74	15,0	6	8	0,04	0,02	81,07	0,08	0,95	3,50
5373	102	3	Sukamantri	>500	Mahoni	Swietenia macrophylla King	Meliaceae	0,61	7,6	3	5	0,04	0,00	12,89	0,01	0,15	0,56
5374	102	3	Sukamantri	>500	Afrika	Maesopsis eminii Engl.	Rhamnaceae	0,43	7,3	7	7,5	0,04	0,00	12,55	0,01	0,15	0,54
5375	102	3	Sukamantri	>500	Mahoni	Swietenia macrophylla King	Meliaceae	0,61	8,6	2	4	0,04	0,01	13,04	0,01	0,15	0,56
5376	102	3	Sukamantri	>500	Afrika	Maesopsis eminii Engl.	Rhamnaceae	0,43	2,1	9	16	0,04	0,00	2,48	0,00	0,03	0,11
5377	102	3	Sukamantri	>500	Afrika	Maesopsis eminii Engl.	Rhamnaceae	0,43	19,1	7	8	0,04	0,03	77,12	0,08	0,91	3,33
5378	102	3	Sukamantri	>500	Afrika	Maesopsis eminii Engl.	Rhamnaceae	0,43	20,4	8	9	0,04	0,03	96,69	0,10	1,14	4,17
5379	102	3	Sukamantri	>500	Afrika	Maesopsis eminii Engl.	Rhamnaceae	0,43	11,1	6	7	0,04	0,01	25,42	0,03	0,30	1,10
5380	102	3	Sukamantri	>500	Sengon	Paraserianthes falcataria (L.) I.C.	Leguminosae / Fabaceae	0,33	3,5	4	5	0,04	0,00	1,76	0,00	0,02	0,08
5381	102	3	Sukamantri	>500	Mahoni	Swietenia macrophylla King	Meliaceae	0,61	13,0	4	7	0,04	0,01	46,80	0,05	0,55	2,02
5382	102	3	Sukamantri	>500	Sawo / sawo manila	Manilkara zapota (L.) P.Royen	Sapotaceae	0,81	5,4	2	4	0,04	0,00	7,24	0,01	0,09	0,31
5383	102	3	Sukamantri	>500	Afrika	Maesopsis eminii Engl.	Rhamnaceae	0,43	13,4	7	8	0,04	0,01	40,12	0,04	0,47	1,73
5384	102	3	Sukamantri	>500	Afrika	Maesopsis eminii Engl.	Rhamnaceae	0,43	13,4	8	9	0,04	0,01	44,69	0,04	0,53	1,93
5385	102	3	Sukamantri	>500	Puspa	Schima wallichii Choisy	Theaceae	0,74	13,4	4	7	0,04	0,01	58,38	0,06	0,69	2,52
5386	102	3	Sukamantri	>500	Afrika	Maesopsis eminii Engl.	Rhamnaceae	0,43	14,6	7	8	0,04	0,02	47,40	0,05	0,56	2,04
5387	102	3	Sukamantri	>500	Mahoni	Swietenia macrophylla King	Meliaceae	0,61	7,0	3	4,5	0,04	0,00	9,98	0,01	0,12	0,43
5388	102	3	Sukamantri	>500	Mahoni	Swietenia macrophylla King	Meliaceae	0,61	8,0	3	5	0,04	0,00	13,89	0,01	0,16	0,60
5389	102	3	Sukamantri	>500	Afrika	Maesopsis eminii Engl.	Rhamnaceae	0,43	13,7	9	10	0,04	0,01	51,39	0,05	0,60	2,22
5390	102	3	Sukamantri	>500	Afrika	Maesopsis eminii Engl.	Rhamnaceae	0,43	15,0	7	8	0,04	0,02	49,30	0,05	0,58	2,13
5391	102	3	Sukamantri	>500	Afrika	Maesopsis eminii Engl.	Rhamnaceae	0,43	19,7	10	11	0,04	0,03	109,64	0,11	1,29	4,73
5392	102	3	Sukamantri	>500	Afrika	Maesopsis eminii Engl.	Rhamnaceae	0,43	7,3	6	7	0,04	0,00	11,78	0,01	0,14	0,51
5393	102	3	Sukamantri	>500	Afrika	Maesopsis eminii Engl.	Rhamnaceae	0,43	9,9	7	8	0,04	0,01	23,00	0,02	0,27	0,99
5394	102	3	Sukamantri	>500	Afrika	Maesopsis eminii Engl.	Rhamnaceae	0,43	15,3	8	9	0,04	0,02	57,08	0,06	0,67	2,46
5395	102	3	Sukamantri	>500	Afrika	Maesopsis eminii Engl.	Rhamnaceae	0,43	16,9	10	11	0,04	0,02	82,26	0,08	0,97	3,55
5396	102	3	Sukamantri	>500	Mahoni	Swietenia macrophylla King	Meliaceae	0,61	12,1	7	10	0,04	0,01	56,45	0,06	0,66	2,43
5397	102	3	Sukamantri	>500	Afrika	Maesopsis eminii Engl.	Rhamnaceae	0,43	18,5	10	11	0,04	0,03	97,03	0,10	1,14	4,18
5398	102	3	Sukamantri	>500	Huru keling	Litsea sp.	Lauraceae	0,425	7,3	3	5	0,04	0,00	8,56	0,01	0,10	0,37
5399	102	3	Sukamantri	>500	Afrika	Maesopsis eminii Engl.	Rhamnaceae	0,43	5,4	4	5	0,04	0,00	4,98	0,00	0,06	0,21
5400	102	3	Sukamantri	>500	Sengon	Paraserianthes falcataria (L.) I.C.	Leguminosae / Fabaceae	0,33	5,4	7	8	0,04	0,00	6,00	0,01	0,07	0,26
5401	102	3	Sukamantri	>500	Mahoni	Swietenia macrophylla King	Meliaceae	0,61	8,9	4	5,5	0,04	0,01	18,66	0,02	0,22	0,80
5402	102	3	Sukamantri	>500	Afrika	Maesopsis eminii Engl.	Rhamnaceae	0,43	11,5	8	9	0,04	0,01	33,70	0,03	0,40	1,45
5403	102	3	Sukamantri	>500	Afrika	Maesopsis eminii Engl.	Rhamnaceae	0,43	5,4	5	6	0,04	0,00	5,88	0,01	0,07	0,25
5404	102	3	Sukamantri	>500	Mahoni	Swietenia macrophylla King	Meliaceae	0,61	4,1	2	3	0,04	0,00	2,63	0,00	0,03	0,11
5405	102	3	Sukamantri	>500	Afrika	Maesopsis eminii Engl.	Rhamnaceae	0,43	4,8	4	5	0,04	0,00	3,96	0,00	0,05	0,17
5406	102	3	Sukamantri	>500	Huru keling	Litsea sp.	Lauraceae	0,425	8,3	4	5	0,04	0,01	10,72	0,01	0,13	0,46
5407	102	3	Sukamantri	>500	Huru keling	Litsea sp.	Lauraceae	0,425	8,9	7	8	0,04	0,01	18,89	0,02	0,22	0,81
5408	102	3	Sukamantri	>500	Afrika	Maesopsis eminii Engl.	Rhamnaceae	0,43	17,2	9	10	0,04	0,02	78,00	0,08	0,92	3,36
5409	102	3	Sukamantri	>500	Afrika	Maesopsis eminii Engl.	Rhamnaceae	0,43	11,8	9	10	0,04	0,01	39,02	0,04	0,46	1,68
5410	102	3	Sukamantri	>500	Afrika	Maesopsis eminii Engl.	Rhamnaceae	0,43	7,6	7	8	0,04	0,00	14,39	0,01	0,17	0,62
5411	102	3	Sukamantri	>500	Afrika	Maesopsis eminii Engl.	Rhamnaceae	0,43	17,5	9	10	0,04	0,02	80,67	0,08	0,95	3,48
5412	102	3	Sukamantri	>500	Afrika	Maesopsis eminii Engl.	Rhamnaceae	0,43	8,0	7	8	0,04	0,00	15,51	0,02	0,18	0,67
5413	102	3	Sukamantri	>500	Afrika	Maesopsis eminii Engl.	Rhamnaceae	0,43	5,4	6	6,5	0,04	0,00	6,33	0,01	0,07	0,27
5414	102	3	Sukamantri	>500	Huru keling	Litsea sp.	Lauraceae	0,425	5,4	3	4	0,04	0,00	4,01	0,00	0,05	0,17
5415	102	3	Sukamantri	>500	Huru keling	Litsea sp.	Lauraceae	0,425	7,0	5	6	0,04	0,00	9,33	0,01	0,11	0,40
5416	102	3	Sukamantri	>500	Huru keling	Litsea sp.	Lauraceae	0,425	6,4	5	6,5	0,04	0,00	8,43	0,01	0,10	0,36
5417	102	3	Sukamantri	>500	Huru keling	Litsea sp.	Lauraceae	0,425	11,1	7	8	0,04	0,01	28,42	0,03	0,33	1,23
5418	102	3	Sukamantri	>500	Huru keling	Litsea sp.	Lauraceae	0,425	11,1	7,5	9	0,04	0,01	31,66	0,03	0,37	1,37
5419	102	3	Sukamantri	>500	Huru keling	Litsea sp.	Lauraceae	0,425	10,8	7	8	0,04	0,01	26,95	0,03	0,32	1,16
5420	102	3	Sukamantri	>500	Huru keling	Litsea sp.	Lauraceae	0,425	8,9	6	7,5	0,04	0,01	17,80	0,02	0,21	0,77
5421	102	3	Sukamantri	>500	Afrika	Maesopsis eminii Engl.	Rhamnaceae	0,43	12,7	9	10	0,04	0,01	45,01	0,05	0,53	1,94
5422	102	3	Sukamantri	>500	Afrika	Maesopsis eminii Engl.	Rhamnaceae	0,43	4,8	4	5	0,04	0,00	3,96	0,00	0,05	0,17
5423	102	3	Sukamantri	>500	Mahoni	Swietenia macrophylla King	Meliaceae	0,61	4,1	2	3,5	0,04	0,00	3,02	0,00	0,04	0,13
5424	102	3	Sukamantri	>500	Afrika	Maesopsis eminii Engl.	Rhamnaceae	0,43	4,1	3	4	0,04	0,00	2,48	0,00	0,03	0,11
5425	103	1	Sukamantri	>500	Ganitri	Elaeocarpus ganitrus Roxb. ex G.	Elaeocarpaceae	0,49	15,0	10	11	0,04	0,02	74,39	0,07	0,87	3,21
5426	103	1	Sukamantri	>500	Ganitri	Elaeocarpus ganitrus Roxb. ex G.	Elaeocarpaceae	0,49	9,2	7	8	0,04	0,01	22,94	0,02	0,27	0,99
5427	103	1	Sukamantri	>500	Ganitri	Elaeocarpus ganitrus Roxb. ex G.	Elaeocarpaceae	0,49	20,4	8	10	0,04	0,03	120,02	0,12	1,41	5,18
5428	103	1	Sukamantri	>500	Ganitri	Elaeocarpus ganitrus Roxb. ex G.	Elaeocarpaceae	0,49	12,1	8	9	0,04	0,01	41,94	0,04	0,49	1,81
5429	103	1	Sukamantri	>500	Ganitri	Elaeocarpus ganitrus Roxb. ex G.	Elaeocarpaceae	0,49	12,4	9	10	0,04	0,01	48,44	0,05	0,57	2,09
5430	103	1	Sukamantri	>500	Ganitri	Elaeocarpus ganitrus Roxb. ex G.	Elaeocarpaceae	0,49	7,0	7	8	0,04	0,00	13,83	0,01	0,16	0,60
5431	103	1	Sukamantri	>500	Ganitri	Elaeocarpus ganitrus Roxb. ex G.	Elaeocarpaceae	0,49	10,2	8	9	0,04	0,01	30,61	0,03	0,36	1,32
5432	103	1	Sukamantri	>500	Karet	Hevea brasiliensis (Willd. ex A.Ju	Euphorbiaceae	0,53	4,5	2	3	0,04	0,00	2,64	0,00	0,03	0,11
5433	103	1	Sukamantri	>500	Ganitri	Elaeocarpus ganitrus Roxb. ex G.	Elaeocarpaceae	0,49	13,0	8,5	9	0,04	0,01	48,20	0,05	0,57	2,08
5434	103	1	Sukamantri	>500	Ganitri	Elaeocarpus ganitrus Roxb. ex G.	Elaeocarpaceae	0,49	10,2	10	11	0,04	0,01	36,79	0,04	0,43	1,59
5435	103	1	Sukamantri	>500	Afrika	Maesopsis eminii Engl.	Rhamnaceae	0,43	6,7	2	6	0,04	0,00	8,66	0,01	0,10	0,37
5436	103	1	Sukamantri	>500	Ganitri	Elaeocarpus ganitrus Roxb. ex G.	Elaeocarpaceae	0,49	7,0	9	10	0,04	0,00	16,97	0,02	0,20	0,73
5437	103	1	Sukamantri	>500	Ganitri	Elaeocarpus ganitrus Roxb. ex G.	Elaeocarpaceae	0,49	6,4	5	6	0,04	0,00	8,93	0,01	0,10	0,38
5438	103	1	Sukamantri	>500	Ganitri	Elaeocarpus ganitrus Roxb. ex G.	Elaeocarpaceae	0,49	3,2	3	4	0,04	0,00	1,73	0,00	0,02	0,07
5439	103	1	Sukamantri	>500	Ganitri	Elaeocarpus ganitrus Roxb. ex G.	Elaeocarpaceae	0,49	7,6	7	8	0,04	0,00	16,22	0,02	0,19	0,70
5440	103	1	Sukamantri	>500	Ganitri	Elaeocarpus ganitrus Roxb. ex G.	Elaeocarpaceae	0,49	8,6	10	11	0,04	0,01	26,95	0,03	0,32	1,16
5441	103	1	Sukamantri	>500	Ganitri	Elaeocarpus ganitrus Roxb. ex G.	Elaeocarpaceae	0,49	9,9	10	11	0,04	0,01	34,71	0,03	0,41	1,50
5442	103	1	Sukamantri	>500	Ganitri	Elaeocarpus ganitrus Roxb. ex G.	Elaeocarpaceae	0,49	8,9	9	9,5	0,04	0,01	25,18	0,03	0,30	1,09
5443	103	1	Sukamantri	>500	Ganitri	Elaeocarpus ganitrus Roxb. ex G.	Elaeocarpaceae	0,49	11,8	10	11	0,04	0,01	48,00	0,05	0,56	2,07
5444	103	1	Sukamantri	>500	Ganitri	Elaeocarpus ganitrus Roxb. ex G.	Elaeocarpaceae	0,49	7,3	8	9	0,04	0,00	16,72	0,02	0,20	0,72
5445	103	1	Sukamantri	>500	Ganitri	Elaeocarpus ganitrus Roxb. ex G.	Elaeocarpaceae	0,49	9,5	10	11	0,04	0,01	32,68	0,03	0,38	1,41
5446	103	1	Sukamantri	>500	Karet	Hevea brasiliensis (Willd. ex A.Ju	Euphorbiaceae	0,53	4,1	3	4	0,04	0,00	3,00	0,00	0,04	0,13
5447	103	1	Sukamantri	>500	Ganitri	Elaeocarpus ganitrus Roxb. ex G.	Elaeocarpaceae	0,49	12,7	10	11	0,04	0,01	55,36	0,06	0,65	2,39
5448	103	1	Sukamantri	>500	Ganitri	Elaeocarpus ganitrus Roxb. ex G.	Elaeocarpaceae	0,49	8,9	6	7	0,04	0,01	19,04	0,02	0,22	0,82
5449	103	1	Sukamantri	>500	Ganitri	Elaeocarpus ganitrus Roxb. ex G.	Elaeocarpaceae	0,49	9,2	10	11	0,04	0,01	30,72	0,03	0,36	1,32
5450	103	1	Sukamantri	>500	Ganitri	Elaeocarpus ganitrus Roxb. ex G.	Elaeocarpaceae	0,49	12,1	10	11	0,04	0,01	50,40	0,05	0,59	2,17

5451	103	1	Sukamantri	>500	Karet	Hevea brasiliensis (Willd. ex A.Ju	Euphorbiaceae		0,53	7,6	3	7	0,04	0,00	15,42	0,02	0,18	0,67
5452	103	1	Sukamantri	>500	Ganitri	Elaeocarpus ganitrus Roxb. ex G.	Elaeocarpaceae		0,49	10,2	9	10	0,04	0,01	33,71	0,03	0,40	1,45
5453	103	1	Sukamantri	>500	Puspa	Schima wallichii Choisy	Theaceae		0,74	14,3	9	11	0,04	0,02	100,22	0,10	1,18	4,32
5454	103	1	Sukamantri	>500	Ganitri	Elaeocarpus ganitrus Roxb. ex G.	Elaeocarpaceae		0,49	8,0	7	8	0,04	0,00	17,48	0,02	0,21	0,75
5455	103	1	Sukamantri	>500	Ganitri	Elaeocarpus ganitrus Roxb. ex G.	Elaeocarpaceae		0,49	6,4	3	5	0,04	0,00	7,55	0,01	0,09	0,33
5456	103	1	Sukamantri	>500	Ganitri	Elaeocarpus ganitrus Roxb. ex G.	Elaeocarpaceae		0,49	7,3	6	7	0,04	0,00	13,28	0,01	0,16	0,57
5457	103	1	Sukamantri	>500	Ganitri	Elaeocarpus ganitrus Roxb. ex G.	Elaeocarpaceae		0,49	11,1	10	11,5	0,04	0,01	45,15	0,05	0,53	1,95
5458	103	1	Sukamantri	>500	Ganitri	Elaeocarpus ganitrus Roxb. ex G.	Elaeocarpaceae		0,49	11,5	10	12	0,04	0,01	49,43	0,05	0,58	2,13
5459	104	3	Sukamantri	>500	Gmelina	Gmelina arborea Roxb.	Lamiaceae / Verbenaceae		0,47	22,3	9	12	0,04	0,04	160,88	0,16	1,89	6,94
5460	104	3	Sukamantri	>500	Gmelina	Gmelina arborea Roxb.	Lamiaceae / Verbenaceae		0,47	11,5	5	7	0,04	0,01	29,04	0,03	0,34	1,25
5461	104	3	Sukamantri	>500	Gmelina	Gmelina arborea Roxb.	Lamiaceae / Verbenaceae		0,47	17,8	9	12	0,04	0,02	106,90	0,11	1,26	4,61
5462	104	3	Sukamantri	>500	Gmelina	Gmelina arborea Roxb.	Lamiaceae / Verbenaceae		0,47	19,4	8	12	0,04	0,03	125,03	0,13	1,47	5,39
5463	104	3	Sukamantri	>500	Manglid	Magnolia champaca (L.) Baill. ex	Magnoliaceae		0,53	9,9	3	9	0,04	0,01	31,03	0,03	0,36	1,34
5464	104	3	Sukamantri	>500	Huru keling	Litsea sp.	Lauraceae		0,425	12,7	5	8	0,04	0,01	36,30	0,04	0,43	1,57
5465	104	3	Sukamantri	>500	Gmelina	Gmelina arborea Roxb.	Lamiaceae / Verbenaceae		0,47	30,9	11	14	0,04	0,07	336,81	0,34	3,96	14,52
5466	104	3	Sukamantri	>500	Gmelina	Gmelina arborea Roxb.	Lamiaceae / Verbenaceae		0,47	38,5	10	14	0,04	0,12	504,98	0,50	5,93	21,78
5467	104	3	Sukamantri	>500	Gmelina	Gmelina arborea Roxb.	Lamiaceae / Verbenaceae		0,47	19,4	11	13	0,04	0,03	134,54	0,13	1,58	5,80
5468	104	3	Sukamantri	>500	Gmelina	Gmelina arborea Roxb.	Lamiaceae / Verbenaceae		0,47	18,5	11	13	0,04	0,03	122,67	0,12	1,44	5,29
5469	104	3	Sukamantri	>500	Gmelina	Gmelina arborea Roxb.	Lamiaceae / Verbenaceae		0,47	11,1	7	9	0,04	0,01	34,72	0,03	0,41	1,50
5470	104	3	Sukamantri	>500	Gmelina	Gmelina arborea Roxb.	Lamiaceae / Verbenaceae		0,47	22,3	10	14	0,04	0,04	185,28	0,19	2,18	7,99
5471	104	3	Sukamantri	>500	Afrika	Maesopsis eminii Engl.	Rhamnaceae		0,43	11,1	10	12	0,04	0,01	41,65	0,04	0,49	1,80
5472	104	3	Sukamantri	>500	Afrika	Maesopsis eminii Engl.	Rhamnaceae		0,43	8,6	6	9	0,04	0,01	19,89	0,02	0,23	0,86
5473	104	3	Sukamantri	>500	Gmelina	Gmelina arborea Roxb.	Lamiaceae / Verbenaceae		0,47	20,4	7	9	0,04	0,03	104,90	0,10	1,23	4,52
5474	104	3	Sukamantri	>500	Afrika	Maesopsis eminii Engl.	Rhamnaceae		0,43	17,2	11	12	0,04	0,02	92,18	0,09	1,08	3,98
5475	104	3	Sukamantri	>500	Huru keling	Litsea sp.	Lauraceae		0,425	10,5	5	7	0,04	0,01	22,58	0,02	0,27	0,97
5476	104	3	Sukamantri	>500	Huru keling	Litsea sp.	Lauraceae		0,425	12,7	5	7	0,04	0,01	32,12	0,03	0,38	1,39
5477	104	3	Sukamantri	>500	Manglid	Magnolia champaca (L.) Baill. ex	Magnoliaceae		0,53	8,3	4	7	0,04	0,01	17,86	0,02	0,21	0,77
5478	104	3	Sukamantri	>500	Afrika	Maesopsis eminii Engl.	Rhamnaceae		0,43	16,5	12	13	0,04	0,02	92,57	0,09	1,09	3,99
5479	104	3	Sukamantri	>500	Manglid	Magnolia champaca (L.) Baill. ex	Magnoliaceae		0,53	4,1	3	4	0,04	0,00	3,00	0,00	0,04	0,13
5480	104	3	Sukamantri	>500	Manglid	Magnolia champaca (L.) Baill. ex	Magnoliaceae		0,53	27,4	10	12	0,04	0,06	261,87	0,26	3,08	11,29
5481	104	3	Sukamantri	>500	Tisuk	Hibiscus macrophyllus Roxb. ex l	Malvaceae		0,4	7,0	3	5	0,04	0,00	7,47	0,01	0,09	0,32
5482	104	3	Sukamantri	>500	Tisuk	Hibiscus macrophyllus Roxb. ex l	Malvaceae		0,4	23,9	8	12	0,04	0,04	157,49	0,16	1,85	6,79
5483	104	3	Sukamantri	>500	Manglid	Magnolia champaca (L.) Baill. ex	Magnoliaceae		0,53	18,1	6	10	0,04	0,03	104,31	0,10	1,23	4,50
5484	104	3	Sukamantri	>500	Manglid	Magnolia champaca (L.) Baill. ex	Magnoliaceae		0,53	5,1	3	5	0,04	0,00	5,39	0,01	0,06	0,23
5485	104	3	Sukamantri	>500	Manglid	Magnolia champaca (L.) Baill. ex	Magnoliaceae		0,53	8,0	3	6	0,04	0,00	14,43	0,01	0,17	0,62
5486	104	3	Sukamantri	>500	Nangka	Artocarpus heterophyllus Lam.	Moraceae		0,61	7,6	4	5,5	0,04	0,00	14,07	0,01	0,17	0,61
5487	104	3	Sukamantri	>500	Huru keling	Litsea sp.	Lauraceae		0,425	17,2	7	11	0,04	0,02	84,21	0,08	0,99	3,63
5488	104	3	Sukamantri	>500	Afrika	Maesopsis eminii Engl.	Rhamnaceae		0,43	24,8	14	16	0,04	0,05	235,32	0,24	2,77	10,15
5489	104	3	Sukamantri	>500	Manglid	Magnolia champaca (L.) Baill. ex	Magnoliaceae		0,53	15,9	11	13	0,04	0,02	104,34	0,10	1,23	4,50
5490	104	3	Sukamantri	>500	Afrika	Maesopsis eminii Engl.	Rhamnaceae		0,43	23,9	13	15	0,04	0,04	206,44	0,21	2,43	8,90
5491	104	3	Sukamantri	>500	Suren	Toona sureni (Blume) Merr.	Meliaceae		0,39	11,8	4	7	0,04	0,01	25,74	0,03	0,30	1,11
5492	104	3	Sukamantri	>500	Afrika	Maesopsis eminii Engl.	Rhamnaceae		0,43	21,6	13	14	0,04	0,04	161,95	0,16	1,90	6,98
5493	104	3	Sukamantri	>500	Manglid	Magnolia champaca (L.) Baill. ex	Magnoliaceae		0,53	16,2	8	12	0,04	0,02	100,54	0,10	1,18	4,34
5494	104	3	Sukamantri	>500	Afrika	Maesopsis eminii Engl.	Rhamnaceae		0,43	21,0	13	15	0,04	0,03	163,34	0,16	1,92	7,04
5495	104	3	Sukamantri	>500	Tisuk	Hibiscus macrophyllus Roxb. ex l	Malvaceae		0,4	10,8	9	11	0,04	0,01	34,13	0,03	0,40	1,47
5496	104	3	Sukamantri	>500	Tisuk	Hibiscus macrophyllus Roxb. ex l	Malvaceae		0,4	9,9	7	9	0,04	0,01	23,98	0,02	0,28	1,03
5497	104	3	Sukamantri	>500	Tisuk	Hibiscus macrophyllus Roxb. ex l	Malvaceae		0,4	13,7	9	12	0,04	0,01	56,84	0,06	0,67	2,45
5498	104	3	Sukamantri	>500	Sp8	Sp8		0	0,5	9,2	2,5	5	0,04	0,01	15,20	0,02	0,18	0,66
5499	104	3	Sukamantri	>500	Manglid	Magnolia champaca (L.) Baill. ex	Magnoliaceae		0,53	15,0	10	13	0,04	0,02	93,16	0,09	1,09	4,02
5500	104	3	Sukamantri	>500	Gmelina	Gmelina arborea Roxb.	Lamiaceae / Verbenaceae		0,47	38,2	10	16	0,04	0,11	562,08	0,56	6,60	24,24
5501	104	3	Sukamantri	>500	Afrika	Maesopsis eminii Engl.	Rhamnaceae		0,43	14,3	10	11	0,04	0,02	60,95	0,06	0,72	2,63
5502	104	3	Sukamantri	>500	Puspa	Schima wallichii Choisy	Theaceae		0,74	12,4	3	10	0,04	0,01	70,66	0,07	0,83	3,05
5503	104	3	Sukamantri	>500	Manglid	Magnolia champaca (L.) Baill. ex	Magnoliaceae		0,53	11,1	9	11	0,04	0,01	46,58	0,05	0,55	2,01
5504	104	3	Sukamantri	>500	Ganitri	Elaeocarpus ganitrus Roxb. ex G.	Elaeocarpaceae		0,49	12,4	3	11	0,04	0,01	52,85	0,05	0,62	2,28
5505	104	3	Sukamantri	>500	Ganitri	Elaeocarpus ganitrus Roxb. ex G.	Elaeocarpaceae		0,49	6,0	7	9	0,04	0,00	11,78	0,01	0,14	0,51
5506	104	3	Sukamantri	>500	Ganitri	Elaeocarpus ganitrus Roxb. ex G.	Elaeocarpaceae		0,49	5,4	5	6	0,04	0,00	6,63	0,01	0,08	0,29
5507	104	3	Sukamantri	>500	Tisuk	Hibiscus macrophyllus Roxb. ex l	Malvaceae		0,4	12,4	10	13	0,04	0,01	51,15	0,05	0,60	2,21
5508	104	3	Sukamantri	>500	Afrika	Maesopsis eminii Engl.	Rhamnaceae		0,43	14,6	13	14	0,04	0,02	79,14	0,08	0,93	3,41
5509	104	3	Sukamantri	>500	Afrika	Maesopsis eminii Engl.	Rhamnaceae		0,43	23,2	14	15	0,04	0,04	196,46	0,20	2,31	8,47
5510	105	1	Sukamantri	>500	Alpukat	Persea americana Mill.	Lauraceae		0,54	6,4	3	4	0,04	0,00	6,73	0,01	0,08	0,29
5511	105	1	Sukamantri	>500	Afrika	Maesopsis eminii Engl.	Rhamnaceae		0,43	7,6	3	4	0,04	0,00	7,63	0,01	0,09	0,33
5512	105	1	Sukamantri	>500	Afrika	Maesopsis eminii Engl.	Rhamnaceae		0,43	9,9	3	4	0,04	0,01	12,19	0,01	0,14	0,53
5513	105	1	Sukamantri	>500	Afrika	Maesopsis eminii Engl.	Rhamnaceae		0,43	8,0	3	4,5	0,04	0,00	9,16	0,01	0,11	0,39
5514	105	1	Sukamantri	>500	Afrika	Maesopsis eminii Engl.	Rhamnaceae		0,43	8,0	4	4,5	0,04	0,00	9,16	0,01	0,11	0,39
5515	105	1	Sukamantri	>500	Afrika	Maesopsis eminii Engl.	Rhamnaceae		0,43	3,2	1,5	3	0,04	0,00	1,18	0,00	0,01	0,05
5516	105	1	Sukamantri	>500	Sengon	Paraserianthes falcataria (L.) I.C.	Leguminosae / Fabaceae		0,33	21,3	6	9	0,04	0,04	82,52	0,08	0,97	3,56
5517	105	1	Sukamantri	>500	Sengon	Paraserianthes falcataria (L.) I.C.	Leguminosae / Fabaceae		0,33	12,7	9	12	0,04	0,01	41,74	0,04	0,49	1,80
5518	105	1	Sukamantri	>500	Afrika	Maesopsis eminii Engl.	Rhamnaceae		0,43	7,0	2	5	0,04	0,00	7,98	0,01	0,09	0,34
5519	105	1	Sukamantri	>500	Afrika	Maesopsis eminii Engl.	Rhamnaceae		0,43	3,5	2	3,5	0,04	0,00	1,62	0,00	0,02	0,07
5520	105	1	Sukamantri	>500	Afrika	Maesopsis eminii Engl.	Rhamnaceae		0,43	5,1	2	3,5	0,04	0,00	3,21	0,00	0,04	0,14
5521	105	1	Sukamantri	>500	Afrika	Maesopsis eminii Engl.	Rhamnaceae		0,43	5,7	2	4	0,04	0,00	4,50	0,00	0,05	0,19
5522	105	1	Sukamantri	>500	Afrika	Maesopsis eminii Engl.	Rhamnaceae		0,43	3,2	1,5	3	0,04	0,00	1,18	0,00	0,01	0,05
5523	105	1	Sukamantri	>500	Afrika	Maesopsis eminii Engl.	Rhamnaceae		0,43	6,0	3	4,5	0,04	0,00	5,54	0,01	0,07	0,24
5524	105	1	Sukamantri	>500	Sengon	Paraserianthes falcataria (L.) I.C.	Leguminosae / Fabaceae		0,33	14,6	6	8	0,04	0,02	37,19	0,04	0,44	1,60
5525	105	1	Sukamantri	>500	Afrika	Maesopsis eminii Engl.	Rhamnaceae		0,43	5,4	3	4,5	0,04	0,00	4,52	0,00	0,05	0,19
5526	105	1	Sukamantri	>500	Sengon	Paraserianthes falcataria (L.) I.C.	Leguminosae / Fabaceae		0,33	17,2	9	12	0,04	0,02	72,34	0,07	0,85	3,12
5527	105	1	Sukamantri	>500	Afrika	Maesopsis eminii Engl.	Rhamnaceae		0,43	8,0	3	4,5	0,04	0,00	9,16	0,01	0,11	0,39
5528	105	1	Sukamantri	>500	Alpukat	Persea americana Mill.	Lauraceae		0,54	4,8	1,5	3	0,04	0,00	3,05	0,00	0,04	0,13
5529	105	1	Sukamantri	>500	Afrika	Maesopsis eminii Engl.	Rhamnaceae		0,43	7,6	3	4,5	0,04	0,00	8,50	0,01	0,10	0,37
5530	105	1	Sukamantri	>500	Afrika	Maesopsis eminii Engl.	Rhamnaceae		0,43	7,3	3	4,5	0,04	0,00	7,86	0,01	0,09	0,34
5531	105	1	Sukamantri	>500	Afrika	Maesopsis eminii Engl.	Rhamnaceae		0,43	3,8	2	3	0,04	0,00	1,65	0,00	0,02	0,07
5532	105	1	Sukamantri	>500	Afrika	Maesopsis eminii Engl.	Rhamnaceae		0,43	5,1	2	3	0,04	0,00	2,79	0,00	0,03	0,12
5533	105	1	Sukamantri	>500	Afrika	Maesopsis eminii Engl.	Rhamnaceae		0,43	5,1	3	4	0,04	0,00	3,63	0,00	0,04	0,16
5534	105	1	Sukamantri	>500	Tisuk	Hibiscus macrophyllus Roxb. ex l	Malvaceae		0,4	8,9	2,5	5	0,04	0,01	11,62	0,01	0,14	0,50
5535	105	1	Sukamantri	>500	Afrika	Maesopsis eminii Engl.	Rhamnaceae		0,43	3,8	1,5	3	0,04	0,00	1,65	0,00	0,02	0,07
5536	105	1	Sukamantri	>500	Sengon	Paraserianthes falcataria (L.) I.C.	Leguminosae / Fabaceae		0,33	18,1	6	10	0,04	0,03	67,58	0,07	0,79	2,91
5537	105	1	Sukamantri	>500	Sengon	Paraserianthes falcataria (L.) I.C.	Leguminosae / Fabaceae		0,33	12,4	6	9	0,04	0,01	30,62	0,03	0,36	1,32
5538	105	1	Sukamantri	>500	Afrika	Maesopsis eminii Engl.	Rhamnaceae		0,43	3,5	1,5	3	0,04	0,00	1,40	0,00	0,02	0,06
5539	105	1	Sukamantri	>500	Tisuk	Hibiscus macrophyllus Roxb. ex l	Malvaceae		0,4	12,7	3	8	0,04	0,01	34,34	0,03	0,40	1,48
5540	105	1	Sukamantri	>500	Sengon	Paraserianthes falcataria (L.) I.C.	Leguminosae / Fabaceae		0,33	13,4	8	10	0,04	0,01	38,63	0,04	0,45	1,67
5541	105	1	Sukamantri	>500	Sengon	Paraserianthes falcataria (L.) I.C.	Leguminosae / Fabaceae		0,33	15,6	7	10	0,04	0,02	51,23	0,05	0,60	2,21
5542	105	1	Sukamantri	>500	Sengon	Paraserianthes falcataria (L.) I.C.	Leguminosae / Fabaceae		0,33	15,3	7	10	0,04	0,02	49,33	0,05	0,58	2,13
5543	105	1	Sukamantri	>500	Sengon	Paraserianthes falcataria (L.) I.C.	Leguminosae / Fabaceae		0,33	15,3	8	10	0,04	0,02	49,33	0,05	0,58	2,13
5544	105	1	Sukamantri	>500	Afrika	Maesopsis eminii Engl.	Rhamnaceae		0,43	3,8	2	4	0,04	0,00	2,14	0,00	0,03	0,09
5545	105	1	Sukamantri	>500	Afrika	Maesopsis eminii Engl.	Rhamnaceae		0,43	7,6	4	6	0,04	0,00	11,06	0,01	0,13	0,48
5546	105	1	Sukamantri	>500	Afrika	Maesopsis eminii Engl.	Rhamnaceae		0,43	9,5	4	6	0,04	0,01	16,64	0,02	0,20	0,72
5547	105	1	Sukamantri	>500	Ganitri	Elaeocarpus ganitrus Roxb. ex G.	Elaeocarpaceae		0,49	5,1	2	4	0,04	0,00	4,09	0,00	0,05	0,18
5548	105	1	Sukamantri	>500	Afrika	Maesopsis eminii Engl.	Rhamnaceae		0,43	6,4	30	4,5	0,04	0,00	6,08	0,01	0,07	0,26
5549	105	1	Sukamantri	>500	Afrika	Maesopsis eminii Engl.	Rhamnaceae		0,43	7,0	3	5	0,04	0,00	7,98	0,01	0,09	0,34
5550	105	1	Sukamantri	>500	Afrika	Maesopsis eminii Engl.	Rhamnaceae		0,43	5,4	2,5	3,5	0,04	0,00	3,59	0,00	0,04	0,15
5551	105	1	Sukamantri	>500	Afrika	Maesopsis eminii Engl.	Rhamnaceae		0,43	3,5	2	3	0,04	0,00	1,40	0,00	0,02	0,06
5552	105	1	Sukamantri	>500	Afrika	Maesopsis eminii Engl.	Rhamnaceae		0,43	5,7	3	4,5	0,04	0,00	5,02	0,01	0,06	0,22
5553	105	1	Sukamantri	>500	Afrika	Maesopsis eminii Engl.	Rhamnaceae		0,43	4,5	2,5	3,5	0,04	0,00	2,51	0,00	0,03	0,11
5554	105	1	Sukamantri	>500	Sengon	Paraserianthes falcataria (L.) I.C.	Leguminosae / Fabaceae		0,33	15,9	8	9	0,04	0,02	48,27	0,05	0,57	2,08
5555	105	1	Sukamantri	>500	Sengon	Paraserianthes falcataria (L.) I.C.	Leguminosae / Fabaceae		0,33	8,6	5	7	0,04	0,01	12,40	0,01	0,15	0,53
5556	105	1	Sukamantri	>500	Tisuk	Hibiscus macrophyllus Roxb. ex l	Malvaceae		0,4	13,7	4	8	0,04	0,01	39,21	0,04	0,46	1,69
5557	105	1	Sukamantri	>500	Afrika	Maesopsis eminii Engl.	Rhamnaceae		0,43	4,8	2,5	3,5	0,04	0,00	2,85	0,00	0,03	0,12
5558	105	1	Sukamantri	>500	Afrika	Maesopsis eminii Engl.	Rhamnaceae		0,43	6,0	3	4,5	0,04	0,00	5,54	0,01	0,07	0,24
5559	105	1	Sukamantri	>500	Sengon	Paraserianthes falcataria (L.) I.C.	Leguminosae / Fabaceae		0,33	15,0	7	11	0,04	0,02	51,79	0,05	0,61	2,23
5560	105	1	Sukamantri	>500	Afrika	Maesopsis eminii Engl.	Rhamnaceae		0,43	7,3	4	6	0,04	0,00	10,23	0,01	0,12	0,44
5561	105	1	Sukamantri	>500	Tisuk	Hibiscus macrophyllus Roxb. ex l	Malvaceae		0,4	10,5	2	6	0,04	0,01	18,55	0,02	0,22	0,80
5562	105	1	Sukamantri	>500	Afrika	Maesopsis eminii Engl.	Rhamnaceae		0,43	8,6	3	4	0,04	0,01	9,47	0,01	0,11	0,41
5563	105	1	Sukamantri	>500	Sengon	Paraserianthes falcataria (L.) I.C.	Leguminosae / Fabaceae		0,33	16,2	7	12	0,04	0,02	65,14	0,07	0,77	2,81
5564	105	1	Sukamantri	>500	Ganitri	Elaeocarpus ganitrus Roxb. ex G.	Elaeocarpaceae		0,49	3,5	1,5	3	0,04	0,00	1,58	0,00	0,02	0,07
5565	105	1	Sukamantri	>500	Afrika	Maesopsis eminii Engl.	Rhamnaceae		0,43	2,9	1,5	3	0,04	0,00	0,97	0,00	0,01	0,04
5566	105	1	Sukamantri	>500	Afrika	Maesopsis eminii Engl.	Rhamnaceae		0,43	3,5	2	3,5	0,04	0,00	1,62	0,00	0,02	0,07
5567	105	1	Sukamantri	>500	Afrika	Maesopsis eminii Engl.	Rhamnaceae		0,43	6,7	3	4	0,04	0,00	5,97	0,01	0,07	0,26
5568	105	1	Sukamantri	>500	Afrika	Maesopsis eminii Engl.	Rhamnaceae		0,43	3,5	2	3,5	0,04	0,00	1,62	0,00	0,02	0,07
5569	105	1	Sukamantri	>500	Tisuk	Hibiscus macrophyllus Roxb. ex l	Malvaceae		0,4	8,3	3	6	0,04	0,01	11,98	0,01	0,14	0,52
5570	105	1	Sukamantri	>500	Tisuk	Hibiscus macrophyllus Roxb. ex l	Malvaceae		0,4	11,1	3	7	0,04	0,01	23,79	0,02	0,28	1,03
5571	105	1	Sukamantri	>500	Tisuk	Hibiscus macrophyllus Roxb. ex l	Malvaceae		0,4	10,2	3	6	0,04	0,01	17,53	0,02	0,21	0,76
5572	105	1	Sukamantri	>500	Sengon	Paraserianthes falcataria (L.) I.C.	Leguminosae / Fabaceae		0,33	11,1	5	8	0,04	0,01	22,54	0,02	0,26	0,97
5573	105	1	Sukamantri	>500	Sengon	Paraserianthes falcataria (L.) I.C.	Leguminosae / Fabaceae		0,33	4,1	5	6	0,04	0,00	2,82	0,00	0,03	0,12
5574	105	1	Sukamantri	>500	Sengon	Paraserianthes falcataria (L.) I.C.	Leguminosae / Fabaceae		0,33	13,7	6	10	0,04	0,01	40,33	0,04	0,47	1,74

5575	105	1	Sukamantri	>500	Tisuk	Hibiscus macrophyllus Roxb. ex	Malvaceae	0,4	14,3	3	8	0,04	0,02	42,61	0,04	0,50	1,84
5576	105	1	Sukamantri	>500	Alpukat	Persea americana Mill.	Lauraceae	0,54	3,8	2	3	0,04	0,00	2,03	0,00	0,02	0,09
5577	105	1	Sukamantri	>500	Sengon	Paraserianthes falcataria (L.) I.C.	Leguminosae / Fabaceae	0,33	17,5	4	8	0,04	0,02	51,60	0,05	0,61	2,23
5578	106	1	Sukamantri	>500	Ganitri	Elaeocarpus ganitrus Roxb. ex G.	Elaeocarpaceae	0,49	20,7	12	16	0,04	0,03	189,92	0,19	2,23	8,19
5579	106	1	Sukamantri	>500	Ganitri	Elaeocarpus ganitrus Roxb. ex G.	Elaeocarpaceae	0,49	16,9	10	15	0,04	0,02	123,17	0,12	1,45	5,31
5580	106	1	Sukamantri	>500	Ganitri	Elaeocarpus ganitrus Roxb. ex G.	Elaeocarpaceae	0,49	15,3	8	14	0,04	0,02	96,43	0,10	1,13	4,16
5581	106	1	Sukamantri	>500	Ganitri	Elaeocarpus ganitrus Roxb. ex G.	Elaeocarpaceae	0,49	18,5	8	13	0,04	0,03	127,44	0,13	1,50	5,50
5582	106	1	Sukamantri	>500	Ganitri	Elaeocarpus ganitrus Roxb. ex G.	Elaeocarpaceae	0,49	20,4	9	15	0,04	0,03	174,00	0,17	2,04	7,50
5583	106	1	Sukamantri	>500	Ganitri	Elaeocarpus ganitrus Roxb. ex G.	Elaeocarpaceae	0,49	15,9	7	13	0,04	0,02	97,10	0,10	1,14	4,19
5584	106	1	Sukamantri	>500	Ganitri	Elaeocarpus ganitrus Roxb. ex G.	Elaeocarpaceae	0,49	7,3	6	9	0,04	0,00	16,72	0,02	0,20	0,72
5585	106	1	Sukamantri	>500	Ganitri	Elaeocarpus ganitrus Roxb. ex G.	Elaeocarpaceae	0,49	10,2	9	12	0,04	0,01	39,84	0,04	0,47	1,72
5586	106	1	Sukamantri	>500	Ganitri	Elaeocarpus ganitrus Roxb. ex G.	Elaeocarpaceae	0,49	18,8	8	13	0,04	0,03	131,50	0,13	1,55	5,67
5587	106	1	Sukamantri	>500	Ganitri	Elaeocarpus ganitrus Roxb. ex G.	Elaeocarpaceae	0,49	17,5	9	15	0,04	0,02	131,82	0,13	1,55	5,68
5588	106	1	Sukamantri	>500	Ganitri	Elaeocarpus ganitrus Roxb. ex G.	Elaeocarpaceae	0,49	18,8	9	14	0,04	0,03	140,73	0,14	1,65	6,07
5589	106	1	Sukamantri	>500	Ganitri	Elaeocarpus ganitrus Roxb. ex G.	Elaeocarpaceae	0,49	15,9	9	13	0,04	0,02	97,10	0,10	1,14	4,19
5590	106	1	Sukamantri	>500	Ganitri	Elaeocarpus ganitrus Roxb. ex G.	Elaeocarpaceae	0,49	13,7	9	13	0,04	0,01	73,66	0,07	0,87	3,18
5591	106	1	Sukamantri	>500	Ganitri	Elaeocarpus ganitrus Roxb. ex G.	Elaeocarpaceae	0,49	9,9	9	12	0,04	0,01	37,59	0,04	0,44	1,62
5592	106	1	Sukamantri	>500	Ganitri	Elaeocarpus ganitrus Roxb. ex G.	Elaeocarpaceae	0,49	11,5	8	12	0,04	0,01	49,43	0,05	0,58	2,13
5593	106	1	Sukamantri	>500	Ganitri	Elaeocarpus ganitrus Roxb. ex G.	Elaeocarpaceae	0,49	14,0	9	12	0,04	0,02	71,40	0,07	0,84	3,08
5594	106	1	Sukamantri	>500	Ganitri	Elaeocarpus ganitrus Roxb. ex G.	Elaeocarpaceae	0,49	17,5	10	12	0,04	0,02	107,45	0,11	1,26	4,63
5595	106	1	Sukamantri	>500	Afrika	Maesopsis eminii Engl.	Rhamnaceae	0,43	10,5	9	11	0,04	0,01	34,53	0,03	0,41	1,49
5596	106	1	Sukamantri	>500	Ganitri	Elaeocarpus ganitrus Roxb. ex G.	Elaeocarpaceae	0,49	16,9	10	14	0,04	0,02	115,63	0,12	1,36	4,99
5597	106	1	Sukamantri	>500	Ganitri	Elaeocarpus ganitrus Roxb. ex G.	Elaeocarpaceae	0,49	11,1	9	12	0,04	0,01	46,95	0,05	0,55	2,02
5598	106	1	Sukamantri	>500	Mahoni	Swietenia macrophylla King	Meliaceae	0,61	8,9	4	7	0,04	0,01	23,27	0,02	0,27	1,00
5599	106	1	Sukamantri	>500	Ganitri	Elaeocarpus ganitrus Roxb. ex G.	Elaeocarpaceae	0,49	13,7	9	12	0,04	0,01	68,45	0,07	0,80	2,95
5600	106	1	Sukamantri	>500	Mahoni	Swietenia macrophylla King	Meliaceae	0,61	8,9	3,5	7,5	0,04	0,01	24,79	0,02	0,29	1,07
5601	106	1	Sukamantri	>500	Ganitri	Elaeocarpus ganitrus Roxb. ex G.	Elaeocarpaceae	0,49	12,1	7	11	0,04	0,01	50,40	0,05	0,59	2,17
5602	106	1	Sukamantri	>500	Ganitri	Elaeocarpus ganitrus Roxb. ex G.	Elaeocarpaceae	0,49	15,3	5	9	0,04	0,02	64,34	0,06	0,76	2,77
5603	106	1	Sukamantri	>500	Ganitri	Elaeocarpus ganitrus Roxb. ex G.	Elaeocarpaceae	0,49	13,7	8	10	0,04	0,01	57,92	0,06	0,68	2,50
5604	106	1	Sukamantri	>500	Afrika	Maesopsis eminii Engl.	Rhamnaceae	0,43	14,6	10	10,5	0,04	0,02	60,81	0,06	0,71	2,62
5605	106	1	Sukamantri	>500	Ganitri	Elaeocarpus ganitrus Roxb. ex G.	Elaeocarpaceae	0,49	14,0	9	12	0,04	0,02	71,40	0,07	0,84	3,08
5606	106	1	Sukamantri	>500	Puspa	Schima wallichii Choisy	Theaceae	0,74	13,0	4	9	0,04	0,01	70,31	0,07	0,83	3,03
5607	106	1	Sukamantri	>500	Ganitri	Elaeocarpus ganitrus Roxb. ex G.	Elaeocarpaceae	0,49	12,1	7	11	0,04	0,01	50,40	0,05	0,59	2,17
5608	106	1	Sukamantri	>500	Ganitri	Elaeocarpus ganitrus Roxb. ex G.	Elaeocarpaceae	0,49	13,7	7	11	0,04	0,01	63,21	0,06	0,74	2,73
5609	106	1	Sukamantri	>500	Ganitri	Elaeocarpus ganitrus Roxb. ex G.	Elaeocarpaceae	0,49	13,0	7	11	0,04	0,01	57,93	0,06	0,68	2,50
5610	106	1	Sukamantri	>500	Afrika	Maesopsis eminii Engl.	Rhamnaceae	0,43	13,4	9	11	0,04	0,01	53,71	0,05	0,63	2,32
5611	106	1	Sukamantri	>500	Afrika	Maesopsis eminii Engl.	Rhamnaceae	0,43	11,8	9	10	0,04	0,01	39,02	0,04	0,46	1,68
5612	106	1	Sukamantri	>500	Ganitri	Elaeocarpus ganitrus Roxb. ex G.	Elaeocarpaceae	0,49	13,7	8	12	0,04	0,01	68,45	0,07	0,80	2,95
5613	106	1	Sukamantri	>500	Ganitri	Elaeocarpus ganitrus Roxb. ex G.	Elaeocarpaceae	0,49	16,5	10	13	0,04	0,02	104,33	0,10	1,23	4,50
5614	106	1	Sukamantri	>500	Ganitri	Elaeocarpus ganitrus Roxb. ex G.	Elaeocarpaceae	0,49	11,8	9	10,5	0,04	0,01	45,99	0,05	0,54	1,98
5615	106	1	Sukamantri	>500	Ganitri	Elaeocarpus ganitrus Roxb. ex G.	Elaeocarpaceae	0,49	14,0	9	12,5	0,04	0,02	74,12	0,07	0,87	3,20
5616	106	1	Sukamantri	>500	Afrika	Maesopsis eminii Engl.	Rhamnaceae	0,43	8,0	10	11	0,04	0,00	20,76	0,02	0,24	0,90
5617	106	1	Sukamantri	>500	Sengon	Paraserianthes falcataria (L.) I.C.	Leguminosae / Fabaceae	0,33	54,1	9	17	0,04	0,23	813,49	0,81	9,56	35,08
5618	106	1	Sukamantri	>500	Ganitri	Elaeocarpus ganitrus Roxb. ex G.	Elaeocarpaceae	0,49	10,2	10	12	0,04	0,01	39,84	0,04	0,47	1,72
5619	106	1	Sukamantri	>500	Afrika	Maesopsis eminii Engl.	Rhamnaceae	0,43	8,9	9	10	0,04	0,01	23,42	0,02	0,28	1,01
5620	106	1	Sukamantri	>500	Afrika	Maesopsis eminii Engl.	Rhamnaceae	0,43	12,7	11	12	0,04	0,01	53,20	0,05	0,63	2,29
5621	106	1	Sukamantri	>500	Ganitri	Elaeocarpus ganitrus Roxb. ex G.	Elaeocarpaceae	0,49	12,7	10	11	0,04	0,01	55,36	0,06	0,65	2,39
5622	106	1	Sukamantri	>500	Ganitri	Elaeocarpus ganitrus Roxb. ex G.	Elaeocarpaceae	0,49	11,5	8	11	0,04	0,01	45,65	0,05	0,54	1,97
5623	106	1	Sukamantri	>500	Ganitri	Elaeocarpus ganitrus Roxb. ex G.	Elaeocarpaceae	0,49	13,7	9	12	0,04	0,01	68,45	0,07	0,80	2,95
5624	107	1	Sukamantri	>500	Manglid	Magnolia champaca (L.) Baill. ex	Magnoliaceae	0,53	12,7	8	10	0,04	0,01	54,52	0,05	0,64	2,35
5625	107	1	Sukamantri	>500	Karet	Hevea brasiliensis (Willd. ex A.Ju	Euphorbiaceae	0,53	10,8	4	5	0,04	0,01	21,45	0,02	0,25	0,93
5626	107	1	Sukamantri	>500	Karet	Hevea brasiliensis (Willd. ex A.Ju	Euphorbiaceae	0,53	8,9	3	5	0,04	0,01	15,03	0,02	0,18	0,65
5627	107	1	Sukamantri	>500	Manglid	Magnolia champaca (L.) Baill. ex	Magnoliaceae	0,53	19,1	10	12	0,04	0,03	135,41	0,14	1,59	5,84
5628	107	1	Sukamantri	>500	Karet	Hevea brasiliensis (Willd. ex A.Ju	Euphorbiaceae	0,53	11,8	6	8	0,04	0,01	38,52	0,04	0,45	1,66
5629	107	1	Sukamantri	>500	Manglid	Magnolia champaca (L.) Baill. ex	Magnoliaceae	0,53	19,7	10	12	0,04	0,03	143,80	0,14	1,69	6,20
5630	107	1	Sukamantri	>500	Manglid	Magnolia champaca (L.) Baill. ex	Magnoliaceae	0,53	16,5	11	12	0,04	0,02	104,18	0,10	1,22	4,49
5631	107	1	Sukamantri	>500	Manglid	Magnolia champaca (L.) Baill. ex	Magnoliaceae	0,53	21,3	10	12	0,04	0,04	165,75	0,17	1,95	7,15
5632	107	1	Sukamantri	>500	Karet	Hevea brasiliensis (Willd. ex A.Ju	Euphorbiaceae	0,53	10,5	4	8	0,04	0,01	31,24	0,03	0,37	1,35
5633	107	1	Sukamantri	>500	Manglid	Magnolia champaca (L.) Baill. ex	Magnoliaceae	0,53	21,6	12	15	0,04	0,04	208,94	0,21	2,45	9,01
5634	107	1	Sukamantri	>500	Manglid	Magnolia champaca (L.) Baill. ex	Magnoliaceae	0,53	12,7	3	6	0,04	0,01	34,14	0,03	0,40	1,47
5635	107	1	Sukamantri	>500	Manglid	Magnolia champaca (L.) Baill. ex	Magnoliaceae	0,53	7,3	6	8	0,04	0,00	16,12	0,02	0,19	0,70
5636	107	1	Sukamantri	>500	Karet	Hevea brasiliensis (Willd. ex A.Ju	Euphorbiaceae	0,53	5,4	3	6	0,04	0,00	7,12	0,01	0,08	0,31
5637	107	1	Sukamantri	>500	Manglid	Magnolia champaca (L.) Baill. ex	Magnoliaceae	0,53	22,9	13	16	0,04	0,04	246,13	0,25	2,89	10,61
5638	107	1	Sukamantri	>500	Huru keling	Litsea sp.	Lauraceae	0,425	23,2	12	14	0,04	0,04	182,47	0,18	2,14	7,87
5639	107	1	Sukamantri	>500	Karet	Hevea brasiliensis (Willd. ex A.Ju	Euphorbiaceae	0,53	7,6	6	8	0,04	0,00	17,43	0,02	0,20	0,75
5640	107	1	Sukamantri	>500	Mahoni	Swietenia macrophylla King	Meliaceae	0,61	17,2	7	11	0,04	0,02	117,26	0,12	1,38	5,06
5641	107	1	Sukamantri	>500	Manglid	Magnolia champaca (L.) Baill. ex	Magnoliaceae	0,53	12,1	10	11	0,04	0,01	54,15	0,05	0,64	2,34
5642	107	1	Sukamantri	>500	Puspa	Schima wallichii Choisy	Theaceae	0,74	17,2	4	12	0,04	0,02	151,57	0,15	1,78	6,54
5643	107	1	Sukamantri	>500	Manglid	Magnolia champaca (L.) Baill. ex	Magnoliaceae	0,53	13,0	11	12	0,04	0,01	67,41	0,07	0,79	2,91
5644	107	1	Sukamantri	>500	Mahoni	Swietenia macrophylla King	Meliaceae	0,61	18,5	5	11	0,04	0,03	133,66	0,13	1,57	5,76
5645	107	1	Sukamantri	>500	Manglid	Magnolia champaca (L.) Baill. ex	Magnoliaceae	0,53	20,0	12	15	0,04	0,03	181,66	0,18	2,13	7,83
5646	107	1	Sukamantri	>500	Karet	Hevea brasiliensis (Willd. ex A.Ju	Euphorbiaceae	0,53	4,1	8	9	0,04	0,00	6,32	0,01	0,07	0,27
5647	107	1	Sukamantri	>500	Karet	Hevea brasiliensis (Willd. ex A.Ju	Euphorbiaceae	0,53	12,7	7	11	0,04	0,01	59,49	0,06	0,70	2,57
5648	107	1	Sukamantri	>500	Karet	Hevea brasiliensis (Willd. ex A.Ju	Euphorbiaceae	0,53	5,7	6	8	0,04	0,00	10,29	0,01	0,12	0,44
5649	107	1	Sukamantri	>500	Huru keling	Litsea sp.	Lauraceae	0,425	27,0	9	14	0,04	0,06	241,14	0,24	2,83	10,40
5650	107	1	Sukamantri	>500	Mahoni	Swietenia macrophylla King	Meliaceae	0,61	16,2	4	8	0,04	0,02	78,88	0,08	0,93	3,40
5651	107	1	Sukamantri	>500	Mahoni	Swietenia macrophylla King	Meliaceae	0,61	13,0	4	9	0,04	0,01	58,91	0,06	0,69	2,54
5652	107	1	Sukamantri	>500	Manglid	Magnolia champaca (L.) Baill. ex	Magnoliaceae	0,53	7,6	4	5	0,04	0,00	11,33	0,01	0,13	0,49
5653	107	1	Sukamantri	>500	Sengon	Paraserianthes falcataria (L.) I.C.	Leguminosae / Fabaceae	0,33	4,5	4	5	0,04	0,00	2,74	0,00	0,03	0,12
5654	107	1	Sukamantri	>500	Manglid	Magnolia champaca (L.) Baill. ex	Magnoliaceae	0,53	13,7	12	11	0,04	0,01	67,92	0,07	0,80	2,93
5655	107	1	Sukamantri	>500	Manglid	Magnolia champaca (L.) Baill. ex	Magnoliaceae	0,53	9,5	7	8,5	0,04	0,01	27,73	0,03	0,33	1,20
5656	107	1	Sukamantri	>500	Huru keling	Litsea sp.	Lauraceae	0,425	15,9	10	11	0,04	0,02	73,14	0,07	0,86	3,15
5657	107	1	Sukamantri	>500	Karet	Hevea brasiliensis (Willd. ex A.Ju	Euphorbiaceae	0,53	5,7	3	5	0,04	0,00	6,69	0,01	0,08	0,29
5658	107	1	Sukamantri	>500	Karet	Hevea brasiliensis (Willd. ex A.Ju	Euphorbiaceae	0,53	5,7	3	5	0,04	0,00	6,69	0,01	0,08	0,29
5659	107	1	Sukamantri	>500	Manglid	Magnolia champaca (L.) Baill. ex	Magnoliaceae	0,53	22,0	10	14	0,04	0,04	201,46	0,20	2,37	8,69
5660	107	1	Sukamantri	>500	Manglid	Magnolia champaca (L.) Baill. ex	Magnoliaceae	0,53	19,1	12	15	0,04	0,03	166,12	0,17	1,95	7,16
5661	107	1	Sukamantri	>500	Karet	Hevea brasiliensis (Willd. ex A.Ju	Euphorbiaceae	0,53	4,1	2	4	0,04	0,00	3,00	0,00	0,04	0,13
5662	107	1	Sukamantri	>500	Manglid	Magnolia champaca (L.) Baill. ex	Magnoliaceae	0,53	19,4	11	15	0,04	0,03	171,23	0,17	2,01	7,38
5663	107	1	Sukamantri	>500	Karet	Hevea brasiliensis (Willd. ex A.Ju	Euphorbiaceae	0,53	9,5	4	7	0,04	0,01	23,21	0,02	0,27	1,00
5664	107	1	Sukamantri	>500	Manglid	Magnolia champaca (L.) Baill. ex	Magnoliaceae	0,53	11,5	8	10	0,04	0,01	44,95	0,04	0,53	1,94
5665	107	1	Sukamantri	>500	Manglid	Magnolia champaca (L.) Baill. ex	Magnoliaceae	0,53	18,5	11	14	0,04	0,03	146,56	0,15	1,72	6,32
5666	107	1	Sukamantri	>500	Karet	Hevea brasiliensis (Willd. ex A.Ju	Euphorbiaceae	0,53	5,4	2	5	0,04	0,00	6,03	0,01	0,07	0,26
5667	107	1	Sukamantri	>500	Huru keling	Litsea sp.	Lauraceae	0,425	16,9	10	12	0,04	0,02	88,13	0,09	1,04	3,80
5668	107	1	Sukamantri	>500	Manglid	Magnolia champaca (L.) Baill. ex	Magnoliaceae	0,53	12,1	9	11	0,04	0,01	54,15	0,05	0,64	2,34
5669	107	1	Sukamantri	>500	Karet	Hevea brasiliensis (Willd. ex A.Ju	Euphorbiaceae	0,53	5,7	3	4,5	0,04	0,00	6,08	0,01	0,07	0,26
5670	107	1	Sukamantri	>500	Sengon	Paraserianthes falcataria (L.) I.C.	Leguminosae / Fabaceae	0,33	12,7	10	12	0,04	0,01	41,74	0,04	0,49	1,80
5671	107	1	Sukamantri	>500	Mahoni	Swietenia macrophylla King	Meliaceae	0,61	10,5	3	6	0,04	0,01	27,30	0,03	0,32	1,18
5672	107	1	Sukamantri	>500	Karet	Hevea brasiliensis (Willd. ex A.Ju	Euphorbiaceae	0,53	8,0	4	8	0,04	0,00	18,79	0,02	0,22	0,81
5673	107	1	Sukamantri	>500	Manglid	Magnolia champaca (L.) Baill. ex	Magnoliaceae	0,53	14,6	9	11	0,04	0,02	76,85	0,08	0,90	3,31
5674	107	1	Sukamantri	>500	Manglid	Magnolia champaca (L.) Baill. ex	Magnoliaceae	0,53	13,7	12	14	0,04	0,01	84,71	0,08	1,00	3,65
5675	107	1	Sukamantri	>500	Manglid	Magnolia champaca (L.) Baill. ex	Magnoliaceae	0,53	11,8	6	10	0,04	0,01	47,26	0,05	0,56	2,04
5676	107	1	Sukamantri	>500	Karet	Hevea brasiliensis (Willd. ex A.Ju	Euphorbiaceae	0,53	5,4	6	8	0,04	0,00	9,27	0,01	0,11	0,40
5677	107	1	Sukamantri	>500	Karet	Hevea brasiliensis (Willd. ex A.Ju	Euphorbiaceae	0,53	8,9	4	7	0,04	0,01	20,46	0,02	0,24	0,88
5678	107	1	Sukamantri	>500	Manglid	Magnolia champaca (L.) Baill. ex	Magnoliaceae	0,53	19,4	11	14	0,04	0,03	160,74	0,16	1,89	6,93
5679	107	1	Sukamantri	>500	Karet	Hevea brasiliensis (Willd. ex A.Ju	Euphorbiaceae	0,53	4,1	2	5	0,04	0,00	3,69	0,00	0,04	0,16
5680	107	1	Sukamantri	>500	Manglid	Magnolia champaca (L.) Baill. ex	Magnoliaceae	0,53	21,6	12	15	0,04	0,04	208,94	0,21	2,45	9,01
5681	107	1	Sukamantri	>500	Manglid	Magnolia champaca (L.) Baill. ex	Magnoliaceae	0,53	19,1	12	15	0,04	0,03	166,12	0,17	1,95	7,16
5682	107	1	Sukamantri	>500	Huru keling	Litsea sp.	Lauraceae	0,425	19,1	9	14	0,04	0,03	127,39	0,13	1,50	5,49
5683	107	1	Sukamantri	>500	Puspa	Schima wallichii Choisy	Theaceae	0,74	23,2	3	12	0,04	0,04	263,31	0,26	3,09	11,35
5684	108	3	Sukamantri	>500	Ganitri	Elaeocarpus ganitrus Roxb. ex G.	Elaeocarpaceae	0,49	9,5	10	11	0,04	0,01	32,68	0,03	0,38	1,41
5685	108	3	Sukamantri	>500	Ganitri	Elaeocarpus ganitrus Roxb. ex G.	Elaeocarpaceae	0,49	14,3	12	13	0,04	0,02	80,06	0,08	0,94	3,45
5686	108	3	Sukamantri	>500	Ganitri	Elaeocarpus ganitrus Roxb. ex G.	Elaeocarpaceae	0,49	6,4	9	10	0,04	0,00	14,25	0,01	0,17	0,61
5687	108	3	Sukamantri	>500	Ganitri	Elaeocarpus ganitrus Roxb. ex G.	Elaeocarpaceae	0,49	12,4	12	13	0,04	0,01	61,59	0,06	0,72	2,66
5688	108	3	Sukamantri	>500	Puspa	Schima wallichii Choisy	Theaceae	0,74	12,4	11	12	0,04	0,01	83,50	0,08	0,98	3,60
5689	108	3	Sukamantri	>500	Ganitri	Elaeocarpus ganitrus Roxb. ex G.	Elaeocarpaceae	0,49	5,7	6	8	0,04	0,00	9,58	0,01	0,11	0,41
5690	108	3	Sukamantri	>500	Ganitri	Elaeocarpus ganitrus Roxb. ex G.	Elaeocarpaceae	0,49	3,2	4	5	0,04	0,00	2,12	0,00	0,02	0,09
5691	108	3	Sukamantri	>500	Afrika	Maesopsis eminii Engl.	Rhamnaceae	0,43	5,7	10	11	0,04	0,00	11,37	0,01	0,13	0,49
5692	108	3	Sukamantri	>500	Ganitri	Elaeocarpus ganitrus Roxb. ex G.	Elaeocarpaceae	0,49	4,8	7	8	0,04	0,00	6,86	0,01	0,08	0,30
5693	108	3	Sukamantri	>500	Ganitri	Elaeocarpus ganitrus Roxb. ex G.	Elaeocarpaceae	0,49	11,1	8	11	0,04	0,01	43,35	0,04	0,51	1,87
5694	108	3	Sukamantri	>500	Ganitri	Elaeocarpus ganitrus Roxb. ex G.	Elaeocarpaceae	0,49	5,7	6	7,5	0,04	0,00	9,03	0,01	0,11	0,39
5695	108	3	Sukamantri	>500	Cengkeh	Syzygium aromaticum (L.) Merr. &	Myrtaceae	0,79	12,7	7	10	0,04	0,01	78,58	0,08	0,92	3,39
5696	108	3	Sukamantri	>500	Ganitri	Elaeocarpus ganitrus Roxb. ex G.	Elaeocarpaceae	0,49	14,0	14	16	0,04	0,02	92,92	0,09	1,09	4,01
5697	108	3	Sukamantri	>500	Ganitri	Elaeocarpus ganitrus Roxb. ex G.	Elaeocarpaceae	0,49	4,5	6	7	0,04	0,00	5,35	0,01	0,06	0,23
5698	108	3	Sukamantri	>500	Ganitri	Elaeocarpus ganitrus Roxb. ex G.	Elaeocarpaceae	0,49	8,6	10	12	0,04	0,01	29,18	0,03	0,34	1,26

5699	108	3	Sukamantri	>500	Ganitri	Elaeocarpus ganitrus Roxb. ex G.	Elaeocarpaceae	0,49	14,0	13	15	0,04	0,02	87,59	0,09	1,03	3,78
5700	108	3	Sukamantri	>500	Ganitri	Elaeocarpus ganitrus Roxb. ex G.	Elaeocarpaceae	0,49	9,5	10	13	0,04	0,01	38,09	0,04	0,45	1,64
5701	108	3	Sukamantri	>500	Ganitri	Elaeocarpus ganitrus Roxb. ex G.	Elaeocarpaceae	0,49	8,0	7	8	0,04	0,00	17,48	0,02	0,21	0,75
5702	108	3	Sukamantri	>500	Ganitri	Elaeocarpus ganitrus Roxb. ex G.	Elaeocarpaceae	0,49	10,5	9	10	0,04	0,01	35,67	0,04	0,42	1,54
5703	108	3	Sukamantri	>500	Ganitri	Elaeocarpus ganitrus Roxb. ex G.	Elaeocarpaceae	0,49	9,9	10	12	0,04	0,01	37,59	0,04	0,44	1,62
5704	108	3	Sukamantri	>500	Ganitri	Elaeocarpus ganitrus Roxb. ex G.	Elaeocarpaceae	0,49	21,6	10	13	0,04	0,04	170,56	0,17	2,00	7,35
5705	108	3	Sukamantri	>500	Ganitri	Elaeocarpus ganitrus Roxb. ex G.	Elaeocarpaceae	0,49	8,9	10	12	0,04	0,01	31,19	0,03	0,37	1,35
5706	108	3	Sukamantri	>500	Afrika	Maesopsis eminii Engl.	Rhamnaceae	0,43	19,7	14	16	0,04	0,03	154,53	0,15	1,82	6,66
5707	108	3	Sukamantri	>500	Afrika	Maesopsis eminii Engl.	Rhamnaceae	0,43	23,9	13	15	0,04	0,04	206,44	0,21	2,43	8,90
5708	108	3	Sukamantri	>500	Afrika	Maesopsis eminii Engl.	Rhamnaceae	0,43	5,7	8	9	0,04	0,00	9,47	0,01	0,11	0,41
5709	108	3	Sukamantri	>500	Cengkeh	Syzygium aromaticum (L.) Merr. a	Myrtaceae	0,79	23,2	7	12	0,04	0,04	279,57	0,28	3,28	12,06
5710	108	3	Sukamantri	>500	Ganitri	Elaeocarpus ganitrus Roxb. ex G.	Elaeocarpaceae	0,49	12,1	13	14	0,04	0,01	62,86	0,06	0,74	2,71
5711	108	3	Sukamantri	>500	Ganitri	Elaeocarpus ganitrus Roxb. ex G.	Elaeocarpaceae	0,49	7,6	12	13	0,04	0,00	25,31	0,03	0,30	1,09
5712	108	3	Sukamantri	>500	Nangka	Artocarpus heterophyllus Lam.	Moraceae	0,61	5,1	3	6	0,04	0,00	7,25	0,01	0,09	0,31
5713	108	3	Sukamantri	>500	Ganitri	Elaeocarpus ganitrus Roxb. ex G.	Elaeocarpaceae	0,49	9,2	11	12	0,04	0,01	33,26	0,03	0,39	1,43
5714	108	3	Sukamantri	>500	Puspa	Schima wallichii Choisy	Theaceae	0,74	20,0	10	14	0,04	0,03	231,52	0,23	2,72	9,98
5715	108	3	Sukamantri	>500	Aren	Arenga pinnata (Wurmb) Merr.	Arecaceae	0,71	42,6	3	9	0,04	0,14	73,80	0,07	0,87	3,18
5716	108	3	Sukamantri	>500	Ganitri	Elaeocarpus ganitrus Roxb. ex G.	Elaeocarpaceae	0,49	13,7	10	12	0,04	0,01	68,45	0,07	0,80	2,95
5717	108	3	Sukamantri	>500	Cengkeh	Syzygium aromaticum (L.) Merr. a	Myrtaceae	0,79	11,5	6	10	0,04	0,01	64,79	0,06	0,76	2,79
5718	108	3	Sukamantri	>500	Afrika	Maesopsis eminii Engl.	Rhamnaceae	0,43	10,2	10	12	0,04	0,01	35,35	0,04	0,42	1,52
5719	108	3	Sukamantri	>500	Ganitri	Elaeocarpus ganitrus Roxb. ex G.	Elaeocarpaceae	0,49	5,1	6	7	0,04	0,00	6,83	0,01	0,08	0,29
5720	108	3	Sukamantri	>500	Ganitri	Elaeocarpus ganitrus Roxb. ex G.	Elaeocarpaceae	0,49	6,7	7	8,5	0,04	0,00	13,43	0,01	0,16	0,58
5721	108	3	Sukamantri	>500	Ganitri	Elaeocarpus ganitrus Roxb. ex G.	Elaeocarpaceae	0,49	10,2	8	9	0,04	0,01	30,61	0,03	0,36	1,32
5722	108	3	Sukamantri	>500	Afrika	Maesopsis eminii Engl.	Rhamnaceae	0,43	8,3	9	10	0,04	0,01	20,45	0,02	0,24	0,88
5723	108	3	Sukamantri	>500	Ganitri	Elaeocarpus ganitrus Roxb. ex G.	Elaeocarpaceae	0,49	9,5	9	10	0,04	0,01	29,95	0,03	0,35	1,29
5724	108	3	Sukamantri	>500	Ganitri	Elaeocarpus ganitrus Roxb. ex G.	Elaeocarpaceae	0,49	14,0	12	13,5	0,04	0,02	79,53	0,08	0,93	3,43
5725	108	3	Sukamantri	>500	Ganitri	Elaeocarpus ganitrus Roxb. ex G.	Elaeocarpaceae	0,49	14,0	12	13	0,04	0,02	76,83	0,08	0,90	3,31
5726	108	3	Sukamantri	>500	Ganitri	Elaeocarpus ganitrus Roxb. ex G.	Elaeocarpaceae	0,49	4,8	6	7	0,04	0,00	6,07	0,01	0,07	0,26
5727	108	3	Sukamantri	>500	Afrika	Maesopsis eminii Engl.	Rhamnaceae	0,43	8,3	10	11	0,04	0,01	22,31	0,02	0,26	0,96
5728	108	3	Sukamantri	>500	Ganitri	Elaeocarpus ganitrus Roxb. ex G.	Elaeocarpaceae	0,49	14,3	12	13	0,04	0,02	80,06	0,08	0,94	3,45
5729	108	3	Sukamantri	>500	Ganitri	Elaeocarpus ganitrus Roxb. ex G.	Elaeocarpaceae	0,49	8,0	10	11	0,04	0,00	23,40	0,02	0,27	1,01
5730	108	3	Sukamantri	>500	Puspa	Schima wallichii Choisy	Theaceae	0,74	19,7	9	15	0,04	0,03	239,50	0,24	2,81	10,33
5731	108	3	Sukamantri	>500	Ganitri	Elaeocarpus ganitrus Roxb. ex G.	Elaeocarpaceae	0,49	5,1	6	7	0,04	0,00	6,83	0,01	0,08	0,29
5732	108	3	Sukamantri	>500	Ganitri	Elaeocarpus ganitrus Roxb. ex G.	Elaeocarpaceae	0,49	6,4	7	8	0,04	0,00	11,62	0,01	0,14	0,50
5733	108	3	Sukamantri	>500	Ganitri	Elaeocarpus ganitrus Roxb. ex G.	Elaeocarpaceae	0,49	4,1	5	6	0,04	0,00	4,05	0,00	0,05	0,17
5734	108	3	Sukamantri	>500	Ganitri	Elaeocarpus ganitrus Roxb. ex G.	Elaeocarpaceae	0,49	4,5	5	6	0,04	0,00	4,64	0,00	0,05	0,20
5735	108	3	Sukamantri	>500	Ganitri	Elaeocarpus ganitrus Roxb. ex G.	Elaeocarpaceae	0,49	7,0	9	10	0,04	0,00	16,97	0,02	0,20	0,73
5736	108	3	Sukamantri	>500	Puspa	Schima wallichii Choisy	Theaceae	0,74	23,9	10	14	0,04	0,04	318,64	0,32	3,74	13,74
5737	108	3	Sukamantri	>500	Puspa	Schima wallichii Choisy	Theaceae	0,74	26,1	9	14	0,04	0,05	375,23	0,38	4,41	16,18
5738	108	3	Sukamantri	>500	Ganitri	Elaeocarpus ganitrus Roxb. ex G.	Elaeocarpaceae	0,49	6,0	8	9	0,04	0,00	11,78	0,01	0,14	0,51
5739	108	3	Sukamantri	>500	Ganitri	Elaeocarpus ganitrus Roxb. ex G.	Elaeocarpaceae	0,49	11,8	12	14	0,04	0,01	59,86	0,06	0,70	2,58
5740	108	3	Sukamantri	>500	Ganitri	Elaeocarpus ganitrus Roxb. ex G.	Elaeocarpaceae	0,49	6,0	7	8	0,04	0,00	10,57	0,01	0,12	0,46
5741	108	3	Sukamantri	>500	Cengkeh	Syzygium aromaticum (L.) Merr. a	Myrtaceae	0,79	22,3	6	10	0,04	0,04	219,06	0,22	2,57	9,45
5742	108	3	Sukamantri	>500	Nangka	Artocarpus heterophyllus Lam.	Moraceae	0,61	12,1	3	9	0,04	0,01	51,25	0,05	0,60	2,21
5743	108	3	Sukamantri	>500	Ganitri	Elaeocarpus ganitrus Roxb. ex G.	Elaeocarpaceae	0,49	8,3	10	11	0,04	0,01	25,15	0,03	0,30	1,08
5744	108	3	Sukamantri	>500	Ganitri	Elaeocarpus ganitrus Roxb. ex G.	Elaeocarpaceae	0,49	6,0	6	8	0,04	0,00	10,57	0,01	0,12	0,46
5745	108	3	Sukamantri	>500	Ganitri	Elaeocarpus ganitrus Roxb. ex G.	Elaeocarpaceae	0,49	11,8	11	13	0,04	0,01	55,93	0,06	0,66	2,41
5746	108	3	Sukamantri	>500	Ganitri	Elaeocarpus ganitrus Roxb. ex G.	Elaeocarpaceae	0,49	4,1	6	7	0,04	0,00	4,67	0,00	0,05	0,20
5747	108	3	Sukamantri	>500	Ganitri	Elaeocarpus ganitrus Roxb. ex G.	Elaeocarpaceae	0,49	7,0	7	8	0,04	0,00	13,83	0,01	0,16	0,60
5748	108	3	Sukamantri	>500	Ganitri	Elaeocarpus ganitrus Roxb. ex G.	Elaeocarpaceae	0,49	8,0	10	11	0,04	0,00	23,40	0,02	0,27	1,01
5749	108	3	Sukamantri	>500	Ganitri	Elaeocarpus ganitrus Roxb. ex G.	Elaeocarpaceae	0,49	10,2	11	12	0,04	0,01	39,84	0,04	0,47	1,72
5750	108	3	Sukamantri	>500	Ganitri	Elaeocarpus ganitrus Roxb. ex G.	Elaeocarpaceae	0,49	15,3	11	12,5	0,04	0,02	86,92	0,09	1,02	3,75
5751	108	3	Sukamantri	>500	Afrika	Maesopsis eminii Engl.	Rhamnaceae	0,43	7,0	16	11	0,04	0,00	16,43	0,02	0,19	0,71
5752	108	3	Sukamantri	>500	Cengkeh	Syzygium aromaticum (L.) Merr. a	Myrtaceae	0,79	15,3	5	10	0,04	0,02	109,74	0,11	1,29	4,73
5753	108	3	Sukamantri	>500	Ganitri	Elaeocarpus ganitrus Roxb. ex G.	Elaeocarpaceae	0,49	13,4	12	13	0,04	0,01	70,55	0,07	0,83	3,04
5754	108	3	Sukamantri	>500	Ganitri	Elaeocarpus ganitrus Roxb. ex G.	Elaeocarpaceae	0,49	17,2	13	14	0,04	0,02	119,66	0,12	1,41	5,16
5755	108	3	Sukamantri	>500	Ganitri	Elaeocarpus ganitrus Roxb. ex G.	Elaeocarpaceae	0,49	8,0	7	8	0,04	0,00	17,48	0,02	0,21	0,75
5756	108	3	Sukamantri	>500	Wangkal	Albizia procera Benth	Leguminosae / Fabaceae	0,77	10,2	4	7	0,04	0,01	36,79	0,04	0,43	1,59
5757	108	3	Sukamantri	>500	Ganitri	Elaeocarpus ganitrus Roxb. ex G.	Elaeocarpaceae	0,49	14,3	12	13	0,04	0,02	80,06	0,08	0,94	3,45
5758	108	3	Sukamantri	>500	Afrika	Maesopsis eminii Engl.	Rhamnaceae	0,43	6,4	7	8	0,04	0,00	10,31	0,01	0,12	0,44
5759	108	3	Sukamantri	>500	Ganitri	Elaeocarpus ganitrus Roxb. ex G.	Elaeocarpaceae	0,49	18,8	13	14	0,04	0,03	140,73	0,14	1,65	6,07
5760	108	3	Sukamantri	>500	Ganitri	Elaeocarpus ganitrus Roxb. ex G.	Elaeocarpaceae	0,49	20,4	13	14	0,04	0,03	163,35	0,16	1,92	7,04
5761	108	3	Sukamantri	>500	Ganitri	Elaeocarpus ganitrus Roxb. ex G.	Elaeocarpaceae	0,49	17,5	13	14	0,04	0,02	123,75	0,12	1,45	5,34
5762	108	3	Sukamantri	>500	Afrika	Maesopsis eminii Engl.	Rhamnaceae	0,43	4,8	5	6	0,04	0,00	4,67	0,00	0,05	0,20
5763	108	3	Sukamantri	>500	Ganitri	Elaeocarpus ganitrus Roxb. ex G.	Elaeocarpaceae	0,49	6,7	6	7	0,04	0,00	11,24	0,01	0,13	0,48
5764	108	3	Sukamantri	>500	Afrika	Maesopsis eminii Engl.	Rhamnaceae	0,43	14,0	10	11	0,04	0,02	58,49	0,06	0,69	2,52
5765	108	3	Sukamantri	>500	Ganitri	Elaeocarpus ganitrus Roxb. ex G.	Elaeocarpaceae	0,49	6,4	8	9	0,04	0,00	12,94	0,01	0,15	0,56
5766	108	3	Sukamantri	>500	Ganitri	Elaeocarpus ganitrus Roxb. ex G.	Elaeocarpaceae	0,49	8,3	8	9	0,04	0,01	20,92	0,02	0,25	0,90
5767	108	3	Sukamantri	>500	Ganitri	Elaeocarpus ganitrus Roxb. ex G.	Elaeocarpaceae	0,49	15,6	11	12	0,04	0,02	86,96	0,09	1,02	3,75
5768	108	3	Sukamantri	>500	Ganitri	Elaeocarpus ganitrus Roxb. ex G.	Elaeocarpaceae	0,49	8,3	8	9	0,04	0,01	20,92	0,02	0,25	0,90
5769	108	3	Sukamantri	>500	Ganitri	Elaeocarpus ganitrus Roxb. ex G.	Elaeocarpaceae	0,49	13,4	12	13	0,04	0,01	70,55	0,07	0,83	3,04
5770	108	3	Sukamantri	>500	Ganitri	Elaeocarpus ganitrus Roxb. ex G.	Elaeocarpaceae	0,49	15,3	12	13	0,04	0,02	90,10	0,09	1,06	3,89
5771	108	3	Sukamantri	>500	Ganitri	Elaeocarpus ganitrus Roxb. ex G.	Elaeocarpaceae	0,49	12,1	11	12	0,04	0,01	54,58	0,05	0,64	2,35
5772	108	3	Sukamantri	>500	Cengkeh	Syzygium aromaticum (L.) Merr. a	Myrtaceae	0,79	18,1	5	11	0,04	0,03	164,07	0,16	1,93	7,08
5773	108	3	Sukamantri	>500	Ganitri	Elaeocarpus ganitrus Roxb. ex G.	Elaeocarpaceae	0,49	7,0	6	7	0,04	0,00	12,24	0,01	0,14	0,53
5774	108	3	Sukamantri	>500	Ganitri	Elaeocarpus ganitrus Roxb. ex G.	Elaeocarpaceae	0,49	9,2	8	9	0,04	0,01	25,56	0,03	0,30	1,10
5775	108	3	Sukamantri	>500	Ganitri	Elaeocarpus ganitrus Roxb. ex G.	Elaeocarpaceae	0,49	4,5	5	6	0,04	0,00	4,64	0,00	0,05	0,20
5776	108	3	Sukamantri	>500	Ganitri	Elaeocarpus ganitrus Roxb. ex G.	Elaeocarpaceae	0,49	8,0	11	12	0,04	0,00	25,35	0,03	0,30	1,09
5777	108	3	Sukamantri	>500	Pinang	Areca catechu L.	Arecaceae	0,66	4,5	2	4	0,04	0,00	4,21	0,00	0,05	0,18
5778	108	3	Sukamantri	>500	Aren	Arenga pinnata (Wurmb) Merr.	Arecaceae	0,71	36,9	3	9	0,04	0,11	73,80	0,07	0,87	3,18
5779	108	3	Sukamantri	>500	Ganitri	Elaeocarpus ganitrus Roxb. ex G.	Elaeocarpaceae	0,49	10,5	11	12	0,04	0,01	42,15	0,04	0,50	1,82
5780	108	3	Sukamantri	>500	Ganitri	Elaeocarpus ganitrus Roxb. ex G.	Elaeocarpaceae	0,49	11,8	12	13	0,04	0,01	55,93	0,06	0,66	2,41
5781	108	3	Sukamantri	>500	Ganitri	Elaeocarpus ganitrus Roxb. ex G.	Elaeocarpaceae	0,49	10,2	6	9	0,04	0,01	30,61	0,03	0,36	1,32
5782	108	3	Sukamantri	>500	Ganitri	Elaeocarpus ganitrus Roxb. ex G.	Elaeocarpaceae	0,49	15,3	12	13	0,04	0,02	90,10	0,09	1,06	3,89
5783	108	3	Sukamantri	>500	Ganitri	Elaeocarpus ganitrus Roxb. ex G.	Elaeocarpaceae	0,49	12,7	12	13	0,04	0,01	64,52	0,06	0,76	2,78
5784	108	3	Sukamantri	>500	Ganitri	Elaeocarpus ganitrus Roxb. ex G.	Elaeocarpaceae	0,49	15,3	12	13	0,04	0,02	90,10	0,09	1,06	3,89
5785	109	1	Sukamantri	>500	Akasia mangium	Acacia mangium Willd.	Leguminosae / Fabaceae	0,5	48,0	9	14	0,04	0,18	801,89	0,80	9,42	34,58
5786	109	1	Sukamantri	>500	Akasia mangium	Acacia mangium Willd.	Leguminosae / Fabaceae	0,5	21,0	10	14	0,04	0,03	176,05	0,18	2,07	7,59
5787	109	1	Sukamantri	>500	Akasia mangium	Acacia mangium Willd.	Leguminosae / Fabaceae	0,5	39,8	9	15	0,04	0,12	604,25	0,60	7,10	26,06
5788	109	1	Sukamantri	>500	Gmelina	Gmelina arborea Roxb.	Lamiaceae / Verbenaceae	0,47	10,8	6	7,5	0,04	0,01	27,86	0,03	0,33	1,20
5789	109	1	Sukamantri	>500	Nangka	Artocarpus heterophyllus Lam.	Moraceae	0,61	6,0	2	6	0,04	0,00	9,93	0,01	0,12	0,43
5790	109	1	Sukamantri	>500	Akasia mangium	Acacia mangium Willd.	Leguminosae / Fabaceae	0,5	12,1	10	14	0,04	0,01	64,03	0,06	0,75	2,76
5791	109	1	Sukamantri	>500	Akasia mangium	Acacia mangium Willd.	Leguminosae / Fabaceae	0,5	37,9	10	16	0,04	0,11	585,80	0,59	6,88	25,26
5792	109	1	Sukamantri	>500	Akasia mangium	Acacia mangium Willd.	Leguminosae / Fabaceae	0,5	16,9	5	8	0,04	0,02	70,55	0,07	0,83	3,04
5793	109	1	Sukamantri	>500	Nangka	Artocarpus heterophyllus Lam.	Moraceae	0,61	4,8	2	6	0,04	0,00	6,44	0,01	0,08	0,28
5794	109	1	Sukamantri	>500	Akasia mangium	Acacia mangium Willd.	Leguminosae / Fabaceae	0,5	31,2	9	14	0,04	0,08	363,21	0,36	4,27	15,66
5795	109	1	Sukamantri	>500	Akasia mangium	Acacia mangium Willd.	Leguminosae / Fabaceae	0,5	9,2	6	9	0,04	0,01	26,04	0,03	0,31	1,12
5796	109	1	Sukamantri	>500	Akasia mangium	Acacia mangium Willd.	Leguminosae / Fabaceae	0,5	26,4	12	15	0,04	0,05	285,38	0,29	3,35	12,31
5797	109	1	Sukamantri	>500	Akasia mangium	Acacia mangium Willd.	Leguminosae / Fabaceae	0,5	12,7	6	10	0,04	0,01	51,68	0,05	0,61	2,23
5798	109	1	Sukamantri	>500	Akasia mangium	Acacia mangium Willd.	Leguminosae / Fabaceae	0,5	19,7	7	11	0,04	0,03	125,88	0,13	1,48	5,43
5799	109	1	Sukamantri	>500	Akasia mangium	Acacia mangium Willd.	Leguminosae / Fabaceae	0,5	14,0	6	10	0,04	0,02	61,54	0,06	0,72	2,65
5800	109	1	Sukamantri	>500	Akasia mangium	Acacia mangium Willd.	Leguminosae / Fabaceae	0,5	32,5	12	16	0,04	0,08	441,68	0,44	5,19	19,05
5801	109	1	Sukamantri	>500	Akasia mangium	Acacia mangium Willd.	Leguminosae / Fabaceae	0,5	25,1	11	16	0,04	0,05	276,57	0,28	3,25	11,93
5802	109	1	Sukamantri	>500	Akasia mangium	Acacia mangium Willd.	Leguminosae / Fabaceae	0,5	17,8	9	16	0,04	0,02	147,24	0,15	1,73	6,35
5803	109	1	Sukamantri	>500	Akasia mangium	Acacia mangium Willd.	Leguminosae / Fabaceae	0,5	13,4	11	15	0,04	0,01	81,93	0,08	0,96	3,53
5804	109	1	Sukamantri	>500	Akasia mangium	Acacia mangium Willd.	Leguminosae / Fabaceae	0,5	13,7	11	14	0,04	0,01	80,31	0,08	0,94	3,46
5805	109	1	Sukamantri	>500	Akasia mangium	Acacia mangium Willd.	Leguminosae / Fabaceae	0,5	35,0	12	18	0,04	0,10	564,98	0,56	6,64	24,36
5806	109	1	Sukamantri	>500	Akasia mangium	Acacia mangium Willd.	Leguminosae / Fabaceae	0,5	23,9	11	16	0,04	0,04	251,46	0,25	2,95	10,84
5807	109	1	Sukamantri	>500	Akasia mangium	Acacia mangium Willd.	Leguminosae / Fabaceae	0,5	15,9	8	12	0,04	0,02	91,92	0,09	1,08	3,96
5808	109	1	Sukamantri	>500	Akasia mangium	Acacia mangium Willd.	Leguminosae / Fabaceae	0,5	13,4	6	8	0,04	0,01	46,07	0,05	0,54	1,99
5809	109	1	Sukamantri	>500	Akasia mangium	Acacia mangium Willd.	Leguminosae / Fabaceae	0,5	26,4	11	17	0,04	0,05	320,05	0,32	3,76	13,80
5810	109	1	Sukamantri	>500	Akasia mangium	Acacia mangium Willd.	Leguminosae / Fabaceae	0,5	16,5	6	9	0,04	0,02	75,89	0,08	0,89	3,27
5811	109	1	Sukamantri	>500	Akasia mangium	Acacia mangium Willd.	Leguminosae / Fabaceae	0,5	26,1	13	17	0,04	0,05	313,02	0,31	3,68	13,50
5812	109	1	Sukamantri	>500	Akasia mangium	Acacia mangium Willd.	Leguminosae / Fabaceae	0,5	32,8	10	16	0,04	0,08	449,64	0,45	5,28	19,39
5813	109	1	Sukamantri	>500	Akasia mangium	Acacia mangium Willd.	Leguminosae / Fabaceae	0,5	41,7	10	15	0,04	0,14	658,44	0,66	7,74	28,39
5814	110	1	Sukamantri	>500	Afrika	Maesopsis eminii Engl.	Rhamnaceae	0,43	15,3	10	11	0,04	0,02	68,60	0,07	0,81	2,96
5815	110	1	Sukamantri	>500	Afrika	Maesopsis eminii Engl.	Rhamnaceae	0,43	13,0	9	10	0,04	0,01	47,10	0,05	0,55	2,03
5816	110	1	Sukamantri	>500	Afrika	Maesopsis eminii Engl.	Rhamnaceae	0,43	10,8	5	6	0,04	0,01	20,93	0,02	0,25	0,90
5817	110	1	Sukamantri	>500	Afrika	Maesopsis eminii Engl.	Rhamnaceae	0,43	18,8	8	9	0,04	0,03	83,30	0,08	0,98	3,59
5818	110	1	Sukamantri	>500	Afrika	Maesopsis eminii Engl.	Rhamnaceae	0,43	15,6	8	9	0,04	0,02	59,28	0,06	0,70	2,56
5819	110	1	Sukamantri	>500	Afrika	Maesopsis eminii Engl.	Rhamnaceae	0,43	4,8	4	6	0,04	0,00	4,67	0,00	0,05	0,20
5820	110	1	Sukamantri	>500	Gmelina	Gmelina arborea Roxb.	Lamiaceae / Verbenaceae	0,47	14,3	9	11	0,04	0,02	66,12	0,07	0,78	2,85
5821	110	1	Sukamantri	>500	Afrika	Maesopsis eminii Engl.	Rhamnaceae	0,43	6,4	5	6	0,04	0,00	7,92	0,01	0,09	0,34
5822	110	1	Sukamantri	>500	Afrika	Maesopsis eminii Engl.	Rhamnaceae	0,43	8,6	5	6	0,04	0,01	13,72	0,01	0,16	0,59

5823	110	1	Sukamantri	>500	Gmelina	Gmelina arborea Roxb.	Lamiaceae / Verbenaceae	0,47	12,1	5	8	0,04	0,01	36,24	0,04	0,43	1,56
5824	110	1	Sukamantri	>500	Afrika	Maesopsis eminii Engl.	Rhamnaceae	0,43	4,8	5	6	0,04	0,00	4,67	0,00	0,05	0,20
5825	110	1	Sukamantri	>500	Afrika	Maesopsis eminii Engl.	Rhamnaceae	0,43	14,3	10	11	0,04	0,02	60,95	0,06	0,72	2,63
5826	110	1	Sukamantri	>500	Afrika	Maesopsis eminii Engl.	Rhamnaceae	0,43	14,3	9	10	0,04	0,02	55,85	0,06	0,66	2,41
5827	110	1	Sukamantri	>500	Afrika	Maesopsis eminii Engl.	Rhamnaceae	0,43	7,0	7	8	0,04	0,00	12,27	0,01	0,14	0,53
5828	110	1	Sukamantri	>500	Afrika	Maesopsis eminii Engl.	Rhamnaceae	0,43	7,0	7	8	0,04	0,00	12,27	0,01	0,14	0,53
5829	110	1	Sukamantri	>500	Afrika	Maesopsis eminii Engl.	Rhamnaceae	0,43	8,6	7	8	0,04	0,01	17,86	0,02	0,21	0,77
5830	110	1	Sukamantri	>500	Afrika	Maesopsis eminii Engl.	Rhamnaceae	0,43	7,3	7	8	0,04	0,00	13,31	0,01	0,16	0,57
5831	110	1	Sukamantri	>500	Afrika	Maesopsis eminii Engl.	Rhamnaceae	0,43	13,4	7	8	0,04	0,01	40,12	0,04	0,47	1,73
5832	110	1	Sukamantri	>500	Afrika	Maesopsis eminii Engl.	Rhamnaceae	0,43	15,9	8	9	0,04	0,02	61,51	0,06	0,72	2,65
5833	110	1	Sukamantri	>500	Afrika	Maesopsis eminii Engl.	Rhamnaceae	0,43	12,1	8	9	0,04	0,01	37,21	0,04	0,44	1,60
5834	110	1	Sukamantri	>500	Afrika	Maesopsis eminii Engl.	Rhamnaceae	0,43	14,3	7	8	0,04	0,02	45,53	0,05	0,53	1,96
5835	110	1	Sukamantri	>500	Afrika	Maesopsis eminii Engl.	Rhamnaceae	0,43	6,4	6	7	0,04	0,00	9,12	0,01	0,11	0,39
5836	110	1	Sukamantri	>500	Afrika	Maesopsis eminii Engl.	Rhamnaceae	0,43	7,0	6	7	0,04	0,00	10,86	0,01	0,13	0,47
5837	110	1	Sukamantri	>500	Afrika	Maesopsis eminii Engl.	Rhamnaceae	0,43	12,1	8	9	0,04	0,01	37,21	0,04	0,44	1,60
5838	110	1	Sukamantri	>500	Afrika	Maesopsis eminii Engl.	Rhamnaceae	0,43	17,5	8	9	0,04	0,02	73,25	0,07	0,86	3,16
5839	110	1	Sukamantri	>500	Afrika	Maesopsis eminii Engl.	Rhamnaceae	0,43	9,2	6	7	0,04	0,01	18,01	0,02	0,21	0,78
5840	110	1	Sukamantri	>500	Afrika	Maesopsis eminii Engl.	Rhamnaceae	0,43	5,7	5	6	0,04	0,00	6,53	0,01	0,08	0,28
5841	110	1	Sukamantri	>500	Afrika	Maesopsis eminii Engl.	Rhamnaceae	0,43	8,3	6	7	0,04	0,01	14,75	0,01	0,17	0,64
5842	110	1	Sukamantri	>500	Afrika	Maesopsis eminii Engl.	Rhamnaceae	0,43	7,0	5	6	0,04	0,00	9,43	0,01	0,11	0,41
5843	110	1	Sukamantri	>500	Afrika	Maesopsis eminii Engl.	Rhamnaceae	0,43	20,4	10	11	0,04	0,03	116,20	0,12	1,37	5,01
5844	110	1	Sukamantri	>500	Afrika	Maesopsis eminii Engl.	Rhamnaceae	0,43	12,4	9	10	0,04	0,01	42,97	0,04	0,50	1,85
5845	110	1	Sukamantri	>500	Afrika	Maesopsis eminii Engl.	Rhamnaceae	0,43	6,0	5	6	0,04	0,00	7,21	0,01	0,08	0,31
5846	110	1	Sukamantri	>500	Afrika	Maesopsis eminii Engl.	Rhamnaceae	0,43	8,6	4	5	0,04	0,01	11,61	0,01	0,14	0,50
5847	110	1	Sukamantri	>500	Afrika	Maesopsis eminii Engl.	Rhamnaceae	0,43	15,6	7	8	0,04	0,02	53,22	0,05	0,63	2,29
5848	110	1	Sukamantri	>500	Afrika	Maesopsis eminii Engl.	Rhamnaceae	0,43	5,4	4	5	0,04	0,00	4,98	0,00	0,06	0,21
5849	110	1	Sukamantri	>500	Afrika	Maesopsis eminii Engl.	Rhamnaceae	0,43	3,8	3	4	0,04	0,00	2,14	0,00	0,03	0,09
5850	110	1	Sukamantri	>500	Afrika	Maesopsis eminii Engl.	Rhamnaceae	0,43	4,5	3	4	0,04	0,00	2,84	0,00	0,03	0,12
5851	110	1	Sukamantri	>500	Afrika	Maesopsis eminii Engl.	Rhamnaceae	0,43	11,5	8	9	0,04	0,01	33,70	0,03	0,40	1,45
5852	110	1	Sukamantri	>500	Afrika	Maesopsis eminii Engl.	Rhamnaceae	0,43	13,7	8	9	0,04	0,01	46,66	0,05	0,55	2,01
5853	110	1	Sukamantri	>500	Afrika	Maesopsis eminii Engl.	Rhamnaceae	0,43	13,0	8	9	0,04	0,01	42,76	0,04	0,50	1,84
5854	110	1	Sukamantri	>500	Afrika	Maesopsis eminii Engl.	Rhamnaceae	0,43	12,1	10	11	0,04	0,01	44,72	0,04	0,53	1,93
5855	110	1	Sukamantri	>500	Afrika	Maesopsis eminii Engl.	Rhamnaceae	0,43	5,1	6	7	0,04	0,00	6,06	0,01	0,07	0,26
5856	110	1	Sukamantri	>500	Afrika	Maesopsis eminii Engl.	Rhamnaceae	0,43	8,0	7	8	0,04	0,00	15,51	0,02	0,18	0,67
5857	110	1	Sukamantri	>500	Afrika	Maesopsis eminii Engl.	Rhamnaceae	0,43	4,1	4	5	0,04	0,00	3,04	0,00	0,04	0,13
5858	110	1	Sukamantri	>500	Afrika	Maesopsis eminii Engl.	Rhamnaceae	0,43	10,8	7	8	0,04	0,01	27,24	0,03	0,32	1,17
5859	110	1	Sukamantri	>500	Afrika	Maesopsis eminii Engl.	Rhamnaceae	0,43	12,1	10	11	0,04	0,01	44,72	0,04	0,53	1,93
5860	110	1	Sukamantri	>500	Afrika	Maesopsis eminii Engl.	Rhamnaceae	0,43	12,7	10	11	0,04	0,01	49,12	0,05	0,58	2,12
5861	110	1	Sukamantri	>500	Afrika	Maesopsis eminii Engl.	Rhamnaceae	0,43	14,3	10	11	0,04	0,02	60,95	0,06	0,72	2,63
5862	110	1	Sukamantri	>500	Afrika	Maesopsis eminii Engl.	Rhamnaceae	0,43	7,0	7	8	0,04	0,00	12,27	0,01	0,14	0,53
5863	110	1	Sukamantri	>500	Afrika	Maesopsis eminii Engl.	Rhamnaceae	0,43	10,5	10	11	0,04	0,01	34,53	0,03	0,41	1,49
5864	110	1	Sukamantri	>500	Afrika	Maesopsis eminii Engl.	Rhamnaceae	0,43	9,5	9	10	0,04	0,01	26,57	0,03	0,31	1,15
5865	110	1	Sukamantri	>500	Afrika	Maesopsis eminii Engl.	Rhamnaceae	0,43	12,1	9	10	0,04	0,01	40,98	0,04	0,48	1,77
5866	110	1	Sukamantri	>500	Puspa	Schima wallichii Choisy	Theaceae	0,74	5,1	2	4	0,04	0,00	5,97	0,01	0,07	0,26
5867	110	1	Sukamantri	>500	Aren	Arenga pinnata (Wurmb) Merr.	Arecaceae	0,71	52,5	4	10	0,04	0,22	81,50	0,08	0,96	3,51
5868	110	1	Sukamantri	>500	Afrika	Maesopsis eminii Engl.	Rhamnaceae	0,43	13,0	10	11	0,04	0,01	51,39	0,05	0,60	2,22
5869	110	1	Sukamantri	>500	Afrika	Maesopsis eminii Engl.	Rhamnaceae	0,43	8,9	8	9	0,04	0,01	21,26	0,02	0,25	0,92
5870	110	1	Sukamantri	>500	Afrika	Maesopsis eminii Engl.	Rhamnaceae	0,43	9,2	8	9	0,04	0,01	22,68	0,02	0,27	0,98
5871	110	1	Sukamantri	>500	Afrika	Maesopsis eminii Engl.	Rhamnaceae	0,43	4,1	10	11	0,04	0,00	6,27	0,01	0,07	0,27
5872	110	1	Sukamantri	>500	Afrika	Maesopsis eminii Engl.	Rhamnaceae	0,43	7,3	10	11	0,04	0,00	17,82	0,02	0,21	0,77
5873	110	1	Sukamantri	>500	Puspa	Schima wallichii Choisy	Theaceae	0,74	4,8	1	4	0,04	0,00	5,30	0,01	0,06	0,23
5874	110	1	Sukamantri	>500	Afrika	Maesopsis eminii Engl.	Rhamnaceae	0,43	6,4	5	6	0,04	0,00	7,92	0,01	0,09	0,34
5875	110	1	Sukamantri	>500	Afrika	Maesopsis eminii Engl.	Rhamnaceae	0,43	9,5	6	7	0,04	0,01	19,17	0,02	0,23	0,83
5876	110	1	Sukamantri	>500	Afrika	Maesopsis eminii Engl.	Rhamnaceae	0,43	9,5	9	10	0,04	0,01	26,57	0,03	0,31	1,15
5877	110	1	Sukamantri	>500	Afrika	Maesopsis eminii Engl.	Rhamnaceae	0,43	7,0	4	5	0,04	0,00	7,98	0,01	0,09	0,34
5878	110	1	Sukamantri	>500	Afrika	Maesopsis eminii Engl.	Rhamnaceae	0,43	9,2	10	11	0,04	0,01	27,25	0,03	0,32	1,18
5879	110	1	Sukamantri	>500	Afrika	Maesopsis eminii Engl.	Rhamnaceae	0,43	15,6	11	12	0,04	0,02	77,15	0,08	0,91	3,33
5880	110	1	Sukamantri	>500	Afrika	Maesopsis eminii Engl.	Rhamnaceae	0,43	8,0	9	9	0,04	0,00	17,28	0,02	0,20	0,75
5881	110	1	Sukamantri	>500	Afrika	Maesopsis eminii Engl.	Rhamnaceae	0,43	7,0	8	9	0,04	0,00	13,67	0,01	0,16	0,59
5882	110	1	Sukamantri	>500	Afrika	Maesopsis eminii Engl.	Rhamnaceae	0,43	7,0	9	10	0,04	0,00	15,06	0,02	0,18	0,65
5883	110	1	Sukamantri	>500	Afrika	Maesopsis eminii Engl.	Rhamnaceae	0,43	4,5	6	7	0,04	0,00	4,74	0,00	0,06	0,20
5884	110	1	Sukamantri	>500	Afrika	Maesopsis eminii Engl.	Rhamnaceae	0,43	12,4	10	11	0,04	0,01	46,89	0,05	0,55	2,02
5885	110	1	Sukamantri	>500	Afrika	Maesopsis eminii Engl.	Rhamnaceae	0,43	12,7	10	11	0,04	0,01	49,12	0,05	0,58	2,12
5886	110	1	Sukamantri	>500	Afrika	Maesopsis eminii Engl.	Rhamnaceae	0,43	12,7	10	11	0,04	0,01	49,12	0,05	0,58	2,12
5887	110	1	Sukamantri	>500	Afrika	Maesopsis eminii Engl.	Rhamnaceae	0,43	6,4	5	6	0,04	0,00	7,92	0,01	0,09	0,34
5888	111	1	Sukamantri	>500	Afrika	Maesopsis eminii Engl.	Rhamnaceae	0,43	12,4	8	9	0,01	0,00	39,02	0,04	1,83	6,73
5889	111	1	Sukamantri	>500	Afrika	Maesopsis eminii Engl.	Rhamnaceae	0,43	8,9	8	9	0,01	0,00	21,26	0,02	1,00	3,67
5890	111	1	Sukamantri	>500	Afrika	Maesopsis eminii Engl.	Rhamnaceae	0,43	8,9	8	9	0,01	0,00	21,26	0,02	1,00	3,67
5891	111	1	Sukamantri	>500	Afrika	Maesopsis eminii Engl.	Rhamnaceae	0,43	7,3	8	9	0,01	0,00	14,83	0,01	0,70	2,56
5892	111	1	Sukamantri	>500	Afrika	Maesopsis eminii Engl.	Rhamnaceae	0,43	9,2	9	10	0,01	0,00	24,97	0,02	1,17	4,31
5893	111	1	Sukamantri	>500	Afrika	Maesopsis eminii Engl.	Rhamnaceae	0,43	7,0	8	9	0,01	0,00	13,67	0,01	0,64	2,36
5894	111	1	Sukamantri	>500	Afrika	Maesopsis eminii Engl.	Rhamnaceae	0,43	13,7	10	11	0,01	0,00	56,08	0,06	2,64	9,67
5895	111	1	Sukamantri	>500	Afrika	Maesopsis eminii Engl.	Rhamnaceae	0,43	14,3	10	11	0,01	0,00	60,95	0,06	2,86	10,51
5896	111	1	Sukamantri	>500	Afrika	Maesopsis eminii Engl.	Rhamnaceae	0,43	16,5	10	11	0,01	0,01	79,43	0,08	3,73	13,70
5897	111	1	Sukamantri	>500	Afrika	Maesopsis eminii Engl.	Rhamnaceae	0,43	13,0	9	10	0,01	0,00	47,10	0,05	2,21	8,12
5898	111	1	Sukamantri	>500	Afrika	Maesopsis eminii Engl.	Rhamnaceae	0,43	13,4	9	10	0,01	0,00	49,22	0,05	2,31	8,49
5899	111	1	Sukamantri	>500	Afrika	Maesopsis eminii Engl.	Rhamnaceae	0,43	8,0	7	8	0,01	0,00	15,51	0,02	0,73	2,68
5900	111	1	Sukamantri	>500	Afrika	Maesopsis eminii Engl.	Rhamnaceae	0,43	4,5	6	7	0,01	0,00	4,74	0,00	0,22	0,82
5901	111	1	Sukamantri	>500	Afrika	Maesopsis eminii Engl.	Rhamnaceae	0,43	12,4	8	9	0,01	0,00	39,02	0,04	1,83	6,73
5902	111	1	Sukamantri	>500	Afrika	Maesopsis eminii Engl.	Rhamnaceae	0,43	9,5	6	7	0,01	0,00	19,17	0,02	0,90	3,31
5903	111	1	Sukamantri	>500	Afrika	Maesopsis eminii Engl.	Rhamnaceae	0,43	9,5	7	8	0,01	0,00	21,66	0,02	1,02	3,74
5904	111	1	Sukamantri	>500	Afrika	Maesopsis eminii Engl.	Rhamnaceae	0,43	6,4	7	8	0,01	0,00	10,31	0,01	0,48	1,78
5905	111	1	Sukamantri	>500	Afrika	Maesopsis eminii Engl.	Rhamnaceae	0,43	8,6	8	9	0,01	0,00	19,89	0,02	0,94	3,43
5906	111	1	Sukamantri	>500	Afrika	Maesopsis eminii Engl.	Rhamnaceae	0,43	11,8	8	9	0,01	0,00	35,43	0,04	1,67	6,11
5907	111	1	Sukamantri	>500	Puspa	Schima wallichii Choisy	Theaceae	0,74	31,2	9	13	0,04	0,08	486,00	0,49	5,71	20,96
5908	111	1	Sukamantri	>500	Puspa	Schima wallichii Choisy	Theaceae	0,74	32,8	6	12	0,04	0,08	494,75	0,49	5,81	21,33
5909	111	1	Sukamantri	>500	Puspa	Schima wallichii Choisy	Theaceae	0,74	24,2	7	11	0,04	0,05	261,76	0,26	3,08	11,29
5910	112	2	Sukamantri	>500	Ganitri	Elaeocarpus ganitrus Roxb. ex G.	Elaeocarpaceae	0,49	9,2	8	9	0,01	0,00	25,56	0,03	1,20	4,41
5911	112	2	Sukamantri	>500	Suren	Toona sureni (Blume) Merr.	Meliaceae	0,39	33,1	6	11	0,01	0,02	258,62	0,26	12,15	44,61
5912	112	2	Sukamantri	>500	Ganitri	Elaeocarpus ganitrus Roxb. ex G.	Elaeocarpaceae	0,49	8,0	6	7	0,01	0,00	15,47	0,02	0,73	2,67
5913	112	2	Sukamantri	>500	Ganitri	Elaeocarpus ganitrus Roxb. ex G.	Elaeocarpaceae	0,49	8,0	7	8	0,01	0,00	17,48	0,02	0,82	3,02
5914	112	2	Sukamantri	>500	Ganitri	Elaeocarpus ganitrus Roxb. ex G.	Elaeocarpaceae	0,49	12,4	9	10	0,01	0,00	48,44	0,05	2,28	8,35
5915	112	2	Sukamantri	>500	Ganitri	Elaeocarpus ganitrus Roxb. ex G.	Elaeocarpaceae	0,49	8,0	6	7	0,01	0,00	15,47	0,02	0,73	2,67
5916	112	2	Sukamantri	>500	Ganitri	Elaeocarpus ganitrus Roxb. ex G.	Elaeocarpaceae	0,49	5,4	6	7	0,01	0,00	7,63	0,01	0,36	1,32
5917	112	2	Sukamantri	>500	Ganitri	Elaeocarpus ganitrus Roxb. ex G.	Elaeocarpaceae	0,49	11,5	8	9	0,01	0,00	37,98	0,04	1,79	6,55
5918	112	2	Sukamantri	>500	Ganitri	Elaeocarpus ganitrus Roxb. ex G.	Elaeocarpaceae	0,49	4,8	5	6	0,01	0,00	5,27	0,01	0,25	0,91
5919	112	2	Sukamantri	>500	Ganitri	Elaeocarpus ganitrus Roxb. ex G.	Elaeocarpaceae	0,49	12,4	9	10	0,01	0,00	48,44	0,05	2,28	8,35
5920	112	2	Sukamantri	>500	Ganitri	Elaeocarpus ganitrus Roxb. ex G.	Elaeocarpaceae	0,49	10,5	8	9	0,01	0,00	32,38	0,03	1,52	5,59
5921	112	2	Sukamantri	>500	Ganitri	Elaeocarpus ganitrus Roxb. ex G.	Elaeocarpaceae	0,49	8,6	8	9	0,01	0,00	22,42	0,02	1,05	3,87
5922	112	2	Sukamantri	>500	Ganitri	Elaeocarpus ganitrus Roxb. ex G.	Elaeocarpaceae	0,49	10,8	8	9	0,01	0,00	34,21	0,03	1,61	5,90
5923	112	2	Sukamantri	>500	Afrika	Maesopsis eminii Engl.	Rhamnaceae	0,43	14,0	8	9	0,01	0,00	48,67	0,05	2,29	8,40
5924	112	2	Sukamantri	>500	Ganitri	Elaeocarpus ganitrus Roxb. ex G.	Elaeocarpaceae	0,49	3,5	2	3	0,01	0,00	1,58	0,00	0,07	0,27
5925	112	2	Sukamantri	>500	Afrika	Maesopsis eminii Engl.	Rhamnaceae	0,43	17,2	10	11	0,01	0,01	85,12	0,09	4,00	14,68
5926	112	2	Sukamantri	>500	Mahoni	Swietenia macrophylla King	Meliaceae	0,61	10,5	4	7	0,01	0,00	31,44	0,03	1,48	5,42
5927	112	2	Sukamantri	>500	Afrika	Maesopsis eminii Engl.	Rhamnaceae	0,43	9,5	6	7	0,01	0,00	19,17	0,02	0,90	3,31
5928	112	2	Sukamantri	>500	Ganitri	Elaeocarpus ganitrus Roxb. ex G.	Elaeocarpaceae	0,49	9,2	6	7	0,01	0,00	20,30	0,02	0,95	3,50
5929	112	2	Sukamantri	>500	Afrika	Maesopsis eminii Engl.	Rhamnaceae	0,43	15,3	9	10	0,01	0,00	62,87	0,06	2,95	10,84
5930	112	2	Sukamantri	>500	Ganitri	Elaeocarpus ganitrus Roxb. ex G.	Elaeocarpaceae	0,49	10,2	8	9	0,01	0,00	30,61	0,03	1,44	5,28
5931	112	2	Sukamantri	>500	Ganitri	Elaeocarpus ganitrus Roxb. ex G.	Elaeocarpaceae	0,49	8,3	7	8	0,01	0,00	18,78	0,02	0,88	3,24
5932	112	2	Sukamantri	>500	Suren	Toona sureni (Blume) Merr.	Meliaceae	0,39	22,3	5	7	0,01	0,01	82,77	0,08	3,89	14,28
5933	112	2	Sukamantri	>500	Afrika	Maesopsis eminii Engl.	Rhamnaceae	0,43	12,1	8	9	0,01	0,00	37,21	0,04	1,75	6,42
5934	112	2	Sukamantri	>500	Mahoni	Swietenia macrophylla King	Meliaceae	0,61	10,5	4	6	0,01	0,00	27,30	0,03	1,28	4,71
5935	112	2	Sukamantri	>500	Afrika	Maesopsis eminii Engl.	Rhamnaceae	0,43	15,0	9	10	0,01	0,00	60,49	0,06	2,84	10,43
5936	112	2	Sukamantri	>500	Sengon	Paraserianthes falcataria (L.) I.C.	Leguminosae / Fabaceae	0,33	21,0	9	11	0,01	0,01	96,47	0,10	4,53	16,64
5937	112	2	Sukamantri	>500	Ganitri	Elaeocarpus ganitrus Roxb. ex G.	Elaeocarpaceae	0,49	5,1	5	6	0,01	0,00	5,93	0,01	0,28	1,02
5938	112	2	Sukamantri	>500	Mangga	Mangifera indica L.	Anacardiaceae	0,6	13,7	3	5	0,01	0,00	36,96	0,04	1,74	6,37
5939	112	2	Sukamantri	>500	Afrika	Maesopsis eminii Engl.	Rhamnaceae	0,43	16,9	8	9	0,01	0,01	68,44	0,07	3,22	11,81
5940	112	2	Sukamantri	>500	Afrika	Maesopsis eminii Engl.	Rhamnaceae	0,43	24,8	9	11	0,04	0,05	166,96	0,17	1,96	7,20
5941	112	2	Sukamantri	>500	Afrika	Maesopsis eminii Engl.	Rhamnaceae	0,43	25,8	4	7	0,04	0,05	118,26	0,12	1,39	5,10
5942	112	2	Sukamantri	>500	Afrika	Maesopsis eminii Engl.	Rhamnaceae	0,43	23,9	11	13	0,04	0,04	181,08	0,18	2,13	7,81
5943	112	2	Sukamantri	>500	Afrika	Maesopsis eminii Engl.	Rhamnaceae	0,43	20,0	11	12,5	0,04	0,03	126,92	0,13	1,49	5,47
5944	112	2	Sukamantri	>500	Afrika	Maesopsis eminii Engl.	Rhamnaceae	0,43	19,1	10	12	0,04	0,03	111,81	0,11	1,31	4,82
5945	112	2	Sukamantri	>500	Nangka	Artocarpus heterophyllus Lam.	Moraceae	0,61	19,1	7	11	0,04	0,03	142,22	0,14	1,67	6,13
5946	112	2	Sukamantri	>500	Afrika	Maesopsis eminii Engl.	Rhamnaceae	0,43	12,4	8	9	0,04	0,01	39,02	0,04	0,46	1,68

5947	112	2	Sukamantri	>500	Afrika	Maesopsis eminii Engl.	Rhamnaceae		0,43	18,1	9	10	0,04	0,03	86,13	0,09	1,01	3,71
5948	112	2	Sukamantri	>500	Afrika	Maesopsis eminii Engl.	Rhamnaceae		0,43	11,1	7	8	0,04	0,01	28,73	0,03	0,34	1,24
5949	112	2	Sukamantri	>500	Ganitri	Elaeocarpus ganitrus Roxb. ex G.	Elaeocarpaceae		0,49	10,8	6	8	0,04	0,01	30,71	0,03	0,36	1,32
5950	112	2	Sukamantri	>500	Afrika	Maesopsis eminii Engl.	Rhamnaceae		0,43	10,2	7	8	0,04	0,01	24,38	0,02	0,29	1,05
5951	112	2	Sukamantri	>500	Afrika	Maesopsis eminii Engl.	Rhamnaceae		0,43	15,3	7	8	0,04	0,02	51,24	0,05	0,60	2,21
5952	112	2	Sukamantri	>500	Ganitri	Elaeocarpus ganitrus Roxb. ex G.	Elaeocarpaceae		0,49	10,5	4	5	0,04	0,01	18,90	0,02	0,22	0,82
5953	112	2	Sukamantri	>500	Afrika	Maesopsis eminii Engl.	Rhamnaceae		0,43	11,1	7	8	0,04	0,01	28,73	0,03	0,34	1,24
5954	112	2	Sukamantri	>500	Afrika	Maesopsis eminii Engl.	Rhamnaceae		0,43	10,8	8	9	0,04	0,01	30,35	0,03	0,36	1,31
5955	112	2	Sukamantri	>500	Afrika	Maesopsis eminii Engl.	Rhamnaceae		0,43	12,7	8	9	0,04	0,01	40,87	0,04	0,48	1,76
5956	112	2	Sukamantri	>500	Afrika	Maesopsis eminii Engl.	Rhamnaceae		0,43	18,8	9	10	0,04	0,03	91,74	0,09	1,08	3,96
5957	112	2	Sukamantri	>500	Jeruk ragi	Citrus microcarpa	Rutaceae		0,7	10,2	4	6	0,04	0,01	29,27	0,03	0,34	1,26
5958	112	2	Sukamantri	>500	Afrika	Maesopsis eminii Engl.	Rhamnaceae		0,43	10,2	5	7	0,04	0,01	21,57	0,02	0,25	0,93
5959	112	2	Sukamantri	>500	Afrika	Maesopsis eminii Engl.	Rhamnaceae		0,43	12,1	8	9	0,04	0,01	37,21	0,04	0,44	1,60
5960	112	2	Sukamantri	>500	Afrika	Maesopsis eminii Engl.	Rhamnaceae		0,43	15,0	6	7	0,04	0,02	43,63	0,04	0,51	1,88
5961	112	2	Sukamantri	>500	Afrika	Maesopsis eminii Engl.	Rhamnaceae		0,43	17,2	9	10	0,04	0,02	78,00	0,08	0,92	3,36
5962	112	2	Sukamantri	>500	Afrika	Maesopsis eminii Engl.	Rhamnaceae		0,43	15,9	9	10	0,04	0,02	67,75	0,07	0,80	2,92
5963	112	2	Sukamantri	>500	Afrika	Maesopsis eminii Engl.	Rhamnaceae		0,43	16,5	9	10	0,04	0,02	72,79	0,07	0,86	3,14
5964	112	2	Sukamantri	>500	Afrika	Maesopsis eminii Engl.	Rhamnaceae		0,43	14,3	7	10	0,04	0,02	55,85	0,06	0,66	2,41
5965	112	2	Sukamantri	>500	Afrika	Maesopsis eminii Engl.	Rhamnaceae		0,43	16,9	10	11	0,04	0,02	82,26	0,08	0,97	3,55
5966	112	2	Sukamantri	>500	Ganitri	Elaeocarpus ganitrus Roxb. ex G.	Elaeocarpaceae		0,49	14,0	7	9	0,04	0,02	54,86	0,05	0,64	2,37
5967	112	2	Sukamantri	>500	Ganitri	Elaeocarpus ganitrus Roxb. ex G.	Elaeocarpaceae		0,49	10,8	8	9	0,04	0,01	34,21	0,03	0,40	1,48
5968	112	2	Sukamantri	>500	Ganitri	Elaeocarpus ganitrus Roxb. ex G.	Elaeocarpaceae		0,49	15,3	7	9	0,04	0,02	64,34	0,06	0,76	2,77
5969	112	2	Sukamantri	>500	Ganitri	Elaeocarpus ganitrus Roxb. ex G.	Elaeocarpaceae		0,49	10,2	7	8	0,04	0,01	27,48	0,03	0,32	1,18
5970	112	2	Sukamantri	>500	Ganitri	Elaeocarpus ganitrus Roxb. ex G.	Elaeocarpaceae		0,49	10,8	7	8	0,04	0,01	30,71	0,03	0,36	1,32
5971	112	2	Sukamantri	>500	Ganitri	Elaeocarpus ganitrus Roxb. ex G.	Elaeocarpaceae		0,49	12,1	7	8	0,04	0,01	37,65	0,04	0,44	1,62
5972	113	1	Sukamantri	>500	Ganitri	Elaeocarpus ganitrus Roxb. ex G.	Elaeocarpaceae		0,49	15,6	9	11	0,04	0,02	80,30	0,08	0,94	3,46
5973	113	1	Sukamantri	>500	Ganitri	Elaeocarpus ganitrus Roxb. ex G.	Elaeocarpaceae		0,49	11,1	5	7	0,04	0,01	28,65	0,03	0,34	1,24
5974	113	1	Sukamantri	>500	Ganitri	Elaeocarpus ganitrus Roxb. ex G.	Elaeocarpaceae		0,49	12,7	6	7	0,04	0,01	36,60	0,04	0,43	1,58
5975	113	1	Sukamantri	>500	Ganitri	Elaeocarpus ganitrus Roxb. ex G.	Elaeocarpaceae		0,49	14,0	6	7	0,04	0,02	43,58	0,04	0,51	1,88
5976	113	1	Sukamantri	>500	Ganitri	Elaeocarpus ganitrus Roxb. ex G.	Elaeocarpaceae		0,49	8,3	5	7	0,04	0,01	16,62	0,02	0,20	0,72
5977	113	1	Sukamantri	>500	Ganitri	Elaeocarpus ganitrus Roxb. ex G.	Elaeocarpaceae		0,49	7,6	5	6	0,04	0,00	12,46	0,01	0,15	0,54
5978	113	1	Sukamantri	>500	Ganitri	Elaeocarpus ganitrus Roxb. ex G.	Elaeocarpaceae		0,49	9,2	5	6	0,04	0,01	17,63	0,02	0,21	0,76
5979	113	1	Sukamantri	>500	Ganitri	Elaeocarpus ganitrus Roxb. ex G.	Elaeocarpaceae		0,49	4,1	3	4	0,04	0,00	2,80	0,00	0,03	0,12
5980	113	1	Sukamantri	>500	Afrika	Maesopsis eminii Engl.	Rhamnaceae		0,43	14,0	12	13	0,04	0,02	68,16	0,07	0,80	2,94
5981	113	1	Sukamantri	>500	Afrika	Maesopsis eminii Engl.	Rhamnaceae		0,43	21,3	12	14	0,04	0,04	157,62	0,16	1,85	6,80
5982	113	1	Sukamantri	>500	Ganitri	Elaeocarpus ganitrus Roxb. ex G.	Elaeocarpaceae		0,49	11,8	6	9	0,04	0,01	39,94	0,04	0,47	1,72
5983	113	1	Sukamantri	>500	Ganitri	Elaeocarpus ganitrus Roxb. ex G.	Elaeocarpaceae		0,49	16,2	9	12	0,04	0,02	93,57	0,09	1,10	4,03
5984	113	1	Sukamantri	>500	Afrika	Maesopsis eminii Engl.	Rhamnaceae		0,43	12,7	10	11	0,04	0,01	49,12	0,05	0,58	2,12
5985	113	1	Sukamantri	>500	Ganitri	Elaeocarpus ganitrus Roxb. ex G.	Elaeocarpaceae		0,49	4,8	3	4	0,04	0,00	3,63	0,00	0,04	0,16
5986	113	1	Sukamantri	>500	Ganitri	Elaeocarpus ganitrus Roxb. ex G.	Elaeocarpaceae		0,49	8,0	5	7	0,04	0,00	15,47	0,02	0,18	0,67
5987	113	1	Sukamantri	>500	Ganitri	Elaeocarpus ganitrus Roxb. ex G.	Elaeocarpaceae		0,49	4,8	2	3	0,04	0,00	2,79	0,00	0,03	0,12
5988	113	1	Sukamantri	>500	Ganitri	Elaeocarpus ganitrus Roxb. ex G.	Elaeocarpaceae		0,49	5,4	5	4	0,04	0,00	4,57	0,00	0,05	0,20
5989	113	1	Sukamantri	>500	Ganitri	Elaeocarpus ganitrus Roxb. ex G.	Elaeocarpaceae		0,49	10,8	7	9	0,04	0,01	34,21	0,03	0,40	1,48
5990	113	1	Sukamantri	>500	Ganitri	Elaeocarpus ganitrus Roxb. ex G.	Elaeocarpaceae		0,49	9,5	7	8	0,04	0,01	24,41	0,02	0,29	1,05
5991	113	1	Sukamantri	>500	Ganitri	Elaeocarpus ganitrus Roxb. ex G.	Elaeocarpaceae		0,49	8,0	7	9	0,04	0,00	19,47	0,02	0,23	0,84
5992	113	1	Sukamantri	>500	Ganitri	Elaeocarpus ganitrus Roxb. ex G.	Elaeocarpaceae		0,49	9,9	7	8,5	0,04	0,01	27,41	0,03	0,32	1,18
5993	113	1	Sukamantri	>500	Ganitri	Elaeocarpus ganitrus Roxb. ex G.	Elaeocarpaceae		0,49	9,2	7	9	0,04	0,01	25,56	0,03	0,30	1,10
5994	113	1	Sukamantri	>500	Ganitri	Elaeocarpus ganitrus Roxb. ex G.	Elaeocarpaceae		0,49	4,8	5	3	0,04	0,00	2,79	0,00	0,03	0,12
5995	113	1	Sukamantri	>500	Ganitri	Elaeocarpus ganitrus Roxb. ex G.	Elaeocarpaceae		0,49	11,8	7	10	0,04	0,01	43,98	0,04	0,52	1,90
5996	113	1	Sukamantri	>500	Ganitri	Elaeocarpus ganitrus Roxb. ex G.	Elaeocarpaceae		0,49	8,9	7	10	0,04	0,01	26,40	0,03	0,31	1,14
5997	113	1	Sukamantri	>500	Afrika	Maesopsis eminii Engl.	Rhamnaceae		0,43	5,4	3	5	0,04	0,00	4,98	0,00	0,06	0,21
5998	113	1	Sukamantri	>500	Ganitri	Elaeocarpus ganitrus Roxb. ex G.	Elaeocarpaceae		0,49	14,0	9	13	0,04	0,02	76,83	0,08	0,90	3,31
5999	113	1	Sukamantri	>500	Ganitri	Elaeocarpus ganitrus Roxb. ex G.	Elaeocarpaceae		0,49	8,6	6	8	0,04	0,01	20,13	0,02	0,24	0,87
6000	113	1	Sukamantri	>500	Ganitri	Elaeocarpus ganitrus Roxb. ex G.	Elaeocarpaceae		0,49	6,0	5	7	0,04	0,00	9,36	0,01	0,11	0,40
6001	113	1	Sukamantri	>500	Afrika	Maesopsis eminii Engl.	Rhamnaceae		0,43	13,4	10	13	0,04	0,01	62,60	0,06	0,74	2,70
6002	113	1	Sukamantri	>500	Ganitri	Elaeocarpus ganitrus Roxb. ex G.	Elaeocarpaceae		0,49	7,0	4	7	0,04	0,00	12,24	0,01	0,14	0,53
6003	113	1	Sukamantri	>500	Ganitri	Elaeocarpus ganitrus Roxb. ex G.	Elaeocarpaceae		0,49	3,8	3	4	0,04	0,00	2,41	0,00	0,03	0,10
6004	113	1	Sukamantri	>500	Ganitri	Elaeocarpus ganitrus Roxb. ex G.	Elaeocarpaceae		0,49	9,2	9	10	0,04	0,01	28,15	0,03	0,33	1,21
6005	113	1	Sukamantri	>500	Ganitri	Elaeocarpus ganitrus Roxb. ex G.	Elaeocarpaceae		0,49	11,8	5	6	0,04	0,01	27,55	0,03	0,32	1,19
6006	113	1	Sukamantri	>500	Ganitri	Elaeocarpus ganitrus Roxb. ex G.	Elaeocarpaceae		0,49	17,8	9	11	0,04	0,02	102,55	0,10	1,20	4,42
6007	113	1	Sukamantri	>500	Ganitri	Elaeocarpus ganitrus Roxb. ex G.	Elaeocarpaceae		0,49	15,6	8	9	0,04	0,02	66,81	0,07	0,79	2,88
6008	113	1	Sukamantri	>500	Padali	Radermachera gigantea (Blume)	Bignoniaceae		0,48	5,4	4	5	0,04	0,00	5,50	0,01	0,06	0,24
6009	113	1	Sukamantri	>500	Afrika	Maesopsis eminii Engl.	Rhamnaceae		0,43	5,4	4	5	0,04	0,00	4,98	0,00	0,06	0,21
6010	113	1	Sukamantri	>500	Ganitri	Elaeocarpus ganitrus Roxb. ex G.	Elaeocarpaceae		0,49	6,4	5	6	0,04	0,00	8,93	0,01	0,10	0,38
6011	113	1	Sukamantri	>500	Ganitri	Elaeocarpus ganitrus Roxb. ex G.	Elaeocarpaceae		0,49	12,4	6	8	0,04	0,01	39,48	0,04	0,46	1,70
6012	113	1	Sukamantri	>500	Ganitri	Elaeocarpus ganitrus Roxb. ex G.	Elaeocarpaceae		0,49	6,4	4	6	0,04	0,00	8,93	0,01	0,10	0,38
6013	113	1	Sukamantri	>500	Ganitri	Elaeocarpus ganitrus Roxb. ex G.	Elaeocarpaceae		0,49	18,1	8	9	0,04	0,03	88,14	0,09	1,04	3,80
6014	113	1	Sukamantri	>500	Ganitri	Elaeocarpus ganitrus Roxb. ex G.	Elaeocarpaceae		0,49	3,2	3	4	0,04	0,00	1,73	0,00	0,02	0,07
6015	113	1	Sukamantri	>500	Ganitri	Elaeocarpus ganitrus Roxb. ex G.	Elaeocarpaceae		0,49	12,7	5	9	0,04	0,01	46,07	0,05	0,54	1,99
6016	113	1	Sukamantri	>500	Ganitri	Elaeocarpus ganitrus Roxb. ex G.	Elaeocarpaceae		0,49	5,4	5	7	0,04	0,00	7,63	0,01	0,09	0,33
6017	113	1	Sukamantri	>500	Ganitri	Elaeocarpus ganitrus Roxb. ex G.	Elaeocarpaceae		0,49	5,4	5	7	0,04	0,00	7,63	0,01	0,09	0,33
6018	113	1	Sukamantri	>500	Ganitri	Elaeocarpus ganitrus Roxb. ex G.	Elaeocarpaceae		0,49	6,4	6	7	0,04	0,00	10,28	0,01	0,12	0,44
6019	113	1	Sukamantri	>500	Ganitri	Elaeocarpus ganitrus Roxb. ex G.	Elaeocarpaceae		0,49	11,5	7	9	0,04	0,01	37,98	0,04	0,45	1,64
6020	113	1	Sukamantri	>500	Ganitri	Elaeocarpus ganitrus Roxb. ex G.	Elaeocarpaceae		0,49	7,3	4	6	0,04	0,00	11,53	0,01	0,14	0,50
6021	113	1	Sukamantri	>500	Sengon	Paraserianthes falcataria (L.) I.C.	Leguminosae / Fabaceae		0,33	6,0	7	8	0,04	0,00	7,36	0,01	0,09	0,32
6022	113	1	Sukamantri	>500	Ganitri	Elaeocarpus ganitrus Roxb. ex G.	Elaeocarpaceae		0,49	4,8	3	5	0,04	0,00	4,46	0,00	0,05	0,19
6023	113	1	Sukamantri	>500	Ganitri	Elaeocarpus ganitrus Roxb. ex G.	Elaeocarpaceae		0,49	6,0	4	7	0,04	0,00	9,36	0,01	0,11	0,40
6024	113	1	Sukamantri	>500	Ganitri	Elaeocarpus ganitrus Roxb. ex G.	Elaeocarpaceae		0,49	18,1	10	12	0,04	0,03	114,72	0,11	1,35	4,95
6025	113	1	Sukamantri	>500	Ganitri	Elaeocarpus ganitrus Roxb. ex G.	Elaeocarpaceae		0,49	7,3	6	8	0,04	0,00	15,01	0,02	0,18	0,65
6026	114	2	Sukamantri	>500	Puspa	Schima wallichii Choisy	Theaceae		0,74	9,9	5	7	0,04	0,01	33,47	0,03	0,39	1,44
6027	114	2	Sukamantri	>500	Puspa	Schima wallichii Choisy	Theaceae		0,74	3,2	2	3	0,04	0,00	1,94	0,00	0,02	0,08
6028	114	2	Sukamantri	>500	Renggang	Sp4		0	0,5	4,5	3	5	0,04	0,00	4,00	0,00	0,05	0,17
6029	114	2	Sukamantri	>500	Puspa	Schima wallichii Choisy	Theaceae		0,74	8,9	4	6	0,04	0,01	24,12	0,02	0,28	1,04
6030	114	2	Sukamantri	>500	Huru keling	Litsea sp.	Lauraceae		0,425	11,1	4	6	0,04	0,01	21,84	0,02	0,26	0,94
6031	114	2	Sukamantri	>500	Huru keling	Litsea sp.	Lauraceae		0,425	15,9	6	8	0,04	0,02	54,63	0,05	0,64	2,36
6032	114	2	Sukamantri	>500	Puspa	Schima wallichii Choisy	Theaceae		0,74	4,8	2	3	0,04	0,00	4,07	0,00	0,05	0,18
6033	114	2	Sukamantri	>500	Puspa	Schima wallichii Choisy	Theaceae		0,74	13,7	4	8	0,04	0,01	68,88	0,07	0,81	2,97
6034	114	2	Sukamantri	>500	Puspa	Schima wallichii Choisy	Theaceae		0,74	10,2	4	7	0,04	0,01	35,47	0,04	0,42	1,53
6035	114	2	Sukamantri	>500	Renggang	Sp4		0	0,5	8,0	3	4	0,04	0,00	9,44	0,01	0,11	0,41
6036	114	2	Sukamantri	>500	Puspa	Schima wallichii Choisy	Theaceae		0,74	4,5	2	3,5	0,04	0,00	4,13	0,00	0,05	0,18
6037	114	2	Sukamantri	>500	Huru keling	Litsea sp.	Lauraceae		0,425	12,7	7	9	0,04	0,01	40,44	0,04	0,48	1,74
6038	114	2	Sukamantri	>500	Mahoni	Swietenia macrophylla King	Meliaceae		0,61	8,3	3	4	0,04	0,01	12,17	0,01	0,14	0,52
6039	114	2	Sukamantri	>500	Mahoni	Swietenia macrophylla King	Meliaceae		0,61	4,8	3	4	0,04	0,00	4,44	0,00	0,05	0,19
6040	114	2	Sukamantri	>500	Puspa	Schima wallichii Choisy	Theaceae		0,74	14,3	6	7	0,04	0,02	66,24	0,07	0,78	2,86
6041	114	2	Sukamantri	>500	Kilunglum	Sp3		0	0,5	8,9	3	5	0,04	0,01	14,25	0,01	0,17	0,61
6042	114	2	Sukamantri	>500	Kilunglum	Sp3		0	0,5	7,6	2	4	0,04	0,00	8,76	0,01	0,10	0,38
6043	114	2	Sukamantri	>500	Puspa	Schima wallichii Choisy	Theaceae		0,74	11,8	7	8	0,04	0,01	52,30	0,05	0,61	2,26
6044	114	2	Sukamantri	>500	Puspa	Schima wallichii Choisy	Theaceae		0,74	13,0	7	9	0,04	0,01	70,31	0,07	0,83	3,03
6045	114	2	Sukamantri	>500	Puspa	Schima wallichii Choisy	Theaceae		0,74	15,9	7	9	0,04	0,02	101,14	0,10	1,19	4,36
6046	114	2	Sukamantri	>500	Puspa	Schima wallichii Choisy	Theaceae		0,74	9,2	7	9	0,04	0,01	37,29	0,04	0,44	1,61
6047	114	2	Sukamantri	>500	Kilunglum	Sp3		0	0,5	11,1	3	6	0,04	0,01	25,35	0,03	0,30	1,09
6048	114	2	Sukamantri	>500	Kijangkung	Sp2		0	0,5	12,4	5	7	0,04	0,01	35,59	0,04	0,42	1,53
6049	114	2	Sukamantri	>500	Puspa	Schima wallichii Choisy	Theaceae		0,74	5,7	3	4	0,04	0,00	7,40	0,01	0,09	0,32
6050	114	2	Sukamantri	>500	Kilunglum	Sp3		0	0,5	6,7	1	3	0,04	0,00	5,27	0,01	0,06	0,23
6051	114	2	Sukamantri	>500	Puspa	Schima wallichii Choisy	Theaceae		0,74	12,1	6	7	0,04	0,01	48,60	0,05	0,57	2,10
6052	114	2	Sukamantri	>500	Kilunglum	Sp3		0	0,5	8,9	4	6	0,04	0,01	16,84	0,02	0,20	0,73
6053	114	2	Sukamantri	>500	Puspa	Schima wallichii Choisy	Theaceae		0,74	7,6	4	5	0,04	0,00	15,39	0,02	0,18	0,66
6054	114	2	Sukamantri	>500	Puspa	Schima wallichii Choisy	Theaceae		0,74	7,6	5	6	0,04	0,00	18,18	0,02	0,21	0,78
6055	114	2	Sukamantri	>500	Kilunglum	Sp3		0	0,5	9,9	2	4	0,04	0,01	14,00	0,01	0,16	0,60
6056	114	2	Sukamantri	>500	Kilunglum	Sp3		0	0,5	7,6	2	4	0,04	0,00	8,76	0,01	0,10	0,38
6057	114	2	Sukamantri	>500	Puspa	Schima wallichii Choisy	Theaceae		0,74	8,6	4	7	0,04	0,01	25,98	0,03	0,31	1,12
6058	114	2	Sukamantri	>500	Kilunglum	Sp3		0	0,5	7,6	3	5	0,04	0,00	10,74	0,01	0,13	0,46
6059	114	2	Sukamantri	>500	Puspa	Schima wallichii Choisy	Theaceae		0,74	6,4	6	7,5	0,04	0,00	15,97	0,02	0,19	0,69
6060	114	2	Sukamantri	>500	Puspa	Schima wallichii Choisy	Theaceae		0,74	9,2	5	6	0,04	0,01	25,72	0,03	0,30	1,11
6061	114	2	Sukamantri	>500	Puspa	Schima wallichii Choisy	Theaceae		0,74	12,4	6	8	0,04	0,01	57,60	0,06	0,68	2,48
6062	114	2	Sukamantri	>500	Puspa	Schima wallichii Choisy	Theaceae		0,74	10,5	6	7	0,04	0,01	37,53	0,04	0,44	1,62
6063	114	2	Sukamantri	>500	Puspa	Schima wallichii Choisy	Theaceae		0,74	12,7	7	8,5	0,04	0,01	63,78	0,06	0,75	2,75
6064	114	2	Sukamantri	>500	Puspa	Schima wallichii Choisy	Theaceae		0,74	10,8	7	9	0,04	0,01	49,90	0,05	0,59	2,15
6065	114	2	Sukamantri	>500	Puspa	Schima wallichii Choisy	Theaceae		0,74	13,0	7	9	0,04	0,01	70,31	0,07	0,83	3,03
6066	114	2	Sukamantri	>500	Puspa	Schima wallichii Choisy	Theaceae		0,74	10,2	7	8	0,04	0,01	40,09	0,04	0,47	1,73
6067	114	2	Sukamantri	>500	Puspa	Schima wallichii Choisy	Theaceae		0,74	12,4	7	9	0,04	0,01	64,16	0,06	0,75	2,77
6068	114	2	Sukamantri	>500	Puspa	Schima wallichii Choisy	Theaceae		0,74	11,5	6	8	0,04	0,01	49,74	0,05	0,58	2,14
6069	114	2	Sukamantri	>500	Puspa	Schima wallichii Choisy	Theaceae		0,74	9,5	5	7	0,04	0,01	31,52	0,03	0,37	1,36
6070	114	2	Sukamantri	>500	Kilunglum	Sp3		0	0,5	5,7	2	4	0,04	0,00	5,17	0,01	0,06	0,22

6071	114	2	Sukamantri	>500	Puspa	Schima wallichii Choisy	Theaceae		0,74	8,0	4	6	0,04	0,00	19,60	0,02	0,23	0,84
6072	114	2	Sukamantri	>500	Puspa	Schima wallichii Choisy	Theaceae		0,74	10,2	4	7	0,04	0,01	35,47	0,04	0,42	1,53
6073	114	2	Sukamantri	>500	Kilunglum	Sp3		0	0,5	8,9	2	4	0,04	0,01	11,62	0,01	0,14	0,50
6074	114	2	Sukamantri	>500	Puspa	Schima wallichii Choisy	Theaceae		0,74	9,9	3	6	0,04	0,01	29,06	0,03	0,34	1,25
6075	114	2	Sukamantri	>500	Ganitri	Elaeocarpus ganitrus Roxb. ex G.	Elaeocarpaceae		0,49	4,8	2	4	0,04	0,00	3,63	0,00	0,04	0,16
6076	114	2	Sukamantri	>500	Kanyere	Bridelia stipularis (L.) Blume	Phyllanthaceae		0,44	15,9	4	7	0,04	0,02	49,91	0,05	0,59	2,15
6077	114	2	Sukamantri	>500	Afrika	Maesopsis eminii Engl.	Rhamnaceae		0,43	11,1	7	8,5	0,04	0,01	30,37	0,03	0,36	1,31
6078	114	2	Sukamantri	>500	Ganitri	Elaeocarpus ganitrus Roxb. ex G.	Elaeocarpaceae		0,49	7,0	3	5	0,04	0,00	8,99	0,01	0,11	0,39
6079	114	2	Sukamantri	>500	Afrika	Maesopsis eminii Engl.	Rhamnaceae		0,43	15,9	8	9	0,04	0,02	61,51	0,06	0,72	2,65
6080	114	2	Sukamantri	>500	Afrika	Maesopsis eminii Engl.	Rhamnaceae		0,43	10,8	6	7	0,04	0,01	24,11	0,02	0,28	1,04
6081	114	2	Sukamantri	>500	Puspa	Schima wallichii Choisy	Theaceae		0,74	8,6	3	5	0,04	0,01	19,09	0,02	0,22	0,82
6082	114	2	Sukamantri	>500	Puspa	Schima wallichii Choisy	Theaceae		0,74	6,0	2	4	0,04	0,00	8,18	0,01	0,10	0,35
6083	114	2	Sukamantri	>500	Kiteja	Daphniphylum glaucescens Blu	Daphniphyllaceae		0,5	6,7	2	4	0,04	0,00	6,86	0,01	0,08	0,30
6084	114	2	Sukamantri	>500	Puspa	Schima wallichii Choisy	Theaceae		0,74	5,7	2	6	0,04	0,00	10,73	0,01	0,13	0,46
6085	114	2	Sukamantri	>500	Puspa	Schima wallichii Choisy	Theaceae		0,74	5,7	4	5	0,04	0,00	9,08	0,01	0,11	0,39
6086	114	2	Sukamantri	>500	Huru keling	Litsea sp.	Lauraceae		0,425	5,4	4	5	0,04	0,00	4,92	0,00	0,06	0,21
6087	115	2	Sukamantri	>500	Huru keling	Litsea sp.	Lauraceae		0,425	20,7	9	11	0,04	0,03	118,28	0,12	1,39	5,10
6088	115	2	Sukamantri	>500	Huru keling	Litsea sp.	Lauraceae		0,425	16,9	8	10	0,04	0,02	74,58	0,07	0,88	3,22
6089	115	2	Sukamantri	>500	Mahoni	Swietenia macrophylla King	Meliaceae		0,61	12,7	4	7	0,04	0,01	44,73	0,04	0,53	1,93
6090	115	2	Sukamantri	>500	Ganitri	Elaeocarpus ganitrus Roxb. ex G.	Elaeocarpaceae		0,49	5,7	5	7	0,04	0,00	8,47	0,01	0,10	0,37
6091	115	2	Sukamantri	>500	Mahoni	Swietenia macrophylla King	Meliaceae		0,61	8,0	3	6	0,04	0,00	16,42	0,02	0,19	0,71
6092	115	2	Sukamantri	>500	Ganitri	Elaeocarpus ganitrus Roxb. ex G.	Elaeocarpaceae		0,49	4,1	5	6	0,04	0,00	4,05	0,00	0,05	0,17
6093	115	2	Sukamantri	>500	Mahoni	Swietenia macrophylla King	Meliaceae		0,61	16,9	4	9	0,04	0,02	94,28	0,09	1,11	4,07
6094	115	2	Sukamantri	>500	Huru keling	Litsea sp.	Lauraceae		0,425	2,9	2	3	0,04	0,00	0,96	0,00	0,01	0,04
6095	115	2	Sukamantri	>500	Mahoni	Swietenia macrophylla King	Meliaceae		0,61	9,2	4	5	0,04	0,01	18,23	0,02	0,21	0,79
6096	115	2	Sukamantri	>500	Ganitri	Elaeocarpus ganitrus Roxb. ex G.	Elaeocarpaceae		0,49	10,5	6	9	0,04	0,01	32,38	0,03	0,38	1,40
6097	115	2	Sukamantri	>500	Ganitri	Elaeocarpus ganitrus Roxb. ex G.	Elaeocarpaceae		0,49	3,2	2	3	0,04	0,00	1,33	0,00	0,02	0,06
6098	115	2	Sukamantri	>500	Afrika	Maesopsis eminii Engl.	Rhamnaceae		0,43	18,1	9	10	0,04	0,03	86,13	0,09	1,01	3,71
6099	115	2	Sukamantri	>500	Afrika	Maesopsis eminii Engl.	Rhamnaceae		0,43	15,3	9	10	0,04	0,02	62,87	0,06	0,74	2,71
6100	115	2	Sukamantri	>500	Afrika	Maesopsis eminii Engl.	Rhamnaceae		0,43	13,7	9	10	0,04	0,01	51,39	0,05	0,60	2,22
6101	115	2	Sukamantri	>500	Ganitri	Elaeocarpus ganitrus Roxb. ex G.	Elaeocarpaceae		0,49	8,0	5	6	0,04	0,00	13,43	0,01	0,16	0,58
6102	115	2	Sukamantri	>500	Ganitri	Elaeocarpus ganitrus Roxb. ex G.	Elaeocarpaceae		0,49	7,0	4	5	0,04	0,00	8,99	0,01	0,11	0,39
6103	115	2	Sukamantri	>500	Afrika	Maesopsis eminii Engl.	Rhamnaceae		0,43	6,4	7	8	0,04	0,00	10,31	0,01	0,12	0,44
6104	115	2	Sukamantri	>500	Afrika	Maesopsis eminii Engl.	Rhamnaceae		0,43	6,0	9	10	0,04	0,00	11,51	0,01	0,14	0,50
6105	115	2	Sukamantri	>500	Ganitri	Elaeocarpus ganitrus Roxb. ex G.	Elaeocarpaceae		0,49	13,0	9	10	0,04	0,01	53,08	0,05	0,62	2,29
6106	115	2	Sukamantri	>500	Ganitri	Elaeocarpus ganitrus Roxb. ex G.	Elaeocarpaceae		0,49	7,3	5	8	0,04	0,00	15,01	0,02	0,18	0,65
6107	115	2	Sukamantri	>500	Puspa	Schima wallichii Choisy	Theaceae		0,74	16,2	7	10	0,04	0,02	115,51	0,12	1,36	4,98
6108	115	2	Sukamantri	>500	Puspa	Schima wallichii Choisy	Theaceae		0,74	16,2	7	9	0,04	0,02	104,88	0,10	1,23	4,52
6109	115	2	Sukamantri	>500	Nangka	Artocarpus heterophyllus Lam.	Moraceae		0,61	5,7	3	4	0,04	0,00	6,20	0,01	0,07	0,27
6110	115	2	Sukamantri	>500	Afrika	Maesopsis eminii Engl.	Rhamnaceae		0,43	11,1	10	11	0,04	0,01	38,46	0,04	0,45	1,66
6111	115	2	Sukamantri	>500	Ganitri	Elaeocarpus ganitrus Roxb. ex G.	Elaeocarpaceae		0,49	4,5	4	6	0,04	0,00	4,64	0,00	0,05	0,20
6112	115	2	Sukamantri	>500	Ganitri	Elaeocarpus ganitrus Roxb. ex G.	Elaeocarpaceae		0,49	8,9	7	9	0,04	0,01	23,97	0,02	0,28	1,03
6113	115	2	Sukamantri	>500	Afrika	Maesopsis eminii Engl.	Rhamnaceae		0,43	18,8	10	12	0,04	0,03	108,42	0,11	1,27	4,68
6114	115	2	Sukamantri	>500	Afrika	Maesopsis eminii Engl.	Rhamnaceae		0,43	15,9	10	12	0,04	0,02	80,06	0,08	0,94	3,45
6115	115	2	Sukamantri	>500	Afrika	Maesopsis eminii Engl.	Rhamnaceae		0,43	4,5	4	5	0,04	0,00	3,49	0,00	0,04	0,15
6116	115	2	Sukamantri	>500	Afrika	Maesopsis eminii Engl.	Rhamnaceae		0,43	7,6	9	11	0,04	0,00	19,27	0,02	0,23	0,83
6117	115	2	Sukamantri	>500	Afrika	Maesopsis eminii Engl.	Rhamnaceae		0,43	11,8	8	10	0,04	0,01	39,02	0,04	0,46	1,68
6118	115	2	Sukamantri	>500	Afrika	Maesopsis eminii Engl.	Rhamnaceae		0,43	8,6	9	10	0,04	0,01	21,91	0,02	0,26	0,94
6119	115	2	Sukamantri	>500	Afrika	Maesopsis eminii Engl.	Rhamnaceae		0,43	20,7	10	12	0,04	0,03	129,47	0,13	1,52	5,58
6120	115	2	Sukamantri	>500	Afrika	Maesopsis eminii Engl.	Rhamnaceae		0,43	20,4	10	12	0,04	0,03	125,84	0,13	1,48	5,43
6121	115	2	Sukamantri	>500	Huru keling	Litsea sp.	Lauraceae		0,425	15,0	9	10	0,04	0,02	59,84	0,06	0,70	2,58
6122	115	2	Sukamantri	>500	Ganitri	Elaeocarpus ganitrus Roxb. ex G.	Elaeocarpaceae		0,49	4,1	4	5	0,04	0,00	3,43	0,00	0,04	0,15
6123	115	2	Sukamantri	>500	Manglid	Magnolia champaca (L.) Baill. ex	Magnoliaceae		0,53	7,6	5	6	0,04	0,00	13,39	0,01	0,16	0,58
6124	115	2	Sukamantri	>500	Mahoni	Swietenia macrophylla King	Meliaceae		0,61	19,7	6	10	0,04	0,03	138,40	0,14	1,63	5,97
6125	115	2	Sukamantri	>500	Ganitri	Elaeocarpus ganitrus Roxb. ex G.	Elaeocarpaceae		0,49	3,2	4	5	0,04	0,00	2,12	0,00	0,02	0,09
6126	115	2	Sukamantri	>500	Alpukat	Persea americana Mill.	Lauraceae		0,54	5,1	3	5	0,04	0,00	5,49	0,01	0,06	0,24
6127	115	2	Sukamantri	>500	Ganitri	Elaeocarpus ganitrus Roxb. ex G.	Elaeocarpaceae		0,49	9,5	7	9	0,04	0,01	27,20	0,03	0,32	1,17
6128	115	2	Sukamantri	>500	Ganitri	Elaeocarpus ganitrus Roxb. ex G.	Elaeocarpaceae		0,49	10,5	7	10	0,04	0,01	35,67	0,04	0,42	1,54
6129	115	2	Sukamantri	>500	Manglid	Magnolia champaca (L.) Baill. ex	Magnoliaceae		0,53	3,2	3	4	0,04	0,00	1,86	0,00	0,02	0,08
6130	115	2	Sukamantri	>500	Manglid	Magnolia champaca (L.) Baill. ex	Magnoliaceae		0,53	3,2	4	5	0,04	0,00	2,28	0,00	0,03	0,10
6131	115	2	Sukamantri	>500	Ganitri	Elaeocarpus ganitrus Roxb. ex G.	Elaeocarpaceae		0,49	7,6	7	8	0,04	0,00	16,22	0,02	0,19	0,70
6132	115	2	Sukamantri	>500	Huru keling	Litsea sp.	Lauraceae		0,425	5,4	5	6	0,04	0,00	5,82	0,01	0,07	0,25
6133	115	2	Sukamantri	>500	Ganitri	Elaeocarpus ganitrus Roxb. ex G.	Elaeocarpaceae		0,49	3,2	3	4	0,04	0,00	1,73	0,00	0,02	0,07
6134	115	2	Sukamantri	>500	Ganitri	Elaeocarpus ganitrus Roxb. ex G.	Elaeocarpaceae		0,49	7,0	7	8	0,04	0,00	13,83	0,01	0,16	0,60
6135	115	2	Sukamantri	>500	Alpukat	Persea americana Mill.	Lauraceae		0,54	15,0	6	9	0,04	0,02	67,66	0,07	0,80	2,92
6136	115	2	Sukamantri	>500	Ganitri	Elaeocarpus ganitrus Roxb. ex G.	Elaeocarpaceae		0,49	4,1	3	4	0,04	0,00	2,80	0,00	0,03	0,12
6137	115	2	Sukamantri	>500	Ganitri	Elaeocarpus ganitrus Roxb. ex G.	Elaeocarpaceae		0,49	5,7	7	8	0,04	0,00	9,58	0,01	0,11	0,41
6138	115	2	Sukamantri	>500	Ganitri	Elaeocarpus ganitrus Roxb. ex G.	Elaeocarpaceae		0,49	20,4	11	14	0,04	0,03	163,35	0,16	1,92	7,04
6139	115	2	Sukamantri	>500	Ganitri	Elaeocarpus ganitrus Roxb. ex G.	Elaeocarpaceae		0,49	3,2	4	5	0,04	0,00	2,12	0,00	0,02	0,09
6140	115	2	Sukamantri	>500	Manglid	Magnolia champaca (L.) Baill. ex	Magnoliaceae		0,53	3,2	2	3	0,04	0,00	1,43	0,00	0,02	0,06
6141	115	2	Sukamantri	>500	Ganitri	Elaeocarpus ganitrus Roxb. ex G.	Elaeocarpaceae		0,49	5,4	6	7	0,04	0,00	7,63	0,01	0,09	0,33
6142	115	2	Sukamantri	>500	Ganitri	Elaeocarpus ganitrus Roxb. ex G.	Elaeocarpaceae		0,49	16,5	9	10	0,04	0,02	82,05	0,08	0,96	3,54
6143	115	2	Sukamantri	>500	Ganitri	Elaeocarpus ganitrus Roxb. ex G.	Elaeocarpaceae		0,49	3,2	4	5	0,04	0,00	2,12	0,00	0,02	0,09
6144	115	2	Sukamantri	>500	Ganitri	Elaeocarpus ganitrus Roxb. ex G.	Elaeocarpaceae		0,49	13,7	10	11	0,04	0,01	63,21	0,06	0,74	2,73
6145	115	2	Sukamantri	>500	Mahoni	Swietenia macrophylla King	Meliaceae		0,61	18,1	6	10	0,04	0,03	118,64	0,12	1,39	5,12
6146	115	2	Sukamantri	>500	Ganitri	Elaeocarpus ganitrus Roxb. ex G.	Elaeocarpaceae		0,49	2,9	4	5	0,04	0,00	1,75	0,00	0,02	0,08
6147	115	2	Sukamantri	>500	Ganitri	Elaeocarpus ganitrus Roxb. ex G.	Elaeocarpaceae		0,49	13,4	10	12	0,04	0,01	65,56	0,07	0,77	2,83
6148	115	2	Sukamantri	>500	Ganitri	Elaeocarpus ganitrus Roxb. ex G.	Elaeocarpaceae		0,49	5,4	4	5,5	0,04	0,00	6,12	0,01	0,07	0,26
6149	115	2	Sukamantri	>500	Ganitri	Elaeocarpus ganitrus Roxb. ex G.	Elaeocarpaceae		0,49	4,5	4	5	0,04	0,00	3,93	0,00	0,05	0,17
6150	115	2	Sukamantri	>500	Ganitri	Elaeocarpus ganitrus Roxb. ex G.	Elaeocarpaceae		0,49	2,5	2	3	0,04	0,00	0,88	0,00	0,01	0,04
6151	115	2	Sukamantri	>500	Ganitri	Elaeocarpus ganitrus Roxb. ex G.	Elaeocarpaceae		0,49	9,2	8	9	0,04	0,01	25,56	0,03	0,30	1,10
6152	115	2	Sukamantri	>500	Ganitri	Elaeocarpus ganitrus Roxb. ex G.	Elaeocarpaceae		0,49	8,9	8	9	0,04	0,01	23,97	0,02	0,28	1,03
6153	115	2	Sukamantri	>500	Ganitri	Elaeocarpus ganitrus Roxb. ex G.	Elaeocarpaceae		0,49	3,2	2	3	0,04	0,00	1,33	0,00	0,02	0,06
6154	115	2	Sukamantri	>500	Ganitri	Elaeocarpus ganitrus Roxb. ex G.	Elaeocarpaceae		0,49	7,0	7	8	0,04	0,00	13,83	0,01	0,16	0,60
6155	115	2	Sukamantri	>500	Afrika	Maesopsis eminii Engl.	Rhamnaceae		0,43	8,3	6	7	0,04	0,01	14,75	0,01	0,17	0,64
6156	115	2	Sukamantri	>500	Afrika	Maesopsis eminii Engl.	Rhamnaceae		0,43	15,3	12	13	0,04	0,02	79,94	0,08	0,94	3,45
6157	115	2	Sukamantri	>500	Afrika	Maesopsis eminii Engl.	Rhamnaceae		0,43	3,2	3	4	0,04	0,00	1,53	0,00	0,02	0,07
6158	115	2	Sukamantri	>500	Huru keling	Litsea sp.	Lauraceae		0,425	23,9	12	14	0,04	0,04	191,73	0,19	2,25	8,27
6159	115	2	Sukamantri	>500	Huru keling	Litsea sp.	Lauraceae		0,425	12,4	9	11	0,04	0,01	46,39	0,05	0,55	2,00
6160	115	2	Sukamantri	>500	Huru keling	Litsea sp.	Lauraceae		0,425	22,3	12	14	0,04	0,04	168,96	0,17	1,99	7,29
6161	115	2	Sukamantri	>500	Huru keling	Litsea sp.	Lauraceae		0,425	7,6	6	8	0,04	0,00	14,24	0,01	0,17	0,61
6162	115	2	Sukamantri	>500	Huru keling	Litsea sp.	Lauraceae		0,425	10,8	8	10	0,04	0,01	33,07	0,03	0,39	1,43
6163	115	2	Sukamantri	>500	Afrika	Maesopsis eminii Engl.	Rhamnaceae		0,43	16,9	12	14	0,04	0,02	102,59	0,10	1,21	4,42
6164	115	2	Sukamantri	>500	Afrika	Maesopsis eminii Engl.	Rhamnaceae		0,43	14,6	10	11	0,04	0,02	63,45	0,06	0,75	2,74
6165	115	2	Sukamantri	>500	Afrika	Maesopsis eminii Engl.	Rhamnaceae		0,43	17,5	12	14	0,04	0,02	109,79	0,11	1,29	4,73
6166	115	2	Sukamantri	>500	Afrika	Maesopsis eminii Engl.	Rhamnaceae		0,43	15,3	9	11	0,04	0,02	68,60	0,07	0,81	2,96
6167	115	2	Sukamantri	>500	Afrika	Maesopsis eminii Engl.	Rhamnaceae		0,43	10,2	9	10	0,04	0,01	29,91	0,03	0,35	1,29
6168	115	2	Sukamantri	>500	Afrika	Maesopsis eminii Engl.	Rhamnaceae		0,43	16,5	12	14	0,04	0,02	99,07	0,10	1,16	4,27
6169	115	2	Sukamantri	>500	Huru keling	Litsea sp.	Lauraceae		0,425	8,9	9	10	0,04	0,01	23,17	0,02	0,27	1,00
6170	115	2	Sukamantri	>500	Ganitri	Elaeocarpus ganitrus Roxb. ex G.	Elaeocarpaceae		0,49	3,8	4	55	0,04	0,00	26,64	0,03	0,31	1,15
6171	115	2	Sukamantri	>500	Ganitri	Elaeocarpus ganitrus Roxb. ex G.	Elaeocarpaceae		0,49	3,8	4	6	0,04	0,00	3,50	0,00	0,04	0,15
6172	115	2	Sukamantri	>500	Afrika	Maesopsis eminii Engl.	Rhamnaceae		0,43	19,7	10	11	0,04	0,03	109,64	0,11	1,29	4,73
6173	115	2	Sukamantri	>500	Afrika	Maesopsis eminii Engl.	Rhamnaceae		0,43	20,7	10	11	0,04	0,03	119,55	0,12	1,40	5,16
6174	115	2	Sukamantri	>500	Ganitri	Elaeocarpus ganitrus Roxb. ex G.	Elaeocarpaceae		0,49	3,8	7	8	0,04	0,00	4,56	0,00	0,05	0,20
6175	115	2	Sukamantri	>500	Ganitri	Elaeocarpus ganitrus Roxb. ex G.	Elaeocarpaceae		0,49	10,2	7	9	0,04	0,01	30,61	0,03	0,36	1,32
6176	115	2	Sukamantri	>500	Ganitri	Elaeocarpus ganitrus Roxb. ex G.	Elaeocarpaceae		0,49	4,5	6	7	0,04	0,00	5,35	0,01	0,06	0,23
6177	115	2	Sukamantri	>500	Ganitri	Elaeocarpus ganitrus Roxb. ex G.	Elaeocarpaceae		0,49	8,9	8	9	0,04	0,01	23,97	0,02	0,28	1,03
6178	115	2	Sukamantri	>500	Ganitri	Elaeocarpus ganitrus Roxb. ex G.	Elaeocarpaceae		0,49	11,8	8	9	0,04	0,01	39,94	0,04	0,47	1,72
6179	115	2	Sukamantri	>500	Huru keling	Litsea sp.	Lauraceae		0,425	16,5	10	11	0,04	0,02	78,59	0,08	0,92	3,39
6180	115	2	Sukamantri	>500	Ganitri	Elaeocarpus ganitrus Roxb. ex G.	Elaeocarpaceae		0,49	14,3	10	13	0,04	0,02	80,06	0,08	0,94	3,45
6181	115	2	Sukamantri	>500	Ganitri	Elaeocarpus ganitrus Roxb. ex G.	Elaeocarpaceae		0,49	14,6	9	11	0,04	0,02	71,52	0,07	0,84	3,08
6182	115	2	Sukamantri	>500	Ganitri	Elaeocarpus ganitrus Roxb. ex G.	Elaeocarpaceae		0,49	6,4	6	8	0,04	0,00	11,62	0,01	0,14	0,50
6183	115	2	Sukamantri	>500	Afrika	Maesopsis eminii Engl.	Rhamnaceae		0,43	7,0	9	10	0,04	0,00	15,06	0,02	0,18	0,65
6184	115	2	Sukamantri	>500	Afrika	Maesopsis eminii Engl.	Rhamnaceae		0,43	8,6	10	11	0,04	0,01	23,91	0,02	0,28	1,03
6185	115	2	Sukamantri	>500	Afrika	Maesopsis eminii Engl.	Rhamnaceae		0,43	8,6	9	10	0,04	0,01	21,91	0,02	0,26	0,94
6186	115	2	Sukamantri	>500	Ganitri	Elaeocarpus ganitrus Roxb. ex G.	Elaeocarpaceae		0,49	5,7	5	6	0,04	0,00	7,36	0,01	0,09	0,32
6187	115	2	Sukamantri	>500	Afrika	Maesopsis eminii Engl.	Rhamnaceae		0,43	20,0	10	11	0,04	0,03	112,90	0,11	1,33	4,87
6188	115	2	Sukamantri	>500	Afrika	Maesopsis eminii Engl.	Rhamnaceae		0,43	15,9	10	11	0,04	0,02	73,93	0,07	0,87	3,19
6189	116	2	Sukamantri	>500	Afrika	Maesopsis eminii Engl.	Rhamnaceae		0,43	10,5	10	11	0,04	0,01	34,53	0,03	0,41	1,49
6190	116	2	Sukamantri	>500	Afrika	Maesopsis eminii Engl.	Rhamnaceae		0,43	12,4	9	10	0,04	0,01	42,97	0,04	0,50	1,85
6191	116	2	Sukamantri	>500	Afrika	Maesopsis eminii Engl.	Rhamnaceae		0,43	8,0	8	9	0,04	0,00	17,28	0,02	0,20	0,75
6192	116	2	Sukamantri	>500	Afrika	Maesopsis eminii Engl.	Rhamnaceae		0,43	10,5	10	11	0,04	0,01	34,53	0,03	0,41	1,49
6193	116	2	Sukamantri	>500	Afrika	Maesopsis eminii Engl.	Rhamnaceae		0,43	18,8	12	13	0,04	0,03	116,67	0,12	1,37	5,03
6194	116	2	Sukamantri	>500	Afrika	Maesopsis eminii Engl.	Rhamnaceae		0,43	15,0	12	13	0,04	0,02	76,92	0,08	0,90	3,32

6195	116	2	Sukamantri	>500	Afrika	Maesopsis eminii Engl.	Rhamnaceae	0,43	3,8	5	6	0,04	0,00	3,11	0,00	0,04	0,13
6196	116	2	Sukamantri	>500	Afrika	Maesopsis eminii Engl.	Rhamnaceae	0,43	7,6	8	9	0,04	0,00	16,03	0,02	0,19	0,69
6197	116	2	Sukamantri	>500	Afrika	Maesopsis eminii Engl.	Rhamnaceae	0,43	6,7	8	9	0,04	0,00	12,55	0,01	0,15	0,54
6198	116	2	Sukamantri	>500	Afrika	Maesopsis eminii Engl.	Rhamnaceae	0,43	20,7	12	13	0,04	0,03	139,32	0,14	1,64	6,01
6199	116	2	Sukamantri	>500	Afrika	Maesopsis eminii Engl.	Rhamnaceae	0,43	13,0	10	11	0,04	0,01	51,39	0,05	0,60	2,22
6200	116	2	Sukamantri	>500	Afrika	Maesopsis eminii Engl.	Rhamnaceae	0,43	16,9	10	11	0,04	0,02	82,26	0,08	0,97	3,55
6201	116	2	Sukamantri	>500	Afrika	Maesopsis eminii Engl.	Rhamnaceae	0,43	7,3	9	10	0,04	0,00	16,33	0,02	0,19	0,70
6202	116	2	Sukamantri	>500	Kaliandra	Calliandra calothyrsus Meisn.	Leguminosae / Fabaceae	0,65	7,0	6	8	0,04	0,00	17,92	0,02	0,21	0,77
6203	116	2	Sukamantri	>500	Gmelina	Gmelina arborea Roxb.	Lamiaceae / Verbenaceae	0,47	4,1	6	8	0,04	0,00	5,08	0,01	0,06	0,22
6204	116	2	Sukamantri	>500	Afrika	Maesopsis eminii Engl.	Rhamnaceae	0,43	4,5	8	9	0,04	0,00	5,97	0,01	0,07	0,26
6205	116	2	Sukamantri	>500	Afrika	Maesopsis eminii Engl.	Rhamnaceae	0,43	9,9	9	10	0,04	0,01	28,22	0,03	0,33	1,22
6206	116	2	Sukamantri	>500	Afrika	Maesopsis eminii Engl.	Rhamnaceae	0,43	7,3	8	9	0,04	0,00	14,83	0,01	0,17	0,64
6207	116	2	Sukamantri	>500	Afrika	Maesopsis eminii Engl.	Rhamnaceae	0,43	7,6	9	10	0,04	0,00	17,66	0,02	0,21	0,76
6208	116	2	Sukamantri	>500	Puspa	Schima wallichii Choisy	Theaceae	0,74	5,4	3	5	0,04	0,00	8,18	0,01	0,10	0,35
6209	116	2	Sukamantri	>500	Puspa	Schima wallichii Choisy	Theaceae	0,74	4,5	3	5	0,04	0,00	5,73	0,01	0,07	0,25
6210	116	2	Sukamantri	>500	Kaliandra	Calliandra calothyrsus Meisn.	Leguminosae / Fabaceae	0,65	7,0	6	7,5	0,04	0,00	16,89	0,02	0,20	0,73
6211	116	2	Sukamantri	>500	Kaliandra	Calliandra calothyrsus Meisn.	Leguminosae / Fabaceae	0,65	6,4	6	7,5	0,04	0,00	14,18	0,01	0,17	0,61
6212	116	2	Sukamantri	>500	Gmelina	Gmelina arborea Roxb.	Lamiaceae / Verbenaceae	0,47	4,5	4	5	0,04	0,00	3,78	0,00	0,04	0,16
6213	116	2	Sukamantri	>500	Afrika	Maesopsis eminii Engl.	Rhamnaceae	0,43	14,0	11	12	0,04	0,02	63,34	0,06	0,74	2,73
6214	116	2	Sukamantri	>500	Kaliandra	Calliandra calothyrsus Meisn.	Leguminosae / Fabaceae	0,65	12,1	5	8	0,04	0,01	48,77	0,05	0,57	2,10
6215	116	2	Sukamantri	>500	Kaliandra	Calliandra calothyrsus Meisn.	Leguminosae / Fabaceae	0,65	10,2	5	8	0,04	0,01	35,60	0,04	0,42	1,54
6216	116	2	Sukamantri	>500	Gmelina	Gmelina arborea Roxb.	Lamiaceae / Verbenaceae	0,47	5,1	5	6	0,04	0,00	5,71	0,01	0,07	0,25
6217	116	2	Sukamantri	>500	Gmelina	Gmelina arborea Roxb.	Lamiaceae / Verbenaceae	0,47	9,2	7	8	0,04	0,01	22,09	0,02	0,26	0,95
6218	116	2	Sukamantri	>500	Afrika	Maesopsis eminii Engl.	Rhamnaceae	0,43	12,1	9	10	0,04	0,01	40,98	0,04	0,48	1,77
6219	116	2	Sukamantri	>500	Gmelina	Gmelina arborea Roxb.	Lamiaceae / Verbenaceae	0,47	9,5	7	8	0,04	0,01	23,50	0,02	0,28	1,01
6220	116	2	Sukamantri	>500	Gmelina	Gmelina arborea Roxb.	Lamiaceae / Verbenaceae	0,47	3,5	5	6	0,04	0,00	2,87	0,00	0,03	0,12
6221	116	2	Sukamantri	>500	Afrika	Maesopsis eminii Engl.	Rhamnaceae	0,43	7,3	8	9	0,04	0,00	14,83	0,01	0,17	0,64
6222	116	2	Sukamantri	>500	Gmelina	Gmelina arborea Roxb.	Lamiaceae / Verbenaceae	0,47	6,4	6	7	0,04	0,00	9,89	0,01	0,12	0,43
6223	116	2	Sukamantri	>500	Afrika	Maesopsis eminii Engl.	Rhamnaceae	0,43	16,5	10	11	0,04	0,02	79,43	0,08	0,93	3,43
6224	116	2	Sukamantri	>500	Afrika	Maesopsis eminii Engl.	Rhamnaceae	0,43	12,4	12	13	0,04	0,01	54,65	0,05	0,64	2,36
6225	116	2	Sukamantri	>500	Gmelina	Gmelina arborea Roxb.	Lamiaceae / Verbenaceae	0,47	6,4	7	8	0,04	0,00	11,18	0,01	0,13	0,48
6226	116	2	Sukamantri	>500	Afrika	Maesopsis eminii Engl.	Rhamnaceae	0,43	10,8	10	11	0,04	0,01	36,47	0,04	0,43	1,57
6227	116	2	Sukamantri	>500	Afrika	Maesopsis eminii Engl.	Rhamnaceae	0,43	12,4	10	11	0,04	0,01	46,89	0,05	0,55	2,02
6228	116	2	Sukamantri	>500	Afrika	Maesopsis eminii Engl.	Rhamnaceae	0,43	12,1	11	12	0,04	0,01	48,43	0,05	0,57	2,09
6229	116	2	Sukamantri	>500	Gmelina	Gmelina arborea Roxb.	Lamiaceae / Verbenaceae	0,47	4,1	5	6	0,04	0,00	3,90	0,00	0,05	0,17
6230	116	2	Sukamantri	>500	Gmelina	Gmelina arborea Roxb.	Lamiaceae / Verbenaceae	0,47	4,1	5	6	0,04	0,00	3,90	0,00	0,05	0,17
6231	116	2	Sukamantri	>500	Afrika	Maesopsis eminii Engl.	Rhamnaceae	0,43	18,5	11	12	0,04	0,03	105,08	0,11	1,23	4,53
6232	116	2	Sukamantri	>500	Afrika	Maesopsis eminii Engl.	Rhamnaceae	0,43	6,4	8	9	0,04	0,00	11,48	0,01	0,13	0,50
6233	116	2	Sukamantri	>500	Afrika	Maesopsis eminii Engl.	Rhamnaceae	0,43	10,5	10	11	0,04	0,01	34,53	0,03	0,41	1,49
6234	116	2	Sukamantri	>500	Gmelina	Gmelina arborea Roxb.	Lamiaceae / Verbenaceae	0,47	7,6	6	8	0,04	0,00	15,61	0,02	0,18	0,67
6235	116	2	Sukamantri	>500	Afrika	Maesopsis eminii Engl.	Rhamnaceae	0,43	15,6	10	12	0,04	0,02	77,15	0,08	0,91	3,33
6236	116	2	Sukamantri	>500	Darangdang	Ficus obscura Blume	Moraceae	0,393	9,5	4	7	0,04	0,01	17,65	0,02	0,21	0,76
6237	116	2	Sukamantri	>500	Afrika	Maesopsis eminii Engl.	Rhamnaceae	0,43	4,5	6	7	0,04	0,00	4,74	0,00	0,06	0,20
6238	116	2	Sukamantri	>500	Afrika	Maesopsis eminii Engl.	Rhamnaceae	0,43	19,1	11	13	0,04	0,03	120,32	0,12	1,41	5,19
6239	116	2	Sukamantri	>500	Gmelina	Gmelina arborea Roxb.	Lamiaceae / Verbenaceae	0,47	3,8	4	5	0,04	0,00	2,85	0,00	0,03	0,12
6240	116	2	Sukamantri	>500	Afrika	Maesopsis eminii Engl.	Rhamnaceae	0,43	10,8	10	11	0,04	0,01	36,47	0,04	0,43	1,57
6241	116	2	Sukamantri	>500	Afrika	Maesopsis eminii Engl.	Rhamnaceae	0,43	11,8	11	12	0,04	0,01	46,12	0,05	0,54	1,99
6242	116	2	Sukamantri	>500	Gmelina	Gmelina arborea Roxb.	Lamiaceae / Verbenaceae	0,47	10,2	8	10	0,04	0,01	32,45	0,03	0,38	1,40
6243	116	2	Sukamantri	>500	Gmelina	Gmelina arborea Roxb.	Lamiaceae / Verbenaceae	0,47	7,6	8	10	0,04	0,00	19,16	0,02	0,23	0,83
6244	116	2	Sukamantri	>500	Afrika	Maesopsis eminii Engl.	Rhamnaceae	0,43	22,3	12	13	0,04	0,04	159,58	0,16	1,88	6,88
6245	116	2	Sukamantri	>500	Afrika	Maesopsis eminii Engl.	Rhamnaceae	0,43	9,5	10	11	0,04	0,01	29,00	0,03	0,34	1,25
6246	116	2	Sukamantri	>500	Afrika	Maesopsis eminii Engl.	Rhamnaceae	0,43	11,1	11	12	0,04	0,01	41,65	0,04	0,49	1,80
6247	116	2	Sukamantri	>500	Gmelina	Gmelina arborea Roxb.	Lamiaceae / Verbenaceae	0,47	11,1	8	11	0,04	0,01	41,73	0,04	0,49	1,80
6248	116	2	Sukamantri	>500	Afrika	Maesopsis eminii Engl.	Rhamnaceae	0,43	5,4	10	11	0,04	0,00	10,24	0,01	0,12	0,44
6249	116	2	Sukamantri	>500	Afrika	Maesopsis eminii Engl.	Rhamnaceae	0,43	11,8	10	11	0,04	0,01	42,58	0,04	0,50	1,84
6250	116	2	Sukamantri	>500	Afrika	Maesopsis eminii Engl.	Rhamnaceae	0,43	7,6	8	9	0,04	0,00	16,03	0,02	0,19	0,69
6251	116	2	Sukamantri	>500	Afrika	Maesopsis eminii Engl.	Rhamnaceae	0,43	8,3	9	10	0,04	0,01	20,45	0,02	0,24	0,88
6252	116	2	Sukamantri	>500	Afrika	Maesopsis eminii Engl.	Rhamnaceae	0,43	8,0	9	10	0,04	0,00	19,03	0,02	0,22	0,82
6253	116	2	Sukamantri	>500	Afrika	Maesopsis eminii Engl.	Rhamnaceae	0,43	6,4	8	9	0,04	0,00	11,48	0,01	0,13	0,50
6254	116	2	Sukamantri	>500	Afrika	Maesopsis eminii Engl.	Rhamnaceae	0,43	7,0	9	10	0,04	0,00	15,06	0,02	0,18	0,65
6255	116	2	Sukamantri	>500	Kaliandra	Calliandra calothyrsus Meisn.	Leguminosae / Fabaceae	0,65	8,6	5	7	0,04	0,01	23,07	0,02	0,27	0,99
6256	116	2	Sukamantri	>500	Afrika	Maesopsis eminii Engl.	Rhamnaceae	0,43	8,6	9	10	0,04	0,01	21,91	0,02	0,26	0,94
6257	116	2	Sukamantri	>500	Afrika	Maesopsis eminii Engl.	Rhamnaceae	0,43	8,0	8	9	0,04	0,00	17,28	0,02	0,20	0,75
6258	116	2	Sukamantri	>500	Afrika	Maesopsis eminii Engl.	Rhamnaceae	0,43	16,5	10	11	0,04	0,02	79,43	0,08	0,93	3,43
6259	116	2	Sukamantri	>500	Gmelina	Gmelina arborea Roxb.	Lamiaceae / Verbenaceae	0,47	7,6	5	7	0,04	0,00	13,82	0,01	0,16	0,60
6260	116	2	Sukamantri	>500	Gmelina	Gmelina arborea Roxb.	Lamiaceae / Verbenaceae	0,47	8,3	6	8	0,04	0,01	18,08	0,02	0,21	0,78
6261	116	2	Sukamantri	>500	Suren	Toona sureni (Blume) Merr.	Meliaceae	0,39	12,7	8	9	0,04	0,01	37,38	0,04	0,44	1,61
6262	116	2	Sukamantri	>500	Suren	Toona sureni (Blume) Merr.	Meliaceae	0,39	9,2	5	6	0,04	0,01	14,30	0,01	0,17	0,62
6263	116	2	Sukamantri	>500	Afrika	Maesopsis eminii Engl.	Rhamnaceae	0,43	7,6	8	9	0,04	0,00	16,03	0,02	0,19	0,69
6264	116	2	Sukamantri	>500	Afrika	Maesopsis eminii Engl.	Rhamnaceae	0,43	9,9	9	10	0,04	0,01	28,22	0,03	0,33	1,22
6265	116	2	Sukamantri	>500	Afrika	Maesopsis eminii Engl.	Rhamnaceae	0,43	8,3	9	10	0,04	0,01	20,45	0,02	0,24	0,88
6266	116	2	Sukamantri	>500	Afrika	Maesopsis eminii Engl.	Rhamnaceae	0,43	14,6	10	11	0,04	0,02	63,45	0,06	0,75	2,74
6267	116	2	Sukamantri	>500	Gmelina	Gmelina arborea Roxb.	Lamiaceae / Verbenaceae	0,47	11,8	7	8	0,04	0,01	34,51	0,03	0,41	1,49
6268	116	2	Sukamantri	>500	Afrika	Maesopsis eminii Engl.	Rhamnaceae	0,43	19,7	10	12	0,04	0,03	118,73	0,12	1,40	5,12
6269	116	2	Sukamantri	>500	Afrika	Maesopsis eminii Engl.	Rhamnaceae	0,43	7,3	7	8	0,04	0,00	13,31	0,01	0,16	0,57
6270	116	2	Sukamantri	>500	Afrika	Maesopsis eminii Engl.	Rhamnaceae	0,43	17,8	10	11	0,04	0,02	90,99	0,09	1,07	3,92
6271	116	2	Sukamantri	>500	Afrika	Maesopsis eminii Engl.	Rhamnaceae	0,43	11,1	7	8	0,04	0,01	28,73	0,03	0,34	1,24
6272	116	2	Sukamantri	>500	Kaliandra	Calliandra calothyrsus Meisn.	Leguminosae / Fabaceae	0,65	8,6	3	6	0,04	0,01	20,04	0,02	0,24	0,86
6273	116	2	Sukamantri	>500	Gmelina	Gmelina arborea Roxb.	Lamiaceae / Verbenaceae	0,47	5,1	4	6	0,04	0,00	5,71	0,01	0,07	0,25
6274	116	2	Sukamantri	>500	Afrika	Maesopsis eminii Engl.	Rhamnaceae	0,43	8,6	7	8	0,04	0,01	17,86	0,02	0,21	0,77
6275	116	2	Sukamantri	>500	Afrika	Maesopsis eminii Engl.	Rhamnaceae	0,43	15,0	10	11	0,04	0,02	66,00	0,07	0,78	2,85
6276	116	2	Sukamantri	>500	Afrika	Maesopsis eminii Engl.	Rhamnaceae	0,43	17,2	10	11	0,04	0,02	85,12	0,09	1,00	3,67
6277	116	2	Sukamantri	>500	Afrika	Maesopsis eminii Engl.	Rhamnaceae	0,43	13,7	8	10	0,04	0,01	51,39	0,05	0,60	2,22
6278	116	2	Sukamantri	>500	Gmelina	Gmelina arborea Roxb.	Lamiaceae / Verbenaceae	0,47	5,4	4	6	0,04	0,00	6,38	0,01	0,07	0,28
6279	116	2	Sukamantri	>500	Gmelina	Gmelina arborea Roxb.	Lamiaceae / Verbenaceae	0,47	5,4	4	5	0,04	0,00	5,40	0,01	0,06	0,23
6280	116	2	Sukamantri	>500	Gmelina	Gmelina arborea Roxb.	Lamiaceae / Verbenaceae	0,47	6,4	4	5	0,04	0,00	7,27	0,01	0,09	0,31
6281	116	2	Sukamantri	>500	Gmelina	Gmelina arborea Roxb.	Lamiaceae / Verbenaceae	0,47	13,7	8	10	0,04	0,01	55,75	0,06	0,66	2,40
6282	116	2	Sukamantri	>500	Afrika	Maesopsis eminii Engl.	Rhamnaceae	0,43	5,4	7	8	0,04	0,00	7,65	0,01	0,09	0,33
6283	116	2	Sukamantri	>500	Gmelina	Gmelina arborea Roxb.	Lamiaceae / Verbenaceae	0,47	15,0	7	11	0,04	0,02	71,61	0,07	0,84	3,09
6284	116	2	Sukamantri	>500	Afrika	Maesopsis eminii Engl.	Rhamnaceae	0,43	21,3	10	12	0,04	0,04	136,86	0,14	1,61	5,90
6285	116	2	Sukamantri	>500	Afrika	Maesopsis eminii Engl.	Rhamnaceae	0,43	9,9	8	9	0,04	0,01	25,62	0,03	0,30	1,10
6286	116	2	Sukamantri	>500	Afrika	Maesopsis eminii Engl.	Rhamnaceae	0,43	11,5	9	10	0,04	0,01	37,11	0,04	0,44	1,60
6287	116	2	Sukamantri	>500	Gmelina	Gmelina arborea Roxb.	Lamiaceae / Verbenaceae	0,47	4,8	5	7	0,04	0,00	5,84	0,01	0,07	0,25
6288	116	2	Sukamantri	>500	Afrika	Maesopsis eminii Engl.	Rhamnaceae	0,43	11,8	12	13	0,04	0,01	49,62	0,05	0,58	2,14
6289	116	2	Sukamantri	>500	Afrika	Maesopsis eminii Engl.	Rhamnaceae	0,43	15,0	11	12	0,04	0,02	71,48	0,07	0,84	3,08
6290	116	2	Sukamantri	>500	Gmelina	Gmelina arborea Roxb.	Lamiaceae / Verbenaceae	0,47	6,4	6	7	0,04	0,00	9,89	0,01	0,12	0,43
6291	116	2	Sukamantri	>500	Afrika	Maesopsis eminii Engl.	Rhamnaceae	0,43	7,3	9	10	0,04	0,00	16,33	0,02	0,19	0,70
6292	116	2	Sukamantri	>500	Afrika	Maesopsis eminii Engl.	Rhamnaceae	0,43	10,8	10	11	0,04	0,01	36,47	0,04	0,43	1,57
6293	116	2	Sukamantri	>500	Afrika	Maesopsis eminii Engl.	Rhamnaceae	0,43	19,1	12	13	0,04	0,03	120,32	0,12	1,41	5,19
6294	116	2	Sukamantri	>500	Gmelina	Gmelina arborea Roxb.	Lamiaceae / Verbenaceae	0,47	15,0	7	11	0,04	0,02	71,61	0,07	0,84	3,09
6295	116	2	Sukamantri	>500	Gmelina	Gmelina arborea Roxb.	Lamiaceae / Verbenaceae	0,47	9,2	4	5	0,04	0,01	14,36	0,01	0,17	0,62
6296	116	2	Sukamantri	>500	Afrika	Maesopsis eminii Engl.	Rhamnaceae	0,43	13,7	10	11	0,04	0,01	56,08	0,06	0,66	2,42
6297	116	2	Sukamantri	>500	Gmelina	Gmelina arborea Roxb.	Lamiaceae / Verbenaceae	0,47	5,7	4	6	0,04	0,00	7,08	0,01	0,08	0,31
6298	116	2	Sukamantri	>500	Gmelina	Gmelina arborea Roxb.	Lamiaceae / Verbenaceae	0,47	7,3	5	7	0,04	0,00	12,78	0,01	0,15	0,55
6299	116	2	Sukamantri	>500	Afrika	Maesopsis eminii Engl.	Rhamnaceae	0,43	15,9	9	10	0,04	0,02	67,75	0,07	0,80	2,92
6300	116	2	Sukamantri	>500	Gmelina	Gmelina arborea Roxb.	Lamiaceae / Verbenaceae	0,47	8,0	4	7	0,04	0,00	14,89	0,01	0,17	0,64
6301	116	2	Sukamantri	>500	Gmelina	Gmelina arborea Roxb.	Lamiaceae / Verbenaceae	0,47	23,2	9	12	0,04	0,04	173,74	0,17	2,04	7,49
6302	117	1	Sukamantri	>500	Ganitri	Elaeocarpus ganitrus Roxb. ex G.	Elaeocarpaceae	0,49	6,7	4	8	0,01	0,00	12,70	0,01	0,60	2,19
6303	117	1	Sukamantri	>500	Afrika	Maesopsis eminii Engl.	Rhamnaceae	0,43	9,5	9	10	0,01	0,00	26,57	0,03	1,25	4,58
6304	117	1	Sukamantri	>500	Afrika	Maesopsis eminii Engl.	Rhamnaceae	0,43	11,1	9,5	10,5	0,01	0,00	36,86	0,04	1,73	6,36
6305	117	1	Sukamantri	>500	Afrika	Maesopsis eminii Engl.	Rhamnaceae	0,43	10,2	9	10	0,01	0,00	29,91	0,03	1,41	5,16
6306	117	1	Sukamantri	>500	Afrika	Maesopsis eminii Engl.	Rhamnaceae	0,43	4,8	5	6	0,01	0,00	4,67	0,00	0,22	0,81
6307	117	1	Sukamantri	>500	Afrika	Maesopsis eminii Engl.	Rhamnaceae	0,43	10,5	9	10	0,01	0,00	31,64	0,03	1,49	5,46
6308	117	1	Sukamantri	>500	Ganitri	Elaeocarpus ganitrus Roxb. ex G.	Elaeocarpaceae	0,49	7,6	6	9	0,01	0,00	18,07	0,02	0,85	3,12
6309	117	1	Sukamantri	>500	Afrika	Maesopsis eminii Engl.	Rhamnaceae	0,43	11,1	10	11	0,01	0,00	38,46	0,04	1,81	6,63
6310	117	1	Sukamantri	>500	Afrika	Maesopsis eminii Engl.	Rhamnaceae	0,43	12,7	10	11	0,01	0,00	49,12	0,05	2,31	8,47
6311	117	1	Sukamantri	>500	Ganitri	Elaeocarpus ganitrus Roxb. ex G.	Elaeocarpaceae	0,49	4,8	4	5	0,01	0,00	4,46	0,00	0,21	0,77
6312	117	1	Sukamantri	>500	Afrika	Maesopsis eminii Engl.	Rhamnaceae	0,43	11,5	9	10	0,01	0,00	37,11	0,04	1,74	6,40
6313	117	1	Sukamantri	>500	Afrika	Maesopsis eminii Engl.	Rhamnaceae	0,43	10,5	7	8	0,01	0,00	25,79	0,03	1,21	4,45
6314	117	1	Sukamantri	>500	Ganitri	Elaeocarpus ganitrus Roxb. ex G.	Elaeocarpaceae	0,49	7,6	6	9	0,01	0,00	18,07	0,02	0,85	3,12
6315	117	1	Sukamantri	>500	Afrika	Maesopsis eminii Engl.	Rhamnaceae	0,43	9,5	9	10	0,01	0,00	26,57	0,03	1,25	4,58
6316	117	1	Sukamantri	>500	Afrika	Maesopsis eminii Engl.	Rhamnaceae	0,43	5,1	5	6	0,01	0,00	5,26	0,01	0,25	0,91
6317	117	1	Sukamantri	>500	Ganitri	Elaeocarpus ganitrus Roxb. ex G.	Elaeocarpaceae	0,49	11,1	6	10	0,01	0,00	39,73	0,04	1,87	6,85
6318	117	1	Sukamantri	>500	Afrika	Maesopsis eminii Engl.	Rhamnaceae	0,43	8,9	9	10	0,01	0,00	23,42	0,02	1,10	4,04

6319	117	1	Sukamantri	>500	Ganitri	Elaeocarpus ganitrus Roxb. ex G.	Elaeocarpaceae	0,49	6,4	4	5	0,01	0,00	7,55	0,01	0,35	1,30
6320	117	1	Sukamantri	>500	Ganitri	Elaeocarpus ganitrus Roxb. ex G.	Elaeocarpaceae	0,49	7,6	4	7	0,01	0,00	14,35	0,01	0,67	2,48
6321	117	1	Sukamantri	>500	Afrika	Maesopsis eminii Engl.	Rhamnaceae	0,43	6,0	6	7	0,01	0,00	8,30	0,01	0,39	1,43
6322	117	1	Sukamantri	>500	Afrika	Maesopsis eminii Engl.	Rhamnaceae	0,43	7,0	7	8	0,01	0,00	12,27	0,01	0,58	2,12
6323	117	1	Sukamantri	>500	Afrika	Maesopsis eminii Engl.	Rhamnaceae	0,43	10,5	8	9	0,01	0,00	28,73	0,03	1,35	4,96
6324	117	1	Sukamantri	>500	Ganitri	Elaeocarpus ganitrus Roxb. ex G.	Elaeocarpaceae	0,49	4,5	5	6	0,01	0,00	4,64	0,00	0,22	0,80
6325	117	1	Sukamantri	>500	Afrika	Maesopsis eminii Engl.	Rhamnaceae	0,43	7,3	8	9	0,01	0,00	14,83	0,01	0,70	2,56
6326	117	1	Sukamantri	>500	Afrika	Maesopsis eminii Engl.	Rhamnaceae	0,43	6,4	7	8	0,01	0,00	10,31	0,01	0,48	1,78
6327	117	1	Sukamantri	>500	Ganitri	Elaeocarpus ganitrus Roxb. ex G.	Elaeocarpaceae	0,49	6,7	4	7	0,01	0,00	11,24	0,01	0,53	1,94
6328	117	1	Sukamantri	>500	Afrika	Maesopsis eminii Engl.	Rhamnaceae	0,43	9,5	7	8	0,01	0,00	21,66	0,02	1,02	3,74
6329	117	1	Sukamantri	>500	Afrika	Maesopsis eminii Engl.	Rhamnaceae	0,43	18,5	8	9	0,04	0,03	80,74	0,08	0,95	3,48
6330	117	1	Sukamantri	>500	Afrika	Maesopsis eminii Engl.	Rhamnaceae	0,43	11,5	8	9	0,04	0,01	33,70	0,03	0,40	1,45
6331	117	1	Sukamantri	>500	Afrika	Maesopsis eminii Engl.	Rhamnaceae	0,43	10,2	9	10	0,04	0,01	29,91	0,03	0,35	1,29
6332	117	1	Sukamantri	>500	Puspa	Schima wallichii Choisy	Theaceae	0,74	13,7	6	10	0,04	0,01	84,50	0,08	0,99	3,64
6333	117	1	Sukamantri	>500	Afrika	Maesopsis eminii Engl.	Rhamnaceae	0,43	11,5	9	10	0,04	0,01	37,11	0,04	0,44	1,60
6334	117	1	Sukamantri	>500	Afrika	Maesopsis eminii Engl.	Rhamnaceae	0,43	10,8	8	9	0,04	0,01	30,35	0,03	0,36	1,31
6335	117	1	Sukamantri	>500	Ganitri	Elaeocarpus ganitrus Roxb. ex G.	Elaeocarpaceae	0,49	10,2	6	10	0,04	0,01	33,71	0,03	0,40	1,45
6336	117	1	Sukamantri	>500	Afrika	Maesopsis eminii Engl.	Rhamnaceae	0,43	12,4	8	9	0,04	0,01	39,02	0,04	0,46	1,68
6337	117	1	Sukamantri	>500	Afrika	Maesopsis eminii Engl.	Rhamnaceae	0,43	10,2	9	10	0,04	0,01	29,91	0,03	0,35	1,29
6338	117	1	Sukamantri	>500	Afrika	Maesopsis eminii Engl.	Rhamnaceae	0,43	12,7	10	11	0,04	0,01	49,12	0,05	0,58	2,12
6339	117	1	Sukamantri	>500	Afrika	Maesopsis eminii Engl.	Rhamnaceae	0,43	10,2	9	10,5	0,04	0,01	31,28	0,03	0,37	1,35
6340	117	1	Sukamantri	>500	Afrika	Maesopsis eminii Engl.	Rhamnaceae	0,43	15,0	10	11	0,04	0,02	66,00	0,07	0,78	2,85
6341	117	1	Sukamantri	>500	Afrika	Maesopsis eminii Engl.	Rhamnaceae	0,43	17,2	11	12	0,04	0,02	92,18	0,09	1,08	3,98
6342	117	1	Sukamantri	>500	Afrika	Maesopsis eminii Engl.	Rhamnaceae	0,43	11,1	4	8	0,04	0,01	28,73	0,03	0,34	1,24
6343	117	1	Sukamantri	>500	Afrika	Maesopsis eminii Engl.	Rhamnaceae	0,43	11,5	9	10	0,04	0,01	37,11	0,04	0,44	1,60
6344	117	1	Sukamantri	>500	Afrika	Maesopsis eminii Engl.	Rhamnaceae	0,43	16,5	8	9	0,04	0,02	66,10	0,07	0,78	2,85
6345	117	1	Sukamantri	>500	Afrika	Maesopsis eminii Engl.	Rhamnaceae	0,43	10,2	8	9	0,04	0,01	27,16	0,03	0,32	1,17
6346	117	1	Sukamantri	>500	Afrika	Maesopsis eminii Engl.	Rhamnaceae	0,43	12,1	7	8	0,04	0,01	33,40	0,03	0,39	1,44
6347	117	1	Sukamantri	>500	Afrika	Maesopsis eminii Engl.	Rhamnaceae	0,43	16,2	12	13	0,04	0,02	89,33	0,09	1,05	3,85
6348	117	1	Sukamantri	>500	Afrika	Maesopsis eminii Engl.	Rhamnaceae	0,43	13,0	8	9	0,04	0,01	42,76	0,04	0,50	1,84
6349	117	1	Sukamantri	>500	Puspa	Schima wallichii Choisy	Theaceae	0,74	15,0	9	10,5	0,04	0,02	104,00	0,10	1,22	4,48
6350	117	1	Sukamantri	>500	Puspa	Schima wallichii Choisy	Theaceae	0,74	16,9	9	11	0,04	0,02	135,25	0,14	1,59	5,83
6351	117	1	Sukamantri	>500	Afrika	Maesopsis eminii Engl.	Rhamnaceae	0,43	13,7	8	9	0,04	0,01	46,66	0,05	0,55	2,01
6352	117	1	Sukamantri	>500	Puspa	Schima wallichii Choisy	Theaceae	0,74	16,2	7	9	0,04	0,02	104,88	0,10	1,23	4,52
6353	117	1	Sukamantri	>500	Afrika	Maesopsis eminii Engl.	Rhamnaceae	0,43	15,0	8	9	0,04	0,02	54,92	0,05	0,65	2,37
6354	117	1	Sukamantri	>500	Afrika	Maesopsis eminii Engl.	Rhamnaceae	0,43	15,0	7	8	0,04	0,02	49,30	0,05	0,58	2,13
6355	117	1	Sukamantri	>500	Afrika	Maesopsis eminii Engl.	Rhamnaceae	0,43	13,7	10	11	0,04	0,01	56,08	0,06	0,66	2,42
6356	117	1	Sukamantri	>500	Afrika	Maesopsis eminii Engl.	Rhamnaceae	0,43	10,2	9	10	0,04	0,01	29,91	0,03	0,35	1,29
6357	117	1	Sukamantri	>500	Afrika	Maesopsis eminii Engl.	Rhamnaceae	0,43	20,0	11	13	0,04	0,03	131,57	0,13	1,55	5,67
6358	118	1	Sukamantri	>500	Mahoni	Swietenia macrophylla King	Meliaceae	0,61	18,1	7	11	0,04	0,03	129,47	0,13	1,52	5,58
6359	118	1	Sukamantri	>500	Afrika	Maesopsis eminii Engl.	Rhamnaceae	0,43	6,7	3	5	0,04	0,00	7,33	0,01	0,09	0,32
6360	118	1	Sukamantri	>500	Mahoni	Swietenia macrophylla King	Meliaceae	0,61	16,9	6	9	0,04	0,02	94,28	0,09	1,11	4,07
6361	118	1	Sukamantri	>500	Mahoni	Swietenia macrophylla King	Meliaceae	0,61	23,2	9	14	0,04	0,04	254,06	0,25	2,99	10,96
6362	118	1	Sukamantri	>500	Mahoni	Swietenia macrophylla King	Meliaceae	0,61	15,9	60	10	0,04	0,02	93,32	0,09	1,10	4,02
6363	118	1	Sukamantri	>500	Mahoni	Swietenia macrophylla King	Meliaceae	0,61	15,0	6	9	0,04	0,02	75,66	0,08	0,89	3,26
6364	118	1	Sukamantri	>500	Mahoni	Swietenia macrophylla King	Meliaceae	0,61	23,2	7	14	0,04	0,04	254,06	0,25	2,99	10,96
6365	118	1	Sukamantri	>500	Mahoni	Swietenia macrophylla King	Meliaceae	0,61	8,9	4	7	0,04	0,01	23,27	0,02	0,27	1,00
6366	118	1	Sukamantri	>500	Mahoni	Swietenia macrophylla King	Meliaceae	0,61	8,0	4	7	0,04	0,00	18,91	0,02	0,22	0,82
6367	118	1	Sukamantri	>500	Mahoni	Swietenia macrophylla King	Meliaceae	0,61	26,4	8	14	0,04	0,05	321,43	0,32	3,78	13,86
6368	118	1	Sukamantri	>500	Mahoni	Swietenia macrophylla King	Meliaceae	0,61	18,5	8	14	0,04	0,03	166,70	0,17	1,96	7,19
6369	118	1	Sukamantri	>500	Mahoni	Swietenia macrophylla King	Meliaceae	0,61	13,0	7	10	0,04	0,01	64,88	0,06	0,76	2,80
6370	118	1	Sukamantri	>500	Mahoni	Swietenia macrophylla King	Meliaceae	0,61	25,5	9	12	0,04	0,05	260,90	0,26	3,07	11,25
6371	118	1	Sukamantri	>500	Mahoni	Swietenia macrophylla King	Meliaceae	0,61	18,8	9	11	0,04	0,03	137,91	0,14	1,62	5,95
6372	118	1	Sukamantri	>500	Mahoni	Swietenia macrophylla King	Meliaceae	0,61	17,8	7	11	0,04	0,02	125,34	0,13	1,47	5,40
6373	118	1	Sukamantri	>500	Mahoni	Swietenia macrophylla King	Meliaceae	0,61	16,9	7	11	0,04	0,02	113,31	0,11	1,33	4,89
6374	118	1	Sukamantri	>500	Mahoni	Swietenia macrophylla King	Meliaceae	0,61	30,2	7	13	0,04	0,07	384,63	0,38	4,52	16,59
6375	118	1	Sukamantri	>500	Mahoni	Swietenia macrophylla King	Meliaceae	0,61	12,4	4	5	0,04	0,01	31,37	0,03	0,37	1,35
6376	118	1	Sukamantri	>500	Mahoni	Swietenia macrophylla King	Meliaceae	0,61	25,8	8	14	0,04	0,05	307,38	0,31	3,61	13,26
6377	118	1	Sukamantri	>500	Mahoni	Swietenia macrophylla King	Meliaceae	0,61	21,0	8	12	0,04	0,03	183,41	0,18	2,16	7,91
6378	118	1	Sukamantri	>500	Mahoni	Swietenia macrophylla King	Meliaceae	0,61	14,0	6	10	0,04	0,02	73,84	0,07	0,87	3,18
6379	118	1	Sukamantri	>500	Cengkeh	Syzygium aromaticum (L.) Merr. a	Myrtaceae	0,79	15,9	6	10	0,04	0,02	118,27	0,12	1,39	5,10
6380	118	1	Sukamantri	>500	Mahoni	Swietenia macrophylla King	Meliaceae	0,61	13,0	6	10	0,04	0,01	64,88	0,06	0,76	2,80
6381	118	1	Sukamantri	>500	Mahoni	Swietenia macrophylla King	Meliaceae	0,61	12,7	70	10	0,04	0,01	62,01	0,06	0,73	2,67
6382	118	1	Sukamantri	>500	Mahoni	Swietenia macrophylla King	Meliaceae	0,61	25,5	8	14	0,04	0,05	300,47	0,30	3,53	12,96
6383	118	1	Sukamantri	>500	Mahoni	Swietenia macrophylla King	Meliaceae	0,61	17,5	5	10	0,04	0,02	111,13	0,11	1,31	4,79
6384	118	1	Sukamantri	>500	Mahoni	Swietenia macrophylla King	Meliaceae	0,61	14,3	5	10	0,04	0,02	76,94	0,08	0,90	3,32
6385	118	1	Sukamantri	>500	Mahoni	Swietenia macrophylla King	Meliaceae	0,61	22,3	60	10	0,04	0,04	172,86	0,17	2,03	7,45
6386	118	1	Sukamantri	>500	Mahoni	Swietenia macrophylla King	Meliaceae	0,61	21,3	7	10	0,04	0,04	159,53	0,16	1,87	6,88
6387	118	1	Sukamantri	>500	Afrika	Maesopsis eminii Engl.	Rhamnaceae	0,43	19,4	12	13	0,04	0,03	124,01	0,12	1,46	5,35
6388	118	1	Sukamantri	>500	Afrika	Maesopsis eminii Engl.	Rhamnaceae	0,43	28,6	12	14	0,04	0,06	270,65	0,27	3,18	11,67
6389	118	1	Sukamantri	>500	Afrika	Maesopsis eminii Engl.	Rhamnaceae	0,43	36,0	15	17	0,04	0,10	490,58	0,49	5,76	21,15
6390	118	1	Sukamantri	>500	Mahoni	Swietenia macrophylla King	Meliaceae	0,61	21,0	7	10	0,04	0,03	155,20	0,16	1,82	6,69
6391	118	1	Sukamantri	>500	Mahoni	Swietenia macrophylla King	Meliaceae	0,61	12,4	4	7	0,04	0,01	42,70	0,04	0,50	1,84
6392	118	1	Sukamantri	>500	Mahoni	Swietenia macrophylla King	Meliaceae	0,61	30,9	12	15	0,04	0,07	455,56	0,46	5,35	19,64
6393	118	1	Sukamantri	>500	Afrika	Maesopsis eminii Engl.	Rhamnaceae	0,43	21,0	12	13	0,04	0,03	143,27	0,14	1,68	6,18
6394	118	1	Sukamantri	>500	Mahoni	Swietenia macrophylla King	Meliaceae	0,61	16,9	6	9	0,04	0,02	94,28	0,09	1,11	4,07
6395	118	1	Sukamantri	>500	Mahoni	Swietenia macrophylla King	Meliaceae	0,61	17,5	6	10	0,04	0,02	111,13	0,11	1,31	4,79
6396	118	1	Sukamantri	>500	Afrika	Maesopsis eminii Engl.	Rhamnaceae	0,43	28,6	12	15	0,04	0,06	288,30	0,29	3,39	12,43
6397	118	1	Sukamantri	>500	Mahoni	Swietenia macrophylla King	Meliaceae	0,61	12,7	7	9	0,04	0,01	56,30	0,06	0,66	2,43
6398	118	1	Sukamantri	>500	Afrika	Maesopsis eminii Engl.	Rhamnaceae	0,43	26,7	13	15	0,04	0,06	254,07	0,25	2,99	10,96
6399	118	1	Sukamantri	>500	Mahoni	Swietenia macrophylla King	Meliaceae	0,61	7,0	8	11	0,04	0,00	22,63	0,02	0,27	0,98
6400	118	1	Sukamantri	>500	Mahoni	Swietenia macrophylla King	Meliaceae	0,61	17,2	6	10	0,04	0,02	107,46	0,11	1,26	4,63
6401	118	1	Sukamantri	>500	Mahoni	Swietenia macrophylla King	Meliaceae	0,61	18,1	4	6	0,04	0,03	74,31	0,07	0,87	3,20
6402	118	1	Sukamantri	>500	Mahoni	Swietenia macrophylla King	Meliaceae	0,61	18,1	8	10	0,04	0,03	118,64	0,12	1,39	5,12
6403	118	1	Sukamantri	>500	Mahoni	Swietenia macrophylla King	Meliaceae	0,61	20,0	8	10	0,04	0,03	142,52	0,14	1,67	6,15
6404	118	1	Sukamantri	>500	Mahoni	Swietenia macrophylla King	Meliaceae	0,61	34,0	14	16	0,04	0,09	578,48	0,58	6,80	24,95
6405	118	1	Sukamantri	>500	Mahoni	Swietenia macrophylla King	Meliaceae	0,61	23,2	7	10	0,04	0,04	186,68	0,19	2,19	8,05
6406	118	1	Sukamantri	>500	Afrika	Maesopsis eminii Engl.	Rhamnaceae	0,43	33,1	13	15	0,04	0,09	375,73	0,38	4,41	16,20
6407	119	2	Sukamantri	>500	Afrika	Maesopsis eminii Engl.	Rhamnaceae	0,43	24,8	10	12	0,04	0,05	180,81	0,18	2,12	7,80
6408	119	2	Sukamantri	>500	Mahoni	Swietenia macrophylla King	Meliaceae	0,61	30,9	6	10	0,04	0,07	314,23	0,31	3,69	13,55
6409	119	2	Sukamantri	>500	Kaliandra	Calliandra calothyrsus Meisn.	Leguminosae / Fabaceae	0,65	6,0	2	4	0,04	0,00	7,26	0,01	0,09	0,31
6410	119	2	Sukamantri	>500	Afrika	Maesopsis eminii Engl.	Rhamnaceae	0,43	20,0	10	11	0,04	0,03	112,90	0,11	1,33	4,87
6411	119	2	Sukamantri	>500	Kaliandra	Calliandra calothyrsus Meisn.	Leguminosae / Fabaceae	0,65	12,4	4	7	0,04	0,01	45,26	0,05	0,53	1,95
6412	119	2	Sukamantri	>500	Kopi	Coffea canephora Pierre ex A.Fro	Rubiaceae	0,41	3,8	2	4	0,04	0,00	4,38	0,00	0,05	0,19
6413	119	2	Sukamantri	>500	Afrika	Maesopsis eminii Engl.	Rhamnaceae	0,43	13,7	10	11	0,04	0,01	56,08	0,06	0,66	2,42
6414	119	2	Sukamantri	>500	Kopi	Coffea canephora Pierre ex A.Fro	Rubiaceae	0,41	3,8	2	3	0,04	0,00	4,38	0,00	0,05	0,19
6415	119	2	Sukamantri	>500	Mahoni	Swietenia macrophylla King	Meliaceae	0,61	26,1	6	9	0,04	0,05	209,74	0,21	2,46	9,04
6416	119	2	Sukamantri	>500	Puspa	Schima wallichii Choisy	Theaceae	0,74	7,6	6	8	0,04	0,00	23,67	0,02	0,28	1,02
6417	119	2	Sukamantri	>500	Puspa	Schima wallichii Choisy	Theaceae	0,74	9,2	4	7	0,04	0,01	29,62	0,03	0,35	1,28
6418	119	2	Sukamantri	>500	Mahoni	Swietenia macrophylla King	Meliaceae	0,61	30,5	10	14	0,04	0,07	419,62	0,42	4,93	18,10
6419	119	2	Sukamantri	>500	Kaliandra	Calliandra calothyrsus Meisn.	Leguminosae / Fabaceae	0,65	6,7	4	6	0,04	0,00	12,64	0,01	0,15	0,55
6420	119	2	Sukamantri	>500	Nangka	Artocarpus heterophyllus Lam.	Moraceae	0,61	24,8	9	14	0,04	0,05	286,85	0,29	3,37	12,37
6421	119	2	Sukamantri	>500	Kaliandra	Calliandra calothyrsus Meisn.	Leguminosae / Fabaceae	0,65	6,4	5	7	0,04	0,00	13,32	0,01	0,16	0,57
6422	119	2	Sukamantri	>500	Puspa	Schima wallichii Choisy	Theaceae	0,74	13,7	8	11	0,04	0,01	92,21	0,09	1,08	3,98
6423	119	2	Sukamantri	>500	Kaliandra	Calliandra calothyrsus Meisn.	Leguminosae / Fabaceae	0,65	6,0	4	6	0,04	0,00	10,52	0,01	0,12	0,45
6424	119	2	Sukamantri	>500	Kaliandra	Calliandra calothyrsus Meisn.	Leguminosae / Fabaceae	0,65	4,8	4	6	0,04	0,00	6,83	0,01	0,08	0,29
6425	119	2	Sukamantri	>500	Afrika	Maesopsis eminii Engl.	Rhamnaceae	0,43	6,4	7	8	0,04	0,00	10,31	0,01	0,12	0,44
6426	119	2	Sukamantri	>500	Afrika	Maesopsis eminii Engl.	Rhamnaceae	0,43	15,3	11	12	0,04	0,02	74,29	0,07	0,87	3,20
6427	119	2	Sukamantri	>500	Afrika	Maesopsis eminii Engl.	Rhamnaceae	0,43	5,4	6	7	0,04	0,00	6,77	0,01	0,08	0,29
6428	119	2	Sukamantri	>500	Afrika	Maesopsis eminii Engl.	Rhamnaceae	0,43	8,0	8	9	0,04	0,00	17,28	0,02	0,20	0,75
6429	119	2	Sukamantri	>500	Nangka	Artocarpus heterophyllus Lam.	Moraceae	0,61	11,8	3	5	0,04	0,01	28,49	0,03	0,33	1,23
6430	119	2	Sukamantri	>500	Kaliandra	Calliandra calothyrsus Meisn.	Leguminosae / Fabaceae	0,65	4,1	3	5	0,04	0,00	4,44	0,00	0,05	0,19
6431	119	2	Sukamantri	>500	Afrika	Maesopsis eminii Engl.	Rhamnaceae	0,43	16,9	10	11	0,04	0,02	82,26	0,08	0,97	3,55
6432	119	2	Sukamantri	>500	Afrika	Maesopsis eminii Engl.	Rhamnaceae	0,43	12,4	8	9	0,04	0,01	39,02	0,04	0,46	1,68
6433	119	2	Sukamantri	>500	Afrika	Maesopsis eminii Engl.	Rhamnaceae	0,43	21,0	12	13	0,04	0,03	143,27	0,14	1,68	6,18
6434	119	2	Sukamantri	>500	Padali	Radermachera gigantea (Blume)	Bignoniaceae	0,48	10,8	7	9	0,04	0,01	33,57	0,03	0,39	1,45
6435	119	2	Sukamantri	>500	Afrika	Maesopsis eminii Engl.	Rhamnaceae	0,43	6,4	5	6	0,04	0,00	7,92	0,01	0,09	0,34
6436	119	2	Sukamantri	>500	Afrika	Maesopsis eminii Engl.	Rhamnaceae	0,43	12,1	10	11	0,04	0,01	44,72	0,04	0,53	1,93
6437	119	2	Sukamantri	>500	Kaliandra	Calliandra calothyrsus Meisn.	Leguminosae / Fabaceae	0,65	6,7	4	7	0,04	0,00	14,56	0,01	0,17	0,63
6438	119	2	Sukamantri	>500	Puspa	Schima wallichii Choisy	Theaceae	0,74	13,4	6	10	0,04	0,01	80,93	0,08	0,95	3,49
6439	119	2	Sukamantri	>500	Afrika	Maesopsis eminii Engl.	Rhamnaceae	0,43	17,8	12	13	0,04	0,02	106,03	0,11	1,25	4,57
6440	119	2	Sukamantri	>500	Afrika	Maesopsis eminii Engl.	Rhamnaceae	0,43	17,5	10	11	0,04	0,02	88,03	0,09	1,03	3,80
6441	119	2	Sukamantri	>500	Afrika	Maesopsis eminii Engl.	Rhamnaceae	0,43	5,1	5	6	0,04	0,00	5,26	0,01	0,06	0,23
6442	119	2	Sukamantri	>500	Afrika	Maesopsis eminii Engl.	Rhamnaceae	0,43	6,0	7	8	0,04	0,00	9,38	0,01	0,11	0,40

6443	119	2	Sukamantri	>500	Afrika	Maesopsis eminii Engl.	Rhamnaceae	0,43	27,7	15	15	0,04	0,06	270,94	0,27	3,18	11,68
6444	119	2	Sukamantri	>500	Afrika	Maesopsis eminii Engl.	Rhamnaceae	0,43	9,5	9	10	0,04	0,01	26,57	0,03	0,31	1,15
6445	119	2	Sukamantri	>500	Kaliandra	Calliandra calothyrsus Meisn.	Leguminosae / Fabaceae	0,65	5,4	4	6	0,04	0,00	8,58	0,01	0,10	0,37
6446	119	2	Sukamantri	>500	Afrika	Maesopsis eminii Engl.	Rhamnaceae	0,43	9,2	9	10	0,04	0,01	24,97	0,02	0,29	1,08
6447	119	2	Sukamantri	>500	Puspa	Schima wallichii Choisy	Theaceae	0,74	13,7	9	11	0,04	0,01	92,21	0,09	1,08	3,98
6448	119	2	Sukamantri	>500	Huni	Antidesma bunius (L.) Spreng.	Phyllanthaceae	0,51	20,0	4	8	0,04	0,03	98,60	0,10	1,16	4,25
6449	119	2	Sukamantri	>500	Mahoni	Swietenia macrophylla King	Meliaceae	0,61	20,4	7	10	0,04	0,03	146,69	0,15	1,72	6,33
6450	119	2	Sukamantri	>500	Nangka	Artocarpus heterophyllus Lam.	Moraceae	0,61	4,5	4	5	0,04	0,00	4,80	0,00	0,06	0,21
6451	119	2	Sukamantri	>500	Mahoni	Swietenia macrophylla King	Meliaceae	0,61	29,6	7	12	0,04	0,07	343,77	0,34	4,04	14,82
6452	119	2	Sukamantri	>500	Afrika	Maesopsis eminii Engl.	Rhamnaceae	0,43	20,0	13	14	0,04	0,03	140,81	0,14	1,65	6,07
6453	119	2	Sukamantri	>500	Afrika	Maesopsis eminii Engl.	Rhamnaceae	0,43	22,3	13	15	0,04	0,04	181,93	0,18	2,14	7,85
6454	119	2	Sukamantri	>500	Mahoni	Swietenia macrophylla King	Meliaceae	0,61	16,9	6	11	0,04	0,02	113,31	0,11	1,33	4,89
6455	119	2	Sukamantri	>500	Kaliandra	Calliandra calothyrsus Meisn.	Leguminosae / Fabaceae	0,65	6,0	5	7	0,04	0,00	12,12	0,01	0,14	0,52
6456	119	2	Sukamantri	>500	Afrika	Maesopsis eminii Engl.	Rhamnaceae	0,43	16,5	12	13	0,04	0,02	92,57	0,09	1,09	3,99
6457	119	2	Sukamantri	>500	Afrika	Maesopsis eminii Engl.	Rhamnaceae	0,43	8,9	10	11	0,04	0,01	25,56	0,03	0,30	1,10
6458	119	2	Sukamantri	>500	Puspa	Schima wallichii Choisy	Theaceae	0,74	9,2	8	9	0,04	0,01	37,29	0,04	0,44	1,61
6459	119	2	Sukamantri	>500	Afrika	Maesopsis eminii Engl.	Rhamnaceae	0,43	26,4	14	15	0,04	0,05	248,56	0,25	2,92	10,72
6460	119	2	Sukamantri	>500	Afrika	Maesopsis eminii Engl.	Rhamnaceae	0,43	20,4	13	13	0,04	0,03	135,42	0,14	1,59	5,84
6461	119	2	Sukamantri	>500	Afrika	Maesopsis eminii Engl.	Rhamnaceae	0,43	9,5	9	10	0,04	0,01	26,57	0,03	0,31	1,15
6462	119	2	Sukamantri	>500	Afrika	Maesopsis eminii Engl.	Rhamnaceae	0,43	16,9	12	13	0,04	0,02	95,86	0,10	1,13	4,13
6463	119	2	Sukamantri	>500	Afrika	Maesopsis eminii Engl.	Rhamnaceae	0,43	8,6	9	10	0,04	0,01	21,91	0,02	0,26	0,94
6464	119	2	Sukamantri	>500	Manglid	Magnolia champaca (L.) Baill. ex	Magnoliaceae	0,53	8,9	6	10	0,04	0,01	28,36	0,03	0,33	1,22
6465	119	2	Sukamantri	>500	Afrika	Maesopsis eminii Engl.	Rhamnaceae	0,43	10,2	8	9	0,04	0,01	27,16	0,03	0,32	1,17
6466	119	2	Sukamantri	>500	Afrika	Maesopsis eminii Engl.	Rhamnaceae	0,43	24,5	12	14	0,04	0,05	203,37	0,20	2,39	8,77
6467	119	2	Sukamantri	>500	Cangcaratan	Nauclea subdita (Korth.) Steud	Rubiaceae	0,32	12,4	10	11	0,04	0,01	35,78	0,04	0,42	1,54
6468	119	2	Sukamantri	>500	Cangcaratan	Nauclea subdita (Korth.) Steud	Rubiaceae	0,32	19,1	9	11	0,04	0,03	78,76	0,08	0,93	3,40
6469	119	2	Sukamantri	>500	Afrika	Maesopsis eminii Engl.	Rhamnaceae	0,43	26,1	11	14	0,04	0,05	228,21	0,23	2,68	9,84
6470	119	2	Sukamantri	>500	Cangcaratan	Nauclea subdita (Korth.) Steud	Rubiaceae	0,32	5,7	3	5	0,04	0,00	4,21	0,00	0,05	0,18
6471	119	2	Sukamantri	>500	Kilalayu	Lepisanthes tetraphylla Radlk.	Sapindaceae	0,69	6,0	2,5	4	0,04	0,00	7,67	0,01	0,09	0,33
6472	119	2	Sukamantri	>500	Puspa	Schima wallichii Choisy	Theaceae	0,74	15,0	7	10	0,04	0,02	99,45	0,10	1,17	4,29
6473	119	2	Sukamantri	>500	Mahoni	Swietenia macrophylla King	Meliaceae	0,61	31,8	8	13	0,04	0,08	422,53	0,42	4,96	18,22
6474	119	2	Sukamantri	>500	Kaliandra	Calliandra calothyrsus Meisn.	Leguminosae / Fabaceae	0,65	11,1	4	7	0,04	0,01	37,12	0,04	0,44	1,60
6475	119	2	Sukamantri	>500	Kaliandra	Calliandra calothyrsus Meisn.	Leguminosae / Fabaceae	0,65	5,4	4	6	0,04	0,00	8,58	0,01	0,10	0,37
6476	119	2	Sukamantri	>500	Kaliandra	Calliandra calothyrsus Meisn.	Leguminosae / Fabaceae	0,65	6,7	3	5	0,04	0,00	10,70	0,01	0,13	0,46
6477	119	2	Sukamantri	>500	Afrika	Maesopsis eminii Engl.	Rhamnaceae	0,43	9,5	9	10	0,04	0,01	26,57	0,03	0,31	1,15
6478	119	2	Sukamantri	>500	Mahoni	Swietenia macrophylla King	Meliaceae	0,61	22,6	7	10	0,04	0,04	177,41	0,18	2,08	7,65
6479	119	2	Sukamantri	>500	Padali	Radermachera gigantea (Blume)	Bignoniaceae	0,48	9,2	4	6	0,04	0,01	17,30	0,02	0,20	0,75
6480	119	2	Sukamantri	>500	Mahoni	Swietenia macrophylla King	Meliaceae	0,61	12,7	4	6	0,04	0,01	38,84	0,04	0,46	1,67
6481	120	1	Sukamantri	>500	Ganitri	Elaeocarpus ganitrus Roxb. ex G.	Elaeocarpaceae	0,49	6,4	9	10	0,01	0,00	14,25	0,01	0,67	2,46
6482	120	1	Sukamantri	>500	Ganitri	Elaeocarpus ganitrus Roxb. ex G.	Elaeocarpaceae	0,49	12,7	8	11	0,01	0,00	55,36	0,06	2,60	9,55
6483	120	1	Sukamantri	>500	Ganitri	Elaeocarpus ganitrus Roxb. ex G.	Elaeocarpaceae	0,49	3,2	3	4	0,01	0,00	1,73	0,00	0,08	0,30
6484	120	1	Sukamantri	>500	Ganitri	Elaeocarpus ganitrus Roxb. ex G.	Elaeocarpaceae	0,49	15,9	9	11	0,01	0,00	83,32	0,08	3,92	14,37
6485	120	1	Sukamantri	>500	Ganitri	Elaeocarpus ganitrus Roxb. ex G.	Elaeocarpaceae	0,49	11,1	8	10	0,01	0,00	39,73	0,04	1,87	6,85
6486	120	1	Sukamantri	>500	Ganitri	Elaeocarpus ganitrus Roxb. ex G.	Elaeocarpaceae	0,49	7,0	8	9	0,01	0,00	15,41	0,02	0,72	2,66
6487	120	1	Sukamantri	>500	Ganitri	Elaeocarpus ganitrus Roxb. ex G.	Elaeocarpaceae	0,49	5,7	7	8	0,01	0,00	9,58	0,01	0,45	1,65
6488	120	1	Sukamantri	>500	Ganitri	Elaeocarpus ganitrus Roxb. ex G.	Elaeocarpaceae	0,49	5,4	7	9	0,01	0,00	9,61	0,01	0,45	1,66
6489	120	1	Sukamantri	>500	Ganitri	Elaeocarpus ganitrus Roxb. ex G.	Elaeocarpaceae	0,49	12,1	9	10	0,01	0,00	46,19	0,05	2,17	7,97
6490	120	1	Sukamantri	>500	Ganitri	Elaeocarpus ganitrus Roxb. ex G.	Elaeocarpaceae	0,49	12,7	10	11	0,01	0,00	55,36	0,06	2,60	9,55
6491	120	1	Sukamantri	>500	Ganitri	Elaeocarpus ganitrus Roxb. ex G.	Elaeocarpaceae	0,49	6,0	6	7,5	0,01	0,00	9,97	0,01	0,47	1,72
6492	120	1	Sukamantri	>500	Ganitri	Elaeocarpus ganitrus Roxb. ex G.	Elaeocarpaceae	0,49	8,3	9	10	0,01	0,00	23,04	0,02	1,08	3,97
6493	120	1	Sukamantri	>500	Ganitri	Elaeocarpus ganitrus Roxb. ex G.	Elaeocarpaceae	0,49	10,5	9	10	0,01	0,00	35,67	0,04	1,68	6,15
6494	120	1	Sukamantri	>500	Ganitri	Elaeocarpus ganitrus Roxb. ex G.	Elaeocarpaceae	0,49	2,5	2,5	3,5	0,01	0,00	1,02	0,00	0,05	0,18
6495	120	1	Sukamantri	>500	Ganitri	Elaeocarpus ganitrus Roxb. ex G.	Elaeocarpaceae	0,49	8,9	7	10	0,01	0,00	26,40	0,03	1,24	4,55
6496	120	1	Sukamantri	>500	Ganitri	Elaeocarpus ganitrus Roxb. ex G.	Elaeocarpaceae	0,49	10,5	10	11	0,01	0,00	38,92	0,04	1,83	6,71
6497	120	1	Sukamantri	>500	Ganitri	Elaeocarpus ganitrus Roxb. ex G.	Elaeocarpaceae	0,49	8,9	7	9	0,01	0,00	23,97	0,02	1,13	4,13
6498	120	1	Sukamantri	>500	Ganitri	Elaeocarpus ganitrus Roxb. ex G.	Elaeocarpaceae	0,49	8,9	8	9	0,01	0,00	23,97	0,02	1,13	4,13
6499	120	1	Sukamantri	>500	Ganitri	Elaeocarpus ganitrus Roxb. ex G.	Elaeocarpaceae	0,49	9,5	6	8	0,01	0,00	24,41	0,02	1,15	4,21
6500	120	1	Sukamantri	>500	Manglid	Magnolia champaca (L.) Baill. ex	Magnoliaceae	0,53	9,5	6	8	0,01	0,00	26,23	0,03	1,23	4,53
6501	120	1	Sukamantri	>500	Ganitri	Elaeocarpus ganitrus Roxb. ex G.	Elaeocarpaceae	0,49	10,2	7	9	0,01	0,00	30,61	0,03	1,44	5,28
6502	120	1	Sukamantri	>500	Ganitri	Elaeocarpus ganitrus Roxb. ex G.	Elaeocarpaceae	0,49	7,3	7	9	0,01	0,00	16,72	0,02	0,79	2,88
6503	120	1	Sukamantri	>500	Ganitri	Elaeocarpus ganitrus Roxb. ex G.	Elaeocarpaceae	0,49	5,4	6	8	0,01	0,00	8,62	0,01	0,41	1,49
6504	120	1	Sukamantri	>500	Ganitri	Elaeocarpus ganitrus Roxb. ex G.	Elaeocarpaceae	0,49	10,2	8	9	0,01	0,00	30,61	0,03	1,44	5,28
6505	120	1	Sukamantri	>500	Ganitri	Elaeocarpus ganitrus Roxb. ex G.	Elaeocarpaceae	0,49	4,1	5	6.5	0,01	0,00	#VALUE!	#VALUE!	#VALUE!	#VALUE!
6506	120	1	Sukamantri	>500	Ganitri	Elaeocarpus ganitrus Roxb. ex G.	Elaeocarpaceae	0,49	12,7	9	11	0,01	0,00	55,36	0,06	2,60	9,55
6507	120	1	Sukamantri	>500	Ganitri	Elaeocarpus ganitrus Roxb. ex G.	Elaeocarpaceae	0,49	9,5	10	11	0,01	0,00	32,68	0,03	1,54	5,64
6508	120	1	Sukamantri	>500	Ganitri	Elaeocarpus ganitrus Roxb. ex G.	Elaeocarpaceae	0,49	7,6	7	8	0,01	0,00	16,22	0,02	0,76	2,80
6509	120	1	Sukamantri	>500	Ganitri	Elaeocarpus ganitrus Roxb. ex G.	Elaeocarpaceae	0,49	6,7	7	8	0,01	0,00	12,70	0,01	0,60	2,19
6510	120	1	Sukamantri	>500	Ganitri	Elaeocarpus ganitrus Roxb. ex G.	Elaeocarpaceae	0,49	6,0	4	6	0,01	0,00	8,12	0,01	0,38	1,40
6511	120	1	Sukamantri	>500	Ganitri	Elaeocarpus ganitrus Roxb. ex G.	Elaeocarpaceae	0,49	4,8	4	6	0,01	0,00	5,27	0,01	0,25	0,91
6512	120	1	Sukamantri	>500	Ganitri	Elaeocarpus ganitrus Roxb. ex G.	Elaeocarpaceae	0,49	6,0	5	6,5	0,01	0,00	8,74	0,01	0,41	1,51
6513	120	1	Sukamantri	>500	Ganitri	Elaeocarpus ganitrus Roxb. ex G.	Elaeocarpaceae	0,49	3,5	3	4	0,01	0,00	2,06	0,00	0,10	0,36
6514	120	1	Sukamantri	>500	Ganitri	Elaeocarpus ganitrus Roxb. ex G.	Elaeocarpaceae	0,49	11,1	6	10	0,04	0,01	39,73	0,04	0,47	1,71
6515	120	1	Sukamantri	>500	Ganitri	Elaeocarpus ganitrus Roxb. ex G.	Elaeocarpaceae	0,49	10,5	9	10	0,04	0,01	35,67	0,04	0,42	1,54
6516	120	1	Sukamantri	>500	Ganitri	Elaeocarpus ganitrus Roxb. ex G.	Elaeocarpaceae	0,49	11,5	8	10	0,04	0,01	41,83	0,04	0,49	1,80
6517	120	1	Sukamantri	>500	Ganitri	Elaeocarpus ganitrus Roxb. ex G.	Elaeocarpaceae	0,49	10,2	7	9	0,04	0,01	30,61	0,03	0,36	1,32
6518	120	1	Sukamantri	>500	Ganitri	Elaeocarpus ganitrus Roxb. ex G.	Elaeocarpaceae	0,49	11,8	5	9	0,04	0,01	39,94	0,04	0,47	1,72
6519	120	1	Sukamantri	>500	Ganitri	Elaeocarpus ganitrus Roxb. ex G.	Elaeocarpaceae	0,49	12,4	6	10	0,04	0,01	48,44	0,05	0,57	2,09
6520	120	1	Sukamantri	>500	Ganitri	Elaeocarpus ganitrus Roxb. ex G.	Elaeocarpaceae	0,49	12,7	10	12	0,04	0,01	59,96	0,06	0,70	2,59
6521	120	1	Sukamantri	>500	Ganitri	Elaeocarpus ganitrus Roxb. ex G.	Elaeocarpaceae	0,49	11,5	8	11	0,04	0,01	45,65	0,05	0,54	1,97
6522	120	1	Sukamantri	>500	Ganitri	Elaeocarpus ganitrus Roxb. ex G.	Elaeocarpaceae	0,49	10,8	8	11	0,04	0,01	41,11	0,04	0,48	1,77
6523	120	1	Sukamantri	>500	Ganitri	Elaeocarpus ganitrus Roxb. ex G.	Elaeocarpaceae	0,49	10,2	7	10	0,04	0,01	33,71	0,03	0,40	1,45
6524	120	1	Sukamantri	>500	Ganitri	Elaeocarpus ganitrus Roxb. ex G.	Elaeocarpaceae	0,49	12,4	10	11	0,04	0,01	52,85	0,05	0,62	2,28
6525	120	1	Sukamantri	>500	Ganitri	Elaeocarpus ganitrus Roxb. ex G.	Elaeocarpaceae	0,49	15,9	10	12	0,04	0,02	90,24	0,09	1,06	3,89
6526	120	1	Sukamantri	>500	Ganitri	Elaeocarpus ganitrus Roxb. ex G.	Elaeocarpaceae	0,49	14,0	10	12	0,04	0,02	71,40	0,07	0,84	3,08
6527	120	1	Sukamantri	>500	Ganitri	Elaeocarpus ganitrus Roxb. ex G.	Elaeocarpaceae	0,49	10,8	9	11	0,04	0,01	41,11	0,04	0,48	1,77
6528	120	1	Sukamantri	>500	Ganitri	Elaeocarpus ganitrus Roxb. ex G.	Elaeocarpaceae	0,49	11,5	9	10	0,04	0,01	41,83	0,04	0,49	1,80
6529	120	1	Sukamantri	>500	Ganitri	Elaeocarpus ganitrus Roxb. ex G.	Elaeocarpaceae	0,49	10,5	8	9	0,04	0,01	32,38	0,03	0,38	1,40
6530	120	1	Sukamantri	>500	Ganitri	Elaeocarpus ganitrus Roxb. ex G.	Elaeocarpaceae	0,49	10,2	7	9	0,04	0,01	30,61	0,03	0,36	1,32
6531	120	1	Sukamantri	>500	Ganitri	Elaeocarpus ganitrus Roxb. ex G.	Elaeocarpaceae	0,49	15,9	8	11	0,04	0,02	83,32	0,08	0,98	3,59
6532	120	1	Sukamantri	>500	Ganitri	Elaeocarpus ganitrus Roxb. ex G.	Elaeocarpaceae	0,49	11,8	7	11	0,04	0,01	48,00	0,05	0,56	2,07
6533	120	1	Sukamantri	>500	Ganitri	Elaeocarpus ganitrus Roxb. ex G.	Elaeocarpaceae	0,49	15,6	7	11	0,04	0,02	80,30	0,08	0,94	3,46
6534	120	1	Sukamantri	>500	Ganitri	Elaeocarpus ganitrus Roxb. ex G.	Elaeocarpaceae	0,49	13,0	10	11	0,04	0,01	57,93	0,06	0,68	2,50
6535	120	1	Sukamantri	>500	Ganitri	Elaeocarpus ganitrus Roxb. ex G.	Elaeocarpaceae	0,49	13,4	10	12	0,04	0,01	65,56	0,07	0,77	2,83
6536	120	1	Sukamantri	>500	Ganitri	Elaeocarpus ganitrus Roxb. ex G.	Elaeocarpaceae	0,49	13,0	9	11	0,04	0,01	57,93	0,06	0,68	2,50
6537	120	1	Sukamantri	>500	Ganitri	Elaeocarpus ganitrus Roxb. ex G.	Elaeocarpaceae	0,49	10,2	9	11	0,04	0,01	36,79	0,04	0,43	1,59
6538	120	1	Sukamantri	>500	Ganitri	Elaeocarpus ganitrus Roxb. ex G.	Elaeocarpaceae	0,49	11,5	10	12	0,04	0,01	49,43	0,05	0,58	2,13
6539	120	1	Sukamantri	>500	Ganitri	Elaeocarpus ganitrus Roxb. ex G.	Elaeocarpaceae	0,49	10,5	9	12	0,04	0,01	42,15	0,04	0,50	1,82
6540	120	1	Sukamantri	>500	Ganitri	Elaeocarpus ganitrus Roxb. ex G.	Elaeocarpaceae	0,49	16,5	9	14	0,04	0,02	111,66	0,11	1,31	4,82
6541	120	1	Sukamantri	>500	Ganitri	Elaeocarpus ganitrus Roxb. ex G.	Elaeocarpaceae	0,49	12,7	7	11	0,04	0,01	55,36	0,06	0,65	2,39
6542	120	1	Sukamantri	>500	Ganitri	Elaeocarpus ganitrus Roxb. ex G.	Elaeocarpaceae	0,49	12,4	9	12	0,04	0,01	57,24	0,06	0,67	2,47
6543	120	1	Sukamantri	>500	Ganitri	Elaeocarpus ganitrus Roxb. ex G.	Elaeocarpaceae	0,49	10,8	8	11	0,04	0,01	41,11	0,04	0,48	1,77
6544	120	1	Sukamantri	>500	Ganitri	Elaeocarpus ganitrus Roxb. ex G.	Elaeocarpaceae	0,49	14,6	7	11	0,04	0,02	71,52	0,07	0,84	3,08
6545	121	3	Sukamantri	>500	Afrika	Maesopsis eminii Engl.	Rhamnaceae	0,43	8,3	10	11	0,04	0,01	22,31	0,02	0,26	0,96
6546	121	3	Sukamantri	>500	Afrika	Maesopsis eminii Engl.	Rhamnaceae	0,43	13,0	11	12	0,04	0,01	55,66	0,06	0,65	2,40
6547	121	3	Sukamantri	>500	Alpukat	Persea americana Mill.	Lauraceae	0,54	5,7	3	5	0,04	0,00	6,81	0,01	0,08	0,29
6548	121	3	Sukamantri	>500	Afrika	Maesopsis eminii Engl.	Rhamnaceae	0,43	3,2	3	3,5	0,04	0,00	1,36	0,00	0,02	0,06
6549	121	3	Sukamantri	>500	Alpukat	Persea americana Mill.	Lauraceae	0,54	4,5	3	4	0,04	0,00	3,50	0,00	0,04	0,15
6550	121	3	Sukamantri	>500	Limus	Mangifera foetida Lour.	Anacardiaceae	0,73	4,8	2	3,5	0,04	0,00	4,63	0,00	0,05	0,20
6551	121	3	Sukamantri	>500	Afrika	Maesopsis eminii Engl.	Rhamnaceae	0,43	14,0	10	11	0,04	0,02	58,49	0,06	0,69	2,52
6552	121	3	Sukamantri	>500	Afrika	Maesopsis eminii Engl.	Rhamnaceae	0,43	5,1	4	5	0,04	0,00	4,45	0,00	0,05	0,19
6553	121	3	Sukamantri	>500	Afrika	Maesopsis eminii Engl.	Rhamnaceae	0,43	8,9	10	11	0,04	0,01	25,56	0,03	0,30	1,10
6554	121	3	Sukamantri	>500	Afrika	Maesopsis eminii Engl.	Rhamnaceae	0,43	8,0	7	8	0,04	0,00	15,51	0,02	0,18	0,67
6555	121	3	Sukamantri	>500	Afrika	Maesopsis eminii Engl.	Rhamnaceae	0,43	11,5	8	10	0,04	0,01	37,11	0,04	0,44	1,60
6556	121	3	Sukamantri	>500	Tisuk	Hibiscus macrophyllus Roxb. ex I	Malvaceae	0,4	5,1	4	5	0,04	0,00	4,17	0,00	0,05	0,18
6557	121	3	Sukamantri	>500	Afrika	Maesopsis eminii Engl.	Rhamnaceae	0,43	15,6	12	13	0,04	0,02	83,02	0,08	0,98	3,58
6558	121	3	Sukamantri	>500	Afrika	Maesopsis eminii Engl.	Rhamnaceae	0,43	15,9	9	10	0,04	0,02	67,75	0,07	0,80	2,92
6559	121	3	Sukamantri	>500	Afrika	Maesopsis eminii Engl.	Rhamnaceae	0,43	13,4	9	10	0,04	0,01	49,22	0,05	0,58	2,12
6560	121	3	Sukamantri	>500	Afrika	Maesopsis eminii Engl.	Rhamnaceae	0,43	7,6	8	9	0,04	0,00	16,03	0,02	0,19	0,69
6561	121	3	Sukamantri	>500	Afrika	Maesopsis eminii Engl.	Rhamnaceae	0,43	15,0	10	11	0,04	0,02	66,00	0,07	0,78	2,85
6562	121	3	Sukamantri	>500	Afrika	Maesopsis eminii Engl.	Rhamnaceae	0,43	10,8	9	10	0,04	0,01	33,42	0,03	0,39	1,44
6563	121	3	Sukamantri	>500	Tisuk	Hibiscus macrophyllus Roxb. ex I	Malvaceae	0,4	4,1	4	6	0,04	0,00	3,37	0,00	0,04	0,15
6564	121	3	Sukamantri	>500	Afrika	Maesopsis eminii Engl.	Rhamnaceae	0,43	10,8	10	11	0,04	0,01	36,47	0,04	0,43	1,57
6565	121	3	Sukamantri	>500	Afrika	Maesopsis eminii Engl.	Rhamnaceae	0,43	14,3	10	11	0,04	0,02	60,95	0,06	0,72	2,63
6566	121	3	Sukamantri	>500	Afrika	Maesopsis eminii Engl.	Rhamnaceae	0,43	14,3	10	11	0,04	0,02	60,95	0,06	0,72	2,63

6567	121	3	Sukamantri	>500	Afrika	Maesopsis eminii Engl.	Rhamnaceae		0,43	12,4	9	10	0,04	0,01	42,97	0,04	0,50	1,85
6568	121	3	Sukamantri	>500	Afrika	Maesopsis eminii Engl.	Rhamnaceae		0,43	10,8	10	11	0,04	0,01	36,47	0,04	0,43	1,57
6569	121	3	Sukamantri	>500	Afrika	Maesopsis eminii Engl.	Rhamnaceae		0,43	5,4	6	8	0,04	0,00	7,65	0,01	0,09	0,33
6570	121	3	Sukamantri	>500	Afrika	Maesopsis eminii Engl.	Rhamnaceae		0,43	11,8	9	10	0,04	0,01	39,02	0,04	0,46	1,68
6571	121	3	Sukamantri	>500	Afrika	Maesopsis eminii Engl.	Rhamnaceae		0,43	3,2	3	4	0,04	0,00	1,53	0,00	0,02	0,07
6572	121	3	Sukamantri	>500	Afrika	Maesopsis eminii Engl.	Rhamnaceae		0,43	6,7	7	8	0,04	0,00	11,27	0,01	0,13	0,49
6573	121	3	Sukamantri	>500	Afrika	Maesopsis eminii Engl.	Rhamnaceae		0,43	17,5	10	11	0,04	0,02	88,03	0,09	1,03	3,80
6574	121	3	Sukamantri	>500	Tisuk	Hibiscus macrophyllus Roxb. ex I	Malvaceae		0,4	6,4	5	6,5	0,04	0,00	7,97	0,01	0,09	0,34
6575	121	3	Sukamantri	>500	Afrika	Maesopsis eminii Engl.	Rhamnaceae		0,43	16,9	10	11	0,04	0,02	82,26	0,08	0,97	3,55
6576	121	3	Sukamantri	>500	Afrika	Maesopsis eminii Engl.	Rhamnaceae		0,43	14,3	10	11	0,04	0,02	60,95	0,06	0,72	2,63
6577	121	3	Sukamantri	>500	Afrika	Maesopsis eminii Engl.	Rhamnaceae		0,43	15,3	10	12	0,04	0,02	74,29	0,07	0,87	3,20
6578	121	3	Sukamantri	>500	Pinus	Pinus merkusii Jungh. & de Vries	Pinaceae		0,55	6,4	2	6	0,04	0,00	9,92	0,01	0,12	0,43
6579	121	3	Sukamantri	>500	Pinus	Pinus merkusii Jungh. & de Vries	Pinaceae		0,55	10,2	2	7	0,04	0,01	27,03	0,03	0,32	1,17
6580	121	3	Sukamantri	>500	Afrika	Maesopsis eminii Engl.	Rhamnaceae		0,43	20,4	10	12	0,04	0,03	125,84	0,13	1,48	5,43
6581	121	3	Sukamantri	>500	Afrika	Maesopsis eminii Engl.	Rhamnaceae		0,43	18,1	10	11	0,04	0,03	93,98	0,09	1,10	4,05
6582	121	3	Sukamantri	>500	Pinus	Pinus merkusii Jungh. & de Vries	Pinaceae		0,55	5,1	2	4	0,04	0,00	4,55	0,00	0,05	0,20
6583	121	3	Sukamantri	>500	Pinus	Pinus merkusii Jungh. & de Vries	Pinaceae		0,55	8,9	2	6	0,04	0,01	18,38	0,02	0,22	0,79
6584	121	3	Sukamantri	>500	Pinus	Pinus merkusii Jungh. & de Vries	Pinaceae		0,55	9,9	2,5	7	0,04	0,01	25,50	0,03	0,30	1,10
6585	121	3	Sukamantri	>500	Afrika	Maesopsis eminii Engl.	Rhamnaceae		0,43	19,4	11	12	0,04	0,03	115,25	0,12	1,35	4,97
6586	121	3	Sukamantri	>500	Pinus	Pinus merkusii Jungh. & de Vries	Pinaceae		0,55	10,5	2	7	0,04	0,01	28,60	0,03	0,34	1,23
6587	121	3	Sukamantri	>500	Afrika	Maesopsis eminii Engl.	Rhamnaceae		0,43	19,4	10	12	0,04	0,03	115,25	0,12	1,35	4,97
6588	121	3	Sukamantri	>500	Afrika	Maesopsis eminii Engl.	Rhamnaceae		0,43	15,0	10	12	0,04	0,02	71,48	0,07	0,84	3,08
6589	121	3	Sukamantri	>500	Pinus	Pinus merkusii Jungh. & de Vries	Pinaceae		0,55	8,6	3	7	0,04	0,01	19,80	0,02	0,23	0,85
6590	121	3	Sukamantri	>500	Afrika	Maesopsis eminii Engl.	Rhamnaceae		0,43	8,9	9	11	0,04	0,01	25,56	0,03	0,30	1,10
6591	121	3	Sukamantri	>500	Afrika	Maesopsis eminii Engl.	Rhamnaceae		0,43	17,5	10	11,5	0,04	0,02	91,69	0,09	1,08	3,95
6592	121	3	Sukamantri	>500	Afrika	Maesopsis eminii Engl.	Rhamnaceae		0,43	16,5	10	11,5	0,04	0,02	82,74	0,08	0,97	3,57
6593	121	3	Sukamantri	>500	Afrika	Maesopsis eminii Engl.	Rhamnaceae		0,43	6,7	7	8	0,04	0,00	11,27	0,01	0,13	0,49
6594	121	3	Sukamantri	>500	Pinus	Pinus merkusii Jungh. & de Vries	Pinaceae		0,55	7,3	2	6	0,04	0,00	12,82	0,01	0,15	0,55
6595	121	3	Sukamantri	>500	Jabon	Neolamarckia cadamba (Roxb.) I	Rubiaceae		0,42	9,5	8	10	0,04	0,01	26,01	0,03	0,31	1,12
6596	121	3	Sukamantri	>500	Pinus	Pinus merkusii Jungh. & de Vries	Pinaceae		0,55	6,0	2	5	0,04	0,00	7,64	0,01	0,09	0,33
6597	121	3	Sukamantri	>500	Afrika	Maesopsis eminii Engl.	Rhamnaceae		0,43	10,8	9	10	0,04	0,01	33,42	0,03	0,39	1,44
6598	121	3	Sukamantri	>500	Pinus	Pinus merkusii Jungh. & de Vries	Pinaceae		0,55	5,1	2	6	0,04	0,00	6,59	0,01	0,08	0,28
6599	121	3	Sukamantri	>500	Sengon	Paraserianthes falcataria (L.) I.C.	Leguminosae / Fabaceae		0,33	6,4	6	8	0,04	0,00	8,09	0,01	0,10	0,35
6600	122	2	Sukamantri	>500	Afrika	Maesopsis eminii Engl.	Rhamnaceae		0,43	14,6	5	7	0,04	0,02	41,94	0,04	0,49	1,81
6601	122	2	Sukamantri	>500	Afrika	Maesopsis eminii Engl.	Rhamnaceae		0,43	10,2	6	7	0,04	0,01	21,57	0,02	0,25	0,93
6602	122	2	Sukamantri	>500	Puspa	Schima wallichii Choisy	Theaceae		0,74	8,0	3	5	0,04	0,00	16,58	0,02	0,19	0,72
6603	122	2	Sukamantri	>500	Puspa	Schima wallichii Choisy	Theaceae		0,74	11,1	3	7	0,04	0,01	41,80	0,04	0,49	1,80
6604	122	2	Sukamantri	>500	Afrika	Maesopsis eminii Engl.	Rhamnaceae		0,43	11,8	6	7	0,04	0,01	28,15	0,03	0,33	1,21
6605	122	2	Sukamantri	>500	Puspa	Schima wallichii Choisy	Theaceae		0,74	9,9	3	5	0,04	0,01	24,59	0,02	0,29	1,06
6606	122	2	Sukamantri	>500	Puspa	Schima wallichii Choisy	Theaceae		0,74	7,3	3	6	0,04	0,00	16,82	0,02	0,20	0,73
6607	122	2	Sukamantri	>500	Afrika	Maesopsis eminii Engl.	Rhamnaceae		0,43	8,0	4	6	0,04	0,00	11,92	0,01	0,14	0,51
6608	122	2	Sukamantri	>500	Afrika	Maesopsis eminii Engl.	Rhamnaceae		0,43	7,0	7	8	0,04	0,00	12,27	0,01	0,14	0,53
6609	122	2	Sukamantri	>500	Afrika	Maesopsis eminii Engl.	Rhamnaceae		0,43	15,0	8	9	0,04	0,02	54,92	0,05	0,65	2,37
6610	122	2	Sukamantri	>500	Mahoni	Swietenia macrophylla King	Meliaceae		0,61	19,1	4	8	0,04	0,03	106,24	0,11	1,25	4,58
6611	122	2	Sukamantri	>500	Afrika	Maesopsis eminii Engl.	Rhamnaceae		0,43	7,6	6	7	0,04	0,00	12,74	0,01	0,15	0,55
6612	122	2	Sukamantri	>500	Renggang	Sp4		0	0,5	3,8	2	3	0,04	0,00	1,89	0,00	0,02	0,08
6613	122	2	Sukamantri	>500	Puspa	Schima wallichii Choisy	Theaceae		0,74	5,7	3	4	0,04	0,00	7,40	0,01	0,09	0,32
6614	122	2	Sukamantri	>500	Puspa	Schima wallichii Choisy	Theaceae		0,74	4,1	1	4	0,04	0,00	4,08	0,00	0,05	0,18
6615	122	2	Sukamantri	>500	Puspa	Schima wallichii Choisy	Theaceae		0,74	15,9	7	12	0,04	0,02	131,64	0,13	1,55	5,68
6616	122	2	Sukamantri	>500	Puspa	Schima wallichii Choisy	Theaceae		0,74	13,0	7	12	0,04	0,01	91,51	0,09	1,08	3,95
6617	122	2	Sukamantri	>500	Puspa	Schima wallichii Choisy	Theaceae		0,74	9,5	5	7	0,04	0,01	31,52	0,03	0,37	1,36
6618	122	2	Sukamantri	>500	Gmelina	Gmelina arborea Roxb.	Lamiaceae / Verbenaceae		0,47	4,5	2	4	0,04	0,00	3,08	0,00	0,04	0,13
6619	122	2	Sukamantri	>500	Gmelina	Gmelina arborea Roxb.	Lamiaceae / Verbenaceae		0,47	3,2	2	3	0,04	0,00	1,28	0,00	0,02	0,06
6620	122	2	Sukamantri	>500	Gmelina	Gmelina arborea Roxb.	Lamiaceae / Verbenaceae		0,47	4,1	2	3	0,04	0,00	2,07	0,00	0,02	0,09
6621	122	2	Sukamantri	>500	Puspa	Schima wallichii Choisy	Theaceae		0,74	7,6	4	7	0,04	0,00	20,94	0,02	0,25	0,90
6622	122	2	Sukamantri	>500	Tisuk	Hibiscus macrophyllus Roxb. ex I	Malvaceae		0,4	3,2	3	4	0,04	0,00	1,44	0,00	0,02	0,06
6623	122	2	Sukamantri	>500	Puspa	Schima wallichii Choisy	Theaceae		0,74	5,7	3	5	0,04	0,00	9,08	0,01	0,11	0,39
6624	122	2	Sukamantri	>500	Afrika	Maesopsis eminii Engl.	Rhamnaceae		0,43	18,5	9	10	0,04	0,03	88,92	0,09	1,04	3,83
6625	122	2	Sukamantri	>500	Puspa	Schima wallichii Choisy	Theaceae		0,74	12,7	3	7	0,04	0,01	53,38	0,05	0,63	2,30
6626	122	2	Sukamantri	>500	Puspa	Schima wallichii Choisy	Theaceae		0,74	5,1	3	6	0,04	0,00	8,65	0,01	0,10	0,37
6627	122	2	Sukamantri	>500	Puspa	Schima wallichii Choisy	Theaceae		0,74	6,4	3	7	0,04	0,00	14,99	0,01	0,18	0,65
6628	122	2	Sukamantri	>500	Puspa	Schima wallichii Choisy	Theaceae		0,74	16,2	6	9	0,04	0,02	104,88	0,10	1,23	4,52
6629	122	2	Sukamantri	>500	Afrika	Maesopsis eminii Engl.	Rhamnaceae		0,43	6,0	5	6	0,04	0,00	7,21	0,01	0,08	0,31
6630	122	2	Sukamantri	>500	Sengon	Paraserianthes falcataria (L.) I.C.	Leguminosae / Fabaceae		0,33	9,5	9	10	0,04	0,01	20,85	0,02	0,25	0,90
6631	122	2	Sukamantri	>500	Tisuk	Hibiscus macrophyllus Roxb. ex I	Malvaceae		0,4	9,5	10	11	0,04	0,01	27,14	0,03	0,32	1,17
6632	122	2	Sukamantri	>500	Mahoni	Swietenia macrophylla King	Meliaceae		0,61	19,1	6	10	0,04	0,03	130,33	0,13	1,53	5,62
6633	122	2	Sukamantri	>500	Afrika	Maesopsis eminii Engl.	Rhamnaceae		0,43	9,9	9	10	0,04	0,01	28,22	0,03	0,33	1,22
6634	122	2	Sukamantri	>500	Puspa	Schima wallichii Choisy	Theaceae		0,74	10,5	3	8	0,04	0,01	42,41	0,04	0,50	1,83
6635	122	2	Sukamantri	>500	Gmelina	Gmelina arborea Roxb.	Lamiaceae / Verbenaceae		0,47	3,8	3	4	0,04	0,00	2,32	0,00	0,03	0,10
6636	122	2	Sukamantri	>500	Puspa	Schima wallichii Choisy	Theaceae		0,74	4,8	3	5	0,04	0,00	6,50	0,01	0,08	0,28
6637	122	2	Sukamantri	>500	Puspa	Schima wallichii Choisy	Theaceae		0,74	4,1	2,5	4	0,04	0,00	4,08	0,00	0,05	0,18
6638	122	2	Sukamantri	>500	Afrika	Maesopsis eminii Engl.	Rhamnaceae		0,43	13,4	8	10	0,04	0,01	49,22	0,05	0,58	2,12
6639	122	2	Sukamantri	>500	Afrika	Maesopsis eminii Engl.	Rhamnaceae		0,43	9,5	6	7	0,04	0,01	19,17	0,02	0,23	0,83
6640	122	2	Sukamantri	>500	Puspa	Schima wallichii Choisy	Theaceae		0,74	5,7	3	5	0,04	0,00	9,08	0,01	0,11	0,39
6641	122	2	Sukamantri	>500	Afrika	Maesopsis eminii Engl.	Rhamnaceae		0,43	7,6	7	8	0,04	0,00	14,39	0,01	0,17	0,62
6642	122	2	Sukamantri	>500	Afrika	Maesopsis eminii Engl.	Rhamnaceae		0,43	5,7	6	7	0,04	0,00	7,52	0,01	0,09	0,32
6643	122	2	Sukamantri	>500	Sengon	Paraserianthes falcataria (L.) I.C.	Leguminosae / Fabaceae		0,33	4,5	5	6	0,04	0,00	3,23	0,00	0,04	0,14
6644	122	2	Sukamantri	>500	Afrika	Maesopsis eminii Engl.	Rhamnaceae		0,43	8,0	6	7	0,04	0,00	13,72	0,01	0,16	0,59
6645	122	2	Sukamantri	>500	Afrika	Maesopsis eminii Engl.	Rhamnaceae		0,43	5,4	5	6	0,04	0,00	5,88	0,01	0,07	0,25
6646	122	2	Sukamantri	>500	Afrika	Maesopsis eminii Engl.	Rhamnaceae		0,43	11,1	10	11	0,04	0,01	38,46	0,04	0,45	1,66
6647	122	2	Sukamantri	>500	Mahoni	Swietenia macrophylla King	Meliaceae		0,61	13,4	4	8	0,04	0,01	55,27	0,06	0,65	2,38
6648	122	2	Sukamantri	>500	Gmelina	Gmelina arborea Roxb.	Lamiaceae / Verbenaceae		0,47	3,2	2	3	0,04	0,00	1,28	0,00	0,02	0,06
6649	122	2	Sukamantri	>500	Afrika	Maesopsis eminii Engl.	Rhamnaceae		0,43	8,9	8	9	0,04	0,01	21,26	0,02	0,25	0,92
6650	122	2	Sukamantri	>500	Gmelina	Gmelina arborea Roxb.	Lamiaceae / Verbenaceae		0,47	3,5	2	3	0,04	0,00	1,52	0,00	0,02	0,07
6651	122	2	Sukamantri	>500	Alpukat	Persea americana Mill.	Lauraceae		0,54	11,1	6	7	0,04	0,01	31,32	0,03	0,37	1,35
6652	122	2	Sukamantri	>500	Afrika	Maesopsis eminii Engl.	Rhamnaceae		0,43	6,4	6	7	0,04	0,00	9,12	0,01	0,11	0,39
6653	122	2	Sukamantri	>500	Mahoni	Swietenia macrophylla King	Meliaceae		0,61	20,7	6	9	0,04	0,03	137,03	0,14	1,61	5,91
6654	122	2	Sukamantri	>500	Tisuk	Hibiscus macrophyllus Roxb. ex I	Malvaceae		0,4	19,1	10	13	0,04	0,03	112,60	0,11	1,32	4,86
6655	122	2	Sukamantri	>500	Alpukat	Persea americana Mill.	Lauraceae		0,54	10,2	5	7	0,04	0,01	26,58	0,03	0,31	1,15
6656	122	2	Sukamantri	>500	Afrika	Maesopsis eminii Engl.	Rhamnaceae		0,43	22,3	12	14	0,04	0,04	170,78	0,17	2,01	7,36
6657	122	2	Sukamantri	>500	Gmelina	Gmelina arborea Roxb.	Lamiaceae / Verbenaceae		0,47	3,8	2,5	3,5	0,04	0,00	2,06	0,00	0,02	0,09
6658	122	2	Sukamantri	>500	Gmelina	Gmelina arborea Roxb.	Lamiaceae / Verbenaceae		0,47	4,8	3	5	0,04	0,00	4,29	0,00	0,05	0,19
6659	122	2	Sukamantri	>500	Afrika	Maesopsis eminii Engl.	Rhamnaceae		0,43	9,2	9	10	0,04	0,01	24,97	0,02	0,29	1,08
6660	122	2	Sukamantri	>500	Tisuk	Hibiscus macrophyllus Roxb. ex I	Malvaceae		0,4	27,4	11	15	0,04	0,06	248,26	0,25	2,92	10,71
6661	122	2	Sukamantri	>500	Tisuk	Hibiscus macrophyllus Roxb. ex I	Malvaceae		0,4	18,5	10	14	0,04	0,03	113,26	0,11	1,33	4,88
6662	123	2	Sukamantri	>500	Mahoni	Swietenia macrophylla King	Meliaceae		0,61	7,0	5	7	0,02	0,00	14,96	0,01	0,35	1,29
6663	123	2	Sukamantri	>500	Mahoni	Swietenia macrophylla King	Meliaceae		0,61	6,4	5	7	0,02	0,00	12,56	0,01	0,30	1,08
6664	123	2	Sukamantri	>500	Afrika	Maesopsis eminii Engl.	Rhamnaceae		0,43	17,2	12	13	0,02	0,01	99,20	0,10	2,33	8,56
6665	123	2	Sukamantri	>500	Huru keling	Litsea sp.	Lauraceae		0,425	11,5	11	12	0,02	0,01	43,39	0,04	1,02	3,74
6666	123	2	Sukamantri	>500	Mahoni	Swietenia macrophylla King	Meliaceae		0,61	7,0	5	7	0,02	0,00	14,96	0,01	0,35	1,29
6667	123	2	Sukamantri	>500	Afrika	Maesopsis eminii Engl.	Rhamnaceae		0,43	19,1	6	8	0,02	0,01	77,12	0,08	1,81	6,65
6668	123	2	Sukamantri	>500	Afrika	Maesopsis eminii Engl.	Rhamnaceae		0,43	21,3	12	13	0,02	0,02	147,27	0,15	3,46	12,70
6669	123	2	Sukamantri	>500	Afrika	Maesopsis eminii Engl.	Rhamnaceae		0,43	21,0	12	13	0,02	0,02	143,27	0,14	3,37	12,36
6670	123	2	Sukamantri	>500	Afrika	Maesopsis eminii Engl.	Rhamnaceae		0,43	23,2	13	14	0,02	0,02	184,43	0,18	4,33	15,91
6671	123	2	Sukamantri	>500	Mahoni	Swietenia macrophylla King	Meliaceae		0,61	6,4	4	6	0,02	0,00	10,91	0,01	0,26	0,94
6672	123	2	Sukamantri	>500	Afrika	Maesopsis eminii Engl.	Rhamnaceae		0,43	20,0	13	14	0,02	0,02	140,81	0,14	3,31	12,14
6673	123	2	Sukamantri	>500	Afrika	Maesopsis eminii Engl.	Rhamnaceae		0,43	6,7	8	9	0,02	0,00	12,55	0,01	0,30	1,08
6674	123	2	Sukamantri	>500	Ganitri	Elaeocarpus ganitrus Roxb. ex G.	Elaeocarpaceae		0,49	5,7	8	9	0,02	0,00	10,67	0,01	0,25	0,92
6675	123	2	Sukamantri	>500	Ganitri	Elaeocarpus ganitrus Roxb. ex G.	Elaeocarpaceae		0,49	6,7	8	9	0,02	0,00	14,15	0,01	0,33	1,22
6676	123	2	Sukamantri	>500	Afrika	Maesopsis eminii Engl.	Rhamnaceae		0,43	8,3	9	10	0,02	0,00	20,45	0,02	0,48	1,76
6677	123	2	Sukamantri	>500	Afrika	Maesopsis eminii Engl.	Rhamnaceae		0,43	12,7	12	13	0,02	0,01	57,24	0,06	1,35	4,94
6678	123	2	Sukamantri	>500	Afrika	Maesopsis eminii Engl.	Rhamnaceae		0,43	23,2	14	16	0,02	0,02	208,43	0,21	4,90	17,98
6679	123	2	Sukamantri	>500	Afrika	Maesopsis eminii Engl.	Rhamnaceae		0,43	7,0	10	11	0,02	0,00	16,43	0,02	0,39	1,42
6680	123	2	Sukamantri	>500	Afrika	Maesopsis eminii Engl.	Rhamnaceae		0,43	5,4	6	7	0,02	0,00	6,77	0,01	0,16	0,58
6681	123	2	Sukamantri	>500	Huru keling	Litsea sp.	Lauraceae		0,425	6,0	2	6	0,02	0,00	7,13	0,01	0,17	0,62
6682	123	2	Sukamantri	>500	Afrika	Maesopsis eminii Engl.	Rhamnaceae		0,43	15,0	13	14	0,02	0,01	82,32	0,08	1,93	7,10
6683	123	2	Sukamantri	>500	Afrika	Maesopsis eminii Engl.	Rhamnaceae		0,43	8,9	10	11	0,02	0,00	25,56	0,03	0,60	2,20
6684	123	2	Sukamantri	>500	Mahoni	Swietenia macrophylla King	Meliaceae		0,61	8,9	4	6	0,02	0,00	20,21	0,02	0,47	1,74
6685	123	2	Sukamantri	>500	Afrika	Maesopsis eminii Engl.	Rhamnaceae		0,43	3,8	5	6	0,02	0,00	3,11	0,00	0,07	0,27
6686	123	2	Sukamantri	>500	Ganitri	Elaeocarpus ganitrus Roxb. ex G.	Elaeocarpaceae		0,49	10,8	10	11	0,02	0,00	41,11	0,04	0,97	3,55
6687	123	2	Sukamantri	>500	Ganitri	Elaeocarpus ganitrus Roxb. ex G.	Elaeocarpaceae		0,49	9,2	10	11	0,02	0,00	30,72	0,03	0,72	2,65
6688	123	2	Sukamantri	>500	Afrika	Maesopsis eminii Engl.	Rhamnaceae		0,43	22,0	12	13	0,02	0,02	155,42	0,16	3,65	13,40
6689	123	2	Sukamantri	>500	Afrika	Maesopsis eminii Engl.	Rhamnaceae		0,43	9,5	9	10	0,02	0,00	26,57	0,03	0,62	2,29
6690	123	2	Sukamantri	>500	Afrika	Maesopsis eminii Engl.	Rhamnaceae		0,43	4,8	4	6	0,02	0,00	4,67	0,00	0,11	0,40

6691	123	2	Sukamantri	>500	Afrika	Maesopsis eminii Engl.	Rhamnaceae	0,43	8,6	10	11	0,02	0,00	23,91	0,02	0,56	2,06
6692	123	2	Sukamantri	>500	Afrika	Maesopsis eminii Engl.	Rhamnaceae	0,43	4,1	6	7	0,02	0,00	4,14	0,00	0,10	0,36
6693	123	2	Sukamantri	>500	Afrika	Maesopsis eminii Engl.	Rhamnaceae	0,43	3,5	3	4	0,02	0,00	1,83	0,00	0,04	0,16
6694	123	2	Sukamantri	>500	Afrika	Maesopsis eminii Engl.	Rhamnaceae	0,43	4,1	3	4	0,02	0,00	2,48	0,00	0,06	0,21
6695	123	2	Sukamantri	>500	Afrika	Maesopsis eminii Engl.	Rhamnaceae	0,43	6,4	7	8	0,02	0,00	10,31	0,01	0,24	0,89
6696	123	2	Sukamantri	>500	Afrika	Maesopsis eminii Engl.	Rhamnaceae	0,43	3,5	6	7	0,02	0,00	3,05	0,00	0,07	0,26
6697	123	2	Sukamantri	>500	Afrika	Maesopsis eminii Engl.	Rhamnaceae	0,43	6,0	7	8	0,02	0,00	9,38	0,01	0,22	0,81
6698	123	2	Sukamantri	>500	Afrika	Maesopsis eminii Engl.	Rhamnaceae	0,43	6,0	7	8	0,02	0,00	9,38	0,01	0,22	0,81
6699	123	2	Sukamantri	>500	Afrika	Maesopsis eminii Engl.	Rhamnaceae	0,43	5,7	7	8	0,02	0,00	8,50	0,01	0,20	0,73
6700	123	2	Sukamantri	>500	Afrika	Maesopsis eminii Engl.	Rhamnaceae	0,43	19,4	12	13	0,02	0,01	124,01	0,12	2,91	10,70
6701	123	2	Sukamantri	>500	Afrika	Maesopsis eminii Engl.	Rhamnaceae	0,43	5,7	7	8	0,02	0,00	8,50	0,01	0,20	0,73
6702	123	2	Sukamantri	>500	Afrika	Maesopsis eminii Engl.	Rhamnaceae	0,43	6,7	8	9	0,02	0,00	12,55	0,01	0,30	1,08
6703	123	2	Sukamantri	>500	Afrika	Maesopsis eminii Engl.	Rhamnaceae	0,43	4,8	8	9	0,02	0,00	6,78	0,01	0,16	0,58
6704	123	2	Sukamantri	>500	Afrika	Maesopsis eminii Engl.	Rhamnaceae	0,43	17,8	10	11	0,02	0,01	90,99	0,09	2,14	7,85
6705	123	2	Sukamantri	>500	Afrika	Maesopsis eminii Engl.	Rhamnaceae	0,43	18,5	10	11	0,02	0,01	97,03	0,10	2,28	8,37
6706	123	2	Sukamantri	>500	Afrika	Maesopsis eminii Engl.	Rhamnaceae	0,43	6,7	8	9	0,02	0,00	12,55	0,01	0,30	1,08
6707	123	2	Sukamantri	>500	Afrika	Maesopsis eminii Engl.	Rhamnaceae	0,43	7,3	9	10	0,02	0,00	16,33	0,02	0,38	1,41
6708	123	2	Sukamantri	>500	Afrika	Maesopsis eminii Engl.	Rhamnaceae	0,43	4,5	6	7	0,02	0,00	4,74	0,00	0,11	0,41
6709	123	2	Sukamantri	>500	Afrika	Maesopsis eminii Engl.	Rhamnaceae	0,43	11,5	10	11	0,02	0,01	40,50	0,04	0,95	3,49
6710	123	2	Sukamantri	>500	Afrika	Maesopsis eminii Engl.	Rhamnaceae	0,43	5,1	5	6	0,02	0,00	5,26	0,01	0,12	0,45
6711	123	2	Sukamantri	>500	Afrika	Maesopsis eminii Engl.	Rhamnaceae	0,43	9,9	9	10	0,02	0,00	28,22	0,03	0,66	2,43
6712	123	2	Sukamantri	>500	Afrika	Maesopsis eminii Engl.	Rhamnaceae	0,43	6,4	6	7	0,02	0,00	9,12	0,01	0,21	0,79
6713	123	2	Sukamantri	>500	Afrika	Maesopsis eminii Engl.	Rhamnaceae	0,43	22,6	11	12	0,02	0,02	152,20	0,15	3,58	13,13
6714	123	2	Sukamantri	>500	Afrika	Maesopsis eminii Engl.	Rhamnaceae	0,43	20,7	13	14	0,02	0,02	149,10	0,15	3,50	12,86
6715	123	2	Sukamantri	>500	Ganitri	Elaeocarpus ganitrus Roxb. ex G.	Elaeocarpaceae	0,49	19,4	12	13	0,02	0,01	139,78	0,14	3,28	12,06
6716	123	2	Sukamantri	>500	Afrika	Maesopsis eminii Engl.	Rhamnaceae	0,43	23,9	12	14	0,02	0,02	193,79	0,19	4,55	16,71
6717	123	2	Sukamantri	>500	Sengon	Paraserianthes falcataria (L.) I.C.	Leguminosae / Fabaceae	0,33	22,9	11	14	0,02	0,02	141,11	0,14	3,32	12,17
6718	123	2	Sukamantri	>500	Afrika	Maesopsis eminii Engl.	Rhamnaceae	0,43	22,6	10	11	0,02	0,02	140,54	0,14	3,30	12,12
6719	123	2	Sukamantri	>500	Ganitri	Elaeocarpus ganitrus Roxb. ex G.	Elaeocarpaceae	0,49	20,0	10	11	0,02	0,02	127,25	0,13	2,99	10,97
6720	123	2	Sukamantri	>500	Suren	Toona sureni (Blume) Merr.	Meliaceae	0,39	11,5	8	10	0,02	0,01	33,94	0,03	0,80	2,93
6721	123	2	Sukamantri	>500	Suren	Toona sureni (Blume) Merr.	Meliaceae	0,39	10,5	8	9	0,02	0,00	26,27	0,03	0,62	2,27
6722	123	2	Sukamantri	>500	Afrika	Maesopsis eminii Engl.	Rhamnaceae	0,43	10,5	9	10	0,02	0,00	31,64	0,03	0,74	2,73
6723	123	2	Sukamantri	>500	Afrika	Maesopsis eminii Engl.	Rhamnaceae	0,43	16,9	10	11	0,02	0,01	82,26	0,08	1,93	7,09
6724	123	2	Sukamantri	>500	Afrika	Maesopsis eminii Engl.	Rhamnaceae	0,43	10,2	10	11	0,02	0,00	32,64	0,03	0,77	2,81
6725	123	2	Sukamantri	>500	Afrika	Maesopsis eminii Engl.	Rhamnaceae	0,43	10,2	10	11	0,02	0,00	32,64	0,03	0,77	2,81
6726	123	2	Sukamantri	>500	Afrika	Maesopsis eminii Engl.	Rhamnaceae	0,43	17,8	13	14	0,02	0,01	113,48	0,11	2,67	9,79
6727	123	2	Sukamantri	>500	Afrika	Maesopsis eminii Engl.	Rhamnaceae	0,43	15,9	11	12	0,02	0,01	80,06	0,08	1,88	6,90
6728	123	2	Sukamantri	>500	Afrika	Maesopsis eminii Engl.	Rhamnaceae	0,43	12,7	11	12	0,02	0,01	53,20	0,05	1,25	4,59
6729	123	2	Sukamantri	>500	Ganitri	Elaeocarpus ganitrus Roxb. ex G.	Elaeocarpaceae	0,49	13,0	10	11	0,02	0,01	57,93	0,06	1,36	5,00
6730	123	2	Sukamantri	>500	Afrika	Maesopsis eminii Engl.	Rhamnaceae	0,43	18,8	12	13	0,02	0,01	116,67	0,12	2,74	10,06
6731	123	2	Sukamantri	>500	Afrika	Maesopsis eminii Engl.	Rhamnaceae	0,43	14,6	10	11	0,02	0,01	63,45	0,06	1,49	5,47
6732	123	2	Sukamantri	>500	Afrika	Maesopsis eminii Engl.	Rhamnaceae	0,43	12,7	10	11	0,02	0,01	49,12	0,05	1,15	4,24
6733	123	2	Sukamantri	>500	Afrika	Maesopsis eminii Engl.	Rhamnaceae	0,43	13,0	12	13	0,02	0,01	59,89	0,06	1,41	5,17
6734	124	1	Sukamantri	>500	Ganitri	Elaeocarpus ganitrus Roxb. ex G.	Elaeocarpaceae	0,49	3,8	3	4	0,01	0,00	2,41	0,00	0,11	0,42
6735	124	1	Sukamantri	>500	Ganitri	Elaeocarpus ganitrus Roxb. ex G.	Elaeocarpaceae	0,49	3,5	2	3	0,01	0,00	1,58	0,00	0,07	0,27
6736	124	1	Sukamantri	>500	Ganitri	Elaeocarpus ganitrus Roxb. ex G.	Elaeocarpaceae	0,49	4,1	3	4	0,01	0,00	2,80	0,00	0,13	0,48
6737	124	1	Sukamantri	>500	Ganitri	Elaeocarpus ganitrus Roxb. ex G.	Elaeocarpaceae	0,49	3,2	2,5	3,5	0,01	0,00	1,53	0,00	0,07	0,26
6738	124	1	Sukamantri	>500	Ganitri	Elaeocarpus ganitrus Roxb. ex G.	Elaeocarpaceae	0,49	4,8	2	4	0,01	0,00	3,63	0,00	0,17	0,63
6739	124	1	Sukamantri	>500	Afrika	Maesopsis eminii Engl.	Rhamnaceae	0,43	8,9	7	8	0,01	0,00	19,09	0,02	0,90	3,29
6740	124	1	Sukamantri	>500	Afrika	Maesopsis eminii Engl.	Rhamnaceae	0,43	5,4	5	6	0,01	0,00	5,88	0,01	0,28	1,01
6741	124	1	Sukamantri	>500	Afrika	Maesopsis eminii Engl.	Rhamnaceae	0,43	5,7	6	7	0,01	0,00	7,52	0,01	0,35	1,30
6742	124	1	Sukamantri	>500	Afrika	Maesopsis eminii Engl.	Rhamnaceae	0,43	5,4	5	6	0,01	0,00	5,88	0,01	0,28	1,01
6743	124	1	Sukamantri	>500	Afrika	Maesopsis eminii Engl.	Rhamnaceae	0,43	7,3	6	7	0,01	0,00	11,78	0,01	0,55	2,03
6744	124	1	Sukamantri	>500	Afrika	Maesopsis eminii Engl.	Rhamnaceae	0,43	12,7	9	10	0,01	0,00	45,01	0,05	2,12	7,76
6745	124	1	Sukamantri	>500	Afrika	Maesopsis eminii Engl.	Rhamnaceae	0,43	4,5	6	7	0,01	0,00	4,74	0,00	0,22	0,82
6746	124	1	Sukamantri	>500	Ganitri	Elaeocarpus ganitrus Roxb. ex G.	Elaeocarpaceae	0,49	5,1	2	3	0,01	0,00	3,14	0,00	0,15	0,54
6747	124	1	Sukamantri	>500	Ganitri	Elaeocarpus ganitrus Roxb. ex G.	Elaeocarpaceae	0,49	4,8	3	5	0,01	0,00	4,46	0,00	0,21	0,77
6748	124	1	Sukamantri	>500	Ganitri	Elaeocarpus ganitrus Roxb. ex G.	Elaeocarpaceae	0,49	4,5	3	5,5	0,01	0,00	4,29	0,00	0,20	0,74
6749	124	1	Sukamantri	>500	Ganitri	Elaeocarpus ganitrus Roxb. ex G.	Elaeocarpaceae	0,49	5,7	4	6	0,01	0,00	7,36	0,01	0,35	1,27
6750	124	1	Sukamantri	>500	Ganitri	Elaeocarpus ganitrus Roxb. ex G.	Elaeocarpaceae	0,49	4,5	3	5	0,01	0,00	3,93	0,00	0,18	0,68
6751	124	1	Sukamantri	>500	Afrika	Maesopsis eminii Engl.	Rhamnaceae	0,43	11,5	9	10	0,01	0,00	37,11	0,04	1,74	6,40
6752	124	1	Sukamantri	>500	Afrika	Maesopsis eminii Engl.	Rhamnaceae	0,43	7,3	7	8	0,01	0,00	13,31	0,01	0,63	2,30
6753	124	1	Sukamantri	>500	Afrika	Maesopsis eminii Engl.	Rhamnaceae	0,43	6,7	5	6	0,01	0,00	8,66	0,01	0,41	1,49
6754	124	1	Sukamantri	>500	Afrika	Maesopsis eminii Engl.	Rhamnaceae	0,43	5,1	4	5	0,01	0,00	4,45	0,00	0,21	0,77
6755	124	1	Sukamantri	>500	Afrika	Maesopsis eminii Engl.	Rhamnaceae	0,43	11,1	9	10	0,01	0,00	35,25	0,04	1,66	6,08
6756	124	1	Sukamantri	>500	Afrika	Maesopsis eminii Engl.	Rhamnaceae	0,43	11,8	9,5	10	0,01	0,00	39,02	0,04	1,83	6,73
6757	124	1	Sukamantri	>500	Afrika	Maesopsis eminii Engl.	Rhamnaceae	0,43	4,8	5	6	0,01	0,00	4,67	0,00	0,22	0,81
6758	124	1	Sukamantri	>500	Afrika	Maesopsis eminii Engl.	Rhamnaceae	0,43	5,4	6	7	0,01	0,00	6,77	0,01	0,32	1,17
6759	124	1	Sukamantri	>500	Afrika	Maesopsis eminii Engl.	Rhamnaceae	0,43	9,5	7	8	0,01	0,00	21,66	0,02	1,02	3,74
6760	124	1	Sukamantri	>500	Afrika	Maesopsis eminii Engl.	Rhamnaceae	0,43	6,0	6	7	0,01	0,00	8,30	0,01	0,39	1,43
6761	124	1	Sukamantri	>500	Ganitri	Elaeocarpus ganitrus Roxb. ex G.	Elaeocarpaceae	0,49	4,1	2	4	0,01	0,00	2,80	0,00	0,13	0,48
6762	124	1	Sukamantri	>500	Ganitri	Elaeocarpus ganitrus Roxb. ex G.	Elaeocarpaceae	0,49	4,1	2	4,5	0,01	0,00	3,11	0,00	0,15	0,54
6763	124	1	Sukamantri	>500	Ganitri	Elaeocarpus ganitrus Roxb. ex G.	Elaeocarpaceae	0,49	4,8	5	4,5	0,01	0,00	4,05	0,00	0,19	0,70
6764	124	1	Sukamantri	>500	Ganitri	Elaeocarpus ganitrus Roxb. ex G.	Elaeocarpaceae	0,49	4,5	3	4	0,01	0,00	3,20	0,00	0,15	0,55
6765	124	1	Sukamantri	>500	Ganitri	Elaeocarpus ganitrus Roxb. ex G.	Elaeocarpaceae	0,49	3,8	3	4	0,01	0,00	2,41	0,00	0,11	0,42
6766	124	1	Sukamantri	>500	Ganitri	Elaeocarpus ganitrus Roxb. ex G.	Elaeocarpaceae	0,49	7,0	4	5,5	0,01	0,00	9,81	0,01	0,46	1,69
6767	124	1	Sukamantri	>500	Ganitri	Elaeocarpus ganitrus Roxb. ex G.	Elaeocarpaceae	0,49	5,4	7	5	0,01	0,00	5,61	0,01	0,26	0,97
6768	124	1	Sukamantri	>500	Afrika	Maesopsis eminii Engl.	Rhamnaceae	0,43	8,9	7	8	0,01	0,00	19,09	0,02	0,90	3,29
6769	124	1	Sukamantri	>500	Afrika	Maesopsis eminii Engl.	Rhamnaceae	0,43	7,6	5	6	0,01	0,00	11,06	0,01	0,52	1,91
6770	124	1	Sukamantri	>500	Afrika	Maesopsis eminii Engl.	Rhamnaceae	0,43	9,9	7	8	0,01	0,00	23,00	0,02	1,08	3,97
6771	124	1	Sukamantri	>500	Afrika	Maesopsis eminii Engl.	Rhamnaceae	0,43	13,7	8	9	0,01	0,00	46,66	0,05	2,19	8,05
6772	124	1	Sukamantri	>500	Afrika	Maesopsis eminii Engl.	Rhamnaceae	0,43	10,5	8	9	0,01	0,00	28,73	0,03	1,35	4,96
6773	124	1	Sukamantri	>500	Afrika	Maesopsis eminii Engl.	Rhamnaceae	0,43	4,5	4	5	0,01	0,00	3,49	0,00	0,16	0,60
6774	124	1	Sukamantri	>500	Afrika	Maesopsis eminii Engl.	Rhamnaceae	0,43	5,4	5	6	0,01	0,00	5,88	0,01	0,28	1,01
6775	124	1	Sukamantri	>500	Afrika	Maesopsis eminii Engl.	Rhamnaceae	0,43	6,4	6	7	0,01	0,00	9,12	0,01	0,43	1,57
6776	124	1	Sukamantri	>500	Ganitri	Elaeocarpus ganitrus Roxb. ex G.	Elaeocarpaceae	0,49	4,8	5	3	0,01	0,00	2,79	0,00	0,13	0,48
6777	124	1	Sukamantri	>500	Ganitri	Elaeocarpus ganitrus Roxb. ex G.	Elaeocarpaceae	0,49	3,5	2,5	3,5	0,01	0,00	1,82	0,00	0,09	0,31
6778	124	1	Sukamantri	>500	Ganitri	Elaeocarpus ganitrus Roxb. ex G.	Elaeocarpaceae	0,49	3,5	3	4	0,01	0,00	2,06	0,00	0,10	0,36
6779	124	1	Sukamantri	>500	Ganitri	Elaeocarpus ganitrus Roxb. ex G.	Elaeocarpaceae	0,49	5,4	4	5,5	0,01	0,00	6,12	0,01	0,29	1,06
6780	124	1	Sukamantri	>500	Ganitri	Elaeocarpus ganitrus Roxb. ex G.	Elaeocarpaceae	0,49	5,7	4	5,5	0,01	0,00	6,79	0,01	0,32	1,17
6781	125	3	Sukamantri	>500	Lamerang	Ficus fulva Reinw. ex Blume	Moraceae	0,39	18,5	7	10	0,04	0,03	81,31	0,08	0,96	3,51
6782	125	3	Sukamantri	>500	Afrika	Maesopsis eminii Engl.	Rhamnaceae	0,43	3,2	2,5	3,5	0,04	0,00	1,36	0,00	0,02	0,06
6783	125	3	Sukamantri	>500	Afrika	Maesopsis eminii Engl.	Rhamnaceae	0,43	10,2	6	7	0,04	0,01	21,57	0,02	0,25	0,93
6784	125	3	Sukamantri	>500	Afrika	Maesopsis eminii Engl.	Rhamnaceae	0,43	6,4	5	6	0,04	0,00	7,92	0,01	0,09	0,34
6785	125	3	Sukamantri	>500	Ganitri	Elaeocarpus ganitrus Roxb. ex G.	Elaeocarpaceae	0,49	7,6	2	4	0,04	0,00	8,60	0,01	0,10	0,37
6786	125	3	Sukamantri	>500	Sengon	Paraserianthes falcataria (L.) I.C.	Leguminosae / Fabaceae	0,33	3,2	2	3	0,04	0,00	0,92	0,00	0,01	0,04
6787	125	3	Sukamantri	>500	Afrika	Maesopsis eminii Engl.	Rhamnaceae	0,43	4,1	2	3	0,04	0,00	1,91	0,00	0,02	0,08
6788	125	3	Sukamantri	>500	Sengon	Paraserianthes falcataria (L.) I.C.	Leguminosae / Fabaceae	0,33	15,6	8	12	0,04	0,02	60,54	0,06	0,71	2,61
6789	125	3	Sukamantri	>500	Ganitri	Elaeocarpus ganitrus Roxb. ex G.	Elaeocarpaceae	0,49	6,4	2,5	4	0,04	0,00	6,16	0,01	0,07	0,27
6790	125	3	Sukamantri	>500	Ganitri	Elaeocarpus ganitrus Roxb. ex G.	Elaeocarpaceae	0,49	4,8	3	4	0,04	0,00	3,63	0,00	0,04	0,16
6791	125	3	Sukamantri	>500	Ganitri	Elaeocarpus ganitrus Roxb. ex G.	Elaeocarpaceae	0,49	2,9	2,5	3	0,04	0,00	1,10	0,00	0,01	0,05
6792	125	3	Sukamantri	>500	Afrika	Maesopsis eminii Engl.	Rhamnaceae	0,43	2,9	2	2,5	0,04	0,00	0,82	0,00	0,01	0,04
6793	125	3	Sukamantri	>500	Ganitri	Elaeocarpus ganitrus Roxb. ex G.	Elaeocarpaceae	0,49	4,5	3	4	0,04	0,00	3,20	0,00	0,04	0,14
6794	125	3	Sukamantri	>500	Afrika	Maesopsis eminii Engl.	Rhamnaceae	0,43	22,3	8	10	0,04	0,04	125,49	0,13	1,47	5,41
6795	125	3	Sukamantri	>500	Afrika	Maesopsis eminii Engl.	Rhamnaceae	0,43	12,4	7	8	0,04	0,01	35,03	0,04	0,41	1,51
6796	125	3	Sukamantri	>500	Afrika	Maesopsis eminii Engl.	Rhamnaceae	0,43	17,2	7	8	0,04	0,02	63,58	0,06	0,75	2,74
6797	125	3	Sukamantri	>500	Ganitri	Elaeocarpus ganitrus Roxb. ex G.	Elaeocarpaceae	0,49	8,6	2	5	0,04	0,01	13,09	0,01	0,15	0,56
6798	125	3	Sukamantri	>500	Ganitri	Elaeocarpus ganitrus Roxb. ex G.	Elaeocarpaceae	0,49	6,7	2	4	0,04	0,00	6,73	0,01	0,08	0,29
6799	125	3	Sukamantri	>500	Ganitri	Elaeocarpus ganitrus Roxb. ex G.	Elaeocarpaceae	0,49	9,2	3	6	0,04	0,01	17,63	0,02	0,21	0,76
6800	125	3	Sukamantri	>500	Ganitri	Elaeocarpus ganitrus Roxb. ex G.	Elaeocarpaceae	0,49	7,6	4	6	0,04	0,00	12,46	0,01	0,15	0,54
6801	125	3	Sukamantri	>500	Afrika	Maesopsis eminii Engl.	Rhamnaceae	0,43	19,7	8	10	0,04	0,03	100,47	0,10	1,18	4,33
6802	125	3	Sukamantri	>500	Sengon	Paraserianthes falcataria (L.) I.C.	Leguminosae / Fabaceae	0,33	3,2	4	4,6	0,04	0,00	1,37	0,00	0,02	0,06
6803	125	3	Sukamantri	>500	Sengon	Paraserianthes falcataria (L.) I.C.	Leguminosae / Fabaceae	0,33	5,7	4	6	0,04	0,00	5,12	0,01	0,06	0,22
6804	125	3	Sukamantri	>500	Sengon	Paraserianthes falcataria (L.) I.C.	Leguminosae / Fabaceae	0,33	3,2	3	4	0,04	0,00	1,20	0,00	0,01	0,05
6805	125	3	Sukamantri	>500	Kiteja	Daphiniphylum glaucescens Blu	Daphniphyllaceae	0,5	15,6	3	5	0,04	0,02	39,73	0,04	0,47	1,71
6806	125	3	Sukamantri	>500	Huru Kacang	Neolitsea triplinervia Merr.	Lauraceae	0,56	5,7	3	4	0,04	0,00	5,74	0,01	0,07	0,25
6807	125	3	Sukamantri	>500	Afrika	Maesopsis eminii Engl.	Rhamnaceae	0,43	16,5	8	10	0,04	0,02	72,79	0,07	0,86	3,14
6808	125	3	Sukamantri	>500	Sengon	Paraserianthes falcataria (L.) I.C.	Leguminosae / Fabaceae	0,33	6,4	6	7	0,04	0,00	7,16	0,01	0,08	0,31
6809	125	3	Sukamantri	>500	Afrika	Maesopsis eminii Engl.	Rhamnaceae	0,43	20,7	12	13	0,04	0,03	139,32	0,14	1,64	6,01
6810	125	3	Sukamantri	>500	Ganitri	Elaeocarpus ganitrus Roxb. ex G.	Elaeocarpaceae	0,49	4,8	2,5	3,5	0,04	0,00	3,22	0,00	0,04	0,14
6811	125	3	Sukamantri	>500	Ganitri	Elaeocarpus ganitrus Roxb. ex G.	Elaeocarpaceae	0,49	5,7	2	4	0,04	0,00	5,08	0,01	0,06	0,22
6812	125	3	Sukamantri	>500	Waru	Hibiscus tilliaceus L.	Malvaceae	0,49	13,4	3	6	0,04	0,01	34,75	0,03	0,41	1,50
6813	125	3	Sukamantri	>500	Mangga	Mangifera indica L.	Anacardiaceae	0,6	6,0	2	3	0,04	0,00	5,18	0,01	0,06	0,22
6814	125	3	Sukamantri	>500	Sengon	Paraserianthes falcataria (L.) I.C.	Leguminosae / Fabaceae	0,33	9,5	3	7	0,04	0,01	15,04	0,02	0,18	0,65

6815	125	3	Sukamantri	>500	Mareme	Glochidion borneense (Müll. Arg.	Phyllanthaceae		0,46	6,7	3	4	0,04	0,00	6,35	0,01	0,07	0,27
6816	125	3	Sukamantri	>500	Afrika	Maesopsis eminii Engl.	Rhamnaceae		0,43	17,8	10	11	0,04	0,02	90,99	0,09	1,07	3,92
6817	125	3	Sukamantri	>500	Huru Kacang	Neolitsea triplinervia Merr.	Lauraceae		0,56	8,3	3	5	0,04	0,01	13,80	0,01	0,16	0,60
6818	125	3	Sukamantri	>500	Afrika	Maesopsis eminii Engl.	Rhamnaceae		0,43	19,4	7	11	0,04	0,03	106,42	0,11	1,25	4,59
6819	125	3	Sukamantri	>500	Afrika	Maesopsis eminii Engl.	Rhamnaceae		0,43	21,6	10	13	0,04	0,04	151,32	0,15	1,78	6,53
6820	125	3	Sukamantri	>500	Tisuk	Hibiscus macrophyllus Roxb. ex I	Malvaceae		0,4	3,2	2	3	0,04	0,00	1,10	0,00	0,01	0,05
6821	125	3	Sukamantri	>500	Alpukat	Persea americana Mill.	Lauraceae		0,54	6,0	3	6	0,04	0,00	8,88	0,01	0,10	0,38
6822	125	3	Sukamantri	>500	Alpukat	Persea americana Mill.	Lauraceae		0,54	7,6	3	6	0,04	0,00	13,62	0,01	0,16	0,59
6823	125	3	Sukamantri	>500	Puspa	Schima wallichii Choisy	Theaceae		0,74	5,4	2	4	0,04	0,00	6,67	0,01	0,08	0,29
6824	125	3	Sukamantri	>500	Puspa	Schima wallichii Choisy	Theaceae		0,74	5,1	2	4	0,04	0,00	5,97	0,01	0,07	0,26
6825	125	3	Sukamantri	>500	Sengon	Paraserianthes falcataria (L.) I.C.	Leguminosae / Fabaceae		0,33	12,4	5	7	0,04	0,01	24,32	0,02	0,29	1,05
6826	126	2	Sukamantri	>500	Afrika	Maesopsis eminii Engl.	Rhamnaceae		0,43	15,6	13	14	0,04	0,02	88,85	0,09	1,04	3,83
6827	126	2	Sukamantri	>500	Puspa	Schima wallichii Choisy	Theaceae		0,74	15,6	6	12	0,04	0,02	126,85	0,13	1,49	5,47
6828	126	2	Sukamantri	>500	Puspa	Schima wallichii Choisy	Theaceae		0,74	8,0	5	9	0,04	0,00	28,41	0,03	0,33	1,23
6829	126	2	Sukamantri	>500	Afrika	Maesopsis eminii Engl.	Rhamnaceae		0,43	17,2	13	14	0,04	0,02	106,16	0,11	1,25	4,58
6830	126	2	Sukamantri	>500	Puspa	Schima wallichii Choisy	Theaceae		0,74	15,9	4	10	0,04	0,02	111,39	0,11	1,31	4,80
6831	126	2	Sukamantri	>500	Puspa	Schima wallichii Choisy	Theaceae		0,74	5,1	3	5	0,04	0,00	7,32	0,01	0,09	0,32
6832	126	2	Sukamantri	>500	Puspa	Schima wallichii Choisy	Theaceae		0,74	4,5	3	6	0,04	0,00	6,77	0,01	0,08	0,29
6833	126	2	Sukamantri	>500	Afrika	Maesopsis eminii Engl.	Rhamnaceae		0,43	7,3	6	7	0,04	0,00	11,78	0,01	0,14	0,51
6834	126	2	Sukamantri	>500	Puspa	Schima wallichii Choisy	Theaceae		0,74	4,5	2	4	0,04	0,00	4,67	0,00	0,05	0,20
6835	126	2	Sukamantri	>500	Puspa	Schima wallichii Choisy	Theaceae		0,74	19,1	7	14	0,04	0,03	211,72	0,21	2,49	9,13
6836	126	2	Sukamantri	>500	Limus	Mangifera foetida Lour.	Anacardiaceae		0,73	3,2	1,5	3	0,04	0,00	1,91	0,00	0,02	0,08
6837	126	2	Sukamantri	>500	Puspa	Schima wallichii Choisy	Theaceae		0,74	14,3	8	10	0,04	0,02	91,84	0,09	1,08	3,96
6838	126	2	Sukamantri	>500	Afrika	Maesopsis eminii Engl.	Rhamnaceae		0,43	22,0	12	13	0,04	0,04	155,42	0,16	1,83	6,70
6839	126	2	Sukamantri	>500	Tisuk	Hibiscus macrophyllus Roxb. ex I	Malvaceae		0,4	18,1	10	12	0,04	0,03	95,26	0,10	1,12	4,11
6840	126	2	Sukamantri	>500	Puspa	Schima wallichii Choisy	Theaceae		0,74	12,1	6	9	0,04	0,01	61,18	0,06	0,72	2,64
6841	126	2	Sukamantri	>500	Puspa	Schima wallichii Choisy	Theaceae		0,74	14,6	7	10	0,04	0,02	95,61	0,10	1,12	4,12
6842	126	2	Sukamantri	>500	Puspa	Schima wallichii Choisy	Theaceae		0,74	15,9	6	12	0,04	0,02	131,64	0,13	1,55	5,68
6843	126	2	Sukamantri	>500	Puspa	Schima wallichii Choisy	Theaceae		0,74	11,5	5	11	0,04	0,01	66,59	0,07	0,78	2,87
6844	126	2	Sukamantri	>500	Puspa	Schima wallichii Choisy	Theaceae		0,74	5,4	3	5	0,04	0,00	8,18	0,01	0,10	0,35
6845	126	2	Sukamantri	>500	Afrika	Maesopsis eminii Engl.	Rhamnaceae		0,43	6,4	7	8	0,04	0,00	10,31	0,01	0,12	0,44
6846	126	2	Sukamantri	>500	Afrika	Maesopsis eminii Engl.	Rhamnaceae		0,43	5,7	7	7,5	0,04	0,00	8,01	0,01	0,09	0,35
6847	126	2	Sukamantri	>500	Puspa	Schima wallichii Choisy	Theaceae		0,74	3,2	1,8	4	0,04	0,00	2,52	0,00	0,03	0,11
6848	126	2	Sukamantri	>500	Cengkeh	Syzygium aromaticum (L.) Merr. a	Myrtaceae		0,79	3,2	1	3	0,04	0,00	2,06	0,00	0,02	0,09
6849	126	2	Sukamantri	>500	Afrika	Maesopsis eminii Engl.	Rhamnaceae		0,43	20,4	12	13	0,04	0,03	135,42	0,14	1,59	5,84
6850	126	2	Sukamantri	>500	Nangka	Artocarpus heterophyllus Lam.	Moraceae		0,61	8,9	3	5	0,04	0,01	17,10	0,02	0,20	0,74
6851	126	2	Sukamantri	>500	Afrika	Maesopsis eminii Engl.	Rhamnaceae		0,43	22,3	10	11	0,04	0,04	136,93	0,14	1,61	5,90
6852	126	2	Sukamantri	>500	Puspa	Schima wallichii Choisy	Theaceae		0,74	9,5	3	7	0,04	0,01	31,52	0,03	0,37	1,36
6853	126	2	Sukamantri	>500	Tisuk	Hibiscus macrophyllus Roxb. ex I	Malvaceae		0,4	23,2	12	16	0,04	0,04	195,07	0,20	2,29	8,41
6854	126	2	Sukamantri	>500	Afrika	Maesopsis eminii Engl.	Rhamnaceae		0,43	15,0	7	8	0,04	0,02	49,30	0,05	0,58	2,13
6855	126	2	Sukamantri	>500	Waru	Hibiscus tilliaceus L.	Malvaceae		0,49	7,6	5	7	0,04	0,00	14,35	0,01	0,17	0,62
6856	126	2	Sukamantri	>500	Afrika	Maesopsis eminii Engl.	Rhamnaceae		0,43	13,4	11	12	0,04	0,01	58,17	0,06	0,68	2,51
6857	126	2	Sukamantri	>500	Afrika	Maesopsis eminii Engl.	Rhamnaceae		0,43	9,5	7	8	0,04	0,01	21,66	0,02	0,25	0,93
6858	126	2	Sukamantri	>500	Tisuk	Hibiscus macrophyllus Roxb. ex I	Malvaceae		0,4	20,7	9	13	0,04	0,03	130,39	0,13	1,53	5,62
6859	126	2	Sukamantri	>500	Afrika	Maesopsis eminii Engl.	Rhamnaceae		0,43	10,8	3	6	0,04	0,01	20,93	0,02	0,25	0,90
6860	126	2	Sukamantri	>500	Afrika	Maesopsis eminii Engl.	Rhamnaceae		0,43	11,5	13	15	0,04	0,01	53,80	0,05	0,63	2,32
6861	126	2	Sukamantri	>500	Alpukat	Persea americana Mill.	Lauraceae		0,54	17,2	7	11	0,04	0,02	104,87	0,10	1,23	4,52
6862	126	2	Sukamantri	>500	Afrika	Maesopsis eminii Engl.	Rhamnaceae		0,43	31,8	12	16	0,04	0,08	370,98	0,37	4,36	16,00
6863	126	2	Sukamantri	>500	Puspa	Schima wallichii Choisy	Theaceae		0,74	20,0	6	10	0,04	0,03	170,11	0,17	2,00	7,34
6864	126	2	Sukamantri	>500	Afrika	Maesopsis eminii Engl.	Rhamnaceae		0,43	22,3	14	15	0,04	0,04	181,93	0,18	2,14	7,85
6865	126	2	Sukamantri	>500	Afrika	Maesopsis eminii Engl.	Rhamnaceae		0,43	8,3	7	8	0,04	0,01	16,67	0,02	0,20	0,72
6866	126	2	Sukamantri	>500	Manglid	Magnolia champaca (L.) Baill. ex	Magnoliaceae		0,53	6,4	2	5	0,04	0,00	8,12	0,01	0,10	0,35
6867	126	2	Sukamantri	>500	Waru	Hibiscus tilliaceus L.	Malvaceae		0,49	12,1	5	9	0,04	0,01	41,94	0,04	0,49	1,81
6868	126	2	Sukamantri	>500	Puspa	Schima wallichii Choisy	Theaceae		0,74	11,1	5	8	0,04	0,01	47,24	0,05	0,56	2,04
6869	126	2	Sukamantri	>500	Puspa	Schima wallichii Choisy	Theaceae		0,74	13,7	7	10	0,04	0,01	84,50	0,08	0,99	3,64
6870	126	2	Sukamantri	>500	Puspa	Schima wallichii Choisy	Theaceae		0,74	17,2	7	10	0,04	0,02	128,26	0,13	1,51	5,53
6871	126	2	Sukamantri	>500	Puspa	Schima wallichii Choisy	Theaceae		0,74	11,5	3	9	0,04	0,01	55,41	0,06	0,65	2,39
6872	126	2	Sukamantri	>500	Puspa	Schima wallichii Choisy	Theaceae		0,74	22,9	7	14	0,04	0,04	295,68	0,30	3,47	12,75
6873	126	2	Sukamantri	>500	Puspa	Schima wallichii Choisy	Theaceae		0,74	16,2	11	14	0,04	0,02	157,20	0,16	1,85	6,78
6874	126	2	Sukamantri	>500	Afrika	Maesopsis eminii Engl.	Rhamnaceae		0,43	7,0	7	8	0,04	0,00	12,27	0,01	0,14	0,53
6875	126	2	Sukamantri	>500	Afrika	Maesopsis eminii Engl.	Rhamnaceae		0,43	19,1	12	13	0,04	0,03	120,32	0,12	1,41	5,19
6876	126	2	Sukamantri	>500	Afrika	Maesopsis eminii Engl.	Rhamnaceae		0,43	15,0	12	13	0,04	0,02	76,92	0,08	0,90	3,32
6877	126	2	Sukamantri	>500	Puspa	Schima wallichii Choisy	Theaceae		0,74	19,1	6	14	0,04	0,03	211,72	0,21	2,49	9,13
6878	126	2	Sukamantri	>500	Afrika	Maesopsis eminii Engl.	Rhamnaceae		0,43	17,8	12	13	0,04	0,02	106,03	0,11	1,25	4,57
6879	126	2	Sukamantri	>500	Afrika	Maesopsis eminii Engl.	Rhamnaceae		0,43	5,7	6	7	0,04	0,00	7,52	0,01	0,09	0,32
6880	126	2	Sukamantri	>500	Afrika	Maesopsis eminii Engl.	Rhamnaceae		0,43	5,1	5	6	0,04	0,00	5,26	0,01	0,06	0,23
6881	126	2	Sukamantri	>500	Puspa	Schima wallichii Choisy	Theaceae		0,74	19,7	7	13	0,04	0,03	210,07	0,21	2,47	9,06
6882	126	2	Sukamantri	>500	Tisuk	Hibiscus macrophyllus Roxb. ex I	Malvaceae		0,4	11,8	6	11	0,04	0,01	39,85	0,04	0,47	1,72
6883	126	2	Sukamantri	>500	Puspa	Schima wallichii Choisy	Theaceae		0,74	18,8	9	12	0,04	0,03	178,27	0,18	2,09	7,69
6884	126	2	Sukamantri	>500	Afrika	Maesopsis eminii Engl.	Rhamnaceae		0,43	16,5	12	13	0,04	0,02	92,57	0,09	1,09	3,99
6885	126	2	Sukamantri	>500	Mareme	Glochidion borneense (Müll. Arg.	Phyllanthaceae		0,46	8,6	6	9	0,04	0,01	21,16	0,02	0,25	0,91
6886	126	2	Sukamantri	>500	Puspa	Schima wallichii Choisy	Theaceae		0,74	16,2	3	10	0,04	0,02	115,51	0,12	1,36	4,98
6887	126	2	Sukamantri	>500	Puspa	Schima wallichii Choisy	Theaceae		0,74	15,0	3	10	0,04	0,02	99,45	0,10	1,17	4,29
6888	126	2	Sukamantri	>500	Afrika	Maesopsis eminii Engl.	Rhamnaceae		0,43	25,8	14	16	0,04	0,05	252,17	0,25	2,96	10,87
6889	126	2	Sukamantri	>500	Afrika	Maesopsis eminii Engl.	Rhamnaceae		0,43	5,4	6	7	0,04	0,00	6,77	0,01	0,08	0,29
6890	126	2	Sukamantri	>500	Afrika	Maesopsis eminii Engl.	Rhamnaceae		0,43	27,4	14	16	0,04	0,06	281,42	0,28	3,31	12,14
6891	126	2	Sukamantri	>500	Afrika	Maesopsis eminii Engl.	Rhamnaceae		0,43	25,5	14	16	0,04	0,05	246,50	0,25	2,90	10,63
6892	126	2	Sukamantri	>500	Afrika	Maesopsis eminii Engl.	Rhamnaceae		0,43	15,0	10	11	0,04	0,02	66,00	0,07	0,78	2,85
6893	126	2	Sukamantri	>500	Afrika	Maesopsis eminii Engl.	Rhamnaceae		0,43	30,2	12	14	0,04	0,07	298,83	0,30	3,51	12,89
6894	126	2	Sukamantri	>500	Puspa	Schima wallichii Choisy	Theaceae		0,74	18,5	4	9	0,04	0,03	132,75	0,13	1,56	5,72
6895	126	2	Sukamantri	>500	Nangka	Artocarpus heterophyllus Lam.	Moraceae		0,61	11,1	3	6	0,04	0,01	30,41	0,03	0,36	1,31
6896	126	2	Sukamantri	>500	Afrika	Maesopsis eminii Engl.	Rhamnaceae		0,43	27,0	13	15	0,04	0,06	259,64	0,26	3,05	11,20
6897	126	2	Sukamantri	>500	Afrika	Maesopsis eminii Engl.	Rhamnaceae		0,43	25,5	13	15	0,04	0,05	232,35	0,23	2,73	10,02
6898	126	2	Sukamantri	>500	Afrika	Maesopsis eminii Engl.	Rhamnaceae		0,43	25,5	13	15	0,04	0,05	232,35	0,23	2,73	10,02
6899	126	2	Sukamantri	>500	Puspa	Schima wallichii Choisy	Theaceae		0,74	19,1	30	10	0,04	0,03	155,56	0,16	1,83	6,71
6900	126	2	Sukamantri	>500	Afrika	Maesopsis eminii Engl.	Rhamnaceae		0,43	11,1	7	8	0,04	0,01	28,73	0,03	0,34	1,24
6901	126	2	Sukamantri	>500	Afrika	Maesopsis eminii Engl.	Rhamnaceae		0,43	14,3	7	8	0,04	0,02	45,53	0,05	0,53	1,96
6902	126	2	Sukamantri	>500	Puspa	Schima wallichii Choisy	Theaceae		0,74	18,1	7	9	0,04	0,03	128,58	0,13	1,51	5,54
6903	126	2	Sukamantri	>500	Petai	Parkia speciosa Hassk.	Leguminosae / Fabaceae		0,45	44,9	6	12	0,04	0,16	557,65	0,56	6,55	24,05
6904	127	2	Sukamantri	>500	Tisuk	Hibiscus macrophyllus Roxb. ex I	Malvaceae		0,4	17,2	12	14	0,04	0,02	99,36	0,10	1,17	4,28
6905	127	2	Sukamantri	>500	Afrika	Maesopsis eminii Engl.	Rhamnaceae		0,43	8,6	10	11	0,04	0,01	23,91	0,02	0,28	1,03
6906	127	2	Sukamantri	>500	Kareumbi	Homalanthus populneus (Giesel	Euphorbiaceae		0,38	6,4	4	7	0,04	0,00	8,14	0,01	0,10	0,35
6907	127	2	Sukamantri	>500	Afrika	Maesopsis eminii Engl.	Rhamnaceae		0,43	15,0	12	13	0,04	0,02	76,92	0,08	0,90	3,32
6908	127	2	Sukamantri	>500	Puspa	Schima wallichii Choisy	Theaceae		0,74	6,4	4	6	0,04	0,00	13,02	0,01	0,15	0,56
6909	127	2	Sukamantri	>500	Puspa	Schima wallichii Choisy	Theaceae		0,74	8,3	6	7	0,04	0,01	24,25	0,02	0,28	1,05
6910	127	2	Sukamantri	>500	Puspa	Schima wallichii Choisy	Theaceae		0,74	7,3	4	6	0,04	0,00	16,82	0,02	0,20	0,73
6911	127	2	Sukamantri	>500	Waru	Hibiscus tilliaceus L.	Malvaceae		0,49	3,2	1,5	3	0,04	0,00	1,33	0,00	0,02	0,06
6912	127	2	Sukamantri	>500	Puspa	Schima wallichii Choisy	Theaceae		0,74	14,0	4	10	0,04	0,02	88,13	0,09	1,04	3,80
6913	127	2	Sukamantri	>500	Puspa	Schima wallichii Choisy	Theaceae		0,74	8,3	7	8,5	0,04	0,01	28,97	0,03	0,34	1,25
6914	127	2	Sukamantri	>500	Puspa	Schima wallichii Choisy	Theaceae		0,74	13,7	12	14	0,04	0,01	115,00	0,12	1,35	4,96
6915	127	2	Sukamantri	>500	Puspa	Schima wallichii Choisy	Theaceae		0,74	19,7	7	14	0,04	0,03	224,83	0,22	2,64	9,70
6916	127	2	Sukamantri	>500	Puspa	Schima wallichii Choisy	Theaceae		0,74	4,8	4	5	0,04	0,00	6,50	0,01	0,08	0,28
6917	127	2	Sukamantri	>500	Puspa	Schima wallichii Choisy	Theaceae		0,74	8,0	5	7	0,04	0,00	22,57	0,02	0,27	0,97
6918	127	2	Sukamantri	>500	Nangka	Artocarpus heterophyllus Lam.	Moraceae		0,61	5,4	3	8	0,04	0,00	10,54	0,01	0,12	0,45
6919	127	2	Sukamantri	>500	Puspa	Schima wallichii Choisy	Theaceae		0,74	15,9	4	11	0,04	0,02	121,55	0,12	1,43	5,24
6920	127	2	Sukamantri	>500	Puspa	Schima wallichii Choisy	Theaceae		0,74	10,8	6	10	0,04	0,01	54,95	0,05	0,65	2,37
6921	127	2	Sukamantri	>500	Puspa	Schima wallichii Choisy	Theaceae		0,74	16,2	7	9	0,04	0,02	104,88	0,10	1,23	4,52
6922	127	2	Sukamantri	>500	Puspa	Schima wallichii Choisy	Theaceae		0,74	3,2	2	4	0,04	0,00	2,52	0,00	0,03	0,11
6923	127	2	Sukamantri	>500	Puspa	Schima wallichii Choisy	Theaceae		0,74	10,5	6	9	0,04	0,01	47,24	0,05	0,56	2,04
6924	127	2	Sukamantri	>500	Puspa	Schima wallichii Choisy	Theaceae		0,74	10,8	4	8	0,04	0,01	44,80	0,04	0,53	1,93
6925	127	2	Sukamantri	>500	Mahoni	Swietenia macrophylla King	Meliaceae		0,61	38,8	10	17	0,04	0,12	777,65	0,78	9,14	33,53
6926	127	2	Sukamantri	>500	Puspa	Schima wallichii Choisy	Theaceae		0,74	13,4	3	11	0,04	0,01	88,32	0,09	1,04	3,81
6927	127	2	Sukamantri	>500	Waru	Hibiscus tilliaceus L.	Malvaceae		0,49	3,8	4	5	0,04	0,00	2,96	0,00	0,03	0,13
6928	127	2	Sukamantri	>500	Afrika	Maesopsis eminii Engl.	Rhamnaceae		0,43	15,9	13	14	0,04	0,02	92,20	0,09	1,08	3,98
6929	127	2	Sukamantri	>500	Puspa	Schima wallichii Choisy	Theaceae		0,74	15,9	7	14	0,04	0,02	151,60	0,15	1,78	6,54
6930	127	2	Sukamantri	>500	Waru	Hibiscus tilliaceus L.	Malvaceae		0,49	5,4	3	6	0,04	0,00	6,63	0,01	0,08	0,29
6931	127	2	Sukamantri	>500	Puspa	Schima wallichii Choisy	Theaceae		0,74	7,3	4	8	0,04	0,00	21,89	0,02	0,26	0,94
6932	127	2	Sukamantri	>500	Renggang	Sp4		0	0,5	4,8	2	3,5	0,04	0,00	3,28	0,00	0,04	0,14
6933	127	2	Sukamantri	>500	Puspa	Schima wallichii Choisy	Theaceae		0,74	7,3	3,5	5,5	0,04	0,00	15,53	0,02	0,18	0,67
6934	127	2	Sukamantri	>500	Puspa	Schima wallichii Choisy	Theaceae		0,74	17,2	4	13	0,04	0,02	163,10	0,16	1,92	7,03
6935	127	2	Sukamantri	>500	Puspa	Schima wallichii Choisy	Theaceae		0,74	15,9	8	12	0,04	0,02	131,64	0,13	1,55	5,68
6936	127	2	Sukamantri	>500	Waru	Hibiscus tilliaceus L.	Malvaceae		0,49	3,2	3	4	0,04	0,00	1,73	0,00	0,02	0,07
6937	127	2	Sukamantri	>500	Tisuk	Hibiscus macrophyllus Roxb. ex I	Malvaceae		0,4	26,7	11	14	0,04	0,06	223,22	0,22	2,62	9,63
6938	127	2	Sukamantri	>500	Mahoni	Swietenia macrophylla King	Meliaceae		0,61	43,6	8	15	0,04	0,15	857,54	0,86	10,08	36,98

6939	127	2	Sukamantri	>500	Nangka	Artocarpus heterophyllus Lam.	Moraceae		0,61	3,2	2	3,5	0,04	0,00	1,87	0,00	0,02	0,08
6940	127	2	Sukamantri	>500	Puspa	Schima wallichii Choisy	Theaceae		0,74	5,7	4	6	0,04	0,00	10,73	0,01	0,13	0,46
6941	127	2	Sukamantri	>500	Puspa	Schima wallichii Choisy	Theaceae		0,74	3,2	2	3	0,04	0,00	1,94	0,00	0,02	0,08
6942	127	2	Sukamantri	>500	Renggang	Sp4		0	0,5	3,2	2	3,5	0,04	0,00	1,56	0,00	0,02	0,07
6943	127	2	Sukamantri	>500	Nangka	Artocarpus heterophyllus Lam.	Moraceae		0,61	4,1	2	4	0,04	0,00	3,42	0,00	0,04	0,15
6944	127	2	Sukamantri	>500	Puspa	Schima wallichii Choisy	Theaceae		0,74	10,8	6	10	0,04	0,01	54,95	0,05	0,65	2,37
6945	127	2	Sukamantri	>500	Renggang	Sp4		0	0,5	3,2	2	3	0,04	0,00	1,35	0,00	0,02	0,06
6946	127	2	Sukamantri	>500	Nangka	Artocarpus heterophyllus Lam.	Moraceae		0,61	2,9	2	3	0,04	0,00	1,34	0,00	0,02	0,06
6947	127	2	Sukamantri	>500	Mahoni	Swietenia macrophylla King	Meliaceae		0,61	44,9	6	14	0,04	0,16	848,60	0,85	9,97	36,59
6948	127	2	Sukamantri	>500	Mahoni	Swietenia macrophylla King	Meliaceae		0,61	44,9	8	16	0,04	0,16	959,01	0,96	11,27	41,36
6949	127	2	Sukamantri	>500	Mahoni	Swietenia macrophylla King	Meliaceae		0,61	27,0	9	14	0,04	0,06	335,76	0,34	3,95	14,48
6950	127	2	Sukamantri	>500	Puspa	Schima wallichii Choisy	Theaceae		0,74	13,7	4	10	0,04	0,01	84,50	0,08	0,99	3,64
6951	127	2	Sukamantri	>500	Cempedak	Artocarpus integer (Thunb.) Merr	Moraceae		0,56	38,5	2	4	0,04	0,12	188,20	0,19	2,21	8,12
6952	127	2	Sukamantri	>500	Puspa	Schima wallichii Choisy	Theaceae		0,74	6,4	4	5	0,04	0,00	11,02	0,01	0,13	0,48
6953	127	2	Sukamantri	>500	Renggang	Sp4		0	0,5	3,2	3	4	0,04	0,00	1,76	0,00	0,02	0,08
6954	127	2	Sukamantri	>500	Puspa	Schima wallichii Choisy	Theaceae		0,74	13,0	3	9	0,04	0,01	70,31	0,07	0,83	3,03
6955	127	2	Sukamantri	>500	Puspa	Schima wallichii Choisy	Theaceae		0,74	16,9	8	12	0,04	0,02	146,47	0,15	1,72	6,32
6956	127	2	Sukamantri	>500	Mahoni	Swietenia macrophylla King	Meliaceae		0,61	29,3	10	15	0,04	0,07	413,47	0,41	4,86	17,83
6957	127	2	Sukamantri	>500	Afrika	Maesopsis eminii Engl.	Rhamnaceae		0,43	10,8	9	10	0,04	0,01	33,42	0,03	0,39	1,44
6958	127	2	Sukamantri	>500	Mahoni	Swietenia macrophylla King	Meliaceae		0,61	35,0	7	14	0,04	0,10	538,48	0,54	6,33	23,22
6959	127	2	Sukamantri	>500	Afrika	Maesopsis eminii Engl.	Rhamnaceae		0,43	37,2	14	16	0,04	0,11	494,61	0,49	5,81	21,33
6960	127	2	Sukamantri	>500	Puspa	Schima wallichii Choisy	Theaceae		0,74	15,6	3	10	0,04	0,02	107,34	0,11	1,26	4,63
6961	127	2	Sukamantri	>500	Puspa	Schima wallichii Choisy	Theaceae		0,74	17,2	3	11	0,04	0,02	139,96	0,14	1,64	6,04
6962	127	2	Sukamantri	>500	Afrika	Maesopsis eminii Engl.	Rhamnaceae		0,43	38,8	14	16	0,04	0,12	534,02	0,53	6,27	23,03
6963	127	2	Sukamantri	>500	Afrika	Maesopsis eminii Engl.	Rhamnaceae		0,43	21,0	12	13	0,04	0,03	143,27	0,14	1,68	6,18
6964	127	2	Sukamantri	>500	Afrika	Maesopsis eminii Engl.	Rhamnaceae		0,43	14,6	12	13	0,04	0,02	73,95	0,07	0,87	3,19
6965	127	2	Sukamantri	>500	Afrika	Maesopsis eminii Engl.	Rhamnaceae		0,43	16,9	9	10	0,04	0,02	75,38	0,08	0,89	3,25
6966	127	2	Sukamantri	>500	Puspa	Schima wallichii Choisy	Theaceae		0,74	20,4	6	11	0,04	0,03	191,06	0,19	2,24	8,24
6967	127	2	Sukamantri	>500	Puspa	Schima wallichii Choisy	Theaceae		0,74	20,0	7	13	0,04	0,03	216,32	0,22	2,54	9,33
6968	127	2	Sukamantri	>500	Mahoni	Swietenia macrophylla King	Meliaceae		0,61	62,0	10	19	0,04	0,30	2.033,12	2,03	23,89	87,67
6969	128	3	Sukamantri	>500	Puspa	Schima wallichii Choisy	Theaceae		0,74	25,1	9	15	0,04	0,05	373,33	0,37	4,39	16,10
6970	128	3	Sukamantri	>500	Suren	Toona sureni (Blume) Merr.	Meliaceae		0,39	13,0	7	9	0,04	0,01	39,11	0,04	0,46	1,69
6971	128	3	Sukamantri	>500	Puspa	Schima wallichii Choisy	Theaceae		0,74	19,4	6	10	0,04	0,03	160,35	0,16	1,88	6,91
6972	128	3	Sukamantri	>500	Puspa	Schima wallichii Choisy	Theaceae		0,74	8,9	4	8	0,04	0,01	31,39	0,03	0,37	1,35
6973	128	3	Sukamantri	>500	Sengon	Paraserianthes falcataria (L.) I.C.	Leguminosae / Fabaceae		0,33	22,6	7	14	0,04	0,04	137,54	0,14	1,62	5,93
6974	128	3	Sukamantri	>500	Puspa	Schima wallichii Choisy	Theaceae		0,74	14,0	5	9	0,04	0,02	80,03	0,08	0,94	3,45
6975	128	3	Sukamantri	>500	Puspa	Schima wallichii Choisy	Theaceae		0,74	12,1	4	10	0,04	0,01	67,38	0,07	0,79	2,91
6976	128	3	Sukamantri	>500	Puspa	Schima wallichii Choisy	Theaceae		0,74	9,9	3	6	0,04	0,01	29,06	0,03	0,34	1,25
6977	128	3	Sukamantri	>500	Puspa	Schima wallichii Choisy	Theaceae		0,74	15,0	5	8	0,04	0,02	81,07	0,08	0,95	3,50
6978	128	3	Sukamantri	>500	Mahoni	Swietenia macrophylla King	Meliaceae		0,61	12,1	5	8	0,04	0,01	46,01	0,05	0,54	1,98
6979	128	3	Sukamantri	>500	Puspa	Schima wallichii Choisy	Theaceae		0,74	13,0	6	9	0,04	0,01	70,31	0,07	0,83	3,03
6980	128	3	Sukamantri	>500	Kilunglum	Sp3		0	0,5	14,3	4	9	0,04	0,02	58,23	0,06	0,68	2,51
6981	128	3	Sukamantri	>500	Suren	Toona sureni (Blume) Merr.	Meliaceae		0,39	11,1	7	10	0,04	0,01	32,23	0,03	0,38	1,39
6982	128	3	Sukamantri	>500	Puspa	Schima wallichii Choisy	Theaceae		0,74	7,6	4	8	0,04	0,00	23,67	0,02	0,28	1,02
6983	128	3	Sukamantri	>500	Suren	Toona sureni (Blume) Merr.	Meliaceae		0,39	12,4	7	10	0,04	0,01	39,30	0,04	0,46	1,69
6984	128	3	Sukamantri	>500	Puspa	Schima wallichii Choisy	Theaceae		0,74	4,5	2	4	0,04	0,00	4,67	0,00	0,05	0,20
6985	128	3	Sukamantri	>500	Puspa	Schima wallichii Choisy	Theaceae		0,74	4,8	2	6	0,04	0,00	7,69	0,01	0,09	0,33
6986	128	3	Sukamantri	>500	Afrika	Maesopsis eminii Engl.	Rhamnaceae		0,43	22,3	14	16	0,04	0,04	193,01	0,19	2,27	8,32
6987	128	3	Sukamantri	>500	Puspa	Schima wallichii Choisy	Theaceae		0,74	5,1	3	6	0,04	0,00	8,65	0,01	0,10	0,37
6988	128	3	Sukamantri	>500	Puspa	Schima wallichii Choisy	Theaceae		0,74	20,0	7	11	0,04	0,03	185,63	0,19	2,18	8,00
6989	128	3	Sukamantri	>500	Puspa	Schima wallichii Choisy	Theaceae		0,74	12,1	7	11	0,04	0,01	73,52	0,07	0,86	3,17
6990	128	3	Sukamantri	>500	Puspa	Schima wallichii Choisy	Theaceae		0,74	20,0	7	12	0,04	0,03	201,03	0,20	2,36	8,67
6991	128	3	Sukamantri	>500	Ganitri	Elaeocarpus ganitrus Roxb. ex G.	Elaeocarpaceae		0,49	12,7	8	14	0,04	0,01	69,05	0,07	0,81	2,98
6992	128	3	Sukamantri	>500	Tisuk	Hibiscus macrophyllus Roxb. ex I	Malvaceae		0,4	14,3	7	11	0,04	0,02	57,04	0,06	0,67	2,46
6993	128	3	Sukamantri	>500	Suren	Toona sureni (Blume) Merr.	Meliaceae		0,39	18,8	9	12	0,04	0,03	99,14	0,10	1,16	4,28
6994	128	3	Sukamantri	>500	Mangga	Mangifera indica L.	Anacardiaceae		0,6	16,9	5	8	0,04	0,02	83,37	0,08	0,98	3,60
6995	128	3	Sukamantri	>500	Tisuk	Hibiscus macrophyllus Roxb. ex I	Malvaceae		0,4	19,7	8	14	0,04	0,03	127,97	0,13	1,50	5,52
6996	128	3	Sukamantri	>500	Mahoni	Swietenia macrophylla King	Meliaceae		0,61	16,2	5	9	0,04	0,02	87,87	0,09	1,03	3,79
6997	128	3	Sukamantri	>500	Puspa	Schima wallichii Choisy	Theaceae		0,74	24,5	12	18	0,04	0,05	420,94	0,42	4,95	18,15
6998	128	3	Sukamantri	>500	Puspa	Schima wallichii Choisy	Theaceae		0,74	7,3	5	8	0,04	0,00	21,89	0,02	0,26	0,94
6999	128	3	Sukamantri	>500	Puspa	Schima wallichii Choisy	Theaceae		0,74	7,0	5	7	0,04	0,00	17,86	0,02	0,21	0,77
7000	128	3	Sukamantri	>500	Mahoni	Swietenia macrophylla King	Meliaceae		0,61	13,0	4	6	0,04	0,01	40,63	0,04	0,48	1,75
7001	128	3	Sukamantri	>500	Mahoni	Swietenia macrophylla King	Meliaceae		0,61	8,6	4	6	0,04	0,01	18,90	0,02	0,22	0,82
7002	128	3	Sukamantri	>500	Mahoni	Swietenia macrophylla King	Meliaceae		0,61	22,9	5	9	0,04	0,04	165,27	0,17	1,94	7,13
7003	128	3	Sukamantri	>500	Sengon	Paraserianthes falcataria (L.) I.C.	Leguminosae / Fabaceae		0,33	5,4	6	7	0,04	0,00	5,31	0,01	0,06	0,23
7004	128	3	Sukamantri	>500	Puspa	Schima wallichii Choisy	Theaceae		0,74	5,7	2	4	0,04	0,00	7,40	0,01	0,09	0,32
7005	128	3	Sukamantri	>500	Sengon	Paraserianthes falcataria (L.) I.C.	Leguminosae / Fabaceae		0,33	10,8	4	8	0,04	0,01	21,38	0,02	0,25	0,92
7006	129	3	Sukamantri	>500	Pinus	Pinus merkusii Jungh. & de Vriese	Pinaceae		0,55	11,1	2	6	0,04	0,01	27,66	0,03	0,32	1,19
7007	129	3	Sukamantri	>500	Pinus	Pinus merkusii Jungh. & de Vriese	Pinaceae		0,55	11,5	2	6	0,04	0,01	29,12	0,03	0,34	1,26
7008	129	3	Sukamantri	>500	Puspa	Schima wallichii Choisy	Theaceae		0,74	17,5	3,5	6,5	0,04	0,02	89,39	0,09	1,05	3,85
7009	129	3	Sukamantri	>500	Sengon	Paraserianthes falcataria (L.) I.C.	Leguminosae / Fabaceae		0,33	5,1	2	4	0,04	0,00	2,85	0,00	0,03	0,12
7010	129	3	Sukamantri	>500	Puspa	Schima wallichii Choisy	Theaceae		0,74	8,3	3	8	0,04	0,01	27,40	0,03	0,32	1,18
7011	129	3	Sukamantri	>500	Puspa	Schima wallichii Choisy	Theaceae		0,74	13,4	4	8	0,04	0,01	65,97	0,07	0,78	2,84
7012	129	3	Sukamantri	>500	Jengkol	Archidendron pauciflorum (Benth	Leguminosae / Fabaceae		0,4	6,0	2	5	0,04	0,00	5,71	0,01	0,07	0,25
7013	129	3	Sukamantri	>500	Tisuk	Hibiscus macrophyllus Roxb. ex I	Malvaceae		0,4	2,9	2	2,5	0,04	0,00	0,77	0,00	0,01	0,03
7014	129	3	Sukamantri	>500	Tisuk	Hibiscus macrophyllus Roxb. ex I	Malvaceae		0,4	3,2	2	3	0,04	0,00	1,10	0,00	0,01	0,05
7015	129	3	Sukamantri	>500	Jengkol	Archidendron pauciflorum (Benth	Leguminosae / Fabaceae		0,4	19,4	4	6	0,04	0,03	57,16	0,06	0,67	2,47
7016	129	3	Sukamantri	>500	Puspa	Schima wallichii Choisy	Theaceae		0,74	4,8	2	3,5	0,04	0,00	4,69	0,00	0,06	0,20
7017	129	3	Sukamantri	>500	Puspa	Schima wallichii Choisy	Theaceae		0,74	4,8	2	3	0,04	0,00	4,07	0,00	0,05	0,18
7018	129	3	Sukamantri	>500	Puspa	Schima wallichii Choisy	Theaceae		0,74	3,2	2	3,5	0,04	0,00	2,23	0,00	0,03	0,10
7019	129	3	Sukamantri	>500	Puspa	Schima wallichii Choisy	Theaceae		0,74	3,2	2	3,5	0,04	0,00	2,23	0,00	0,03	0,10
7020	129	3	Sukamantri	>500	Puspa	Schima wallichii Choisy	Theaceae		0,74	4,8	2	3,5	0,04	0,00	4,69	0,00	0,06	0,20
7021	129	3	Sukamantri	>500	Puspa	Schima wallichii Choisy	Theaceae		0,74	8,9	6	8	0,04	0,01	31,39	0,03	0,37	1,35
7022	129	3	Sukamantri	>500	Puspa	Schima wallichii Choisy	Theaceae		0,74	10,8	6	9	0,04	0,01	49,90	0,05	0,59	2,15
7023	129	3	Sukamantri	>500	Puspa	Schima wallichii Choisy	Theaceae		0,74	12,1	6	10	0,04	0,01	67,38	0,07	0,79	2,91
7024	129	3	Sukamantri	>500	Puspa	Schima wallichii Choisy	Theaceae		0,74	13,7	6	10	0,04	0,01	84,50	0,08	0,99	3,64
7025	129	3	Sukamantri	>500	Puspa	Schima wallichii Choisy	Theaceae		0,74	9,5	6	8	0,04	0,01	35,62	0,04	0,42	1,54
7026	129	3	Sukamantri	>500	Picung	Pangium edule Reinw. ex Blume	Achariaceae		0,5	2,9	2	3	0,04	0,00	1,12	0,00	0,01	0,05
7027	129	3	Sukamantri	>500	Afrika	Maesopsis eminii Engl.	Rhamnaceae		0,43	17,5	6	8	0,04	0,02	65,76	0,07	0,77	2,84
7028	129	3	Sukamantri	>500	Petai	Parkia speciosa Hassk.	Leguminosae / Fabaceae		0,45	3,2	2	3	0,04	0,00	1,23	0,00	0,01	0,05
7029	129	3	Sukamantri	>500	Puspa	Schima wallichii Choisy	Theaceae		0,74	3,2	2	3	0,04	0,00	1,94	0,00	0,02	0,08
7030	129	3	Sukamantri	>500	Puspa	Schima wallichii Choisy	Theaceae		0,74	6,7	2	5	0,04	0,00	12,05	0,01	0,14	0,52
7031	129	3	Sukamantri	>500	Puspa	Schima wallichii Choisy	Theaceae		0,74	6,4	2	5	0,04	0,00	11,02	0,01	0,13	0,48
7032	129	3	Sukamantri	>500	Manggis	Mangostana garcinia Gaertn.	Clusiaceae		0,81	4,5	1	3	0,04	0,00	3,90	0,00	0,05	0,17
7033	129	3	Sukamantri	>500	Mahoni	Swietenia macrophylla King	Meliaceae		0,61	8,6	6	7	0,04	0,01	21,77	0,02	0,26	0,94
7034	129	3	Sukamantri	>500	Alpukat	Persea americana Mill.	Lauraceae		0,54	14,3	4	7	0,04	0,02	49,63	0,05	0,58	2,14
7035	129	3	Sukamantri	>500	Nangka	Artocarpus heterophyllus Lam.	Moraceae		0,61	12,7	4	6	0,04	0,01	38,84	0,04	0,46	1,67
7036	129	3	Sukamantri	>500	Puspa	Schima wallichii Choisy	Theaceae		0,74	11,1	4	10	0,04	0,01	57,95	0,06	0,68	2,50
7037	129	3	Sukamantri	>500	Puspa	Schima wallichii Choisy	Theaceae		0,74	12,4	4	10	0,04	0,01	70,66	0,07	0,83	3,05
7038	129	3	Sukamantri	>500	Mahoni	Swietenia macrophylla King	Meliaceae		0,61	10,2	2,5	6	0,04	0,01	25,80	0,03	0,30	1,11
7039	129	3	Sukamantri	>500	Puspa	Schima wallichii Choisy	Theaceae		0,74	18,1	5	11	0,04	0,03	154,53	0,15	1,82	6,66
7040	130	3	Sukamantri	>500	Puspa	Schima wallichii Choisy	Theaceae		0,74	6,7	2	4	0,04	0,00	9,82	0,01	0,12	0,42
7041	130	3	Sukamantri	>500	Puspa	Schima wallichii Choisy	Theaceae		0,74	5,4	4	5	0,04	0,00	8,18	0,01	0,10	0,35
7042	130	3	Sukamantri	>500	Puspa	Schima wallichii Choisy	Theaceae		0,74	8,3	4	6	0,04	0,01	21,05	0,02	0,25	0,91
7043	130	3	Sukamantri	>500	Puspa	Schima wallichii Choisy	Theaceae		0,74	5,7	4	6	0,04	0,00	10,73	0,01	0,13	0,46
7044	130	3	Sukamantri	>500	Puspa	Schima wallichii Choisy	Theaceae		0,74	5,1	4	6	0,04	0,00	8,65	0,01	0,10	0,37
7045	130	3	Sukamantri	>500	Puspa	Schima wallichii Choisy	Theaceae		0,74	6,4	4	3	0,04	0,00	6,90	0,01	0,08	0,30
7046	130	3	Sukamantri	>500	Puspa	Schima wallichii Choisy	Theaceae		0,74	17,2	4	7	0,04	0,02	92,51	0,09	1,09	3,99
7047	130	3	Sukamantri	>500	Puspa	Schima wallichii Choisy	Theaceae		0,74	7,3	2	4	0,04	0,00	11,60	0,01	0,14	0,50
7048	130	3	Sukamantri	>500	Kihaji	Dysoxylum macrocarpum Blume	Meliaceae		0,73	11,5	4	8	0,04	0,01	49,12	0,05	0,58	2,12
7049	130	3	Sukamantri	>500	Puspa	Schima wallichii Choisy	Theaceae		0,74	16,9	4	9	0,04	0,02	112,54	0,11	1,32	4,85
7050	130	3	Sukamantri	>500	Puspa	Schima wallichii Choisy	Theaceae		0,74	7,3	3	6	0,04	0,00	16,82	0,02	0,20	0,73
7051	130	3	Sukamantri	>500	Puspa	Schima wallichii Choisy	Theaceae		0,74	8,3	3	7	0,04	0,01	24,25	0,02	0,28	1,05
7052	130	3	Sukamantri	>500	Puspa	Schima wallichii Choisy	Theaceae		0,74	8,3	3	7	0,04	0,01	24,25	0,02	0,28	1,05
7053	130	3	Sukamantri	>500	Puspa	Schima wallichii Choisy	Theaceae		0,74	16,5	4	8	0,04	0,02	97,56	0,10	1,15	4,21
7054	130	3	Sukamantri	>500	Huru Keling	Litsea sp.	Lauraceae		0,425	6,7	3,5	5	0,04	0,00	7,25	0,01	0,09	0,31
7055	130	3	Sukamantri	>500	Huru Keling	Litsea sp.	Lauraceae		0,425	6,4	3,5	5	0,04	0,00	6,63	0,01	0,08	0,29
7056	130	3	Sukamantri	>500	Puspa	Schima wallichii Choisy	Theaceae		0,74	5,7	3	6	0,04	0,00	10,73	0,01	0,13	0,46
7057	130	3	Sukamantri	>500	Puspa	Schima wallichii Choisy	Theaceae		0,74	6,0	3	6	0,04	0,00	11,85	0,01	0,14	0,51
7058	130	3	Sukamantri	>500	Puspa	Schima wallichii Choisy	Theaceae		0,74	7,6	3	5	0,04	0,00	15,39	0,02	0,18	0,66
7059	130	3	Sukamantri	>500	Puspa	Schima wallichii Choisy	Theaceae		0,74	22,6	4	10	0,04	0,04	211,76	0,21	2,49	9,13
7060	130	3	Sukamantri	>500	Puspa	Schima wallichii Choisy	Theaceae		0,74	6,7	3	5	0,04	0,00	12,05	0,01	0,14	0,52
7061	130	3	Sukamantri	>500	Tisuk	Hibiscus macrophyllus Roxb. ex I	Malvaceae		0,4	15,9	5	7	0,04	0,02	45,73	0,05	0,54	1,97
7062	130	3	Sukamantri	>500	Puspa	Schima wallichii Choisy	Theaceae		0,74	6,7	2	4	0,04	0,00	9,82	0,01	0,12	0,42

7063	130	3	Sukamantri	>500	Puspa	Schima wallichii Choisy	Theaceae		0,74	9,9	2	5	0,04	0,01	24,59	0,02	0,29	1,06
7064	130	3	Sukamantri	>500	Puspa	Schima wallichii Choisy	Theaceae		0,74	7,0	2	6	0,04	0,00	15,50	0,02	0,18	0,67
7065	130	3	Sukamantri	>500	Puspa	Schima wallichii Choisy	Theaceae		0,74	8,9	4	7	0,04	0,01	27,77	0,03	0,33	1,20
7066	130	3	Sukamantri	>500	Puspa	Schima wallichii Choisy	Theaceae		0,74	6,4	2	4	0,04	0,00	8,98	0,01	0,11	0,39
7067	130	3	Sukamantri	>500	Puspa	Schima wallichii Choisy	Theaceae		0,74	8,0	3	5	0,04	0,00	16,58	0,02	0,19	0,72
7068	130	3	Sukamantri	>500	Puspa	Schima wallichii Choisy	Theaceae		0,74	11,8	3	5	0,04	0,01	34,00	0,03	0,40	1,47
7069	130	3	Sukamantri	>500	Puspa	Schima wallichii Choisy	Theaceae		0,74	12,7	2,5	5,5	0,04	0,01	42,80	0,04	0,50	1,85
7070	131	2	Sukamantri	>500	Huru Kacang	Neolitsea triplinervia Merr.	Lauraceae		0,56	8,0	2	6	0,04	0,00	15,18	0,02	0,18	0,65
7071	131	2	Sukamantri	>500	Puspa	Schima wallichii Choisy	Theaceae		0,74	11,5	5	9	0,04	0,01	55,41	0,06	0,65	2,39
7072	131	2	Sukamantri	>500	Kihaji	Dysoxylum macrocarpum Blume	Meliaceae		0,73	4,5	3	5	0,04	0,00	5,66	0,01	0,07	0,24
7073	131	2	Sukamantri	>500	Huru Keling	Litsea sp.	Lauraceae		0,425	4,8	2	4	0,04	0,00	3,19	0,00	0,04	0,14
7074	131	2	Sukamantri	>500	Puspa	Schima wallichii Choisy	Theaceae		0,74	9,9	4	7	0,04	0,01	33,47	0,03	0,39	1,44
7075	131	2	Sukamantri	>500	Sampang	Melicope denhamii (Seem.) T.G.I	Rutaceae		0,366	15,0	7	11	0,04	0,02	56,95	0,06	0,67	2,46
7076	131	2	Sukamantri	>500	Kihaji	Dysoxylum macrocarpum Blume	Meliaceae		0,73	7,3	4	7	0,04	0,00	19,13	0,02	0,22	0,82
7077	131	2	Sukamantri	>500	Puspa	Schima wallichii Choisy	Theaceae		0,74	4,8	4	5,5	0,04	0,00	7,10	0,01	0,08	0,31
7078	131	2	Sukamantri	>500	Kihaji	Dysoxylum macrocarpum Blume	Meliaceae		0,73	2,9	2	2,5	0,04	0,00	1,34	0,00	0,02	0,06
7079	131	2	Sukamantri	>500	Afrika	Maesopsis eminii Engl.	Rhamnaceae		0,43	5,1	7	8	0,04	0,00	6,85	0,01	0,08	0,30
7080	131	2	Sukamantri	>500	Puspa	Schima wallichii Choisy	Theaceae		0,74	7,3	7	9	0,04	0,00	24,38	0,02	0,29	1,05
7081	131	2	Sukamantri	>500	Puspa	Schima wallichii Choisy	Theaceae		0,74	10,8	9	10	0,04	0,01	54,95	0,05	0,65	2,37
7082	131	2	Sukamantri	>500	Tisuk	Hibiscus macrophyllus Roxb. ex I	Malvaceae		0,4	26,7	12	15	0,04	0,06	237,79	0,24	2,79	10,25
7083	131	2	Sukamantri	>500	Puspa	Schima wallichii Choisy	Theaceae		0,74	8,3	6	9	0,04	0,01	30,52	0,03	0,36	1,32
7084	131	2	Sukamantri	>500	Afrika	Maesopsis eminii Engl.	Rhamnaceae		0,43	9,5	11	12	0,04	0,01	31,40	0,03	0,37	1,35
7085	131	2	Sukamantri	>500	Puspa	Schima wallichii Choisy	Theaceae		0,74	8,6	5	8	0,04	0,01	29,36	0,03	0,35	1,27
7086	131	2	Sukamantri	>500	Mahoni	Swietenia macrophylla King	Meliaceae		0,61	12,4	3	9	0,04	0,01	53,75	0,05	0,63	2,32
7087	131	2	Sukamantri	>500	Suren	Toona sureni (Blume) Merr.	Meliaceae		0,39	8,9	6	7	0,04	0,01	15,45	0,02	0,18	0,67
7088	131	2	Sukamantri	>500	Puspa	Schima wallichii Choisy	Theaceae		0,74	4,1	3	5	0,04	0,00	5,00	0,01	0,06	0,22
7089	131	2	Sukamantri	>500	Puspa	Schima wallichii Choisy	Theaceae		0,74	4,5	3	5	0,04	0,00	5,73	0,01	0,07	0,25
7090	131	2	Sukamantri	>500	Afrika	Maesopsis eminii Engl.	Rhamnaceae		0,43	15,6	9	11	0,04	0,02	71,24	0,07	0,84	3,07
7091	131	2	Sukamantri	>500	Afrika	Maesopsis eminii Engl.	Rhamnaceae		0,43	23,2	13	15	0,04	0,04	196,46	0,20	2,31	8,47
7092	131	2	Sukamantri	>500	Afrika	Maesopsis eminii Engl.	Rhamnaceae		0,43	14,0	11	12	0,04	0,02	63,34	0,06	0,74	2,73
7093	131	2	Sukamantri	>500	Suren	Toona sureni (Blume) Merr.	Meliaceae		0,39	6,0	6	7	0,04	0,00	7,59	0,01	0,09	0,33
7094	131	2	Sukamantri	>500	Puspa	Schima wallichii Choisy	Theaceae		0,74	9,9	7	10	0,04	0,01	46,40	0,05	0,55	2,00
7095	131	2	Sukamantri	>500	Afrika	Maesopsis eminii Engl.	Rhamnaceae		0,43	12,4	14	15	0,04	0,01	62,30	0,06	0,73	2,69
7096	131	2	Sukamantri	>500	Kihaji	Dysoxylum macrocarpum Blume	Meliaceae		0,73	17,5	7	13	0,04	0,02	166,59	0,17	1,96	7,18
7097	131	2	Sukamantri	>500	Afrika	Maesopsis eminii Engl.	Rhamnaceae		0,43	5,1	12	65	0,04	0,00	46,66	0,05	0,55	2,01
7098	131	2	Sukamantri	>500	Tisuk	Hibiscus macrophyllus Roxb. ex I	Malvaceae		0,4	24,8	15	18	0,04	0,05	245,33	0,25	2,88	10,58
7099	131	2	Sukamantri	>500	Afrika	Maesopsis eminii Engl.	Rhamnaceae		0,43	11,5	11	12	0,04	0,01	43,86	0,04	0,52	1,89
7100	131	2	Sukamantri	>500	Puspa	Schima wallichii Choisy	Theaceae		0,74	6,4	6	7	0,04	0,00	14,99	0,01	0,18	0,65
7101	131	2	Sukamantri	>500	Puspa	Schima wallichii Choisy	Theaceae		0,74	7,3	6	8	0,04	0,00	21,89	0,02	0,26	0,94
7102	131	2	Sukamantri	>500	Puspa	Schima wallichii Choisy	Theaceae		0,74	5,1	5	6	0,04	0,00	8,65	0,01	0,10	0,37
7103	131	2	Sukamantri	>500	Puspa	Schima wallichii Choisy	Theaceae		0,74	5,1	5	6	0,04	0,00	8,65	0,01	0,10	0,37
7104	131	2	Sukamantri	>500	Afrika	Maesopsis eminii Engl.	Rhamnaceae		0,43	26,1	13	15	0,04	0,05	243,10	0,24	2,86	10,48
7105	131	2	Sukamantri	>500	Nangka	Artocarpus heterophyllus Lam.	Moraceae		0,61	34,4	12	16	0,04	0,09	588,42	0,59	6,91	25,37
7106	131	2	Sukamantri	>500	Puspa	Schima wallichii Choisy	Theaceae		0,74	9,9	5	8	0,04	0,01	37,82	0,04	0,44	1,63
7107	131	2	Sukamantri	>500	Puspa	Schima wallichii Choisy	Theaceae		0,74	8,0	5	9	0,04	0,00	28,41	0,03	0,33	1,23
7108	131	2	Sukamantri	>500	Puspa	Schima wallichii Choisy	Theaceae		0,74	6,4	2	4	0,04	0,00	8,98	0,01	0,11	0,39
7109	131	2	Sukamantri	>500	Manglid	Magnolia champaca (L.) Baill. ex	Magnoliaceae		0,53	7,0	6	7	0,04	0,00	13,15	0,01	0,15	0,57
7110	131	2	Sukamantri	>500	Kihaji	Dysoxylum macrocarpum Blume	Meliaceae		0,73	10,5	4	7	0,04	0,01	37,06	0,04	0,44	1,60
7111	131	2	Sukamantri	>500	Afrika	Maesopsis eminii Engl.	Rhamnaceae		0,43	12,4	10	11	0,04	0,01	46,89	0,05	0,55	2,02
7112	131	2	Sukamantri	>500	Puspa	Schima wallichii Choisy	Theaceae		0,74	11,5	6	9	0,04	0,01	55,41	0,06	0,65	2,39
7113	131	2	Sukamantri	>500	Tisuk	Hibiscus macrophyllus Roxb. ex I	Malvaceae		0,4	8,9	7	9	0,04	0,01	19,90	0,02	0,23	0,86
7114	131	2	Sukamantri	>500	Puspa	Schima wallichii Choisy	Theaceae		0,74	8,9	6	9	0,04	0,01	34,96	0,03	0,41	1,51
7115	131	2	Sukamantri	>500	Puspa	Schima wallichii Choisy	Theaceae		0,74	10,5	4	9	0,04	0,01	47,24	0,05	0,56	2,04
7116	131	2	Sukamantri	>500	Nangka	Artocarpus heterophyllus Lam.	Moraceae		0,61	19,1	9	12	0,04	0,03	154,02	0,15	1,81	6,64
7117	131	2	Sukamantri	>500	Puspa	Schima wallichii Choisy	Theaceae		0,74	5,4	4	6	0,04	0,00	9,67	0,01	0,11	0,42
7118	131	2	Sukamantri	>500	Kilunglum	Sp3		0	0,5	9,9	2,5	6	0,04	0,01	20,29	0,02	0,24	0,88
7119	131	2	Sukamantri	>500	Afrika	Maesopsis eminii Engl.	Rhamnaceae		0,43	11,8	7	8	0,04	0,01	31,81	0,03	0,37	1,37
7120	131	2	Sukamantri	>500	Sampang	Melicope denhamii (Seem.) T.G.I	Rutaceae		0,366	11,5	2,5	4,5	0,04	0,01	15,41	0,02	0,18	0,66
7121	131	2	Sukamantri	>500	Puspa	Schima wallichii Choisy	Theaceae		0,74	9,9	3	7	0,04	0,01	33,47	0,03	0,39	1,44
7122	131	2	Sukamantri	>500	Puspa	Schima wallichii Choisy	Theaceae		0,74	12,4	3	7	0,04	0,01	50,97	0,05	0,60	2,20
7123	131	2	Sukamantri	>500	Puspa	Schima wallichii Choisy	Theaceae		0,74	9,9	5	9	0,04	0,01	42,13	0,04	0,50	1,82
7124	131	2	Sukamantri	>500	Afrika	Maesopsis eminii Engl.	Rhamnaceae		0,43	11,1	10	11	0,04	0,01	38,46	0,04	0,45	1,66
7125	131	2	Sukamantri	>500	Puspa	Schima wallichii Choisy	Theaceae		0,74	12,1	5	10	0,04	0,01	67,38	0,07	0,79	2,91
7126	131	2	Sukamantri	>500	Kihaji	Dysoxylum macrocarpum Blume	Meliaceae		0,73	5,7	5	7	0,04	0,00	12,21	0,01	0,14	0,53
7127	131	2	Sukamantri	>500	Afrika	Maesopsis eminii Engl.	Rhamnaceae		0,43	22,0	14	16	0,04	0,04	187,98	0,19	2,21	8,11
7128	131	2	Sukamantri	>500	Huru keling	Litsea sp.	Lauraceae		0,425	8,6	2	7	0,04	0,01	15,63	0,02	0,18	0,67
7129	131	2	Sukamantri	>500	Puspa	Schima wallichii Choisy	Theaceae		0,74	10,2	7	11	0,04	0,01	53,66	0,05	0,63	2,31
7130	131	2	Sukamantri	>500	Puspa	Schima wallichii Choisy	Theaceae		0,74	5,4	3	5	0,04	0,00	8,18	0,01	0,10	0,35
7131	131	2	Sukamantri	>500	Afrika	Maesopsis eminii Engl.	Rhamnaceae		0,43	18,1	11	14	0,04	0,03	117,22	0,12	1,38	5,05
7132	131	2	Sukamantri	>500	Afrika	Maesopsis eminii Engl.	Rhamnaceae		0,43	22,6	13	15	0,04	0,04	186,72	0,19	2,19	8,05
7133	131	2	Sukamantri	>500	Puspa	Schima wallichii Choisy	Theaceae		0,74	6,7	7	8,5	0,04	0,00	19,59	0,02	0,23	0,84
7134	131	2	Sukamantri	>500	Afrika	Maesopsis eminii Engl.	Rhamnaceae		0,43	25,5	12	15	0,04	0,05	232,35	0,23	2,73	10,02
7135	131	2	Sukamantri	>500	Huru keling	Litsea sp.	Lauraceae		0,425	12,4	5	7	0,04	0,01	30,67	0,03	0,36	1,32
7136	131	2	Sukamantri	>500	Manglid	Magnolia champaca (L.) Baill. ex	Magnoliaceae		0,53	33,1	12	16	0,04	0,09	482,76	0,48	5,67	20,82
7137	132	1	Sukamantri	>500	Afrika	Maesopsis eminii Engl.	Rhamnaceae		0,43	13,0	10	11	0,04	0,01	51,39	0,05	0,60	2,22
7138	132	1	Sukamantri	>500	Afrika	Maesopsis eminii Engl.	Rhamnaceae		0,43	8,9	9	10	0,04	0,01	23,42	0,02	0,28	1,01
7139	132	1	Sukamantri	>500	Afrika	Maesopsis eminii Engl.	Rhamnaceae		0,43	5,7	7	8	0,04	0,00	8,50	0,01	0,10	0,37
7140	132	1	Sukamantri	>500	Afrika	Maesopsis eminii Engl.	Rhamnaceae		0,43	6,0	7	8	0,04	0,00	9,38	0,01	0,11	0,40
7141	132	1	Sukamantri	>500	Afrika	Maesopsis eminii Engl.	Rhamnaceae		0,43	13,4	10	11	0,04	0,01	53,71	0,05	0,63	2,32
7142	132	1	Sukamantri	>500	Afrika	Maesopsis eminii Engl.	Rhamnaceae		0,43	9,9	7	8	0,04	0,01	23,00	0,02	0,27	0,99
7143	132	1	Sukamantri	>500	Afrika	Maesopsis eminii Engl.	Rhamnaceae		0,43	6,7	5	6	0,04	0,00	8,66	0,01	0,10	0,37
7144	132	1	Sukamantri	>500	Afrika	Maesopsis eminii Engl.	Rhamnaceae		0,43	15,6	11	12	0,04	0,02	77,15	0,08	0,91	3,33
7145	132	1	Sukamantri	>500	Sengon	Paraserianthes falcataria (L.) I.C.	Leguminosae / Fabaceae		0,33	8,0	6	7	0,04	0,00	10,77	0,01	0,13	0,46
7146	132	1	Sukamantri	>500	Afrika	Maesopsis eminii Engl.	Rhamnaceae		0,43	8,9	7	8	0,04	0,01	19,09	0,02	0,22	0,82
7147	132	1	Sukamantri	>500	Afrika	Maesopsis eminii Engl.	Rhamnaceae		0,43	9,5	6	7	0,04	0,01	19,17	0,02	0,23	0,83
7148	132	1	Sukamantri	>500	Afrika	Maesopsis eminii Engl.	Rhamnaceae		0,43	9,5	6	8	0,04	0,01	21,66	0,02	0,25	0,93
7149	132	1	Sukamantri	>500	Afrika	Maesopsis eminii Engl.	Rhamnaceae		0,43	7,6	6	7	0,04	0,00	12,74	0,01	0,15	0,55
7150	132	1	Sukamantri	>500	Afrika	Maesopsis eminii Engl.	Rhamnaceae		0,43	16,5	10	11	0,04	0,02	79,43	0,08	0,93	3,43
7151	132	1	Sukamantri	>500	Afrika	Maesopsis eminii Engl.	Rhamnaceae		0,43	9,2	9	10	0,04	0,01	24,97	0,02	0,29	1,08
7152	132	1	Sukamantri	>500	Afrika	Maesopsis eminii Engl.	Rhamnaceae		0,43	7,0	7	8	0,04	0,00	12,27	0,01	0,14	0,53
7153	132	1	Sukamantri	>500	Pisitan	Dysoxylum parasiticum (Osbeck	Meliaceae		0,6	6,4	2,5	4,5	0,04	0,00	8,26	0,01	0,10	0,36
7154	132	1	Sukamantri	>500	Afrika	Maesopsis eminii Engl.	Rhamnaceae		0,43	13,0	11	12	0,04	0,01	55,66	0,06	0,65	2,40
7155	132	1	Sukamantri	>500	Afrika	Maesopsis eminii Engl.	Rhamnaceae		0,43	7,6	9	10	0,04	0,00	17,66	0,02	0,21	0,76
7156	132	1	Sukamantri	>500	Afrika	Maesopsis eminii Engl.	Rhamnaceae		0,43	12,1	9	10	0,04	0,01	40,98	0,04	0,48	1,77
7157	132	1	Sukamantri	>500	Afrika	Maesopsis eminii Engl.	Rhamnaceae		0,43	6,7	7	8	0,04	0,00	11,27	0,01	0,13	0,49
7158	132	1	Sukamantri	>500	Afrika	Maesopsis eminii Engl.	Rhamnaceae		0,43	4,8	6	7	0,04	0,00	5,38	0,01	0,06	0,23
7159	132	1	Sukamantri	>500	Afrika	Maesopsis eminii Engl.	Rhamnaceae		0,43	10,8	8	9	0,04	0,01	30,35	0,03	0,36	1,31
7160	132	1	Sukamantri	>500	Afrika	Maesopsis eminii Engl.	Rhamnaceae		0,43	10,8	9	10	0,04	0,01	33,42	0,03	0,39	1,44
7161	132	1	Sukamantri	>500	Afrika	Maesopsis eminii Engl.	Rhamnaceae		0,43	14,0	10	11	0,04	0,02	58,49	0,06	0,69	2,52
7162	132	1	Sukamantri	>500	Afrika	Maesopsis eminii Engl.	Rhamnaceae		0,43	9,5	9	10	0,04	0,01	26,57	0,03	0,31	1,15
7163	132	1	Sukamantri	>500	Afrika	Maesopsis eminii Engl.	Rhamnaceae		0,43	8,0	8	9	0,04	0,00	17,28	0,02	0,20	0,75
7164	132	1	Sukamantri	>500	Afrika	Maesopsis eminii Engl.	Rhamnaceae		0,43	12,7	9	10	0,04	0,01	45,01	0,05	0,53	1,94
7165	132	1	Sukamantri	>500	Afrika	Maesopsis eminii Engl.	Rhamnaceae		0,43	4,1	4	5	0,04	0,00	3,04	0,00	0,04	0,13
7166	132	1	Sukamantri	>500	Afrika	Maesopsis eminii Engl.	Rhamnaceae		0,43	15,9	10	11	0,04	0,02	73,93	0,07	0,87	3,19
7167	132	1	Sukamantri	>500	Afrika	Maesopsis eminii Engl.	Rhamnaceae		0,43	11,1	9	10	0,04	0,01	35,25	0,04	0,41	1,52
7168	132	1	Sukamantri	>500	Afrika	Maesopsis eminii Engl.	Rhamnaceae		0,43	14,0	9	10	0,04	0,02	53,60	0,05	0,63	2,31
7169	132	1	Sukamantri	>500	Afrika	Maesopsis eminii Engl.	Rhamnaceae		0,43	11,8	9	10	0,04	0,01	39,02	0,04	0,46	1,68
7170	132	1	Sukamantri	>500	Sengon	Paraserianthes falcataria (L.) I.C.	Leguminosae / Fabaceae		0,33	6,7	6	7	0,04	0,00	7,83	0,01	0,09	0,34
7171	132	1	Sukamantri	>500	Afrika	Maesopsis eminii Engl.	Rhamnaceae		0,43	13,7	10	11	0,04	0,01	56,08	0,06	0,66	2,42
7172	132	1	Sukamantri	>500	Afrika	Maesopsis eminii Engl.	Rhamnaceae		0,43	24,2	12	13	0,04	0,05	185,52	0,19	2,18	8,00
7173	132	1	Sukamantri	>500	Afrika	Maesopsis eminii Engl.	Rhamnaceae		0,43	9,5	7	8	0,04	0,01	21,66	0,02	0,25	0,93
7174	132	1	Sukamantri	>500	Afrika	Maesopsis eminii Engl.	Rhamnaceae		0,43	28,6	12	13	0,04	0,06	252,88	0,25	2,97	10,90
7175	132	1	Sukamantri	>500	Afrika	Maesopsis eminii Engl.	Rhamnaceae		0,43	7,3	7	8	0,04	0,00	13,31	0,01	0,16	0,57
7176	132	1	Sukamantri	>500	Afrika	Maesopsis eminii Engl.	Rhamnaceae		0,43	10,2	8	9	0,04	0,01	27,16	0,03	0,32	1,17
7177	132	1	Sukamantri	>500	Afrika	Maesopsis eminii Engl.	Rhamnaceae		0,43	8,3	8	9	0,04	0,01	18,56	0,02	0,22	0,80
7178	132	1	Sukamantri	>500	Afrika	Maesopsis eminii Engl.	Rhamnaceae		0,43	14,6	9	10	0,04	0,02	58,15	0,06	0,68	2,51
7179	132	1	Sukamantri	>500	Afrika	Maesopsis eminii Engl.	Rhamnaceae		0,43	7,6	7	8	0,04	0,00	14,39	0,01	0,17	0,62
7180	132	1	Sukamantri	>500	Afrika	Maesopsis eminii Engl.	Rhamnaceae		0,43	10,8	8	9	0,04	0,01	30,35	0,03	0,36	1,31
7181	132	1	Sukamantri	>500	Afrika	Maesopsis eminii Engl.	Rhamnaceae		0,43	12,1	9	12	0,04	0,01	48,43	0,05	0,57	2,09
7182	132	1	Sukamantri	>500	Afrika	Maesopsis eminii Engl.	Rhamnaceae		0,43	19,1	10	11	0,04	0,03	103,24	0,10	1,21	4,45
7183	132	1	Sukamantri	>500	Sengon	Paraserianthes falcataria (L.) I.C.	Leguminosae / Fabaceae		0,33	4,5	6	7	0,04	0,00	3,72	0,00	0,04	0,16
7184	132	1	Sukamantri	>500	Afrika	Maesopsis eminii Engl.	Rhamnaceae		0,43	13,7	10	11	0,04	0,01	56,08	0,06	0,66	2,42
7185	132	1	Sukamantri	>500	Afrika	Maesopsis eminii Engl.	Rhamnaceae		0,43	10,2	9	10	0,04	0,01	29,91	0,03	0,35	1,29
7186	132	1	Sukamantri	>500	Afrika	Maesopsis eminii Engl.	Rhamnaceae		0,43	8,0	9	10	0,04	0,00	19,03	0,02	0,22	0,82

7187	132	1	Sukamantri	>500	Afrika	Maesopsis eminii Engl.	Rhamnaceae		0,43	15,9	12	14	0,04	0,02	92,20	0,09	1,08	3,98
7188	132	1	Sukamantri	>500	Afrika	Maesopsis eminii Engl.	Rhamnaceae		0,43	8,3	9	10	0,04	0,01	20,45	0,02	0,24	0,88
7189	132	1	Sukamantri	>500	Afrika	Maesopsis eminii Engl.	Rhamnaceae		0,43	13,7	10	11	0,04	0,01	56,08	0,06	0,66	2,42
7190	132	1	Sukamantri	>500	Afrika	Maesopsis eminii Engl.	Rhamnaceae		0,43	28,3	11	14	0,04	0,06	265,16	0,27	3,12	11,43
7191	132	1	Sukamantri	>500	Afrika	Maesopsis eminii Engl.	Rhamnaceae		0,43	15,3	9	10	0,04	0,02	62,87	0,06	0,74	2,71
7192	132	1	Sukamantri	>500	Afrika	Maesopsis eminii Engl.	Rhamnaceae		0,43	8,0	6	7,5	0,04	0,00	14,62	0,01	0,17	0,63
7193	132	1	Sukamantri	>500	Afrika	Maesopsis eminii Engl.	Rhamnaceae		0,43	24,5	12	14	0,04	0,05	203,37	0,20	2,39	8,77
7194	132	1	Sukamantri	>500	Afrika	Maesopsis eminii Engl.	Rhamnaceae		0,43	12,7	10	11	0,04	0,01	49,12	0,05	0,58	2,12
7195	132	1	Sukamantri	>500	Afrika	Maesopsis eminii Engl.	Rhamnaceae		0,43	9,2	9	10	0,04	0,01	24,97	0,02	0,29	1,08
7196	132	1	Sukamantri	>500	Afrika	Maesopsis eminii Engl.	Rhamnaceae		0,43	19,7	11	12	0,04	0,03	118,73	0,12	1,40	5,12
7197	132	1	Sukamantri	>500	Afrika	Maesopsis eminii Engl.	Rhamnaceae		0,43	25,8	12	13	0,04	0,05	208,49	0,21	2,45	8,99
7198	132	1	Sukamantri	>500	Afrika	Maesopsis eminii Engl.	Rhamnaceae		0,43	9,5	7	8	0,04	0,01	21,66	0,02	0,25	0,93
7199	132	1	Sukamantri	>500	Afrika	Maesopsis eminii Engl.	Rhamnaceae		0,43	6,4	7	8	0,04	0,00	10,31	0,01	0,12	0,44
7200	132	1	Sukamantri	>500	Afrika	Maesopsis eminii Engl.	Rhamnaceae		0,43	8,0	6	7	0,04	0,00	13,72	0,01	0,16	0,59
7201	132	1	Sukamantri	>500	Sengon	Paraserianthes falcataria (L.) I.C	Leguminosae / Fabaceae		0,33	34,4	9	14	0,04	0,09	296,59	0,30	3,48	12,79
7202	132	1	Sukamantri	>500	Afrika	Maesopsis eminii Engl.	Rhamnaceae		0,43	18,8	12	13	0,04	0,03	116,67	0,12	1,37	5,03
7203	133	3	Sukamantri	>500	Sengon	Paraserianthes falcataria (L.) I.C	Leguminosae / Fabaceae		0,33	5,7	4	6	0,04	0,00	5,12	0,01	0,06	0,22
7204	133	3	Sukamantri	>500	Manglid	Magnolia champaca (L.) Baill. ex	Magnoliaceae		0,53	6,7	3,5	4,5	0,04	0,00	8,06	0,01	0,09	0,35
7205	133	3	Sukamantri	>500	Afrika	Maesopsis eminii Engl.	Rhamnaceae		0,43	5,1	4	4,5	0,04	0,00	4,04	0,00	0,05	0,17
7206	133	3	Sukamantri	>500	Afrika	Maesopsis eminii Engl.	Rhamnaceae		0,43	3,2	2,5	3	0,04	0,00	1,18	0,00	0,01	0,05
7207	133	3	Sukamantri	>500	Sengon	Paraserianthes falcataria (L.) I.C	Leguminosae / Fabaceae		0,33	8,6	5	6	0,04	0,01	10,77	0,01	0,13	0,46
7208	133	3	Sukamantri	>500	Afrika	Maesopsis eminii Engl.	Rhamnaceae		0,43	7,0	4	5	0,04	0,00	7,98	0,01	0,09	0,34
7209	133	3	Sukamantri	>500	Manglid	Magnolia champaca (L.) Baill. ex	Magnoliaceae		0,53	3,2	2	2,5	0,04	0,00	1,21	0,00	0,01	0,05
7210	133	3	Sukamantri	>500	Afrika	Maesopsis eminii Engl.	Rhamnaceae		0,43	5,1	4	5	0,04	0,00	4,45	0,00	0,05	0,19
7211	133	3	Sukamantri	>500	Afrika	Maesopsis eminii Engl.	Rhamnaceae		0,43	4,1	4	5	0,04	0,00	3,04	0,00	0,04	0,13
7212	133	3	Sukamantri	>500	Afrika	Maesopsis eminii Engl.	Rhamnaceae		0,43	4,5	3	4	0,04	0,00	2,84	0,00	0,03	0,12
7213	133	3	Sukamantri	>500	Afrika	Maesopsis eminii Engl.	Rhamnaceae		0,43	5,4	3	4	0,04	0,00	4,06	0,00	0,05	0,17
7214	133	3	Sukamantri	>500	Afrika	Maesopsis eminii Engl.	Rhamnaceae		0,43	3,8	3	3	0,04	0,00	1,65	0,00	0,02	0,07
7215	133	3	Sukamantri	>500	Sengon	Paraserianthes falcataria (L.) I.C	Leguminosae / Fabaceae		0,33	7,6	4	5	0,04	0,00	7,34	0,01	0,09	0,32
7216	133	3	Sukamantri	>500	Afrika	Maesopsis eminii Engl.	Rhamnaceae		0,43	4,5	3	4	0,04	0,00	2,84	0,00	0,03	0,12
7217	133	3	Sukamantri	>500	Afrika	Maesopsis eminii Engl.	Rhamnaceae		0,43	6,4	4	5	0,04	0,00	6,70	0,01	0,08	0,29
7218	133	3	Sukamantri	>500	Afrika	Maesopsis eminii Engl.	Rhamnaceae		0,43	5,4	3,5	4,5	0,04	0,00	4,52	0,00	0,05	0,19
7219	133	3	Sukamantri	>500	Afrika	Maesopsis eminii Engl.	Rhamnaceae		0,43	5,7	3	4	0,04	0,00	4,50	0,00	0,05	0,19
7220	133	3	Sukamantri	>500	Sengon	Paraserianthes falcataria (L.) I.C	Leguminosae / Fabaceae		0,33	5,7	3	4	0,04	0,00	3,53	0,00	0,04	0,15
7221	133	3	Sukamantri	>500	Afrika	Maesopsis eminii Engl.	Rhamnaceae		0,43	4,8	3,5	4	0,04	0,00	3,22	0,00	0,04	0,14
7222	133	3	Sukamantri	>500	Afrika	Maesopsis eminii Engl.	Rhamnaceae		0,43	7,0	4	5	0,04	0,00	7,98	0,01	0,09	0,34
7223	133	3	Sukamantri	>500	Afrika	Maesopsis eminii Engl.	Rhamnaceae		0,43	10,2	6	7	0,04	0,01	21,57	0,02	0,25	0,93
7224	133	3	Sukamantri	>500	Sengon	Paraserianthes falcataria (L.) I.C	Leguminosae / Fabaceae		0,33	7,3	5	6	0,04	0,00	8,03	0,01	0,09	0,35
7225	133	3	Sukamantri	>500	Sengon	Paraserianthes falcataria (L.) I.C	Leguminosae / Fabaceae		0,33	15,3	7	10	0,04	0,02	49,33	0,05	0,58	2,13
7226	133	3	Sukamantri	>500	Afrika	Maesopsis eminii Engl.	Rhamnaceae		0,43	4,5	3,5	5	0,04	0,00	3,49	0,00	0,04	0,15
7227	133	3	Sukamantri	>500	Afrika	Maesopsis eminii Engl.	Rhamnaceae		0,43	5,1	4	6	0,04	0,00	5,26	0,01	0,06	0,23
7228	133	3	Sukamantri	>500	Afrika	Maesopsis eminii Engl.	Rhamnaceae		0,43	6,4	4	6	0,04	0,00	7,92	0,01	0,09	0,34
7229	133	3	Sukamantri	>500	Sengon	Paraserianthes falcataria (L.) I.C	Leguminosae / Fabaceae		0,33	8,3	6	7	0,04	0,01	11,57	0,01	0,14	0,50
7230	133	3	Sukamantri	>500	Afrika	Maesopsis eminii Engl.	Rhamnaceae		0,43	7,6	4	5	0,04	0,00	9,36	0,01	0,11	0,40
7231	133	3	Sukamantri	>500	Afrika	Maesopsis eminii Engl.	Rhamnaceae		0,43	8,6	5	6	0,04	0,01	13,72	0,01	0,16	0,59
7232	133	3	Sukamantri	>500	Afrika	Maesopsis eminii Engl.	Rhamnaceae		0,43	5,4	4	5	0,04	0,00	4,98	0,00	0,06	0,21
7233	133	3	Sukamantri	>500	Afrika	Maesopsis eminii Engl.	Rhamnaceae		0,43	6,0	4	5	0,04	0,00	6,10	0,01	0,07	0,26
7234	133	3	Sukamantri	>500	Afrika	Maesopsis eminii Engl.	Rhamnaceae		0,43	6,7	4	5	0,04	0,00	7,33	0,01	0,09	0,32
7235	133	3	Sukamantri	>500	Afrika	Maesopsis eminii Engl.	Rhamnaceae		0,43	6,4	4	5	0,04	0,00	6,70	0,01	0,08	0,29
7236	133	3	Sukamantri	>500	Afrika	Maesopsis eminii Engl.	Rhamnaceae		0,43	6,7	5	6	0,04	0,00	8,66	0,01	0,10	0,37
7237	133	3	Sukamantri	>500	Afrika	Maesopsis eminii Engl.	Rhamnaceae		0,43	4,8	3	4	0,04	0,00	3,22	0,00	0,04	0,14
7238	133	3	Sukamantri	>500	Afrika	Maesopsis eminii Engl.	Rhamnaceae		0,43	6,4	3,5	5	0,04	0,00	6,70	0,01	0,08	0,29
7239	133	3	Sukamantri	>500	Sengon	Paraserianthes falcataria (L.) I.C	Leguminosae / Fabaceae		0,33	11,1	6	9	0,04	0,01	25,11	0,03	0,30	1,08
7240	133	3	Sukamantri	>500	Afrika	Maesopsis eminii Engl.	Rhamnaceae		0,43	7,0	4,5	5,5	0,04	0,00	8,71	0,01	0,10	0,38
7241	133	3	Sukamantri	>500	Afrika	Maesopsis eminii Engl.	Rhamnaceae		0,43	7,6	5	6	0,04	0,00	11,06	0,01	0,13	0,48
7242	133	3	Sukamantri	>500	Afrika	Maesopsis eminii Engl.	Rhamnaceae		0,43	14,6	9	11	0,04	0,02	63,45	0,06	0,75	2,74
7243	133	3	Sukamantri	>500	Afrika	Maesopsis eminii Engl.	Rhamnaceae		0,43	6,7	4	5	0,04	0,00	7,33	0,01	0,09	0,32
7244	133	3	Sukamantri	>500	Afrika	Maesopsis eminii Engl.	Rhamnaceae		0,43	7,0	5	6	0,04	0,00	9,43	0,01	0,11	0,41
7245	133	3	Sukamantri	>500	Sengon	Paraserianthes falcataria (L.) I.C	Leguminosae / Fabaceae		0,33	6,7	5	6	0,04	0,00	6,79	0,01	0,08	0,29
7246	133	3	Sukamantri	>500	Afrika	Maesopsis eminii Engl.	Rhamnaceae		0,43	6,4	4	5	0,04	0,00	6,70	0,01	0,08	0,29
7247	133	3	Sukamantri	>500	Sengon	Paraserianthes falcataria (L.) I.C	Leguminosae / Fabaceae		0,33	10,5	7	10	0,04	0,01	24,83	0,02	0,29	1,07
7248	133	3	Sukamantri	>500	Afrika	Maesopsis eminii Engl.	Rhamnaceae		0,43	5,4	4	5	0,04	0,00	4,98	0,00	0,06	0,21
7249	133	3	Sukamantri	>500	Afrika	Maesopsis eminii Engl.	Rhamnaceae		0,43	5,7	4	5	0,04	0,00	5,52	0,01	0,06	0,24
7250	133	3	Sukamantri	>500	Afrika	Maesopsis eminii Engl.	Rhamnaceae		0,43	5,1	4	5	0,04	0,00	4,45	0,00	0,05	0,19
7251	133	3	Sukamantri	>500	Sengon	Paraserianthes falcataria (L.) I.C	Leguminosae / Fabaceae		0,33	10,2	7	9	0,04	0,01	21,31	0,02	0,25	0,92
7252	133	3	Sukamantri	>500	Sengon	Paraserianthes falcataria (L.) I.C	Leguminosae / Fabaceae		0,33	3,8	6	7	0,04	0,00	2,81	0,00	0,03	0,12
7253	133	3	Sukamantri	>500	Sengon	Paraserianthes falcataria (L.) I.C	Leguminosae / Fabaceae		0,33	10,5	7	9	0,04	0,01	22,55	0,02	0,26	0,97
7254	133	3	Sukamantri	>500	Afrika	Maesopsis eminii Engl.	Rhamnaceae		0,43	6,4	4	6	0,04	0,00	7,92	0,01	0,09	0,34
7255	133	3	Sukamantri	>500	Afrika	Maesopsis eminii Engl.	Rhamnaceae		0,43	4,8	4	6	0,04	0,00	4,67	0,00	0,05	0,20
7256	133	3	Sukamantri	>500	Afrika	Maesopsis eminii Engl.	Rhamnaceae		0,43	5,1	4	5	0,04	0,00	4,45	0,00	0,05	0,19
7257	133	3	Sukamantri	>500	Afrika	Maesopsis eminii Engl.	Rhamnaceae		0,43	6,7	4,5	5	0,04	0,00	7,33	0,01	0,09	0,32
7258	133	3	Sukamantri	>500	Sengon	Paraserianthes falcataria (L.) I.C	Leguminosae / Fabaceae		0,33	6,7	5	7	0,04	0,00	7,83	0,01	0,09	0,34
7259	134	3	Sukamantri	>500	Kareumbi	Homalanthus populneus (Giesel	Euphorbiaceae		0,38	5,1	3	6	0,04	0,00	4,70	0,00	0,06	0,20
7260	134	3	Sukamantri	>500	Puspa	Schima wallichii Choisy	Theaceae		0,74	6,0	2	4	0,04	0,00	8,18	0,01	0,10	0,35
7261	134	3	Sukamantri	>500	Alpukat	Persea americana Mill.	Lauraceae		0,54	20,4	5	12	0,04	0,03	155,04	0,16	1,82	6,69
7262	134	3	Sukamantri	>500	Nangka	Artocarpus heterophyllus Lam.	Moraceae		0,61	8,0	4	6	0,04	0,00	16,42	0,02	0,19	0,71
7263	134	3	Sukamantri	>500	Afrika	Maesopsis eminii Engl.	Rhamnaceae		0,43	4,1	4	5	0,04	0,00	3,04	0,00	0,04	0,13
7264	134	3	Sukamantri	>500	Afrika	Maesopsis eminii Engl.	Rhamnaceae		0,43	4,8	5	6	0,04	0,00	4,67	0,00	0,05	0,20
7265	134	3	Sukamantri	>500	Afrika	Maesopsis eminii Engl.	Rhamnaceae		0,43	12,4	8	9	0,04	0,01	39,02	0,04	0,46	1,68
7266	134	3	Sukamantri	>500	Mahoni	Swietenia macrophylla King	Meliaceae		0,61	20,4	5	9	0,04	0,03	133,20	0,13	1,57	5,74
7267	134	3	Sukamantri	>500	Huru Kacang	Neolitsea triplinervia Merr.	Lauraceae		0,56	4,8	3	6	0,04	0,00	5,95	0,01	0,07	0,26
7268	134	3	Sukamantri	>500	Renggang	Sp4		0	0,5	3,8	1,8	3	0,04	0,00	1,89	0,00	0,02	0,08
7269	134	3	Sukamantri	>500	Puspa	Schima wallichii Choisy	Theaceae		0,74	6,4	2	5	0,04	0,00	11,02	0,01	0,13	0,48
7270	134	3	Sukamantri	>500	Nangka	Artocarpus heterophyllus Lam.	Moraceae		0,61	14,0	2,5	5	0,04	0,02	39,13	0,04	0,46	1,69
7271	134	3	Sukamantri	>500	Alpukat	Persea americana Mill.	Lauraceae		0,54	16,9	8	11	0,04	0,02	101,34	0,10	1,19	4,37
7272	134	3	Sukamantri	>500	Huru Kacang	Neolitsea triplinervia Merr.	Lauraceae		0,56	12,1	9	11	0,04	0,01	56,96	0,06	0,67	2,46
7273	134	3	Sukamantri	>500	Nangka	Artocarpus heterophyllus Lam.	Moraceae		0,61	16,2	4	6	0,04	0,02	60,61	0,06	0,71	2,61
7274	134	3	Sukamantri	>500	Afrika	Maesopsis eminii Engl.	Rhamnaceae		0,43	10,2	7	8	0,04	0,01	24,38	0,02	0,29	1,05
7275	134	3	Sukamantri	>500	Huru Kacang	Neolitsea triplinervia Merr.	Lauraceae		0,56	10,2	8	9	0,04	0,01	34,59	0,03	0,41	1,49
7276	134	3	Sukamantri	>500	Huru Kacang	Neolitsea triplinervia Merr.	Lauraceae		0,56	4,1	4	5	0,04	0,00	3,88	0,00	0,05	0,17
7277	134	3	Sukamantri	>500	Puspa	Schima wallichii Choisy	Theaceae		0,74	6,0	3	5	0,04	0,00	10,03	0,01	0,12	0,43
7278	134	3	Sukamantri	>500	Kiceuhai	Turpinia sphaerocarpa Hassk.	Staphyleaceae		0,46	10,5	3,5	7	0,04	0,01	24,28	0,02	0,29	1,05
7279	134	3	Sukamantri	>500	Kiceuhai	Turpinia sphaerocarpa Hassk.	Staphyleaceae		0,46	8,6	3	5	0,04	0,01	12,35	0,01	0,15	0,53
7280	134	3	Sukamantri	>500	Mareme	Glochidion borneense (Müll. Arg.	Phyllanthaceae		0,46	4,5	2	4	0,04	0,00	3,02	0,00	0,04	0,13
7281	134	3	Sukamantri	>500	Puspa	Schima wallichii Choisy	Theaceae		0,74	7,0	3,5	7	0,04	0,00	17,86	0,02	0,21	0,77
7282	134	3	Sukamantri	>500	Afrika	Maesopsis eminii Engl.	Rhamnaceae		0,43	9,5	9	10	0,04	0,01	26,57	0,03	0,31	1,15
7283	134	3	Sukamantri	>500	Afrika	Maesopsis eminii Engl.	Rhamnaceae		0,43	10,8	9	10	0,04	0,01	33,42	0,03	0,39	1,44
7284	134	3	Sukamantri	>500	Afrika	Maesopsis eminii Engl.	Rhamnaceae		0,43	9,2	8	9	0,04	0,01	22,68	0,02	0,27	0,98
7285	134	3	Sukamantri	>500	Afrika	Maesopsis eminii Engl.	Rhamnaceae		0,43	6,7	6	7	0,04	0,00	9,97	0,01	0,12	0,43
7286	134	3	Sukamantri	>500	Cengkeh	Syzygium aromaticum (L.) Merr. &	Myrtaceae		0,79	21,3	6	10	0,04	0,04	202,17	0,20	2,38	8,72
7287	134	3	Sukamantri	>500	Tisuk	Hibiscus macrophyllus Roxb. ex	Malvaceae		0,4	8,6	3	7	0,04	0,01	14,79	0,01	0,17	0,64
7288	134	3	Sukamantri	>500	Kihaji	Dysoxylum macrocarpum Blume	Meliaceae		0,73	5,4	3	5	0,04	0,00	8,08	0,01	0,09	0,35
7289	134	3	Sukamantri	>500	Alpukat	Persea americana Mill.	Lauraceae		0,54	8,6	4	8	0,04	0,01	22,00	0,02	0,26	0,95
7290	134	3	Sukamantri	>500	Alpukat	Persea americana Mill.	Lauraceae		0,54	12,1	8	10	0,04	0,01	50,48	0,05	0,59	2,18
7291	134	3	Sukamantri	>500	Alpukat	Persea americana Mill.	Lauraceae		0,54	15,0	6	9	0,04	0,02	67,66	0,07	0,80	2,92
7292	134	3	Sukamantri	>500	Alpukat	Persea americana Mill.	Lauraceae		0,54	8,9	5	7	0,04	0,01	20,81	0,02	0,24	0,90
7293	134	3	Sukamantri	>500	Mahoni	Swietenia macrophylla King	Meliaceae		0,61	14,0	7	10	0,04	0,02	73,84	0,07	0,87	3,18
7294	134	3	Sukamantri	>500	Kanyere	Bridelia stipularis (L.) Blume	Phyllanthaceae		0,44	13,7	6	11	0,04	0,01	57,27	0,06	0,67	2,47
7295	134	3	Sukamantri	>500	Kihaji	Dysoxylum macrocarpum Blume	Meliaceae		0,73	7,3	4	7	0,04	0,00	19,13	0,02	0,22	0,82
7296	134	3	Sukamantri	>500	Kihaji	Dysoxylum macrocarpum Blume	Meliaceae		0,73	8,6	4	7	0,04	0,01	25,66	0,03	0,30	1,11
7297	134	3	Sukamantri	>500	Petai	Parkia speciosa Hassk.	Leguminosae / Fabaceae		0,45	5,4	4	5	0,04	0,00	5,19	0,01	0,06	0,22
7298	134	3	Sukamantri	>500	Kihaji	Dysoxylum macrocarpum Blume	Meliaceae		0,73	7,6	4	6	0,04	0,00	17,96	0,02	0,21	0,77
7299	134	3	Sukamantri	>500	Sengon	Paraserianthes falcataria (L.) I.C	Leguminosae / Fabaceae		0,33	3,5	4	5	0,04	0,00	1,76	0,00	0,02	0,08
7300	134	3	Sukamantri	>500	Afrika	Maesopsis eminii Engl.	Rhamnaceae		0,43	10,8	8	9	0,04	0,01	30,35	0,03	0,36	1,31
7301	134	3	Sukamantri	>500	Nangka	Artocarpus heterophyllus Lam.	Moraceae		0,61	14,6	6	9	0,04	0,02	72,73	0,07	0,85	3,14
7302	135	2	Sukamantri	>500	Afrika	Maesopsis eminii Engl.	Rhamnaceae		0,43	8,9	7	8	0,04	0,01	19,09	0,02	0,22	0,82
7303	135	2	Sukamantri	>500	Manglid	Magnolia champaca (L.) Baill. ex	Magnoliaceae		0,53	3,5	3	4	0,04	0,00	2,21	0,00	0,03	0,10
7304	135	2	Sukamantri	>500	Afrika	Maesopsis eminii Engl.	Rhamnaceae		0,43	11,1	9	10	0,04	0,01	35,25	0,04	0,41	1,52
7305	135	2	Sukamantri	>500	Afrika	Maesopsis eminii Engl.	Rhamnaceae		0,43	9,9	9	10	0,04	0,01	28,22	0,03	0,33	1,22
7306	135	2	Sukamantri	>500	Tisuk	Hibiscus macrophyllus Roxb. ex	Malvaceae		0,4	13,4	7	10	0,04	0,01	46,07	0,05	0,54	1,99
7307	135	2	Sukamantri	>500	Nangka	Artocarpus heterophyllus Lam.	Moraceae		0,61	9,5	5	6	0,04	0,01	22,93	0,02	0,27	0,99
7308	135	2	Sukamantri	>500	Kelapa	Cocos nucifera L.	Arecaceae		0,5	25,5	8	11	0,04	0,05	89,20	0,09	1,05	3,85
7309	135	2	Sukamantri	>500	Tisuk	Hibiscus macrophyllus Roxb. ex	Malvaceae		0,4	7,0	4	6	0,04	0,00	8,82	0,01	0,10	0,38
7310	135	2	Sukamantri	>500	Alpukat	Persea americana Mill.	Lauraceae		0,54	5,4	3	4	0,04	0,00	5,00	0,00	0,06	0,22

7311	135	2	Sukamantri	>500	Kelapa	Cocos nucifera L.	Arecaceae	0,5	34,0	5	8	0,04	0,09	66,10	0,07	0,78	2,85
7312	135	2	Sukamantri	>500	Alpukat	Persea americana Mill.	Lauraceae	0,54	9,2	4	5	0,04	0,01	16,31	0,02	0,19	0,70
7313	135	2	Sukamantri	>500	Afrika	Maesopsis eminii Engl.	Rhamnaceae	0,43	8,9	6	8	0,04	0,01	19,09	0,02	0,22	0,82
7314	135	2	Sukamantri	>500	Afrika	Maesopsis eminii Engl.	Rhamnaceae	0,43	3,2	3	4	0,04	0,00	1,53	0,00	0,02	0,07
7315	135	2	Sukamantri	>500	Afrika	Maesopsis eminii Engl.	Rhamnaceae	0,43	11,5	8	9	0,04	0,01	33,70	0,03	0,40	1,45
7316	135	2	Sukamantri	>500	Afrika	Maesopsis eminii Engl.	Rhamnaceae	0,43	16,9	9	10	0,04	0,02	75,38	0,08	0,89	3,25
7317	135	2	Sukamantri	>500	Afrika	Maesopsis eminii Engl.	Rhamnaceae	0,43	7,3	7	8	0,04	0,00	13,31	0,01	0,16	0,57
7318	135	2	Sukamantri	>500	Afrika	Maesopsis eminii Engl.	Rhamnaceae	0,43	4,1	3	4	0,04	0,00	2,48	0,00	0,03	0,11
7319	135	2	Sukamantri	>500	Afrika	Maesopsis eminii Engl.	Rhamnaceae	0,43	11,1	8	10	0,04	0,01	35,25	0,04	0,41	1,52
7320	135	2	Sukamantri	>500	Afrika	Maesopsis eminii Engl.	Rhamnaceae	0,43	9,5	7	8	0,04	0,01	21,66	0,02	0,25	0,93
7321	135	2	Sukamantri	>500	Afrika	Maesopsis eminii Engl.	Rhamnaceae	0,43	9,5	7,5	9	0,04	0,01	24,13	0,02	0,28	1,04
7322	135	2	Sukamantri	>500	Mahoni	Swietenia macrophylla King	Meliaceae	0,61	9,9	7	9	0,04	0,01	35,30	0,04	0,41	1,52
7323	135	2	Sukamantri	>500	Kelapa	Cocos nucifera L.	Arecaceae	0,5	25,5	2	5	0,04	0,05	43,00	0,04	0,51	1,85
7324	135	2	Sukamantri	>500	Tisuk	Hibiscus macrophyllus Roxb. ex I	Malvaceae	0,4	3,8	3	4	0,04	0,00	2,01	0,00	0,02	0,09
7325	135	2	Sukamantri	>500	Limus	Mangifera foetida Lour.	Anacardiaceae	0,73	4,1	3	4	0,04	0,00	4,03	0,00	0,05	0,17
7326	135	2	Sukamantri	>500	Afrika	Maesopsis eminii Engl.	Rhamnaceae	0,43	8,6	7	8	0,04	0,01	17,86	0,02	0,21	0,77
7327	135	2	Sukamantri	>500	Afrika	Maesopsis eminii Engl.	Rhamnaceae	0,43	4,1	6	6,5	0,04	0,00	3,87	0,00	0,05	0,17
7328	135	2	Sukamantri	>500	Cengkeh	Syzygium aromaticum (L.) Merr. a	Myrtaceae	0,79	12,4	6	9	0,04	0,01	68,12	0,07	0,80	2,94
7329	135	2	Sukamantri	>500	Afrika	Maesopsis eminii Engl.	Rhamnaceae	0,43	8,9	7	8	0,04	0,01	19,09	0,02	0,22	0,82
7330	135	2	Sukamantri	>500	Afrika	Maesopsis eminii Engl.	Rhamnaceae	0,43	5,7	7	8	0,04	0,00	8,50	0,01	0,10	0,37
7331	135	2	Sukamantri	>500	Afrika	Maesopsis eminii Engl.	Rhamnaceae	0,43	8,9	7	8	0,04	0,01	19,09	0,02	0,22	0,82
7332	135	2	Sukamantri	>500	Petai	Parkia speciosa Hassk.	Leguminosae / Fabaceae	0,45	12,1	6	9	0,04	0,01	38,79	0,04	0,46	1,67
7333	135	2	Sukamantri	>500	Cengkeh	Syzygium aromaticum (L.) Merr. a	Myrtaceae	0,79	13,4	6	8	0,04	0,01	70,04	0,07	0,82	3,02
7334	135	2	Sukamantri	>500	Afrika	Maesopsis eminii Engl.	Rhamnaceae	0,43	16,9	6	7	0,04	0,02	54,37	0,05	0,64	2,34
7335	135	2	Sukamantri	>500	Afrika	Maesopsis eminii Engl.	Rhamnaceae	0,43	6,0	3	5	0,04	0,00	6,10	0,01	0,07	0,26
7336	135	2	Sukamantri	>500	Tisuk	Hibiscus macrophyllus Roxb. ex I	Malvaceae	0,4	3,2	3,5	4	0,04	0,00	1,44	0,00	0,02	0,06
7337	135	2	Sukamantri	>500	Afrika	Maesopsis eminii Engl.	Rhamnaceae	0,43	5,4	4	5	0,04	0,00	4,98	0,00	0,06	0,21
7338	135	2	Sukamantri	>500	Afrika	Maesopsis eminii Engl.	Rhamnaceae	0,43	8,6	6	7	0,04	0,01	15,80	0,02	0,19	0,68
7339	135	2	Sukamantri	>500	Alpukat	Persea americana Mill.	Lauraceae	0,54	4,1	3	4	0,04	0,00	3,06	0,00	0,04	0,13
7340	135	2	Sukamantri	>500	Afrika	Maesopsis eminii Engl.	Rhamnaceae	0,43	10,8	6	7	0,04	0,01	24,11	0,02	0,28	1,04
7341	135	2	Sukamantri	>500	Afrika	Maesopsis eminii Engl.	Rhamnaceae	0,43	4,5	4	5	0,04	0,00	3,49	0,00	0,04	0,15
7342	135	2	Sukamantri	>500	Sengon	Paraserianthes falcataria (L.) I.C	Leguminosae / Fabaceae	0,33	11,8	8	9	0,04	0,01	27,80	0,03	0,33	1,20
7343	135	2	Sukamantri	>500	Nangka	Artocarpus heterophyllus Lam.	Moraceae	0,61	5,4	2	4	0,04	0,00	5,59	0,01	0,07	0,24
7344	135	2	Sukamantri	>500	Afrika	Maesopsis eminii Engl.	Rhamnaceae	0,43	10,5	7	8	0,04	0,01	25,79	0,03	0,30	1,11
7345	135	2	Sukamantri	>500	Afrika	Maesopsis eminii Engl.	Rhamnaceae	0,43	6,0	5	6	0,04	0,00	7,21	0,01	0,08	0,31
7346	135	2	Sukamantri	>500	Sengon	Paraserianthes falcataria (L.) I.C	Leguminosae / Fabaceae	0,33	4,1	4	5	0,04	0,00	2,39	0,00	0,03	0,10
7347	135	2	Sukamantri	>500	Sengon	Paraserianthes falcataria (L.) I.C	Leguminosae / Fabaceae	0,33	13,7	5	9	0,04	0,01	36,62	0,04	0,43	1,58
7348	135	2	Sukamantri	>500	Sengon	Paraserianthes falcataria (L.) I.C	Leguminosae / Fabaceae	0,33	5,7	5	6	0,04	0,00	5,12	0,01	0,06	0,22
7349	135	2	Sukamantri	>500	Sengon	Paraserianthes falcataria (L.) I.C	Leguminosae / Fabaceae	0,33	6,0	4	5	0,04	0,00	4,79	0,00	0,06	0,21
7350	135	2	Sukamantri	>500	Afrika	Maesopsis eminii Engl.	Rhamnaceae	0,43	4,5	3	4,5	0,04	0,00	3,17	0,00	0,04	0,14
7351	135	2	Sukamantri	>500	Afrika	Maesopsis eminii Engl.	Rhamnaceae	0,43	3,8	3	4	0,04	0,00	2,14	0,00	0,03	0,09
7352	135	2	Sukamantri	>500	Afrika	Maesopsis eminii Engl.	Rhamnaceae	0,43	7,0	3,5	6	0,04	0,00	9,43	0,01	0,11	0,41
7353	135	2	Sukamantri	>500	Afrika	Maesopsis eminii Engl.	Rhamnaceae	0,43	5,4	4	6	0,04	0,00	5,88	0,01	0,07	0,25
7354	135	2	Sukamantri	>500	Afrika	Maesopsis eminii Engl.	Rhamnaceae	0,43	6,7	4	5,5	0,04	0,00	8,00	0,01	0,09	0,34
7355	135	2	Sukamantri	>500	Afrika	Maesopsis eminii Engl.	Rhamnaceae	0,43	5,4	3	5	0,04	0,00	4,98	0,00	0,06	0,21
7356	135	2	Sukamantri	>500	Afrika	Maesopsis eminii Engl.	Rhamnaceae	0,43	3,2	3	4	0,04	0,00	1,53	0,00	0,02	0,07
7357	135	2	Sukamantri	>500	Afrika	Maesopsis eminii Engl.	Rhamnaceae	0,43	6,7	3,5	5	0,04	0,00	7,33	0,01	0,09	0,32
7358	135	2	Sukamantri	>500	Afrika	Maesopsis eminii Engl.	Rhamnaceae	0,43	4,8	3	4	0,04	0,00	3,22	0,00	0,04	0,14
7359	135	2	Sukamantri	>500	Sengon	Paraserianthes falcataria (L.) I.C	Leguminosae / Fabaceae	0,33	7,6	8	9	0,04	0,00	12,58	0,01	0,15	0,54
7360	135	2	Sukamantri	>500	Afrika	Maesopsis eminii Engl.	Rhamnaceae	0,43	6,4	4	5,5	0,04	0,00	7,31	0,01	0,09	0,32
7361	135	2	Sukamantri	>500	Afrika	Maesopsis eminii Engl.	Rhamnaceae	0,43	3,2	3	4	0,04	0,00	1,53	0,00	0,02	0,07
7362	135	2	Sukamantri	>500	Afrika	Maesopsis eminii Engl.	Rhamnaceae	0,43	4,1	3	4	0,04	0,00	2,48	0,00	0,03	0,11
7363	135	2	Sukamantri	>500	Afrika	Maesopsis eminii Engl.	Rhamnaceae	0,43	6,0	3,5	5	0,04	0,00	6,10	0,01	0,07	0,26
7364	135	2	Sukamantri	>500	Sengon	Paraserianthes falcataria (L.) I.C	Leguminosae / Fabaceae	0,33	3,8	3	5	0,04	0,00	2,06	0,00	0,02	0,09
7365	135	2	Sukamantri	>500	Afrika	Maesopsis eminii Engl.	Rhamnaceae	0,43	24,5	12	14	0,04	0,05	203,37	0,20	2,39	8,77
7366	136	2	Sukamantri	>500	Tangkalak/Huru tangkalak	Litsea glutinosa (Lour.) C.B.Rob.	Lauraceae	0,52	13,0	9	12	0,01	0,00	66,24	0,07	3,11	11,43
7367	136	2	Sukamantri	>500	Afrika	Maesopsis eminii Engl.	Rhamnaceae	0,43	12,7	11	12	0,01	0,00	53,20	0,05	2,50	9,18
7368	136	2	Sukamantri	>500	Puspa	Schima wallichii Choisy	Theaceae	0,74	11,8	7	11	0,01	0,00	70,02	0,07	3,29	12,08
7369	136	2	Sukamantri	>500	Puspa	Schima wallichii Choisy	Theaceae	0,74	3,2	2	3	0,01	0,00	1,94	0,00	0,09	0,33
7370	136	2	Sukamantri	>500	Afrika	Maesopsis eminii Engl.	Rhamnaceae	0,43	23,2	12	14	0,01	0,01	184,43	0,18	8,67	31,81
7371	136	2	Sukamantri	>500	Afrika	Maesopsis eminii Engl.	Rhamnaceae	0,43	20,7	12	14	0,01	0,01	149,10	0,15	7,01	25,72
7372	136	2	Sukamantri	>500	Afrika	Maesopsis eminii Engl.	Rhamnaceae	0,43	7,6	9	10	0,01	0,00	17,66	0,02	0,83	3,05
7373	136	2	Sukamantri	>500	Afrika	Maesopsis eminii Engl.	Rhamnaceae	0,43	4,5	7	8	0,01	0,00	5,36	0,01	0,25	0,92
7374	136	2	Sukamantri	>500	Afrika	Maesopsis eminii Engl.	Rhamnaceae	0,43	4,5	7	8	0,01	0,00	5,36	0,01	0,25	0,92
7375	136	2	Sukamantri	>500	Tisuk	Hibiscus macrophyllus Roxb. ex I	Malvaceae	0,4	23,2	3	4	0,01	0,01	54,79	0,05	2,58	9,45
7376	136	2	Sukamantri	>500	Puspa	Schima wallichii Choisy	Theaceae	0,74	9,2	5	6	0,01	0,00	25,72	0,03	1,21	4,44
7377	136	2	Sukamantri	>500	Tangkalak/Huru tangkalak	Litsea glutinosa (Lour.) C.B.Rob.	Lauraceae	0,52	22,3	7	11	0,01	0,01	162,97	0,16	7,66	28,11
7378	136	2	Sukamantri	>500	Afrika	Maesopsis eminii Engl.	Rhamnaceae	0,43	4,1	14	5,5	0,01	0,00	3,32	0,00	0,16	0,57
7379	136	2	Sukamantri	>500	Afrika	Maesopsis eminii Engl.	Rhamnaceae	0,43	8,0	10	11	0,01	0,00	20,76	0,02	0,98	3,58
7380	136	2	Sukamantri	>500	Afrika	Maesopsis eminii Engl.	Rhamnaceae	0,43	5,7	7	8	0,01	0,00	8,50	0,01	0,40	1,47
7381	136	2	Sukamantri	>500	Afrika	Maesopsis eminii Engl.	Rhamnaceae	0,43	7,6	9	10	0,01	0,00	17,66	0,02	0,83	3,05
7382	136	2	Sukamantri	>500	Afrika	Maesopsis eminii Engl.	Rhamnaceae	0,43	6,0	7	8	0,01	0,00	9,38	0,01	0,44	1,62
7383	136	2	Sukamantri	>500	Afrika	Maesopsis eminii Engl.	Rhamnaceae	0,43	4,8	5	7	0,01	0,00	5,38	0,01	0,25	0,93
7384	136	2	Sukamantri	>500	Afrika	Maesopsis eminii Engl.	Rhamnaceae	0,43	5,1	4	5	0,01	0,00	4,45	0,00	0,21	0,77
7385	136	2	Sukamantri	>500	Afrika	Maesopsis eminii Engl.	Rhamnaceae	0,43	4,1	4	5	0,01	0,00	3,04	0,00	0,14	0,52
7386	136	2	Sukamantri	>500	Puspa	Schima wallichii Choisy	Theaceae	0,74	11,1	5	9	0,01	0,00	52,62	0,05	2,47	9,08
7387	136	2	Sukamantri	>500	Afrika	Maesopsis eminii Engl.	Rhamnaceae	0,43	15,6	12	14	0,01	0,00	88,85	0,09	4,18	15,33
7388	136	2	Sukamantri	>500	Afrika	Maesopsis eminii Engl.	Rhamnaceae	0,43	6,0	8	9	0,01	0,00	10,45	0,01	0,49	1,80
7389	136	2	Sukamantri	>500	Afrika	Maesopsis eminii Engl.	Rhamnaceae	0,43	5,4	8	9	0,01	0,00	8,52	0,01	0,40	1,47
7390	136	2	Sukamantri	>500	Afrika	Maesopsis eminii Engl.	Rhamnaceae	0,43	15,0	11	12	0,01	0,00	71,48	0,07	3,36	12,33
7391	136	2	Sukamantri	>500	Puspa	Schima wallichii Choisy	Theaceae	0,74	8,6	7	10	0,01	0,00	36,02	0,04	1,69	6,21
7392	136	2	Sukamantri	>500	Puspa	Schima wallichii Choisy	Theaceae	0,74	5,4	3	7	0,01	0,00	11,13	0,01	0,52	1,92
7393	136	2	Sukamantri	>500	Puspa	Schima wallichii Choisy	Theaceae	0,74	17,8	6	10	0,01	0,01	137,09	0,14	6,44	23,65
7394	136	2	Sukamantri	>500	Tisuk	Hibiscus macrophyllus Roxb. ex I	Malvaceae	0,4	3,8	5	6	0,01	0,00	2,91	0,00	0,14	0,50
7395	136	2	Sukamantri	>500	Afrika	Maesopsis eminii Engl.	Rhamnaceae	0,43	7,6	7	8	0,01	0,00	14,39	0,01	0,68	2,48
7396	136	2	Sukamantri	>500	Afrika	Maesopsis eminii Engl.	Rhamnaceae	0,43	4,5	6	7	0,01	0,00	4,74	0,00	0,22	0,82
7397	136	2	Sukamantri	>500	Puspa	Schima wallichii Choisy	Theaceae	0,74	5,4	5	7	0,01	0,00	11,13	0,01	0,52	1,92
7398	136	2	Sukamantri	>500	Afrika	Maesopsis eminii Engl.	Rhamnaceae	0,43	20,0	12	14	0,04	0,03	140,81	0,14	1,65	6,07
7399	136	2	Sukamantri	>500	Afrika	Maesopsis eminii Engl.	Rhamnaceae	0,43	22,6	11	13	0,04	0,04	163,78	0,16	1,92	7,06
7400	136	2	Sukamantri	>500	Sengon	Paraserianthes falcataria (L.) I.C	Leguminosae / Fabaceae	0,33	21,6	12	15	0,04	0,04	135,37	0,14	1,59	5,84
7401	136	2	Sukamantri	>500	Sengon	Paraserianthes falcataria (L.) I.C	Leguminosae / Fabaceae	0,33	25,5	14	18	0,04	0,05	215,46	0,22	2,53	9,29
7402	136	2	Sukamantri	>500	Cengkeh	Syzygium aromaticum (L.) Merr. a	Myrtaceae	0,79	25,1	7	12	0,04	0,05	323,09	0,32	3,80	13,93
7403	136	2	Sukamantri	>500	Cengkeh	Syzygium aromaticum (L.) Merr. a	Myrtaceae	0,79	21,3	12	14	0,04	0,04	275,15	0,28	3,23	11,87
7404	136	2	Sukamantri	>500	Afrika	Maesopsis eminii Engl.	Rhamnaceae	0,43	11,5	10	11	0,04	0,01	40,50	0,04	0,48	1,75
7405	136	2	Sukamantri	>500	Afrika	Maesopsis eminii Engl.	Rhamnaceae	0,43	12,1	10	11	0,04	0,01	44,72	0,04	0,53	1,93
7406	136	2	Sukamantri	>500	Afrika	Maesopsis eminii Engl.	Rhamnaceae	0,43	12,4	10	11	0,04	0,01	46,89	0,05	0,55	2,02
7407	136	2	Sukamantri	>500	Afrika	Maesopsis eminii Engl.	Rhamnaceae	0,43	17,2	10	11	0,04	0,02	85,12	0,09	1,00	3,67
7408	136	2	Sukamantri	>500	Afrika	Maesopsis eminii Engl.	Rhamnaceae	0,43	10,8	9	10	0,04	0,01	33,42	0,03	0,39	1,44
7409	136	2	Sukamantri	>500	Afrika	Maesopsis eminii Engl.	Rhamnaceae	0,43	18,8	11	12	0,04	0,03	108,42	0,11	1,27	4,68
7410	136	2	Sukamantri	>500	Afrika	Maesopsis eminii Engl.	Rhamnaceae	0,43	12,4	11	12	0,04	0,01	50,79	0,05	0,60	2,19
7411	136	2	Sukamantri	>500	Afrika	Maesopsis eminii Engl.	Rhamnaceae	0,43	11,1	10	10	0,04	0,01	35,25	0,04	0,41	1,52
7412	136	2	Sukamantri	>500	Afrika	Maesopsis eminii Engl.	Rhamnaceae	0,43	12,1	10	11	0,04	0,01	44,72	0,04	0,53	1,93
7413	136	2	Sukamantri	>500	Afrika	Maesopsis eminii Engl.	Rhamnaceae	0,43	11,5	10	11	0,04	0,01	40,50	0,04	0,48	1,75
7414	136	2	Sukamantri	>500	Afrika	Maesopsis eminii Engl.	Rhamnaceae	0,43	14,3	10	11	0,04	0,02	60,95	0,06	0,72	2,63
7415	136	2	Sukamantri	>500	Afrika	Maesopsis eminii Engl.	Rhamnaceae	0,43	14,0	11	12	0,04	0,02	63,34	0,06	0,74	2,73
7416	136	2	Sukamantri	>500	Afrika	Maesopsis eminii Engl.	Rhamnaceae	0,43	13,7	10	11	0,04	0,01	56,08	0,06	0,66	2,42
7417	136	2	Sukamantri	>500	Ganitri	Elaeocarpus ganitrus Roxb. ex G.	Elaeocarpaceae	0,49	17,8	11	17	0,04	0,02	152,80	0,15	1,80	6,59
7418	136	2	Sukamantri	>500	Ganitri	Elaeocarpus ganitrus Roxb. ex G.	Elaeocarpaceae	0,49	10,8	8	11	0,04	0,01	41,11	0,04	0,48	1,77
7419	136	2	Sukamantri	>500	Afrika	Maesopsis eminii Engl.	Rhamnaceae	0,43	10,8	10	11	0,04	0,01	36,47	0,04	0,43	1,57
7420	136	2	Sukamantri	>500	Afrika	Maesopsis eminii Engl.	Rhamnaceae	0,43	13,4	9	10	0,04	0,01	49,22	0,05	0,58	2,12
7421	136	2	Sukamantri	>500	Puspa	Schima wallichii Choisy	Theaceae	0,74	10,5	4	10	0,04	0,01	52,03	0,05	0,61	2,24
7422	136	2	Sukamantri	>500	Afrika	Maesopsis eminii Engl.	Rhamnaceae	0,43	15,3	10	11	0,04	0,02	68,60	0,07	0,81	2,96
7423	136	2	Sukamantri	>500	Afrika	Maesopsis eminii Engl.	Rhamnaceae	0,43	10,8	10	11	0,04	0,01	36,47	0,04	0,43	1,57
7424	136	2	Sukamantri	>500	Afrika	Maesopsis eminii Engl.	Rhamnaceae	0,43	16,9	10	11	0,04	0,02	82,26	0,08	0,97	3,55
7425	136	2	Sukamantri	>500	Mahoni	Swietenia macrophylla King	Meliaceae	0,61	12,7	4	9	0,04	0,01	56,30	0,06	0,66	2,43
7426	136	2	Sukamantri	>500	Cengkeh	Syzygium aromaticum (L.) Merr. a	Myrtaceae	0,79	10,5	6	8	0,04	0,01	45,03	0,05	0,53	1,94
7427	136	2	Sukamantri	>500	Afrika	Maesopsis eminii Engl.	Rhamnaceae	0,43	14,6	9	11	0,04	0,02	63,45	0,06	0,75	2,74
7428	137	3	Sukamantri	>500	Ganitri	Elaeocarpus ganitrus Roxb. ex G.	Elaeocarpaceae	0,49	5,1	6	8	0,01	0,00	7,72	0,01	0,36	1,33
7429	137	3	Sukamantri	>500	Ganitri	Elaeocarpus ganitrus Roxb. ex G.	Elaeocarpaceae	0,49	11,5	11	14	0,01	0,00	56,93	0,06	2,68	9,82
7430	137	3	Sukamantri	>500	Ganitri	Elaeocarpus ganitrus Roxb. ex G.	Elaeocarpaceae	0,49	10,2	10	12	0,01	0,00	39,84	0,04	1,87	6,87
7431	137	3	Sukamantri	>500	Ganitri	Elaeocarpus ganitrus Roxb. ex G.	Elaeocarpaceae	0,49	2,5	3	3,5	0,01	0,00	1,02	0,00	0,05	0,18
7432	137	3	Sukamantri	>500	Ganitri	Elaeocarpus ganitrus Roxb. ex G.	Elaeocarpaceae	0,49	7,0	9	11	0,01	0,00	18,52	0,02	0,87	3,19
7433	137	3	Sukamantri	>500	Ganitri	Elaeocarpus ganitrus Roxb. ex G.	Elaeocarpaceae	0,49	6,7	6	9	0,01	0,00	14,15	0,01	0,67	2,44
7434	137	3	Sukamantri	>500	Sengon	Paraserianthes falcataria (L.) I.C	Leguminosae / Fabaceae	0,33	5,4	10	11	0,01	0,00	8,04	0,01	0,38	1,39

7435	137	3	Sukamantri	>500	Ganitri	Elaeocarpus ganitrus Roxb. ex G.	Elaeocarpaceae	0,49	11,8	11	14	0,01	0,00	59,86	0,06	2,81	10,33
7436	137	3	Sukamantri	>500	Ganitri	Elaeocarpus ganitrus Roxb. ex G.	Elaeocarpaceae	0,49	5,1	9	11	0,01	0,00	10,33	0,01	0,49	1,78
7437	137	3	Sukamantri	>500	Nangka	Artocarpus heterophyllus Lam.	Moraceae	0,61	8,6	3	5	0,01	0,00	16,00	0,02	0,75	2,76
7438	137	3	Sukamantri	>500	Ganitri	Elaeocarpus ganitrus Roxb. ex G.	Elaeocarpaceae	0,49	3,2	4	5	0,01	0,00	2,12	0,00	0,10	0,37
7439	137	3	Sukamantri	>500	Ganitri	Elaeocarpus ganitrus Roxb. ex G.	Elaeocarpaceae	0,49	9,2	9	13	0,01	0,00	35,80	0,04	1,68	6,17
7440	137	3	Sukamantri	>500	Ganitri	Elaeocarpus ganitrus Roxb. ex G.	Elaeocarpaceae	0,49	4,5	7	9	0,01	0,00	6,73	0,01	0,32	1,16
7441	137	3	Sukamantri	>500	Ganitri	Elaeocarpus ganitrus Roxb. ex G.	Elaeocarpaceae	0,49	10,5	10	14	0,01	0,00	48,54	0,05	2,28	8,37
7442	137	3	Sukamantri	>500	Ganitri	Elaeocarpus ganitrus Roxb. ex G.	Elaeocarpaceae	0,49	12,7	9	13	0,01	0,00	64,52	0,06	3,03	11,13
7443	137	3	Sukamantri	>500	Benda/teureup	Artocarpus elasticus Reinw. ex B	Moraceae	0,37	21,3	7	14	0,01	0,01	137,35	0,14	6,46	23,69
7444	137	3	Sukamantri	>500	Afrika	Maesopsis eminii Engl.	Rhamnaceae	0,43	17,8	15	17	0,01	0,01	135,57	0,14	6,37	23,38
7445	137	3	Sukamantri	>500	Afrika	Maesopsis eminii Engl.	Rhamnaceae	0,43	24,2	16	19	0,01	0,01	262,64	0,26	12,34	45,30
7446	137	3	Sukamantri	>500	Afrika	Maesopsis eminii Engl.	Rhamnaceae	0,43	21,6	14	16	0,01	0,01	183,02	0,18	8,60	31,57
7447	137	3	Sukamantri	>500	Ganitri	Elaeocarpus ganitrus Roxb. ex G.	Elaeocarpaceae	0,49	5,1	8	9	0,01	0,00	8,60	0,01	0,40	1,48
7448	137	3	Sukamantri	>500	Ganitri	Elaeocarpus ganitrus Roxb. ex G.	Elaeocarpaceae	0,49	13,0	12	15	0,01	0,00	76,96	0,08	3,62	13,27
7449	137	3	Sukamantri	>500	Ganitri	Elaeocarpus ganitrus Roxb. ex G.	Elaeocarpaceae	0,49	5,1	9	11	0,01	0,00	10,33	0,01	0,49	1,78
7450	137	3	Sukamantri	>500	Afrika	Maesopsis eminii Engl.	Rhamnaceae	0,43	22,9	15	18	0,01	0,01	226,38	0,23	10,64	39,05
7451	137	3	Sukamantri	>500	Ganitri	Elaeocarpus ganitrus Roxb. ex G.	Elaeocarpaceae	0,49	8,0	11	13	0,01	0,00	27,27	0,03	1,28	4,70
7452	137	3	Sukamantri	>500	Ganitri	Elaeocarpus ganitrus Roxb. ex G.	Elaeocarpaceae	0,49	6,4	11	14	0,01	0,00	19,39	0,02	0,91	3,35
7453	137	3	Sukamantri	>500	Sengon	Paraserianthes falcataria (L.) I.C.	Leguminosae / Fabaceae	0,33	7,6	12	13	0,01	0,00	17,62	0,02	0,83	3,04
7454	137	3	Sukamantri	>500	Alpukat	Persea americana Mill.	Lauraceae	0,54	8,6	6	10	0,01	0,00	26,99	0,03	1,27	4,66
7455	137	3	Sukamantri	>500	Ganitri	Elaeocarpus ganitrus Roxb. ex G.	Elaeocarpaceae	0,49	8,0	11	13	0,01	0,00	27,27	0,03	1,28	4,70
7456	137	3	Sukamantri	>500	Ganitri	Elaeocarpus ganitrus Roxb. ex G.	Elaeocarpaceae	0,49	10,8	11	13	0,01	0,00	47,90	0,05	2,25	8,26
7457	137	3	Sukamantri	>500	Sawo / sawo manila	Manilkara zapota (L.) P.Royen	Sapotaceae	0,81	3,2	3	4	0,01	0,00	2,74	0,00	0,13	0,47
7458	137	3	Sukamantri	>500	Ganitri	Elaeocarpus ganitrus Roxb. ex G.	Elaeocarpaceae	0,49	4,5	5	7	0,01	0,00	5,35	0,01	0,25	0,92
7459	137	3	Sukamantri	>500	Ganitri	Elaeocarpus ganitrus Roxb. ex G.	Elaeocarpaceae	0,49	10,2	10	14	0,01	0,00	45,88	0,05	2,16	7,91
7460	137	3	Sukamantri	>500	Ganitri	Elaeocarpus ganitrus Roxb. ex G.	Elaeocarpaceae	0,49	7,3	10	13	0,01	0,00	23,41	0,02	1,10	4,04
7461	137	3	Sukamantri	>500	Rambutan	Nephelium lappaceum L.	Sapindaceae	0,71	6,4	6	9	0,01	0,00	18,17	0,02	0,85	3,13
7462	137	3	Sukamantri	>500	Nangka	Artocarpus heterophyllus Lam.	Moraceae	0,61	27,4	6	10	0,04	0,06	252,05	0,25	2,96	10,87
7463	137	3	Sukamantri	>500	Afrika	Maesopsis eminii Engl.	Rhamnaceae	0,43	22,0	15	17	0,04	0,04	198,72	0,20	2,33	8,57
7464	137	3	Sukamantri	>500	Afrika	Maesopsis eminii Engl.	Rhamnaceae	0,43	28,6	15	18	0,04	0,06	340,70	0,34	4,00	14,69
7465	137	3	Sukamantri	>500	Afrika	Maesopsis eminii Engl.	Rhamnaceae	0,43	30,2	13	17	0,04	0,07	356,99	0,36	4,19	15,39
7466	137	3	Sukamantri	>500	Afrika	Maesopsis eminii Engl.	Rhamnaceae	0,43	22,3	11	15	0,04	0,04	181,93	0,18	2,14	7,85
7467	137	3	Sukamantri	>500	Aren	Arenga pinnata (Wurmb) Merr.	Arecaceae	0,71	30,2	7	13	0,04	0,07	104,60	0,10	1,23	4,51
7468	138	2	Sukamantri	>500	Mahoni	Swietenia macrophylla King	Meliaceae	0,61	16,9	6	10	0,04	0,02	103,84	0,10	1,22	4,48
7469	138	2	Sukamantri	>500	Afrika	Maesopsis eminii Engl.	Rhamnaceae	0,43	20,7	12	14	0,04	0,03	149,10	0,15	1,75	6,43
7470	138	2	Sukamantri	>500	Afrika	Maesopsis eminii Engl.	Rhamnaceae	0,43	17,2	9	14	0,04	0,02	106,16	0,11	1,25	4,58
7471	138	2	Sukamantri	>500	Mahoni	Swietenia macrophylla King	Meliaceae	0,61	15,0	5	10	0,04	0,02	83,32	0,08	0,98	3,59
7472	138	2	Sukamantri	>500	Mahoni	Swietenia macrophylla King	Meliaceae	0,61	7,6	5	8	0,04	0,00	19,83	0,02	0,23	0,85
7473	138	2	Sukamantri	>500	Puspa	Schima wallichii Choisy	Theaceae	0,74	8,3	8	14	0,04	0,01	45,75	0,05	0,54	1,97
7474	138	2	Sukamantri	>500	Puspa	Schima wallichii Choisy	Theaceae	0,74	17,2	10	16	0,04	0,02	197,27	0,20	2,32	8,51
7475	138	2	Sukamantri	>500	Puspa	Schima wallichii Choisy	Theaceae	0,74	15,9	8	14	0,04	0,02	151,60	0,15	1,78	6,54
7476	138	2	Sukamantri	>500	Puspa	Schima wallichii Choisy	Theaceae	0,74	14,3	5	8	0,04	0,02	74,86	0,07	0,88	3,23
7477	138	2	Sukamantri	>500	Afrika	Maesopsis eminii Engl.	Rhamnaceae	0,43	23,9	9	12	0,04	0,04	168,27	0,17	1,98	7,26
7478	138	2	Sukamantri	>500	Afrika	Maesopsis eminii Engl.	Rhamnaceae	0,43	21,0	12	15	0,04	0,03	163,34	0,16	1,92	7,04
7479	138	2	Sukamantri	>500	Mahoni	Swietenia macrophylla King	Meliaceae	0,61	11,1	4	7	0,04	0,01	35,02	0,04	0,41	1,51
7480	138	2	Sukamantri	>500	Afrika	Maesopsis eminii Engl.	Rhamnaceae	0,43	13,7	9	11	0,04	0,01	56,08	0,06	0,66	2,42
7481	138	2	Sukamantri	>500	Mahoni	Swietenia macrophylla King	Meliaceae	0,61	17,8	5	10	0,04	0,02	114,86	0,11	1,35	4,95
7482	138	2	Sukamantri	>500	Mahoni	Swietenia macrophylla King	Meliaceae	0,61	12,7	5	9	0,04	0,01	56,30	0,06	0,66	2,43
7483	138	2	Sukamantri	>500	Afrika	Maesopsis eminii Engl.	Rhamnaceae	0,43	13,4	9	11	0,04	0,01	53,71	0,05	0,63	2,32
7484	138	2	Sukamantri	>500	Afrika	Maesopsis eminii Engl.	Rhamnaceae	0,43	22,9	12	13	0,04	0,04	168,03	0,17	1,97	7,25
7485	138	2	Sukamantri	>500	Mahoni	Swietenia macrophylla King	Meliaceae	0,61	9,2	6	7	0,04	0,01	24,81	0,02	0,29	1,07
7486	138	2	Sukamantri	>500	Mahoni	Swietenia macrophylla King	Meliaceae	0,61	17,5	6	9	0,04	0,02	100,90	0,10	1,19	4,35
7487	138	2	Sukamantri	>500	Mahoni	Swietenia macrophylla King	Meliaceae	0,61	9,2	4	6	0,04	0,01	21,55	0,02	0,25	0,93
7488	138	2	Sukamantri	>500	Mahoni	Swietenia macrophylla King	Meliaceae	0,61	12,1	4	5	0,04	0,01	29,92	0,03	0,35	1,29
7489	138	2	Sukamantri	>500	Mahoni	Swietenia macrophylla King	Meliaceae	0,61	15,0	4	8	0,04	0,02	67,92	0,07	0,80	2,93
7490	138	2	Sukamantri	>500	Mahoni	Swietenia macrophylla King	Meliaceae	0,61	15,9	6	8	0,04	0,02	76,07	0,08	0,89	3,28
7491	138	2	Sukamantri	>500	Mahoni	Swietenia macrophylla King	Meliaceae	0,61	22,6	6	9	0,04	0,04	161,09	0,16	1,89	6,95
7492	138	2	Sukamantri	>500	Afrika	Maesopsis eminii Engl.	Rhamnaceae	0,43	12,7	10	11	0,04	0,01	49,12	0,05	0,58	2,12
7493	138	2	Sukamantri	>500	Mahoni	Swietenia macrophylla King	Meliaceae	0,61	19,7	6	10	0,04	0,03	138,40	0,14	1,63	5,97
7494	138	2	Sukamantri	>500	Mahoni	Swietenia macrophylla King	Meliaceae	0,61	14,3	7	9	0,04	0,02	69,86	0,07	0,82	3,01
7495	138	2	Sukamantri	>500	Afrika	Maesopsis eminii Engl.	Rhamnaceae	0,43	18,8	11	12	0,04	0,03	108,42	0,11	1,27	4,68
7496	138	2	Sukamantri	>500	Afrika	Maesopsis eminii Engl.	Rhamnaceae	0,43	31,8	12	14	0,04	0,08	328,27	0,33	3,86	14,16
7497	138	2	Sukamantri	>500	Afrika	Maesopsis eminii Engl.	Rhamnaceae	0,43	6,7	8	9	0,04	0,00	12,55	0,01	0,15	0,54
7498	138	2	Sukamantri	>500	Petai	Parkia speciosa Hassk.	Leguminosae / Fabaceae	0,45	14,3	6	9	0,04	0,02	52,87	0,05	0,62	2,28
7499	138	2	Sukamantri	>500	Afrika	Maesopsis eminii Engl.	Rhamnaceae	0,43	22,0	10	11	0,04	0,04	133,37	0,13	1,57	5,75
7500	138	2	Sukamantri	>500	Afrika	Maesopsis eminii Engl.	Rhamnaceae	0,43	16,2	13	14	0,04	0,02	95,61	0,10	1,12	4,12
7501	138	2	Sukamantri	>500	Mahoni	Swietenia macrophylla King	Meliaceae	0,61	15,0	6	9	0,04	0,02	75,66	0,08	0,89	3,26
7502	138	2	Sukamantri	>500	Mareme	Glochidion borneense (Müll. Arg.	Phyllanthaceae	0,46	16,9	9	11	0,04	0,02	87,50	0,09	1,03	3,77
7503	138	2	Sukamantri	>500	Mahoni	Swietenia macrophylla King	Meliaceae	0,61	12,1	4	6	0,04	0,01	35,35	0,04	0,42	1,52
7504	138	2	Sukamantri	>500	Afrika	Maesopsis eminii Engl.	Rhamnaceae	0,43	18,1	10	11	0,04	0,03	93,98	0,09	1,10	4,05
7505	138	2	Sukamantri	>500	Mahoni	Swietenia macrophylla King	Meliaceae	0,61	19,7	7	11	0,04	0,03	151,03	0,15	1,77	6,51
7506	138	2	Sukamantri	>500	Afrika	Maesopsis eminii Engl.	Rhamnaceae	0,43	22,3	12	14	0,04	0,04	170,78	0,17	2,01	7,36
7507	138	2	Sukamantri	>500	Mahoni	Swietenia macrophylla King	Meliaceae	0,61	11,8	4	8	0,04	0,01	43,82	0,04	0,51	1,89
7508	139	1	Sukamantri	>500	Manglid	Magnolia champaca (L.) Baill. ex	Magnoliaceae	0,53	22,3	10	16	0,01	0,01	233,75	0,23	10,99	40,32
7509	139	1	Sukamantri	>500	Manglid	Magnolia champaca (L.) Baill. ex	Magnoliaceae	0,53	10,8	10	13	0,01	0,00	51,48	0,05	2,42	8,88
7510	139	1	Sukamantri	>500	Manglid	Magnolia champaca (L.) Baill. ex	Magnoliaceae	0,53	9,5	10	13	0,01	0,00	40,93	0,04	1,92	7,06
7511	139	1	Sukamantri	>500	Manglid	Magnolia champaca (L.) Baill. ex	Magnoliaceae	0,53	21,6	9	16	0,01	0,01	221,66	0,22	10,42	38,23
7512	139	1	Sukamantri	>500	Manglid	Magnolia champaca (L.) Baill. ex	Magnoliaceae	0,53	7,3	5	6	0,01	0,00	12,39	0,01	0,58	2,14
7513	139	1	Sukamantri	>500	Alpukat	Persea americana Mill.	Lauraceae	0,54	6,4	4	6	0,01	0,00	9,76	0,01	0,46	1,68
7514	139	1	Sukamantri	>500	Manglid	Magnolia champaca (L.) Baill. ex	Magnoliaceae	0,53	8,6	8	10	0,01	0,00	26,53	0,03	1,25	4,58
7515	139	1	Sukamantri	>500	Manglid	Magnolia champaca (L.) Baill. ex	Magnoliaceae	0,53	8,9	8	9	0,01	0,00	25,75	0,03	1,21	4,44
7516	139	1	Sukamantri	>500	Manglid	Magnolia champaca (L.) Baill. ex	Magnoliaceae	0,53	8,0	8	9	0,01	0,00	20,93	0,02	0,98	3,61
7517	139	1	Sukamantri	>500	Afrika	Maesopsis eminii Engl.	Rhamnaceae	0,43	4,5	4	5	0,01	0,00	3,49	0,00	0,16	0,60
7518	139	1	Sukamantri	>500	Manglid	Magnolia champaca (L.) Baill. ex	Magnoliaceae	0,53	15,9	11	13	0,01	0,00	104,34	0,10	4,90	18,00
7519	139	1	Sukamantri	>500	Manglid	Magnolia champaca (L.) Baill. ex	Magnoliaceae	0,53	7,0	7	8,5	0,01	0,00	15,71	0,02	0,74	2,71
7520	139	1	Sukamantri	>500	Manglid	Magnolia champaca (L.) Baill. ex	Magnoliaceae	0,53	9,9	10	13	0,01	0,00	43,46	0,04	2,04	7,50
7521	139	1	Sukamantri	>500	Manglid	Magnolia champaca (L.) Baill. ex	Magnoliaceae	0,53	10,8	10	13	0,01	0,00	51,48	0,05	2,42	8,88
7522	139	1	Sukamantri	>500	Manglid	Magnolia champaca (L.) Baill. ex	Magnoliaceae	0,53	9,9	10	12	0,01	0,00	40,39	0,04	1,90	6,97
7523	139	1	Sukamantri	>500	Manglid	Magnolia champaca (L.) Baill. ex	Magnoliaceae	0,53	16,2	11	13	0,01	0,01	108,19	0,11	5,09	18,66
7524	139	1	Sukamantri	>500	Manglid	Magnolia champaca (L.) Baill. ex	Magnoliaceae	0,53	8,6	9	10	0,01	0,00	26,53	0,03	1,25	4,58
7525	139	1	Sukamantri	>500	Manglid	Magnolia champaca (L.) Baill. ex	Magnoliaceae	0,53	11,1	10	12	0,01	0,00	50,45	0,05	2,37	8,70
7526	139	1	Sukamantri	>500	Manglid	Magnolia champaca (L.) Baill. ex	Magnoliaceae	0,53	14,0	10	14	0,01	0,00	88,35	0,09	4,15	15,24
7527	139	1	Sukamantri	>500	Manglid	Magnolia champaca (L.) Baill. ex	Magnoliaceae	0,53	7,6	7	8	0,01	0,00	17,43	0,02	0,82	3,01
7528	139	1	Sukamantri	>500	Manglid	Magnolia champaca (L.) Baill. ex	Magnoliaceae	0,53	5,1	5	7	0,01	0,00	7,34	0,01	0,34	1,27
7529	139	1	Sukamantri	>500	Manglid	Magnolia champaca (L.) Baill. ex	Magnoliaceae	0,53	5,7	5	7	0,01	0,00	9,11	0,01	0,43	1,57
7530	139	1	Sukamantri	>500	Manglid	Magnolia champaca (L.) Baill. ex	Magnoliaceae	0,53	8,0	8	11	0,01	0,00	25,15	0,03	1,18	4,34
7531	139	1	Sukamantri	>500	Manglid	Magnolia champaca (L.) Baill. ex	Magnoliaceae	0,53	2,9	2	3	0,01	0,00	1,18	0,00	0,06	0,20
7532	139	1	Sukamantri	>500	Manglid	Magnolia champaca (L.) Baill. ex	Magnoliaceae	0,53	17,2	12	15	0,01	0,01	136,96	0,14	6,44	23,62
7533	139	1	Sukamantri	>500	Manglid	Magnolia champaca (L.) Baill. ex	Magnoliaceae	0,53	21,6	12	17	0,04	0,04	234,32	0,23	2,75	10,10
7534	139	1	Sukamantri	>500	Manglid	Magnolia champaca (L.) Baill. ex	Magnoliaceae	0,53	23,5	13	18	0,04	0,04	288,28	0,29	3,39	12,43
7535	139	1	Sukamantri	>500	Manglid	Magnolia champaca (L.) Baill. ex	Magnoliaceae	0,53	24,8	13	18	0,04	0,05	317,47	0,32	3,73	13,69
7536	139	1	Sukamantri	>500	Manglid	Magnolia champaca (L.) Baill. ex	Magnoliaceae	0,53	22,6	12	18	0,04	0,04	267,23	0,27	3,14	11,52
7537	139	1	Sukamantri	>500	Manglid	Magnolia champaca (L.) Baill. ex	Magnoliaceae	0,53	20,0	14	18	0,04	0,03	214,67	0,21	2,52	9,26
7538	139	1	Sukamantri	>500	Manglid	Magnolia champaca (L.) Baill. ex	Magnoliaceae	0,53	24,2	12	17	0,04	0,05	287,27	0,29	3,38	12,39
7539	139	1	Sukamantri	>500	Manglid	Magnolia champaca (L.) Baill. ex	Magnoliaceae	0,53	20,7	11	17	0,04	0,03	215,73	0,22	2,53	9,30
7540	139	1	Sukamantri	>500	Manglid	Magnolia champaca (L.) Baill. ex	Magnoliaceae	0,53	21,0	12	17	0,04	0,03	221,85	0,22	2,61	9,57
7541	139	1	Sukamantri	>500	Manglid	Magnolia champaca (L.) Baill. ex	Magnoliaceae	0,53	20,0	11	16	0,04	0,03	192,72	0,19	2,26	8,31
7542	140	2	Sukamantri	>500	Sengon	Paraserianthes falcataria (L.) I.C.	Leguminosae / Fabaceae	0,33	4,1	5	7	0,01	0,00	3,25	0,00	0,15	0,56
7543	140	2	Sukamantri	>500	Sengon	Paraserianthes falcataria (L.) I.C.	Leguminosae / Fabaceae	0,33	15,0	10	14	0,01	0,00	64,60	0,06	3,04	11,14
7544	140	2	Sukamantri	>500	Tisuk	Hibiscus macrophyllus Roxb. ex I	Malvaceae	0,4	4,8	6	7	0,01	0,00	5,04	0,01	0,24	0,87
7545	140	2	Sukamantri	>500	Afrika	Maesopsis eminii Engl.	Rhamnaceae	0,43	20,7	13	17	0,01	0,01	178,12	0,18	8,37	30,72
7546	140	2	Sukamantri	>500	Tisuk	Hibiscus macrophyllus Roxb. ex I	Malvaceae	0,4	3,8	4	5	0,01	0,00	2,46	0,00	0,12	0,42
7547	140	2	Sukamantri	>500	Afrika	Maesopsis eminii Engl.	Rhamnaceae	0,43	11,1	9	10	0,01	0,00	35,25	0,04	1,66	6,08
7548	140	2	Sukamantri	>500	Tisuk	Hibiscus macrophyllus Roxb. ex I	Malvaceae	0,4	9,5	8	13	0,01	0,00	31,63	0,03	1,49	5,46
7549	140	2	Sukamantri	>500	Sengon	Paraserianthes falcataria (L.) I.C.	Leguminosae / Fabaceae	0,33	13,4	11	14	0,01	0,00	52,57	0,05	2,47	9,07
7550	140	2	Sukamantri	>500	Tisuk	Hibiscus macrophyllus Roxb. ex I	Malvaceae	0,4	5,7	7	8	0,01	0,00	7,95	0,01	0,37	1,37
7551	140	2	Sukamantri	>500	Alpukat	Persea americana Mill.	Lauraceae	0,54	7,0	5	6	0,01	0,00	11,62	0,01	0,55	2,00
7552	140	2	Sukamantri	>500	Sengon	Paraserianthes falcataria (L.) I.C.	Leguminosae / Fabaceae	0,33	8,3	9	10	0,01	0,00	16,04	0,02	0,75	2,77
7553	140	2	Sukamantri	>500	Tisuk	Hibiscus macrophyllus Roxb. ex I	Malvaceae	0,4	4,5	6	7	0,01	0,00	4,44	0,00	0,21	0,77
7554	140	2	Sukamantri	>500	Tisuk	Hibiscus macrophyllus Roxb. ex I	Malvaceae	0,4	3,5	4	5,5	0,01	0,00	2,29	0,00	0,11	0,39
7555	140	2	Sukamantri	>500	Sengon	Paraserianthes falcataria (L.) I.C.	Leguminosae / Fabaceae	0,33	4,1	7	9	0,01	0,00	4,09	0,00	0,19	0,71
7556	140	2	Sukamantri	>500	Huru Kacang	Neolitsea triplinervia Merr.	Lauraceae	0,56	4,5	3	4,2	0,01	0,00	3,78	0,00	0,18	0,65
7557	140	2	Sukamantri	>500	Afrika	Maesopsis eminii Engl.	Rhamnaceae	0,43	11,8	10	11	0,01	0,00	42,58	0,04	2,00	7,35
7558	140	2	Sukamantri	>500	Afrika	Maesopsis eminii Engl.	Rhamnaceae	0,43	7,0	6	8	0,01	0,00	12,27	0,01	0,58	2,12

7559	140	2	Sukamantri	>500	Saga	Adenanthera pavonina L.	Leguminosae / Fabaceae	0,7	7,0	7	8	0,01	0,00	19,18	0,02	0,90	3,31
7560	140	2	Sukamantri	>500	Saga	Adenanthera pavonina L.	Leguminosae / Fabaceae	0,7	6,7	7	8	0,01	0,00	17,61	0,02	0,83	3,04
7561	140	2	Sukamantri	>500	Sengon	Paraserianthes falcataria (L.) I.C.	Leguminosae / Fabaceae	0,33	3,2	5	6	0,01	0,00	1,75	0,00	0,08	0,30
7562	140	2	Sukamantri	>500	Sengon	Paraserianthes falcataria (L.) I.C.	Leguminosae / Fabaceae	0,33	8,0	6	9	0,01	0,00	13,56	0,01	0,64	2,34
7563	140	2	Sukamantri	>500	Tisuk	Hibiscus macrophyllus Roxb. ex I	Malvaceae	0,4	6,7	6	10	0,01	0,00	12,94	0,01	0,61	2,23
7564	140	2	Sukamantri	>500	Saga	Adenanthera pavonina L.	Leguminosae / Fabaceae	0,7	18,5	8	13	0,01	0,01	176,69	0,18	8,30	30,48
7565	140	2	Sukamantri	>500	Sengon	Paraserianthes falcataria (L.) I.C.	Leguminosae / Fabaceae	0,33	11,8	10	14	0,01	0,00	41,68	0,04	1,96	7,19
7566	140	2	Sukamantri	>500	Aren	Arenga pinnata (Wurmb) Merr.	Arecaceae	0,71	39,1	5	11	0,01	0,03	89,20	0,09	4,19	15,39
7567	140	2	Sukamantri	>500	Sengon	Paraserianthes falcataria (L.) I.C.	Leguminosae / Fabaceae	0,33	13,0	10	12	0,01	0,00	43,67	0,04	2,05	7,53
7568	140	2	Sukamantri	>500	Saga	Adenanthera pavonina L.	Leguminosae / Fabaceae	0,7	7,6	6	10	0,01	0,00	27,59	0,03	1,30	4,76
7569	140	2	Sukamantri	>500	Saga	Adenanthera pavonina L.	Leguminosae / Fabaceae	0,7	7,0	4	10	0,01	0,00	23,53	0,02	1,11	4,06
7570	140	2	Sukamantri	>500	Saga	Adenanthera pavonina L.	Leguminosae / Fabaceae	0,7	21,3	8	13	0,01	0,01	230,13	0,23	10,82	39,69
7571	140	2	Sukamantri	>500	Manglid	Magnolia champaca (L.) Baill. ex	Magnoliaceae	0,53	30,9	12	17	0,04	0,07	449,17	0,45	5,28	19,37
7572	140	2	Sukamantri	>500	Manglid	Magnolia champaca (L.) Baill. ex	Magnoliaceae	0,53	28,6	15	19	0,04	0,06	433,58	0,43	5,09	18,70
7573	140	2	Sukamantri	>500	Afrika	Maesopsis eminii Engl.	Rhamnaceae	0,43	21,0	16	18	0,04	0,03	193,02	0,19	2,27	8,32
7574	140	2	Sukamantri	>500	Afrika	Maesopsis eminii Engl.	Rhamnaceae	0,43	19,4	13	15	0,04	0,03	141,38	0,14	1,66	6,10
7575	140	2	Sukamantri	>500	Tisuk	Hibiscus macrophyllus Roxb. ex I	Malvaceae	0,4	16,9	9	14	0,04	0,02	96,01	0,10	1,13	4,14
7576	140	2	Sukamantri	>500	Sengon	Paraserianthes falcataria (L.) I.C.	Leguminosae / Fabaceae	0,33	17,2	11	15	0,04	0,02	88,74	0,09	1,04	3,83
7577	140	2	Sukamantri	>500	Puspa	Schima wallichii Choisy	Theaceae	0,74	12,1	4	12	0,04	0,01	79,62	0,08	0,94	3,43
7578	140	2	Sukamantri	>500	Puspa	Schima wallichii Choisy	Theaceae	0,74	11,8	4	10	0,04	0,01	64,16	0,06	0,75	2,77
7579	140	2	Sukamantri	>500	Tisuk	Hibiscus macrophyllus Roxb. ex I	Malvaceae	0,4	10,5	9	12	0,04	0,01	35,00	0,03	0,41	1,51
7580	140	2	Sukamantri	>500	Afrika	Maesopsis eminii Engl.	Rhamnaceae	0,43	12,1	11	12	0,04	0,01	48,43	0,05	0,57	2,09
7581	140	2	Sukamantri	>500	Afrika	Maesopsis eminii Engl.	Rhamnaceae	0,43	10,8	10	11	0,04	0,01	36,47	0,04	0,43	1,57
7582	140	2	Sukamantri	>500	Sengon	Paraserianthes falcataria (L.) I.C.	Leguminosae / Fabaceae	0,33	12,7	10	14	0,04	0,01	48,07	0,05	0,56	2,07
7583	140	2	Sukamantri	>500	Sengon	Paraserianthes falcataria (L.) I.C.	Leguminosae / Fabaceae	0,33	10,8	11	14	0,04	0,01	35,69	0,04	0,42	1,54
7584	140	2	Sukamantri	>500	Afrika	Maesopsis eminii Engl.	Rhamnaceae	0,43	21,0	12	17	0,04	0,03	183,18	0,18	2,15	7,90
7585	140	2	Sukamantri	>500	Saga	Adenanthera pavonina L.	Leguminosae / Fabaceae	0,7	17,2	10	14	0,04	0,02	165,89	0,17	1,95	7,15
7586	140	2	Sukamantri	>500	Puspa	Schima wallichii Choisy	Theaceae	0,74	15,0	9	10	0,04	0,02	99,45	0,10	1,17	4,29
7587	140	2	Sukamantri	>500	Afrika	Maesopsis eminii Engl.	Rhamnaceae	0,43	17,5	15	16	0,04	0,02	124,08	0,12	1,46	5,35
7588	140	2	Sukamantri	>500	Puspa	Schima wallichii Choisy	Theaceae	0,74	11,5	5	10	0,04	0,01	61,02	0,06	0,72	2,63
7589	141	2	Sukamantri	>500	Mahoni	Swietenia macrophylla King	Meliaceae	0,61	15,9	3	7	0,01	0,00	67,31	0,07	3,16	11,61
7590	141	2	Sukamantri	>500	Mahoni	Swietenia macrophylla King	Meliaceae	0,61	17,2	5	9	0,01	0,01	97,57	0,10	4,59	16,83
7591	141	2	Sukamantri	>500	Mahoni	Swietenia macrophylla King	Meliaceae	0,61	9,2	4	7	0,01	0,00	24,81	0,02	1,17	4,28
7592	141	2	Sukamantri	>500	Afrika	Maesopsis eminii Engl.	Rhamnaceae	0,43	21,3	9	11	0,01	0,01	126,37	0,13	5,94	21,80
7593	141	2	Sukamantri	>500	Afrika	Maesopsis eminii Engl.	Rhamnaceae	0,43	22,6	10	12	0,01	0,01	152,20	0,15	7,15	26,25
7594	141	2	Sukamantri	>500	Afrika	Maesopsis eminii Engl.	Rhamnaceae	0,43	8,9	8	9	0,01	0,00	21,26	0,02	1,00	3,67
7595	141	2	Sukamantri	>500	Puspa	Schima wallichii Choisy	Theaceae	0,74	19,4	6	10	0,01	0,01	160,35	0,16	7,54	27,66
7596	141	2	Sukamantri	>500	Afrika	Maesopsis eminii Engl.	Rhamnaceae	0,43	3,5	4	5	0,01	0,00	2,24	0,00	0,11	0,39
7597	141	2	Sukamantri	>500	Mahoni	Swietenia macrophylla King	Meliaceae	0,61	9,5	4	6	0,01	0,00	22,93	0,02	1,08	3,95
7598	141	2	Sukamantri	>500	Mahoni	Swietenia macrophylla King	Meliaceae	0,61	3,8	2	3	0,01	0,00	2,27	0,00	0,11	0,39
7599	141	2	Sukamantri	>500	Afrika	Maesopsis eminii Engl.	Rhamnaceae	0,43	10,2	9	10	0,01	0,00	29,91	0,03	1,41	5,16
7600	141	2	Sukamantri	>500	Mahoni	Swietenia macrophylla King	Meliaceae	0,61	3,2	2	2,5	0,01	0,00	1,37	0,00	0,06	0,24
7601	141	2	Sukamantri	>500	Mahoni	Swietenia macrophylla King	Meliaceae	0,61	3,5	2	4	0,01	0,00	2,52	0,00	0,12	0,43
7602	141	2	Sukamantri	>500	Mahoni	Swietenia macrophylla King	Meliaceae	0,61	8,9	4	7	0,01	0,00	23,27	0,02	1,09	4,01
7603	141	2	Sukamantri	>500	Mahoni	Swietenia macrophylla King	Meliaceae	0,61	3,8	2	3	0,01	0,00	2,27	0,00	0,11	0,39
7604	141	2	Sukamantri	>500	Mahoni	Swietenia macrophylla King	Meliaceae	0,61	5,7	3,5	5	0,01	0,00	7,61	0,01	0,36	1,31
7605	141	2	Sukamantri	>500	Nangka	Artocarpus heterophyllus Lam.	Moraceae	0,61	4,5	3	3,5	0,01	0,00	3,46	0,00	0,16	0,60
7606	141	2	Sukamantri	>500	Gmelina	Gmelina arborea Roxb.	Lamiaceae / Verbenaceae	0,47	13,7	7	11	0,01	0,00	60,84	0,06	2,86	10,49
7607	141	2	Sukamantri	>500	Afrika	Maesopsis eminii Engl.	Rhamnaceae	0,43	6,0	8	9	0,01	0,00	10,45	0,01	0,49	1,80
7608	141	2	Sukamantri	>500	Mahoni	Swietenia macrophylla King	Meliaceae	0,61	4,1	3	4,5	0,01	0,00	3,81	0,00	0,18	0,66
7609	141	2	Sukamantri	>500	Afrika	Maesopsis eminii Engl.	Rhamnaceae	0,43	9,5	8	9	0,01	0,00	24,13	0,02	1,13	4,16
7610	141	2	Sukamantri	>500	Gmelina	Gmelina arborea Roxb.	Lamiaceae / Verbenaceae	0,47	14,6	7	11	0,01	0,00	68,84	0,07	3,24	11,87
7611	141	2	Sukamantri	>500	Mahoni	Swietenia macrophylla King	Meliaceae	0,61	3,2	2,5	3	0,01	0,00	1,62	0,00	0,08	0,28
7612	141	2	Sukamantri	>500	Afrika	Maesopsis eminii Engl.	Rhamnaceae	0,43	18,1	10	12	0,01	0,01	101,78	0,10	4,78	17,56
7613	141	2	Sukamantri	>500	Mahoni	Swietenia macrophylla King	Meliaceae	0,61	11,1	5	7	0,01	0,00	35,02	0,04	1,65	6,04
7614	141	2	Sukamantri	>500	Mahoni	Swietenia macrophylla King	Meliaceae	0,61	9,9	4	6	0,01	0,00	24,35	0,02	1,14	4,20
7615	141	2	Sukamantri	>500	Nangka	Artocarpus heterophyllus Lam.	Moraceae	0,61	11,8	3	8	0,01	0,00	43,82	0,04	2,06	7,56
7616	141	2	Sukamantri	>500	Afrika	Maesopsis eminii Engl.	Rhamnaceae	0,43	8,3	9	10	0,01	0,00	20,45	0,02	0,96	3,53
7617	141	2	Sukamantri	>500	Gmelina	Gmelina arborea Roxb.	Lamiaceae / Verbenaceae	0,47	12,4	8	12	0,01	0,00	55,10	0,06	2,59	9,50
7618	141	2	Sukamantri	>500	Gmelina	Gmelina arborea Roxb.	Lamiaceae / Verbenaceae	0,47	21,0	11	14	0,04	0,03	166,35	0,17	1,95	7,17
7619	141	2	Sukamantri	>500	Mahoni	Swietenia macrophylla King	Meliaceae	0,61	28,6	7	13	0,04	0,06	348,36	0,35	4,09	15,02
7620	141	2	Sukamantri	>500	Nangka	Artocarpus heterophyllus Lam.	Moraceae	0,61	21,3	3	9	0,04	0,04	144,86	0,14	1,70	6,25
7621	141	2	Sukamantri	>500	Mahoni	Swietenia macrophylla King	Meliaceae	0,61	21,3	4	12	0,04	0,04	188,53	0,19	2,22	8,13
7622	141	2	Sukamantri	>500	Gmelina	Gmelina arborea Roxb.	Lamiaceae / Verbenaceae	0,47	21,0	10	14	0,04	0,03	166,35	0,17	1,95	7,17
7623	141	2	Sukamantri	>500	Suren	Toona sureni (Blume) Merr.	Meliaceae	0,39	27,7	7	14	0,04	0,06	232,59	0,23	2,73	10,03
7624	141	2	Sukamantri	>500	Gmelina	Gmelina arborea Roxb.	Lamiaceae / Verbenaceae	0,47	20,0	7	13	0,04	0,03	142,73	0,14	1,68	6,16
7625	141	2	Sukamantri	>500	Suren	Toona sureni (Blume) Merr.	Meliaceae	0,39	26,1	6	12	0,04	0,05	181,21	0,18	2,13	7,81
7626	141	2	Sukamantri	>500	Afrika	Maesopsis eminii Engl.	Rhamnaceae	0,43	34,0	9	14	0,04	0,09	371,59	0,37	4,37	16,02
7627	141	2	Sukamantri	>500	Afrika	Maesopsis eminii Engl.	Rhamnaceae	0,43	43,3	10	15	0,04	0,15	614,21	0,61	7,22	26,49
7628	141	2	Sukamantri	>500	Afrika	Maesopsis eminii Engl.	Rhamnaceae	0,43	23,5	12	15	0,04	0,04	201,42	0,20	2,37	8,69
7629	142	2	Sukamantri	>500	Sengon	Paraserianthes falcataria (L.) I.C.	Leguminosae / Fabaceae	0,33	37,2	12	18	0,01	0,03	432,34	0,43	20,32	74,57
7630	142	2	Sukamantri	>500	Afrika	Maesopsis eminii Engl.	Rhamnaceae	0,43	18,1	13	18	0,01	0,01	147,56	0,15	6,94	25,45
7631	142	2	Sukamantri	>500	Afrika	Maesopsis eminii Engl.	Rhamnaceae	0,43	16,9	10	11	0,01	0,01	82,26	0,08	3,87	14,19
7632	142	2	Sukamantri	>500	Afrika	Maesopsis eminii Engl.	Rhamnaceae	0,43	10,8	9	10	0,01	0,00	33,42	0,03	1,57	5,77
7633	142	2	Sukamantri	>500	Afrika	Maesopsis eminii Engl.	Rhamnaceae	0,43	6,4	6	7	0,01	0,00	9,12	0,01	0,43	1,57
7634	142	2	Sukamantri	>500	Afrika	Maesopsis eminii Engl.	Rhamnaceae	0,43	9,5	7	8	0,01	0,00	21,66	0,02	1,02	3,74
7635	142	2	Sukamantri	>500	Afrika	Maesopsis eminii Engl.	Rhamnaceae	0,43	9,5	10	11	0,01	0,00	29,00	0,03	1,36	5,00
7636	142	2	Sukamantri	>500	Afrika	Maesopsis eminii Engl.	Rhamnaceae	0,43	7,0	7	8	0,01	0,00	12,27	0,01	0,58	2,12
7637	142	2	Sukamantri	>500	Afrika	Maesopsis eminii Engl.	Rhamnaceae	0,43	13,4	8	9	0,01	0,00	44,69	0,04	2,10	7,71
7638	142	2	Sukamantri	>500	Afrika	Maesopsis eminii Engl.	Rhamnaceae	0,43	13,4	10	11	0,01	0,00	53,71	0,05	2,52	9,27
7639	142	2	Sukamantri	>500	Afrika	Maesopsis eminii Engl.	Rhamnaceae	0,43	15,0	11	12	0,01	0,00	71,48	0,07	3,36	12,33
7640	142	2	Sukamantri	>500	Alpukat	Persea americana Mill.	Lauraceae	0,54	6,0	3	4	0,01	0,00	6,13	0,01	0,29	1,06
7641	142	2	Sukamantri	>500	Alpukat	Persea americana Mill.	Lauraceae	0,54	12,7	4	9	0,01	0,00	50,36	0,05	2,37	8,69
7642	142	2	Sukamantri	>500	Manglid	Magnolia champaca (L.) Baill. ex	Magnoliaceae	0,53	5,7	4	6	0,01	0,00	7,91	0,01	0,37	1,36
7643	142	2	Sukamantri	>500	Gmelina	Gmelina arborea Roxb.	Lamiaceae / Verbenaceae	0,47	4,5	3	4	0,01	0,00	3,08	0,00	0,14	0,53
7644	142	2	Sukamantri	>500	Afrika	Maesopsis eminii Engl.	Rhamnaceae	0,43	4,5	7	8	0,01	0,00	5,36	0,01	0,25	0,92
7645	142	2	Sukamantri	>500	Afrika	Maesopsis eminii Engl.	Rhamnaceae	0,43	8,6	8	9	0,01	0,00	19,89	0,02	0,94	3,43
7646	142	2	Sukamantri	>500	Sengon	Paraserianthes falcataria (L.) I.C.	Leguminosae / Fabaceae	0,33	13,7	12	14	0,01	0,00	54,88	0,05	2,58	9,47
7647	142	2	Sukamantri	>500	Suren	Toona sureni (Blume) Merr.	Meliaceae	0,39	4,5	3	4	0,01	0,00	2,60	0,00	0,12	0,45
7648	142	2	Sukamantri	>500	Manglid	Magnolia champaca (L.) Baill. ex	Magnoliaceae	0,53	3,2	2	3	0,01	0,00	1,43	0,00	0,07	0,25
7649	142	2	Sukamantri	>500	Afrika	Maesopsis eminii Engl.	Rhamnaceae	0,43	9,5	12	13	0,01	0,00	33,79	0,03	1,59	5,83
7650	142	2	Sukamantri	>500	Afrika	Maesopsis eminii Engl.	Rhamnaceae	0,43	8,6	10	11	0,01	0,00	23,91	0,02	1,12	4,12
7651	142	2	Sukamantri	>500	Afrika	Maesopsis eminii Engl.	Rhamnaceae	0,43	28,3	12	14	0,01	0,02	265,16	0,27	12,46	45,74
7652	142	2	Sukamantri	>500	Afrika	Maesopsis eminii Engl.	Rhamnaceae	0,43	16,2	15	16	0,01	0,01	108,05	0,11	5,08	18,64
7653	142	2	Sukamantri	>500	Afrika	Maesopsis eminii Engl.	Rhamnaceae	0,43	11,5	10	11	0,01	0,00	40,50	0,04	1,90	6,99
7654	142	2	Sukamantri	>500	Sengon	Paraserianthes falcataria (L.) I.C.	Leguminosae / Fabaceae	0,33	18,5	14	17	0,01	0,01	113,44	0,11	5,33	19,57
7655	142	2	Sukamantri	>500	Kilunglum	Sp3	0	0,5	24,2	2	9	0,01	0,01	152,09	0,15	7,15	26,23
7656	142	2	Sukamantri	>500	Afrika	Maesopsis eminii Engl.	Rhamnaceae	0,43	22,3	14	15	0,04	0,04	181,93	0,18	2,14	7,85
7657	142	2	Sukamantri	>500	Afrika	Maesopsis eminii Engl.	Rhamnaceae	0,43	25,5	16	18	0,04	0,05	274,58	0,27	3,23	11,84
7658	142	2	Sukamantri	>500	Afrika	Maesopsis eminii Engl.	Rhamnaceae	0,43	25,5	14	16	0,04	0,05	246,50	0,25	2,90	10,63
7659	142	2	Sukamantri	>500	Afrika	Maesopsis eminii Engl.	Rhamnaceae	0,43	20,0	15	17	0,04	0,03	168,21	0,17	1,98	7,25
7660	142	2	Sukamantri	>500	Afrika	Maesopsis eminii Engl.	Rhamnaceae	0,43	24,8	15	16	0,04	0,05	235,32	0,24	2,77	10,15
7661	142	2	Sukamantri	>500	Afrika	Maesopsis eminii Engl.	Rhamnaceae	0,43	20,7	15	16	0,04	0,03	168,50	0,17	1,98	7,27
7662	142	2	Sukamantri	>500	Afrika	Maesopsis eminii Engl.	Rhamnaceae	0,43	20,4	16	17	0,04	0,03	173,14	0,17	2,03	7,47
7663	142	2	Sukamantri	>500	Afrika	Maesopsis eminii Engl.	Rhamnaceae	0,43	21,6	14	16	0,04	0,04	183,02	0,18	2,15	7,89
7664	142	2	Sukamantri	>500	Afrika	Maesopsis eminii Engl.	Rhamnaceae	0,43	23,9	15	17	0,04	0,04	231,52	0,23	2,72	9,98
7665	142	2	Sukamantri	>500	Afrika	Maesopsis eminii Engl.	Rhamnaceae	0,43	26,7	15	17	0,04	0,06	284,94	0,28	3,35	12,29
7666	142	2	Sukamantri	>500	Afrika	Maesopsis eminii Engl.	Rhamnaceae	0,43	22,6	15	17	0,04	0,04	209,40	0,21	2,46	9,03
7667	142	2	Sukamantri	>500	Afrika	Maesopsis eminii Engl.	Rhamnaceae	0,43	26,7	15	17	0,04	0,06	284,94	0,28	3,35	12,29
7668	142	2	Sukamantri	>500	Afrika	Maesopsis eminii Engl.	Rhamnaceae	0,43	23,9	14	16	0,04	0,04	219,01	0,22	2,57	9,44
7669	143	2	Sukamantri	>500	Pinus	Pinus merkusii Jungh. & de Vriese	Pinaceae	0,55	3,2	2	3	0,01	0,00	1,48	0,00	0,07	0,25
7670	143	2	Sukamantri	>500	Ganitri	Elaeocarpus ganitrus Roxb. ex G.	Elaeocarpaceae	0,49	10,2	4	7	0,01	0,00	24,32	0,02	1,14	4,19
7671	143	2	Sukamantri	>500	Ganitri	Elaeocarpus ganitrus Roxb. ex G.	Elaeocarpaceae	0,49	17,2	15	17	0,01	0,01	142,95	0,14	6,72	24,66
7672	143	2	Sukamantri	>500	Ganitri	Elaeocarpus ganitrus Roxb. ex G.	Elaeocarpaceae	0,49	12,4	10	13	0,01	0,00	61,59	0,06	2,89	10,62
7673	143	2	Sukamantri	>500	Gmelina	Gmelina arborea Roxb.	Lamiaceae / Verbenaceae	0,47	22,0	14	17	0,01	0,01	215,59	0,22	10,13	37,19
7674	143	2	Sukamantri	>500	Ganitri	Elaeocarpus ganitrus Roxb. ex G.	Elaeocarpaceae	0,49	12,1	9	12	0,01	0,00	54,58	0,05	2,57	9,41
7675	143	2	Sukamantri	>500	Gmelina	Gmelina arborea Roxb.	Lamiaceae / Verbenaceae	0,47	29,3	14	18	0,01	0,02	384,81	0,38	18,09	66,38
7676	143	2	Sukamantri	>500	Ganitri	Elaeocarpus ganitrus Roxb. ex G.	Elaeocarpaceae	0,49	14,0	8	12	0,01	0,00	71,40	0,07	3,36	12,32
7677	143	2	Sukamantri	>500	Gmelina	Gmelina arborea Roxb.	Lamiaceae / Verbenaceae	0,47	26,7	14	7	0,01	0,01	137,14	0,14	6,45	23,65
7678	143	2	Sukamantri	>500	Ganitri	Elaeocarpus ganitrus Roxb. ex G.	Elaeocarpaceae	0,49	14,6	10	13	0,01	0,00	83,35	0,08	3,92	14,38
7679	143	2	Sukamantri	>500	Ganitri	Elaeocarpus ganitrus Roxb. ex G.	Elaeocarpaceae	0,49	12,4	10	12	0,01	0,00	57,24	0,06	2,69	9,87
7680	143	2	Sukamantri	>500	Ganitri	Elaeocarpus ganitrus Roxb. ex G.	Elaeocarpaceae	0,49	16,9	15	17	0,01	0,01	138,13	0,14	6,49	23,83
7681	143	2	Sukamantri	>500	Ganitri	Elaeocarpus ganitrus Roxb. ex G.	Elaeocarpaceae	0,49	15,6	15	17	0,01	0,00	119,64	0,12	5,62	20,64
7682	143	2	Sukamantri	>500	Nangka	Artocarpus heterophyllus Lam.	Moraceae	0,61	7,6	3	6	0,01	0,00	15,23	0,02	0,72	2,63

7683	143	2	Sukamantri	>500	Ganitri	Elaeocarpus ganitrus Roxb. ex G.	Elaeocarpaceae	0,49	8,6	7	8	0,01	0,00	20,13	0,02	0,95	3,47
7684	143	2	Sukamantri	>500	Gmelina	Gmelina arborea Roxb.	Lamiaceae / Verbenaceae	0,47	13,4	12	15	0,01	0,00	77,42	0,08	3,64	13,35
7685	143	2	Sukamantri	>500	Ganitri	Elaeocarpus ganitrus Roxb. ex G.	Elaeocarpaceae	0,49	10,5	12	14	0,01	0,00	48,54	0,05	2,28	8,37
7686	143	2	Sukamantri	>500	Ganitri	Elaeocarpus ganitrus Roxb. ex G.	Elaeocarpaceae	0,49	18,1	14	17	0,01	0,01	157,83	0,16	7,42	27,22
7687	143	2	Sukamantri	>500	Pinus	Pinus merkusii Jungh. & de Vriese	Pinaceae	0,55	4,5	4	6	0,01	0,00	5,16	0,01	0,24	0,89
7688	143	2	Sukamantri	>500	Ganitri	Elaeocarpus ganitrus Roxb. ex G.	Elaeocarpaceae	0,49	11,5	12	14	0,01	0,00	56,93	0,06	2,68	9,82
7689	143	2	Sukamantri	>500	Ganitri	Elaeocarpus ganitrus Roxb. ex G.	Elaeocarpaceae	0,49	18,5	13	15	0,01	0,00	145,29	0,15	6,83	25,06
7690	143	2	Sukamantri	>500	Ganitri	Elaeocarpus ganitrus Roxb. ex G.	Elaeocarpaceae	0,49	10,2	9	12	0,01	0,00	39,84	0,04	1,87	6,87
7691	143	2	Sukamantri	>500	Ganitri	Elaeocarpus ganitrus Roxb. ex G.	Elaeocarpaceae	0,49	16,9	13	15	0,01	0,01	123,17	0,12	5,79	21,25
7692	143	2	Sukamantri	>500	Gmelina	Gmelina arborea Roxb.	Lamiaceae / Verbenaceae	0,47	32,8	12	17	0,04	0,08	449,13	0,45	5,28	19,37
7693	143	2	Sukamantri	>500	Afrika	Maesopsis eminii Engl.	Rhamnaceae	0,43	28,3	13	15	0,04	0,06	282,46	0,28	3,32	12,18
7694	143	2	Sukamantri	>500	Afrika	Maesopsis eminii Engl.	Rhamnaceae	0,43	20,7	13	15	0,04	0,03	158,83	0,16	1,87	6,85
7695	143	2	Sukamantri	>500	Ganitri	Elaeocarpus ganitrus Roxb. ex G.	Elaeocarpaceae	0,49	20,0	13	17	0,04	0,03	189,59	0,19	2,23	8,18
7696	143	2	Sukamantri	>500	Ganitri	Elaeocarpus ganitrus Roxb. ex G.	Elaeocarpaceae	0,49	22,6	12	17	0,04	0,04	236,01	0,24	2,77	10,18
7697	143	2	Sukamantri	>500	Gmelina	Gmelina arborea Roxb.	Lamiaceae / Verbenaceae	0,47	21,6	11	15	0,04	0,04	187,16	0,19	2,20	8,07
7698	143	2	Sukamantri	>500	Gmelina	Gmelina arborea Roxb.	Lamiaceae / Verbenaceae	0,47	31,2	12	17	0,04	0,08	409,99	0,41	4,82	17,68
7699	143	2	Sukamantri	>500	Ganitri	Elaeocarpus ganitrus Roxb. ex G.	Elaeocarpaceae	0,49	22,6	15	18	0,04	0,04	248,70	0,25	2,92	10,72
7700	143	2	Sukamantri	>500	Gmelina	Gmelina arborea Roxb.	Lamiaceae / Verbenaceae	0,47	36,9	14	17	0,04	0,11	558,39	0,56	6,56	24,08
7701	143	2	Sukamantri	>500	Ganitri	Elaeocarpus ganitrus Roxb. ex G.	Elaeocarpaceae	0,49	21,6	15	18	0,04	0,04	229,79	0,23	2,70	9,91
7702	144	3	Sukamantri	>500	Kopi	Coffea canephora Pierre ex A.Fro	Rubiaceae	0,41	5,7	1	2,5	0,04	0,00	10,06	0,01	0,12	0,43
7703	144	3	Sukamantri	>500	Kopi	Coffea canephora Pierre ex A.Fro	Rubiaceae	0,41	5,4	1	2	0,04	0,00	8,95	0,01	0,11	0,39
7704	144	3	Sukamantri	>500	Kopi	Coffea canephora Pierre ex A.Fro	Rubiaceae	0,41	3,2	1	1,8	0,04	0,00	3,01	0,00	0,04	0,13
7705	144	3	Sukamantri	>500	Kopi	Coffea canephora Pierre ex A.Fro	Rubiaceae	0,41	5,7	1	1,5	0,04	0,00	10,06	0,01	0,12	0,43
7706	144	3	Sukamantri	>500	Kopi	Coffea canephora Pierre ex A.Fro	Rubiaceae	0,41	6,4	2	3,5	0,04	0,00	12,48	0,01	0,15	0,54
7707	144	3	Sukamantri	>500	Kopi	Coffea canephora Pierre ex A.Fro	Rubiaceae	0,41	6,4	3	4	0,04	0,00	12,48	0,01	0,15	0,54
7708	144	3	Sukamantri	>500	Kopi	Coffea canephora Pierre ex A.Fro	Rubiaceae	0,41	4,8	0,5	1	0,04	0,00	6,92	0,01	0,08	0,30
7709	144	3	Sukamantri	>500	Kopi	Coffea canephora Pierre ex A.Fro	Rubiaceae	0,41	5,4	1,5	2	0,04	0,00	8,95	0,01	0,11	0,39
7710	144	3	Sukamantri	>500	Kopi	Coffea canephora Pierre ex A.Fro	Rubiaceae	0,41	5,7	1,2	1,5	0,04	0,00	10,06	0,01	0,12	0,43
7711	144	3	Sukamantri	>500	Kopi	Coffea canephora Pierre ex A.Fro	Rubiaceae	0,41	5,1	2,5	3	0,04	0,00	7,90	0,01	0,09	0,34
7712	144	3	Sukamantri	>500	Kihujan	Samanea saman (Jacq.) Merr.	Leguminosae / Fabaceae	0,45	8,9	2	4	0,04	0,01	10,55	0,01	0,12	0,45
7713	144	3	Sukamantri	>500	Cengkeh	Syzygium aromaticum (L.) Merr. &	Myrtaceae	0,79	3,2	2	3	0,04	0,00	2,06	0,00	0,02	0,09
7714	144	3	Sukamantri	>500	Kihujan	Samanea saman (Jacq.) Merr.	Leguminosae / Fabaceae	0,45	6,0	3	4	0,04	0,00	5,18	0,01	0,06	0,22
7715	144	3	Sukamantri	>500	Kihujan	Samanea saman (Jacq.) Merr.	Leguminosae / Fabaceae	0,45	5,1	2	3,5	0,04	0,00	3,35	0,00	0,04	0,14
7716	144	3	Sukamantri	>500	Kihujan	Samanea saman (Jacq.) Merr.	Leguminosae / Fabaceae	0,45	5,1	2	2,5	0,04	0,00	2,46	0,00	0,03	0,11
7717	144	3	Sukamantri	>500	Kihujan	Samanea saman (Jacq.) Merr.	Leguminosae / Fabaceae	0,45	5,4	2	3,5	0,04	0,00	3,74	0,00	0,04	0,16
7718	144	3	Sukamantri	>500	Cengkeh	Syzygium aromaticum (L.) Merr. &	Myrtaceae	0,79	3,2	0,5	2	0,04	0,00	1,42	0,00	0,02	0,06
7719	144	3	Sukamantri	>500	Petai	Parkia speciosa Hassk.	Leguminosae / Fabaceae	0,45	14,3	3	6	0,04	0,02	36,47	0,04	0,43	1,57
7720	144	3	Sukamantri	>500	Petai	Parkia speciosa Hassk.	Leguminosae / Fabaceae	0,45	14,3	3	7	0,04	0,02	42,00	0,04	0,49	1,81
7721	144	3	Sukamantri	>500	Kihujan	Samanea saman (Jacq.) Merr.	Leguminosae / Fabaceae	0,45	11,5	4	7	0,04	0,01	27,91	0,03	0,33	1,20
7722	144	3	Sukamantri	>500	Kihujan	Samanea saman (Jacq.) Merr.	Leguminosae / Fabaceae	0,45	3,8	2	3	0,04	0,00	1,72	0,00	0,02	0,07
7723	144	3	Sukamantri	>500	Cengkeh	Syzygium aromaticum (L.) Merr. &	Myrtaceae	0,79	3,2	1	3	0,04	0,00	2,06	0,00	0,02	0,09
7724	144	3	Sukamantri	>500	Durian	Durio zibethinus L.	Malvaceae	0,57	10,5	3	6	0,04	0,01	25,66	0,03	0,30	1,11
7725	144	3	Sukamantri	>500	Kihujan	Samanea saman (Jacq.) Merr.	Leguminosae / Fabaceae	0,45	9,5	3	7	0,04	0,01	19,98	0,02	0,23	0,86
7726	144	3	Sukamantri	>500	Kihujan	Samanea saman (Jacq.) Merr.	Leguminosae / Fabaceae	0,45	3,2	2	3	0,04	0,00	1,23	0,00	0,01	0,05
7727	144	3	Sukamantri	>500	Durian	Durio zibethinus L.	Malvaceae	0,57	17,8	5	8	0,04	0,02	87,99	0,09	1,03	3,79
7728	144	3	Sukamantri	>500	Mangga	Mangifera indica L.	Anacardiaceae	0,6	13,7	2,5	5	0,04	0,01	36,96	0,04	0,43	1,59
7729	144	3	Sukamantri	>500	Cengkeh	Syzygium aromaticum (L.) Merr. &	Myrtaceae	0,79	7,0	2	3	0,04	0,00	8,72	0,01	0,10	0,38
7730	145	3	Sukamantri	>500	Alpukat	Persea americana Mill.	Lauraceae	0,54	10,2	4	7	0,0025	0,00	26,58	0,03	5,00	18,34
7731	145	3	Sukamantri	>500	Nangka	Artocarpus heterophyllus Lam.	Moraceae	0,61	3,8	2	3	0,0025	0,00	2,27	0,00	0,43	1,56
7732	145	3	Sukamantri	>500	Alpukat	Persea americana Mill.	Lauraceae	0,54	7,0	3	5	0,0025	0,00	9,83	0,01	1,85	6,78
7733	145	3	Sukamantri	>500	Kopi	Coffea canephora Pierre ex A.Fro	Rubiaceae	0,41	2,9	1,2	2	0,0025	0,00	2,43	0,00	0,46	1,68
7734	145	3	Sukamantri	>500	Kopi	Coffea canephora Pierre ex A.Fro	Rubiaceae	0,41	2,2	1,8	2,5	0,0025	0,00	1,45	0,00	0,27	1,00
7735	145	3	Sukamantri	>500	Kopi	Coffea canephora Pierre ex A.Fro	Rubiaceae	0,41	2,5	1,2	2	0,0025	0,00	1,91	0,00	0,36	1,32
7736	145	3	Sukamantri	>500	Kopi	Coffea canephora Pierre ex A.Fro	Rubiaceae	0,41	2,9	1,5	2	0,0025	0,00	2,43	0,00	0,46	1,68
7737	145	3	Sukamantri	>500	Kopi	Coffea canephora Pierre ex A.Fro	Rubiaceae	0,41	2,2	1	1,2	0,0025	0,00	1,45	0,00	0,27	1,00
7738	145	3	Sukamantri	>500	Kopi	Coffea canephora Pierre ex A.Fro	Rubiaceae	0,41	3,2	1,8	2,5	0,0025	0,00	3,01	0,00	0,57	2,08
7739	145	3	Sukamantri	>500	Puspa	Schima wallichii Choisy	Theaceae	0,74	13,4	5	8	0,04	0,01	65,97	0,07	0,78	2,84
7740	145	3	Sukamantri	>500	Alpukat	Persea americana Mill.	Lauraceae	0,54	12,4	5	8	0,04	0,01	43,16	0,04	0,51	1,86
7741	145	3	Sukamantri	>500	Puspa	Schima wallichii Choisy	Theaceae	0,74	14,0	4	9	0,04	0,02	80,03	0,08	0,94	3,45
7742	145	3	Sukamantri	>500	Sengon	Paraserianthes falcataria (L.) I.C.	Leguminosae / Fabaceae	0,33	16,2	7	13	0,04	0,02	70,10	0,07	0,82	3,02
7743	145	3	Sukamantri	>500	Alpukat	Persea americana Mill.	Lauraceae	0,54	16,9	4	10	0,04	0,02	92,87	0,09	1,09	4,00
7744	145	3	Sukamantri	>500	Nangka	Artocarpus heterophyllus Lam.	Moraceae	0,61	7,3	2	6	0,04	0,00	14,09	0,01	0,17	0,61
7745	145	3	Sukamantri	>500	Alpukat	Persea americana Mill.	Lauraceae	0,54	5,4	3	5	0,04	0,00	6,13	0,01	0,07	0,26
7746	145	3	Sukamantri	>500	Sengon	Paraserianthes falcataria (L.) I.C.	Leguminosae / Fabaceae	0,33	6,0	4	6	0,04	0,00	5,66	0,01	0,07	0,24
7747	145	3	Sukamantri	>500	Alpukat	Persea americana Mill.	Lauraceae	0,54	8,6	3	7	0,04	0,01	19,47	0,02	0,23	0,84
7748	145	3	Sukamantri	>500	Alpukat	Persea americana Mill.	Lauraceae	0,54	6,7	3,5	7	0,04	0,00	12,29	0,01	0,14	0,53
7749	145	3	Sukamantri	>500	Sengon	Paraserianthes falcataria (L.) I.C.	Leguminosae / Fabaceae	0,33	5,1	5	7	0,04	0,00	4,75	0,00	0,06	0,21
7750	145	3	Sukamantri	>500	Sengon	Paraserianthes falcataria (L.) I.C.	Leguminosae / Fabaceae	0,33	3,2	3	4	0,04	0,00	1,20	0,00	0,01	0,05
7751	145	3	Sukamantri	>500	Afrika	Maesopsis eminii Engl.	Rhamnaceae	0,43	5,1	3	4	0,04	0,00	3,63	0,00	0,04	0,16
7752	145	3	Sukamantri	>500	Alpukat	Persea americana Mill.	Lauraceae	0,54	8,6	3	6	0,04	0,01	16,91	0,02	0,20	0,73
7753	145	3	Sukamantri	>500	Alpukat	Persea americana Mill.	Lauraceae	0,54	10,5	3	9	0,04	0,01	35,40	0,04	0,42	1,53
7754	145	3	Sukamantri	>500	Puspa	Schima wallichii Choisy	Theaceae	0,74	12,4	3	9	0,04	0,01	64,16	0,06	0,75	2,77
7755	145	3	Sukamantri	>500	Alpukat	Persea americana Mill.	Lauraceae	0,54	12,7	4	9	0,04	0,01	50,36	0,05	0,59	2,17
7756	145	3	Sukamantri	>500	Afrika	Maesopsis eminii Engl.	Rhamnaceae	0,43	5,4	3	3,5	0,04	0,00	3,59	0,00	0,04	0,15
7757	145	3	Sukamantri	>500	Puspa	Schima wallichii Choisy	Theaceae	0,74	4,5	2	4	0,04	0,00	4,67	0,00	0,05	0,20
7758	145	3	Sukamantri	>500	Puspa	Schima wallichii Choisy	Theaceae	0,74	4,1	2	4	0,04	0,00	4,08	0,00	0,05	0,18
7759	145	3	Sukamantri	>500	Afrika	Maesopsis eminii Engl.	Rhamnaceae	0,43	6,0	4	5	0,04	0,00	6,10	0,01	0,07	0,26
7760	145	3	Sukamantri	>500	Mangga	Mangifera indica L.	Anacardiaceae	0,6	8,0	2	4	0,04	0,00	11,15	0,01	0,13	0,48
7761	145	3	Sukamantri	>500	Afrika	Maesopsis eminii Engl.	Rhamnaceae	0,43	5,7	4	5	0,04	0,00	5,52	0,01	0,06	0,24
7762	145	3	Sukamantri	>500	Alpukat	Persea americana Mill.	Lauraceae	0,54	8,0	2,5	5,5	0,04	0,00	13,56	0,01	0,16	0,58
7763	145	3	Sukamantri	>500	Afrika	Maesopsis eminii Engl.	Rhamnaceae	0,43	4,1	3	4	0,04	0,00	2,48	0,00	0,03	0,11
7764	145	3	Sukamantri	>500	Alpukat	Persea americana Mill.	Lauraceae	0,54	12,4	2	6	0,04	0,01	33,16	0,03	0,39	1,43
7765	145	3	Sukamantri	>500	Alpukat	Persea americana Mill.	Lauraceae	0,54	9,5	2	6	0,04	0,01	20,51	0,02	0,24	0,88
7766	145	3	Sukamantri	>500	Afrika	Maesopsis eminii Engl.	Rhamnaceae	0,43	6,7	4	5	0,04	0,00	7,33	0,01	0,09	0,32
7767	146	3	Sukamantri	>500	Kopi	Coffea canephora Pierre ex A.Fro	Rubiaceae	0,41	1,9	0,5	1	0,0025	0,00	1,06	0,00	0,20	0,73
7768	146	3	Sukamantri	>500	Kopi	Coffea canephora Pierre ex A.Fro	Rubiaceae	0,41	1,9	0,5	1	0,0025	0,00	1,06	0,00	0,20	0,73
7769	146	3	Sukamantri	>500	Kopi	Coffea canephora Pierre ex A.Fro	Rubiaceae	0,41	2,2	0,5	1,2	0,0025	0,00	1,45	0,00	0,27	1,00
7770	146	3	Sukamantri	>500	Kopi	Coffea canephora Pierre ex A.Fro	Rubiaceae	0,41	1,9	0,5	1	0,0025	0,00	1,06	0,00	0,20	0,73
7771	146	3	Sukamantri	>500	Kopi	Coffea canephora Pierre ex A.Fro	Rubiaceae	0,41	2,2	0,5	1,2	0,0025	0,00	1,45	0,00	0,27	1,00
7772	146	3	Sukamantri	>500	Kopi	Coffea canephora Pierre ex A.Fro	Rubiaceae	0,41	3,2	0,5	1,2	0,0025	0,00	3,01	0,00	0,57	2,08
7773	146	3	Sukamantri	>500	Afrika	Maesopsis eminii Engl.	Rhamnaceae	0,43	7,3	3	7	0,04	0,00	11,78	0,01	0,14	0,51
7774	146	3	Sukamantri	>500	Nangka	Artocarpus heterophyllus Lam.	Moraceae	0,61	9,5	4	5	0,04	0,01	19,40	0,02	0,23	0,84
7775	146	3	Sukamantri	>500	Afrika	Maesopsis eminii Engl.	Rhamnaceae	0,43	9,9	8	9,5	0,04	0,01	26,92	0,03	0,32	1,16
7776	146	3	Sukamantri	>500	Afrika	Maesopsis eminii Engl.	Rhamnaceae	0,43	9,2	8	9	0,04	0,01	22,68	0,02	0,27	0,98
7777	146	3	Sukamantri	>500	Manglid	Magnolia champaca (L.) Baill. ex	Magnoliaceae	0,53	9,2	5	8	0,04	0,01	24,65	0,02	0,29	1,06
7778	146	3	Sukamantri	>500	Nangka	Artocarpus heterophyllus Lam.	Moraceae	0,61	5,1	3	5	0,04	0,00	6,13	0,01	0,07	0,26
7779	146	3	Sukamantri	>500	Afrika	Maesopsis eminii Engl.	Rhamnaceae	0,43	9,5	4	7	0,04	0,01	19,17	0,02	0,23	0,83
7780	146	3	Sukamantri	>500	Afrika	Maesopsis eminii Engl.	Rhamnaceae	0,43	10,5	4	7	0,04	0,01	22,82	0,02	0,27	0,98
7781	146	3	Sukamantri	>500	Nangka	Artocarpus heterophyllus Lam.	Moraceae	0,61	10,5	3	6	0,04	0,01	27,30	0,03	0,32	1,18
7782	146	3	Sukamantri	>500	Puspa	Schima wallichii Choisy	Theaceae	0,74	9,5	3	7	0,04	0,01	31,52	0,03	0,37	1,36
7783	146	3	Sukamantri	>500	Afrika	Maesopsis eminii Engl.	Rhamnaceae	0,43	7,0	3	4	0,04	0,00	6,50	0,01	0,08	0,28
7784	146	3	Sukamantri	>500	Alpukat	Persea americana Mill.	Lauraceae	0,54	6,0	3	4	0,04	0,00	6,13	0,01	0,07	0,26
7785	146	3	Sukamantri	>500	Afrika	Maesopsis eminii Engl.	Rhamnaceae	0,43	15,0	10	11	0,04	0,02	66,00	0,07	0,78	2,85
7786	146	3	Sukamantri	>500	Afrika	Maesopsis eminii Engl.	Rhamnaceae	0,43	12,1	8	10	0,04	0,01	40,98	0,04	0,48	1,77
7787	146	3	Sukamantri	>500	Sengon	Paraserianthes falcataria (L.) I.C.	Leguminosae / Fabaceae	0,33	15,6	6	9	0,04	0,02	46,52	0,05	0,55	2,01
7788	146	3	Sukamantri	>500	Afrika	Maesopsis eminii Engl.	Rhamnaceae	0,43	12,1	9	10	0,04	0,01	40,98	0,04	0,48	1,77
7789	146	3	Sukamantri	>500	Afrika	Maesopsis eminii Engl.	Rhamnaceae	0,43	8,0	7	8	0,04	0,00	15,51	0,02	0,18	0,67
7790	146	3	Sukamantri	>500	Afrika	Maesopsis eminii Engl.	Rhamnaceae	0,43	8,3	8	9	0,04	0,01	18,56	0,02	0,22	0,80
7791	146	3	Sukamantri	>500	Manglid	Magnolia champaca (L.) Baill. ex	Magnoliaceae	0,53	14,3	9	12	0,04	0,02	79,94	0,08	0,94	3,45
7792	146	3	Sukamantri	>500	Nangka	Artocarpus heterophyllus Lam.	Moraceae	0,61	10,5	4	6	0,04	0,01	27,30	0,03	0,32	1,18
7793	147	3	Sukamantri	>500	Kopi	Coffea canephora Pierre ex A.Fro	Rubiaceae	0,41	2,2	0,4	0,8	0,0025	0,00	1,45	0,00	0,27	1,00
7794	147	3	Sukamantri	>500	Kopi	Coffea canephora Pierre ex A.Fro	Rubiaceae	0,41	2,9	0,5	1	0,0025	0,00	2,43	0,00	0,46	1,68
7795	147	3	Sukamantri	>500	Kopi	Coffea canephora Pierre ex A.Fro	Rubiaceae	0,41	3,2	0,5	1	0,0025	0,00	3,01	0,00	0,57	2,08
7796	147	3	Sukamantri	>500	Kopi	Coffea canephora Pierre ex A.Fro	Rubiaceae	0,41	2,5	0,5	1,4	0,0025	0,00	1,91	0,00	0,36	1,32
7797	147	3	Sukamantri	>500	Manglid	Magnolia champaca (L.) Baill. ex	Magnoliaceae	0,53	3,8	2	3	0,04	0,00	1,99	0,00	0,02	0,09
7798	147	3	Sukamantri	>500	Puspa	Schima wallichii Choisy	Theaceae	0,74	14,0	5	8	0,04	0,02	71,84	0,07	0,84	3,10
7799	147	3	Sukamantri	>500	Nangka	Artocarpus heterophyllus Lam.	Moraceae	0,61	9,9	5	9	0,04	0,01	35,30	0,04	0,41	1,52
7800	147	3	Sukamantri	>500	Afrika	Maesopsis eminii Engl.	Rhamnaceae	0,43	15,6	9	11	0,04	0,02	71,24	0,07	0,84	3,07
7801	147	3	Sukamantri	>500	Afrika	Maesopsis eminii Engl.	Rhamnaceae	0,43	10,2	5	7	0,04	0,01	21,57	0,02	0,25	0,93
7802	147	3	Sukamantri	>500	Afrika	Maesopsis eminii Engl.	Rhamnaceae	0,43	13,4	7	8	0,04	0,01	40,12	0,04	0,47	1,73
7803	147	3	Sukamantri	>500	Manglid	Magnolia champaca (L.) Baill. ex	Magnoliaceae	0,53	4,1	2	3	0,04	0,00	2,31	0,00	0,03	0,10
7804	147	3	Sukamantri	>500	Padali	Radermachera gigantea (Blume)	Bignoniaceae	0,48	11,8	6	8	0,04	0,01	35,18	0,04	0,41	1,52
7805	147	3	Sukamantri	>500	Manglid	Magnolia champaca (L.) Baill. ex	Magnoliaceae	0,53	4,1	3	4	0,04	0,00	3,00	0,00	0,04	0,13
7806	147	3	Sukamantri	>500	Mahoni	Swietenia macrophylla King	Meliaceae	0,61	17,2	6	10	0,04	0,02	107,46	0,11	1,26	4,63

7807	147	3	Sukamantri	>500	Afrika	Maesopsis eminii Engl.	Rhamnaceae	0,43	11,8	6	7	0,04	0,01	28,15	0,03	0,33	1,21
7808	147	3	Sukamantri	>500	Afrika	Maesopsis eminii Engl.	Rhamnaceae	0,43	13,0	6	8	0,04	0,01	38,39	0,04	0,45	1,66
7809	147	3	Sukamantri	>500	Sengon	Paraserianthes falcataria (L.) I.C.	Leguminosae / Fabaceae	0,33	14,3	7	10	0,04	0,02	43,83	0,04	0,51	1,89
7810	147	3	Sukamantri	>500	Nangka	Artocarpus heterophyllus Lam.	Moraceae	0,61	17,5	4	8	0,04	0,02	90,58	0,09	1,06	3,91
7811	147	3	Sukamantri	>500	Sengon	Paraserianthes falcataria (L.) I.C.	Leguminosae / Fabaceae	0,33	15,0	9	13	0,04	0,02	60,36	0,06	0,71	2,60
7812	147	3	Sukamantri	>500	Nangka	Artocarpus heterophyllus Lam.	Moraceae	0,61	12,1	4	8	0,04	0,01	46,01	0,05	0,54	1,98
7813	147	3	Sukamantri	>500	Afrika	Maesopsis eminii Engl.	Rhamnaceae	0,43	14,0	6	8	0,04	0,02	43,69	0,04	0,51	1,88
7814	147	3	Sukamantri	>500	Jambu air	Syzygium aqueum (Burm.f.) Alstc	Myrtaceae	0,8	6,4	4	5,5	0,04	0,00	12,91	0,01	0,15	0,56
7815	147	3	Sukamantri	>500	Afrika	Maesopsis eminii Engl.	Rhamnaceae	0,43	11,1	5	6	0,04	0,01	22,07	0,02	0,26	0,95
7816	147	3	Sukamantri	>500	Afrika	Maesopsis eminii Engl.	Rhamnaceae	0,43	11,1	5	6	0,04	0,01	22,07	0,02	0,26	0,95
7817	147	3	Sukamantri	>500	Afrika	Maesopsis eminii Engl.	Rhamnaceae	0,43	13,0	6	7	0,04	0,01	33,97	0,03	0,40	1,46
7818	147	3	Sukamantri	>500	Afrika	Maesopsis eminii Engl.	Rhamnaceae	0,43	14,3	5	6	0,04	0,02	34,98	0,03	0,41	1,51
7819	147	3	Sukamantri	>500	Afrika	Maesopsis eminii Engl.	Rhamnaceae	0,43	10,8	6	7	0,04	0,01	24,11	0,02	0,28	1,04
7820	147	3	Sukamantri	>500	Afrika	Maesopsis eminii Engl.	Rhamnaceae	0,43	11,1	6	7	0,04	0,01	25,42	0,03	0,30	1,10
7821	147	3	Sukamantri	>500	Afrika	Maesopsis eminii Engl.	Rhamnaceae	0,43	10,5	5	7	0,04	0,01	22,82	0,02	0,27	0,98
7822	147	3	Sukamantri	>500	Afrika	Maesopsis eminii Engl.	Rhamnaceae	0,43	3,8	2	3,5	0,04	0,00	1,90	0,00	0,02	0,08
7823	147	3	Sukamantri	>500	Aren	Arenga pinnata (Wurmb) Merr.	Arecaceae	0,71	47,7	7	11	0,04	0,18	89,20	0,09	1,05	3,85
7824	147	3	Sukamantri	>500	Sengon	Paraserianthes falcataria (L.) I.C.	Leguminosae / Fabaceae	0,33	2,2	2	3	0,04	0,00	0,48	0,00	0,01	0,02
7825	147	3	Sukamantri	>500	Sengon	Paraserianthes falcataria (L.) I.C.	Leguminosae / Fabaceae	0,33	3,2	2	3	0,04	0,00	0,92	0,00	0,01	0,04
7826	147	3	Sukamantri	>500	Afrika	Maesopsis eminii Engl.	Rhamnaceae	0,43	12,4	6	7	0,04	0,01	31,00	0,03	0,36	1,34
7827	147	3	Sukamantri	>500	Afrika	Maesopsis eminii Engl.	Rhamnaceae	0,43	13,4	5	6	0,04	0,01	30,83	0,03	0,36	1,33
7828	147	3	Sukamantri	>500	Mahoni	Swietenia macrophylla King	Meliaceae	0,61	22,0	8	11	0,04	0,04	183,73	0,18	2,16	7,92
7829	147	3	Sukamantri	>500	Aren	Arenga pinnata (Wurmb) Merr.	Arecaceae	0,71	47,7	3	9	0,04	0,18	73,80	0,07	0,87	3,18
7830	147	3	Sukamantri	>500	Sengon	Paraserianthes falcataria (L.) I.C.	Leguminosae / Fabaceae	0,33	4,1	3	4,5	0,04	0,00	2,17	0,00	0,03	0,09
7831	147	3	Sukamantri	>500	Nangka	Artocarpus heterophyllus Lam.	Moraceae	0,61	15,0	4	9	0,04	0,02	75,66	0,08	0,89	3,26
7832	147	3	Sukamantri	>500	Mahoni	Swietenia macrophylla King	Meliaceae	0,61	15,3	4	8	0,04	0,02	70,59	0,07	0,83	3,04
7833	148	3	Sukamantri	>500	Kopi	Coffea canephora Pierre ex A.Frc	Rubiaceae	0,41	4,8	5	1	0,0025	0,00	6,92	0,01	1,30	4,78
7834	148	3	Sukamantri	>500	Kopi	Coffea canephora Pierre ex A.Frc	Rubiaceae	0,41	4,8	1	1,2	0,0025	0,00	6,92	0,01	1,30	4,78
7835	148	3	Sukamantri	>500	Kopi	Coffea canephora Pierre ex A.Frc	Rubiaceae	0,41	5,1	0,5	1,2	0,0025	0,00	7,90	0,01	1,49	5,45
7836	148	3	Sukamantri	>500	Kopi	Coffea canephora Pierre ex A.Frc	Rubiaceae	0,41	4,8	1	1,8	0,0025	0,00	6,92	0,01	1,30	4,78
7837	148	3	Sukamantri	>500	Kopi	Coffea canephora Pierre ex A.Frc	Rubiaceae	0,41	4,1	0,8	1,4	0,0025	0,00	5,16	0,01	0,97	3,56
7838	148	3	Sukamantri	>500	Kopi	Coffea canephora Pierre ex A.Frc	Rubiaceae	0,41	4,8	0,8	1,6	0,0025	0,00	6,92	0,01	1,30	4,78
7839	148	3	Sukamantri	>500	Kopi	Coffea canephora Pierre ex A.Frc	Rubiaceae	0,41	3,8	0,5	0,8	0,0025	0,00	4,38	0,00	0,82	3,02
7840	148	3	Sukamantri	>500	Kopi	Coffea canephora Pierre ex A.Frc	Rubiaceae	0,41	4,1	0,8	1,4	0,0025	0,00	5,16	0,01	0,97	3,56
7841	148	3	Sukamantri	>500	Kopi	Coffea canephora Pierre ex A.Frc	Rubiaceae	0,41	4,5	1	1,8	0,0025	0,00	6,01	0,01	1,13	4,15
7842	148	3	Sukamantri	>500	Puspa	Schima wallichii Choisy	Theaceae	0,74	18,8	4	9	0,04	0,03	136,97	0,14	1,61	5,91
7843	148	3	Sukamantri	>500	Puspa	Schima wallichii Choisy	Theaceae	0,74	24,2	3	9	0,04	0,05	217,81	0,22	2,56	9,39
7844	148	3	Sukamantri	>500	Puspa	Schima wallichii Choisy	Theaceae	0,74	15,6	5	14	0,04	0,02	146,09	0,15	1,72	6,30
7845	148	3	Sukamantri	>500	Puspa	Schima wallichii Choisy	Theaceae	0,74	16,2	5	15	0,04	0,02	167,46	0,17	1,97	7,22
7846	148	3	Sukamantri	>500	Puspa	Schima wallichii Choisy	Theaceae	0,74	17,5	5	14	0,04	0,02	180,52	0,18	2,12	7,78
7847	148	3	Sukamantri	>500	Puspa	Schima wallichii Choisy	Theaceae	0,74	14,3	5	14	0,04	0,02	124,99	0,12	1,47	5,39
7848	148	3	Sukamantri	>500	Puspa	Schima wallichii Choisy	Theaceae	0,74	17,5	5	14	0,04	0,02	180,52	0,18	2,12	7,78
7849	148	3	Sukamantri	>500	Puspa	Schima wallichii Choisy	Theaceae	0,74	28,3	3	13	0,04	0,06	407,37	0,41	4,79	17,57
7850	148	3	Sukamantri	>500	Sengon	Paraserianthes falcataria (L.) I.C.	Leguminosae / Fabaceae	0,33	39,8	10	17	0,04	0,12	463,14	0,46	5,44	19,97
7851	148	3	Sukamantri	>500	Sengon	Paraserianthes falcataria (L.) I.C.	Leguminosae / Fabaceae	0,33	15,3	7	11	0,04	0,02	53,83	0,05	0,63	2,32
7852	148	3	Sukamantri	>500	Sengon	Paraserianthes falcataria (L.) I.C.	Leguminosae / Fabaceae	0,33	18,8	8	18	0,04	0,03	123,34	0,12	1,45	5,32
7853	148	3	Sukamantri	>500	Puspa	Schima wallichii Choisy	Theaceae	0,74	25,8	2	10	0,04	0,05	269,58	0,27	3,17	11,62
7854	148	3	Sukamantri	>500	Puspa	Schima wallichii Choisy	Theaceae	0,74	12,1	5	13	0,04	0,01	85,68	0,09	1,01	3,69
7855	148	3	Sukamantri	>500	Puspa	Schima wallichii Choisy	Theaceae	0,74	14,6	5	14	0,04	0,02	130,13	0,13	1,53	5,61
7856	148	3	Sukamantri	>500	Puspa	Schima wallichii Choisy	Theaceae	0,74	17,5	5	15	0,04	0,02	192,30	0,19	2,26	8,29
7857	148	3	Sukamantri	>500	Puspa	Schima wallichii Choisy	Theaceae	0,74	17,8	5	15	0,04	0,02	198,75	0,20	2,34	8,57
7858	148	3	Sukamantri	>500	Puspa	Schima wallichii Choisy	Theaceae	0,74	15,0	3	12	0,04	0,02	117,53	0,12	1,38	5,07
7859	148	3	Sukamantri	>500	Puspa	Schima wallichii Choisy	Theaceae	0,74	22,3	3	14	0,04	0,04	280,81	0,28	3,30	12,11
7860	148	3	Sukamantri	>500	Puspa	Schima wallichii Choisy	Theaceae	0,74	11,1	3	6	0,04	0,01	36,30	0,04	0,43	1,57
7861	148	3	Sukamantri	>500	Aren	Arenga pinnata (Wurmb) Merr.	Arecaceae	0,71	46,1	3	9	0,04	0,17	73,80	0,07	0,87	3,18
7862	148	3	Sukamantri	>500	Nangka	Artocarpus heterophyllus Lam.	Moraceae	0,61	10,8	3	5	0,04	0,01	24,40	0,02	0,29	1,05
7863	148	3	Sukamantri	>500	Manglid	Magnolia champaca (L.) Baill. ex	Magnoliaceae	0,53	27,0	9	16	0,04	0,06	333,60	0,33	3,92	14,39
7864	148	3	Sukamantri	>500	Tisuk	Hibiscus macrophyllus Roxb. ex I	Malvaceae	0,4	22,0	10	16	0,04	0,04	175,93	0,18	2,07	7,59
7865	149	1	Sukamantri	>500	Ganitri	Elaeocarpus ganitrus Roxb. ex G.	Elaeocarpaceae	0,49	2,5	2	3	0,01	0,00	0,88	0,00	0,04	0,15
7866	149	1	Sukamantri	>500	Ganitri	Elaeocarpus ganitrus Roxb. ex G.	Elaeocarpaceae	0,49	5,1	4	5	0,01	0,00	5,02	0,01	0,24	0,87
7867	149	1	Sukamantri	>500	Ganitri	Elaeocarpus ganitrus Roxb. ex G.	Elaeocarpaceae	0,49	9,9	5	7	0,01	0,00	22,94	0,02	1,08	3,96
7868	149	1	Sukamantri	>500	Sengon	Paraserianthes falcataria (L.) I.C.	Leguminosae / Fabaceae	0,33	4,5	5	6	0,01	0,00	3,23	0,00	0,15	0,56
7869	149	1	Sukamantri	>500	Ganitri	Elaeocarpus ganitrus Roxb. ex G.	Elaeocarpaceae	0,49	4,5	2	3	0,01	0,00	2,46	0,00	0,12	0,42
7870	149	1	Sukamantri	>500	Ganitri	Elaeocarpus ganitrus Roxb. ex G.	Elaeocarpaceae	0,49	5,1	5	6	0,01	0,00	5,93	0,01	0,28	1,02
7871	149	1	Sukamantri	>500	Afrika	Maesopsis eminii Engl.	Rhamnaceae	0,43	16,5	7	10	0,01	0,01	72,79	0,07	3,42	12,56
7872	149	1	Sukamantri	>500	Afrika	Maesopsis eminii Engl.	Rhamnaceae	0,43	15,0	7	10	0,01	0,00	60,49	0,06	2,84	10,43
7873	149	1	Sukamantri	>500	Kilunglum	Sp3	0	0,5	3,2	3	4,5	0,01	0,00	1,96	0,00	0,09	0,34
7874	149	1	Sukamantri	>500	Ganitri	Elaeocarpus ganitrus Roxb. ex G.	Elaeocarpaceae	0,49	6,4	30	6	0,01	0,00	8,93	0,01	0,42	1,54
7875	149	1	Sukamantri	>500	Ganitri	Elaeocarpus ganitrus Roxb. ex G.	Elaeocarpaceae	0,49	4,1	4	5,5	0,01	0,00	3,74	0,00	0,18	0,65
7876	149	1	Sukamantri	>500	Ganitri	Elaeocarpus ganitrus Roxb. ex G.	Elaeocarpaceae	0,49	4,1	4	5	0,01	0,00	3,43	0,00	0,16	0,59
7877	149	1	Sukamantri	>500	Afrika	Maesopsis eminii Engl.	Rhamnaceae	0,43	11,5	8	9	0,01	0,00	33,70	0,03	1,58	5,81
7878	149	1	Sukamantri	>500	Afrika	Maesopsis eminii Engl.	Rhamnaceae	0,43	12,4	8	9	0,01	0,00	39,02	0,04	1,83	6,73
7879	149	1	Sukamantri	>500	Afrika	Maesopsis eminii Engl.	Rhamnaceae	0,43	10,8	8	9	0,01	0,00	30,35	0,03	1,43	5,23
7880	149	1	Sukamantri	>500	Ganitri	Elaeocarpus ganitrus Roxb. ex G.	Elaeocarpaceae	0,49	3,8	2	3,5	0,01	0,00	2,14	0,00	0,10	0,37
7881	149	1	Sukamantri	>500	Nangka	Artocarpus heterophyllus Lam.	Moraceae	0,61	3,2	1,8	3	0,01	0,00	1,62	0,00	0,08	0,28
7882	149	1	Sukamantri	>500	Ganitri	Elaeocarpus ganitrus Roxb. ex G.	Elaeocarpaceae	0,49	5,1	3	5	0,01	0,00	5,02	0,01	0,24	0,87
7883	149	1	Sukamantri	>500	Sengon	Paraserianthes falcataria (L.) I.C.	Leguminosae / Fabaceae	0,33	18,5	10	13	0,01	0,01	88,73	0,09	4,17	15,30
7884	149	1	Sukamantri	>500	Ganitri	Elaeocarpus ganitrus Roxb. ex G.	Elaeocarpaceae	0,49	6,0	4	5,5	0,01	0,00	7,50	0,01	0,35	1,29
7885	149	1	Sukamantri	>500	Afrika	Maesopsis eminii Engl.	Rhamnaceae	0,43	4,8	6	7	0,01	0,00	5,38	0,01	0,25	0,93
7886	149	1	Sukamantri	>500	Ganitri	Elaeocarpus ganitrus Roxb. ex G.	Elaeocarpaceae	0,49	4,5	2	4	0,01	0,00	3,20	0,00	0,15	0,55
7887	149	1	Sukamantri	>500	Ganitri	Elaeocarpus ganitrus Roxb. ex G.	Elaeocarpaceae	0,49	3,8	2	4	0,01	0,00	2,41	0,00	0,11	0,42
7888	149	1	Sukamantri	>500	Afrika	Maesopsis eminii Engl.	Rhamnaceae	0,43	8,0	9	10	0,01	0,00	19,03	0,02	0,89	3,28
7889	149	1	Sukamantri	>500	Afrika	Maesopsis eminii Engl.	Rhamnaceae	0,43	8,6	9	10	0,01	0,00	21,91	0,02	1,03	3,78
7890	149	1	Sukamantri	>500	Ganitri	Elaeocarpus ganitrus Roxb. ex G.	Elaeocarpaceae	0,49	2,5	2	3	0,01	0,00	0,88	0,00	0,04	0,15
7891	149	1	Sukamantri	>500	Afrika	Maesopsis eminii Engl.	Rhamnaceae	0,43	8,9	7	8	0,01	0,00	19,09	0,02	0,90	3,29
7892	149	1	Sukamantri	>500	Ganitri	Elaeocarpus ganitrus Roxb. ex G.	Elaeocarpaceae	0,49	2,9	2	3	0,01	0,00	1,10	0,00	0,05	0,19
7893	149	1	Sukamantri	>500	Afrika	Maesopsis eminii Engl.	Rhamnaceae	0,43	9,2	7	8	0,01	0,00	20,36	0,02	0,96	3,51
7894	149	1	Sukamantri	>500	Afrika	Maesopsis eminii Engl.	Rhamnaceae	0,43	18,8	8	9	0,01	0,01	83,30	0,08	3,92	14,37
7895	149	1	Sukamantri	>500	Ganitri	Elaeocarpus ganitrus Roxb. ex G.	Elaeocarpaceae	0,49	5,4	2	4	0,01	0,00	4,57	0,00	0,21	0,79
7896	149	1	Sukamantri	>500	Ganitri	Elaeocarpus ganitrus Roxb. ex G.	Elaeocarpaceae	0,49	9,5	3	7	0,01	0,00	21,60	0,02	1,02	3,73
7897	149	1	Sukamantri	>500	Sengon	Paraserianthes falcataria (L.) I.C.	Leguminosae / Fabaceae	0,33	3,8	5	6	0,01	0,00	2,44	0,00	0,11	0,42
7898	149	1	Sukamantri	>500	Sengon	Paraserianthes falcataria (L.) I.C.	Leguminosae / Fabaceae	0,33	4,1	5	6	0,01	0,00	2,82	0,00	0,13	0,49
7899	149	1	Sukamantri	>500	Afrika	Maesopsis eminii Engl.	Rhamnaceae	0,43	4,1	4	5	0,01	0,00	3,04	0,00	0,14	0,52
7900	149	1	Sukamantri	>500	Kihaji	Dysoxylum macrocarpum Blume	Meliaceae	0,73	6,7	3	5	0,01	0,00	11,90	0,01	0,56	2,05
7901	149	1	Sukamantri	>500	Afrika	Maesopsis eminii Engl.	Rhamnaceae	0,43	8,6	6	7	0,01	0,00	15,80	0,02	0,74	2,73
7902	149	1	Sukamantri	>500	Afrika	Maesopsis eminii Engl.	Rhamnaceae	0,43	15,0	8	9	0,01	0,00	54,92	0,05	2,58	9,47
7903	149	1	Sukamantri	>500	Afrika	Maesopsis eminii Engl.	Rhamnaceae	0,43	14,0	8	9	0,01	0,00	48,67	0,05	2,29	8,40
7904	149	1	Sukamantri	>500	Afrika	Maesopsis eminii Engl.	Rhamnaceae	0,43	3,2	5	6	0,01	0,00	2,22	0,00	0,10	0,38
7905	149	1	Sukamantri	>500	Manglid	Magnolia champaca (L.) Baill. ex	Magnoliaceae	0,53	3,8	2	4	0,01	0,00	2,59	0,00	0,12	0,45
7906	149	1	Sukamantri	>500	Ganitri	Elaeocarpus ganitrus Roxb. ex G.	Elaeocarpaceae	0,49	5,7	4	5	0,01	0,00	6,23	0,01	0,29	1,07
7907	149	1	Sukamantri	>500	Afrika	Maesopsis eminii Engl.	Rhamnaceae	0,43	10,2	6	7,5	0,01	0,00	22,98	0,02	1,08	3,96
7908	149	1	Sukamantri	>500	Afrika	Maesopsis eminii Engl.	Rhamnaceae	0,43	12,1	7	8	0,04	0,01	33,40	0,03	0,39	1,44
7909	149	1	Sukamantri	>500	Sengon	Paraserianthes falcataria (L.) I.C.	Leguminosae / Fabaceae	0,33	10,2	7	8	0,04	0,01	19,13	0,02	0,22	0,82
7910	149	1	Sukamantri	>500	Afrika	Maesopsis eminii Engl.	Rhamnaceae	0,43	15,0	9	10	0,04	0,02	60,49	0,06	0,71	2,61
7911	149	1	Sukamantri	>500	Afrika	Maesopsis eminii Engl.	Rhamnaceae	0,43	12,1	8	9	0,04	0,01	37,21	0,04	0,44	1,60
7912	149	1	Sukamantri	>500	Afrika	Maesopsis eminii Engl.	Rhamnaceae	0,43	13,0	9	10	0,04	0,01	47,10	0,05	0,55	2,03
7913	149	1	Sukamantri	>500	Afrika	Maesopsis eminii Engl.	Rhamnaceae	0,43	10,5	9	10	0,04	0,01	31,64	0,03	0,37	1,36
7914	149	1	Sukamantri	>500	Afrika	Maesopsis eminii Engl.	Rhamnaceae	0,43	19,7	9	10	0,04	0,03	100,47	0,10	1,18	4,33
7915	149	1	Sukamantri	>500	Afrika	Maesopsis eminii Engl.	Rhamnaceae	0,43	10,2	7	8,5	0,04	0,01	25,77	0,03	0,30	1,11
7916	149	1	Sukamantri	>500	Afrika	Maesopsis eminii Engl.	Rhamnaceae	0,43	12,1	7	9	0,04	0,01	37,21	0,04	0,44	1,60
7917	149	1	Sukamantri	>500	Sengon	Paraserianthes falcataria (L.) I.C.	Leguminosae / Fabaceae	0,33	30,2	6	11	0,04	0,07	188,01	0,19	2,21	8,11
7918	149	1	Sukamantri	>500	Afrika	Maesopsis eminii Engl.	Rhamnaceae	0,43	15,3	9	11	0,04	0,02	68,60	0,07	0,81	2,96
7919	149	1	Sukamantri	>500	Sengon	Paraserianthes falcataria (L.) I.C.	Leguminosae / Fabaceae	0,33	13,0	10	12	0,04	0,01	43,67	0,04	0,51	1,88
7920	149	1	Sukamantri	>500	Afrika	Maesopsis eminii Engl.	Rhamnaceae	0,43	10,2	9	10	0,04	0,01	29,91	0,03	0,35	1,29
7921	149	1	Sukamantri	>500	Afrika	Maesopsis eminii Engl.	Rhamnaceae	0,43	17,2	10	12	0,04	0,02	92,18	0,09	1,08	3,98
7922	149	1	Sukamantri	>500	Afrika	Maesopsis eminii Engl.	Rhamnaceae	0,43	17,5	10	12	0,04	0,02	95,33	0,10	1,12	4,11
7923	149	1	Sukamantri	>500	Afrika	Maesopsis eminii Engl.	Rhamnaceae	0,43	16,2	9	10	0,04	0,02	70,25	0,07	0,83	3,03
7924	149	1	Sukamantri	>500	Afrika	Maesopsis eminii Engl.	Rhamnaceae	0,43	11,1	7	9	0,04	0,01	32,00	0,03	0,38	1,38
7925	149	1	Sukamantri	>500	Sengon	Paraserianthes falcataria (L.) I.C.	Leguminosae / Fabaceae	0,33	11,1	10	12	0,04	0,01	32,68	0,03	0,38	1,41
7926	150	1	Sukamantri	>500	Afrika	Maesopsis eminii Engl.	Rhamnaceae	0,43	6,7	9	10	0,01	0,00	13,83	0,01	0,65	2,38
7927	150	1	Sukamantri	>500	Ganitri	Elaeocarpus ganitrus Roxb. ex G.	Elaeocarpaceae	0,49	6,0	7	10	0,01	0,00	12,97	0,01	0,61	2,24
7928	150	1	Sukamantri	>500	Ganitri	Elaeocarpus ganitrus Roxb. ex G.	Elaeocarpaceae	0,49	8,6	9	11	0,01	0,00	26,95	0,03	1,27	4,65
7929	150	1	Sukamantri	>500	Ganitri	Elaeocarpus ganitrus Roxb. ex G.	Elaeocarpaceae	0,49	4,8	5	7	0,01	0,00	6,07	0,01	0,29	1,05
7930	150	1	Sukamantri	>500	Ganitri	Elaeocarpus ganitrus Roxb. ex G.	Elaeocarpaceae	0,49	8,0	9	11	0,01	0,00	23,40	0,02	1,10	4,04

7931	150	1	Sukamantri	>500	Nangka	Artocarpus heterophyllus Lam.	Moraceae	0,61	21,0	7	11	0,01	0,01	169,36	0,17	7,96	29,21
7932	150	1	Sukamantri	>500	Ganitri	Elaeocarpus ganitrus Roxb. ex G.	Elaeocarpaceae	0,49	5,1	6	8	0,01	0,00	7,72	0,01	0,36	1,33
7933	150	1	Sukamantri	>500	Ganitri	Elaeocarpus ganitrus Roxb. ex G.	Elaeocarpaceae	0,49	4,5	4	6	0,01	0,00	4,64	0,00	0,22	0,80
7934	150	1	Sukamantri	>500	Ganitri	Elaeocarpus ganitrus Roxb. ex G.	Elaeocarpaceae	0,49	3,2	3	4,5	0,01	0,00	1,93	0,00	0,09	0,33
7935	150	1	Sukamantri	>500	Ganitri	Elaeocarpus ganitrus Roxb. ex G.	Elaeocarpaceae	0,49	3,2	3	4,5	0,01	0,00	1,93	0,00	0,09	0,33
7936	150	1	Sukamantri	>500	Afrika	Maesopsis eminii Engl.	Rhamnaceae	0,43	6,4	8	9	0,01	0,00	11,48	0,01	0,54	1,98
7937	150	1	Sukamantri	>500	Ganitri	Elaeocarpus ganitrus Roxb. ex G.	Elaeocarpaceae	0,49	4,1	3	4,5	0,01	0,00	3,11	0,00	0,15	0,54
7938	150	1	Sukamantri	>500	Ganitri	Elaeocarpus ganitrus Roxb. ex G.	Elaeocarpaceae	0,49	7,3	3	7	0,01	0,00	13,28	0,01	0,62	2,29
7939	150	1	Sukamantri	>500	Ganitri	Elaeocarpus ganitrus Roxb. ex G.	Elaeocarpaceae	0,49	10,8	10	12	0,01	0,00	44,52	0,04	2,09	7,68
7940	150	1	Sukamantri	>500	Ganitri	Elaeocarpus ganitrus Roxb. ex G.	Elaeocarpaceae	0,49	4,8	7	9	0,01	0,00	7,64	0,01	0,36	1,32
7941	150	1	Sukamantri	>500	Afrika	Maesopsis eminii Engl.	Rhamnaceae	0,43	5,4	15	17	0,01	0,00	15,26	0,02	0,72	2,63
7942	150	1	Sukamantri	>500	Ganitri	Elaeocarpus ganitrus Roxb. ex G.	Elaeocarpaceae	0,49	6,7	7	9	0,01	0,00	14,15	0,01	0,67	2,44
7943	150	1	Sukamantri	>500	Afrika	Maesopsis eminii Engl.	Rhamnaceae	0,43	15,6	10	12	0,01	0,00	77,15	0,08	3,63	13,31
7944	150	1	Sukamantri	>500	Ganitri	Elaeocarpus ganitrus Roxb. ex G.	Elaeocarpaceae	0,49	7,3	7	10	0,01	0,00	18,41	0,02	0,87	3,18
7945	150	1	Sukamantri	>500	Ganitri	Elaeocarpus ganitrus Roxb. ex G.	Elaeocarpaceae	0,49	7,3	7	10	0,01	0,00	18,41	0,02	0,87	3,18
7946	150	1	Sukamantri	>500	Ganitri	Elaeocarpus ganitrus Roxb. ex G.	Elaeocarpaceae	0,49	4,1	5	6	0,01	0,00	4,05	0,00	0,19	0,70
7947	150	1	Sukamantri	>500	Mahoni afrika	Khaya anthotheca (Welw.) C.DC.	Meliaceae	0,49	6,4	5	6,5	0,01	0,00	9,60	0,01	0,45	1,66
7948	150	1	Sukamantri	>500	Ganitri	Elaeocarpus ganitrus Roxb. ex G.	Elaeocarpaceae	0,49	5,1	5	7	0,01	0,00	6,83	0,01	0,32	1,18
7949	150	1	Sukamantri	>500	Ganitri	Elaeocarpus ganitrus Roxb. ex G.	Elaeocarpaceae	0,49	7,3	11	13	0,01	0,00	23,41	0,02	1,10	4,04
7950	150	1	Sukamantri	>500	Ganitri	Elaeocarpus ganitrus Roxb. ex G.	Elaeocarpaceae	0,49	11,5	8	12	0,01	0,00	49,43	0,05	2,32	8,53
7951	150	1	Sukamantri	>500	Ganitri	Elaeocarpus ganitrus Roxb. ex G.	Elaeocarpaceae	0,49	4,8	6	7	0,01	0,00	6,07	0,01	0,29	1,05
7952	150	1	Sukamantri	>500	Ganitri	Elaeocarpus ganitrus Roxb. ex G.	Elaeocarpaceae	0,49	4,1	4	3,3	0,01	0,00	2,34	0,00	0,11	0,40
7953	150	1	Sukamantri	>500	Ganitri	Elaeocarpus ganitrus Roxb. ex G.	Elaeocarpaceae	0,49	9,2	8	11	0,01	0,00	30,72	0,03	1,44	5,30
7954	150	1	Sukamantri	>500	Afrika	Maesopsis eminii Engl.	Rhamnaceae	0,43	14,0	10	12	0,01	0,00	63,34	0,06	2,98	10,93
7955	150	1	Sukamantri	>500	Ganitri	Elaeocarpus ganitrus Roxb. ex G.	Elaeocarpaceae	0,49	3,8	2	3	0,01	0,00	1,86	0,00	0,09	0,32
7956	150	1	Sukamantri	>500	Ganitri	Elaeocarpus ganitrus Roxb. ex G.	Elaeocarpaceae	0,49	8,3	7	10	0,01	0,00	23,04	0,02	1,08	3,97
7957	150	1	Sukamantri	>500	Ganitri	Elaeocarpus ganitrus Roxb. ex G.	Elaeocarpaceae	0,49	5,7	4	6	0,01	0,00	7,36	0,01	0,35	1,27
7958	150	1	Sukamantri	>500	Ganitri	Elaeocarpus ganitrus Roxb. ex G.	Elaeocarpaceae	0,49	9,2	7	10	0,01	0,00	28,15	0,03	1,32	4,86
7959	150	1	Sukamantri	>500	Sengon	Paraserianthes falcataria (L.) I.C.	Leguminosae / Fabaceae	0,33	13,4	12	14	0,01	0,00	52,57	0,05	2,47	9,07
7960	150	1	Sukamantri	>500	Ganitri	Elaeocarpus ganitrus Roxb. ex G.	Elaeocarpaceae	0,49	15,3	10	15	0,01	0,00	102,72	0,10	4,83	17,72
7961	150	1	Sukamantri	>500	Ganitri	Elaeocarpus ganitrus Roxb. ex G.	Elaeocarpaceae	0,49	10,5	7	12	0,01	0,00	42,15	0,04	1,98	7,27
7962	150	1	Sukamantri	>500	Afrika	Maesopsis eminii Engl.	Rhamnaceae	0,43	22,6	15	17	0,04	0,04	209,40	0,21	2,46	9,03
7963	150	1	Sukamantri	>500	Afrika	Maesopsis eminii Engl.	Rhamnaceae	0,43	24,8	15	17	0,04	0,05	248,76	0,25	2,92	10,73
7964	150	1	Sukamantri	>500	Afrika	Maesopsis eminii Engl.	Rhamnaceae	0,43	23,9	15	18	0,04	0,04	243,96	0,24	2,87	10,52
7965	150	1	Sukamantri	>500	Afrika	Maesopsis eminii Engl.	Rhamnaceae	0,43	26,4	14	17	0,04	0,05	278,75	0,28	3,28	12,02
7966	150	1	Sukamantri	>500	Afrika	Maesopsis eminii Engl.	Rhamnaceae	0,43	28,0	16	20	0,04	0,06	360,09	0,36	4,23	15,53
7967	150	1	Sukamantri	>500	Afrika	Maesopsis eminii Engl.	Rhamnaceae	0,43	19,4	16	19	0,04	0,03	175,57	0,18	2,06	7,57
7968	150	1	Sukamantri	>500	Afrika	Maesopsis eminii Engl.	Rhamnaceae	0,43	24,5	17	20	0,04	0,05	281,95	0,28	3,31	12,16
7969	150	1	Sukamantri	>500	Afrika	Maesopsis eminii Engl.	Rhamnaceae	0,43	19,1	15	17	0,04	0,03	153,83	0,15	1,81	6,63
7970	150	1	Sukamantri	>500	Nangka	Artocarpus heterophyllus Lam.	Moraceae	0,61	23,9	6	14	0,04	0,04	266,96	0,27	3,14	11,51
7971	150	1	Sukamantri	>500	Afrika	Maesopsis eminii Engl.	Rhamnaceae	0,43	20,0	14	16	0,04	0,03	159,13	0,16	1,87	6,86
